# Antineoplastic 4-piperidone-1-phosphonothioates with potential multi-targeted inhibitory properties

**DOI:** 10.1038/s41598-025-25796-6

**Published:** 2025-11-18

**Authors:** Mohamed S. Bekheit, Siva S. Panda, Benson M Kariuki, Walid Fayad, Ahmed A. F. Soliman, Hanaa Farag, Adel S. Girgis

**Affiliations:** 1 Department of Pesticide Chemistry, National Research Centre, Dokki, 12622 Giza Egypt; 2 Department of Chemistry and Biochemistry & Department of Biochemistry and Molecular Biology, Augusta University, Augusta, GA 30912 USA; 3 School of Chemistry, Cardiff University, Main Building, Park Place, CF10 3AT Cardiff UK; 4 Drug Bioassay-Cell Culture Laboratory, Pharmacognosy Department, National Research Centre, Dokki, 12622 Giza Egypt

**Keywords:** 4-Piperidone-1-phosphonothioate, Cancer, MDM2-p53, Topoisomerase, Molecular modeling, Breast cancer, Cancer screening, Skin cancer, Cancer, Chemistry

## Abstract

A set of 3,5-bis(ylidene)-4-piperidone-1-phosphonothioates **20a‒l** was synthesized in high yields through dehydrochlorination reaction of 3,5-bis(ylidene)-4-piperidones **18a‒l** and diethyl chlorothiophosphate **19** in DMF containing quantitative amounts of TEA at 0 °C. Most of the synthesized agents exhibit effective antiproliferation properties against a variety of cancer cell lines (MCF7/breast, HCT116/colon, and A431/skin), outperforming the efficacy of the clinically approved drugs. Compound **20c** (R = 3-ClC_6_H_4_) is the most effective analog discovered with a sub-micromolar activity against MCF7 (IC_50_ = 0.650, 3.15 and 3.97 µM, for **20c**, 5-fluorouracil, and sunitinib, respectively) as well as remarkable efficacy against HCT116, and A431 (IC_50_ = 1.445 and 2.920 µM respectively for **20c**, compared to 20.43 and 23.44 µM respectively for 5-fluorouracil). The synthesized analogs demonstrating considerable anti-MCF7 properties were evaluated for their biochemical properties against MDM2, p53, and topoisomerase I/II. Compound **20k** [R = 3,4,5-(MeO)_3_C_6_H_2_] is the most effective anti-MDM2 agent with a potency higher than the standard reference (% inhibition of 65.3, for **20k**, and 42.2 for doxorubicin; i.e. 1.55-fold). Biochemical activation of p53 by the synthesized analogs is consistent with the anti-MDM2 properties. Compound **20i** (R = 4-MeOC_6_H_4_) is the most effective topo-I and topo-IIα inhibitor with potency close to that of the standard references (Dxd, and etoposide). Some of the synthesized agents showed selectivity for topo-IIα over topo-I. Molecular docking results (PDB: 4OAS) are consistent with the observed properties against MDM2. Based on the biological and biochemical findings, some of the synthesized compounds could serve as promising multi-targeted inhibitors.

**Supplementary Information:**

The online version contains supplementary material available at 10.1038/s41598-025-25796-6.

## Introduction

The multi-targeted therapeutic approach continues to gain popularity in medicinal chemistry. The technique enables the design of effective and versatile agents that can overcome drawbacks of single-target therapeutics such as off-target (i.e. poor target selectivity), drug resistance, and side effect problems. This can be attained by, for example, constructing a molecular structure capable of interacting and/or binding with two or more cellular targets to render the net bio-observations with high potency and broad efficacy. In other words, the multi-targeted agent can effectively act as a dual inhibitor impacting different and/or independent receptors, thereby maximizing the potential therapeutic output^1–3^.

Adoption of a specific pharmacophoric unit or conjugation of two or more pharmacophoric residues capable of interaction with various receptors responsible for controlling specific cellular biochemical pathways has been the subject of numerous reports. Many biologically active agents have been designed this way and experimentally demonstrated as antibacterial^4^, antifungal^5^, antiviral^6,7^, anticancer^8,9^, and agents against Alzheimer’s disease^10^. The current study is directed towards design and generation of novel 4-piperidone-1-phosphonothioates manifesting potential antineoplastic properties. Notably, analogs of this heterocyclic scaffold have shown distinguished antitumor behavior against many cancer types,^11–17^ besides diverse biological observations as anti-inflammatory^18,19^, and anticholinesterases evidencing activity against Alzheimer’s disease^20,21^.

Considering that several receptors and signals have been identified as controlling elements in cancer progression, multi-targeted therapeutics could be more favorable for combating cancer than the mono-targeted compounds^22,23^. Interest in conjugation of thiophosphate function with the targeted heterocycle is due to its electronic constitution that could give rise to interaction with the amino acids within the protein active site of the targeted protein, thus providing high potency towards the intended mode of action.

**Fig. 1:**
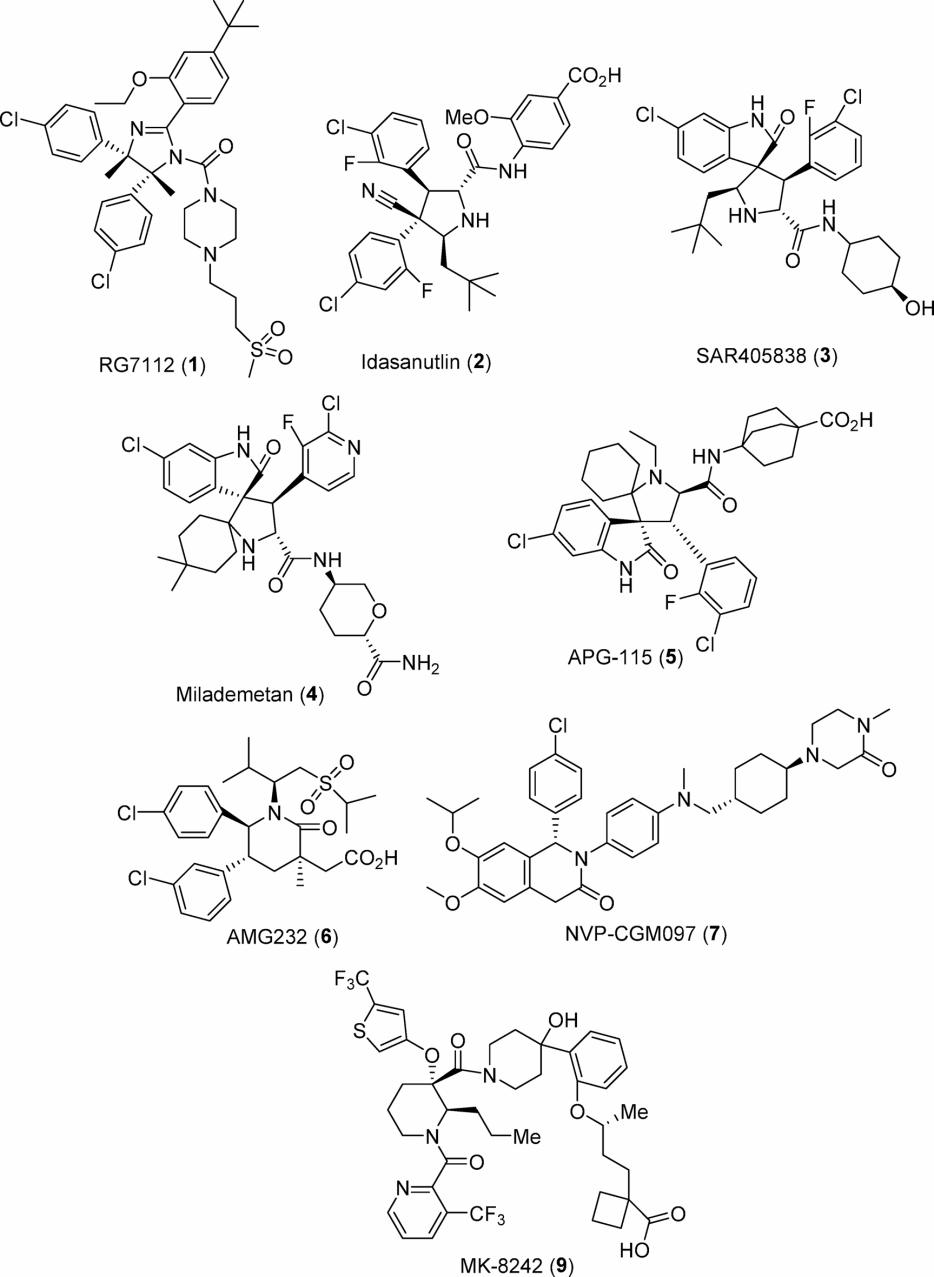
MDM2 inhibitors in clinical trials.

Cancer progression and development can be inhibited by p53 (guardian of the genome), which is a tumor suppressor that acts by suppressing cells with mutated DNA. It is believed that in 50% of cancers, the gene encoding p53 is mutated, and so the function of the p53 protein is lost. In the rest of the cancers, p53 is inhibited due to numerous biochemical mechanisms. For example, MDM2 (murine double minute 2) protein overexpression auto-regulates the p53 and can render it inactive. A possible therapeutic pathway for combating many cancer types is therefore interference with the protein-protein interaction between MDM2 and p53 to restore the activity of p53. Several small molecules have been identified as effective agents towards inhibition of co-crystallization of MDM2 with p53 (Fig. 1). Although many of them revealed satisfactory observations in clinical trials, none has been awarded approval for clinical practice^24,25^. Some piperidone analogs have been attributed with activity towards p53 making them potential therapeutics against cancer^26–32^ and hence their incorporation in the current design.

Topoisomerases are enzymes that can alter the topological structure of DNA by either single strand breaking (topoisomerase I) or double strand breaking (topoisomerase II). These changes lead to unwinding DNA double helix (solving the DNA supercoiling), which is a crucial step in separation the DNA double strands necessary during many biological processes, including cellular replication. Their primary role involves breaking and rejoining either single or double DNA strands for strand separation and release of DNA torsion strain caused by supercoiling. Generally, topoisomerases are essential enzymes for vital cellular functions including replication, transcription, and maintenance of genome stability^33–35^. This makes the topoisomerase inhibitors promising agents against many diseases, including cancer, which is among the deadliest threats to human life with limited treatment options often associated with serious side effects^36^.

**Fig. 2:**
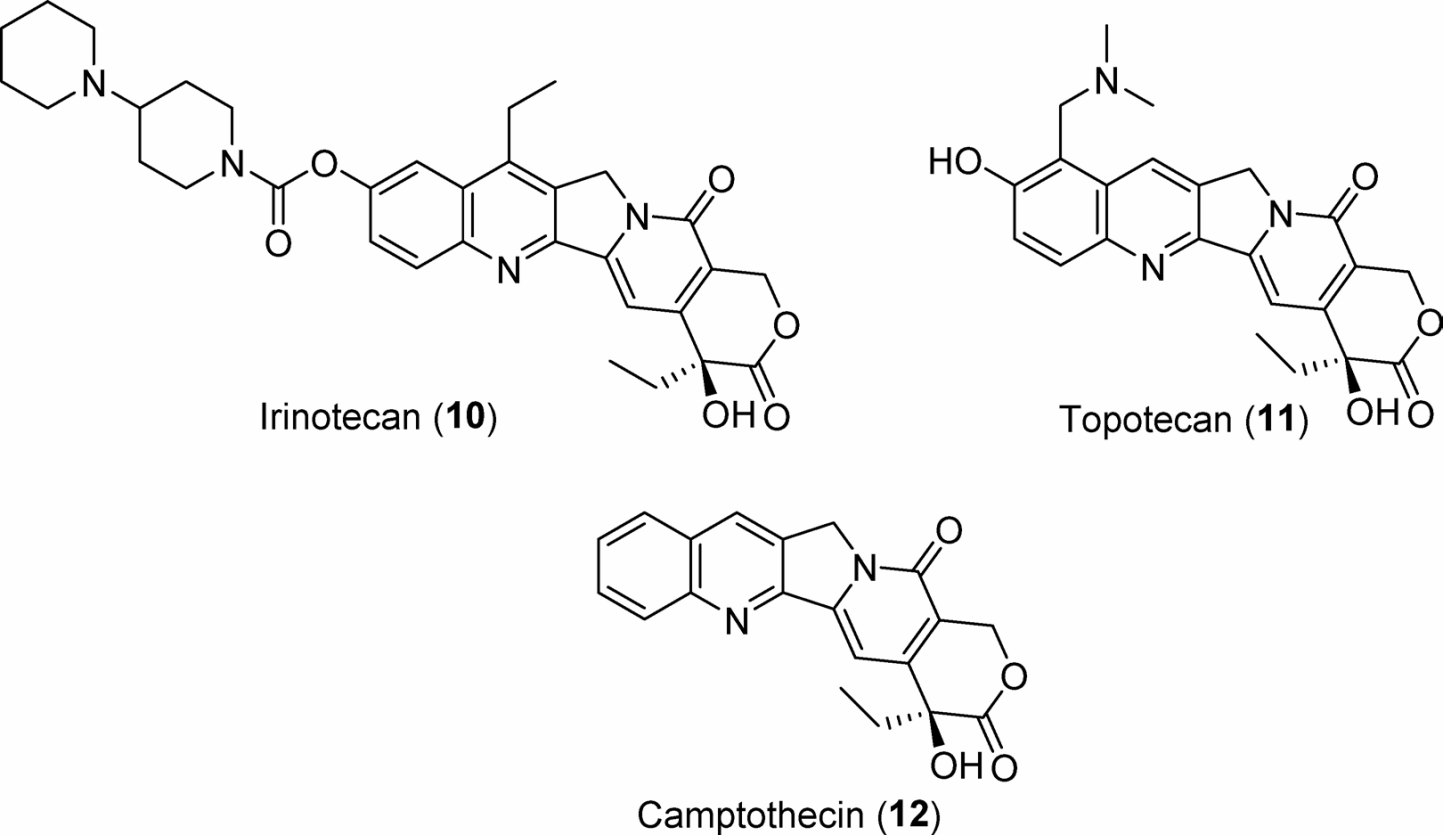
Topoisomerase I inhibitors.

**Fig. 3:**
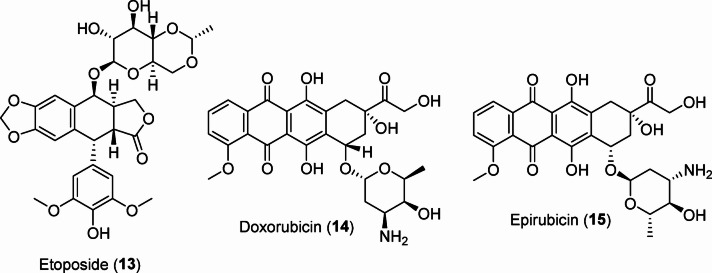
Therapeutic drugs with topoisomerase II inhibitory properties.

Some topoisomerase inhibitors have been identified, including irinotecan **10** (FDA approval in 1996 for colorectal and pancreatic cancers)^37^, topotecan **11** (FDA approved in 2014 for ovarian, small cell lung, and cervical cancers)^38,39^, and camptothecin **12**, which is a natural product isolated from the Chinese tree *Camptotheca acuminata*. Camptothecin showed promise in preliminary clinical trials but was not awarded drug approval due to the revealed adverse effects^40^ (Fig. 2). Other notable inhibitors include etoposide **13** (approved in USA in 1983 against small cell lung cancer)^41,42^, doxorubicin **14** (is a broad spectrum natural therapeutic isolated from the culture of *Streptomyces peucetius*, awarded FDA approval in 1974 against many types of cancers including breast, ovary, lung, lymphoma, sarcoma, and leukemia)^43,44^, and epirubicin **15** (used against breast cancer for patients had surgery for removing the tumor)^45,46^ (Fig. 3). These drugs target topoisomerases I and II, respectively (Figs. 2 and 3).

Topoisomerase I/II inhibitory properties have been observed for many 3,5-diylidene-4-piperidone analogs^47–50^. This is a key factor driving the investigating into the topoisomerase(s) inhibitory properties of the discovered antiproliferation agents. Therefore, the current work focuses on exploration of the antiproliferation properties of the newly synthesized 3,5-diylidene-4-piperidone-1-phosphonothioates and their ability to inhibit MDM2-p53 and topoisomerase I/II, aiming to identify novel multi-targeted inhibitory agent(s) with broad-spectrum antitumor activity and diverse mode of action.

## Results and discussion

### Chemical synthesis

**Fig. 4:**
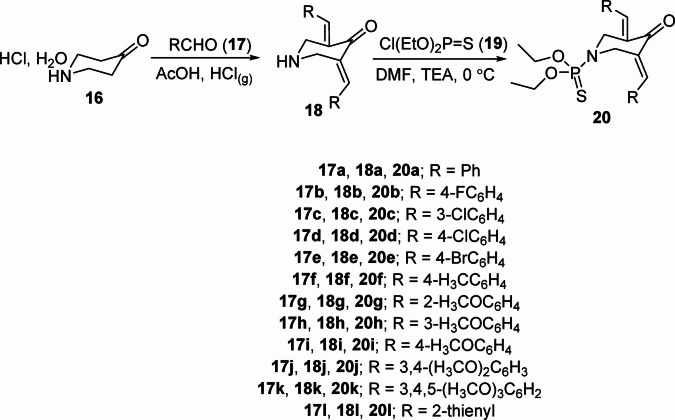
Synthesis of the targeted 3,5-bis(ylidene)-4-piperidone-1-phosphonothioates (**20a‒l**).

The starting 3,5-bis(ylidene)-4-piperidones **18a‒l** were prepared through condensation of 4-piperidone monohydrate hydrochloride **16** with the appropriate aromatic aldehyde **17a‒l** in glacial acetic acid with the acid of HCl “gas” as dehydrating agent^23^. Dehydrochlorination reaction of **18a‒l** with diethyl chlorothiophosphate **19** in *N*,*N*-dimethylformamide (DMF) containing quantitative amounts of triethylamine (TEA) at 0 °C, gave the corresponding 3,5-bis(ylidene)-4-piperidone-1-phosphonothioates **20a‒l** in high yields (70‒96%) (Fig. 4). Different spectroscopic techniques were used to assign the chemical structure of the synthesized agents. IR spectrum of **20a** (an example of the prepared analogs) reveals the unsaturated carbonyl as a strong band at *ν*_max_ = 1670 cm^− 1^. ^1^H-NMR spectrum of **20a** shows the ethoxy thiophosphate at *δ*_H_ = 1.04, 3.74‒3.85, in addition to the piperidinyl methylene protons at *δ*_H_ = 4.59, and 4.62 ppm. ^13^C-NMR spectrum of **20a** exhibits the ethoxy thiophosphate carbons at *δ*_C_ = 15.32, 15.38; 62.54, 62.58, and the piperidinyl methylene carbons at *δ*_C_ = 46.06, 46.09 ppm. The *E*-configuration in both the ylidene linkages was established through the singlet signal at *δ*_H_ = 7.72^46,47^. Mass spectrum (EI, 70 eV) of **20a** reveals its parent ion peak “*m/z* (%): 427 (M, 28)” (Supplementary Figs. S1‒S37). Single X-ray studies of **20a** and **20b** add conclusive evidence for the geometrical configuration.

### Crystal structures of 20a and 20b

**Fig. 5:**
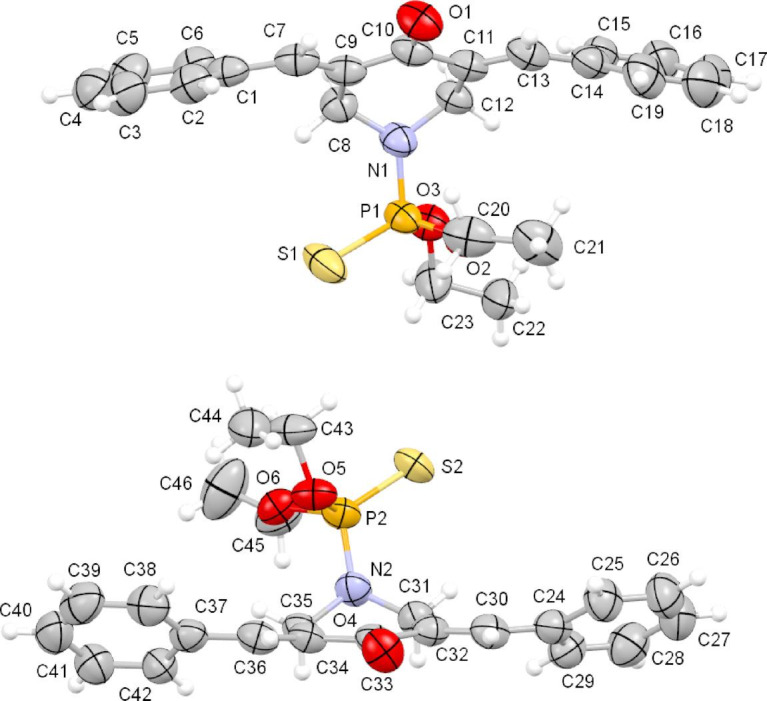
An ortep representation of the two independent molecules in the crystal structure of **20a** (molecules: top = M11, bottom = M12).

The asymmetric unit of the crystal structure of **20a** comprises two crystallographically independent molecules (M11 and M12, as shown in Fig. 5). Each independent molecule contains two ylidene groups [(C7‒C9) and (C11‒C13) in M11, (C30‒C32) and (C34‒C36) in M12]. Each molecule in **20a** has a piperidinone group namely (C8‒C12, N1, O1) in M11, and (C31–C35, N2, O4) in M12. Each molecule also has a diethyl thiophosphate group [(C20–C23, O2, O3, P1, S1) in M11, and (C43–C46, O5, O6, P2, S2) in M12]. Both piperidinone groups are planar, apart from the nitrogen atoms, which are displaced by 0.66 Å from the plane through the rest of the atoms. The diethyl thiophosphate groups are bonded axial to the piperidinone rings as observed in, for example, (3*E*,5*E*)-3,5-dibenzylidene-1-[3-(piperidin-1-yl)propanoyl]piperidin-4-one^51^ which also has the benzylidene-piperidinone-benzylidene system. The two independent molecules differ slightly in conformation as illustrated by benzylidene-piperidinone-benzylidene twist angles of 35.71(13)° and 39.41 (13)° for M11 and twist angles 33.00(15)° and 41.61(11)° for M12.

**Fig. 6:**
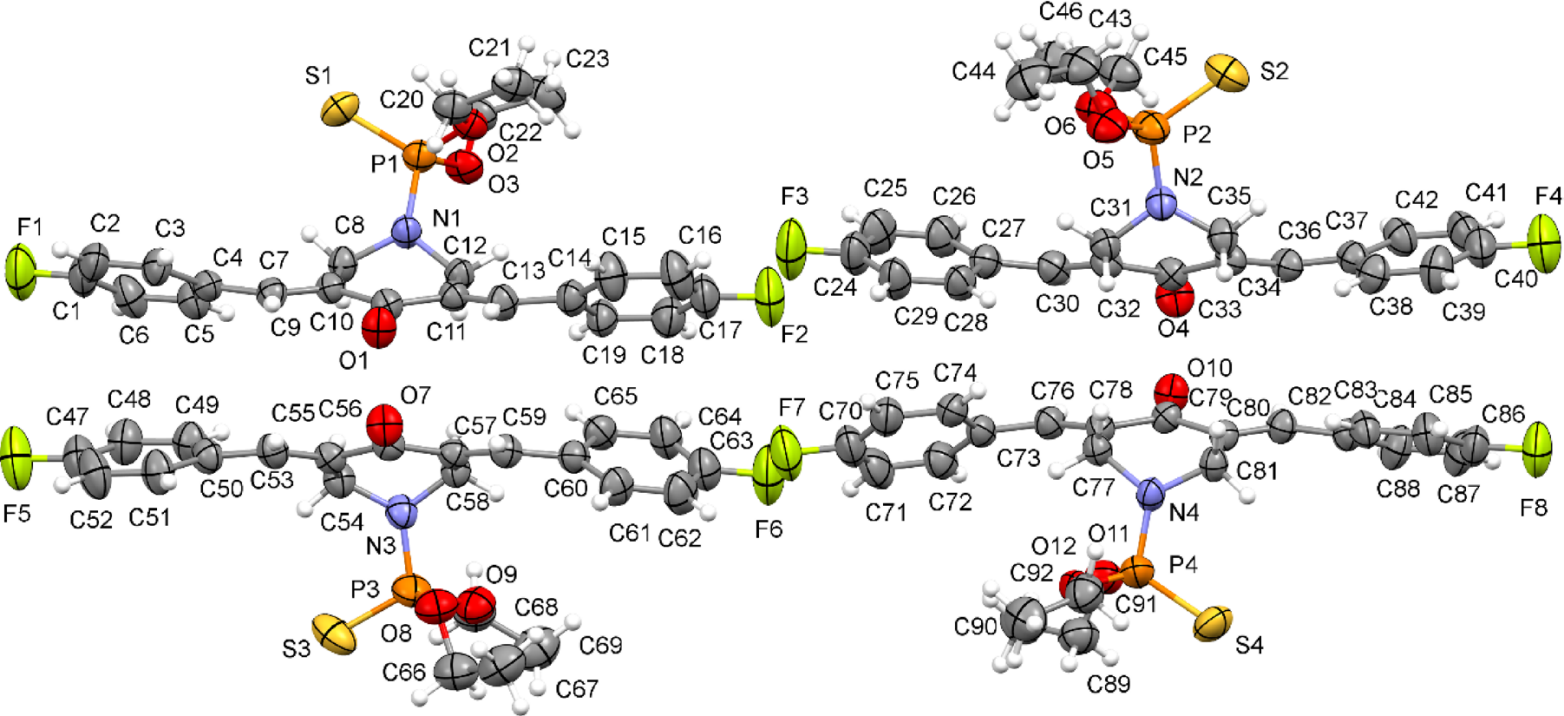
An ortep representation of the four independent molecules in the crystal structure of **20b** (molecules: top left = M21, top right = M22, bottom left = M23, bottom right = M24).

The asymmetric unit of the crystal structure of **20b** comprises four crystallographically independent molecules (M21, M22, M23, and M24, as shown in Fig. 6). Each independent molecule contains two ylidene groups [C7‒C9) and (C11‒C13) in M21, (C30‒C32) and (C34‒C36) in M22, (C53‒C55) and (C57‒C59) in M23, (C76‒C78), and (C80‒C82) in M24]. There are two fluorophenyl groups in each independent molecule; (C1–C6, F1), and (C14‒C19, F2) in M21; (C24‒C29, F3), and (C37–C42, F4) in M22; (C47‒C52, F5), and (C60‒C65, F6) in M23; and (C70‒C75, F7), and (C83‒C88, F8) in M24. Each molecule also has a piperidinone group [(C8‒C12, N1, O1) in M21, (C31–C35, N2, O4) in M22, (C54‒C58, N3, O7) in M23, and (C77‒C81, N4, O10) in M24]. and a diethyl thiophosphate group [(C20–C23, O2, O3, P1, S1) in M21, (C43–C46, O5, O6, P2, S2) in M22, (C66‒C69, O8, P3, S3) in M23, and (C89‒C92, O11, P4, S4) in M24]. All four piperidinone groups in **20b** are planar, apart from the nitrogen atoms, which are displaced by about 0.64 Å from the plane through the rest of the atoms of the group. The diethyl thiophosphate groups are all bonded axially to the piperidinone rings as observed in **20a**. The four independent molecules in **20b** have slightly different conformations with 4-(fluorobenzylidene)-piperidinone-4-(fluorobenzylidene) twist angles of 35.55(14)° and 41.43 (15)° for M21, 45.96(15)° and 36.61(15)° for M22, 31.69(14)° and 45.43(15)° for M23 and 48.90(13)° and 29.65(15)° for M24 (Supplementary Table S1).

### Antiproliferation properties

The antiproliferation properties for the synthesized 3,5-bis(ylidene)-4-piperidone-1-phosphonothioates (**20a‒l**) were assessed against cancer (MCF7, HCT116, A431; breast, colon, and skin/squamous, respectively) and normal (RPE1, retinal pigment epithelium) cell lines by the standard MTT technique^52^. The clinically approved anticancer drugs, 5-fluorouracil and sunitinib^53–56^, were used as standard references for comparison (Table 1, Supplementary Figs. S38‒S41).

### MCF7

Most of the synthesized 4-piperidone-1-thiophosphonates exhibit efficacy against MCF7 with higher potency than that of the two anticancer drugs tested. Compound **20c** (R = 3-ClC_6_H_4_) is the most effective agent discovered, with a sub-micromolar value, against MCF7 (IC_50_ = 0.65). The effectiveness is 4.8- and 6.1-fold relative to the standard drugs used (3.15 and 3.97 µM for 5-fluorouracil and sunitinib, respectively). Analogs **20d**, **20e**, and **20k** [R = 4-ClC_6_H_4_, 4-BrC_6_H_4_, and 3,4,5-(MeO)_3_C_6_H_2_, respectively] also show promising potency (IC_50_ = 1.083‒1.273 µM) with compounds **20b**, **20h**, and **20j** [R = 4-FC_6_H_4_, 3-MeOC_6_H_4_, and 3,4-(MeO)_2_C_6_H_3_, respectively] close behind (IC_50_ = 1.482‒1.760 µM).

SARs (structure-activity relationships) based on the exhibited biological observations indicate that the synthesized analogs possessing electron-withdrawing substituted ylidenes (fluoro-, chloro-, and bromobenzylidene) have more enhanced anti-MCF7 properties than those with electron-donating residues (methyl-, and methoxybenzylidene). This is evident from comparison of the IC_50_ values for compounds **20b**, **20d**, and **20e** (1.760, 1.273, and 1.210 µM, respectively), with those for **20f**, and **20i** (2.289, and 11.270 µM, respectively). Higher electron-withdrawing power of the substituent linked to the benzylidene residue, enhances the antiproliferation properties, and so the potency of the synthesized agents with a halogen substituent is **20e** > **20d** > **20b** (i.e. F > Cl > Br).

In additionally, the position of the substituent at either ortho- or meta-position relative to the benzylidene linkage is more preferable than the para-position for enhanced anti-MCF7 properties as noted in the synthesized analogs **20c**, and **20d** (IC_50_ = 0.650, and 1.273 µM, respectively), and **20g**, **20h**, and **20i** (IC_50_ = 4.185, 1.583, and 11.270 µM, respectively). Generally, compounds possessing the ylidene of (un)substituted benzylidene is associated with greater anti-proliferation properties in comparison to the 2-thienylidene heterocycle.

### HCT116

The synthesized agents show higher anti-HCT116 properties than the standard reference drugs used, noting that compounds **20i**, and **20L** (R= 4-MeOC_6_H_4_, and 2-thienyl respectively) are exceptions. Compound **20c** (R = 3-ClC_6_H_4_) is the most potent anti-HCT116 of the compounds. The value for **20c** (IC_50_ = 1.445 µM) is 14- and 6.7-fold the values for the standard drugs 5-fluorouracil and sunitinib (20.43 and 9.67 µM respectively). The remaining synthesized analogs also exhibit significant anti-HCT116 properties with IC_50_ values in the range 1.460‒2.283 µM.

SARs for the tested analogs indicate that the halogenated benzylidene-containing compounds (i.e., those with an electron-withdrawing function) have higher anti-HCT116 activity than those containing an electron-donating function. Thus, compounds **20b**, **20d**, and **20e** have higher anti-HCT116 activity (IC_50_ = 1.617, 1.482, and 1.596 respectively) than **20f**, and **20i** (IC_50_ = 2.283, and > 25.000 µM, respectively). A similar observation is made for **20c**, and **20h** (IC_50_ = 1.445, and 1.738 µM, respectively).

Additionally, substituent location in the ortho- or meta- ring position leads to better anti-HCT116 properties than the para-substituted analogs, irrespective of whether the substituent is electron donating or withdrawing. This is illustrated by compounds **20c**, and **20d** (IC_50_ = 1.445, and 1.482 µM, respectively) and by compound **20g**, **20h**, and **20i** (IC_50_ = 2.251, 1.738, and > 25.000 µM, respectively). The same observation as for MCF7 relating to thienylidene-containing compounds exhibiting poor antiproliferation properties relative to those with benzylidene analogs also applies to anti-HCT116 properties.

### A431

Most of the prepared 4-piperidone-1-thiophosphonates have higher potency against the A431 cell line than 5-fluorouracil which is a clinically approved drug against skin cancer (with IC_50_ = 23.44 µM). Compound **20i**, and **20L** (R = 4-MeOC_6_H_4_, and 2-thienyl, respectively) are exceptions. The most potent analog is **20k** [R = 3,4,5-(MeO)_3_C_6_H_2_, IC_50_ = 1.097 µM] with a value 21-fold the standard drug. Compounds **20c‒20e**, **20g**, **20h**, and **20j** also display promising anti-A431 potency (IC_50_ = 1.572‒2.936 µM).

SARs relating to anti-A431 properties point towards the effect of para-halogen substituted benzylidene-containing compounds (compound **20b** is an exception), showing higher potency than analogs with para-electron donating benzylidenes (methyl or methoxy). This is generally observed for all the tested cancer cell lines, as illustrated by compounds **20d**, and **20e** (IC_50_ = 1.869, and 1.934, respectively) in comparison to **20f**, and **20L** (IC_50_ = 5.222, and > 25.000 µM, respectively). Also notable is the poor antiproliferation performance by the thenylidene-containing compound **20L** against all the tested cell lines.

**Table 1:** Antiproliferation properties of the tested compounds.

Compd.	IC_50_, (µM) ± SEM, (SI)*			
	MCF7	HCT116	A431	RPE1
**20a**	2.707 ± 0.165 (1.591)	2.087 ± 0.104 (2.064)	15.950 ± 0.452 (0.270)	4.307 ± 0.150
**20b**	1.760 ± 0.053 (4.206)	1.617 ± 0.055 (4.578)	5.360 ± 0.352 (1.381)	7.403 ± 0.189
**20c**	0.650 ± 0.029 (4.702)	1.445 ± 0.032 (2.115)	2.920 ± 0.204 (1.047)	3.056 ± 0.149
**20d**	1.273 ± 0.012 (1.930)	1.482 ± 0.057 (1.658)	1.869 ± 0.062 (1.315)	2.457 ± 0.163
**20e**	1.210 ± 0.024 (1.967)	1.596 ± 0.055 (1.491)	1.934 ± 0.016 (1.231)	2.380 ± 0.109
**20f**	2.289 ± 0.274 (> 10.922)	2.283 ± 0.031 (> 10.951)	5.222 ± 0.757 (> 4.787)	> 25.000 ± 1.006
**20 g**	4.185 ± 0.037 (2.459)	2.251 ± 0.032 (4.571)	2.609 ± 0.035 (3.944)	10.290 ± 0.193
**20 h**	1.583 ± 0.051 (2.008)	1.738 ± 0.114 (1.829)	2.936 ± 0.121 (1.082)	3.178 ± 0.096
**20i**	11.270 ± 0.022 (> 2.218)	> 25.000 ± 0.270 (---)	> 25.000 ± 0.900 (---)	> 25.000 ± 0.768
**20j**	1.482 ± 0.068 (1.064)	1.460 ± 0.024 (1.080)	1.572 ± 0.032 (1.003)	1.577 ± 0.066
**20k**	1.083 ± 0.008 (1.951)	1.938 ± 0.033 (1.090)	1.097 ± 0.031 (1.926)	2.113 ± 0.024
**20L**	> 25.000 ± 0.238 (---)	> 25.000 ± 0.239 (---)	> 25.000 ± 0.774 (---)	> 25.000 ± 1.134
**5-Fluorouracil** ^49^	3.15 ± 0.44	20.43 ± 1.99	23.44 ± 2.09	---
**Sunitinib** ^49^	3.97 ± 0.32	9.67 ± 0.22	---	---

*Selectivity index (SI) = IC_50_ of RPE1/IC_50_ of the cancer cell line.

### Biochemical/enzymatic inhibitory properties

The synthesized analogs showing significant antiproliferation activity were tested for their biochemical and enzymatic properties against MDM2, p53, and topoisomerase I/II in order to explore the multi-targeted effects of the agents in MCF7 (breast cancer cell). The % inhibition/activation values were determined for each agent utilizing the IC_50_ value observed against the MCF7 cell line (Table 1) and for doxorubicin (standard reference drug, at IC_50_ = 0.1016 µM).

### MDM2

**Fig. 7:**
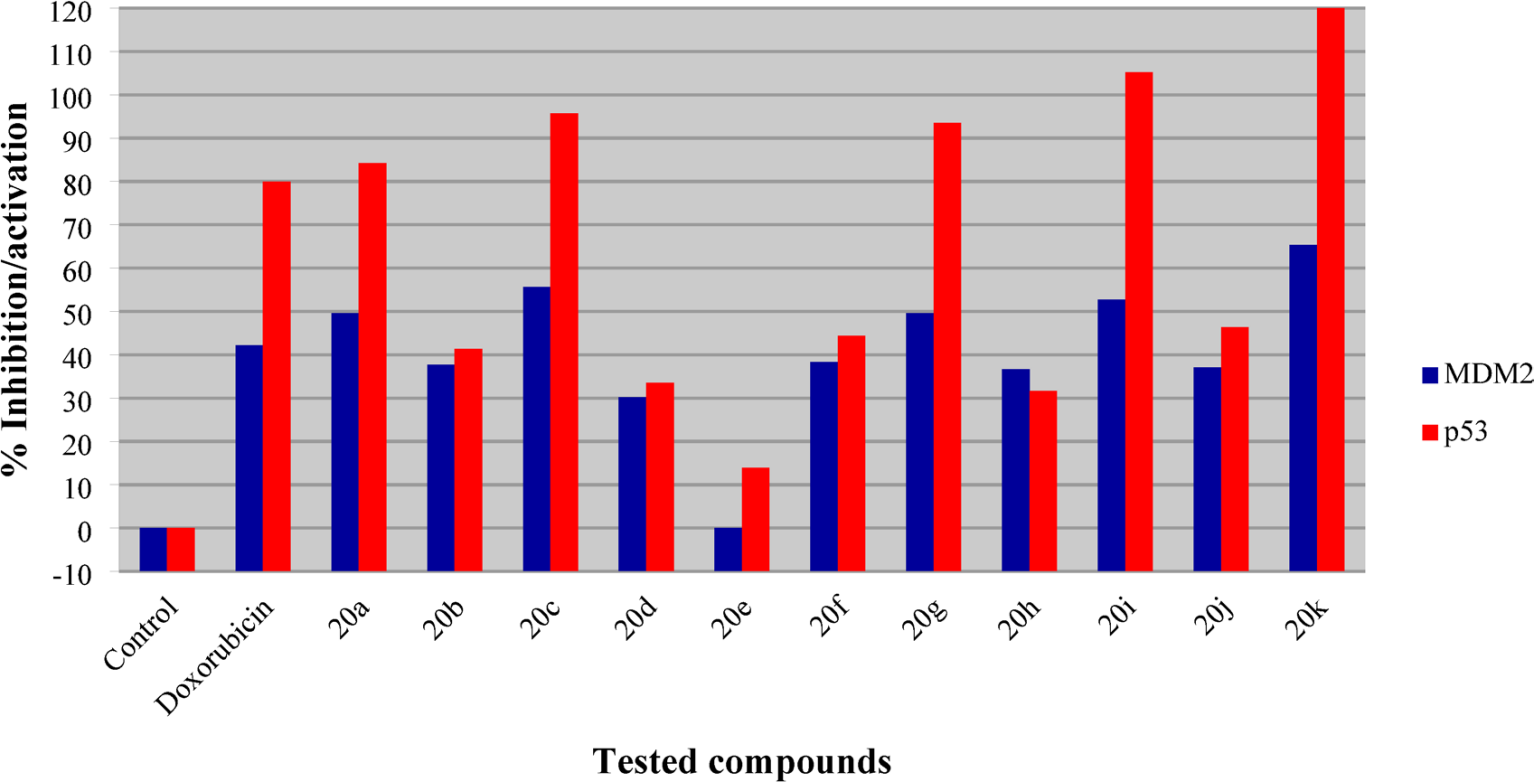
% Inhibition/activation of MDM2 and p53 by the synthesized agent **20a‒k**, and doxorubicin (standard reference).

Compounds with antiproliferation properties against MCF7 (**20a‒k**) were tested for their effects on MDM2 using a standard technique^57^. By utilizing the IC_50_ values, a fair comparison of analogs with different molecular weights was achieved. The tested agents demonstrated significant inhibitory effects on MDM2, exhibiting a range of efficacies. Notably, compound **20e** (R = 4-BrC_6_H_4_) showed no anti-MDM2 effect (refer to Table 2, and Fig. 7). In contrast, compound **20k** [R = 3,4,5-(MeO)_3_C_6_H_2_] emerged as the most effective anti-MDM2 agent, exhibiting a potency 1.55 times greater than the standard reference (% inhibition = 65.3 for **20k** compared to 42.2 for doxorubicin). Additionally, compounds **20c**, and **20i** demonstrated anti-MDM2 potencies that were also higher than the standard reference with % inhibition of 55.6, and 52.7, respectively. Compounds **20a**, and **20 g** showed promising but lower anti-MDM2 efficacy, each achieving a % inhibition of 49.6.

SAR analyses grounded in the identified biochemical properties suggested that para-substituted benzylidenes featuring electron-donating groups exhibit superior anti-MDM2 activity in comparison to those containing electron-withdrawing groups. This is exemplified by compounds **20f** and **20i**, which demonstrate % inhibition values of 38.3, and 52.7 respectively, in contrast to compounds **20b**, **20d**, and **20e**, which display % inhibition values of 37.7, 30.2, and 00.0, respectively. Furthermore, the enhancement of the anti-MDM2 effect correlates with the increasing the electron-withdrawing capability of the halogen substituents, following the order of F > Cl > Br. This trend elucidates the inhibition sequence of **20b** > **20d** > **20e**.

### p53

Investigation of the activation of p53 by the synthesized agents, with antiproliferation properties against MCF7 (**20a‒k**), was also undertaken by the standard technique utilizing the IC_50_ obtained against this tested cell line^58^. From the results (Table 2; Fig. 7) it is clear that some of the synthesized analogs have activation properties higher than that of the doxorubicin standard reference (which has % activation = 79.9). Compound **20k** [R = 3,4,5-(MeO)_3_C_6_H_2_] is the most promising of the compounds (% activation = 120.0) with a value 1.5-fold that of doxorubicin. Close behind are compound **20i** (R = 4-MeOC_6_H_4_, % activation = 105.2) as well as **20a**, **20c**, and **20g** with % activation values in the range 95.7‒84.2. Overall, the activation of p53 mirrors the observations against MDM2. This is consistent with p53 activation being mainly due to inhibition of the protein-protein MDM2-p53 interaction.

SARs noted from the exhibited biochemical observations are in accord with the anti-MDM2 properties of these compounds. The para-substituted benzylidenes with electron-donating groups have better p53 activation properties than those with electron-withdrawing groups as shown by **20f**, and **20i** (% activation = 44.4, and 105.2 respectively) compared with **20b**, **20d**, and **20e** (% activation = 41.3, 33.5, and 13.9, respectively). Additionally, the increment in electron-withdrawing power of the halogen atom attached to the benzylidene function is reflected in increased p53 activation as shown by compounds **20b**, **20d**, and **20e**.

**Table 2:** % Inhibition/activation of MDM2 and p53 of the synthesized compounds **20a‒k**, and doxorubicin (standard reference).

Compd.	MDM2		P53	
	Conc. (ng/ml) ± SD	% Inhibition	Conc. (pg/ml) ± SD	% Activation
**Control**	0.593 ± 0.006	---	97.660 ± 3.257	---
**Doxorubicin**	0.342 ± 0.013	42.2	175.733 ± 0.416	79.9
**20a**	0.299 ± 0.002	49.6	179.933 ± 2.550	84.2
**20b**	0.369 ± 0.004	37.7	137.967 ± 0.306	41.3
**20c**	0.263 ± 0.034	55.6	191.100 ± 2.722	95.7
**20d**	0.414 ± 0.006	30.2	130.400 ± 2.272	33.5
**20e**	0.593 ± 0.001	00.0	111.200 ± 3.081	13.9
**20f**	0.366 ± 0.003	38.3	141.067 ± 9.714	44.4
**20g**	0.299 ± 0.010	49.6	188.933 ± 2.967	93.5
**20h**	0.376 ± 0.023	36.6	128.533 ± 7.866	31.6
**20i**	0.280 ± 0.006	52.7	200.400 ± 5.507	105.2
**20j**	0.373 ± 0.005	37.1	142.867 ± 0.702	46.3
**20k**	0.206 ± 0.011	65.3	214.833 ± 3.539	120.0

### Topo-I

**Fig. 8:**
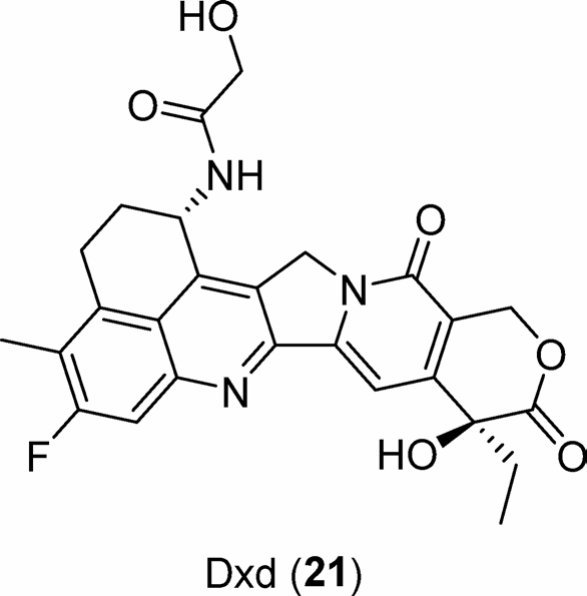
Dxd (**21**), potent topoisomerase-I (topo-I) inhibitor.

**Fig. 9:**
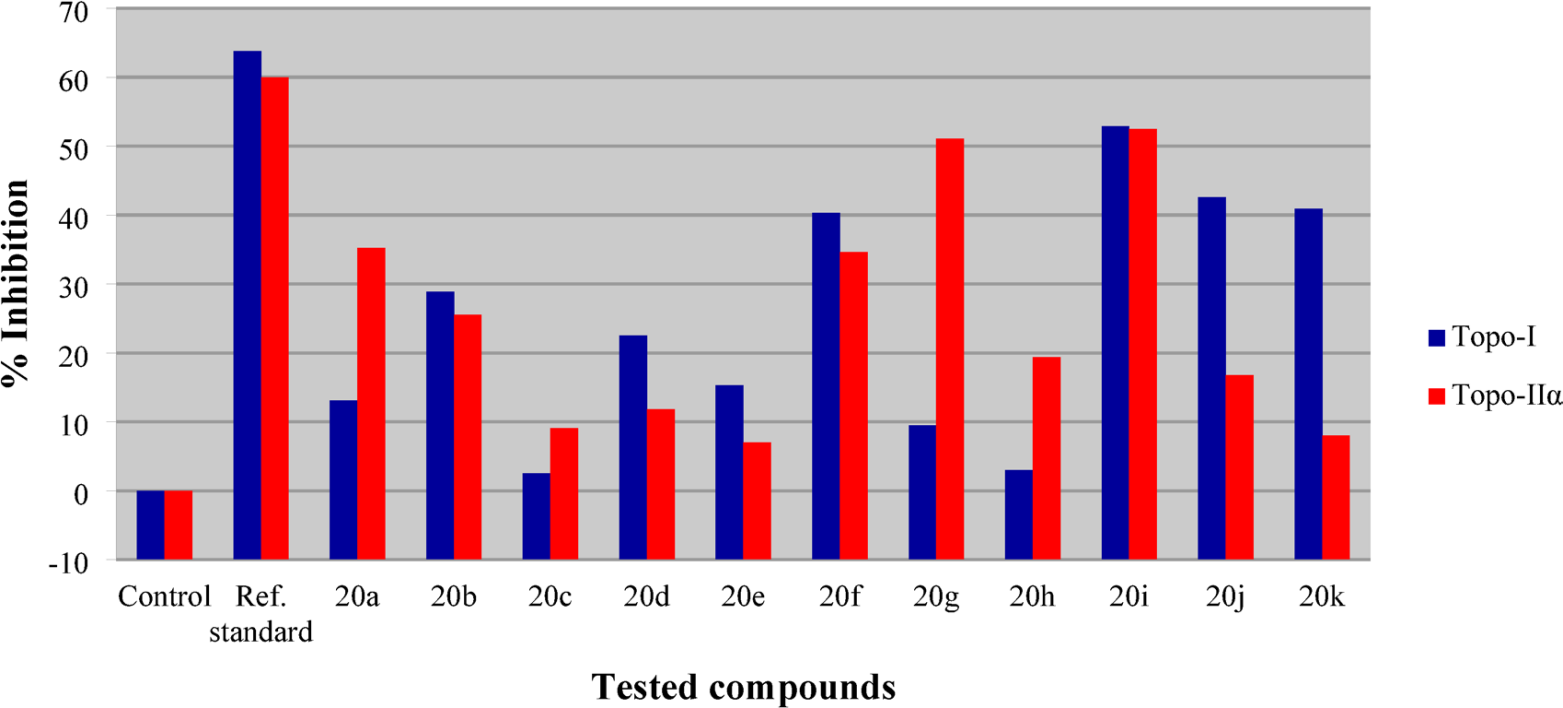
% Inhibition of topo-I/IIα by the synthesized agents **20a‒k**, and standard references (Dxd, and etoposide, respectively).

The antiproliferative active agents **20a‒k** were screened for anti-topoisomerase-I (topo-I) effects utilizing the observed IC_50_ against MCF7 and the results were compared with the reference standard Dxd **21** (Fig. 8) which is a potent topo-I inhibitor (IC_50_ = 0.40 µM)^59^. The standard technique was used^60^. The results show that some of the synthesized agents have considerable inhibitory properties relative to the standard (Table 3; Fig. 9). Compound **20i** (R = 4-MeOC_6_H_4_) is the most promising agent tested with % inhibition of topo-I value 52.9 compared to 63.8 for Dxd. Compounds **20f**, **20j**, and **20k** [R = 4-MeC_6_H_4_, 3,4-(MeO)_2_C_6_H_3_, 3,4,5-(MeO)_3_C_6_H_2_, respectively] also show substantial but milder topo-I inhibitory properties, with % inhibition in the range 42.6‒40.3.

SARs extracted from the biochemical observations show that the benzylidene-containing compounds with electron-donating groups have higher topo-I inhibitory effects than those containing electron-withdrawing groups, as shown in compounds **20b**, **20d**, and **20e** (% inhibition = 28.9, 22.5 and 15.3, respectively) relative to **20f** and **20i** (% inhibition = 40.3, and 52.9, respectively). The same is also observed for the pair **20c** and **20h** (% inhibition = 2.5, and 3.0, respectively). The increment in the electron withdrawing power of the halogen atom attached to the benzylidene linkage (F > Cl > Br) is associated with enhancement of topo-I inhibitory properties as seen in **20b**, **20d**, and **20e**. The position of the substituent attached to the benzylidene linkage also influences the topo-I inhibitory properties. The para-substitution is generally preferable than the meta-substitution as shown by pair **20c**, and **20d** (% inhibition = 2.5, and 22.5, respectively) as well as **20h**, and **20i** (% inhibition = 3.0, and 52.9, respectively).

### Topo-IIα

Topoisomerase-IIα (topo-IIα) inhibitory properties were investigated by the standard technique^61^ utilizing etoposide **13** (IC_50_ = 59.2 µM) as a standard reference. Some of the synthesized agents reveal promising biochemical properties (Table 3; Fig. 9). Compound **20i** (R = 4-MeOC_6_H_4_) is the most effective against topo-IIα (% inhibition = 52.5 for **20i** and 60.0 for etoposide). Compound **20g** (R = 2-MeOC_6_H_4_) is close behind with a % inhibition of 51.1.

SARs based on the observed biochemical results are similar to those for topo-I inhibitory properties. The electron-withdrawing effect of the halogen atom on topo-IIα inhibitory properties is observed in the order of F > Cl > Br as noted in compounds **20b**, **20d**, and **20e** (% inhibition = 25.5, 11.8, and 7.0, respectively). Additionally, the para-substituted benzylidene compounds are better topo-IIα inhibitors than the meta-substituted analogs as noted for pairs **20d**, and **20c** (% inhibition = 11.8, and 9.1, respectively), as well as **20i**, and **20h** (% inhibition = 52.5, and 19.4, respectively). Moreover, compounds with benzylidene-containing electron-donating group have higher efficacies against topo-IIα than those with electron-withdrawing function, as illustrated by compounds **20b**, and **20f** with % inhibition values 25.5, and 34.6 respectively. It is also notable that some of the synthesized agents are more effective/selective towards topo-IIα than to topo-I as shown in compounds **20a**, **20g**, and **20h** (% inhibition = 13.1/35.2, 9.5/51.1 and 3.0/19.4 against topo-I/topo-IIα, respectively).

**Table 3:** % Inhibition of topo-I/IIα of the synthesized compounds **2oa‒k**, and reference standards (Dxd, and etoposide).

Compd.	Topo-I		Topo-IIα	
	Conc. (ng/ml) ± SD	% Inhibition	Conc. (ng/l) ± SD	% Inhibition
**Control**	0.624 ± 0.005	---	311.867 ± 5.325	---
Ref. standard*	0.226 ± 0.003	63.8	124.900 ± 4.011	60.0
**20a**	0.542 ± 0.004	13.1	202.000 ± 4.034	35.2
**20b**	0.444 ± 0.016	28.9	232.367 ± 7.447	25.5
**20c**	0.609 ± 0.016	2.5	283.487 ± 7.454	9.1
**20d**	0.484 ± 0.007	22.5	275 0.000 ± 7.281	11.8
**20e**	0.529 ± 0.006	15.3	290.000 ± 4.681	7.0
**20f**	0.373 ± 0.002	40.3	204.033 ± 1.501	34.6
**20g**	0.565 ± 0.007	9.5	152.433 ± 4.043	51.1
**20h**	0.607 ± 0.008	3.0	251.500 ± 4.677	19.4
**20i**	0.294 ± 0.022	52.9	148.233 ± 0.833	52.5
**20j**	0.358 ± 0.011	42.6	259.467 ± 5.498	16.8
**20k**	0.369 ± 0.011	40.9	286.917 ± 7.087	8.0

* Ref. standard of Topo-I/IIα are Dxd, etoposide, respectively.

### Molecular Docking studies

Molecular modeling is a branch of computational study that is increasingly gaining interest in medicinal chemistry studies^62^. The study of the synthesized agents **20a‒l** was performed using Discovery Studio 4.1 software (standard CDOCKER technique, utilizing PDB: 4OAS, resolution: 1.70 Å, RMS gradient: 0.09155)^26,63,64^. The docking results (Table 4, Supplementary Figs. S42, S43) indicate that all the tested agents are involved in interactions with the amino acids of the protein active site with varied docking score values ranged from ‒38.367 to ‒56.577 kcal mol^− 1^. Compound **20k** has the highest docking score affinity ‒56.577 kcal mol^− 1^, followed by **20j** at ‒54.749 kcal/mol and **20i** at ‒48.021 kcal/mol. These values are close to or better than the docking score of doxorubicin, which is ‒50.250 kcal/mol serving as a standard reference drug. Although the co-crystallized ligand achieved the most favorable score at ‒68.725 kcal/mol, several synthesized analogs exhibited comparable interaction profiles, indicating promising therapeutic potential. The molecular docking data align with the observed antiproliferative effects across all tested cell lines (MCF7, HCT116, and A431).

Hydrogen bonding has emerged as a critical factor in determining binding stability. A notable observation is the consistent interaction of ARG97 with the piperidinyl nitrogen, which occurs in six out of eleven active compounds (**20b–20d**, **20h–20j**). This is particularly significant, as ARG97 is the only residue involved in hydrogen bonding with the co-crystallized ligand, highlighting its central role in ligand recognition and stabilization. Interestingly, the most potent compounds, **20a** and **20k**, showed hydrogen bonding between the piperidinyl carbonyl group (C = O) and ARG97, suggesting an alternative yet equally effective mode of interaction. Compounds **20e** and **20f** formed hydrogen bonding interaction between the thiophosphonate P-O group with ARG97. Compound **20g** uniquely interacted via the methoxy oxygen of the benzylidene moiety. These variations in hydrogen bonding patterns exemplify the structural diversity of the analogs and their adaptive binding strategies.

All compounds exhibited a diverse range of non-bonding interactions, including π-cation and π-alkyl interactions, primarily involving the ylidene phenyl group along with residues such as GLN18, ARG97, and MET62. These interactions enhance electrostatic complementarity and provide hydrophobic stabilization. Additionally, π-sulfur and π-π interactions, observed in compounds like **20d**, **20f**, **20i**, and **20k**, engage with amino acid residues of the protein active site such as TYR67 and HIS96, contributing to the specificity and binding strength. Alkyl interactions also play a role, as the phosphonothioate methyl groups frequently interact with hydrophobic residues (VAL93, ILE61, LEU54, MET62), anchoring the ligands more securely within the active site. Collectively, these interactions optimize ligand fit and enhance the overall docking score, showcasing a well-balanced interplay of hydrophilic and hydrophobic forces.

The docking results align well with biochemical assays focused on MDM2, particularly in MCF7 cell lines. Compounds that exhibited strong docking scores and favorable interaction profiles also displayed significant antiproliferative activity. Any minor discrepancies between the computational predictions and experimental results can be attributed to variations in assay conditions, such as solvent effects, protein dynamics, and cellular uptake, which are not fully represented in docking simulations.

The SAR analysis emphasizes the importance of certain features: electron-rich functional groups (such as phosphonothioate and halogens) are crucial for hydrogen bonding and π-type interactions. Aromatic systems, like ylidene phenyl, contribute to π-stacking and hydrophobic interactions. Additionally, the strategic placement of donor and acceptor atoms is essential for engaging key residues such as ARG97 and GLN18. Compounds incorporating these characteristics, such as **20k**, **20j**, and **20i**, demonstrate superior binding affinity and biological activity, thereby validating the design strategy.

**Fig. 10:**
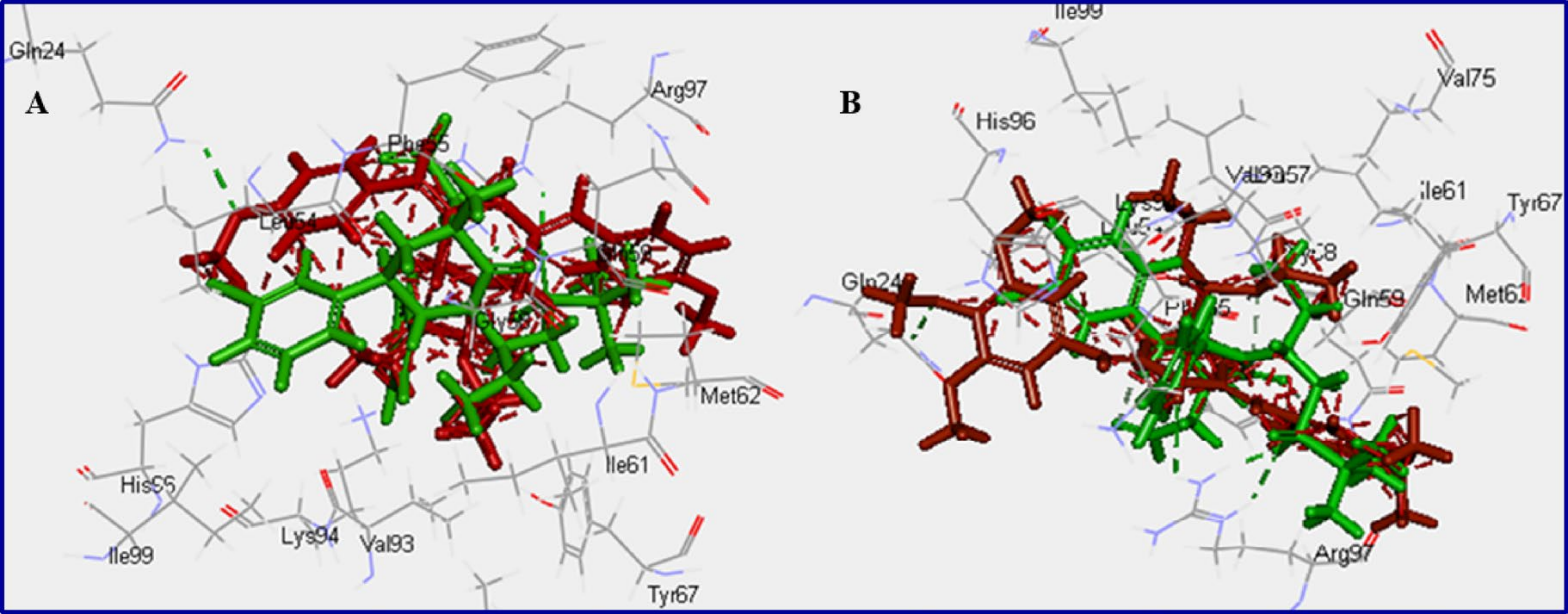
(**A**) Alignment of compound **20i** (deep red), and co-crystallized ligand (green); (**B**) Alignment of compound **20k** (deep red), and co-crystallized ligand (green).

Although the co-crystallized ligand demonstrates the highest binding potency, several synthesized compounds, specifically **20k**, **20j**, and **20i**, show docking scores that are either comparable to or exceed those of doxorubicin. This suggests their potential as alternative or complementary therapeutic agents. Additionally, the interaction profiles of these compounds closely resemble those of the reference ligands, highlighting their effective mimicry of essential binding characteristics (Fig. 10).

**Table 4:** CDOCKER interaction observations and energy scores (kcal mol^− 1^) of **20a‒l** in the active site of PDB ID: 4OAS.

Compd.	Docking score	Hydrogen bonding	Non-bonding interaction
**20a**	‒44.276	piperidinyl C = O … ARG97	*π-cation interaction*: ylidene phenyl …. GLN18 *π-alkyl & alkyl interactions*: ylidene phenyl … MET62, ylidene Ph … LEU54, phosphonothioate Me … MET62, phosphonothioate Me … TYR67, phosphonothioate Me … VAL93, phosphonothioate Me … ILE61, phosphonothioate Me … LEU57, phosphonothioate Me … LEU54
**20b**	‒43.651	piperidinyl N … ARG97, phosphonothioate P‒O … ARG97, F … GLN24	*π-cation interaction*: ylidene phenyl …. GLN18 *π-alkyl & alkyl interactions*: ylidene phenyl … MET62, ylidene Ph … ARG97, phosphonothioate Me … VAL93, phosphonothioate Me … ILE61, phosphonothioate Me … TYR67
**20c**	‒43.679	piperidinyl N … ARG97, phosphonothioate P‒O … ARG97	*π-cation interaction*: ylidene phenyl …. GLN18 *π-alkyl & alkyl interactions*: ylidene phenyl … ARG97, ylidene phenyl … MET62, ylidene Ph … LEU54, phosphonothioate Me … HIS96, phosphonothioate Me … TYR67
**20d**	‒45.147	piperidinyl N … ARG97	*π-σ interaction*: ylidene phenyl …. ARG97 *π-sulfur interaction*: phosphonothioate P = S … TYR67 *π-alkyl & alkyl interactions*: ylidene phenyl … LEU54, ylidene phenyl … MET62, Cl … ILE99, Cl … HIS96, Cl … MET62, phosphonothioate Me … HIS96, phosphonothioate Me … ILE61, phosphonothioate Me … VAL93, phosphonothioate Me … MET62
**20e**	‒43.935	phosphonothioate P‒O … ARG97, piperidinyl C = O … GLN18	*π-sulfur interaction*: phosphonothioate P = S … HIS96 *π-alkyl & alkyl interactions*: ylidene phenyl …ARG97, Br … ARG97, Br … MET62, Br … HIS96, phosphonothioate Me … ILE99, phosphonothioate Me … VAL93, phosphonothioate Me … MET62
**20f**	‒45.313	phosphonothioate P‒O … ARG97, piperidinyl N … ARG97	*π-cation interaction*: ylidenr Ph … ARG97 *π-σ interaction*: ylidene Ph … LEU54 *π-π interaction*: ylidene Ph … HIS96 *π-alkyl & alkyl interactions*: ylidene Me … LEU54, ylidene Me … ILE99, ylidene Me … MET62, ylidene Ph … MET62, phosphonothioate Me … TYR67, phosphonothioate Me … HIS96, phosphonothioate Me … VAL93, phosphonothioate Me … LYS94
**20g**	‒41.956	ylidene OMe … ARG97	*π-sulfur interaction*: ylidenr Ph … MET62 *π-alkyl & alkyl interactions*: ylidene Ph … VAL93, ylidene Ph … ILE61, phosphonothioate Me … LEU54, phosphonothioate Me … MET62, phosphonothioate Me … PHE55
**20h**	‒47.048	piperidinyl N … ARG97	*π-cation interaction*: ylidenr Ph … GLN18 *π-sulfur interaction*: ylidene Ph … MET62 *π-alkyl & alkyl interactions*: ylidene Ph … ARG97, phosphonothioate Me … ILE61, phosphonothioate Me … HIS96
**20i**	‒48.021	piperidinyl N … ARG97, ylidene OMe … GLN24	*π-sulfur interaction*: ylidene Ph … MET62, phosphonothioate P = S … TYR67 *π-alkyl & alkyl interactions*: ylidene Ph … ARG97, ylidene Ph … LEU54, phosphonothioate Me … VAL75, phosphonothioate Me … ILE61, phosphonothioate Me … VAL93, phosphonothioate Me … TYR67, phosphonothioate Me … HIS96
**20j**	‒54.749	piperidinyl N … ARG97	*π-alkyl & alkyl interactions*: ylidene Ph … ARG97, ylidene Ph … MET62, ylidene Ph … LEU54, phosphonothioate Me … VAL75, phosphonothioate Me … ILE61, phosphonothioate Me … VAL93, 2 phosphonothioate Me … TYR67
**20k**	‒56.577	piperidinyl C = O … ARG97, 2 ylidene OMe … GLN24	*π-carbon interaction*: ylidene Ph … ARG97 *π-sulfur interaction*: ylidene Ph … MET62 *π- π interaction*: ylidene Ph … HIS96 *π-alkyl & alkyl interactions*: ylidene Ph … LEU54, phosphonothioate Me … LEU54, phosphonothioate Me … ILE99, phosphonothioate Me … LEU57, phosphonothioate Me … ILE61, phosphonothioate Me … TYR67, phosphonothioate Me … VAL93, phosphonothioate Me … MET62
**20L**	‒38.367	phosphonothioate P = S … ARG97	*π-alkyl & alkyl interactions*: thienyl … ILE99, thienyl … LEU57, 2 phosphonothioate Me … VAL93, phosphonothioate Me … ILE61, phosphonothioate Me … MET62, phosphonothioate Me … TYR67, phosphonothioate Me … VAL75
Co-crystallized ligand	‒68.725	sulfonyl S = O … ARG97	*π-cation interaction*: carboxykic OH …. GLN18, phenyl … ARG97 *π-alkyl & alkyl interactions*: Cl … MET62, Cl … LEU57, Cl … ILE61, Cl … ILE99, alkyl Me … LEU54, alkyl Me … HIS96
Doxorubicin	‒50.250	OH … ARG97, C = O … GLN18, OMe … HIS96	*π-cation interaction*: NH_2_ … TYR67 *π-alkyl & alkyl interactions*: Me … LEU57, Me … LEU54

### Molecular dynamic studies

Molecular dynamic simulation is a computational technique that has gained continuous interest over the last decade(s) due its accessibility for assessing the stability of the docking interaction(s) taking place between a specific docked tested analog in the active site of an enzyme/protein. This process provides feedback on the potential for inhibition of biochemical pathways, thereby indicating the target agent’s efficacy and potency against the targeted disease. The RMSD and RMSF (root mean square deviation and fluctuation, respectively) are two important metrics commonly derived from molecular dynamic simulation studies that demonstrate the stability and flexibility of a docked agent over the course of simulation period^64^.

**Fig. 11:**
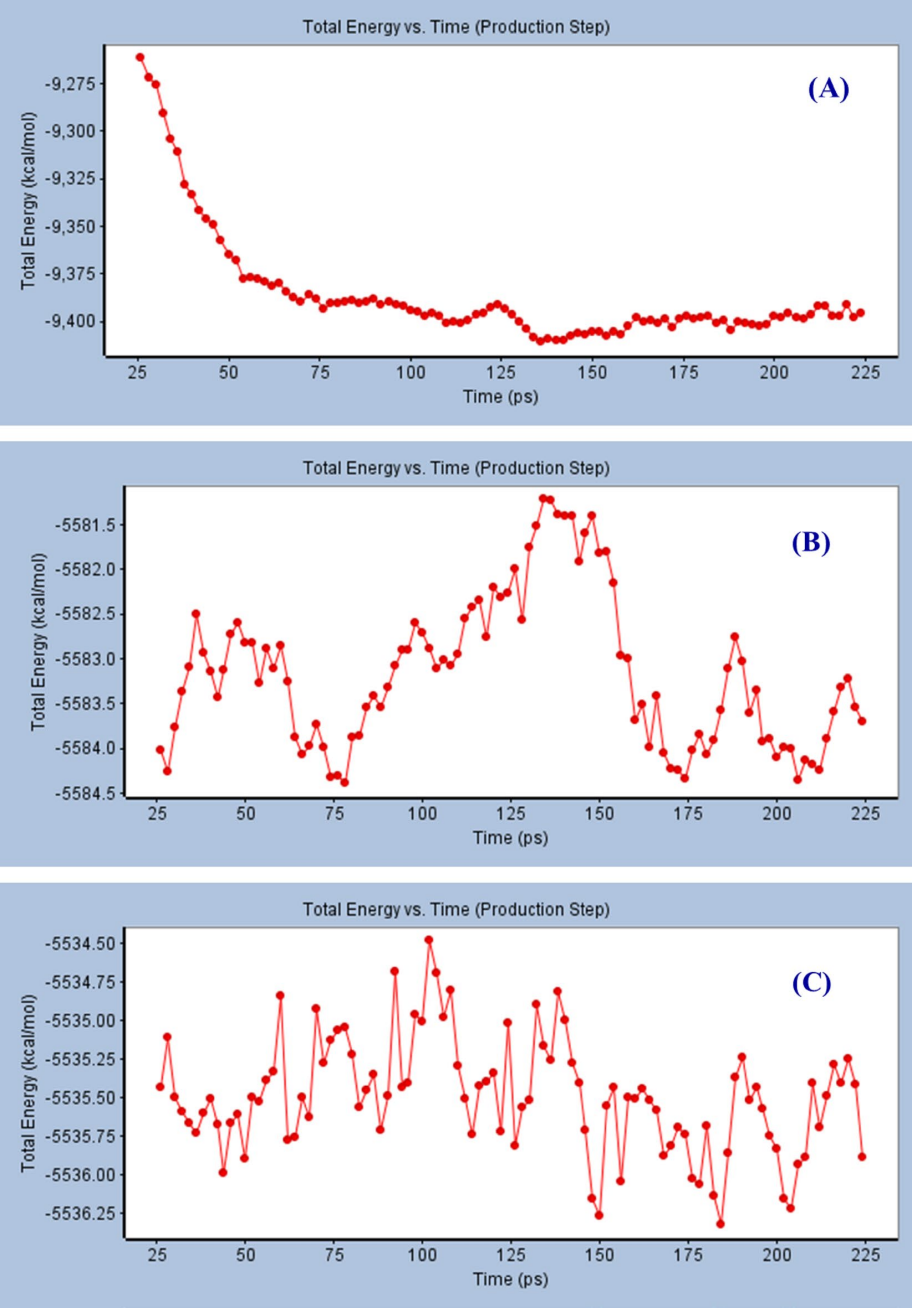
Total energy versus time in the production step during interaction of; (A): protein backbone of PDB: 4OAS, (B, and C): the best conformation poses of compounds **20i**, and **20k** respectively docked in the active site of PDB: 4OAS.

The total energy of the protein backbone during the production step of molecular dynamic studies, taking place by the standard technique (Discovery Studio 4.1) reveals a smooth descending within the simulation period 26‒110 *p*s (total energy = -9261.74 ‒ -9400.89 kcal mol^− 1^), followed by a slight increment till 124 *p*s (total energy = -9391.24). Finally, smooth stability of the total energy was shown with few fluctuations till the end of simulation period study (224 *p*s, total energy = -9395.87 kcal mol^− 1^) (Fig. 11, Supplementary Tables S2‒S4).

Molecular dynamic simulation studies of compound **20i** that, reveals promising docking score (‒48.021 kcal mol^− 1^), and efficacy against MDM2 (% inhibition = 52.9), exhibits narrow total energy range within the entire simulation period (-5581.22 ‒ -5584.39 i.e. 3.17 kcal mol^− 1^). An increment in energy was observed within the simulation time period (28‒36 *p*s, energy = -5584.26 ‒ -5582.51 kcal mol^− 1^), followed by energy depression at 78 *p*s (energy = -5584.39 kcal mol^− 1^). The maximum energy evaluation was shown at 134 *p*s (energy = -5581.22 kcal mol^− 1^).

Similar observations were also shown by compound **20k** that possesses the highest docking score (‒56.577) with potent anti-MDM2 properties (% inhibition = 65.3). Narrow total energy range was observed during the simulation period (energy = -5534.48 ‒ -5536.32 kcal mol^− 1^, i.e. 1.84 kcal mol^− 1^). The minimum energy level was shown at 184 *p*s while the minimum level was shown at 102 *p*s. The total energy narrow range behavior throughout the molecular simulation studies of compounds **20i** and **20k** is good evidence for high stability of the docking interaction taking place between the tested effective agents in the protein active site.

**Fig. 12:**
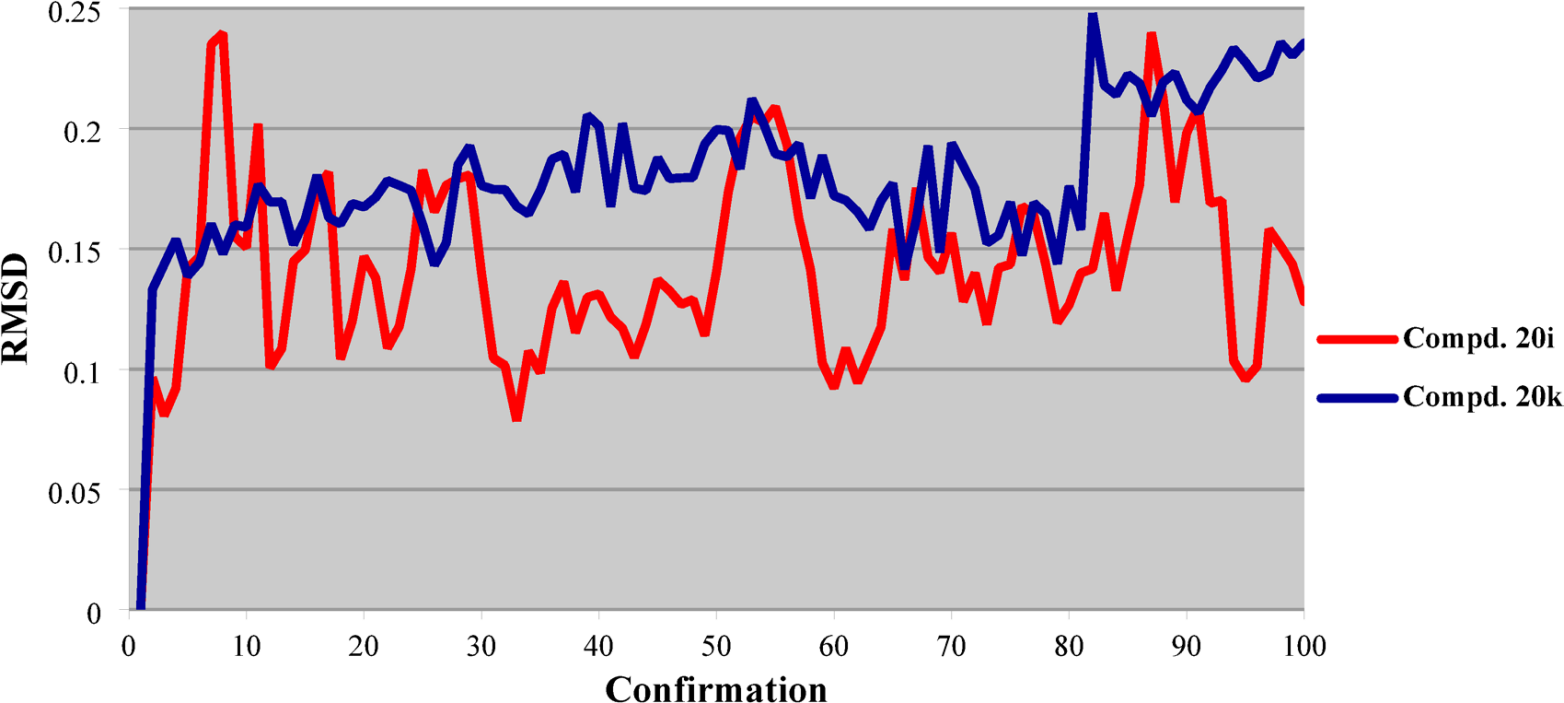
RMSD of the best conformation pose of compounds **20i**, and **20k** docked in the active site of PDB: 4OAS.

RMSD of compounds **20i** and **20k** is within a narrow range (0.079‒0.240, 0.133‒0.248 and averages = 0.142, 0.180, respectively). These observations are expected due to the narrow range of total energy revealed by these analogs within the production step. The highest RMSD for compounds **20i** and **20k** are shown at conformational steps 87, and 82, respectively (Fig. 12, supplementary Tables S5, and S6).

**Fig. 13:**
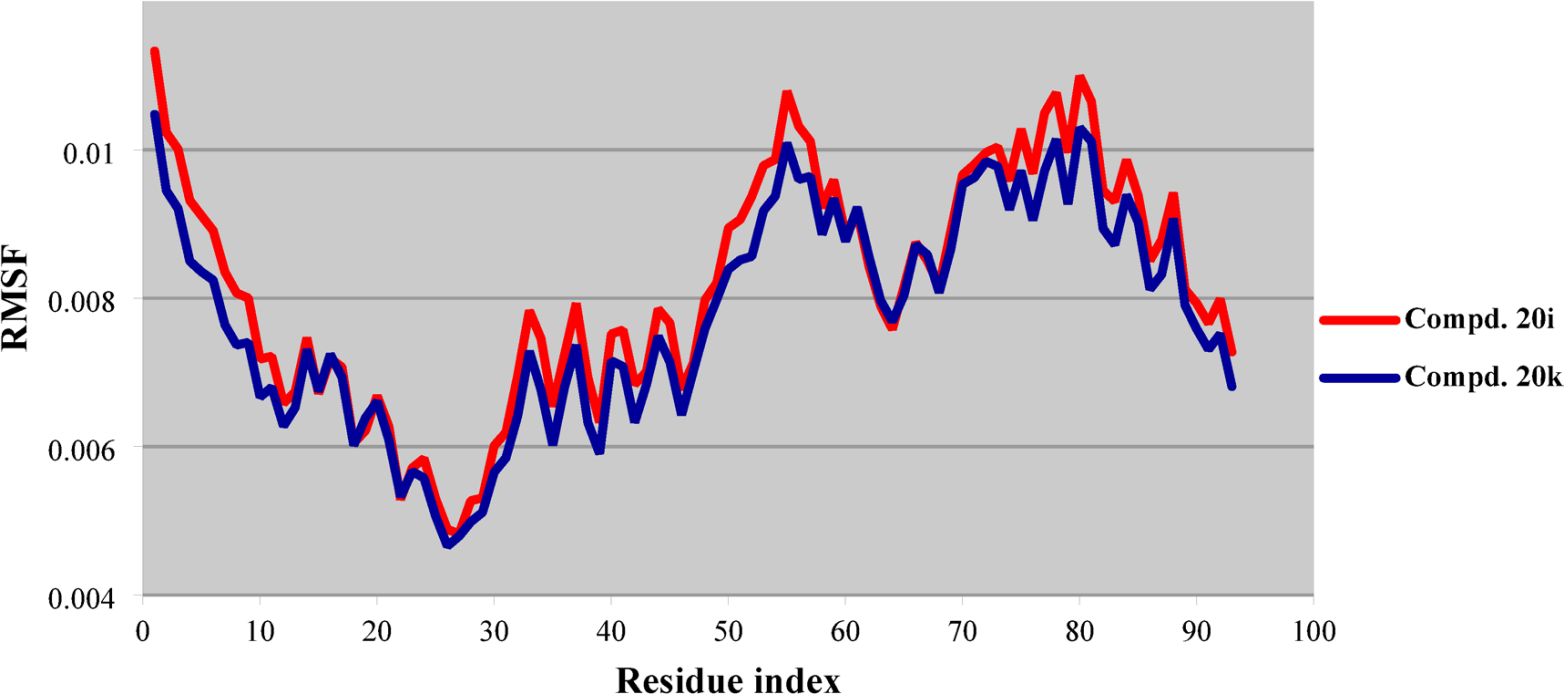
RMSF of the best conformation pose of compounds **20i**, and **20k** docked in the active site of PDB: 4OAS.

RMSF of the best poses due to the docked compounds **20i** and **20k** in the protein active site (PDB: 4OAS) also reveal similar observations to that exhibited by RMSD revealing narrow pattern (0.0048‒0.0113 “corresponding to GLN44, and GLN18” and 0.0047‒0.0105 “corresponding to ALA43, and GLN18”, respectively) (Fig. 13, Supplementary Tables S7, and S8). The most important amino acids (ARG97, and GLN24) revealing interactions with the docked agents **20i** and **20k** reveal RMSF values 0.0110, 0.0084, and 0.0103, 0.0076, respectively. All the above observations evidence the stability and robustness of the docking behavior of the tested compounds which is consistent with both docking scores and interactions with the amino acids of the protein active site.

## Conclusion

The findings of the study indicate that the targeted 3,5-bis(ylidene)-4-piperidone-1-phosphonothioates (**20a‒l**) can be synthesized with high yields ranging from 70% to 96%. This is achieved through a readily accessible dehydrochlorination reaction of 3,5-bis(ylidene)-4-piperidones (**18a‒l**) with diethyl chlorothiophosphate **19** in DMF containing TEA at 0 °C. Most of the targeted agents exhibit significant efficacy against MCF7 cells, demonstrating a higher potency than that of the standard reference drugs employed in the study. Notably, compound **20c** (R = 3-ClC_6_H_4_) emerged as the most effective agent against MCF7, with potency values 4.8- and 6.1-fold greater than those of the standard drugs (IC_50_ = 0.65 µM for **20c**, compared to 3.15 and 3.97 for 5-fluorouracil and sunitinib respectively). Additionally, several synthesized analogs exhibit high potency against HCT116, with **20c** maintaining its status as the most potent with values 14- and 6-, 7-fold greater than those of the standard drugs (IC_50_ = 1.445 for µM for **20c** versus to 20.43 and 9.67 for 5-fluorouracil and sunitinib respectively). Furthermore, some of the synthesized compounds demonstrate remarkable efficacy against A431. The most potent analog is **20k** [R = 3,4,5-(MeO)_3_C_6_H_2_] with an efficacy 21-fold superior to that of the standard drug (IC_50_ = 1.097 µM for **20k** and 23.44 µM for 5-fluorouracil). Compound **20k** also shows efficacy agent against MDM2 presenting a potency 1.55-fold higher than the standard reference used (% inhibition = 65.3 for **20k**, and 42.2 for doxorubicin). The results of biochemical activation testing of p53 align with the anti-MDM2 activity findings, indicating that p53 activation is primarily due to inhibition of protein-protein interaction between MDM2 and p53. Additionally, compound **20i** has the most topo-I and topo-IIα inhibitory effect of the compounds tested with potency close to that of the standard references used (Dxd, and etoposide). Interestingly, some of the synthesized agents (**20a**, **20g**, and **20h**) demonstrate selectivity towards topo-IIα relative to topo-I. MDM2 functions as a negative regulator of p53, a critical tumor suppressor protein that instrumental in the intrinsic (mitochondrial) pathway of apoptosis. Moreover, topoisomerase inhibitors (notably topo-I and topo-II) are known to induce DNA damage, serving as a potent trigger for p53-mediated intrinsic apoptosis. Based on the biochemical observation of the current study, it is evident that the compounds investigated suggest an involvement in the intrinsic pathway of apoptosis. Molecular docking observations (PDB: 4OAS) support the properties exhibited against MDM2. Based on the entirety of the biological and biochemical results, several synthesized compounds can be considered for nomination as multi-targeted inhibitors in line with the main goal of the study. Finally, the observed antiproliferation properties of the synthesized agents, especially the potent analogs, may result from the combined action of various enzymes involved in different mechanisms of action, including those examined in this research.

### Experimental

#### Chemical synthesis

Melting points were determined on a capillary melting point apparatus (Stuart SMP3) equipped with a digital thermometer. IR spectra (KBr) were recorded on a Shimadzu FT-IR 8400 S spectrophotometer. Reactions were monitored using thin layer chromatography (TLC) on 0.2 mm silica gel F254 plates (Merck) utilizing various solvents for elution. The chemical structures of the synthesized compounds were characterized by nuclear magnetic resonance spectra (^1^H-, ^13^C-NMR) and determined on Bruker and JEOL NMR spectrometers (500 MHz, 125 MHz for 1H and 13C, respectively). ^13^C NMR spectra are fully decoupled. Chemical shifts were reported in parts per million (ppm) using the deuterated solvent peak or tetramethylsilane as an internal standard. Mass spectra were recorded on a direct probe controller inlet part to single quadrppole mass analyzer in Thermo Scientific GCMS, model ISQ LT (EI, 70 eV). Supplementary Figs. S1‒S37 reveal the spectral illustrations of the synthesized agents.

#### Synthesis of O, O-diethyl [3,5-bis((E)-ylidene)-4-oxopiperidin-1-yl]phosphonothioates 20a‒l (general procedure)

To a stirring solution of 3,5-(bis(*E*)-ylidene)-4-piperidones **18a‒l** (2.5 mmol) in DMF (10 ml) containing TEA (0.4 ml, 3 mmol) in an ice bath (0 °C), diethyl chlorothiophosphate **19** (0.44 ml, 3 mmol) in DMF (10 ml) was added dropwise (within 10 min). The mixture was stirred at the same reaction conditions for additional 2 h and stored overnight at room temperature (20–25 °C). After the completion of the reaction (TLC), the reaction mixture was poured into ice-cold water (200 ml) containing NaCl (1.0 g). The separated solid was collected, washed with tap water, dried and crystallized from a suitable solvent affording the corresponding **20a‒l**.

#### *O*,*O*-Diethyl [3,5-di((*E*)-benzylidene)-4-oxopiperidin-1-yl]phosphonothioate (**20a**)

Obtained from the reaction of **18a** and **19**, as pale yellow microcrystals from methanol, mp 130–132 °C, and yield 96% (1.02 g). IR: *ν*_max_/cm^− 1^ 3082, 3024, 2978, 2932, 2897, 2859, 1670, 1612, 1585, 1485. ^1^H-NMR (DMSO-*d*_*6*_) *δ* (ppm): 1.04 (t, *J* = 7.0 Hz, 6 H, 2CH_3_), 3.74‒3.85 (m, 4 H, 2OCH_2_), 4.59 (s, 2 H, NCH_2_), 4.62 (s, 2 H, NCH_2_), 7.45‒7.52 (m, 10 H, arom. H), 7.72 (s, 2 H, 2 olefinic CH). ^13^C-NMR (DMSO-*d*_*6*_) *δ* (ppm): 15.32, 15.38 (CH_3_), 46.06, 46.09 (NCH_2_), 62.54, 62.58 (OCH_2_), 128.8, 129.4, 130.3, 132.92, 132.95, 134.3, 135.6 (arom. C + olefinic C), 185.8 (CO). MS, m/z (%): 427 (M, 28), 223 (100). Anal. Calcd. for C_23_H_26_NO_3_PS (427.50): C, 64.62; H, 6.13; N, 3.28. Found: C,64.41; H,6.04; N, 3.17.

#### *O*,*O*-Diethyl [3,5-bis((*E*)-4-fluorobenzylidene)-4-oxopiperidin-1-yl]phosphonothioate (**20b**)

Obtained from the reaction of **18b** and **19**, as yellow microcrystals from methanol, mp 96–97 °C, and yield 95% (1.10 g). IR: *ν*_max_/cm^− 1^ 2982, 2928, 2901, 2859, 1674, 1620, 1591, 1582, 1508. ^1^H-NMR (CDCl_3_) *δ* (ppm): 1.15 (t, *J* = 7.2 Hz, 6 H, 2CH_3_), 3.85‒3.97 (m, 4 H, 2OCH_2_), 4.53 (s, 2 H, NCH_2_), 4.55 (s, 2 H, NCH_2_), 7.13 (t, *J* = 8.6 Hz, 4 H, arom. H), 7.40 (dd, *J* = 5.2, 8.6 Hz, 4 H, arom. H), 7.77 (s, 2 H, 2 olefinic CH). ^13^C-NMR (CDCl_3_) *δ* (ppm): 15.7, 15.8 (CH_3_), 46.7 (NCH_2_), 63.4 (OCH_2_), 116.0, 116.2, 131.01, 131.04, 132.31, 132.35, 132.43, 132.5, 135.9, 162.2, 164.2 (arom. C + olefinic C), 186.8 (CO). MS, m/z (%): 463 (M, 13), 152 (100). Anal. Calcd. for C_23_H_24_F_2_NO_3_PS (463.48): C, 59.60; H, 5.22; N, 3.02. Found: C, 59.52; H, 5.12; N, 2.98.

#### *O*,*O*-Diethyl [3,5-bis((*E*)-3-chlorobenzylidene)-4-oxopiperidin-1-yl]phosphonothioate (**20c**)

Obtained from the reaction of **18c** and **19**, as yellow microcrystals from methanol, mp 126–128 °C, and yield 85% (1.05 g). IR: *ν*_max_/cm^− 1^ 2986, 2897, 2847, 1667, 1609, 1582, 1562, 1474. ^1^H-NMR (DMSO-*d*_*6*_) *δ* (ppm): 1.06 (t, *J* = 7.2 Hz, 6 H, 2CH_3_), 3.73‒3.86 (m, 4 H, 2OCH_2_), 4.56 (s, 2 H, NCH_2_), 4.58 (s, 2 H, NCH_2_), 7.45‒7.55 (m, 6 H, arom. H), 7.59 (s, 2 H, arom, H), 7.68 (s, 2 H, 2 olefinic CH). ^13^C-NMR (DMSO-*d*_*6*_) *δ* (ppm): 15.28, 15.34 (CH_3_), 45.85, 45.88 (NCH_2_), 62.60, 62.64 (OCH_2_), 128.6, 129.1, 129.7, 130.5, 133.4, 134.05, 134.08, 134.18, 136.4 (arom. C + olefinic C), 185.6 (CO). MS, m/z (%): 496 [(M + 1), 15], 498 (13), 86 (100). Anal. Calcd. for C_23_H_24_Cl_2_NO_3_PS (496.38): C, 55.65; H, 4.87; N, 2.82. Found: C, 55.84; H, 4.81; N, 2.69.

#### *O*,*O*-Diethyl [3,5-bis((*E*)-4-chlorobenzylidene)-4-oxopiperidin-1-yl]phosphonothioate (**20d**)

Obtained from the reaction of **18d** and **19**, as yellow microcrystals from methanol, mp 122–124 °C, and yield 87% (1.07 g). IR: *ν*_max_/cm^− 1^ 2982, 2932, 2897, 1670, 1612, 1585, 1562, 1489. ^1^H-NMR (DMSO-*d*_*6*_) *δ* (ppm): 1.05 (t, *J* = 7.2 Hz, 6 H, 2CH_3_), 3.75‒3.86 (m, 4 H, 2OCH_2_), 4.56 (s, 2 H, NCH_2_), 4.58 (s, 2 H, NCH_2_), 7.54 (d, *J* = 8.5 Hz, 4 H, arom. H), 7.57 (d, *J* = 8.4 Hz, 4 H, arom. H), 7.68 (s, 2 H, 2 olefinic CH). ^13^C-NMR (DMSO-*d*_*6*_) *δ* (ppm): 15.21, 15.25, 15.29, 15.36 (CH_3_), 45.89, 45.96, 45.99 (NCH_2_), 62.51, 62.58, 62.62 (OCH_2_), 128.7, 128.8, 131.9, 132.0, 133.02, 133.04, 133.1, 133.36, 133.43, 133.46, 134.05, 134.13, 134.2, 134.3 (arom. C + olefinic C), 185.6 (CO). MS, m/z (%): 495 (M, 14), 496 (63), 497 (21), 172 (100). Anal. Calcd. for C_23_H_24_Cl_2_NO_3_PS (496.38): C, 55.65; H, 4.87; N, 2.82. Found: C, 55.76; H, 4.94; N, 2.91.

#### *O*,*O*-Diethyl [3,5-bis((*E*)-4-bromobenzylidene)-4-oxopiperidin-1-yl]phosphonothioate (**20e**)

Obtained from the reaction of **18e** and **19**, as yellow microcrystals from methanol, mp 145–146 °C, and yield 85% (1.24 g). IR: *ν*_max_/cm^− 1^ 2982, 2932, 2897, 1670, 1612, 1585, 1558, 1485. ^1^H-NMR (DMSO-*d*_*6*_) *δ* (ppm): 1.05 (t, *J* = 7.1 Hz, 6 H, 2CH_3_), 3.72‒3.87 (m, 4 H, 2OCH_2_), 4.55 (s, 2 H, NCH_2_), 4.57 (s, 2 H, NCH_2_), 7.46 (d, *J* = 8.0 Hz, 4 H, arom. H), 7.66 (s, 2 H, 2 olefinic CH), 7.70 (d, *J* = 8.1 Hz, 4 H, arom. H). ^13^C-NMR (DMSO-*d*_*6*_) *δ* (ppm): 15.3, 15.4 (CH_3_), 45.98, 46.01 (NCH_2_), 62.60, 62.63 (OCH_2_), 123.0, 131.8, 132.2, 133.47, 133.52, 134.4 (arom. C + olefinic C), 185.7 (CO). MS, m/z (%): 582 (M, 33), 585 (53), 586 (27), 587 (43), 305 (100). Anal. Calcd. for C_23_H_24_Br_2_NO_3_PS (585.29): C, 47.20; H, 4.13; N, 2.39. Found: C, 47.07; H, 3.99; N, 2.33.

#### *O*,*O*-Diethyl [3,5-bis((*E*)-4-methylbenzylidene)-4-oxopiperidin-1-yl]phosphonothioate (**20f**)

Obtained from the reaction of **18f** and **19**, as yellow microcrystals from methanol, mp 160–161 °C, and yield 75% (0.85 g). IR: *ν*_max_/cm^− 1^ 2978, 2924, 2897, 2859, 1670, 1609, 1582, 1562, 1508. ^1^H-NMR (DMSO-*d*_*6*_) *δ* (ppm): 1.05 (t, *J* = 7.1 Hz, 6 H, 2CH_3_), 2.37 (s, 6 H, 2ArCH_3_), 3.74‒3.85 (m, 4 H, 2OCH_2_), 4.58 (s, 2 H, NCH_2_), 4.60 (s, 2 H, NCH_2_), 7.32 (d, *J* = 7.8 Hz, 4 H, arom. H), 7.41 (d, *J* = 7.8 Hz, 4 H, arom. H), 7.67 (s, 2 H, 2 olefinic CH). ^13^C-NMR (DMSO-*d*_*6*_) *δ* (ppm): 15.3, 15.4 (CH_3_), 20.9 (ArCH_3_), 46.13, 46.16 (NCH_2_), 62.49, 62.53 (OCH_2_), 129.4, 130.4, 131.6, 132.15, 132.18, 135.6, 139.4 (arom. C + olefinic C), 185.7 (CO). MS, m/z (%): 455 (M, 99), 227 (100). Anal. Calcd. for C_25_H_30_NO_3_PS (455.55): C, 65.91; H, 6.64; N, 3.07. Found: C, 65.98; H, 6.75; N, 3.15.

#### *O*,*O*-Diethyl [3,5-bis((*E*)-2-methoxybenzylidene)-4-oxopiperidin-1-yl]phosphonothioate (**20g**)

Obtained from the reaction of **18g** and **19**, as yellow microcrystals from methanol, mp 114–115 °C, and yield 70% (0.85 g). IR: *ν*_max_/cm^− 1^ 2982, 2940, 2901, 2835, 1659, 1601, 1566, 1485. ^1^H-NMR (DMSO-*d*_*6*_) *δ* (ppm): 1.03 (t, *J* = 6.9 Hz, 6 H, 2CH_3_), 3.73‒3.86 (m, 10 H, 2OCH_2_ + 2OCH_3_), 4.46 (s, 2 H, NCH_2_), 4.48 (s, 2 H, NCH_2_), 7.06 (t, *J* = 7.6 Hz, 2 H, arom. H), 7.13 (d, *J* = 8.3 Hz, 2 H, arom. H), 7.29 (d, *J* = 7.5 Hz, 2 H, arom. H), 7.44 (t, *J* = 8.0 Hz, 2 H, arom. H), 7.87 (s, 2 H, 2 olefinic CH). ^13^C-NMR (DMSO-*d*_*6*_) *δ* (ppm): 15.18, 15.24 (CH_3_), 46.10, 46.13 (NCH_2_), 55.6 (OCH_3_), 62.41, 62.45 (OCH_2_), 111,4, 120.0, 120.2, 122.9, 130.1, 131.24, 131.26, 132.50, 132.53, 158.0 (arom. C + olefinic C), 185.8 (CO). MS, m/z (%): 487 (M, 56), 195 (100). Anal. Calcd. for C_25_H_30_NO_5_PS (487.55): C, 61.59; H, 6.20; N, 2.87. Found: C, 61.71; H, 6.25; N, 2.98.

#### *O*,*O*-Diethyl [3,5-bis((*E*)-3-methoxybenzylidene)-4-oxopiperidin-1-yl]phosphonothioate (**20h**)

Obtained from the reaction of **18h** and **19**, as yellow microcrystals from methanol, mp 112–113 °C, and yield 95% (1.15 g). IR: *ν*_max_/cm^− 1^ 2978, 2936, 2893, 2835, 1674, 1610, 1574, 1489. ^1^H-NMR (DMSO-*d*_*6*_) *δ* (ppm):1.06 (t, *J* = 6.7 Hz, 6 H, 2CH_3_), 3.75‒3.88 (m, 10 H, 2OCH_2_ + 2OCH_3_), 4.59 (s, 2 H, NCH_2_), 4.61 (s, 2 H, NCH_2_), 7.03‒7.08 (m, 6 H, arom. H), 7.42 (t, *J* = 8.2 Hz, 2 H, arom. H), 7.69 (s, 2 H, 2 olefinic CH). ^13^C-NMR (DMSO-*d*_*6*_) *δ* (ppm): 15.32, 15.38 (CH_3_), 46.03, 46.06 (NCH_2_), 55.2 (OCH_3_), 62.54, 62.58 (OCH_2_), 115.2, 115.6, 122.4, 129.8, 133.08, 133.12, 135.60, 135.63, 159.3 (arom. C + olefinic C), 185.8 (CO). MS, m/z (%): 487 (M, 20), 85 (100). Anal. Calcd. for C_25_H_30_NO_5_PS (487.55): C, 61.59; H, 6.20; N, 2.87. Found: C, 61.64; H, 6.26; N, 2.94.

#### *O*,*O*-Diethyl [3,5-bis((*E*)-4-methoxybenzylidene)-4-oxopiperidin-1-yl]phosphonothioate (**20i**)

Obtained from the reaction of **18i** and **19**, as yellow microcrystals from methanol, mp 157–158 °C, and yield 86% (1.04 g). IR: *ν*_max_/cm^− 1^ 2982, 2932, 2901, 2835, 1670, 1605, 1582, 1512. ^1^H-NMR (DMSO-*d*_*6*_) *δ* (ppm): 1.07 (t, *J* = 7.2 Hz, 6 H, 2CH_3_), 3.73‒3.88 (m, 10 H, 2OCH_2_ + 2OCH_3_), 4.58 (s, 2 H, NCH_2_), 4.60 (s, 2 H, NCH_2_), 7.07 (d, *J* = 6.4 Hz, 4 H, arom. H), 7.48 (d, *J* = 6.8 Hz, 4 H, arom. H), 7.66 (s, 2 H, 2 olefinic CH). ^13^C-NMR (DMSO-*d*_*6*_) *δ* (ppm): 15.3, 15.4 (CH_3_), 46.12, 46.15 (NCH_2_), 55.3 (OCH_3_), 62.48, 62.51 (OCH_2_), 114.4, 126.9, 130.87, 130.90, 132.3, 135.2, 160.2 (arom. C + olefinic C), 185.5 (CO). MS, m/z (%): 487 (M, 24), 157 (100). Anal. Calcd. for C_25_H_30_NO_5_PS (487.55): C, 61.59; H, 6.20; N, 2.87. Found: C, 61.47; H, 6.23; N, 3.01.

#### *O*,*O*-Diethyl [3,5-bis((*E*)-3,4-dimethoxybenzylidene)-4-oxopiperidin-1-yl]phosphonothioate (**20j**)

Obtained from the reaction of **18j** and **19**, as yellow microcrystals from methanol, mp 146–148 °C, and yield 93% (1.27 g). IR: *ν*_max_/cm^− 1^ 2932, 2839, 1670, 1597, 1578, 1512. ^1^H-NMR (DMSO-*d*_*6*_) *δ* (ppm): 1.08 (t, *J* = 7.0 Hz, 6 H, 2CH_3_), 3.81‒3.83 (br d, 16 H, 2OCH_2_ + 4OCH_3_), 4.61 (s, 2 H, NCH_2_), 4.63 (s, 2 H, NCH_2_), 7.09‒7.13 (br d, 6 H, arom. H), 7.67 (s, 2 H, 2 olefinic CH). ^13^C-NMR (DMSO-*d*_*6*_) *δ* (ppm): 15.4, 15.5 (CH_3_), 46.1 (NCH_2_), 55.55, 55.58 (OCH_3_), 62.49, 62.53 (OCH_2_), 111.8, 114.2, 123.8, 127.1, 130.95, 130.99, 135.7, 148.6, 150.1 (arom. C + olefinic C), 185.4 (CO). MS, m/z (%): 547 (M, 30), 520 (100). Anal. Calcd. for C_27_H_34_NO_7_PS (547.60): C, 59.22; H, 6.26; N, 2.56. Found: C, 59.31; H, 6.32; N, 2.68.

#### *O*,*O*-Diethyl [4-oxo-3,5-bis((*E*)-3,4,5-trimethoxybenzylidene]piperidin-1-yl)phosphonothioate (**20k**)

Obtained from the reaction of **18k** and **19**, as yellow microcrystals from methanol, mp 150–151 °C, and yield 92% (1.39 g). IR: *ν*_max_/cm^− 1^ 2978, 2940, 2901, 2835, 1663, 1609, 1578, 1504. ^1^H-NMR (DMSO-*d*_*6*_) *δ* (ppm): 1.09 (t, *J* = 7.2 Hz, 6 H, 2CH_3_), 3.73 (s, 6 H, 2OCH_3_), 3.80‒3.89 (m, 16 H, 2OCH_2_ + 4OCH_3_), 4.65 (s, 2 H, NCH_2_), 4.68 (s, 2 H, NCH_2_), 6.84 (s, 4 H, arom. H), 7.67 (s, 2 H, 2 olefinic CH). ^13^C-NMR (DMSO-*d*_*6*_) *δ* (ppm): 15.37, 15.43 (CH_3_), 46.0 (NCH_2_), 56.1, 60.1 (OCH_3_), 62.54, 62.58 (OCH_2_), 108.2, 129.8, 132.1, 135.9, 138.8, 152.8 (arom. C + olefinic C), 185.5 (CO). MS, m/z (%): 607 (M, 17], 222 (100). Anal. Calcd. for C_29_H_38_NO_9_PS (607.65): C, 57.32; H, 6.30; N, 2.31. Found: C, 57.45; H, 6.44; N, 2.37.

#### *O*,*O*-Diethyl [(3*E*,5*E*)-4-oxo-3,5-bis(thiophen-2-ylmethylene)piperidin-1-yl]phosphonothioate (**20L**)

Obtained from the reaction of **18L** and **19**, as dark yellow microcrystals from methanol, mp 94–96 °C, and yield 88% (0.96 g). IR: *ν*_max_/cm^− 1^ 2978, 2932, 2897, 2824, 1659, 1593, 1562, 1504. ^1^H-NMR (DMSO-*d*_*6*_) *δ* (ppm): 1.18 (t, *J* = 6.2 Hz, 6 H, 2CH_3_), 3.88‒3.99 (m, 4 H, 2OCH_2_), 4.66 (s, 2 H, NCH_2_), 4.68 (s, 2 H, NCH_2_), 7.31 (s, 2 H, arom. H), 7.66 (s, 2 H, 2 olefinic CH), 7.92 (s, 2 H, arom. H), 8.00 (d, *J* = 5.2 Hz, 2 H, arom. H). ^13^C-NMR (DMSO-*d*_*6*_) *δ* (ppm): 15.46, 15.52 (CH_3_), 45.67, 45.70 (NCH_2_), 62.69, 62.74 (OCH_2_), 127.83, 127.88, 128.4, 129.36, 129.40, 132.4, 134.69, 134.72, 137.5 (arom. C + olefinic C), 184.7 (CO). MS, m/z (%): 439 (M, 22), 281 (100). Anal. Calcd. for C_19_H_22_NO_3_PS_3_ (439.54): C, 51.92; H, 5.05; N, 3.19. Found: C, 51.83; H, 4.99; N, 3.32.

### Antiproliferation properties

The cell lines used in the current study were kindly gifted by Prof. Stig Linder, Karolinska Institute, Stockholm, Sweden, originally purchased from ATCC. The synthesized compounds **20a‒l** were screened for their antiproliferation properties against MCF7 (breast), HCT116 (colon), and A431 (skin/squamous) cancer cell lines by the standard mitochondrial dependent reduction of yellow MTT [3-(4,5-dimethylthiazol-2-yl)-2,5-diphenyl-tetrazolium bromide] to purple formazan technique^52^. 5-Fluorouracil and sunitinib were used as standard reference drugs. Cells were suspended in DMEM medium for MCF7, A431, and McCoy’s 5 A for HCT116 in addition to 1% antibiotic–antimycotic mixture (10000 µg ml^− 1^ potassium penicillin, 10000 µg ml^− 1^ streptomycin sulfate and 25 µg ml^− 1^ amphotericin B), 10% fetal bovine serum and 1% L-glutamine at 37 °C, under 5% CO_2_ and 95% humidity. Cells were seeded at concentration of 30000 cells per well in fresh complete growth medium in 96-well tissue culture microtiter plates for 24 h. Media was aspirated, fresh complete medium was added and cells were incubated with different concentrations of the tested compound to give a final concentration of [25, 12.5, 6.25, and 3.125 µM, in addition to 1.5625, 0.78125, 0.390625, and 0.195313 µM in case of high potent analogs). 0.5% DMSO was used as a negative control. Triplicate wells were prepared for each individual dose. After 72 h of incubation, the medium was aspirated, 40 µl MTT salt (2.5 mg ml^− 1^) was added to each well and incubated for further 4 h at 37 °C. To stop the reaction and dissolve the crystals formed, 150 µl of 10% sodium dodecyl sulfate (SDS) in deionized water was added to each well and incubated overnight at 37 °C. The absorbance was then measured at 570 nm and a reference wavelength of 595 nm.

Data were recorded as mean values for experiments performed in triplicates for each individual dose measured by MTT assay. Control experiments did not exhibit a significant change compared to the DMSO vehicle. The cell surviving fraction was calculated according to the following equation.

$$\:Surviving\:fraction\:=\frac{Optical \:density\left(O.D.\right) of \: treated \: cells}{O.D. \: of \: control \: cells}$$

The agents synthesized were also tested against RPE1 (normal human immortalized retinal pigment epithelial cell line) cell (in DMEM-F12 medium) to determine the toxicity/selectivity towards normal cells relative to the cancer cell lines utilized.

The IC_50_ (concentration required to produce 50% inhibition of cell growth compared to the control experiment) was determined using Graph-Pad PRISM version-5 software. Statistical calculations for determination of the mean and standard deviation (SD) values were determined by SPSS 16 software. The observed anti-proliferative properties are presented in Table 1 (Supplementary Figs. S38‒S41).

### Biochemical/enzymatic and computational studies

Presented in the supplementary file.

## Supplementary Information

Below is the link to the electronic supplementary material.

Supplementary Material 1

Supplementary Material 2

Supplementary Material 3

Supplementary Material 4

Supplementary Material 5

## Data Availability

All data generated or analyzed during this study are included in this submitted article and its supplementary information file. Crystallographic data for the structures reported in this paper have been deposited at the Cambridge Crystallographic Data Centre (CCDC) in the CSD under reference CCDC 2440500 and CCDC2440501. These data can be obtained free of charge from the CCDC via [https://www.ccdc.cam.ac.uk/structures/](https:/www.ccdc.cam.ac.uk/structures).
