# Supplementary material for: Antineoplastic 4-piperidone-1-phosphonothioates with potential multi-targeted inhibitory properties
Source: Sci Rep. 2025 Nov 18;15:40363. doi: 10.1038/s41598-025-25796-6 (PMC12627723; doi:10.1038/s41598-025-25796-6)

checkCIF/PLATON report

Structure factors have been supplied for datablock(s) bmk2351b

THIS REPORT IS FOR GUIDANCE ONLY. IF USED AS PART OF A REVIEW PROCEDURE FOR PUBLICATION, IT SHOULD NOT REPLACE THE EXPERTISE OF AN EXPERIENCED CRYSTALLOGRAPHIC REFEREE.

No syntax errors found.   CIF dictionary   Interpreting this report

Datablock: bmk2351b

Bond precision:	C-C = 0.0083 A		Wavelength=1.54184
Cell:	a=32.9400(5)	b=12.5414(2)	c=22.4554(4)
	alpha=90	beta=90	gamma=90
Temperature:	293 K		

	Calculated	Reported
Volume	9276.6(3)	9276.6(3)
Space group	P n a 21	P n a 21
Hall group	P 2c -2n	P 2c -2n
Moiety formula	C23 H24 F2 N O3 P S	?
Sum formula	C23 H24 F2 N O3 P S	C23 H24 F2 N O3 P S
Mr	463.46	463.46
Dx,g cm-3	1.327	1.327
Z	16	16
Mu (mm-1)	2.245	2.245
F000	3872.0	3872.0
F000’	3893.51	
h,k,lmax	40,15,27	40,15,27
Nref	18485[9491]	15591
Tmin,Tmax	0.760,0.799	0.450,1.000
Tmin’	0.253	

Correction method= # Reported T Limits: Tmin=0.450 Tmax=1.000
AbsCorr = GAUSSIAN

Data completeness= 1.64/0.84   Theta(max)= 72.795

R(reflections)= 0.0567(12652)   wR2(reflections)= 0.1709(15591)

S = 1.027   Npar= 1389

The following ALERTS were generated. Each ALERT has the format
test-name_ALERT_alert-type_alert-level.
Click on the hyperlinks for more details of the test.

Alert level C

```
PLAT048_ALERT_1_C MoietyFormula Not Given (or Incomplete) ........     Please Check
PLAT089_ALERT_3_C Poor Data / Parameter Ratio (Zmax < 18) ........       6.72 Note
PLAT242_ALERT_2_C Low    'MainMol' Ueq as Compared to Neighbors of        C17 Check
PLAT242_ALERT_2_C Low    'MainMol' Ueq as Compared to Neighbors of        C24 Check
PLAT242_ALERT_2_C Low    'MainMol' Ueq as Compared to Neighbors of        C40 Check
PLAT242_ALERT_2_C Low    'MainMol' Ueq as Compared to Neighbors of        C47 Check
PLAT242_ALERT_2_C Low    'MainMol' Ueq as Compared to Neighbors of        C63 Check
PLAT242_ALERT_2_C Low    'MainMol' Ueq as Compared to Neighbors of        C70 Check
PLAT242_ALERT_2_C Low    'MainMol' Ueq as Compared to Neighbors of        C86 Check
PLAT340_ALERT_3_C Low Bond Precision on  C-C Bonds ...............    0.00834 Ang.
PLAT911_ALERT_3_C Missing FCF Refl Between Thmin & STh/L=    0.600         11 Report
                1 15  0,   2 15  0,   3 15  0,   2 15  1,   1  6  9,   2  6  9,
                0  6 10,   1  6 10,   2  6 10,   3  6 10,   6  0 19,
PLAT987_ALERT_1_C The Flack x is >> 0 -  Do a BASF/TWIN Refinement     Please Check
```

Alert level G

```
PLAT002_ALERT_2_G Number of Distance or Angle Restraints on AtSite         64 Note
PLAT003_ALERT_2_G Number of Uiso or U(i,j)  Restrained non-H-Atoms         64 Report
PLAT168_ALERT_4_G The CIF-Embedded .res File Contains EXYZ Records          4 Report
PLAT171_ALERT_4_G The CIF-Embedded .res File Contains EADP Records          4 Report
PLAT175_ALERT_4_G The CIF-Embedded .res File Contains SAME Records          4 Report
PLAT178_ALERT_4_G The CIF-Embedded .res File Contains SIMU Records          4 Report
PLAT186_ALERT_4_G The CIF-Embedded .res File Contains ISOR Records          4 Report
PLAT188_ALERT_3_G A Non-default SIMU Restraint Value has been used     0.0050 Report
PLAT188_ALERT_3_G A Non-default SIMU Restraint Value has been used     0.0050 Report
PLAT188_ALERT_3_G A Non-default SIMU Restraint Value has been used     0.0050 Report
PLAT188_ALERT_3_G A Non-default SIMU Restraint Value has been used     0.0050 Report
PLAT189_ALERT_3_G A Non-default SAME Restraint Value for First Par     0.0050 Report
PLAT189_ALERT_3_G A Non-default SAME Restraint Value for First Par     0.0050 Report
PLAT189_ALERT_3_G A Non-default SAME Restraint Value for First Par     0.0050 Report
PLAT189_ALERT_3_G A Non-default SAME Restraint Value for First Par     0.0050 Report
PLAT199_ALERT_1_G Reported _cell_measurement_temperature ..... (K)        293 Check
PLAT200_ALERT_1_G Reported   _diffrn_ambient_temperature ..... (K)        293 Check
PLAT230_ALERT_2_G Hirshfeld Test Diff for    P4        --O11       .      7.5 s.u.
PLAT230_ALERT_2_G Hirshfeld Test Diff for    P4        --O12       .      6.0 s.u.
PLAT301_ALERT_3_G Main Residue  Disorder ..............(Resd    1)        26% Note
PLAT301_ALERT_3_G Main Residue  Disorder ..............(Resd    2)        26% Note
PLAT301_ALERT_3_G Main Residue  Disorder ..............(Resd    3)        26% Note
PLAT301_ALERT_3_G Main Residue  Disorder ..............(Resd    4)        26% Note
PLAT811_ALERT_5_G No ADDSYM Analysis: Too Many Excluded Atoms ....          ! Info
PLAT860_ALERT_3_G Number of Least-Squares Restraints .............       1317 Note
PLAT883_ALERT_1_G Absent Datum for _atom_sites_solution_primary ..     Please Do !
PLAT910_ALERT_3_G Missing # of FCF Reflection(s) Below Theta(Min).          2 Note
                2  0  0,   2  0  1,
PLAT912_ALERT_4_G Missing # of FCF Reflections Above STh/L=  0.600        145 Note
PLAT915_ALERT_3_G No Flack x Check Done: Low Friedel Pair Coverage         70 %
PLAT941_ALERT_3_G Average HKL Measurement Multiplicity ...........        4.1 Low
PLAT969_ALERT_5_G The 'Henn et al.' R-Factor-gap value ...........      3.808 Note
              Predicted wR2: Based on SigI**2  4.49 or SHELX Weight 16.64
```

```
PLAT978_ALERT_2_G Number C-C Bonds with Positive Residual Density.          0 Info
PLAT992_ALERT_5_G Repd & Actual _reflns_number_gt Values Differ by          8 Check
```

```
   0 ALERT level A = Most likely a serious problem - resolve or explain
   0 ALERT level B = A potentially serious problem, consider carefully
  12 ALERT level C = Check. Ensure it is not caused by an omission or oversight
  33 ALERT level G = General information/check it is not something unexpected

   5 ALERT type 1 CIF construction/syntax error, inconsistent or missing data
  12 ALERT type 2 Indicator that the structure model may be wrong or deficient
  19 ALERT type 3 Indicator that the structure quality may be low
   6 ALERT type 4 Improvement, methodology, query or suggestion
   3 ALERT type 5 Informative message, check
```

---

checkCIF publication errors

Alert level A

```
PUBL004_ALERT_1_A  The contact author's name and address are missing,
            _publ_contact_author_name and _publ_contact_author_address.
PUBL005_ALERT_1_A  _publ_contact_author_email, _publ_contact_author_fax and
            _publ_contact_author_phone are all missing.
            At least one of these should be present.
PUBL006_ALERT_1_A  _publ_requested_journal is missing
            e.g. 'Acta Crystallographica Section C'
PUBL008_ALERT_1_A  _publ_section_title is missing. Title of paper.
PUBL009_ALERT_1_A  _publ_author_name is missing. List of author(s) name(s).
PUBL010_ALERT_1_A  _publ_author_address is missing. Author(s) address(es).
PUBL012_ALERT_1_A  _publ_section_abstract is missing.
            Abstract of paper in English.
```

Alert level G

```
PUBL017_ALERT_1_G The _publ_section_references section is missing or
            empty.
```

```
   7 ALERT level A = Data missing that is essential or data in wrong format
   1 ALERT level G = General alerts. Data that may be required is missing
```

Publication of your CIF

You should attempt to resolve as many as possible of the alerts in all categories. Often the minor alerts point to easily fixed oversights, errors and omissions in your CIF or refinement strategy, so attention to these fine details can be worthwhile. In order to resolve some of the more serious problems it may be necessary to carry out additional measurements or structure refinements. However, the nature of your study may justify the reported deviations from journal submission requirements and the more serious of these should be commented upon in the discussion or experimental section of a paper or in the "special_details" fields of the CIF. *checkCIF* was carefully designed to identify outliers and unusual parameters, but every test has its limitations and alerts that are not important in a particular case may appear. Conversely, the absence of alerts does not guarantee there are no aspects of the results needing attention. It is up to the individual to critically assess their own results and, if necessary, seek expert advice.

If level A alerts remain, which you believe to be justified deviations, and you intend to submit this CIF for publication in a journal, you should additionally insert an explanation in your CIF using the Validation Reply Form (VRF) below. This will allow your explanation to be considered as part of the review process.

```
# start Validation Reply Form
_vrf_PUBL004_GLOBAL
;
PROBLEM: The contact author's name and address are missing,
RESPONSE: ...
;
_vrf_PUBL005_GLOBAL
;
PROBLEM: _publ_contact_author_email, _publ_contact_author_fax and
RESPONSE: ...
;
_vrf_PUBL006_GLOBAL
;
PROBLEM: _publ_requested_journal is missing
RESPONSE: ...
;
_vrf_PUBL008_GLOBAL
;
PROBLEM: _publ_section_title is missing. Title of paper.
RESPONSE: ...
;
_vrf_PUBL009_GLOBAL
;
PROBLEM: _publ_author_name is missing. List of author(s) name(s).
RESPONSE: ...
;
_vrf_PUBL010_GLOBAL
;
PROBLEM: _publ_author_address is missing. Author(s) address(es).
RESPONSE: ...
;
_vrf_PUBL012_GLOBAL
;
```

```
PROBLEM: _publ_section_abstract is missing.
RESPONSE: ...
;
# end Validation Reply Form
```

If you wish to submit your CIF for publication in Acta Crystallographica Section C or E, you should upload your CIF via the web. If you wish to submit your CIF for publication in IUCrData you should upload your CIF via the web. If your CIF is to form part of a submission to another IUCr journal, you will be asked, either during electronic submission or by the Co-editor handling your paper, to upload your CIF via our web site.

PLATON version of 02/02/2025; check.def file version of 02/02/2025

Datablock bmk2351b - ellipsoid plot

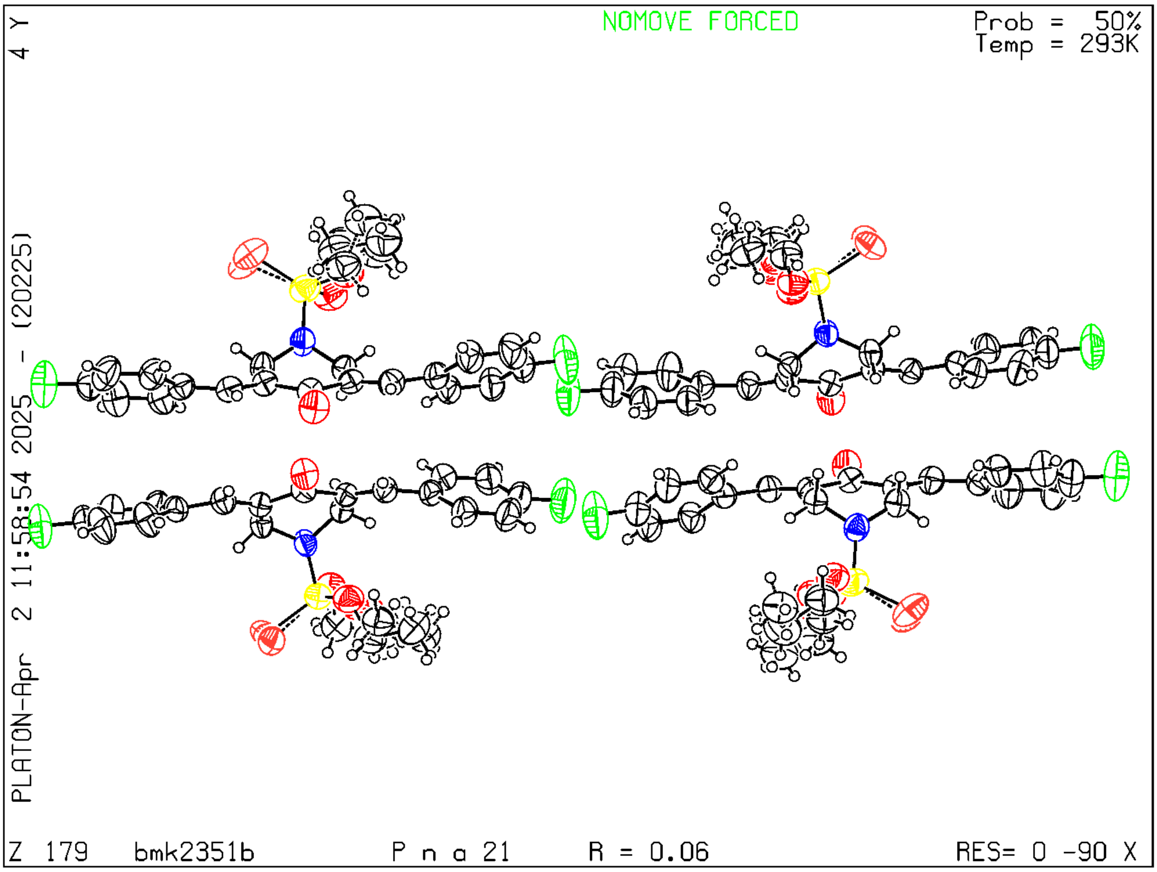

Supplement: Supplementary file 2 — Supplementary Material 2 [file 41598_2025_25796_MOESM2_ESM.pdf]
