# Supplementary material for: Antineoplastic 4-piperidone-1-phosphonothioates with potential multi-targeted inhibitory properties
Source: Sci Rep. 2025 Nov 18;15:40363. doi: 10.1038/s41598-025-25796-6 (PMC12627723; doi:10.1038/s41598-025-25796-6)
Supplement: Supplementary file 3 — Supplementary Material 3 [file 41598_2025_25796_MOESM3_ESM.docx]

data_bmk2342b

_audit_creation_method 'SHELXL-2019/3'

_shelx_SHELXL_version_number '2019/3'

_chemical_name_systematic ?

_chemical_name_common ?

_chemical_melting_point ?

_chemical_formula_moiety ?

_chemical_formula_sum

'C23 H26 N O3 P S'

_chemical_formula_weight 427.48

loop_

_atom_type_symbol

_atom_type_description

_atom_type_scat_dispersion_real

_atom_type_scat_dispersion_imag

_atom_type_scat_source

'C' 'C' 0.0033 0.0016

'International Tables Vol C Tables 4.2.6.8 and 6.1.1.4'

'H' 'H' 0.0000 0.0000

'International Tables Vol C Tables 4.2.6.8 and 6.1.1.4'

'N' 'N' 0.0061 0.0033

'International Tables Vol C Tables 4.2.6.8 and 6.1.1.4'

'O' 'O' 0.0106 0.0060

'International Tables Vol C Tables 4.2.6.8 and 6.1.1.4'

'P' 'P' 0.1023 0.0942

'International Tables Vol C Tables 4.2.6.8 and 6.1.1.4'

'S' 'S' 0.1246 0.1234

'International Tables Vol C Tables 4.2.6.8 and 6.1.1.4'

_space_group_crystal_system monoclinic

_space_group_IT_number 14

_space_group_name_H-M_alt 'P 21/c'

_space_group_name_Hall '-P 2ybc'

_shelx_space_group_comment

;

The symmetry employed for this shelxl refinement is uniquely defined

by the following loop, which should always be used as a source of

symmetry information in preference to the above space-group names.

They are only intended as comments.

;

loop_

_space_group_symop_operation_xyz

'x, y, z'

'-x, y+1/2, -z+1/2'

'-x, -y, -z'

'x, -y-1/2, z-1/2'

_cell_length_a 15.9985(6)

_cell_length_b 23.6855(8)

_cell_length_c 12.8351(5)

_cell_angle_alpha 90

_cell_angle_beta 112.788(4)

_cell_angle_gamma 90

_cell_volume 4484.0(3)

_cell_formula_units_Z 8

_cell_measurement_temperature 293(2)

_cell_measurement_reflns_used 15147

_cell_measurement_theta_min 3.6920

_cell_measurement_theta_max 26.2150

_exptl_crystal_description block

_exptl_crystal_colour colourless

_exptl_crystal_density_meas ?

_exptl_crystal_density_method ?

_exptl_crystal_density_diffrn 1.266

_exptl_crystal_F_000 1808

_exptl_transmission_factor_min ?

_exptl_transmission_factor_max ?

_exptl_crystal_size_max 0.440

_exptl_crystal_size_mid 0.410

_exptl_crystal_size_min 0.280

_exptl_absorpt_coefficient_mu 0.239

_shelx_estimated_absorpt_T_min 0.902

_shelx_estimated_absorpt_T_max 0.936

_exptl_absorpt_correction_T_min 0.255

_exptl_absorpt_correction_T_max 1.000

_exptl_absorpt_correction_type gaussian

_exptl_absorpt_process_details

;

CrysAlisPro 1.171.42.90a (Rigaku Oxford Diffraction, 2023)

Numerical absorption correction based on gaussian integration over

a multifaceted crystal model

Empirical absorption correction using spherical harmonics,

implemented in SCALE3 ABSPACK scaling algorithm.

;

_diffrn_ambient_temperature 293(2)

_diffrn_radiation_wavelength 0.71073

_diffrn_radiation_type MoK\a

_diffrn_source ?

_diffrn_measurement_device_type 'SuperNova, Dual, Cu at home/near, Atlas'

_diffrn_measurement_method '\w scans'

_diffrn_detector_area_resol_mean 10.5082

_diffrn_reflns_number 57048

_diffrn_reflns_av_unetI/netI 0.0361

_diffrn_reflns_av_R_equivalents 0.0524

_diffrn_reflns_limit_h_min -21

_diffrn_reflns_limit_h_max 22

_diffrn_reflns_limit_k_min -32

_diffrn_reflns_limit_k_max 32

_diffrn_reflns_limit_l_min -17

_diffrn_reflns_limit_l_max 17

_diffrn_reflns_theta_min 3.441

_diffrn_reflns_theta_max 29.779

_diffrn_reflns_theta_full 25.242

_diffrn_measured_fraction_theta_max 0.891

_diffrn_measured_fraction_theta_full 0.997

_diffrn_reflns_Laue_measured_fraction_max 0.891

_diffrn_reflns_Laue_measured_fraction_full 0.997

_diffrn_reflns_point_group_measured_fraction_max 0.891

_diffrn_reflns_point_group_measured_fraction_full 0.997

_reflns_number_total 11415

_reflns_number_gt 5914

_reflns_threshold_expression 'I > 2\s(I)'

_reflns_Friedel_coverage 0.000

_reflns_Friedel_fraction_max .

_reflns_Friedel_fraction_full .

_reflns_special_details

;

Reflections were merged by SHELXL according to the crystal

class for the calculation of statistics and refinement.

_reflns_Friedel_fraction is defined as the number of unique

Friedel pairs measured divided by the number that would be

possible theoretically, ignoring centric projections and

systematic absences.

;

_computing_data_collection 'CrysAlisPro 1.171.42.90a (Rigaku OD, 2023)'

_computing_cell_refinement 'CrysAlisPro 1.171.42.90a (Rigaku OD, 2023)'

_computing_data_reduction 'CrysAlisPro 1.171.42.90a (Rigaku OD, 2023)'

_computing_structure_solution 'SHELXT'

_computing_structure_refinement 'SHELXL-2019/2 (Sheldrick, 2019)'

_computing_molecular_graphics ?

_computing_publication_material ?

_refine_special_details ?

_refine_ls_structure_factor_coef Fsqd

_refine_ls_matrix_type full

_refine_ls_weighting_scheme calc

_refine_ls_weighting_details

'w=1/[\s^2^(Fo^2^)+(0.1273P)^2^+1.8653P] where P=(Fo^2^+2Fc^2^)/3'

_atom_sites_solution_primary ?

_atom_sites_solution_secondary ?

_atom_sites_solution_hydrogens geom

_refine_ls_hydrogen_treatment constr

_refine_ls_extinction_method none

_refine_ls_extinction_coef .

_refine_ls_number_reflns 11415

_refine_ls_number_parameters 677

_refine_ls_number_restraints 652

_refine_ls_R_factor_all 0.1403

_refine_ls_R_factor_gt 0.0838

_refine_ls_wR_factor_ref 0.2925

_refine_ls_wR_factor_gt 0.2493

_refine_ls_goodness_of_fit_ref 1.078

_refine_ls_restrained_S_all 1.053

_refine_ls_shift/su_max 0.000

_refine_ls_shift/su_mean 0.000

loop_

_atom_site_label

_atom_site_type_symbol

_atom_site_fract_x

_atom_site_fract_y

_atom_site_fract_z

_atom_site_U_iso_or_equiv

_atom_site_adp_type

_atom_site_occupancy

_atom_site_site_symmetry_order

_atom_site_calc_flag

_atom_site_refinement_flags_posn

_atom_site_refinement_flags_adp

_atom_site_refinement_flags_occupancy

_atom_site_disorder_assembly

_atom_site_disorder_group

C1 C 0.8726(2) 0.10601(18) 0.0866(3) 0.0765(10) Uani 1 1 d . . . . .

C2 C 0.9009(3) 0.0664(2) 0.0267(4) 0.0930(12) Uani 1 1 d . . . . .

H2 H 0.913870 0.078122 -0.034529 0.112 Uiso 1 1 calc R U . . .

C3 C 0.9099(3) 0.0100(2) 0.0564(5) 0.1126(16) Uani 1 1 d . . . . .

H3 H 0.930541 -0.015364 0.016515 0.135 Uiso 1 1 calc R U . . .

C4 C 0.8888(4) -0.0088(2) 0.1439(5) 0.1128(15) Uani 1 1 d . . . . .

H4 H 0.894340 -0.046859 0.163063 0.135 Uiso 1 1 calc R U . . .

C5 C 0.8591(4) 0.0296(2) 0.2039(4) 0.1098(15) Uani 1 1 d . . . . .

H5 H 0.844528 0.017326 0.263627 0.132 Uiso 1 1 calc R U . . .

C6 C 0.8512(3) 0.0852(2) 0.1753(3) 0.0907(12) Uani 1 1 d . . . . .

H6 H 0.830875 0.110312 0.216128 0.109 Uiso 1 1 calc R U . . .

C7 C 0.8625(2) 0.16445(19) 0.0491(3) 0.0779(10) Uani 1 1 d . . . . .

H7 H 0.848121 0.169450 -0.027778 0.094 Uiso 1 1 calc R U . . .

C8 C 0.9028(3) 0.21502(16) 0.2353(3) 0.0746(9) Uani 1 1 d . . . . .

H8A H 0.851284 0.212670 0.257159 0.089 Uiso 1 1 calc R U . . .

H8B H 0.942718 0.183429 0.268972 0.089 Uiso 1 1 calc R U . . .

C9 C 0.8706(2) 0.21224(17) 0.1081(3) 0.0705(9) Uani 1 1 d . . . . .

C10 C 0.8509(2) 0.26652(17) 0.0431(3) 0.0737(10) Uani 1 1 d . . . . .

C11 C 0.8654(2) 0.32059(17) 0.1073(3) 0.0727(9) Uani 1 1 d . . . . .

C12 C 0.8939(2) 0.31775(16) 0.2332(3) 0.0730(9) Uani 1 1 d . . . . .

H12A H 0.927370 0.351532 0.267927 0.088 Uiso 1 1 calc R U . . .

H12B H 0.840815 0.315370 0.251997 0.088 Uiso 1 1 calc R U . . .

C13 C 0.8568(3) 0.3692(2) 0.0493(3) 0.0857(11) Uani 1 1 d . . . . .

H13 H 0.843636 0.365060 -0.027458 0.103 Uiso 1 1 calc R U . . .

C14 C 0.8649(3) 0.42752(19) 0.0893(3) 0.0842(11) Uani 1 1 d . . . . .

C15 C 0.8398(3) 0.44530(18) 0.1769(4) 0.0884(11) Uani 1 1 d . . . . .

H15 H 0.818887 0.418685 0.214346 0.106 Uiso 1 1 calc R U . . .

C16 C 0.8452(3) 0.5010(2) 0.2096(5) 0.1052(14) Uani 1 1 d . . . . .

H16 H 0.826102 0.511620 0.266610 0.126 Uiso 1 1 calc R U . . .

C17 C 0.8786(4) 0.5408(2) 0.1585(6) 0.1209(17) Uani 1 1 d . . . . .

H17 H 0.884250 0.578344 0.181777 0.145 Uiso 1 1 calc R U . . .

C18 C 0.9036(4) 0.5240(3) 0.0716(6) 0.135(2) Uani 1 1 d . . . . .

H18 H 0.926494 0.550577 0.036412 0.162 Uiso 1 1 calc R U . . .

C19 C 0.8954(4) 0.4686(3) 0.0360(5) 0.1137(16) Uani 1 1 d . . . . .

H19 H 0.910482 0.458820 -0.024682 0.136 Uiso 1 1 calc R U . . .

C31 C 0.5977(2) 0.21103(15) 0.3308(3) 0.0719(9) Uani 1 1 d . . . . .

H31A H 0.556615 0.179775 0.323351 0.086 Uiso 1 1 calc R U . . .

H31B H 0.648821 0.207000 0.402495 0.086 Uiso 1 1 calc R U . . .

C32 C 0.6308(2) 0.20882(16) 0.2363(3) 0.0681(8) Uani 1 1 d . . . . .

C33 C 0.6516(2) 0.26352(16) 0.1919(3) 0.0695(9) Uani 1 1 d . . . . .

C34 C 0.6385(2) 0.31745(16) 0.2448(3) 0.0686(8) Uani 1 1 d . . . . .

C35 C 0.6106(2) 0.31308(15) 0.3440(3) 0.0708(8) Uani 1 1 d . . . . .

H35A H 0.664037 0.309385 0.413327 0.085 Uiso 1 1 calc R U . . .

H35B H 0.578720 0.347117 0.349190 0.085 Uiso 1 1 calc R U . . .

C36 C 0.6450(2) 0.36597(18) 0.1953(3) 0.0787(10) Uani 1 1 d . . . . .

H36 H 0.657055 0.362032 0.130413 0.094 Uiso 1 1 calc R U . . .

C37 C 0.6361(2) 0.42396(17) 0.2269(3) 0.0768(9) Uani 1 1 d . . . . .

C38 C 0.6023(3) 0.4646(2) 0.1428(4) 0.0987(13) Uani 1 1 d . . . . .

H38 H 0.584991 0.454110 0.067583 0.118 Uiso 1 1 calc R U . . .

C39 C 0.5937(4) 0.5207(2) 0.1690(5) 0.1163(17) Uani 1 1 d . . . . .

H39 H 0.569935 0.547124 0.111433 0.140 Uiso 1 1 calc R U . . .

C40 C 0.6205(3) 0.5373(2) 0.2804(6) 0.1097(15) Uani 1 1 d . . . . .

H40 H 0.614675 0.574726 0.298624 0.132 Uiso 1 1 calc R U . . .

C41 C 0.6562(3) 0.4972(2) 0.3640(4) 0.0993(13) Uani 1 1 d . . . . .

H41 H 0.675305 0.508055 0.439344 0.119 Uiso 1 1 calc R U . . .

C42 C 0.6641(3) 0.44187(17) 0.3384(3) 0.0810(10) Uani 1 1 d . . . . .

H42 H 0.688537 0.415827 0.396594 0.097 Uiso 1 1 calc R U . . .

N1 N 0.9510(2) 0.26788(12) 0.2756(2) 0.0770(8) Uani 1 1 d . . . . .

N2 N 0.55140(19) 0.26379(12) 0.3301(3) 0.0732(8) Uani 1 1 d . . . . .

O1 O 0.8223(2) 0.26657(13) -0.0597(2) 0.0977(9) Uani 1 1 d . . . . .

O4 O 0.6795(2) 0.26437(12) 0.1163(2) 0.0916(8) Uani 1 1 d . . . . .

C20 C 1.1109(17) 0.3144(8) 0.2090(15) 0.107(5) Uani 0.60(2) 1 d D U P A 1

H20A H 1.057074 0.297415 0.152914 0.129 Uiso 0.60(2) 1 calc R U P A 1

H20B H 1.161670 0.289258 0.220886 0.129 Uiso 0.60(2) 1 calc R U P A 1

C21 C 1.1285(17) 0.3701(9) 0.167(2) 0.120(5) Uani 0.60(2) 1 d D U P A 1

H21A H 1.075319 0.393334 0.146740 0.181 Uiso 0.60(2) 1 calc R U P A 1

H21B H 1.143204 0.364474 0.102081 0.181 Uiso 0.60(2) 1 calc R U P A 1

H21C H 1.178257 0.388452 0.225482 0.181 Uiso 0.60(2) 1 calc R U P A 1

C22 C 1.1474(9) 0.3717(4) 0.5884(15) 0.114(5) Uani 0.60(2) 1 d D U P A 1

H22A H 1.166859 0.385249 0.530980 0.170 Uiso 0.60(2) 1 calc R U P A 1

H22B H 1.195990 0.375586 0.661145 0.170 Uiso 0.60(2) 1 calc R U P A 1

H22C H 1.096169 0.393365 0.586823 0.170 Uiso 0.60(2) 1 calc R U P A 1

C23 C 1.1220(12) 0.3129(4) 0.5679(8) 0.102(4) Uani 0.60(2) 1 d D U P A 1

H23A H 1.173721 0.291342 0.568451 0.123 Uiso 0.60(2) 1 calc R U P A 1

H23B H 1.105808 0.298871 0.628616 0.123 Uiso 0.60(2) 1 calc R U P A 1

O2 O 1.0984(7) 0.3215(4) 0.3130(7) 0.080(2) Uani 0.60(2) 1 d D U P A 1

O3 O 1.0485(7) 0.3044(5) 0.4638(6) 0.082(2) Uani 0.60(2) 1 d D U P A 1

P1 P 1.0559(5) 0.2725(3) 0.3601(5) 0.0703(12) Uani 0.60(2) 1 d D U P A 1

S1 S 1.1204(10) 0.2020(4) 0.3933(15) 0.118(3) Uani 0.60(2) 1 d D U P A 1

C20A C 1.129(3) 0.3152(12) 0.208(3) 0.114(7) Uani 0.40(2) 1 d D U P A 2

H20C H 1.084765 0.294443 0.145654 0.137 Uiso 0.40(2) 1 calc R U P A 2

H20D H 1.182585 0.292009 0.241167 0.137 Uiso 0.40(2) 1 calc R U P A 2

C21A C 1.152(3) 0.3691(17) 0.166(4) 0.164(13) Uani 0.40(2) 1 d D U P A 2

H21D H 1.101836 0.394624 0.145157 0.247 Uiso 0.40(2) 1 calc R U P A 2

H21E H 1.166175 0.361540 0.100561 0.247 Uiso 0.40(2) 1 calc R U P A 2

H21F H 1.204064 0.385865 0.223769 0.247 Uiso 0.40(2) 1 calc R U P A 2

C23A C 1.1321(13) 0.3597(8) 0.6349(11) 0.092(5) Uani 0.40(2) 1 d D U P A 2

H23C H 1.081593 0.384993 0.603172 0.137 Uiso 0.40(2) 1 calc R U P A 2

H23D H 1.185236 0.380869 0.679178 0.137 Uiso 0.40(2) 1 calc R U P A 2

H23E H 1.119166 0.332594 0.682167 0.137 Uiso 0.40(2) 1 calc R U P A 2

C22A C 1.1477(9) 0.3307(11) 0.5445(12) 0.094(5) Uani 0.40(2) 1 d D U P A 2

H22D H 1.178532 0.355871 0.511471 0.112 Uiso 0.40(2) 1 calc R U P A 2

H22E H 1.186628 0.298314 0.575247 0.112 Uiso 0.40(2) 1 calc R U P A 2

O2A O 1.0918(16) 0.3270(7) 0.2910(18) 0.123(6) Uani 0.40(2) 1 d D U P A 2

O3A O 1.0650(10) 0.3123(9) 0.4593(12) 0.110(6) Uani 0.40(2) 1 d D U P A 2

P1A P 1.0596(8) 0.2784(5) 0.3510(9) 0.090(3) Uani 0.40(2) 1 d D U P A 2

S1A S 1.1295(16) 0.2097(7) 0.378(2) 0.102(3) Uani 0.40(2) 1 d D U P A 2

C43 C 0.3438(7) 0.3251(7) 0.0906(10) 0.103(4) Uani 0.540(8) 1 d D U P B 1

H43A H 0.311474 0.339436 0.135172 0.124 Uiso 0.540(8) 1 calc R U P B 1

H43B H 0.309051 0.293973 0.045206 0.124 Uiso 0.540(8) 1 calc R U P B 1

C44 C 0.3509(16) 0.3694(9) 0.0165(16) 0.109(5) Uani 0.540(8) 1 d D U P B 1

H44A H 0.386297 0.399991 0.061061 0.163 Uiso 0.540(8) 1 calc R U P B 1

H44B H 0.291420 0.382782 -0.029711 0.163 Uiso 0.540(8) 1 calc R U P B 1

H44C H 0.379810 0.354754 -0.030919 0.163 Uiso 0.540(8) 1 calc R U P B 1

C45 C 0.4164(11) 0.3103(6) 0.4529(9) 0.114(4) Uani 0.540(8) 1 d D U P B 1

H45A H 0.385204 0.274722 0.448659 0.137 Uiso 0.540(8) 1 calc R U P B 1

H45B H 0.477410 0.306346 0.509551 0.137 Uiso 0.540(8) 1 calc R U P B 1

C46 C 0.371(2) 0.3526(10) 0.487(2) 0.157(9) Uani 0.540(8) 1 d D U P B 1

H46A H 0.404991 0.387258 0.498305 0.236 Uiso 0.540(8) 1 calc R U P B 1

H46B H 0.366577 0.341671 0.556521 0.236 Uiso 0.540(8) 1 calc R U P B 1

H46C H 0.311860 0.358019 0.429834 0.236 Uiso 0.540(8) 1 calc R U P B 1

O5 O 0.4314(4) 0.3046(3) 0.1654(5) 0.0822(18) Uani 0.540(8) 1 d D U P B 1

O6 O 0.4214(4) 0.3214(2) 0.3448(5) 0.0778(17) Uani 0.540(8) 1 d D U P B 1

P2 P 0.4432(5) 0.2720(2) 0.2771(4) 0.0727(10) Uani 0.540(8) 1 d D U P B 1

S2 S 0.3736(5) 0.2042(3) 0.2615(8) 0.0935(19) Uani 0.540(8) 1 d D U P B 1

C43A C 0.3798(10) 0.3167(7) 0.1049(10) 0.101(4) Uani 0.460(8) 1 d D U P B 2

H43C H 0.324567 0.294644 0.073288 0.122 Uiso 0.460(8) 1 calc R U P B 2

H43D H 0.427604 0.296179 0.092538 0.122 Uiso 0.460(8) 1 calc R U P B 2

C44A C 0.366(2) 0.3710(11) 0.0479(19) 0.128(9) Uani 0.460(8) 1 d D U P B 2

H44D H 0.321343 0.392120 0.063432 0.192 Uiso 0.460(8) 1 calc R U P B 2

H44E H 0.346487 0.365065 -0.032088 0.192 Uiso 0.460(8) 1 calc R U P B 2

H44F H 0.422465 0.391530 0.074664 0.192 Uiso 0.460(8) 1 calc R U P B 2

C45A C 0.3782(12) 0.3115(7) 0.4518(15) 0.108(5) Uani 0.460(8) 1 d D U P B 2

H45C H 0.325926 0.293268 0.395350 0.130 Uiso 0.460(8) 1 calc R U P B 2

H45D H 0.389138 0.293558 0.523845 0.130 Uiso 0.460(8) 1 calc R U P B 2

C46A C 0.358(2) 0.3685(9) 0.459(3) 0.129(8) Uani 0.460(8) 1 d D U P B 2

H46D H 0.412782 0.389699 0.491933 0.193 Uiso 0.460(8) 1 calc R U P B 2

H46E H 0.322821 0.372146 0.504906 0.193 Uiso 0.460(8) 1 calc R U P B 2

H46F H 0.323040 0.382729 0.384443 0.193 Uiso 0.460(8) 1 calc R U P B 2

O5A O 0.4039(4) 0.3239(2) 0.2253(5) 0.086(2) Uani 0.460(8) 1 d D U P B 2

O6A O 0.4553(5) 0.3021(4) 0.4234(7) 0.094(3) Uani 0.460(8) 1 d D U P B 2

P2A P 0.4442(6) 0.2732(3) 0.3089(6) 0.0772(14) Uani 0.460(8) 1 d D U P B 2

S2A S 0.3747(9) 0.2053(4) 0.2753(11) 0.120(4) Uani 0.460(8) 1 d D U P B 2

C24 C 0.6315(2) 0.10254(18) 0.2115(3) 0.0792(10) Uani 1 1 d . . . . .

C25 C 0.6053(3) 0.0639(2) 0.1235(4) 0.0974(13) Uani 1 1 d . . . . .

H25 H 0.593447 0.076421 0.050493 0.117 Uiso 1 1 calc R U . . .

C26 C 0.5966(4) 0.0072(2) 0.1421(5) 0.1124(16) Uani 1 1 d . . . . .

H26 H 0.579249 -0.017989 0.081865 0.135 Uiso 1 1 calc R U . . .

C27 C 0.6131(4) -0.0120(2) 0.2477(5) 0.1106(15) Uani 1 1 d . . . . .

H27 H 0.606153 -0.050053 0.259918 0.133 Uiso 1 1 calc R U . . .

C28 C 0.6404(4) 0.0255(2) 0.3369(4) 0.1078(14) Uani 1 1 d . . . . .

H28 H 0.652938 0.012557 0.409771 0.129 Uiso 1 1 calc R U . . .

C29 C 0.6490(3) 0.08199(19) 0.3184(3) 0.0865(11) Uani 1 1 d . . . . .

H29 H 0.666985 0.106832 0.379209 0.104 Uiso 1 1 calc R U . . .

C30 C 0.6417(2) 0.16127(17) 0.1855(3) 0.0763(9) Uani 1 1 d . . . . .

H30 H 0.658239 0.166828 0.124338 0.092 Uiso 1 1 calc R U . . .

loop_

_atom_site_aniso_label

_atom_site_aniso_U_11

_atom_site_aniso_U_22

_atom_site_aniso_U_33

_atom_site_aniso_U_23

_atom_site_aniso_U_13

_atom_site_aniso_U_12

C1 0.0583(18) 0.097(3) 0.069(2) -0.0194(19) 0.0195(15) -0.0031(17)

C2 0.089(3) 0.101(3) 0.095(3) -0.023(2) 0.042(2) -0.005(2)

C3 0.107(3) 0.107(4) 0.131(4) -0.037(3) 0.055(3) -0.005(3)

C4 0.106(3) 0.103(4) 0.125(4) -0.015(3) 0.040(3) -0.012(3)

C5 0.119(4) 0.109(4) 0.103(3) -0.014(3) 0.046(3) -0.018(3)

C6 0.091(3) 0.103(3) 0.082(2) -0.018(2) 0.038(2) -0.007(2)

C7 0.0633(19) 0.112(3) 0.0577(18) -0.0130(19) 0.0224(15) 0.0011(19)

C8 0.082(2) 0.079(2) 0.0589(18) -0.0035(16) 0.0230(16) 0.0011(18)

C9 0.0576(17) 0.098(3) 0.0539(17) -0.0063(16) 0.0192(14) 0.0034(16)

C10 0.0654(19) 0.099(3) 0.0542(18) 0.0010(17) 0.0208(15) 0.0111(17)

C11 0.0604(18) 0.093(3) 0.0616(18) 0.0070(17) 0.0206(14) 0.0111(17)

C12 0.0687(19) 0.082(2) 0.0619(18) 0.0022(16) 0.0183(15) 0.0103(17)

C13 0.076(2) 0.108(3) 0.071(2) 0.016(2) 0.0255(17) 0.016(2)

C14 0.068(2) 0.097(3) 0.083(2) 0.026(2) 0.0247(18) 0.0125(19)

C15 0.075(2) 0.085(3) 0.109(3) 0.020(2) 0.039(2) 0.0067(19)

C16 0.101(3) 0.087(3) 0.128(4) 0.017(3) 0.045(3) 0.010(2)

C17 0.114(4) 0.091(3) 0.148(5) 0.031(3) 0.040(4) 0.008(3)

C18 0.124(4) 0.134(5) 0.149(5) 0.063(4) 0.054(4) 0.000(4)

C19 0.121(4) 0.115(4) 0.111(4) 0.041(3) 0.052(3) 0.016(3)

C31 0.074(2) 0.076(2) 0.073(2) -0.0019(16) 0.0368(17) -0.0029(16)

C32 0.0589(17) 0.083(2) 0.0596(17) -0.0068(16) 0.0200(14) -0.0015(15)

C33 0.0630(18) 0.087(2) 0.0587(17) -0.0083(16) 0.0242(14) -0.0095(16)

C34 0.0554(17) 0.083(2) 0.0674(18) -0.0040(16) 0.0239(14) -0.0102(15)

C35 0.0672(19) 0.079(2) 0.0695(19) -0.0099(16) 0.0302(15) -0.0088(16)

C36 0.070(2) 0.099(3) 0.068(2) -0.0002(19) 0.0274(16) -0.0119(19)

C37 0.0632(19) 0.083(2) 0.085(2) 0.0092(19) 0.0290(17) -0.0027(17)

C38 0.096(3) 0.103(4) 0.095(3) 0.019(2) 0.035(2) -0.007(2)

C39 0.105(3) 0.102(4) 0.141(5) 0.045(3) 0.046(3) 0.012(3)

C40 0.103(3) 0.080(3) 0.149(5) 0.005(3) 0.051(3) -0.001(2)

C41 0.098(3) 0.088(3) 0.111(3) -0.005(3) 0.040(3) -0.008(2)

C42 0.077(2) 0.074(2) 0.085(2) 0.0009(19) 0.0236(18) -0.0042(18)

N1 0.0770(18) 0.079(2) 0.0598(15) -0.0028(13) 0.0099(13) 0.0087(14)

N2 0.0642(16) 0.077(2) 0.0866(19) -0.0061(14) 0.0377(14) -0.0072(13)

O1 0.107(2) 0.123(3) 0.0564(14) 0.0040(13) 0.0234(13) 0.0184(17)

O4 0.108(2) 0.104(2) 0.0805(16) -0.0119(14) 0.0570(15) -0.0147(16)

C20 0.095(9) 0.154(10) 0.080(7) -0.014(7) 0.041(5) 0.018(7)

C21 0.116(10) 0.153(11) 0.119(8) 0.040(8) 0.076(7) 0.032(7)

C22 0.110(7) 0.076(6) 0.126(11) -0.012(7) 0.014(7) -0.001(5)

C23 0.129(9) 0.078(5) 0.074(5) -0.002(4) 0.011(5) -0.007(5)

O2 0.074(4) 0.100(5) 0.064(3) -0.017(3) 0.024(3) -0.001(4)

O3 0.086(4) 0.103(5) 0.054(4) -0.004(3) 0.022(3) 0.005(3)

P1 0.074(2) 0.076(2) 0.0496(15) -0.0089(15) 0.0115(13) 0.0119(16)

S1 0.113(6) 0.104(4) 0.097(4) -0.004(3) -0.004(3) 0.045(4)

C20A 0.106(13) 0.140(14) 0.115(12) -0.004(11) 0.064(9) 0.040(10)

C21A 0.16(3) 0.19(2) 0.18(2) 0.013(17) 0.105(19) 0.011(19)

C23A 0.108(10) 0.082(9) 0.072(7) 0.005(6) 0.021(6) 0.013(7)

C22A 0.074(7) 0.115(13) 0.076(7) -0.010(8) 0.012(5) -0.009(7)

O2A 0.111(10) 0.138(10) 0.131(11) 0.039(8) 0.059(8) 0.030(8)

O3A 0.086(7) 0.115(9) 0.093(9) -0.043(7) -0.006(6) 0.009(6)

P1A 0.069(3) 0.094(4) 0.091(5) 0.012(3) 0.014(3) 0.008(3)

S1A 0.091(3) 0.097(4) 0.100(6) 0.002(3) 0.016(4) 0.021(3)

C43 0.059(6) 0.140(9) 0.094(6) 0.022(6) 0.010(5) 0.001(6)

C44 0.100(8) 0.104(9) 0.100(11) -0.003(7) 0.013(9) 0.000(7)

C45 0.110(11) 0.137(9) 0.095(7) 0.019(6) 0.040(7) 0.028(9)

C46 0.205(18) 0.143(18) 0.171(15) 0.032(12) 0.124(14) 0.061(13)

O5 0.060(3) 0.118(4) 0.067(3) 0.010(3) 0.023(2) -0.003(3)

O6 0.077(3) 0.091(4) 0.075(4) 0.010(3) 0.040(3) 0.012(2)

P2 0.0581(13) 0.0876(19) 0.074(2) -0.0019(15) 0.0274(19) -0.0072(11)

S2 0.071(3) 0.090(4) 0.113(3) -0.012(2) 0.028(2) -0.030(2)

C43A 0.067(9) 0.114(9) 0.099(8) -0.010(7) 0.006(8) -0.008(8)

C44A 0.149(17) 0.102(12) 0.087(12) 0.015(9) -0.004(10) -0.013(10)

C45A 0.120(11) 0.107(9) 0.128(9) -0.023(7) 0.082(9) -0.023(8)

C46A 0.131(12) 0.101(11) 0.182(19) -0.016(13) 0.090(13) 0.017(10)

O5A 0.071(3) 0.091(4) 0.089(5) -0.022(3) 0.021(3) -0.001(3)

O6A 0.076(4) 0.123(6) 0.095(6) -0.026(5) 0.047(4) -0.008(4)

P2A 0.0604(16) 0.089(2) 0.081(3) -0.0150(19) 0.027(2) -0.0081(13)

S2A 0.106(6) 0.125(7) 0.151(7) -0.005(4) 0.073(5) -0.021(4)

C24 0.067(2) 0.094(3) 0.075(2) -0.0162(19) 0.0263(17) -0.0002(18)

C25 0.115(3) 0.093(3) 0.083(3) -0.016(2) 0.037(2) -0.002(2)

C26 0.130(4) 0.089(3) 0.109(4) -0.024(3) 0.036(3) -0.004(3)

C27 0.126(4) 0.078(3) 0.128(4) -0.004(3) 0.049(3) 0.003(3)

C28 0.118(4) 0.108(4) 0.100(3) 0.008(3) 0.044(3) 0.015(3)

C29 0.089(3) 0.091(3) 0.076(2) -0.006(2) 0.0282(19) 0.010(2)

C30 0.071(2) 0.097(3) 0.0630(18) -0.0144(18) 0.0277(16) -0.0045(18)

_geom_special_details

;

All esds (except the esd in the dihedral angle between two l.s. planes)

are estimated using the full covariance matrix. The cell esds are taken

into account individually in the estimation of esds in distances, angles

and torsion angles; correlations between esds in cell parameters are only

used when they are defined by crystal symmetry. An approximate (isotropic)

treatment of cell esds is used for estimating esds involving l.s. planes.

;

loop_

_geom_bond_atom_site_label_1

_geom_bond_atom_site_label_2

_geom_bond_distance

_geom_bond_site_symmetry_2

_geom_bond_publ_flag

C1 C2 1.395(5) . ?

C1 C6 1.400(5) . ?

C1 C7 1.454(6) . ?

C2 C3 1.379(7) . ?

C2 H2 0.9300 . ?

C3 C4 1.366(7) . ?

C3 H3 0.9300 . ?

C4 C5 1.390(7) . ?

C4 H4 0.9300 . ?

C5 C6 1.359(7) . ?

C5 H5 0.9300 . ?

C6 H6 0.9300 . ?

C7 C9 1.340(5) . ?

C7 H7 0.9300 . ?

C8 N1 1.456(5) . ?

C8 C9 1.510(4) . ?

C8 H8A 0.9700 . ?

C8 H8B 0.9700 . ?

C9 C10 1.498(5) . ?

C10 O1 1.218(4) . ?

C10 C11 1.491(5) . ?

C11 C13 1.349(5) . ?

C11 C12 1.502(4) . ?

C12 N1 1.463(4) . ?

C12 H12A 0.9700 . ?

C12 H12B 0.9700 . ?

C13 C14 1.460(6) . ?

C13 H13 0.9300 . ?

C14 C19 1.383(6) . ?

C14 C15 1.398(6) . ?

C15 C16 1.377(6) . ?

C15 H15 0.9300 . ?

C16 C17 1.370(7) . ?

C16 H16 0.9300 . ?

C17 C18 1.382(8) . ?

C17 H17 0.9300 . ?

C18 C19 1.377(9) . ?

C18 H18 0.9300 . ?

C19 H19 0.9300 . ?

C31 N2 1.451(5) . ?

C31 C32 1.501(4) . ?

C31 H31A 0.9700 . ?

C31 H31B 0.9700 . ?

C32 C30 1.346(5) . ?

C32 C33 1.504(5) . ?

C33 O4 1.215(4) . ?

C33 C34 1.499(5) . ?

C34 C36 1.336(5) . ?

C34 C35 1.507(5) . ?

C35 N2 1.470(4) . ?

C35 H35A 0.9700 . ?

C35 H35B 0.9700 . ?

C36 C37 1.455(6) . ?

C36 H36 0.9300 . ?

C37 C38 1.390(6) . ?

C37 C42 1.391(5) . ?

C38 C39 1.390(7) . ?

C38 H38 0.9300 . ?

C39 C40 1.381(8) . ?

C39 H39 0.9300 . ?

C40 C41 1.380(7) . ?

C40 H40 0.9300 . ?

C41 C42 1.369(6) . ?

C41 H41 0.9300 . ?

C42 H42 0.9300 . ?

N1 P1 1.611(7) . ?

N1 P1A 1.648(12) . ?

N2 P2 1.608(8) . ?

N2 P2A 1.644(10) . ?

C20 O2 1.434(9) . ?

C20 C21 1.494(10) . ?

C20 H20A 0.9700 . ?

C20 H20B 0.9700 . ?

C21 H21A 0.9600 . ?

C21 H21B 0.9600 . ?

C21 H21C 0.9600 . ?

C22 C23 1.446(9) . ?

C22 H22A 0.9600 . ?

C22 H22B 0.9600 . ?

C22 H22C 0.9600 . ?

C23 O3 1.412(8) . ?

C23 H23A 0.9700 . ?

C23 H23B 0.9700 . ?

O2 P1 1.580(6) . ?

O3 P1 1.574(5) . ?

P1 S1 1.923(4) . ?

C20A O2A 1.431(10) . ?

C20A C21A 1.493(11) . ?

C20A H20C 0.9700 . ?

C20A H20D 0.9700 . ?

C21A H21D 0.9600 . ?

C21A H21E 0.9600 . ?

C21A H21F 0.9600 . ?

C23A C22A 1.452(10) . ?

C23A H23C 0.9600 . ?

C23A H23D 0.9600 . ?

C23A H23E 0.9600 . ?

C22A O3A 1.420(9) . ?

C22A H22D 0.9700 . ?

C22A H22E 0.9700 . ?

O2A P1A 1.579(8) . ?

O3A P1A 1.580(7) . ?

P1A S1A 1.927(7) . ?

C43 O5 1.441(9) . ?

C43 C44 1.450(11) . ?

C43 H43A 0.9700 . ?

C43 H43B 0.9700 . ?

C44 H44A 0.9600 . ?

C44 H44B 0.9600 . ?

C44 H44C 0.9600 . ?

C45 C46 1.400(13) . ?

C45 O6 1.444(11) . ?

C45 H45A 0.9700 . ?

C45 H45B 0.9700 . ?

C46 H46A 0.9600 . ?

C46 H46B 0.9600 . ?

C46 H46C 0.9600 . ?

O5 P2 1.575(6) . ?

O6 P2 1.576(6) . ?

P2 S2 1.919(5) . ?

C43A O5A 1.451(11) . ?

C43A C44A 1.453(12) . ?

C43A H43C 0.9700 . ?

C43A H43D 0.9700 . ?

C44A H44D 0.9600 . ?

C44A H44E 0.9600 . ?

C44A H44F 0.9600 . ?

C45A C46A 1.402(14) . ?

C45A O6A 1.432(12) . ?

C45A H45C 0.9700 . ?

C45A H45D 0.9700 . ?

C46A H46D 0.9600 . ?

C46A H46E 0.9600 . ?

C46A H46F 0.9600 . ?

O5A P2A 1.572(7) . ?

O6A P2A 1.568(7) . ?

P2A S2A 1.907(6) . ?

C24 C29 1.378(5) . ?

C24 C25 1.386(5) . ?

C24 C30 1.454(6) . ?

C25 C26 1.381(7) . ?

C25 H25 0.9300 . ?

C26 C27 1.354(7) . ?

C26 H26 0.9300 . ?

C27 C28 1.379(7) . ?

C27 H27 0.9300 . ?

C28 C29 1.374(7) . ?

C28 H28 0.9300 . ?

C29 H29 0.9300 . ?

C30 H30 0.9300 . ?

loop_

_geom_angle_atom_site_label_1

_geom_angle_atom_site_label_2

_geom_angle_atom_site_label_3

_geom_angle

_geom_angle_site_symmetry_1

_geom_angle_site_symmetry_3

_geom_angle_publ_flag

C2 C1 C6 116.4(4) . . ?

C2 C1 C7 118.5(4) . . ?

C6 C1 C7 125.0(4) . . ?

C3 C2 C1 121.3(4) . . ?

C3 C2 H2 119.3 . . ?

C1 C2 H2 119.3 . . ?

C4 C3 C2 120.7(5) . . ?

C4 C3 H3 119.7 . . ?

C2 C3 H3 119.7 . . ?

C3 C4 C5 119.2(5) . . ?

C3 C4 H4 120.4 . . ?

C5 C4 H4 120.4 . . ?

C6 C5 C4 120.0(5) . . ?

C6 C5 H5 120.0 . . ?

C4 C5 H5 120.0 . . ?

C5 C6 C1 122.3(4) . . ?

C5 C6 H6 118.9 . . ?

C1 C6 H6 118.9 . . ?

C9 C7 C1 130.0(3) . . ?

C9 C7 H7 115.0 . . ?

C1 C7 H7 115.0 . . ?

N1 C8 C9 109.5(3) . . ?

N1 C8 H8A 109.8 . . ?

C9 C8 H8A 109.8 . . ?

N1 C8 H8B 109.8 . . ?

C9 C8 H8B 109.8 . . ?

H8A C8 H8B 108.2 . . ?

C7 C9 C10 117.2(3) . . ?

C7 C9 C8 124.4(4) . . ?

C10 C9 C8 118.3(3) . . ?

O1 C10 C11 120.7(3) . . ?

O1 C10 C9 120.9(3) . . ?

C11 C10 C9 118.4(3) . . ?

C13 C11 C10 118.0(3) . . ?

C13 C11 C12 123.7(4) . . ?

C10 C11 C12 118.2(3) . . ?

N1 C12 C11 108.8(3) . . ?

N1 C12 H12A 109.9 . . ?

C11 C12 H12A 109.9 . . ?

N1 C12 H12B 109.9 . . ?

C11 C12 H12B 109.9 . . ?

H12A C12 H12B 108.3 . . ?

C11 C13 C14 129.6(4) . . ?

C11 C13 H13 115.2 . . ?

C14 C13 H13 115.2 . . ?

C19 C14 C15 116.9(5) . . ?

C19 C14 C13 119.5(4) . . ?

C15 C14 C13 123.6(4) . . ?

C16 C15 C14 122.0(4) . . ?

C16 C15 H15 119.0 . . ?

C14 C15 H15 119.0 . . ?

C17 C16 C15 120.3(5) . . ?

C17 C16 H16 119.9 . . ?

C15 C16 H16 119.9 . . ?

C16 C17 C18 118.4(6) . . ?

C16 C17 H17 120.8 . . ?

C18 C17 H17 120.8 . . ?

C19 C18 C17 121.6(5) . . ?

C19 C18 H18 119.2 . . ?

C17 C18 H18 119.2 . . ?

C18 C19 C14 120.8(5) . . ?

C18 C19 H19 119.6 . . ?

C14 C19 H19 119.6 . . ?

N2 C31 C32 111.4(3) . . ?

N2 C31 H31A 109.4 . . ?

C32 C31 H31A 109.4 . . ?

N2 C31 H31B 109.4 . . ?

C32 C31 H31B 109.4 . . ?

H31A C31 H31B 108.0 . . ?

C30 C32 C31 124.9(3) . . ?

C30 C32 C33 116.7(3) . . ?

C31 C32 C33 118.4(3) . . ?

O4 C33 C34 120.4(3) . . ?

O4 C33 C32 121.3(3) . . ?

C34 C33 C32 118.2(3) . . ?

C36 C34 C33 117.9(3) . . ?

C36 C34 C35 124.3(3) . . ?

C33 C34 C35 117.6(3) . . ?

N2 C35 C34 109.9(3) . . ?

N2 C35 H35A 109.7 . . ?

C34 C35 H35A 109.7 . . ?

N2 C35 H35B 109.7 . . ?

C34 C35 H35B 109.7 . . ?

H35A C35 H35B 108.2 . . ?

C34 C36 C37 130.2(3) . . ?

C34 C36 H36 114.9 . . ?

C37 C36 H36 114.9 . . ?

C38 C37 C42 117.4(4) . . ?

C38 C37 C36 119.3(4) . . ?

C42 C37 C36 123.2(4) . . ?

C37 C38 C39 121.3(5) . . ?

C37 C38 H38 119.3 . . ?

C39 C38 H38 119.3 . . ?

C40 C39 C38 120.2(5) . . ?

C40 C39 H39 119.9 . . ?

C38 C39 H39 119.9 . . ?

C41 C40 C39 118.5(5) . . ?

C41 C40 H40 120.7 . . ?

C39 C40 H40 120.7 . . ?

C42 C41 C40 121.4(5) . . ?

C42 C41 H41 119.3 . . ?

C40 C41 H41 119.3 . . ?

C41 C42 C37 121.1(4) . . ?

C41 C42 H42 119.5 . . ?

C37 C42 H42 119.5 . . ?

C8 N1 C12 113.2(3) . . ?

C8 N1 P1 124.6(3) . . ?

C12 N1 P1 122.2(3) . . ?

C8 N1 P1A 129.1(5) . . ?

C12 N1 P1A 117.5(5) . . ?

C31 N2 C35 112.3(3) . . ?

C31 N2 P2 124.8(3) . . ?

C35 N2 P2 119.5(3) . . ?

C31 N2 P2A 128.0(3) . . ?

C35 N2 P2A 119.6(3) . . ?

O2 C20 C21 110.2(8) . . ?

O2 C20 H20A 109.6 . . ?

C21 C20 H20A 109.6 . . ?

O2 C20 H20B 109.6 . . ?

C21 C20 H20B 109.6 . . ?

H20A C20 H20B 108.1 . . ?

C20 C21 H21A 109.5 . . ?

C20 C21 H21B 109.5 . . ?

H21A C21 H21B 109.5 . . ?

C20 C21 H21C 109.5 . . ?

H21A C21 H21C 109.5 . . ?

H21B C21 H21C 109.5 . . ?

C23 C22 H22A 109.5 . . ?

C23 C22 H22B 109.5 . . ?

H22A C22 H22B 109.5 . . ?

C23 C22 H22C 109.5 . . ?

H22A C22 H22C 109.5 . . ?

H22B C22 H22C 109.5 . . ?

O3 C23 C22 112.4(8) . . ?

O3 C23 H23A 109.1 . . ?

C22 C23 H23A 109.1 . . ?

O3 C23 H23B 109.1 . . ?

C22 C23 H23B 109.1 . . ?

H23A C23 H23B 107.8 . . ?

C20 O2 P1 120.4(7) . . ?

C23 O3 P1 124.1(8) . . ?

O3 P1 O2 98.9(4) . . ?

O3 P1 N1 101.7(5) . . ?

O2 P1 N1 106.4(5) . . ?

O3 P1 S1 116.1(5) . . ?

O2 P1 S1 116.7(5) . . ?

N1 P1 S1 114.8(6) . . ?

O2A C20A C21A 109.8(12) . . ?

O2A C20A H20C 109.7 . . ?

C21A C20A H20C 109.7 . . ?

O2A C20A H20D 109.7 . . ?

C21A C20A H20D 109.7 . . ?

H20C C20A H20D 108.2 . . ?

C20A C21A H21D 109.5 . . ?

C20A C21A H21E 109.5 . . ?

H21D C21A H21E 109.5 . . ?

C20A C21A H21F 109.5 . . ?

H21D C21A H21F 109.5 . . ?

H21E C21A H21F 109.5 . . ?

C22A C23A H23C 109.5 . . ?

C22A C23A H23D 109.5 . . ?

H23C C23A H23D 109.5 . . ?

C22A C23A H23E 109.5 . . ?

H23C C23A H23E 109.5 . . ?

H23D C23A H23E 109.5 . . ?

O3A C22A C23A 111.3(10) . . ?

O3A C22A H22D 109.4 . . ?

C23A C22A H22D 109.4 . . ?

O3A C22A H22E 109.4 . . ?

C23A C22A H22E 109.4 . . ?

H22D C22A H22E 108.0 . . ?

C20A O2A P1A 121.8(12) . . ?

C22A O3A P1A 123.4(10) . . ?

O2A P1A O3A 98.1(8) . . ?

O2A P1A N1 107.0(11) . . ?

O3A P1A N1 106.2(7) . . ?

O2A P1A S1A 115.4(8) . . ?

O3A P1A S1A 116.1(8) . . ?

N1 P1A S1A 112.7(10) . . ?

O5 C43 C44 112.2(11) . . ?

O5 C43 H43A 109.2 . . ?

C44 C43 H43A 109.2 . . ?

O5 C43 H43B 109.2 . . ?

C44 C43 H43B 109.2 . . ?

H43A C43 H43B 107.9 . . ?

C43 C44 H44A 109.5 . . ?

C43 C44 H44B 109.5 . . ?

H44A C44 H44B 109.5 . . ?

C43 C44 H44C 109.5 . . ?

H44A C44 H44C 109.5 . . ?

H44B C44 H44C 109.5 . . ?

C46 C45 O6 113.4(11) . . ?

C46 C45 H45A 108.9 . . ?

O6 C45 H45A 108.9 . . ?

C46 C45 H45B 108.9 . . ?

O6 C45 H45B 108.9 . . ?

H45A C45 H45B 107.7 . . ?

C45 C46 H46A 109.5 . . ?

C45 C46 H46B 109.5 . . ?

H46A C46 H46B 109.5 . . ?

C45 C46 H46C 109.5 . . ?

H46A C46 H46C 109.5 . . ?

H46B C46 H46C 109.5 . . ?

C43 O5 P2 120.9(7) . . ?

C45 O6 P2 120.0(7) . . ?

O5 P2 O6 99.4(4) . . ?

O5 P2 N2 100.0(4) . . ?

O6 P2 N2 106.8(4) . . ?

O5 P2 S2 117.1(5) . . ?

O6 P2 S2 115.8(5) . . ?

N2 P2 S2 115.4(4) . . ?

O5A C43A C44A 111.1(13) . . ?

O5A C43A H43C 109.4 . . ?

C44A C43A H43C 109.4 . . ?

O5A C43A H43D 109.4 . . ?

C44A C43A H43D 109.4 . . ?

H43C C43A H43D 108.0 . . ?

C43A C44A H44D 109.5 . . ?

C43A C44A H44E 109.5 . . ?

H44D C44A H44E 109.5 . . ?

C43A C44A H44F 109.5 . . ?

H44D C44A H44F 109.5 . . ?

H44E C44A H44F 109.5 . . ?

C46A C45A O6A 114.4(12) . . ?

C46A C45A H45C 108.7 . . ?

O6A C45A H45C 108.7 . . ?

C46A C45A H45D 108.7 . . ?

O6A C45A H45D 108.7 . . ?

H45C C45A H45D 107.6 . . ?

C45A C46A H46D 109.5 . . ?

C45A C46A H46E 109.5 . . ?

H46D C46A H46E 109.5 . . ?

C45A C46A H46F 109.5 . . ?

H46D C46A H46F 109.5 . . ?

H46E C46A H46F 109.5 . . ?

C43A O5A P2A 120.4(7) . . ?

C45A O6A P2A 120.7(9) . . ?

O6A P2A O5A 100.0(5) . . ?

O6A P2A N2 99.9(5) . . ?

O5A P2A N2 110.1(5) . . ?

O6A P2A S2A 115.1(6) . . ?

O5A P2A S2A 116.1(6) . . ?

N2 P2A S2A 113.7(6) . . ?

C29 C24 C25 117.3(4) . . ?

C29 C24 C30 124.7(4) . . ?

C25 C24 C30 118.0(4) . . ?

C26 C25 C24 121.3(4) . . ?

C26 C25 H25 119.3 . . ?

C24 C25 H25 119.3 . . ?

C27 C26 C25 120.4(5) . . ?

C27 C26 H26 119.8 . . ?

C25 C26 H26 119.8 . . ?

C26 C27 C28 119.4(5) . . ?

C26 C27 H27 120.3 . . ?

C28 C27 H27 120.3 . . ?

C29 C28 C27 120.2(5) . . ?

C29 C28 H28 119.9 . . ?

C27 C28 H28 119.9 . . ?

C28 C29 C24 121.3(4) . . ?

C28 C29 H29 119.3 . . ?

C24 C29 H29 119.3 . . ?

C32 C30 C24 130.0(3) . . ?

C32 C30 H30 115.0 . . ?

C24 C30 H30 115.0 . . ?

loop_

_geom_torsion_atom_site_label_1

_geom_torsion_atom_site_label_2

_geom_torsion_atom_site_label_3

_geom_torsion_atom_site_label_4

_geom_torsion

_geom_torsion_site_symmetry_1

_geom_torsion_site_symmetry_2

_geom_torsion_site_symmetry_3

_geom_torsion_site_symmetry_4

_geom_torsion_publ_flag

C6 C1 C2 C3 -2.1(6) . . . . ?

C7 C1 C2 C3 -178.6(4) . . . . ?

C1 C2 C3 C4 1.9(7) . . . . ?

C2 C3 C4 C5 -0.8(8) . . . . ?

C3 C4 C5 C6 0.0(8) . . . . ?

C4 C5 C6 C1 -0.3(8) . . . . ?

C2 C1 C6 C5 1.4(6) . . . . ?

C7 C1 C6 C5 177.6(4) . . . . ?

C2 C1 C7 C9 -151.6(4) . . . . ?

C6 C1 C7 C9 32.2(6) . . . . ?

C1 C7 C9 C10 -176.8(3) . . . . ?

C1 C7 C9 C8 6.6(6) . . . . ?

N1 C8 C9 C7 148.2(3) . . . . ?

N1 C8 C9 C10 -28.4(4) . . . . ?

C7 C9 C10 O1 5.3(5) . . . . ?

C8 C9 C10 O1 -177.8(3) . . . . ?

C7 C9 C10 C11 -175.9(3) . . . . ?

C8 C9 C10 C11 0.9(5) . . . . ?

O1 C10 C11 C13 -8.0(5) . . . . ?

C9 C10 C11 C13 173.2(3) . . . . ?

O1 C10 C11 C12 175.3(3) . . . . ?

C9 C10 C11 C12 -3.5(5) . . . . ?

C13 C11 C12 N1 -143.4(4) . . . . ?

C10 C11 C12 N1 33.1(4) . . . . ?

C10 C11 C13 C14 178.4(4) . . . . ?

C12 C11 C13 C14 -5.2(6) . . . . ?

C11 C13 C14 C19 149.8(4) . . . . ?

C11 C13 C14 C15 -33.0(6) . . . . ?

C19 C14 C15 C16 -0.2(6) . . . . ?

C13 C14 C15 C16 -177.5(4) . . . . ?

C14 C15 C16 C17 -2.1(7) . . . . ?

C15 C16 C17 C18 2.1(8) . . . . ?

C16 C17 C18 C19 0.2(9) . . . . ?

C17 C18 C19 C14 -2.5(9) . . . . ?

C15 C14 C19 C18 2.5(7) . . . . ?

C13 C14 C19 C18 179.9(5) . . . . ?

N2 C31 C32 C30 -151.4(3) . . . . ?

N2 C31 C32 C33 27.0(4) . . . . ?

C30 C32 C33 O4 -2.2(5) . . . . ?

C31 C32 C33 O4 179.2(3) . . . . ?

C30 C32 C33 C34 178.8(3) . . . . ?

C31 C32 C33 C34 0.3(4) . . . . ?

O4 C33 C34 C36 10.0(5) . . . . ?

C32 C33 C34 C36 -171.0(3) . . . . ?

O4 C33 C34 C35 -175.4(3) . . . . ?

C32 C33 C34 C35 3.6(4) . . . . ?

C36 C34 C35 N2 140.4(3) . . . . ?

C33 C34 C35 N2 -33.8(4) . . . . ?

C33 C34 C36 C37 -179.7(3) . . . . ?

C35 C34 C36 C37 6.1(6) . . . . ?

C34 C36 C37 C38 -149.0(4) . . . . ?

C34 C36 C37 C42 34.4(6) . . . . ?

C42 C37 C38 C39 -2.2(6) . . . . ?

C36 C37 C38 C39 -179.1(4) . . . . ?

C37 C38 C39 C40 1.2(8) . . . . ?

C38 C39 C40 C41 0.5(8) . . . . ?

C39 C40 C41 C42 -0.9(8) . . . . ?

C40 C41 C42 C37 -0.2(7) . . . . ?

C38 C37 C42 C41 1.7(6) . . . . ?

C36 C37 C42 C41 178.5(4) . . . . ?

C9 C8 N1 C12 62.5(4) . . . . ?

C9 C8 N1 P1 -118.6(4) . . . . ?

C9 C8 N1 P1A -111.8(6) . . . . ?

C11 C12 N1 C8 -64.9(4) . . . . ?

C11 C12 N1 P1 116.2(4) . . . . ?

C11 C12 N1 P1A 110.0(6) . . . . ?

C32 C31 N2 C35 -60.4(4) . . . . ?

C32 C31 N2 P2 98.5(4) . . . . ?

C32 C31 N2 P2A 115.7(4) . . . . ?

C34 C35 N2 C31 63.9(4) . . . . ?

C34 C35 N2 P2 -96.3(4) . . . . ?

C34 C35 N2 P2A -112.6(4) . . . . ?

C21 C20 O2 P1 165.1(14) . . . . ?

C22 C23 O3 P1 112.7(17) . . . . ?

C23 O3 P1 O2 -79.2(10) . . . . ?

C23 O3 P1 N1 171.9(9) . . . . ?

C23 O3 P1 S1 46.5(11) . . . . ?

C20 O2 P1 O3 -174.3(15) . . . . ?

C20 O2 P1 N1 -69.2(15) . . . . ?

C20 O2 P1 S1 60.5(16) . . . . ?

C8 N1 P1 O3 -119.4(5) . . . . ?

C12 N1 P1 O3 59.4(6) . . . . ?

C8 N1 P1 O2 137.5(4) . . . . ?

C12 N1 P1 O2 -43.7(5) . . . . ?

C8 N1 P1 S1 6.8(7) . . . . ?

C12 N1 P1 S1 -174.4(6) . . . . ?

C21A C20A O2A P1A 180(3) . . . . ?

C23A C22A O3A P1A -179(2) . . . . ?

C20A O2A P1A O3A 159(3) . . . . ?

C20A O2A P1A N1 -92(3) . . . . ?

C20A O2A P1A S1A 34(3) . . . . ?

C22A O3A P1A O2A -68.1(19) . . . . ?

C22A O3A P1A N1 -178.6(17) . . . . ?

C22A O3A P1A S1A 55(2) . . . . ?

C8 N1 P1A O2A 132.1(8) . . . . ?

C12 N1 P1A O2A -42.0(8) . . . . ?

C8 N1 P1A O3A -123.9(9) . . . . ?

C12 N1 P1A O3A 62.0(9) . . . . ?

C8 N1 P1A S1A 4.2(11) . . . . ?

C12 N1 P1A S1A -169.8(8) . . . . ?

C44 C43 O5 P2 160.0(13) . . . . ?

C46 C45 O6 P2 -162.3(18) . . . . ?

C43 O5 P2 O6 -66.9(11) . . . . ?

C43 O5 P2 N2 -175.9(10) . . . . ?

C43 O5 P2 S2 58.6(12) . . . . ?

C45 O6 P2 O5 174.9(9) . . . . ?

C45 O6 P2 N2 -81.6(9) . . . . ?

C45 O6 P2 S2 48.5(11) . . . . ?

C31 N2 P2 O5 -105.0(4) . . . . ?

C35 N2 P2 O5 52.6(4) . . . . ?

C31 N2 P2 O6 151.9(3) . . . . ?

C35 N2 P2 O6 -50.5(4) . . . . ?

C31 N2 P2 S2 21.5(6) . . . . ?

C35 N2 P2 S2 179.1(4) . . . . ?

C44A C43A O5A P2A -165.3(17) . . . . ?

C46A C45A O6A P2A -114.6(18) . . . . ?

C45A O6A P2A O5A 76.4(13) . . . . ?

C45A O6A P2A N2 -171.0(12) . . . . ?

C45A O6A P2A S2A -48.8(15) . . . . ?

C43A O5A P2A O6A -178.6(9) . . . . ?

C43A O5A P2A N2 76.9(9) . . . . ?

C43A O5A P2A S2A -54.1(11) . . . . ?

C31 N2 P2A O6A 120.3(5) . . . . ?

C35 N2 P2A O6A -63.8(5) . . . . ?

C31 N2 P2A O5A -135.1(4) . . . . ?

C35 N2 P2A O5A 40.8(5) . . . . ?

C31 N2 P2A S2A -2.8(7) . . . . ?

C35 N2 P2A S2A 173.1(5) . . . . ?

C29 C24 C25 C26 0.4(7) . . . . ?

C30 C24 C25 C26 178.3(4) . . . . ?

C24 C25 C26 C27 0.3(8) . . . . ?

C25 C26 C27 C28 -1.1(9) . . . . ?

C26 C27 C28 C29 1.2(8) . . . . ?

C27 C28 C29 C24 -0.5(8) . . . . ?

C25 C24 C29 C28 -0.3(6) . . . . ?

C30 C24 C29 C28 -178.0(4) . . . . ?

C31 C32 C30 C24 -4.8(6) . . . . ?

C33 C32 C30 C24 176.8(3) . . . . ?

C29 C24 C30 C32 -31.5(6) . . . . ?

C25 C24 C30 C32 150.8(4) . . . . ?

loop_

_geom_hbond_atom_site_label_D

_geom_hbond_atom_site_label_H

_geom_hbond_atom_site_label_A

_geom_hbond_distance_DH

_geom_hbond_distance_HA

_geom_hbond_distance_DA

_geom_hbond_angle_DHA

_geom_hbond_site_symmetry_A

_geom_hbond_publ_flag

C8 H8A O1 0.97 2.61 3.376(5) 135.8 4_566 yes

C8 H8B S1^a 0.97 2.70 3.295(16) 119.9 . yes

C8 H8B S1A^b 0.97 2.83 3.37(2) 115.9 . yes

C31 H31A S2^a 0.97 2.78 3.347(9) 117.9 . yes

C31 H31A S2A^b 0.97 2.80 3.360(14) 117.7 . yes

C21^a H21B^a S1^a 0.96 3.01 3.86(3) 149.5 4_565 yes

C23^a H23A^a S1^a 0.97 2.96 3.448(16) 112.1 . yes

C23A^b H23E^b S1A^b 0.96 2.65 3.54(3) 154.5 4_566 yes

C43^a H43B^a S1^a 0.97 2.89 3.549(18) 125.9 4_465 yes

C44^a H44C^a S2^a 0.96 2.98 3.85(3) 151.6 4_565 yes

C45^a H45A^a S2^a 0.97 2.87 3.393(16) 114.6 . yes

C46^a H46B^a S2^a 0.96 2.81 3.76(2) 170.5 4_566 yes

C45A^b H45C^b S2A^b 0.97 2.87 3.370(19) 112.8 . yes

_refine_diff_density_max 0.335

_refine_diff_density_min -0.331

_refine_diff_density_rms 0.052

_shelx_res_file

;

TITL bmk2342b in P21/c

shelx.res

created by SHELXL-2019/3 at 08:58:11 on 02-Apr-2025

CELL 0.71073 15.9985 23.6855 12.8351 90.000 112.788 90.000

ZERR 8.00 0.0006 0.0008 0.0005 0.000 0.004 0.000

LATT 1

SYMM - X, 1/2 + Y, 1/2 - Z

SFAC C H N O P S

UNIT 184 208 8 24 8 8

MERG 2

rem EXTI 0.00202

simu c43 > s2a

isor c43 > s2a

simu c20 > s1a

isor c20 > s1a

MPLA C1 C2 C3 C4 C5 C6 C7

MPLA 6 C8 C9 C10 C11 C12 o1 N1

MPLA C13 C14 C15 C16 C17 C18 C19

MPLA C24 C25 C26 C27 C28 C29 C30

MPLA 6 C31 C32 C33 C34 C35 o4 N2

MPLA C36 C37 C38 C39 C40 C41 C42

EQIV $1 x, -y+1/2, z+1/2

HTAB C8 O1_$1

HTAB C8 S1

HTAB C8 S1A

HTAB C31 S2

HTAB C31 S2A

EQIV $2 x, -y+1/2, z-1/2

HTAB C21 S1_$2

HTAB C23 S1

HTAB C23A S1A_$1

EQIV $3 x-1, -y+1/2, z-1/2

HTAB C43 S1_$3

HTAB C44 S2_$2

HTAB C45 S2

HTAB C46 S2_$1

HTAB C45A S2A

FMAP 2

PLAN 10

SIZE 0.280 0.410 0.440

ACTA

BOND $H

CONF

LIST 4

L.S. 4

WGHT 0.127300 1.865300

FVAR 2.66247 0.54001 0.60070

C1 1 0.872623 0.106006 0.086600 11.00000 0.05833 0.09728 =

0.06929 -0.01942 0.01950 -0.00307

C2 1 0.900869 0.066352 0.026686 11.00000 0.08863 0.10123 =

0.09537 -0.02346 0.04235 -0.00467

AFIX 43

H2 2 0.913870 0.078122 -0.034529 11.00000 -1.20000

AFIX 0

C3 1 0.909896 0.010047 0.056430 11.00000 0.10710 0.10732 =

0.13143 -0.03663 0.05508 -0.00512

AFIX 43

H3 2 0.930541 -0.015364 0.016515 11.00000 -1.20000

AFIX 0

C4 1 0.888833 -0.008782 0.143887 11.00000 0.10583 0.10271 =

0.12512 -0.01467 0.03958 -0.01154

AFIX 43

H4 2 0.894340 -0.046859 0.163063 11.00000 -1.20000

AFIX 0

C5 1 0.859063 0.029649 0.203906 11.00000 0.11936 0.10923 =

0.10316 -0.01371 0.04553 -0.01806

AFIX 43

H5 2 0.844528 0.017326 0.263627 11.00000 -1.20000

AFIX 0

C6 1 0.851196 0.085227 0.175323 11.00000 0.09100 0.10316 =

0.08191 -0.01756 0.03787 -0.00681

AFIX 43

H6 2 0.830875 0.110312 0.216128 11.00000 -1.20000

AFIX 0

C7 1 0.862487 0.164453 0.049104 11.00000 0.06328 0.11187 =

0.05774 -0.01301 0.02241 0.00112

AFIX 43

H7 2 0.848121 0.169450 -0.027778 11.00000 -1.20000

AFIX 0

C8 1 0.902799 0.215019 0.235253 11.00000 0.08225 0.07863 =

0.05889 -0.00347 0.02299 0.00107

AFIX 23

H8A 2 0.851284 0.212670 0.257159 11.00000 -1.20000

H8B 2 0.942718 0.183429 0.268972 11.00000 -1.20000

AFIX 0

C9 1 0.870633 0.212241 0.108143 11.00000 0.05764 0.09774 =

0.05395 -0.00626 0.01915 0.00337

C10 1 0.850932 0.266517 0.043141 11.00000 0.06545 0.09934 =

0.05423 0.00099 0.02083 0.01111

C11 1 0.865443 0.320586 0.107253 11.00000 0.06037 0.09341 =

0.06165 0.00698 0.02064 0.01107

C12 1 0.893881 0.317752 0.233194 11.00000 0.06872 0.08199 =

0.06188 0.00223 0.01825 0.01030

AFIX 23

H12A 2 0.927370 0.351532 0.267927 11.00000 -1.20000

H12B 2 0.840815 0.315370 0.251997 11.00000 -1.20000

AFIX 0

C13 1 0.856757 0.369249 0.049325 11.00000 0.07557 0.10800 =

0.07088 0.01575 0.02553 0.01565

AFIX 43

H13 2 0.843636 0.365060 -0.027458 11.00000 -1.20000

AFIX 0

C14 1 0.864893 0.427516 0.089262 11.00000 0.06790 0.09749 =

0.08319 0.02587 0.02471 0.01249

C15 1 0.839835 0.445297 0.176932 11.00000 0.07478 0.08480 =

0.10850 0.01980 0.03866 0.00672

AFIX 43

H15 2 0.818887 0.418685 0.214346 11.00000 -1.20000

AFIX 0

C16 1 0.845206 0.501006 0.209618 11.00000 0.10088 0.08709 =

0.12844 0.01696 0.04516 0.01036

AFIX 43

H16 2 0.826102 0.511620 0.266610 11.00000 -1.20000

AFIX 0

C17 1 0.878567 0.540849 0.158545 11.00000 0.11447 0.09063 =

0.14791 0.03053 0.04016 0.00755

AFIX 43

H17 2 0.884250 0.578344 0.181777 11.00000 -1.20000

AFIX 0

C18 1 0.903574 0.523960 0.071646 11.00000 0.12445 0.13362 =

0.14868 0.06252 0.05373 -0.00039

AFIX 43

H18 2 0.926494 0.550577 0.036412 11.00000 -1.20000

AFIX 0

C19 1 0.895355 0.468637 0.035983 11.00000 0.12139 0.11480 =

0.11078 0.04148 0.05152 0.01577

AFIX 43

H19 2 0.910482 0.458820 -0.024682 11.00000 -1.20000

AFIX 0

C31 1 0.597729 0.211027 0.330828 11.00000 0.07361 0.07629 =

0.07337 -0.00190 0.03678 -0.00290

AFIX 23

H31A 2 0.556615 0.179775 0.323351 11.00000 -1.20000

H31B 2 0.648821 0.207000 0.402495 11.00000 -1.20000

AFIX 0

C32 1 0.630840 0.208816 0.236303 11.00000 0.05895 0.08318 =

0.05960 -0.00677 0.01997 -0.00146

C33 1 0.651614 0.263523 0.191918 11.00000 0.06302 0.08748 =

0.05869 -0.00831 0.02425 -0.00951

C34 1 0.638531 0.317445 0.244818 11.00000 0.05544 0.08294 =

0.06742 -0.00402 0.02387 -0.01017

C35 1 0.610612 0.313084 0.343997 11.00000 0.06717 0.07919 =

0.06945 -0.00986 0.03015 -0.00880

AFIX 23

H35A 2 0.664037 0.309385 0.413327 11.00000 -1.20000

H35B 2 0.578720 0.347117 0.349190 11.00000 -1.20000

AFIX 0

C36 1 0.644967 0.365966 0.195320 11.00000 0.06959 0.09898 =

0.06822 -0.00021 0.02738 -0.01193

AFIX 43

H36 2 0.657055 0.362032 0.130413 11.00000 -1.20000

AFIX 0

C37 1 0.636065 0.423963 0.226868 11.00000 0.06316 0.08277 =

0.08492 0.00916 0.02904 -0.00266

C38 1 0.602260 0.464634 0.142846 11.00000 0.09627 0.10280 =

0.09490 0.01885 0.03465 -0.00651

AFIX 43

H38 2 0.584991 0.454110 0.067583 11.00000 -1.20000

AFIX 0

C39 1 0.593744 0.520720 0.169050 11.00000 0.10492 0.10192 =

0.14097 0.04458 0.04647 0.01231

AFIX 43

H39 2 0.569935 0.547124 0.111433 11.00000 -1.20000

AFIX 0

C40 1 0.620534 0.537337 0.280378 11.00000 0.10255 0.08004 =

0.14854 0.00484 0.05077 -0.00070

AFIX 43

H40 2 0.614675 0.574726 0.298624 11.00000 -1.20000

AFIX 0

C41 1 0.656206 0.497240 0.364016 11.00000 0.09846 0.08761 =

0.11115 -0.00490 0.03972 -0.00783

AFIX 43

H41 2 0.675305 0.508055 0.439344 11.00000 -1.20000

AFIX 0

C42 1 0.664069 0.441874 0.338421 11.00000 0.07693 0.07405 =

0.08496 0.00090 0.02356 -0.00416

AFIX 43

H42 2 0.688537 0.415827 0.396594 11.00000 -1.20000

AFIX 0

N1 3 0.950960 0.267881 0.275563 11.00000 0.07695 0.07923 =

0.05984 -0.00276 0.00992 0.00870

N2 3 0.551403 0.263790 0.330100 11.00000 0.06423 0.07652 =

0.08658 -0.00607 0.03772 -0.00720

O1 4 0.822313 0.266569 -0.059692 11.00000 0.10671 0.12252 =

0.05643 0.00402 0.02343 0.01841

O4 4 0.679497 0.264367 0.116300 11.00000 0.10848 0.10438 =

0.08048 -0.01192 0.05701 -0.01473

PART 1

C20 1 1.110899 0.314373 0.209003 31.00000 0.09457 0.15372 =

0.07970 -0.01411 0.04057 0.01807

AFIX 23

H20A 2 1.057074 0.297415 0.152914 31.00000 -1.20000

H20B 2 1.161670 0.289258 0.220886 31.00000 -1.20000

AFIX 0

C21 1 1.128491 0.370138 0.167083 31.00000 0.11646 0.15343 =

0.11893 0.03967 0.07567 0.03208

AFIX 137

H21A 2 1.075319 0.393334 0.146740 31.00000 -1.50000

H21B 2 1.143204 0.364474 0.102081 31.00000 -1.50000

H21C 2 1.178257 0.388452 0.225482 31.00000 -1.50000

AFIX 0

C22 1 1.147392 0.371721 0.588444 31.00000 0.11009 0.07613 =

0.12559 -0.01158 0.01387 -0.00118

AFIX 137

H22A 2 1.166859 0.385249 0.530980 31.00000 -1.50000

H22B 2 1.195990 0.375586 0.661145 31.00000 -1.50000

H22C 2 1.096169 0.393365 0.586823 31.00000 -1.50000

AFIX 0

C23 1 1.122016 0.312906 0.567931 31.00000 0.12857 0.07754 =

0.07430 -0.00196 0.01080 -0.00665

AFIX 23

H23A 2 1.173721 0.291342 0.568451 31.00000 -1.20000

H23B 2 1.105808 0.298871 0.628616 31.00000 -1.20000

AFIX 0

O2 4 1.098413 0.321492 0.312991 31.00000 0.07414 0.09962 =

0.06365 -0.01748 0.02424 -0.00077

O3 4 1.048463 0.304422 0.463793 31.00000 0.08567 0.10274 =

0.05369 -0.00379 0.02189 0.00473

P1 5 1.055856 0.272491 0.360149 31.00000 0.07402 0.07585 =

0.04961 -0.00890 0.01146 0.01189

S1 6 1.120364 0.201965 0.393268 31.00000 0.11287 0.10381 =

0.09700 -0.00377 -0.00402 0.04524

PART 2

same .01 c20 > s1

C20A 1 1.128725 0.315188 0.208033 -31.00000 0.10626 0.13996 =

0.11483 -0.00388 0.06375 0.03986

AFIX 23

H20C 2 1.084765 0.294443 0.145654 -31.00000 -1.20000

H20D 2 1.182585 0.292009 0.241167 -31.00000 -1.20000

AFIX 0

C21A 1 1.152305 0.369120 0.165590 -31.00000 0.15804 0.19460 =

0.17699 0.01288 0.10502 0.01109

AFIX 137

H21D 2 1.101836 0.394624 0.145157 -31.00000 -1.50000

H21E 2 1.166175 0.361540 0.100561 -31.00000 -1.50000

H21F 2 1.204064 0.385865 0.223769 -31.00000 -1.50000

AFIX 0

C23A 1 1.132104 0.359745 0.634908 -31.00000 0.10823 0.08226 =

0.07169 0.00458 0.02097 0.01275

AFIX 137

H23C 2 1.081593 0.384993 0.603172 -31.00000 -1.50000

H23D 2 1.185236 0.380869 0.679178 -31.00000 -1.50000

H23E 2 1.119166 0.332594 0.682167 -31.00000 -1.50000

AFIX 0

C22A 1 1.147704 0.330682 0.544501 -31.00000 0.07392 0.11520 =

0.07579 -0.01046 0.01160 -0.00870

AFIX 23

H22D 2 1.178532 0.355871 0.511471 -31.00000 -1.20000

H22E 2 1.186628 0.298314 0.575247 -31.00000 -1.20000

AFIX 0

O2A 4 1.091825 0.327010 0.290963 -31.00000 0.11073 0.13797 =

0.13099 0.03949 0.05917 0.03014

O3A 4 1.064962 0.312339 0.459280 -31.00000 0.08555 0.11543 =

0.09306 -0.04279 -0.00645 0.00863

P1A 5 1.059623 0.278361 0.350959 -31.00000 0.06949 0.09362 =

0.09149 0.01240 0.01357 0.00821

S1A 6 1.129487 0.209696 0.378293 -31.00000 0.09052 0.09698 =

0.09977 0.00202 0.01586 0.02128

PART 1

C43 1 0.343850 0.325080 0.090591 21.00000 0.05892 0.13981 =

0.09362 0.02170 0.00989 0.00093

AFIX 23

H43A 2 0.311474 0.339436 0.135172 21.00000 -1.20000

H43B 2 0.309051 0.293973 0.045206 21.00000 -1.20000

AFIX 0

C44 1 0.350943 0.369396 0.016495 21.00000 0.09980 0.10360 =

0.09981 -0.00332 0.01285 -0.00001

AFIX 137

H44A 2 0.386297 0.399991 0.061061 21.00000 -1.50000

H44B 2 0.291420 0.382782 -0.029711 21.00000 -1.50000

H44C 2 0.379810 0.354754 -0.030919 21.00000 -1.50000

AFIX 0

C45 1 0.416391 0.310295 0.452864 21.00000 0.11037 0.13688 =

0.09480 0.01926 0.04012 0.02813

AFIX 23

H45A 2 0.385204 0.274722 0.448659 21.00000 -1.20000

H45B 2 0.477410 0.306346 0.509551 21.00000 -1.20000

AFIX 0

C46 1 0.371419 0.352640 0.487070 21.00000 0.20518 0.14320 =

0.17056 0.03155 0.12442 0.06061

AFIX 137

H46A 2 0.404991 0.387258 0.498305 21.00000 -1.50000

H46B 2 0.366577 0.341671 0.556521 21.00000 -1.50000

H46C 2 0.311860 0.358019 0.429834 21.00000 -1.50000

AFIX 0

O5 4 0.431422 0.304633 0.165364 21.00000 0.05990 0.11802 =

0.06715 0.00956 0.02278 -0.00307

O6 4 0.421400 0.321432 0.344803 21.00000 0.07713 0.09106 =

0.07483 0.00992 0.04008 0.01201

P2 5 0.443205 0.271996 0.277094 21.00000 0.05812 0.08756 =

0.07401 -0.00189 0.02743 -0.00717

S2 6 0.373632 0.204231 0.261453 21.00000 0.07134 0.08956 =

0.11281 -0.01225 0.02811 -0.03014

PART 2

same .01 c43 > s2

C43A 1 0.379802 0.316707 0.104939 -21.00000 0.06689 0.11421 =

0.09916 -0.01017 0.00626 -0.00761

AFIX 23

H43C 2 0.324567 0.294644 0.073288 -21.00000 -1.20000

H43D 2 0.427604 0.296179 0.092538 -21.00000 -1.20000

AFIX 0

C44A 1 0.366386 0.370958 0.047898 -21.00000 0.14899 0.10243 =

0.08735 0.01460 -0.00450 -0.01331

AFIX 137

H44D 2 0.321343 0.392120 0.063432 -21.00000 -1.50000

H44E 2 0.346487 0.365065 -0.032088 -21.00000 -1.50000

H44F 2 0.422465 0.391530 0.074664 -21.00000 -1.50000

AFIX 0

C45A 1 0.378214 0.311471 0.451780 -21.00000 0.11977 0.10685 =

0.12834 -0.02326 0.08168 -0.02274

AFIX 23

H45C 2 0.325926 0.293268 0.395350 -21.00000 -1.20000

H45D 2 0.389138 0.293558 0.523845 -21.00000 -1.20000

AFIX 0

C46A 1 0.357589 0.368505 0.458820 -21.00000 0.13083 0.10110 =

0.18198 -0.01576 0.09015 0.01666

AFIX 137

H46D 2 0.412782 0.389699 0.491933 -21.00000 -1.50000

H46E 2 0.322821 0.372146 0.504906 -21.00000 -1.50000

H46F 2 0.323040 0.382729 0.384443 -21.00000 -1.50000

AFIX 0

O5A 4 0.403900 0.323903 0.225337 -21.00000 0.07065 0.09089 =

0.08869 -0.02198 0.02120 -0.00053

O6A 4 0.455313 0.302119 0.423408 -21.00000 0.07573 0.12265 =

0.09476 -0.02601 0.04668 -0.00767

P2A 5 0.444217 0.273231 0.308861 -21.00000 0.06038 0.08937 =

0.08137 -0.01495 0.02689 -0.00813

S2A 6 0.374707 0.205327 0.275303 -21.00000 0.10628 0.12462 =

0.15145 -0.00549 0.07315 -0.02068

PART 0

C24 1 0.631501 0.102537 0.211500 11.00000 0.06690 0.09449 =

0.07507 -0.01619 0.02630 -0.00015

C25 1 0.605343 0.063897 0.123535 11.00000 0.11478 0.09265 =

0.08347 -0.01613 0.03692 -0.00160

AFIX 43

H25 2 0.593447 0.076421 0.050493 11.00000 -1.20000

AFIX 0

C26 1 0.596589 0.007187 0.142146 11.00000 0.13035 0.08888 =

0.10850 -0.02387 0.03576 -0.00384

AFIX 43

H26 2 0.579249 -0.017989 0.081865 11.00000 -1.20000

AFIX 0

C27 1 0.613112 -0.011956 0.247669 11.00000 0.12568 0.07768 =

0.12836 -0.00406 0.04899 0.00250

AFIX 43

H27 2 0.606153 -0.050053 0.259918 11.00000 -1.20000

AFIX 0

C28 1 0.640357 0.025530 0.336855 11.00000 0.11808 0.10753 =

0.09962 0.00809 0.04423 0.01514

AFIX 43

H28 2 0.652938 0.012557 0.409771 11.00000 -1.20000

AFIX 0

C29 1 0.648983 0.081988 0.318363 11.00000 0.08932 0.09126 =

0.07557 -0.00618 0.02824 0.01001

AFIX 43

H29 2 0.666985 0.106832 0.379209 11.00000 -1.20000

AFIX 0

C30 1 0.641682 0.161270 0.185503 11.00000 0.07095 0.09659 =

0.06299 -0.01437 0.02768 -0.00451

AFIX 43

H30 2 0.658239 0.166828 0.124338 11.00000 -1.20000

AFIX 0

HKLF 4

REM bmk2342b in P21/c

REM wR2 = 0.2925, GooF = S = 1.078, Restrained GooF = 1.053 for all data

REM R1 = 0.0838 for 5914 Fo > 4sig(Fo) and 0.1403 for all 11415 data

REM 677 parameters refined using 652 restraints

END

WGHT 0.1270 1.8660

REM Highest difference peak 0.335, deepest hole -0.331, 1-sigma level 0.052

Q1 1 1.1247 0.3043 0.4138 11.00000 0.05 0.33

Q2 1 0.3685 0.2987 0.2477 11.00000 0.05 0.31

Q3 1 0.4434 0.2159 0.2806 11.00000 0.05 0.26

Q4 1 0.8825 0.2692 -0.0394 11.00000 0.05 0.25

Q5 1 0.4426 0.2744 0.4988 11.00000 0.05 0.21

Q6 1 0.8790 0.0653 -0.0833 11.00000 0.05 0.20

Q7 1 1.0589 0.2977 0.7202 11.00000 0.05 0.18

Q8 1 1.2198 0.3199 0.5386 11.00000 0.05 0.18

Q9 1 0.6453 0.0860 0.0525 11.00000 0.05 0.18

Q10 1 1.0576 0.2214 0.3176 11.00000 0.05 0.18

;

_shelx_res_checksum 38843

_shelx_hkl_file

;

0 0 2 432719. 1522.62 10

0 0 3 15.4166 14.0984 4

0 0 -3 21.9320 15.8390 4

0 0 3 7.28615 13.1009 1

0 0 -3 24.1003 19.9188 1

0 0 -3 23.7374 19.7917 10

0 0 3 12.3978 17.0182 10

0 0 -3 10.0863 7.33310 5

0 0 4 5739.40 240.939 4

0 0 -4 5516.37 242.724 1

0 0 4 5392.29 227.314 1

0 0 -4 5721.07 247.758 10

0 0 4 6047.77 256.919 10

0 0 -4 6022.70 165.397 5

0 0 5 763.234 110.188 4

0 0 -5 817.324 120.579 1

0 0 5 560.709 96.6111 1

0 0 5 673.928 112.942 10

0 0 -5 943.293 127.735 10

0 0 -5 619.497 64.6780 5

0 0 6 2598.89 2014.32 1

0 0 6 2846.53 2288.02 10

0 0 -6 3107.27 1454.29 5

0 0 7 669.538 116.796 1

0 0 7 925.288 143.378 10

0 0 -7 1085.10 95.6699 5

0 0 -8 1246.44 96.5641 5

0 0 8 867.066 126.922 1

0 0 -8 1411.74 183.924 10

0 0 8 1142.99 158.257 10

0 0 -9 24.0249 15.2824 5

0 0 -9-5.68513 30.9920 2

0 0 -9 9.58256 39.4106 10

0 0 9 8.14931 37.7508 10

0 0 -9-29.5775 33.8524 6

0 0 -10 3673.74 145.661 5

0 0 -10 3450.16 236.620 2

0 0 10 4025.87 344.003 10

0 0 -10 4352.95 356.730 10

0 0 -10 4319.01 414.381 6

0 0 -11-1.96349 20.1846 2

0 0 11 29.0176 39.0212 1

0 0 -11-4.05245 34.7984 10

0 0 11-37.2385 42.9136 10

0 0 12 301.669 104.651 1

0 0 -12 441.967 122.953 10

0 0 -12 564.349 155.489 6

0 0 -13 34.5187 44.1821 10

0 0 -13-35.0619 85.2526 6

0 0 -14 156.418 97.3278 10

0 0 -14 133.194 93.4431 6

0 0 -15 46.1103 61.3441 10

0 -1 -2 2084.98 114.040 4

0 1 -2 1570.55 75.5826 5

0 -1 2 1432.12 72.3476 1

0 1 2 1451.64 97.1451 1

0 1 -2 1771.14 99.2780 10

0 1 2 1679.05 98.2228 10

0 -1 -2 1809.81 96.1830 10

0 -1 2 1531.81 95.8351 10

0 -1 2 1657.07 79.5342 5

0 -1 -2 1565.26 74.0848 8

0 -1 -3 2739.61 154.700 4

0 -1 3 2620.49 136.418 4

0 1 3 2342.96 144.091 1

0 -1 3 2214.44 115.650 1

0 1 -3 2518.65 125.948 1

0 -1 -3 2546.97 153.671 1

0 1 -3 2606.00 149.074 10

0 -1 3 2660.04 146.536 10

0 -1 -3 2728.71 147.375 10

0 1 3 2743.48 148.752 10

0 -1 -3 2534.93 106.784 8

0 1 3 2753.95 175.260 6

0 1 -3 2573.90 107.615 5

0 -1 4 19623.3 427.328 4

0 -1 -4 19117.0 464.291 4

0 -1 4 18396.3 388.928 1

0 -1 -4 19232.8 465.919 1

0 1 4 18660.8 451.108 1

0 -1 -4 20426.1 466.812 10

0 -1 4 19516.1 452.684 10

0 1 4 19244.3 460.250 10

0 1 -4 19017.2 457.635 10

0 1 -4 19239.2 321.216 5

0 -1 5 185.076 54.6460 4

0 -1 -5 240.174 83.4995 4

0 1 -5 243.937 41.0517 5

0 -1 5 193.737 48.1181 1

0 -1 -5 258.290 71.4475 1

0 1 5 190.100 62.5284 1

0 1 -5 318.726 76.1295 10

0 1 5 266.798 68.1046 10

0 -1 -5 324.257 71.0051 10

0 -1 5 225.284 68.7582 10

0 -1 6 725.519 106.022 4

0 1 -6 605.781 66.7446 5

0 -1 6 661.128 89.3907 1

0 1 6 715.933 109.696 1

0 -1 -6 919.493 133.429 1

0 -1 6 782.611 118.361 10

0 1 -6 863.151 124.692 10

0 1 6 731.146 116.289 10

0 -1 -6 865.648 126.780 10

0 -1 -6 834.053 73.4896 5

0 1 -7 957.302 87.0795 5

0 -1 7 1085.74 135.962 4

0 1 7 875.159 125.365 1

0 -1 -7 1015.52 134.248 1

0 -1 7 981.515 118.926 1

0 1 7 1043.71 144.074 10

0 -1 -7 1159.14 147.087 10

0 -1 7 1157.06 150.200 10

0 1 -7 1018.67 140.765 10

0 -1 -7 944.231 79.0532 5

0 1 -8 6723.80 233.305 5

0 -1 8 6885.35 344.230 1

0 -1 -8 6300.46 272.681 2

0 1 8 6960.33 368.480 1

0 -1 -8 7510.70 410.604 10

0 1 -8 7440.33 411.975 10

0 1 8 7430.90 412.229 10

0 -1 8 7526.36 410.184 10

0 -1 -8 7253.87 224.112 5

0 1 -9 28.1070 16.3567 5

0 -1 -9 58.8493 22.2635 5

0 -1 -9 57.4678 33.2117 2

0 -1 9-14.1188 37.0985 1

0 -1 9 50.9917 61.1248 10

0 -1 -9 39.7364 55.3486 10

0 1 9-8.01704 42.5025 10

0 1 -9-10.9583 46.8652 10

0 -1 -9 64.4369 59.6097 6

0 1 -9 95.4997 85.4827 6

0 -1 -10 28.9931 16.8452 5

0 -1 -10 12.5525 18.5484 2

0 1 10 107.984 74.4541 1

0 1 -10-26.4180 32.0481 2

0 1 -10-0.92185 35.1485 10

0 -1 10 11.2151 35.2689 10

0 1 10-0.25810 35.9612 10

0 -1 -10 2.93640 34.8753 10

0 -1 -10 20.0573 64.9972 6

0 1 -10-9.81446 49.9310 6

0 -1 -11 253.573 61.4895 2

0 1 -11 228.388 64.4536 2

0 1 11 185.818 87.4446 1

0 1 -11 274.703 101.359 10

0 1 11 187.039 75.8895 10

0 -1 -11 391.345 121.721 10

0 -1 -11 283.160 108.536 6

0 1 -11 122.088 79.0578 6

0 -1 12 1029.73 170.179 1

0 1 12 943.448 175.817 1

0 -1 -12 1180.07 206.647 10

0 1 -12 1127.04 202.535 10

0 -1 -12 1290.80 249.519 6

0 1 -12 1123.26 240.246 6

0 1 -13 132.839 80.7633 10

0 -1 -13 7.27958 52.9569 10

0 -1 -13 98.1653 86.8113 6

0 1 -13-16.4944 61.8471 6

0 1 -14 124.629 74.0649 10

0 -1 -14-19.2331 66.0379 10

0 -1 -14-68.5073 94.6450 6

0 1 -14 38.1558 78.1954 6

0 1 -15 35.3205 77.2648 10

0 -1 -15 12.1711 49.5007 10

0 -2 2 32923.9 412.481 5

0 2 -2 31692.6 407.070 5

0 2 2 34688.0 510.717 1

0 2 -2 34690.4 457.499 10

0 -2 2 35413.1 449.836 10

0 -2 -2 35080.9 435.693 10

0 2 2 34336.6 443.450 10

0 2 2 32996.6 524.404 6

0 -2 -2 35952.0 440.502 8

0 -2 -3 6219.10 283.399 1

0 2 3 4022.68 194.567 10

0 -2 -3 4324.97 193.103 10

0 2 -3 3838.66 221.273 10

0 -2 -3 2589.78 138.125 8

0 2 3 3831.21 218.266 6

0 2 -4 5562.30 191.295 5

0 -2 4 6221.37 232.765 4

0 -2 -4 5952.25 270.621 4

0 -2 -4 6217.43 281.080 1

0 2 4 6042.79 276.470 1

0 -2 4 5840.73 200.062 1

0 2 -4 6350.05 273.037 10

0 -2 4 6425.38 264.744 10

0 -2 -4 6546.82 262.765 10

0 2 4 6208.31 261.360 10

0 -2 -4 5948.36 205.443 8

0 2 4 6485.66 315.923 6

0 -2 4 6231.10 204.514 5

0 -2 5 101.226 40.2423 4

0 2 -5 172.990 39.2552 5

0 -2 -5 214.317 68.3468 4

0 2 -5 171.092 52.6840 10

0 2 5 185.874 54.0891 10

0 -2 -5 181.706 56.3549 10

0 -2 5 123.570 42.6347 10

0 2 5 163.989 51.8439 1

0 -2 5 112.555 30.3082 1

0 -2 -5 154.349 60.6662 1

0 -2 -6 22952.2 645.897 4

0 2 -6 20074.9 408.741 5

0 -2 6 21433.5 535.840 4

0 2 -6 21371.6 604.877 10

0 2 6 22553.1 622.668 10

0 -2 -6 23362.7 609.229 10

0 -2 6 22160.2 596.133 10

0 -2 6 20401.8 483.426 1

0 2 6 20531.1 586.722 1

0 -2 -6 25799.5 694.421 1

0 -2 -6 18952.4 359.696 2

0 2 -7 2731.19 155.707 5

0 -2 -7 2790.04 240.831 4

0 -2 7 2715.29 208.814 4

0 -2 7 2330.09 184.352 1

0 2 7 2700.22 224.319 1

0 -2 -7 2336.16 137.497 2

0 -2 -7 2602.19 237.981 1

0 -2 7 2850.64 232.677 10

0 2 -7 2500.45 227.269 10

0 -2 -7 2869.04 231.363 10

0 2 7 2736.06 232.538 10

0 2 -8 1202.33 105.031 5

0 -2 -8 1159.63 164.729 1

0 -2 8 1095.26 129.985 1

0 -2 -8 1127.38 101.711 2

0 2 -8 1180.18 166.847 10

0 -2 -8 1404.37 176.571 10

0 -2 8 1311.05 170.586 10

0 2 8 1146.91 162.002 10

0 -2 -8 1162.51 90.7148 5

0 2 -9 758.345 84.6161 5

0 -2 9 697.735 119.014 1

0 -2 -9 764.679 89.2545 2

0 2 9 784.283 143.691 10

0 2 -9 893.848 155.732 10

0 -2 -9 831.444 144.212 10

0 -2 9 801.820 143.113 10

0 2 -9 764.867 166.399 6

0 -2 -9 925.798 170.581 6

0 -2 -9 861.930 76.5806 5

0 -2 -10 562.294 53.7772 5

0 -2 10 573.836 106.622 1

0 -2 -10 628.988 83.8158 2

0 2 10 520.025 118.584 1

0 -2 10 688.292 144.896 10

0 2 -10 813.253 157.365 10

0 -2 -10 725.508 145.739 10

0 2 10 588.458 133.019 10

0 -2 -10 528.368 142.307 6

0 2 -10 723.546 178.651 6

0 2 -11 52.7848 46.8109 2

0 2 11 47.8968 52.9084 1

0 -2 -11 44.5911 23.9188 2

0 -2 -11 64.5517 54.1857 10

0 2 11 93.3109 67.6187 10

0 2 -11 82.7440 74.2740 10

0 2 -11 188.486 101.059 6

0 -2 -11 38.4641 54.3609 6

0 2 12 319.219 109.350 1

0 -2 12 365.448 110.829 1

0 2 -12 363.299 112.820 10

0 -2 -12 364.464 109.533 10

0 -2 -12 366.967 157.022 6

0 2 -12 327.102 137.521 6

0 -2 -13 100.682 63.1155 10

0 2 -13 135.364 87.5119 10

0 -2 -13 8.61759 64.3964 6

0 2 -13 156.691 100.656 6

0 -2 -14 80.9324 62.0095 10

0 2 -14 178.120 102.687 10

0 -2 -14 196.326 139.215 6

0 2 -14 30.7854 49.2717 6

0 -2 -15 93.8869 67.4316 10

0 2 -15 46.1623 50.9257 10

0 3 -2 234469. 1264.75 5

0 -3 -2 252852. 1472.76 1

0 3 -2 251918. 1282.92 10

0 -3 -2 241235. 917.964 9

0 3 2 246929. 1265.09 10

0 -3 2 251663. 1262.30 10

0 -3 -2 233005. 1283.27 8

0 3 2 253914. 1488.31 6

0 3 -3 3824.20 171.965 5

0 -3 -3 3876.08 204.573 4

0 -3 3 3829.91 121.492 1

0 3 3 3549.63 202.091 1

0 -3 -3 3970.55 211.545 1

0 -3 -3 4131.41 184.748 10

0 3 3 4380.48 195.431 10

0 -3 3 4450.90 193.292 10

0 3 -3 4329.47 198.559 10

0 -3 -3 3861.69 175.931 8

0 3 3 4453.92 233.887 6

0 -3 3 4090.96 181.823 5

0 3 -4 6984.42 243.065 5

0 -3 -4 7910.58 326.290 4

0 3 4 6873.67 301.119 1

0 -3 -4 8109.67 352.269 1

0 -3 4 6736.27 198.425 1

0 -3 4 7851.13 293.424 10

0 -3 -4 7793.52 286.383 10

0 3 -4 7588.14 300.982 10

0 3 4 7071.85 286.729 10

0 3 4 7680.35 346.764 6

0 -3 4 7093.58 247.555 5

0 -3 -4 7688.65 260.535 8

0 3 -5 8040.38 273.064 5

0 -3 -5 8189.02 364.716 4

0 -3 5 8563.89 304.836 4

0 -3 -5 8151.12 372.389 1

0 -3 5 8072.33 252.655 1

0 3 5 7690.49 348.200 1

0 -3 -5 7765.66 148.464 2

0 3 5 8601.29 352.424 10

0 -3 5 8563.55 343.177 10

0 3 -5 8625.61 362.943 10

0 -3 -5 8632.97 333.929 10

0 3 5 8709.53 418.103 6

0 -3 5 8207.24 272.816 5

0 -3 -5 7892.91 283.168 8

0 3 -6 13389.0 360.781 5

0 -3 6 14201.0 432.576 4

0 -3 -6 14551.2 528.867 4

0 -3 -6 14391.6 473.677 10

0 3 6 14325.0 493.927 10

0 3 -6 14507.1 515.666 10

0 -3 6 14664.0 487.632 10

0 -3 6 12778.1 361.146 1

0 -3 -6 12903.0 231.974 2

0 3 6 13185.5 483.914 1

0 -3 -6 14605.3 533.455 1

0 -3 7 31.5229 34.8295 4

0 3 -7-7.96372 30.3332 5

0 -3 -7 69.5793 54.3507 4

0 3 7 57.6917 39.0763 10

0 3 -7 22.3860 42.8895 10

0 -3 -7-7.57984 33.6513 10

0 -3 7 23.7395 25.4744 10

0 3 7-52.3578 60.0283 1

0 -3 7 14.3467 21.2328 1

0 -3 -7 24.3830 16.4541 2

0 -3 -7-16.9429 47.5971 1

0 3 -8 29.6354 16.7672 5

0 -3 -8 18.1512 42.3809 4

0 -3 8 7.32524 28.8367 4

0 -3 -8 15.4132 15.4041 2

0 -3 -8 80.2903 53.7961 1

0 -3 8 24.4931 27.5669 1

0 3 -8 61.0340 60.6229 10

0 -3 8-21.6497 34.6998 10

0 3 8 130.736 63.9806 10

0 -3 -8-4.22773 33.4911 10

0 3 -9 5203.71 239.968 5

0 -3 9 4777.00 278.842 1

0 3 9 5102.76 361.016 1

0 -3 -9 4953.81 188.392 2

0 -3 9 5796.20 380.269 10

0 3 9 5228.02 375.724 10

0 -3 -9 5219.39 354.620 10

0 3 -9 5348.75 382.860 10

0 -3 -9 5107.57 191.416 5

0 -3 -9 5671.28 441.898 6

0 3 -9 5482.81 451.266 6

0 3 10 646.234 144.835 1

0 -3 10 613.435 105.986 1

0 3 -10 691.404 151.348 10

0 -3 10 688.026 137.044 10

0 3 10 658.556 141.693 10

0 -3 -10 665.337 133.880 10

0 -3 -10 796.877 175.824 6

0 3 -10 903.427 197.521 6

0 -3 -10 567.591 57.0703 5

0 -3 11 0.17861 44.1774 1

0 3 11 55.1397 54.7715 1

0 3 -11-44.9106 60.7654 2

0 -3 -11 12.2417 52.5159 10

0 3 11 90.2577 71.7706 10

0 3 -11 28.5226 46.0516 10

0 -3 -11 29.4049 61.6120 6

0 3 -11 10.1330 56.0628 6

0 -3 -12 927.750 177.103 10

0 3 -12 883.656 182.584 10

0 -3 -12 1120.18 236.686 6

0 3 -12 1013.20 226.674 6

0 3 -13 165.609 87.8202 10

0 -3 -13 43.5494 47.8829 10

0 -3 -13 149.237 101.668 6

0 3 -13-66.5499 73.0176 6

0 3 -14 26.4101 47.4957 10

0 -3 -14 134.740 83.0698 10

0 3 -14-130.530 135.437 6

0 -3 -14-53.3609 70.7823 6

0 3 -15 29.8919 72.7358 10

0 -3 -15-38.1169 47.6956 10

0 3 -16 64.3271 59.4210 10

0 4 0 668210. 1841.93 1

0 -4 -1 128482. 833.110 3

0 4 1 134566. 868.776 3

0 4 1 132160. 754.013 9

0 4 -1 126572. 701.445 7

0 -4 2 136733. 1131.80 5

0 -4 -2 126647. 898.864 3

0 4 -2 117869. 1032.97 5

0 -4 -2 127812. 712.151 9

0 4 2 124210. 733.540 9

0 4 2 130651. 1108.92 6

0 -4 -2 122066. 1037.48 8

0 -4 2 129696. 699.499 7

0 -4 3 7411.69 2780.01 5

0 -4 -3 5548.88 2673.50 1

0 -4 -3 6016.33 1797.02 9

0 -4 3 9691.57 3154.02 10

0 4 -3 9600.47 3113.00 10

0 4 3 5207.51 2339.34 10

0 4 3 6315.88 3091.41 6

0 4 -4 9855.27 309.467 5

0 -4 -4 11357.6 404.051 4

0 -4 -4 10765.8 425.975 1

0 -4 4 9666.44 216.047 1

0 4 4 11266.1 406.983 1

0 4 -4 10517.9 366.144 10

0 4 4 11029.4 361.737 10

0 -4 4 11229.2 356.726 10

0 -4 -4 10525.4 337.617 10

0 -4 4 10526.0 333.118 5

0 -4 -4 10765.4 339.643 8

0 4 4 10524.2 420.525 6

0 4 -5 3735.49 201.890 5

0 -4 -5 4354.92 278.159 4

0 -4 5 3721.15 159.989 1

0 4 5 3881.26 264.734 1

0 -4 -5 3918.08 265.361 1

0 -4 -5 4273.73 238.351 10

0 -4 5 4074.22 239.698 10

0 4 -5 4524.85 264.883 10

0 4 5 4268.24 255.568 10

0 4 5 4548.85 304.898 6

0 -4 -5 3830.84 216.836 8

0 -4 5 4333.02 219.295 5

0 -4 6 27.8380 22.2018 4

0 -4 -6 73.0924 48.2881 4

0 4 -6 14.5167 16.7252 5

0 -4 6 28.6865 16.3583 1

0 4 6 64.8769 38.1357 1

0 -4 -6 3.70611 40.7281 1

0 -4 6-6.93225 24.3851 10

0 -4 -6 24.5451 32.3856 10

0 4 6 35.1719 29.1316 10

0 4 -6 4.44470 32.9731 10

0 -4 6 80.7282 35.9377 5

0 4 6 50.8344 52.1481 6

0 -4 -7 13022.8 555.720 4

0 -4 7 13452.3 451.697 4

0 4 -7 12509.0 398.276 5

0 -4 7 12212.3 356.520 1

0 -4 -7 12560.4 548.188 1

0 4 -7 13472.6 543.629 10

0 -4 -7 13637.3 499.749 10

0 4 7 13417.8 525.533 10

0 -4 7 13940.1 521.659 10

0 -4 8 3520.85 248.919 4

0 -4 -8 3576.18 312.533 4

0 4 -8 3177.71 202.672 5

0 -4 -8 3219.80 293.231 1

0 4 8 2709.97 268.049 1

0 -4 8 3129.19 194.269 1

0 4 8 3549.70 293.198 10

0 -4 8 3796.93 287.993 10

0 4 -8 3730.16 302.886 10

0 -4 -8 3592.09 274.853 10

0 -4 -8 3704.89 331.645 6

0 -4 9 52.2046 43.1565 4

0 -4 -9 20.5232 34.6122 4

0 4 -9 57.2962 34.8127 5

0 4 9-18.3401 42.2071 1

0 -4 9-9.47522 32.2848 1

0 -4 -9 50.5628 71.8235 1

0 4 9 5.49647 30.9598 10

0 -4 9 5.58401 36.1749 10

0 -4 -9-13.9805 40.7444 10

0 4 -9 24.8640 44.6141 10

0 -4 -9-43.3131 43.2487 6

0 4 10 2559.12 273.250 1

0 -4 10 2097.89 191.512 1

0 4 -10 2486.66 282.533 10

0 4 10 2853.33 297.977 10

0 -4 10 2801.46 279.428 10

0 -4 -10 2828.63 277.738 10

0 -4 -10 3066.65 337.919 6

0 -4 -10 2506.02 127.994 5

0 4 -10 2418.43 320.090 6

0 -4 11 135.864 45.8470 1

0 4 11 89.9175 77.1805 1

0 -4 -11 227.023 84.8567 10

0 4 11 134.189 72.1721 10

0 4 -11 142.257 77.0755 10

0 -4 -11 247.887 109.135 6

0 4 -11 188.114 114.185 6

0 4 -12 89.1313 54.6626 2

0 -4 -12 108.183 57.1287 10

0 4 -12 192.852 107.556 10

0 4 -12 181.347 119.472 6

0 -4 -12 276.459 127.247 6

0 4 -13 8.33135 56.1330 10

0 -4 -13 0.16974 37.7008 10

0 -4 -13 89.8959 84.3426 6

0 4 -13 43.5379 80.3357 6

0 4 -14 65.5714 60.8135 10

0 -4 -14 280.956 118.425 10

0 -4 -14 360.945 160.809 6

0 4 -14 85.7779 80.9175 6

0 4 -15 259.653 128.177 10

0 4 -16 29.2966 71.6779 10

0 5 0 2480.01 147.320 1

0 -5 0 2177.07 147.337 1

0 -5 -1 4065.99 195.972 1

0 5 -1 3904.82 132.780 1

0 -5 1 3819.17 133.799 1

0 5 1 4296.68 173.491 3

0 -5 -1 4375.77 174.561 3

0 -5 1 4132.47 209.268 5

0 5 -1 4180.99 143.326 7

0 5 2 65648.0 721.341 3

0 -5 2 73255.6 900.551 5

0 5 -2 75999.8 923.793 5

0 -5 -2 65118.3 695.481 3

0 -5 -2 68862.1 882.870 1

0 -5 2 68742.0 449.073 1

0 -5 -2 67689.0 575.083 9

0 5 2 65691.1 589.244 9

0 -5 2 67089.7 555.233 7

0 -5 -2 73528.3 884.910 8

0 5 2 64864.1 847.222 6

0 5 -3 260.130 50.6722 5

0 -5 -3 325.410 52.5742 3

0 -5 3 297.043 61.6688 5

0 -5 -3 224.645 59.7292 1

0 -5 -3 329.127 38.7244 9

0 5 -3 389.452 63.9970 10

0 5 3 320.985 57.8935 10

0 -5 3 303.900 39.4804 7

0 -5 -3 378.003 69.5621 8

0 5 3 246.734 58.3269 6

0 -5 4 10683.3 373.701 5

0 5 -4 10912.4 360.757 5

0 -5 -4 12408.5 453.782 4

0 -5 4 10633.2 202.218 1

0 5 4 10866.7 410.551 1

0 -5 -4 11219.2 269.534 9

0 5 -4 11797.0 402.752 10

0 5 4 11196.8 369.587 10

0 -5 4 10821.3 363.887 10

0 5 4 11242.7 450.937 6

0 -5 -4 9610.70 356.047 8

0 5 -5 6850.16 299.130 5

0 -5 -5 7108.96 364.580 4

0 5 5 6712.98 353.865 1

0 -5 -5 6383.81 359.530 1

0 -5 5 6466.51 188.440 1

0 5 5 7261.85 333.036 10

0 -5 5 7656.34 331.577 10

0 5 -5 7432.81 344.362 10

0 5 5 7449.42 402.367 6

0 -5 5 7357.13 310.823 5

0 -5 5 6837.04 180.063 8

0 -5 -5 6995.42 306.868 8

0 5 -6 866.088 106.870 5

0 -5 -6 1251.17 173.873 4

0 -5 -6 1116.52 165.586 1

0 -5 6 838.531 78.8324 1

0 -5 -6 1041.36 131.342 10

0 5 -6 1029.23 144.056 10

0 -5 6 886.171 121.380 10

0 5 6 987.080 137.700 10

0 -5 -6 918.684 123.322 8

0 5 6 986.601 158.842 6

0 -5 6 827.527 117.513 5

0 -5 -7 876.408 150.081 4

0 5 -7 687.676 98.9879 5

0 -5 7 837.955 115.789 4

0 -5 7 722.801 79.7602 1

0 -5 -7 912.631 159.292 1

0 5 -7 910.010 149.344 10

0 -5 -7 837.381 127.383 10

0 5 7 724.195 124.072 10

0 -5 7 851.165 131.304 10

0 5 7 785.757 155.305 6

0 5 -8 185.130 59.9479 5

0 -5 8 108.731 43.0956 4

0 -5 -8 160.550 71.2627 4

0 5 8 112.894 48.8605 1

0 -5 -8 183.047 67.8468 1

0 -5 8 129.707 30.5628 1

0 5 8 175.194 67.5283 10

0 5 -8 168.887 65.8311 10

0 -5 -8 205.898 69.8717 10

0 -5 8 202.942 71.5034 10

0 -5 -8 215.174 81.6656 6

0 -5 9 2803.17 231.614 4

0 -5 -9 2734.29 292.661 4

0 5 9 2819.94 283.272 1

0 -5 -9 2565.26 282.191 1

0 -5 9 2593.99 179.714 1

0 5 -9 2745.17 283.808 10

0 -5 -9 3012.97 271.543 10

0 5 9 3029.23 291.194 10

0 -5 9 3017.40 277.615 10

0 -5 -9 2595.98 295.706 6

0 -5 -10 487.620 125.372 1

0 5 10 305.320 96.5364 1

0 -5 10 436.472 80.8916 1

0 -5 -10 502.905 125.514 10

0 5 -10 402.240 120.452 10

0 5 10 436.634 118.424 10

0 -5 10 486.291 120.383 10

0 -5 -10 390.149 54.9573 5

0 -5 -10 409.051 137.223 6

0 5 -10 427.591 131.696 6

0 -5 -11 929.821 63.1267 5

0 -5 11 1132.64 132.788 1

0 5 -11 1221.32 208.521 10

0 -5 -11 1340.85 198.475 10

0 5 11 1311.87 218.472 10

0 -5 -11 958.260 65.6346 5

0 5 -11 1277.27 242.565 6

0 -5 -11 1145.60 221.990 6

0 5 -12 804.134 165.335 2

0 5 -12 1026.18 206.399 10

0 -5 -12 1049.83 186.226 10

0 -5 -12 998.425 215.051 6

0 5 -12 863.771 207.113 6

0 5 -13 27.8811 67.0397 10

0 -5 -13 66.6515 57.8278 6

0 5 -13-89.6811 83.8925 6

0 5 -14 33.6427 48.8665 10

0 -5 -14 102.442 76.6990 6

0 5 -14 27.7111 94.4507 6

0 5 -15-48.9594 73.9890 10

0 5 -16 2.30506 53.5228 10

0 6 0 97928.9 865.285 1

0 6 0 92032.5 896.861 3

0 -6 -1 442.287 69.3453 1

0 6 1 280.918 51.9501 1

0 6 -1 265.308 39.8107 1

0 6 -1 310.188 53.5309 3

0 -6 1 250.351 40.6964 1

0 -6 -1 317.314 52.5667 3

0 6 1 305.982 51.2697 3

0 -6 1 225.371 52.7337 5

0 6 2 860.830 88.0973 3

0 -6 2 652.736 93.0730 5

0 -6 -2 773.622 80.8294 3

0 -6 -2 802.826 106.196 1

0 -6 2 684.370 53.1777 1

0 6 2 702.903 65.5611 9

0 6 -2 745.706 65.1584 7

0 -6 -2 715.972 94.5963 8

0 -6 -3 1852.92 140.923 3

0 6 -3 2348.29 193.734 5

0 -6 -3 2011.17 118.036 9

0 6 -3 3059.25 192.863 10

0 6 3 1102.78 178.361 6

0 -6 -3 2030.14 171.499 8

0 -6 3 1831.57 118.964 7

0 6 -4 1683.08 151.641 5

0 -6 -4 1612.26 132.386 3

0 -6 4 1469.17 149.106 5

0 -6 -4 1500.89 174.875 1

0 6 -4 1616.52 151.371 10

0 6 4 1457.79 139.207 10

0 -6 -4 1583.84 103.321 9

0 -6 4 1607.74 101.247 7

0 -6 -4 1178.92 146.541 8

0 6 4 1576.18 167.955 6

0 6 -5 7206.42 329.388 5

0 -6 -5 7579.71 385.089 4

0 -6 -5 6748.06 383.821 1

0 -6 5 7080.69 175.780 1

0 6 5 7337.64 375.770 1

0 6 5 7678.65 351.916 10

0 -6 -5 7426.09 243.150 9

0 6 -5 7708.12 359.234 10

0 -6 5 7626.14 337.467 10

0 6 5 7985.48 425.396 6

0 -6 5 7819.18 347.108 5

0 -6 -5 6759.51 324.130 8

0 -6 5 7371.17 225.967 8

0 -6 -6 637.933 129.349 4

0 6 -6 680.830 107.270 5

0 -6 6 669.858 63.6800 1

0 -6 -6 868.423 160.369 1

0 -6 6 694.283 118.185 10

0 6 6 736.469 119.187 10

0 6 -6 920.692 146.016 10

0 -6 6 783.663 63.0084 8

0 -6 -6 779.496 117.230 8

0 -6 6 756.992 129.543 5

0 6 6 783.798 149.224 6

0 -6 7 4445.09 260.983 4

0 6 -7 4051.80 254.222 5

0 -6 -7 4388.88 343.470 4

0 -6 7 3694.19 162.834 1

0 6 7 3519.63 304.902 1

0 -6 -7 3989.23 322.624 1

0 6 7 4286.41 305.895 10

0 -6 7 4633.36 302.042 10

0 6 -7 4627.31 326.710 10

0 -6 -7 4145.01 268.402 8

0 -6 7 4335.41 271.618 5

0 6 7 4635.43 378.533 6

0 -6 8 38.6562 26.0993 4

0 -6 -8 97.6768 74.1577 4

0 -6 8 28.2253 23.1577 1

0 6 8 29.3216 55.8179 1

0 -6 -8 118.731 107.371 1

0 6 -8-15.6675 52.7928 10

0 -6 8-3.94026 42.2598 10

0 6 8 15.2433 26.6491 10

0 6 8 61.9477 36.9632 6

0 -6 -8 32.9357 58.8509 6

0 -6 9 120.360 52.8867 4

0 -6 -9 62.9781 63.6283 4

0 -6 9 95.1154 43.9250 1

0 6 9 66.9258 66.4191 1

0 -6 -9 245.733 160.988 1

0 -6 9 107.312 44.3757 10

0 6 -9 61.6787 46.3041 10

0 6 9 67.2209 38.6638 10

0 -6 -9 138.076 65.3251 6

0 -6 -10 565.511 147.509 4

0 -6 10 709.193 129.752 4

0 -6 -10 635.294 162.794 1

0 6 10 576.359 139.992 1

0 -6 10 664.032 91.4366 1

0 6 -10 636.007 144.689 10

0 6 10 680.482 150.049 10

0 -6 -10 700.941 166.500 6

0 -6 -11 47.9792 23.9391 5

0 -6 11 10.9057 28.7047 1

0 6 -11 59.9275 60.6428 10

0 6 -11 122.237 96.7482 6

0 -6 -11 64.1631 22.0690 5

0 -6 -11 4.26426 60.4793 6

0 6 -12 140.007 88.5346 10

0 -6 -12 64.4921 69.7797 6

0 6 -12 174.891 124.503 6

0 6 -13 169.986 90.8757 10

0 -6 -13 158.628 90.1674 6

0 6 -13 104.049 76.7282 6

0 6 -14 28.9415 50.6345 10

0 6 -14 117.703 77.4036 6

0 -6 -14 157.732 126.840 6

0 6 -15 91.9592 61.3907 10

0 6 -16 2.85852 50.0977 10

0 7 0 1845.34 149.627 1

0 -7 0 3579.62 201.312 1

0 -7 0 2626.75 208.287 5

0 7 1 1548.31 136.226 1

0 -7 1 1665.46 112.037 1

0 -7 -1 1795.98 144.721 1

0 7 -1 1521.15 102.650 1

0 7 1 1443.36 118.481 3

0 7 -1 1487.67 125.555 3

0 -7 -1 1479.21 122.196 3

0 -7 1 1763.38 163.046 5

0 -7 -2 7623.02 268.881 3

0 -7 2 7884.15 345.404 5

0 7 2 7751.40 280.376 3

0 -7 -2 7995.09 338.832 1

0 -7 2 7683.98 208.012 1

0 7 -2 7756.40 204.630 1

0 7 2 7723.21 238.456 9

0 -7 -2 8209.99 344.075 8

0 7 -2 7614.95 228.375 7

0 -7 3 181.373 58.7835 5

0 -7 -3 156.965 42.1554 3

0 -7 3 177.451 27.1607 1

0 -7 -3 151.560 60.6189 1

0 7 -3 180.953 52.1056 10

0 7 3 144.963 33.9410 9

0 -7 -3 155.609 34.9088 9

0 -7 3 154.329 32.4159 7

0 -7 -3 95.7594 45.6427 8

0 7 3 138.749 48.8003 6

0 -7 4 1281.30 158.167 5

0 -7 -4 1032.99 110.241 3

0 7 -4 971.904 126.015 5

0 -7 -4 1614.03 183.618 1

0 -7 4 1010.51 52.4439 1

0 7 -4 1303.49 148.714 10

0 7 4 963.468 122.523 10

0 -7 -4 1052.97 90.3296 9

0 -7 -4 1325.95 164.878 8

0 -7 4 1056.69 85.3821 7

0 7 4 1055.57 148.034 6

0 7 -5 12179.5 448.585 5

0 -7 -5 13200.8 421.252 3

0 -7 -5 12385.6 508.117 4

0 -7 5 12753.4 477.372 5

0 -7 -5 11312.5 509.658 1

0 7 5 12144.3 447.764 10

0 -7 -5 12441.8 318.775 9

0 7 -5 12636.0 476.055 10

0 -7 -5 11312.6 437.113 8

0 7 5 13231.1 562.488 6

0 -7 5 12110.2 305.654 7

0 -7 5 11737.2 325.385 8

0 -7 -6 870.088 150.712 4

0 7 -6 879.674 144.165 5

0 -7 -6 1909.92 231.869 1

0 -7 6 779.835 56.8213 1

0 7 6 815.596 127.273 10

0 -7 -6 879.253 92.9695 9

0 7 -6 877.211 143.700 10

0 -7 -6 1825.65 189.233 8

0 -7 6 807.645 143.430 5

0 7 6 889.048 155.151 6

0 -7 6 840.580 79.3963 8

0 -7 -7 375.904 119.221 4

0 7 7 166.507 71.8682 1

0 -7 7 165.775 30.6999 1

0 7 7 178.361 67.8377 10

0 -7 7 173.675 68.3859 10

0 -7 -7 269.657 58.2975 9

0 7 -7 190.384 69.6336 10

0 7 7 118.811 71.4770 6

0 -7 -7 435.468 108.446 8

0 -7 7 192.890 75.3781 5

0 -7 8 767.589 115.728 4

0 -7 -8 748.191 152.983 4

0 7 8 1008.02 172.180 1

0 -7 -8 934.543 168.381 1

0 -7 8 780.673 72.3430 1

0 7 -8 841.255 151.342 10

0 -7 8 1046.45 157.196 10

0 7 8 761.923 140.897 10

0 -7 8 788.195 123.328 5

0 7 8 894.686 181.398 6

0 -7 -8 728.108 150.198 6

0 -7 -9 17.9301 43.1024 4

0 -7 9-39.9465 32.2042 4

0 -7 -9-55.7791 78.9779 1

0 -7 9-8.91414 21.9923 1

0 7 9 34.4651 60.4643 1

0 7 9 105.335 68.7537 10

0 -7 9 92.6085 63.9129 10

0 7 -9 19.6838 40.3583 10

0 -7 -9 29.8978 38.5047 6

0 -7 10 837.692 135.845 4

0 -7 -10 956.615 209.214 1

0 -7 10 407.282 72.6588 1

0 7 10 773.670 157.018 10

0 7 -10 892.856 174.874 10

0 -7 -10 784.411 169.166 6

0 -7 11 702.736 89.7128 1

0 7 -11 811.943 172.741 10

0 -7 -11 772.417 186.829 6

0 7 -12 503.404 151.707 10

0 -7 -12 461.510 144.803 6

0 7 -12 694.706 190.838 6

0 7 -13 257.803 107.231 10

0 7 -13 279.141 128.445 6

0 -7 -13 194.514 103.408 6

0 7 -14 53.8476 87.7525 10

0 7 -14-29.4905 71.3026 6

0 -7 -14-115.953 101.832 6

0 7 -15-28.3359 65.3840 10

0 8 0 47222.6 687.280 1

0 -8 0 49811.0 739.787 1

0 8 0 45532.7 729.359 3

0 -8 0 51157.6 911.382 5

0 8 1 566.286 85.0204 1

0 8 -1 524.944 63.8767 1

0 -8 -1 500.273 79.7005 1

0 -8 1 542.766 71.2818 1

0 -8 1 509.364 92.3699 5

0 8 1 649.695 86.0992 3

0 8 -1 629.746 87.3089 3

0 -8 -1 594.876 82.5840 3

0 -8 2 14559.1 506.999 5

0 8 2 13136.7 395.642 3

0 -8 -2 13565.0 387.048 3

0 8 -2 13825.0 299.790 1

0 -8 2 15047.6 319.100 1

0 -8 -2 13797.5 480.198 1

0 8 2 13977.9 449.173 1

0 8 2 13624.6 341.996 9

0 8 -2 13682.2 329.313 7

0 -8 -2 14818.3 494.765 8

0 -8 -3 1707.29 143.679 3

0 -8 3 3531.44 290.221 5

0 -8 -3 4594.29 336.565 1

0 -8 3 2755.59 126.769 1

0 -8 -3 3694.54 319.223 8

0 8 3 2002.58 194.697 6

0 -8 4 1251.98 153.324 5

0 -8 -4 1271.06 126.736 3

0 -8 4 1042.21 60.1728 1

0 -8 -4 1148.13 152.050 1

0 8 4 1174.94 134.514 10

0 -8 -4 1239.73 97.3544 9

0 -8 4 1119.26 114.471 8

0 -8 4 1106.16 90.8260 7

0 -8 -4 1042.93 139.912 8

0 8 4 1065.66 149.280 6

0 -8 -5 3842.19 231.745 3

0 -8 5 3563.66 266.145 5

0 -8 -5 3056.53 265.533 1

0 -8 -5 3421.62 168.258 9

0 8 -5 3559.04 259.583 10

0 8 5 3534.06 252.195 10

0 -8 5 3469.20 167.596 7

0 -8 5 3411.44 191.643 8

0 8 5 3869.18 306.808 6

0 -8 -5 3109.51 237.836 8

0 -8 -6 3661.78 317.133 4

0 -8 -6 3126.07 225.312 3

0 8 6 3261.72 289.528 1

0 -8 -6 3924.24 364.868 1

0 8 -6 3277.27 270.143 10

0 8 6 2958.53 247.742 10

0 -8 -6 3061.24 171.225 9

0 -8 6 3213.15 259.406 5

0 -8 -6 4435.44 300.340 8

0 8 6 3302.00 308.869 6

0 -8 6 2808.47 163.565 8

0 -8 6 2863.56 159.104 7

0 -8 -7 8901.95 499.136 4

0 8 7 8649.95 487.630 1

0 -8 -7 8477.58 498.998 1

0 8 -7 8673.58 458.981 10

0 8 7 8793.45 456.157 10

0 -8 -7 8340.11 301.642 9

0 -8 -7 7543.30 400.720 8

0 8 7 9057.21 550.929 6

0 -8 7 8943.96 435.348 5

0 -8 -8 1433.15 217.672 4

0 -8 8 1291.04 151.851 4

0 8 8 1184.19 190.578 1

0 -8 8 1109.68 67.3875 1

0 -8 -8 1398.76 254.948 1

0 -8 -8 1306.94 123.140 9

0 8 -8 1344.09 200.786 10

0 8 8 1174.82 178.170 10

0 -8 8 1315.34 175.576 5

0 -8 -8 1293.58 196.448 6

0 -8 -9 72.4900 82.2804 4

0 -8 9-37.5703 41.5822 4

0 -8 -9 103.837 188.928 1

0 -8 9 13.4446 15.2396 1

0 8 -9-93.9036 74.3700 10

0 8 9-35.8596 59.6528 10

0 -8 -9 43.7673 34.8711 9

0 -8 -9-6.69374 42.1731 6

0 -8 10-7.60042 25.6239 4

0 -8 -10-73.8586 83.5818 1

0 -8 10 5.26417 21.7108 1

0 8 -10-11.1565 48.8589 10

0 8 10-12.3669 37.0143 10

0 -8 -10 20.8870 31.4479 9

0 -8 -10-42.1340 53.5740 6

0 -8 -11-16.3148 60.4459 1

0 -8 11-14.5983 18.3155 1

0 8 -11-36.6458 72.4292 10

0 -8 -11-51.8299 36.6596 9

0 -8 -11-15.8627 44.4509 6

0 8 -12 619.065 182.854 10

0 -8 -12 536.464 101.707 9

0 8 -12 571.003 183.236 6

0 -8 -12 526.751 159.509 6

0 8 -13 890.771 208.256 10

0 -8 -13 830.182 207.109 6

0 8 -13 728.106 215.674 6

0 8 -14 496.941 163.382 10

0 8 -14 362.811 163.664 6

0 -8 -14 339.061 127.199 6

0 8 -15 204.772 81.5971 10

0 -9 0 665.104 120.758 1

0 9 0 422.831 92.7868 1

0 -9 0 405.304 106.802 5

0 9 0 292.101 79.1395 3

0 -9 -1 584.958 93.7735 1

0 9 -1 473.837 82.5238 3

0 -9 1 591.431 78.3293 1

0 9 -1 495.371 68.9711 1

0 9 1 430.216 86.0016 1

0 -9 1 557.160 106.171 5

0 -9 -1 411.474 79.7941 3

0 9 1 311.168 65.5752 3

0 -9 2 4907.56 307.191 5

0 9 2 5460.44 265.587 3

0 -9 -2 5059.53 249.814 3

0 -9 2 4656.82 192.004 1

0 9 -2 4608.65 188.422 1

0 9 -2 5205.91 275.258 3

0 -9 -2 4741.16 282.508 1

0 9 2 5204.29 226.408 9

0 -9 -2 4807.74 293.948 8

0 9 -2 5097.59 213.527 7

0 9 3 22657.4 569.035 3

0 -9 -3 21979.7 526.188 3

0 -9 3 22457.9 680.073 5

0 -9 -3 21708.7 657.241 1

0 -9 3 20809.2 363.540 1

0 9 3 21796.1 454.997 9

0 -9 -3 20638.2 638.474 8

0 -9 4 950.747 64.4423 1

0 -9 -4 1269.76 189.275 1

0 -9 4 1196.16 160.367 5

0 -9 -4 1055.56 122.329 3

0 9 4 983.599 147.476 6

0 -9 -4 1141.98 157.721 8

0 -9 4 1304.96 145.420 8

0 -9 5 183.530 111.548 5

0 -9 -5 203.724 71.6933 3

0 9 -5 138.750 72.5503 10

0 9 5 114.298 57.7145 10

0 -9 5 72.5513 53.6239 8

0 -9 -5 72.7175 38.9530 9

0 -9 5 83.4529 35.1965 7

0 9 5 101.358 59.0571 6

0 -9 -6 1219.95 190.934 4

0 -9 -6 1712.35 176.290 3

0 9 6 1134.99 187.344 1

0 -9 -6 1518.63 238.048 1

0 9 6 1177.94 166.118 10

0 -9 -6 1632.29 133.534 9

0 9 -6 1506.29 197.783 10

0 -9 6 1314.79 129.873 8

0 -9 6 1338.78 177.772 5

0 -9 6 1220.61 109.740 7

0 9 6 1366.66 216.322 6

0 -9 -6 1108.22 170.170 8

0 -9 -7 63.3947 77.6360 4

0 -9 -7 77.9651 42.2000 3

0 -9 7 216.366 127.580 5

0 9 7 258.978 136.767 1

0 9 7 9.28119 32.3594 10

0 9 -7 90.2579 77.9200 10

0 -9 -7 39.7474 35.5166 9

0 -9 7 41.1361 26.5996 7

0 9 7 23.3464 46.4174 6

0 -9 7 82.9361 43.5049 8

0 -9 -7 311.240 123.152 8

0 -9 -8 2109.88 206.849 3

0 -9 -8 1727.93 242.606 4

0 -9 -8 1836.13 249.073 1

0 9 8 1739.90 247.247 1

0 9 8 2023.22 236.550 10

0 9 -8 2034.97 246.639 10

0 -9 -8 2093.83 155.724 9

0 -9 -8 1869.38 240.377 6

0 -9 -8 1648.33 201.250 8

0 -9 8 1825.06 141.399 7

0 -9 8 2141.50 230.493 5

0 -9 9 5732.95 331.437 4

0 -9 -9 5137.78 343.416 3

0 -9 -9 4696.46 418.405 1

0 -9 -9 5366.16 272.900 9

0 9 9 5101.34 397.446 10

0 9 -9 5027.41 411.518 10

0 -9 9 5001.54 346.993 5

0 -9 -9 5262.24 420.953 6

0 -9 10 101.459 58.6792 4

0 -9 -10 7.42800 49.4223 1

0 9 10-2.76374 40.2809 10

0 9 -10 25.1439 57.1122 10

0 -9 -10 65.9180 36.0885 9

0 -9 -10 46.2440 44.5991 6

0 -9 11 368.596 81.0562 4

0 -9 -11 352.847 131.990 1

0 9 -11 559.813 161.308 10

0 -9 -11 457.238 89.8752 9

0 -9 -11 470.132 134.618 6

0 9 -12 261.832 104.590 10

0 -9 -12 512.474 96.1773 9

0 -9 -12 662.673 189.015 6

0 -9 -13 985.768 184.837 3

0 9 -13 1069.79 223.437 10

0 9 -13 1218.68 287.914 6

0 -9 -13 1398.61 275.800 6

0 -9 -14 325.463 109.706 3

0 9 -14 476.054 162.733 10

0 9 -14 459.582 171.881 6

0 -9 -14 395.345 158.657 6

0 -9 -15 113.983 64.4955 3

0 9 -15 35.1550 48.0166 10

0 -10 0 94074.8 1155.05 1

0 10 0 86868.0 1047.90 1

0 -10 0 95100.3 1420.50 5

0 10 0 84232.4 1131.71 3

0 10 1 8550.00 360.687 1

0 -10 -1 8214.87 365.692 1

0 10 -1 8130.84 294.220 1

0 -10 1 8011.93 310.924 1

0 10 -1 8807.30 370.763 3

0 -10 -1 8265.38 412.584 5

0 10 1 9029.85 362.425 3

0 -10 1 8416.91 424.369 5

0 -10 -1 8328.37 405.723 8

0 10 2 14826.4 468.368 3

0 10 -2 14416.6 482.608 3

0 -10 2 16869.8 596.700 5

0 -10 -2 14646.9 452.759 3

0 -10 2 17163.2 410.389 1

0 10 -2 14476.2 365.578 1

0 -10 -2 15208.6 545.453 1

0 10 2 13770.7 398.743 9

0 10 -2 14865.4 391.532 7

0 -10 -2 16058.0 571.226 8

0 -10 -3 779.631 114.208 3

0 -10 3 1705.01 197.413 5

0 10 3 857.440 121.193 3

0 -10 -3 1898.76 201.609 1

0 -10 3 1868.16 122.936 1

0 10 3 835.744 93.7182 9

0 10 -3 967.814 102.015 7

0 -10 -3 2139.64 215.790 8

0 -10 -4 209.491 62.0553 3

0 -10 4 160.763 71.9692 5

0 -10 -4 223.831 81.6984 1

0 -10 4 165.499 29.7178 1

0 10 4 225.848 50.8968 9

0 -10 -4 137.868 55.8332 8

0 10 4 185.276 65.2511 6

0 -10 4 240.234 65.6127 8

0 -10 -5 5651.60 295.278 3

0 -10 5 5902.17 378.306 5

0 -10 5 5321.71 132.727 1

0 -10 -5 5225.86 372.321 1

0 10 5 5310.02 148.270 2

0 10 -5 6016.76 352.859 10

0 10 5 5599.81 339.131 10

0 -10 -5 5989.54 242.518 9

0 -10 -5 4612.56 330.836 8

0 -10 5 5100.65 281.795 8

0 10 5 6002.72 400.867 6

0 -10 5 5543.34 224.858 7

0 -10 -6 13387.7 478.711 3

0 -10 -6 11757.3 591.299 4

0 -10 6 14050.3 603.635 5

0 -10 -6 12916.0 634.843 1

0 10 6 12661.9 600.748 1

0 10 6 12832.6 535.421 10

0 -10 -6 12372.6 354.949 9

0 10 -6 14333.0 587.989 10

0 -10 6 11348.3 401.516 8

0 10 6 14336.9 666.467 6

0 -10 -6 14662.4 592.297 8

0 -10 6 12389.0 345.674 7

0 -10 -7 10354.8 444.077 3

0 -10 -7 9723.35 553.400 4

0 10 7 10421.2 557.730 1

0 -10 -7 9534.00 558.060 1

0 10 -7 10585.0 536.152 10

0 10 7 9907.72 507.850 10

0 -10 -7 10045.1 328.867 9

0 -10 7 9509.30 345.430 8

0 -10 -7 8941.01 476.157 8

0 -10 7 10527.8 520.446 5

0 10 7 10706.1 618.274 6

0 -10 7 9348.59 313.551 7

0 -10 -8 1814.27 246.455 4

0 -10 -8 2178.76 214.532 3

0 -10 -8 2677.32 319.122 1

0 10 8 2265.05 256.435 10

0 -10 -8 2139.24 162.633 9

0 10 -8 2341.80 268.341 10

0 -10 8 2342.12 258.773 5

0 -10 8 2236.56 157.375 7

0 -10 -8 2253.73 243.968 8

0 -10 9 445.150 93.4316 4

0 -10 -9 426.635 105.660 3

0 -10 -9 771.188 215.733 1

0 10 9 383.174 111.330 10

0 -10 -9 461.158 80.2933 9

0 10 -9 424.819 122.694 10

0 -10 9 423.567 115.675 5

0 -10 9 462.393 76.6447 7

0 -10 10 138.667 65.1238 4

0 -10 -10 118.825 53.4155 5

0 -10 -10 146.733 79.6572 3

0 -10 -10-36.6630 78.0646 1

0 10 -10 25.6537 50.2557 10

0 -10 -10 227.158 62.0761 9

0 10 10 85.7163 57.7134 10

0 -10 -10 84.9773 56.3885 6

0 -10 10 140.103 43.1171 7

0 -10 -11 308.732 97.8899 3

0 -10 11 190.822 71.7264 4

0 -10 -11 319.962 140.511 1

0 10 -11 186.580 81.0235 10

0 -10 -11 191.761 57.8709 9

0 -10 -11 107.455 54.3472 6

0 -10 -12 358.097 115.814 3

0 10 -12 252.079 110.903 10

0 -10 -12 221.885 119.904 6

0 -10 -13 462.155 129.601 3

0 10 -13 348.924 141.322 10

0 -10 -13 458.304 149.836 6

0 -10 -14 87.3993 80.8869 3

0 10 -14-102.838 89.0248 10

0 10 -15 28.3131 52.3347 10

0 11 0 2197.83 196.766 1

0 -11 0 2763.52 274.760 5

0 -11 0 2672.06 263.330 8

0 -11 1 24864.3 586.898 1

0 -11 -1 24024.7 663.109 1

0 11 -1 24138.9 535.412 1

0 11 1 25066.7 644.871 1

0 11 -1 27845.0 687.860 3

0 -11 -1 25334.5 759.255 5

0 11 1 27855.9 669.502 3

0 -11 1 23935.4 758.830 5

0 -11 -1 24292.3 729.111 8

0 11 2 18227.0 541.683 3

0 -11 -2 16857.2 512.571 3

0 -11 2 18546.5 659.627 5

0 11 -2 17356.6 554.174 3

0 -11 2 18893.9 463.065 1

0 -11 -2 18585.4 617.311 1

0 11 -2 16773.8 415.869 1

0 11 2 17311.8 473.367 9

0 11 -2 16751.2 441.341 7

0 -11 -2 17883.2 636.104 8

0 -11 3 12708.9 558.701 5

0 11 3 12096.5 459.191 3

0 -11 -3 11712.9 424.549 3

0 -11 -3 12771.0 540.964 1

0 -11 3 12019.6 334.604 1

0 11 3 12115.0 378.850 9

0 -11 3 13102.0 501.514 8

0 11 -3 12258.9 369.134 7

0 -11 -3 12568.6 547.202 8

0 -11 -4 536.953 89.8241 3

0 -11 4 627.878 125.333 5

0 -11 4 585.364 62.1846 1

0 -11 -4 526.063 114.754 1

0 11 4 566.237 82.9039 9

0 11 4 670.451 123.918 6

0 -11 4 599.768 105.314 8

0 -11 -4 448.652 107.767 8

0 -11 -5 2777.41 217.266 3

0 -11 5 3397.66 310.299 5

0 11 5 2989.33 142.738 2

0 -11 -5 3841.45 336.107 1

0 -11 5 2404.31 103.441 1

0 11 5 2351.63 227.628 10

0 11 -5 2711.24 248.950 10

0 -11 -5 2479.82 268.438 8

0 -11 5 2399.41 208.730 8

0 11 5 2487.02 269.163 6

0 -11 -6 1018.79 137.091 3

0 -11 -6 813.874 152.204 4

0 -11 6 920.954 163.093 5

0 -11 -6 711.713 166.748 1

0 11 6 741.769 161.866 1

0 11 -6 921.303 146.504 10

0 11 6 840.978 140.787 10

0 -11 6 855.081 116.523 8

0 -11 -6 931.413 102.601 9

0 -11 6 851.835 90.2638 7

0 11 6 923.062 172.134 6

0 -11 -6 822.783 154.646 8

0 -11 -7 1370.03 166.970 3

0 -11 -7 1231.28 203.871 4

0 -11 -7 1625.85 278.718 1

0 11 7 1538.48 235.855 1

0 11 -7 1588.08 224.133 10

0 11 7 1289.42 184.364 10

0 -11 -7 1352.55 125.089 9

0 -11 -7 1605.44 225.132 8

0 -11 7 1334.66 142.147 8

0 -11 7 1244.92 117.971 7

0 -11 7 1470.99 205.240 5

0 -11 -8 144.907 63.0886 3

0 -11 8 96.0827 60.0096 5

0 11 -8 20.8972 48.3972 10

0 11 8 120.009 63.2884 10

0 -11 -8 129.574 43.6255 9

0 -11 -8 121.498 64.3596 8

0 -11 8 95.8207 33.2877 7

0 -11 9 7861.06 400.427 4

0 -11 -9 7189.97 414.028 3

0 -11 -9 6179.57 507.886 1

0 11 -9 6436.55 476.422 10

0 11 9 6925.87 486.432 10

0 -11 -9 7160.48 307.269 9

0 -11 9 7623.20 488.350 5

0 -11 9 6835.48 287.878 7

0 -11 10 141.603 43.5506 4

0 -11 -10 147.865 61.7788 3

0 -11 -10 105.279 41.5353 5

0 -11 -10 113.026 84.2656 1

0 11 -10 236.499 103.217 10

0 -11 10 239.704 51.6865 7

0 -11 -10 166.743 52.0884 9

0 -11 10 203.177 81.5205 5

0 -11 11 425.227 98.9259 4

0 -11 -11 417.179 119.227 3

0 -11 -11 431.243 76.8161 9

0 11 -11 495.255 159.662 10

0 -11 -11 418.972 163.241 1

0 -11 -12 111.056 70.1854 3

0 11 -12 102.988 96.6477 10

0 -11 -12 110.222 99.0363 1

0 -11 -13 884.169 178.987 3

0 11 -13 720.197 185.614 10

0 -11 -14-33.2919 49.0255 3

0 11 -14 54.6942 67.9104 10

0 11 -15 72.5072 54.9458 10

0 -12 0 14846.8 514.894 1

0 12 0 11713.2 424.772 1

0 12 0 10853.7 450.348 3

0 -12 0 13765.2 597.189 5

0 -12 0 13054.4 554.459 8

0 -12 -1 23141.4 681.447 1

0 12 -1 23839.1 568.193 1

0 12 1 24073.8 652.042 1

0 -12 1 23848.3 606.743 1

0 12 -1 26932.9 711.956 3

0 12 1 27518.2 698.953 3

0 -12 -1 25010.5 787.594 5

0 -12 1 24888.2 802.783 5

0 -12 -1 23871.5 757.339 8

0 -12 1 23734.1 739.253 8

0 12 -2 28.1166 41.5421 3

0 12 2 31.0476 33.0637 1

0 -12 2 64.8915 23.7733 1

0 12 -2 55.4350 27.8015 1

0 -12 -2 26.1990 53.3410 1

0 12 2-17.2745 36.2531 3

0 -12 -2-18.1199 41.3931 3

0 -12 2-5.16478 42.4452 5

0 -12 -2 53.3734 25.8208 8

0 12 2 70.7896 30.5843 9

0 12 -2 53.1180 27.9676 7

0 -12 2 46.3592 56.6332 8

0 -12 -3 1888.00 189.386 3

0 -12 3 2934.20 307.234 5

0 12 -3 1828.26 206.354 3

0 12 3 1498.87 172.185 3

0 -12 -3 2911.84 313.006 1

0 -12 3 3607.50 204.300 1

0 12 3 1665.99 150.105 9

0 -12 3 3769.77 296.320 8

0 12 -3 1892.94 158.702 7

0 -12 -3 3577.29 305.660 8

0 -12 4 45.8126 52.8339 5

0 -12 -4 135.732 65.6038 3

0 -12 -4 139.156 84.7118 1

0 -12 4 181.499 48.6745 1

0 12 4 96.6733 42.2616 9

0 -12 -4 84.1966 62.5729 8

0 -12 4 146.401 68.7122 8

0 12 4 140.883 80.4467 6

0 -12 5 4288.38 351.020 5

0 -12 -5 4262.76 274.879 3

0 12 5 3611.25 171.840 2

0 -12 5 3523.33 143.737 1

0 -12 -5 3756.19 332.414 1

0 12 -5 4428.10 320.380 10

0 12 5 4516.50 363.197 6

0 -12 5 3725.55 269.772 8

0 -12 -5 3220.77 300.268 8

0 -12 -6 976.844 140.903 3

0 -12 -6 733.531 157.906 4

0 -12 6 768.821 157.728 5

0 -12 -6 1127.69 213.820 1

0 12 6 871.110 176.123 1

0 12 -6 879.610 154.881 10

0 12 6 671.587 132.755 10

0 12 6 779.099 166.172 6

0 -12 -6 727.865 148.271 8

0 -12 6 863.025 132.341 8

0 -12 7 198.822 84.4815 5

0 -12 -7 268.399 72.5565 3

0 -12 -7 349.433 120.872 1

0 12 7 290.427 93.5766 10

0 12 -7 214.588 83.4142 10

0 -12 -7 215.783 87.3460 8

0 -12 7 188.457 58.9475 8

0 -12 -8 1969.26 211.673 3

0 -12 -8 1536.80 248.761 1

0 12 -8 1911.36 251.179 10

0 12 8 1945.67 249.616 10

0 -12 -8 1762.19 154.237 9

0 -12 -8 1314.85 202.998 8

0 -12 8 1802.13 144.840 7

0 -12 8 1700.84 232.203 5

0 -12 -9 387.517 92.2536 5

0 -12 -9 476.030 104.800 3

0 -12 -9 439.863 135.066 1

0 12 -9 381.442 125.343 10

0 12 9 364.631 116.879 10

0 -12 9 443.580 126.130 5

0 -12 -9 457.034 82.2596 9

0 -12 9 421.918 71.5253 7

0 -12 -10 126.327 53.3459 5

0 -12 10 46.2827 34.9919 4

0 -12 -10 176.217 81.1243 3

0 -12 -10 121.315 78.5679 1

0 12 -10 212.581 109.896 10

0 -12 10 82.6474 45.8393 7

0 -12 -10 143.225 52.0302 9

0 -12 10 60.8766 37.6282 5

0 -12 -11 23.4725 42.9669 3

0 -12 11 94.7020 48.8827 4

0 -12 -11 56.2437 65.2176 1

0 12 -11 95.8595 73.0145 10

0 -12 -11 55.5719 32.9239 9

0 -12 -12-13.4010 44.8699 3

0 12 -12 45.6422 71.5163 10

0 -12 -12-41.6338 59.3178 1

0 -12 -13 28.9075 48.6674 3

0 12 -13 171.690 102.667 10

0 -12 -14 55.8900 53.4492 3

0 12 -14-172.856 99.1116 10

0 12 -15 31.7956 115.569 10

0 13 0 1530.84 170.923 1

0 13 0 1438.09 180.409 3

0 -13 1 5550.51 299.548 1

0 13 1 5608.21 327.145 1

0 13 -1 5631.40 288.566 1

0 -13 -1 5450.16 343.490 1

0 13 -1 6282.21 354.363 3

0 -13 -1 5519.26 386.098 5

0 13 1 6336.41 349.810 3

0 -13 1 5551.47 396.330 5

0 -13 -1 5366.47 375.057 8

0 -13 1 5646.36 376.668 8

0 13 -2 1015.49 145.840 3

0 -13 -2 999.450 165.769 5

0 13 2 1062.68 143.017 3

0 -13 -2 1186.78 147.548 3

0 -13 2 979.985 168.981 5

0 -13 -2 832.431 138.869 1

0 13 -2 839.776 102.351 1

0 13 2 884.960 140.468 1

0 -13 2 971.972 119.793 1

0 13 2 1056.24 128.622 9

0 13 -2 951.592 115.594 7

0 -13 2 1020.81 156.873 8

0 -13 -2 941.387 158.159 8

0 -13 -3 23345.5 655.252 3

0 -13 3 21807.0 793.582 5

0 13 -3 24732.9 730.346 3

0 13 3 24786.1 707.303 3

0 13 -3 21487.8 499.895 1

0 -13 -3 20329.5 724.261 1

0 -13 3 20513.4 511.895 1

0 13 3 23206.8 583.499 9

0 -13 3 21026.9 702.963 8

0 -13 -3 20587.4 756.808 8

0 13 -3 23572.9 562.127 7

0 -13 4 23.4619 39.8027 5

0 -13 -4 37.1627 35.3040 3

0 -13 4 32.0557 25.1273 1

0 -13 -4-48.2953 64.0336 1

0 13 4 24.2642 27.1569 9

0 13 4-8.43334 46.9200 6

0 -13 4 72.7611 45.6624 8

0 -13 -4 73.1022 75.3894 8

0 -13 -5 1391.81 163.606 3

0 -13 5 1210.64 196.930 5

0 -13 -5 1181.18 206.821 1

0 -13 5 1096.67 86.6667 1

0 13 5 1104.40 188.102 1

0 13 -5 1241.05 176.133 10

0 -13 5 1129.60 158.193 8

0 -13 -5 1171.42 190.909 8

0 13 5 1255.24 196.170 6

0 -13 6 8.64157 35.7974 5

0 -13 -6 43.3205 35.9971 3

0 -13 -6-69.0123 80.5960 1

0 13 6-8.00922 54.5853 1

0 13 6 4.85164 35.1018 10

0 13 -6 24.8814 55.2274 10

0 -13 -6-6.73908 62.4727 8

0 -13 6-29.1442 42.6791 8

0 13 6 10.6774 46.0713 6

0 -13 -7 2303.38 224.564 3

0 -13 -7 2238.82 288.468 1

0 13 7 2165.24 250.294 10

0 13 -7 1981.68 250.710 10

0 -13 7 1782.19 188.935 8

0 -13 7 1971.39 269.012 5

0 -13 -7 1870.74 247.370 8

0 -13 -8 600.459 120.573 3

0 -13 -8 520.874 116.834 5

0 -13 8 618.496 144.290 5

0 -13 -8 468.134 153.286 1

0 13 8 535.887 132.968 10

0 13 -8 441.466 131.789 10

0 -13 -8 478.499 126.174 8

0 -13 -9 713.169 134.411 5

0 -13 9 758.327 164.348 5

0 -13 -9 704.095 129.258 3

0 -13 -9 824.321 185.412 1

0 13 -9 860.486 189.182 10

0 -13 10 252.473 82.2999 4

0 -13 -10 230.791 79.4017 3

0 -13 -10 180.842 112.965 1

0 13 -10 155.031 92.7697 10

0 -13 -10 257.080 66.1756 9

0 -13 10 174.559 90.1756 5

0 -13 -11 345.892 105.585 3

0 13 -11 355.564 128.706 10

0 -13 -11 291.725 121.343 1

0 -13 -12 107.066 64.7657 3

0 13 -12 51.1885 54.0034 10

0 -13 -12-16.5622 106.558 1

0 -13 -13 116.040 76.1339 3

0 13 -13 48.3457 72.8231 10

0 -13 -14-97.6510 90.5723 3

0 13 -14-82.2903 74.7647 10

0 -14 0 10058.0 460.635 1

0 14 0 8300.25 385.723 1

0 14 0 7625.82 406.698 3

0 -14 0 9491.75 535.798 5

0 -14 0 9006.00 502.211 8

0 14 -1 5335.33 293.892 1

0 14 1 4984.27 318.116 1

0 -14 1 5252.59 312.857 1

0 -14 -1 5432.37 356.197 1

0 14 -1 6241.62 367.929 3

0 -14 -1 5710.66 410.874 5

0 14 1 6157.77 358.851 3

0 -14 1 5493.56 404.656 5

0 -14 -1 5026.04 375.148 8

0 -14 1 5217.20 373.848 8

0 14 2 672.589 131.157 1

0 -14 -2 757.195 137.614 1

0 -14 2 749.797 111.542 1

0 14 -2 652.863 98.0416 1

0 -14 -2 737.162 156.310 5

0 -14 -2 606.282 111.722 3

0 14 2 678.846 122.479 3

0 14 -2 745.231 130.499 3

0 -14 2 716.158 154.549 5

0 -14 2 736.250 145.041 8

0 -14 -2 771.089 154.073 8

0 14 -2 607.578 101.549 7

0 14 2 577.194 102.830 9

0 14 3 2842.94 255.459 3

0 -14 3 5032.59 404.587 5

0 -14 -3 5697.17 412.488 5

0 14 -3 3427.29 280.716 3

0 -14 -3 3165.77 249.388 3

0 -14 3 4717.57 258.307 1

0 14 3 5087.14 366.679 1

0 -14 -3 4098.54 365.528 1

0 14 -3 3360.00 210.793 1

0 14 3 3074.48 222.367 9

0 14 -3 3149.50 219.658 7

0 -14 3 3826.44 348.726 8

0 -14 -3 4550.71 368.544 8

0 -14 -4 225.493 77.9088 3

0 14 4 374.330 98.2956 3

0 -14 -4 218.956 91.0179 5

0 -14 4 253.019 87.7158 5

0 14 4 271.663 99.9415 1

0 -14 4 186.856 44.8375 1

0 -14 -4 337.875 114.401 1

0 14 -4 349.905 101.725 3

0 14 4 218.171 60.8111 9

0 14 -4 240.783 90.3554 10

0 14 4 270.905 95.1232 6

0 -14 4 145.486 61.4487 8

0 -14 -4 69.5583 49.8263 8

0 -14 5 786.295 168.864 5

0 -14 -5 406.885 99.2537 3

0 14 5 574.058 186.185 1

0 -14 5 357.655 55.3280 1

0 -14 -5 552.736 165.993 1

0 14 5 310.005 68.6112 9

0 14 -5 589.409 133.507 10

0 -14 -5 698.368 174.027 8

0 -14 5 508.966 147.998 8

0 14 5 332.636 108.529 6

0 -14 6 1.41630 40.3796 5

0 -14 -6-35.6258 53.0050 5

0 -14 -6-42.6049 52.0866 3

0 14 6-8.09574 12.3570 2

0 -14 -6 77.3028 67.4672 1

0 14 6 20.8525 26.3684 10

0 14 -6-70.7288 68.1122 10

0 -14 6 4.88483 37.3511 8

0 -14 -6-8.24204 47.1062 8

0 -14 -7 7440.99 413.757 3

0 -14 -7 6020.54 413.130 5

0 -14 -7 6607.34 510.871 1

0 14 7 6646.33 457.141 10

0 14 -7 7334.56 488.571 10

0 -14 7 6999.73 508.731 5

0 -14 -7 5777.41 444.359 8

0 -14 7 5930.21 366.106 8

0 -14 -8 1389.79 199.481 5

0 -14 -8 1708.08 203.884 3

0 -14 -8 1488.08 269.722 1

0 14 -8 1672.79 241.736 10

0 14 8 1742.10 241.586 10

0 -14 -8 1667.93 239.959 8

0 -14 8 2223.70 292.038 5

0 -14 8 1649.51 183.674 8

0 -14 9 279.808 104.244 5

0 -14 -9 284.164 92.0798 5

0 -14 -9 298.064 89.1897 3

0 -14 -9 351.025 134.396 1

0 14 -9 260.720 99.0674 10

0 -14 -10 104.521 52.9141 3

0 -14 10 41.4702 41.6702 4

0 -14 -10 35.2626 53.2757 1

0 14 -10 35.6513 45.4982 10

0 -14 10-13.3978 66.6013 5

0 -14 -11 251.213 87.0827 3

0 14 -11 237.807 122.044 10

0 -14 -11 275.155 120.476 1

0 -14 -12 13.9609 41.7049 3

0 14 -12-93.3590 79.5209 10

0 -14 -12-184.762 108.758 1

0 -14 -13 80.0910 65.6568 3

0 14 -13 30.4150 68.6784 10

0 -14 -14 44.7341 61.0267 3

0 14 -14-30.1610 49.0118 10

0 15 0 549.406 118.917 1

0 -15 0 882.136 184.618 5

0 15 0 515.384 122.600 3

0 -15 0 614.750 151.471 8

0 -15 -1 929.340 162.808 1

0 -15 1 1040.67 149.842 1

0 -15 -1 508.890 124.508 3

0 15 1 692.013 127.770 1

0 15 -1 796.963 120.859 1

0 15 1 195.062 81.3362 3

0 -15 -1 940.051 186.268 5

0 15 -1 286.686 91.5687 3

0 -15 1 1017.48 186.241 5

0 -15 -1 943.650 180.072 8

0 -15 1 893.472 154.640 8

0 -15 -2 4331.00 370.694 5

0 -15 2 4178.66 366.235 5

0 15 2 4788.03 328.661 3

0 -15 -2 4089.76 295.126 3

0 15 -2 4780.80 337.911 3

0 -15 2 3806.38 267.915 1

0 15 -2 4081.42 250.668 1

0 -15 -2 3790.46 324.437 1

0 15 2 4253.73 321.394 1

0 15 2 4123.57 276.227 9

0 15 -2 4873.06 282.411 7

0 -15 2 4011.15 343.388 8

0 -15 -2 4438.29 369.125 8

0 -15 -3 13866.9 663.296 5

0 15 3 14234.1 576.348 3

0 -15 -3 13291.9 530.700 3

0 -15 3 13394.2 676.634 5

0 15 -3 13540.8 581.091 3

0 -15 3 13029.1 453.200 1

0 15 3 14115.9 617.706 1

0 -15 -3 12963.6 620.552 1

0 15 -3 12459.9 425.500 1

0 15 3 12907.7 478.869 9

0 15 -3 13563.3 464.644 7

0 -15 3 13658.0 626.621 8

0 -15 -3 12649.4 638.791 8

0 -15 4 828.464 169.879 5

0 15 4 981.377 153.716 3

0 -15 -4 1118.58 202.330 5

0 -15 -4 801.957 134.267 3

0 15 -4 761.426 143.788 3

0 15 4 1091.37 181.521 1

0 -15 -4 1045.85 194.452 1

0 -15 4 982.446 117.978 1

0 15 4 809.096 117.685 9

0 15 -4 848.448 153.173 10

0 -15 -4 698.265 163.824 8

0 -15 4 838.300 155.033 8

0 -15 -5 6490.89 450.603 5

0 -15 5 7728.46 525.651 5

0 -15 -5 6717.33 388.460 3

0 -15 5 5936.27 246.397 1

0 -15 -5 6465.13 491.824 1

0 15 -5 7046.71 445.379 3

0 15 -5 6962.16 440.335 10

0 15 5 6418.34 330.817 9

0 -15 -5 6181.16 457.478 8

0 -15 5 6032.64 406.700 8

0 -15 6 278.971 106.673 5

0 -15 -6 200.214 78.5492 3

0 -15 -6 226.100 92.2374 5

0 15 6 193.899 44.7825 2

0 -15 -6 408.674 150.315 1

0 -15 6 270.136 39.3745 1

0 15 -6 193.355 61.7154 10

0 15 6 143.044 70.5712 10

0 -15 -6 234.314 97.2614 8

0 -15 6 257.435 91.0325 8

0 -15 -7 1369.19 203.759 5

0 -15 -7 1690.99 200.060 3

0 -15 7 1872.88 274.248 5

0 -15 -7 1673.94 265.082 1

0 15 7 1673.83 237.536 10

0 15 -7 2007.97 262.287 10

0 -15 7 1535.23 196.408 8

0 -15 -8 72.1238 49.4183 3

0 -15 8 66.4913 64.1435 5

0 -15 -8 124.962 63.4664 5

0 -15 -8 115.296 72.1583 1

0 15 -8 211.714 103.218 10

0 -15 8 117.006 52.3572 8

0 -15 -9 529.801 126.176 5

0 -15 9 499.304 148.820 5

0 -15 -9 611.386 131.386 3

0 15 -9 620.676 163.129 10

0 -15 -9 478.758 139.585 1

0 -15 -10 90.7178 68.9573 3

0 15 -10 166.110 98.1329 10

0 -15 -10 35.4700 54.4746 1

0 -15 -11 33.9283 50.9421 3

0 15 -11-55.3366 80.3442 10

0 -15 -11 46.1906 71.2506 1

0 -15 -12 24.0577 49.2451 3

0 15 -12-31.6356 67.5930 10

0 -15 -12-77.9419 114.404 1

0 -15 -13 69.6998 62.9954 3

0 15 -13 0.26187 53.8902 10

0 -15 -14-97.1896 104.002 3

0 15 -14 47.0061 78.8580 10

0 16 0 918.638 142.172 1

0 -16 0 1309.18 186.739 1

0 -16 0 967.302 186.535 5

0 16 0 907.840 154.269 3

0 -16 0 971.842 180.287 8

0 -16 1 31886.0 840.759 1

0 -16 -1 31345.9 911.406 1

0 16 1 31203.0 851.079 1

0 16 -1 31540.1 774.766 1

0 -16 1 32076.7 1055.06 5

0 16 1 37123.7 956.922 3

0 -16 -1 36296.4 910.080 3

0 16 -1 37296.0 979.658 3

0 -16 -1 32745.6 1060.40 5

0 -16 1 33391.8 1032.75 8

0 -16 -1 32967.4 1048.65 8

0 -16 2 3131.89 330.778 5

0 -16 -2 3287.23 337.838 5

0 16 -2 2926.60 275.136 3

0 16 2 3053.29 271.607 3

0 -16 -2 2931.08 255.613 3

0 16 2 3151.75 283.211 1

0 -16 -2 3009.99 305.586 1

0 -16 2 3188.60 247.447 1

0 16 -2 2913.50 226.308 1

0 16 2 2800.84 235.727 9

0 -16 -2 3282.31 333.248 8

0 16 -2 3112.97 239.437 7

0 -16 2 3118.86 311.819 8

0 16 3 1452.80 192.515 3

0 -16 -3 1332.07 227.895 5

0 -16 -3 1124.00 157.263 3

0 16 -3 1484.60 200.596 3

0 -16 3 1123.27 212.048 5

0 -16 -3 1322.20 209.563 1

0 16 3 1385.66 202.268 1

0 16 -3 1206.34 134.701 1

0 -16 3 1355.62 155.162 1

0 16 3 1316.38 156.285 9

0 16 -3 1438.40 156.115 7

0 -16 3 1617.23 222.113 8

0 -16 -3 1091.47 197.934 8

0 16 4 82.5846 49.3414 3

0 -16 4 125.367 78.0088 5

0 -16 -4 46.1220 34.1310 5

0 -16 -4 100.363 69.8841 3

0 16 4 68.4252 81.2913 1

0 16 -4 51.0702 56.4547 3

0 -16 -4 130.271 95.6709 1

0 -16 4 43.7613 38.0274 1

0 16 -4 154.334 81.3529 10

0 16 4 38.6912 34.8093 9

0 -16 -4-17.2475 61.4745 8

0 16 -4 74.7901 51.0161 7

0 -16 4-21.9498 51.3779 8

0 -16 -5 1783.95 244.250 5

0 -16 -5 1934.29 214.970 3

0 -16 5 1806.62 261.053 5

0 -16 5 1752.95 143.502 1

0 -16 -5 1811.31 260.066 1

0 16 -5 1973.70 240.986 10

0 16 5 1823.10 183.287 9

0 -16 5 1592.11 213.455 8

0 -16 6 295.592 119.926 5

0 -16 -6 86.2237 92.9615 5

0 -16 -6 203.074 79.9156 3

0 -16 6 76.2481 19.2870 1

0 -16 -6 134.912 105.106 1

0 16 6 167.778 46.6420 2

0 16 6 119.907 65.7316 10

0 16 -6 139.682 64.0409 10

0 -16 6 121.720 67.8342 8

0 -16 -7 633.436 146.153 5

0 -16 -7 818.310 139.309 3

0 -16 -7 981.104 210.070 1

0 16 -7 764.666 162.266 10

0 -16 7 603.528 120.875 8

0 -16 -8 108.331 62.8296 3

0 -16 -8 70.4387 39.0514 5

0 -16 -8 78.2750 61.7980 1

0 16 -8 131.364 89.0788 10

0 -16 8 102.020 63.7605 8

0 -16 -9 330.191 98.5822 3

0 -16 -9 253.489 99.3264 5

0 16 -9 241.423 106.077 10

0 -16 -9 298.415 132.070 1

0 -16 -10-3.52950 56.1330 3

0 16 -10 115.786 86.9824 10

0 -16 -10 13.2759 93.2895 1

0 -16 -11 56.0516 39.1470 3

0 16 -11-12.3399 71.3814 10

0 -16 -11 84.4427 87.5685 1

0 -16 -12 2.95682 39.1087 3

0 16 -12 38.7930 82.3141 10

0 -16 -12-97.8979 96.7172 1

0 -16 -13 94.3306 62.1223 3

0 16 -13-20.2626 67.2804 10

0 17 0 240.584 85.3840 1

0 -17 0 385.951 119.501 1

0 17 0 249.705 89.3618 3

0 -17 0 295.356 103.266 5

0 -17 0 192.875 81.8398 8

0 -17 1 3611.23 304.418 1

0 -17 -1 3864.59 337.936 1

0 -17 -1 4152.59 330.733 3

0 17 1 3941.85 315.943 1

0 17 -1 3888.51 280.673 1

0 -17 1 3711.54 376.055 5

0 17 -1 4447.71 347.285 3

0 -17 -1 3992.93 375.850 5

0 17 1 4264.96 331.683 3

0 -17 1 3843.86 363.295 8

0 -17 -1 4006.84 377.916 8

0 17 -2 873.480 157.394 3

0 -17 -2 1291.88 213.385 5

0 17 2 863.667 148.727 3

0 -17 2 1196.39 221.401 5

0 17 -2 994.296 138.784 1

0 17 2 1044.24 173.607 1

0 -17 2 1069.70 159.254 1

0 -17 -2 825.947 145.823 3

0 -17 -2 955.821 176.601 1

0 17 2 983.511 150.437 9

0 -17 -2 1165.60 206.467 8

0 17 -2 913.037 134.246 7

0 -17 2 938.211 183.403 8

0 17 3 8587.67 485.590 3

0 -17 -3 8343.78 556.573 5

0 -17 3 8714.19 583.264 5

0 -17 -3 8548.55 453.110 3

0 17 3 8661.15 508.024 1

0 17 -3 7780.49 365.150 1

0 -17 3 7715.37 395.247 1

0 -17 -3 8266.01 542.184 1

0 17 3 7921.35 404.041 9

0 -17 3 8357.24 525.077 8

0 17 -3 8483.41 400.615 7

0 17 4 453.575 122.087 3

0 -17 -4 309.447 103.832 3

0 -17 -4 820.035 207.567 1

0 -17 4 761.608 127.979 1

0 17 4 393.142 90.8885 9

0 17 -4 512.562 127.362 10

0 17 -4 520.748 104.352 7

0 -17 4 567.133 176.651 8

0 -17 -5 125.058 51.7771 5

0 -17 -5 127.061 70.1599 3

0 -17 5 125.113 47.9460 1

0 17 5 75.5488 36.0765 9

0 17 -5 166.319 99.8980 10

0 -17 5 231.243 120.114 8

0 -17 -6-10.5225 49.3806 5

0 -17 -6 50.3710 52.7679 3

0 17 6 58.6689 32.8481 2

0 -17 -6 1.52444 85.9433 1

0 -17 6 35.1748 17.6081 1

0 17 -6 44.6606 60.9585 10

0 -17 6 50.0309 37.5111 8

0 -17 -7 864.476 156.895 3

0 -17 -7 621.888 149.135 5

0 -17 -7 933.751 218.198 1

0 17 -7 927.848 190.300 10

0 -17 7 729.426 146.080 8

0 -17 -8-2.52486 31.7234 5

0 -17 -8 65.5321 50.7118 3

0 -17 -8 69.5441 60.6584 1

0 17 -8 61.6910 70.3210 10

0 -17 8-38.9520 52.0401 8

0 -17 -9 317.625 94.3127 3

0 17 -9 320.691 125.445 10

0 -17 -9 225.500 133.475 1

0 -17 -10 103.889 67.0794 3

0 17 -10 14.9988 53.8139 10

0 -17 -10 61.1061 100.994 1

0 -17 -11 27.6121 38.2229 3

0 17 -11 104.641 93.5322 10

0 -17 -12 23.9367 36.0684 3

0 17 -12-60.5466 46.8063 10

0 -17 -12 66.4818 98.1695 1

0 -17 -13 204.175 102.324 3

0 17 -13 0.27873 83.0541 10

0 18 0 734.472 134.154 1

0 -18 0 869.822 162.229 1

0 18 0 701.703 144.955 3

0 -18 0 594.276 133.964 8

0 18 -1 37091.4 913.511 1

0 -18 1 35864.1 971.874 1

0 -18 -1 36909.3 1060.54 1

0 18 1 38084.3 999.478 1

0 18 1 43979.0 1111.80 3

0 18 -1 42003.2 969.500 7

0 -18 1 37614.7 1181.97 8

0 -18 -2 1426.48 188.482 3

0 18 2 1580.03 212.571 3

0 -18 -2 1926.75 277.580 5

0 18 -2 1619.26 180.151 1

0 -18 -2 1581.11 239.404 1

0 18 2 1776.87 224.969 1

0 -18 2 1901.57 205.735 1

0 18 2 1516.30 189.326 9

0 -18 2 1837.84 262.558 8

0 18 -2 1552.90 181.509 7

0 -18 -3 4852.82 435.095 5

0 -18 -3 3156.84 292.073 3

0 -18 3 4106.06 301.678 1

0 -18 -3 4352.57 415.661 1

0 18 -3 3481.63 259.852 1

0 18 3 3135.03 263.474 9

0 -18 3 4097.12 381.063 8

0 18 -3 3448.21 266.846 7

0 -18 -4 543.760 147.491 5

0 -18 -4 837.500 152.561 3

0 -18 -4 388.413 120.307 1

0 -18 4 667.463 108.747 1

0 18 4 697.702 122.755 9

0 18 -4 861.045 162.351 10

0 18 -4 618.416 114.358 7

0 -18 4 550.837 130.139 8

0 -18 -5 80.8553 63.0002 3

0 -18 5 65.8186 44.4680 1

0 -18 -5 235.512 159.496 1

0 18 5 21.0966 33.3805 9

0 18 -5 24.7326 47.1111 10

0 -18 5 161.580 115.874 8

0 -18 -6 218.697 88.0704 3

0 18 6 150.458 69.0992 2

0 -18 -6 203.873 133.255 1

0 -18 6 82.7546 33.5647 1

0 18 -6 170.530 84.7119 10

0 -18 6 112.049 50.7679 8

0 -18 -7 976.329 170.342 3

0 -18 -7 950.156 222.491 1

0 18 -7 962.998 194.540 10

0 -18 7 728.502 155.380 8

0 -18 -8 45.4112 34.6306 3

0 -18 -8 119.064 108.520 1

0 18 -8-0.95313 51.8134 10

0 -18 8 34.0899 34.8865 8

0 -18 -9 63.7950 47.9000 3

0 18 -9 64.5075 73.1587 10

0 -18 -9-16.3610 72.1131 1

0 -18 -10 82.7147 64.2469 3

0 18 -10-0.22411 51.3961 10

0 -18 -10 79.2575 124.859 1

0 -18 -11 68.7835 59.0051 3

0 18 -11 52.7269 67.1570 10

0 -18 -11-129.979 97.7537 1

0 -18 -12 0.16516 37.2261 3

0 18 -12 72.7068 48.3838 10

0 -18 -12 123.412 133.761 1

0 -18 -13 78.2291 69.6855 3

0 -19 0 320.566 120.727 1

0 19 0 120.170 69.1431 1

0 -19 1 812.412 138.289 1

0 19 1 828.880 154.674 1

0 -19 -1 903.115 173.414 1

0 19 -1 871.207 133.781 1

0 19 -1 1026.15 160.248 7

0 -19 -2 156.179 103.469 1

0 -19 2 79.6537 47.9310 1

0 19 2 138.610 77.0684 1

0 -19 -2 89.1853 68.8996 3

0 19 -2 90.0788 48.1552 1

0 19 -2 85.8152 59.0876 7

0 19 2 123.574 61.9107 9

0 -19 3 1244.30 174.434 1

0 -19 -3 1212.43 239.298 1

0 19 -3 485.127 106.892 1

0 -19 -3 414.155 123.896 3

0 -19 3 1159.56 213.185 8

0 19 3 149.869 74.7204 9

0 19 -3 360.416 100.918 7

0 -19 -4 4314.85 345.880 3

0 -19 -4 3894.84 407.054 1

0 -19 4 3701.53 275.367 1

0 19 4 4257.43 311.888 9

0 19 -4 4568.86 391.413 10

0 -19 4 4013.99 385.055 8

0 19 -4 4281.78 302.580 7

0 -19 -5 19.0903 43.3430 3

0 -19 5 83.4285 41.2631 1

0 19 -5-56.1769 72.3742 10

0 19 5 91.4253 48.9224 9

0 -19 5 139.735 74.3947 8

0 -19 -6-43.6164 62.4362 3

0 -19 6 11.9874 22.5286 1

0 -19 -6 73.0730 63.1581 1

0 19 6 8.58194 29.4141 2

0 19 -6-31.5046 56.6339 10

0 -19 6-109.312 73.0064 8

0 -19 -7 340.221 103.968 3

0 -19 -7 384.146 154.788 1

0 19 -7 302.003 105.849 10

0 -19 7 209.507 87.4299 8

0 -19 -8 212.390 86.0826 3

0 19 -8 192.149 104.768 10

0 -19 -8 20.8427 67.5824 1

0 -19 -9 92.2727 69.3306 3

0 19 -9-18.8915 75.0589 10

0 -19 -9 77.1967 76.8746 1

0 -19 -10 34.8814 53.0274 3

0 19 -10-24.0650 47.4175 10

0 -19 -10-56.4605 79.5265 1

0 -19 -11 41.5789 51.7781 3

0 19 -11-68.2398 74.0453 10

0 -19 -11 32.0557 68.9399 1

0 -19 -12-33.3053 47.7493 3

0 19 -12 134.928 114.474 10

0 -19 -13-264.315 108.458 3

0 -20 0 4078.11 368.413 1

0 20 0 3472.08 306.911 1

0 -20 -1 6102.15 456.011 1

0 -20 1 6400.27 436.658 1

0 20 1 6376.01 438.749 1

0 20 -1 6358.73 403.770 1

0 20 -1 7658.26 451.578 7

0 -20 2 420.041 108.956 1

0 -20 -2 235.306 88.4394 3

0 -20 -2 315.215 100.553 1

0 20 -2 422.265 99.5195 1

0 20 2 404.324 104.625 9

0 20 -2 382.112 93.2699 7

0 20 -3 45.5028 45.9521 1

0 -20 -3 15.6065 59.6207 1

0 -20 -3 98.1181 66.1123 3

0 -20 3 17.7896 34.8790 1

0 20 3 11.0909 40.9522 9

0 20 -3 20.8975 47.6614 7

0 -20 4 93.4711 51.3177 1

0 -20 -4 1.96262 46.1231 3

0 -20 -4 36.5904 49.5119 1

0 20 4 19.3813 23.6357 9

0 20 -4 32.4561 31.3061 7

0 -20 -5 291.689 99.1888 3

0 -20 5 351.175 92.7376 1

0 20 5 306.941 88.2145 9

0 20 -5 502.125 148.383 10

0 -20 -6 77.2340 55.1213 3

0 -20 6 49.1992 28.3020 1

0 20 6 55.6582 56.4144 2

0 -20 -6 220.108 138.878 1

0 20 -6 8.53718 40.1682 10

0 -20 6 106.079 88.9534 8

0 -20 -7-38.1518 46.1303 3

0 -20 -7-101.613 115.015 1

0 20 -7-21.2571 52.4699 10

0 -20 7-117.301 96.5411 8

0 -20 -8 94.1469 56.5404 3

0 20 -8 166.474 77.1397 10

0 -20 -8 212.854 127.835 1

0 -20 -9 350.421 113.901 3

0 20 -9 313.519 144.149 10

0 -20 -9 496.915 169.952 1

0 -20 -10-60.6997 51.6152 3

0 20 -10 75.2267 75.8882 10

0 -20 -10-64.1431 95.7030 1

0 -20 -11 48.0109 55.2103 3

0 20 -11 57.7886 80.1258 10

0 -20 -11 9.16091 85.9935 1

0 -20 -12-58.2485 47.3991 3

0 21 0 78.1009 44.4008 1

0 -21 0 215.639 101.143 1

0 21 -1 195.298 82.0425 1

0 -21 1 185.935 88.5838 1

0 -21 -1 364.143 132.712 1

0 21 -1 150.157 63.6151 7

0 -21 -2-9.95084 38.0589 3

0 -21 2 77.3540 43.4725 1

0 21 -2 19.5363 33.5819 1

0 21 2 58.3485 57.2548 9

0 21 -2 80.8353 65.2482 7

0 -21 3 302.687 89.8590 1

0 21 -3 395.446 101.229 1

0 -21 -3 329.356 114.167 3

0 -21 -3 305.836 131.338 1

0 21 -3 264.477 84.2434 7

0 21 3 340.397 101.339 9

0 -21 -4 910.911 209.803 1

0 -21 -4 715.890 148.625 3

0 -21 4 820.361 146.003 1

0 21 4 612.969 130.533 9

0 21 -4 694.299 156.854 10

0 21 -4 653.163 130.950 7

0 -21 -5 164.157 86.2027 3

0 21 5 35.2736 28.3376 9

0 21 -5 108.565 58.5606 10

0 -21 -5 20.5301 79.4854 1

0 21 5 77.3636 64.7614 2

0 -21 5-6.53705 42.1474 1

0 -21 -6 20.3956 42.8434 3

0 21 -6 121.839 87.0685 10

0 -21 -6 4.18269 71.4707 1

0 -21 6 68.5198 31.7314 1

0 -21 -7 149.747 81.1474 3

0 21 -7 110.371 72.4251 10

0 -21 -7 169.387 125.791 1

0 -21 -8 31.4220 40.4782 3

0 21 -8 33.6589 56.7130 10

0 -21 -8-30.9913 95.6488 1

0 -21 -9 139.452 78.2664 3

0 21 -9 16.8511 71.4656 10

0 -21 -9 73.7862 110.144 1

0 -21 -10 70.4052 58.8896 3

0 21 -10 192.069 124.414 10

0 -21 -10 26.8150 59.7706 1

0 -21 -11 28.4860 50.2915 3

0 21 -11-72.6870 76.1403 10

0 -21 -12 88.1990 62.5616 3

0 -22 0 2036.87 280.315 1

0 -22 -1 1057.75 203.376 1

0 -22 1 949.518 181.487 1

0 22 -1 1046.68 177.279 1

0 22 -1 1292.76 194.989 7

0 -22 -2 770.573 183.070 1

0 -22 2 692.821 156.905 1

0 22 -2 591.917 129.778 1

0 22 -2 647.687 139.533 7

0 -22 3 310.263 104.535 1

0 -22 -3 221.202 90.9422 3

0 -22 -3 315.063 135.118 1

0 22 -3 188.134 74.3575 1

0 22 -3 228.540 88.4009 7

0 22 3 199.489 78.9098 9

0 -22 -4 96.1233 95.6264 1

0 -22 4-4.76974 50.0162 1

0 -22 -4-20.9722 38.8741 3

0 22 4-26.6321 43.2925 9

0 22 -4-17.3312 52.3676 10

0 22 -4-12.7793 47.5536 7

0 22 -5 145.884 95.5739 10

0 22 5 172.742 76.2834 9

0 -22 -5 171.318 86.4492 3

0 22 5 96.3593 57.9827 2

0 -22 -5 116.676 71.9469 1

0 -22 5 141.388 55.7543 1

0 -22 -6 299.145 99.2561 3

0 22 -6 289.237 137.409 10

0 -22 -6 422.557 170.425 1

0 -22 -7 44.7598 65.9763 3

0 22 -7 6.78102 55.7281 10

0 -22 -7 47.5197 88.3109 1

0 -22 -8 31.4326 54.1454 3

0 22 -8-15.5997 55.8945 10

0 -22 -8 131.079 110.379 1

0 -22 -9 39.5805 71.6828 3

0 22 -9 0.24834 58.9999 10

0 -22 -9-65.6772 102.758 1

0 -22 -10 58.7186 81.9205 3

0 22 -10 19.6601 50.6884 10

0 -22 -11-17.2169 48.0962 3

0 -22 -12-350.528 127.942 3

0 -23 0 160.715 82.8198 1

0 23 0 81.7860 54.2611 7

0 -23 -1 38.2591 58.4740 1

0 -23 1-14.1215 45.5399 1

0 23 -1 3.83751 33.6700 1

0 23 -1 34.0925 47.5328 7

0 23 -2 92.5108 51.1592 1

0 -23 -2 134.493 99.7493 1

0 -23 2 138.410 68.4853 1

0 23 -2 96.7973 48.5304 7

0 -23 3 97.9950 47.8512 1

0 23 -3 54.7105 54.6344 1

0 -23 -3 147.560 88.4742 3

0 -23 -3 53.8049 46.7337 1

0 23 -3 44.3388 41.5618 7

0 -23 4 415.123 111.874 1

0 -23 -4 498.961 141.900 1

0 -23 -4 425.061 133.167 3

0 23 -4 207.502 100.034 10

0 23 -4 438.620 111.237 7

0 23 -5 137.300 106.699 10

0 -23 5 97.3563 32.1342 1

0 -23 -5 102.643 68.1469 3

0 -23 -5 183.141 115.659 1

0 -23 -6-26.5578 51.4434 3

0 23 -6 95.9368 85.8990 10

0 -23 -6 12.7319 60.0622 1

0 -23 -7-141.435 81.6436 3

0 23 -7-11.3692 69.4167 10

0 -23 -7-35.0242 64.1005 1

0 -23 -8 196.696 105.027 3

0 23 -8 58.4840 60.8221 10

0 -23 -8 170.831 109.615 1

0 -23 -9 37.3793 55.7695 3

0 23 -9-130.694 92.1219 10

0 -23 -9 25.5382 58.5164 1

0 -23 -10-144.325 91.5340 3

0 -23 -11 49.5994 43.8196 3

0 -24 0 345.716 123.331 1

0 24 0 324.166 118.715 7

0 -24 -1 315.790 114.088 1

0 -24 1 501.005 150.192 1

0 24 -1 506.022 128.855 7

0 -24 2 56.0769 52.2814 1

0 24 -2 14.1919 48.8417 1

0 -24 -2-10.8005 80.3447 1

0 24 -2 35.0997 46.0222 7

0 -24 -3 124.829 63.4356 1

0 -24 3 40.5549 52.8442 1

0 24 -3 188.009 100.559 7

0 -24 -4-41.1446 56.1423 1

0 -24 -4 56.5568 55.3939 3

0 -24 4 37.1320 43.6853 1

0 24 -4-3.74105 67.2112 10

0 24 -4-43.0320 65.1197 7

0 24 -5 65.9684 77.4337 10

0 -24 -5 9.90116 51.8004 3

0 -24 -5-104.111 106.763 1

0 -24 5-81.9722 71.7652 1

0 24 -6-60.1857 66.0235 10

0 -24 -6-76.9799 76.6947 3

0 -24 -7 23.0926 44.8207 3

0 24 -7-33.7641 77.7010 10

0 -24 -7 85.8609 102.742 1

0 -24 -8 45.5458 64.1113 3

0 24 -8 95.6891 86.0587 10

0 -24 -8-30.5274 70.0192 1

0 -24 -9 5.53935 61.8035 3

0 24 -9 11.9363 58.6775 10

0 -24 -10 87.5327 76.1232 3

0 -24 -11-40.1448 54.0595 3

0 -25 0 180.343 92.3166 1

0 25 0 187.180 90.2805 7

0 -25 1-83.3351 71.5152 1

0 -25 -1 72.3781 93.3386 1

0 25 -1 116.356 71.1388 7

0 -25 2 71.6367 66.7887 1

0 -25 -2 135.072 100.993 1

0 25 -2 119.358 66.9254 7

0 -25 -3 338.572 129.733 1

0 -25 3 245.616 100.600 1

0 25 -3 120.685 67.6151 7

0 -25 -4 79.4993 39.7561 3

0 -25 -4 90.0690 117.317 1

0 -25 4-12.2253 53.1278 1

0 25 -4 210.385 119.018 10

0 25 -4 112.984 64.9860 7

0 25 -5-43.6341 60.6444 10

0 -25 -5 55.1369 60.7484 3

0 25 -6 67.8785 90.0651 10

0 -25 -6 5.83809 56.5233 3

0 -25 -6 26.8413 54.0742 1

0 25 -7-69.7805 65.7029 10

0 -25 -7-29.7041 58.6671 3

0 -25 -7-9.05895 59.7078 1

0 -25 -8-103.935 75.1285 3

0 25 -8-161.342 114.661 10

0 -25 -8-169.723 131.063 1

0 -25 -9-18.6465 61.7134 3

0 -25 -10-213.684 103.386 3

0 -26 0 291.063 130.265 1

0 26 0 480.633 137.043 7

0 -26 1 329.625 128.774 1

0 -26 -1 522.300 173.886 1

0 26 -1 397.761 124.995 7

0 -26 -2 31.0240 77.3555 1

0 -26 2-83.5612 88.8491 1

0 26 -2 13.4706 55.8774 7

0 -26 -3 91.0306 90.7188 1

0 -26 3 121.808 74.3349 1

0 26 -3 0.15376 42.0311 7

0 -26 4-27.4025 77.0434 1

0 -26 -4-63.9111 56.6471 1

0 26 -4-73.2699 74.3241 10

0 26 -4-2.37245 46.9340 7

0 26 -5-87.0780 76.2236 10

0 -26 -5-69.5307 81.4782 1

0 -26 -5 70.0930 77.0730 3

0 26 -6 21.0721 77.4248 10

0 -26 -6 53.3755 66.5889 1

0 -26 -6 62.5873 82.4066 3

0 26 -7 70.2534 48.0043 10

0 -26 -7 24.6625 70.6914 3

0 -26 -8 15.1943 41.8014 3

0 -26 -9 21.6580 77.3850 3

0 -27 0 254.926 123.492 1

0 27 0 125.666 92.0145 7

0 -27 -1 115.399 92.5602 1

0 -27 1 49.8188 75.1183 1

0 27 -1 142.491 89.1460 7

0 27 1 238.289 105.763 7

0 -27 2 162.037 88.8062 1

0 -27 -2 208.060 119.277 1

0 27 -2 162.337 70.5897 7

0 -27 3-11.9460 67.0299 1

0 -27 -3-12.4616 102.857 1

0 27 -3 50.3096 63.0579 7

0 -27 -4-12.7740 60.3675 1

0 27 -4-38.5500 56.1435 7

0 -27 -5-118.354 112.496 1

0 -27 -5 79.5897 66.7100 3

0 27 -5-32.4996 72.1126 10

0 -27 -6 130.766 74.3818 1

0 -27 -6-139.475 79.0979 3

0 27 -6-156.409 101.064 10

0 -27 -7 30.8944 60.9632 3

0 -27 -8 42.1165 45.0948 3

0 -28 0 277.700 118.072 1

0 28 0 193.883 104.770 7

0 -28 -1 485.946 172.890 1

0 -28 1 240.850 95.8050 1

0 28 1 445.448 151.825 7

0 28 -1 224.712 108.686 7

0 -28 -2 49.8647 94.3357 1

0 -28 2-5.66674 56.7675 1

0 28 -2 25.5108 41.9453 7

0 -28 -3 154.724 101.563 1

0 28 -3 110.072 85.6625 7

0 -28 -4-42.8806 79.7433 1

0 28 -4-87.2797 101.519 10

0 -28 -5 25.7759 91.4472 1

0 -28 -6 105.487 83.4315 3

0 -28 -7 160.980 94.1678 3

0 -29 0 31.6111 69.8212 1

0 29 0 73.6961 74.2353 7

0 -29 -1 46.4801 122.865 1

0 -29 1 59.5591 53.1791 1

0 29 -1-135.588 91.7949 7

0 29 1 113.717 93.0816 7

0 -29 2 71.8618 75.2669 1

0 -29 -2 62.4083 94.2169 1

0 29 -2-79.5471 66.0000 7

0 29 2-39.2860 79.1326 7

0 -29 -3 104.584 96.9710 1

0 29 -3 77.7704 79.9200 10

0 29 -3-30.9600 73.5934 7

0 -29 -4-18.7969 96.8624 1

0 -30 0 593.839 193.227 1

0 30 0 226.309 113.453 7

0 -30 -1 23.5617 83.0417 1

0 -30 1 46.3185 63.5755 1

0 30 -1-0.20597 50.7452 7

0 30 1-0.22083 78.6789 7

0 -30 -2-0.28884 65.6865 1

0 30 -2 104.699 91.8260 7

0 30 2 14.5440 48.5941 7

0 -30 -3-63.7539 78.9869 1

0 30 -3 143.471 90.7734 7

0 -30 -4-193.359 153.060 1

0 -31 0-0.27239 66.4211 1

0 31 0 144.432 104.741 7

0 -31 -1 80.6346 78.6682 1

0 31 -1-54.5416 62.7629 7

0 31 1 92.6278 76.2250 7

0 -31 -2-138.438 102.635 1

0 31 -2 31.4280 66.3044 7

0 -31 -3 139.074 110.541 1

0 31 -3-11.2195 49.3891 7

0 -32 0 70.2277 92.9173 1

0 32 0 104.856 106.661 7

0 -32 -1-235.811 134.561 1

0 32 -1 12.9028 70.3289 7

0 -32 -2-49.4480 107.531 1

0 32 -2 31.6082 78.8208 7

1 0 -16 133.423 114.894 10

1 0 -15 37.4371 58.7245 10

1 0 -15 18.3380 51.5242 6

1 0 -14 33.4854 46.5017 10

1 0 -14 172.834 109.788 6

1 0 -13 8.30668 41.2312 10

1 0 -13-13.4893 57.7596 6

-1 0 12 2475.66 273.092 1

1 0 -12 3131.73 339.650 10

1 0 -12 3353.28 397.037 6

-1 0 11-32.8820 39.3803 1

1 0 -11-56.2642 58.5697 10

1 0 -11 48.7969 53.6213 6

1 0 -10 8197.76 491.780 10

-1 0 10 9149.25 519.687 10

1 0 -10 9056.16 584.712 6

-1 0 9 33.3930 52.1134 10

1 0 -9-89.3728 68.4546 10

1 0 -9 12.7288 33.4249 6

1 0 -8 7589.64 417.741 10

-1 0 8 7356.91 413.751 10

-1 0 8 6980.41 367.830 1

-1 0 7 2.84521 26.0135 4

-1 0 7 1.33341 23.9701 1

-1 0 7 20.8559 32.7948 10

1 0 -7 58.5318 40.3883 10

-1 0 6 36601.6 776.261 4

-1 0 6 34157.9 718.068 1

-1 0 6 36012.9 799.650 10

1 0 -6 38098.0 804.732 10

-1 0 6 35592.2 466.822 8

-1 0 5 11.2740 21.9080 4

-1 0 5 5.76913 18.7754 1

1 0 -5-31.4430 24.9238 10

-1 0 5-3.64955 19.8216 10

-1 0 5-15.7763 13.8386 8

-1 0 4 39.9005 30.0784 4

-1 0 4 20.6663 19.6597 10

1 0 -4 30.4712 16.0314 10

-1 0 4 35.8690 24.3637 1

-1 0 3 2.81959 12.4880 4

1 0 -3 6.68192 15.8468 10

-1 0 3-17.0040 22.7604 10

-1 0 3 10.3915 13.4298 1

-1 0 -2 902580. 2012.81 1

-1 0 -2 900615. 2136.90 2

-1 0 -3-13.1239 15.2639 4

-1 0 -3-6.11612 19.9452 10

1 0 3-28.9790 25.7540 10

-1 0 -3 3.30977 11.1693 2

-1 0 -3-7.39974 18.0327 1

1 0 3 34.3517 20.8689 1

-1 0 -4 9838.91 304.161 4

1 0 4 9589.33 291.187 1

-1 0 -4 9361.11 278.741 2

-1 0 -4 9949.08 310.189 1

-1 0 -4 9851.97 313.181 10

-1 0 -5 4.46713 21.6756 4

1 0 5-3.48838 17.5582 1

-1 0 -5-18.3935 25.5965 2

-1 0 -5 31.1040 33.5820 1

1 0 5 15.9306 17.7801 10

-1 0 -5-5.28328 18.7020 10

-1 0 -5 11.0241 15.6992 5

-1 0 -6 929.890 120.013 1

1 0 6 634.359 92.1422 1

-1 0 -6 706.393 88.4812 2

-1 0 -6 895.520 121.771 10

1 0 6 792.987 110.473 10

-1 0 -6 860.579 93.5116 5

-1 0 -7-19.8009 23.5285 5

1 0 7-69.5190 48.3677 1

-1 0 -7 22.1974 38.1332 1

-1 0 -7 22.7908 25.9276 2

1 0 7-31.2133 31.7583 10

-1 0 -7 1.09892 21.6156 10

1 0 8 2652.16 215.869 1

-1 0 -8 2372.47 179.973 2

-1 0 -8 2375.48 224.040 1

-1 0 -8 2746.75 246.876 10

1 0 8 2795.59 244.788 10

-1 0 -8 2741.92 295.712 6

-1 0 -8 2449.37 169.057 5

-1 0 -9-1.30591 19.3952 5

-1 0 -9-42.8012 39.2919 2

-1 0 -9 6.00504 38.3466 10

1 0 9 14.5000 34.1386 10

-1 0 -9 66.4897 49.6332 6

-1 0 -10 1883.48 143.082 5

-1 0 -10 1715.36 165.610 2

-1 0 -10 2144.35 248.350 10

1 0 10 1942.73 228.494 10

-1 0 -10 1892.18 283.096 6

-1 0 -11 5.87711 16.2100 5

-1 0 -11-15.1272 28.9139 2

1 0 11 6.48170 30.6742 1

1 0 11-28.5576 44.7258 10

-1 0 -11 2.69763 43.1734 10

-1 0 -11-70.8178 46.7871 6

1 0 12 451.045 115.726 1

-1 0 -12 261.930 96.7692 10

-1 0 -12 211.680 90.9490 6

-1 0 -13-37.8616 63.2275 10

-1 0 -13 101.673 93.6703 6

-1 0 -14 14.3993 61.7893 10

-1 0 -14 192.167 137.043 6

1 1 -16-126.204 97.0648 10

1 -1 -16 7.91878 49.6962 10

1 -1 -15-55.5795 97.9718 10

1 1 -15 26.6637 44.1391 10

1 -1 -15-69.8960 95.4611 6

1 1 -15-49.8486 49.2492 6

1 -1 -14-99.0952 94.1102 10

1 1 -14 68.6374 59.4646 10

1 -1 -14 82.7730 83.2294 6

1 1 -14 98.1783 115.893 6

1 -1 -13 6.69490 48.8531 10

1 1 -13-98.0920 54.9956 10

1 -1 -13-34.2702 93.9045 6

1 1 -13-0.47901 45.0423 6

1 1 -12 6.45588 51.0850 2

-1 -1 12-100.828 64.8151 1

-1 1 12-91.7044 88.4784 1

1 -1 -12 36.5486 45.3642 10

1 1 -12 34.3811 47.6106 10

1 -1 -12 13.3122 70.3506 6

1 1 -12-37.0804 64.2135 6

-1 -1 11 22.7188 46.9279 1

-1 1 11 38.4942 45.0899 1

1 -1 -11 13.2232 31.0344 2

1 1 -11-58.0220 75.7050 10

1 -1 -11-53.0369 38.6328 10

1 -1 -11-72.3568 77.1939 6

1 1 -11-8.67040 59.4645 6

-1 1 10 1942.98 222.683 1

-1 1 10 2531.64 275.138 10

-1 -1 10 2300.29 263.839 10

1 -1 -10 2228.72 258.056 10

1 1 -10 2140.63 256.933 10

1 -1 -10 1923.95 271.889 6

1 1 -10 2086.07 283.941 6

-1 -1 9 131.147 60.7738 1

-1 -1 9 21.7687 39.5392 10

1 1 -9 80.9718 57.9581 10

1 -1 -9 46.4861 32.4737 10

-1 1 9 100.528 58.3197 10

1 1 -9 88.4967 65.5962 6

1 -1 -9 88.8441 71.8575 6

-1 -1 8 2718.63 234.854 4

-1 1 8 3217.85 276.341 10

-1 -1 8 3049.59 265.446 10

1 -1 -8 3234.50 268.516 10

1 1 -8 3029.45 266.801 10

-1 1 8 2559.12 235.149 1

-1 -1 8 2653.60 219.574 1

-1 1 7 2269.88 214.344 4

-1 -1 7 2465.41 209.746 4

-1 -1 7 2130.56 198.744 1

-1 1 7 2288.51 212.957 1

-1 -1 7 2472.59 219.680 10

-1 1 7 2637.59 232.521 10

1 -1 -7 2495.81 219.704 10

1 1 -7 2696.96 233.905 10

-1 -1 6 5015.27 284.208 4

-1 1 6 5050.66 297.271 4

-1 -1 6 4492.80 256.571 1

-1 1 6 4715.38 273.565 1

1 -1 -6 5244.07 296.365 10

-1 1 6 5178.48 303.375 10

1 1 -6 5464.73 307.565 10

-1 -1 6 4802.07 150.906 8

-1 -1 5 2607.28 188.346 4

-1 1 5 2557.63 192.903 4

-1 -1 5 2583.53 177.387 1

-1 1 5 2674.93 194.343 1

1 1 -5 2678.16 197.182 10

1 -1 -5 2857.89 201.029 10

-1 1 5 2845.99 203.311 10

-1 -1 5 2688.34 196.124 10

-1 1 5 2534.69 136.327 8

-1 -1 4-7.84297 16.5805 4

-1 1 4-2.27071 19.2985 4

-1 -1 4 0.81610 17.3349 1

-1 1 4 2.20125 20.2942 1

-1 -1 4 11.4682 14.1990 10

-1 1 4 16.5427 19.6812 10

1 1 -4 1.93515 20.5141 10

-1 1 4-3.16387 11.7898 8

1 -1 -3 780.631 88.0898 4

1 -1 -3 1609.70 124.896 10

1 1 -3 1921.03 134.069 10

-1 -1 3 756.916 123.084 10

-1 1 3 1737.97 129.284 10

-1 1 3 1242.71 122.482 6

-1 1 -2 17142.7 240.555 4

-1 -1 -2 19059.7 355.498 1

1 1 2 17396.9 251.327 2

1 -1 2 16660.4 233.978 1

-1 1 -2 17833.6 244.471 1

1 1 2 15998.8 306.707 1

-1 -1 -2 16885.9 237.574 2

1 -1 2 16896.5 282.672 10

1 1 2 15664.4 268.785 10

-1 1 -2 16505.6 289.393 10

-1 -1 -2 16231.1 268.640 10

1 1 2 16277.5 377.386 6

-1 1 -2 17225.0 309.192 5

-1 1 -3 298.934 64.4341 4

-1 -1 -3 492.382 91.2738 4

1 1 3 150.581 80.8376 1

-1 -1 -3 209.345 44.0727 2

-1 -1 -3 323.270 80.9702 1

1 -1 3 101.922 50.8094 1

-1 1 -3 168.576 55.4126 1

-1 -1 -3 364.327 57.7080 10

-1 1 -3 289.049 58.3551 10

1 1 3 124.875 52.0060 10

1 -1 3 115.528 54.7926 10

-1 1 -3 355.654 61.1500 5

-1 -1 -4 269.050 52.3800 4

-1 -1 -4 207.663 37.2576 2

1 1 4 182.200 45.5115 1

-1 -1 -4 233.475 51.6874 1

1 -1 4 185.614 45.1983 1

-1 1 -4 237.496 45.0947 1

1 1 4 240.269 51.1481 10

-1 1 -4 283.364 56.0308 10

-1 -1 -4 223.515 45.9301 10

1 -1 4 185.968 42.6246 10

-1 -1 -4 172.332 28.9116 8

-1 1 -4 197.399 40.5250 5

-1 -1 -5 1860.26 158.210 4

-1 1 -5 1881.58 147.696 1

-1 -1 -5 1923.74 174.115 1

1 -1 5 1702.08 133.478 1

1 1 5 1576.20 144.546 1

-1 -1 -5 1600.71 112.456 2

1 1 5 1861.33 157.012 10

-1 1 -5 1869.28 157.001 10

1 -1 5 2040.87 160.761 10

-1 -1 -5 1902.53 155.929 10

-1 1 -5 1732.13 127.987 5

-1 -1 -5 1763.07 99.7908 8

-1 -1 -6 13142.4 465.971 4

-1 1 -6 12984.1 417.235 1

-1 -1 -6 12294.8 337.838 2

1 -1 6 12525.2 389.318 1

1 1 6 12497.0 437.422 1

-1 -1 -6 13260.1 467.480 1

-1 1 -6 14171.2 475.622 10

1 -1 6 14377.7 471.821 10

-1 -1 -6 13594.4 455.576 10

1 1 6 13423.4 457.825 10

-1 1 -6 13860.9 368.601 5

-1 -1 -7 1450.48 167.291 4

1 1 7 1115.09 139.374 1

-1 1 -7 1224.37 143.410 1

-1 -1 -7 1142.02 112.123 2

-1 1 -7 1140.62 131.533 2

1 -1 7 1192.54 133.820 1

-1 -1 -7 1229.85 158.707 1

-1 -1 -7 1290.39 154.864 10

1 1 7 1325.17 154.099 10

-1 1 -7 1419.07 167.426 10

1 -1 7 1414.91 161.668 10

-1 1 -7 1296.49 120.332 5

-1 -1 -7 1328.33 118.168 5

-1 -1 -8 458.996 70.5969 5

-1 1 -8 373.936 65.5130 5

1 -1 8 422.110 88.2423 1

-1 -1 -8 459.583 105.175 1

-1 -1 -8 395.171 68.4476 2

-1 1 -8 398.726 81.9357 2

1 1 8 397.654 88.6266 1

1 -1 8 521.831 108.154 10

1 1 8 422.192 95.6927 10

-1 -1 -8 445.378 102.278 10

-1 1 -8 414.173 94.3058 10

-1 1 -8 530.106 131.621 6

-1 -1 -8 515.770 134.860 6

-1 -1 -9 528.588 76.8366 5

-1 1 -9 538.382 82.4092 5

1 -1 9 547.286 96.7415 1

-1 -1 -9 535.968 85.7119 2

-1 1 -9 551.261 95.5287 2

-1 -1 -9 629.463 126.733 10

1 1 9 634.345 125.241 10

-1 1 -9 641.401 134.145 10

1 -1 9 557.071 116.964 10

-1 -1 -9 706.488 165.078 6

-1 1 -9 678.078 166.999 6

-1 1 -10 577.751 83.8236 5

-1 -1 -10 553.164 74.0046 5

-1 -1 -10 573.698 93.0581 2

1 1 10 618.551 130.686 1

-1 1 -10 417.442 90.0261 2

1 1 10 607.444 130.246 10

-1 -1 -10 672.817 130.704 10

1 -1 10 642.278 135.033 10

-1 1 -10 727.305 146.673 10

-1 -1 -10 514.252 155.643 6

-1 1 -10 581.645 168.474 6

-1 1 -11 49.3066 31.4421 5

-1 -1 -11 15.0155 21.1442 5

1 1 11 24.9587 46.6335 1

-1 1 -11 35.1883 39.5775 2

1 -1 11-19.6993 40.9355 10

-1 1 -11 39.2398 57.0089 10

-1 -1 -11 21.3804 45.0855 10

1 1 11-12.8100 51.9469 10

-1 1 -11-72.5265 72.5657 6

-1 -1 -11 170.534 102.670 6

1 1 12-38.5657 34.7702 1

1 -1 12-54.7115 67.2678 1

-1 1 -12 37.1465 62.3037 10

-1 -1 -12-80.3395 65.6054 10

-1 1 -12-94.0389 78.9026 6

-1 -1 -12-22.8251 79.4919 6

-1 1 -13-47.9765 62.9889 10

-1 -1 -13 47.5448 34.6180 10

-1 1 -13-19.3061 50.2722 6

-1 -1 -13 23.4027 49.2541 6

-1 -1 -14 671.129 171.740 10

-1 1 -14 680.738 171.929 10

-1 1 -14 756.945 222.713 6

-1 -1 -14 791.861 226.820 6

-1 1 -15 66.8443 81.3182 10

1 -2 -16 185.473 118.991 10

1 2 -16-85.8436 100.032 10

1 -2 -15-36.6256 64.2702 10

1 2 -15-87.3519 94.2830 10

1 -2 -15 127.283 110.599 6

1 2 -15 13.3119 68.3367 6

1 -2 -14 199.079 104.567 10

1 2 -14 143.458 90.2051 10

1 -2 -14 263.514 139.197 6

1 2 -14 176.216 119.074 6

1 -2 -13 36.5663 38.8209 10

1 2 -13 167.446 98.2720 10

1 2 -13 67.6348 68.3495 6

1 -2 -13 74.4061 82.9306 6

-1 2 12 1732.56 237.219 1

-1 -2 12 1749.38 208.527 1

1 -2 -12 2016.66 265.590 10

1 2 -12 1948.31 269.655 10

1 -2 -12 1910.92 298.333 6

1 2 -12 1996.89 308.810 6

1 -2 -11-16.1100 27.0421 2

-1 2 11-41.5875 55.5559 1

1 2 -11-5.82814 41.2641 10

1 -2 -11 42.5945 56.8307 10

1 2 -11 23.2637 59.0875 6

1 -2 -11 72.2890 65.3092 6

-1 2 10 7445.82 449.217 1

1 -2 -10 7520.82 290.948 2

1 -2 -10 8393.36 497.330 10

-1 2 10 8835.95 518.790 10

1 2 -10 8581.56 515.279 10

-1 -2 10 9165.79 518.095 10

1 2 -10 8105.30 562.369 6

1 -2 -10 8632.05 568.057 6

-1 -2 9 270.266 74.0978 1

1 -2 -9 247.918 84.5612 10

-1 -2 9 433.156 110.907 10

-1 2 9 360.508 100.060 10

1 2 -9 348.643 107.579 10

1 -2 -9 243.802 82.7193 6

1 2 -9 285.174 109.890 6

-1 -2 8 17891.4 602.469 4

1 2 -8 18031.7 653.918 10

1 -2 -8 18599.9 646.076 10

-1 -2 8 17492.2 639.787 10

-1 2 8 18435.0 670.459 10

-1 -2 8 16140.9 524.947 1

-1 -2 7 121.410 47.8465 4

1 -2 -7-6.58813 31.2556 10

1 2 -7 81.3367 46.6005 10

-1 2 7 49.6449 36.2184 10

-1 -2 7 72.8812 49.4610 10

-1 2 7 64.7468 32.4008 1

-1 -2 7 68.0587 36.7997 1

-1 2 6 248.956 70.5119 4

-1 -2 6 311.722 72.7968 4

-1 -2 6 251.538 57.8764 1

-1 2 6 261.874 69.0697 1

-1 2 6 314.141 74.0077 10

1 -2 -6 321.873 83.8423 10

-1 -2 6 327.397 68.1141 10

1 2 -6 300.805 73.4495 10

1 -2 -5 330.865 81.6941 4

-1 2 5 293.933 75.6359 4

-1 -2 5 351.619 70.1295 4

-1 2 5 236.774 70.1643 1

-1 -2 5 302.885 57.3897 1

1 -2 -5 276.466 70.7686 1

1 2 -5 381.813 82.4667 10

1 -2 -5 346.910 73.5404 10

-1 -2 5 286.747 67.3948 10

-1 2 5 251.690 61.4734 10

-1 2 5 302.399 57.1912 8

-1 2 5 335.325 78.6326 6

-1 -2 5 269.952 40.5084 5

1 -2 -4 10924.3 376.580 4

-1 2 4 11872.0 387.618 4

-1 2 4 10892.3 374.434 1

1 -2 -4 11189.2 383.026 1

-1 -2 4 11871.3 369.557 10

-1 2 4 11428.5 369.295 10

1 2 -4 11754.3 376.783 10

1 -2 -4 11911.2 366.062 10

-1 -2 4 11518.6 244.056 5

-1 2 4 11238.2 299.551 8

-1 2 4 11718.2 407.640 6

1 -2 -3 10806.6 334.390 4

-1 2 3 11000.1 329.367 4

-1 -2 3 10827.4 228.917 5

1 -2 -3 11205.8 344.468 1

-1 2 3 10498.6 334.412 1

-1 2 3 11741.9 329.736 10

-1 -2 3 11774.7 324.418 10

1 -2 -3 11369.1 313.461 10

1 2 -3 11247.2 325.115 10

-1 2 3 11298.7 345.901 6

-1 2 3 11130.2 286.311 8

1 -2 -2 73.8595 15.5341 5

1 -2 -2 61.2221 22.2286 4

1 -2 -2 24.0456 25.7222 1

-1 2 2 88.1721 24.7642 10

-1 -2 2 79.4651 23.2367 10

-1 2 2 92.7403 23.2124 8

-1 2 2 80.4396 24.2917 6

1 2 2 4031.02 170.589 1

-1 -2 -2 4122.70 137.789 10

1 2 2 4108.70 141.272 10

1 -2 2 4291.95 149.993 10

-1 2 -2 4128.16 151.053 10

1 -2 2 4183.26 168.738 5

-1 2 -2 4369.77 170.399 5

1 2 2 4127.22 193.230 6

1 -2 3 61272.8 539.986 4

1 2 3 57826.4 741.053 1

-1 -2 -3 57616.0 748.154 1

-1 2 -3 59240.7 528.115 1

1 -2 3 62204.0 684.144 10

1 2 3 63751.5 694.186 10

-1 2 -3 61373.8 706.251 10

-1 -2 -3 63211.3 655.626 10

1 2 3 65045.6 914.784 6

-1 2 -3 59110.7 681.147 5

1 -2 4 27179.5 442.398 4

-1 -2 -4 27173.0 568.593 4

1 2 4 25333.8 541.120 1

-1 2 -4 27279.5 438.591 1

-1 -2 -4 26822.8 349.524 2

-1 -2 -4 27304.6 581.044 1

1 -2 4 25978.8 396.874 1

1 -2 4 28242.8 530.984 10

1 2 4 27267.9 527.394 10

-1 -2 -4 28451.6 522.233 10

-1 2 -4 28286.8 557.971 10

1 2 4 28782.0 688.571 6

-1 2 -4 28525.0 498.266 5

-1 -2 -5 659.752 105.228 4

-1 2 -5 489.193 76.3689 1

-1 -2 -5 433.492 56.5430 2

1 -2 5 388.734 60.7945 1

1 2 5 450.186 84.8816 1

-1 -2 -5 419.836 122.039 1

-1 -2 -5 487.610 80.9396 10

-1 2 -5 482.379 87.9003 10

1 2 5 401.937 75.1556 10

1 -2 5 420.090 76.9595 10

-1 2 -5 511.045 81.1101 5

-1 -2 -5 403.914 59.7680 8

-1 -2 -6 6583.29 344.228 4

1 -2 6 5789.49 248.455 1

-1 -2 -6 6038.55 213.579 2

1 2 6 6143.82 321.744 1

-1 -2 -6 7270.87 360.565 1

1 -2 6 6702.45 323.366 10

-1 -2 -6 6584.08 319.236 10

1 2 6 6452.87 323.142 10

-1 2 -6 6972.70 338.831 10

-1 -2 -6 6392.19 231.029 8

-1 2 -6 6302.93 273.117 5

-1 -2 -7 764.027 125.719 4

-1 2 -7 652.624 90.6835 5

1 -2 7 613.506 89.9215 1

-1 -2 -7 709.813 79.5832 2

-1 -2 -7 746.235 128.379 1

1 2 7 678.498 117.014 1

-1 2 -7 799.254 128.602 10

1 -2 7 792.048 119.912 10

1 2 7 739.428 117.489 10

-1 -2 -7 764.759 117.114 10

-1 2 -8 2402.19 174.663 5

-1 -2 -8 2196.81 228.259 4

-1 -2 -8 2558.27 250.455 1

1 -2 8 2168.05 181.078 1

-1 -2 -8 2234.07 146.739 2

-1 2 -8 2067.93 199.222 2

1 2 8 2287.95 221.884 10

1 -2 8 2399.73 223.983 10

-1 2 -8 2277.53 226.699 10

-1 -2 -8 2349.91 222.456 10

-1 -2 -8 2563.66 284.265 6

-1 2 -8 2601.62 289.680 6

-1 -2 -8 2199.35 158.013 5

-1 2 -9 619.525 89.9778 5

-1 -2 -9 757.707 88.0037 2

-1 2 -9 637.407 113.087 2

-1 -2 -9 804.159 149.817 1

1 -2 9 692.170 110.647 1

-1 2 -9 912.077 156.625 10

1 2 9 772.193 140.381 10

1 -2 9 734.509 128.177 10

-1 -2 -9 719.882 127.761 10

-1 -2 -9 778.164 165.951 6

-1 -2 -9 701.790 90.2560 5

-1 2 -9 763.119 177.152 6

-1 2 -10 49.4285 22.1701 5

-1 -2 -10 42.1046 19.2290 5

1 2 10 39.6203 39.9839 1

-1 2 -10 68.5169 54.7991 2

1 -2 10-5.29989 44.0552 1

-1 2 -10 156.740 67.4101 10

-1 -2 -10 94.3363 69.9322 10

1 2 10 41.6603 45.3535 10

1 -2 10 160.931 80.7860 10

-1 2 -10 181.312 96.0751 6

-1 -2 -10 148.277 98.4014 6

-1 2 -11 136.851 40.7682 5

-1 -2 -11 141.124 33.0287 5

1 2 11 28.6425 36.6802 1

-1 2 -11 162.388 55.1615 2

-1 -2 -11 199.786 79.6893 10

1 2 11 193.796 82.5251 10

-1 2 -11 210.791 84.7739 10

1 -2 11 147.623 77.1441 10

-1 -2 -11 138.833 83.6325 6

-1 2 -11 137.018 90.0290 6

1 2 12 145.481 85.7783 1

-1 -2 -12 348.241 104.519 10

-1 2 -12 200.080 85.6091 10

-1 2 -12 431.718 162.158 6

-1 -2 -12 241.334 98.0292 6

-1 -2 -13-85.5513 67.7768 10

-1 2 -13 19.2475 46.2377 10

-1 -2 -13 21.9629 42.2698 6

-1 2 -13 49.3182 63.0246 6

-1 -2 -14-28.3248 58.9793 10

-1 2 -14 17.5731 68.9795 10

-1 2 -14 20.1656 77.8647 6

-1 -2 -14 145.560 103.288 6

-1 2 -15 181.248 129.547 10

1 -3 -16-22.0557 65.8513 10

1 3 -16 146.072 122.375 10

1 3 -15 1.84582 40.6318 10

1 -3 -15 42.9099 67.7132 10

1 3 -15-187.746 107.620 6

1 -3 -15-91.2246 113.228 6

1 3 -14 11.7630 43.7412 10

1 -3 -14-56.1487 84.2528 10

1 3 -14 29.7480 70.7358 6

1 -3 -14 171.615 119.189 6

1 3 -13 17.4279 39.9587 10

1 -3 -13-16.1676 42.3146 10

1 -3 -13 7.90869 50.6632 6

1 -3 -12-19.7395 37.1887 10

1 3 -12 32.3595 43.1772 10

1 -3 -12 90.6096 78.0572 6

1 3 -12 13.4424 86.5517 6

-1 3 11 135.130 72.2048 1

1 -3 -11 80.3880 31.1480 2

1 3 -11 39.6084 54.3155 10

1 -3 -11 111.799 52.6115 10

1 -3 -11 132.511 85.1929 6

1 3 -11 182.555 62.0137 6

-1 -3 10 103.061 45.1342 1

-1 3 10 116.196 68.1929 1

1 -3 -10 118.565 30.2903 2

-1 -3 10 192.084 79.5214 10

1 3 -10 176.504 88.0020 10

-1 3 10 243.383 101.015 10

1 -3 -10 136.544 72.2858 10

1 -3 -10 170.775 82.1097 6

1 3 -10 213.064 96.6719 6

-1 3 9 486.217 108.679 1

-1 -3 9 535.969 98.7579 1

1 -3 -9 439.155 60.2685 2

1 3 -9 484.615 111.287 10

-1 -3 9 493.813 116.207 10

-1 3 9 428.645 102.550 10

1 -3 -9 519.244 111.004 10

1 -3 -9 391.904 116.713 6

1 3 -9 449.677 122.506 6

-1 -3 8 2349.13 219.316 4

-1 3 8 2785.57 261.250 10

1 -3 -8 2595.48 242.556 10

-1 -3 8 2652.89 249.324 10

1 3 -8 2768.71 257.807 10

-1 -3 8 2312.42 190.308 1

-1 -3 7 13784.7 496.246 4

1 -3 -7 13153.3 543.844 4

1 -3 -7 13460.2 548.205 1

-1 -3 7 12155.7 414.988 1

-1 3 7 12800.8 529.007 1

-1 3 7 13506.8 532.099 10

1 3 -7 13071.1 531.155 10

-1 -3 7 13803.3 526.363 10

1 -3 -7 14087.6 515.956 10

1 -3 -6 1779.61 186.505 4

-1 -3 6 1867.24 170.029 4

-1 3 6 1681.42 182.829 1

-1 -3 6 1654.83 144.355 1

1 -3 -6 1723.24 184.970 1

1 3 -6 1900.51 187.518 10

-1 -3 6 1821.48 175.667 10

1 -3 -6 1830.16 172.508 10

-1 3 6 1896.36 184.453 10

-1 -3 6 1623.52 116.684 5

-1 3 6 1874.32 207.603 6

1 -3 -5 1606.81 162.850 4

-1 -3 5 1686.44 130.716 1

-1 3 5 1647.14 166.067 1

1 -3 -5 1679.13 169.065 1

1 3 -5 1757.59 166.454 10

-1 -3 5 1808.35 163.667 10

-1 3 5 1765.81 163.804 10

1 -3 -5 1760.57 157.684 10

-1 3 5 1620.29 131.794 8

-1 -3 5 1812.20 117.009 5

-1 3 5 1760.17 182.549 6

1 -3 -4 3543.39 224.846 4

-1 -3 4 3323.50 156.625 5

1 -3 -4 3831.72 243.211 1

-1 3 4 3142.94 215.377 1

-1 -3 4 3666.86 206.754 10

-1 3 4 3587.17 207.716 10

1 -3 -4 3687.52 203.175 10

1 3 -4 3583.75 212.728 10

-1 3 4 3355.29 182.320 8

-1 3 4 3435.15 224.825 6

1 -3 -3 1762.31 145.841 4

1 -3 -3 2382.63 179.630 1

-1 3 3 1833.68 155.357 10

-1 -3 3 3544.51 185.900 10

1 -3 -3 2572.73 161.304 10

-1 3 3 1249.26 174.329 6

1 -3 -2 25.7048 16.6516 5

1 -3 -2 19.1175 19.2610 4

1 -3 -2 53.4166 34.1958 1

-1 3 2 43.0151 29.4879 10

1 -3 -2 43.6115 14.3226 9

-1 -3 2 15.2231 16.2721 10

-1 3 2 58.9448 28.9204 8

1 -3 -2 11.0842 19.0015 8

-1 3 2 18.0544 16.5911 6

-1 -3 -1 25174.5 406.694 1

-1 -3 -1 24411.7 298.688 3

-1 3 -1 24505.5 426.038 5

1 3 1 25409.5 306.419 3

1 3 1 24485.4 402.151 6

-1 3 -1 24128.2 352.766 8

1 -3 1 24926.1 437.194 5

-1 3 -2 58677.8 706.704 5

1 3 2 60355.7 731.913 1

-1 -3 -2 60742.5 710.072 1

1 3 2 57892.3 569.391 10

-1 3 -2 59551.2 613.881 10

-1 3 -2 57727.2 491.521 8

1 -3 2 56296.7 701.971 5

1 3 2 59631.7 771.764 6

-1 3 -3 6632.19 265.745 5

1 3 3 8474.29 367.319 1

-1 3 -3 5554.68 222.759 10

-1 -3 -3 4364.56 184.042 10

1 -3 3 5626.64 222.245 10

1 3 3 4256.53 187.734 10

1 3 3 3858.90 238.434 6

1 -3 3 4196.41 205.500 5

-1 3 -4 16452.9 409.898 5

1 -3 4 16270.4 327.114 4

-1 -3 -4 15402.6 455.996 1

1 3 4 16681.7 480.847 1

1 3 4 15668.9 409.939 10

-1 3 -4 16023.4 427.379 10

-1 -3 -4 17032.0 403.550 10

1 -3 4 16644.1 414.144 10

1 3 4 16582.4 536.703 6

1 -3 4 16508.1 408.134 5

1 -3 5 17.9167 21.7911 4

-1 -3 -5 98.1957 60.9551 4

-1 3 -5 40.2501 41.5380 5

1 -3 5 9.13927 22.0672 1

1 3 5 75.3673 47.2840 1

-1 -3 -5 54.5992 50.1125 1

1 -3 5 4.00911 19.7440 10

1 3 5-3.53641 20.4361 10

-1 -3 -5 10.0124 25.4628 10

-1 3 -5 21.6548 36.2131 10

-1 -3 -5 24.2533 40.4989 8

1 3 5 30.8507 39.0715 6

-1 -3 -6 15231.1 535.354 4

1 -3 6 14894.5 412.136 4

-1 3 -6 14868.0 426.098 5

-1 -3 -6 16602.9 573.861 1

1 -3 6 12994.1 349.611 1

1 3 6 15007.1 515.588 1

1 3 6 14975.3 494.772 10

-1 3 -6 15824.7 524.141 10

-1 -3 -6 15316.8 474.740 10

1 -3 6 16073.0 499.675 10

-1 -3 -6 14276.0 384.747 8

-1 3 -7 5825.74 275.336 5

-1 -3 -7 6341.82 368.264 4

1 -3 7 5793.16 251.379 1

-1 -3 -7 5626.35 356.034 1

-1 -3 -7 6128.30 326.627 10

1 3 7 6344.72 350.121 10

-1 3 -7 6587.21 367.739 10

1 -3 7 6585.37 347.993 10

-1 -3 -8 3075.54 279.347 4

-1 3 -8 2897.60 197.583 5

-1 -3 -8 2683.69 263.101 1

1 -3 8 2739.19 186.634 1

1 -3 8 3282.67 262.168 10

-1 3 -8 3174.24 267.554 10

-1 -3 -8 3068.05 251.482 10

1 3 8 2908.31 254.183 10

-1 3 -8 3266.75 326.490 6

-1 -3 -8 3233.47 321.406 6

-1 3 -9 522.901 92.5700 5

-1 -3 -9 537.990 124.711 4

1 3 9 548.762 120.911 1

1 -3 9 561.827 92.7068 1

-1 3 -9 444.212 97.5540 2

-1 -3 -9 552.133 134.574 1

1 3 9 666.384 133.133 10

-1 3 -9 649.154 134.802 10

-1 -3 -9 620.734 127.347 10

1 -3 9 567.653 117.722 10

-1 -3 -9 583.958 84.7472 5

-1 3 -9 674.595 161.138 6

-1 -3 -9 566.319 143.094 6

-1 -3 -10 291.930 57.6288 5

-1 3 -10 233.358 51.7870 5

-1 3 -10 211.982 73.1938 2

1 -3 10 266.575 66.5777 1

-1 -3 -10 233.283 117.774 1

1 3 10 254.457 86.2983 1

1 -3 10 295.994 95.7556 10

-1 -3 -10 275.073 84.8035 10

1 3 10 254.290 83.5120 10

-1 3 -10 341.574 112.330 10

-1 -3 -10 422.344 132.399 6

-1 3 -10 219.271 105.868 6

-1 -3 -11 185.609 37.1002 5

-1 3 -11 180.085 54.0905 5

-1 3 -11 91.1636 47.8561 2

1 3 11 28.2534 39.4425 1

1 -3 11 261.451 78.1599 1

1 3 11 207.932 78.5584 10

-1 3 -11 252.029 94.6032 10

-1 -3 -11 240.436 90.9121 10

-1 -3 -11 200.483 101.131 6

-1 3 -11 283.963 128.565 6

-1 -3 -12-15.6944 54.9265 10

-1 3 -12 72.9245 69.2557 10

-1 3 -12 54.4603 85.3876 6

-1 -3 -12-55.3151 77.3446 6

-1 -3 -13-27.7213 69.9290 10

-1 3 -13-27.5133 61.0672 10

-1 3 -13 11.0429 58.0684 6

-1 -3 -13 112.822 96.5110 6

-1 -3 -14 73.5765 57.2143 10

-1 3 -14 110.487 90.9457 10

-1 -3 -14 79.2485 69.5090 6

-1 3 -14 173.231 113.329 6

1 4 -16-185.279 109.393 10

1 -4 -15 126.159 70.3514 10

1 4 -15 132.577 110.079 10

1 -4 -15 29.3342 88.3016 6

1 4 -15 230.326 109.298 6

1 -4 -14-1.33791 57.4123 10

1 4 -14 157.716 99.0141 10

1 -4 -14 240.067 125.062 6

1 4 -14 77.0686 75.1661 6

1 -4 -13 64.0881 36.7091 10

1 4 -13 26.0414 56.6207 10

1 4 -13-32.8404 54.7725 6

1 -4 -13-74.2123 84.6431 6

1 4 -12 805.715 178.405 10

1 -4 -12 824.519 171.919 10

1 4 -12 803.058 213.899 6

1 -4 -12 655.853 164.192 6

-1 4 11 44.1594 60.3617 1

-1 -4 11 44.7242 52.6906 1

1 4 -11 78.4795 71.2457 10

1 -4 -11 102.242 69.1813 10

1 4 -11-31.4557 60.3171 6

1 -4 -11 96.1541 94.6381 6

-1 -4 10 2786.09 218.046 1

-1 4 10 2965.69 300.668 1

-1 4 10 3236.42 320.326 10

-1 -4 10 3557.12 322.185 10

1 4 -10 3227.15 331.600 10

1 -4 -10 3220.09 302.467 10

1 4 -10 3553.14 374.433 6

1 -4 -10 3148.54 343.522 6

-1 -4 9 152.634 68.0353 4

-1 -4 9 89.6685 49.1493 1

-1 4 9 65.9572 71.4089 1

1 4 -9 203.444 85.7071 10

-1 -4 9 91.6830 58.6342 10

1 -4 -9 122.824 62.8457 10

-1 4 9 89.4263 56.2146 10

1 -4 -9 71.2349 79.4501 6

-1 -4 8 911.493 137.114 4

1 -4 -8 864.229 149.513 4

-1 -4 8 884.609 110.245 1

1 -4 -8 795.592 52.9221 2

1 -4 -8 706.811 137.784 1

1 -4 -8 1071.72 158.036 10

1 4 -8 880.175 149.823 10

-1 -4 8 945.313 145.407 10

-1 4 8 967.684 156.206 10

-1 -4 7 2421.68 210.137 4

1 -4 -7 2657.47 252.609 4

-1 -4 7 2086.36 172.317 1

1 -4 -7 2625.19 248.736 1

-1 -4 7 2579.99 228.730 10

-1 4 7 2559.36 233.487 10

1 -4 -7 2762.26 232.171 10

1 4 -7 2526.09 238.980 10

-1 -4 7 2438.33 157.217 5

-1 4 7 2649.55 270.466 6

1 -4 -6 2042.86 207.357 4

-1 -4 6 2137.89 151.998 1

-1 4 6 1773.36 192.758 1

1 -4 -6 2113.05 208.489 1

-1 -4 6 2237.67 195.308 10

-1 4 6 2310.39 205.740 10

1 -4 -6 2278.55 195.595 10

1 4 -6 2268.17 206.741 10

-1 -4 6 2276.58 154.755 5

-1 4 6 2244.83 229.189 6

1 -4 -5 3996.13 267.545 4

1 -4 -5 3311.69 251.311 1

-1 4 5 3212.09 237.920 1

1 -4 -5 3846.92 233.793 10

1 4 -5 3635.04 244.242 10

-1 -4 5 3668.06 230.626 10

-1 4 5 3613.54 238.457 10

-1 -4 5 3543.20 192.817 5

-1 4 5 3726.70 270.061 6

-1 -4 4 7469.89 265.486 5

1 -4 -4 9078.59 382.292 4

1 -4 -4 6994.60 335.371 1

-1 4 4 7426.54 334.051 1

1 4 -4 8387.47 332.356 10

-1 4 4 7869.52 318.297 10

-1 -4 4 7980.86 306.971 10

1 -4 -4 8196.18 311.206 10

-1 4 4 6972.24 316.521 8

-1 4 4 8115.44 357.685 6

-1 -4 3 12898.1 341.725 5

1 -4 -3 12174.9 298.262 5

1 -4 -3 13272.6 404.181 4

1 -4 -3 13504.3 423.713 1

-1 4 3 14279.4 381.563 10

-1 -4 3 13457.2 358.103 10

1 -4 -3 13935.6 298.449 9

-1 4 3 12916.1 380.293 8

1 -4 -3 13003.8 374.609 8

-1 4 3 13862.7 406.616 6

1 -4 -2 21.6177 18.1482 4

1 -4 -2 8.44282 22.7905 5

-1 4 2 20.3598 11.9684 2

1 -4 -2 2.13593 28.6106 1

-1 -4 2 42.7395 25.2686 10

-1 4 2 23.9314 10.9847 9

-1 4 2 2.59744 18.6998 8

-1 4 2 27.0671 20.3432 6

1 -4 -2 35.3233 28.3085 8

-1 -4 2 12.1665 11.0704 7

1 -4 -1 25167.4 513.174 5

1 4 -1 21286.0 340.268 1

-1 -4 1 18360.0 308.274 7

-1 4 0 597.080 57.6741 1

1 -4 0 604.396 60.6540 1

-1 -4 0 632.972 62.0175 1

-1 4 0 692.450 72.0909 3

1 -4 0 562.839 70.8603 5

1 4 0 622.785 65.7587 3

1 -4 0 585.035 70.2258 8

-1 -4 -1 24017.2 458.152 1

1 -4 1 20526.2 446.064 5

-1 4 -1 22696.0 452.832 5

1 -4 1 20924.6 387.344 8

-1 4 -1 26680.0 431.032 8

-1 -4 -2 127540. 854.814 3

-1 4 -2 134690. 1145.18 5

-1 -4 -2 133954. 1134.00 1

-1 -4 -2 127982. 565.879 9

-1 -4 -2 125169. 1105.90 6

1 -4 2 125906. 1145.38 5

1 -4 2 127756. 534.261 7

1 4 2 129734. 1162.33 6

1 -4 2 129558. 904.465 8

-1 4 -3 65404.0 834.569 5

-1 -4 -3 61254.1 877.231 1

1 4 3 64546.3 884.936 1

-1 -4 -3 68166.0 484.827 9

1 4 3 68286.8 763.186 10

-1 4 -3 69833.7 817.060 10

1 -4 3 70255.1 885.664 5

1 4 3 71002.2 1009.46 6

1 -4 4 10.4377 11.7310 4

-1 4 -4 22.4087 18.5766 5

1 -4 4 1.16359 8.41331 1

-1 -4 -4 16.8107 26.8888 1

1 4 4 0.64489 24.0839 1

1 4 4 27.2939 23.3402 10

1 -4 4 19.6173 19.1588 10

-1 4 -4 63.4894 33.7932 10

1 -4 4 6.55067 21.1123 5

1 4 4 5.43632 26.4115 6

-1 -4 -5 2549.97 216.403 4

1 -4 5 1474.13 113.980 4

-1 4 -5 1925.55 178.147 5

1 4 5 1954.53 217.959 1

-1 -4 -5 2270.10 205.427 1

1 -4 5 1522.48 91.3757 1

-1 -4 -5 1909.54 159.327 10

1 -4 5 1622.84 148.315 10

1 4 5 1571.70 156.142 10

-1 4 -5 2102.81 182.557 10

-1 -4 -5 1520.79 140.783 8

1 -4 5 1852.13 168.597 5

1 4 5 1817.19 205.624 6

1 -4 6 4010.88 207.075 4

-1 4 -6 3867.02 226.363 5

-1 -4 -6 3958.50 281.805 4

1 4 6 3803.03 268.140 1

-1 -4 -6 3993.63 287.156 1

1 -4 6 3478.28 158.023 1

1 -4 6 4025.69 253.579 10

-1 4 -6 3999.22 270.918 10

1 4 6 3701.34 244.802 10

-1 -4 -6 3974.93 241.669 10

1 4 6 4137.80 328.529 6

-1 -4 -6 3475.67 207.317 8

-1 -4 -7 220.963 99.2244 4

-1 4 -7 150.664 61.2256 5

1 -4 7 136.398 39.6516 1

1 4 7 167.895 59.3926 10

1 -4 7 135.242 56.2942 10

-1 -4 -7 192.390 70.6080 10

-1 4 -7 168.370 69.0814 10

-1 -4 -7 207.665 75.9636 8

-1 4 -8 699.746 101.052 5

-1 -4 -8 594.831 127.052 4

-1 -4 -8 534.599 119.312 1

1 4 8 875.524 155.508 1

1 -4 8 639.856 86.6408 1

1 -4 8 738.139 126.599 10

-1 -4 -8 636.620 117.266 10

-1 4 -8 575.818 116.940 10

1 4 8 657.410 118.759 10

-1 -4 -8 794.490 161.801 6

-1 -4 -9 4661.76 372.127 4

-1 4 -9 5110.28 282.142 5

1 -4 9 4991.16 257.409 1

1 4 9 5195.49 377.102 1

-1 -4 -9 4896.56 383.968 1

1 4 9 5584.04 381.851 10

-1 -4 -9 5648.80 359.714 10

1 -4 9 5705.20 374.136 10

-1 4 -9 5281.78 380.323 10

-1 -4 -9 5207.64 425.990 6

-1 4 -9 6053.67 487.016 6

-1 4 -10 556.151 95.9834 5

-1 -4 -10 523.826 144.380 1

-1 4 -10 550.304 121.765 2

1 4 10 436.049 109.213 1

1 -4 10 582.393 101.613 1

1 -4 10 562.006 119.933 10

-1 -4 -10 683.693 134.635 10

1 4 10 655.786 134.884 10

-1 4 -10 707.677 150.757 10

-1 -4 -10 621.686 163.491 6

-1 4 -10 683.671 177.662 6

-1 -4 -10 586.406 83.1496 5

-1 -4 -11-5.98998 14.3207 5

1 -4 11-24.1855 38.3757 1

1 4 11 54.4673 62.3038 1

-1 4 -11-55.7839 52.4679 2

-1 4 -11-18.5195 36.8007 10

1 4 11 16.6161 68.4365 10

-1 -4 -11 36.4419 40.9007 10

-1 4 -11-2.21306 71.2313 6

-1 -4 -11 54.0192 43.5317 6

-1 4 -12 220.378 77.0742 10

-1 -4 -12 162.145 59.9392 10

-1 -4 -12 412.400 137.373 6

-1 4 -12 336.520 135.841 6

-1 4 -13-115.055 87.3463 10

-1 -4 -13-40.2534 34.1507 10

-1 4 -13-40.5375 61.6168 6

-1 -4 -13-45.0816 81.7270 6

-1 4 -14 47.6898 69.5387 10

-1 4 -14-8.42500 69.8761 6

-1 -4 -14 35.7520 49.6784 6

-1 4 -15 171.505 113.096 10

1 5 -16 169.054 134.114 10

1 5 -15 10.7148 44.0666 10

1 5 -14 66.2734 54.1903 10

1 -5 -14 27.7092 43.2885 6

1 5 -14 74.8160 96.0287 6

1 -5 -13 202.163 97.7054 10

1 5 -13 197.871 109.287 10

1 5 -13 220.375 116.709 6

1 -5 -13-15.3241 48.3719 6

-1 -5 12 1111.31 152.951 1

1 5 -12 1296.46 227.223 10

1 -5 -12 1405.45 223.008 10

1 -5 -12 1648.24 267.546 6

1 5 -12 1655.10 295.225 6

-1 -5 11 38.3589 43.6922 1

1 -5 -11 126.825 77.8311 10

1 5 -11 65.6150 71.4325 10

1 5 -11 66.5577 66.3272 6

1 -5 -11 87.4988 58.8618 6

-1 5 10 1098.22 183.844 1

-1 -5 10 847.608 115.879 1

1 -5 -10 1048.53 172.252 10

1 5 -10 1027.84 187.386 10

-1 5 10 877.511 163.278 10

-1 -5 10 1201.39 188.354 10

1 -5 -10 1105.37 203.335 6

1 -5 -9 339.732 107.981 4

1 -5 -9 347.600 45.3639 5

-1 -5 9 385.210 96.7219 4

-1 5 9 300.901 115.670 1

-1 -5 9 343.284 80.1221 1

1 -5 -9 285.467 90.1429 10

-1 5 9 231.552 75.7721 10

1 5 -9 385.743 107.861 10

-1 -5 9 342.647 98.2905 10

1 -5 -9 399.529 116.382 6

-1 -5 8 1632.33 178.002 4

1 -5 -8 1568.69 213.142 4

1 -5 -8 1760.38 218.009 1

-1 -5 8 1542.35 139.959 1

-1 5 8 1525.58 202.153 1

-1 5 8 1874.40 216.559 10

1 -5 -8 1657.59 194.177 10

-1 -5 8 1813.93 204.246 10

1 5 -8 1793.77 216.789 10

1 -5 -8 1686.23 219.776 6

-1 5 8 1923.77 252.009 6

1 -5 -7 227.464 80.3405 4

-1 -5 7 153.111 43.1891 1

1 -5 -7 133.529 70.7433 1

1 -5 -7 178.374 60.2951 10

-1 5 7 182.421 58.4370 10

1 5 -7 121.413 53.7448 10

-1 -5 7 196.409 67.1069 10

-1 -5 7 189.385 55.8851 5

-1 5 7 206.962 81.2184 6

1 -5 -6 30061.0 822.584 4

1 -5 -6 31367.9 845.471 1

-1 -5 6 29220.9 538.773 1

-1 5 6 29637.0 794.297 1

1 5 -6 31810.6 790.864 10

-1 5 6 30606.8 772.600 10

1 -5 -6 31284.4 730.170 10

-1 -5 6 30847.9 737.264 10

-1 -5 6 31024.3 623.337 5

-1 5 6 33265.9 901.539 6

1 -5 -5 802.665 128.153 4

-1 -5 5 731.310 98.5316 5

-1 5 5 744.750 119.182 1

1 -5 -5 720.030 125.288 1

-1 -5 5 895.027 113.236 10

-1 5 5 828.672 116.917 10

1 5 -5 996.205 130.366 10

-1 5 5 712.058 120.925 6

-1 -5 4 28091.9 572.018 5

1 -5 -4 29001.1 687.194 4

1 -5 -4 28022.6 698.925 1

-1 5 4 29831.6 698.154 1

1 -5 -4 29382.8 482.668 9

1 5 -4 30929.8 654.368 10

-1 5 4 28617.7 621.925 10

-1 -5 4 29882.6 606.056 10

-1 5 4 30334.4 703.762 6

1 -5 -4 29274.5 630.261 8

1 -5 -3 932.713 96.3295 5

1 -5 -3 1103.32 121.655 4

-1 -5 3 1029.12 106.644 5

1 -5 -3 1118.16 104.002 3

1 -5 -3 844.021 124.224 1

1 -5 -3 1075.43 85.1547 9

-1 -5 3 1080.90 104.054 10

-1 5 3 1115.71 112.045 10

-1 5 3 1142.67 117.367 6

1 -5 -3 1031.87 112.953 8

-1 5 3 935.846 115.192 8

1 -5 -2 68.1293 36.7283 5

1 -5 -2 155.457 59.3282 1

-1 5 2 100.528 27.1699 2

-1 -5 2 72.9634 23.3952 1

-1 5 2 92.8092 29.4846 9

1 -5 -2 116.195 31.2456 9

1 -5 -2 93.1483 38.5026 8

-1 -5 2 91.9764 28.0271 7

-1 5 1 1802.61 153.382 5

1 -5 -1 2024.13 146.035 5

-1 5 1 1385.41 101.748 3

1 -5 -1 1834.32 142.082 1

1 5 -1 1533.89 98.6124 1

-1 5 1 2344.41 144.444 1

-1 -5 1 1705.82 103.757 1

-1 5 1 1390.13 98.0621 9

1 5 -1 1385.97 94.5993 7

1 -5 -1 1810.55 141.542 8

-1 5 0 196956. 1115.01 1

1 -5 0 195041. 1169.14 1

-1 -5 0 175541. 1127.49 1

1 5 0 173042. 1129.58 1

-1 5 0 173707. 1144.12 3

1 -5 0 185795. 1392.18 5

1 5 0 173528. 1088.14 3

1 -5 0 182939. 1330.33 8

-1 5 0 190637. 1337.93 8

-1 -5 -1 6665.76 255.319 1

1 -5 1 7194.71 183.052 1

-1 5 -1 7784.72 174.015 1

1 -5 1 6814.82 275.711 5

-1 -5 -1 5435.60 189.158 3

-1 5 -1 5671.78 212.470 3

1 5 1 5610.49 190.821 3

1 -5 1 6565.32 249.482 8

1 5 2 37886.3 523.684 3

-1 -5 -2 37596.0 499.792 3

-1 5 -2 39305.8 676.268 5

1 -5 2 39062.4 698.680 5

-1 -5 -2 38872.9 654.917 1

1 5 2 38620.9 373.207 9

1 5 2 38670.9 662.379 6

1 -5 2 37455.3 562.292 8

-1 5 -3 1274.88 128.437 5

-1 -5 -3 1282.50 105.759 3

-1 -5 -3 1803.70 174.129 1

1 5 3 1584.51 203.661 1

1 5 3 1219.96 108.675 10

-1 5 -3 1606.35 131.434 10

-1 -5 -3 1184.08 70.1375 9

1 -5 3 1170.59 124.617 5

-1 -5 -3 1248.20 138.414 6

1 5 3 1059.63 137.072 6

1 -5 3 1236.39 69.1196 7

1 -5 3 1321.35 100.623 8

-1 5 -4 2842.92 192.533 5

-1 -5 -4 2697.08 212.149 1

1 5 4 2459.66 196.856 1

-1 5 -4 2753.31 189.306 10

-1 -5 -4 2701.76 114.616 9

1 5 4 2672.97 175.361 10

1 5 4 2750.53 229.647 6

1 -5 4 2759.87 196.645 5

-1 -5 -5 2356.02 215.605 4

1 -5 5 2157.06 136.124 4

-1 5 -5 2158.80 186.285 5

-1 -5 -5 2687.21 228.177 1

1 -5 5 1864.77 78.3362 1

1 5 5 2328.93 205.734 1

-1 5 -5 2321.18 190.945 10

1 -5 5 2227.70 180.853 10

1 5 5 2222.88 178.236 10

1 -5 5 2377.01 189.345 5

-1 -5 -5 1849.94 161.838 8

1 5 5 2331.13 233.124 6

-1 -5 -6 8206.66 419.936 4

1 -5 6 8058.00 288.089 4

-1 5 -6 7630.30 343.545 5

1 -5 6 7339.62 196.667 1

-1 -5 -6 9201.23 449.380 1

1 -5 6 8274.34 369.170 10

-1 5 -6 8046.55 391.289 10

1 5 6 7940.58 370.896 10

1 5 6 8685.74 483.104 6

-1 -5 -6 7594.42 328.533 8

-1 -5 -7 5993.41 384.708 4

1 -5 7 6026.79 271.936 4

-1 5 -7 5841.47 305.159 5

-1 -5 -7 5726.61 375.601 1

1 -5 7 6475.47 352.072 10

-1 5 -7 5853.40 355.845 10

1 5 7 5552.33 334.330 10

-1 -5 -7 5953.51 322.094 10

-1 -5 -7 5256.23 279.381 8

1 5 7 6224.58 441.001 6

-1 -5 -8-29.0317 51.5305 4

-1 5 -8 28.4952 25.2281 5

-1 -5 -8 2.02109 39.5710 1

1 -5 8 20.4931 23.7740 1

1 5 8 42.3435 37.9038 1

-1 5 -8-34.6409 46.4152 10

-1 -5 -8 49.6226 39.6072 10

1 -5 8 25.0531 29.8999 10

1 5 8 94.1450 55.8375 10

-1 -5 -8 83.6477 57.4907 6

-1 -5 -9 595.930 133.490 4

-1 5 -9 547.444 97.8594 5

1 5 9 477.141 129.334 1

-1 -5 -9 541.122 128.036 1

1 -5 9 576.764 80.6873 1

1 5 9 565.573 125.002 10

1 -5 9 481.099 111.723 10

-1 -5 -9 716.965 131.369 10

-1 5 -9 627.241 138.662 10

-1 -5 -9 688.651 156.008 6

-1 5 -10 922.798 127.645 5

-1 -5 -10 877.596 172.571 4

1 -5 10 916.098 112.981 1

-1 -5 -10 1126.90 198.308 1

-1 5 -10 701.031 137.150 2

1 5 10 807.825 154.749 1

-1 5 -10 1063.92 180.488 10

1 -5 10 996.349 169.435 10

-1 -5 -10 907.917 152.889 10

1 5 10 885.248 159.859 10

-1 5 -10 1044.31 218.961 6

-1 -5 -10 788.427 173.370 6

-1 5 -11 733.968 147.107 2

-1 -5 -11 860.695 175.562 1

1 -5 11 949.928 124.074 1

-1 5 -11 980.050 184.862 10

-1 -5 -11 1014.08 179.868 10

1 5 11 955.038 181.799 10

-1 -5 -11 919.582 99.1566 5

-1 5 -11 974.041 225.648 6

-1 -5 -11 835.332 190.645 6

-1 5 -12 1076.12 204.288 10

-1 -5 -12 1319.08 255.481 6

-1 5 -12 1435.71 285.583 6

-1 5 -13-11.9026 58.4219 10

-1 -5 -13 59.6053 94.8350 6

-1 5 -13-17.0665 54.9653 6

-1 5 -14 170.137 109.512 10

-1 -5 -14 81.2919 50.8119 6

-1 5 -14 31.1685 102.764 6

-1 5 -15-42.2415 41.2588 10

1 6 -16-75.1351 77.5617 10

1 6 -15 284.922 129.151 10

1 6 -14 28.0368 53.3864 10

1 -6 -14 144.574 105.225 6

1 6 -14 50.4601 105.841 6

1 6 -13 42.1261 87.9140 10

1 -6 -13-48.9639 81.0884 6

1 6 -13 88.9250 96.7225 6

1 6 -12 370.557 129.290 10

1 6 -12 284.094 120.445 6

1 -6 -12 377.911 126.787 6

-1 -6 11 11.1918 28.2457 1

1 6 -11-48.9622 67.6718 10

1 -6 -11-18.8474 51.7202 10

1 -6 -11 65.0282 87.6538 6

1 6 -11 3.32348 49.3951 6

1 -6 -10 2974.06 125.130 5

-1 -6 10 3004.02 205.406 1

-1 6 10 3537.02 342.938 1

-1 6 10 3428.01 338.505 10

1 -6 -10 3490.58 316.755 10

1 6 -10 3560.48 346.083 10

1 -6 -10 3406.64 350.455 6

-1 -6 9 39.3831 50.2852 4

1 -6 -9 70.7929 26.5925 5

1 -6 -9 88.8388 39.3157 4

1 -6 -9 91.9621 139.101 1

-1 6 9 122.124 91.6122 1

-1 -6 9 75.9973 42.0288 1

-1 -6 9 69.9935 40.3102 10

-1 6 9 42.4016 27.0915 10

1 6 -9 5.52925 43.9551 10

1 -6 -9 22.9551 48.4534 10

1 -6 -9-35.4658 55.1395 6

1 -6 -8 5355.98 396.648 4

1 -6 -8 4545.90 204.822 5

-1 -6 8 5518.71 333.066 4

1 -6 -8 5150.87 389.681 1

-1 -6 8 4698.78 234.406 1

-1 6 8 5141.89 377.706 1

1 6 -8 5110.68 369.871 10

1 -6 -8 5273.36 341.913 10

-1 -6 8 5709.84 365.583 10

-1 6 8 5074.12 366.717 10

-1 6 8 5699.97 440.722 6

1 -6 -8 4940.86 368.497 6

-1 -6 8 5431.26 289.291 5

1 -6 -7 309.951 93.9998 4

-1 -6 7 379.631 80.1778 5

1 -6 -7 432.673 125.236 1

-1 6 7 328.726 118.315 1

-1 -6 7 287.241 59.8802 1

-1 -6 7 304.213 80.1574 10

-1 6 7 277.644 81.3470 10

1 6 -7 299.165 82.9278 10

-1 6 7 415.903 113.382 6

1 -6 -6 7605.90 422.253 4

1 -6 -6 6670.34 403.821 1

-1 -6 6 7259.58 259.217 1

1 6 -6 7465.70 385.707 10

-1 -6 6 7785.48 373.179 10

-1 6 6 7492.88 385.218 10

-1 -6 6 7277.32 333.228 5

-1 6 6 7643.99 438.305 6

1 -6 -5 1540.90 172.343 4

-1 -6 5 1481.60 145.188 5

1 -6 -5 1427.60 171.007 1

1 -6 -5 1512.55 117.175 9

-1 -6 5 1502.14 150.228 10

-1 6 5 1660.06 163.502 10

1 -6 -5 1503.05 156.380 8

-1 6 5 1507.50 177.894 6

1 -6 -4 4639.45 283.209 4

-1 -6 4 4478.58 246.770 5

1 -6 -4 4847.35 297.853 1

-1 -6 4 4708.59 243.058 10

1 -6 -4 4870.10 201.007 9

-1 6 4 4950.68 265.381 10

1 -6 -4 3968.98 259.449 8

-1 6 4 4728.95 288.502 6

-1 -6 3 2546.03 182.745 5

1 -6 -3 2672.61 165.341 3

1 -6 -3 2395.82 167.311 5

1 -6 -3 2520.79 203.108 1

-1 6 3 2476.45 105.659 2

-1 -6 3 2678.08 121.247 1

1 -6 -3 2640.36 141.953 9

-1 6 3 2707.68 151.629 9

-1 -6 3 2641.41 137.208 7

-1 6 3 2717.92 192.848 6

1 -6 -3 2521.88 190.133 8

1 -6 -2 32.4699 13.5245 3

1 -6 -2 3.04155 18.1728 5

1 -6 -2 43.3363 26.6895 1

-1 -6 2 37.8454 18.2048 1

1 -6 -2 39.9847 25.3752 8

-1 -6 2 43.8283 18.9455 7

1 6 -2 44.2985 19.7017 7

1 -6 -2 36.1808 20.2742 9

-1 6 1 140.432 36.1630 1

1 -6 -1 94.8979 36.3248 1

-1 -6 1 116.396 30.6265 1

1 6 -1 104.985 28.6391 1

1 -6 -1 108.413 41.6124 5

-1 6 1 62.0853 22.7335 3

1 6 -1 92.1556 30.1013 7

-1 6 1 92.5890 29.9282 9

1 -6 -1 91.9329 39.5022 8

-1 -6 0 32.7179 22.6765 1

-1 6 0 5.15362 14.0279 1

1 -6 0 4.20118 22.8896 1

1 6 0 41.9810 33.8049 1

1 6 0 10.7455 16.3494 3

1 -6 0-0.11120 17.1218 5

1 -6 0-3.91221 20.2031 8

1 -6 1 2330.15 126.353 1

-1 6 -1 2726.88 118.869 1

-1 -6 -1 2568.50 167.374 1

1 -6 1 2352.63 175.965 5

-1 -6 -1 1299.57 102.580 3

-1 6 -1 1372.44 115.668 3

1 -6 1 2603.51 167.350 8

1 6 2 3608.74 174.226 3

1 -6 2 3665.50 226.594 5

-1 -6 -2 3855.53 173.569 3

-1 6 -2 3705.14 222.123 5

-1 -6 -2 3351.70 206.208 1

1 6 2 3735.18 132.926 9

-1 6 -2 3744.14 127.206 7

1 6 2 3873.70 223.661 6

1 -6 2 3468.54 187.541 8

1 -6 3 16410.2 501.193 5

-1 6 -3 15601.3 471.410 5

-1 -6 -3 17130.5 391.965 3

-1 -6 -3 14825.1 478.023 1

-1 -6 -3 16850.0 274.592 9

-1 6 -3 17289.6 444.211 10

1 -6 3 16236.3 383.119 8

1 6 3 17117.3 525.004 6

-1 6 -4 7502.51 351.711 5

-1 -6 -4 7720.20 280.397 3

-1 -6 -4 7470.21 389.584 1

-1 -6 -4 7323.67 190.875 9

1 6 4 7309.78 300.360 10

-1 6 -4 7750.84 324.887 10

1 -6 4 7985.61 359.461 5

1 -6 4 7255.72 180.943 7

1 6 4 7432.54 389.440 6

-1 6 -5 1186.01 153.223 5

1 6 5 1167.91 170.707 1

-1 -6 -5 1310.32 181.685 1

-1 -6 -5 1085.46 83.8681 9

1 6 5 974.990 123.279 10

-1 6 -5 1215.71 148.848 10

1 6 5 1138.42 166.687 6

1 -6 5 1240.82 153.703 5

-1 -6 -5 1261.95 156.994 8

1 -6 6 457.021 68.8147 4

-1 6 -6 479.080 90.0059 5

-1 -6 -6 513.251 106.431 4

-1 -6 -6 497.434 111.741 1

1 -6 6 374.305 35.0347 1

1 6 6 529.360 95.2353 10

-1 6 -6 481.663 97.4769 10

-1 -6 -6 409.125 83.8345 8

1 6 6 530.659 119.789 6

1 -6 6 499.875 93.8359 5

1 -6 7 454.459 74.6230 4

-1 6 -7 416.962 79.0929 5

-1 -6 -7 458.703 107.076 4

1 6 7 339.640 105.259 1

1 -6 7 433.052 45.8800 1

-1 -6 -7 486.465 107.945 1

1 6 7 540.378 110.661 10

-1 6 -7 467.753 108.908 10

1 -6 7 634.267 111.991 10

1 6 7 531.939 132.262 6

-1 -6 -7 434.297 85.5274 8

-1 -6 -7 428.510 108.989 6

1 -6 8 538.170 86.7181 4

-1 -6 -8 397.971 107.417 4

-1 6 -8 414.339 94.6619 5

1 6 8 503.145 118.622 1

-1 -6 -8 435.237 115.126 1

1 -6 8 466.514 56.4043 1

1 -6 8 632.958 121.569 10

-1 6 -8 524.411 124.957 10

1 6 8 455.744 104.349 10

-1 -6 -8 439.989 90.5299 8

-1 -6 -8 475.305 123.389 6

1 6 8 448.318 129.649 6

-1 -6 -9 7486.48 492.285 4

-1 6 -9 6826.85 366.069 5

-1 -6 -9 7305.31 489.932 1

1 6 9 7566.05 484.246 1

1 -6 9 7147.75 253.602 1

1 6 9 7555.95 459.415 10

-1 6 -9 7585.95 479.271 10

1 -6 9 8302.53 456.855 10

-1 -6 -9 7584.10 513.404 6

-1 -6 -10 166.060 66.5850 4

-1 6 -10 155.773 44.6355 5

-1 -6 -10 225.570 91.3783 1

1 -6 10 184.838 37.0717 1

1 6 10 229.008 93.8353 10

-1 6 -10 192.340 80.1699 10

1 -6 10 359.969 111.961 10

-1 -6 -10 150.321 68.7000 6

-1 -6 -11 16.5195 73.7617 1

1 -6 11 84.3140 41.6004 1

-1 6 -11 75.8302 38.0936 2

1 6 11 138.302 93.7636 10

-1 6 -11 118.481 75.0279 10

-1 -6 -11 38.6138 67.2074 6

-1 6 -11-5.25990 48.8228 6

-1 -6 -11 49.3944 27.8794 5

-1 6 -12 386.917 132.576 10

-1 6 -12 500.384 193.022 6

-1 -6 -12 447.324 148.813 6

-1 6 -13 25.0187 57.0510 10

-1 -6 -13 14.5357 47.6122 6

-1 6 -13 108.635 64.3479 6

-1 6 -14 19.5899 63.8911 10

-1 6 -14-29.2549 75.1379 6

-1 -6 -14 109.344 45.2173 6

-1 6 -15 153.767 100.099 10

1 7 -16 125.155 134.468 10

1 7 -15-126.700 89.8306 10

1 7 -14 12.9443 55.4712 10

1 7 -14-35.3471 88.0589 6

1 -7 -14 115.611 90.6721 6

1 7 -13 16.9283 63.1251 10

1 -7 -13 214.806 117.594 6

1 7 -13 224.719 123.544 6

-1 -7 12 192.457 85.3666 4

1 7 -12 2.64033 61.6127 10

1 7 -12 187.404 114.645 6

1 -7 -12 33.4249 60.2242 6

-1 -7 11 116.910 41.3717 1

1 7 -11 293.283 111.797 10

1 -7 -11 229.296 88.0522 6

1 -7 -10 45.8527 26.8374 5

-1 -7 10 0.48475 20.2442 1

1 7 -10-27.4070 52.1588 10

-1 7 10 14.8359 48.0063 10

1 -7 -10-72.3102 68.7221 6

1 -7 -9 18.0485 15.9020 5

-1 -7 9 29.0094 27.1140 4

1 -7 -9 79.7406 53.8679 4

-1 7 9 80.1949 46.6851 1

-1 -7 9 61.6744 25.7632 1

1 -7 -9 100.007 76.1962 1

1 7 -9-31.1123 41.5478 10

-1 -7 9 71.0408 42.6325 10

-1 7 9 39.0006 55.7401 10

1 -7 -9 83.1824 54.8538 6

-1 -7 9 76.2198 38.5545 5

1 -7 -8 3217.59 189.579 5

1 -7 -8 3637.39 339.104 4

1 -7 -8 3404.83 337.317 1

-1 -7 8 3219.06 178.503 1

-1 7 8 3749.77 325.289 1

1 7 -8 3811.87 324.928 10

-1 -7 8 3890.19 303.960 10

-1 7 8 3475.78 307.354 10

1 -7 -8 3479.85 314.708 6

-1 -7 8 3298.07 122.333 8

-1 7 8 3992.41 375.332 6

-1 -7 8 3560.78 256.235 5

1 -7 -7 255.782 86.6274 4

-1 -7 7 313.473 77.2699 5

-1 -7 7 304.646 53.8217 1

-1 7 7 243.039 81.0048 1

1 -7 -7 242.082 81.8437 1

-1 -7 7 408.809 98.4040 10

1 7 -7 315.617 87.7988 10

1 -7 -7 308.310 61.8363 9

-1 7 7 267.628 79.9962 10

-1 7 7 401.608 114.660 6

-1 -7 6 4774.01 293.501 5

1 -7 -6 4590.08 337.625 4

1 -7 -6 5084.37 371.506 1

-1 -7 6 4653.99 295.631 10

-1 7 6 4394.27 299.566 10

1 7 -6 4764.23 320.514 10

1 -7 -6 4756.75 226.323 9

1 -7 -6 4875.97 312.723 8

-1 7 6 4665.47 350.889 6

-1 -7 5 2030.71 183.724 5

1 -7 -5 2064.50 214.600 4

1 -7 -5 2426.21 240.459 1

-1 -7 5 2260.06 189.581 10

-1 7 5 2233.45 197.575 10

1 -7 -5 2304.30 149.181 9

1 -7 -5 1885.30 187.201 8

-1 7 5 2220.62 223.151 6

-1 -7 4-1.74847 19.6256 5

1 -7 -4-2.47375 17.1481 4

1 -7 -4-3.76398 20.0378 3

1 -7 -4 26.8191 52.1091 1

-1 7 4 22.2716 30.9803 10

1 -7 -4 2.66584 13.7827 9

-1 7 4 23.8907 24.0015 6

1 -7 -4-11.4939 33.0928 8

-1 -7 4 5.99748 11.6504 7

1 -7 -3 341.084 70.4106 5

1 -7 -3 460.413 76.9610 3

-1 -7 3 464.792 116.199 5

-1 -7 3 483.406 70.1001 1

-1 7 3 477.706 66.6538 2

-1 7 3 427.923 62.5099 9

1 -7 -3 486.680 65.2627 9

-1 -7 3 531.969 68.1146 7

-1 7 3 484.152 88.4225 6

1 -7 -3 562.741 100.020 8

1 -7 -2-9.38465 31.1946 1

-1 -7 2 63.9486 20.6608 1

-1 7 2 22.5697 27.4064 1

1 -7 -2 11.2781 15.6137 3

-1 7 2 8.36121 11.8260 3

1 -7 -2 31.4182 34.0763 5

1 -7 -2 3.02215 17.0186 8

1 7 -2 14.9912 17.5264 7

-1 7 2 34.3104 23.2837 9

-1 7 1 1789.86 146.390 1

1 -7 -1 1927.52 153.005 1

-1 -7 1 1810.47 122.639 1

1 7 -1 1896.28 124.611 1

1 7 -1 1967.93 142.848 3

1 -7 -1 1920.70 164.685 5

-1 7 1 1839.67 139.223 3

1 7 -1 2028.72 131.941 7

1 -7 -1 2027.60 168.786 8

1 -7 0 42876.6 644.066 1

-1 7 0 41420.4 595.277 1

-1 -7 0 39908.2 628.563 1

1 7 0 38757.9 616.213 1

-1 -7 0 38320.9 603.968 3

1 7 0 37786.1 609.840 3

1 -7 0 41826.7 772.313 5

-1 7 0 38574.3 633.851 3

1 -7 0 41325.7 743.364 8

-1 -7 -1 4806.49 240.003 1

1 7 1 4621.01 234.410 1

1 -7 1 4769.45 192.433 1

-1 7 -1 4707.75 173.489 1

-1 -7 -1 4173.27 196.907 3

1 7 1 4141.99 195.393 3

1 -7 1 4581.19 270.385 5

-1 7 -1 4278.29 215.542 3

1 -7 1 4638.92 247.310 8

1 -7 2 293.792 74.4154 5

1 7 2 328.368 56.9017 3

-1 7 -2 378.239 79.6813 5

-1 -7 -2 378.125 57.7536 3

1 -7 2 342.874 42.3935 1

-1 -7 -2 321.799 68.3412 1

-1 7 -2 391.660 37.8244 1

1 7 2 419.381 49.1867 9

1 -7 2 362.638 65.9857 8

-1 7 -2 370.953 43.4816 7

-1 -7 -3 1049.13 119.005 3

-1 7 -3 5603.23 307.116 5

1 -7 3 2878.51 233.457 5

-1 -7 -3 6035.49 336.990 1

-1 7 -3 1864.22 178.411 10

1 -7 3 1541.23 151.966 8

1 7 3 1622.42 175.432 6

-1 7 -4 111.174 49.7971 5

-1 -7 -4 118.964 40.1106 3

1 -7 4 110.775 50.8033 5

-1 -7 -4 176.436 62.6149 1

-1 7 -4 101.309 39.6692 10

1 7 4 91.9088 36.7072 10

1 -7 4 132.234 38.7190 8

-1 -7 -4 86.0246 21.7241 9

1 -7 4 98.7197 22.8588 7

1 7 4 144.340 57.1847 6

-1 -7 -5 2099.49 173.857 3

-1 7 -5 3264.65 255.031 5

-1 -7 -5 7166.43 421.619 1

1 7 5 1578.97 163.462 10

-1 7 -5 2812.62 230.528 10

-1 -7 -5 1688.95 109.822 9

1 -7 5 2238.30 259.839 5

-1 -7 -5 6036.22 341.604 8

1 -7 5 1772.15 103.869 7

1 7 5 1668.78 232.039 6

-1 7 -6 1728.64 178.770 5

-1 -7 -6 1849.47 211.085 4

-1 -7 -6 2033.68 229.271 1

1 7 6 2027.51 223.076 1

1 7 6 1648.55 174.915 10

-1 7 -6 1737.93 190.284 10

-1 -7 -6 1612.17 111.804 9

1 7 6 1857.85 232.717 6

1 -7 6 1766.29 192.553 5

-1 -7 -6 2307.45 201.339 8

-1 7 -7 8222.65 398.666 5

1 -7 7 8710.92 321.986 4

-1 -7 -7 8937.49 493.158 4

-1 -7 -7 8153.11 477.349 1

1 7 7 7877.21 478.914 1

1 7 7 8320.91 425.862 10

-1 7 -7 8687.79 453.072 10

-1 -7 -7 8571.50 281.702 9

1 7 7 8924.46 547.093 6

-1 -7 -7 7446.05 372.646 8

-1 -7 -7 8814.53 488.568 6

1 -7 7 8467.59 417.025 5

-1 7 -8 988.178 143.871 5

-1 -7 -8 1016.29 177.029 4

1 -7 8 1086.33 124.526 4

-1 -7 -8 1110.61 188.758 1

1 7 8 1087.95 181.073 1

1 -7 8 869.195 61.6248 1

-1 7 -8 990.581 168.942 10

-1 -7 -8 1060.85 108.191 9

1 7 8 989.866 156.783 10

-1 -7 -8 1164.21 187.986 6

-1 -7 -8 1044.86 145.378 8

-1 7 -9 44.3361 37.2544 5

-1 -7 -9 18.8467 50.5017 4

1 -7 9 20.6552 14.9039 1

1 7 9-28.2557 79.7566 1

-1 -7 -9-42.7443 87.3764 1

-1 7 -9 38.1189 55.7777 10

1 7 9-77.0407 61.4388 10

-1 -7 -9 45.7449 62.3519 6

-1 -7 -10 1127.79 216.563 1

1 -7 10 1119.89 91.9781 1

1 7 10 1373.82 211.059 10

-1 7 -10 1410.07 220.670 10

-1 -7 -10 1275.63 232.601 6

-1 -7 -11 267.708 95.8759 1

1 7 11 293.841 99.9986 10

-1 7 -11 364.118 122.752 10

-1 -7 -11 483.323 154.548 6

-1 -7 -12 676.857 107.012 9

-1 7 -12 719.504 180.660 10

-1 7 -12 585.330 199.043 6

-1 -7 -12 406.958 155.169 6

-1 7 -13-97.7395 92.5167 10

-1 -7 -13 58.0928 75.7996 6

-1 7 -13 25.4839 90.8794 6

-1 7 -14 124.703 93.2131 10

-1 7 -14-63.1397 66.8167 6

-1 -7 -14-25.3693 45.0708 6

-1 7 -15-123.787 90.4381 10

1 8 -16-105.756 89.2708 10

1 8 -15 150.767 108.485 10

1 8 -14 345.639 139.332 10

1 -8 -14 376.128 147.529 6

1 8 -14 428.978 181.449 6

1 -8 -13 455.288 97.9797 9

1 8 -13 378.926 133.085 10

1 8 -13 495.980 172.630 6

1 -8 -13 440.774 151.595 6

1 -8 -12 288.305 83.5661 9

1 8 -12 227.009 103.794 10

1 -8 -12 354.681 142.816 6

1 -8 -11 506.913 56.4869 5

-1 -8 11 306.104 63.4623 1

1 -8 -11 760.946 125.554 9

1 8 -11 667.044 167.694 10

1 -8 -11 655.643 164.202 6

1 -8 -10 737.203 83.7124 5

-1 -8 10 689.755 81.5461 1

1 -8 -10 798.360 173.353 1

-1 8 10 759.950 167.499 10

1 8 -10 894.445 181.803 10

1 -8 -10 815.996 115.084 9

1 -8 -10 779.407 171.004 6

1 -8 -9 149.915 66.1625 4

-1 -8 9 62.6291 30.5555 4

1 -8 -9 130.243 45.3144 5

-1 -8 9 83.0208 25.7073 1

1 -8 -9 206.338 113.256 1

-1 8 9 129.271 83.0499 1

-1 -8 9 83.3550 60.2837 10

1 -8 -9 117.371 44.6711 9

1 8 -9 76.1026 75.1106 10

-1 8 9 41.3052 38.3353 10

1 -8 -9 79.3265 44.3569 6

-1 -8 9 101.935 47.5529 5

1 -8 -8-45.2392 54.9098 4

-1 -8 8 3.60742 24.4687 5

1 -8 -8 1.95152 21.6472 5

-1 -8 8 50.6006 22.2842 1

1 -8 -8 56.8502 91.1329 1

-1 8 8 3.91061 72.2161 1

-1 -8 8-3.65312 38.3475 10

1 -8 -8-3.12263 26.9634 9

-1 8 8 18.2484 39.9057 10

1 8 -8 31.8484 39.1949 10

-1 8 8 30.1924 30.0731 6

1 -8 -8 47.9507 46.8582 6

-1 -8 8 28.4785 20.2267 8

1 -8 -7 48.8283 48.0379 5

-1 -8 7 46.4763 50.5618 5

1 -8 -7 100.748 72.9544 4

-1 8 7 83.8361 93.5991 1

-1 -8 7 29.0332 21.6560 1

1 8 -7 39.1791 70.9180 10

1 -8 -7 40.0219 36.7914 9

-1 -8 7-6.17949 50.8204 10

-1 8 7 5.87754 28.7352 10

-1 8 7-12.3021 44.5578 6

-1 -8 6 3301.87 254.692 5

1 -8 -6 3723.52 312.534 4

1 -8 -6 3510.61 241.910 3

1 -8 -6 3112.77 292.227 1

1 8 -6 3380.23 276.395 10

1 -8 -6 3443.95 194.091 9

-1 8 6 3222.41 264.451 10

-1 8 6 3573.04 315.418 6

1 -8 -6 3141.36 261.074 8

1 -8 -5 880.101 119.213 3

1 -8 -5 1241.45 188.083 4

-1 -8 5 912.064 150.845 5

1 -8 -5 1743.66 236.151 1

1 -8 -5 881.355 94.6501 9

-1 8 5 721.911 116.557 10

1 -8 -5 1632.89 186.644 8

-1 8 5 975.674 159.350 6

-1 -8 5 817.439 92.1109 7

1 -8 -4 971.362 129.268 5

1 -8 -4 928.487 111.739 3

1 -8 -4 1033.24 145.366 4

-1 -8 4 842.058 124.794 5

-1 8 4 987.865 73.8745 2

1 -8 -4 1195.04 166.009 1

-1 -8 4 983.827 82.9460 1

1 -8 -4 907.285 90.6337 9

-1 -8 4 858.488 88.5266 7

1 -8 -4 1054.90 141.904 8

-1 8 4 944.137 136.079 6

-1 -8 3 3879.67 255.776 5

1 -8 -3 2128.58 222.695 5

1 -8 -3 783.409 100.866 3

-1 -8 3 1686.32 129.817 1

1 -8 -3 3007.69 280.888 1

1 -8 -3 2608.47 218.473 8

1 -8 -3 1221.17 108.973 9

-1 -8 3 1190.35 108.851 7

-1 -8 2 144.451 41.0120 1

1 8 -2 158.923 37.1860 1

-1 8 2 155.846 53.9704 1

1 -8 -2 216.382 74.2816 1

1 -8 -2 190.830 56.5886 5

-1 -8 2 124.009 51.1580 5

1 -8 -2 147.139 44.2262 3

-1 8 2 154.804 43.8478 3

-1 8 2 176.537 43.5766 9

1 -8 -2 192.388 68.0502 8

1 8 -2 156.398 43.2913 7

1 8 -1 62.2764 23.9705 1

1 8 -1 36.3658 22.0131 3

-1 8 1 185.316 50.4947 1

1 -8 -1 129.793 53.1868 1

-1 -8 1 154.338 39.6433 1

-1 8 1 21.7353 19.9934 3

1 -8 -1 187.743 49.9017 5

1 -8 -1 123.813 41.0178 8

1 8 -1 50.0889 17.8463 7

-1 -8 0 46.6710 46.2929 3

1 -8 0 67.8576 57.9371 1

-1 8 0 30.5846 18.7557 1

1 8 0 84.0757 40.9867 1

-1 -8 0 28.8027 26.4397 1

1 -8 0 42.9057 38.3587 5

-1 8 0 25.0683 34.8469 3

1 8 0 37.4116 34.3196 3

1 -8 0 35.7421 38.3205 8

1 8 1 2149.48 169.077 1

-1 8 -1 2515.85 137.452 1

-1 -8 -1 2283.90 178.618 1

1 -8 1 2384.97 150.055 1

1 8 1 1149.73 112.186 3

-1 8 -1 1199.50 123.174 3

1 -8 1 2218.40 194.142 5

-1 -8 -1 1209.47 113.882 3

1 -8 1 2426.75 187.213 8

1 -8 2 7608.11 367.611 5

1 8 2 7084.77 277.606 3

-1 -8 -2 6896.42 270.285 3

-1 -8 -2 7524.04 355.642 1

-1 8 -2 7567.45 197.437 1

-1 8 -2 7099.93 308.412 3

1 -8 2 7682.70 231.618 1

1 8 2 6811.97 224.767 9

-1 8 -2 7053.88 216.268 7

1 -8 2 7513.66 331.097 8

1 -8 3 4226.66 296.018 5

-1 -8 -3 2433.59 165.265 3

-1 8 -3 5831.40 336.744 5

-1 -8 -3 4572.55 314.747 1

1 8 3 2202.82 122.234 9

1 -8 3 2641.48 197.126 8

1 8 3 2315.03 218.171 6

-1 -8 -4 4222.89 223.043 3

-1 8 -4 4892.01 304.278 5

-1 -8 -4 4237.38 306.217 1

1 8 4 4099.69 243.328 10

-1 8 -4 4332.77 260.559 10

1 -8 4 4280.08 298.514 5

1 -8 4 4019.81 221.357 8

1 8 4 4252.94 307.057 6

-1 8 -5 592.609 130.626 5

1 -8 5 106.574 68.2719 5

-1 -8 -5 140.981 57.6426 3

-1 -8 -5 1423.96 211.602 1

-1 8 -5 322.668 96.3928 10

1 8 5 67.8687 39.9458 10

-1 -8 -5 1319.40 170.416 8

1 -8 5 46.8093 27.2936 7

1 8 5 53.1699 58.6611 6

-1 -8 -5 60.1655 31.8243 9

1 -8 6 230.149 70.6624 5

-1 -8 -6 241.126 80.1203 4

-1 -8 -6 279.276 68.2670 3

-1 8 -6 230.064 77.3821 5

1 8 6 169.384 80.4269 1

-1 -8 -6 219.911 87.9766 1

-1 -8 -6 254.832 44.6908 9

-1 8 -6 269.938 77.8083 10

1 8 6 320.350 86.1969 10

1 8 6 257.711 86.7274 6

1 -8 6 234.773 40.3120 7

-1 -8 -6 162.782 62.0914 8

1 -8 7 119.313 45.2447 4

-1 8 -7 142.272 68.8424 5

-1 -8 -7 180.463 106.931 4

-1 -8 -7 115.973 55.9919 3

1 8 7 184.901 82.6354 1

1 8 7 102.031 48.4137 10

-1 8 -7 128.563 77.4847 10

-1 -8 -7 135.405 37.7380 9

-1 -8 -7 142.843 91.1107 6

1 -8 7 142.306 94.1007 5

1 8 7 169.163 91.2532 6

1 -8 7 114.119 32.0217 7

-1 -8 -7 215.208 81.8146 8

1 -8 8 821.327 104.552 4

-1 -8 -8 723.322 148.596 4

-1 8 -8 725.458 119.333 5

-1 -8 -8 701.147 150.001 1

1 8 8 800.101 161.868 1

-1 8 -8 713.606 141.321 10

1 8 8 757.853 139.333 10

-1 -8 -8 765.314 90.2412 9

-1 -8 -8 978.963 178.183 6

-1 -8 -8 667.886 116.981 8

-1 8 -9 9790.01 477.435 5

-1 -8 -9 10435.3 612.491 4

1 -8 9 11138.2 404.420 4

-1 -8 -9 10036.7 604.226 1

-1 8 -9 10952.2 590.327 10

1 8 9 10663.7 554.442 10

-1 -8 -9 11343.3 368.284 9

-1 -8 -9 11198.2 627.193 6

-1 -8 -10 3674.73 379.078 1

-1 8 -10 3575.24 357.361 10

1 8 10 3743.40 350.952 10

-1 -8 -10 4014.01 230.510 9

-1 -8 -10 3921.43 389.430 6

-1 -8 -11 498.832 161.573 1

-1 8 -11 423.486 133.169 10

-1 -8 -11 411.070 75.4610 9

1 8 11 477.832 134.455 10

-1 -8 -11 498.835 149.484 6

-1 -8 -12 46.5303 67.0287 1

-1 8 -12 61.4249 78.1350 10

-1 -8 -12 144.031 111.126 6

-1 8 -12 21.5077 51.3908 6

-1 8 -13 68.7808 59.3111 10

-1 -8 -13-16.8344 51.7499 6

-1 8 -13 76.3400 101.057 6

-1 -8 -14-42.2958 78.7146 3

-1 8 -14-27.5715 78.7608 10

-1 8 -15-43.0142 56.1872 10

1 -9 -15 64.5647 67.5498 3

1 9 -15 16.1651 57.3119 10

1 9 -14 118.717 74.8844 10

1 9 -14 75.1726 69.0212 6

1 -9 -14 168.426 119.047 6

1 9 -13-25.4601 60.7603 10

1 -9 -13 146.469 115.487 6

1 9 -13 140.057 98.7429 6

1 9 -12 244.254 124.042 10

1 -9 -12 205.679 58.5444 9

1 -9 -12 421.892 148.349 6

-1 -9 11 514.485 69.2070 1

1 -9 -11 948.454 130.123 9

1 9 -11 1052.21 201.933 10

1 -9 -11 754.919 178.059 6

1 -9 -10 50.7487 29.0194 5

1 -9 -10-47.3224 85.8859 1

-1 -9 10 83.4433 27.0262 1

1 9 -10 22.8193 54.8226 10

1 -9 -10 40.0208 31.7834 9

-1 9 10 66.7943 67.0212 10

1 -9 -10 136.112 82.2695 6

-1 -9 10 72.8594 39.0035 5

1 -9 -9 85.5043 44.0633 5

1 -9 -9 129.816 117.057 1

-1 -9 9 85.3550 28.5297 1

1 -9 -9 53.9897 27.1060 9

1 9 -9 145.700 86.9052 10

-1 9 9 82.9892 48.8616 10

1 -9 -9 119.823 74.6213 6

-1 -9 9 63.2068 49.1603 5

1 -9 -8 129.724 35.2960 5

1 -9 -8 77.7243 40.4419 4

-1 -9 8 128.581 66.5675 5

1 -9 -8 76.5505 48.5445 3

-1 9 8 118.860 87.2548 1

-1 -9 8 109.490 26.6589 1

1 -9 -8 66.0358 58.6252 1

1 -9 -8 113.545 41.0326 9

-1 9 8 32.8675 45.9254 10

1 9 -8 98.2900 61.4673 10

-1 -9 8 115.562 28.7856 8

1 -9 -8 80.4966 43.5779 6

1 -9 -7 9975.73 432.982 3

1 -9 -7 8218.69 366.735 5

1 -9 -7 9141.75 532.141 4

-1 -9 7 8895.95 258.027 1

1 -9 -7 9286.95 528.760 1

-1 9 7 8819.16 507.088 1

1 9 -7 9754.84 503.281 10

1 -9 -7 9460.79 341.434 9

-1 9 7 9282.66 488.169 10

-1 -9 7 9338.22 331.555 7

-1 9 7 10435.1 585.146 6

-1 -9 7 9927.38 475.933 5

-1 -9 6 163.458 69.2582 5

1 -9 -6 184.246 87.1341 4

1 -9 -6 160.531 55.1564 3

1 -9 -6 103.733 41.3685 5

-1 -9 6 119.838 26.3428 1

1 -9 -6 118.254 68.9023 1

-1 9 6 127.047 57.1316 10

1 -9 -6 107.499 34.8780 9

1 9 -6 114.286 52.8617 10

-1 9 6 93.1347 65.5237 6

-1 -9 6 109.172 36.6731 7

1 -9 -6 67.4838 40.4609 8

-1 -9 5 4336.09 300.408 5

1 -9 -5 5350.61 294.849 3

1 -9 -5 4477.37 353.773 4

1 -9 -5 4239.66 270.473 5

1 -9 -5 5163.78 375.709 1

-1 -9 5 4695.64 175.872 1

1 -9 -5 4726.61 231.055 9

-1 9 5 4841.17 314.555 10

-1 -9 5 4888.50 224.676 7

-1 9 5 4992.58 355.259 6

1 -9 -4 477.907 103.156 5

-1 -9 4 657.066 140.141 5

1 -9 -4 430.802 83.2095 3

-1 9 4 527.006 70.1848 2

1 -9 -4 331.746 105.477 1

-1 -9 4 387.824 66.3214 1

-1 9 4 339.509 68.9254 9

1 -9 -4 610.616 127.902 8

-1 9 4 337.834 93.3786 6

-1 -9 4 409.400 71.8285 7

1 -9 -4 433.742 74.4494 9

1 -9 -3 20634.3 626.998 5

-1 -9 3 20184.8 657.559 5

1 -9 -3 15889.1 573.275 1

-1 -9 3 15945.8 368.111 1

1 -9 -3 16702.0 594.326 8

1 -9 -2 178.283 54.7066 1

1 9 -2 194.598 45.5587 1

-1 -9 2 172.826 40.2701 1

-1 9 2 200.660 62.2933 1

-1 9 2 226.151 53.6856 3

-1 -9 2 174.463 69.2949 5

1 -9 -2 236.495 71.4907 5

-1 9 2 209.466 49.0091 9

1 9 -2 255.125 53.6177 7

1 -9 -2 180.392 65.7607 8

1 9 -1 958.732 101.098 1

-1 -9 1 993.211 103.371 1

-1 9 1 889.671 111.997 1

1 -9 -1 1038.44 127.638 1

1 9 -1 1073.13 122.154 3

-1 9 1 1082.21 122.358 3

1 -9 -1 899.529 130.336 5

1 9 -1 1042.95 107.443 7

1 -9 -1 1043.32 138.491 8

-1 -9 0 1006.86 110.312 1

1 -9 0 986.755 108.660 1

-1 9 0 1085.77 106.925 1

1 9 0 1021.91 109.567 1

-1 9 0 972.783 113.908 3

1 9 0 1025.69 116.795 3

1 -9 0 1107.53 143.585 5

1 -9 0 1008.26 131.661 8

1 -9 1 14971.7 395.141 1

-1 9 -1 14361.0 350.514 1

1 9 1 14498.5 458.939 1

-1 -9 -1 13585.0 446.025 1

-1 -9 -1 16323.7 441.977 3

1 9 1 16132.1 449.506 3

1 -9 1 14681.4 542.921 5

-1 9 -1 15950.3 475.968 3

1 -9 1 15610.6 512.198 8

-1 -9 -2 9827.34 344.575 3

1 9 2 9647.66 350.574 3

-1 9 -2 9967.87 382.910 3

-1 9 -2 13289.5 519.660 5

1 -9 2 11615.1 484.427 5

1 -9 2 11016.7 304.486 1

-1 9 -2 11165.8 270.358 1

-1 -9 -2 11312.6 464.081 1

1 9 2 9703.01 293.512 9

1 -9 2 12267.2 442.422 8

-1 9 -2 9985.10 281.240 7

-1 9 -3 15292.7 560.982 5

1 9 3 16405.1 468.795 3

-1 -9 -3 15945.3 439.720 3

1 -9 3 15396.8 575.537 5

1 -9 3 14152.5 272.792 1

-1 -9 -3 14615.2 529.987 1

1 9 3 15829.6 351.725 9

1 9 3 16298.2 566.220 6

-1 9 -3 15318.9 337.124 7

1 -9 3 14977.7 477.845 8

-1 9 -4 2392.57 224.768 5

1 -9 4 2168.67 225.778 5

-1 -9 -4 2521.05 178.399 3

-1 -9 -4 2109.95 215.748 1

-1 9 -4 2403.88 200.173 10

-1 -9 -4 1879.81 197.758 8

1 -9 4 2399.59 182.513 8

1 9 4 2619.71 245.780 6

-1 -9 -5 2943.47 201.898 3

-1 9 -5 2449.76 236.361 5

-1 -9 -5 2639.01 249.147 1

1 9 5 2904.83 230.320 10

-1 9 -5 2911.83 237.134 10

-1 -9 -5 2516.05 232.467 8

1 -9 5 3109.01 269.367 5

1 -9 5 2813.14 192.092 8

1 9 5 3032.18 283.473 6

-1 9 -6 387.345 101.176 5

1 -9 6 285.636 90.4379 5

-1 -9 -6 233.936 56.8005 3

-1 -9 -6 286.019 106.076 1

1 9 6 234.096 90.6569 1

-1 9 -6 246.379 84.2325 10

1 9 6 231.029 75.0157 10

-1 -9 -6 215.726 42.5706 9

-1 -9 -6 211.939 80.8791 8

1 -9 6 156.205 32.8881 7

1 9 6 233.070 88.1711 6

-1 9 -7 3955.91 302.382 5

-1 -9 -7 4530.92 279.678 3

-1 -9 -7 4072.00 348.551 4

1 9 7 4397.94 363.630 1

-1 -9 -7 3976.40 363.011 1

-1 9 -7 4292.48 331.476 10

-1 -9 -7 4139.34 191.466 9

1 9 7 4513.90 327.513 10

1 9 7 4844.27 417.889 6

1 -9 7 4109.93 329.678 5

1 -9 7 4005.71 178.841 7

-1 -9 -7 4562.20 357.784 6

-1 -9 -7 3660.93 288.578 8

1 -9 8 2455.99 183.556 4

-1 -9 -8 2536.88 296.715 4

-1 -9 -8 2426.16 221.908 3

-1 9 -8 2241.28 231.293 5

-1 -9 -8 2130.20 279.255 1

1 9 8 2259.39 278.684 1

-1 9 -8 2556.46 270.492 10

1 9 8 2246.97 250.274 10

-1 -9 -8 2211.45 151.096 9

-1 -9 -8 2404.91 280.950 6

-1 -9 -8 2170.10 230.847 8

1 -9 8 2359.00 245.592 5

1 -9 8 2283.82 141.612 7

-1 -9 -9 110.648 50.8346 3

1 -9 9 180.438 57.3029 4

-1 -9 -9 154.261 74.3330 1

-1 -9 -9 166.304 46.6699 9

-1 9 -9 114.025 64.1510 10

1 9 9 187.259 74.9790 10

-1 -9 -9 73.2104 42.0126 8

-1 -9 -9 203.377 98.6933 6

1 -9 9 151.975 38.3560 7

-1 -9 -10-2.19631 48.8081 3

-1 -9 -10-55.7944 94.3124 1

-1 -9 -10 29.5294 28.4238 9

-1 9 -10 90.5870 73.1537 10

1 9 10 84.2789 64.2212 10

1 -9 10 68.3690 31.5857 7

-1 -9 -10-20.3654 43.3052 6

-1 -9 -11 379.965 103.605 3

-1 -9 -11 628.369 174.472 1

-1 9 -11 580.269 160.989 10

-1 -9 -11 434.885 75.0151 9

-1 -9 -11 518.527 161.370 6

-1 -9 -12 1001.81 179.154 3

-1 -9 -12 860.782 210.326 1

-1 9 -12 875.882 204.016 10

-1 -9 -12 1223.45 240.232 6

-1 -9 -13 159.690 73.5346 3

-1 9 -13 400.061 144.070 10

-1 9 -13 587.571 200.633 6

-1 -9 -13 376.593 151.013 6

-1 -9 -14 3.89332 44.3634 3

-1 9 -14 52.6982 63.7199 10

-1 9 -15-240.732 123.975 10

1 -10 -15 315.273 117.432 3

1 10 -15 240.644 96.7164 10

1 -10 -14 186.811 92.4922 3

1 10 -14 443.747 159.373 10

1 -10 -14 143.304 90.0900 6

1 10 -14 157.748 120.304 6

1 -10 -13 441.791 134.739 3

1 10 -13 27.0716 78.0671 10

1 -10 -13 294.177 105.337 6

1 -10 -12 154.752 85.2114 3

1 10 -12 34.0366 66.3078 10

1 -10 -12 65.9984 50.7613 9

1 -10 -12 78.2215 48.0935 6

1 -10 -11 1749.93 225.556 3

1 -10 -11 1598.19 170.403 9

1 10 -11 1915.11 287.109 10

1 -10 -11 1769.09 267.074 6

1 -10 -10 171.299 77.8362 3

1 -10 -10 62.8002 23.3762 5

-1 -10 10 38.5087 17.7306 1

1 -10 -10 52.7585 64.8047 1

1 -10 -10 147.233 51.0244 9

1 10 -10 146.123 85.5223 10

-1 -10 10 139.151 58.9442 5

-1 -10 10 118.451 39.1428 7

1 -10 -9 1168.89 172.716 3

1 -10 -9 948.709 128.947 5

-1 -10 9 848.756 63.8968 1

1 -10 -9 1072.19 205.198 1

-1 10 9 1107.36 189.578 10

1 -10 -9 1075.57 128.502 9

1 10 -9 1044.11 197.235 10

-1 -10 9 952.789 162.605 5

-1 -10 9 937.910 116.869 7

1 -10 -8 1638.33 238.656 4

1 -10 -8 1664.71 174.510 5

1 -10 -8 1734.39 200.544 3

-1 -10 8 1900.85 98.7872 1

-1 10 8 1884.64 261.190 1

1 -10 -8 2246.23 300.597 1

-1 -10 8 1558.39 90.3566 1

1 -10 -8 1664.39 153.112 9

1 10 -8 1698.53 224.206 10

-1 10 8 1565.76 215.178 10

-1 -10 8 1767.61 218.448 5

-1 -10 8 1649.69 140.410 8

-1 -10 8 1625.35 145.313 7

-1 -10 7 2189.77 285.308 5

1 -10 -7 2431.42 224.328 3

1 -10 -7 2134.59 206.515 5

1 -10 -7 2189.64 269.388 4

1 -10 -7 2384.33 292.163 1

-1 -10 7 1997.84 103.038 1

-1 10 7 2215.22 333.121 1

1 10 -7 2649.02 279.280 10

-1 10 7 2195.36 241.603 10

1 -10 -7 2136.78 166.955 9

1 -10 -7 3222.95 300.012 8

-1 -10 7 2090.37 171.852 8

-1 10 7 2130.31 274.041 6

-1 -10 7 2027.87 158.221 7

-1 -10 6 45.6394 43.5996 5

1 -10 -6 95.5370 43.9632 5

1 -10 -6 47.4802 34.7193 4

1 -10 -6 30.2058 33.2866 3

-1 -10 6 37.6742 15.4179 1

1 -10 -6-25.2870 77.4295 1

1 10 -6 44.0160 30.1545 10

-1 10 6 4.42250 30.5403 10

-1 10 6 63.5555 43.2310 6

1 -10 -6 19.2129 43.2254 8

-1 -10 6 34.9115 20.9512 7

1 -10 -6 42.9512 27.5234 9

1 -10 -5 1111.46 198.162 4

1 -10 -5 1099.05 139.602 3

1 -10 -5 918.929 131.397 5

-1 -10 5 1279.79 218.382 5

-1 -10 5 996.467 83.7363 1

1 -10 -5 1662.76 262.440 1

-1 10 5 1094.47 156.083 10

1 -10 -5 1078.33 116.336 9

1 -10 -5 972.823 161.507 8

-1 -10 5 967.740 108.135 7

-1 10 5 1115.05 172.333 6

1 -10 -4 6335.66 310.442 3

1 -10 -4 5870.20 339.910 5

-1 -10 4 6578.08 383.893 5

1 -10 -4 6966.88 417.490 1

-1 10 4 6350.19 236.150 2

-1 -10 4 6061.22 213.033 1

-1 10 4 6148.17 275.044 9

1 -10 -4 6563.35 384.791 8

-1 -10 4 6275.57 260.834 7

-1 10 4 6570.32 383.732 6

-1 -10 3 4645.48 3182.63 5

1 -10 -3 9030.02 4427.92 5

-1 -10 3 6690.56 2518.44 1

1 -10 -3 6077.16 4258.72 1

1 -10 -3 6781.80 3858.64 8

1 -10 -2 1369.09 171.731 5

-1 10 2 1400.58 147.049 3

-1 -10 2 1444.54 178.661 5

-1 10 2 1333.90 154.076 1

1 10 -2 1296.98 121.645 1

1 10 -2 1310.43 146.148 3

1 -10 -2 1497.26 173.279 1

-1 -10 2 1352.82 123.799 1

-1 10 2 1242.97 126.793 9

1 -10 -2 1402.91 174.571 8

1 10 -2 1375.54 126.789 7

1 10 -1 3109.37 193.191 1

1 -10 -1 4129.43 262.388 1

-1 10 1 4307.45 250.267 1

-1 -10 1 4265.74 225.034 1

1 -10 -1 4398.83 302.456 5

1 10 -1 2461.77 194.260 3

-1 10 1 2045.64 176.326 3

1 10 -1 2740.37 184.177 7

1 -10 -1 3574.11 272.870 8

1 -10 0 165.668 77.5183 1

-1 10 0 100.943 44.7651 1

1 10 0 139.287 64.1449 1

-1 -10 0 100.369 53.1687 1

-1 10 0 71.8191 44.4559 3

1 10 0 100.936 55.0506 3

1 -10 0 104.689 65.3998 5

1 -10 0 111.053 63.7997 8

1 -10 1 13526.4 402.311 1

1 10 1 13308.8 458.545 1

-1 10 -1 12822.8 353.795 1

-1 -10 -1 12770.2 453.575 1

1 -10 1 14326.6 561.237 5

-1 -10 -1 14462.5 442.908 3

-1 10 -1 15142.7 485.709 3

1 10 1 15223.1 461.664 3

1 -10 1 13704.2 510.789 8

1 10 2 5277.73 270.600 3

-1 10 -2 5185.47 290.418 3

-1 -10 -2 5544.10 275.517 3

1 -10 2 6142.85 368.676 5

-1 -10 -2 5662.57 339.102 1

-1 10 -2 5420.61 205.541 1

1 -10 2 5399.19 239.650 1

1 10 2 5532.36 233.349 9

-1 10 -2 5516.86 222.963 7

1 -10 2 5903.86 328.792 8

1 -10 3 4099.73 361.142 5

-1 10 -3 7808.71 448.542 5

-1 -10 -3 7550.26 399.970 1

1 -10 3 2145.96 143.595 1

1 10 3 1471.47 202.031 6

-1 10 -3 1684.89 126.556 7

1 -10 3 5000.30 314.042 8

-1 10 -4 2678.96 249.241 5

1 -10 4 2482.02 244.603 5

-1 -10 -4 2300.90 178.137 3

-1 -10 -4 2457.03 238.153 1

-1 10 -4 2379.29 208.220 10

1 10 4 2436.35 240.658 6

-1 -10 -4 2200.70 219.365 8

1 -10 4 2230.50 190.654 8

-1 -10 -5 2248.14 188.513 3

-1 10 -5 4379.79 333.230 5

-1 -10 -5 4046.24 342.145 1

1 10 5 2164.34 205.847 10

-1 10 -5 2647.87 237.672 10

-1 -10 -5 3231.33 273.288 8

1 -10 5 2178.43 187.782 8

1 -10 5 3119.23 302.091 5

1 10 5 2524.77 269.304 6

-1 -10 -6 69.6236 53.3727 3

-1 10 -6 54.4893 61.8349 5

1 -10 6 85.1627 61.5118 5

-1 -10 -6 83.6027 79.6503 1

1 10 6 24.7305 40.9132 1

1 10 6 37.4074 33.6223 10

-1 10 -6 91.2737 65.6934 10

1 10 6 48.5017 58.8581 6

-1 -10 -6 87.2702 58.2317 8

-1 10 -7 202.480 155.493 5

-1 -10 -7 96.9277 83.3878 4

-1 -10 -7 41.3488 35.1657 3

1 -10 7 115.668 79.7370 5

1 10 7 74.9920 79.9794 1

1 10 7 55.7869 41.2103 10

-1 10 -7 143.201 77.6092 10

1 10 7-14.8996 53.0500 6

-1 -10 -7 115.964 41.8089 9

-1 -10 -7 224.759 90.1382 8

1 -10 7 80.8005 29.7606 7

1 -10 8 1295.12 137.385 4

-1 -10 -8 1308.88 162.908 3

-1 -10 -8 1079.29 197.460 1

-1 10 -8 1261.99 193.176 10

1 10 8 1096.69 174.818 10

-1 -10 -8 1257.21 110.820 9

-1 -10 -8 1032.69 164.306 8

1 -10 8 1307.74 197.776 5

1 -10 8 1153.57 100.518 7

-1 -10 -9 10284.2 478.360 3

1 -10 9 10464.7 384.355 4

-1 -10 -9 9116.03 605.535 1

-1 -10 -9 10156.4 333.929 9

1 10 9 9868.58 553.193 10

-1 10 -9 10545.1 600.017 10

1 -10 9 9698.80 305.468 7

-1 -10 -9 10879.0 624.616 6

-1 -10 -9 8302.59 475.516 8

-1 -10 -10 2085.88 227.151 3

-1 -10 -10 1818.58 287.743 1

1 10 10 1773.14 243.391 10

-1 -10 -10 1978.83 153.233 9

-1 10 -10 2102.63 284.410 10

1 -10 10 1964.73 141.409 7

-1 -10 -10 1979.04 283.991 6

-1 -10 -11 494.305 120.827 3

-1 -10 -11 523.948 159.038 1

-1 10 -11 520.510 156.751 10

-1 -10 -11 485.268 81.5889 9

-1 -10 -11 450.345 137.627 6

-1 -10 -12 156.042 66.6667 3

-1 -10 -12 227.986 123.339 1

-1 10 -12 162.356 93.2319 10

-1 -10 -12 315.281 141.220 6

-1 -10 -13-31.5716 37.7018 3

-1 10 -13-62.7266 64.5833 10

-1 10 -13-149.388 132.048 6

-1 -10 -13-46.4357 82.2016 6

-1 -10 -14 14.1507 48.4606 3

-1 10 -14 35.0624 50.5927 10

-1 10 -15 98.5891 115.904 10

1 -11 -15-53.1195 57.1782 3

1 11 -15 112.031 85.2790 10

1 -11 -14-34.2024 45.8888 3

1 11 -14 79.1149 81.7589 10

1 11 -14 5.88216 65.5874 6

1 -11 -13 211.034 81.8529 3

1 11 -13 327.328 132.971 10

1 -11 -12 61.7396 57.5006 3

1 11 -12-42.8077 70.2684 10

1 -11 -12 41.3886 41.1880 9

1 -11 -11-0.53468 34.9759 3

1 -11 -11 113.718 90.1067 1

1 11 -11 25.5039 44.4834 10

1 -11 -11 19.6947 27.4709 9

-1 -11 11 26.2127 27.9355 7

1 -11 -10 59.5037 44.7980 3

1 -11 -10 17.0183 23.1520 5

1 -11 -10 65.1777 80.5637 1

1 -11 -10 22.7682 28.8815 9

1 11 -10-14.0206 40.9612 10

-1 -11 10 19.2768 34.0028 5

-1 -11 10 29.8181 22.2074 7

1 -11 -9 68.1353 61.5497 5

-1 -11 9 2.08939 73.4557 5

1 -11 -9 5.25283 38.7639 3

1 11 -9 131.971 88.4374 10

1 -11 -9 25.1807 26.6610 9

-1 11 9-25.2803 54.5984 10

-1 -11 9 55.7664 35.7991 8

-1 -11 9 40.8494 23.4638 7

1 -11 -8 1967.58 199.445 5

1 -11 -8 2157.87 215.108 3

1 -11 -8 2109.30 292.007 1

-1 -11 8 1780.66 75.6297 1

1 11 -8 2018.29 256.026 10

1 -11 -8 2090.32 168.082 9

-1 11 8 2066.15 253.047 10

-1 -11 8 1841.94 164.413 8

-1 -11 8 2313.78 260.106 5

-1 -11 8 1914.64 160.100 7

1 -11 -7 10516.5 461.438 3

-1 -11 7 10585.4 542.336 5

1 -11 -7 9292.42 437.342 5

1 -11 -7 10222.7 584.022 4

-1 -11 7 9783.26 214.107 1

1 -11 -7 9938.02 578.675 1

-1 11 7 10144.7 564.987 1

1 -11 -7 10498.7 375.326 9

-1 11 7 10873.1 544.562 10

1 11 -7 10579.5 549.085 10

1 -11 -7 9859.37 521.923 8

-1 -11 7 10293.8 361.954 7

-1 -11 7 9564.58 389.906 8

-1 11 7 11278.8 634.664 6

1 -11 -6 3640.46 258.739 3

1 -11 -6 4015.96 357.593 4

-1 -11 6 3617.85 306.578 5

1 -11 -6 3256.34 261.023 5

1 -11 -6 4584.25 385.020 1

-1 11 6 4397.51 375.183 1

-1 -11 6 3123.57 138.796 1

1 -11 -6 3563.07 213.459 9

-1 11 6 3540.33 301.962 10

1 11 -6 3801.42 313.662 10

1 -11 -6 3969.43 324.826 8

-1 11 6 3940.79 349.199 6

-1 -11 6 3225.36 196.689 7

1 -11 -5 11769.5 502.804 5

1 -11 -5 12239.7 462.370 3

1 -11 -5 13025.7 595.713 4

-1 -11 5 12276.6 555.825 5

-1 -11 5 12169.9 304.480 1

-1 11 5 12946.8 303.196 2

1 -11 -5 15208.9 665.503 1

-1 11 5 13213.6 553.096 10

-1 -11 5 12070.1 380.610 7

-1 11 5 13993.2 617.224 6

1 -11 -5 14230.6 608.531 8

-1 -11 4 522.486 133.172 5

1 -11 -4 453.702 99.8086 5

1 -11 -4 397.406 87.5191 3

-1 11 4 328.662 67.7794 2

1 -11 -4 546.440 137.320 1

-1 -11 4 452.178 68.0399 1

-1 11 4 360.572 75.1077 9

-1 11 4 377.999 98.2763 6

1 -11 -4 451.941 109.225 8

-1 -11 3 5576.92 394.712 5

1 -11 -3 5747.88 360.047 5

-1 -11 3 5601.69 246.907 1

1 -11 -3 4863.56 369.180 1

1 -11 -3 4759.89 338.544 8

1 11 -3 2985.84 196.391 7

1 11 -2 44.5500 29.6541 1

1 -11 -2 95.7003 56.0952 1

-1 11 2 83.0026 51.5532 1

-1 -11 2 79.4101 34.6766 1

1 11 -2 136.802 64.2283 3

-1 11 2 79.1308 38.4848 3

1 -11 -2 66.2317 51.6343 5

-1 -11 2 75.1766 62.5391 5

1 -11 -2 104.260 69.4675 8

1 11 -2 27.7109 24.3077 7

-1 11 2 83.0398 39.2021 9

1 11 -1 7076.01 309.022 1

1 -11 -1 10180.2 427.689 1

-1 -11 1 10003.6 371.205 1

-1 11 1 10036.6 403.557 1

1 -11 -1 9395.11 483.052 5

1 11 -1 5435.22 270.665 7

1 -11 -1 8437.37 444.334 8

1 11 0 56480.2 929.424 1

1 -11 0 61751.9 987.315 1

-1 -11 0 57587.0 943.060 1

-1 11 0 59036.2 898.493 1

-1 11 0 54923.7 966.634 3

1 -11 0 60882.8 1179.70 5

1 11 0 55041.5 945.323 3

1 -11 0 60063.4 1140.38 8

1 -11 1 8234.94 330.264 1

1 11 1 8049.40 368.280 1

-1 -11 -1 8048.82 379.921 1

-1 11 -1 8545.69 306.457 1

1 -11 1 8363.83 448.436 5

-1 -11 -1 9798.77 382.528 3

-1 11 -1 9475.41 403.857 3

1 11 1 9442.37 383.055 3

1 -11 1 8377.86 418.164 8

-1 -11 -2 338.174 113.457 1

-1 11 -2 214.988 63.3116 3

1 -11 2 306.185 69.0827 1

-1 11 -2 324.003 61.3142 1

1 -11 2 287.593 103.149 5

1 11 2 273.972 72.4629 3

-1 -11 -2 228.070 61.3879 3

1 -11 2 394.164 102.853 8

1 11 2 265.453 56.1323 9

-1 11 -2 170.992 41.6925 7

-1 -11 -3 9101.19 369.742 3

1 -11 3 11405.8 543.209 5

1 11 3 9348.37 391.368 3

-1 11 -3 9403.76 426.951 3

-1 11 -3 10213.9 270.084 1

1 -11 3 9506.72 289.092 1

-1 -11 -3 11353.3 535.720 1

1 11 3 8726.72 302.880 9

1 -11 3 10994.3 464.596 8

-1 11 -3 9411.01 301.027 7

1 -11 4 2403.53 258.731 5

-1 11 -4 2375.56 257.175 5

-1 -11 -4 2292.41 186.727 3

-1 -11 -4 2422.32 261.806 1

-1 11 -4 2402.38 218.667 10

1 11 4 2175.94 147.853 9

-1 -11 -4 2810.64 260.416 8

1 -11 4 2260.71 208.102 8

1 11 4 2416.38 247.746 6

1 -11 5 467.468 114.692 5

-1 -11 -5 593.838 101.805 3

-1 11 -5 1257.10 218.905 5

-1 -11 -5 974.468 215.458 1

1 11 5 432.221 93.5986 10

-1 11 -5 626.540 117.895 10

1 11 5 565.838 134.828 6

1 -11 5 431.273 100.693 8

-1 -11 -5 1018.89 194.595 8

-1 -11 -6 1017.31 130.183 3

-1 11 -6 866.637 166.298 5

1 11 6 1216.18 192.543 1

-1 -11 -6 1166.49 198.428 1

-1 11 -6 1112.14 167.590 10

1 11 6 1205.10 167.010 10

1 -11 6 1238.86 196.227 5

-1 -11 -6 998.111 166.661 8

1 11 6 1165.37 198.357 6

-1 -11 -7 203.915 107.088 4

1 -11 7 245.249 110.785 5

-1 -11 -7 208.333 68.3550 3

1 11 7 264.947 100.463 1

-1 -11 -7 364.416 142.632 1

-1 11 -7 188.127 76.6273 10

1 11 7 175.739 73.0722 10

-1 -11 -7 329.706 107.134 8

-1 -11 -8 1621.47 178.572 3

1 -11 8 1652.16 156.022 4

-1 -11 -8 1679.71 243.452 1

1 11 8 1525.80 215.590 10

-1 11 -8 1535.30 214.339 10

-1 -11 -8 1563.45 125.292 9

-1 -11 -8 1518.16 204.577 8

1 -11 8 1675.29 237.229 5

-1 -11 -9 1939.97 210.879 3

1 -11 9 1870.24 161.923 4

-1 -11 -9 1630.92 272.135 1

-1 -11 -9 1859.69 142.930 9

-1 11 -9 1902.79 255.417 10

1 11 9 1877.99 247.609 10

1 -11 9 1740.01 232.272 5

-1 -11 -9 1413.72 211.275 8

1 -11 9 1866.92 132.511 7

1 -11 10 28.8934 21.2831 4

-1 -11 -10 6.97448 36.0557 3

-1 -11 -10 35.7456 85.5933 1

1 11 10 55.9497 47.4431 10

-1 11 -10 50.7742 48.8349 10

1 -11 10 7.45855 25.1426 7

-1 -11 -10 75.3721 41.2519 9

-1 -11 -11 1008.90 168.394 3

-1 11 -11 960.428 198.290 10

-1 -11 -11 767.305 191.367 1

-1 -11 -12 34.8542 34.8628 3

-1 11 -12-70.0176 78.6398 10

-1 -11 -12 3.60964 66.5298 1

-1 -11 -13 53.7990 49.1243 3

-1 11 -13-41.6428 68.0199 10

-1 11 -14 260.243 132.747 10

-1 11 -15 161.502 113.596 10

1 -12 -15 164.923 89.1032 3

1 12 -15-124.635 94.5838 10

1 -12 -14 28.6721 45.5555 3

1 12 -14-90.2127 95.6614 10

1 -12 -13 15.5144 40.6293 3

1 12 -13 283.137 136.826 10

1 -12 -12 2.63931 38.4774 3

1 -12 -12 16.0691 44.2138 9

1 -12 -11 601.092 129.209 3

1 -12 -11 617.332 165.247 1

1 12 -11 560.588 147.844 10

1 -12 -11 552.239 101.275 9

1 -12 -10 193.967 89.9270 3

1 -12 -10 120.197 58.7221 5

1 -12 -10 2.92882 50.6307 1

1 12 -10 180.593 71.4488 10

-1 -12 10 191.780 90.7078 5

-1 -12 10 114.280 36.6758 7

1 -12 -10 142.110 47.4108 9

1 -12 -9 1190.04 170.244 3

1 -12 -9 1025.80 151.520 5

1 -12 -9 1129.87 242.771 1

1 -12 -9 1094.31 129.845 9

1 12 -9 1219.78 210.012 10

-1 -12 9 1176.34 132.204 7

-1 -12 9 1250.67 206.369 5

-1 -12 9 1247.45 132.530 8

1 -12 -8 1743.23 198.018 5

1 -12 -8 2193.82 222.982 3

1 -12 -8 2186.26 306.502 1

1 -12 -8 1946.45 169.521 9

1 12 -8 2030.04 256.876 10

-1 12 8 1939.96 248.700 10

-1 -12 8 2245.26 264.918 5

-1 -12 8 1899.44 177.215 8

-1 -12 8 1906.33 163.303 7

-1 -12 7 4730.67 377.643 5

1 -12 -7 4523.49 315.412 3

1 -12 -7 4104.59 372.173 4

1 -12 -7 4181.31 315.234 5

1 -12 -7 5464.30 453.406 1

-1 -12 7 3627.79 129.607 1

-1 12 7 4137.20 345.523 10

1 -12 -7 4357.84 250.673 9

1 12 -7 4802.48 381.403 10

-1 -12 7 4008.11 268.740 8

-1 -12 7 4215.25 236.505 7

1 -12 -7 4136.44 367.062 8

1 -12 -6 1072.48 141.758 3

-1 -12 6 1135.59 174.886 5

1 -12 -6 1123.27 156.548 5

1 -12 -6 1323.04 201.126 4

1 -12 -6 1194.57 203.755 1

-1 -12 6 1173.59 88.8129 1

-1 12 6 1047.34 190.138 1

1 12 -6 1245.77 176.725 10

-1 12 6 1321.44 185.029 10

-1 -12 6 1136.48 119.858 7

1 -12 -6 1190.59 187.327 8

-1 12 6 1405.53 211.683 6

-1 -12 5 1369.77 131.910 1

-1 12 5 2578.42 159.228 2

1 -12 -5 3767.81 358.671 1

1 -12 -5 1596.82 178.889 3

-1 -12 5 2611.09 334.630 5

1 -12 -5 1736.12 221.271 5

1 -12 -5 3162.60 304.026 8

-1 12 5 1442.72 218.395 6

1 -12 -4 12576.4 552.467 5

1 -12 -4 12959.0 472.566 3

-1 -12 4 12789.1 583.053 5

-1 12 4 11946.9 379.787 2

1 -12 -4 13614.5 614.850 1

-1 -12 4 12733.1 363.502 1

-1 12 4 12762.1 432.613 9

-1 12 4 13926.4 599.218 6

1 -12 -4 11761.3 566.040 8

1 -12 -3 1721.64 209.079 5

-1 12 3 1829.03 187.522 3

-1 -12 3 1679.72 213.510 5

1 -12 -3 1827.55 213.989 1

-1 -12 3 1758.63 142.054 1

-1 12 3 2215.12 180.107 9

1 -12 -3 1597.36 209.544 8

1 12 -3 2119.80 166.490 7

1 12 -2 25.2788 20.8375 3

1 12 -2-20.6443 31.9989 1

1 -12 -2 53.6523 44.9884 1

-1 12 2 4.28193 23.5268 1

-1 -12 2-30.5854 41.3762 1

-1 12 2-8.50059 18.0514 3

1 -12 -2 7.25808 23.9712 5

-1 -12 2 36.4764 49.8105 5

1 12 -2-34.1830 41.9714 7

-1 12 2-0.68718 27.6577 9

1 -12 -2 26.7435 30.7285 8

-1 12 1 9730.13 410.823 1

-1 -12 1 10248.5 391.683 1

1 12 -1 6988.39 321.112 1

1 -12 -1 9713.77 443.980 1

-1 -12 1 11013.2 536.427 5

1 -12 -1 10355.1 502.300 5

1 -12 -1 8306.90 462.547 8

-1 -12 0 463.664 89.7894 1

1 -12 0 629.141 115.188 1

1 12 0 528.936 101.477 1

-1 12 0 414.832 79.1591 1

1 12 0 477.062 94.7509 3

-1 12 0 471.992 97.1569 3

1 -12 0 420.155 112.122 5

1 -12 0 577.537 125.058 8

-1 -12 -1 10228.4 449.104 1

1 12 1 10810.6 443.540 1

1 -12 1 10932.8 408.244 1

-1 12 -1 10269.0 354.794 1

-1 -12 -1 9975.13 403.388 3

1 -12 1 11069.2 545.378 5

-1 12 -1 10193.8 442.796 3

1 12 1 10506.5 424.666 3

1 -12 1 11086.1 507.644 8

-1 12 -2 895.070 96.4098 1

-1 -12 -2 977.474 152.007 1

1 -12 2 928.792 110.165 1

-1 12 -2 1020.14 141.862 3

-1 -12 -2 849.068 114.154 3

1 12 2 950.714 128.001 3

1 -12 2 965.898 159.264 5

1 12 2 890.728 105.106 9

1 -12 2 932.505 146.152 8

-1 12 -2 1030.72 109.724 7

1 12 3 21857.9 627.377 3

-1 12 -3 21518.7 662.659 3

-1 -12 -3 21449.4 595.473 3

1 -12 3 20877.0 762.071 5

1 -12 3 19724.8 449.534 1

-1 -12 -3 18764.7 668.685 1

-1 12 -3 20515.4 412.425 1

1 12 3 20108.8 490.014 9

1 -12 3 20429.5 658.109 8

-1 12 -3 20668.4 473.031 7

1 -12 4 4292.08 356.056 5

-1 -12 -4 4556.91 275.580 3

1 -12 4 4107.78 161.289 1

-1 -12 -4 4025.31 339.512 1

-1 12 -4 4483.11 304.854 10

1 12 4 4205.10 218.731 9

1 12 4 4322.36 343.810 6

1 -12 4 3964.97 287.232 8

-1 -12 -4 4217.18 329.091 8

-1 -12 -5 1312.22 148.724 3

1 -12 5 1271.16 195.705 5

1 12 5 1305.47 195.311 1

-1 -12 -5 1145.23 194.719 1

-1 12 -5 1511.19 188.712 10

1 -12 5 1414.49 165.753 8

1 12 5 1435.34 217.922 6

-1 -12 -5 1094.54 173.650 8

1 -12 6 84.3233 74.0809 5

-1 -12 -6 59.0134 45.9647 3

1 12 6 84.0203 65.1738 1

-1 -12 -6-10.7725 61.4774 1

1 12 6 45.5855 23.8240 10

-1 12 -6 85.0471 57.6920 10

-1 -12 -6 21.1067 32.5816 8

1 -12 6 64.8167 46.0448 8

1 12 6 70.4884 45.9058 6

-1 -12 -7 262.914 76.3358 3

1 -12 7 288.712 97.6967 5

-1 -12 -7 204.360 114.394 1

-1 12 -7 229.276 87.9911 10

1 12 7 108.521 55.2624 10

-1 -12 -7 267.647 96.5279 8

-1 -12 -8 112.595 66.8798 3

-1 -12 -8-5.12685 85.6011 1

1 12 8 4.87345 37.8244 10

-1 12 -8 81.6895 67.9683 10

-1 -12 -8-11.3587 52.1402 8

1 -12 8 164.977 81.5135 5

1 -12 9 437.517 82.4957 4

-1 -12 -9 392.684 102.111 3

-1 -12 -9 320.790 110.983 1

-1 12 -9 322.166 113.113 10

1 12 9 417.667 120.928 10

1 -12 9 491.369 136.063 5

-1 -12 -9 274.994 92.9317 8

-1 -12 -10 310.684 91.2575 3

1 -12 10 258.252 57.9926 4

-1 -12 -10 320.040 116.451 1

-1 12 -10 319.638 121.083 10

-1 -12 -10 252.901 48.1352 9

-1 -12 -11 328.648 107.858 3

-1 12 -11 344.124 119.566 10

-1 -12 -11 326.343 118.225 1

-1 -12 -12 30.1761 43.1426 3

-1 12 -12 50.0821 76.0592 10

-1 -12 -12-138.756 127.765 1

-1 -12 -13 57.6253 51.2765 3

-1 12 -13-81.5598 75.1519 10

-1 12 -14-214.367 114.989 10

1 -13 -15 78.3158 51.5392 3

1 -13 -14 9.68358 56.8099 3

1 13 -14-97.8414 88.6904 10

1 -13 -13 117.005 81.4760 3

1 13 -13-137.966 95.6257 10

1 -13 -12-95.3621 89.4181 3

1 13 -12 162.458 118.622 10

1 -13 -11 97.3324 67.6466 3

1 -13 -11-58.0343 79.8392 1

1 13 -11-1.04199 74.4993 10

1 -13 -11 8.38444 22.2300 9

1 -13 -10-22.4975 33.9024 5

-1 -13 10-8.86898 28.3287 5

1 -13 -10-73.1920 52.8759 3

1 -13 -10-80.7890 90.0287 1

1 13 -10 32.7500 58.6021 10

1 -13 -10 56.1012 36.8119 9

-1 -13 10 27.6840 30.0065 7

-1 -13 9-2.62667 32.4813 5

1 -13 -9 20.8677 41.1934 3

1 -13 -9-8.83892 26.1300 5

1 -13 -9 51.8276 69.6174 1

1 13 -9-76.3347 87.5951 10

-1 -13 9-33.6342 32.7084 7

1 -13 -9 12.7343 33.6895 9

-1 -13 9 3.22828 23.0305 8

1 -13 -8 1903.80 211.733 5

1 -13 -8 2368.72 237.735 3

1 -13 -8 2348.40 313.330 1

1 -13 -8 2305.57 188.153 9

-1 13 8 2255.70 275.877 10

1 13 -8 2050.23 265.176 10

-1 -13 8 1982.92 167.028 7

-1 -13 8 2160.54 275.072 5

-1 -13 8 1939.45 191.153 8

1 -13 -7 1060.97 161.492 5

-1 -13 7 1463.82 213.419 5

1 -13 -7 1177.57 157.792 3

1 -13 -7 1342.03 237.651 1

-1 -13 7 1255.54 77.5044 1

1 13 -7 1373.05 209.383 10

-1 13 7 1506.30 215.387 10

-1 -13 7 1345.22 166.270 8

1 -13 -7 1328.15 204.398 8

1 -13 -6 2723.78 270.805 5

1 -13 -6 2993.86 251.277 3

-1 -13 6 3388.71 327.818 5

1 -13 -6 2774.65 302.657 4

1 -13 -6 2872.05 323.811 1

-1 -13 6 2609.08 142.222 1

-1 13 6 2992.20 319.910 1

1 13 -6 3153.24 299.857 10

-1 13 6 3184.54 303.372 10

1 -13 -6 3191.56 321.698 8

-1 -13 6 2850.18 245.832 8

-1 13 6 2919.82 317.821 6

1 -13 -5 1111.83 153.748 3

1 -13 -5 1119.10 189.325 5

-1 -13 5 1605.54 265.674 5

-1 13 5 1093.23 181.406 1

-1 -13 5 744.363 90.7509 1

-1 13 5 1473.46 132.350 2

1 -13 -5 1909.61 266.591 1

-1 13 5 874.752 121.209 9

1 13 -5 1039.69 173.211 10

-1 13 5 974.567 182.358 6

1 -13 -5 1586.86 224.757 8

1 -13 -4 151.036 111.124 5

1 -13 -4 94.9885 59.6875 3

1 -13 -4 223.016 108.038 1

-1 -13 4 191.580 71.7780 1

-1 13 4 4.32157 30.4716 9

1 -13 -4 134.699 79.2449 8

-1 -13 3 19709.5 7883.62 5

1 -13 -3 12769.1 6271.10 5

-1 -13 3 18444.3 5141.17 1

1 -13 -3 18196.4 7253.29 1

1 -13 -3 17663.9 7057.75 8

-1 -13 2 766.080 152.597 5

-1 13 2 782.041 124.455 3

1 -13 -2 812.282 155.131 5

1 -13 -2 720.310 151.164 1

-1 13 2 658.387 123.590 1

-1 -13 2 743.739 107.255 1

1 13 -2 680.598 102.745 1

1 13 -2 707.749 123.440 3

-1 13 2 775.886 113.743 9

1 -13 -2 702.161 138.910 8

1 13 -2 748.205 110.249 7

1 13 -1 1876.09 174.334 1

-1 -13 1 2479.45 203.690 1

-1 13 1 2457.99 217.452 1

1 -13 -1 2412.74 238.814 1

1 13 -1 1259.48 159.011 3

1 -13 -1 2317.69 261.823 5

-1 13 1 1169.30 153.880 3

-1 -13 1 2615.17 278.509 5

1 13 -1 1596.43 163.788 7

1 -13 -1 2285.50 245.999 8

1 -13 0 6904.59 364.054 1

-1 -13 0 6681.20 352.457 1

1 13 0 6581.53 343.776 1

-1 13 0 6746.62 325.655 1

1 -13 0 6948.39 434.547 5

-1 13 0 6278.47 354.744 3

1 13 0 6304.17 351.199 3

1 -13 0 6727.87 413.264 8

-1 13 -1 3995.20 231.954 1

1 13 1 4029.19 286.008 1

1 -13 1 4479.50 272.374 1

-1 -13 -1 4326.40 302.664 1

-1 -13 -1 3282.62 242.196 3

1 13 1 3382.26 251.710 3

1 -13 1 4256.70 349.714 5

-1 13 -1 3359.76 262.353 3

1 -13 1 4027.33 325.761 8

-1 -13 -2 475.047 108.247 1

1 -13 2 521.185 89.3723 1

-1 13 -2 509.034 74.7772 1

1 13 2 598.273 113.947 1

-1 -13 -2 521.761 93.1959 3

1 -13 2 423.126 114.541 5

-1 13 -2 706.595 120.328 3

1 13 2 497.335 99.1845 3

1 -13 2 566.330 113.026 8

-1 13 -2 611.114 85.6102 7

1 13 2 522.604 87.4541 9

-1 -13 -3 418.018 112.501 1

-1 13 -3 123.665 47.3332 3

-1 13 -3 270.789 49.6422 1

1 -13 3 176.361 59.5371 1

-1 -13 -3 163.234 51.9905 3

1 13 3 245.634 66.8177 3

1 -13 3 253.089 110.303 5

-1 -13 -3 366.157 113.331 8

1 -13 3 291.801 88.1780 8

-1 13 -3 223.065 56.4505 7

1 13 3 145.113 40.8095 9

-1 13 -4 1031.45 157.187 3

-1 -13 -4 1033.17 139.388 3

1 -13 4 1135.73 194.030 5

1 -13 4 867.140 83.5529 1

-1 -13 -4 1041.97 182.440 1

-1 13 -4 1069.16 161.098 10

1 13 4 951.360 108.178 9

1 13 4 1056.58 179.688 6

-1 -13 -4 814.989 157.943 8

1 -13 4 936.944 148.799 8

1 -13 5 1307.55 211.278 5

-1 -13 -5 1023.82 140.488 3

-1 -13 -5 1204.51 199.602 1

1 13 5 1064.36 185.536 1

-1 13 -5 1138.22 173.059 10

1 13 5 1131.60 189.237 6

1 -13 5 901.719 148.182 8

-1 -13 -5 1365.37 206.207 8

-1 -13 -6 831.435 130.429 3

1 -13 6 944.983 171.728 5

-1 -13 -6 665.140 156.948 1

1 13 6 760.068 59.2337 2

-1 13 -6 810.106 150.174 10

1 13 6 802.093 144.553 10

1 -13 6 781.012 121.918 8

-1 -13 -6 682.716 141.619 8

-1 -13 -7 688.458 119.171 3

1 -13 7 741.650 165.661 5

-1 -13 -7 773.305 177.789 1

1 13 7 703.596 137.571 10

-1 13 -7 733.489 148.093 10

-1 -13 -7 611.114 144.438 8

-1 -13 -8 86.0511 47.9541 3

1 -13 8 45.2313 57.8950 5

-1 -13 -8 73.4346 69.9132 1

1 13 8 60.0638 30.7466 10

-1 13 -8 121.649 77.3332 10

-1 -13 -8 112.964 69.7479 8

1 -13 9 486.169 83.5627 4

-1 -13 -9 456.528 101.752 3

-1 -13 -9 342.398 131.080 1

1 13 9 387.638 116.204 10

-1 13 -9 400.965 124.135 10

-1 -13 -9 385.127 120.561 8

1 -13 9 505.906 141.034 5

-1 -13 -10-2.24635 46.7833 3

1 -13 10 76.4776 39.8851 4

-1 -13 -10-90.5739 83.9887 1

-1 13 -10-40.5044 68.9484 10

1 -13 10-32.4526 69.8630 5

-1 -13 -11 674.936 142.207 3

-1 13 -11 734.512 178.119 10

-1 -13 -11 427.096 149.194 1

-1 -13 -12 116.469 67.2375 3

-1 13 -12-39.9238 64.9398 10

-1 -13 -12-78.5579 72.0276 1

-1 -13 -13-49.2965 37.2210 3

-1 -13 -13-52.0506 67.7088 1

-1 13 -14-17.8399 44.9276 10

1 -14 -14-71.2833 49.4758 3

1 14 -14 20.6169 46.3937 10

1 -14 -13 98.6792 73.0431 3

1 14 -13 81.5591 86.7603 10

1 -14 -12 58.6643 39.4712 3

1 14 -12 98.9249 66.4999 10

1 -14 -11 196.306 87.4053 3

1 -14 -11 376.982 153.559 1

1 14 -11 258.723 100.588 10

1 -14 -11 349.924 81.5685 9

-1 -14 10 636.723 155.263 5

1 -14 -10 820.997 152.131 3

1 -14 -10 509.603 162.059 1

1 -14 -10 834.577 121.558 9

1 14 -10 787.847 188.064 10

-1 -14 10 743.634 110.446 7

-1 -14 9 743.469 172.066 5

1 -14 -9 799.388 146.948 3

1 -14 -9 694.395 131.492 5

1 -14 -9 719.423 185.890 1

1 14 -9 678.003 169.276 10

-1 -14 9 662.724 114.647 8

1 -14 -8 1933.46 216.284 3

-1 -14 8 2035.72 279.576 5

1 -14 -8 1761.26 212.007 5

1 -14 -8 1398.99 258.986 1

1 14 -8 2121.41 275.810 10

1 -14 -8 1561.26 239.858 8

-1 -14 8 1791.39 198.181 8

1 -14 -7 53.1980 53.9884 3

1 -14 -7 90.7822 57.3332 5

-1 -14 7 27.5518 47.7647 5

-1 -14 7 27.7732 16.9471 1

1 -14 -7-1.24768 91.9937 1

-1 14 7-7.91554 42.5038 10

1 14 -7 75.9174 56.5565 10

1 -14 -7 78.1278 71.1238 8

-1 -14 7 18.1126 28.6276 8

-1 -14 6 190.064 85.6682 5

1 -14 -6 152.429 54.7061 3

1 -14 -6 212.355 84.1716 5

1 -14 -6 53.3426 58.5494 1

-1 14 6 153.331 36.0127 2

-1 -14 6 244.053 44.1685 1

-1 14 6 205.393 84.7135 10

1 14 -6 183.532 75.9372 10

-1 -14 6 150.094 63.1770 8

1 -14 -6 219.786 83.4517 8

1 -14 -5 6177.26 356.765 3

1 -14 -5 5938.45 411.174 5

-1 -14 5 6636.93 474.509 5

-1 -14 5 5430.14 244.317 1

1 -14 -5 6557.37 472.404 1

-1 14 5 5799.70 265.920 2

-1 14 5 6567.48 465.547 1

1 14 -5 6243.23 406.498 10

-1 14 5 5591.65 315.544 9

1 -14 -5 5862.08 437.444 8

-1 14 5 6035.58 438.235 6

-1 14 4 5192.16 345.876 3

-1 -14 4 4574.59 381.499 5

1 -14 -4 4716.11 310.527 3

1 -14 -4 4736.55 371.682 5

-1 14 4 5061.09 370.358 1

-1 -14 4 4472.57 239.118 1

1 -14 -4 4412.39 373.552 1

-1 14 4 4711.28 287.803 9

1 -14 -4 4107.20 362.102 8

-1 14 3 1051.76 151.654 3

-1 -14 3 968.197 181.564 5

1 14 -3 1019.97 151.618 3

1 -14 -3 1130.88 186.054 5

1 -14 -3 906.373 136.530 3

-1 -14 3 1015.00 120.557 1

-1 14 3 1097.42 177.581 1

1 -14 -3 810.724 165.979 1

-1 14 3 949.400 130.475 9

1 14 -3 885.202 115.863 7

1 -14 -3 1026.67 178.176 8

1 -14 -2-45.0377 49.8199 1

-1 14 2 45.9772 38.9996 1

1 14 -2 45.0249 38.2617 1

1 14 -2 44.8708 38.5441 3

-1 -14 2 8.68026 36.2210 1

-1 -14 2 18.0000 50.0756 5

1 -14 -2 3.26323 37.3540 5

-1 14 2 61.5434 50.6111 3

1 14 -2 38.1770 28.4585 7

1 -14 -2 35.3456 32.1701 8

-1 14 2 38.8768 31.6719 9

-1 -14 1 2455.49 215.943 1

1 -14 -1 2439.01 235.586 1

-1 14 1 1986.59 211.282 1

1 14 -1 1632.70 169.480 1

1 14 -1 996.957 148.812 3

-1 -14 1 2474.74 285.075 5

1 -14 -1 2587.45 273.708 5

-1 14 1 1112.99 157.985 3

1 -14 -1 1900.65 238.319 8

1 14 -1 1426.35 158.954 7

-1 14 0-22.1094 20.6990 1

-1 -14 0-8.66121 43.1547 1

1 -14 0-3.65477 30.5419 1

1 14 0 9.85200 30.0553 1

-1 -14 0-65.2524 43.8822 5

-1 14 0-14.9266 46.8342 3

1 14 0 6.81901 23.0763 3

1 -14 0-14.9761 56.1935 5

1 -14 0 7.80829 50.9594 8

-1 -14 -1 3282.43 276.129 1

1 -14 1 3443.00 260.742 1

-1 14 -1 3158.37 215.489 1

1 14 1 3150.43 266.324 1

-1 14 -1 2348.06 229.659 3

1 -14 1 3342.18 325.142 5

-1 -14 -1 2478.77 221.105 3

1 14 1 2506.21 227.637 3

1 -14 1 3300.41 306.426 8

1 14 2 2181.98 227.387 1

-1 -14 -2 1972.04 226.766 1

1 -14 2 2053.76 187.751 1

-1 14 -2 1871.83 153.335 1

-1 -14 -2 1665.12 181.943 3

1 14 2 1477.94 175.485 3

-1 14 -2 1753.64 200.208 3

1 -14 2 2116.27 259.353 5

1 14 2 1569.40 159.985 9

-1 14 -2 1714.96 155.462 7

1 -14 2 1827.45 229.896 8

1 -14 3 13688.8 662.907 5

-1 -14 -3 13298.7 513.636 3

1 14 3 13970.9 553.270 3

-1 14 -3 14165.4 579.761 3

-1 -14 -3 13085.3 603.589 1

-1 14 -3 13359.1 384.037 1

1 -14 3 12964.5 420.012 1

1 14 3 14449.2 618.648 1

1 14 3 13292.0 440.051 9

-1 -14 -3 12952.3 614.581 8

-1 14 -3 13932.1 431.507 7

1 -14 3 12847.6 578.693 8

1 -14 4 3474.99 341.191 5

-1 -14 -4 3315.88 259.853 3

-1 14 -4 3466.61 295.010 3

1 -14 4 2950.29 168.054 1

-1 -14 -4 3168.36 312.822 1

1 14 4 3913.38 336.803 1

-1 14 -4 3437.76 286.085 10

1 14 4 3048.82 205.941 9

1 14 4 3134.03 310.436 6

1 -14 4 3050.08 278.601 8

-1 -14 -4 3329.90 328.733 8

1 -14 5 3167.74 340.888 5

-1 -14 -5 3393.53 259.997 3

-1 14 -5 3472.18 298.677 3

1 14 5 3084.53 320.846 1

-1 -14 -5 2741.49 307.704 1

-1 14 -5 3273.41 292.980 10

1 14 5 3142.97 330.131 6

-1 -14 -5 2825.11 299.436 8

1 -14 5 2674.59 253.626 8

-1 -14 -6 376.305 89.1775 3

1 -14 6 529.253 149.486 5

1 14 6 569.641 62.5987 2

-1 -14 -6 399.509 134.886 1

-1 14 -6 456.134 107.137 10

1 14 6 343.596 105.250 10

1 -14 6 365.626 97.8379 8

-1 -14 -6 452.606 124.569 8

1 -14 7 418.681 124.798 5

-1 -14 -7 317.305 87.2174 3

-1 -14 -7 398.989 137.356 1

-1 14 -7 332.835 102.570 10

1 14 7 410.323 114.938 10

-1 -14 -7 313.405 105.305 8

-1 -14 -8-36.4423 52.4148 3

1 -14 8-93.3840 78.7853 5

-1 -14 -8 38.5139 75.6209 1

-1 14 -8 27.8469 45.7132 10

1 14 8-5.19348 33.6125 10

-1 -14 -8 44.5207 69.3960 8

-1 -14 -9 55.6877 42.1588 3

1 -14 9 50.8447 32.0848 4

-1 14 -9 54.6740 60.9462 10

1 -14 9-20.4157 52.3983 5

-1 -14 -10-28.9727 33.5805 3

-1 14 -10 87.4594 74.4045 10

-1 -14 -10-45.3989 72.9523 1

-1 -14 -11-28.4193 55.2453 3

-1 14 -11-130.066 119.301 10

-1 -14 -11 120.854 100.561 1

-1 -14 -12 53.4831 59.9349 3

-1 -14 -12 122.915 112.487 1

-1 -14 -13-22.4199 56.4357 3

-1 14 -13-122.257 89.5158 10

-1 -14 -13-39.3174 71.8907 1

-1 14 -14 38.1566 83.8365 10

1 -15 -14 107.859 83.9982 3

1 15 -14-277.886 137.960 10

1 -15 -13 133.893 70.6475 3

1 15 -13 0.52657 45.7259 10

1 -15 -12 228.997 94.0631 3

1 15 -12 146.244 66.3754 10

1 -15 -11 25.8950 39.7908 3

1 15 -11 73.2964 63.4908 10

1 -15 -11 159.635 125.012 1

-1 -15 10 13.7513 52.8551 5

1 -15 -10 70.4255 49.3578 3

1 -15 -10 26.4548 99.0142 1

1 15 -10 34.1151 45.6349 10

1 -15 -9 759.883 146.528 3

1 -15 -9 617.059 133.168 5

-1 -15 9 671.805 170.804 5

1 -15 -9 702.988 189.431 1

1 15 -9 812.627 178.806 10

-1 -15 9 654.905 126.342 8

-1 -15 8 2617.97 321.136 5

1 -15 -8 2011.10 236.070 5

1 -15 -8 2532.04 255.851 3

1 -15 -8 2075.83 310.193 1

1 15 -8 2956.86 333.883 10

-1 -15 8 2266.12 235.373 8

-1 -15 7 1425.02 227.891 5

1 -15 -7 1131.09 187.325 5

1 -15 -7 1390.69 181.731 3

1 -15 -7 1591.45 259.439 1

-1 -15 7 1419.63 93.0868 1

-1 15 7 1448.75 221.770 10

1 15 -7 1696.52 244.468 10

-1 -15 7 1085.75 165.220 8

-1 -15 6 2062.51 271.910 5

1 -15 -6 2350.58 231.991 3

1 -15 -6 1938.62 246.664 5

1 -15 -6 2416.76 317.454 1

-1 15 6 2006.07 141.107 2

-1 -15 6 2079.99 142.481 1

-1 15 6 2201.37 266.609 10

1 15 -6 2322.92 267.131 10

-1 -15 6 1986.38 224.260 8

1 -15 -6 2230.66 278.159 8

1 -15 -5 4489.76 373.146 5

1 -15 -5 4645.20 322.448 3

-1 -15 5 4756.26 406.965 5

-1 -15 5 4516.21 235.703 1

-1 15 5 4180.55 243.359 2

-1 15 5 4625.33 394.262 1

1 -15 -5 4444.03 414.165 1

-1 15 5 4310.08 284.101 9

1 15 -5 4559.59 361.175 10

-1 15 5 4794.78 402.590 6

1 -15 -5 4372.85 389.367 8

-1 -15 5 4126.42 336.298 8

1 -15 -4 81.4460 88.2273 5

-1 15 4-5.04829 30.1724 3

1 -15 -4 2.45471 37.5318 3

-1 -15 4 141.172 109.596 5

-1 15 4 135.664 110.166 1

-1 -15 4 186.997 64.9522 1

1 15 -4 67.3891 65.8732 3

1 -15 -4 58.9999 50.0295 1

-1 15 4-3.55936 28.7764 9

1 15 -4 17.9083 54.6636 10

1 -15 -4 118.708 84.2590 8

1 -15 -3 2506.94 258.015 3

-1 -15 3 10181.8 573.423 5

1 -15 -3 7606.26 570.217 5

1 15 -3 2479.72 262.978 3

-1 15 3 8313.28 479.560 1

-1 -15 3 9716.69 408.398 1

1 -15 -3 7718.40 553.379 1

1 15 -3 2585.29 231.110 7

1 -15 -3 5113.87 496.824 8

-1 -15 2 25.7699 48.6517 1

-1 15 2 16.1091 32.9180 1

1 15 -2 20.7079 28.0920 1

1 -15 -2 71.1782 51.2136 1

1 -15 -2 37.7050 45.3117 3

1 15 -2 22.5152 29.2337 3

-1 15 2 48.4591 50.7951 3

-1 -15 2 22.9328 34.1217 5

1 -15 -2-7.42583 45.3798 5

1 -15 -2 2.97909 44.9222 8

-1 15 2 26.8232 23.3832 9

1 15 -2 33.8331 32.9491 7

1 -15 -1 2699.81 262.974 1

-1 15 1 2508.95 229.027 1

1 15 -1 2453.50 215.666 1

-1 -15 1 2323.98 218.539 1

1 -15 -1 2516.70 286.497 5

-1 15 1 2933.64 257.225 3

-1 -15 1 2589.55 287.826 5

1 15 -1 2836.57 256.425 3

1 -15 -1 2584.68 290.680 8

1 15 -1 2698.53 228.102 7

1 -15 0-5.75669 35.1982 1

-1 15 0-6.41061 29.5909 1

1 15 0-26.0898 37.1771 1

-1 -15 0-7.73721 35.4827 1

-1 15 0-75.8521 56.6445 3

1 15 0-37.0298 39.8476 3

1 -15 0 24.1290 39.2869 5

-1 -15 0-21.3872 44.7840 5

1 -15 0-51.7929 56.8869 8

-1 15 -1 6830.58 334.974 1

1 -15 1 7587.33 392.490 1

1 15 1 7322.54 412.451 1

-1 -15 -1 7104.88 419.186 1

-1 15 -1 8977.39 461.486 3

-1 -15 -1 8137.72 408.063 3

1 -15 1 7632.04 499.066 5

1 15 1 8476.87 429.213 3

1 -15 1 7481.45 473.845 8

1 -15 2 782.362 122.648 1

-1 15 -2 957.161 115.875 1

1 15 2 980.823 156.656 1

-1 -15 -2 839.027 131.837 3

-1 -15 -2 847.834 159.499 1

1 -15 2 894.292 176.792 5

1 15 2 854.513 141.160 3

-1 15 -2 932.221 153.342 3

1 -15 2 894.956 161.887 8

-1 -15 -2 937.098 168.625 8

-1 15 -2 851.007 116.675 7

1 15 2 937.251 127.089 9

1 15 3 2837.00 256.341 3

1 -15 3 2537.52 296.293 5

-1 15 -3 2769.00 263.859 3

-1 -15 -3 2596.70 233.074 3

-1 15 -3 2623.97 178.781 1

1 -15 3 2682.59 198.496 1

1 15 3 2531.75 267.386 1

-1 -15 -3 2412.94 264.591 1

1 15 3 2630.08 205.786 9

-1 15 -3 2759.78 200.563 7

1 -15 3 2435.29 259.093 8

-1 -15 -3 2354.48 277.694 8

1 -15 4 810.086 176.411 5

-1 -15 -4 758.249 128.813 3

1 15 4 694.621 132.314 3

1 15 4 980.838 185.909 1

-1 -15 -4 822.939 160.900 1

-1 15 -4 748.864 146.513 3

1 -15 4 691.083 92.0584 1

1 15 4 744.505 106.025 9

1 -15 4 745.310 149.458 8

1 15 4 879.905 173.692 6

-1 -15 -4 845.947 168.093 8

1 -15 5 3773.74 380.575 5

-1 -15 -5 3491.84 273.189 3

-1 -15 -5 3569.16 355.613 1

-1 15 -5 3651.11 314.818 10

-1 -15 -5 3269.19 336.576 8

1 -15 5 3163.36 291.210 8

-1 -15 -6 1563.48 188.112 3

1 15 6 1657.62 120.861 2

-1 -15 -6 1379.06 232.633 1

-1 15 -6 1543.18 218.776 10

1 15 6 1418.69 204.772 10

-1 -15 -6 1458.60 221.840 8

1 -15 6 1343.81 184.557 8

1 -15 6 1676.51 254.777 5

1 -15 7 477.339 144.054 5

-1 -15 -7 266.893 84.9823 3

1 15 7 249.700 99.3807 10

-1 15 -7 159.799 87.0535 10

-1 -15 -7 379.716 174.998 1

-1 -15 -8 3.19002 35.2579 3

1 15 8 177.507 85.6960 10

-1 15 -8 165.900 81.0402 10

-1 -15 -8-70.6740 95.4265 1

-1 -15 -9 9.08770 40.5504 3

-1 15 -9-33.1607 82.9589 10

-1 -15 -9 36.3356 53.4608 1

-1 -15 -10 204.165 87.6341 3

-1 15 -10 22.4806 60.0746 10

-1 -15 -10 8.45676 61.1513 1

-1 -15 -11 81.5262 50.5901 3

-1 15 -11 58.2509 100.554 10

-1 -15 -11 141.142 111.125 1

-1 -15 -12 137.859 73.7139 3

-1 15 -12 67.5715 55.6581 10

-1 -15 -12 181.698 109.553 1

-1 -15 -13 63.2807 61.8063 3

-1 15 -13-131.220 96.6462 10

-1 -15 -13 141.580 103.583 1

-1 15 -14 72.3370 86.2233 10

1 -16 -14-42.7978 58.9321 3

1 -16 -13 233.861 100.170 3

1 16 -13 90.9818 100.965 10

1 -16 -12 210.929 95.1644 3

1 16 -12 55.2934 45.7599 10

1 -16 -11-6.37817 39.6696 3

1 16 -11 124.948 102.868 10

1 -16 -11-6.78525 70.3421 1

1 -16 -10 422.283 113.284 3

1 -16 -10 498.362 165.596 1

1 16 -10 341.484 127.189 10

1 -16 -9 828.519 149.577 3

1 -16 -9 652.803 139.509 5

1 -16 -9 806.488 216.647 1

1 16 -9 772.103 181.208 10

-1 -16 9 625.941 124.207 8

1 -16 -8 663.775 139.689 5

1 -16 -8 970.315 158.551 3

1 -16 -8 980.185 218.790 1

1 16 -8 815.777 169.651 10

-1 -16 8 777.647 143.405 8

1 -16 -7 341.320 89.5061 3

-1 -16 7 410.953 138.412 5

1 -16 -7 379.885 114.054 5

-1 -16 7 306.538 46.6425 1

1 -16 -7 417.652 153.162 1

1 16 -7 421.237 118.717 10

-1 -16 7 280.831 92.1585 8

1 -16 -6 457.226 120.416 5

-1 -16 6 385.284 121.660 5

1 -16 -6 534.701 111.697 3

1 -16 -6 407.117 128.671 1

-1 -16 6 396.119 71.2626 1

-1 16 6 466.181 72.5730 2

1 16 -6 292.307 94.9955 10

-1 -16 6 436.296 115.850 8

1 -16 -5 410.504 103.013 3

-1 -16 5 470.121 130.263 5

1 -16 -5 466.095 129.381 5

-1 -16 5 414.739 75.1937 1

-1 16 5 410.248 82.1727 2

1 -16 -5 396.856 151.638 1

1 16 -5 401.152 119.667 10

-1 16 5 400.154 95.1731 9

-1 -16 5 299.327 97.7904 8

1 -16 -4 1777.98 205.050 3

-1 -16 4 1649.93 242.975 5

1 16 -4 1597.44 206.245 3

1 -16 -4 1506.79 227.382 5

-1 16 4 1719.57 218.597 3

-1 -16 4 1685.51 169.258 1

1 -16 -4 1716.65 250.346 1

-1 16 4 1949.14 248.233 1

1 16 -4 1498.87 209.607 10

-1 16 4 1469.61 171.347 9

-1 -16 4 1661.09 222.771 8

1 16 -4 1589.62 174.949 7

1 -16 -4 1661.53 255.026 8

1 -16 -3 260.713 77.4842 1

-1 16 3 260.884 112.874 1

-1 -16 3 161.097 52.7671 1

1 16 -3 77.4808 56.0715 3

-1 -16 3 238.700 114.825 5

-1 16 3 2.09497 49.5468 3

1 -16 -3 45.0565 45.1709 3

1 -16 -3 274.260 110.526 5

-1 16 3-49.0761 46.5635 9

1 16 -3-5.18505 66.5024 7

1 -16 -3 306.331 101.891 8

1 16 -2 372.046 91.4847 1

-1 -16 2 459.466 101.917 1

1 -16 -2 589.612 142.473 1

-1 16 2 485.162 114.807 1

-1 -16 2 466.578 134.524 5

1 -16 -2 569.529 144.054 5

1 -16 -2 424.406 104.974 3

-1 16 2 372.982 91.4845 3

1 16 -2 456.939 114.223 3

1 -16 -2 548.117 151.727 8

-1 16 2 375.051 94.0853 9

1 16 -2 493.666 105.868 7

-1 -16 1 12417.6 5424.76 1

1 -16 -1 11515.6 5646.06 1

-1 16 1 10498.6 4966.92 1

1 16 -1 7044.92 3866.24 1

-1 -16 1 13292.6 6948.37 5

1 -16 -1 11459.3 6339.02 5

1 -16 -1 9079.14 5669.13 8

-1 16 0 189.424 64.1917 1

1 16 0 270.948 80.8003 1

-1 -16 0 339.180 94.8899 1

1 -16 0 198.684 79.0227 1

1 16 0 280.386 78.4339 3

1 -16 0 231.322 92.7383 5

-1 16 0 259.416 83.1317 3

-1 -16 0 347.882 109.989 5

1 -16 0 349.910 112.044 8

1 -16 1 6442.82 377.877 1

-1 -16 -1 6528.29 424.469 1

1 16 1 5882.95 387.135 1

-1 16 -1 5514.55 315.024 1

-1 -16 -1 4216.57 308.280 3

-1 -16 -1 7368.99 504.848 5

-1 16 -1 4330.56 327.393 3

1 -16 1 5801.67 456.070 5

1 16 1 4457.16 329.276 3

1 -16 1 6056.16 443.562 8

-1 -16 -1 6591.06 462.407 8

1 16 2 2707.45 271.829 1

1 -16 2 2756.93 238.734 1

-1 -16 -2 2477.01 280.607 1

-1 16 -2 2626.52 202.469 1

-1 -16 -2 2395.34 226.288 3

1 16 2 2792.71 258.353 3

-1 16 -2 2467.15 251.844 3

1 -16 2 2935.49 319.252 5

-1 -16 -2 2633.55 297.176 8

1 -16 2 2948.79 309.634 8

-1 16 -2 2699.19 213.319 7

1 16 2 2478.21 216.269 9

-1 16 -3 1920.17 222.867 3

1 16 3 1714.31 206.478 3

-1 -16 -3 1675.05 192.583 3

1 -16 3 1836.23 263.641 5

1 16 3 1398.07 206.127 1

-1 16 -3 1629.42 149.445 1

1 -16 3 1594.43 159.896 1

-1 -16 -3 1591.99 216.281 1

1 16 3 1704.37 173.451 9

1 -16 3 1558.08 218.663 8

-1 16 -3 1798.73 167.643 7

-1 -16 -3 1398.85 224.303 8

1 16 4 3951.97 322.790 3

-1 -16 -4 3641.63 288.021 3

1 -16 4 3845.31 387.707 5

-1 -16 -4 3677.37 359.359 1

1 -16 4 3558.25 218.353 1

1 16 4 3484.08 239.370 9

-1 16 -4 3565.27 233.661 7

-1 -16 -4 3807.54 362.566 8

1 -16 4 3588.17 331.042 8

-1 -16 -5 77.3011 56.2992 3

1 -16 5 103.741 78.2054 5

-1 -16 -5 325.315 113.552 1

-1 16 -5 96.3041 65.7486 10

1 16 5 42.5435 29.7651 9

1 -16 5 130.946 91.0581 8

-1 -16 -6 36.9466 34.7213 3

1 16 6 82.6363 34.5118 2

-1 -16 -6 9.50706 55.3306 1

-1 16 -6 15.3603 52.4162 10

1 16 6-0.56194 43.8974 10

1 -16 6 38.4025 55.8121 8

-1 -16 -7 569.487 119.958 3

-1 16 -7 555.415 148.617 10

1 16 7 430.500 125.110 10

-1 -16 -7 491.931 148.613 1

-1 -16 -8 820.991 142.665 3

-1 16 -8 700.798 169.480 10

-1 -16 -8 912.035 213.517 1

-1 -16 -9 138.640 69.0738 3

-1 16 -9 121.882 89.1021 10

-1 -16 -9 15.2980 68.5582 1

-1 -16 -10 364.603 107.433 3

-1 16 -10 181.507 88.4484 10

-1 -16 -10 383.909 142.818 1

-1 -16 -11 559.937 132.891 3

-1 16 -11 518.957 165.593 10

-1 -16 -11 265.007 134.045 1

-1 -16 -12 108.387 66.3415 3

-1 16 -12-86.9860 80.3277 10

-1 -16 -12 94.9308 117.159 1

-1 -16 -13-8.12589 56.9543 3

-1 16 -13-112.001 100.264 10

1 -17 -14-56.1865 60.2123 3

1 -17 -13 91.7972 86.5352 3

1 17 -13 221.146 131.798 10

1 -17 -12 122.146 73.5449 3

1 17 -12 170.398 93.2898 10

1 -17 -11 147.797 79.3817 3

1 17 -11 17.7680 45.5025 10

1 -17 -11 27.0595 53.1147 1

1 -17 -10 5.90819 41.2247 3

1 17 -10-49.7387 66.0124 10

1 -17 -10 110.173 63.7273 1

1 -17 -9 140.216 82.2206 5

1 -17 -9 26.8281 38.3718 3

1 -17 -9 64.2061 83.9099 1

1 17 -9 139.634 97.2096 10

1 -17 -8 547.052 140.051 5

1 -17 -8 671.411 138.321 3

1 -17 -8 486.635 155.335 1

1 17 -8 836.801 187.698 10

-1 -17 8 496.252 124.497 8

1 -17 -7-0.72732 44.6681 3

1 -17 -7 68.8817 62.2691 5

1 -17 -7-3.05437 83.4892 1

-1 -17 7 56.0231 29.0042 1

1 17 -7 54.1225 64.0787 10

-1 -17 7 36.4819 47.6595 8

1 -17 -6 78.5292 66.3950 5

1 -17 -6 32.5859 36.9679 3

-1 -17 6 14.7147 24.2235 1

1 -17 -6 49.3039 73.7549 1

-1 17 6 86.0305 36.3938 2

1 17 -6 111.666 52.3957 10

-1 -17 6 15.3500 53.9917 8

1 -17 -5 4139.72 381.061 5

1 -17 -5 4475.59 346.324 3

1 -17 -5 4170.34 415.416 1

-1 17 5 4059.80 269.571 2

-1 -17 5 4064.77 253.775 1

1 17 -5 4675.59 381.900 10

-1 17 5 4277.90 307.693 9

-1 -17 5 4249.16 369.037 8

-1 17 4 704.584 143.928 3

-1 -17 4 619.297 160.501 5

1 -17 -4 424.001 114.196 5

1 -17 -4 576.217 122.082 3

1 -17 -4 568.326 159.274 1

-1 -17 4 566.450 100.949 1

-1 17 4 638.362 119.743 9

1 17 -4 703.360 149.545 10

-1 -17 4 533.768 130.068 8

1 17 -4 544.485 108.422 7

-1 -17 3 4720.64 494.649 5

1 -17 -3 6578.56 497.654 5

1 -17 -3 2798.66 277.704 3

-1 17 3 2400.90 271.952 3

1 17 -3 3398.25 265.263 1

1 -17 -3 6137.72 476.115 1

-1 -17 3 6347.88 365.575 1

-1 17 3 5429.73 434.186 1

-1 17 3 2264.73 236.357 9

1 17 -3 2632.16 241.832 7

-1 -17 3 5107.44 478.615 8

1 -17 -2 254.681 100.882 1

1 -17 -2 117.308 63.3170 3

-1 17 2 340.548 105.070 1

1 17 -2 205.622 62.5691 1

-1 -17 2 209.966 81.7603 1

1 17 -2 184.770 73.6541 3

-1 17 2 195.695 81.2521 3

-1 -17 2 242.817 93.6441 5

1 -17 -2 211.290 95.3757 5

-1 17 2 95.5656 46.5279 9

1 -17 -2 237.043 110.771 8

1 17 -2 211.826 64.2142 7

-1 -17 2 182.685 71.1582 8

1 17 -1 889.893 148.685 1

-1 17 1 1126.51 167.120 1

1 -17 -1 343.741 101.895 3

1 -17 -1 1242.60 185.906 1

-1 -17 1 1425.21 185.287 1

1 17 -1 358.293 110.215 3

-1 -17 1 1398.57 227.113 5

1 -17 -1 1450.75 236.536 5

-1 17 1 288.391 87.9788 3

1 17 -1 451.554 95.1350 7

-1 -17 1 1582.62 234.885 8

1 -17 -1 1137.75 198.820 8

-1 17 0-5.81613 36.7078 1

-1 -17 0-10.1216 41.3364 1

1 17 0-1.06910 43.6471 1

1 -17 0 16.4761 41.3133 1

-1 -17 0-86.6511 70.3191 5

1 17 0 26.4246 45.3539 3

1 -17 0-14.5021 46.3584 5

-1 17 0-16.7444 31.1976 3

1 -17 0-2.69047 46.1224 8

-1 -17 0-3.71506 75.0087 8

1 17 1 813.416 143.970 1

1 -17 1 1208.38 169.696 1

-1 17 -1 829.883 128.501 1

-1 -17 -1 1193.24 184.722 1

1 -17 1 986.235 198.622 5

-1 -17 -1 1263.28 222.998 5

1 17 1 198.020 80.2984 3

1 -17 1 875.955 183.690 8

-1 -17 -1 1052.09 196.974 8

-1 17 -2 3196.96 304.526 3

1 17 2 3469.13 300.389 3

1 -17 2 3202.69 348.154 5

-1 -17 -2 2779.83 254.476 3

-1 -17 -2 3052.94 333.942 5

1 -17 2 3249.13 264.088 1

-1 17 -2 3119.17 229.182 1

-1 -17 -2 2998.86 301.800 1

1 17 2 3026.03 291.877 1

1 17 2 2948.67 246.547 9

-1 -17 -2 2787.90 318.394 8

-1 17 -2 2974.29 236.288 7

1 -17 2 3073.80 320.911 8

1 17 3 5042.74 369.037 3

-1 -17 -3 4886.84 337.658 3

-1 17 -3 4375.01 259.800 1

1 -17 3 4427.27 286.002 1

-1 -17 -3 4521.41 385.559 1

1 17 3 4626.76 382.902 1

1 17 3 4724.25 297.422 9

1 -17 3 4452.40 380.632 8

-1 17 -3 4770.98 288.549 7

-1 -17 -4 11.2540 34.7458 3

1 17 4-3.73903 36.7879 3

-1 -17 -4-17.8659 75.2760 1

1 -17 4-2.28622 34.8613 1

1 17 4 5.45036 24.1432 9

1 -17 4-21.9935 30.0271 8

-1 17 -4 16.9526 28.4484 7

-1 -17 -5 827.415 143.577 3

-1 -17 -5 647.910 155.175 1

1 17 5 736.038 114.831 9

-1 17 -5 724.972 148.761 10

1 -17 5 604.513 132.834 8

-1 -17 -6 451.641 112.233 3

-1 -17 -6 499.454 150.274 1

1 17 6 518.282 83.3242 2

-1 17 -6 682.111 153.369 10

1 -17 6 495.911 124.270 8

-1 -17 -7 316.493 103.532 3

-1 17 -7 427.935 132.112 10

-1 -17 -7 474.087 160.341 1

-1 -17 -8-20.2503 46.2821 3

-1 -17 -8 133.239 78.7735 5

-1 17 -8 135.479 80.6303 10

-1 -17 -8 5.97038 69.5811 1

-1 -17 -9 280.143 92.2078 3

-1 17 -9 312.767 128.861 10

-1 -17 -9 230.574 127.641 1

-1 -17 -10-107.214 67.9123 3

-1 17 -10 31.4349 70.3369 10

-1 -17 -10-56.1935 104.431 1

-1 -17 -11 15.6031 39.0935 3

-1 17 -11-29.4357 69.2770 10

-1 -17 -12 213.697 103.260 3

-1 17 -12 0.25871 60.5290 10

-1 -17 -12 170.404 97.6143 1

-1 17 -13-28.5351 84.5904 10

1 -18 -13 239.397 108.690 3

1 -18 -12 80.5417 72.4616 3

1 18 -12 126.629 77.6309 10

1 -18 -11-20.2662 35.3388 3

1 -18 -11 75.7771 93.9001 1

1 -18 -10 266.141 100.746 3

1 18 -10 235.434 102.144 10

1 -18 -10 158.555 97.2457 1

1 -18 -9 219.785 88.2759 3

1 -18 -9 251.104 140.934 1

1 18 -9 434.461 150.545 10

1 -18 -8 48.6027 54.3346 3

1 -18 -8-40.5180 41.6030 5

1 -18 -8-20.1693 52.4321 1

1 18 -8-44.5938 55.6605 10

-1 -18 8-18.7262 28.8149 8

1 -18 -7 2456.71 260.894 3

1 -18 -7 1775.43 254.811 5

1 -18 -7 2474.80 348.182 1

-1 -18 7 2226.81 143.253 1

1 18 -7 2432.26 313.793 10

-1 -18 7 2016.20 256.545 8

1 -18 -6 234.467 95.0638 3

1 -18 -6 275.711 113.260 5

-1 18 6 150.225 47.6574 2

1 -18 -6 209.602 105.486 1

-1 -18 6 192.007 53.6105 1

1 18 -6 230.563 94.8648 10

-1 -18 6 205.371 91.2025 8

1 -18 -5 3001.24 337.908 5

1 -18 -5 3028.96 292.505 3

-1 18 5 2506.34 222.260 2

1 -18 -5 2861.66 347.226 1

-1 -18 5 2668.50 211.111 1

1 18 -5 2869.68 310.126 10

-1 18 5 2781.70 256.606 9

-1 -18 5 2510.22 287.581 8

1 -18 -4 58.6450 53.8416 5

1 -18 -4 96.2389 54.2352 3

-1 -18 4 2.12974 27.2931 1

1 -18 -4 142.637 88.5274 1

1 18 -4 89.7528 67.6076 10

-1 18 4 63.2844 36.0944 9

-1 -18 4 72.2849 57.1769 8

1 18 -4 33.4787 29.5754 7

-1 18 3 1602.51 232.041 1

-1 -18 3 1276.58 166.564 1

1 18 -3 877.395 141.582 1

1 -18 -3 1314.87 223.461 1

1 -18 -3 1794.98 260.524 5

1 -18 -3 868.103 159.500 3

-1 18 3 728.982 136.087 9

-1 -18 3 1453.47 246.599 8

1 18 -3 779.179 137.805 7

1 -18 -2 1554.73 245.421 5

1 -18 -2 1202.90 182.139 3

1 18 -2 1331.20 172.423 1

1 -18 -2 1389.06 218.857 1

-1 -18 2 1554.97 199.679 1

-1 18 2 1297.49 196.699 1

-1 18 2 1365.16 185.270 9

1 18 -2 1519.50 186.438 7

-1 -18 2 1590.62 244.274 8

1 18 -1 8195.05 4491.59 1

-1 -18 1 13715.8 6125.99 1

1 -18 -1 12292.5 6363.55 1

-1 18 1 11819.1 5542.15 1

-1 -18 1 13535.1 7043.25 8

-1 18 0 736.594 134.671 1

1 18 0 615.634 141.105 1

-1 -18 0 999.030 170.985 1

1 -18 0 729.115 150.350 1

1 18 0 909.814 157.308 3

1 -18 0 669.950 157.601 8

1 18 1 6471.56 432.368 1

1 -18 1 7157.61 430.568 1

-1 18 -1 6226.23 360.623 1

-1 -18 -1 7024.85 471.331 1

1 -18 1 6620.80 490.734 8

-1 -18 -2 64.0784 51.2487 3

1 18 2 234.822 96.1418 1

-1 18 -2 178.720 57.9552 1

1 -18 2 169.690 68.0262 1

-1 -18 -2 255.243 99.6161 1

1 18 2 161.037 71.4147 3

1 -18 2 185.030 99.9484 8

-1 18 -2 115.286 60.6971 7

1 18 2 89.0448 44.9825 9

-1 -18 -3 8369.01 458.582 3

1 18 3 9546.82 521.270 3

-1 18 -3 8026.74 364.048 1

1 -18 3 7578.10 392.549 1

-1 -18 -3 7325.51 506.802 1

1 18 3 8624.94 421.958 9

1 -18 3 8365.67 550.687 8

-1 18 -3 8469.13 402.054 7

-1 -18 -4 707.726 129.258 3

1 -18 4 729.439 116.966 1

-1 -18 -4 812.661 188.237 1

1 18 4 630.902 113.626 9

-1 18 -4 680.272 112.549 7

1 -18 4 731.096 170.742 8

-1 -18 -5 715.336 140.744 3

-1 -18 -5 1043.54 219.699 1

1 18 5 710.779 114.116 9

-1 18 -5 762.580 169.344 10

1 -18 5 928.863 177.415 8

-1 -18 -6-0.97288 56.0660 3

1 18 6 26.0357 26.8821 2

-1 -18 -6 41.7831 54.8934 1

-1 18 -6-31.4952 51.9544 10

1 -18 6-2.98225 33.6787 8

-1 -18 -7-63.3393 57.7797 3

-1 18 -7 46.3335 50.4023 10

-1 -18 -7 49.6398 71.2362 1

-1 -18 -8 152.973 75.5002 3

-1 18 -8 133.882 75.7470 10

-1 -18 -8 123.991 95.3564 1

-1 -18 -9 75.5633 65.6255 3

-1 18 -9 96.9838 90.1603 10

-1 -18 -9-34.2984 93.0319 1

-1 -18 -10 277.886 101.818 3

-1 18 -10 328.368 134.850 10

-1 -18 -10 235.734 128.212 1

-1 -18 -11 36.2314 61.6885 3

-1 18 -11 135.788 88.6886 10

-1 -18 -11 114.160 111.285 1

-1 -18 -12-79.5480 74.1261 3

-1 18 -12-142.618 124.471 10

1 -19 -13-88.2351 83.0253 3

1 -19 -12 2.61689 50.6680 3

1 19 -12 85.5242 80.0291 10

1 -19 -11-111.505 72.4644 3

1 19 -11 37.6303 59.7013 10

1 -19 -11 130.161 89.1741 1

1 -19 -10 119.031 77.7804 3

1 19 -10-16.3855 53.0096 10

1 -19 -10-46.7690 78.8804 1

1 -19 -9 62.0486 47.8525 3

1 -19 -9 111.679 82.0308 1

1 19 -9 89.8571 82.5674 10

1 -19 -8-78.1018 65.3569 3

1 -19 -8-25.4125 71.0110 1

1 19 -8-54.9698 38.8085 10

-1 -19 8-31.6777 42.1415 8

1 -19 -7 80.1119 44.2817 3

-1 -19 7 4.58741 17.1366 1

1 -19 -7-55.0881 92.1627 1

1 19 -7-78.4816 90.0685 10

-1 -19 7 17.4225 30.7896 8

1 -19 -6 30.4894 49.8917 3

-1 19 6 12.9753 22.8879 2

-1 -19 6 2.76759 22.2894 1

1 19 -6 128.747 92.7186 10

-1 -19 6-29.0604 76.1747 8

1 -19 -5 1200.35 191.710 3

1 -19 -5 1052.18 222.436 1

-1 -19 5 957.269 130.867 1

-1 19 5 915.553 144.304 2

-1 19 5 914.817 148.243 9

1 19 -5 1120.26 205.307 10

-1 -19 5 948.775 201.087 8

1 -19 -4 44.0083 47.0679 3

-1 -19 4-6.77444 51.5478 1

1 -19 -4-17.2637 83.7788 1

1 19 -4 194.726 100.551 10

-1 19 4 32.5959 27.9405 9

1 19 -4 62.1916 44.8985 7

-1 -19 4 46.1824 55.3842 8

1 -19 -3 445.192 106.855 3

1 -19 -3 356.758 130.078 1

-1 -19 3 405.896 102.073 1

1 19 -3 434.321 102.159 7

-1 19 3 449.706 105.326 9

-1 19 2 486.221 130.011 1

-1 -19 2 276.818 93.0313 1

1 -19 -2 256.990 90.8972 3

1 19 -2 409.048 101.158 1

1 -19 -2 413.977 134.788 1

-1 19 2 391.436 108.705 9

1 19 -2 381.791 106.914 7

1 -19 -1 492.920 123.724 1

1 19 -1 551.756 122.801 1

-1 19 1 526.965 116.213 1

-1 -19 1 573.327 132.163 1

-1 19 1 292.885 84.1842 9

1 19 -1 462.824 114.684 7

-1 19 0 381.049 105.223 1

1 -19 0 347.194 117.718 1

-1 -19 0 407.402 129.688 1

1 19 0 280.238 91.7829 1

1 -19 1 221.639 81.3351 1

1 19 1 265.034 89.6798 1

-1 -19 -1 297.562 110.920 1

-1 19 -1 338.644 92.5837 1

-1 -19 -2 187.128 87.2923 3

1 -19 2 201.832 71.6147 1

-1 19 -2 155.805 58.7625 1

-1 -19 -2 273.370 111.132 1

-1 19 -2 255.460 82.1590 7

1 19 2 69.0092 53.7237 9

1 -19 3 1099.19 163.260 1

-1 -19 -3 1224.77 181.108 3

-1 19 -3 1139.05 146.689 1

-1 -19 -3 1183.95 225.722 1

1 19 3 1045.57 154.644 9

1 -19 3 1085.24 207.364 8

-1 19 -3 1109.48 155.693 7

-1 -19 -4 138.594 100.298 1

-1 -19 -4 69.8012 62.3617 3

1 -19 4 160.978 53.6697 1

1 19 4 71.0342 43.4011 9

1 -19 4 201.541 91.1573 8

-1 19 -4 115.351 54.4184 7

-1 -19 -5 87.9311 56.3926 3

-1 19 -5-18.3020 45.3291 10

1 19 5-4.49530 43.6276 9

1 -19 5-27.9849 60.3620 8

-1 -19 -6 1125.52 178.595 3

1 19 6 892.150 123.888 2

-1 -19 -6 913.915 213.178 1

-1 19 -6 1171.19 203.797 10

1 -19 6 835.145 170.029 8

-1 -19 -7 256.996 86.8433 3

-1 19 -7 362.874 126.575 10

-1 -19 -7 323.190 144.861 1

-1 -19 -8 289.644 96.7986 3

-1 19 -8 132.983 63.0275 10

-1 -19 -8 277.940 134.938 1

-1 -19 -9 15.7064 34.2146 3

-1 19 -9 129.528 91.6864 10

-1 -19 -9-28.6373 74.6130 1

-1 -19 -10 0.15209 41.6503 3

-1 19 -10 133.388 101.659 10

-1 -19 -10-30.0192 82.2677 1

-1 -19 -11 0.64332 38.6647 3

-1 -19 -11 1.78986 67.3455 1

-1 -19 -12-42.1097 65.0853 3

-1 19 -12-60.2652 69.4707 10

1 -20 -13 44.8913 68.2141 3

1 -20 -12 93.3335 83.5714 3

1 -20 -11 79.5139 69.4901 3

1 20 -11-7.04988 59.7787 10

1 -20 -11-85.8265 105.829 1

1 -20 -10 213.376 93.5434 3

1 20 -10 331.050 156.487 10

1 -20 -10 229.371 126.953 1

1 -20 -9 136.005 73.9571 3

1 20 -9 262.010 119.115 10

1 -20 -9 273.381 140.367 1

1 -20 -8 13.5536 44.9335 3

1 -20 -8 66.1784 70.6226 1

1 20 -8-76.3176 94.6600 10

1 -20 -7 519.781 119.590 3

-1 -20 7 471.668 75.3943 1

1 -20 -7 636.150 184.401 1

1 20 -7 683.211 174.466 10

-1 -20 7 338.969 106.075 8

1 -20 -6 160.497 90.9256 3

1 20 -6-44.9900 51.3903 10

-1 20 6 136.319 67.9305 9

-1 -20 6-4.14423 30.0269 1

-1 20 6-22.5482 19.9232 2

1 -20 -6 19.0166 97.5230 1

1 -20 -5 1532.08 217.911 3

-1 -20 5 1362.95 172.315 1

-1 20 5 1202.00 169.684 2

1 -20 -5 1476.35 254.375 1

-1 20 5 1414.62 196.146 9

1 20 -5 1590.35 244.067 10

1 -20 -4 34.5712 57.6824 3

-1 -20 4-32.6892 57.9614 1

1 -20 -4 75.0352 71.9263 1

1 20 -4 109.688 91.6737 10

-1 20 4 9.91700 26.9601 9

1 20 -4-21.6690 38.9322 7

1 -20 -3 112.978 81.9390 3

1 -20 -3 396.584 176.503 1

1 20 -3 225.613 87.1236 7

-1 20 3 98.9365 69.6727 9

1 20 -2-17.9152 25.9416 1

-1 -20 2 47.7523 42.9716 1

1 -20 -2-2.41227 60.5629 1

1 -20 -2-77.6888 82.2842 3

-1 20 2-31.8367 46.5308 9

1 20 -2 10.3321 43.8733 7

-1 20 1 2196.16 253.855 1

-1 -20 1 2345.29 276.496 1

1 -20 -1 1916.30 283.080 1

1 20 -1 1337.26 194.391 1

1 20 -1 1046.82 183.625 7

1 -20 0 352.926 113.435 1

1 20 0 207.543 82.7722 1

-1 -20 0 159.885 59.1984 1

-1 20 0 241.360 85.3311 1

1 -20 1 5781.49 415.825 1

-1 -20 -1 5754.09 448.237 1

-1 20 -1 5603.57 372.070 1

-1 -20 -2 117.593 65.2761 1

-1 20 -2 121.072 56.2100 1

1 -20 2 91.7328 51.4293 1

1 20 2 30.0334 33.3807 9

-1 20 -2 107.144 54.2066 7

-1 20 -3 209.547 74.9638 1

-1 -20 -3 133.895 68.9987 3

-1 -20 -3 144.198 82.6863 1

1 -20 3 371.085 107.888 1

-1 20 -3 263.083 84.3015 7

1 20 3 118.117 54.2827 9

-1 -20 -4-18.0462 90.0212 1

-1 -20 -4 0.77323 41.7043 3

1 -20 4 55.8981 44.7655 1

1 20 4-31.0142 24.9893 9

-1 20 -4 39.4996 43.9906 7

-1 -20 -5 1409.28 204.652 3

-1 -20 -5 1402.46 267.002 1

1 20 5 1298.17 170.809 9

-1 20 -5 1711.18 256.514 10

1 -20 5 1577.99 254.088 8

-1 -20 -6 7.14606 48.7688 3

-1 -20 -6-20.5861 60.1013 1

1 20 6-27.7772 34.8670 2

-1 20 -6-95.3617 62.3983 10

1 -20 6 25.0848 39.6849 8

-1 -20 -7 100.084 56.6190 3

-1 20 -7-73.9840 39.1048 10

-1 -20 -7 77.9492 99.8624 1

-1 -20 -8 89.7824 59.2589 3

-1 20 -8 64.2351 56.6093 10

-1 -20 -8 63.1229 87.6259 1

-1 -20 -9 23.5681 40.4212 3

-1 20 -9 6.12346 55.6399 10

-1 -20 -9 74.3082 92.5996 1

-1 -20 -10 78.8466 62.0558 3

-1 -20 -10-12.9034 108.230 1

-1 -20 -11 167.998 90.7696 3

-1 20 -11 236.352 123.895 10

-1 -20 -12-3.71352 42.7845 3

1 -21 -12 142.003 93.2016 3

1 -21 -11-48.3351 87.0506 3

1 21 -11-88.8734 83.8502 10

1 -21 -10 83.4274 72.1092 3

1 21 -10-43.1383 65.3502 10

1 -21 -10-10.9571 69.2828 1

1 -21 -9 77.3467 64.7237 3

1 21 -9-52.5103 40.0370 10

1 -21 -9-132.146 103.699 1

1 -21 -8 92.3869 74.6310 3

1 21 -8 37.8158 49.9347 10

1 -21 -8-116.906 107.691 1

1 -21 -7-13.7614 38.5781 3

1 21 -7-22.9859 50.4244 10

1 -21 -7 46.7105 89.3488 1

1 -21 -6-41.6587 53.7904 3

1 21 -6-13.6979 56.8344 10

-1 21 6 68.6091 51.7157 9

-1 21 6 27.1918 20.2819 2

1 -21 -6 8.53249 54.8398 1

-1 -21 6 41.4786 29.1729 1

1 -21 -5 1349.77 210.995 3

1 -21 -5 1228.33 258.648 1

-1 21 5 1308.05 183.294 2

-1 -21 5 1189.31 164.631 1

-1 21 5 1180.63 184.843 9

1 21 -5 1487.83 241.901 10

-1 -21 4 170.661 63.2529 1

1 -21 -4 399.610 115.270 3

1 -21 -4 241.130 118.503 1

-1 21 4 323.024 93.7568 9

1 21 -4 336.795 119.624 10

1 21 -4 243.860 69.9816 7

1 -21 -3 505.341 145.555 1

1 -21 -3 255.574 98.5922 3

-1 -21 3 533.012 125.505 1

1 21 -3 166.907 70.0369 7

-1 21 3 95.3922 55.4054 9

-1 -21 2 78.5798 45.6932 1

1 -21 -2 127.432 76.2835 3

1 -21 -2 125.099 59.9069 1

1 21 -2 120.516 55.7760 1

1 21 -2 72.8869 59.9653 7

-1 -21 1 413.779 106.500 1

1 21 -1 372.578 105.473 1

1 -21 -1 397.481 126.979 1

1 21 -1 509.360 125.891 7

1 -21 0-122.568 96.6600 1

-1 -21 0-19.0723 51.6482 1

-1 21 0-1.14829 34.9962 1

1 21 0-31.6794 47.9664 7

1 -21 1 1420.64 219.033 1

-1 -21 -1 1332.77 223.732 1

-1 21 -1 1492.49 195.544 1

-1 21 -1 1768.08 214.286 7

-1 -21 -2-9.03101 70.0751 1

1 -21 2-49.7179 47.8021 1

-1 21 -2-46.1264 46.3782 1

1 21 2-23.3743 38.8678 9

-1 21 -2-60.9163 80.3318 7

-1 21 -3 635.205 120.171 1

-1 -21 -3 716.767 149.826 3

1 -21 3 634.586 128.502 1

-1 -21 -3 600.461 162.339 1

1 21 3 623.889 132.969 9

-1 21 -3 677.969 129.224 7

-1 -21 -4 313.382 112.145 3

1 -21 4 223.163 71.5304 1

-1 -21 -4 257.275 121.700 1

1 21 4 278.866 81.4582 9

-1 21 -4 371.026 97.3161 7

-1 21 -5 169.427 50.0071 10

1 21 5 174.877 71.5409 9

-1 -21 -5 234.343 95.3203 3

-1 -21 -5 352.400 145.843 1

-1 -21 -6 381.676 116.753 3

-1 21 -6 339.383 117.215 10

-1 -21 -6 393.868 147.687 1

-1 -21 -7-15.8689 45.5072 3

-1 21 -7-63.9221 82.0275 10

-1 -21 -7 113.957 83.8789 1

-1 -21 -8 89.9211 69.3776 3

-1 21 -8 111.563 98.8361 10

-1 -21 -9 63.9268 71.1469 3

-1 21 -9 26.8044 54.8725 10

-1 -21 -9 47.6751 88.7293 1

-1 -21 -10-1.01031 62.0067 3

-1 21 -10-67.5938 73.6414 10

-1 -21 -10-83.2848 78.6077 1

-1 -21 -11-12.1601 59.5439 3

-1 21 -11-118.746 89.1776 10

1 -22 -12-30.7321 42.5374 3

1 -22 -11 41.5154 61.8089 3

1 -22 -10 33.7936 42.5195 3

1 22 -10-26.8821 53.7011 10

1 -22 -10 4.00706 75.0159 1

1 -22 -9-37.0800 52.2190 3

1 22 -9 11.9247 61.4694 10

1 -22 -9 44.0804 97.3196 1

1 -22 -8 78.5576 43.3250 3

1 22 -8-16.1423 62.2194 10

1 -22 -8-18.1349 56.9133 1

1 -22 -7 48.3666 43.2252 3

1 22 -7 198.309 104.467 10

1 -22 -7 44.1053 61.6580 1

1 -22 -6 15.6215 37.9138 3

1 22 -6-54.4400 91.7450 10

-1 -22 6 44.9928 39.5702 1

1 -22 -6 64.2877 54.4275 1

1 -22 -5-15.2335 46.9443 3

1 -22 -5-19.9925 76.8415 1

-1 -22 5 6.15775 34.0321 1

-1 22 5-25.4469 41.5087 2

1 22 -5-129.656 93.6328 10

-1 -22 4 157.749 79.2546 1

1 -22 -4 108.205 85.6640 1

-1 22 4 150.934 69.9724 2

1 -22 -4 200.762 87.7772 3

1 22 -4 89.7520 61.6056 10

1 22 -4 105.518 58.2473 7

-1 -22 3 496.910 145.207 1

1 -22 -3 367.462 124.571 1

1 -22 -3 227.532 104.972 3

1 22 -3 136.702 66.7982 7

-1 -22 2 168.518 77.4360 1

1 22 -2 80.0567 65.7466 1

1 -22 -2 141.600 81.0338 3

1 -22 -2 94.7647 63.4893 1

1 22 -2 104.392 64.8909 7

-1 -22 1 488.244 134.261 1

1 22 -1 223.916 82.8720 1

1 -22 -1 472.526 141.316 1

1 22 -1 283.822 108.461 7

-1 -22 0 220.757 99.9827 1

-1 22 0 130.218 61.2660 1

1 -22 0 233.506 100.184 1

1 22 0 117.255 69.8855 7

-1 22 -1 22.8730 34.4461 1

1 -22 1 63.0004 70.4131 1

-1 -22 -1 78.8091 74.8277 1

-1 22 -1 82.1329 64.4583 7

-1 22 -2 373.865 101.257 1

1 -22 2 380.770 109.273 1

-1 -22 -2 387.531 134.330 1

-1 22 -2 442.990 118.529 7

1 22 2 337.460 102.971 9

1 -22 3 169.907 68.9209 1

-1 -22 -3 224.839 104.721 1

-1 22 -3 294.314 90.2714 1

-1 -22 -3 185.497 90.7630 3

-1 22 -3 332.028 99.7089 7

1 22 3 228.688 81.5782 9

1 -22 4 319.928 84.3573 1

-1 -22 -4 467.953 165.000 1

-1 -22 -4 402.991 117.304 3

1 22 4 419.451 113.090 9

-1 22 -4 438.627 105.282 7

-1 22 -5-9.66522 59.1527 10

1 22 5-14.3741 27.2135 9

-1 -22 -5 73.7228 105.459 1

-1 -22 -5 4.69041 44.5457 3

-1 -22 -6-48.4847 69.9948 3

-1 22 -6 2.57679 62.7309 10

-1 -22 -6 144.174 113.009 1

-1 -22 -7 137.749 77.1164 3

-1 22 -7 31.5680 55.6076 10

-1 -22 -7 3.88897 87.2620 1

-1 -22 -8 271.535 103.773 3

-1 22 -8-75.2676 91.1326 10

-1 -22 -8 263.236 154.239 1

-1 -22 -9-43.7920 65.6800 3

-1 -22 -9-143.640 95.4347 1

-1 -22 -10-15.3639 62.3341 3

-1 22 -10 170.457 128.403 10

-1 -22 -11 56.4345 42.0271 3

1 -23 -11-0.19796 44.7341 3

1 -23 -10 63.3502 77.6287 3

1 23 -10 60.8256 71.9481 10

1 -23 -9-17.2032 47.9262 3

1 23 -9-54.3360 69.6660 10

1 -23 -8-21.3050 47.4823 3

1 23 -8-36.3053 66.8807 10

1 -23 -8 14.6572 81.3110 1

1 -23 -7-36.9574 46.6622 3

1 23 -7 116.245 94.3567 10

1 -23 -7 63.1051 85.2416 1

1 -23 -6-85.5210 76.7678 3

1 -23 -6-33.9377 72.2188 1

-1 -23 6 39.1480 46.1829 1

1 23 -6 8.77078 57.6208 10

1 -23 -5-32.8930 79.5091 1

-1 -23 5 91.1398 69.3597 1

1 -23 -5 60.7175 78.9499 3

1 23 -5 5.86224 53.2265 10

-1 23 4 2.88440 30.6274 2

-1 -23 4-11.4018 60.2512 1

1 -23 -4 23.0408 59.6336 1

1 -23 -4 54.0293 61.1249 3

1 23 -4-9.98510 53.6586 10

1 23 -4 27.2082 42.6595 7

-1 -23 3 64.2101 66.5092 1

1 -23 -3-4.69276 41.0357 3

1 -23 -3-36.3171 76.5679 1

1 23 -3 20.8544 34.7588 7

1 -23 -2-24.6301 43.9905 1

1 23 -2-7.72285 32.5005 1

-1 -23 2 12.2836 54.0824 1

1 23 -2-19.8221 49.4671 7

1 -23 -1 58.5485 41.7501 1

-1 -23 1 53.5157 58.6299 1

1 23 -1-33.7414 44.9244 7

1 -23 0 8.36871 56.7191 1

-1 -23 0 66.2909 52.4273 1

1 23 0 25.5545 64.2536 7

-1 -23 -1 140.367 71.1597 1

1 -23 1 299.431 109.446 1

-1 23 -1 154.385 67.6548 1

-1 23 -1 197.799 85.9054 7

-1 23 -2 68.8582 62.3073 1

1 -23 2 23.9358 57.9067 1

-1 -23 -2 133.946 83.4747 1

-1 23 -2 80.7633 60.6484 7

-1 -23 -3 127.873 91.4319 1

-1 23 -3 98.7907 55.7408 1

1 -23 3 82.1693 57.9838 1

-1 23 -3 29.2858 33.7467 7

-1 -23 -4 109.771 106.492 1

-1 -23 -4-49.6904 60.2708 3

1 -23 4-45.5952 49.0484 1

1 23 4-31.2824 44.3005 9

-1 23 -4-13.0729 44.6262 7

-1 23 -5 283.802 126.954 10

-1 -23 -5-1.69473 77.1468 1

-1 -23 -5 264.011 98.0072 3

-1 23 -6 148.478 72.4209 10

-1 -23 -6 218.087 90.5360 3

-1 -23 -6 273.102 138.888 1

-1 -23 -7 76.1709 78.9032 3

-1 23 -7-57.0569 70.8262 10

-1 -23 -7-101.939 65.7621 1

-1 -23 -8 169.260 80.9145 3

-1 23 -8 152.338 97.7178 10

-1 -23 -8 322.305 158.993 1

-1 -23 -9 52.4580 71.1514 3

-1 23 -9-88.8433 85.7196 10

-1 -23 -10 28.0813 62.0135 3

-1 -23 -11 17.5914 60.6871 3

1 -24 -11 28.5887 63.8764 3

1 -24 -10 24.9462 64.2699 3

1 -24 -9-125.702 86.3067 3

1 24 -9-111.535 91.1264 10

1 -24 -8-22.2536 65.4683 3

1 24 -8-58.5465 78.1037 10

1 -24 -8 54.9601 111.000 1

1 -24 -7 19.0703 43.5259 3

1 24 -7-87.3430 80.3914 10

1 -24 -7-143.332 111.378 1

1 -24 -6 93.9798 68.5304 3

1 -24 -6-15.1650 54.4694 1

1 24 -6-89.6384 64.7574 10

1 -24 -5 276.975 111.751 3

-1 -24 5 213.894 91.2331 1

1 -24 -5 156.874 100.140 1

1 24 -5 446.183 150.551 10

-1 -24 4 23.6364 60.1328 1

1 -24 -4-40.2036 64.5755 1

1 -24 -4-20.6385 69.6691 3

1 24 -4-27.6046 41.8152 7

-1 -24 3 124.056 69.1567 1

1 -24 -3 67.1899 71.0644 3

1 -24 -3 146.790 104.150 1

1 24 -3-30.8309 47.7137 7

-1 -24 2-5.95970 36.3249 1

1 -24 -2-104.446 102.385 1

1 24 -2 38.1478 43.8493 7

1 -24 -1 105.205 82.7563 1

-1 -24 1 126.333 86.5068 1

1 24 -1 151.228 90.7860 7

1 -24 0 106.047 102.359 1

-1 -24 0 43.1783 62.1066 1

1 24 0 0.31891 61.6461 7

-1 -24 -1 3.40471 57.8854 1

1 -24 1 107.310 84.6257 1

-1 24 -1-17.3151 55.5395 7

-1 -24 -2-82.9163 94.0075 1

-1 24 -2 86.1315 61.0448 1

1 -24 2-93.5666 61.9653 1

-1 24 -2 63.3266 65.2334 7

-1 -24 -3 573.302 167.325 1

1 -24 3 363.935 112.412 1

-1 24 -3 523.365 127.013 7

1 -24 4 79.5670 50.6082 1

-1 -24 -4 9.13973 76.0006 1

-1 -24 -4 24.1102 43.9537 3

-1 24 -4-43.7262 96.3418 10

-1 24 -4 61.3398 64.8660 7

-1 24 -5 469.520 153.585 10

-1 -24 -5 494.629 188.334 1

-1 -24 -5 528.013 144.132 3

-1 24 -6-150.427 114.095 10

-1 -24 -6-28.8816 50.5958 3

-1 -24 -6 39.7108 62.7167 1

-1 24 -7-81.8124 100.872 10

-1 -24 -7-88.9250 92.1631 1

-1 -24 -7-79.7985 71.0720 3

-1 -24 -8 38.2977 62.4907 3

-1 24 -8 54.1853 75.8953 10

-1 -24 -9 6.87794 52.9328 3

-1 -24 -10-14.6790 42.2408 3

1 -25 -10-54.7620 89.2915 3

1 -25 -9 133.443 101.483 3

1 -25 -8 29.8221 66.9390 3

1 25 -8 158.279 119.455 10

1 -25 -8 92.6186 110.914 1

1 -25 -7 241.313 106.360 3

1 -25 -7 291.489 156.422 1

1 25 -7 339.328 148.816 10

1 -25 -6 25.4720 44.9267 3

1 -25 -6-103.647 130.230 1

1 25 -6 122.386 88.0053 10

1 -25 -5-62.0931 71.1548 3

1 -25 -5-3.96767 72.4906 1

-1 -25 5-71.6245 61.6995 1

1 25 -5 95.0288 72.1395 10

1 -25 -4-28.6320 46.7020 3

-1 -25 4 85.0064 45.0470 1

1 -25 -4-117.457 108.492 1

1 25 -4-89.9021 92.0896 10

1 25 -4 6.43392 58.1221 7

-1 -25 3-57.6110 63.7853 1

1 -25 -3 5.47702 65.5189 1

1 25 -3 85.9472 77.1472 7

-1 -25 2 74.6090 87.1825 1

1 -25 -2 25.6075 63.0597 1

1 25 -2-17.1390 39.2634 7

1 -25 -1 112.775 84.3622 1

-1 -25 1 26.7324 45.4753 1

1 25 -1 110.796 83.1046 7

1 -25 0 119.904 86.6300 1

-1 -25 0 175.101 83.5810 1

-1 25 0 26.0222 49.8451 7

1 25 0 55.8793 51.3446 7

-1 -25 -1 481.950 165.081 1

1 -25 1 637.266 168.299 1

-1 25 -1 523.409 138.520 7

1 25 1 441.814 130.553 7

1 -25 2 34.2485 50.0535 1

-1 -25 -2-31.7749 68.0775 1

-1 25 -2-45.0819 52.1721 7

1 -25 3 28.4473 34.0283 1

-1 25 -3 119.414 65.6767 7

-1 -25 -4-155.686 104.699 1

-1 25 -4-14.4201 35.7563 7

-1 25 -5-3.95733 54.7265 10

-1 -25 -5 9.44863 37.1128 3

-1 -25 -5 31.4996 59.3171 1

-1 25 -6 234.656 129.410 10

-1 -25 -6 242.189 95.4078 3

-1 -25 -6 213.067 122.925 1

-1 25 -7 14.2971 52.1713 10

-1 -25 -7 52.2834 52.2103 3

-1 -25 -7-18.0727 88.2517 1

-1 25 -8 107.072 57.0473 10

-1 -25 -8-86.5734 58.4756 3

-1 -25 -9 60.0978 67.1579 3

-1 -25 -10 31.6844 57.0503 3

1 -26 -9-26.4594 49.9086 3

1 -26 -8-53.6608 76.4336 3

1 -26 -7-47.6010 63.8976 1

1 -26 -7-22.6910 49.6542 3

1 26 -7 80.5335 50.8589 10

1 -26 -6 131.650 72.6950 3

1 -26 -6-50.0848 59.7198 1

1 26 -6 57.2797 83.1207 10

1 -26 -5 173.707 96.4328 3

1 -26 -5-91.8117 88.6015 1

1 26 -5 48.0050 78.9286 10

-1 -26 4-134.150 90.7916 1

1 -26 -4 34.9843 69.9715 3

1 -26 -4 70.7012 114.150 1

1 26 -4-65.0555 49.5307 10

1 26 -4 14.0830 53.0678 7

1 -26 -3 30.4875 70.2913 1

-1 -26 3 171.039 88.3422 1

1 26 -3-92.2324 81.8702 7

1 -26 -2-98.4556 84.6131 1

-1 -26 2-12.3366 51.3326 1

-1 -26 1 82.5057 99.4076 1

1 -26 -1 196.906 115.249 1

1 26 -1 185.324 103.836 7

-1 -26 0 34.6423 93.1874 1

1 -26 0 21.2849 64.4442 1

-1 26 0-31.7647 47.7730 7

1 26 0-0.89648 66.2335 7

-1 -26 -1 57.5627 54.5589 1

1 -26 1 53.7434 47.8670 1

1 26 1-0.92205 59.9463 7

-1 26 -1-58.8947 60.6078 7

1 -26 2 70.8252 88.4785 1

-1 -26 -2 6.01739 52.4959 1

-1 26 -2 109.966 68.9837 7

1 -26 3 190.298 86.5435 1

-1 -26 -3 383.006 137.755 1

-1 26 -3 304.733 107.834 7

-1 -26 -4-21.0274 101.038 1

-1 26 -4-175.367 105.618 10

-1 26 -4-13.5380 55.6564 7

-1 -26 -5 32.2080 64.1696 1

-1 -26 -5 85.0900 80.2805 3

-1 26 -5 9.99307 71.7939 10

-1 -26 -6 193.100 121.153 1

-1 -26 -6-23.0308 47.2440 3

-1 26 -6 117.157 112.655 10

-1 -26 -7 1.11906 47.3576 3

-1 26 -7 123.085 117.112 10

-1 -26 -8 4.21914 57.4639 3

-1 -26 -9-218.054 103.835 3

1 -27 -8 7.63394 51.4533 3

1 -27 -7 0.20118 51.2231 3

1 -27 -6 142.729 70.6418 3

1 -27 -6 173.086 119.365 1

1 27 -6 41.0025 88.0471 10

1 -27 -5 100.096 87.3214 1

1 -27 -5 29.9549 51.7328 3

1 27 -5-12.4184 65.5440 10

1 -27 -4-28.6525 131.367 1

1 27 -4-116.532 69.1528 10

1 -27 -3-9.74692 55.9148 1

-1 -27 3 165.711 77.5162 1

1 27 -3 147.536 77.1979 7

1 -27 -2-133.925 99.4963 1

-1 -27 2 15.6793 72.1438 1

1 27 -2 91.2665 68.4825 7

-1 -27 1-34.2439 73.8205 1

1 -27 -1 23.5201 59.8226 1

1 27 -1 2.00402 51.4542 7

-1 -27 0 58.7094 66.2519 1

1 -27 0 38.6294 74.4189 1

-1 27 0 18.9974 47.2634 7

1 27 0-81.6771 78.0364 7

1 -27 1-26.3291 53.8364 1

-1 -27 -1 37.1653 77.5565 1

1 27 1-47.6977 44.8838 7

-1 27 -1-50.6846 63.1846 7

1 -27 2 66.0618 70.1665 1

-1 -27 -2 96.8116 63.3389 1

-1 27 -2 128.410 67.8954 7

1 27 2 130.471 96.9566 7

-1 -27 -3-39.4206 88.5982 1

-1 27 -3-26.7113 49.4202 7

-1 -27 -4 14.8214 84.9182 1

-1 27 -4-171.827 109.645 10

-1 -27 -5 99.7296 106.775 1

-1 27 -5 225.854 127.966 10

-1 -27 -6-47.7040 87.4787 1

-1 -27 -6-62.3375 68.5957 3

-1 -27 -7-75.4218 85.6421 3

-1 -27 -8-145.100 88.4933 3

1 -28 -7 9.85921 45.5397 3

1 -28 -6-190.428 145.621 1

1 -28 -6 102.554 94.5738 3

1 -28 -5 114.252 109.291 3

1 -28 -5-60.1042 73.8108 1

1 28 -5 2.82637 50.3991 10

1 -28 -4-67.7140 108.699 1

1 28 -4-0.24418 62.0700 10

-1 -28 3 123.762 85.7722 1

1 -28 -3 142.235 116.245 1

1 28 -3 79.7542 78.1826 10

1 28 -3 76.2743 76.3853 7

1 -28 -2 70.5185 75.2794 1

-1 -28 2-45.5192 70.9788 1

1 28 -2 126.275 94.1228 7

-1 -28 1 201.712 109.617 1

1 -28 -1-33.2752 65.3987 1

-1 28 1 139.958 87.8119 7

1 28 -1 133.469 71.8277 7

-1 -28 0-28.3385 66.0316 1

1 -28 0-84.0307 74.9931 1

1 28 0 43.7101 58.6451 7

-1 28 0-94.6961 76.8839 7

1 -28 1 20.0964 54.8720 1

-1 -28 -1 50.2190 79.0875 1

1 28 1 82.8702 78.7970 7

-1 28 -1 129.520 85.8420 7

-1 -28 -2 37.1158 63.8090 1

1 -28 2 69.3908 87.8343 1

1 28 2 29.4315 61.0638 7

-1 28 -2-128.642 84.2247 7

-1 -28 -3 15.1093 114.776 1

-1 28 -3 227.890 102.331 7

-1 -28 -4-65.4156 100.103 1

-1 28 -4-36.6590 72.8142 10

-1 -28 -5-61.0083 63.6474 1

-1 -28 -7-54.7304 63.3663 3

1 -29 -5-33.1239 106.852 1

1 -29 -4 135.268 104.748 1

1 -29 -3 122.907 87.0464 1

1 29 -3-108.669 89.5921 10

1 29 -3 0.19113 41.5582 7

1 -29 -2 11.0982 51.2235 1

-1 -29 2-24.6876 78.4902 1

1 29 -2-96.1588 74.2911 7

-1 -29 1 58.0055 77.2312 1

1 29 -1 186.970 107.270 7

-1 29 1 2.02942 55.8915 7

1 -29 0-44.0340 53.4592 1

-1 29 0 118.613 84.6980 7

1 29 0 140.761 84.7325 7

1 -29 1 113.673 101.534 1

-1 -29 -1 1.33408 95.2292 1

-1 29 -1 165.916 94.2847 7

1 29 1-34.4847 64.6305 7

-1 -29 -2 0.28063 61.4893 1

-1 29 -2-17.5889 46.8583 7

1 29 2-121.799 92.8083 7

-1 -29 -3 36.0958 54.2085 1

-1 29 -3 87.9241 56.7806 7

1 29 3 0.91884 73.4020 7

-1 -29 -4-138.518 141.104 1

1 -30 -4-96.8455 99.9670 1

1 -30 -3-124.120 106.193 1

1 30 -3-0.20100 60.7186 7

1 -30 -2-158.949 135.027 1

-1 -30 2-37.8073 83.2452 1

1 30 -2 116.574 88.1603 7

-1 30 2-117.874 98.9927 7

1 -30 -1-98.9397 90.8047 1

-1 -30 1 4.73109 61.7698 1

-1 30 1 91.6299 90.0571 7

1 30 -1-108.304 75.7560 7

-1 -30 0-125.290 81.6740 1

1 30 0 2.63910 65.0973 7

-1 30 0-108.374 85.0457 7

-1 -30 -1-66.2948 77.1173 1

1 -30 1-20.9352 66.9190 1

1 30 1-18.4843 58.9768 7

-1 30 -1 31.6031 77.6214 7

-1 -30 -2 44.7421 56.9629 1

1 30 2 85.3589 59.5528 7

-1 30 -2 16.2856 68.8590 7

-1 -30 -3-68.1353 92.6525 1

-1 30 -3 81.5042 68.3652 7

1 -31 -3 245.861 161.296 1

1 31 -3 65.8783 80.9916 7

1 -31 -2-83.6584 94.5465 1

1 31 -2 35.0525 60.4929 7

-1 31 2 0.69528 65.1438 7

-1 -31 1 97.7820 98.7743 1

1 -31 -1 4.55422 60.3674 1

1 31 -1 12.3289 82.7086 7

-1 31 1-80.0692 62.6460 7

1 -31 0 89.0987 97.8796 1

-1 -31 0-32.2938 76.7381 1

1 31 0-194.594 129.508 7

-1 31 0 55.5044 67.4329 7

-1 -31 -1 99.8113 57.7540 1

1 31 1-299.318 134.227 7

-1 31 -1-106.975 83.4200 7

-1 -31 -2 230.036 173.717 1

-1 31 -2-41.4883 81.4778 7

1 32 -3-197.078 105.761 7

1 -32 -2 18.5899 81.9018 1

1 32 -2 33.7415 70.9273 7

1 -32 -1-396.595 167.210 1

1 32 -1-89.1139 77.0994 7

-1 -32 0 73.6629 59.7798 1

-1 32 0 21.9611 46.6591 7

-1 -32 -1 330.108 186.008 1

-1 32 -1 120.524 96.2926 7

2 0 -16 20.8253 51.4941 10

2 0 -15 13.5333 73.4545 10

2 0 -15 84.8896 98.6362 6

2 0 -14-66.6380 52.3156 10

2 0 -14 30.0632 66.7833 6

-2 0 13-200.215 85.6507 1

2 0 -13-52.8821 69.5837 6

-2 0 12 472.105 127.734 1

2 0 -12 659.291 162.148 10

2 0 -12 725.148 184.292 6

-2 0 11-58.4917 45.7838 1

2 0 -11-38.9241 66.2624 10

2 0 -11-41.9301 66.8142 6

-2 0 10 65.9374 58.7253 4

2 0 -10-38.6388 68.7790 10

-2 0 10-19.1377 27.8372 10

2 0 -10-12.3690 57.3519 6

2 0 -9 44.1933 51.3459 10

-2 0 9-58.4850 56.7967 10

2 0 -9 11.4039 38.2968 6

-2 0 8 9885.45 454.927 1

2 0 -8 10894.4 509.835 10

-2 0 8 10399.9 509.652 10

-2 0 8 9666.16 302.297 8

-2 0 7 28.4111 33.3330 4

-2 0 7 33.3991 41.2898 1

-2 0 7-46.5413 39.8606 10

2 0 -7-37.1205 38.4081 10

-2 0 7-13.9291 19.8046 8

-2 0 6 31396.5 750.565 4

-2 0 6 29982.4 711.025 1

2 0 -6 32173.7 758.663 10

-2 0 6 31350.2 759.511 10

-2 0 5-25.2692 41.7199 4

-2 0 5 39.5403 37.4618 1

-2 0 5 21.2331 26.2939 10

2 0 -5 9.63650 33.9785 10

2 0 -5 6.24801 30.3792 6

-2 0 4 13226.4 410.111 10

2 0 -4 13130.9 401.905 10

2 0 -4 13068.5 396.754 6

-2 0 3 9.37769 17.5706 10

2 0 -3-1.41373 15.5902 6

-2 0 -1 29.0104 15.9607 1

2 0 1 11.7425 12.1427 2

2 0 1 2.50463 6.03537 7

-2 0 -2 123898. 890.559 2

2 0 2 123396. 862.907 1

-2 0 -2 125202. 844.509 1

2 0 2 122017. 657.912 10

-2 0 -3-12.1481 14.0685 1

2 0 3-34.6948 25.2527 1

-2 0 -3-13.8372 15.3655 2

2 0 3 17.8845 13.2812 10

-2 0 -3-0.26201 9.85697 10

-2 0 -3-32.3846 32.2428 6

-2 0 -4 19672.9 429.407 4

-2 0 -4 19154.8 424.152 10

2 0 4 19158.7 414.021 10

-2 0 -4 20100.0 449.033 1

-2 0 -4 19036.5 421.313 2

-2 0 -5 9.96806 19.1595 4

-2 0 -5-13.5492 18.7281 1

-2 0 -5 9.02448 19.7901 2

2 0 5 25.2487 24.5875 1

-2 0 -5-12.4209 20.9325 10

2 0 5-12.1353 21.3874 10

-2 0 -6 1167.73 125.382 4

-2 0 -6 1122.61 127.128 1

2 0 6 1104.19 121.585 1

-2 0 -6 1025.14 110.519 2

-2 0 -6 1074.65 128.916 10

2 0 6 1212.88 131.843 10

-2 0 -6 1170.04 120.319 5

-2 0 -7-5.80922 19.2257 2

2 0 7 5.74473 28.2680 1

-2 0 -7-0.15972 28.7404 1

2 0 7 18.0616 23.5769 10

-2 0 -7-22.9470 49.0065 10

-2 0 -7 14.7771 17.2613 5

-2 0 -8 11885.8 498.384 1

-2 0 -8 11011.6 405.355 2

2 0 8 10750.9 446.130 1

-2 0 -8 12076.4 505.714 10

2 0 8 12578.7 503.840 10

-2 0 -8 12054.2 639.971 6

-2 0 -8 11846.4 417.545 5

-2 0 -9-3.22544 24.3043 5

-2 0 -9-12.2214 28.0732 2

-2 0 -9 5.53024 35.3803 1

2 0 9 30.4433 46.3244 10

-2 0 -9 42.0744 52.5493 10

-2 0 -9 37.6313 57.8066 6

-2 0 -10 1582.60 157.963 5

-2 0 -10 1425.92 154.769 2

2 0 10 1714.20 214.063 10

-2 0 -10 1624.09 209.154 10

-2 0 -10 1755.51 278.928 6

-2 0 -11-18.5989 25.7998 5

2 0 11-21.9678 43.2240 1

2 0 11-46.5704 29.3437 10

-2 0 -11 22.7491 51.1535 10

-2 0 -11 10.4704 53.7058 6

-2 0 -12 241.877 94.5808 10

-2 0 -12 528.660 186.559 6

-2 0 -13-9.91791 34.0782 10

-2 0 -13-16.2372 79.8123 6

-2 0 -14 564.107 154.189 10

-2 0 -14 501.802 179.556 6

2 -1 -16 93.6600 87.0904 10

2 1 -16 134.565 106.138 10

2 -1 -15 82.6352 64.6044 10

2 1 -15-43.5869 80.9989 10

2 1 -15 24.5350 48.9286 6

2 -1 -15-84.8492 84.7809 6

2 -1 -14-16.2019 61.9244 10

2 -1 -14 0.74081 38.3153 6

2 1 -14-150.316 86.3168 6

-2 -1 13-69.4591 87.2539 1

2 -1 -13-103.695 86.0927 10

2 1 -13 14.9648 48.7064 10

2 1 -13-15.6151 81.8732 6

2 -1 -13 88.5049 65.2883 6

-2 1 12-7.97718 44.6321 1

-2 -1 12-10.0678 32.0709 1

2 1 -12-2.70095 66.9508 10

2 -1 -12 32.6796 59.4647 10

2 1 -12 17.7480 57.0952 6

2 -1 -12-42.9201 73.8293 6

-2 -1 11 0.14000 51.5001 4

-2 1 11 87.7276 71.0829 1

-2 -1 11 15.6035 41.9661 1

2 -1 -11-38.5324 53.5284 10

2 1 -11 20.4037 45.8319 10

2 1 -11 63.1484 56.7322 6

2 -1 -11-53.4364 44.1223 6

-2 -1 10 2776.08 281.972 4

-2 1 10 2427.64 264.411 1

2 1 -10 2740.29 286.546 10

-2 1 10 2618.38 279.257 10

-2 -1 10 2906.14 296.464 10

2 -1 -10 2692.82 282.552 10

2 -1 -10 2707.53 310.636 6

2 1 -10 2575.99 309.531 6

-2 1 9 106.412 59.0236 4

-2 -1 9 86.3536 50.8364 1

2 -1 -9 97.2782 56.7267 10

-2 1 9 94.2922 46.4325 10

2 1 -9 132.869 75.5471 10

-2 -1 9 98.8717 53.0024 10

2 1 -9 132.618 65.7379 6

2 -1 -9 23.1556 36.6757 6

-2 1 8 1119.05 158.290 1

-2 -1 8 990.945 146.331 1

-2 1 8 1181.40 173.849 10

-2 -1 8 1064.00 159.224 10

2 1 -8 1121.58 161.743 10

2 -1 -8 1144.76 167.054 10

-2 -1 8 1064.79 93.1149 8

-2 1 8 1001.95 106.532 8

2 1 -8 947.531 159.773 6

-2 -1 7 1476.72 174.949 4

-2 1 7 1611.86 188.356 4

-2 -1 7 1421.11 167.853 1

-2 1 7 1489.33 176.384 1

-2 -1 7 1573.96 181.982 10

2 1 -7 1614.91 184.394 10

2 -1 -7 1675.55 181.059 10

-2 1 7 1607.55 182.589 10

-2 1 7 1373.77 127.792 8

-2 1 6 81660.5 1205.58 4

-2 -1 6 82428.6 1212.25 4

-2 -1 6 78026.4 1122.51 1

-2 1 6 78378.4 1203.64 1

-2 -1 6 84146.5 1235.09 10

2 -1 -6 84615.9 1217.97 10

-2 1 6 83891.5 1252.18 10

2 1 -6 83203.8 1230.45 10

-2 1 6 77894.9 903.346 8

-2 1 5 6578.83 319.083 4

-2 1 5 6145.57 309.043 1

2 -1 -5 6548.73 314.774 10

-2 -1 5 6878.67 324.833 10

2 1 -5 6601.39 316.767 10

-2 1 5 6250.50 309.604 10

2 -1 -5 6641.86 323.942 6

-2 1 4 308512. 1995.78 4

-2 1 4 315530. 1980.51 10

2 1 -4 313745. 1959.40 10

2 -1 -4 313039. 1956.07 10

-2 -1 4 318315. 2000.70 10

2 -1 -4 308260. 1933.21 6

-2 1 3 28252.5 526.220 10

-2 -1 3 27920.2 517.225 10

2 -1 -3 28070.4 491.110 6

-2 1 3 28802.8 505.010 6

-2 -1 2 5012.85 184.445 10

-2 1 2 4992.37 188.246 10

-2 1 2 5108.56 164.345 6

-2 1 2 5032.66 134.526 5

-2 1 -1 3231.81 142.684 2

-2 -1 -1 3331.74 137.480 1

2 -1 1 3408.31 147.150 2

2 1 1 3091.63 135.142 1

-2 -1 -1 3378.87 140.757 6

2 1 1 3266.20 143.918 6

2 1 2 315702. 1332.02 2

-2 1 -2 307495. 1547.50 2

2 1 2 312671. 1501.31 1

-2 1 -2 319497. 1194.19 1

-2 1 -3 15880.8 294.477 4

-2 1 -3 14740.5 297.639 1

2 -1 3 15432.8 311.926 1

-2 -1 -3 14641.7 354.593 1

2 1 3 15098.8 334.281 2

2 1 3 15632.0 372.671 1

-2 1 -3 15080.1 370.849 2

2 1 3 16289.2 321.921 10

-2 -1 -3 16217.5 321.468 10

-2 1 -3 15906.7 339.584 10

2 -1 3 15996.1 321.992 10

-2 -1 -3 16485.3 468.502 6

-2 1 -4 38709.9 557.664 4

-2 -1 -4 38484.5 642.231 4

2 -1 4 37425.1 548.081 1

-2 -1 -4 38300.1 540.060 2

-2 -1 -4 37372.7 643.336 1

-2 1 -4 37483.1 572.399 1

-2 1 -4 36128.1 632.116 2

2 1 4 36010.4 618.730 1

-2 -1 -4 38787.3 595.049 10

-2 1 -4 39175.1 626.118 10

2 -1 4 38711.7 588.939 10

2 1 4 38353.7 592.014 10

-2 1 -4 39866.5 648.670 5

-2 -1 -4 41076.0 836.173 6

-2 -1 -5 375.869 72.6334 4

-2 1 -5 310.850 58.9183 4

2 1 5 182.300 71.8424 1

-2 1 -5 255.273 55.8748 2

-2 -1 -5 304.335 75.2451 1

-2 -1 -5 332.342 57.0715 2

2 -1 5 293.420 55.1468 1

-2 1 -5 408.170 73.5253 1

2 1 5 353.537 64.9113 10

-2 -1 -5 360.876 63.0545 10

-2 1 -5 359.972 67.8792 10

2 -1 5 340.303 67.5473 10

-2 1 -5 329.662 65.8909 5

-2 -1 -6 17508.5 528.313 4

-2 -1 -6 16974.6 424.313 2

-2 1 -6 18377.3 499.275 1

-2 1 -6 16499.6 478.525 2

-2 -1 -6 19035.2 553.869 1

2 1 6 17194.4 510.505 1

2 -1 6 17057.1 454.349 1

2 -1 6 18868.1 521.487 10

-2 1 -6 19330.5 548.386 10

-2 -1 -6 18897.0 524.725 10

2 1 6 18372.7 512.788 10

-2 -1 -6 16734.0 320.621 8

-2 1 -6 18251.7 499.982 5

-2 -1 -7 214.212 70.9496 4

-2 1 -7 229.959 62.0603 5

-2 1 -7 208.507 66.2502 1

2 1 7 121.542 67.8894 1

-2 -1 -7 240.421 52.3909 2

-2 1 -7 188.407 58.5672 2

-2 -1 -7 187.216 70.5709 1

2 -1 7 213.346 59.8572 1

-2 1 -7 230.175 67.6749 10

-2 -1 -7 217.109 61.1717 10

2 -1 7 228.852 65.4639 10

2 1 7 204.667 58.5577 10

-2 1 -7 165.580 72.0453 6

-2 -1 -7 238.472 90.7166 6

-2 -1 -7 212.841 65.6308 5

-2 1 -8 20.9648 25.1387 5

-2 1 -8 16.8023 27.3463 2

-2 -1 -8 106.515 43.6017 2

2 -1 8 25.2635 27.2338 1

-2 1 -8 71.9436 40.5309 1

-2 -1 -8 18.7482 31.1693 1

2 -1 8 33.4574 26.9368 10

-2 1 -8 54.5049 47.0717 10

-2 -1 -8 76.3891 26.5344 10

2 1 8 11.9678 21.6155 10

-2 -1 -8 31.1781 33.7508 5

-2 -1 -8 25.1707 51.8336 6

-2 1 -8 0.45863 53.7222 6

-2 1 -9 81.3932 35.1330 5

-2 -1 -9 82.3422 29.0652 5

-2 1 -9 101.267 46.9835 2

2 -1 9 87.6844 57.5825 1

-2 -1 -9 78.5685 38.8102 1

-2 -1 -9 139.771 59.6083 10

2 1 9 110.702 52.7953 10

-2 1 -9 122.155 64.9017 10

2 -1 9 107.310 55.9570 10

-2 1 -9 145.396 97.5500 6

-2 -1 -9 163.385 81.9957 6

-2 -1 -10 43.5438 28.7548 5

-2 1 -10 128.348 62.5929 5

-2 1 -10 91.9845 41.6978 2

2 1 10 34.6577 48.7337 1

2 1 10 59.3670 51.0879 10

2 -1 10 20.8392 41.8265 10

-2 -1 -10 116.902 72.9354 10

-2 1 -10 94.8554 51.1898 10

-2 1 -10 175.451 95.6323 6

-2 -1 -10 73.2367 47.4077 6

-2 1 -11 166.115 54.4641 5

-2 -1 -11 162.817 48.4723 5

2 1 11 202.090 88.8114 1

-2 1 -11 102.766 60.4539 2

2 -1 11 154.255 63.8014 10

-2 -1 -11 108.929 58.4589 10

-2 1 -11 145.879 68.0650 10

2 1 11 61.3070 26.1051 10

-2 1 -11 108.990 71.7031 6

-2 -1 -11 234.458 117.788 6

-2 1 -12-22.9458 36.3884 10

-2 -1 -12-49.3627 46.9682 10

-2 1 -12 87.3118 87.2218 6

-2 -1 -12 51.2931 67.6344 6

-2 1 -13 3.80960 42.0841 10

-2 -1 -13-4.48722 34.6490 10

-2 -1 -13 68.9753 92.2174 6

-2 1 -13-0.54561 44.7709 6

-2 -1 -14 101.042 65.2326 10

-2 1 -14-109.148 92.2486 10

-2 -1 -14 39.5095 77.3850 6

-2 1 -14-45.0154 50.6045 6

2 2 -16 94.2801 61.6879 10

2 -2 -16 56.5366 54.4453 10

2 2 -15-33.0119 54.1788 10

2 -2 -15-23.6429 51.6084 10

2 -2 -15-24.3853 93.1058 6

2 2 -15 42.3813 62.4453 6

2 2 -14 65.1909 39.2989 10

2 -2 -14-1.56992 61.8019 10

2 -2 -14 123.822 54.0952 6

2 2 -14 68.0849 68.6134 6

-2 -2 13-42.5829 69.5799 1

2 -2 -13-49.9535 62.8687 10

2 2 -13-72.8708 69.1068 10

2 2 -13-129.380 95.3099 6

2 -2 -13 78.2563 77.8275 6

-2 -2 12 43.7068 43.2204 1

-2 2 12-53.2260 54.4628 1

2 -2 -12 15.5150 20.0110 2

2 2 -12-88.1955 72.1713 10

2 -2 -12 66.6601 69.9336 10

2 2 -12-34.9222 60.9278 6

2 -2 -12-61.8829 99.0990 6

-2 -2 11 752.261 147.903 4

-2 2 11 630.817 135.670 1

2 2 -11 641.573 157.234 10

2 -2 -11 583.374 138.025 10

2 2 -11 776.326 192.225 6

2 -2 -11 599.935 158.811 6

-2 -2 10 1464.91 207.462 4

-2 2 10 970.093 189.581 1

2 -2 -10 1611.56 219.101 10

-2 -2 10 1505.16 211.185 10

2 2 -10 1541.72 223.113 10

-2 2 10 1617.76 228.110 10

2 2 -10 1339.83 223.583 6

2 -2 -10 1435.64 228.676 6

-2 -2 9-11.9369 31.2839 1

2 2 -9-24.1256 49.7428 10

2 -2 -9 3.71961 31.2478 10

-2 2 9-7.08264 28.1942 10

-2 -2 9 94.1570 58.7699 10

2 -2 -9 17.4508 42.6667 6

2 2 -9-66.3274 59.5975 6

-2 -2 8 4859.29 330.743 4

-2 -2 8 4423.78 305.685 1

2 -2 -8 4806.63 336.227 10

-2 -2 8 4836.48 339.785 10

-2 2 8 4858.15 351.349 10

2 2 -8 4777.60 340.261 10

-2 2 8 4619.98 242.876 8

2 2 -8 4719.35 370.363 6

-2 -2 8 4709.55 180.774 8

-2 -2 7 915.526 141.605 4

-2 -2 7 774.020 116.462 1

-2 2 7 808.124 133.708 1

2 -2 -7 834.108 127.689 10

-2 -2 7 943.486 137.755 10

-2 2 7 954.274 138.531 10

2 2 -7 951.706 144.639 10

-2 2 7 765.790 103.678 8

-2 2 6 138951. 1611.59 4

-2 2 6 129346. 1594.33 1

-2 -2 6 135335. 1419.71 1

-2 2 6 135421. 1579.44 10

-2 -2 6 139482. 1573.39 10

2 -2 -6 138307. 1565.02 10

2 2 -6 141943. 1614.51 10

-2 2 6 134777. 1247.85 8

-2 -2 6 129954. 760.956 5

-2 -2 5 1573.26 85.9521 5

-2 2 5 1375.68 149.485 4

-2 2 5 1447.30 158.909 1

2 -2 -5 1680.24 160.215 10

-2 -2 5 1656.40 162.000 10

2 2 -5 1736.70 167.632 10

-2 2 5 1706.77 166.885 10

-2 2 5 1688.53 171.981 6

2 -2 -5 1713.26 166.307 6

-2 2 5 1406.54 127.389 8

-2 2 4 6228.57 291.131 4

-2 2 4 6066.97 293.823 1

-2 -2 4 6496.18 281.720 10

-2 2 4 6610.19 292.236 10

2 -2 -4 6537.50 282.630 10

-2 2 4 6587.80 293.510 6

2 -2 -4 6518.27 286.029 6

-2 2 3 1322.83 73.2190 5

-2 2 3 1422.34 145.848 1

-2 2 3 1763.32 137.462 10

-2 -2 3 1532.50 131.946 10

-2 2 3 1639.81 132.405 6

2 -2 -3 1329.30 71.6212 5

2 -2 -3 1863.76 131.800 6

-2 -2 2 43019.8 552.765 10

-2 2 2 43505.7 571.938 10

-2 2 2 44164.5 461.749 5

2 -2 -2 43749.2 465.035 5

-2 2 2 44479.1 513.946 6

-2 2 -1 3309.68 160.644 2

2 2 1 3429.87 155.645 1

-2 -2 -1 3245.83 149.281 1

-2 2 -1 3890.77 125.364 3

-2 -2 -1 3532.42 147.179 6

-2 2 -1 3417.49 167.277 5

2 2 1 3711.93 159.008 6

2 2 2 134254. 1063.59 1

-2 2 -2 134317. 1121.60 2

-2 -2 -2 136855. 1053.93 1

-2 2 -2 130039. 681.975 1

-2 2 -2 127879. 819.772 10

2 2 2 120991. 704.306 10

2 2 2 127104. 1140.14 6

-2 2 -2 125525. 1076.48 5

-2 -2 -2 120076. 1087.44 6

-2 2 -3 1866.42 88.1097 4

2 2 3 1875.71 107.247 2

2 -2 3 1863.88 99.1013 1

-2 -2 -3 1907.79 138.852 1

-2 2 -3 1775.96 144.678 2

2 2 3 1865.66 144.792 1

-2 2 -3 1817.71 94.6248 1

-2 -2 -3 2027.16 109.650 10

-2 2 -3 1923.59 124.552 10

2 -2 3 2092.03 119.790 10

2 2 3 1891.88 110.529 10

-2 2 -3 1831.77 140.567 5

2 2 3 2065.39 170.837 6

-2 -2 -3 2038.38 166.020 6

2 2 4 10215.3 355.452 1

-2 2 -4 9837.06 354.295 2

2 -2 4 9249.67 250.932 1

-2 2 -4 10088.4 286.318 1

-2 2 -4 9427.23 317.109 10

2 2 4 9554.29 300.543 10

2 -2 4 9879.29 304.271 10

-2 -2 -4 9484.20 287.871 10

-2 2 -4 9493.07 333.546 5

-2 -2 -5 258.245 77.7870 4

-2 2 -5 167.363 53.8738 5

2 2 5 136.145 67.4387 1

-2 2 -5 109.695 39.8220 2

2 -2 5 99.3451 41.6110 1

-2 -2 -5 204.876 62.8889 1

-2 2 -5 138.114 46.0550 1

-2 -2 -5 174.458 51.4887 10

-2 2 -5 141.545 43.3917 10

2 -2 5 100.587 43.4238 10

2 2 5 148.539 48.2252 10

-2 -2 -6 17584.4 549.776 4

2 -2 6 17190.0 429.690 1

2 2 6 17627.6 541.760 1

-2 2 -6 16396.5 508.661 2

-2 2 -6 17600.9 462.591 1

-2 -2 -6 17037.9 555.601 1

2 2 6 17556.9 511.657 10

-2 2 -6 18465.8 543.647 10

-2 -2 -6 18692.2 513.096 10

2 -2 6 18057.2 510.795 10

-2 2 -6 18282.1 499.259 5

-2 -2 -6 15822.4 360.428 8

-2 -2 -7 1077.46 145.277 4

-2 -2 -7 1027.85 147.708 1

-2 2 -7 1090.99 123.589 1

2 2 7 1123.54 146.960 1

2 -2 7 1135.06 122.048 1

-2 2 -7 846.898 123.801 2

-2 -2 -7 1118.23 134.552 10

2 -2 7 1129.03 139.608 10

2 2 7 1102.77 136.368 10

-2 2 -7 1171.33 150.037 10

-2 2 -7 1164.62 130.082 5

-2 -2 -7 1040.47 92.4829 8

-2 2 -7 1154.08 181.752 6

-2 -2 -7 1335.07 199.643 6

-2 -2 -8 2497.78 237.656 4

-2 -2 -8 2561.04 243.722 1

-2 2 -8 2096.50 197.517 2

2 -2 8 2401.77 198.053 1

-2 2 -8 2751.95 247.430 10

-2 -2 -8 2722.87 229.225 10

2 -2 8 2801.33 235.582 10

2 2 8 2759.80 235.152 10

-2 2 -8 2607.26 207.450 5

-2 -2 -8 2593.10 195.482 5

-2 2 -8 2796.53 310.826 6

-2 -2 -8 2705.94 297.462 6

-2 2 -9 203.104 54.2105 5

-2 2 -9 250.647 69.3938 2

-2 -2 -9 323.785 109.547 1

2 -2 9 297.309 82.4578 1

2 2 9 272.518 90.2535 1

-2 -2 -9 271.645 78.2341 10

2 -2 9 228.284 72.5725 10

-2 2 -9 170.868 53.4346 10

2 2 9 250.133 77.6280 10

-2 2 -9 328.006 121.255 6

-2 -2 -9 277.687 67.1753 5

-2 -2 -9 446.759 140.730 6

-2 -2 -10 195.035 56.7137 5

-2 2 -10 51.9279 27.1914 5

2 2 10 227.807 84.9881 1

2 -2 10 104.615 37.1646 1

-2 -2 -10 174.962 80.0941 1

-2 2 -10 142.278 58.7149 2

2 2 10 99.2694 55.7387 10

-2 2 -10 103.071 67.5369 10

2 -2 10 101.973 49.4273 10

-2 -2 -10 168.121 67.7816 10

-2 2 -10 200.230 86.4441 6

-2 -2 -10 131.304 74.4764 6

-2 2 -11 133.291 50.8228 5

-2 -2 -11 179.954 55.5865 5

2 2 11 107.621 59.5808 1

-2 2 -11 139.757 58.3962 2

2 2 11 115.522 68.7366 10

-2 -2 -11 165.523 73.7871 10

-2 2 -11 177.671 83.7741 10

2 -2 11 206.947 87.8257 10

-2 -2 -11 296.106 108.702 6

-2 2 -11 194.187 105.802 6

-2 2 -12 51.8821 51.8360 10

-2 -2 -12-6.76109 39.6996 10

-2 -2 -12-67.1450 47.5348 6

-2 2 -12 23.5414 49.1467 6

-2 2 -13-12.4455 46.2154 10

-2 -2 -13 74.1691 68.1844 10

-2 2 -13 171.753 118.740 6

-2 2 -14 142.942 73.4284 10

-2 -2 -14 138.648 68.4318 6

-2 2 -14 103.213 81.2872 6

2 3 -16 164.606 73.8281 10

2 -3 -16-14.4871 48.7652 10

2 -3 -15 41.8310 59.7030 10

2 3 -15-104.212 81.5348 10

2 -3 -15-152.000 95.2625 6

2 3 -15-98.2089 67.9777 6

2 3 -14 90.8800 81.8687 10

2 -3 -14-41.2513 43.1241 10

2 3 -14-40.6153 74.1864 6

2 -3 -14-156.585 108.467 6

2 -3 -13-21.4474 65.2659 10

2 3 -13-57.5032 68.4527 10

2 -3 -13-33.2056 42.3077 6

2 3 -13-55.0914 47.9360 6

-2 -3 12-25.5131 62.6117 4

-2 -3 12 43.0669 58.6290 1

2 -3 -12 44.9097 39.5559 10

2 3 -12 12.3162 58.9574 10

2 3 -12-26.3612 56.4828 6

2 -3 -12 81.4820 56.7540 6

-2 -3 11-20.0960 62.9794 4

2 -3 -11 15.8991 22.8505 2

-2 3 11 27.2377 74.3630 1

2 3 -11-28.9122 51.7844 10

2 -3 -11-11.5835 43.4026 10

2 3 -11-194.855 119.021 6

2 -3 -11 34.8958 38.1328 6

-2 -3 10 1914.93 206.352 1

-2 3 10 1879.41 242.528 1

2 -3 -10 1915.32 238.788 10

-2 -3 10 2255.54 257.890 10

2 3 -10 2112.42 258.516 10

-2 3 10 2219.47 268.774 10

2 3 -10 2054.97 280.033 6

2 -3 -10 2072.10 271.040 6

-2 3 9 257.608 91.9687 1

-2 -3 9 144.725 54.5164 1

2 3 -9 199.330 67.9175 10

-2 3 9 245.000 85.4262 10

2 -3 -9 179.681 73.4411 10

-2 -3 9 270.433 89.4072 10

2 -3 -9 225.198 89.2641 6

2 3 -9 170.518 88.8058 6

-2 -3 8 768.516 130.889 4

-2 -3 8 712.199 120.477 1

2 3 -8 740.982 132.101 10

-2 3 8 884.805 149.802 10

-2 -3 8 885.051 143.158 10

2 -3 -8 857.019 140.770 10

-2 -3 7 366.429 87.5737 4

-2 -3 7 344.776 81.9648 1

-2 3 7 423.197 113.240 1

2 -3 -7 361.680 94.9053 10

-2 3 7 414.899 99.0806 10

-2 -3 7 388.206 96.3848 10

2 3 -7 416.935 109.502 10

-2 3 7 348.962 73.9565 8

-2 -3 7 322.059 59.2669 5

-2 3 6 5552.23 325.361 4

-2 3 6 5080.28 328.610 1

-2 -3 6 5673.21 320.052 10

2 3 -6 5709.66 328.867 10

2 -3 -6 5753.20 317.216 10

-2 3 6 5544.63 324.254 10

-2 3 6 5643.34 349.211 6

-2 3 6 5103.60 258.769 8

-2 -3 6 5426.47 197.903 5

-2 3 5 133.456 53.4431 4

2 -3 -5 180.183 64.2737 4

-2 -3 5 120.727 30.7883 5

2 -3 -5 182.148 26.3406 5

-2 3 5 123.136 49.8704 1

-2 -3 5 199.803 62.0993 10

-2 3 5 143.247 54.3041 10

2 -3 -5 217.469 62.3107 10

-2 3 5 114.603 42.2697 8

-2 3 5 128.334 46.2628 6

2 -3 -5 211.143 64.3256 6

-2 3 4 43171.5 776.133 4

2 -3 -4 42024.7 441.674 5

2 -3 -4 45780.9 820.816 4

-2 3 4 40766.9 780.237 1

-2 3 4 43267.1 753.381 10

-2 -3 4 45810.6 755.328 10

2 -3 -4 44801.6 740.604 10

-2 3 4 42151.0 762.021 6

-2 3 4 43620.5 716.839 8

2 -3 -4 43742.0 739.024 6

-2 3 3 44509.2 505.447 5

2 -3 -3 45062.0 741.195 4

-2 3 3 48100.7 705.782 10

-2 -3 3 46351.2 670.451 10

2 -3 -3 44265.2 501.253 5

2 -3 -3 47336.4 661.955 6

-2 3 3 47487.0 692.947 6

2 -3 -2 235478. 1509.05 4

-2 3 2 241282. 1261.23 5

-2 -3 2 225224. 1274.67 10

2 -3 -2 230748. 1214.36 9

-2 3 2 230110. 1384.12 10

-2 3 2 235451. 1282.49 6

2 -3 -1 21156.6 385.994 4

-2 3 1 21414.9 378.265 4

-2 3 1 23331.3 414.299 5

-2 3 1 21506.8 352.756 3

2 -3 -1 22630.3 365.214 2

-2 3 1 21511.3 358.619 2

2 3 -1 21697.5 336.257 10

-2 3 1 21962.3 362.831 9

-2 -3 1 21172.4 323.047 10

-2 -3 1 21686.8 350.628 7

2 -3 -1 22030.6 400.569 5

-2 -3 0 126.529 31.9770 1

-2 3 0 120.980 32.0822 2

2 -3 0 145.478 38.2477 5

-2 3 0 128.443 26.0851 3

2 -3 0 113.046 23.0663 4

-2 3 0 121.756 22.6853 4

2 -3 0 113.865 24.8114 3

-2 3 0 106.250 26.4990 9

-2 -3 -1 27261.6 461.308 1

-2 3 -1 27235.1 495.160 2

2 3 1 25038.9 283.263 3

-2 -3 -1 24952.2 285.323 3

-2 3 -1 24824.3 364.624 3

2 -3 1 25162.4 490.566 5

2 3 1 23532.7 418.500 6

-2 3 -1 24746.8 472.852 5

-2 -3 -1 24030.5 405.010 6

-2 -3 -2 633.604 51.1475 3

-2 3 -2 666.336 91.0756 2

-2 -3 -2 707.563 86.6356 1

2 3 2 681.313 87.6907 1

2 3 2 597.528 51.8818 10

-2 3 -2 655.578 63.7859 10

2 -3 2 627.135 80.8237 5

-2 -3 -2 606.591 77.4085 6

2 3 2 666.856 84.7475 6

-2 3 -2 628.291 79.5452 5

-2 3 -3 49044.3 787.376 2

-2 -3 -3 45884.8 719.071 1

2 3 3 51099.1 512.174 2

2 3 3 48702.3 746.984 1

2 3 3 52101.1 611.383 10

-2 3 -3 53428.0 671.374 10

-2 -3 -3 52227.0 836.759 6

-2 3 -3 52125.4 765.007 5

2 3 3 53920.5 891.115 6

-2 3 -4 103.369 66.2639 5

2 3 4 110.492 69.6540 1

-2 3 -4 48.5331 28.0739 2

-2 3 -4 227.989 53.5035 1

2 -3 4 101.934 39.8535 1

-2 3 -4 153.470 53.3875 10

2 3 4 211.271 45.8584 10

2 -3 4 83.9647 46.1811 10

-2 -3 -4 150.127 44.1232 10

2 3 4 128.634 61.0321 6

-2 -3 -5 1351.13 144.294 4

-2 3 -5 1371.96 105.539 1

2 -3 5 1277.49 95.1363 1

2 3 5 1307.48 144.066 1

-2 3 -5 1349.66 148.329 2

-2 -3 -5 1274.06 143.971 1

2 -3 5 1412.38 130.382 10

-2 3 -5 1482.73 143.112 10

2 3 5 1351.12 130.172 10

-2 -3 -5 1401.94 125.732 10

-2 3 -5 1372.80 137.386 5

-2 -3 -6 3910.61 270.421 4

-2 -3 -6 3924.19 280.863 1

2 3 6 3968.75 275.673 1

-2 3 -6 3395.52 246.628 2

2 -3 6 3708.70 183.808 1

-2 3 -6 3785.41 200.802 1

2 -3 6 3862.37 237.245 10

-2 3 -6 3903.74 257.691 10

2 3 6 3895.62 244.120 10

-2 -3 -6 3837.73 229.695 10

-2 -3 -6 3526.52 188.477 8

-2 3 -6 4294.09 253.154 5

-2 3 -7 385.426 105.055 5

-2 -3 -7 468.729 117.012 4

-2 3 -7 447.957 109.941 2

-2 -3 -7 922.560 190.178 1

2 -3 7 379.762 71.2004 1

-2 3 -7 508.743 107.074 10

2 -3 7 455.248 94.8475 10

-2 -3 -7 409.671 86.7922 10

2 3 7 432.957 92.8742 10

-2 -3 -7 581.817 137.062 6

-2 -3 -7 466.468 94.8470 8

-2 -3 -8 34.7113 38.5231 4

-2 3 -8 11.0395 26.7073 5

2 -3 8-19.3404 31.2587 1

-2 -3 -8 7.05411 48.0452 1

-2 3 -8-21.4785 34.5431 2

-2 -3 -8 8.31556 24.5705 10

2 -3 8 62.4556 42.3148 10

2 3 8-5.97930 31.1970 10

-2 3 -8-0.30701 39.0150 10

-2 3 -8 56.9400 66.2477 6

-2 -3 -8 110.644 68.9392 6

-2 3 -9-13.6554 39.8313 5

-2 -3 -9 19.1768 35.8677 4

-2 3 -9-26.3005 36.0113 2

-2 -3 -9 61.2704 105.338 1

2 -3 9 30.2361 32.1767 1

2 3 9 18.8702 54.1242 1

-2 3 -9 7.91888 47.9013 10

2 -3 9 80.7000 45.5266 10

-2 -3 -9-5.82329 28.6624 10

2 3 9-13.7665 41.9916 10

-2 -3 -9 25.3843 32.0121 5

-2 -3 -9 1.65960 55.1698 6

-2 3 -9-15.7613 48.2826 6

-2 3 -10 343.029 80.0575 5

-2 3 -10 328.770 89.7752 2

2 3 10 451.756 112.474 1

-2 -3 -10 498.373 128.148 1

2 -3 10 312.781 72.8154 1

-2 -3 -10 440.473 113.635 10

2 -3 10 381.737 100.780 10

2 3 10 357.260 104.868 10

-2 3 -10 388.592 115.377 10

-2 -3 -10 415.490 77.9300 5

-2 -3 -10 545.066 151.767 6

-2 3 -10 369.649 133.273 6

-2 3 -11 707.927 111.312 5

2 3 11 647.875 149.424 1

-2 3 -11 635.467 129.185 2

2 -3 11 791.405 129.585 1

-2 3 -11 895.541 171.767 10

2 3 11 845.203 159.452 10

-2 -3 -11 915.581 163.018 10

2 -3 11 772.931 157.971 10

-2 3 -11 769.313 189.566 6

-2 -3 -11 953.306 217.091 6

-2 -3 -11 840.329 107.830 5

-2 -3 -12 282.448 99.5156 10

-2 3 -12 219.793 103.978 10

-2 3 -12 258.188 132.356 6

-2 -3 -12 181.452 98.6584 6

-2 3 -13-60.2990 62.1876 10

-2 -3 -13-35.1781 58.7641 10

-2 -3 -13 26.9718 62.8732 6

-2 3 -13-107.947 104.251 6

-2 3 -14-128.603 105.338 10

-2 -3 -14 73.9256 49.9827 6

-2 3 -14-187.275 109.757 6

2 4 -16 87.4758 90.6719 10

2 -4 -16 200.549 113.767 10

2 4 -15-93.7894 79.2024 10

2 -4 -15 61.2442 65.6433 10

2 4 -15 169.450 116.188 6

2 -4 -15 34.0020 65.5807 6

2 -4 -14 90.9694 61.1602 10

2 4 -14 69.1867 73.0690 10

2 4 -14 199.396 138.548 6

2 -4 -14-47.4294 72.7921 6

2 -4 -13 100.586 69.7097 10

2 4 -13 46.4713 47.7253 10

2 -4 -13 69.5781 78.3311 6

2 4 -13-77.5257 112.332 6

-2 -4 12 77.1867 50.4214 4

2 4 -12 24.2217 64.9065 10

2 -4 -12 7.83793 42.4545 10

2 -4 -12-112.513 95.9313 6

2 4 -12-121.979 72.8032 6

-2 -4 11 425.459 122.000 4

2 -4 -11 273.630 38.1831 2

-2 4 11 289.609 99.3157 1

2 -4 -11 421.967 121.936 10

2 4 -11 338.827 111.562 10

2 4 -11 520.045 154.076 6

2 -4 -11 422.298 135.938 6

-2 -4 10 853.756 140.643 1

2 -4 -10 860.397 69.6952 2

-2 4 10 853.082 170.578 1

2 4 -10 998.268 182.325 10

2 -4 -10 967.791 166.805 10

-2 4 10 834.473 161.134 10

-2 -4 10 1093.00 175.770 10

2 -4 -10 948.056 189.765 6

2 4 -10 977.561 198.844 6

-2 -4 9 153.953 62.2253 1

-2 4 9 343.749 109.189 1

-2 -4 9 261.102 83.4788 10

-2 4 9 189.968 67.5780 10

2 4 -9 193.057 80.2216 10

2 -4 -9 271.118 76.6840 10

2 -4 -9 275.362 98.6255 6

-2 -4 8 4075.59 305.015 4

-2 -4 8 3777.93 266.376 1

-2 -4 8 4517.53 328.502 10

2 4 -8 4531.70 338.658 10

-2 4 8 4258.54 330.399 10

2 -4 -8 4321.88 316.159 10

2 -4 -7 497.581 115.846 4

-2 -4 7 378.608 76.3914 1

-2 -4 7 377.463 85.3638 10

2 4 -7 468.026 104.391 10

-2 4 7 417.325 98.0697 10

2 -4 -7 419.143 93.3256 10

-2 4 7 411.756 104.726 6

-2 4 7 463.820 87.3714 8

-2 -4 7 391.262 63.9327 5

2 -4 -7 361.583 36.4368 5

-2 -4 6 12822.7 349.570 5

2 -4 -6 12156.9 243.537 5

-2 4 6 12672.2 513.306 4

2 -4 -6 12099.5 513.025 4

-2 4 6 12314.6 506.228 1

2 -4 -6 13377.6 485.329 10

-2 4 6 13315.5 512.189 10

-2 -4 6 13381.9 492.749 10

-2 4 6 12865.8 536.399 6

-2 4 6 12603.7 425.099 8

-2 -4 5 21616.5 449.880 5

2 -4 -5 22144.0 639.079 4

-2 4 5 21832.4 618.126 4

2 -4 -5 20792.9 348.738 5

-2 4 5 21269.2 630.757 1

-2 -4 5 22198.6 586.095 10

-2 4 5 22747.5 613.884 10

2 -4 -5 22758.1 588.664 10

-2 4 5 21128.9 543.181 8

-2 4 5 23139.3 650.067 6

2 -4 -4 10075.0 403.111 4

2 -4 -4 9376.06 246.796 5

-2 4 4 10494.4 417.667 1

2 -4 -4 10473.1 361.244 10

-2 -4 4 10178.3 355.334 10

-2 4 4 10609.5 379.229 10

-2 4 4 10248.3 387.040 6

2 -4 -4 9558.47 354.654 6

-2 4 4 9601.27 351.154 8

2 -4 -3 2450.02 190.984 4

-2 4 3 1236.81 105.789 5

2 -4 -3 1134.08 94.7312 5

2 -4 -3 2558.13 113.524 2

-2 -4 3 2070.84 149.035 10

-2 4 3 1655.13 142.967 10

2 -4 -3 1718.88 117.340 9

-2 4 3 1604.12 140.719 6

-2 4 3 1319.07 201.318 8

2 -4 -2 115288. 976.117 5

2 -4 -2 116359. 1103.59 4

-2 -4 2 115582. 947.259 10

2 -4 -2 117159. 905.160 9

-2 4 2 117245. 928.987 9

-2 -4 2 116561. 902.510 7

-2 4 1 71536.7 720.285 4

-2 4 1 70114.5 785.194 5

2 -4 -1 72285.3 825.337 5

2 -4 -1 69426.0 755.063 4

-2 4 1 79020.1 738.257 3

-2 4 1 70264.9 704.279 2

-2 4 1 70379.3 780.076 1

-2 -4 1 72666.5 727.407 1

2 -4 -1 68496.7 719.884 2

-2 4 1 76161.3 737.414 9

2 4 -1 74058.7 663.560 10

-2 -4 1 75061.2 698.252 7

-2 4 0 100233. 935.636 2

-2 -4 0 98878.7 867.144 1

2 -4 0 99161.7 720.541 4

2 -4 0 99611.5 800.392 3

-2 4 0 97302.5 784.551 3

2 4 0 99313.0 710.905 3

2 -4 0 96769.4 981.849 5

-2 4 -1 20903.0 486.363 2

-2 -4 -1 20728.0 427.717 1

-2 4 -1 21202.4 380.045 3

-2 4 -1 20805.7 474.306 5

2 4 1 22278.9 316.668 3

-2 -4 -1 21821.2 317.200 3

2 4 1 20863.2 420.415 6

2 -4 1 20611.5 482.478 5

-2 4 -1 21377.3 408.906 8

2 4 2 7403.27 282.034 1

-2 -4 -2 7349.54 277.859 1

-2 -4 -2 7298.05 192.892 3

-2 -4 -2 7182.93 271.727 6

2 -4 2 7437.79 298.512 5

-2 4 -2 7366.74 295.315 5

2 4 2 7161.95 291.775 6

-2 4 -2 7332.54 223.796 8

-2 -4 -3 7574.82 222.015 3

-2 -4 -3 6696.51 288.348 1

2 4 3 7036.41 300.676 1

2 4 3 7432.84 238.341 10

-2 4 -3 7677.87 267.762 10

-2 4 -3 7312.91 304.777 5

-2 -4 -3 7459.01 318.438 6

2 -4 3 7759.90 316.969 5

2 4 3 7514.16 340.020 6

-2 4 -3 7224.56 201.645 8

2 4 4 4538.41 155.094 2

2 4 4 4612.61 261.846 1

-2 4 -4 4690.96 140.054 1

-2 -4 -4 5152.01 276.902 1

2 4 4 4856.65 223.590 10

-2 4 -4 4788.35 239.924 10

-2 4 -4 4768.05 259.076 5

2 4 4 4783.38 301.677 6

-2 4 -5 12149.9 425.392 5

-2 4 -5 11725.9 269.028 1

2 4 5 12198.9 466.184 1

2 -4 5 11410.4 248.794 1

-2 -4 -5 11219.8 443.695 1

-2 4 -5 12297.2 423.198 10

-2 -4 -5 13125.3 379.552 10

2 4 5 12836.4 406.905 10

2 4 5 12960.3 552.872 6

-2 -4 -6 259.194 70.5411 4

-2 4 -6 145.030 51.2463 5

-2 -4 -6 155.798 58.6349 1

2 -4 6 184.564 39.7369 1

-2 -4 -6 150.564 43.2994 10

2 -4 6 290.277 67.0838 10

-2 4 -6 154.972 57.8976 10

2 4 6 107.663 40.3706 10

-2 -4 -6 104.484 37.6376 8

-2 -4 -7 4153.20 312.240 4

-2 4 -7 3941.69 257.767 5

-2 4 -7 3653.47 279.390 2

-2 -4 -7 4216.10 314.946 1

2 -4 7 3948.74 192.392 1

2 4 7 4332.63 283.610 10

-2 4 -7 4406.01 304.320 10

2 -4 7 4410.60 282.161 10

-2 -4 -7 4416.71 267.834 10

-2 -4 -7 4528.00 357.439 6

-2 -4 -7 3671.18 218.505 8

-2 4 -8 1871.81 185.574 5

-2 -4 -8 1946.90 228.287 4

2 -4 8 2081.20 154.130 1

2 4 8 2087.21 228.911 1

-2 -4 -8 2060.02 238.191 1

-2 4 -8 1888.69 205.593 2

-2 4 -8 2288.33 231.548 10

2 -4 8 2206.84 213.663 10

2 4 8 2192.21 218.150 10

-2 -4 -8 2536.37 220.112 10

-2 -4 -8 2020.80 161.778 8

-2 -4 -8 2347.26 272.986 6

-2 -4 -9 2172.62 254.211 4

-2 4 -9 2141.25 201.744 5

-2 4 -9 2057.24 217.880 2

-2 -4 -9 2285.28 262.324 1

2 4 9 2267.64 249.262 1

2 -4 9 2403.10 180.972 1

2 4 9 2437.02 244.035 10

-2 -4 -9 2425.98 229.543 10

-2 4 -9 2302.98 252.265 10

2 -4 9 2671.66 250.697 10

-2 4 -9 2583.51 323.627 6

-2 -4 -9 2848.85 325.233 6

-2 -4 -10-1.47476 36.2110 4

-2 4 -10 61.9741 40.9758 5

2 4 10 14.3088 36.4753 1

-2 4 -10-82.4804 69.8772 2

2 -4 10 11.8136 26.3630 1

-2 -4 -10-88.2716 63.8728 1

2 4 10-1.95164 26.1157 10

2 -4 10 67.4701 49.4278 10

-2 -4 -10-35.6601 50.8628 10

-2 4 -10 15.9876 41.6929 10

-2 4 -10-7.27445 47.5668 6

-2 -4 -10 24.9729 58.1290 6

-2 -4 -10-15.6952 20.7770 5

-2 -4 -11 81.3115 38.9780 5

-2 4 -11 81.4547 41.6227 5

2 -4 11 24.6668 38.8576 1

-2 -4 -11 136.358 73.0713 1

-2 4 -11 10.8756 49.2430 2

-2 -4 -11 40.2136 59.9150 10

2 4 11 70.4643 56.0755 10

-2 4 -11 82.0254 86.1219 10

-2 -4 -11 107.177 95.7561 6

-2 4 -11 33.8634 78.7954 6

-2 -4 -12 11.5506 48.9884 10

-2 4 -12-77.2466 56.7853 10

-2 -4 -12 94.4742 77.8763 6

-2 4 -12-106.817 93.0734 6

-2 4 -13 577.876 154.530 10

-2 -4 -13 497.186 162.864 6

-2 4 -13 488.723 196.916 6

-2 4 -14 255.035 130.462 10

-2 4 -14 35.4191 64.2382 6

-2 -4 -14 99.2027 108.474 6

2 5 -16-4.94662 95.8314 10

2 5 -15 107.607 100.165 10

2 -5 -15 65.1295 95.1132 6

2 5 -15-1.48674 55.1971 6

2 -5 -14 151.806 92.5039 10

2 5 -14 123.918 66.3838 10

2 5 -14 159.892 113.218 6

2 -5 -14 271.644 126.584 6

2 5 -13 436.194 149.359 10

2 -5 -13 520.025 141.804 10

2 -5 -13 453.494 148.493 6

2 5 -13 543.471 171.901 6

-2 -5 12-99.9615 73.4656 4

2 -5 -12 96.7480 90.2352 10

2 5 -12 127.111 82.9433 10

2 -5 -12 51.5903 69.7887 6

2 5 -12 98.9849 86.5223 6

-2 -5 11 624.393 136.938 4

-2 -5 11 389.521 87.6343 1

2 -5 -11 420.786 123.847 10

2 5 -11 499.482 133.741 10

2 -5 -11 586.337 164.941 6

2 5 -11 428.718 138.662 6

-2 5 10 150.654 75.9053 1

-2 -5 10 170.919 59.1539 1

2 -5 -10 404.541 109.525 10

2 5 -10 320.417 115.736 10

-2 5 10 232.466 90.3259 10

2 -5 -10 259.660 93.7084 6

-2 5 9 999.466 185.308 1

-2 -5 9 1132.00 144.950 1

2 -5 -9 1339.04 188.455 10

-2 5 9 1203.08 190.194 10

2 5 -9 1416.76 201.564 10

-2 -5 9 1290.84 185.933 10

2 -5 -9 1205.93 189.563 6

2 -5 -8 3396.51 315.473 4

-2 -5 8 3285.91 241.557 1

-2 5 8 3274.68 311.852 1

-2 -5 8 3637.19 291.855 10

2 5 -8 4220.75 335.332 10

2 -5 -8 3796.61 291.269 10

-2 5 8 3915.36 321.701 10

2 -5 -8 3528.63 126.486 5

-2 -5 8 3729.54 209.851 5

-2 5 8 3855.81 346.149 6

2 -5 -8 3756.29 318.283 6

2 -5 -7 1801.27 105.142 5

2 -5 -7 2035.35 222.958 4

-2 -5 7 1654.69 157.237 1

-2 -5 7 1945.04 200.795 10

2 5 -7 2131.45 223.930 10

2 -5 -7 1940.59 196.685 10

-2 5 7 2039.99 216.233 10

-2 5 7 1934.18 227.483 6

-2 -5 7 1996.99 154.472 5

2 -5 -6 1822.82 114.556 5

2 -5 -6 1926.20 222.476 4

-2 -5 6 1653.68 141.706 5

-2 5 6 1612.88 190.182 1

2 -5 -6 1948.69 212.508 1

-2 5 6 2029.28 198.807 10

-2 -5 6 1776.47 177.197 10

2 -5 -6 1744.03 179.852 10

-2 5 6 1910.41 179.443 8

-2 5 6 2006.58 217.579 6

2 -5 -5 31.3752 25.6171 4

-2 -5 5 39.7277 18.2706 5

2 -5 -5 7.59193 16.7687 5

2 -5 -5 39.5461 11.1276 2

2 -5 -5-31.0104 31.5547 1

-2 -5 5 47.2861 31.3125 10

2 -5 -5 16.6622 29.5245 10

-2 5 5 35.7069 25.6741 10

-2 5 5 36.8592 38.7525 8

-2 5 5 69.7057 40.8861 6

2 -5 -4 119691. 1034.83 5

-2 -5 4 120805. 1184.51 5

2 -5 -4 127963. 1467.19 4

2 -5 -4 123594. 735.624 2

2 -5 -4 129245. 1520.50 1

-2 -5 4 136329. 1316.85 10

-2 5 4 131490. 1373.71 10

2 -5 -4 133156. 1122.88 9

2 -5 -4 131385. 1351.89 6

-2 5 4 132963. 1431.74 6

-2 5 4 126625. 1328.74 8

2 -5 -3 425.435 80.5213 4

2 -5 -3 314.537 57.7769 5

2 -5 -3 464.806 97.6062 1

2 -5 -3 461.905 56.4683 2

2 -5 -3 529.941 67.0387 9

-2 -5 3 521.961 75.9694 10

-2 5 3 474.127 77.3231 10

-2 5 3 479.106 98.1025 8

-2 5 3 549.959 81.5495 6

2 -5 -2 65122.2 813.596 5

-2 5 2 67470.6 846.453 5

2 -5 -2 56306.4 807.848 4

2 -5 -2 64074.8 906.707 1

-2 5 2 60019.5 636.722 2

-2 5 2 58250.4 695.913 9

2 -5 -2 56690.2 659.275 9

-2 -5 2 56592.8 656.408 7

2 -5 -1 12305.2 332.471 4

-2 5 1 12491.2 367.516 5

2 -5 -1 12297.1 373.236 5

-2 5 1 13355.5 333.986 3

-2 5 1 12165.7 349.338 1

-2 5 1 11933.2 318.625 2

-2 -5 1 12109.0 306.594 1

-2 5 1 13133.4 326.887 9

-2 -5 1 12528.2 302.734 7

-2 5 0 22.9193 23.6012 1

2 5 0 25.5853 20.8573 3

-2 -5 0 30.3466 27.0182 1

-2 5 0 14.1767 24.5426 2

2 -5 0 14.6591 27.3009 5

2 -5 0 18.4516 18.7561 4

-2 5 0 30.3071 24.7440 3

2 -5 0 20.4800 21.5423 3

-2 5 0 51.2498 30.2621 8

-2 5 -1 21084.0 318.934 1

-2 -5 -1 20668.3 455.395 1

-2 5 -1 23756.7 443.256 3

2 5 1 24007.9 378.684 3

-2 5 -1 21519.9 512.329 5

-2 -5 -1 23003.0 369.716 3

2 -5 1 22653.5 536.471 5

-2 5 -1 21535.6 447.707 8

-2 -5 -2 4841.85 236.267 1

2 5 2 4792.15 238.452 1

-2 5 -2 5166.06 259.400 5

-2 -5 -2 5308.38 177.724 3

2 5 2 5200.38 257.328 6

2 -5 2 5438.59 272.823 5

-2 5 -2 5087.96 204.416 8

-2 -5 -3 15381.2 336.557 3

-2 5 -3 14720.3 455.212 5

2 5 3 14599.6 449.850 1

-2 -5 -3 13645.5 429.441 1

2 5 3 14552.3 352.783 10

-2 5 -3 15953.5 403.651 10

-2 -5 -3 15103.3 459.284 6

-2 5 -3 14751.0 324.280 8

2 -5 3 15717.0 480.359 5

2 5 3 15864.6 513.429 6

-2 5 -4 5896.53 295.774 5

-2 -5 -4 6227.83 235.720 3

2 -5 4 6210.70 144.152 4

2 5 4 5926.93 164.704 2

2 5 4 5999.18 314.303 1

-2 -5 -4 5688.98 301.480 1

2 5 4 6348.54 260.174 10

-2 -5 -4 6227.97 140.306 9

-2 5 -4 5985.96 276.136 10

2 -5 4 6432.06 315.701 5

2 5 4 5992.08 346.910 6

-2 5 -5 857.998 117.825 5

2 5 5 610.168 113.253 1

-2 -5 -5 841.187 137.735 1

2 5 5 759.986 101.632 10

-2 5 -5 724.952 107.406 10

2 5 5 652.097 125.035 6

-2 -5 -6 1759.08 194.618 4

-2 5 -6 1849.97 175.774 5

2 -5 6 1710.18 95.4800 1

-2 -5 -6 1773.90 192.902 1

-2 5 -6 1794.26 180.332 10

2 5 6 2035.84 180.561 10

2 5 6 1750.54 222.246 6

-2 -5 -6 1624.00 148.453 8

-2 -5 -7 413.033 119.948 4

-2 5 -7 418.184 101.475 5

-2 -5 -7 636.692 166.604 1

2 -5 7 301.619 55.2045 1

2 5 7 528.420 142.137 1

-2 5 -7 384.192 92.5712 10

2 5 7 340.327 75.2755 10

-2 -5 -7 251.739 94.1351 6

-2 -5 -7 383.547 95.7313 8

-2 -5 -8 4911.72 366.805 4

-2 5 -8 5305.39 318.003 5

2 5 8 5273.41 377.766 1

-2 5 -8 4904.93 348.559 2

2 -5 8 5259.93 223.263 1

-2 -5 -8 5281.30 387.696 1

-2 5 -8 5667.92 374.799 10

2 5 8 5654.69 356.792 10

-2 -5 -8 4926.71 276.400 8

-2 -5 -8 6025.49 439.612 6

-2 -5 -9 846.054 158.675 4

-2 5 -9 846.741 135.560 5

-2 5 -9 731.452 136.396 2

2 5 9 761.739 150.464 1

2 -5 9 779.639 96.7528 1

-2 -5 -9 861.229 168.677 1

-2 5 -9 924.050 162.142 10

2 5 9 860.998 149.272 10

-2 -5 -9 961.558 194.450 6

-2 -5 -10 1591.56 236.533 4

-2 5 -10 1682.92 180.673 5

-2 5 -10 1407.31 198.391 2

2 -5 10 1689.28 148.123 1

-2 -5 -10 1485.03 232.784 1

2 5 10 1674.61 233.824 1

2 5 10 1674.27 217.815 10

-2 5 -10 1953.42 247.442 10

-2 -5 -10 1681.75 254.696 6

-2 5 -10 1714.40 290.940 6

-2 5 -11 722.090 120.914 5

-2 5 -11 578.163 142.348 2

-2 -5 -11 784.431 172.461 1

2 5 11 649.329 144.048 10

-2 5 -11 742.658 168.107 10

-2 -5 -11 560.167 98.1917 5

-2 -5 -11 707.431 179.922 6

-2 5 -11 650.800 182.485 6

-2 5 -12 70.8795 40.1042 10

-2 -5 -12 1.58926 44.5091 6

-2 5 -13-44.1331 63.3127 10

-2 -5 -13 158.744 113.958 6

-2 5 -13 71.9936 100.769 6

-2 5 -14-313.587 137.370 10

2 6 -16 82.7128 118.108 10

2 6 -15-45.8999 77.0706 10

2 -6 -15 168.165 109.833 6

2 6 -15-82.9337 105.022 6

2 6 -14 44.4622 54.8058 10

2 6 -14-25.8834 56.5531 6

2 -6 -14 22.3039 63.4617 6

-2 -6 13-19.7654 61.0164 4

2 6 -13-17.7392 44.6869 10

2 6 -13-32.7547 104.819 6

2 -6 -13-71.1244 68.3419 6

-2 -6 12 178.995 85.4862 4

-2 -6 12 77.8457 34.2727 1

2 6 -12 227.634 96.6968 10

2 -6 -12 212.900 101.039 10

2 6 -12 169.310 101.669 6

2 -6 -12 119.895 77.1393 6

-2 -6 11 244.222 79.1105 4

-2 -6 11 221.737 68.8516 1

2 -6 -11 230.808 96.9555 10

2 6 -11 98.4777 50.2431 10

2 6 -11 194.989 80.7930 6

2 -6 -11 217.272 100.826 6

-2 6 10 2118.49 270.647 1

-2 -6 10 1783.56 183.715 1

2 6 -10 2355.46 282.094 10

-2 6 10 2528.29 288.190 10

2 -6 -10 2242.21 257.436 10

2 -6 -10 2025.52 266.977 6

-2 -6 9 942.696 127.148 1

-2 6 9 1127.14 189.785 1

-2 6 9 1201.90 189.773 10

2 -6 -9 1142.20 174.417 10

2 6 -9 1161.69 193.397 10

-2 -6 9 1147.26 173.136 10

-2 -6 9 1011.17 117.436 5

2 -6 -9 976.435 178.627 6

2 -6 -9 1016.82 71.7765 5

2 -6 -8 3064.66 310.770 4

2 -6 -8 3230.96 152.659 5

-2 6 8 3612.57 319.568 1

-2 -6 8 3295.38 223.160 1

2 6 -8 3620.99 317.877 10

-2 -6 8 3391.57 283.406 10

-2 6 8 3496.99 307.228 10

2 -6 -8 3724.34 293.866 10

-2 6 8 3479.93 337.195 6

-2 -6 8 3348.37 222.561 5

2 -6 -8 3332.13 297.641 6

2 -6 -7 7208.51 445.256 4

2 -6 -7 6423.24 236.074 5

-2 -6 7 7081.78 329.529 5

-2 6 7 6549.15 418.289 1

2 -6 -7 7216.84 442.313 1

-2 -6 7 7587.40 396.867 10

-2 6 7 7145.30 416.158 10

2 6 -7 7631.04 431.293 10

-2 6 7 7599.78 462.116 6

-2 -6 6 5621.61 278.111 5

2 -6 -6 5392.13 226.782 5

2 -6 -6 5653.84 369.923 4

2 -6 -6 5422.72 368.686 1

-2 -6 6 5876.60 332.537 10

-2 6 6 5867.30 350.520 10

-2 6 6 6094.50 386.228 6

-2 6 6 5291.25 312.315 8

2 -6 -5 2484.77 228.813 4

-2 -6 5 2415.18 182.471 5

2 -6 -5 2492.06 159.238 5

2 -6 -5 2680.80 239.402 1

-2 6 5 2745.45 218.459 10

2 -6 -5 2786.32 172.381 9

-2 -6 5 2801.79 209.543 10

-2 6 5 2414.29 208.780 8

-2 6 5 2896.83 239.693 6

-2 -6 4 5963.76 282.955 5

2 -6 -4 5652.88 245.830 5

2 -6 -4 6033.09 343.024 4

2 -6 -4 7118.57 375.501 1

-2 -6 4 6038.14 285.060 10

2 -6 -4 6480.14 253.639 9

-2 6 4 6416.28 312.080 10

-2 6 4 6383.89 327.986 8

-2 6 4 6097.18 320.469 6

2 -6 -3 224.842 87.1825 4

2 -6 -3 51.0375 38.1106 5

-2 6 3 92.4991 37.7722 9

2 -6 -3 144.968 38.5655 9

-2 -6 3 233.906 57.2507 10

-2 6 3 117.848 107.698 8

-2 6 3 93.9146 49.6656 6

-2 -6 3 130.644 42.5640 7

-2 6 2 305.469 73.0475 5

2 -6 -2 258.089 55.3759 4

-2 6 2 212.790 45.8266 3

2 -6 -2 210.999 60.4534 5

-2 6 2 271.367 71.1275 1

-2 6 2 204.020 46.7843 2

-2 -6 2 271.730 48.7386 1

2 -6 -2 283.417 69.7395 1

-2 6 2 206.622 45.8684 9

-2 -6 2 262.790 48.6667 7

2 -6 -2 200.306 46.3117 9

2 6 -2 247.346 47.8451 7

2 -6 -1 6620.08 292.621 5

2 -6 -1 6621.51 259.735 4

-2 6 1 6506.03 287.551 5

-2 6 1 7534.58 266.453 3

2 -6 -1 6819.70 283.125 1

-2 -6 1 6447.88 239.343 1

-2 6 1 6471.87 268.407 1

2 6 -1 6601.19 249.625 1

-2 6 1 6303.11 257.824 2

-2 6 1 7543.05 264.119 9

2 6 -1 7513.44 256.015 7

2 -6 -1 6589.52 302.850 8

-2 6 0 2516.04 141.573 1

2 6 0 2334.11 154.636 1

-2 -6 0 2207.52 147.091 1

2 -6 0 2166.51 140.850 3

-2 6 0 2183.28 144.610 3

2 6 0 2379.54 137.710 3

2 -6 0 2519.12 185.167 5

-2 6 0 2472.15 170.513 8

-2 -6 -1 29353.7 577.974 1

-2 6 -1 31449.8 426.774 1

2 6 1 36563.6 522.934 3

2 -6 1 34955.4 721.931 5

-2 6 -1 36310.3 592.055 3

-2 -6 -1 35446.3 508.850 3

-2 6 -1 31823.6 590.613 8

-2 -6 -2 16783.0 353.058 3

-2 6 -2 19178.2 530.236 5

2 6 2 17157.1 364.959 3

-2 -6 -2 20036.3 512.265 1

-2 6 -2 17469.9 430.084 3

2 6 2 16851.6 230.946 9

-2 6 -2 17346.9 226.467 7

2 6 2 16733.7 482.013 6

2 -6 2 18154.4 533.908 5

-2 6 -2 18725.9 445.191 8

-2 6 -3 128.182 47.9628 5

2 -6 3 94.7447 47.4372 5

-2 -6 -3 79.8603 26.4326 3

-2 -6 -3 111.602 53.9869 1

-2 6 -3 120.971 39.0049 10

2 6 3 81.4162 35.4843 6

2 -6 4 274.870 31.5072 4

-2 -6 -4 202.193 43.5784 3

-2 6 -4 359.785 78.0910 5

-2 -6 -4 263.774 84.6488 1

2 6 4 273.232 73.5463 1

-2 -6 -4 280.082 29.3300 9

2 6 4 212.140 47.2001 10

-2 6 -4 256.933 58.4640 10

2 6 4 285.658 84.4349 6

2 -6 4 251.476 69.7481 5

2 -6 5 354.999 42.7059 4

-2 -6 -5 425.512 76.4283 3

-2 6 -5 489.453 102.098 5

-2 6 -5 536.791 97.0117 10

2 6 5 400.524 79.2282 10

-2 -6 -5 484.686 50.0307 9

2 6 5 344.584 103.679 6

2 -6 5 366.792 37.0406 7

2 -6 5 590.661 119.826 5

-2 6 -6 1446.91 161.163 5

-2 -6 -6 1306.25 170.919 4

-2 -6 -6 1458.91 187.932 1

-2 -6 -6 1527.12 96.7170 9

2 6 6 1573.01 161.683 10

-2 6 -6 1589.00 173.330 10

-2 -6 -6 1312.10 142.754 8

-2 -6 -6 1462.92 186.835 6

2 6 6 1570.45 212.944 6

-2 6 -7 15.6497 25.5844 5

-2 -6 -7 17.8423 42.8275 4

-2 -6 -7-62.2633 64.3467 1

2 6 7-36.9490 48.7764 1

2 6 7 11.1243 26.5118 10

-2 6 -7-33.0532 54.7984 10

-2 -6 -7-44.2781 43.0779 6

-2 -6 -7-6.61778 21.1349 8

2 6 7-43.9014 56.2794 6

-2 6 -8 7258.24 390.663 5

-2 -6 -8 7042.18 451.857 4

2 6 8 7893.75 472.107 1

-2 -6 -8 7663.31 481.841 1

2 -6 8 6785.84 210.864 1

-2 6 -8 7907.92 453.077 10

2 6 8 7740.03 420.630 10

-2 -6 -8 6898.36 351.112 8

-2 -6 -8 7606.39 497.821 6

-2 -6 -9 3814.21 357.606 4

-2 6 -9 3704.49 280.648 5

-2 6 -9 3266.78 304.158 2

2 -6 9 3848.96 176.310 1

2 6 9 3733.34 360.448 1

-2 -6 -9 4230.29 378.489 1

-2 6 -9 4339.81 358.999 10

2 6 9 3873.62 318.813 10

-2 -6 -9 4096.24 385.536 6

-2 6 -10 730.245 127.209 5

-2 -6 -10 838.728 174.236 4

-2 6 -10 776.067 154.131 2

-2 -6 -10 868.306 186.026 1

2 -6 10 901.737 93.3741 1

2 6 10 991.511 171.336 10

-2 6 -10 813.132 167.212 10

-2 -6 -10 811.145 179.605 6

-2 6 -11 39.7900 26.0380 5

-2 -6 -11-39.7561 102.114 1

-2 6 -11 43.8024 65.9170 2

2 6 11-83.7260 82.7937 10

-2 6 -11 18.3627 51.4586 10

-2 -6 -11 11.5897 31.2135 5

-2 -6 -11 159.156 96.1981 6

-2 6 -11-177.962 115.283 6

-2 -6 -12 61.1978 82.9905 1

-2 6 -12 34.2762 72.7113 10

-2 -6 -12 80.0819 77.0218 6

-2 6 -12 97.0717 111.431 6

-2 6 -13 646.929 179.066 10

-2 -6 -13 518.842 164.408 6

-2 6 -14 170.722 120.547 10

2 7 -16-49.7663 49.3130 10

2 7 -15-5.02385 42.3657 10

2 -7 -15 101.796 83.7501 6

2 7 -15-21.6448 58.7572 6

2 7 -14 50.5973 55.6160 10

2 7 -14-13.3772 75.9638 6

2 -7 -14-108.184 115.545 6

-2 -7 13 93.3585 74.0665 4

2 7 -13 117.908 94.5513 10

2 -7 -13-165.858 105.029 6

2 7 -13-51.3489 61.1445 6

-2 -7 12 743.772 155.257 4

-2 -7 12 543.748 104.113 1

2 7 -12 603.872 157.719 10

2 -7 -12 606.072 169.341 6

2 7 -12 535.191 164.445 6

-2 -7 11 342.539 120.466 4

-2 -7 11 250.518 73.1976 1

2 7 -11 427.669 134.996 10

2 -7 -11 214.200 92.4386 6

-2 -7 10 1877.43 171.085 1

-2 7 10 2120.33 268.676 10

2 7 -10 1991.83 268.868 10

2 -7 -10 1891.05 92.6226 5

2 -7 -10 2116.81 271.499 6

2 -7 -9 2176.55 273.439 4

2 -7 -9 1950.91 125.729 5

-2 -7 9 1793.55 172.171 1

-2 7 9 1934.13 245.590 1

-2 7 9 2329.78 269.794 10

2 7 -9 2228.39 266.856 10

-2 -7 9 2216.87 244.550 10

2 -7 -9 1892.75 241.112 6

-2 -7 9 2250.77 193.906 5

-2 -7 8 701.886 106.476 5

2 -7 -8 675.744 81.8352 5

2 -7 -8 665.650 139.403 4

-2 -7 8 713.452 101.721 1

-2 7 8 662.795 148.144 1

2 -7 -8 794.212 153.914 1

-2 7 8 799.301 150.032 10

2 7 -8 708.029 151.306 10

-2 -7 8 835.235 143.783 10

-2 7 8 699.457 151.068 6

2 -7 -8 799.463 151.939 6

-2 -7 7 110.591 48.3479 5

2 -7 -7 94.5531 45.7617 4

2 -7 -7 89.6834 31.7797 5

-2 7 7 170.028 77.0584 1

2 -7 -7 116.192 71.3225 1

2 7 -7 121.737 63.0225 10

2 -7 -7 105.778 41.4567 9

-2 7 7 224.404 80.1649 10

-2 -7 7 95.4159 33.7736 10

-2 7 7 166.226 65.6952 6

-2 -7 6 1635.24 161.054 5

2 -7 -6 1438.72 127.388 5

2 -7 -6 1656.71 198.931 4

2 -7 -6 1601.80 201.265 1

-2 7 6 1598.26 181.767 10

2 -7 -6 1557.85 137.728 9

-2 -7 6 1532.79 165.179 10

-2 7 6 1741.15 206.905 6

2 -7 -5 3774.64 217.529 5

2 -7 -5 4087.13 298.812 4

-2 -7 5 3825.71 245.834 5

2 -7 -5 3999.20 309.476 1

-2 -7 5 4152.46 261.961 10

2 -7 -5 4202.52 218.740 9

-2 7 5 4176.97 276.231 10

-2 7 5 3835.34 282.886 6

-2 7 5 3976.92 274.504 8

2 -7 -4 1295.39 130.666 5

2 -7 -4 1401.07 163.512 4

-2 -7 4 1512.48 151.101 5

2 -7 -4 1380.89 176.276 1

-2 -7 4 1630.55 150.111 10

-2 7 4 1310.94 145.057 10

2 -7 -4 1782.24 133.668 9

-2 7 4 1357.53 158.668 8

-2 7 4 1372.14 153.704 6

-2 -7 4 1354.60 117.423 7

2 -7 -3 3123.72 227.740 4

2 -7 -3 2946.69 200.913 5

2 -7 -3 3222.72 240.937 1

-2 -7 3 3161.00 165.011 1

-2 7 3 3060.66 151.948 2

-2 7 3 3295.26 188.091 9

2 -7 -3 3295.22 176.879 9

-2 7 3 2861.81 222.035 8

2 -7 -3 3363.40 239.627 8

-2 -7 3 3213.79 172.389 7

2 -7 -2 6280.86 303.507 5

-2 7 2 7219.05 321.889 5

2 -7 -2 6100.89 288.159 4

-2 7 2 5554.78 256.292 3

2 -7 -2 6601.25 333.489 1

-2 -7 2 6084.12 231.661 1

-2 7 2 6304.61 311.671 1

-2 7 2 5979.33 240.733 2

2 7 -2 5678.51 249.793 10

-2 7 2 5584.60 242.428 9

2 -7 -2 6192.50 321.146 8

-2 -7 2 5823.14 230.955 7

2 -7 -2 5993.75 244.015 9

2 7 -2 5961.77 241.779 7

2 -7 -1 6196.11 304.190 5

-2 7 1 6660.90 315.161 5

-2 7 1 6161.47 259.678 3

2 -7 -1 6430.53 288.243 1

2 7 -1 6067.14 251.661 1

-2 -7 1 5973.92 241.992 1

-2 7 1 6184.37 276.812 1

-2 7 1 6424.49 261.180 9

2 7 -1 6365.66 250.963 7

2 -7 -1 6079.43 309.983 8

2 -7 0 31.8191 31.7281 1

-2 7 0 71.6595 37.0271 1

2 7 0 10.1360 28.5050 1

-2 -7 0 78.4608 39.5033 1

2 7 0 38.2533 26.1676 3

-2 7 0 65.2098 36.1455 3

2 -7 0 54.8717 42.0924 3

2 -7 0 19.1777 27.7532 5

2 -7 0 2.98723 34.1111 8

-2 7 0 63.8875 46.8150 8

-2 -7 -1 29017.7 601.847 1

-2 7 -1 29934.4 445.937 1

-2 -7 -1 34357.7 549.315 3

2 7 1 33764.3 552.578 3

-2 7 -1 34164.5 619.872 3

2 -7 1 32366.3 736.315 5

2 -7 1 32377.1 667.074 8

-2 7 -1 31864.1 634.565 8

-2 7 -2 23731.0 535.170 3

-2 -7 -2 23880.7 459.284 3

-2 7 -2 25552.4 657.231 5

2 7 2 22982.6 455.600 3

-2 7 -2 26552.8 307.562 1

-2 -7 -2 25050.3 609.458 1

2 7 2 23079.5 322.924 9

2 7 2 23472.6 593.647 6

-2 7 -2 23882.9 316.177 7

-2 7 -2 27270.0 560.304 8

2 -7 2 25628.3 688.949 5

-2 7 -3 24259.6 642.871 5

-2 -7 -3 25955.6 488.535 3

2 -7 3 26668.3 704.178 5

2 7 3 26108.1 691.106 6

-2 -7 -4 23220.0 490.327 3

-2 -7 -4 25045.8 698.145 1

2 7 4 21956.2 527.820 10

-2 7 -4 23520.2 591.614 10

2 7 4 23942.6 734.321 6

2 -7 4 24141.8 690.976 5

-2 -7 -5 9546.05 334.542 3

2 -7 5 9246.56 221.410 4

-2 7 -5 9123.84 416.728 5

-2 -7 -5 9411.00 450.645 1

2 7 5 8249.18 169.263 2

-2 7 -5 9882.83 411.918 10

2 7 5 9222.54 372.538 10

-2 -7 -5 9294.09 202.115 9

2 -7 5 10351.3 467.646 5

2 7 5 9723.16 509.884 6

2 -7 5 9449.10 181.623 7

2 -7 6 10.2919 15.9999 4

-2 -7 -6-2.62310 25.5156 3

-2 7 -6 6.39161 24.4165 5

-2 -7 -6-32.0355 36.2693 1

2 7 6 44.4448 37.1082 1

-2 7 -6-18.7965 34.9748 10

2 7 6-21.7610 34.7670 10

-2 -7 -6-0.52801 12.3418 9

2 -7 6-5.35610 10.2637 7

-2 -7 -6-16.0303 29.8324 8

-2 -7 -6 43.8247 46.2232 6

2 -7 6-3.83535 26.1912 5

2 7 6 53.2072 65.7725 6

-2 7 -7 162.346 95.1398 5

-2 -7 -7 107.761 74.8098 4

2 7 7 67.7512 85.6057 1

-2 -7 -7 246.563 162.733 1

2 7 7 85.5862 53.3274 10

-2 -7 -7 73.3951 27.3154 9

-2 7 -7 62.2590 60.0658 10

-2 -7 -7-0.62027 44.4987 6

-2 -7 -7 193.564 116.139 8

2 7 7 53.5415 40.3096 6

-2 7 -8 834.948 135.282 5

-2 -7 -8 792.505 149.452 4

-2 -7 -8 851.704 157.440 1

2 7 8 882.684 162.870 1

-2 -7 -8 1016.48 93.6680 9

2 7 8 964.583 149.942 10

-2 7 -8 885.145 159.817 10

-2 -7 -8 1067.74 187.620 6

-2 -7 -8 816.731 123.660 8

-2 7 -9 2377.62 236.228 5

-2 -7 -9 2441.80 290.915 4

2 7 9 2329.22 285.053 1

-2 7 -9 2085.86 248.244 2

-2 -7 -9 2502.10 289.291 1

-2 7 -9 2483.69 273.717 10

-2 -7 -9 2661.63 167.456 9

2 7 9 2373.45 251.856 10

-2 -7 -9 2433.79 220.048 8

-2 -7 -9 2786.57 316.120 6

-2 7 -10-10.9519 33.5384 5

-2 7 -10 20.0129 34.8837 2

-2 -7 -10-110.209 73.1308 1

-2 7 -10 37.3608 37.0705 10

-2 -7 -10-19.9865 24.2872 9

2 7 10 37.4614 61.3353 10

-2 -7 -10-37.1991 70.4268 6

-2 7 -11 1062.73 161.231 5

-2 -7 -11 1118.24 222.819 1

-2 7 -11 1229.76 218.171 10

-2 -7 -11 1095.44 121.116 9

2 7 11 1074.91 191.117 10

-2 7 -11 1437.19 284.168 6

-2 -7 -11 1036.03 217.343 6

-2 -7 -12-56.9515 57.7781 1

-2 7 -12 177.448 105.343 10

-2 -7 -12-139.342 121.609 6

-2 7 -12-76.8614 88.9363 6

-2 7 -13-170.731 108.757 10

-2 -7 -13 32.9938 59.9579 6

-2 7 -14-145.077 108.248 10

2 8 -16-45.0127 60.0437 10

2 8 -15 348.474 150.794 10

2 8 -14 189.821 115.746 10

2 8 -14-45.7727 105.808 6

2 -8 -14 61.7377 55.8030 6

-2 -8 13 47.9166 35.6022 4

2 8 -13 122.024 77.3600 10

2 -8 -13 67.8002 64.5792 9

2 8 -13 127.403 95.9229 6

2 -8 -13 129.072 92.9179 6

-2 -8 12 2417.93 276.295 4

2 8 -12 1988.80 293.218 10

2 -8 -12 2343.88 231.284 9

2 -8 -12 2199.08 309.185 6

-2 -8 11 3205.74 318.083 4

-2 -8 11 2364.73 188.441 1

2 -8 -11 2898.55 243.651 9

2 8 -11 2566.27 316.231 10

2 -8 -11 2931.42 336.008 6

2 -8 -10 213.375 42.6742 5

-2 -8 10 200.018 59.5965 1

2 8 -10 202.036 86.8887 10

2 -8 -10 240.611 62.5715 9

-2 -8 10 177.358 59.1136 5

2 -8 -10 208.147 99.4887 6

2 -8 -9 2077.97 274.238 4

2 -8 -9 1746.14 136.760 5

-2 -8 9 1841.01 165.667 1

-2 8 9 2037.99 260.954 1

-2 -8 9 1995.11 232.306 10

2 -8 -9 2034.86 184.537 9

-2 8 9 1835.79 245.021 10

2 8 -9 2171.16 261.010 10

2 -8 -9 1824.06 240.897 6

-2 -8 9 1982.02 196.408 5

-2 -8 8-16.3432 32.4990 5

2 -8 -8-13.3989 28.7333 5

2 -8 -8 49.5642 66.6757 4

-2 -8 8-53.0496 36.0188 1

-2 8 8 7.06536 46.5893 1

2 -8 -8 120.270 95.2229 1

2 8 -8 19.2133 38.6424 10

-2 -8 8-4.23607 38.9046 10

2 -8 -8-4.24278 31.9232 9

-2 8 8-5.74311 34.5270 10

-2 8 8-57.9904 80.0406 6

2 -8 -8-62.1208 67.9754 6

2 -8 -7 1178.88 182.633 4

2 -8 -7 1079.04 120.485 5

-2 -8 7 1172.18 152.038 5

-2 8 7 1251.07 189.128 1

2 -8 -7 1312.58 198.602 1

2 -8 -7 1220.46 133.062 9

-2 8 7 1214.50 173.955 10

-2 -8 7 1239.70 161.012 10

2 8 -7 1223.21 175.836 10

-2 8 7 1250.26 194.490 6

2 -8 -6 181.068 49.1416 5

2 -8 -6 120.656 58.9969 4

-2 -8 6 208.278 60.8859 5

2 -8 -6 158.632 86.0559 1

-2 -8 6 243.064 69.9247 10

-2 8 6 207.490 76.5124 10

2 -8 -6 177.276 49.1954 9

-2 8 6 152.260 69.5369 6

2 -8 -5 493.597 100.136 3

2 -8 -5 234.044 60.3594 5

-2 -8 5 244.632 83.5657 5

2 -8 -5 221.528 91.5144 1

2 -8 -5 452.705 79.9294 9

-2 8 5 353.087 92.7027 10

-2 8 5 321.109 100.028 6

-2 -8 5 363.169 71.9923 7

-2 -8 4 18452.3 573.843 5

2 -8 -4 18288.4 524.430 5

2 -8 -4 19853.0 632.124 4

2 -8 -4 21830.6 702.961 1

-2 -8 4 21285.5 433.701 1

2 -8 -4 20607.2 472.356 9

2 -8 -4 22533.3 674.638 8

-2 8 4 20875.0 624.056 6

-2 8 4 19536.5 688.162 8

-2 -8 4 20342.3 468.747 7

2 -8 -3 12550.7 467.529 4

2 -8 -3 12346.9 438.975 5

-2 8 3 12151.8 330.332 2

2 -8 -3 12349.0 495.713 1

-2 -8 3 12088.0 330.002 1

2 -8 -3 13237.7 375.483 9

-2 8 3 13938.2 403.934 9

-2 -8 3 13184.7 367.545 7

2 -8 -3 12578.0 490.260 8

2 -8 -2 15408.7 500.548 5

-2 8 2 13917.5 428.199 3

-2 8 2 15799.9 491.520 1

2 -8 -2 15170.0 510.960 1

-2 -8 2 14223.9 372.059 1

-2 8 2 13804.1 407.349 2

-2 8 2 13768.2 398.902 9

2 8 -2 13994.2 389.672 7

2 -8 -2 15180.4 525.370 8

2 -8 -1 6357.07 325.807 5

-2 8 1 7002.37 297.079 3

-2 8 1 6462.04 334.322 5

-2 8 1 6080.07 280.103 1

-2 -8 1 6171.00 257.100 1

2 -8 -1 6237.07 303.881 1

2 8 -1 6178.73 266.468 1

2 8 -1 6975.53 284.404 3

-2 8 1 6733.08 284.749 9

2 -8 -1 6089.17 332.788 8

2 8 -1 6939.91 276.464 7

-2 -8 0 9844.67 345.959 1

2 -8 0 10034.5 345.288 1

2 8 0 9127.80 339.361 1

-2 8 0 10629.4 327.009 1

-2 8 0 8911.19 333.103 3

2 -8 0 10407.0 425.709 5

2 8 0 9083.64 315.643 3

2 -8 0 9792.29 399.744 8

-2 8 0 10749.2 404.712 8

-2 -8 -1 23895.5 574.822 1

-2 8 -1 24683.8 435.814 1

2 8 1 28952.4 550.104 3

-2 8 -1 27861.5 599.767 3

2 -8 1 26908.0 712.989 5

-2 -8 -1 28361.2 538.206 3

-2 8 -1 25390.3 603.469 8

2 -8 1 26603.8 647.528 8

2 8 2 78804.0 908.347 3

-2 8 -2 85803.4 1250.42 5

-2 8 -2 79569.3 1031.91 3

2 -8 2 90358.0 1341.17 5

-2 -8 -2 80770.9 912.201 3

-2 -8 -2 83521.2 1144.47 1

-2 8 -2 85761.8 627.906 1

2 8 2 80352.3 689.143 9

-2 8 -2 81160.7 660.331 7

2 8 2 82556.5 1170.43 6

2 -8 2 83188.1 1113.72 8

-2 8 -3 13089.6 498.508 5

-2 -8 -3 11610.5 346.527 3

-2 -8 -3 12251.7 465.014 1

2 8 3 11195.3 235.259 9

2 -8 3 13091.7 521.737 5

2 8 3 12081.2 485.204 6

-2 8 -4 7654.45 395.027 5

-2 -8 -4 8188.28 302.133 3

-2 -8 -4 8409.49 414.761 1

2 8 4 7555.26 325.521 10

-2 8 -4 8084.48 356.601 10

2 -8 4 8317.77 422.945 5

2 8 4 8158.55 435.609 6

-2 -8 -5 770.805 112.427 3

-2 8 -5 3352.65 279.743 5

2 8 5 2248.85 302.624 1

-2 -8 -5 2992.67 332.202 1

2 8 5 726.072 112.753 10

-2 8 -5 1177.78 157.826 10

2 8 5 602.007 135.278 6

2 -8 5 1108.58 170.678 5

-2 8 -6 1399.93 176.181 5

-2 -8 -6 992.243 123.665 3

2 -8 6 954.348 78.6856 4

2 8 6 1302.21 211.672 1

-2 -8 -6 1530.01 232.659 1

-2 8 -6 1150.20 160.191 10

-2 -8 -6 972.837 73.8428 9

2 8 6 846.419 127.539 10

2 -8 6 923.329 63.8177 7

2 8 6 967.761 173.509 6

-2 -8 -6 1126.46 155.592 8

2 -8 6 938.185 161.972 5

-2 -8 -6 1020.27 165.093 6

-2 8 -7-1.52647 33.3023 5

-2 -8 -7 40.4460 26.3437 4

-2 -8 -7 10.7811 24.0788 3

-2 -8 -7 19.3610 58.0781 1

2 8 7-27.7335 39.8967 1

-2 -8 -7 18.3651 13.7291 9

-2 8 -7 5.88893 39.1135 10

2 8 7 23.6266 24.8977 10

2 8 7-79.4644 75.5684 6

-2 -8 -7-16.8483 33.9814 8

-2 -8 -7-16.9155 45.2963 6

2 -8 7 0.45494 11.1335 7

2 -8 7-4.94141 26.0785 5

-2 -8 -8 648.775 110.272 3

-2 8 -8 691.459 129.861 5

-2 -8 -8 659.332 148.132 4

-2 -8 -8 845.431 173.920 1

2 8 8 593.795 138.702 1

-2 8 -8 570.096 124.375 10

-2 -8 -8 698.409 76.3391 9

2 8 8 630.559 131.326 10

2 -8 8 651.880 63.6324 7

-2 -8 -8 489.923 103.556 8

-2 -8 -8 659.618 151.671 6

-2 -8 -9 2712.67 307.294 4

-2 -8 -9 3032.29 253.054 3

-2 8 -9 2781.28 265.364 5

-2 -8 -9 2969.39 352.292 1

-2 -8 -9 3362.10 178.778 9

2 8 9 2978.68 284.666 10

-2 8 -9 3185.53 317.528 10

-2 -8 -9 2587.76 248.895 8

2 -8 9 3007.28 149.827 7

-2 -8 -9 2955.99 328.086 6

-2 -8 -10 83.2039 50.9023 3

-2 8 -10 101.620 64.5404 5

-2 -8 -10 148.009 82.1814 1

-2 -8 -10 137.914 44.6028 9

2 8 10 200.536 97.5471 10

-2 8 -10 146.108 83.5237 10

-2 -8 -10 51.0830 60.9211 6

-2 -8 -11 1085.08 171.227 3

-2 -8 -11 930.859 207.150 1

-2 8 -11 1005.07 206.015 10

-2 -8 -11 1024.00 110.559 9

-2 -8 -11 915.158 210.359 6

-2 -8 -12 459.217 119.956 3

-2 -8 -12 288.939 95.6903 1

-2 8 -12 422.199 141.932 10

-2 8 -12 576.172 203.968 6

-2 -8 -12 263.794 97.7741 6

-2 -8 -13 557.341 127.537 3

-2 8 -13 575.009 179.620 10

-2 8 -13 635.307 214.380 6

-2 -8 -13 691.410 208.641 6

-2 8 -14 60.4620 72.4229 10

2 -9 -16 34.1742 44.8079 3

2 9 -15 95.7434 106.576 10

2 9 -14 131.130 96.6881 10

2 9 -14 26.9527 63.9691 6

2 -9 -14-131.304 99.8700 6

-2 -9 13 54.4148 38.0476 4

2 -9 -13 1.26954 42.3472 9

2 9 -13-56.9416 94.9862 10

2 -9 -13-58.6832 61.4918 6

2 9 -13 79.3306 91.0281 6

-2 -9 12 321.698 94.2748 4

2 -9 -12 357.878 87.2715 9

2 9 -12 438.620 151.397 10

2 -9 -12 385.516 132.221 6

2 -9 -11 193.900 34.2099 5

-2 -9 11 413.443 122.732 4

-2 -9 11 276.657 56.6201 1

2 -9 -11 199.425 68.9273 9

2 9 -11 273.277 118.451 10

2 -9 -11 184.360 85.7133 6

2 -9 -10 284.005 57.6435 5

-2 -9 10 321.793 63.2490 1

2 -9 -10 333.707 78.6602 9

2 9 -10 487.294 144.690 10

-2 -9 10 298.307 82.7791 5

-2 -9 10 256.203 38.4661 8

2 -9 -10 165.769 96.0057 6

-2 -9 9 61.6573 55.2819 5

2 -9 -9 10.5593 23.2519 5

2 -9 -9 53.5066 50.7950 1

-2 -9 9 13.9747 21.6588 1

2 -9 -9-10.3019 29.4759 9

-2 9 9-53.4889 71.1788 10

2 9 -9-53.3037 41.4811 10

2 -9 -9-29.4181 66.4510 6

-2 -9 8 159.457 66.2800 5

2 -9 -8 120.281 41.5082 5

2 -9 -8 103.369 49.3985 4

2 -9 -8 129.100 70.8610 1

-2 -9 8 152.406 41.0583 1

-2 9 8 125.749 66.0616 1

-2 -9 8 108.095 58.7704 10

-2 9 8 129.776 64.6986 10

2 -9 -8 91.8603 35.7026 9

2 9 -8 152.211 84.1345 10

-2 9 8 10.1677 44.1826 6

-2 -9 7 952.482 146.518 5

2 -9 -7 791.114 109.786 5

2 -9 -7 901.048 171.497 4

2 -9 -7 984.196 142.358 3

-2 9 7 873.795 172.508 1

2 -9 -7 834.447 172.494 1

-2 9 7 1029.04 165.181 10

-2 -9 7 936.243 144.686 10

2 -9 -7 919.069 115.652 9

2 9 -7 983.288 158.244 10

-2 9 7 1085.39 187.724 6

2 -9 -6 137.636 57.8255 3

2 -9 -6 146.781 52.5415 5

2 -9 -6 127.033 61.0755 4

-2 -9 6 161.760 67.7528 5

2 -9 -6 174.057 87.9376 1

-2 9 6 142.079 60.7136 10

2 -9 -6 178.828 50.4886 9

-2 -9 6 143.281 43.6302 7

-2 9 6 142.883 62.5215 6

-2 -9 5 22416.9 676.677 5

2 -9 -5 21872.0 602.325 5

2 -9 -5 25644.8 639.003 3

2 -9 -5 23874.3 759.922 4

-2 -9 5 23429.7 462.320 1

2 -9 -5 23258.3 786.317 1

-2 9 5 25218.5 732.657 10

2 -9 -5 24814.3 544.557 9

2 -9 -5 23913.4 742.654 8

-2 -9 5 24105.6 534.605 7

-2 9 5 25100.4 765.001 6

2 -9 -4 64484.1 1191.74 4

-2 -9 4 58031.5 1079.17 5

2 -9 -4 65184.8 1055.32 5

-2 9 4 61282.7 719.652 2

-2 -9 4 63755.1 772.340 1

2 -9 -4 70288.8 1306.76 1

2 -9 -4 64174.8 866.692 9

-2 9 4 62424.1 916.152 9

-2 -9 4 60550.9 828.698 7

2 -9 -4 60836.5 1162.16 8

-2 9 4 64366.1 1145.32 6

-2 9 3 48310.3 868.543 3

2 -9 -3 43163.6 909.966 4

2 -9 -3 41665.6 858.817 5

-2 -9 3 43623.3 655.986 1

2 -9 -3 43478.1 955.673 1

-2 9 3 42758.8 669.169 2

-2 9 3 47698.8 781.212 9

2 -9 -3 45399.4 982.332 8

-2 -9 3 44280.0 702.790 7

-2 9 2 851.151 111.409 3

2 -9 -2 1814.40 190.096 5

-2 9 2 1855.45 161.282 2

-2 -9 2 1682.74 138.354 1

-2 9 2 1739.13 178.783 1

2 -9 -2 1372.14 193.680 1

-2 9 2 980.764 115.465 9

2 -9 -2 1762.89 187.365 8

2 9 -2 1026.60 110.316 7

2 9 -1 5727.07 271.435 1

-2 9 1 7892.55 337.276 1

2 -9 -1 7372.87 341.989 1

-2 -9 1 7030.57 289.513 1

2 9 -1 4876.28 255.056 3

2 -9 -1 7094.76 365.397 5

-2 9 1 4494.27 250.128 3

2 9 -1 5191.31 253.140 7

-2 9 1 4889.00 256.310 9

2 -9 -1 6455.53 358.933 8

-2 9 0 892.965 102.353 1

2 -9 0 855.903 107.982 1

-2 -9 0 867.536 110.425 1

2 9 0 733.044 101.780 1

-2 9 0 881.123 114.453 3

2 -9 0 911.929 132.818 5

2 9 0 883.503 105.401 3

2 -9 0 789.492 120.192 8

-2 9 -1 26000.7 473.000 1

2 -9 1 25693.8 523.297 1

-2 -9 -1 25458.4 626.064 1

-2 -9 -1 24606.1 530.523 3

2 -9 1 25836.4 736.188 5

2 9 1 24866.2 544.656 3

-2 9 -1 24814.3 597.940 3

2 -9 1 26164.9 682.208 8

2 -9 2 10989.1 490.759 5

-2 9 -2 9698.48 380.940 3

2 9 2 9590.91 337.366 3

-2 -9 -2 10031.7 336.981 3

-2 -9 -2 10382.6 427.295 1

-2 9 -2 11381.8 255.434 1

2 9 2 9588.44 268.095 9

2 -9 2 10517.3 423.172 8

-2 9 -2 9659.32 260.378 7

2 -9 3 397.662 95.4470 5

2 9 3 417.544 73.9482 3

-2 -9 -3 473.941 74.8020 3

-2 9 -3 416.721 94.7705 5

-2 9 -3 516.195 93.8580 3

-2 -9 -3 338.734 95.8837 1

2 9 3 420.673 50.4877 9

2 9 3 579.672 110.642 6

-2 9 -4 2191.66 221.470 5

-2 -9 -4 1930.54 153.331 3

-2 -9 -4 2183.53 223.255 1

-2 9 -4 1961.47 181.684 10

2 -9 4 1700.19 200.326 5

2 9 4 2113.14 229.261 6

-2 -9 -5 2014.47 165.218 3

-2 9 -5 2256.51 228.751 5

-2 -9 -5 2103.23 229.037 1

2 9 5 2216.23 237.497 1

-2 9 -5 1928.65 195.520 10

2 9 5 1894.23 178.887 10

2 9 5 2190.67 253.996 6

2 -9 5 2090.54 231.991 5

-2 -9 -6 687.747 101.410 3

-2 9 -6 509.886 112.595 5

2 -9 6 613.468 65.0034 4

-2 -9 -6 712.020 145.002 1

2 9 6 607.483 132.309 1

-2 9 -6 582.710 119.671 10

2 9 6 702.103 119.640 10

-2 -9 -6 488.759 101.864 8

2 9 6 623.575 141.627 6

2 -9 6 636.547 133.838 5

-2 9 -7 2342.75 242.086 5

-2 -9 -7 2349.19 202.969 3

2 -9 7 2274.35 127.628 4

-2 -9 -7 2027.11 249.895 4

2 9 7 2338.89 268.248 1

-2 -9 -7 2311.68 260.187 1

2 9 7 2448.07 234.852 10

-2 9 -7 2526.65 254.919 10

-2 -9 -7 2358.82 124.787 9

-2 -9 -7 2337.68 262.045 6

-2 -9 -7 2094.98 217.673 8

2 9 7 2258.08 291.118 6

2 -9 7 2402.75 254.730 5

-2 -9 -8 799.702 166.645 4

-2 -9 -8 989.605 140.661 3

-2 9 -8 933.756 157.762 5

-2 -9 -8 993.162 182.038 1

2 9 8 1038.66 161.072 10

-2 9 -8 1025.29 172.171 10

-2 -9 -8 962.717 85.6883 9

2 -9 8 983.737 75.2951 7

-2 -9 -8 986.492 182.081 6

-2 -9 -8 848.970 141.898 8

-2 9 -9 131.252 64.5672 5

-2 -9 -9 256.464 76.7552 3

-2 -9 -9 60.3664 72.9195 1

-2 -9 -9 165.506 35.4936 9

-2 9 -9 201.837 93.9191 10

2 9 9 64.9722 48.4924 10

-2 -9 -9 314.335 122.793 6

-2 -9 -9 186.693 70.0669 8

2 -9 9 183.901 32.7187 7

-2 -9 -10 9.38259 29.7759 3

-2 9 -10-15.9657 38.7868 5

-2 -9 -10-4.72538 54.6286 1

-2 -9 -10 41.0226 23.1493 9

2 9 10 68.6840 42.8796 10

-2 9 -10 15.9554 42.0783 10

-2 -9 -10 82.0119 71.0903 6

2 -9 10 33.8322 23.7865 7

-2 -9 -11 1071.74 165.586 3

-2 -9 -11 958.195 234.128 1

-2 9 -11 1088.91 210.657 10

-2 -9 -11 1247.68 241.039 6

-2 -9 -12 201.390 80.5390 3

-2 -9 -12 132.217 91.7383 1

-2 9 -12 157.314 90.9094 10

-2 9 -12 272.368 136.503 6

-2 -9 -12 224.683 124.071 6

-2 -9 -13 164.597 88.0026 3

-2 9 -13 241.075 113.146 10

-2 9 -13 297.530 138.643 6

-2 -9 -13 357.867 143.007 6

-2 9 -14 57.3554 68.0106 10

2 -10 -16 176.807 109.656 3

2 -10 -15 252.209 108.785 3

2 10 -15 242.195 124.712 10

2 -10 -14-104.136 73.5183 3

2 10 -14-8.36601 89.5052 10

2 10 -14-18.8157 64.5601 6

2 -10 -14-2.40358 47.3579 6

2 -10 -13 226.629 100.542 3

-2 -10 13 154.157 88.2326 4

2 10 -13 21.8793 53.5513 10

2 -10 -13 136.272 57.6149 9

2 -10 -13-59.1650 62.5681 6

-2 -10 12 418.837 119.756 4

2 -10 -12 460.861 98.2867 9

2 10 -12 523.532 158.020 10

2 -10 -12 582.675 172.491 6

-2 -10 11 2083.46 261.860 4

-2 -10 11 1195.26 115.538 1

2 -10 -11 1793.58 189.266 9

2 10 -11 1722.45 279.731 10

-2 -10 11 1493.37 183.176 5

2 -10 -10 135.265 81.2527 3

2 -10 -10 64.0608 31.1444 5

-2 -10 10 37.0995 30.0572 5

-2 -10 10 89.1315 37.0801 1

2 -10 -10 82.6234 60.4101 9

2 10 -10 90.4932 86.3527 10

-2 -10 10 30.4662 16.4980 8

2 -10 -9 1590.87 155.847 5

2 -10 -9 2016.34 220.159 3

-2 -10 9 1768.92 146.588 1

2 -10 -9 1894.84 268.947 1

2 10 -9 2016.77 263.882 10

-2 10 9 1891.43 253.863 10

2 -10 -9 1709.46 169.566 9

-2 -10 9 1798.89 212.617 5

2 -10 -8 185.865 85.3875 4

-2 -10 8 101.666 57.7619 5

2 -10 -8 156.331 69.2029 3

2 -10 -8 95.1731 42.6016 5

-2 10 8 164.580 96.1720 1

2 -10 -8 309.933 131.029 1

2 -10 -8 113.314 45.4581 9

2 10 -8 92.3233 65.2634 10

-2 10 8 118.779 58.2805 10

-2 -10 8 95.9788 35.4579 7

2 -10 -7 2418.82 277.379 4

-2 -10 7 2146.62 223.969 5

2 -10 -7 1995.42 186.343 5

2 -10 -7 2240.92 216.764 3

-2 10 7 2014.21 255.007 1

2 -10 -7 2448.14 289.328 1

-2 10 7 2037.37 235.527 10

2 10 -7 2041.07 235.949 10

2 -10 -7 2346.32 180.976 9

-2 10 7 2683.38 298.144 6

-2 -10 7 2209.28 174.630 7

2 -10 -6 12284.5 474.391 5

-2 -10 6 13137.7 554.659 5

2 -10 -6 13607.1 632.506 4

2 -10 -6 14056.2 508.994 3

-2 -10 6 13151.9 345.346 1

2 -10 -6 13521.7 652.535 1

2 -10 -6 13295.0 422.198 9

-2 10 6 13826.2 583.401 10

-2 10 6 14604.1 644.755 6

-2 -10 6 13141.6 416.229 7

2 -10 -5 80.5458 58.8372 4

-2 -10 5 52.7212 56.2637 5

2 -10 -5 18.1753 41.2067 5

2 -10 -5 14.4540 35.9354 3

-2 -10 5 51.4782 26.5284 1

2 -10 -5 20.0340 60.2622 1

-2 10 5 4.29010 25.0971 10

2 -10 -5 65.6439 53.0484 8

-2 10 5 17.6014 32.3143 6

2 -10 -5 95.2838 39.9385 9

-2 -10 5 50.7553 36.2846 7

2 -10 -4 1686.57 180.140 5

-2 -10 4 1785.96 203.455 5

2 -10 -4 1549.23 193.424 4

-2 10 4 1693.13 127.868 2

-2 -10 4 1741.16 136.611 1

2 -10 -4 1554.54 205.844 1

2 -10 -4 1745.93 149.217 9

-2 10 4 1721.40 158.517 9

-2 10 4 1913.96 205.171 6

2 -10 -4 1592.90 198.815 8

-2 -10 4 1611.61 142.086 7

-2 10 3 30126.3 717.297 3

2 -10 -3 26091.9 716.584 5

-2 10 3 25900.1 562.291 2

2 -10 -3 27030.8 785.318 1

-2 -10 3 27233.4 544.438 1

-2 10 3 29312.5 643.677 9

2 10 -3 28878.6 617.786 7

2 -10 -3 27209.5 794.489 8

2 -10 -2 16648.6 582.724 5

-2 10 2 16259.4 512.935 3

-2 10 2 18068.9 569.466 1

2 10 -2 15437.3 448.140 1

2 -10 -2 16993.7 591.892 1

-2 -10 2 16747.3 439.702 1

-2 10 2 16607.0 480.998 9

2 -10 -2 17032.1 620.115 8

2 10 -2 16669.5 462.708 7

2 -10 -1 5421.86 304.241 1

-2 -10 1 5493.16 271.304 1

-2 10 1 5519.21 311.149 1

2 10 -1 4052.80 237.159 1

2 -10 -1 5105.70 337.650 5

2 10 -1 2833.37 204.806 3

-2 10 1 2460.45 196.404 3

-2 10 1 2839.70 208.760 9

2 -10 -1 4296.12 311.068 8

2 10 -1 3119.13 206.792 7

-2 10 0 6077.82 269.799 1

-2 -10 0 5716.51 291.028 1

2 -10 0 6007.60 294.343 1

2 10 0 5498.29 287.163 1

-2 10 0 5512.96 294.158 3

2 -10 0 5873.26 353.071 5

2 10 0 5632.52 283.332 3

2 -10 0 5836.70 343.988 8

-2 -10 -1 23165.6 626.664 1

2 -10 1 25166.6 550.913 1

-2 10 -1 23288.0 477.060 1

2 -10 1 25085.8 761.394 5

-2 -10 -1 26672.5 584.759 3

-2 10 -1 27232.1 659.954 3

2 10 1 26502.2 601.372 3

2 -10 1 25234.5 704.744 8

-2 -10 -2 48613.3 953.268 1

-2 10 -2 50293.4 584.375 1

2 10 2 48583.5 809.476 3

-2 -10 -2 48953.5 796.881 3

-2 10 -2 48261.1 893.626 3

2 -10 2 50232.5 974.033 8

-2 10 -3 38756.3 828.115 3

-2 10 -3 36991.8 920.966 5

2 10 3 39288.8 748.564 3

2 -10 3 38091.6 982.962 5

-2 -10 -3 38345.1 710.036 3

-2 10 -3 39175.6 393.275 1

2 10 3 36894.4 523.710 9

2 -10 3 37205.4 815.155 8

2 10 3 39372.7 937.345 6

-2 10 -3 37310.8 506.954 7

-2 10 -4 452.953 116.217 5

2 -10 4 190.180 67.3489 5

-2 -10 -4 267.781 61.9931 3

-2 -10 -4 302.018 108.293 1

-2 10 -4 214.637 73.6710 10

2 10 4 242.238 80.0512 6

-2 -10 -5 983.087 127.299 3

-2 10 -5 2751.18 268.676 5

2 10 5 1114.42 221.990 1

-2 -10 -5 2986.60 291.759 1

2 10 5 1013.89 138.158 10

-2 10 -5 1246.67 170.527 10

2 -10 5 1481.76 212.015 5

2 10 5 1102.53 191.348 6

-2 -10 -6 5573.93 298.072 3

-2 10 -6 5133.13 370.147 5

-2 -10 -6 5576.14 426.403 1

2 10 6 5183.76 389.758 1

2 10 6 5329.51 330.523 10

-2 10 -6 5429.65 357.504 10

2 10 6 5806.17 446.295 6

2 -10 6 5907.39 409.193 5

-2 -10 -6 5354.57 365.287 8

2 -10 7 4438.43 181.412 4

-2 -10 -7 4426.19 279.224 3

-2 -10 -7 4136.12 366.769 4

-2 10 -7 3976.13 326.902 5

-2 -10 -7 4507.43 382.470 1

2 10 7 4250.05 370.663 1

2 10 7 4339.69 319.476 10

-2 10 -7 4553.20 349.687 10

2 -10 7 4587.35 369.290 5

-2 -10 -7 3933.94 309.474 8

-2 10 -8 579.925 148.793 5

-2 -10 -8 579.506 108.086 3

-2 -10 -8 628.041 182.920 1

-2 10 -8 661.068 142.555 10

-2 -10 -8 577.263 64.9132 9

2 10 8 574.211 126.340 10

-2 -10 -8 668.310 139.646 8

2 -10 8 524.375 133.425 5

-2 -10 -9 3606.30 277.381 3

-2 10 -9 3021.24 304.358 5

-2 -10 -9 3654.38 386.524 1

-2 10 -9 3679.53 346.788 10

-2 -10 -9 3455.94 165.629 9

2 10 9 3406.34 315.467 10

-2 -10 -9 3521.06 361.290 6

-2 -10 -9 3215.05 296.250 8

-2 -10 -10 43.8407 30.7894 3

-2 -10 -10 48.0109 52.5844 1

-2 10 -10 63.9116 55.0762 10

2 10 10 128.884 78.6818 10

-2 -10 -10 166.058 75.1808 6

-2 -10 -10 105.045 35.3272 9

-2 -10 -11 405.728 105.458 3

-2 -10 -11 436.445 146.352 1

-2 10 -11 663.657 166.018 10

-2 -10 -11 662.472 183.999 6

-2 -10 -12 154.743 72.2581 3

-2 -10 -12 206.188 106.548 1

-2 10 -12 251.762 120.042 10

-2 -10 -12 387.897 156.740 6

-2 -10 -13 227.366 102.983 3

-2 -10 -13 166.425 106.832 1

-2 10 -13-81.6138 73.0332 10

-2 10 -13 255.908 148.486 6

-2 -10 -13 141.227 91.6755 6

-2 10 -14-51.2773 85.0644 10

2 -11 -15 6.22700 57.7281 3

2 11 -15-83.5148 92.2107 10

2 -11 -14-39.3430 62.6420 3

2 11 -14-143.945 98.0990 10

2 11 -14 39.4435 50.7683 6

2 -11 -13 154.444 70.8961 3

2 11 -13 19.5847 52.2629 10

2 -11 -12 97.8935 64.6524 3

-2 -11 12 33.1624 38.0766 4

2 11 -12-33.8397 55.6660 10

2 -11 -12 100.125 46.5097 9

-2 -11 11 251.177 104.708 4

2 -11 -11 217.623 91.9033 3

-2 -11 11 120.653 27.7861 1

2 11 -11 283.781 119.466 10

2 -11 -11 168.137 61.1499 9

-2 -11 11 247.272 87.7453 5

-2 -11 11 227.492 70.7764 7

2 -11 -10 253.410 89.2505 3

2 -11 -10 177.034 58.8789 5

-2 -11 10 126.581 72.9500 5

-2 -11 10 151.774 33.5353 1

2 11 -10 142.341 87.0916 10

2 -11 -10 266.297 74.4875 9

-2 -11 10 142.375 49.1047 7

-2 -11 10 135.628 40.2271 8

2 -11 -9 392.746 108.363 3

-2 -11 9 389.024 113.786 5

2 -11 -9 432.833 92.8143 5

-2 -11 9 461.532 64.4064 1

-2 -11 9 405.836 67.0400 1

2 -11 -9 451.062 158.307 1

2 11 -9 579.217 147.369 10

2 -11 -9 462.913 88.4575 9

-2 -11 9 359.713 76.9169 7

-2 -11 9 450.922 83.6265 8

2 -11 -8 232.369 71.8934 3

2 -11 -8 260.994 71.1554 5

-2 -11 8 245.764 86.3751 5

-2 -11 8 268.244 46.5053 1

2 -11 -8 320.461 122.571 1

2 -11 -8 283.068 66.8236 9

-2 11 8 270.362 87.9681 10

2 11 -8 212.594 92.0985 10

-2 -11 8 288.799 70.9320 7

-2 -11 7 904.334 159.829 5

2 -11 -7 864.624 136.608 5

2 -11 -7 884.714 177.084 4

2 -11 -7 781.301 126.445 3

-2 11 7 886.999 185.944 1

2 -11 -7 902.437 189.754 1

-2 -11 7 849.094 87.4863 1

2 -11 -7 906.567 114.145 9

2 11 -7 884.346 165.727 10

-2 11 7 912.523 167.374 10

-2 -11 7 852.407 111.745 7

-2 11 7 855.107 172.567 6

2 -11 -6 2444.21 273.214 4

2 -11 -6 2143.92 216.957 5

2 -11 -6 2429.18 214.776 3

-2 -11 6 2290.82 240.626 5

-2 -11 6 2217.89 144.864 1

2 -11 -6 2586.01 292.648 1

2 11 -6 2620.08 261.205 10

-2 11 6 2547.44 257.438 10

2 -11 -6 2403.40 182.044 9

-2 -11 6 2349.77 178.305 7

-2 11 6 2582.48 277.053 6

2 -11 -5 5652.70 390.747 4

2 -11 -5 5271.84 332.192 5

-2 -11 5 5274.10 358.291 5

2 -11 -5 5897.33 326.380 3

-2 -11 5 5689.37 237.122 1

2 -11 -5 5385.27 393.610 1

-2 11 5 6252.01 385.463 10

2 -11 -5 5700.43 281.964 9

-2 -11 5 5565.80 274.988 7

-2 11 5 6376.21 404.725 6

2 -11 -5 5298.10 384.103 8

2 -11 -4 4461.03 336.740 4

-2 -11 4 4793.89 362.283 5

2 -11 -4 4964.02 328.114 5

2 -11 -4 4820.49 369.287 1

-2 -11 4 4701.92 229.454 1

-2 11 4 4186.51 220.137 2

-2 11 4 4418.16 259.925 9

2 -11 -4 4512.09 346.782 8

-2 11 3 15430.7 538.041 3

2 -11 -3 13289.5 539.053 5

-2 -11 3 13390.3 394.149 1

-2 11 3 12464.6 424.097 2

2 -11 -3 13757.2 581.069 1

-2 11 3 14307.5 466.806 9

2 -11 -3 13609.8 578.914 8

2 11 -3 14330.4 450.342 7

2 -11 -2 15813.1 604.415 5

-2 11 2 14518.5 507.258 3

-2 11 2 16898.4 564.319 1

2 11 -2 13877.2 447.945 1

2 11 -2 14429.9 499.609 3

-2 -11 2 16033.3 456.672 1

2 -11 -2 16614.1 598.016 1

-2 11 2 13944.6 464.725 9

2 -11 -2 15154.9 605.976 8

2 11 -2 14153.1 452.428 7

-2 11 1 3054.71 230.798 1

2 -11 -1 2528.47 223.075 1

-2 -11 1 2889.00 204.965 1

2 11 -1 1724.01 164.667 1

2 -11 -1 2717.99 245.884 5

2 -11 -1 2376.96 233.520 8

2 11 -1 1272.82 141.707 7

2 11 0 236.620 73.9792 1

2 -11 0 305.524 90.4573 1

-2 11 0 224.736 70.5176 1

-2 -11 0 314.538 100.804 1

-2 11 0 246.462 81.6865 3

2 -11 0 236.668 92.1620 5

2 11 0 245.894 79.8210 3

2 -11 0 217.713 86.9892 8

-2 11 -1 13577.6 385.372 1

-2 -11 -1 13816.9 500.810 1

2 -11 1 14173.4 443.780 1

-2 -11 -1 15087.0 462.902 3

2 11 1 15483.7 483.137 3

-2 11 -1 15750.6 528.895 3

2 -11 1 15125.0 617.020 5

2 -11 1 14579.0 564.919 8

2 -11 2 16729.4 422.055 1

-2 -11 -2 17249.9 584.681 1

-2 11 -2 16896.0 365.796 1

2 -11 2 17909.6 677.973 5

2 11 2 16343.1 492.653 3

-2 -11 -2 15754.0 476.202 3

-2 11 -2 16279.5 539.550 3

2 -11 2 16605.5 592.064 8

-2 11 -3 5692.20 384.864 5

-2 11 -3 6265.22 346.781 3

2 -11 3 6032.95 410.002 5

-2 -11 -3 6356.63 304.756 3

2 11 3 6202.15 314.206 3

-2 -11 -3 5354.20 349.180 1

-2 11 -3 6268.00 181.076 1

2 11 3 6238.77 231.394 9

-2 11 -3 6121.67 221.539 7

2 11 3 6452.51 390.235 6

2 -11 3 6176.80 347.244 8

-2 11 -4 17525.1 680.425 5

-2 -11 -4 16522.6 499.521 3

-2 -11 -4 17007.1 658.609 1

-2 11 -4 17134.9 592.076 3

-2 11 -4 17628.9 589.327 10

2 -11 4 18029.4 724.267 5

2 11 4 16161.3 662.217 6

2 -11 5 94.9273 65.1872 5

-2 -11 -5 11.6773 30.1146 3

-2 11 -5 169.285 112.481 5

-2 -11 -5 104.140 120.826 1

2 11 5 268.955 97.6956 1

2 11 5 16.7479 35.7499 10

-2 11 -5 28.1863 44.5223 10

2 11 5 16.7062 29.5147 6

2 -11 6 34.8687 54.7044 5

-2 -11 -6 89.7345 44.0193 3

-2 11 -6 158.633 80.6441 5

-2 -11 -6 209.675 89.8072 1

2 11 6 119.796 86.8458 1

2 11 6 62.4107 42.8769 10

-2 11 -6 60.7983 32.2551 10

-2 -11 -6 231.741 95.6627 8

2 11 6 102.170 72.1211 6

2 -11 7 1156.68 97.9399 4

-2 11 -7 1004.02 168.944 5

-2 -11 -7 1137.67 143.947 3

-2 -11 -7 976.877 173.217 1

2 11 7 1096.65 163.347 10

-2 11 -7 1187.87 186.162 10

2 -11 7 1221.81 197.976 5

-2 -11 -7 963.138 155.402 8

-2 -11 -8 8.65820 34.8962 3

-2 11 -8 37.6954 56.1020 5

2 -11 8 8.16098 22.0358 4

-2 -11 -8 24.6288 79.3414 1

2 11 8-25.2578 39.0482 10

-2 11 -8 124.612 76.9095 10

2 -11 8 14.2844 46.6713 5

-2 -11 -8 39.3041 43.2540 8

-2 11 -9-42.4538 70.8281 5

-2 -11 -9 94.8053 54.4213 3

-2 -11 -9-128.836 92.5979 1

-2 11 -9-2.01412 65.7426 10

2 11 9-14.2745 39.6227 10

-2 -11 -9 35.9014 28.5987 8

-2 -11 -10-23.0215 40.2371 3

2 11 10-11.9497 40.3945 10

-2 11 -10 45.9633 55.7015 10

-2 -11 -10-82.8106 51.8201 8

-2 -11 -11 666.554 126.957 3

-2 11 -11 848.668 191.349 10

-2 -11 -11 497.570 156.237 1

-2 -11 -12 1.95116 30.4950 3

-2 -11 -12 1.96015 93.1845 1

-2 11 -12-8.79175 48.4363 10

-2 -11 -13-12.7659 49.7222 3

-2 -11 -13 0.26443 56.8561 1

-2 11 -13 41.5781 63.0897 10

-2 11 -13 135.597 134.721 6

-2 11 -14 264.856 141.047 10

2 -12 -15 238.418 113.531 3

2 12 -15 179.048 107.922 10

2 -12 -14-76.7339 58.3794 3

2 12 -14-217.453 143.494 10

2 -12 -13 43.7384 42.0723 3

2 12 -13 23.3624 52.6318 10

2 -12 -12 47.7740 44.4935 3

-2 -12 12 163.685 89.4684 4

2 12 -12-1.32989 47.7283 10

2 -12 -12 49.7181 44.8174 9

2 -12 -11 66.7035 69.7916 3

2 12 -11-98.1351 86.1331 10

2 -12 -11 21.8069 26.6274 9

-2 -12 11 50.8325 55.3504 5

-2 -12 11 49.5789 31.3176 7

2 -12 -10 217.125 85.3263 3

2 -12 -10 173.924 60.7204 5

-2 -12 10 162.341 70.3641 5

-2 -12 10 174.958 36.7448 1

-2 -12 10 141.381 24.8795 1

2 -12 -10 336.678 148.790 1

2 12 -10 228.699 102.365 10

2 -12 -10 87.0053 46.9928 9

-2 -12 10 119.827 47.1388 7

-2 -12 10 95.8111 37.5348 8

2 -12 -9 1527.58 176.034 5

2 -12 -9 1754.17 212.086 3

-2 -12 9 1809.56 112.434 1

-2 -12 9 1684.15 122.324 1

2 -12 -9 1807.23 281.132 1

2 -12 -9 1900.06 181.641 9

2 12 -9 2046.14 277.463 10

-2 -12 9 1779.00 172.979 7

-2 -12 9 1614.84 164.206 8

-2 -12 9 1940.49 249.033 5

-2 -12 8 598.413 132.453 5

2 -12 -8 585.041 114.106 5

2 -12 -8 642.455 132.360 3

-2 -12 8 709.182 79.1902 1

2 -12 -8 510.727 161.501 1

2 12 -8 688.631 156.472 10

-2 12 8 607.382 142.994 10

-2 -12 8 561.020 96.0948 7

2 -12 -8 603.113 101.839 9

2 -12 -7 1177.87 204.557 4

-2 -12 7 1256.58 196.282 5

2 -12 -7 1109.55 170.548 5

2 -12 -7 1258.62 165.895 3

-2 12 7 1517.00 232.005 1

2 -12 -7 1715.22 290.808 1

-2 -12 7 1216.92 106.638 1

2 12 -7 1193.87 194.444 10

2 -12 -7 1189.34 139.696 9

-2 12 7 1195.13 184.939 10

-2 -12 7 1148.41 132.390 7

-2 12 7 1312.23 218.898 6

-2 -12 6 2583.51 271.617 5

2 -12 -6 2482.81 233.782 5

2 -12 -6 2667.97 230.683 3

2 -12 -6 2291.77 268.744 4

2 -12 -6 2493.07 306.042 1

-2 12 6 2534.24 277.617 1

-2 -12 6 2395.97 151.547 1

-2 12 6 2740.89 276.171 10

2 12 -6 2516.03 260.697 10

2 -12 -6 2649.64 197.554 9

2 -12 -6 2692.33 288.993 8

-2 -12 6 2436.93 188.171 7

-2 12 6 2658.70 286.877 6

-2 12 5 9089.64 303.326 2

2 -12 -5 9405.21 535.536 1

-2 -12 5 8658.21 306.432 1

-2 -12 5 8662.03 485.123 5

2 -12 -5 9290.77 414.010 3

2 -12 -5 8602.55 445.028 5

2 -12 -5 8844.67 501.324 4

-2 12 5 9403.88 508.111 6

2 -12 -5 9103.23 508.912 8

-2 -12 5 8819.36 350.788 7

2 -12 -4 791.795 146.064 5

2 -12 -4 686.719 115.354 3

2 -12 -4 671.637 137.414 4

-2 -12 4 620.820 131.251 5

-2 12 4 737.895 101.430 2

2 -12 -4 816.276 169.314 1

-2 -12 4 741.880 95.6966 1

-2 12 4 760.287 111.629 9

2 -12 -4 699.020 143.330 8

2 -12 -3 522.560 113.935 5

-2 12 3 289.914 80.7657 3

-2 -12 3 697.465 158.349 5

-2 -12 3 480.040 90.8820 1

-2 12 3 410.215 93.3462 1

2 -12 -3 683.607 164.441 1

-2 12 3 417.639 104.835 2

-2 12 3 273.242 63.0857 9

2 12 -3 395.018 89.3082 7

2 -12 -3 521.862 120.796 8

2 -12 -2 364.598 88.0235 1

-2 12 2 371.507 87.3576 1

2 12 -2 413.207 80.8399 1

2 12 -2 380.809 87.6247 3

-2 -12 2 478.362 76.9631 1

-2 12 2 510.142 99.2567 3

2 -12 -2 370.861 90.9880 5

2 12 -2 399.404 75.6238 7

-2 12 2 434.409 86.6508 9

2 -12 -2 442.893 117.996 8

-2 12 1 10104.9 412.780 1

-2 -12 1 9897.84 399.138 1

2 -12 -1 9913.60 458.041 1

2 12 -1 9429.76 396.849 1

-2 12 1 9070.87 407.260 3

2 -12 -1 10044.0 512.810 5

2 12 -1 9003.58 400.649 3

-2 12 1 9227.86 405.101 9

2 -12 -1 9643.19 496.434 8

2 12 -1 9718.42 391.999 7

2 12 0 1500.67 161.689 1

-2 -12 0 1399.02 153.239 1

2 -12 0 1543.15 164.002 1

-2 12 0 1549.64 146.313 1

2 12 0 1326.82 151.172 3

2 -12 0 1499.09 194.597 5

-2 12 0 1491.49 167.463 3

2 -12 0 1411.05 185.870 8

2 12 1 1055.27 145.394 1

-2 -12 -1 726.398 108.683 3

2 -12 1 1091.72 131.619 1

-2 -12 -1 1335.53 159.450 1

-2 12 -1 1241.98 121.749 1

2 12 1 695.563 108.827 3

-2 12 -1 745.964 119.552 3

2 -12 1 1172.99 177.556 5

2 -12 1 1148.27 167.159 8

-2 -12 -2 1269.32 165.379 1

-2 12 -2 1320.60 107.530 1

2 -12 2 1506.34 134.517 1

-2 12 -2 1652.47 178.601 3

2 -12 2 1373.31 192.856 5

2 12 2 1521.83 156.897 3

-2 -12 -2 1817.46 169.045 3

2 -12 2 1582.89 188.577 8

2 -12 3 7651.65 473.136 5

-2 12 -3 9946.21 541.362 5

2 12 3 3966.27 263.853 3

-2 -12 -3 4470.22 273.258 3

-2 12 -3 4395.90 307.806 3

-2 -12 -3 8167.99 493.527 1

-2 12 -3 6667.49 219.999 1

2 12 3 3842.55 199.116 9

2 -12 3 6960.76 402.775 8

2 12 3 4505.05 346.609 6

-2 12 -3 4753.65 212.374 7

2 -12 4 125.837 61.5271 5

-2 -12 -4 80.4903 36.9607 3

-2 12 -4 280.462 93.0626 5

-2 12 -4 154.365 68.6753 3

-2 -12 -4 209.307 77.1703 1

-2 12 -4 72.4776 44.4984 10

2 12 4 76.7220 31.3066 9

2 12 4 23.2650 24.0549 6

2 -12 4 108.555 54.3294 8

-2 12 -5 3515.19 327.497 5

-2 -12 -5 3609.78 247.176 3

2 12 5 3863.34 345.665 1

-2 -12 -5 3805.77 337.099 1

2 12 5 3310.95 265.970 10

-2 12 -5 3841.92 296.610 10

-2 -12 -5 3781.59 318.175 8

2 -12 5 4037.57 362.118 5

2 12 5 3819.76 353.424 6

-2 -12 -6 1714.67 172.715 3

-2 12 -6 1632.98 234.824 5

2 12 6 1561.03 232.123 1

-2 -12 -6 1939.07 258.688 1

-2 12 -6 1657.64 207.724 10

2 12 6 1546.44 188.513 10

-2 -12 -6 1475.21 205.093 8

2 -12 6 1761.37 243.878 5

-2 -12 -7 3454.93 256.682 3

-2 12 -7 3466.51 326.977 5

-2 -12 -7 3298.67 329.698 1

-2 12 -7 3901.74 337.099 10

2 12 7 3267.35 293.542 10

2 -12 7 3426.61 341.337 5

-2 -12 -7 2733.26 279.023 8

-2 12 -8 316.427 101.307 5

-2 -12 -8 476.408 100.665 3

2 -12 8 344.839 46.7294 4

-2 -12 -8 323.645 124.324 1

-2 12 -8 430.320 124.551 10

2 12 8 379.555 111.163 10

-2 -12 -8 269.159 91.1151 8

2 -12 8 424.748 131.446 5

-2 -12 -9 1119.15 159.685 3

-2 -12 -9 974.824 202.225 1

2 12 9 1246.08 203.718 10

-2 12 -9 1061.73 203.416 10

-2 -12 -9 1030.48 181.446 8

-2 -12 -10 17.4646 45.3830 3

2 12 10-90.6377 87.1706 10

-2 12 -10 75.1935 52.6908 10

-2 -12 -10 140.438 106.968 1

-2 -12 -11 123.570 64.1632 3

-2 12 -11-86.9523 69.7957 10

-2 -12 -11-41.9245 70.8984 1

-2 -12 -12 0.52986 29.3440 3

-2 12 -12 15.2361 55.2239 10

-2 -12 -12 64.4603 74.0529 1

-2 12 -13 209.874 113.567 10

-2 -12 -13-13.3996 80.6517 1

-2 12 -14 120.966 109.545 10

2 -13 -15 0.19514 66.7264 3

2 -13 -14 0.18027 44.4716 3

2 13 -14-141.237 115.984 10

2 -13 -13 223.254 95.6243 3

2 13 -13 325.351 132.647 10

-2 -13 12 27.4535 52.9703 4

2 -13 -12-4.73646 49.8124 3

2 13 -12 10.5208 51.2870 10

2 -13 -12-85.4491 52.6126 9

2 -13 -11 42.9902 59.4878 3

2 13 -11 82.7232 79.5657 10

2 -13 -11 77.9391 48.3023 9

2 -13 -10-38.3251 37.3156 5

-2 -13 10-10.7964 37.4291 5

2 -13 -10-5.29254 44.5838 3

2 -13 -10 133.366 123.575 1

2 13 -10-0.75613 62.2115 10

-2 -13 10-46.5547 34.6360 8

-2 -13 10-39.8734 38.0087 7

2 -13 -10-12.3832 31.1733 9

2 -13 -9-14.2046 25.1730 5

-2 -13 9-11.6294 40.7181 5

2 -13 -9-21.8324 50.9845 3

-2 -13 9 14.3047 19.5392 1

-2 -13 9-23.3913 25.8141 1

2 -13 -9 72.1522 78.2011 1

2 13 -9 34.6705 68.9228 10

-2 -13 9-23.0670 34.5044 7

2 -13 -9 59.9989 43.7091 9

-2 -13 9-5.76102 33.5953 8

2 -13 -8 408.158 104.616 3

2 -13 -8 348.294 94.0483 5

-2 -13 8 345.181 104.562 5

-2 -13 8 374.479 52.1616 1

2 -13 -8 370.441 128.195 1

2 13 -8 617.919 162.943 10

2 -13 -8 404.878 86.3379 9

-2 -13 8 350.279 80.3309 7

2 -13 -7-16.1626 55.8036 3

2 -13 -7-17.8705 43.4477 5

-2 -13 7-42.5205 48.8573 5

-2 -13 7 26.8906 24.9992 1

2 -13 -7 16.6612 58.3305 1

-2 13 7 101.586 67.7675 10

2 13 -7-5.15012 62.1319 10

2 -13 -7-47.3616 51.1230 9

-2 -13 7 22.2959 34.3176 7

2 -13 -6 26.6040 37.3799 4

2 -13 -6 21.5411 45.7212 5

-2 -13 6-9.72010 55.6242 5

2 -13 -6 35.7577 60.5530 3

2 -13 -6 150.564 74.0946 1

-2 -13 6 14.7217 20.3971 1

-2 13 6-9.61049 39.4851 1

-2 13 6-70.7693 57.7029 10

2 13 -6 54.9307 61.3832 10

2 -13 -6 90.9755 51.8436 8

-2 -13 6 63.8585 29.4153 7

-2 13 6 44.1451 48.6032 6

-2 -13 5 5432.24 398.044 5

2 -13 -5 5113.10 360.994 5

2 -13 -5 5588.99 411.582 4

2 -13 -5 5929.42 340.272 3

-2 -13 5 4689.44 247.553 1

-2 13 5 5759.07 406.402 1

2 -13 -5 5481.96 425.906 1

-2 13 5 5405.78 251.240 2

-2 13 5 5993.90 332.968 9

2 -13 -5 5784.50 426.350 8

-2 13 5 6468.62 434.927 6

2 -13 -4 7807.04 452.376 5

2 -13 -4 8468.95 478.125 4

2 -13 -4 8312.96 398.793 3

-2 -13 4 8716.99 504.722 5

-2 13 4 9783.94 504.047 1

-2 13 4 7536.30 339.035 2

2 -13 -4 8392.36 498.752 1

-2 -13 4 8467.46 329.523 1

-2 13 4 8682.65 395.214 9

2 -13 -4 9598.56 538.766 8

-2 13 3 71504.1 1249.50 3

2 -13 -3 60928.5 1277.79 5

-2 -13 3 61948.3 1331.01 5

-2 13 3 58974.5 1013.25 2

-2 -13 3 59860.0 925.289 1

2 -13 -3 63992.9 1329.93 1

-2 13 3 60217.9 1193.44 1

-2 13 3 69116.2 1115.73 9

2 -13 -3 63516.7 1366.37 8

2 13 -3 70979.1 1084.30 7

2 -13 -2 437.503 119.083 1

-2 -13 2 495.315 94.2421 1

2 13 -2 308.613 82.6366 3

2 13 -2 336.256 78.5208 1

-2 13 2 385.961 102.691 1

-2 13 2 253.122 68.8690 3

2 -13 -2 566.103 133.236 5

-2 13 2 347.288 87.4652 9

2 13 -2 400.522 83.9862 7

2 -13 -2 401.589 102.532 8

-2 13 1 6232.42 339.448 1

-2 -13 1 6175.96 325.903 1

2 13 -1 6418.03 342.113 1

2 -13 -1 6458.01 385.244 1

2 13 -1 7052.81 375.311 3

2 -13 -1 6715.57 439.244 5

-2 13 1 7334.37 382.367 3

2 13 -1 7357.46 354.241 7

-2 13 1 7098.94 368.939 9

2 -13 -1 7262.72 443.788 8

2 13 0 148.146 54.8084 1

-2 13 0 165.773 56.9885 1

2 -13 0 144.154 57.3578 1

-2 -13 0 198.377 65.0356 1

-2 13 0 93.5848 39.9254 3

2 -13 0 140.711 68.7860 5

2 13 0 123.118 48.3662 3

2 -13 0 224.365 82.0929 8

-2 -13 -1 4713.68 322.238 1

-2 13 -1 5068.70 259.000 1

2 13 1 4588.57 311.807 1

2 -13 1 4948.54 291.015 1

-2 -13 -1 3396.36 244.872 3

2 13 1 3331.48 250.252 3

2 -13 1 4685.82 372.427 5

-2 13 -1 3727.51 279.290 3

2 -13 1 4475.26 345.331 8

2 -13 2 732.095 101.945 1

-2 -13 -2 723.025 135.201 1

-2 13 -2 744.614 86.6875 1

-2 13 -2 864.453 135.833 3

2 -13 2 799.837 159.432 5

-2 -13 -2 891.343 123.469 3

2 13 2 821.717 119.809 3

-2 13 -2 775.548 94.3757 7

2 -13 2 718.008 135.856 8

2 -13 3 1155.16 192.943 5

-2 13 -3 1275.38 166.329 3

2 13 3 1234.72 150.470 3

-2 -13 -3 1127.27 136.065 3

-2 13 -3 1139.85 184.925 5

-2 -13 -3 974.418 155.995 1

-2 13 -3 1130.92 93.5626 1

2 13 3 1174.54 115.783 9

2 -13 3 1071.36 163.602 8

2 13 3 1307.26 187.010 6

-2 13 -3 1142.76 110.039 7

2 -13 4 8787.09 540.723 5

-2 13 -4 9398.07 469.465 3

-2 13 -4 8223.75 499.644 5

-2 -13 -4 8455.30 386.831 3

-2 -13 -4 7655.05 470.370 1

2 13 4 7997.67 287.948 9

2 13 4 8387.18 503.620 6

2 -13 4 7939.24 433.176 8

-2 -13 -5 165.730 64.2311 3

-2 13 -5 930.156 182.128 5

2 -13 5 349.951 109.876 5

2 13 5 402.762 121.694 1

-2 13 -5 199.864 83.0984 3

-2 -13 -5 519.495 179.781 1

-2 13 -5 274.815 88.8974 10

2 13 5 189.507 89.1287 6

-2 -13 -5 773.584 158.119 8

-2 13 -6 43.7758 73.1890 5

2 -13 6 26.9176 59.6857 5

-2 -13 -6 39.4536 32.7029 3

-2 -13 -6-33.9172 69.7445 1

2 13 6 55.5553 37.4934 10

-2 13 -6 55.4760 47.6424 10

-2 -13 -6 76.2574 61.6824 8

-2 -13 -7 164.187 62.7151 3

2 -13 7 161.285 80.8431 5

-2 13 -7 254.854 116.083 5

-2 -13 -7 135.835 87.7223 1

-2 13 -7 168.180 76.8242 10

2 13 7 218.056 79.8165 10

-2 -13 -7 160.793 81.1207 8

-2 -13 -8 250.773 79.7583 3

2 -13 8 304.890 49.8363 4

-2 -13 -8 244.419 103.136 1

2 13 8 303.922 99.6855 10

-2 13 -8 224.350 92.2877 10

2 -13 8 291.980 105.972 5

-2 -13 -8 186.032 87.1401 8

-2 -13 -9 664.662 127.576 3

-2 -13 -9 563.194 161.734 1

-2 13 -9 490.507 142.143 10

2 13 9 490.078 125.993 10

2 -13 9 422.910 129.462 5

-2 -13 -9 433.256 126.254 8

-2 -13 -10 7.30517 32.2519 3

-2 13 -10 140.334 88.5332 10

-2 -13 -10 24.2741 58.5024 1

-2 -13 -11 464.605 118.741 3

-2 13 -11 350.550 143.061 10

-2 -13 -11 251.435 117.529 1

-2 -13 -12 34.6403 53.6291 3

-2 13 -12 123.939 98.5921 10

-2 -13 -12 168.030 116.235 1

-2 13 -13-22.8630 106.321 10

-2 -13 -13-83.9839 69.5736 1

-2 13 -14-55.0020 51.5835 10

2 -14 -15 81.5262 70.7637 3

2 -14 -14 99.8501 68.8602 3

2 14 -14 21.8469 97.4807 10

2 -14 -13 107.120 77.3291 3

2 14 -13 209.095 106.159 10

2 -14 -12-25.8763 48.3544 3

-2 -14 12 22.8799 52.2793 4

2 14 -12 39.0973 57.1443 10

2 -14 -12 44.0722 51.6419 9

2 -14 -11-24.2946 57.5618 3

2 14 -11 11.3067 45.4808 10

2 -14 -11 7.82253 40.9786 9

2 -14 -10-67.5994 68.8467 3

2 -14 -10 26.9721 28.3734 5

-2 -14 10 11.2738 35.0236 5

2 -14 -10 111.703 110.206 1

2 14 -10 10.5476 64.2815 10

-2 -14 10-9.58525 22.2335 8

-2 -14 10 28.9639 30.4872 7

2 -14 -10 41.9423 39.5954 9

2 -14 -9 111.429 70.1715 3

2 -14 -9 89.9776 61.0645 5

-2 -14 9 59.2131 49.7117 5

-2 -14 9 20.7247 11.3524 1

-2 -14 9 42.2222 19.7101 1

2 -14 -9 138.812 115.485 1

2 14 -9 103.344 69.0245 10

2 -14 -9 54.7511 49.9602 9

-2 -14 9 44.8307 27.0479 8

-2 -14 9 106.288 54.8188 7

2 -14 -8 478.393 112.206 5

2 -14 -8 725.584 146.442 3

-2 -14 8 599.407 149.550 5

2 -14 -8 708.932 183.491 1

-2 -14 8 518.978 64.0737 1

2 14 -8 582.454 145.701 10

-2 -14 8 595.410 98.8508 7

2 -14 -8 635.543 107.336 9

-2 -14 8 623.289 122.554 8

2 -14 -7 2177.91 240.513 5

-2 -14 7 2149.84 268.329 5

2 -14 -7 2483.12 245.438 3

-2 -14 7 2175.60 147.770 1

2 -14 -7 2328.38 319.246 1

-2 14 7 2538.62 291.743 10

2 14 -7 2222.90 272.683 10

2 -14 -6 2168.31 220.681 3

-2 -14 6 2195.76 267.683 5

2 -14 -6 1823.97 224.417 5

-2 -14 6 1892.82 149.058 1

2 -14 -6 2370.10 296.944 1

2 14 -6 2229.34 257.464 10

-2 14 6 2217.90 263.841 10

2 -14 -6 2073.58 271.119 8

-2 -14 5 5741.53 429.089 5

2 -14 -5 5318.18 385.591 5

2 -14 -5 6018.06 354.345 3

2 -14 -5 6304.43 436.805 4

-2 14 5 6014.24 415.950 1

-2 -14 5 5615.22 265.942 1

2 -14 -5 5552.70 437.623 1

-2 14 5 5337.04 269.255 2

2 14 -5 6334.51 410.260 10

-2 14 5 6064.42 346.176 9

-2 14 5 6509.67 451.469 6

2 -14 -5 5938.64 445.976 8

2 -14 -4 721.532 123.628 3

-2 -14 4 830.174 169.684 5

2 -14 -4 836.142 159.904 5

-2 -14 4 748.355 106.095 1

-2 14 4 660.196 104.418 2

-2 14 4 799.213 141.441 1

2 -14 -4 570.331 140.827 1

-2 14 4 734.919 119.543 9

2 -14 -4 720.167 157.268 8

-2 14 3 1111.19 166.411 3

2 14 -3 1205.41 172.647 3

-2 -14 3 1248.85 200.272 5

2 -14 -3 996.698 171.886 5

-2 -14 3 967.425 125.180 1

-2 14 3 1002.78 167.838 1

2 -14 -3 1129.87 198.384 1

-2 14 3 1120.68 141.933 9

2 14 -3 1121.63 143.040 7

2 -14 -3 1041.39 183.457 8

-2 14 2 1085.38 155.759 3

2 -14 -2 977.141 172.014 5

2 14 -2 1052.51 149.883 3

-2 14 2 963.555 149.248 1

-2 -14 2 895.792 126.646 1

2 14 -2 923.463 128.414 1

2 -14 -2 1135.09 178.378 1

-2 14 2 998.807 139.456 9

2 -14 -2 928.281 167.578 8

2 14 -2 1045.69 132.627 7

2 14 -1 5757.73 339.443 1

-2 14 1 5618.72 332.079 1

-2 -14 1 5422.95 325.575 1

2 -14 -1 5988.93 380.828 1

2 -14 -1 5926.60 424.826 5

2 14 -1 6251.31 363.393 3

-2 14 1 6481.38 377.063 3

2 14 -1 6333.24 340.850 7

2 -14 -1 6045.45 420.975 8

-2 14 1 6182.98 358.188 9

2 14 0 19.6737 27.1901 1

-2 -14 0 10.8756 29.0386 1

2 -14 0-30.2787 45.6199 1

-2 14 0-19.0470 32.3218 1

-2 14 0 26.2614 42.7360 3

2 -14 0 64.8461 64.1558 5

2 14 0 24.8966 34.0245 3

2 -14 0-13.7295 41.4961 8

-2 14 -1 9570.46 370.736 1

2 -14 1 10119.4 436.375 1

-2 -14 -1 9329.26 460.300 1

2 14 1 10279.9 487.000 1

2 14 1 11676.3 484.858 3

2 -14 1 10244.3 566.299 5

-2 14 -1 11564.7 509.140 3

2 -14 1 10427.1 543.844 8

2 -14 2 803.191 112.405 1

-2 -14 -2 781.137 145.586 1

-2 14 -2 703.498 92.1780 1

2 14 2 845.579 147.019 1

-2 -14 -2 835.228 125.813 3

2 -14 2 968.998 175.199 5

2 14 2 1031.14 144.148 3

-2 14 -2 1021.47 156.164 3

2 -14 2 850.153 150.967 8

-2 14 -2 946.015 109.081 7

2 -14 3 3249.95 382.861 5

-2 -14 -3 2838.50 231.558 3

-2 14 -3 2742.31 263.500 3

2 14 3 2464.66 233.491 3

-2 14 -3 3743.98 193.105 1

-2 -14 -3 5093.84 395.910 1

2 14 3 4781.42 369.171 1

2 14 3 2329.58 173.433 9

2 -14 3 3761.40 334.413 8

-2 14 -3 2800.04 185.427 7

2 -14 4 301.704 111.031 5

-2 14 -4 119.350 55.2971 5

-2 -14 -4 144.907 56.1317 3

-2 14 -4 213.495 89.3913 3

2 14 4 112.404 74.5424 1

-2 -14 -4 341.210 122.181 1

2 14 4 162.428 44.6505 9

2 -14 4 169.105 71.9539 8

2 14 4 111.883 73.6577 6

-2 -14 -5 983.806 142.619 3

2 -14 5 863.605 180.271 5

-2 14 -5 1022.57 186.305 5

-2 14 -5 982.338 159.534 3

-2 -14 -5 806.023 165.373 1

-2 14 -5 1011.48 161.835 10

-2 -14 -5 812.736 159.474 8

-2 -14 -6 1283.05 161.504 3

-2 -14 -6 1190.54 207.362 1

-2 14 -6 1250.60 191.331 10

2 14 6 1094.79 170.147 10

2 -14 6 1260.70 219.932 5

-2 -14 -6 1113.87 193.810 8

2 -14 7 735.143 178.475 5

-2 -14 -7 510.737 104.590 3

-2 -14 -7 705.750 180.508 1

2 14 7 552.055 122.720 10

-2 14 -7 579.667 138.556 10

-2 -14 -7 545.803 139.916 8

-2 -14 -8 1155.03 162.250 3

2 -14 8 1039.34 94.5428 4

-2 -14 -8 764.788 186.024 1

-2 14 -8 1073.66 198.183 10

2 14 8 898.197 172.614 10

2 -14 8 911.081 196.953 5

-2 -14 -9 152.600 67.0436 3

2 14 9 11.1094 45.2694 10

-2 14 -9 84.3459 68.1876 10

-2 -14 -9 104.722 67.1425 1

-2 -14 -10 77.2472 45.4574 3

-2 14 -10 27.8145 66.3271 10

-2 -14 -10 172.594 106.421 1

-2 -14 -11-48.6773 55.8677 3

-2 14 -11 5.54484 44.2272 10

-2 -14 -11-33.6798 92.0281 1

-2 -14 -12-23.1795 47.8946 3

-2 14 -12-144.228 103.151 10

-2 -14 -12-100.141 78.6585 1

-2 14 -13-144.478 103.250 10

-2 -14 -13 43.8494 92.7953 1

-2 14 -14 30.3897 49.1326 10

2 -15 -15-18.4443 38.3573 3

2 -15 -14 37.3357 61.7761 3

2 15 -14 105.162 68.2851 10

2 -15 -13 73.6680 42.7538 3

2 15 -13 117.254 104.032 10

2 -15 -12 42.2642 47.7861 3

2 15 -12 13.1553 58.9078 10

2 -15 -11 90.3158 80.9541 3

2 15 -11 28.5591 58.5141 10

2 -15 -11 0.55649 22.9614 9

-2 -15 10 38.3868 36.1259 5

2 -15 -10-36.8023 70.4387 3

2 -15 -10 85.2395 71.0758 1

2 15 -10 73.6857 79.4122 10

-2 -15 10-9.85132 36.4336 7

2 -15 -10 67.9498 47.1366 9

2 -15 -9 155.954 67.3898 3

-2 -15 9 212.068 127.014 5

2 -15 -9 145.082 53.0050 5

-2 -15 9 160.224 29.0914 1

-2 -15 9 85.4255 20.1662 1

2 -15 -9 302.992 151.094 1

2 15 -9 205.379 105.508 10

-2 -15 9 136.341 52.9984 7

-2 -15 9 70.1465 29.2724 8

2 -15 -8 174.956 71.3977 3

2 -15 -8 252.977 84.8573 5

-2 -15 8 206.866 88.0436 5

2 -15 -8 262.637 128.181 1

-2 -15 8 153.683 38.9305 1

2 15 -8 293.024 119.726 10

-2 -15 8 306.926 94.0903 8

-2 -15 7 312.883 109.517 5

2 -15 -7 264.507 98.7804 5

2 -15 -7 484.755 120.656 3

2 -15 -7 192.526 108.555 1

-2 -15 7 254.799 56.3819 1

2 15 -7 330.087 121.273 10

2 -15 -6 271.631 106.400 5

-2 -15 6 199.197 96.4447 5

2 -15 -6 274.410 85.0546 3

-2 -15 6 296.441 64.8744 1

2 -15 -6 297.008 111.394 1

2 15 -6 312.785 96.9463 10

-2 15 6 338.975 112.550 10

2 -15 -6 331.469 122.773 8

2 -15 -5 2359.47 281.512 4

-2 -15 5 2452.50 287.858 5

2 -15 -5 2529.26 235.773 3

2 -15 -5 2314.49 265.513 5

-2 15 5 2319.84 268.732 1

2 -15 -5 2608.28 310.699 1

-2 -15 5 2483.50 185.907 1

-2 15 5 2166.43 179.819 2

-2 15 5 2486.64 228.311 9

2 15 -5 2786.76 276.213 10

2 -15 -5 2488.33 293.735 8

-2 15 5 2586.81 293.932 6

-2 15 4 8055.61 462.158 3

2 -15 -4 7335.11 405.198 3

-2 -15 4 8642.52 550.610 5

2 -15 -4 7038.30 467.286 5

-2 -15 4 7949.94 358.844 1

2 -15 -4 7759.20 511.019 1

-2 15 4 7174.19 368.315 2

-2 15 4 7883.01 489.610 1

-2 15 4 7378.97 390.889 9

2 15 -4 7530.58 434.248 10

2 -15 -4 7779.05 517.132 8

2 -15 -3 27332.5 923.417 5

-2 -15 3 28709.0 989.905 5

2 15 -3 32960.0 890.619 3

-2 15 3 33241.5 907.966 3

2 -15 -3 31144.3 832.361 3

2 -15 -3 28198.8 941.393 1

-2 15 3 27323.3 850.393 1

-2 -15 3 26825.1 679.963 1

-2 15 3 31094.8 802.445 9

2 -15 -3 28963.5 983.163 8

2 15 -3 31330.3 778.039 7

2 15 -2 1433.73 181.742 3

2 -15 -2 2089.50 258.241 5

-2 15 2 1378.27 184.218 3

-2 -15 2 2441.70 292.450 5

-2 -15 2 1988.42 197.174 1

-2 15 2 1605.45 221.505 1

2 15 -2 1507.95 175.285 1

2 -15 -2 1788.96 249.687 1

-2 15 2 1175.21 159.736 9

2 -15 -2 1787.59 234.391 8

2 15 -2 1385.34 166.646 7

2 15 -1 1634.29 185.612 1

-2 -15 1 2595.33 238.269 1

2 -15 -1 2311.59 247.395 1

-2 15 1 2453.66 230.514 1

-2 15 1 638.259 130.978 3

2 -15 -1 2325.36 272.170 5

2 15 -1 782.309 140.988 3

2 15 -1 1088.12 155.630 7

2 -15 -1 1927.11 260.634 8

-2 15 1 722.358 133.661 9

-2 -15 0-18.6875 43.3805 1

2 15 0 22.3766 41.4866 1

-2 15 0-31.8341 36.1567 1

2 -15 0 32.9181 53.5926 1

2 15 0 34.7320 40.8152 3

2 -15 0 34.3157 59.5660 5

-2 15 0 22.1409 47.0618 3

2 -15 0 45.5298 57.6766 8

-2 -15 -1 2586.66 255.135 1

2 -15 1 2262.38 213.244 1

-2 15 -1 2402.17 195.646 1

2 15 1 2016.43 228.838 1

-2 15 -1 1543.54 193.405 3

2 -15 1 2179.28 267.221 5

2 15 1 1355.91 175.627 3

2 -15 1 2152.90 261.288 8

-2 -15 -2 358.841 92.8224 3

2 -15 2 277.442 70.4630 1

-2 15 -2 311.980 73.4249 1

2 15 2 373.110 118.447 1

-2 -15 -2 412.945 123.564 1

-2 15 -2 333.722 98.1200 3

2 15 2 300.709 79.8164 3

2 -15 2 415.952 124.799 5

-2 15 -2 331.003 74.6702 7

2 -15 2 310.995 106.607 8

-2 15 -3 249.695 48.4656 1

-2 -15 -3 229.239 82.3262 1

2 15 3 446.784 124.873 1

2 15 3 403.808 90.0716 3

-2 15 -3 280.147 84.4100 3

2 -15 3 317.402 115.281 5

-2 -15 -3 328.841 84.0458 3

2 15 3 258.923 60.1136 9

-2 15 -3 299.415 57.4684 7

2 -15 3 328.000 111.390 8

-2 -15 -4 725.259 124.601 3

2 15 4 863.305 141.080 3

2 -15 4 787.274 176.518 5

-2 -15 -4 856.544 173.997 1

-2 15 -4 934.927 154.612 3

2 15 4 818.347 105.771 9

2 15 4 890.146 177.814 6

2 -15 4 749.758 147.122 8

-2 -15 -4 740.730 152.287 8

-2 -15 -5 586.471 110.073 3

2 -15 5 700.182 178.238 5

-2 -15 -5 739.869 167.736 1

-2 15 -5 666.109 137.077 10

-2 -15 -5 709.337 150.109 8

2 -15 6 178.807 96.3820 5

-2 -15 -6 255.529 72.3653 3

-2 15 -6 129.353 76.9481 10

2 15 6 116.598 69.6879 10

-2 -15 -6 232.590 87.7211 1

-2 -15 -7 152.379 66.0538 3

-2 15 -7 81.2284 69.3220 10

2 15 7 117.777 68.9008 10

-2 -15 -7 26.0314 65.2476 1

-2 -15 -8 167.113 70.0107 3

-2 15 -8-85.9318 82.7847 10

2 15 8 212.764 94.8954 10

-2 -15 -8 181.503 91.6074 1

-2 -15 -9-48.2291 45.1845 3

-2 15 -9 39.6807 80.7950 10

-2 -15 -9 45.3975 72.4342 1

-2 -15 -10 15.4661 41.5318 3

-2 15 -10 4.13326 41.0935 10

-2 -15 -10 53.2689 70.3357 1

-2 -15 -11 222.131 88.6545 3

-2 15 -11 148.992 94.0488 10

-2 -15 -11 68.3637 95.4346 1

-2 -15 -12-8.10376 34.7031 3

-2 15 -12 3.11634 43.0116 10

-2 15 -13 103.072 119.603 10

-2 -15 -13 200.282 124.190 1

2 -16 -14 106.613 98.0396 3

2 -16 -13 346.199 118.967 3

2 16 -13 171.453 122.747 10

2 -16 -12 5.87206 37.3369 3

2 16 -12-83.0568 105.395 10

2 -16 -11-3.33473 42.7409 3

2 16 -11 2.07091 46.3517 10

2 -16 -10 45.3641 51.9844 3

2 16 -10-59.6143 66.9053 10

2 -16 -9-11.7695 33.3128 5

2 -16 -9-4.99733 42.1586 3

2 -16 -9 78.9372 84.4862 1

2 16 -9-30.4448 48.2828 10

-2 -16 9 30.4841 25.0174 8

2 -16 -8 6.20966 28.8262 5

-2 -16 8-60.1765 58.8053 5

2 -16 -8-25.3502 55.0603 3

-2 -16 8-8.31520 18.4090 1

2 -16 -8-41.1926 66.9677 1

2 16 -8-64.7380 65.6200 10

-2 -16 8 10.6701 36.2625 8

2 -16 -7 207.871 89.8108 5

2 -16 -7 151.178 67.7699 3

-2 -16 7 211.106 95.4386 5

2 16 -7 272.724 88.4274 10

2 -16 -7 307.380 142.427 1

-2 -16 7 229.344 50.1386 1

2 -16 -6 1083.74 186.242 5

2 -16 -6 1285.19 178.326 3

-2 -16 6 1231.56 229.142 5

2 16 -6 1375.22 209.273 10

-2 -16 6 1321.28 136.954 1

-2 16 6 1266.15 132.109 2

2 -16 -6 1567.18 263.637 1

2 -16 -5-37.7528 54.4121 3

2 -16 -5 93.7574 65.3057 5

-2 -16 5 91.0447 85.6167 5

-2 16 5 37.4547 28.5656 2

2 -16 -5-31.9301 63.9257 1

-2 -16 5 10.3508 28.4683 1

2 16 -5-43.6503 78.7075 10

-2 16 5 44.0984 23.7874 9

2 -16 -4 322.099 104.363 5

2 -16 -4 284.212 99.0524 3

-2 -16 4 440.774 132.991 5

-2 16 4 401.695 103.293 3

-2 16 4 260.896 92.4737 1

-2 -16 4 285.868 77.6873 1

2 -16 -4 357.014 121.230 1

2 16 -4 358.062 105.137 3

-2 16 4 272.961 80.1739 2

-2 16 4 291.776 79.0541 9

2 16 -4 217.021 85.3774 10

2 -16 -4 298.230 108.814 8

-2 -16 3 1165.50 209.838 5

2 16 -3 1633.60 207.091 3

2 -16 -3 1037.86 183.687 5

2 -16 -3 1398.84 178.440 3

-2 16 3 1471.02 197.397 3

-2 16 3 1183.12 176.930 1

-2 -16 3 1176.42 148.269 1

2 -16 -3 1357.20 222.057 1

-2 16 3 1272.22 171.662 9

2 -16 -3 1263.63 210.725 8

2 16 -3 1250.74 168.379 7

2 -16 -2 2510.93 241.139 3

-2 -16 2 3178.05 331.828 5

-2 16 2 2907.43 273.192 3

2 16 -2 2657.09 259.352 3

2 -16 -2 2921.87 318.473 5

2 16 -2 2579.19 233.572 1

-2 16 2 2816.13 272.839 1

-2 -16 2 2869.13 244.793 1

2 -16 -2 2913.76 299.876 1

-2 16 2 2644.68 247.062 9

2 -16 -2 2977.93 320.650 8

2 16 -2 2751.77 243.195 7

-2 16 1 10427.0 481.820 1

2 16 -1 10189.8 477.836 1

2 -16 -1 10899.5 553.553 1

-2 -16 1 9682.30 467.897 1

2 -16 -1 10822.9 614.159 5

2 -16 -1 10693.2 504.202 3

-2 16 1 11750.0 544.888 3

2 16 -1 11818.9 541.362 3

2 16 -1 11185.7 495.228 7

2 -16 -1 10735.8 611.055 8

-2 16 1 10584.4 506.472 9

-2 16 0 1112.98 144.814 1

2 -16 0 1156.63 171.434 1

2 16 0 993.162 160.935 1

-2 -16 0 988.185 152.741 1

-2 16 0 1094.98 164.064 3

2 16 0 1039.38 160.079 3

2 -16 0 977.704 180.983 5

2 -16 0 1127.74 193.605 8

2 16 1 671.273 139.592 1

-2 16 -1 644.878 106.042 1

-2 -16 -1 918.659 153.015 1

2 -16 1 733.929 129.168 1

-2 16 -1 527.473 115.772 3

2 16 1 480.959 109.318 3

2 -16 1 824.114 166.135 5

2 -16 1 739.113 160.408 8

-2 -16 -2 58.4367 52.6001 1

-2 -16 -2 130.636 57.8387 3

2 -16 2 129.164 47.2930 1

2 16 2 88.4401 52.8805 1

-2 16 -2 77.9672 19.9245 1

2 -16 2 90.4176 82.5350 5

2 16 2 101.750 41.4582 3

-2 16 -2 158.335 78.8538 3

2 16 2 87.0401 42.5770 9

2 -16 2 74.5735 58.5931 8

-2 16 -2 96.9329 40.2078 7

2 16 3 359.546 116.541 1

-2 -16 -3 748.502 182.961 1

-2 -16 -3 154.181 86.0871 3

-2 16 -3 461.930 78.0151 1

-2 16 -3 205.826 89.5332 3

2 16 3 112.642 70.0685 3

2 -16 3 497.955 155.388 5

2 16 3 101.939 46.7704 9

2 -16 3 263.271 139.642 8

-2 16 -3 284.210 69.8449 7

-2 -16 -4-33.2159 50.1214 3

2 16 4 37.8565 48.6432 3

-2 -16 -4 4.40784 48.9248 1

2 16 4-18.4324 26.1484 9

2 -16 4 74.8518 63.6587 8

-2 -16 -5-2.13156 44.6503 3

-2 16 -5-51.9556 45.1496 10

-2 -16 -6 281.305 88.4825 3

2 16 6 391.361 108.889 10

-2 16 -6 458.775 130.610 10

-2 -16 -6 370.653 133.839 1

-2 -16 -7-23.3040 43.7710 3

2 16 7-3.05696 50.1052 10

-2 16 -7-41.3468 49.1701 10

-2 -16 -7-74.6665 62.9235 1

-2 -16 -8 40.1275 38.2746 3

-2 16 -8 35.3470 47.9259 10

-2 -16 -8 46.9105 65.9908 1

-2 -16 -9 47.2731 44.4364 3

-2 16 -9 60.6063 60.4111 10

-2 -16 -9 40.2925 54.2307 1

-2 -16 -10 6.29095 44.2502 3

-2 16 -10-50.0988 98.0561 10

-2 -16 -10-39.3781 82.5542 1

-2 -16 -11 113.160 77.1372 3

-2 16 -11-61.7795 80.1427 10

-2 -16 -11 90.3260 53.3531 1

-2 -16 -12 51.6752 51.2244 3

-2 16 -12-58.5503 87.8009 10

-2 -16 -12 155.229 144.557 1

-2 16 -13-58.5950 66.2358 10

2 -17 -14-70.3039 76.6384 3

2 -17 -13-59.0470 67.0061 3

2 17 -13 0.28175 57.5247 10

2 -17 -12 24.1557 54.9123 3

2 17 -12 6.81257 56.6937 10

2 -17 -11-37.6386 72.6018 3

2 17 -11 120.269 116.091 10

2 -17 -10 143.845 74.0637 3

2 -17 -10 25.5674 68.0638 1

2 17 -10 2.57953 64.6391 10

2 -17 -9 23.2592 24.2379 5

2 -17 -9-8.41344 44.9119 3

2 -17 -9 5.93847 77.6946 1

2 17 -9-30.1498 50.4509 10

-2 -17 9 24.9599 31.1383 8

2 -17 -8 6.23775 46.2564 5

2 -17 -8 42.4508 60.2408 3

-2 -17 8-17.9337 26.0467 1

2 -17 -8-148.969 108.814 1

2 17 -8-5.50336 43.0830 10

-2 -17 8-7.36669 32.8786 8

2 -17 -7 409.513 116.382 5

2 -17 -7 406.544 108.423 3

-2 -17 7 314.381 59.6965 1

2 -17 -7 392.426 154.965 1

2 17 -7 416.422 132.471 10

-2 -17 7 260.877 87.5828 8

2 -17 -6 264.156 111.527 5

2 -17 -6 275.859 87.9821 3

2 17 -6 319.423 109.008 10

-2 17 6 287.264 64.9899 2

-2 -17 6 257.974 61.3417 1

2 -17 -6 227.692 109.779 1

2 -17 -5 3876.39 317.623 3

2 -17 -5 3218.69 341.100 5

-2 17 5 3199.79 241.338 2

2 -17 -5 3826.43 395.271 1

-2 -17 5 3359.19 235.116 1

2 17 -5 3736.96 345.437 10

-2 17 5 3902.70 301.865 9

2 -17 -4 1451.23 191.668 3

-2 -17 4 1478.44 248.921 5

2 -17 -4 1599.74 239.905 5

2 -17 -4 1380.58 228.785 1

-2 17 4 1905.30 247.093 1

-2 -17 4 1484.00 168.164 1

-2 17 4 1416.35 180.955 2

-2 17 4 1404.49 182.135 9

2 17 -4 1505.60 203.588 10

2 17 -4 1574.39 187.134 7

-2 17 3 19534.7 748.346 3

2 -17 -3 16037.5 775.131 5

2 17 -3 17720.0 701.466 3

-2 -17 3 16617.0 802.894 5

2 -17 -3 18170.4 674.970 3

-2 17 3 16879.1 709.937 1

2 -17 -3 17129.9 766.351 1

-2 -17 3 15971.9 571.410 1

-2 17 3 17961.9 652.926 9

2 17 -3 18138.4 631.234 7

2 -17 -2 1703.58 250.473 5

-2 17 2 1861.50 227.740 3

2 -17 -2 1662.72 208.090 3

-2 -17 2 1636.39 250.275 5

2 17 -2 1629.27 207.711 3

2 17 -2 1725.32 203.212 1

2 -17 -2 1551.52 226.445 1

-2 -17 2 1325.06 174.600 1

-2 17 2 1564.88 203.343 1

-2 17 2 1701.30 203.849 9

2 17 -2 1742.27 197.369 7

2 -17 -2 1946.12 271.419 8

2 -17 -1 3462.08 317.259 1

-2 -17 1 3032.49 270.592 1

-2 17 1 3110.62 272.947 1

2 17 -1 3185.79 282.719 1

2 -17 -1 3320.33 288.815 3

-2 17 1 3436.84 305.445 3

2 17 -1 3582.00 311.967 3

2 -17 -1 3240.50 343.736 5

-2 -17 1 3246.76 344.281 5

-2 17 1 3662.86 305.112 9

2 -17 -1 3036.61 336.556 8

2 17 -1 3540.52 288.829 7

-2 -17 0 186.139 72.1063 1

2 17 0 250.905 92.4149 1

-2 17 0 289.092 82.5344 1

2 -17 0 88.4486 51.8015 1

2 17 0 107.979 40.8800 3

2 -17 0 198.130 96.9425 5

-2 17 0 166.880 60.0956 3

2 -17 0 241.610 107.208 8

-2 -17 -1 1041.90 178.427 1

2 17 1 793.505 151.955 1

-2 17 -1 926.949 134.667 1

2 -17 1 947.285 157.566 1

2 17 1 783.920 143.858 3

2 -17 1 1055.09 202.157 5

-2 17 -1 876.183 155.913 3

2 -17 1 1128.22 204.936 8

-2 -17 -2 68.8770 57.4893 3

2 -17 2 13.3586 44.8051 1

-2 -17 -2 9.30575 48.8260 1

-2 17 -2-14.0334 30.3119 1

2 17 2 13.7232 41.7054 1

2 17 2-64.2226 48.2706 3

2 17 2 7.00156 30.9130 9

-2 17 -2 45.4158 31.4071 7

2 -17 2 53.5080 51.2987 8

-2 17 -3 652.272 93.6623 1

-2 -17 -3 607.305 125.659 3

-2 -17 -3 656.543 165.031 1

2 17 3 504.215 116.118 3

-2 17 -3 523.220 91.1360 7

2 17 3 569.763 104.286 9

2 -17 3 365.319 118.311 8

-2 -17 -4 669.227 124.484 3

2 17 4 604.543 129.488 3

-2 -17 -4 479.917 139.744 1

2 17 4 559.624 95.6849 9

-2 17 -4 481.585 85.0799 7

2 -17 4 595.441 146.073 8

-2 -17 -5 841.061 141.773 3

2 17 5 704.618 103.265 9

-2 17 -5 853.434 161.278 10

-2 -17 -5 579.743 144.471 1

-2 -17 -6 372.598 94.6028 3

-2 17 -6 459.473 129.649 10

2 17 6 535.540 134.355 10

-2 -17 -6 391.161 135.347 1

-2 -17 -7 131.596 63.0145 3

-2 17 -7 183.342 97.5794 10

-2 -17 -7 23.6139 50.6739 1

-2 -17 -8-11.9189 36.6902 3

-2 17 -8-33.1031 57.3304 10

-2 -17 -8-4.03552 50.6571 1

-2 -17 -9-13.8557 32.5177 3

-2 -17 -9-35.7375 77.1397 1

-2 -17 -10 146.684 69.8903 3

-2 17 -10 69.5842 87.4639 10

-2 -17 -10-46.2000 64.2919 1

-2 -17 -11-96.5823 69.9322 3

-2 17 -11-86.3710 77.3116 10

-2 -17 -11 134.247 108.013 1

-2 17 -12-83.6783 93.2792 10

-2 -17 -12 94.5332 107.150 1

-2 17 -13 116.137 107.734 10

2 -18 -14 38.6424 64.0534 3

2 -18 -13 0.18528 46.6953 3

2 -18 -12 26.7614 59.7338 3

2 18 -12-117.398 99.4690 10

2 -18 -11 32.6992 55.1081 3

2 18 -11-192.045 111.017 10

2 -18 -10 17.6204 36.3123 3

2 18 -10 120.454 87.3687 10

2 -18 -9 161.157 70.6058 3

2 -18 -9 159.574 105.375 1

2 18 -9 57.0697 46.4797 10

2 -18 -8 11.8111 38.7658 3

2 -18 -8 12.1935 37.7464 5

-2 -18 8 42.4800 29.5300 1

2 -18 -8 54.6320 66.4386 1

2 18 -8-21.4674 52.9423 10

-2 -18 8-48.8082 61.8176 8

2 -18 -7 843.793 174.463 5

2 -18 -7 917.829 162.999 3

-2 -18 7 897.935 117.194 1

2 -18 -7 1067.40 243.751 1

2 18 -7 871.294 192.256 10

-2 -18 7 840.513 165.097 8

2 -18 -6 273.625 109.797 5

2 -18 -6 330.862 99.9518 3

2 -18 -6 334.196 110.431 1

-2 -18 6 215.814 63.1693 1

-2 18 6 219.557 55.8900 2

2 18 -6 285.482 111.039 10

-2 -18 6 253.328 81.5793 8

2 -18 -5 4810.81 423.093 5

2 -18 -5 5602.18 395.756 3

-2 -18 5 4575.09 288.882 1

-2 18 5 4399.86 298.481 2

2 -18 -5 5180.89 472.964 1

-2 18 5 5071.78 360.092 9

2 18 -5 5262.38 414.248 10

2 -18 -4-113.828 67.1868 3

2 -18 -4 33.0603 38.5019 5

-2 18 4 0.74635 30.7810 2

2 -18 -4-127.329 108.902 1

-2 -18 4-57.1505 49.9168 1

2 18 -4-50.7111 54.4555 10

-2 18 4-2.85216 25.0069 9

2 18 -4-18.0709 59.2589 7

2 -18 -3 3455.81 301.626 3

2 -18 -3 3522.99 366.005 5

-2 -18 3 2920.43 256.959 1

-2 18 3 3256.95 312.851 1

2 -18 -3 3593.52 360.480 1

-2 18 3 3453.59 294.875 9

2 18 -3 3617.25 288.812 7

2 -18 -2 2369.14 252.830 3

-2 -18 2 2565.15 249.335 1

2 -18 -2 2323.50 283.540 1

-2 18 2 2461.32 257.830 1

2 18 -2 2269.91 237.087 1

-2 18 2 2347.72 247.848 9

2 18 -2 2596.29 249.068 7

-2 -18 1 9576.45 503.428 1

2 -18 -1 9956.51 559.842 1

2 18 -1 9645.60 497.346 1

-2 18 1 9707.25 494.825 1

2 18 -1 10074.6 539.246 3

2 -18 -1 9824.63 515.260 3

-2 18 1 9710.83 512.805 9

2 18 -1 10230.0 511.740 7

-2 -18 0 717.235 143.840 1

-2 18 0 732.985 134.038 1

2 18 0 623.404 130.403 1

2 -18 0 638.319 130.377 1

2 18 0 651.636 131.689 3

2 -18 0 886.183 190.084 8

2 18 0 760.870 145.841 7

2 18 1 1700.00 228.064 1

-2 -18 -1 1942.30 250.659 1

-2 18 -1 1585.06 180.054 1

2 -18 1 1719.00 211.690 1

2 18 1 1781.70 220.804 3

2 -18 1 1949.90 282.691 8

-2 -18 -2 219.912 90.1694 1

-2 18 -2 239.766 66.1609 1

2 -18 2 236.538 86.5255 1

-2 -18 -2 161.339 74.1783 3

2 18 2 234.439 83.7096 3

2 18 2 201.784 68.9041 9

-2 18 -2 195.048 67.0632 7

2 -18 2 127.903 66.6112 8

-2 -18 -3 354.196 99.6420 3

-2 -18 -3 284.451 97.0064 1

2 -18 3 254.642 74.3777 1

-2 18 -3 257.625 62.8835 1

2 18 3 218.100 85.4528 3

2 -18 3 206.659 97.0252 8

2 18 3 205.066 69.7531 9

-2 18 -3 204.447 63.0187 7

2 18 4 67.6254 59.0647 3

-2 -18 -4-47.1296 49.9417 1

-2 -18 -4 58.3178 55.5822 3

2 18 4-16.0179 33.4209 9

2 -18 4-30.5946 50.2737 8

-2 18 -4 35.7507 42.1778 7

-2 -18 -5 3049.69 279.348 3

-2 18 -5 3846.06 355.684 10

2 18 5 3157.04 229.726 9

-2 -18 -5 3098.81 360.695 1

-2 -18 -6 90.4190 58.9917 3

-2 18 -6-5.43855 44.3747 10

-2 -18 -6 65.2732 51.9435 1

-2 -18 -7 297.090 107.959 3

-2 18 -7 197.188 84.1181 10

-2 -18 -7 205.628 105.685 1

-2 -18 -8-86.2004 58.0011 3

-2 18 -8 80.1280 89.3749 10

-2 -18 -8 83.1398 69.4437 1

-2 -18 -9 19.6728 55.7480 3

-2 18 -9 92.7735 89.1146 10

-2 -18 -9 107.156 102.099 1

-2 -18 -10 47.4349 53.0370 3

-2 18 -10-37.0608 63.1726 10

-2 -18 -10-108.085 94.7006 1

-2 -18 -11-62.0894 60.6518 3

-2 18 -11-19.1611 52.4211 10

-2 -18 -11 61.7271 100.444 1

-2 18 -12-110.811 90.8688 10

2 -19 -13-41.3651 78.6464 3

2 -19 -12-43.2514 53.1507 3

2 19 -12-40.7185 76.7615 10

2 -19 -11 112.195 82.4202 3

2 19 -11 86.3691 99.8754 10

2 -19 -10-14.6293 43.6089 3

2 19 -10 153.831 98.7233 10

2 -19 -10 69.1545 64.1081 1

2 -19 -9 64.6295 62.1251 3

2 -19 -9 38.1653 98.8831 1

2 19 -9 56.5601 42.0904 10

2 -19 -8 4.46282 38.8780 3

-2 -19 8 24.4432 22.0236 1

2 -19 -8-32.6517 51.6254 1

2 19 -8 96.9660 71.8345 10

-2 -19 8-52.2616 56.2738 8

2 -19 -7 268.371 90.6074 3

-2 -19 7 173.958 60.9055 1

2 -19 -7 277.778 115.295 1

2 19 -7 287.135 119.727 10

-2 -19 7 221.079 90.4006 8

2 -19 -6 415.871 106.932 3

-2 -19 6 416.007 89.8323 1

-2 19 6 418.202 87.0643 2

2 -19 -6 496.634 137.914 1

2 19 -6 416.638 124.141 10

-2 19 6 533.953 116.879 9

-2 -19 6 330.246 99.0240 8

2 -19 -5 3605.03 328.035 3

-2 19 5 2986.74 254.332 2

2 -19 -5 3249.32 381.371 1

-2 -19 5 3492.64 266.441 1

-2 19 5 3546.97 312.156 9

2 19 -5 3851.87 366.232 10

-2 -19 5 3187.23 349.855 8

2 -19 -4 1420.61 198.505 3

-2 19 4 1061.91 177.740 2

2 -19 -4 1630.17 257.872 1

-2 -19 4 1367.68 177.864 1

2 19 -4 1352.61 211.423 10

-2 19 4 1321.13 188.116 9

2 19 -4 1285.04 178.249 7

-2 -19 3 309.401 91.1742 1

2 -19 -3 173.391 91.2362 1

2 -19 -3 320.435 100.332 3

2 19 -3 302.927 91.7649 7

-2 19 3 275.044 96.5934 9

2 -19 -2 78.1122 46.2961 3

2 19 -2 103.429 58.4158 1

-2 -19 2 186.400 78.1322 1

-2 19 2 101.576 56.9752 1

2 -19 -2 112.930 65.2150 1

-2 19 2 26.8896 42.1208 9

2 19 -2 169.976 85.0236 7

-2 -19 1 202.463 87.9435 1

2 -19 -1-65.6980 60.8376 3

-2 19 1 45.3191 58.7118 1

2 19 -1 102.577 69.8851 1

2 -19 -1 31.2444 53.4338 1

2 19 -1 45.5665 46.5159 7

-2 19 1 74.6644 46.8904 9

2 19 0 306.431 107.389 1

-2 -19 0 349.204 125.808 1

-2 19 0 168.263 72.7654 1

2 -19 0 224.581 79.4253 1

2 19 0 263.881 100.100 7

-2 -19 -1 1614.89 259.470 1

-2 19 -1 1996.10 202.565 1

2 -19 1 2184.79 244.526 1

2 -19 2 1619.71 198.728 1

-2 19 -2 1371.89 162.794 1

-2 -19 -2 1620.53 240.741 1

2 19 2 1345.34 176.773 9

-2 19 -2 1668.70 188.582 7

-2 -19 -3 2838.87 272.880 3

-2 19 -3 2616.57 209.323 1

-2 -19 -3 2531.00 313.113 1

2 19 3 2876.21 241.398 9

-2 19 -3 2968.98 237.886 7

2 -19 3 3009.69 340.566 8

-2 -19 -4 699.779 141.484 3

-2 -19 -4 636.410 168.890 1

2 19 4 574.192 104.662 9

2 -19 4 620.838 152.423 8

-2 19 -4 568.595 104.445 7

-2 -19 -5 892.553 152.711 3

-2 19 -5 1065.77 192.889 10

2 19 5 1080.74 142.079 9

-2 -19 -5 1241.40 235.802 1

-2 -19 -6 499.159 119.065 3

-2 19 -6 399.414 130.696 10

-2 -19 -6 330.583 130.353 1

-2 -19 -7 239.103 79.7167 3

-2 19 -7 190.830 94.9472 10

-2 -19 -7 235.925 125.650 1

-2 -19 -8-22.3701 36.5586 3

-2 19 -8-50.7010 63.9109 10

-2 -19 -8 11.2393 58.0350 1

-2 -19 -9 86.7455 79.9973 3

-2 19 -9-46.6125 57.0307 10

-2 -19 -9 168.685 120.123 1

-2 -19 -10 12.5896 36.6820 3

-2 19 -10 2.32723 58.1681 10

-2 -19 -11-34.7363 46.4305 3

-2 19 -11-49.0239 55.0449 10

-2 19 -12 3.28708 83.3408 10

2 -20 -13-72.2510 48.7248 3

2 -20 -12 165.930 95.3383 3

2 -20 -11 160.762 89.1992 3

2 20 -11 190.515 110.363 10

2 -20 -10 45.6494 68.9410 3

2 20 -10 62.3592 84.9897 10

2 -20 -10 75.0235 81.0113 1

2 -20 -9-58.6566 65.6822 3

2 20 -9 131.286 94.8428 10

2 -20 -9 119.329 110.447 1

2 -20 -8 9.10836 48.2679 3

2 20 -8-68.7706 51.6767 10

2 -20 -8-74.2963 124.736 1

2 -20 -7-20.2736 53.7863 3

2 20 -7 23.6104 49.4521 10

-2 -20 7 46.4817 30.6766 1

2 -20 -7-8.08707 67.3220 1

2 -20 -6-3.15418 38.2921 3

2 20 -6-31.1811 52.5284 10

-2 20 6 102.487 53.9822 9

-2 -20 6 32.7123 41.5658 1

-2 20 6 6.57184 26.7372 2

2 -20 -6-1.79298 73.3994 1

2 -20 -5 1228.50 197.045 3

-2 20 5 854.841 145.225 2

-2 -20 5 1028.47 150.701 1

2 -20 -5 1234.19 242.410 1

-2 20 5 1121.55 179.590 9

2 20 -5 1193.26 218.135 10

2 -20 -4 116.195 61.9650 3

-2 20 4 120.127 65.4310 2

-2 -20 4 49.1225 49.0198 1

2 -20 -4-21.7277 78.8806 1

-2 20 4 67.0324 53.3669 9

2 20 -4 94.1490 62.1764 10

2 20 -4 49.6724 60.2370 7

2 -20 -3 2985.75 302.761 3

2 -20 -3 2643.38 322.550 1

-2 -20 3 2486.85 255.873 1

-2 20 3 3001.73 292.518 9

2 20 -3 3174.55 292.169 7

2 -20 -2 693.323 158.535 1

2 -20 -2 870.596 166.522 3

2 20 -2 712.927 140.272 1

-2 -20 2 732.392 145.205 1

2 20 -2 787.604 145.431 7

-2 20 1 2931.53 288.654 1

-2 -20 1 3104.18 310.359 1

2 20 -1 2241.49 264.564 1

2 -20 -1 3120.31 330.116 1

2 20 -1 2107.81 250.980 7

2 -20 0 1474.00 222.889 1

-2 -20 0 1097.18 202.762 1

2 20 0 1278.93 198.852 1

-2 20 0 1323.70 183.477 1

2 20 0 1526.19 205.635 7

-2 -20 -1 699.755 160.128 1

-2 20 -1 619.479 123.396 1

2 -20 1 660.413 134.121 1

2 -20 2 1349.96 193.558 1

-2 20 -2 1380.31 164.890 1

-2 -20 -2 1537.99 242.939 1

2 20 2 1332.83 177.811 9

-2 20 -2 1415.56 179.471 7

-2 -20 -3 2688.22 274.807 3

-2 20 -3 2533.21 214.067 1

-2 -20 -3 2384.90 309.245 1

2 20 3 2677.90 244.709 9

-2 20 -3 2549.69 226.809 7

-2 -20 -4-45.9758 53.7955 3

-2 -20 -4 52.8276 91.4317 1

2 20 4 109.479 64.7164 9

-2 20 -4 40.2130 37.6176 7

-2 20 -5 440.177 130.793 10

2 20 5 382.275 86.0473 9

-2 -20 -5 395.615 121.482 1

-2 -20 -5 336.518 112.251 3

-2 -20 -6 408.879 111.456 3

-2 20 -6 255.717 108.171 10

-2 -20 -6 606.702 171.316 1

-2 -20 -7 97.2279 53.6669 3

-2 20 -7 115.756 73.2530 10

-2 -20 -7 18.0484 77.9717 1

-2 -20 -8 1.70952 33.7239 3

-2 -20 -8-89.2842 100.590 1

-2 -20 -9-8.23584 35.0816 3

-2 20 -9 36.2479 46.0771 10

-2 -20 -9-29.8263 68.5819 1

-2 -20 -10 3.15131 42.7367 3

-2 20 -10 89.9969 97.4587 10

-2 -20 -10-81.9537 89.4655 1

-2 -20 -11 132.833 78.4552 3

-2 20 -11-50.9185 62.4138 10

2 -21 -12-55.8821 55.7318 3

2 -21 -11-72.0743 82.0941 3

2 21 -11-77.6457 99.3694 10

2 -21 -10 32.0999 42.1667 3

2 21 -10-60.0755 76.5585 10

2 -21 -10 1.47367 112.195 1

2 -21 -9-15.6507 44.8312 3

2 21 -9 73.8561 102.314 10

2 -21 -9-153.170 123.207 1

2 -21 -8 100.374 75.3508 3

2 21 -8 35.1629 53.7502 10

2 -21 -8 112.236 91.0326 1

2 21 -7 44.9991 40.0224 10

-2 -21 7-15.1591 36.7461 1

2 -21 -7-63.2514 92.0488 1

2 -21 -6-5.10588 42.8021 3

-2 21 6 22.6869 54.6702 9

2 21 -6 22.4228 54.0350 10

2 -21 -6-68.9577 85.8523 1

-2 21 6-37.3679 32.3026 2

-2 -21 6-28.1553 40.3367 1

2 -21 -5 1099.58 186.520 3

-2 -21 5 978.621 156.603 1

2 -21 -5 980.245 220.064 1

-2 21 5 818.408 144.159 2

2 21 -5 1061.27 205.768 10

2 -21 -4 707.899 154.673 3

-2 21 4 538.495 126.797 2

2 -21 -4 816.658 194.256 1

-2 -21 4 642.482 141.868 1

2 21 -4 581.484 147.194 10

2 21 -4 622.916 129.385 7

-2 -21 3 1585.51 212.293 1

-2 21 3 1590.91 228.532 2

2 -21 -3 1416.23 249.638 1

2 -21 -3 1712.57 234.471 3

2 21 -3 1741.16 223.176 7

-2 -21 2 298.681 105.521 1

2 -21 -2 324.725 99.4394 3

2 21 -2 319.027 102.130 1

2 -21 -2 292.986 109.213 1

2 21 -2 313.168 90.4194 7

-2 -21 1 586.959 137.302 1

2 -21 -1 545.800 147.678 1

-2 21 1 530.709 130.535 1

2 21 -1 305.880 101.331 1

2 21 -1 164.644 75.3506 7

2 -21 0 127.190 79.8118 1

-2 21 0 136.224 56.1184 1

-2 -21 0 150.996 80.9304 1

2 21 0 89.5982 57.9385 7

-2 -21 -1 710.363 169.187 1

2 -21 1 608.301 142.826 1

-2 21 -1 411.837 100.309 1

-2 21 -2 1531.43 184.354 1

2 -21 2 1634.81 210.723 1

-2 -21 -2 1948.18 280.035 1

2 21 2 1439.65 193.645 9

-2 21 -2 1473.46 187.152 7

-2 -21 -3 501.461 158.030 1

-2 21 -3 432.333 95.2764 1

2 21 3 408.374 102.128 9

-2 21 -3 439.900 104.767 7

-2 -21 -4 286.228 136.326 1

-2 -21 -4 111.223 69.0147 3

2 21 4 149.865 68.2038 9

-2 21 -4 132.380 56.3372 7

-2 21 -5 152.751 103.045 10

2 21 5 108.879 49.7848 9

-2 -21 -5 208.610 69.3686 3

-2 -21 -5 145.296 97.7782 1

-2 21 -6 12.9890 45.2448 10

-2 -21 -6 21.3566 58.5178 1

-2 -21 -6 19.6533 69.6771 3

-2 -21 -7 13.7937 42.8694 3

-2 21 -7-59.4868 91.1001 10

-2 -21 -7 4.25008 91.7519 1

-2 -21 -8-22.8457 71.4193 3

-2 21 -8 199.339 93.7911 10

-2 -21 -8 21.0881 80.7613 1

-2 -21 -9 21.3418 35.8515 3

-2 21 -9 86.2588 75.0974 10

-2 -21 -10 117.696 74.3893 3

-2 21 -10 107.739 76.3206 10

2 -22 -12-105.417 107.724 3

2 -22 -11 197.806 102.974 3

2 -22 -10-129.069 81.0724 3

2 22 -10 6.93456 47.1229 10

2 -22 -10 117.119 134.540 1

2 -22 -9-43.4875 72.4229 3

2 22 -9 142.764 107.343 10

2 -22 -9 80.3865 68.4823 1

2 -22 -8 6.75349 38.5490 3

2 22 -8 60.4068 73.7249 10

2 -22 -8 179.127 126.777 1

2 -22 -7 230.658 93.4334 3

2 22 -7 303.833 128.301 10

2 -22 -7 18.5215 79.8061 1

2 -22 -6-31.1531 52.7171 3

2 22 -6 60.6660 71.1205 10

-2 -22 6 29.4864 33.6725 1

2 -22 -5 402.782 114.387 3

2 -22 -5 332.529 129.973 1

-2 -22 5 433.574 106.426 1

-2 22 5 392.690 103.481 2

2 22 -5 416.293 137.521 10

-2 22 4 197.794 74.3981 2

2 -22 -4 318.820 107.173 3

2 -22 -4 248.981 123.482 1

-2 -22 4 155.644 67.5061 1

2 22 -4 281.501 103.647 10

2 22 -4 247.393 91.2840 7

2 -22 -3 1771.65 287.202 1

-2 22 3 1589.22 243.099 2

-2 -22 3 1850.51 237.071 1

2 -22 -3 1864.51 253.314 3

2 22 -3 1937.53 245.924 7

-2 -22 2 587.313 141.280 1

2 -22 -2 472.508 133.803 3

2 -22 -2 730.633 181.258 1

2 22 -2 441.110 116.132 1

2 22 -2 499.011 128.958 7

-2 -22 1 144.383 81.4660 1

2 -22 -1 27.3707 65.6711 1

2 22 -1 101.483 75.9030 7

-2 -22 0 614.384 154.497 1

-2 22 0 553.019 122.789 1

2 -22 0 558.419 140.385 1

2 22 0 717.597 158.490 7

-2 22 -1 193.388 71.7287 1

2 -22 1 311.409 112.565 1

-2 -22 -1 154.155 86.0376 1

-2 22 -1 280.552 89.8357 7

-2 22 -2 453.458 100.600 1

2 -22 2 484.253 125.180 1

-2 -22 -2 416.600 141.975 1

2 22 2 489.644 109.091 9

-2 22 -2 723.860 135.807 7

-2 -22 -3 887.997 210.199 1

-2 22 -3 745.967 122.113 1

-2 22 -3 804.324 136.375 7

2 22 3 762.736 144.568 9

-2 -22 -4 504.770 133.230 3

-2 -22 -4 467.784 163.421 1

2 22 4 369.986 96.1577 9

-2 22 -4 366.748 97.5467 7

-2 22 -5-1.31005 55.7665 10

-2 -22 -5 24.4438 61.3344 1

-2 -22 -5-30.7992 48.8792 3

-2 22 -6 93.5664 73.3496 10

-2 -22 -6 122.134 88.8936 1

-2 -22 -6 12.6695 51.9567 3

-2 -22 -7 142.602 81.9843 3

-2 22 -7 139.596 94.1126 10

-2 -22 -7 292.854 162.321 1

-2 -22 -8 40.6394 38.1261 3

-2 22 -8-62.0163 89.8107 10

-2 -22 -8-25.3463 100.545 1

-2 -22 -9 72.5417 67.2994 3

-2 22 -9 119.214 94.1337 10

-2 -22 -9 177.357 139.694 1

-2 -22 -10-48.5508 52.2822 3

-2 22 -10 20.8874 93.4525 10

2 -23 -11-212.470 114.344 3

2 -23 -10-217.720 107.575 3

2 23 -10 2.50416 61.3141 10

2 -23 -9-125.818 94.8967 3

2 23 -9-2.60069 59.8481 10

2 -23 -9-21.3448 99.6567 1

2 -23 -8-3.00600 46.5272 3

2 23 -8 51.0193 86.1379 10

2 -23 -8 109.517 105.148 1

2 -23 -7 152.456 92.5658 3

2 23 -7 198.106 110.760 10

2 -23 -7 145.511 117.687 1

2 -23 -6 115.130 67.0970 3

2 23 -6-25.7559 51.6485 10

2 -23 -6 229.930 133.344 1

-2 -23 6 99.5578 60.0577 1

2 -23 -5-57.8999 67.5533 3

-2 23 5-40.8751 44.6636 2

-2 -23 5 17.6448 30.9470 1

2 -23 -5-28.6371 61.1358 1

2 23 -5 27.8733 46.5885 10

-2 -23 4 246.175 91.1575 1

2 -23 -4 237.327 88.8491 3

2 -23 -4 284.061 132.848 1

-2 23 4 271.975 97.6548 2

2 23 -4 337.917 127.212 10

2 23 -4 356.066 108.391 7

2 -23 -3-51.6618 74.5227 3

2 -23 -3 60.9389 63.2474 1

-2 -23 3 2.52456 55.1625 1

2 23 -3 143.671 75.6771 7

2 -23 -2 316.707 128.940 1

-2 -23 2 194.501 51.7488 1

2 23 -2 378.101 110.179 7

-2 -23 1 4.57019 64.8481 1

2 -23 -1 19.0121 47.8840 1

2 23 -1 77.9899 59.0414 7

-2 -23 0-45.8402 74.3709 1

2 -23 0 10.9514 37.8590 1

2 23 0 23.7513 64.6452 7

-2 23 -1 287.344 109.772 1

-2 -23 -1 209.739 122.917 1

2 -23 1 143.417 54.8927 1

-2 23 -1 96.7849 60.4893 7

2 23 1 20.8140 66.2870 7

2 -23 2 525.753 134.433 1

-2 -23 -2 632.891 187.754 1

-2 23 -2 484.355 109.389 1

-2 23 -2 335.864 97.2306 7

2 23 2 403.699 125.364 9

-2 23 -3 32.2944 43.2403 7

2 23 3 0.37009 27.5811 9

-2 -23 -4 288.358 125.010 1

-2 -23 -4 195.230 72.2238 3

-2 23 -4 261.555 83.7655 7

-2 23 -5-85.3408 100.150 10

-2 -23 -5-35.5573 46.2967 3

-2 23 -6-31.6458 53.0166 10

-2 -23 -6 0.15498 44.2670 3

-2 23 -7 55.3418 43.0459 10

-2 -23 -7 82.3446 67.6668 3

-2 -23 -7 193.647 138.165 1

-2 -23 -8 30.0001 57.9717 3

-2 23 -8-93.5058 68.9640 10

-2 -23 -8-62.3494 98.8903 1

-2 -23 -9-10.5181 48.2846 3

-2 23 -9 54.0610 61.2720 10

-2 -23 -10 85.5735 74.6584 3

2 -24 -10-0.20048 47.5993 3

2 -24 -9 63.1944 76.8673 3

2 24 -9 47.0722 87.6617 10

2 -24 -9 72.1236 119.422 1

2 -24 -8 48.5339 46.5397 3

2 24 -8 102.706 65.4169 10

2 -24 -8 90.8201 128.303 1

2 -24 -7-91.7544 84.4105 3

2 24 -7 3.74912 60.3610 10

2 -24 -7-34.1864 70.1935 1

2 -24 -6 198.800 86.0727 3

-2 -24 6 30.2124 42.3082 1

2 -24 -6 103.905 95.2879 1

2 24 -6 126.586 94.4355 10

2 -24 -5-62.4343 72.2219 1

2 -24 -5 55.0901 52.9176 3

-2 -24 5-61.9875 68.4949 1

2 24 -5-21.4339 55.3926 10

2 -24 -4 25.6440 40.3517 3

-2 -24 4 91.2373 64.6928 1

2 -24 -4-50.8241 78.0612 1

2 24 -4-64.4381 76.9542 10

2 24 -4 53.2606 35.7136 7

2 -24 -3 213.785 96.4138 3

2 -24 -3 325.663 121.062 1

-2 -24 3 228.089 99.1729 1

2 24 -3 415.464 127.000 7

-2 -24 2 83.9593 51.7565 1

2 -24 -2-83.8714 89.2834 1

2 24 -2 19.6886 43.2081 7

2 -24 -1 85.1231 45.6177 1

-2 -24 1 43.5073 70.0288 1

2 24 -1 162.265 71.0634 7

2 -24 0 861.188 192.081 1

-2 -24 0 917.664 197.301 1

2 24 0 985.942 194.017 7

-2 24 -1 51.6519 37.1318 1

2 -24 1 136.913 67.7576 1

-2 -24 -1 255.624 116.983 1

2 24 1 146.190 72.5312 7

-2 24 -1 203.049 86.9815 7

2 -24 2 718.276 155.499 1

-2 -24 -2 476.994 151.268 1

-2 24 -2 358.379 96.4444 1

-2 24 -2 654.218 139.138 7

-2 -24 -3 472.879 168.989 1

-2 24 -3 265.625 87.7428 7

-2 -24 -4 150.354 100.911 1

-2 24 -4 21.6059 29.5283 7

-2 24 -5-28.3919 61.7353 10

-2 -24 -5 47.1766 59.5823 1

-2 -24 -5 8.55072 38.9031 3

-2 24 -6 19.8916 60.2106 10

-2 -24 -6-32.4760 72.0320 1

-2 -24 -6-29.8177 67.1686 3

-2 24 -7 213.070 74.9616 10

-2 -24 -7 104.511 73.4776 3

-2 24 -8 85.4903 65.3709 10

-2 -24 -8 114.437 69.9971 3

-2 -24 -9 47.1650 64.5687 3

-2 -24 -10-221.385 100.974 3

2 -25 -10-23.6531 48.0670 3

2 -25 -9 6.56427 46.4595 3

2 -25 -8 101.085 73.9951 3

2 25 -8-81.9138 80.7308 10

2 -25 -8 0.93396 63.4829 1

2 -25 -7 77.4844 65.7496 3

2 -25 -7 149.688 122.504 1

2 25 -7 47.7948 68.1611 10

2 -25 -6-55.1078 63.8306 1

2 -25 -6 10.1612 44.3410 3

2 25 -6 105.510 100.601 10

-2 -25 5 60.9714 33.2086 1

2 -25 -5 1.61363 90.5820 1

2 -25 -5 185.278 98.1052 3

2 25 -5 13.9415 45.2735 10

-2 -25 4-15.5335 45.2886 1

2 -25 -4 82.4274 68.3180 3

2 -25 -4 24.6884 55.9874 1

2 25 -4 62.5177 70.4593 10

2 25 -4-99.6338 85.7796 7

2 -25 -3 1.45107 49.8375 1

-2 -25 3 3.83506 56.2081 1

2 -25 -3 212.535 108.153 3

2 25 -3 159.649 92.0192 10

2 25 -3 112.917 73.0734 7

2 -25 -2 407.572 146.275 1

-2 -25 2 254.012 111.126 1

2 25 -2 98.1275 58.6418 7

-2 -25 1 170.819 84.8451 1

2 -25 -1 75.9335 68.9120 1

2 25 -1-9.35024 49.2411 7

2 -25 0 7.07577 47.0685 1

-2 -25 0 96.0573 59.8611 1

2 25 0 11.6166 59.7526 7

-2 -25 -1 165.395 100.636 1

2 -25 1 136.565 96.9773 1

2 25 1 186.741 102.234 7

-2 25 -1 95.4762 84.2294 7

2 -25 2 130.815 86.7457 1

-2 -25 -2 101.972 93.6526 1

-2 25 -2 227.083 96.3548 7

-2 -25 -3 37.5620 52.7053 1

-2 25 -3-69.7991 63.4312 7

-2 -25 -4 218.131 123.039 1

-2 25 -4 61.4285 42.3269 7

-2 25 -5-63.0972 41.2563 10

-2 -25 -5 115.667 78.2170 1

-2 25 -6 34.4836 56.8361 10

-2 -25 -6-77.7780 51.6214 3

-2 -25 -6 42.1763 75.7827 1

-2 25 -7 75.0037 66.3370 10

-2 -25 -7 25.1275 87.3272 1

-2 -25 -7-45.8577 50.6881 3

-2 -25 -8-74.0057 70.4387 3

-2 -25 -9 28.5662 43.8770 3

2 -26 -9-114.971 90.7058 3

2 -26 -8 157.126 80.0961 3

2 -26 -7-51.4946 81.0715 1

2 -26 -7-62.8373 63.9283 3

2 26 -7-47.1362 77.8382 10

2 -26 -6 81.3855 86.0473 3

2 -26 -6 99.8573 78.7473 1

2 26 -6-13.8769 71.7015 10

2 -26 -5 62.5142 78.4984 3

2 -26 -5 170.995 106.141 1

2 26 -5 89.5269 96.8913 10

-2 -26 4-25.0475 35.7402 1

2 -26 -4-43.5061 66.7613 3

2 26 -4 15.0184 42.7690 10

-2 -26 3 83.0524 76.4958 1

2 -26 -3 92.7237 82.5750 1

2 26 -3 43.3221 69.6049 10

2 26 -3-41.7577 42.2903 7

2 -26 -2 0.23883 61.4927 1

-2 -26 2 1.52281 54.5858 1

2 26 -2 41.1574 51.2045 7

-2 -26 1 184.564 117.471 1

2 -26 -1 114.539 83.9762 1

2 26 -1 149.573 100.199 7

2 -26 0 428.015 145.657 1

-2 -26 0 472.242 154.103 1

-2 26 0 248.820 105.745 7

2 26 0 310.136 131.228 7

-2 -26 -1-2.02084 51.4195 1

2 -26 1 49.6115 52.6390 1

2 26 1 39.1162 61.8957 7

-2 26 -1-38.7661 39.9765 7

-2 -26 -2 102.254 99.0155 1

2 26 2 157.370 104.691 7

-2 26 -2 56.0054 55.9787 7

-2 -26 -3 197.442 116.260 1

-2 26 -3 60.8098 53.1059 7

-2 -26 -4-68.8855 131.115 1

-2 26 -4 11.5820 54.7376 10

-2 26 -4 65.9033 55.7639 7

-2 26 -5 36.5715 55.8218 10

-2 -26 -5-57.2296 74.8808 1

-2 26 -6 23.8154 61.4172 10

-2 -26 -6-119.107 81.2156 3

-2 -26 -6-93.9930 108.146 1

-2 -26 -7 12.5338 49.8849 3

-2 -26 -8-105.983 86.6732 3

-2 -26 -9 85.7984 75.0179 3

2 -27 -8 44.2557 74.5212 3

2 -27 -7-158.462 101.525 1

2 -27 -7 32.0055 84.7002 3

2 -27 -6-27.5074 76.0262 3

2 -27 -6-37.0325 101.609 1

2 27 -6-34.8796 51.2392 10

2 -27 -5 109.548 99.9376 3

2 -27 -5 98.8846 123.100 1

2 27 -5-52.8317 87.2462 10

-2 -27 4 114.616 106.172 1

2 -27 -4 99.2706 67.7020 3

2 -27 -4 364.346 177.528 1

2 27 -4 410.734 162.669 10

-2 -27 3 79.0518 89.4562 1

2 -27 -3 39.8193 69.7418 1

2 27 -3 188.009 108.789 10

2 27 -3 285.582 113.656 7

-2 -27 2 159.301 94.6810 1

2 -27 -2 173.622 125.666 1

2 27 -2 111.767 82.2968 7

-2 -27 1-56.9960 68.6845 1

2 -27 -1-190.982 130.501 1

2 27 -1-9.68969 45.7399 7

-2 -27 0 11.0586 51.3292 1

2 -27 0-115.909 87.7795 1

-2 27 0-10.5265 39.8050 7

2 27 0-25.6563 55.7890 7

2 -27 1-119.741 83.2636 1

-2 -27 -1-108.426 90.3480 1

2 27 1-49.3044 93.6262 7

-2 27 -1-1.30348 48.2599 7

-2 -27 -2 49.0153 53.5537 1

-2 27 -2-74.3044 60.2981 7

2 27 2-14.3564 43.8650 7

-2 -27 -3 45.2200 52.6153 1

-2 27 -3 33.0208 54.0829 7

-2 -27 -4 192.196 144.471 1

-2 27 -4-194.270 114.946 10

-2 -27 -5 305.677 157.344 1

-2 27 -5-159.655 112.149 10

-2 -27 -7-69.3188 52.8323 3

-2 -27 -8-91.1292 72.5512 3

2 -28 -6-93.8108 101.053 1

2 -28 -6 74.6021 79.6098 3

2 -28 -5 95.3514 83.8554 1

2 -28 -5 56.7535 72.3291 3

2 28 -5 133.201 75.6420 10

2 -28 -4 74.2430 112.733 1

2 28 -4 108.885 83.5850 10

-2 -28 3 140.340 106.572 1

2 -28 -3 64.5634 67.1842 1

2 28 -3-30.1393 102.875 10

2 28 -3-39.6918 71.0048 7

-2 -28 2-24.1799 53.9777 1

2 -28 -2 0.26170 55.6191 1

2 28 -2 5.45972 46.0482 7

-2 -28 1 25.1577 76.3328 1

2 -28 -1 165.380 123.201 1

2 28 -1 249.775 112.557 7

-2 -28 0-30.8767 86.6658 1

2 -28 0-29.3753 108.946 1

-2 28 0 74.0780 67.5400 7

2 28 0-83.3978 77.2006 7

-2 -28 -1-20.5582 73.5854 1

2 28 1 48.3486 93.1646 7

-2 28 -1-69.2757 57.4456 7

-2 28 -2-59.9658 61.5550 7

2 28 2 66.5588 68.0902 7

-2 -28 -3-84.0727 98.5974 1

-2 28 -3 36.9687 44.2813 7

2 28 3-38.2023 82.8489 7

-2 -28 -4-147.006 132.016 1

-2 28 -4-75.8561 103.540 10

2 -29 -5-55.0953 103.646 1

2 -29 -4-165.751 137.608 1

2 -29 -3-132.997 132.236 1

-2 -29 3 128.744 82.0356 1

2 29 -3 178.913 119.330 10

2 29 -3 207.675 97.9689 7

-2 -29 2-147.979 123.729 1

2 -29 -2 118.255 93.1744 1

2 29 -2 57.5410 65.0741 7

-2 -29 1 91.6150 87.9113 1

2 -29 -1-29.2998 55.1246 1

-2 29 1-137.124 90.4174 7

2 29 -1-88.8957 83.5263 7

-2 -29 0-66.0102 87.8401 1

2 -29 0-22.0390 89.2103 1

-2 29 0 30.3282 57.0114 7

2 29 0 48.7359 66.5368 7

-2 -29 -1-63.1219 73.3461 1

2 29 1 99.1222 87.4779 7

-2 29 -1-0.17994 70.2454 7

-2 -29 -2-166.978 117.119 1

2 29 2-45.7778 66.6266 7

-2 29 -2 90.2691 82.5124 7

-2 -29 -3 140.780 104.605 1

2 29 3-112.897 117.346 7

-2 -29 -4-77.8936 84.9605 1

2 -30 -4 98.9036 106.296 1

2 -30 -3 227.502 150.876 1

2 30 -3-211.271 118.263 7

-2 -30 2-56.4995 74.8190 1

2 -30 -2-52.0671 67.6369 1

2 30 -2 29.2027 77.7523 7

2 -30 -1 85.8680 109.396 1

-2 -30 1-48.8840 78.1216 1

-2 30 1 90.7296 79.1351 7

2 30 -1-31.9163 57.0917 7

-2 -30 0 41.8700 85.0765 1

-2 30 0 79.6868 95.6278 7

-2 -30 -1-149.233 113.554 1

2 30 1-87.6386 82.7026 7

-2 30 -1 18.5506 46.9745 7

-2 -30 -2-152.322 141.714 1

-2 30 -2 0.18008 50.5589 7

-2 -30 -3-64.5348 127.871 1

2 -31 -3 87.3763 122.606 1

2 31 -3-104.930 104.161 7

2 31 -2 0.22680 68.3118 7

-2 31 2 32.8607 64.1165 7

-2 -31 1 19.7378 61.9638 1

2 31 -1-132.789 93.2853 7

-2 31 1 0.21726 76.4861 7

-2 -31 0-185.309 116.221 1

-2 31 0-42.7616 60.4131 7

2 31 0-119.490 84.4522 7

-2 -31 -1-150.058 113.081 1

-2 31 -1-42.8656 79.1565 7

-2 -31 -2 218.033 179.246 1

2 32 -3 85.1378 93.3443 7

2 32 -2-225.575 116.703 7

-2 -32 1 21.7425 64.5665 1

-2 32 1 236.749 136.138 7

-2 -32 0-47.9145 77.1735 1

-2 32 0-34.2882 65.9827 7

-2 -32 -1 25.6267 65.7829 1

-2 32 -1-208.309 104.903 7

3 0 -17 159.098 125.207 10

3 0 -16-89.8474 93.1270 10

3 0 -15 24.5870 63.1062 10

3 0 -15 47.7345 76.5566 6

3 0 -14 373.618 133.851 10

3 0 -14 320.484 142.974 6

-3 0 13 50.8646 67.4673 1

3 0 -13 15.1019 45.6104 10

3 0 -13 83.8725 88.4603 6

-3 0 12 413.072 120.500 1

3 0 -12 743.215 178.761 10

3 0 -12 546.540 161.910 6

-3 0 11-121.976 71.1925 4

-3 0 11 17.9233 43.8341 1

3 0 -11-1.97922 42.4614 10

3 0 -11-4.51900 51.0151 6

-3 0 10 9780.88 551.548 4

-3 0 10 9284.20 514.078 1

-3 0 10 9686.09 549.484 10

3 0 -10 9733.07 547.984 10

-3 0 10 9300.36 334.421 8

3 0 -10 9530.95 572.596 6

-3 0 9 31.4296 57.9512 4

3 0 -9-78.3242 72.9223 10

-3 0 9-31.5295 41.1063 10

-3 0 9-13.4201 30.9977 8

3 0 -9-34.2401 37.6438 6

-3 0 8 22933.2 741.760 1

-3 0 8 24056.0 783.491 10

3 0 -8 22232.8 748.250 10

3 0 -8 23068.0 773.633 6

-3 0 7 5.15984 34.8682 4

-3 0 7-49.0431 46.8650 1

-3 0 7-21.3580 30.3930 10

3 0 -7 20.5438 33.2704 10

-3 0 6 2232.66 216.066 4

-3 0 6 2330.96 206.210 1

-3 0 6 2130.63 201.404 10

3 0 -6 2063.45 195.011 6

-3 0 5 10.4590 28.2290 10

3 0 -5-10.3500 23.2100 6

-3 0 4 205.340 53.9130 10

3 0 -4 213.836 46.9083 6

-3 0 3 13.8202 13.4018 10

3 0 -3 8.49261 14.3855 6

3 0 -2 997275. 2760.36 10

-3 0 2 992940. 2770.41 10

3 0 -2 999999. 1915.70 6

-3 0 1 4.68046 10.0431 10

3 0 -1-2.65771 13.0110 10

3 0 0 41.5962 16.8881 9

-3 0 0 27.8072 9.12793 9

-3 0 -1-22.1710 26.4386 1

3 0 1-0.79199 10.2703 7

-3 0 -1 26.6202 17.9802 9

-3 0 -2 56271.1 647.510 1

3 0 2 56064.6 701.472 2

-3 0 -2 54231.4 433.363 10

-3 0 -2 57003.7 790.842 6

-3 0 -3 21.6166 20.9249 1

3 0 3 11.5501 17.2653 2

3 0 3 43.5290 21.2366 1

3 0 3 7.66564 11.5115 10

-3 0 -3 5.24858 9.95289 10

-3 0 -4 19581.3 451.564 1

3 0 4 18104.7 446.619 1

-3 0 -4 19127.1 453.744 2

-3 0 -4 18311.9 396.128 10

3 0 4 18643.6 382.836 10

-3 0 -4 19358.9 600.573 6

-3 0 -5 31.7202 25.8274 4

-3 0 -5 10.0296 18.3197 1

-3 0 -5 0.91775 17.6394 2

3 0 5 33.3455 34.4002 1

-3 0 -5 17.0442 36.0905 10

3 0 5 16.4802 14.2510 10

-3 0 -5-24.1419 35.3935 6

-3 0 -6 18048.1 509.298 4

3 0 6 16624.9 507.063 1

-3 0 -6 17883.9 502.366 2

-3 0 -6 19016.2 547.028 1

3 0 6 17623.9 484.007 10

-3 0 -6 20824.1 555.259 10

-3 0 -6 21645.2 775.229 6

-3 0 -6 20635.8 584.576 5

-3 0 -7-14.0483 38.4570 4

-3 0 -7-3.85116 29.4851 1

3 0 7-40.2074 44.6082 1

-3 0 -7-8.43939 16.3481 2

3 0 7-22.5528 24.1580 10

-3 0 -7-54.1367 43.5998 10

-3 0 -7-99.5775 82.0485 6

-3 0 -7-16.7171 43.3298 5

3 0 8 2255.72 209.442 1

-3 0 -8 2247.17 225.810 1

3 0 8 2445.49 211.142 10

-3 0 -8 2377.74 220.401 10

-3 0 -8 2226.57 205.058 5

-3 0 -8 2554.12 305.374 6

-3 0 -9-16.5233 29.1730 5

-3 0 -9 35.1604 57.6276 1

3 0 9 41.7425 48.7580 10

-3 0 -9 2.71773 34.9651 10

-3 0 -9-6.35328 64.6283 6

3 0 10 1240.89 179.865 1

-3 0 -10 1516.41 200.292 1

-3 0 -10 1749.71 216.810 10

3 0 10 1543.87 195.629 10

-3 0 -10 1684.44 283.926 6

-3 0 -10 1554.09 174.013 5

-3 0 -11 15.5538 25.7859 5

3 0 11-29.7116 51.9955 1

3 0 11 105.462 54.4849 10

-3 0 -11 14.3599 31.7380 10

-3 0 -11-5.48415 69.3703 6

-3 0 -12-7.95893 34.2191 10

-3 0 -12-70.5211 81.1317 6

-3 0 -13-50.4882 55.0218 10

-3 0 -13-32.8034 98.5209 6

3 1 -17 5.27974 69.4047 10

3 -1 -17-32.7013 63.8041 10

3 -1 -16-91.6248 88.8745 10

3 1 -16-85.5994 83.5672 10

3 -1 -15-22.6026 42.4932 10

3 1 -15-81.8065 74.9914 10

3 -1 -15-44.3696 98.9785 6

3 1 -15 22.7898 60.9163 6

3 1 -14 1252.18 251.387 10

3 -1 -14 1342.02 241.970 10

3 -1 -14 1335.03 266.163 6

3 1 -14 1114.59 262.430 6

-3 -1 13 2.81875 44.6704 1

3 1 -13 17.1678 48.6906 10

3 -1 -13 55.0415 58.4889 10

3 -1 -13 41.5722 59.7190 6

3 1 -13-74.9215 43.3634 6

-3 -1 12 2680.76 314.151 4

-3 -1 12 2377.41 275.677 1

-3 1 12 2168.30 272.100 1

3 -1 -12 2550.74 314.839 10

3 1 -12 2594.79 313.366 10

3 1 -12 2572.99 340.412 6

3 -1 -12 2455.54 330.128 6

-3 -1 11 531.630 147.844 4

-3 1 11 559.376 142.825 4

-3 1 11 353.717 111.757 1

-3 -1 11 318.062 102.506 1

3 1 -11 332.900 110.357 10

3 -1 -11 514.024 130.718 10

3 1 -11 512.049 151.672 6

3 -1 -11 333.322 117.577 6

-3 -1 10 86.5804 56.0202 4

-3 1 10 77.7225 63.8741 4

-3 1 10 69.0573 55.2366 1

3 1 -10 94.0522 62.9175 10

-3 -1 10 52.5026 43.2132 10

-3 1 10-5.90057 37.1147 10

3 -1 -10 20.1623 38.7710 10

-3 -1 10 73.8781 39.9202 8

3 1 -10 77.7887 72.5927 6

-3 1 10 83.2843 41.9803 8

3 -1 -10 64.6442 53.6916 6

-3 1 9-60.9690 59.8827 4

-3 -1 9 64.5119 54.5890 10

3 1 -9-19.1497 47.0366 10

-3 1 9 40.4037 32.9234 10

3 -1 -9 65.1468 54.2595 10

3 -1 -9 3.98590 56.7684 6

-3 -1 9 29.4560 27.4665 8

-3 1 9 9.19514 22.6068 8

3 1 -9-2.55494 46.0223 6

-3 -1 8 5555.90 358.324 1

-3 1 8 6130.79 394.260 10

3 -1 -8 5932.98 379.237 10

3 1 -8 6006.26 391.663 10

-3 -1 8 6057.73 387.815 10

3 1 -8 5552.11 381.129 6

3 -1 -8 5922.28 388.300 6

-3 1 8 5877.64 287.272 8

-3 -1 7 672.479 123.592 4

-3 1 7 644.321 117.829 1

-3 -1 7 782.889 124.049 1

3 -1 -7 776.179 131.495 10

-3 -1 7 761.400 127.159 10

-3 1 7 791.089 131.906 10

-3 1 6 4536.75 303.174 4

-3 1 6 4418.25 293.410 1

-3 -1 6 4673.23 298.577 10

-3 1 6 4787.28 302.643 10

3 1 -6 4695.00 292.505 6

3 -1 -6 4334.67 279.749 6

-3 1 5 112.029 63.3571 4

-3 -1 5 134.296 69.0725 10

-3 1 5 146.707 67.5263 10

3 -1 -5 80.5826 47.6676 6

3 1 -5 160.026 63.4438 6

-3 1 4 64.1893 24.8977 10

-3 -1 4 61.1343 32.6134 10

-3 1 4 24.8285 20.0953 6

3 -1 -4 10.9552 24.4650 6

-3 1 4 43.4117 12.4926 5

-3 -1 3 3529.57 192.158 10

-3 1 3 3597.99 198.115 10

3 -1 -3 3593.40 163.135 6

-3 1 3 3504.57 164.221 6

-3 1 2 13267.4 327.037 10

3 1 -2 13530.2 320.949 10

-3 -1 2 13704.4 326.529 10

3 -1 -2 13658.6 321.960 10

3 -1 -2 13032.7 233.657 6

-3 1 2 13848.4 246.126 6

-3 1 1 1132.29 79.0802 10

-3 -1 1 1160.79 76.6446 10

3 -1 -1 1145.68 77.4311 10

3 1 -1 1142.62 76.2293 10

3 -1 0 71220.3 448.563 3

3 -1 0 71313.4 602.737 9

3 1 0 68381.8 523.956 9

-3 1 0 69768.2 610.099 9

-3 -1 0 68060.2 477.830 6

3 1 0 68545.9 496.626 6

-3 -1 0 68338.1 598.763 7

-3 1 -1 15420.1 207.421 3

-3 -1 -1 14349.9 337.683 1

3 -1 1 14301.2 348.348 2

-3 1 -1 15268.8 291.772 9

-3 -1 -1 14822.7 219.729 9

-3 -1 -1 15131.0 324.879 6

3 1 1 15220.2 280.294 7

-3 1 -1 15141.8 218.975 7

3 1 2 146.693 58.2685 1

-3 -1 -2 399.024 72.9005 1

-3 1 -2 427.601 54.1656 1

3 -1 2 561.682 82.2772 2

3 1 2 313.213 57.4746 2

-3 1 -2 277.400 61.9226 2

-3 1 -2 222.407 32.8627 10

3 -1 2 163.820 29.9343 7

-3 -1 -2 267.877 63.2329 6

3 -1 3 819.429 121.280 1

3 1 3 1464.42 127.968 1

-3 1 -3 1907.65 160.386 2

-3 -1 -3 4019.70 210.491 1

-3 1 -3 3018.56 174.594 1

3 1 3 2902.82 176.129 2

-3 -1 -3 990.233 78.7982 10

-3 1 -3 1063.12 90.9555 10

-3 -1 -3 821.293 118.541 6

-3 1 -4 23034.9 426.137 4

-3 1 -4 23186.0 461.837 1

3 1 4 22310.1 525.957 1

-3 1 -4 23017.9 529.660 2

-3 -1 -4 22939.8 513.426 1

3 -1 4 21578.1 455.153 1

-3 -1 -4 22160.1 421.917 10

3 -1 4 23373.7 435.952 10

-3 1 -4 21979.5 456.885 10

3 1 4 22446.3 420.647 10

-3 -1 -4 22705.9 644.095 6

-3 -1 -5 754.925 100.411 4

-3 1 -5 742.430 86.7379 4

-3 -1 -5 725.098 101.943 1

-3 1 -5 670.769 85.4198 1

3 1 5 798.072 108.643 1

-3 1 -5 682.455 95.9034 2

3 -1 5 806.370 97.4612 1

-3 1 -5 736.670 93.4932 10

3 1 5 915.717 99.0655 10

-3 -1 -5 916.765 100.012 10

3 -1 5 815.033 92.6838 10

-3 1 -5 852.954 137.675 6

-3 -1 -5 782.608 131.345 6

-3 1 -5 714.798 104.648 5

-3 1 -6 319.816 65.6642 4

-3 -1 -6 363.956 76.5554 4

3 -1 6 294.111 60.4927 1

-3 1 -6 328.343 63.2133 1

3 1 6 265.895 70.8728 1

-3 -1 -6 262.816 61.7255 1

-3 1 -6 322.179 74.2393 2

-3 1 -6 322.509 72.0419 10

3 1 6 375.265 73.0986 10

-3 -1 -6 376.799 77.6608 10

3 -1 6 367.124 68.2513 10

-3 1 -6 381.954 97.8732 6

-3 1 -6 290.936 75.7136 5

-3 -1 -6 246.612 80.8040 6

-3 -1 -7 2410.08 211.249 4

3 -1 7 2351.02 191.319 1

3 1 7 2370.30 209.623 1

-3 1 -7 2255.94 194.744 2

-3 1 -7 2389.35 197.487 1

-3 -1 -7 2354.82 208.838 1

3 1 7 2603.72 204.579 10

-3 1 -7 2526.92 211.421 10

-3 -1 -7 2608.00 206.951 10

3 -1 7 2749.37 207.026 10

-3 1 -7 2580.41 212.848 5

-3 1 -7 2863.22 299.799 6

-3 -1 -7 2155.78 118.894 8

-3 -1 -7 2529.21 276.165 6

-3 -1 -8 1820.03 199.617 4

-3 1 -8 2113.77 200.841 2

-3 -1 -8 2104.05 223.975 1

-3 1 -8 2255.85 232.748 1

3 -1 8 2144.65 198.820 1

3 1 8 2143.42 201.623 10

3 -1 8 2116.60 200.194 10

-3 -1 -8 2164.95 204.617 10

-3 1 -8 2039.39 208.758 10

-3 -1 -8 2295.77 205.456 5

-3 -1 -8 2416.15 290.535 6

-3 1 -8 2325.61 290.014 6

-3 1 -8 2221.90 205.690 5

-3 1 -9 12.3038 26.3351 5

3 -1 9 16.8921 26.4927 1

-3 1 -9 32.5053 35.8549 1

-3 1 -9 47.6595 38.2983 2

-3 -1 -9 11.1907 51.7621 1

3 -1 9 46.0238 43.7670 10

3 1 9 37.3046 32.4972 10

-3 -1 -9 12.7735 38.6823 10

-3 1 -9 63.2818 50.7941 10

-3 -1 -9-17.3189 65.6525 6

-3 -1 -9 98.7245 57.9060 5

-3 1 -9 5.30816 42.6098 6

-3 -1 -10 138.790 56.8121 5

-3 1 -10 149.937 61.1257 5

3 1 10 77.7272 36.3997 1

-3 -1 -10 81.0534 50.0202 1

-3 -1 -10 211.161 81.9915 10

-3 1 -10 125.886 63.2674 10

3 -1 10 204.416 81.4888 10

3 1 10 143.766 67.7853 10

-3 1 -10 185.772 99.0887 6

-3 -1 -10 149.589 92.8968 6

-3 1 -11 51.0750 38.1309 5

-3 -1 -11-20.5945 36.8762 5

3 1 11 152.441 71.0900 1

3 -1 11 148.493 80.6782 1

3 1 11 38.1759 37.3247 10

-3 1 -11 0.27703 30.5920 10

3 -1 11-103.230 71.6932 10

-3 -1 -11-24.2487 54.4091 10

-3 -1 -11 20.5061 49.8409 6

-3 1 -11 21.6246 83.9094 6

-3 -1 -12-40.9286 43.1742 10

-3 1 -12 45.7495 44.2714 10

-3 -1 -12 8.06436 72.5584 6

-3 1 -12-13.6775 76.8866 6

-3 -1 -13-58.4138 77.5224 10

-3 1 -13-37.6119 57.4207 10

-3 -1 -13-124.428 83.8975 6

-3 1 -13 23.1326 59.5641 6

3 -2 -17 25.6154 50.4178 10

3 2 -16 47.0240 53.4576 10

3 -2 -16 15.6784 56.4267 10

3 2 -15 100.648 91.8207 10

3 -2 -15 66.7256 70.6898 10

3 -2 -15 203.774 121.766 6

3 2 -15-59.9288 98.7292 6

3 -2 -14 45.6003 43.7208 10

3 2 -14 33.8663 57.1371 10

3 -2 -14 88.6644 89.5305 6

3 2 -14 99.1145 81.3714 6

-3 -2 13-112.575 109.492 1

3 2 -13-91.7652 81.5049 10

3 -2 -13 21.8176 61.4148 10

3 -2 -13-36.6358 65.9295 6

3 2 -13 78.9906 70.7903 6

-3 -2 12 260.391 102.698 4

-3 -2 12 330.878 87.4642 1

-3 2 12 166.399 79.2696 1

3 2 -12 269.864 110.669 10

3 -2 -12 333.560 116.493 10

3 2 -12 344.686 116.762 6

3 -2 -12 263.396 104.043 6

-3 -2 11 199.639 94.2949 4

-3 -2 11 178.479 88.4636 1

-3 2 11 53.0784 62.6212 1

3 -2 -11 210.779 99.7813 10

3 2 -11-1.10378 58.4522 10

3 2 -11 97.9140 85.9756 6

3 -2 -11 110.406 79.5042 6

-3 -2 10 1328.13 200.535 4

-3 2 10 1327.86 205.940 4

-3 2 10 1110.70 182.162 1

3 -2 -10 1552.83 222.338 10

-3 -2 10 1587.68 219.879 10

-3 2 10 1591.62 230.137 10

3 2 -10 1493.77 221.450 10

3 -2 -10 1345.03 218.597 6

3 2 -10 1291.42 219.930 6

-3 -2 10 1419.34 113.963 8

-3 2 9-1.66007 34.5676 4

-3 -2 9 22.7078 35.2668 1

-3 2 9 25.7538 39.7262 1

3 -2 -9-47.8411 42.6532 10

-3 -2 9-3.30902 46.3171 10

3 2 -9 32.4364 47.5294 10

-3 2 9 7.55916 40.4902 10

-3 2 9-2.02080 35.8643 8

3 2 -9 12.6327 54.2754 6

3 -2 -9-75.3648 72.3751 6

-3 -2 8 11065.6 485.383 1

3 -2 -8 11953.7 535.454 10

-3 2 8 11453.5 545.222 10

-3 -2 8 11269.9 522.799 10

3 2 -8 11568.4 544.501 10

3 -2 -8 11634.1 544.928 6

3 2 -8 11198.2 544.893 6

-3 2 8 10425.7 410.770 8

-3 2 7 20.5352 33.2255 1

-3 -2 7 21.5286 36.3589 1

-3 2 7 47.2925 32.3320 10

3 -2 -7 84.1261 68.9669 10

-3 -2 7 89.4734 49.6659 10

-3 2 7 78.2920 38.9759 8

-3 2 6 894.330 143.911 4

-3 -2 6 994.518 70.8747 5

-3 2 6 1012.68 154.082 1

-3 2 6 989.548 140.164 10

-3 -2 6 982.982 137.831 10

3 -2 -6 880.927 132.283 6

-3 2 5 4716.11 289.014 4

-3 2 5 4733.14 291.780 1

-3 2 5 5091.01 288.760 10

-3 -2 5 4885.03 279.958 10

-3 2 5 4889.41 275.295 6

3 -2 -5 4866.96 266.758 6

-3 2 4 8320.05 203.318 5

-3 2 4 8681.83 345.784 10

-3 -2 4 8585.44 333.151 10

-3 2 4 8445.43 318.082 6

3 -2 -4 8405.77 308.063 6

-3 -2 3 80677.4 911.737 10

-3 2 3 81500.5 948.435 10

-3 2 3 83317.1 838.692 6

3 -2 -3 82094.6 796.284 6

-3 2 3 79537.4 695.442 5

3 2 -2 2513.83 142.796 10

-3 2 2 2417.95 142.027 10

-3 -2 2 2235.63 130.597 10

-3 2 2 2473.18 134.477 5

-3 2 2 2467.08 112.108 6

-3 2 1 452.336 55.2437 9

3 2 -1 488.000 55.7551 10

-3 -2 1 556.365 53.4429 10

-3 2 0 87.1250 21.4681 3

3 -2 0 72.3980 20.0782 3

-3 -2 0 91.2058 17.0185 10

3 2 0 137.009 20.0863 10

3 2 0 75.4482 20.7619 6

-3 2 0 103.100 28.8182 9

3 2 0 82.5742 24.8881 7

-3 -2 0 84.5459 25.4897 7

3 -2 0 106.409 30.6941 9

3 -2 1 185.131 44.7296 2

-3 2 -1 169.638 41.7350 2

3 2 1 132.019 33.2084 1

-3 -2 -1 172.023 39.4740 1

-3 2 -1 207.962 33.1256 3

3 2 1 164.156 35.6795 6

-3 -2 -1 199.913 37.7940 6

-3 2 -1 174.700 43.3279 5

-3 2 -1 185.048 21.7447 7

3 2 1 206.619 36.4439 7

-3 -2 -2 41067.6 621.211 1

-3 2 -2 40299.0 652.901 2

3 -2 2 41346.5 706.155 2

-3 2 -2 38684.2 472.483 1

3 2 2 39502.9 639.182 1

-3 2 -2 39222.4 444.070 10

-3 -2 -2 36768.3 628.420 6

-3 2 -2 37246.7 663.591 5

3 2 2 36271.0 645.865 6

-3 2 -3 12539.8 227.258 4

3 2 3 12532.7 320.405 2

-3 2 -3 11851.8 380.572 2

3 2 3 11904.4 381.103 1

-3 2 -3 11995.6 267.111 1

-3 -2 -3 12214.9 363.447 1

-3 2 -3 12496.4 309.718 10

3 2 3 12726.6 265.731 10

-3 -2 -3 13087.2 255.442 10

-3 -2 -3 12568.6 429.295 6

-3 2 -3 12106.8 400.366 5

-3 2 -4 5049.22 178.997 4

3 2 4 5326.30 230.128 2

3 -2 4 4799.09 204.894 1

-3 2 -4 4973.21 260.538 2

3 2 4 5185.19 272.597 1

-3 2 -4 5407.78 203.645 1

-3 -2 -4 5523.64 264.625 1

-3 2 -4 5015.58 224.466 10

-3 -2 -4 5001.54 195.291 10

3 2 4 4722.48 197.060 10

-3 -2 -4 5531.51 319.366 6

-3 2 -4 5379.76 277.746 5

-3 2 -5 56.3662 41.8857 2

-3 2 -5 141.310 48.9692 1

-3 -2 -5 70.9610 60.9417 1

3 -2 5 21.0308 29.8670 1

3 2 5 18.2419 49.1735 1

3 2 5 27.5184 19.4394 10

-3 -2 -5 3.99851 21.3816 10

3 -2 5 32.5925 28.6939 10

-3 2 -5 53.7866 30.3391 10

-3 2 -5 62.9937 47.8570 5

-3 -2 -5 0.68277 27.6961 6

-3 -2 -6 1384.59 153.440 4

3 -2 6 1184.40 124.575 1

-3 -2 -6 1411.19 162.260 1

3 2 6 1363.03 159.120 1

-3 2 -6 1272.09 125.012 1

-3 2 -6 1161.50 143.025 2

-3 -2 -6 1319.10 132.327 10

3 2 6 1343.24 135.001 10

-3 2 -6 1378.76 146.360 10

3 -2 6 1329.36 133.129 10

-3 2 -6 1289.89 149.901 5

-3 -2 -6 1305.20 189.298 6

-3 -2 -7 138.064 59.6874 4

-3 2 -7 119.759 46.9633 5

3 -2 7 166.030 51.3372 1

-3 -2 -7 113.805 54.5611 1

-3 2 -7 142.300 51.4397 1

-3 2 -7 125.358 47.8860 2

-3 2 -7 147.417 62.3953 10

3 -2 7 118.769 47.1215 10

3 2 7 154.643 48.4057 10

-3 -2 -7 142.194 45.8495 10

-3 2 -7 265.904 109.012 6

-3 -2 -7 76.9784 27.4122 8

-3 -2 -7 120.687 63.1729 6

-3 -2 -8 97.9904 42.1076 4

-3 2 -8 167.047 57.2709 5

3 -2 8 130.429 55.4896 1

-3 2 -8 195.684 72.2363 1

-3 2 -8 133.253 45.9592 2

-3 -2 -8 201.065 84.2022 1

3 -2 8 239.842 70.7116 10

-3 -2 -8 123.191 46.6222 10

-3 2 -8 206.917 64.2099 10

3 2 8 143.070 49.6962 10

-3 2 -8 210.816 90.7031 6

-3 -2 -8 39.0137 46.1387 6

-3 -2 -9 1446.69 193.417 4

-3 2 -9 1577.76 183.038 2

3 2 9 1443.94 187.264 1

-3 -2 -9 1633.55 213.735 1

3 -2 9 1415.94 167.606 1

3 -2 9 1576.72 184.423 10

3 2 9 1405.32 174.445 10

-3 2 -9 1749.52 210.561 10

-3 -2 -9 1637.00 185.726 10

-3 2 -9 1785.39 280.033 6

-3 -2 -9 1601.21 254.158 6

-3 -2 -9 1796.82 188.835 5

-3 2 -9 1667.06 182.554 5

-3 2 -10 1272.92 158.090 5

-3 2 -10 1225.68 170.805 2

3 2 10 1429.30 203.357 1

-3 -2 -10 1366.16 202.997 1

3 -2 10 1450.38 174.645 1

-3 -2 -10 1519.33 194.300 10

3 -2 10 1325.75 177.797 10

-3 2 -10 1469.67 201.896 10

3 2 10 1430.90 190.276 10

-3 2 -10 1507.98 259.965 6

-3 -2 -10 1805.54 285.970 6

-3 -2 -10 1412.65 165.897 5

-3 2 -11 12.9845 26.7021 5

-3 -2 -11 34.9025 26.5193 5

3 2 11 139.805 75.9776 1

-3 -2 -11-51.2880 60.1008 1

3 -2 11 92.8808 56.9696 10

-3 -2 -11-0.50304 51.0489 10

3 2 11-18.4843 37.8960 10

-3 2 -11 42.0655 55.8244 10

-3 2 -11 33.7445 51.5173 6

-3 -2 -11 123.792 80.0221 6

-3 2 -12-60.1106 60.6772 10

-3 -2 -12 103.983 74.7785 10

-3 -2 -12-132.196 103.207 6

-3 2 -12-113.639 104.142 6

-3 2 -13 120.742 78.7627 10

-3 -2 -13 17.6693 31.2713 10

-3 -2 -13 87.5831 55.7380 6

3 -3 -17-40.8719 112.490 10

3 3 -16 14.2118 50.8824 10

3 -3 -16-27.7358 57.2889 10

3 3 -15 67.4776 48.5701 10

3 -3 -15-66.1666 66.7220 10

3 -3 -15-56.9678 74.3587 6

3 3 -15 79.5286 70.8085 6

3 -3 -14 437.097 140.184 10

3 3 -14 436.777 143.127 10

3 3 -14 442.111 168.710 6

3 -3 -14 263.615 117.703 6

-3 -3 13 25.4845 50.9025 4

-3 -3 13-74.0932 53.4166 1

3 -3 -13 40.4000 47.5309 10

3 3 -13-42.3875 71.9181 10

3 3 -13-26.9959 97.1257 6

3 -3 -13-10.2205 58.3465 6

-3 -3 12 3242.30 338.897 4

-3 -3 12 2942.29 292.848 1

3 3 -12 3203.84 359.576 10

3 -3 -12 3089.01 337.166 10

3 3 -12 2814.19 359.775 6

3 -3 -12 3081.96 356.488 6

-3 -3 11 83.9087 58.4516 4

-3 3 11 132.117 48.8321 1

3 -3 -11 82.3347 51.9040 10

3 3 -11 85.7680 67.2386 10

3 -3 -11 183.976 85.3183 6

3 3 -11 23.0662 53.8373 6

-3 -3 10 3982.36 348.761 4

-3 3 10 4052.45 359.509 1

-3 -3 10 4508.48 369.787 10

3 -3 -10 4079.41 347.929 10

-3 3 10 4198.17 372.472 10

3 3 -10 3966.66 355.181 10

-3 -3 10 4103.95 183.356 8

3 -3 -10 3805.52 354.248 6

3 3 -10 3982.91 381.518 6

-3 3 9-8.17156 35.8395 4

-3 3 9-17.7126 71.0857 1

-3 -3 9-7.74228 32.8760 1

-3 3 9 30.3107 38.3327 10

-3 -3 9 20.5880 40.7660 10

3 3 -9 65.1647 68.8496 10

3 -3 -9-1.98736 39.1295 10

3 -3 -9 43.0507 39.0923 6

-3 3 9 29.1123 40.7870 8

3 3 -9-65.1959 75.7833 6

-3 -3 8 80.5123 49.9684 1

3 3 -8 72.2698 47.6062 10

-3 3 8 26.5036 41.0971 10

-3 -3 8 101.213 47.5928 10

3 -3 -8 44.1073 34.5267 10

-3 3 8 86.4924 33.4556 8

3 -3 -8 96.8878 55.5520 6

-3 3 7 7775.56 422.427 10

3 -3 -7 8013.41 404.103 10

-3 -3 7 8277.41 423.941 10

-3 3 7 7609.18 355.376 8

-3 -3 7 8042.56 232.401 5

-3 3 6 135.394 48.6992 4

-3 -3 6 90.6759 26.5055 5

-3 3 6 75.0583 44.5467 1

-3 -3 6 82.5637 40.1478 10

-3 3 6 85.3286 40.7157 10

3 -3 -6 97.8516 43.5723 6

-3 3 6 68.8382 22.6278 6

-3 3 6 45.5666 31.1945 8

-3 3 5 983.680 133.123 1

-3 3 5 975.735 130.308 10

-3 -3 5 865.773 115.759 10

3 -3 -5 912.724 115.480 6

-3 3 5 907.089 120.065 6

-3 3 4 2889.91 135.350 5

-3 3 4 3021.68 221.870 1

-3 3 4 3183.94 209.973 10

-3 -3 4 3043.77 200.398 10

3 -3 -4 3086.11 133.778 5

3 -3 -4 3143.88 188.601 6

-3 3 4 3051.91 198.693 6

-3 3 3 542.234 70.1073 5

-3 3 3 383.716 71.4303 10

-3 -3 3 353.074 72.4222 10

-3 3 3 446.250 65.3021 6

3 -3 -3 372.725 56.7378 5

-3 3 2 47571.3 695.637 4

-3 3 2 43251.6 634.942 10

3 3 -2 44512.1 610.380 10

-3 -3 2 44154.7 588.762 10

-3 3 2 46448.7 620.223 5

3 -3 -2 44969.6 604.918 5

-3 3 1 34322.7 511.816 4

3 -3 -1 35644.5 458.742 3

3 -3 -1 34588.9 525.569 2

3 3 -1 34524.3 449.439 10

-3 3 1 36563.5 526.367 9

-3 -3 1 33669.0 423.694 10

-3 -3 1 35398.5 501.179 7

3 -3 -1 34071.2 561.330 5

3 -3 0 124277. 823.344 3

-3 3 0 120514. 809.911 3

-3 3 0 122375. 1055.82 2

-3 3 0 120534. 951.439 9

3 3 0 120047. 645.819 10

3 -3 0 118277. 1099.29 5

-3 -3 0 120885. 909.328 7

3 3 0 122272. 926.498 7

3 3 0 120115. 814.976 6

-3 3 -1 7979.08 292.291 2

-3 -3 -1 7901.86 279.958 1

3 3 1 7840.32 283.514 1

-3 3 -1 7810.57 209.576 3

3 3 1 7998.61 143.582 3

-3 -3 -1 7907.66 144.784 3

3 3 1 7267.89 253.202 6

-3 -3 -1 7794.01 240.874 6

-3 3 -1 7745.73 300.273 5

3 3 1 7985.45 241.395 7

-3 -3 -2 81136.6 515.313 3

3 3 2 82606.9 981.678 1

-3 3 -2 79252.2 625.471 1

-3 3 -2 88213.3 1027.95 2

-3 -3 -2 86432.0 940.592 1

3 3 2 76233.6 529.357 10

-3 3 -2 81591.4 1026.13 5

-3 -3 -2 77579.3 914.567 6

3 3 2 76168.1 963.641 6

-3 -3 -3 6658.00 288.167 1

-3 3 -3 4072.65 241.167 2

3 3 3 3571.74 223.008 1

3 3 3 3065.62 157.032 2

-3 3 -3 2437.81 145.601 10

-3 -3 -3 1692.43 165.044 6

3 3 3 1748.06 170.466 6

-3 3 -3 3183.59 218.409 5

-3 3 -4 3527.48 232.222 2

-3 -3 -4 3783.82 233.339 1

-3 3 -4 3764.49 157.951 1

3 3 4 3701.37 237.190 1

3 3 4 3662.57 179.369 2

-3 3 -4 3688.21 200.486 10

-3 -3 -4 3737.32 167.242 10

3 3 4 3655.30 177.533 10

3 3 4 3910.77 276.366 6

-3 -3 -4 3921.04 266.815 6

-3 3 -4 3805.18 240.317 5

3 -3 5 782.751 83.3223 1

-3 -3 -5 705.675 110.119 1

-3 3 -5 633.655 77.8091 1

-3 3 -5 634.596 105.133 2

3 3 5 598.219 105.509 1

3 3 5 730.557 92.5220 10

-3 -3 -5 780.105 87.2331 10

-3 3 -5 687.591 97.5220 10

-3 3 -5 759.874 112.135 5

-3 -3 -5 741.132 124.256 6

-3 3 -6 144.723 56.7264 5

-3 -3 -6 53.3676 30.4810 4

-3 -3 -6 119.856 48.8054 1

3 -3 6 120.534 39.1825 1

-3 3 -6 132.252 48.4855 2

3 3 6 132.140 50.4083 1

-3 3 -6 106.716 36.5218 1

3 3 6 117.970 41.7183 10

-3 -3 -6 134.814 49.7669 10

-3 3 -6 157.163 61.3278 10

-3 -3 -6 169.102 73.4038 6

-3 -3 -7 13886.4 543.869 4

-3 3 -7 13055.5 509.720 2

3 -3 7 14018.5 414.818 1

-3 3 -7 13443.0 424.057 1

-3 -3 -7 13538.4 547.862 1

-3 -3 -7 15483.3 492.089 10

3 3 7 14723.3 492.524 10

-3 3 -7 15117.3 541.150 10

3 -3 7 15851.5 511.601 10

-3 -3 -7 12281.0 369.736 8

-3 -3 -7 15327.3 680.475 6

-3 3 -7 14326.1 520.098 5

-3 -3 -8 11.1628 31.7732 4

-3 3 -8-18.0883 24.5520 5

-3 3 -8 31.9645 39.6429 1

-3 3 -8 13.6232 39.0776 2

-3 -3 -8 24.2193 39.8132 1

3 -3 8 17.4876 38.1761 1

3 3 8 27.8339 36.1220 1

-3 -3 -8 13.9406 33.7058 10

-3 3 -8 23.6009 31.6004 10

3 -3 8 31.9892 21.7035 10

3 3 8-11.3639 25.6792 10

-3 3 -8-23.1681 54.2550 6

-3 -3 -8-11.6023 15.6983 8

-3 -3 -8-27.5426 48.9457 6

-3 -3 -9 178.657 72.0906 4

-3 3 -9 203.876 65.4100 5

-3 -3 -9 122.676 94.1961 1

3 -3 9 148.996 57.7232 1

3 3 9 156.836 68.1937 1

-3 3 -9 151.026 60.3280 2

3 3 9 238.846 82.8706 10

3 -3 9 213.523 65.4468 10

-3 3 -9 214.213 89.3930 10

-3 -3 -9 244.058 79.3892 10

-3 -3 -9 162.557 92.2809 6

-3 -3 -9 126.439 55.1605 5

-3 3 -9 318.405 118.204 6

-3 3 -10 1505.94 176.428 5

3 3 10 1329.77 212.109 1

-3 -3 -10 1683.02 238.822 1

3 -3 10 1606.45 173.572 1

-3 3 -10 1303.86 184.296 2

-3 -3 -10 1867.64 213.216 10

3 3 10 1519.67 194.966 10

3 -3 10 1435.46 190.668 10

-3 3 -10 1724.02 229.093 10

-3 -3 -10 1722.22 185.903 5

-3 3 -10 1809.91 294.212 6

-3 -3 -10 1742.53 282.990 6

-3 -3 -11 75.9099 49.6528 5

-3 3 -11 81.6670 44.1756 5

3 3 11 27.6197 39.6960 1

-3 -3 -11 114.289 72.6215 1

-3 -3 -11 67.2122 32.0137 10

3 3 11-5.55063 48.2513 10

-3 3 -11 79.9471 51.0243 10

-3 -3 -11 63.9976 64.4162 6

-3 3 -11 81.4924 84.6160 6

-3 -3 -12 23.2750 49.9545 10

-3 3 -12 36.5205 49.9594 10

-3 -3 -12 28.7251 52.9836 6

-3 3 -12 27.6730 58.6138 6

-3 3 -13-20.3235 75.2675 10

-3 -3 -13-171.655 125.583 6

-3 3 -13 22.0573 95.8928 6

3 -4 -16 117.587 110.580 10

3 4 -16 57.5075 93.6744 10

3 4 -15 55.5344 73.3041 10

3 -4 -15 113.129 80.1211 10

3 -4 -15 54.6830 62.8726 6

3 4 -15 72.1010 90.7848 6

3 -4 -14 5.14843 64.0222 10

3 4 -14-29.2472 61.2174 10

3 4 -14 12.2800 63.9232 6

3 -4 -14 23.9392 47.9581 6

-3 -4 13 416.038 126.743 4

3 -4 -13 292.732 109.898 10

3 4 -13 290.152 120.810 10

3 4 -13 416.822 152.200 6

3 -4 -13 250.886 112.757 6

-3 -4 12 1486.20 230.819 4

-3 -4 12 1079.19 173.847 1

3 -4 -12 1027.94 82.2832 2

3 -4 -12 1267.44 214.238 10

3 4 -12 1481.33 246.813 10

3 4 -12 1542.15 268.246 6

3 -4 -12 1364.91 244.872 6

-3 -4 11 88.7218 88.0064 4

-3 4 11-3.55753 49.1600 1

3 -4 -11 24.8143 43.2548 10

3 4 -11 80.9849 42.4178 10

3 -4 -11 58.5637 61.5669 6

3 4 -11-39.4090 57.2071 6

-3 -4 10 4979.44 394.101 4

-3 -4 10 4341.83 322.245 1

-3 4 10 4275.71 376.872 1

3 -4 -10 4595.50 363.949 10

-3 4 10 4751.21 401.919 10

3 4 -10 4646.43 388.618 10

3 4 -10 4266.00 391.246 6

3 -4 -10 4470.70 388.803 6

-3 -4 9 1274.25 169.530 1

-3 4 9 1275.60 197.640 1

-3 -4 9 1386.50 195.343 10

-3 4 9 1340.63 201.996 10

3 4 -9 1122.94 178.971 10

3 -4 -9 1300.78 187.447 10

3 -4 -9 1125.72 179.800 6

-3 4 9 1156.12 154.832 8

-3 4 8 1934.42 228.549 4

-3 -4 8 2017.41 198.144 1

-3 4 8 1736.98 231.837 1

-3 -4 8 2151.29 226.276 10

3 -4 -8 1891.05 213.573 10

3 4 -8 1974.25 224.711 10

-3 4 8 1784.16 208.935 10

-3 -4 8 2035.73 128.066 5

3 -4 -8 1920.55 220.224 6

-3 4 8 1931.58 188.663 8

-3 -4 7 240.319 49.5893 5

-3 4 7 253.251 77.1305 10

-3 -4 7 308.387 84.7845 10

3 -4 -7 316.656 83.0143 10

-3 4 7 299.156 72.0336 8

-3 4 7 313.978 93.1091 6

-3 -4 6 110.387 34.9592 5

-3 4 6 144.740 55.7048 1

-3 -4 6 142.699 57.1239 10

-3 4 6 131.970 60.7088 10

-3 4 6 75.4537 42.0304 8

-3 4 6 139.178 59.7490 6

3 -4 -6 86.7226 22.9317 5

3 -4 -6 56.3338 40.2047 6

-3 -4 5 801.873 145.179 10

-3 4 5 1061.62 147.735 10

3 -4 -5 641.820 74.7125 5

-3 4 5 972.907 143.203 6

3 -4 -5 991.990 136.906 6

-3 4 5 816.689 131.820 8

3 -4 -4 18598.8 560.132 4

-3 4 4 18522.8 386.983 5

-3 -4 4 19975.8 510.342 10

-3 4 4 19535.2 532.780 10

-3 4 4 20244.9 518.034 6

3 -4 -4 18965.8 381.062 5

3 -4 -4 19355.8 482.842 6

3 -4 -3 119769. 1285.03 4

-3 4 3 117233. 1023.43 5

-3 -4 3 124166. 1137.37 10

3 -4 -3 123777. 1079.12 9

-3 4 3 126196. 1242.57 10

3 -4 -3 118528. 1017.63 5

-3 4 3 123255. 1108.61 6

-3 4 2 104430. 1018.64 5

3 -4 -2 105186. 1093.52 4

-3 4 2 101742. 1063.81 4

3 -4 -2 106651. 907.573 2

3 4 -2 102057. 960.602 10

-3 -4 2 100945. 896.816 10

-3 4 2 98351.7 949.395 9

-3 -4 2 100734. 932.631 7

3 -4 -2 105716. 1018.51 5

-3 4 1 14258.8 341.457 4

3 -4 -1 13855.4 310.602 3

-3 4 1 13918.4 326.182 3

3 -4 -1 14802.3 369.048 2

-3 4 1 14204.7 359.532 2

-3 4 1 14155.3 345.981 9

3 4 -1 13595.7 296.707 10

-3 -4 1 14017.3 332.774 7

-3 4 1 14699.1 398.608 5

3 -4 -1 14184.5 392.202 5

-3 4 0 89.8619 28.8151 2

-3 -4 0 82.6840 31.8057 1

3 -4 0 93.9779 29.7472 3

-3 4 0 112.361 25.9160 4

-3 4 0 94.6779 23.9449 3

3 -4 0 123.834 41.6105 5

-3 4 0 87.6810 26.6803 9

-3 -4 -1 14801.1 396.858 1

-3 4 -1 15415.4 427.802 2

-3 -4 -1 16591.7 258.020 3

3 4 1 17326.5 262.463 3

-3 4 -1 16761.3 344.266 3

3 -4 1 16110.8 458.773 5

3 4 1 16824.1 395.817 6

-3 4 -1 15902.2 446.863 5

-3 4 -2 34093.3 678.522 2

-3 -4 -2 33528.9 625.108 1

3 4 2 33487.6 654.799 1

-3 4 -2 33661.0 517.994 3

-3 -4 -2 32822.1 380.457 3

-3 -4 -2 32378.7 595.028 6

3 -4 2 32887.2 676.070 5

-3 4 -2 32772.6 669.370 5

3 4 2 32984.4 656.947 6

-3 -4 -3 30719.5 416.138 3

3 4 3 29847.9 654.862 1

-3 4 -3 28431.4 332.098 1

-3 4 -3 29561.0 676.021 2

-3 -4 -3 28191.0 615.480 1

-3 4 -3 31364.4 535.779 10

3 4 3 29943.8 448.679 10

-3 -4 -3 30474.7 662.164 6

-3 4 -3 30776.3 674.669 5

3 4 3 31782.6 728.501 6

-3 -4 -4 5880.02 299.719 1

-3 4 -4 5772.09 178.174 1

-3 4 -4 5389.85 302.544 2

3 4 4 5341.81 203.957 2

3 4 4 5999.14 317.217 1

-3 4 -4 5572.06 256.446 10

3 4 4 5302.81 218.351 10

3 4 4 5786.99 342.211 6

-3 -4 -4 5272.85 308.708 6

-3 4 -4 5715.24 191.322 8

-3 4 -4 5504.20 299.031 5

3 4 5 1832.58 189.739 1

-3 -4 -5 2427.93 214.206 1

-3 4 -5 2114.50 125.206 1

-3 4 -5 1648.35 175.621 2

-3 4 -5 1863.90 165.917 10

3 4 5 1767.52 143.345 10

-3 4 -5 1822.42 183.734 5

-3 -4 -5 2015.50 208.130 6

-3 4 -5 1893.01 89.8877 8

3 4 5 1718.45 208.877 6

-3 -4 -6 471.229 97.6510 4

-3 4 -6 431.064 92.5882 5

-3 -4 -6 372.869 93.5851 1

-3 4 -6 432.032 62.1496 1

-3 4 -6 445.336 95.7420 2

3 4 6 418.424 78.1527 10

-3 4 -6 422.979 87.2453 10

-3 -4 -6 483.342 109.767 6

-3 -4 -7 526.404 109.597 4

-3 4 -7 608.235 116.633 5

3 -4 7 571.641 82.0405 1

-3 -4 -7 637.925 126.486 1

-3 4 -7 576.697 84.1028 1

-3 4 -7 599.742 120.921 2

3 4 7 501.103 93.2990 10

-3 -4 -7 660.271 103.681 10

-3 4 -7 678.558 121.943 10

-3 -4 -7 713.589 153.760 6

-3 -4 -7 540.524 86.8161 8

-3 -4 -8 3509.26 302.175 4

-3 4 -8 3559.95 274.497 5

3 4 8 3616.71 315.022 1

-3 4 -8 3090.32 268.379 2

-3 -4 -8 3627.27 327.886 1

3 -4 8 3315.25 205.429 1

3 4 8 3655.74 269.056 10

-3 -4 -8 3760.22 263.406 10

-3 4 -8 3553.70 288.493 10

-3 -4 -8 2987.52 207.844 8

-3 -4 -8 3563.14 348.613 6

-3 -4 -9 1907.73 232.354 4

-3 4 -9 2013.35 207.108 5

-3 4 -9 1692.04 208.134 2

3 4 9 1901.38 243.610 1

-3 -4 -9 1967.96 244.644 1

3 -4 9 1774.10 160.096 1

3 4 9 1958.97 209.191 10

-3 4 -9 1996.69 234.575 10

-3 -4 -9 2046.42 206.871 10

-3 -4 -9 1901.26 267.617 6

-3 4 -9 1786.17 273.872 6

-3 4 -10-17.4049 52.4316 5

-3 -4 -10-78.5534 77.4715 4

3 -4 10 27.8329 28.0970 1

-3 4 -10 22.4187 35.4819 2

-3 -4 -10 49.7347 58.3979 1

3 4 10-0.72228 41.5604 1

3 4 10-59.2351 55.3890 10

-3 4 -10 33.4384 43.8797 10

-3 -4 -10 13.8645 38.4221 10

-3 -4 -10 1.72463 48.3706 6

-3 4 -10 8.52459 54.9093 6

-3 -4 -10 15.2433 39.2422 5

-3 4 -11 93.7951 51.6572 5

-3 -4 -11 154.032 76.1983 1

-3 4 -11 228.776 94.4972 10

3 4 11 176.666 77.1070 10

-3 -4 -11 224.622 122.627 6

-3 -4 -11 135.987 60.9570 5

-3 4 -11 166.266 116.575 6

-3 -4 -12 102.649 67.4697 1

-3 4 -12 128.906 63.2308 10

-3 4 -12 221.066 131.040 6

-3 -4 -12 178.937 113.254 6

-3 4 -13 51.8417 86.7610 10

-3 -4 -13-8.18806 54.1192 6

-3 4 -13-70.4999 80.8720 6

3 5 -16-75.5018 80.0004 10

3 -5 -15-89.6476 77.3253 10

3 5 -15 118.327 77.1370 10

3 -5 -15-43.7744 73.5618 6

3 5 -15 69.2726 93.6877 6

-3 -5 14 443.372 140.932 4

3 -5 -14 433.420 142.410 10

3 5 -14 542.939 175.063 10

3 5 -14 569.268 187.705 6

3 -5 -14 398.968 140.136 6

-3 -5 13 105.917 66.5904 4

3 -5 -13 218.699 106.063 10

3 5 -13 71.4874 76.8412 10

3 -5 -13 221.202 103.452 6

3 5 -13-9.79427 68.6798 6

-3 -5 12 57.7869 43.9855 4

3 5 -12 189.118 84.0578 10

3 5 -12 66.4608 58.9323 6

3 -5 -12 117.374 76.2271 6

-3 -5 11-58.7938 66.9574 4

3 -5 -11-23.0830 55.2279 10

3 5 -11 55.3141 41.9715 10

3 -5 -11-11.6526 65.0412 6

3 5 -11 86.3808 71.5482 6

-3 -5 10 644.424 142.319 4
[truncated: 1,259,767 more chars]
