# Supplementary material for: Antineoplastic 4-piperidone-1-phosphonothioates with potential multi-targeted inhibitory properties
Source: Sci Rep. 2025 Nov 18;15:40363. doi: 10.1038/s41598-025-25796-6 (PMC12627723; doi:10.1038/s41598-025-25796-6)
Supplement: Supplementary file 4 — Supplementary Material 4 [file 41598_2025_25796_MOESM4_ESM.docx]

data_bmk2351b

_audit_creation_method 'SHELXL-2019/3'

_shelx_SHELXL_version_number '2019/3'

_chemical_name_systematic ?

_chemical_name_common ?

_chemical_melting_point ?

_chemical_formula_moiety ?

_chemical_formula_sum

'C23 H24 F2 N O3 P S'

_chemical_formula_weight 463.46

loop_

_atom_type_symbol

_atom_type_description

_atom_type_scat_dispersion_real

_atom_type_scat_dispersion_imag

_atom_type_scat_source

'C' 'C' 0.0181 0.0091

'International Tables Vol C Tables 4.2.6.8 and 6.1.1.4'

'H' 'H' 0.0000 0.0000

'International Tables Vol C Tables 4.2.6.8 and 6.1.1.4'

'N' 'N' 0.0311 0.0180

'International Tables Vol C Tables 4.2.6.8 and 6.1.1.4'

'O' 'O' 0.0492 0.0322

'International Tables Vol C Tables 4.2.6.8 and 6.1.1.4'

'F' 'F' 0.0727 0.0534

'International Tables Vol C Tables 4.2.6.8 and 6.1.1.4'

'P' 'P' 0.2955 0.4335

'International Tables Vol C Tables 4.2.6.8 and 6.1.1.4'

'S' 'S' 0.3331 0.5567

'International Tables Vol C Tables 4.2.6.8 and 6.1.1.4'

_space_group_crystal_system orthorhombic

_space_group_IT_number 33

_space_group_name_H-M_alt 'P n a 21'

_space_group_name_Hall 'P 2c -2n'

_shelx_space_group_comment

;

The symmetry employed for this shelxl refinement is uniquely defined

by the following loop, which should always be used as a source of

symmetry information in preference to the above space-group names.

They are only intended as comments.

;

loop_

_space_group_symop_operation_xyz

'x, y, z'

'-x, -y, z+1/2'

'x+1/2, -y+1/2, z'

'-x+1/2, y+1/2, z+1/2'

_cell_length_a 32.9400(5)

_cell_length_b 12.5414(2)

_cell_length_c 22.4554(4)

_cell_angle_alpha 90

_cell_angle_beta 90

_cell_angle_gamma 90

_cell_volume 9276.6(3)

_cell_formula_units_Z 16

_cell_measurement_temperature 293(2)

_cell_measurement_reflns_used 15892

_cell_measurement_theta_min 3.7120

_cell_measurement_theta_max 72.5310

_exptl_crystal_description needle

_exptl_crystal_colour colourless

_exptl_crystal_density_meas ?

_exptl_crystal_density_method ?

_exptl_crystal_density_diffrn 1.327

_exptl_crystal_F_000 3872

_exptl_transmission_factor_min ?

_exptl_transmission_factor_max ?

_exptl_crystal_size_max 0.590

_exptl_crystal_size_mid 0.120

_exptl_crystal_size_min 0.100

_exptl_absorpt_coefficient_mu 2.245

_shelx_estimated_absorpt_T_min 0.351

_shelx_estimated_absorpt_T_max 0.807

_exptl_absorpt_correction_T_min 0.450

_exptl_absorpt_correction_T_max 1.000

_exptl_absorpt_correction_type gaussian

_exptl_absorpt_process_details

;

CrysAlisPro 1.171.42.90a (Rigaku Oxford Diffraction, 2023)

Numerical absorption correction based on gaussian integration over

a multifaceted crystal model

Empirical absorption correction using spherical harmonics,

implemented in SCALE3 ABSPACK scaling algorithm.

;

_diffrn_ambient_temperature 293(2)

_diffrn_radiation_wavelength 1.54184

_diffrn_radiation_type CuK\a

_diffrn_source ?

_diffrn_measurement_device_type 'SuperNova, Dual, Cu at home/near, Atlas'

_diffrn_measurement_method '\w scans'

_diffrn_detector_area_resol_mean 10.5082

_diffrn_reflns_number 37910

_diffrn_reflns_av_unetI/netI 0.0291

_diffrn_reflns_av_R_equivalents 0.0307

_diffrn_reflns_limit_h_min -36

_diffrn_reflns_limit_h_max 40

_diffrn_reflns_limit_k_min -12

_diffrn_reflns_limit_k_max 15

_diffrn_reflns_limit_l_min -27

_diffrn_reflns_limit_l_max 26

_diffrn_reflns_theta_min 3.771

_diffrn_reflns_theta_max 72.795

_diffrn_reflns_theta_full 67.684

_diffrn_measured_fraction_theta_max 0.983

_diffrn_measured_fraction_theta_full 0.998

_diffrn_reflns_Laue_measured_fraction_max 0.983

_diffrn_reflns_Laue_measured_fraction_full 0.998

_diffrn_reflns_point_group_measured_fraction_max 0.843

_diffrn_reflns_point_group_measured_fraction_full 0.875

_reflns_number_total 15591

_reflns_number_gt 12652

_reflns_threshold_expression 'I > 2\s(I)'

_reflns_Friedel_coverage 0.671

_reflns_Friedel_fraction_max 0.696

_reflns_Friedel_fraction_full 0.745

_reflns_special_details

;

Reflections were merged by SHELXL according to the crystal

class for the calculation of statistics and refinement.

_reflns_Friedel_fraction is defined as the number of unique

Friedel pairs measured divided by the number that would be

possible theoretically, ignoring centric projections and

systematic absences.

;

_computing_data_collection 'CrysAlisPro 1.171.42.90a (Rigaku OD, 2023)'

_computing_cell_refinement 'CrysAlisPro 1.171.42.90a (Rigaku OD, 2023)'

_computing_data_reduction 'CrysAlisPro 1.171.42.90a (Rigaku OD, 2023)'

_computing_structure_solution 'SHELXT 2014/5 (Sheldrick, 2014)'

_computing_structure_refinement 'SHELXL-2019/2 (Sheldrick, 2019)'

_computing_molecular_graphics ?

_computing_publication_material ?

_refine_special_details ?

_refine_ls_structure_factor_coef Fsqd

_refine_ls_matrix_type full

_refine_ls_weighting_scheme calc

_refine_ls_weighting_details

'w=1/[\s^2^(Fo^2^)+(0.0952P)^2^+2.3591P] where P=(Fo^2^+2Fc^2^)/3'

_atom_sites_solution_primary ?

_atom_sites_solution_secondary ?

_atom_sites_solution_hydrogens geom

_refine_ls_hydrogen_treatment constr

_refine_ls_extinction_method none

_refine_ls_extinction_coef .

_refine_ls_abs_structure_details

;

Flack x determined using 4278 quotients [(I+)-(I-)]/[(I+)+(I-)]

(Parsons, Flack and Wagner, Acta Cryst. B69 (2013) 249-259).

;

_refine_ls_abs_structure_Flack 0.082(9)

_chemical_absolute_configuration ?

_refine_ls_number_reflns 15591

_refine_ls_number_parameters 1389

_refine_ls_number_restraints 1317

_refine_ls_R_factor_all 0.0705

_refine_ls_R_factor_gt 0.0567

_refine_ls_wR_factor_ref 0.1709

_refine_ls_wR_factor_gt 0.1536

_refine_ls_goodness_of_fit_ref 1.027

_refine_ls_restrained_S_all 1.059

_refine_ls_shift/su_max 0.001

_refine_ls_shift/su_mean 0.000

loop_

_atom_site_label

_atom_site_type_symbol

_atom_site_fract_x

_atom_site_fract_y

_atom_site_fract_z

_atom_site_U_iso_or_equiv

_atom_site_adp_type

_atom_site_occupancy

_atom_site_site_symmetry_order

_atom_site_calc_flag

_atom_site_refinement_flags_posn

_atom_site_refinement_flags_adp

_atom_site_refinement_flags_occupancy

_atom_site_disorder_assembly

_atom_site_disorder_group

C1 C 0.19257(18) 0.3890(7) 0.6002(4) 0.091(2) Uani 1 1 d . . . . .

C2 C 0.20259(19) 0.2887(7) 0.6134(4) 0.0871(19) Uani 1 1 d . . . . .

H2 H 0.183175 0.240045 0.626206 0.105 Uiso 1 1 calc R U . . .

C3 C 0.24302(17) 0.2596(5) 0.6073(3) 0.0723(15) Uani 1 1 d . . . . .

H3 H 0.250741 0.190049 0.616115 0.087 Uiso 1 1 calc R U . . .

C4 C 0.27247(15) 0.3327(4) 0.5883(2) 0.0578(11) Uani 1 1 d . . . . .

C5 C 0.26004(17) 0.4339(5) 0.5747(3) 0.0725(14) Uani 1 1 d . . . . .

H5 H 0.278966 0.483792 0.561763 0.087 Uiso 1 1 calc R U . . .

C6 C 0.21932(19) 0.4632(5) 0.5800(4) 0.088(2) Uani 1 1 d . . . . .

H6 H 0.210731 0.531576 0.570010 0.106 Uiso 1 1 calc R U . . .

C7 C 0.31382(15) 0.2939(4) 0.5795(2) 0.0568(11) Uani 1 1 d . . . . .

H7 H 0.316068 0.221809 0.570302 0.068 Uiso 1 1 calc R U . . .

C8 C 0.35410(14) 0.4601(4) 0.6036(3) 0.0579(11) Uani 1 1 d . . . . .

H8A H 0.351477 0.508369 0.570082 0.069 Uiso 1 1 calc R U . . .

H8B H 0.332969 0.477182 0.632171 0.069 Uiso 1 1 calc R U . . .

C9 C 0.34917(14) 0.3474(3) 0.5828(2) 0.0506(10) Uani 1 1 d . . . . .

C10 C 0.38644(15) 0.2856(4) 0.5660(2) 0.0548(11) Uani 1 1 d . . . . .

C11 C 0.42678(15) 0.3382(4) 0.5721(2) 0.0554(10) Uani 1 1 d . . . . .

C12 C 0.42748(14) 0.4534(4) 0.5906(2) 0.0575(11) Uani 1 1 d . . . . .

H12A H 0.453097 0.469605 0.609871 0.069 Uiso 1 1 calc R U . . .

H12B H 0.424916 0.498587 0.555751 0.069 Uiso 1 1 calc R U . . .

C13 C 0.45957(16) 0.2762(4) 0.5649(3) 0.0644(13) Uani 1 1 d . . . . .

H13 H 0.454178 0.204569 0.557675 0.077 Uiso 1 1 calc R U . . .

C14 C 0.50238(16) 0.3046(5) 0.5669(3) 0.0682(13) Uani 1 1 d . . . . .

C15 C 0.5299(2) 0.2271(6) 0.5861(4) 0.095(2) Uani 1 1 d . . . . .

H15 H 0.520174 0.161904 0.599700 0.114 Uiso 1 1 calc R U . . .

C16 C 0.5709(2) 0.2452(7) 0.5852(5) 0.105(3) Uani 1 1 d . . . . .

H16 H 0.588951 0.193347 0.598603 0.126 Uiso 1 1 calc R U . . .

C17 C 0.58489(18) 0.3405(7) 0.5644(4) 0.095(2) Uani 1 1 d . . . . .

C18 C 0.5591(2) 0.4199(6) 0.5458(4) 0.0872(18) Uani 1 1 d . . . . .

H18 H 0.569330 0.484842 0.532613 0.105 Uiso 1 1 calc R U . . .

C19 C 0.51798(17) 0.4017(5) 0.5470(3) 0.0722(14) Uani 1 1 d . . . . .

H19 H 0.500284 0.454836 0.534361 0.087 Uiso 1 1 calc R U . . .

C20 C 0.4302(4) 0.3284(8) 0.7454(7) 0.073(2) Uani 0.505(13) 1 d D U P A 1

H20A H 0.419526 0.288852 0.711744 0.088 Uiso 0.505(13) 1 calc R U P A 1

H20B H 0.410631 0.325104 0.777558 0.088 Uiso 0.505(13) 1 calc R U P A 1

C21 C 0.4701(5) 0.2810(11) 0.7651(8) 0.082(3) Uani 0.505(13) 1 d D U P A 1

H21A H 0.490138 0.292241 0.734751 0.122 Uiso 0.505(13) 1 calc R U P A 1

H21B H 0.466730 0.205926 0.771776 0.122 Uiso 0.505(13) 1 calc R U P A 1

H21C H 0.478689 0.314720 0.801341 0.122 Uiso 0.505(13) 1 calc R U P A 1

C22 C 0.4343(4) 0.6927(9) 0.7354(7) 0.079(2) Uani 0.505(13) 1 d D U P A 1

H22A H 0.417746 0.675340 0.769821 0.095 Uiso 0.505(13) 1 calc R U P A 1

H22B H 0.426529 0.762929 0.721424 0.095 Uiso 0.505(13) 1 calc R U P A 1

C23 C 0.4771(4) 0.6925(15) 0.7521(8) 0.098(4) Uani 0.505(13) 1 d D U P A 1

H23A H 0.485036 0.621795 0.763585 0.147 Uiso 0.505(13) 1 calc R U P A 1

H23B H 0.481270 0.740469 0.784805 0.147 Uiso 0.505(13) 1 calc R U P A 1

H23C H 0.493186 0.715134 0.718714 0.147 Uiso 0.505(13) 1 calc R U P A 1

O2 O 0.4374(2) 0.4372(7) 0.7288(5) 0.0654(17) Uani 0.505(13) 1 d D U P A 1

O3 O 0.4276(3) 0.6147(6) 0.6888(4) 0.0716(15) Uani 0.505(13) 1 d D U P A 1

P1 P 0.40289(4) 0.50806(10) 0.70029(7) 0.0625(3) Uani 0.505(13) 1 d D U P A 1

S1 S 0.3535(3) 0.5211(10) 0.7460(6) 0.080(2) Uani 0.505(13) 1 d D U P A 1

C20A C 0.4319(4) 0.3208(8) 0.7304(8) 0.074(2) Uani 0.495(13) 1 d D U P A 2

H20C H 0.417706 0.285407 0.698119 0.089 Uiso 0.495(13) 1 calc R U P A 2

H20D H 0.415275 0.316170 0.765995 0.089 Uiso 0.495(13) 1 calc R U P A 2

C21A C 0.4725(6) 0.2685(12) 0.7409(9) 0.088(4) Uani 0.495(13) 1 d D U P A 2

H21D H 0.488729 0.274244 0.705612 0.133 Uiso 0.495(13) 1 calc R U P A 2

H21E H 0.468464 0.194660 0.750496 0.133 Uiso 0.495(13) 1 calc R U P A 2

H21F H 0.486009 0.303390 0.773383 0.133 Uiso 0.495(13) 1 calc R U P A 2

C22A C 0.4441(5) 0.6599(10) 0.7547(5) 0.078(2) Uani 0.495(13) 1 d D U P A 2

H22C H 0.459322 0.604515 0.774942 0.094 Uiso 0.495(13) 1 calc R U P A 2

H22D H 0.422460 0.683273 0.780991 0.094 Uiso 0.495(13) 1 calc R U P A 2

C23A C 0.4708(5) 0.7494(12) 0.7414(7) 0.088(3) Uani 0.495(13) 1 d D U P A 2

H23D H 0.498115 0.724115 0.736956 0.132 Uiso 0.495(13) 1 calc R U P A 2

H23E H 0.469607 0.800200 0.773278 0.132 Uiso 0.495(13) 1 calc R U P A 2

H23F H 0.462168 0.782768 0.705014 0.132 Uiso 0.495(13) 1 calc R U P A 2

O2A O 0.4393(3) 0.4303(7) 0.7155(5) 0.0664(17) Uani 0.495(13) 1 d D U P A 2

O3A O 0.4269(3) 0.6178(6) 0.7002(4) 0.0714(15) Uani 0.495(13) 1 d D U P A 2

P1A P 0.40289(4) 0.50806(10) 0.70029(7) 0.0625(3) Uani 0.495(13) 1 d D U P A 2

S1A S 0.3569(3) 0.4992(11) 0.7535(6) 0.082(2) Uani 0.495(13) 1 d D U P A 2

C24 C 0.67231(18) 0.6125(6) 0.5981(4) 0.093(2) Uani 1 1 d . . . . .

C25 C 0.6839(2) 0.7101(6) 0.6168(4) 0.099(2) Uani 1 1 d . . . . .

H25 H 0.665150 0.756598 0.633622 0.118 Uiso 1 1 calc R U . . .

C26 C 0.7236(2) 0.7399(5) 0.6106(4) 0.0845(19) Uani 1 1 d . . . . .

H26 H 0.731595 0.807733 0.622573 0.101 Uiso 1 1 calc R U . . .

C27 C 0.75219(17) 0.6701(4) 0.5867(3) 0.0663(13) Uani 1 1 d . . . . .

C28 C 0.73899(17) 0.5700(5) 0.5685(3) 0.0716(15) Uani 1 1 d . . . . .

H28 H 0.757504 0.522121 0.552360 0.086 Uiso 1 1 calc R U . . .

C29 C 0.69885(18) 0.5409(6) 0.5741(4) 0.087(2) Uani 1 1 d . . . . .

H29 H 0.690055 0.473950 0.561862 0.104 Uiso 1 1 calc R U . . .

C30 C 0.79390(17) 0.7072(4) 0.5786(3) 0.0641(13) Uani 1 1 d . . . . .

H30 H 0.796673 0.779463 0.570160 0.077 Uiso 1 1 calc R U . . .

C31 C 0.83189(15) 0.5366(4) 0.5995(3) 0.0587(11) Uani 1 1 d . . . . .

H31A H 0.807967 0.516117 0.621959 0.070 Uiso 1 1 calc R U . . .

H31B H 0.833462 0.491894 0.564319 0.070 Uiso 1 1 calc R U . . .

C32 C 0.82892(15) 0.6518(4) 0.5816(2) 0.0554(11) Uani 1 1 d . . . . .

C33 C 0.86757(15) 0.7107(3) 0.5698(3) 0.0563(11) Uani 1 1 d . . . . .

C34 C 0.90694(15) 0.6561(4) 0.5816(2) 0.0551(11) Uani 1 1 d . . . . .

C35 C 0.90584(15) 0.5428(4) 0.6036(3) 0.0627(12) Uani 1 1 d . . . . .

H35A H 0.907783 0.494413 0.570044 0.075 Uiso 1 1 calc R U . . .

H35B H 0.928937 0.529971 0.629468 0.075 Uiso 1 1 calc R U . . .

C36 C 0.94063(16) 0.7156(4) 0.5749(2) 0.0590(11) Uani 1 1 d . . . . .

H36 H 0.936049 0.786947 0.565810 0.071 Uiso 1 1 calc R U . . .

C37 C 0.98322(17) 0.6852(4) 0.5798(3) 0.0630(12) Uani 1 1 d . . . . .

C38 C 0.9984(2) 0.5863(5) 0.5648(3) 0.0779(16) Uani 1 1 d . . . . .

H38 H 0.980687 0.532710 0.552806 0.093 Uiso 1 1 calc R U . . .

C39 C 1.0394(2) 0.5656(6) 0.5673(4) 0.095(2) Uani 1 1 d . . . . .

H39 H 1.049701 0.499327 0.556468 0.113 Uiso 1 1 calc R U . . .

C40 C 1.0648(2) 0.6460(7) 0.5862(4) 0.094(2) Uani 1 1 d . . . . .

C41 C 1.0522(2) 0.7435(7) 0.5993(4) 0.098(2) Uani 1 1 d . . . . .

H41 H 1.070588 0.796579 0.609877 0.118 Uiso 1 1 calc R U . . .

C42 C 1.0109(2) 0.7640(5) 0.5969(3) 0.0788(17) Uani 1 1 d . . . . .

H42 H 1.001425 0.831557 0.606806 0.095 Uiso 1 1 calc R U . . .

C43 C 0.8258(5) 0.3416(11) 0.7659(6) 0.096(2) Uani 0.569(11) 1 d D U P B 1

H43A H 0.814101 0.399236 0.788968 0.116 Uiso 0.569(11) 1 calc R U P B 1

H43B H 0.848213 0.311657 0.788395 0.116 Uiso 0.569(11) 1 calc R U P B 1

C44 C 0.7957(5) 0.2609(12) 0.7554(8) 0.107(4) Uani 0.569(11) 1 d D U P B 1

H44A H 0.777554 0.284207 0.724576 0.161 Uiso 0.569(11) 1 calc R U P B 1

H44B H 0.780649 0.248494 0.791325 0.161 Uiso 0.569(11) 1 calc R U P B 1

H44C H 0.808791 0.196029 0.743252 0.161 Uiso 0.569(11) 1 calc R U P B 1

C45 C 0.8406(4) 0.6681(8) 0.7560(7) 0.090(2) Uani 0.569(11) 1 d D U P B 1

H45A H 0.857727 0.711977 0.730864 0.108 Uiso 0.569(11) 1 calc R U P B 1

H45B H 0.854716 0.652806 0.792952 0.108 Uiso 0.569(11) 1 calc R U P B 1

C46 C 0.7983(5) 0.7227(10) 0.7678(8) 0.103(3) Uani 0.569(11) 1 d D U P B 1

H46A H 0.783387 0.727073 0.731190 0.154 Uiso 0.569(11) 1 calc R U P B 1

H46B H 0.802517 0.793090 0.783410 0.154 Uiso 0.569(11) 1 calc R U P B 1

H46C H 0.783216 0.681118 0.796113 0.154 Uiso 0.569(11) 1 calc R U P B 1

O5 O 0.8404(3) 0.3820(6) 0.7103(4) 0.0882(16) Uani 0.569(11) 1 d D U P B 1

O6 O 0.8282(2) 0.5695(6) 0.7254(5) 0.0875(17) Uani 0.569(11) 1 d D U P B 1

P2 P 0.86424(6) 0.49331(13) 0.70621(9) 0.0782(4) Uani 0.569(11) 1 d D U P B 1

S2 S 0.91299(18) 0.5030(7) 0.7539(4) 0.0979(19) Uani 0.569(11) 1 d D U P B 1

C43A C 0.8319(5) 0.3091(10) 0.7428(10) 0.093(2) Uani 0.431(11) 1 d D U P B 2

H43C H 0.850322 0.322497 0.775709 0.112 Uiso 0.431(11) 1 calc R U P B 2

H43D H 0.837076 0.237733 0.727957 0.112 Uiso 0.431(11) 1 calc R U P B 2

C44A C 0.7906(6) 0.3170(18) 0.7631(10) 0.107(4) Uani 0.431(11) 1 d D U P B 2

H44D H 0.785119 0.388901 0.775418 0.160 Uiso 0.431(11) 1 calc R U P B 2

H44E H 0.786524 0.269587 0.796132 0.160 Uiso 0.431(11) 1 calc R U P B 2

H44F H 0.772526 0.297845 0.731300 0.160 Uiso 0.431(11) 1 calc R U P B 2

C45A C 0.8340(7) 0.6744(9) 0.7341(8) 0.093(2) Uani 0.431(11) 1 d D U P B 2

H45C H 0.817606 0.697404 0.700645 0.111 Uiso 0.431(11) 1 calc R U P B 2

H45D H 0.861784 0.698127 0.728371 0.111 Uiso 0.431(11) 1 calc R U P B 2

C46A C 0.8162(7) 0.7154(12) 0.7948(8) 0.102(4) Uani 0.431(11) 1 d D U P B 2

H46D H 0.827222 0.673885 0.826934 0.153 Uiso 0.431(11) 1 calc R U P B 2

H46E H 0.787204 0.708361 0.794422 0.153 Uiso 0.431(11) 1 calc R U P B 2

H46F H 0.823361 0.788963 0.800271 0.153 Uiso 0.431(11) 1 calc R U P B 2

O5A O 0.8388(4) 0.3851(7) 0.6966(5) 0.0881(17) Uani 0.431(11) 1 d D U P B 2

O6A O 0.8318(3) 0.5581(7) 0.7426(5) 0.0855(18) Uani 0.431(11) 1 d D U P B 2

P2A P 0.86424(6) 0.49331(13) 0.70621(9) 0.0782(4) Uani 0.431(11) 1 d D U P B 2

S2A S 0.9174(2) 0.4745(9) 0.7412(5) 0.097(2) Uani 0.431(11) 1 d D U P B 2

C47 C 0.1796(2) 0.6117(6) 0.4320(5) 0.097(2) Uani 1 1 d . . . . .

C48 C 0.2063(2) 0.6938(6) 0.4454(4) 0.091(2) Uani 1 1 d . . . . .

H48 H 0.197060 0.761611 0.455109 0.110 Uiso 1 1 calc R U . . .

C49 C 0.24731(18) 0.6706(4) 0.4436(3) 0.0754(15) Uani 1 1 d . . . . .

H49 H 0.265944 0.724595 0.451309 0.090 Uiso 1 1 calc R U . . .

C50 C 0.26138(16) 0.5695(4) 0.4307(3) 0.0649(13) Uani 1 1 d . . . . .

C51 C 0.2326(2) 0.4901(5) 0.4179(4) 0.0842(19) Uani 1 1 d . . . . .

H51 H 0.241244 0.421315 0.409070 0.101 Uiso 1 1 calc R U . . .

C52 C 0.1918(2) 0.5131(6) 0.4182(5) 0.104(3) Uani 1 1 d . . . . .

H52 H 0.172881 0.460577 0.408989 0.125 Uiso 1 1 calc R U . . .

C53 C 0.30387(16) 0.5370(4) 0.4337(3) 0.0630(12) Uani 1 1 d . . . . .

H53 H 0.307919 0.465490 0.442797 0.076 Uiso 1 1 calc R U . . .

C54 C 0.33988(16) 0.7083(4) 0.4046(3) 0.0621(12) Uani 1 1 d . . . . .

H54A H 0.317086 0.722576 0.378414 0.075 Uiso 1 1 calc R U . . .

H54B H 0.337794 0.755458 0.438647 0.075 Uiso 1 1 calc R U . . .

C55 C 0.33809(15) 0.5932(3) 0.4255(2) 0.0557(11) Uani 1 1 d . . . . .

C56 C 0.37694(16) 0.5356(4) 0.4364(3) 0.0567(11) Uani 1 1 d . . . . .

C57 C 0.41586(14) 0.5934(4) 0.4256(2) 0.0541(10) Uani 1 1 d . . . . .

C58 C 0.41335(15) 0.7102(4) 0.4093(3) 0.0574(11) Uani 1 1 d . . . . .

H58A H 0.411938 0.752974 0.445289 0.069 Uiso 1 1 calc R U . . .

H58B H 0.437508 0.730945 0.387516 0.069 Uiso 1 1 calc R U . . .

C59 C 0.45054(16) 0.5377(4) 0.4272(3) 0.0609(12) Uani 1 1 d . . . . .

H59 H 0.447340 0.465070 0.434099 0.073 Uiso 1 1 calc R U . . .

C60 C 0.49221(16) 0.5722(4) 0.4199(3) 0.0607(12) Uani 1 1 d . . . . .

C61 C 0.52039(18) 0.5054(5) 0.3918(3) 0.0739(16) Uani 1 1 d . . . . .

H61 H 0.511905 0.439113 0.378117 0.089 Uiso 1 1 calc R U . . .

C62 C 0.5599(2) 0.5347(6) 0.3840(4) 0.090(2) Uani 1 1 d . . . . .

H62 H 0.578068 0.489324 0.364988 0.108 Uiso 1 1 calc R U . . .

C63 C 0.57223(19) 0.6313(6) 0.4045(4) 0.0873(19) Uani 1 1 d . . . . .

C64 C 0.54643(18) 0.7003(5) 0.4344(4) 0.0854(19) Uani 1 1 d . . . . .

H64 H 0.555765 0.764787 0.449628 0.102 Uiso 1 1 calc R U . . .

C65 C 0.50635(18) 0.6698(5) 0.4409(3) 0.0719(14) Uani 1 1 d . . . . .

H65 H 0.488326 0.715824 0.459737 0.086 Uiso 1 1 calc R U . . .

C66 C 0.4132(4) 0.5955(9) 0.2257(5) 0.099(2) Uani 0.728(6) 1 d D U P C 1

H66A H 0.424273 0.658378 0.206455 0.119 Uiso 0.728(6) 1 calc R U P C 1

H66B H 0.390253 0.570225 0.202750 0.119 Uiso 0.728(6) 1 calc R U P C 1

C67 C 0.4447(5) 0.5112(9) 0.2306(6) 0.114(3) Uani 0.728(6) 1 d D U P C 1

H67A H 0.465813 0.534645 0.256929 0.170 Uiso 0.728(6) 1 calc R U P C 1

H67B H 0.455910 0.497219 0.191923 0.170 Uiso 0.728(6) 1 calc R U P C 1

H67C H 0.432606 0.447258 0.246021 0.170 Uiso 0.728(6) 1 calc R U P C 1

C68 C 0.4157(4) 0.9225(7) 0.2776(6) 0.093(2) Uani 0.728(6) 1 d D U P C 1

H68A H 0.415200 0.958032 0.315965 0.112 Uiso 0.728(6) 1 calc R U P C 1

H68B H 0.391038 0.939739 0.256101 0.112 Uiso 0.728(6) 1 calc R U P C 1

C69 C 0.4528(4) 0.9547(11) 0.2424(7) 0.116(3) Uani 0.728(6) 1 d D U P C 1

H69A H 0.476703 0.942134 0.265731 0.174 Uiso 0.728(6) 1 calc R U P C 1

H69B H 0.451075 1.029031 0.232487 0.174 Uiso 0.728(6) 1 calc R U P C 1

H69C H 0.454110 0.913340 0.206428 0.174 Uiso 0.728(6) 1 calc R U P C 1

O8 O 0.4011(2) 0.6198(5) 0.2858(3) 0.0888(14) Uani 0.728(6) 1 d D U P C 1

O9 O 0.42011(19) 0.8067(5) 0.2848(3) 0.0853(13) Uani 0.728(6) 1 d D U P C 1

P3 P 0.38224(6) 0.73771(15) 0.30067(9) 0.0835(4) Uani 0.728(6) 1 d D U P C 1

S3 S 0.33161(15) 0.7708(4) 0.2622(3) 0.1019(16) Uani 0.728(6) 1 d D U P C 1

C66A C 0.4173(8) 0.5659(11) 0.2593(13) 0.096(2) Uani 0.272(6) 1 d D U P C 2

H66C H 0.389470 0.549782 0.248445 0.116 Uiso 0.272(6) 1 calc R U P C 2

H66D H 0.424697 0.522758 0.293485 0.116 Uiso 0.272(6) 1 calc R U P C 2

C67A C 0.4449(12) 0.544(2) 0.2088(14) 0.109(5) Uani 0.272(6) 1 d D U P C 2

H67D H 0.453648 0.610713 0.191668 0.164 Uiso 0.272(6) 1 calc R U P C 2

H67E H 0.430930 0.503161 0.179215 0.164 Uiso 0.272(6) 1 calc R U P C 2

H67F H 0.468147 0.505424 0.222709 0.164 Uiso 0.272(6) 1 calc R U P C 2

C68A C 0.4145(8) 0.9123(18) 0.2534(10) 0.092(2) Uani 0.272(6) 1 d D U P C 2

H68C H 0.394230 0.965962 0.244356 0.111 Uiso 0.272(6) 1 calc R U P C 2

H68D H 0.417328 0.865059 0.219489 0.111 Uiso 0.272(6) 1 calc R U P C 2

C69A C 0.4547(9) 0.964(3) 0.2689(14) 0.098(4) Uani 0.272(6) 1 d D U P C 2

H69D H 0.462204 0.944356 0.308749 0.148 Uiso 0.272(6) 1 calc R U P C 2

H69E H 0.452212 1.039650 0.266013 0.148 Uiso 0.272(6) 1 calc R U P C 2

H69F H 0.475142 0.939193 0.241628 0.148 Uiso 0.272(6) 1 calc R U P C 2

O8A O 0.4216(3) 0.6778(10) 0.2730(7) 0.0896(17) Uani 0.272(6) 1 d D U P C 2

O9A O 0.4034(4) 0.8523(8) 0.3072(6) 0.0889(19) Uani 0.272(6) 1 d D U P C 2

P3A P 0.38224(6) 0.73771(15) 0.30067(9) 0.0835(4) Uani 0.272(6) 1 d D U P C 2

S3A S 0.3337(3) 0.7399(12) 0.2527(8) 0.090(3) Uani 0.272(6) 1 d D U P C 2

C70 C 0.67227(19) 0.3842(7) 0.4228(4) 0.090(2) Uani 1 1 d . . . . .

C71 C 0.6853(2) 0.4796(7) 0.4012(4) 0.095(2) Uani 1 1 d . . . . .

H71 H 0.667167 0.526434 0.383139 0.114 Uiso 1 1 calc R U . . .

C72 C 0.72518(19) 0.5052(5) 0.4066(3) 0.0758(16) Uani 1 1 d . . . . .

H72 H 0.734169 0.571007 0.392895 0.091 Uiso 1 1 calc R U . . .

C73 C 0.75332(15) 0.4342(4) 0.4326(2) 0.0600(12) Uani 1 1 d . . . . .

C74 C 0.73858(17) 0.3379(5) 0.4536(3) 0.0686(13) Uani 1 1 d . . . . .

H74 H 0.756442 0.289607 0.470895 0.082 Uiso 1 1 calc R U . . .

C75 C 0.69791(19) 0.3119(6) 0.4493(4) 0.0837(17) Uani 1 1 d . . . . .

H75 H 0.688179 0.247418 0.463913 0.100 Uiso 1 1 calc R U . . .

C76 C 0.79556(15) 0.4675(4) 0.4382(3) 0.0598(12) Uani 1 1 d . . . . .

H76 H 0.799492 0.539209 0.446907 0.072 Uiso 1 1 calc R U . . .

C77 C 0.83043(14) 0.2945(3) 0.4133(2) 0.0537(10) Uani 1 1 d . . . . .

H77A H 0.834619 0.249167 0.447792 0.064 Uiso 1 1 calc R U . . .

H77B H 0.804789 0.274972 0.395099 0.064 Uiso 1 1 calc R U . . .

C78 C 0.82917(13) 0.4102(4) 0.4325(2) 0.0513(10) Uani 1 1 d . . . . .

C79 C 0.86919(15) 0.4649(4) 0.4401(2) 0.0542(11) Uani 1 1 d . . . . .

C80 C 0.90697(14) 0.4087(3) 0.4221(2) 0.0506(10) Uani 1 1 d . . . . .

C81 C 0.90330(14) 0.2966(4) 0.3967(3) 0.0572(11) Uani 1 1 d . . . . .

H81A H 0.924022 0.286004 0.366623 0.069 Uiso 1 1 calc R U . . .

H81B H 0.907831 0.245032 0.428239 0.069 Uiso 1 1 calc R U . . .

C82 C 0.94177(15) 0.4629(4) 0.4284(3) 0.0604(12) Uani 1 1 d . . . . .

H82 H 0.938610 0.533169 0.440782 0.072 Uiso 1 1 calc R U . . .

C83 C 0.98378(15) 0.4301(4) 0.4190(3) 0.0598(11) Uani 1 1 d . . . . .

C84 C 0.99812(17) 0.3270(5) 0.4242(3) 0.0720(14) Uani 1 1 d . . . . .

H84 H 0.979924 0.272064 0.431986 0.086 Uiso 1 1 calc R U . . .

C85 C 1.03874(18) 0.3038(6) 0.4180(4) 0.0851(18) Uani 1 1 d . . . . .

H85 H 1.048091 0.234215 0.422323 0.102 Uiso 1 1 calc R U . . .

C86 C 1.06511(18) 0.3851(6) 0.4053(4) 0.0862(19) Uani 1 1 d . . . . .

C87 C 1.0527(2) 0.4878(6) 0.3989(4) 0.097(2) Uani 1 1 d . . . . .

H87 H 1.071241 0.541557 0.390129 0.116 Uiso 1 1 calc R U . . .

C88 C 1.01216(19) 0.5103(5) 0.4055(4) 0.0835(19) Uani 1 1 d . . . . .

H88 H 1.003256 0.580183 0.400988 0.100 Uiso 1 1 calc R U . . .

C89 C 0.8109(3) 0.4260(8) 0.2416(5) 0.0883(18) Uani 0.777(6) 1 d D U P D 1

H89A H 0.806058 0.364188 0.216628 0.106 Uiso 0.777(6) 1 calc R U P D 1

H89B H 0.828872 0.474059 0.220379 0.106 Uiso 0.777(6) 1 calc R U P D 1

C90 C 0.7727(4) 0.4794(9) 0.2540(6) 0.106(3) Uani 0.777(6) 1 d D U P D 1

H90A H 0.755862 0.433870 0.277998 0.159 Uiso 0.777(6) 1 calc R U P D 1

H90B H 0.759030 0.495004 0.217282 0.159 Uiso 0.777(6) 1 calc R U P D 1

H90C H 0.777846 0.544615 0.275111 0.159 Uiso 0.777(6) 1 calc R U P D 1

C91 C 0.8258(3) 0.0919(7) 0.2826(5) 0.0906(19) Uani 0.777(6) 1 d D U P D 1

H91A H 0.834189 0.065064 0.321153 0.109 Uiso 0.777(6) 1 calc R U P D 1

H91B H 0.848164 0.081412 0.255044 0.109 Uiso 0.777(6) 1 calc R U P D 1

C92 C 0.7902(4) 0.0298(8) 0.2615(7) 0.102(3) Uani 0.777(6) 1 d D U P D 1

H92A H 0.768591 0.035546 0.290128 0.154 Uiso 0.777(6) 1 calc R U P D 1

H92B H 0.797657 -0.043682 0.256993 0.154 Uiso 0.777(6) 1 calc R U P D 1

H92C H 0.781169 0.057532 0.223927 0.154 Uiso 0.777(6) 1 calc R U P D 1

O11 O 0.82891(17) 0.3943(4) 0.2951(3) 0.0796(12) Uani 0.777(6) 1 d D U P D 1

O12 O 0.81742(16) 0.2045(4) 0.2874(3) 0.0766(12) Uani 0.777(6) 1 d D U P D 1

P4 P 0.85330(5) 0.27980(15) 0.29987(8) 0.0775(4) Uani 0.777(6) 1 d D U P D 1

S4 S 0.90043(15) 0.2653(5) 0.2502(2) 0.0923(16) Uani 0.777(6) 1 d D U P D 1

C89A C 0.8035(7) 0.4288(14) 0.2645(17) 0.087(2) Uani 0.223(6) 1 d D U P D 2

H89C H 0.822826 0.449391 0.234072 0.104 Uiso 0.223(6) 1 calc R U P D 2

H89D H 0.808070 0.472681 0.299429 0.104 Uiso 0.223(6) 1 calc R U P D 2

C90A C 0.7624(8) 0.444(2) 0.243(2) 0.096(5) Uani 0.223(6) 1 d D U P D 2

H90D H 0.762376 0.496580 0.211878 0.145 Uiso 0.223(6) 1 calc R U P D 2

H90E H 0.745368 0.467370 0.275007 0.145 Uiso 0.223(6) 1 calc R U P D 2

H90F H 0.752232 0.377673 0.227438 0.145 Uiso 0.223(6) 1 calc R U P D 2

C91A C 0.8323(9) 0.0940(17) 0.2605(13) 0.087(2) Uani 0.223(6) 1 d D U P D 2

H91C H 0.848574 0.030043 0.264941 0.105 Uiso 0.223(6) 1 calc R U P D 2

H91D H 0.841885 0.131301 0.225339 0.105 Uiso 0.223(6) 1 calc R U P D 2

C92A C 0.7893(11) 0.063(3) 0.2510(19) 0.098(5) Uani 0.223(6) 1 d D U P D 2

H92D H 0.788078 0.006681 0.221850 0.147 Uiso 0.223(6) 1 calc R U P D 2

H92E H 0.774154 0.123017 0.237183 0.147 Uiso 0.223(6) 1 calc R U P D 2

H92F H 0.778021 0.037643 0.287859 0.147 Uiso 0.223(6) 1 calc R U P D 2

O11A O 0.8085(3) 0.3207(11) 0.2790(8) 0.0795(17) Uani 0.223(6) 1 d D U P D 2

O12A O 0.8386(5) 0.1611(7) 0.3117(7) 0.0868(19) Uani 0.223(6) 1 d D U P D 2

P4A P 0.85330(5) 0.27980(15) 0.29987(8) 0.0775(4) Uani 0.223(6) 1 d D U P D 2

S4A S 0.8964(4) 0.2866(15) 0.2422(7) 0.072(3) Uani 0.223(6) 1 d D U P D 2

N1 N 0.39409(13) 0.4740(3) 0.6315(2) 0.0600(10) Uani 1 1 d . . . . .

N2 N 0.86843(13) 0.5218(3) 0.6362(2) 0.0631(11) Uani 1 1 d . . . . .

N3 N 0.37763(14) 0.7294(3) 0.3730(2) 0.0632(11) Uani 1 1 d . . . . .

N4 N 0.86356(12) 0.2788(3) 0.3706(2) 0.0590(10) Uani 1 1 d . . . . .

O1 O 0.38370(12) 0.1952(3) 0.5460(2) 0.0724(11) Uani 1 1 d . . . . .

O4 O 0.86670(13) 0.8005(3) 0.5485(2) 0.0770(12) Uani 1 1 d . . . . .

O7 O 0.37754(13) 0.4441(3) 0.4547(2) 0.0737(11) Uani 1 1 d . . . . .

O10 O 0.87022(12) 0.5553(3) 0.4611(2) 0.0683(10) Uani 1 1 d . . . . .

F1 F 0.15253(12) 0.4173(5) 0.6058(3) 0.136(2) Uani 1 1 d . . . . .

F2 F 0.62528(12) 0.3580(5) 0.5623(4) 0.140(2) Uani 1 1 d . . . . .

F3 F 0.63277(12) 0.5836(5) 0.6041(4) 0.145(2) Uani 1 1 d . . . . .

F4 F 1.10559(13) 0.6240(5) 0.5875(4) 0.145(2) Uani 1 1 d . . . . .

F5 F 0.13922(13) 0.6338(4) 0.4338(4) 0.144(2) Uani 1 1 d . . . . .

F6 F 0.61153(13) 0.6602(4) 0.3973(3) 0.139(2) Uani 1 1 d . . . . .

F7 F 0.63238(12) 0.3597(5) 0.4186(3) 0.139(2) Uani 1 1 d . . . . .

F8 F 1.10506(11) 0.3629(4) 0.4005(3) 0.1262(18) Uani 1 1 d . . . . .

loop_

_atom_site_aniso_label

_atom_site_aniso_U_11

_atom_site_aniso_U_22

_atom_site_aniso_U_33

_atom_site_aniso_U_23

_atom_site_aniso_U_13

_atom_site_aniso_U_12

C1 0.047(3) 0.124(6) 0.101(5) -0.023(4) 0.001(3) -0.002(3)

C2 0.059(3) 0.112(5) 0.090(5) -0.010(4) 0.013(3) -0.018(3)

C3 0.057(3) 0.079(3) 0.081(4) 0.003(3) 0.009(3) -0.018(3)

C4 0.054(2) 0.065(3) 0.055(3) -0.003(2) 0.000(2) -0.007(2)

C5 0.058(3) 0.077(3) 0.083(4) 0.000(3) -0.008(3) -0.005(2)

C6 0.065(3) 0.084(4) 0.115(6) -0.014(4) -0.024(4) 0.011(3)

C7 0.055(2) 0.055(2) 0.060(3) 0.003(2) 0.000(2) -0.0044(19)

C8 0.049(2) 0.053(2) 0.072(3) -0.004(2) 0.001(2) 0.0029(19)

C9 0.051(2) 0.047(2) 0.054(3) 0.0004(19) -0.0004(19) -0.0024(17)

C10 0.056(3) 0.048(2) 0.060(3) 0.000(2) -0.002(2) 0.0013(19)

C11 0.053(2) 0.057(2) 0.056(3) -0.003(2) -0.001(2) -0.001(2)

C12 0.050(2) 0.059(2) 0.063(3) -0.010(2) 0.002(2) -0.0059(19)

C13 0.055(3) 0.063(3) 0.076(4) -0.010(3) 0.000(2) 0.007(2)

C14 0.053(3) 0.075(3) 0.076(4) -0.009(3) 0.000(2) 0.008(2)

C15 0.063(3) 0.080(4) 0.141(7) -0.001(4) -0.009(4) 0.017(3)

C16 0.063(4) 0.105(5) 0.148(8) -0.014(5) -0.021(4) 0.019(4)

C17 0.049(3) 0.119(5) 0.118(6) -0.014(5) -0.006(3) 0.000(3)

C18 0.066(4) 0.098(4) 0.098(5) 0.000(4) 0.005(3) -0.008(3)

C19 0.053(3) 0.083(3) 0.081(4) 0.006(3) 0.000(3) 0.006(3)

C20 0.086(4) 0.066(3) 0.069(4) -0.011(3) -0.001(4) 0.001(3)

C21 0.099(6) 0.071(5) 0.074(7) -0.013(6) -0.004(6) 0.012(5)

C22 0.092(4) 0.070(4) 0.076(4) -0.013(3) -0.006(3) -0.010(3)

C23 0.107(7) 0.096(7) 0.090(7) -0.008(6) -0.016(6) -0.014(6)

O2 0.073(3) 0.061(3) 0.062(3) -0.008(3) -0.006(3) -0.001(2)

O3 0.086(3) 0.060(2) 0.069(3) -0.016(2) -0.004(3) -0.011(2)

P1 0.0650(7) 0.0605(6) 0.0621(7) -0.0103(6) -0.0003(6) -0.0019(5)

S1 0.086(3) 0.082(4) 0.071(4) -0.009(3) 0.022(3) 0.008(2)

C20A 0.085(4) 0.069(3) 0.069(5) -0.008(4) 0.000(4) 0.000(3)

C21A 0.097(6) 0.081(6) 0.087(8) -0.007(6) 0.001(7) 0.014(5)

C22A 0.091(4) 0.072(4) 0.072(4) -0.010(3) -0.007(4) -0.011(3)

C23A 0.108(7) 0.073(6) 0.083(6) -0.007(6) -0.016(6) -0.016(6)

O2A 0.073(3) 0.062(3) 0.064(3) -0.003(3) -0.005(3) 0.001(2)

O3A 0.086(3) 0.061(3) 0.068(3) -0.015(3) -0.004(3) -0.009(2)

P1A 0.0650(7) 0.0605(6) 0.0621(7) -0.0103(6) -0.0003(6) -0.0019(5)

S1A 0.075(3) 0.097(5) 0.074(3) -0.003(3) 0.009(2) 0.014(3)

C24 0.052(3) 0.118(5) 0.111(6) -0.031(5) 0.001(3) 0.013(3)

C25 0.076(4) 0.099(5) 0.121(7) -0.030(5) 0.008(4) 0.029(4)

C26 0.071(4) 0.082(4) 0.100(5) -0.018(4) 0.001(4) 0.017(3)

C27 0.060(3) 0.073(3) 0.066(3) -0.004(3) -0.002(2) 0.013(2)

C28 0.058(3) 0.081(3) 0.076(4) -0.022(3) 0.000(3) 0.008(2)

C29 0.059(3) 0.097(4) 0.104(5) -0.034(4) -0.001(3) 0.004(3)

C30 0.066(3) 0.058(2) 0.069(3) -0.006(2) -0.005(3) 0.013(2)

C31 0.054(2) 0.058(2) 0.065(3) 0.003(2) -0.004(2) -0.003(2)

C32 0.060(3) 0.054(2) 0.053(3) -0.002(2) -0.004(2) 0.0004(19)

C33 0.062(3) 0.045(2) 0.062(3) -0.004(2) -0.003(2) 0.0018(19)

C34 0.060(3) 0.048(2) 0.057(3) -0.001(2) -0.003(2) -0.0038(19)

C35 0.053(2) 0.054(2) 0.081(4) 0.007(2) -0.003(3) 0.001(2)

C36 0.065(3) 0.052(2) 0.060(3) 0.002(2) -0.004(2) -0.010(2)

C37 0.064(3) 0.062(3) 0.063(3) 0.004(2) -0.005(2) -0.011(2)

C38 0.071(3) 0.072(3) 0.090(4) 0.002(3) 0.007(3) -0.010(3)

C39 0.067(4) 0.085(4) 0.131(7) 0.004(4) 0.010(4) 0.008(3)

C40 0.060(3) 0.120(6) 0.101(5) 0.014(5) -0.005(4) -0.008(4)

C41 0.073(4) 0.111(5) 0.110(6) 0.000(5) -0.024(4) -0.021(4)

C42 0.069(3) 0.079(3) 0.089(4) 0.001(3) -0.012(3) -0.016(3)

C43 0.112(4) 0.090(4) 0.088(4) 0.020(4) 0.003(4) -0.016(4)

C44 0.125(7) 0.089(6) 0.108(7) 0.020(6) 0.020(6) -0.022(6)

C45 0.107(4) 0.084(3) 0.079(4) 0.006(4) -0.010(4) -0.011(3)

C46 0.115(7) 0.095(6) 0.099(7) -0.002(6) -0.002(6) -0.012(6)

O5 0.107(3) 0.080(3) 0.078(3) 0.025(3) -0.002(3) -0.018(3)

O6 0.103(3) 0.086(3) 0.074(3) 0.003(3) -0.003(3) -0.006(3)

P2 0.0861(9) 0.0780(8) 0.0706(9) 0.0140(7) -0.0088(8) -0.0082(7)

S2 0.109(3) 0.100(4) 0.084(4) 0.004(3) -0.030(2) 0.001(2)

C43A 0.109(4) 0.085(4) 0.086(4) 0.018(4) 0.006(4) -0.016(4)

C44A 0.115(7) 0.097(7) 0.108(7) 0.011(7) 0.016(7) -0.016(7)

C45A 0.109(4) 0.088(4) 0.081(4) 0.011(4) -0.006(4) -0.013(4)

C46A 0.122(8) 0.098(6) 0.086(7) 0.003(7) -0.005(7) -0.015(7)

O5A 0.106(3) 0.079(3) 0.079(3) 0.024(3) -0.001(3) -0.017(3)

O6A 0.101(3) 0.083(3) 0.073(3) 0.008(3) -0.006(3) -0.009(3)

P2A 0.0861(9) 0.0780(8) 0.0706(9) 0.0140(7) -0.0088(8) -0.0082(7)

S2A 0.106(4) 0.101(5) 0.083(5) 0.009(4) -0.038(3) 0.003(3)

C47 0.060(3) 0.102(5) 0.129(7) 0.014(5) -0.007(4) -0.001(3)

C48 0.069(4) 0.085(4) 0.120(6) 0.000(4) 0.005(4) 0.002(3)

C49 0.066(3) 0.064(3) 0.096(4) -0.008(3) 0.003(3) -0.007(2)

C50 0.060(3) 0.063(3) 0.072(3) 0.000(3) -0.003(3) -0.010(2)

C51 0.067(3) 0.069(3) 0.117(6) -0.006(3) -0.006(4) -0.012(3)

C52 0.070(4) 0.092(5) 0.151(8) 0.005(5) -0.020(5) -0.024(3)

C53 0.061(3) 0.056(2) 0.072(3) -0.006(2) -0.005(3) -0.005(2)

C54 0.058(3) 0.053(2) 0.075(3) 0.004(2) -0.003(2) 0.001(2)

C55 0.059(3) 0.048(2) 0.060(3) -0.005(2) -0.004(2) -0.0007(19)

C56 0.061(3) 0.051(2) 0.058(3) 0.001(2) -0.003(2) -0.0004(19)

C57 0.056(2) 0.053(2) 0.053(3) -0.002(2) -0.001(2) 0.0013(19)

C58 0.051(2) 0.057(2) 0.064(3) 0.003(2) -0.001(2) 0.0008(19)

C59 0.063(3) 0.056(2) 0.064(3) 0.000(2) -0.001(2) 0.008(2)

C60 0.059(3) 0.064(3) 0.060(3) -0.001(2) -0.001(2) 0.009(2)

C61 0.067(3) 0.068(3) 0.087(4) -0.012(3) 0.004(3) 0.014(3)

C62 0.065(4) 0.094(4) 0.110(6) -0.020(4) 0.002(4) 0.021(3)

C63 0.060(3) 0.093(4) 0.109(6) -0.007(4) -0.006(3) 0.004(3)

C64 0.059(3) 0.088(4) 0.110(5) -0.020(4) -0.008(3) 0.005(3)

C65 0.067(3) 0.075(3) 0.074(4) -0.015(3) -0.002(3) 0.012(3)

C66 0.118(4) 0.097(4) 0.083(4) 0.004(3) 0.003(4) -0.003(3)

C67 0.130(6) 0.109(6) 0.101(7) 0.000(5) 0.024(6) -0.008(6)

C68 0.103(4) 0.088(3) 0.088(4) -0.004(3) 0.001(4) 0.002(3)

C69 0.129(6) 0.104(6) 0.116(7) 0.013(6) 0.018(6) 0.005(5)

O8 0.106(3) 0.088(3) 0.073(3) 0.009(2) 0.004(2) 0.002(2)

O9 0.092(3) 0.085(2) 0.079(3) 0.017(2) 0.005(2) 0.002(2)

P3 0.0889(10) 0.0944(10) 0.0673(9) 0.0177(8) -0.0089(8) 0.0014(8)

S3 0.116(2) 0.093(3) 0.097(3) 0.020(2) -0.045(2) -0.0078(18)

C66A 0.112(4) 0.095(4) 0.083(4) 0.009(4) 0.004(4) -0.002(4)

C67A 0.127(8) 0.108(8) 0.092(8) 0.007(8) 0.008(8) -0.001(8)

C68A 0.102(4) 0.089(4) 0.086(4) 0.006(4) 0.000(4) 0.001(4)

C69A 0.112(8) 0.095(7) 0.088(8) 0.001(8) 0.004(8) 0.000(7)

O8A 0.100(3) 0.092(3) 0.076(3) 0.009(3) 0.001(3) -0.002(3)

O9A 0.097(3) 0.092(3) 0.077(3) 0.012(3) -0.001(3) -0.001(3)

P3A 0.0889(10) 0.0944(10) 0.0673(9) 0.0177(8) -0.0089(8) 0.0014(8)

S3A 0.100(5) 0.092(6) 0.077(4) -0.003(5) -0.032(4) 0.008(4)

C70 0.053(3) 0.116(5) 0.102(5) -0.019(4) -0.011(3) 0.001(3)

C71 0.065(4) 0.119(5) 0.101(6) -0.014(5) -0.021(4) 0.026(4)

C72 0.065(3) 0.080(3) 0.082(4) -0.004(3) -0.005(3) 0.015(3)

C73 0.052(2) 0.068(3) 0.060(3) -0.009(2) -0.002(2) 0.011(2)

C74 0.059(3) 0.082(3) 0.064(3) -0.003(3) 0.004(2) 0.007(3)

C75 0.064(3) 0.097(4) 0.090(5) -0.010(4) 0.004(3) -0.004(3)

C76 0.056(3) 0.060(3) 0.064(3) -0.006(2) 0.000(2) 0.007(2)

C77 0.048(2) 0.052(2) 0.061(3) -0.004(2) -0.003(2) 0.0017(18)

C78 0.049(2) 0.054(2) 0.051(2) 0.002(2) -0.0010(19) 0.0017(18)

C79 0.057(3) 0.049(2) 0.057(3) 0.001(2) 0.004(2) -0.0020(18)

C80 0.049(2) 0.047(2) 0.055(3) 0.003(2) -0.0008(19) -0.0012(17)

C81 0.046(2) 0.055(2) 0.071(3) -0.006(2) 0.003(2) 0.0037(18)

C82 0.057(3) 0.057(2) 0.067(3) 0.000(2) 0.002(2) -0.007(2)

C83 0.052(2) 0.067(3) 0.061(3) -0.003(2) 0.003(2) -0.011(2)

C84 0.056(3) 0.077(3) 0.083(4) 0.010(3) -0.007(3) -0.007(2)

C85 0.058(3) 0.089(4) 0.108(5) 0.002(4) -0.006(3) 0.001(3)

C86 0.051(3) 0.110(5) 0.098(5) -0.014(4) 0.010(3) -0.005(3)

C87 0.065(4) 0.101(5) 0.125(7) -0.007(4) 0.024(4) -0.025(3)

C88 0.064(3) 0.073(3) 0.114(6) -0.007(3) 0.022(4) -0.015(3)

C89 0.095(3) 0.093(3) 0.077(3) -0.007(3) 0.003(3) 0.003(3)

C90 0.092(5) 0.130(6) 0.096(6) 0.003(6) -0.024(5) -0.002(5)

C91 0.087(3) 0.095(3) 0.091(4) 0.010(3) 0.004(3) 0.002(3)

C92 0.111(6) 0.087(6) 0.110(6) 0.006(5) -0.005(5) -0.017(5)

O11 0.087(3) 0.083(2) 0.068(2) -0.011(2) -0.003(2) 0.006(2)

O12 0.070(2) 0.086(2) 0.074(2) -0.016(2) -0.008(2) 0.000(2)

P4 0.0663(8) 0.1057(10) 0.0605(8) -0.0135(8) 0.0006(7) 0.0038(7)

S4 0.092(2) 0.111(3) 0.074(2) -0.0122(19) 0.028(2) 0.0022(17)

C89A 0.090(4) 0.094(4) 0.077(4) -0.009(4) 0.000(4) 0.003(4)

C90A 0.094(8) 0.109(8) 0.086(8) -0.005(8) -0.012(8) -0.007(8)

C91A 0.084(4) 0.093(4) 0.084(4) -0.002(4) 0.001(4) 0.002(4)

C92A 0.105(8) 0.089(8) 0.100(8) 0.003(8) -0.002(8) -0.004(8)

O11A 0.078(3) 0.091(3) 0.070(3) -0.010(3) -0.002(3) -0.001(3)

O12A 0.080(3) 0.105(3) 0.076(3) -0.009(3) -0.001(3) 0.005(3)

P4A 0.0663(8) 0.1057(10) 0.0605(8) -0.0135(8) 0.0006(7) 0.0038(7)

S4A 0.064(4) 0.080(5) 0.074(5) -0.005(4) -0.004(4) -0.002(4)

N1 0.051(2) 0.064(2) 0.066(3) -0.017(2) 0.0017(19) -0.0006(17)

N2 0.055(2) 0.062(2) 0.073(3) 0.016(2) -0.002(2) -0.0011(18)

N3 0.058(2) 0.063(2) 0.070(3) 0.010(2) -0.004(2) 0.0002(19)

N4 0.046(2) 0.065(2) 0.066(3) -0.014(2) 0.0047(18) 0.0012(17)

O1 0.068(2) 0.0497(18) 0.100(3) -0.0148(19) 0.003(2) -0.0008(15)

O4 0.084(3) 0.0467(18) 0.100(3) 0.015(2) -0.004(2) 0.0023(17)

O7 0.076(2) 0.0451(17) 0.100(3) 0.0073(19) -0.001(2) 0.0013(15)

O10 0.069(2) 0.0455(16) 0.090(3) -0.0072(18) 0.004(2) -0.0016(15)

F1 0.052(2) 0.171(4) 0.186(6) -0.038(4) 0.001(3) 0.013(2)

F2 0.048(2) 0.165(4) 0.208(7) -0.013(4) -0.004(3) -0.006(2)

F3 0.052(2) 0.159(4) 0.222(7) -0.064(5) 0.015(3) 0.002(2)

F4 0.061(2) 0.175(5) 0.200(7) 0.001(5) -0.009(3) 0.005(3)

F5 0.058(2) 0.140(4) 0.233(8) 0.013(5) -0.015(3) 0.007(2)

F6 0.057(2) 0.145(4) 0.214(7) -0.046(4) 0.010(3) -0.005(2)

F7 0.058(2) 0.177(5) 0.181(6) -0.018(4) -0.022(3) -0.006(3)

F8 0.0480(19) 0.151(4) 0.179(5) -0.007(4) 0.012(3) -0.002(2)

_geom_special_details

;

All esds (except the esd in the dihedral angle between two l.s. planes)

are estimated using the full covariance matrix. The cell esds are taken

into account individually in the estimation of esds in distances, angles

and torsion angles; correlations between esds in cell parameters are only

used when they are defined by crystal symmetry. An approximate (isotropic)

treatment of cell esds is used for estimating esds involving l.s. planes.

;

loop_

_geom_bond_atom_site_label_1

_geom_bond_atom_site_label_2

_geom_bond_distance

_geom_bond_site_symmetry_2

_geom_bond_publ_flag

C1 C2 1.333(11) . ?

C1 C6 1.360(11) . ?

C1 F1 1.371(7) . ?

C2 C3 1.388(9) . ?

C2 H2 0.9300 . ?

C3 C4 1.401(7) . ?

C3 H3 0.9300 . ?

C4 C5 1.368(8) . ?

C4 C7 1.460(7) . ?

C5 C6 1.396(8) . ?

C5 H5 0.9300 . ?

C6 H6 0.9300 . ?

C7 C9 1.346(7) . ?

C7 H7 0.9300 . ?

C8 N1 1.468(6) . ?

C8 C9 1.498(6) . ?

C8 H8A 0.9700 . ?

C8 H8B 0.9700 . ?

C9 C10 1.500(7) . ?

C10 O1 1.222(6) . ?

C10 C11 1.490(7) . ?

C11 C13 1.341(7) . ?

C11 C12 1.503(7) . ?

C12 N1 1.456(6) . ?

C12 H12A 0.9700 . ?

C12 H12B 0.9700 . ?

C13 C14 1.455(8) . ?

C13 H13 0.9300 . ?

C14 C19 1.394(8) . ?

C14 C15 1.397(9) . ?

C15 C16 1.371(10) . ?

C15 H15 0.9300 . ?

C16 C17 1.363(12) . ?

C16 H16 0.9300 . ?

C17 F2 1.349(7) . ?

C17 C18 1.374(10) . ?

C18 C19 1.375(8) . ?

C18 H18 0.9300 . ?

C19 H19 0.9300 . ?

C20 O2 1.434(8) . ?

C20 C21 1.507(11) . ?

C20 H20A 0.9700 . ?

C20 H20B 0.9700 . ?

C21 H21A 0.9600 . ?

C21 H21B 0.9600 . ?

C21 H21C 0.9600 . ?

C22 O3 1.449(11) . ?

C22 C23 1.459(13) . ?

C22 H22A 0.9700 . ?

C22 H22B 0.9700 . ?

C23 H23A 0.9600 . ?

C23 H23B 0.9600 . ?

C23 H23C 0.9600 . ?

O2 P1 1.577(6) . ?

O3 P1 1.587(5) . ?

P1 N1 1.630(5) . ?

P1 S1 1.929(4) . ?

C20A O2A 1.434(8) . ?

C20A C21A 1.507(11) . ?

C20A H20C 0.9700 . ?

C20A H20D 0.9700 . ?

C21A H21D 0.9600 . ?

C21A H21E 0.9600 . ?

C21A H21F 0.9600 . ?

C22A O3A 1.449(11) . ?

C22A C23A 1.456(13) . ?

C22A H22C 0.9700 . ?

C22A H22D 0.9700 . ?

C23A H23D 0.9600 . ?

C23A H23E 0.9600 . ?

C23A H23F 0.9600 . ?

O2A P1A 1.583(5) . ?

O3A P1A 1.587(5) . ?

P1A N1 1.630(5) . ?

P1A S1A 1.933(5) . ?

C24 C25 1.350(11) . ?

C24 F3 1.358(8) . ?

C24 C29 1.364(9) . ?

C25 C26 1.365(10) . ?

C25 H25 0.9300 . ?

C26 C27 1.394(8) . ?

C26 H26 0.9300 . ?

C27 C28 1.389(8) . ?

C27 C30 1.462(8) . ?

C28 C29 1.378(8) . ?

C28 H28 0.9300 . ?

C29 H29 0.9300 . ?

C30 C32 1.348(7) . ?

C30 H30 0.9300 . ?

C31 N2 1.470(7) . ?

C31 C32 1.504(7) . ?

C31 H31A 0.9700 . ?

C31 H31B 0.9700 . ?

C32 C33 1.495(7) . ?

C33 O4 1.224(6) . ?

C33 C34 1.491(7) . ?

C34 C36 1.346(7) . ?

C34 C35 1.505(7) . ?

C35 N2 1.457(7) . ?

C35 H35A 0.9700 . ?

C35 H35B 0.9700 . ?

C36 C37 1.458(8) . ?

C36 H36 0.9300 . ?

C37 C38 1.380(8) . ?

C37 C42 1.399(8) . ?

C38 C39 1.377(9) . ?

C38 H38 0.9300 . ?

C39 C40 1.377(11) . ?

C39 H39 0.9300 . ?

C40 C41 1.323(12) . ?

C40 F4 1.372(8) . ?

C41 C42 1.387(10) . ?

C41 H41 0.9300 . ?

C42 H42 0.9300 . ?

C43 O5 1.429(12) . ?

C43 C44 1.437(14) . ?

C43 H43A 0.9700 . ?

C43 H43B 0.9700 . ?

C44 H44A 0.9600 . ?

C44 H44B 0.9600 . ?

C44 H44C 0.9600 . ?

C45 O6 1.472(10) . ?

C45 C46 1.575(15) . ?

C45 H45A 0.9700 . ?

C45 H45B 0.9700 . ?

C46 H46A 0.9600 . ?

C46 H46B 0.9600 . ?

C46 H46C 0.9600 . ?

O5 P2 1.604(6) . ?

O6 P2 1.585(7) . ?

P2 N2 1.618(6) . ?

P2 S2 1.934(4) . ?

C43A O5A 1.427(13) . ?

C43A C44A 1.440(14) . ?

C43A H43C 0.9700 . ?

C43A H43D 0.9700 . ?

C44A H44D 0.9600 . ?

C44A H44E 0.9600 . ?

C44A H44F 0.9600 . ?

C45A O6A 1.473(10) . ?

C45A C46A 1.571(15) . ?

C45A H45C 0.9700 . ?

C45A H45D 0.9700 . ?

C46A H46D 0.9600 . ?

C46A H46E 0.9600 . ?

C46A H46F 0.9600 . ?

O5A P2A 1.609(6) . ?

O6A P2A 1.570(7) . ?

P2A N2 1.618(6) . ?

P2A S2A 1.933(5) . ?

C47 C52 1.337(11) . ?

C47 F5 1.360(8) . ?

C47 C48 1.388(10) . ?

C48 C49 1.381(9) . ?

C48 H48 0.9300 . ?

C49 C50 1.382(8) . ?

C49 H49 0.9300 . ?

C50 C51 1.404(8) . ?

C50 C53 1.459(8) . ?

C51 C52 1.375(10) . ?

C51 H51 0.9300 . ?

C52 H52 0.9300 . ?

C53 C55 1.342(7) . ?

C53 H53 0.9300 . ?

C54 N3 1.455(7) . ?

C54 C55 1.518(7) . ?

C54 H54A 0.9700 . ?

C54 H54B 0.9700 . ?

C55 C56 1.490(7) . ?

C56 O7 1.220(6) . ?

C56 C57 1.492(7) . ?

C57 C59 1.339(7) . ?

C57 C58 1.513(7) . ?

C58 N3 1.452(7) . ?

C58 H58A 0.9700 . ?

C58 H58B 0.9700 . ?

C59 C60 1.448(8) . ?

C59 H59 0.9300 . ?

C60 C65 1.392(8) . ?

C60 C61 1.400(7) . ?

C61 C62 1.363(9) . ?

C61 H61 0.9300 . ?

C62 C63 1.358(10) . ?

C62 H62 0.9300 . ?

C63 F6 1.354(8) . ?

C63 C64 1.387(10) . ?

C64 C65 1.382(9) . ?

C64 H64 0.9300 . ?

C65 H65 0.9300 . ?

C66 O8 1.439(11) . ?

C66 C67 1.483(14) . ?

C66 H66A 0.9700 . ?

C66 H66B 0.9700 . ?

C67 H67A 0.9600 . ?

C67 H67B 0.9600 . ?

C67 H67C 0.9600 . ?

C68 O9 1.469(11) . ?

C68 C69 1.509(14) . ?

C68 H68A 0.9700 . ?

C68 H68B 0.9700 . ?

C69 H69A 0.9600 . ?

C69 H69B 0.9600 . ?

C69 H69C 0.9600 . ?

O8 P3 1.639(6) . ?

O9 P3 1.559(6) . ?

P3 N3 1.636(5) . ?

P3 S3 1.924(3) . ?

C66A O8A 1.444(13) . ?

C66A C67A 1.480(15) . ?

C66A H66C 0.9700 . ?

C66A H66D 0.9700 . ?

C67A H67D 0.9600 . ?

C67A H67E 0.9600 . ?

C67A H67F 0.9600 . ?

C68A O9A 1.468(12) . ?

C68A C69A 1.511(15) . ?

C68A H68C 0.9700 . ?

C68A H68D 0.9700 . ?

C69A H69D 0.9600 . ?

C69A H69E 0.9600 . ?

C69A H69F 0.9600 . ?

O8A P3A 1.624(8) . ?

O9A P3A 1.605(8) . ?

P3A N3 1.636(5) . ?

P3A S3A 1.927(6) . ?

C70 F7 1.353(7) . ?

C70 C71 1.360(11) . ?

C70 C75 1.374(10) . ?

C71 C72 1.359(10) . ?

C71 H71 0.9300 . ?

C72 C73 1.411(8) . ?

C72 H72 0.9300 . ?

C73 C74 1.384(8) . ?

C73 C76 1.458(7) . ?

C74 C75 1.382(8) . ?

C74 H74 0.9300 . ?

C75 H75 0.9300 . ?

C76 C78 1.326(7) . ?

C76 H76 0.9300 . ?

C77 N4 1.466(6) . ?

C77 C78 1.515(6) . ?

C77 H77A 0.9700 . ?

C77 H77B 0.9700 . ?

C78 C79 1.496(7) . ?

C79 O10 1.228(6) . ?

C79 C80 1.487(7) . ?

C80 C82 1.340(7) . ?

C80 C81 1.521(6) . ?

C81 N4 1.452(6) . ?

C81 H81A 0.9700 . ?

C81 H81B 0.9700 . ?

C82 C83 1.459(7) . ?

C82 H82 0.9300 . ?

C83 C84 1.381(8) . ?

C83 C88 1.406(7) . ?

C84 C85 1.376(8) . ?

C84 H84 0.9300 . ?

C85 C86 1.370(10) . ?

C85 H85 0.9300 . ?

C86 F8 1.349(7) . ?

C86 C87 1.359(11) . ?

C87 C88 1.374(10) . ?

C87 H87 0.9300 . ?

C88 H88 0.9300 . ?

C89 O11 1.398(11) . ?

C89 C90 1.453(13) . ?

C89 H89A 0.9700 . ?

C89 H89B 0.9700 . ?

C90 H90A 0.9600 . ?

C90 H90B 0.9600 . ?

C90 H90C 0.9600 . ?

C91 O12 1.443(10) . ?

C91 C92 1.486(12) . ?

C91 H91A 0.9700 . ?

C91 H91B 0.9700 . ?

C92 H92A 0.9600 . ?

C92 H92B 0.9600 . ?

C92 H92C 0.9600 . ?

O11 P4 1.649(6) . ?

O12 P4 1.538(5) . ?

P4 N4 1.624(5) . ?

P4 S4 1.920(3) . ?

C89A O11A 1.404(12) . ?

C89A C90A 1.450(14) . ?

C89A H89C 0.9700 . ?

C89A H89D 0.9700 . ?

C90A H90D 0.9600 . ?

C90A H90E 0.9600 . ?

C90A H90F 0.9600 . ?

C91A O12A 1.440(12) . ?

C91A C92A 1.485(13) . ?

C91A H91C 0.9700 . ?

C91A H91D 0.9700 . ?

C92A H92D 0.9600 . ?

C92A H92E 0.9600 . ?

C92A H92F 0.9600 . ?

O11A P4A 1.631(8) . ?

O12A P4A 1.587(8) . ?

P4A N4 1.624(5) . ?

P4A S4A 1.924(6) . ?

loop_

_geom_angle_atom_site_label_1

_geom_angle_atom_site_label_2

_geom_angle_atom_site_label_3

_geom_angle

_geom_angle_site_symmetry_1

_geom_angle_site_symmetry_3

_geom_angle_publ_flag

C2 C1 C6 124.0(6) . . ?

C2 C1 F1 117.5(7) . . ?

C6 C1 F1 118.4(7) . . ?

C1 C2 C3 117.7(6) . . ?

C1 C2 H2 121.2 . . ?

C3 C2 H2 121.2 . . ?

C2 C3 C4 121.5(6) . . ?

C2 C3 H3 119.3 . . ?

C4 C3 H3 119.3 . . ?

C5 C4 C3 117.9(5) . . ?

C5 C4 C7 123.9(5) . . ?

C3 C4 C7 118.0(5) . . ?

C4 C5 C6 120.9(6) . . ?

C4 C5 H5 119.6 . . ?

C6 C5 H5 119.6 . . ?

C1 C6 C5 118.1(6) . . ?

C1 C6 H6 121.0 . . ?

C5 C6 H6 121.0 . . ?

C9 C7 C4 129.3(4) . . ?

C9 C7 H7 115.3 . . ?

C4 C7 H7 115.3 . . ?

N1 C8 C9 110.0(4) . . ?

N1 C8 H8A 109.7 . . ?

C9 C8 H8A 109.7 . . ?

N1 C8 H8B 109.7 . . ?

C9 C8 H8B 109.7 . . ?

H8A C8 H8B 108.2 . . ?

C7 C9 C8 125.6(4) . . ?

C7 C9 C10 115.9(4) . . ?

C8 C9 C10 118.5(4) . . ?

O1 C10 C11 120.7(5) . . ?

O1 C10 C9 120.7(4) . . ?

C11 C10 C9 118.5(4) . . ?

C13 C11 C10 116.8(4) . . ?

C13 C11 C12 125.3(5) . . ?

C10 C11 C12 117.7(4) . . ?

N1 C12 C11 109.4(4) . . ?

N1 C12 H12A 109.8 . . ?

C11 C12 H12A 109.8 . . ?

N1 C12 H12B 109.8 . . ?

C11 C12 H12B 109.8 . . ?

H12A C12 H12B 108.2 . . ?

C11 C13 C14 129.4(5) . . ?

C11 C13 H13 115.3 . . ?

C14 C13 H13 115.3 . . ?

C19 C14 C15 117.8(6) . . ?

C19 C14 C13 124.2(5) . . ?

C15 C14 C13 117.9(6) . . ?

C16 C15 C14 121.4(7) . . ?

C16 C15 H15 119.3 . . ?

C14 C15 H15 119.3 . . ?

C17 C16 C15 118.8(7) . . ?

C17 C16 H16 120.6 . . ?

C15 C16 H16 120.6 . . ?

F2 C17 C16 119.2(7) . . ?

F2 C17 C18 118.7(8) . . ?

C16 C17 C18 122.1(6) . . ?

C17 C18 C19 118.9(7) . . ?

C17 C18 H18 120.6 . . ?

C19 C18 H18 120.6 . . ?

C18 C19 C14 121.0(6) . . ?

C18 C19 H19 119.5 . . ?

C14 C19 H19 119.5 . . ?

O2 C20 C21 108.0(8) . . ?

O2 C20 H20A 110.1 . . ?

C21 C20 H20A 110.1 . . ?

O2 C20 H20B 110.1 . . ?

C21 C20 H20B 110.1 . . ?

H20A C20 H20B 108.4 . . ?

C20 C21 H21A 109.5 . . ?

C20 C21 H21B 109.5 . . ?

H21A C21 H21B 109.5 . . ?

C20 C21 H21C 109.5 . . ?

H21A C21 H21C 109.5 . . ?

H21B C21 H21C 109.5 . . ?

O3 C22 C23 109.3(9) . . ?

O3 C22 H22A 109.8 . . ?

C23 C22 H22A 109.8 . . ?

O3 C22 H22B 109.8 . . ?

C23 C22 H22B 109.8 . . ?

H22A C22 H22B 108.3 . . ?

C22 C23 H23A 109.5 . . ?

C22 C23 H23B 109.5 . . ?

H23A C23 H23B 109.5 . . ?

C22 C23 H23C 109.5 . . ?

H23A C23 H23C 109.5 . . ?

H23B C23 H23C 109.5 . . ?

C20 O2 P1 121.6(7) . . ?

C22 O3 P1 121.9(8) . . ?

O2 P1 O3 99.8(4) . . ?

O2 P1 N1 111.4(5) . . ?

O3 P1 N1 99.1(4) . . ?

O2 P1 S1 116.1(4) . . ?

O3 P1 S1 116.6(4) . . ?

N1 P1 S1 112.1(5) . . ?

O2A C20A C21A 107.6(8) . . ?

O2A C20A H20C 110.2 . . ?

C21A C20A H20C 110.2 . . ?

O2A C20A H20D 110.2 . . ?

C21A C20A H20D 110.2 . . ?

H20C C20A H20D 108.5 . . ?

C20A C21A H21D 109.5 . . ?

C20A C21A H21E 109.5 . . ?

H21D C21A H21E 109.5 . . ?

C20A C21A H21F 109.5 . . ?

H21D C21A H21F 109.5 . . ?

H21E C21A H21F 109.5 . . ?

O3A C22A C23A 110.0(9) . . ?

O3A C22A H22C 109.7 . . ?

C23A C22A H22C 109.7 . . ?

O3A C22A H22D 109.7 . . ?

C23A C22A H22D 109.7 . . ?

H22C C22A H22D 108.2 . . ?

C22A C23A H23D 109.5 . . ?

C22A C23A H23E 109.5 . . ?

H23D C23A H23E 109.5 . . ?

C22A C23A H23F 109.5 . . ?

H23D C23A H23F 109.5 . . ?

H23E C23A H23F 109.5 . . ?

C20A O2A P1A 120.8(7) . . ?

C22A O3A P1A 120.6(7) . . ?

O2A P1A O3A 99.1(4) . . ?

O2A P1A N1 100.2(5) . . ?

O3A P1A N1 108.3(4) . . ?

O2A P1A S1A 115.1(4) . . ?

O3A P1A S1A 116.2(5) . . ?

N1 P1A S1A 115.6(5) . . ?

C25 C24 F3 118.9(6) . . ?

C25 C24 C29 122.5(6) . . ?

F3 C24 C29 118.6(7) . . ?

C24 C25 C26 119.2(6) . . ?

C24 C25 H25 120.4 . . ?

C26 C25 H25 120.4 . . ?

C25 C26 C27 120.9(6) . . ?

C25 C26 H26 119.5 . . ?

C27 C26 H26 119.5 . . ?

C28 C27 C26 118.0(6) . . ?

C28 C27 C30 123.0(5) . . ?

C26 C27 C30 118.9(5) . . ?

C29 C28 C27 120.9(5) . . ?

C29 C28 H28 119.6 . . ?

C27 C28 H28 119.6 . . ?

C24 C29 C28 118.5(6) . . ?

C24 C29 H29 120.8 . . ?

C28 C29 H29 120.8 . . ?

C32 C30 C27 129.3(5) . . ?

C32 C30 H30 115.3 . . ?

C27 C30 H30 115.3 . . ?

N2 C31 C32 109.0(4) . . ?

N2 C31 H31A 109.9 . . ?

C32 C31 H31A 109.9 . . ?

N2 C31 H31B 109.9 . . ?

C32 C31 H31B 109.9 . . ?

H31A C31 H31B 108.3 . . ?

C30 C32 C33 117.7(4) . . ?

C30 C32 C31 124.3(5) . . ?

C33 C32 C31 117.8(4) . . ?

O4 C33 C34 120.8(5) . . ?

O4 C33 C32 120.2(5) . . ?

C34 C33 C32 118.8(4) . . ?

C36 C34 C33 116.3(4) . . ?

C36 C34 C35 125.5(5) . . ?

C33 C34 C35 118.1(4) . . ?

N2 C35 C34 110.8(4) . . ?

N2 C35 H35A 109.5 . . ?

C34 C35 H35A 109.5 . . ?

N2 C35 H35B 109.5 . . ?

C34 C35 H35B 109.5 . . ?

H35A C35 H35B 108.1 . . ?

C34 C36 C37 129.8(5) . . ?

C34 C36 H36 115.1 . . ?

C37 C36 H36 115.1 . . ?

C38 C37 C42 117.7(6) . . ?

C38 C37 C36 124.5(5) . . ?

C42 C37 C36 117.6(5) . . ?

C39 C38 C37 121.0(6) . . ?

C39 C38 H38 119.5 . . ?

C37 C38 H38 119.5 . . ?

C38 C39 C40 118.1(7) . . ?

C38 C39 H39 121.0 . . ?

C40 C39 H39 121.0 . . ?

C41 C40 F4 119.2(7) . . ?

C41 C40 C39 123.7(7) . . ?

F4 C40 C39 117.0(8) . . ?

C40 C41 C42 118.0(7) . . ?

C40 C41 H41 121.0 . . ?

C42 C41 H41 121.0 . . ?

C41 C42 C37 121.3(7) . . ?

C41 C42 H42 119.3 . . ?

C37 C42 H42 119.3 . . ?

O5 C43 C44 109.8(10) . . ?

O5 C43 H43A 109.7 . . ?

C44 C43 H43A 109.7 . . ?

O5 C43 H43B 109.7 . . ?

C44 C43 H43B 109.7 . . ?

H43A C43 H43B 108.2 . . ?

C43 C44 H44A 109.5 . . ?

C43 C44 H44B 109.5 . . ?

H44A C44 H44B 109.5 . . ?

C43 C44 H44C 109.5 . . ?

H44A C44 H44C 109.5 . . ?

H44B C44 H44C 109.5 . . ?

O6 C45 C46 101.4(8) . . ?

O6 C45 H45A 111.5 . . ?

C46 C45 H45A 111.5 . . ?

O6 C45 H45B 111.5 . . ?

C46 C45 H45B 111.5 . . ?

H45A C45 H45B 109.3 . . ?

C45 C46 H46A 109.5 . . ?

C45 C46 H46B 109.5 . . ?

H46A C46 H46B 109.5 . . ?

C45 C46 H46C 109.5 . . ?

H46A C46 H46C 109.5 . . ?

H46B C46 H46C 109.5 . . ?

C43 O5 P2 121.5(7) . . ?

C45 O6 P2 115.2(7) . . ?

O6 P2 O5 98.2(4) . . ?

O6 P2 N2 101.3(4) . . ?

O5 P2 N2 106.8(4) . . ?

O6 P2 S2 115.7(4) . . ?

O5 P2 S2 115.4(4) . . ?

N2 P2 S2 116.9(4) . . ?

O5A C43A C44A 109.6(11) . . ?

O5A C43A H43C 109.8 . . ?

C44A C43A H43C 109.8 . . ?

O5A C43A H43D 109.8 . . ?

C44A C43A H43D 109.8 . . ?

H43C C43A H43D 108.2 . . ?

C43A C44A H44D 109.5 . . ?

C43A C44A H44E 109.5 . . ?

H44D C44A H44E 109.5 . . ?

C43A C44A H44F 109.5 . . ?

H44D C44A H44F 109.5 . . ?

H44E C44A H44F 109.5 . . ?

O6A C45A C46A 101.2(9) . . ?

O6A C45A H45C 111.5 . . ?

C46A C45A H45C 111.5 . . ?

O6A C45A H45D 111.5 . . ?

C46A C45A H45D 111.5 . . ?

H45C C45A H45D 109.4 . . ?

C45A C46A H46D 109.5 . . ?

C45A C46A H46E 109.5 . . ?

H46D C46A H46E 109.5 . . ?

C45A C46A H46F 109.5 . . ?

H46D C46A H46F 109.5 . . ?

H46E C46A H46F 109.5 . . ?

C43A O5A P2A 123.3(10) . . ?

C45A O6A P2A 114.3(8) . . ?

O6A P2A O5A 98.8(5) . . ?

O6A P2A N2 116.7(5) . . ?

O5A P2A N2 95.7(5) . . ?

O6A P2A S2A 117.8(5) . . ?

O5A P2A S2A 115.0(5) . . ?

N2 P2A S2A 110.2(5) . . ?

C52 C47 F5 119.3(7) . . ?

C52 C47 C48 123.1(6) . . ?

F5 C47 C48 117.6(7) . . ?

C49 C48 C47 117.2(6) . . ?

C49 C48 H48 121.4 . . ?

C47 C48 H48 121.4 . . ?

C48 C49 C50 121.8(6) . . ?

C48 C49 H49 119.1 . . ?

C50 C49 H49 119.1 . . ?

C49 C50 C51 117.9(5) . . ?

C49 C50 C53 124.6(5) . . ?

C51 C50 C53 117.4(5) . . ?

C52 C51 C50 120.7(6) . . ?

C52 C51 H51 119.6 . . ?

C50 C51 H51 119.6 . . ?

C47 C52 C51 119.2(6) . . ?

C47 C52 H52 120.4 . . ?

C51 C52 H52 120.4 . . ?

C55 C53 C50 130.8(5) . . ?

C55 C53 H53 114.6 . . ?

C50 C53 H53 114.6 . . ?

N3 C54 C55 110.9(4) . . ?

N3 C54 H54A 109.5 . . ?

C55 C54 H54A 109.5 . . ?

N3 C54 H54B 109.5 . . ?

C55 C54 H54B 109.5 . . ?

H54A C54 H54B 108.0 . . ?

C53 C55 C56 116.4(4) . . ?

C53 C55 C54 125.0(5) . . ?

C56 C55 C54 118.6(4) . . ?

O7 C56 C55 121.7(5) . . ?

O7 C56 C57 119.8(5) . . ?

C55 C56 C57 118.4(4) . . ?

C59 C57 C56 118.4(4) . . ?

C59 C57 C58 123.9(5) . . ?

C56 C57 C58 117.5(4) . . ?

N3 C58 C57 109.9(4) . . ?

N3 C58 H58A 109.7 . . ?

C57 C58 H58A 109.7 . . ?

N3 C58 H58B 109.7 . . ?

C57 C58 H58B 109.7 . . ?

H58A C58 H58B 108.2 . . ?

C57 C59 C60 130.5(5) . . ?

C57 C59 H59 114.7 . . ?

C60 C59 H59 114.7 . . ?

C65 C60 C61 117.1(5) . . ?

C65 C60 C59 122.8(5) . . ?

C61 C60 C59 120.1(5) . . ?

C62 C61 C60 122.0(6) . . ?

C62 C61 H61 119.0 . . ?

C60 C61 H61 119.0 . . ?

C63 C62 C61 118.8(6) . . ?

C63 C62 H62 120.6 . . ?

C61 C62 H62 120.6 . . ?

F6 C63 C62 119.0(6) . . ?

F6 C63 C64 118.4(6) . . ?

C62 C63 C64 122.5(6) . . ?

C65 C64 C63 117.6(6) . . ?

C65 C64 H64 121.2 . . ?

C63 C64 H64 121.2 . . ?

C64 C65 C60 121.8(5) . . ?

C64 C65 H65 119.1 . . ?

C60 C65 H65 119.1 . . ?

O8 C66 C67 106.0(9) . . ?

O8 C66 H66A 110.5 . . ?

C67 C66 H66A 110.5 . . ?

O8 C66 H66B 110.5 . . ?

C67 C66 H66B 110.5 . . ?

H66A C66 H66B 108.7 . . ?

C66 C67 H67A 109.5 . . ?

C66 C67 H67B 109.5 . . ?

H67A C67 H67B 109.5 . . ?

C66 C67 H67C 109.5 . . ?

H67A C67 H67C 109.5 . . ?

H67B C67 H67C 109.5 . . ?

O9 C68 C69 104.0(9) . . ?

O9 C68 H68A 110.9 . . ?

C69 C68 H68A 110.9 . . ?

O9 C68 H68B 110.9 . . ?

C69 C68 H68B 110.9 . . ?

H68A C68 H68B 109.0 . . ?

C68 C69 H69A 109.5 . . ?

C68 C69 H69B 109.5 . . ?

H69A C69 H69B 109.5 . . ?

C68 C69 H69C 109.5 . . ?

H69A C69 H69C 109.5 . . ?

H69B C69 H69C 109.5 . . ?

C66 O8 P3 119.1(6) . . ?

C68 O9 P3 119.7(6) . . ?

O9 P3 N3 109.6(3) . . ?

O9 P3 O8 98.7(4) . . ?

N3 P3 O8 100.4(3) . . ?

O9 P3 S3 118.1(3) . . ?

N3 P3 S3 112.3(3) . . ?

O8 P3 S3 115.6(3) . . ?

O8A C66A C67A 106.2(11) . . ?

O8A C66A H66C 110.5 . . ?

C67A C66A H66C 110.5 . . ?

O8A C66A H66D 110.5 . . ?

C67A C66A H66D 110.5 . . ?

H66C C66A H66D 108.7 . . ?

C66A C67A H67D 109.5 . . ?

C66A C67A H67E 109.5 . . ?

H67D C67A H67E 109.5 . . ?

C66A C67A H67F 109.5 . . ?

H67D C67A H67F 109.5 . . ?

H67E C67A H67F 109.5 . . ?

O9A C68A C69A 104.3(12) . . ?

O9A C68A H68C 110.9 . . ?

C69A C68A H68C 110.9 . . ?

O9A C68A H68D 110.9 . . ?

C69A C68A H68D 110.9 . . ?

H68C C68A H68D 108.9 . . ?

C68A C69A H69D 109.5 . . ?

C68A C69A H69E 109.5 . . ?

H69D C69A H69E 109.5 . . ?

C68A C69A H69F 109.5 . . ?

H69D C69A H69F 109.5 . . ?

H69E C69A H69F 109.5 . . ?

C66A O8A P3A 116.8(10) . . ?

C68A O9A P3A 119.5(10) . . ?

O9A P3A O8A 95.8(6) . . ?

O9A P3A N3 90.4(5) . . ?

O8A P3A N3 115.2(6) . . ?

O9A P3A S3A 113.5(6) . . ?

O8A P3A S3A 117.1(6) . . ?

N3 P3A S3A 118.7(6) . . ?

F7 C70 C71 118.7(7) . . ?

F7 C70 C75 118.5(7) . . ?

C71 C70 C75 122.8(6) . . ?

C72 C71 C70 118.7(7) . . ?

C72 C71 H71 120.7 . . ?

C70 C71 H71 120.7 . . ?

C71 C72 C73 121.6(7) . . ?

C71 C72 H72 119.2 . . ?

C73 C72 H72 119.2 . . ?

C74 C73 C72 117.4(5) . . ?

C74 C73 C76 123.7(5) . . ?

C72 C73 C76 118.8(5) . . ?

C75 C74 C73 121.5(6) . . ?

C75 C74 H74 119.3 . . ?

C73 C74 H74 119.3 . . ?

C70 C75 C74 118.0(7) . . ?

C70 C75 H75 121.0 . . ?

C74 C75 H75 121.0 . . ?

C78 C76 C73 129.4(5) . . ?

C78 C76 H76 115.3 . . ?

C73 C76 H76 115.3 . . ?

N4 C77 C78 109.6(4) . . ?

N4 C77 H77A 109.8 . . ?

C78 C77 H77A 109.8 . . ?

N4 C77 H77B 109.8 . . ?

C78 C77 H77B 109.8 . . ?

H77A C77 H77B 108.2 . . ?

C76 C78 C79 118.5(4) . . ?

C76 C78 C77 124.7(4) . . ?

C79 C78 C77 116.6(4) . . ?

O10 C79 C80 121.3(4) . . ?

O10 C79 C78 119.4(4) . . ?

C80 C79 C78 119.3(4) . . ?

C82 C80 C79 116.5(4) . . ?

C82 C80 C81 125.2(4) . . ?

C79 C80 C81 118.3(4) . . ?

N4 C81 C80 111.4(4) . . ?

N4 C81 H81A 109.4 . . ?

C80 C81 H81A 109.4 . . ?

N4 C81 H81B 109.4 . . ?

C80 C81 H81B 109.4 . . ?

H81A C81 H81B 108.0 . . ?

C80 C82 C83 130.7(5) . . ?

C80 C82 H82 114.6 . . ?

C83 C82 H82 114.6 . . ?

C84 C83 C88 117.4(5) . . ?

C84 C83 C82 125.2(5) . . ?

C88 C83 C82 117.4(5) . . ?

C85 C84 C83 121.5(6) . . ?

C85 C84 H84 119.3 . . ?

C83 C84 H84 119.3 . . ?

C86 C85 C84 118.6(6) . . ?

C86 C85 H85 120.7 . . ?

C84 C85 H85 120.7 . . ?

F8 C86 C87 118.7(6) . . ?

F8 C86 C85 118.8(7) . . ?

C87 C86 C85 122.5(6) . . ?

C86 C87 C88 118.4(6) . . ?

C86 C87 H87 120.8 . . ?

C88 C87 H87 120.8 . . ?

C87 C88 C83 121.6(6) . . ?

C87 C88 H88 119.2 . . ?

C83 C88 H88 119.2 . . ?

O11 C89 C90 109.5(9) . . ?

O11 C89 H89A 109.8 . . ?

C90 C89 H89A 109.8 . . ?

O11 C89 H89B 109.8 . . ?

C90 C89 H89B 109.8 . . ?

H89A C89 H89B 108.2 . . ?

C89 C90 H90A 109.5 . . ?

C89 C90 H90B 109.5 . . ?

H90A C90 H90B 109.5 . . ?

C89 C90 H90C 109.5 . . ?

H90A C90 H90C 109.5 . . ?

H90B C90 H90C 109.5 . . ?

O12 C91 C92 112.7(8) . . ?

O12 C91 H91A 109.1 . . ?

C92 C91 H91A 109.1 . . ?

O12 C91 H91B 109.1 . . ?

C92 C91 H91B 109.1 . . ?

H91A C91 H91B 107.8 . . ?

C91 C92 H92A 109.5 . . ?

C91 C92 H92B 109.5 . . ?

H92A C92 H92B 109.5 . . ?

C91 C92 H92C 109.5 . . ?

H92A C92 H92C 109.5 . . ?

H92B C92 H92C 109.5 . . ?

C89 O11 P4 120.7(6) . . ?

C91 O12 P4 117.8(5) . . ?

O12 P4 N4 109.5(3) . . ?

O12 P4 O11 98.5(3) . . ?

N4 P4 O11 99.9(3) . . ?

O12 P4 S4 117.2(3) . . ?

N4 P4 S4 113.5(3) . . ?

O11 P4 S4 116.0(3) . . ?

O11A C89A C90A 108.3(13) . . ?

O11A C89A H89C 110.0 . . ?

C90A C89A H89C 110.0 . . ?

O11A C89A H89D 110.0 . . ?

C90A C89A H89D 110.0 . . ?

H89C C89A H89D 108.4 . . ?

C89A C90A H90D 109.5 . . ?

C89A C90A H90E 109.5 . . ?

H90D C90A H90E 109.5 . . ?

C89A C90A H90F 109.5 . . ?

H90D C90A H90F 109.5 . . ?

H90E C90A H90F 109.5 . . ?

O12A C91A C92A 114.1(13) . . ?

O12A C91A H91C 108.7 . . ?

C92A C91A H91C 108.7 . . ?

O12A C91A H91D 108.7 . . ?

C92A C91A H91D 108.7 . . ?

H91C C91A H91D 107.6 . . ?

C91A C92A H92D 109.5 . . ?

C91A C92A H92E 109.5 . . ?

H92D C92A H92E 109.5 . . ?

C91A C92A H92F 109.5 . . ?

H92D C92A H92F 109.5 . . ?

H92E C92A H92F 109.5 . . ?

C89A O11A P4A 118.5(10) . . ?

C91A O12A P4A 117.3(11) . . ?

O12A P4A N4 83.8(6) . . ?

O12A P4A O11A 93.9(6) . . ?

N4 P4A O11A 118.2(7) . . ?

O12A P4A S4A 112.2(6) . . ?

N4 P4A S4A 120.3(6) . . ?

O11A P4A S4A 117.5(7) . . ?

C12 N1 C8 112.9(4) . . ?

C12 N1 P1A 120.7(3) . . ?

C8 N1 P1A 126.5(4) . . ?

C12 N1 P1 120.7(3) . . ?

C8 N1 P1 126.5(4) . . ?

C35 N2 C31 112.8(5) . . ?

C35 N2 P2A 126.9(4) . . ?

C31 N2 P2A 120.2(4) . . ?

C35 N2 P2 126.9(4) . . ?

C31 N2 P2 120.2(4) . . ?

C58 N3 C54 112.9(4) . . ?

C58 N3 P3A 119.6(4) . . ?

C54 N3 P3A 125.1(4) . . ?

C58 N3 P3 119.6(4) . . ?

C54 N3 P3 125.1(4) . . ?

C81 N4 C77 112.7(4) . . ?

C81 N4 P4A 125.6(4) . . ?

C77 N4 P4A 119.0(3) . . ?

C81 N4 P4 125.6(4) . . ?

C77 N4 P4 119.0(3) . . ?

loop_

_geom_torsion_atom_site_label_1

_geom_torsion_atom_site_label_2

_geom_torsion_atom_site_label_3

_geom_torsion_atom_site_label_4

_geom_torsion

_geom_torsion_site_symmetry_1

_geom_torsion_site_symmetry_2

_geom_torsion_site_symmetry_3

_geom_torsion_site_symmetry_4

_geom_torsion_publ_flag

C6 C1 C2 C3 1.6(13) . . . . ?

F1 C1 C2 C3 179.5(7) . . . . ?

C1 C2 C3 C4 0.2(11) . . . . ?

C2 C3 C4 C5 -1.2(10) . . . . ?

C2 C3 C4 C7 -176.2(6) . . . . ?

C3 C4 C5 C6 0.3(10) . . . . ?

C7 C4 C5 C6 175.1(6) . . . . ?

C2 C1 C6 C5 -2.4(13) . . . . ?

F1 C1 C6 C5 179.8(7) . . . . ?

C4 C5 C6 C1 1.3(11) . . . . ?

C5 C4 C7 C9 31.9(9) . . . . ?

C3 C4 C7 C9 -153.4(6) . . . . ?

C4 C7 C9 C8 7.5(9) . . . . ?

C4 C7 C9 C10 -174.8(5) . . . . ?

N1 C8 C9 C7 149.7(5) . . . . ?

N1 C8 C9 C10 -28.0(7) . . . . ?

C7 C9 C10 O1 5.7(8) . . . . ?

C8 C9 C10 O1 -176.5(5) . . . . ?

C7 C9 C10 C11 -176.4(5) . . . . ?

C8 C9 C10 C11 1.5(7) . . . . ?

O1 C10 C11 C13 -11.3(8) . . . . ?

C9 C10 C11 C13 170.7(5) . . . . ?

O1 C10 C11 C12 173.4(5) . . . . ?

C9 C10 C11 C12 -4.5(7) . . . . ?

C13 C11 C12 N1 -140.7(6) . . . . ?

C10 C11 C12 N1 34.1(7) . . . . ?

C10 C11 C13 C14 178.4(6) . . . . ?

C12 C11 C13 C14 -6.8(11) . . . . ?

C11 C13 C14 C19 -34.4(11) . . . . ?

C11 C13 C14 C15 150.1(7) . . . . ?

C19 C14 C15 C16 -0.3(13) . . . . ?

C13 C14 C15 C16 175.4(8) . . . . ?

C14 C15 C16 C17 -0.9(15) . . . . ?

C15 C16 C17 F2 -178.7(9) . . . . ?

C15 C16 C17 C18 1.8(15) . . . . ?

F2 C17 C18 C19 179.0(8) . . . . ?

C16 C17 C18 C19 -1.5(14) . . . . ?

C17 C18 C19 C14 0.2(12) . . . . ?

C15 C14 C19 C18 0.7(11) . . . . ?

C13 C14 C19 C18 -174.8(7) . . . . ?

C21 C20 O2 P1 174.4(11) . . . . ?

C23 C22 O3 P1 110.4(15) . . . . ?

C20 O2 P1 O3 -177.6(10) . . . . ?

C20 O2 P1 N1 -73.7(10) . . . . ?

C20 O2 P1 S1 56.2(11) . . . . ?

C22 O3 P1 O2 -82.3(11) . . . . ?

C22 O3 P1 N1 163.9(9) . . . . ?

C22 O3 P1 S1 43.5(12) . . . . ?

C21A C20A O2A P1A 178.7(12) . . . . ?

C23A C22A O3A P1A 169.3(12) . . . . ?

C20A O2A P1A O3A 169.6(10) . . . . ?

C20A O2A P1A N1 -79.7(10) . . . . ?

C20A O2A P1A S1A 45.0(12) . . . . ?

C22A O3A P1A O2A -66.8(13) . . . . ?

C22A O3A P1A N1 -170.9(11) . . . . ?

C22A O3A P1A S1A 57.0(14) . . . . ?

F3 C24 C25 C26 -179.6(9) . . . . ?

C29 C24 C25 C26 1.2(15) . . . . ?

C24 C25 C26 C27 -1.5(14) . . . . ?

C25 C26 C27 C28 1.0(12) . . . . ?

C25 C26 C27 C30 177.3(8) . . . . ?

C26 C27 C28 C29 -0.1(10) . . . . ?

C30 C27 C28 C29 -176.3(7) . . . . ?

C25 C24 C29 C28 -0.4(14) . . . . ?

F3 C24 C29 C28 -179.6(8) . . . . ?

C27 C28 C29 C24 -0.2(12) . . . . ?

C28 C27 C30 C32 -35.4(10) . . . . ?

C26 C27 C30 C32 148.4(7) . . . . ?

C27 C30 C32 C33 178.6(6) . . . . ?

C27 C30 C32 C31 -5.2(10) . . . . ?

N2 C31 C32 C30 -141.3(5) . . . . ?

N2 C31 C32 C33 34.8(7) . . . . ?

C30 C32 C33 O4 -12.1(8) . . . . ?

C31 C32 C33 O4 171.5(5) . . . . ?

C30 C32 C33 C34 170.9(5) . . . . ?

C31 C32 C33 C34 -5.5(7) . . . . ?

O4 C33 C34 C36 8.3(8) . . . . ?

C32 C33 C34 C36 -174.8(5) . . . . ?

O4 C33 C34 C35 -175.5(5) . . . . ?

C32 C33 C34 C35 1.4(7) . . . . ?

C36 C34 C35 N2 148.3(5) . . . . ?

C33 C34 C35 N2 -27.5(7) . . . . ?

C33 C34 C36 C37 -176.3(5) . . . . ?

C35 C34 C36 C37 7.9(10) . . . . ?

C34 C36 C37 C38 32.5(10) . . . . ?

C34 C36 C37 C42 -152.2(6) . . . . ?

C42 C37 C38 C39 0.8(11) . . . . ?

C36 C37 C38 C39 176.1(7) . . . . ?

C37 C38 C39 C40 1.2(13) . . . . ?

C38 C39 C40 C41 -3.5(14) . . . . ?

C38 C39 C40 F4 -179.1(8) . . . . ?

F4 C40 C41 C42 179.1(8) . . . . ?

C39 C40 C41 C42 3.6(15) . . . . ?

C40 C41 C42 C37 -1.4(13) . . . . ?

C38 C37 C42 C41 -0.7(11) . . . . ?

C36 C37 C42 C41 -176.3(7) . . . . ?

C44 C43 O5 P2 162.4(12) . . . . ?

C46 C45 O6 P2 179.7(9) . . . . ?

C45 O6 P2 O5 150.4(12) . . . . ?

C45 O6 P2 N2 -100.5(11) . . . . ?

C45 O6 P2 S2 27.0(13) . . . . ?

C43 O5 P2 O6 -65.4(12) . . . . ?

C43 O5 P2 N2 -169.9(11) . . . . ?

C43 O5 P2 S2 58.2(12) . . . . ?

C44A C43A O5A P2A 105.3(18) . . . . ?

C46A C45A O6A P2A -152.7(13) . . . . ?

C45A O6A P2A O5A -150.1(14) . . . . ?

C45A O6A P2A N2 -49.1(14) . . . . ?

C45A O6A P2A S2A 85.5(15) . . . . ?

C43A O5A P2A O6A -74.2(12) . . . . ?

C43A O5A P2A N2 167.6(11) . . . . ?

C43A O5A P2A S2A 52.2(13) . . . . ?

C52 C47 C48 C49 0.6(15) . . . . ?

F5 C47 C48 C49 179.8(9) . . . . ?

C47 C48 C49 C50 -1.7(13) . . . . ?

C48 C49 C50 C51 1.4(12) . . . . ?

C48 C49 C50 C53 -173.8(7) . . . . ?

C49 C50 C51 C52 0.1(13) . . . . ?

C53 C50 C51 C52 175.6(8) . . . . ?

F5 C47 C52 C51 -178.4(10) . . . . ?

C48 C47 C52 C51 0.9(16) . . . . ?

C50 C51 C52 C47 -1.2(15) . . . . ?

C49 C50 C53 C55 -29.5(11) . . . . ?

C51 C50 C53 C55 155.3(7) . . . . ?

C50 C53 C55 C56 175.7(6) . . . . ?

C50 C53 C55 C54 -6.1(10) . . . . ?

N3 C54 C55 C53 -152.0(5) . . . . ?

N3 C54 C55 C56 26.2(7) . . . . ?

C53 C55 C56 O7 -3.7(8) . . . . ?

C54 C55 C56 O7 178.0(5) . . . . ?

C53 C55 C56 C57 177.7(5) . . . . ?

C54 C55 C56 C57 -0.6(7) . . . . ?

O7 C56 C57 C59 10.7(8) . . . . ?

C55 C56 C57 C59 -170.7(5) . . . . ?

O7 C56 C57 C58 -173.5(5) . . . . ?

C55 C56 C57 C58 5.1(7) . . . . ?

C59 C57 C58 N3 140.6(5) . . . . ?

C56 C57 C58 N3 -35.0(6) . . . . ?

C56 C57 C59 C60 -178.4(6) . . . . ?

C58 C57 C59 C60 6.0(10) . . . . ?

C57 C59 C60 C65 36.1(10) . . . . ?

C57 C59 C60 C61 -145.2(7) . . . . ?

C65 C60 C61 C62 -1.3(10) . . . . ?

C59 C60 C61 C62 180.0(7) . . . . ?

C60 C61 C62 C63 0.5(12) . . . . ?

C61 C62 C63 F6 179.4(8) . . . . ?

C61 C62 C63 C64 1.6(13) . . . . ?

F6 C63 C64 C65 179.4(8) . . . . ?

C62 C63 C64 C65 -2.8(13) . . . . ?

C63 C64 C65 C60 1.9(11) . . . . ?

C61 C60 C65 C64 0.0(10) . . . . ?

C59 C60 C65 C64 178.8(7) . . . . ?

C67 C66 O8 P3 154.6(8) . . . . ?

C69 C68 O9 P3 -160.5(9) . . . . ?

C68 O9 P3 N3 -89.8(8) . . . . ?

C68 O9 P3 O8 165.8(8) . . . . ?

C68 O9 P3 S3 40.6(8) . . . . ?

C66 O8 P3 O9 -63.0(8) . . . . ?

C66 O8 P3 N3 -174.9(8) . . . . ?

C66 O8 P3 S3 64.0(9) . . . . ?

C67A C66A O8A P3A 150(2) . . . . ?

C69A C68A O9A P3A -142(2) . . . . ?

C68A O9A P3A O8A 67.8(19) . . . . ?

C68A O9A P3A N3 -176.8(18) . . . . ?

C68A O9A P3A S3A -55(2) . . . . ?

C66A O8A P3A O9A 179.4(18) . . . . ?

C66A O8A P3A N3 86.3(18) . . . . ?

C66A O8A P3A S3A -60(2) . . . . ?

F7 C70 C71 C72 178.6(8) . . . . ?

C75 C70 C71 C72 -0.7(13) . . . . ?

C70 C71 C72 C73 1.6(12) . . . . ?

C71 C72 C73 C74 -1.2(10) . . . . ?

C71 C72 C73 C76 -178.8(7) . . . . ?

C72 C73 C74 C75 0.0(9) . . . . ?

C76 C73 C74 C75 177.4(6) . . . . ?

F7 C70 C75 C74 -179.8(7) . . . . ?

C71 C70 C75 C74 -0.5(12) . . . . ?

C73 C74 C75 C70 0.8(10) . . . . ?

C74 C73 C76 C78 39.0(9) . . . . ?

C72 C73 C76 C78 -143.6(6) . . . . ?

C73 C76 C78 C79 179.5(5) . . . . ?

C73 C76 C78 C77 5.5(10) . . . . ?

N4 C77 C78 C76 136.9(5) . . . . ?

N4 C77 C78 C79 -37.3(6) . . . . ?

C76 C78 C79 O10 12.5(8) . . . . ?

C77 C78 C79 O10 -173.0(5) . . . . ?

C76 C78 C79 C80 -167.6(5) . . . . ?

C77 C78 C79 C80 6.9(7) . . . . ?

O10 C79 C80 C82 -1.8(8) . . . . ?

C78 C79 C80 C82 178.2(5) . . . . ?

O10 C79 C80 C81 179.7(5) . . . . ?

C78 C79 C80 C81 -0.3(7) . . . . ?

C82 C80 C81 N4 -153.6(5) . . . . ?

C79 C80 C81 N4 24.8(7) . . . . ?

C79 C80 C82 C83 175.4(6) . . . . ?

C81 C80 C82 C83 -6.2(10) . . . . ?

C80 C82 C83 C84 -27.4(10) . . . . ?

C80 C82 C83 C88 154.8(7) . . . . ?

C88 C83 C84 C85 1.8(11) . . . . ?

C82 C83 C84 C85 -176.1(7) . . . . ?

C83 C84 C85 C86 -1.2(12) . . . . ?

C84 C85 C86 F8 178.7(7) . . . . ?

C84 C85 C86 C87 0.2(13) . . . . ?

F8 C86 C87 C88 -178.2(8) . . . . ?

C85 C86 C87 C88 0.2(14) . . . . ?

C86 C87 C88 C83 0.3(14) . . . . ?

C84 C83 C88 C87 -1.3(12) . . . . ?

C82 C83 C88 C87 176.8(8) . . . . ?

C90 C89 O11 P4 142.2(8) . . . . ?

C92 C91 O12 P4 -171.3(8) . . . . ?

C91 O12 P4 N4 -81.1(7) . . . . ?

C91 O12 P4 O11 175.2(7) . . . . ?

C91 O12 P4 S4 50.0(7) . . . . ?

C89 O11 P4 O12 -63.1(7) . . . . ?

C89 O11 P4 N4 -174.7(7) . . . . ?

C89 O11 P4 S4 62.9(7) . . . . ?

C90A C89A O11A P4A 175(3) . . . . ?

C92A C91A O12A P4A -114(3) . . . . ?

C91A O12A P4A N4 -165.6(17) . . . . ?

C91A O12A P4A O11A 76.4(18) . . . . ?

C91A O12A P4A S4A -45.4(19) . . . . ?

C89A O11A P4A O12A 178(2) . . . . ?

C89A O11A P4A N4 92(2) . . . . ?

C89A O11A P4A S4A -65(2) . . . . ?

C11 C12 N1 C8 -64.5(6) . . . . ?

C11 C12 N1 P1A 115.3(4) . . . . ?

C11 C12 N1 P1 115.3(4) . . . . ?

C9 C8 N1 C12 61.3(6) . . . . ?

C9 C8 N1 P1A -118.4(4) . . . . ?

C9 C8 N1 P1 -118.4(4) . . . . ?

O2A P1A N1 C12 -41.2(6) . . . . ?

O3A P1A N1 C12 62.0(6) . . . . ?

S1A P1A N1 C12 -165.6(5) . . . . ?

O2A P1A N1 C8 138.5(5) . . . . ?

O3A P1A N1 C8 -118.3(6) . . . . ?

S1A P1A N1 C8 14.1(6) . . . . ?

O2 P1 N1 C12 -44.1(6) . . . . ?

O3 P1 N1 C12 60.2(6) . . . . ?

S1 P1 N1 C12 -176.1(5) . . . . ?

O2 P1 N1 C8 135.6(5) . . . . ?

O3 P1 N1 C8 -120.0(6) . . . . ?

S1 P1 N1 C8 3.7(6) . . . . ?

C34 C35 N2 C31 60.7(6) . . . . ?

C34 C35 N2 P2A -115.0(5) . . . . ?

C34 C35 N2 P2 -115.0(5) . . . . ?

C32 C31 N2 C35 -64.2(6) . . . . ?

C32 C31 N2 P2A 111.8(4) . . . . ?

C32 C31 N2 P2 111.8(4) . . . . ?

O6A P2A N2 C35 131.4(6) . . . . ?

O5A P2A N2 C35 -125.7(6) . . . . ?

S2A P2A N2 C35 -6.4(6) . . . . ?

O6A P2A N2 C31 -44.0(6) . . . . ?

O5A P2A N2 C31 58.9(6) . . . . ?

S2A P2A N2 C31 178.2(5) . . . . ?

O6 P2 N2 C35 133.9(5) . . . . ?

O5 P2 N2 C35 -123.8(6) . . . . ?

S2 P2 N2 C35 7.2(6) . . . . ?

O6 P2 N2 C31 -41.5(5) . . . . ?

O5 P2 N2 C31 60.8(6) . . . . ?

S2 P2 N2 C31 -168.1(4) . . . . ?

C57 C58 N3 C54 64.0(6) . . . . ?

C57 C58 N3 P3A -99.1(5) . . . . ?

C57 C58 N3 P3 -99.1(5) . . . . ?

C55 C54 N3 C58 -59.4(6) . . . . ?

C55 C54 N3 P3A 102.6(5) . . . . ?

C55 C54 N3 P3 102.6(5) . . . . ?

O9A P3A N3 C58 -72.7(7) . . . . ?

O8A P3A N3 C58 23.9(7) . . . . ?

S3A P3A N3 C58 170.0(6) . . . . ?

O9A P3A N3 C54 126.5(7) . . . . ?

O8A P3A N3 C54 -136.9(6) . . . . ?

S3A P3A N3 C54 9.2(7) . . . . ?

O9 P3 N3 C58 -43.5(5) . . . . ?

O8 P3 N3 C58 59.7(5) . . . . ?

S3 P3 N3 C58 -176.9(4) . . . . ?

O9 P3 N3 C54 155.7(4) . . . . ?

O8 P3 N3 C54 -101.1(5) . . . . ?

S3 P3 N3 C54 22.3(5) . . . . ?

C80 C81 N4 C77 -58.4(6) . . . . ?

C80 C81 N4 P4A 102.4(5) . . . . ?

C80 C81 N4 P4 102.4(5) . . . . ?

C78 C77 N4 C81 65.1(5) . . . . ?

C78 C77 N4 P4A -97.2(4) . . . . ?

C78 C77 N4 P4 -97.2(4) . . . . ?

O12A P4A N4 C81 120.7(7) . . . . ?

O11A P4A N4 C81 -148.2(6) . . . . ?

S4A P4A N4 C81 8.5(8) . . . . ?

O12A P4A N4 C77 -79.5(7) . . . . ?

O11A P4A N4 C77 11.6(7) . . . . ?

S4A P4A N4 C77 168.3(7) . . . . ?

O12 P4 N4 C81 150.4(4) . . . . ?

O11 P4 N4 C81 -106.8(4) . . . . ?

S4 P4 N4 C81 17.3(5) . . . . ?

O12 P4 N4 C77 -49.8(4) . . . . ?

O11 P4 N4 C77 53.0(4) . . . . ?

S4 P4 N4 C77 177.1(4) . . . . ?

_refine_diff_density_max 0.520

_refine_diff_density_min -0.456

_refine_diff_density_rms 0.047

_shelx_res_file

;

TITL bmk2351b in Pna2(1)

shelx.res

created by SHELXL-2019/3 at 11:38:12 on 02-Apr-2025

CELL 1.54184 32.9400 12.5414 22.4554 90.000 90.000 90.000

ZERR 16.00 0.0005 0.0002 0.0004 0.000 0.000 0.000

LATT -1

SYMM - X, - Y, 1/2 + Z

SYMM 1/2 + X, 1/2 - Y, Z

SYMM 1/2 - X, 1/2 + Y, 1/2 + Z

SFAC C H N O F P S

UNIT 368 384 16 48 32 16 16

MERG 2

eadp p1 p1a

exyz p1 p1a

isor .01 c20 > s1a

simu .005 c20 > s1a

eadp p2 p2a

exyz p2 p2a

isor .01 c43 > s2a

simu .005 c43 > s2a

eadp p3 p3a

exyz p3 p3a

isor .01 c66 > s3a

simu .005 c66 > s3a

eadp p4 p4a

exyz p4 p4a

isor .01 c89 > s4a

simu .005 c89 > s4a

MPLA C1 C2 C3 C4 C5 C6 C7 F1

MPLA 6 C8 C9 C10 C11 C12 O1 N1

MPLA C13 C14 C15 C16 C17 C18 C19 F2

MPLA C24 C25 C26 C27 C28 C29 C30 F3

MPLA 6 C31 C32 C33 C34 C35 O4 n2

MPLA C36 C37 C38 C39 C40 C41 C42 F4

MPLA C47 C48 C49 C50 C51 C52 C53 F5

MPLA 6 C54 C55 C56 C57 C58 O7 n3

MPLA C59 C60 C61 C62 C63 C64 C65 F6

MPLA C70 C71 C72 C73 C74 C75 C76 F7

MPLA 6 C77 C78 C79 C80 C81 O10 n4

MPLA C82 C83 C84 C85 C86 C87 C88 F8

FMAP 2

PLAN 10

SIZE 0.100 0.120 0.590

ACTA

conf

BOND $H

LIST 4

L.S. 4

WGHT 0.095200 2.359100

FVAR 0.06501 0.50526 0.56939 0.72766 0.77701

C1 1 0.192566 0.388996 0.600233 11.00000 0.04669 0.12400 =

0.10085 -0.02254 0.00051 -0.00226

C2 1 0.202594 0.288749 0.613404 11.00000 0.05862 0.11249 =

0.09026 -0.00981 0.01285 -0.01823

AFIX 43

H2 2 0.183175 0.240045 0.626206 11.00000 -1.20000

AFIX 0

C3 1 0.243020 0.259625 0.607341 11.00000 0.05727 0.07886 =

0.08092 0.00294 0.00855 -0.01753

AFIX 43

H3 2 0.250741 0.190049 0.616115 11.00000 -1.20000

AFIX 0

C4 1 0.272474 0.332660 0.588299 11.00000 0.05377 0.06513 =

0.05457 -0.00317 -0.00014 -0.00739

C5 1 0.260039 0.433889 0.574707 11.00000 0.05762 0.07672 =

0.08317 -0.00037 -0.00803 -0.00537

AFIX 43

H5 2 0.278966 0.483792 0.561763 11.00000 -1.20000

AFIX 0

C6 1 0.219316 0.463241 0.580012 11.00000 0.06517 0.08356 =

0.11540 -0.01436 -0.02365 0.01069

AFIX 43

H6 2 0.210731 0.531576 0.570010 11.00000 -1.20000

AFIX 0

C7 1 0.313824 0.293868 0.579506 11.00000 0.05455 0.05541 =

0.06049 0.00298 0.00002 -0.00444

AFIX 43

H7 2 0.316068 0.221809 0.570302 11.00000 -1.20000

AFIX 0

C8 1 0.354099 0.460135 0.603630 11.00000 0.04851 0.05313 =

0.07191 -0.00438 0.00060 0.00294

AFIX 23

H8A 2 0.351477 0.508369 0.570082 11.00000 -1.20000

H8B 2 0.332969 0.477182 0.632171 11.00000 -1.20000

AFIX 0

C9 1 0.349171 0.347395 0.582805 11.00000 0.05092 0.04696 =

0.05394 0.00036 -0.00041 -0.00244

C10 1 0.386441 0.285567 0.565968 11.00000 0.05623 0.04793 =

0.06028 -0.00046 -0.00202 0.00131

C11 1 0.426776 0.338169 0.572138 11.00000 0.05303 0.05698 =

0.05613 -0.00317 -0.00088 -0.00077

C12 1 0.427483 0.453353 0.590586 11.00000 0.05007 0.05904 =

0.06325 -0.00999 0.00161 -0.00592

AFIX 23

H12A 2 0.453097 0.469605 0.609871 11.00000 -1.20000

H12B 2 0.424916 0.498587 0.555751 11.00000 -1.20000

AFIX 0

C13 1 0.459575 0.276182 0.564949 11.00000 0.05453 0.06296 =

0.07571 -0.00995 -0.00038 0.00672

AFIX 43

H13 2 0.454178 0.204569 0.557675 11.00000 -1.20000

AFIX 0

C14 1 0.502380 0.304613 0.566867 11.00000 0.05317 0.07536 =

0.07621 -0.00920 0.00045 0.00827

C15 1 0.529899 0.227099 0.586065 11.00000 0.06336 0.08011 =

0.14064 -0.00070 -0.00880 0.01730

AFIX 43

H15 2 0.520174 0.161904 0.599700 11.00000 -1.20000

AFIX 0

C16 1 0.570944 0.245185 0.585212 11.00000 0.06264 0.10547 =

0.14770 -0.01438 -0.02091 0.01901

AFIX 43

H16 2 0.588951 0.193347 0.598603 11.00000 -1.20000

AFIX 0

C17 1 0.584892 0.340472 0.564396 11.00000 0.04891 0.11906 =

0.11752 -0.01436 -0.00585 -0.00027

C18 1 0.559118 0.419863 0.545795 11.00000 0.06600 0.09762 =

0.09812 0.00021 0.00483 -0.00779

AFIX 43

H18 2 0.569330 0.484842 0.532613 11.00000 -1.20000

AFIX 0

C19 1 0.517976 0.401655 0.546992 11.00000 0.05340 0.08268 =

0.08059 0.00609 0.00018 0.00636

AFIX 43

H19 2 0.500284 0.454836 0.534361 11.00000 -1.20000

PART 1

AFIX 0

C20 1 0.430233 0.328413 0.745375 21.00000 0.08577 0.06555 =

0.06853 -0.01065 -0.00107 0.00105

AFIX 23

H20A 2 0.419526 0.288852 0.711744 21.00000 -1.20000

H20B 2 0.410631 0.325104 0.777558 21.00000 -1.20000

AFIX 0

C21 1 0.470061 0.281026 0.765100 21.00000 0.09891 0.07137 =

0.07423 -0.01342 -0.00409 0.01194

AFIX 137

H21A 2 0.490138 0.292241 0.734751 21.00000 -1.50000

H21B 2 0.466730 0.205926 0.771776 21.00000 -1.50000

H21C 2 0.478689 0.314720 0.801341 21.00000 -1.50000

AFIX 0

C22 1 0.434294 0.692660 0.735425 21.00000 0.09205 0.06983 =

0.07606 -0.01313 -0.00647 -0.00997

AFIX 23

H22A 2 0.417746 0.675340 0.769821 21.00000 -1.20000

H22B 2 0.426529 0.762929 0.721424 21.00000 -1.20000

AFIX 0

C23 1 0.477106 0.692501 0.752054 21.00000 0.10738 0.09584 =

0.08987 -0.00791 -0.01584 -0.01441

AFIX 137

H23A 2 0.485036 0.621795 0.763585 21.00000 -1.50000

H23B 2 0.481270 0.740469 0.784805 21.00000 -1.50000

H23C 2 0.493186 0.715134 0.718714 21.00000 -1.50000

AFIX 0

O2 4 0.437369 0.437247 0.728751 21.00000 0.07298 0.06072 =

0.06242 -0.00802 -0.00625 -0.00114

O3 4 0.427644 0.614682 0.688807 21.00000 0.08571 0.06019 =

0.06903 -0.01582 -0.00437 -0.01139

P1 6 0.402890 0.508063 0.700295 21.00000 0.06501 0.06053 =

0.06210 -0.01032 -0.00027 -0.00192

S1 7 0.353530 0.521099 0.745952 21.00000 0.08597 0.08225 =

0.07055 -0.00941 0.02203 0.00818

PART 2

same .005 c20 > s1

C20A 1 0.431883 0.320783 0.730417 -21.00000 0.08514 0.06881 =

0.06875 -0.00812 0.00029 -0.00032

AFIX 23

H20C 2 0.417706 0.285407 0.698119 -21.00000 -1.20000

H20D 2 0.415275 0.316170 0.765995 -21.00000 -1.20000

AFIX 0

C21A 1 0.472453 0.268527 0.740931 -21.00000 0.09700 0.08125 =

0.08695 -0.00658 0.00057 0.01436

AFIX 137

H21D 2 0.488729 0.274244 0.705612 -21.00000 -1.50000

H21E 2 0.468464 0.194660 0.750496 -21.00000 -1.50000

H21F 2 0.486009 0.303390 0.773383 -21.00000 -1.50000

AFIX 0

C22A 1 0.444090 0.659946 0.754734 -21.00000 0.09074 0.07169 =

0.07226 -0.00971 -0.00655 -0.01070

AFIX 23

H22C 2 0.459322 0.604515 0.774942 -21.00000 -1.20000

H22D 2 0.422460 0.683273 0.780991 -21.00000 -1.20000

AFIX 0

C23A 1 0.470767 0.749375 0.741356 -21.00000 0.10794 0.07297 =

0.08334 -0.00664 -0.01631 -0.01625

AFIX 137

H23D 2 0.498115 0.724115 0.736956 -21.00000 -1.50000

H23E 2 0.469607 0.800200 0.773278 -21.00000 -1.50000

H23F 2 0.462168 0.782768 0.705014 -21.00000 -1.50000

AFIX 0

O2A 4 0.439277 0.430286 0.715506 -21.00000 0.07332 0.06183 =

0.06407 -0.00267 -0.00470 0.00055

O3A 4 0.426888 0.617831 0.700200 -21.00000 0.08566 0.06096 =

0.06764 -0.01452 -0.00352 -0.00905

P1A 6 0.402890 0.508063 0.700295 -21.00000 0.06501 0.06053 =

0.06210 -0.01032 -0.00027 -0.00192

S1A 7 0.356914 0.499155 0.753547 -21.00000 0.07539 0.09705 =

0.07441 -0.00337 0.00930 0.01353

PART 0

C24 1 0.672306 0.612489 0.598137 11.00000 0.05152 0.11807 =

0.11072 -0.03137 0.00058 0.01312

C25 1 0.683927 0.710147 0.616786 11.00000 0.07551 0.09939 =

0.12084 -0.03029 0.00837 0.02853

AFIX 43

H25 2 0.665150 0.756598 0.633622 11.00000 -1.20000

AFIX 0

C26 1 0.723567 0.739926 0.610641 11.00000 0.07099 0.08248 =

0.10016 -0.01778 0.00118 0.01736

AFIX 43

H26 2 0.731595 0.807733 0.622573 11.00000 -1.20000

AFIX 0

C27 1 0.752187 0.670094 0.586722 11.00000 0.06024 0.07250 =

0.06614 -0.00382 -0.00150 0.01277

C28 1 0.738994 0.570046 0.568535 11.00000 0.05783 0.08128 =

0.07567 -0.02230 -0.00017 0.00783

AFIX 43

H28 2 0.757504 0.522121 0.552360 11.00000 -1.20000

AFIX 0

C29 1 0.698853 0.540891 0.574145 11.00000 0.05945 0.09675 =

0.10382 -0.03395 -0.00148 0.00384

AFIX 43

H29 2 0.690055 0.473950 0.561862 11.00000 -1.20000

AFIX 0

C30 1 0.793901 0.707229 0.578596 11.00000 0.06553 0.05803 =

0.06887 -0.00557 -0.00471 0.01275

AFIX 43

H30 2 0.796673 0.779463 0.570160 11.00000 -1.20000

AFIX 0

C31 1 0.831894 0.536553 0.599513 11.00000 0.05364 0.05756 =

0.06499 0.00345 -0.00379 -0.00264

AFIX 23

H31A 2 0.807967 0.516117 0.621959 11.00000 -1.20000

H31B 2 0.833462 0.491894 0.564319 11.00000 -1.20000

AFIX 0

C32 1 0.828922 0.651849 0.581571 11.00000 0.05953 0.05424 =

0.05252 -0.00184 -0.00447 0.00037

C33 1 0.867566 0.710721 0.569816 11.00000 0.06175 0.04497 =

0.06215 -0.00397 -0.00338 0.00185

C34 1 0.906942 0.656092 0.581643 11.00000 0.05982 0.04804 =

0.05729 -0.00111 -0.00252 -0.00383

C35 1 0.905839 0.542755 0.603643 11.00000 0.05274 0.05432 =

0.08093 0.00721 -0.00255 0.00149

AFIX 23

H35A 2 0.907783 0.494413 0.570044 11.00000 -1.20000

H35B 2 0.928937 0.529971 0.629468 11.00000 -1.20000

AFIX 0

C36 1 0.940633 0.715624 0.574937 11.00000 0.06487 0.05211 =

0.05991 0.00190 -0.00396 -0.01032

AFIX 43

H36 2 0.936049 0.786947 0.565810 11.00000 -1.20000

AFIX 0

C37 1 0.983216 0.685224 0.579815 11.00000 0.06351 0.06228 =

0.06319 0.00428 -0.00469 -0.01092

C38 1 0.998421 0.586272 0.564793 11.00000 0.07099 0.07214 =

0.09044 0.00175 0.00684 -0.01038

AFIX 43

H38 2 0.980687 0.532710 0.552806 11.00000 -1.20000

AFIX 0

C39 1 1.039430 0.565634 0.567278 11.00000 0.06738 0.08473 =

0.13148 0.00438 0.01043 0.00751

AFIX 43

H39 2 1.049701 0.499327 0.556468 11.00000 -1.20000

AFIX 0

C40 1 1.064803 0.646016 0.586234 11.00000 0.06044 0.12013 =

0.10094 0.01402 -0.00457 -0.00784

C41 1 1.052243 0.743464 0.599339 11.00000 0.07300 0.11105 =

0.10971 -0.00018 -0.02386 -0.02117

AFIX 43

H41 2 1.070588 0.796579 0.609877 11.00000 -1.20000

AFIX 0

C42 1 1.010916 0.764012 0.596891 11.00000 0.06894 0.07851 =

0.08897 0.00070 -0.01178 -0.01638

AFIX 43

H42 2 1.001425 0.831557 0.606806 11.00000 -1.20000

PART 1

AFIX 0

C43 1 0.825836 0.341627 0.765850 31.00000 0.11171 0.08984 =

0.08786 0.01990 0.00321 -0.01600

AFIX 23

H43A 2 0.814101 0.399236 0.788968 31.00000 -1.20000

H43B 2 0.848213 0.311657 0.788395 31.00000 -1.20000

AFIX 0

C44 1 0.795706 0.260887 0.755382 31.00000 0.12515 0.08923 =

0.10752 0.01967 0.01950 -0.02243

AFIX 137

H44A 2 0.777554 0.284207 0.724576 31.00000 -1.50000

H44B 2 0.780649 0.248494 0.791325 31.00000 -1.50000

H44C 2 0.808791 0.196029 0.743252 31.00000 -1.50000

AFIX 0

C45 1 0.840588 0.668088 0.756025 31.00000 0.10747 0.08360 =

0.07904 0.00620 -0.01050 -0.01129

AFIX 23

H45A 2 0.857727 0.711977 0.730864 31.00000 -1.20000

H45B 2 0.854716 0.652806 0.792952 31.00000 -1.20000

AFIX 0

C46 1 0.798301 0.722668 0.767837 31.00000 0.11499 0.09459 =

0.09857 -0.00174 -0.00185 -0.01225

AFIX 137

H46A 2 0.783387 0.727073 0.731190 31.00000 -1.50000

H46B 2 0.802517 0.793090 0.783410 31.00000 -1.50000

H46C 2 0.783216 0.681118 0.796113 31.00000 -1.50000

AFIX 0

O5 4 0.840430 0.382006 0.710303 31.00000 0.10677 0.07990 =

0.07792 0.02486 -0.00168 -0.01756

O6 4 0.828171 0.569509 0.725425 31.00000 0.10304 0.08553 =

0.07386 0.00306 -0.00317 -0.00612

P2 6 0.864239 0.493309 0.706214 31.00000 0.08608 0.07800 =

0.07057 0.01397 -0.00881 -0.00824

S2 7 0.912990 0.503040 0.753890 31.00000 0.10943 0.09985 =

0.08445 0.00418 -0.02980 0.00119

PART 2

same .005 c43 > s2

C43A 1 0.831926 0.309083 0.742820 -31.00000 0.10856 0.08478 =

0.08608 0.01803 0.00615 -0.01604

AFIX 23

H43C 2 0.850322 0.322497 0.775709 -31.00000 -1.20000

H43D 2 0.837076 0.237733 0.727957 -31.00000 -1.20000

AFIX 0

C44A 1 0.790580 0.317015 0.763107 -31.00000 0.11539 0.09696 =

0.10763 0.01073 0.01558 -0.01553

AFIX 137

H44D 2 0.785119 0.388901 0.775418 -31.00000 -1.50000

H44E 2 0.786524 0.269587 0.796132 -31.00000 -1.50000

H44F 2 0.772526 0.297845 0.731300 -31.00000 -1.50000

AFIX 0

C45A 1 0.834030 0.674407 0.734119 -31.00000 0.10887 0.08829 =

0.08106 0.01089 -0.00563 -0.01323

AFIX 23

H45C 2 0.817606 0.697404 0.700645 -31.00000 -1.20000

H45D 2 0.861784 0.698127 0.728371 -31.00000 -1.20000

AFIX 0

C46A 1 0.816224 0.715388 0.794839 -31.00000 0.12159 0.09766 =

0.08590 0.00267 -0.00530 -0.01517

AFIX 137

H46D 2 0.827222 0.673885 0.826934 -31.00000 -1.50000

H46E 2 0.787204 0.708361 0.794422 -31.00000 -1.50000

H46F 2 0.823361 0.788963 0.800271 -31.00000 -1.50000

AFIX 0

O5A 4 0.838837 0.385094 0.696608 -31.00000 0.10562 0.07942 =

0.07912 0.02384 -0.00134 -0.01731

O6A 4 0.831840 0.558106 0.742576 -31.00000 0.10074 0.08270 =

0.07321 0.00847 -0.00628 -0.00856

P2A 6 0.864239 0.493309 0.706214 -31.00000 0.08608 0.07800 =

0.07057 0.01397 -0.00881 -0.00824

S2A 7 0.917370 0.474531 0.741225 -31.00000 0.10619 0.10113 =

0.08295 0.00916 -0.03819 0.00342

PART 0

C47 1 0.179624 0.611714 0.431964 11.00000 0.05997 0.10156 =

0.12871 0.01415 -0.00655 -0.00078

C48 1 0.206350 0.693800 0.445352 11.00000 0.06914 0.08494 =

0.11978 -0.00039 0.00537 0.00242

AFIX 43

H48 2 0.197060 0.761611 0.455109 11.00000 -1.20000

AFIX 0

C49 1 0.247312 0.670623 0.443587 11.00000 0.06591 0.06431 =

0.09586 -0.00830 0.00341 -0.00675

AFIX 43

H49 2 0.265944 0.724595 0.451309 11.00000 -1.20000

AFIX 0

C50 1 0.261377 0.569450 0.430664 11.00000 0.05956 0.06311 =

0.07207 -0.00020 -0.00349 -0.00970

C51 1 0.232607 0.490133 0.417873 11.00000 0.06668 0.06929 =

0.11666 -0.00589 -0.00636 -0.01197

AFIX 43

H51 2 0.241244 0.421315 0.409070 11.00000 -1.20000

AFIX 0

C52 1 0.191799 0.513083 0.418217 11.00000 0.07000 0.09172 =

0.15055 0.00521 -0.02031 -0.02390

AFIX 43

H52 2 0.172881 0.460577 0.408989 11.00000 -1.20000

AFIX 0

C53 1 0.303873 0.537038 0.433674 11.00000 0.06122 0.05554 =

0.07219 -0.00624 -0.00478 -0.00515

AFIX 43

H53 2 0.307919 0.465490 0.442797 11.00000 -1.20000

AFIX 0

C54 1 0.339881 0.708256 0.404565 11.00000 0.05846 0.05339 =

0.07454 0.00413 -0.00344 0.00053

AFIX 23

H54A 2 0.317086 0.722576 0.378414 11.00000 -1.20000

H54B 2 0.337794 0.755458 0.438647 11.00000 -1.20000

AFIX 0

C55 1 0.338092 0.593222 0.425477 11.00000 0.05944 0.04796 =

0.05957 -0.00493 -0.00431 -0.00073

C56 1 0.376937 0.535608 0.436378 11.00000 0.06104 0.05150 =

0.05761 0.00061 -0.00326 -0.00040

C57 1 0.415863 0.593364 0.425638 11.00000 0.05650 0.05254 =

0.05334 -0.00248 -0.00068 0.00132

C58 1 0.413353 0.710235 0.409347 11.00000 0.05129 0.05746 =

0.06350 0.00293 -0.00103 0.00079

AFIX 23

H58A 2 0.411938 0.752974 0.445289 11.00000 -1.20000

H58B 2 0.437508 0.730945 0.387516 11.00000 -1.20000

AFIX 0

C59 1 0.450536 0.537707 0.427205 11.00000 0.06321 0.05585 =

0.06376 -0.00011 -0.00057 0.00788

AFIX 43

H59 2 0.447340 0.465070 0.434099 11.00000 -1.20000

AFIX 0

C60 1 0.492208 0.572171 0.419916 11.00000 0.05886 0.06351 =

0.05978 -0.00134 -0.00108 0.00942

C61 1 0.520388 0.505431 0.391848 11.00000 0.06676 0.06763 =

0.08732 -0.01170 0.00381 0.01382

AFIX 43

H61 2 0.511905 0.439113 0.378117 11.00000 -1.20000

AFIX 0

C62 1 0.559895 0.534696 0.384025 11.00000 0.06452 0.09444 =

0.11014 -0.02027 0.00158 0.02052

AFIX 43

H62 2 0.578068 0.489324 0.364988 11.00000 -1.20000

AFIX 0

C63 1 0.572232 0.631256 0.404522 11.00000 0.05959 0.09337 =

0.10895 -0.00721 -0.00565 0.00398

C64 1 0.546432 0.700300 0.434442 11.00000 0.05893 0.08754 =

0.10971 -0.02050 -0.00766 0.00464

AFIX 43

H64 2 0.555765 0.764787 0.449628 11.00000 -1.20000

AFIX 0

C65 1 0.506354 0.669818 0.440877 11.00000 0.06650 0.07501 =

0.07424 -0.01451 -0.00179 0.01156

AFIX 43

H65 2 0.488326 0.715824 0.459737 11.00000 -1.20000

PART 1

AFIX 0

C66 1 0.413248 0.595519 0.225740 41.00000 0.11791 0.09744 =

0.08275 0.00404 0.00279 -0.00264

AFIX 23

H66A 2 0.424273 0.658378 0.206455 41.00000 -1.20000

H66B 2 0.390253 0.570225 0.202750 41.00000 -1.20000

AFIX 0

C67 1 0.444665 0.511225 0.230580 41.00000 0.13048 0.10939 =

0.10090 -0.00019 0.02368 -0.00793

AFIX 137

H67A 2 0.465813 0.534645 0.256929 41.00000 -1.50000

H67B 2 0.455910 0.497219 0.191923 41.00000 -1.50000

H67C 2 0.432606 0.447258 0.246021 41.00000 -1.50000

AFIX 0

C68 1 0.415726 0.922530 0.277596 41.00000 0.10287 0.08843 =

0.08826 -0.00412 0.00077 0.00183

AFIX 23

H68A 2 0.415200 0.958032 0.315965 41.00000 -1.20000

H68B 2 0.391038 0.939739 0.256101 41.00000 -1.20000

AFIX 0

C69 1 0.452775 0.954685 0.242354 41.00000 0.12934 0.10362 =

0.11552 0.01329 0.01757 0.00461

AFIX 137

H69A 2 0.476703 0.942134 0.265731 41.00000 -1.50000

H69B 2 0.451075 1.029031 0.232487 41.00000 -1.50000

H69C 2 0.454110 0.913340 0.206428 41.00000 -1.50000

AFIX 0

O8 4 0.401100 0.619769 0.285803 41.00000 0.10614 0.08777 =

0.07257 0.00885 0.00384 0.00168

O9 4 0.420110 0.806702 0.284828 41.00000 0.09206 0.08479 =

0.07890 0.01665 0.00500 0.00198

P3 6 0.382236 0.737713 0.300665 41.00000 0.08891 0.09439 =

0.06730 0.01770 -0.00889 0.00145

S3 7 0.331606 0.770849 0.262195 41.00000 0.11634 0.09264 =

0.09672 0.02044 -0.04531 -0.00782

PART 2

same .005 c66 > s3

C66A 1 0.417297 0.565929 0.259327 -41.00000 0.11196 0.09486 =

0.08252 0.00867 0.00418 -0.00203

AFIX 23

H66C 2 0.389470 0.549782 0.248445 -41.00000 -1.20000

H66D 2 0.424697 0.522758 0.293485 -41.00000 -1.20000

AFIX 0

C67A 1 0.444932 0.544419 0.208794 -41.00000 0.12742 0.10774 =

0.09238 0.00734 0.00810 -0.00145

AFIX 137

H67D 2 0.453648 0.610713 0.191668 -41.00000 -1.50000

H67E 2 0.430930 0.503161 0.179215 -41.00000 -1.50000

H67F 2 0.468147 0.505424 0.222709 -41.00000 -1.50000

AFIX 0

C68A 1 0.414521 0.912320 0.253434 -41.00000 0.10215 0.08943 =

0.08583 0.00645 0.00038 0.00113

AFIX 23

H68C 2 0.394230 0.965962 0.244356 -41.00000 -1.20000

H68D 2 0.417328 0.865059 0.219489 -41.00000 -1.20000

AFIX 0

C69A 1 0.454681 0.963549 0.268862 -41.00000 0.11150 0.09545 =

0.08828 0.00097 0.00354 0.00032

AFIX 137

H69D 2 0.462204 0.944356 0.308749 -41.00000 -1.50000

H69E 2 0.452212 1.039650 0.266013 -41.00000 -1.50000

H69F 2 0.475142 0.939193 0.241628 -41.00000 -1.50000

AFIX 0

O8A 4 0.421644 0.677838 0.272956 -41.00000 0.10047 0.09228 =

0.07602 0.00862 0.00147 -0.00185

O9A 4 0.403428 0.852336 0.307186 -41.00000 0.09678 0.09244 =

0.07741 0.01224 -0.00143 -0.00081

P3A 6 0.382236 0.737713 0.300665 -41.00000 0.08891 0.09439 =

0.06730 0.01770 -0.00889 0.00145

S3A 7 0.333738 0.739874 0.252683 -41.00000 0.10023 0.09199 =

0.07713 -0.00329 -0.03176 0.00793

PART 0

C70 1 0.672266 0.384181 0.422791 11.00000 0.05303 0.11593 =

0.10161 -0.01919 -0.01116 0.00065

C71 1 0.685250 0.479598 0.401231 11.00000 0.06482 0.11862 =

0.10146 -0.01405 -0.02100 0.02591

AFIX 43

H71 2 0.667167 0.526434 0.383139 11.00000 -1.20000

AFIX 0

C72 1 0.725178 0.505167 0.406646 11.00000 0.06531 0.07988 =

0.08236 -0.00358 -0.00516 0.01501

AFIX 43

H72 2 0.734169 0.571007 0.392895 11.00000 -1.20000

AFIX 0

C73 1 0.753320 0.434217 0.432632 11.00000 0.05199 0.06815 =

0.05988 -0.00891 -0.00175 0.01145

C74 1 0.738578 0.337920 0.453562 11.00000 0.05948 0.08223 =

0.06423 -0.00342 0.00366 0.00674

AFIX 43

H74 2 0.756442 0.289607 0.470895 11.00000 -1.20000

AFIX 0

C75 1 0.697908 0.311919 0.449288 11.00000 0.06413 0.09685 =

0.09006 -0.00967 0.00419 -0.00423

AFIX 43

H75 2 0.688179 0.247418 0.463913 11.00000 -1.20000

AFIX 0

C76 1 0.795557 0.467464 0.438167 11.00000 0.05557 0.05951 =

0.06431 -0.00568 -0.00030 0.00741

AFIX 43

H76 2 0.799492 0.539209 0.446907 11.00000 -1.20000

AFIX 0

C77 1 0.830432 0.294482 0.413329 11.00000 0.04842 0.05172 =

0.06093 -0.00362 -0.00268 0.00171

AFIX 23

H77A 2 0.834619 0.249167 0.447792 11.00000 -1.20000

H77B 2 0.804789 0.274972 0.395099 11.00000 -1.20000

AFIX 0

C78 1 0.829170 0.410209 0.432544 11.00000 0.04875 0.05435 =

0.05084 0.00209 -0.00102 0.00168

C79 1 0.869186 0.464936 0.440069 11.00000 0.05651 0.04858 =

0.05749 0.00110 0.00417 -0.00196

C80 1 0.906966 0.408682 0.422059 11.00000 0.04932 0.04742 =

0.05494 0.00260 -0.00081 -0.00124

C81 1 0.903300 0.296602 0.396739 11.00000 0.04598 0.05470 =

0.07095 -0.00575 0.00309 0.00373

AFIX 23

H81A 2 0.924022 0.286004 0.366623 11.00000 -1.20000

H81B 2 0.907831 0.245032 0.428239 11.00000 -1.20000

AFIX 0

C82 1 0.941769 0.462910 0.428372 11.00000 0.05695 0.05707 =

0.06715 0.00044 0.00179 -0.00657

AFIX 43

H82 2 0.938610 0.533169 0.440782 11.00000 -1.20000

AFIX 0

C83 1 0.983779 0.430072 0.419018 11.00000 0.05174 0.06669 =

0.06105 -0.00290 0.00323 -0.01117

C84 1 0.998123 0.327002 0.424168 11.00000 0.05646 0.07700 =

0.08268 0.00999 -0.00706 -0.00742

AFIX 43

H84 2 0.979924 0.272064 0.431986 11.00000 -1.20000

AFIX 0

C85 1 1.038738 0.303751 0.417994 11.00000 0.05790 0.08933 =

0.10799 0.00248 -0.00594 0.00071

AFIX 43

H85 2 1.048091 0.234215 0.422323 11.00000 -1.20000

AFIX 0

C86 1 1.065112 0.385133 0.405338 11.00000 0.05069 0.11035 =

0.09758 -0.01447 0.01027 -0.00453

C87 1 1.052719 0.487815 0.398858 11.00000 0.06470 0.10087 =

0.12474 -0.00687 0.02386 -0.02519

AFIX 43

H87 2 1.071241 0.541557 0.390129 11.00000 -1.20000

AFIX 0

C88 1 1.012159 0.510281 0.405516 11.00000 0.06369 0.07327 =

0.11364 -0.00726 0.02158 -0.01496

AFIX 43

H88 2 1.003256 0.580183 0.400988 11.00000 -1.20000

PART 1

AFIX 0

C89 1 0.810869 0.426046 0.241579 51.00000 0.09504 0.09291 =

0.07700 -0.00740 0.00331 0.00274

AFIX 23

H89A 2 0.806058 0.364188 0.216628 51.00000 -1.20000

H89B 2 0.828872 0.474059 0.220379 51.00000 -1.20000

AFIX 0

C90 1 0.772662 0.479408 0.254050 51.00000 0.09237 0.12977 =

0.09558 0.00341 -0.02429 -0.00231

AFIX 137

H90A 2 0.755862 0.433870 0.277998 51.00000 -1.50000

H90B 2 0.759030 0.495004 0.217282 51.00000 -1.50000

H90C 2 0.777846 0.544615 0.275111 51.00000 -1.50000

AFIX 0

C91 1 0.825814 0.091918 0.282551 51.00000 0.08656 0.09468 =

0.09070 0.01007 0.00410 0.00155

AFIX 23

H91A 2 0.834189 0.065064 0.321153 51.00000 -1.20000

H91B 2 0.848164 0.081412 0.255044 51.00000 -1.20000

AFIX 0

C92 1 0.790151 0.029833 0.261540 51.00000 0.11067 0.08672 =

0.10983 0.00571 -0.00535 -0.01716

AFIX 137

H92A 2 0.768591 0.035546 0.290128 51.00000 -1.50000

H92B 2 0.797657 -0.043682 0.256993 51.00000 -1.50000

H92C 2 0.781169 0.057532 0.223927 51.00000 -1.50000

AFIX 0

O11 4 0.828913 0.394346 0.295062 51.00000 0.08715 0.08325 =

0.06847 -0.01150 -0.00321 0.00613

O12 4 0.817418 0.204519 0.287418 51.00000 0.06997 0.08550 =

0.07436 -0.01629 -0.00784 0.00019

P4 6 0.853299 0.279802 0.299868 51.00000 0.06633 0.10570 =

0.06055 -0.01349 0.00057 0.00383

S4 7 0.900434 0.265329 0.250186 51.00000 0.09207 0.11110 =

0.07377 -0.01217 0.02751 0.00215

PART 2

same .005 c89 > s4

C89A 1 0.803464 0.428778 0.264514 -51.00000 0.08986 0.09360 =

0.07738 -0.00851 -0.00004 0.00270

AFIX 23

H89C 2 0.822826 0.449391 0.234072 -51.00000 -1.20000

H89D 2 0.808070 0.472681 0.299429 -51.00000 -1.20000

AFIX 0

C90A 1 0.762390 0.443875 0.242882 -51.00000 0.09404 0.10933 =

0.08587 -0.00546 -0.01221 -0.00710

AFIX 137

H90D 2 0.762376 0.496580 0.211878 -51.00000 -1.50000

H90E 2 0.745368 0.467370 0.275007 -51.00000 -1.50000

H90F 2 0.752232 0.377673 0.227438 -51.00000 -1.50000

AFIX 0

C91A 1 0.832314 0.094034 0.260491 -51.00000 0.08447 0.09291 =

0.08414 -0.00240 0.00103 0.00153

AFIX 23

H91C 2 0.848574 0.030043 0.264941 -51.00000 -1.20000

H91D 2 0.841885 0.131301 0.225339 -51.00000 -1.20000

AFIX 0

C92A 1 0.789345 0.062563 0.251008 -51.00000 0.10487 0.08940 =

0.09961 0.00331 -0.00175 -0.00416

AFIX 137

H92D 2 0.788078 0.006681 0.221850 -51.00000 -1.50000

H92E 2 0.774154 0.123017 0.237183 -51.00000 -1.50000

H92F 2 0.778021 0.037643 0.287859 -51.00000 -1.50000

AFIX 0

O11A 4 0.808508 0.320657 0.278983 -51.00000 0.07801 0.09088 =

0.06952 -0.01045 -0.00164 -0.00117

O12A 4 0.838643 0.161119 0.311695 -51.00000 0.08002 0.10490 =

0.07558 -0.00925 -0.00062 0.00515

P4A 6 0.853299 0.279802 0.299868 -51.00000 0.06633 0.10570 =

0.06055 -0.01349 0.00057 0.00383

S4A 7 0.896449 0.286569 0.242232 -51.00000 0.06357 0.07996 =

0.07367 -0.00458 -0.00397 -0.00208

PART 0

N1 3 0.394090 0.473992 0.631451 11.00000 0.05050 0.06377 =

0.06584 -0.01733 0.00174 -0.00059

N2 3 0.868426 0.521752 0.636223 11.00000 0.05470 0.06171 =

0.07289 0.01630 -0.00232 -0.00109

N3 3 0.377625 0.729418 0.373039 11.00000 0.05752 0.06266 =

0.06951 0.00984 -0.00426 0.00021

N4 3 0.863560 0.278841 0.370585 11.00000 0.04585 0.06524 =

0.06586 -0.01364 0.00470 0.00120

O1 4 0.383699 0.195153 0.546036 11.00000 0.06784 0.04973 =

0.09953 -0.01478 0.00336 -0.00080

O4 4 0.866702 0.800465 0.548488 11.00000 0.08411 0.04671 =

0.10030 0.01526 -0.00398 0.00232

O7 4 0.377544 0.444059 0.454729 11.00000 0.07583 0.04513 =

0.10004 0.00729 -0.00070 0.00131

O10 4 0.870219 0.555342 0.461056 11.00000 0.06947 0.04549 =

0.08990 -0.00724 0.00378 -0.00158

F1 5 0.152526 0.417282 0.605795 11.00000 0.05172 0.17077 =

0.18607 -0.03755 0.00118 0.01323

F2 5 0.625280 0.358036 0.562280 11.00000 0.04810 0.16461 =

0.20848 -0.01273 -0.00435 -0.00583

F3 5 0.632768 0.583650 0.604084 11.00000 0.05244 0.15942 =

0.22168 -0.06356 0.01490 0.00245

F4 5 1.105588 0.624040 0.587498 11.00000 0.06113 0.17451 =

0.19973 0.00147 -0.00949 0.00518

F5 5 0.139225 0.633792 0.433778 11.00000 0.05756 0.14008 =

0.23298 0.01256 -0.01454 0.00680

F6 5 0.611525 0.660201 0.397307 11.00000 0.05747 0.14477 =

0.21394 -0.04631 0.01023 -0.00468

F7 5 0.632384 0.359652 0.418595 11.00000 0.05842 0.17685 =

0.18088 -0.01770 -0.02211 -0.00598

F8 5 1.105059 0.362916 0.400460 11.00000 0.04803 0.15102 =

0.17949 -0.00714 0.01247 -0.00230

HKLF 4

REM bmk2351b in Pna2(1)

REM wR2 = 0.1709, GooF = S = 1.027, Restrained GooF = 1.059 for all data

REM R1 = 0.0567 for 12652 Fo > 4sig(Fo) and 0.0705 for all 15591 data

REM 1389 parameters refined using 1317 restraints

END

WGHT 0.0952 2.3591

REM Highest difference peak 0.520, deepest hole -0.456, 1-sigma level 0.047

Q1 1 0.8541 0.2227 0.3020 11.00000 0.05 0.52

Q2 1 0.8171 0.2999 0.2889 11.00000 0.05 0.51

Q3 1 0.8292 0.4588 0.7229 11.00000 0.05 0.49

Q4 1 0.8324 0.1194 0.3161 11.00000 0.05 0.48

Q5 1 0.4122 0.8803 0.3095 11.00000 0.05 0.38

Q6 1 0.8864 0.2645 0.2999 11.00000 0.05 0.33

Q7 1 0.8496 0.6383 0.7133 11.00000 0.05 0.33

Q8 1 0.4148 0.7083 0.2780 11.00000 0.05 0.29

Q9 1 0.3694 0.5061 0.7020 11.00000 0.05 0.27

Q10 1 0.3479 0.7543 0.3029 11.00000 0.05 0.26

;

_shelx_res_checksum 70420

_shelx_hkl_file

;

0 1 0 0.00 0.01

0 2 0 0.20 0.05

0 3 0 0.32 0.07

0 4 0 729.15 4.67

0 4 0 711.94 4.72

0 5 0 0.00 0.08

0 5 0 0.00 0.08

0 6 0 2.04 0.22

0 6 0 2.15 0.22

0 7 0 -0.05 0.09

0 7 0 -0.02 0.11

0 8 0 1.23 0.21

0 8 0 0.82 0.17

0 9 0 0.08 0.09

0 9 0 0.06 0.09

0 9 0 0.06 0.05

0 9 0 -0.01 0.05

0 9 0 0.02 0.04

0 10 0 7.67 0.56

0 10 0 7.70 0.29

0 10 0 7.48 0.27

0 10 0 8.12 0.58

0 10 0 8.65 0.31

0 11 0 0.06 0.07

0 11 0 0.00 0.07

0 11 0 -0.06 0.06

0 12 0 0.99 0.11

0 12 0 1.21 0.14

0 13 0 0.06 0.05

0 13 0 0.05 0.06

0 14 0 0.89 0.10

0 -1 -1 0.50 0.04

0 1 1 0.53 0.03

0 1 1 0.48 0.04

0 -1 1 0.52 0.04

0 -2 -1 -0.01 0.02

0 2 1 0.08 0.04

0 -2 1 0.00 0.02

0 2 -1 0.02 0.03

0 3 1 1.88 0.13

0 3 -1 2.04 0.15

0 -3 -1 1.77 0.07

0 4 -1 0.01 0.06

0 4 1 0.01 0.06

0 4 1 0.05 0.07

0 5 1 0.92 0.13

0 5 1 0.98 0.14

0 5 -1 1.00 0.14

0 6 1 -0.13 0.13

0 6 1 0.09 0.11

0 6 -1 0.13 0.11

0 6 -1 -0.03 0.12

0 7 -1 0.42 0.13

0 7 1 0.57 0.14

0 7 1 0.44 0.13

0 7 -1 0.44 0.13

0 8 -1 -0.01 0.11

0 8 -1 -0.05 0.12

0 8 1 0.00 0.08

0 8 1 -0.11 0.13

0 9 -1 1.29 0.23

0 9 1 1.26 0.22

0 9 -1 1.45 0.24

0 9 1 1.33 0.23

0 9 1 1.25 0.12

0 9 -1 1.30 0.11

0 9 -1 1.23 0.11

0 9 1 1.12 0.10

0 9 -1 1.41 0.12

0 9 1 1.45 0.12

0 10 1 -0.02 0.13

0 10 -1 0.12 0.12

0 10 -1 0.07 0.13

0 10 -1 0.04 0.06

0 10 -1 -0.11 0.07

0 10 -1 0.10 0.08

0 10 1 0.02 0.07

0 10 1 -0.13 0.07

0 10 1 0.05 0.05

0 11 1 2.07 0.17

0 11 -1 1.75 0.14

0 11 -1 1.78 0.15

0 11 1 1.79 0.14

0 11 1 1.78 0.15

0 11 -1 1.87 0.16

0 12 -1 0.19 0.09

0 12 1 0.14 0.08

0 12 1 -0.01 0.06

0 12 -1 -0.02 0.05

0 12 -1 -0.01 0.07

0 12 1 0.09 0.08

0 13 1 0.17 0.06

0 13 -1 0.09 0.05

0 13 1 0.05 0.06

0 13 -1 0.08 0.07

0 14 -1 0.03 0.05

0 14 1 0.02 0.05

0 15 -1 0.27 0.05

0 0 2 25.01 0.36

0 -1 2 0.05 0.03

0 1 2 0.03 0.03

0 -2 2 12.04 0.26

0 2 2 12.21 0.22

0 -3 -2 0.03 0.02

0 3 2 0.02 0.04

0 3 -2 0.04 0.06

0 4 2 216.22 1.93

0 4 -2 219.93 2.03

0 -4 -2 210.14 1.39

0 4 2 213.56 1.89

0 5 2 0.11 0.08

0 5 -2 0.05 0.08

0 5 2 0.07 0.08

0 6 2 2.48 0.24

0 6 2 2.68 0.25

0 7 2 -0.02 0.10

0 7 -2 -0.06 0.09

0 7 2 0.08 0.09

0 7 -2 -0.17 0.14

0 8 2 68.12 1.44

0 8 -2 65.32 1.50

0 8 2 68.13 1.42

0 8 -2 67.93 1.53

0 9 -2 0.03 0.04

0 9 2 0.00 0.05

0 9 -2 0.05 0.05

0 9 2 0.00 0.05

0 9 -2 0.05 0.05

0 9 2 -0.09 0.06

0 9 -2 0.02 0.09

0 9 2 -0.05 0.11

0 9 -2 0.15 0.14

0 9 -2 0.02 0.05

0 10 -2 4.51 0.44

0 10 2 5.59 0.25

0 10 -2 4.86 0.23

0 10 2 5.51 0.24

0 10 -2 5.38 0.50

0 10 2 5.99 0.49

0 10 -2 4.38 0.21

0 10 2 5.98 0.25

0 10 -2 5.56 0.25

0 11 -2 0.00 0.06

0 11 2 0.07 0.07

0 11 2 0.00 0.06

0 11 -2 0.07 0.08

0 11 2 0.01 0.06

0 11 -2 -0.04 0.06

0 12 -2 2.81 0.17

0 12 2 2.52 0.18

0 12 -2 2.21 0.15

0 12 2 2.32 0.16

0 12 2 2.73 0.18

0 12 -2 2.46 0.18

0 13 -2 0.03 0.04

0 13 2 0.04 0.05

0 13 -2 -0.04 0.07

0 13 2 -0.02 0.06

0 14 2 0.20 0.06

0 14 -2 0.26 0.07

0 15 -2 0.09 0.04

0 0 3 0.04 0.04

0 -1 3 3.32 0.16

0 1 3 3.23 0.15

0 2 3 0.03 0.04

0 -2 -3 -0.03 0.05

0 -2 3 0.01 0.05

0 -3 3 3.30 0.17

0 -3 -3 3.19 0.16

0 4 3 -0.01 0.05

0 4 -3 -0.03 0.09

0 4 3 -0.02 0.05

0 -4 -3 -0.01 0.03

0 5 3 0.58 0.11

0 5 -3 0.54 0.12

0 5 3 0.67 0.12

0 6 -3 0.12 0.11

0 6 3 -0.10 0.12

0 6 3 0.12 0.11

0 7 -3 1.98 0.25

0 7 3 1.99 0.23

0 7 3 2.00 0.22

0 8 3 0.21 0.14

0 8 -3 -0.05 0.13

0 8 -3 -0.09 0.11

0 9 3 2.07 0.14

0 9 -3 2.05 0.13

0 9 -3 2.08 0.14

0 9 -3 2.06 0.15

0 9 3 2.14 0.28

0 9 3 2.01 0.15

0 9 -3 1.92 0.29

0 9 -3 1.84 0.28

0 9 -3 1.94 0.15

0 9 3 2.35 0.15

0 10 3 -0.04 0.09

0 10 -3 0.05 0.11

0 10 -3 0.20 0.13

0 10 -3 -0.13 0.07

0 10 3 -0.05 0.06

0 10 -3 -0.04 0.05

0 10 3 -0.05 0.06

0 10 3 0.08 0.07

0 10 -3 0.05 0.07

0 11 -3 0.66 0.12

0 11 -3 0.65 0.09

0 11 3 0.67 0.10

0 11 3 0.69 0.11

0 11 -3 0.47 0.07

0 11 3 0.71 0.11

0 12 -3 -0.07 0.05

0 12 -3 -0.14 0.09

0 12 3 -0.03 0.06

0 12 3 0.14 0.08

0 12 3 -0.08 0.07

0 13 -3 0.32 0.08

0 13 -3 0.35 0.06

0 13 3 0.34 0.07

0 13 3 0.41 0.09

0 14 -3 0.09 0.06

0 15 -3 0.12 0.04

0 0 4 14.07 0.37

0 -1 4 0.18 0.07

0 1 4 0.16 0.07

0 2 4 1143.85 6.65

0 -2 4 1187.33 6.80

0 -3 4 0.07 0.06

0 -3 -4 0.00 0.04

0 3 4 0.06 0.04

0 4 4 23.96 0.55

0 -4 -4 22.39 0.48

0 -5 -4 0.03 0.03

0 5 -4 0.06 0.08

0 5 4 0.04 0.07

0 6 4 25.68 0.72

0 6 -4 28.35 0.85

0 7 4 0.02 0.08

0 7 -4 -0.04 0.09

0 8 4 2.72 0.29

0 8 -4 3.79 0.39

0 8 -4 3.98 0.38

0 9 -4 0.05 0.05

0 9 -4 -0.02 0.06

0 9 4 -0.03 0.05

0 9 -4 0.04 0.04

0 9 4 0.00 0.05

0 9 4 -0.05 0.05

0 9 -4 -0.11 0.12

0 9 -4 0.01 0.09

0 9 4 0.08 0.10

0 9 -4 0.03 0.06

0 10 -4 1.70 0.30

0 10 -4 1.80 0.16

0 10 -4 1.96 0.14

0 10 4 2.31 0.30

0 10 4 1.91 0.16

0 10 -4 1.87 0.31

0 10 4 1.60 0.13

0 10 -4 1.68 0.12

0 10 4 2.02 0.15

0 11 4 0.01 0.06

0 11 4 -0.05 0.07

0 11 -4 0.05 0.07

0 11 -4 0.03 0.06

0 11 -4 -0.05 0.05

0 11 4 0.08 0.07

0 12 4 2.27 0.16

0 12 -4 2.29 0.14

0 12 4 2.66 0.18

0 12 4 2.63 0.18

0 12 -4 2.97 0.20

0 13 -4 -0.03 0.06

0 13 4 0.09 0.06

0 13 4 0.02 0.06

0 14 -4 0.24 0.06

0 15 -4 0.04 0.03

0 0 5 0.04 0.07

0 -1 5 23.52 0.56

0 1 5 24.18 0.55

0 -2 5 -0.01 0.07

0 2 5 0.01 0.07

0 -3 -5 7.56 0.32

0 3 5 7.48 0.30

0 -3 5 7.77 0.33

0 4 5 0.01 0.06

0 -4 -5 0.01 0.05

0 5 -5 2.30 0.24

0 -5 -5 2.42 0.17

0 5 5 2.66 0.22

0 6 -5 -0.01 0.11

0 6 5 -0.06 0.09

0 7 5 0.72 0.15

0 7 -5 0.77 0.19

0 8 5 0.07 0.10

0 8 -5 0.17 0.15

0 8 5 0.00 0.04

0 8 5 0.04 0.05

0 9 -5 0.79 0.09

0 9 5 0.86 0.10

0 9 5 0.83 0.10

0 9 -5 0.85 0.10

0 9 -5 0.75 0.21

0 9 -5 0.66 0.19

0 9 5 0.85 0.18

0 9 -5 0.82 0.11

0 9 5 1.01 0.10

0 10 -5 0.03 0.05

0 10 -5 -0.21 0.07

0 10 5 -0.10 0.07

0 10 5 -0.01 0.06

0 10 -5 -0.03 0.07

0 10 -5 0.09 0.13

0 10 -5 0.01 0.13

0 10 -5 0.13 0.09

0 10 5 0.08 0.07

0 11 5 0.18 0.07

0 11 5 0.35 0.09

0 11 -5 0.26 0.09

0 11 5 0.26 0.08

0 12 5 0.08 0.06

0 12 5 -0.01 0.06

0 12 5 0.00 0.06

0 13 5 0.09 0.06

0 13 -5 0.00 0.07

0 14 -5 -0.01 0.05

0 15 -5 0.13 0.04

0 0 6 9999.00 51.41

0 1 6 0.62 0.13

0 -1 6 1.03 0.18

0 2 6 72.30 1.08

0 -2 6 71.25 1.13

0 -3 6 0.01 0.07

0 3 6 -0.03 0.06

0 -3 -6 0.04 0.07

0 -4 6 79.45 1.26

0 -4 -6 77.77 1.17

0 -5 -6 0.02 0.04

0 6 -6 38.58 1.06

0 6 6 38.89 0.89

0 7 -6 -0.01 0.11

0 7 6 0.04 0.07

0 8 6 17.14 0.39

0 8 6 15.98 0.40

0 8 -6 17.58 0.83

0 8 -6 18.48 0.46

0 8 6 16.23 0.37

0 9 6 -0.06 0.05

0 9 -6 -0.03 0.04

0 9 6 0.01 0.05

0 9 -6 -0.10 0.08

0 9 -6 0.14 0.11

0 9 -6 0.02 0.06

0 9 6 0.05 0.05

0 10 -6 14.56 0.36

0 10 6 15.65 0.41

0 10 -6 15.23 0.44

0 10 6 14.60 0.41

0 10 -6 14.67 0.86

0 10 -6 15.27 0.87

0 10 -6 14.31 0.44

0 10 6 15.30 0.40

0 11 -6 -0.08 0.08

0 11 6 0.04 0.06

0 11 6 -0.07 0.07

0 11 6 0.01 0.06

0 12 6 0.99 0.12

0 12 6 0.97 0.11

0 12 -6 1.14 0.14

0 13 -6 -0.10 0.07

0 14 -6 2.18 0.13

0 15 -6 -0.01 0.02

0 0 7 -0.12 0.13

0 1 7 21.89 0.63

0 -1 7 22.06 0.65

0 -2 7 -0.21 0.13

0 2 7 -0.20 0.12

0 -3 7 1.97 0.21

0 3 7 2.06 0.19

0 -3 -7 2.31 0.21

0 -4 -7 0.06 0.07

0 -4 7 -0.14 0.12

0 -5 -7 1.77 0.19

0 6 -7 -0.05 0.10

0 7 -7 1.92 0.28

0 8 7 -0.01 0.04

0 8 7 0.01 0.04

0 8 -7 0.08 0.10

0 8 -7 0.06 0.07

0 8 7 0.02 0.04

0 9 -7 -0.03 0.04

0 9 7 0.01 0.05

0 9 7 -0.07 0.06

0 9 -7 -0.18 0.16

0 9 -7 0.16 0.14

0 9 -7 -0.02 0.07

0 9 7 -0.01 0.05

0 10 7 0.12 0.07

0 10 -7 -0.04 0.07

0 10 7 0.01 0.06

0 10 -7 0.16 0.14

0 10 -7 0.11 0.13

0 10 -7 0.15 0.09

0 10 7 0.04 0.06

0 11 -7 0.04 0.08

0 11 7 0.17 0.07

0 11 7 0.15 0.07

0 12 7 0.09 0.07

0 12 -7 0.11 0.09

0 12 7 0.10 0.07

0 13 -7 -0.12 0.07

0 14 -7 -0.04 0.04

0 15 -7 0.00 0.02

0 0 8 539.43 4.30

0 -1 8 0.14 0.11

0 1 8 0.23 0.11

0 2 8 7.52 0.40

0 -2 8 7.93 0.43

0 3 8 0.07 0.08

0 -3 -8 0.03 0.08

0 -3 8 0.00 0.10

0 -4 -8 105.40 1.62

0 -5 -8 0.11 0.09

0 6 -8 0.79 0.19

0 7 -8 -0.03 0.10

0 8 8 8.64 0.29

0 8 8 8.87 0.28

0 8 -8 8.52 0.62

0 8 -8 8.57 0.33

0 8 8 8.03 0.26

0 9 8 -0.03 0.05

0 9 8 -0.07 0.06

0 9 -8 0.04 0.10

0 9 -8 0.00 0.06

0 9 8 -0.08 0.06

0 10 8 14.08 0.40

0 10 -8 14.67 0.44

0 10 8 15.08 0.39

0 10 -8 14.66 0.46

0 11 8 0.12 0.06

0 11 -8 -0.04 0.07

0 11 8 0.05 0.06

0 12 8 1.00 0.11

0 12 8 1.03 0.11

0 12 -8 0.99 0.13

0 13 -8 0.07 0.06

0 14 -8 0.23 0.05

0 0 9 -0.01 0.10

0 -1 9 11.87 0.56

0 1 9 11.61 0.54

0 -2 9 -0.01 0.11

0 2 9 -0.06 0.10

0 -2 -9 -0.01 0.10

0 3 9 9.49 0.48

0 -3 9 9.07 0.50

0 -3 -9 9.17 0.49

0 -4 -9 0.12 0.11

0 -5 -9 2.82 0.28

0 7 -9 0.16 0.10

0 7 -9 0.20 0.07

0 8 9 -0.01 0.04

0 8 9 0.03 0.04

0 8 -9 0.09 0.14

0 8 -9 0.04 0.07

0 8 -9 -0.02 0.05

0 9 9 1.63 0.13

0 9 9 1.59 0.13

0 9 -9 1.82 0.17

0 9 -9 1.65 0.29

0 10 9 0.08 0.06

0 10 9 -0.01 0.06

0 10 -9 -0.01 0.08

0 11 9 0.56 0.09

0 11 -9 0.49 0.10

0 11 9 0.49 0.08

0 12 -9 0.04 0.07

0 12 9 0.14 0.05

0 13 -9 0.22 0.07

0 14 -9 0.00 0.04

0 0 10 31.04 0.94

0 1 10 0.03 0.12

0 -1 10 0.03 0.11

0 2 10 201.36 2.54

0 -2 -10 198.56 2.57

0 -3 10 -0.04 0.12

0 -3 -10 0.03 0.10

0 -4 -10 2.27 0.27

0 -5 -10 0.01 0.08

0 5 -10 0.10 0.09

0 7 10 0.02 0.03

0 7 -10 -0.08 0.10

0 7 -10 0.00 0.05

0 7 -10 0.02 0.05

0 8 10 24.51 0.47

0 8 10 23.31 0.48

0 8 -10 26.74 0.61

0 8 -10 21.13 0.52

0 8 -10 23.64 1.08

0 9 10 -0.01 0.05

0 9 10 0.01 0.04

0 9 -10 0.10 0.08

0 10 -10 2.03 0.18

0 10 10 2.65 0.16

0 10 10 2.54 0.16

0 10 -10 2.40 0.20

0 11 10 0.03 0.05

0 11 -10 -0.12 0.09

0 11 10 -0.01 0.05

0 12 -10 0.27 0.08

0 13 -10 0.11 0.06

0 14 -10 0.38 0.05

0 0 11 -0.01 0.15

0 -1 11 0.06 0.13

0 1 11 0.04 0.12

0 -2 11 0.25 0.17

0 2 11 -0.01 0.11

0 -2 -11 0.00 0.13

0 -3 11 9.78 0.58

0 -3 -11 10.71 0.60

0 -4 -11 -0.03 0.09

0 5 -11 2.12 0.27

0 -5 -11 2.18 0.28

0 6 -11 -0.03 0.06

0 7 11 0.33 0.06

0 7 -11 0.27 0.14

0 7 -11 0.33 0.08

0 7 -11 0.27 0.07

0 8 11 0.06 0.05

0 8 -11 0.10 0.11

0 8 -11 0.13 0.09

0 8 -11 -0.10 0.07

0 9 11 0.54 0.08

0 9 11 0.58 0.08

0 9 -11 0.55 0.11

0 9 -11 0.46 0.09

0 10 11 0.10 0.06

0 10 11 0.07 0.05

0 10 -11 0.00 0.08

0 11 11 0.24 0.06

0 11 -11 0.04 0.08

0 12 -11 0.02 0.06

0 13 -11 0.13 0.05

0 14 -11 -0.05 0.03

0 0 12 74.10 1.67

0 -1 12 -0.08 0.16

0 -1 -12 -0.03 0.12

0 1 12 -0.13 0.14

0 -2 12 9.20 0.60

0 -2 -12 8.72 0.57

0 -3 -12 -0.04 0.10

0 -4 -12 2.34 0.31

0 4 -12 2.13 0.29

0 -5 -12 0.04 0.08

0 5 -12 -0.06 0.09

0 6 -12 1.84 0.15

0 6 -12 1.76 0.15

0 7 12 0.03 0.04

0 7 -12 -0.09 0.13

0 7 -12 0.01 0.07

0 7 -12 -0.01 0.06

0 8 12 1.07 0.10

0 8 -12 1.06 0.26

0 8 -12 1.12 0.14

0 8 -12 0.72 0.11

0 9 12 0.00 0.04

0 9 -12 0.06 0.08

0 9 -12 -0.02 0.06

0 10 -12 2.53 0.20

0 11 -12 -0.17 0.09

0 12 -12 0.16 0.06

0 13 -12 -0.06 0.04

0 14 -12 0.68 0.06

0 0 -13 0.11 0.13

0 0 13 -0.06 0.15

0 -1 -13 16.34 0.81

0 -1 13 15.54 0.80

0 -2 -13 -0.16 0.18

0 -3 -13 0.22 0.12

0 3 -13 0.16 0.13

0 -4 -13 0.06 0.13

0 4 -13 0.06 0.11

0 5 -13 1.56 0.26

0 -5 -13 1.27 0.25

0 5 -13 1.47 0.14

0 5 13 1.41 0.14

0 5 -13 1.54 0.14

0 6 -13 -0.11 0.07

0 6 -13 -0.01 0.06

0 7 -13 0.10 0.11

0 7 -13 0.04 0.07

0 7 -13 0.11 0.06

0 8 -13 0.11 0.09

0 8 -13 -0.06 0.06

0 9 -13 1.21 0.13

0 9 -13 2.16 0.19

0 10 -13 0.12 0.10

0 11 -13 0.09 0.07

0 12 -13 -0.06 0.06

0 13 -13 0.12 0.04

0 0 -14 46.04 1.45

0 -1 -14 -0.16 0.17

0 1 -14 -0.11 0.14

0 -2 -14 26.22 1.11

0 2 -14 24.86 1.06

0 -3 -14 -0.07 0.17

0 3 -14 -0.05 0.12

0 -4 -14 13.28 0.80

0 4 -14 14.14 0.39

0 4 14 14.10 0.44

0 5 -14 -0.13 0.15

0 5 -14 0.04 0.06

0 5 -14 0.09 0.06

0 5 14 -0.02 0.07

0 6 -14 3.44 0.22

0 6 -14 3.56 0.22

0 7 -14 0.03 0.10

0 7 -14 -0.03 0.07

0 7 -14 -0.05 0.07

0 8 -14 9.33 0.37

0 8 -14 7.69 0.33

0 9 -14 -0.12 0.10

0 9 -14 -0.07 0.06

0 10 -14 13.93 0.43

0 11 -14 -0.05 0.07

0 12 -14 0.09 0.05

0 13 -14 -0.06 0.04

0 0 -15 -0.12 0.19

0 1 -15 3.56 0.44

0 -1 -15 2.97 0.40

0 2 -15 0.02 0.15

0 -2 -15 -0.05 0.15

0 2 15 0.03 0.06

0 3 -15 4.29 0.47

0 -3 -15 4.30 0.48

0 3 15 4.58 0.25

0 4 -15 0.01 0.14

0 -4 -15 -0.14 0.15

0 4 -15 0.10 0.07

0 4 15 0.05 0.06

0 5 -15 2.55 0.37

0 5 -15 2.97 0.20

0 5 -15 2.95 0.20

0 5 15 3.12 0.22

0 6 -15 -0.01 0.06

0 6 -15 -0.02 0.06

0 7 -15 -0.06 0.07

0 7 -15 -0.06 0.07

0 8 -15 0.03 0.07

0 8 -15 0.04 0.06

0 9 -15 0.06 0.08

0 9 -15 0.01 0.05

0 10 -15 0.14 0.09

0 11 -15 0.07 0.05

0 12 -15 -0.04 0.04

0 13 -15 0.18 0.04

0 0 -16 28.36 1.26

0 -1 -16 -0.04 0.13

0 1 -16 0.04 0.12

0 1 16 0.00 0.05

0 -2 -16 4.01 0.49

0 2 -16 3.39 0.45

0 2 16 4.92 0.26

0 -3 -16 0.01 0.13

0 3 -16 -0.16 0.15

0 3 -16 -0.03 0.06

0 3 16 -0.03 0.07

0 -4 -16 15.67 0.96

0 4 -16 16.48 0.44

0 4 16 18.05 0.53

0 5 -16 -0.03 0.07

0 5 16 -0.02 0.09

0 5 -16 -0.05 0.07

0 6 -16 31.13 0.65

0 6 -16 27.86 0.66

0 7 -16 0.00 0.07

0 7 -16 0.02 0.06

0 8 -16 2.74 0.21

0 8 -16 1.96 0.17

0 9 -16 -0.07 0.08

0 9 -16 -0.04 0.06

0 10 -16 0.24 0.09

0 11 -16 0.00 0.05

0 12 -16 0.40 0.06

0 0 -17 0.05 0.14

0 0 17 0.06 0.06

0 -1 -17 3.06 0.45

0 1 -17 3.11 0.44

0 1 17 3.64 0.23

0 2 -17 -0.10 0.18

0 -2 -17 -0.03 0.16

0 2 17 0.00 0.07

0 3 -17 0.87 0.23

0 -3 -17 1.08 0.30

0 3 17 1.00 0.14

0 -4 -17 -0.17 0.19

0 4 -17 0.16 0.12

0 4 -17 0.06 0.06

0 4 17 0.04 0.08

0 4 -17 0.08 0.07

0 5 -17 0.67 0.23

0 5 -17 0.73 0.11

0 5 -17 0.68 0.12

0 5 17 0.73 0.14

0 6 -17 0.15 0.09

0 6 -17 0.10 0.09

0 7 -17 0.21 0.09

0 7 -17 0.23 0.09

0 8 -17 0.26 0.10

0 8 -17 0.12 0.08

0 9 -17 0.56 0.09

0 9 -17 0.84 0.12

0 10 -17 0.11 0.07

0 11 -17 0.31 0.06

0 12 -17 -0.07 0.03

0 0 -18 36.93 1.57

0 0 18 38.64 0.74

0 1 -18 -0.09 0.16

0 -1 -18 -0.04 0.13

0 1 18 0.13 0.08

0 -1 18 -0.02 0.05

0 -2 -18 13.36 0.95

0 2 -18 12.55 0.91

0 2 18 14.95 0.48

0 3 -18 -0.12 0.13

0 -3 -18 -0.02 0.12

0 3 18 -0.04 0.09

0 -4 -18 7.56 0.71

0 4 -18 7.17 0.68

0 4 -18 8.18 0.31

0 4 18 8.77 0.39

0 4 -18 8.36 0.36

0 5 -18 -0.02 0.06

0 5 18 0.06 0.09

0 5 -18 -0.01 0.07

0 6 -18 0.88 0.12

0 6 -18 0.86 0.13

0 7 -18 0.11 0.07

0 7 -18 0.04 0.07

0 8 -18 4.99 0.26

0 8 -18 5.05 0.25

0 9 -18 0.16 0.07

0 9 -18 0.06 0.05

0 10 -18 0.26 0.07

0 11 -18 0.07 0.04

0 0 -19 0.07 0.12

0 0 19 -0.03 0.06

0 -1 -19 4.94 0.60

0 1 -19 4.20 0.55

0 -1 19 4.41 0.25

0 1 19 4.55 0.26

0 -2 19 0.14 0.07

0 2 19 0.01 0.08

0 3 19 0.02 0.09

0 4 -19 0.16 0.10

0 4 19 0.14 0.10

0 5 -19 0.31 0.07

0 5 -19 0.36 0.11

0 6 -19 0.01 0.06

0 6 -19 -0.05 0.07

0 7 -19 0.44 0.09

0 7 -19 0.50 0.11

0 8 -19 0.07 0.06

0 8 -19 0.08 0.06

0 9 -19 0.11 0.06

0 9 -19 0.12 0.05

0 10 -19 0.19 0.06

0 11 -19 0.33 0.05

0 0 20 8.19 0.35

0 1 20 -0.02 0.07

0 -1 20 -0.03 0.06

0 -2 20 0.48 0.09

0 2 20 0.57 0.12

0 3 20 0.15 0.10

0 3 -20 0.14 0.10

0 4 20 13.34 0.48

0 4 -20 12.00 0.43

0 5 -20 0.03 0.05

0 5 -20 0.01 0.08

0 6 -20 2.73 0.17

0 6 -20 3.02 0.21

0 7 -20 0.00 0.05

0 7 -20 -0.01 0.07

0 8 -20 4.78 0.22

0 8 -20 4.91 0.21

0 9 -20 -0.01 0.04

0 10 -20 0.30 0.06

0 0 21 0.02 0.06

0 -1 21 0.19 0.07

0 1 21 0.32 0.10

0 -2 21 0.07 0.06

0 2 21 0.05 0.08

0 3 21 1.41 0.17

0 3 -21 1.41 0.16

0 4 -21 0.24 0.11

0 5 -21 1.50 0.16

0 6 -21 0.03 0.04

0 6 -21 0.11 0.08

0 7 -21 0.03 0.04

0 7 -21 0.02 0.06

0 8 -21 0.05 0.05

0 8 -21 0.05 0.05

0 9 -21 0.06 0.04

0 10 -21 0.00 0.03

0 0 22 14.54 0.45

0 1 22 0.05 0.07

0 -1 22 0.01 0.06

0 2 -22 2.81 0.21

0 2 22 2.76 0.22

0 -2 22 2.60 0.18

0 3 22 -0.01 0.08

0 3 -22 0.06 0.09

0 4 -22 6.90 0.31

0 5 -22 0.01 0.08

0 6 -22 1.59 0.14

0 7 -22 0.06 0.06

0 8 -22 0.94 0.09

0 9 -22 0.05 0.03

0 0 23 0.04 0.05

0 1 23 0.75 0.11

0 1 -23 1.04 0.13

0 -1 23 0.72 0.10

0 2 -23 0.02 0.07

0 2 23 0.02 0.07

0 3 -23 0.38 0.10

0 4 -23 0.20 0.09

0 5 -23 1.03 0.12

0 6 -23 0.23 0.08

0 7 -23 0.08 0.05

0 8 -23 0.05 0.03

0 0 -24 4.15 0.22

0 0 24 3.84 0.21

0 1 -24 0.05 0.07

0 -1 24 0.00 0.04

0 1 24 -0.01 0.06

0 -1 -24 0.03 0.05

0 -2 -24 0.30 0.07

0 2 -24 0.35 0.09

0 -3 -24 0.17 0.06

0 3 -24 -0.01 0.06

0 4 -24 0.64 0.10

0 5 -24 0.12 0.07

0 6 -24 1.80 0.13

0 7 -24 0.04 0.04

0 0 -25 0.12 0.06

0 -1 -25 0.17 0.06

0 1 -25 0.22 0.07

0 -2 -25 0.09 0.05

0 2 -25 0.05 0.06

0 3 -25 0.14 0.07

0 -3 -25 0.21 0.05

0 4 -25 0.09 0.06

0 5 -25 0.29 0.06

0 6 -25 0.05 0.03

0 0 -26 -0.01 0.04

0 -1 -26 0.02 0.04

0 1 -26 -0.02 0.04

0 -2 -26 2.45 0.13

0 2 -26 2.68 0.15

0 3 -26 0.08 0.05

0 4 -26 0.70 0.08

0 5 -26 0.04 0.03

0 0 -27 0.01 0.03

0 -1 -27 -0.01 0.03

0 1 -27 0.06 0.04

0 2 -27 0.01 0.03

0 3 -27 0.10 0.04

-1 1 0 4.78 0.12

1 1 0 4.72 0.13

1 -1 0 4.78 0.12

1 2 0 7.97 0.23

-1 2 0 8.15 0.22

1 3 0 1.65 0.13

-1 3 0 1.61 0.12

1 4 0 22.36 0.55

-1 4 0 23.61 0.54

1 4 0 23.17 0.56

-1 5 0 0.51 0.11

1 5 0 0.63 0.12

1 5 0 0.65 0.13

-1 5 0 0.65 0.12

1 6 0 9.35 0.45

-1 6 0 8.64 0.42

-1 6 0 9.38 0.47

1 6 0 8.81 0.44

1 7 0 1.14 0.19

-1 7 0 1.12 0.18

-1 7 0 1.30 0.20

1 7 0 1.27 0.19

1 8 0 8.15 0.51

-1 8 0 7.90 0.49

1 8 0 8.75 0.52

-1 8 0 8.34 0.52

1 9 0 0.29 0.11

-1 9 0 0.33 0.13

1 9 0 0.26 0.13

-1 9 0 0.33 0.13

-1 9 0 0.21 0.06

-1 9 0 0.30 0.06

-1 9 0 0.26 0.07

1 9 0 0.26 0.07

1 9 0 0.21 0.06

1 9 0 0.18 0.05

-1 10 0 3.49 0.38

1 10 0 3.72 0.40

-1 10 0 3.12 0.18

-1 10 0 3.61 0.41

1 10 0 3.27 0.18

-1 10 0 3.46 0.20

1 10 0 3.41 0.37

1 10 0 3.51 0.20

-1 10 0 3.98 0.21

1 10 0 3.65 0.20

1 11 0 -0.03 0.06

1 11 0 0.01 0.06

-1 11 0 0.03 0.07

-1 11 0 -0.06 0.07

-1 11 0 -0.01 0.06

1 11 0 0.09 0.07

-1 12 0 4.29 0.21

1 12 0 4.38 0.21

1 12 0 4.83 0.24

-1 12 0 5.28 0.24

-1 12 0 5.00 0.25

1 12 0 4.76 0.24

-1 13 0 0.10 0.05

1 13 0 0.07 0.05

1 13 0 0.08 0.07

-1 13 0 0.18 0.08

-1 14 0 0.10 0.06

1 14 0 0.05 0.05

1 1 1 2.90 0.10

1 -1 -1 2.75 0.10

-1 1 1 2.95 0.09

-1 1 1 2.94 0.10

-1 2 1 154.15 1.21

1 -2 -1 144.45 1.04

1 2 1 148.08 1.21

-1 2 1 152.61 1.18

1 2 -1 155.95 1.26

-1 3 -1 2.85 0.17

1 3 -1 2.79 0.17

-1 3 1 2.97 0.16

1 -3 -1 2.62 0.11

1 -3 1 3.20 0.11

1 3 1 2.99 0.17

-1 4 1 16.78 0.49

-1 4 1 18.16 0.47

-1 4 -1 17.16 0.48

1 4 1 18.49 0.49

1 4 -1 16.63 0.48

1 4 1 17.78 0.48

-1 5 1 3.48 0.25

-1 5 -1 3.15 0.24

-1 5 1 3.39 0.24

1 5 1 3.20 0.24

1 5 1 3.46 0.24

1 5 -1 3.11 0.25

-1 6 1 37.33 0.90

1 6 -1 37.26 0.92

1 6 1 37.23 0.89

1 6 -1 38.26 0.92

-1 6 -1 37.76 0.91

-1 6 -1 38.44 0.96

-1 6 1 36.85 0.87

1 6 1 38.94 0.91

1 7 -1 1.73 0.23

-1 7 -1 1.69 0.22

-1 7 -1 1.65 0.23

-1 7 1 1.60 0.21

1 7 1 1.83 0.23

1 7 -1 1.68 0.23

1 7 1 1.80 0.22

-1 7 1 1.80 0.23

1 8 -1 11.11 0.60

-1 8 -1 11.40 0.62

1 8 1 11.46 0.60

-1 8 -1 10.70 0.58

-1 8 1 11.50 0.58

1 8 1 12.14 0.61

1 8 -1 10.58 0.59

-1 8 1 12.46 0.62

-1 9 -1 0.73 0.17

1 9 -1 0.61 0.18

-1 9 1 0.44 0.16

-1 9 1 0.55 0.15

1 9 1 0.63 0.17

1 9 1 0.68 0.18

1 9 -1 0.50 0.16

-1 9 -1 0.80 0.18

1 9 -1 0.66 0.09

-1 9 -1 0.63 0.08

-1 9 -1 0.73 0.09

1 9 1 0.59 0.08

1 9 -1 0.71 0.09

-1 9 1 0.62 0.09

-1 9 -1 0.72 0.09

-1 9 1 0.68 0.09

1 9 -1 0.63 0.08

1 9 1 0.48 0.07

-1 9 1 0.71 0.10

1 9 1 0.65 0.09

1 10 1 0.66 0.18

-1 10 -1 0.40 0.15

1 10 -1 0.66 0.19

-1 10 -1 0.65 0.20

-1 10 1 0.64 0.19

1 10 -1 0.42 0.17

-1 10 -1 0.42 0.07

1 10 -1 0.48 0.07

-1 10 1 0.46 0.08

1 10 1 0.62 0.08

-1 10 -1 0.78 0.11

1 10 -1 0.65 0.10

-1 10 -1 0.79 0.10

1 10 1 0.67 0.10

-1 10 1 0.65 0.10

1 10 1 0.68 0.10

-1 10 1 0.71 0.10

1 10 -1 0.84 0.11

-1 11 -1 -0.02 0.06

1 11 -1 0.01 0.07

-1 11 -1 -0.02 0.07

1 11 1 0.00 0.06

-1 11 -1 0.11 0.07

-1 11 1 0.05 0.06

1 11 -1 -0.02 0.05

-1 11 1 -0.09 0.07

-1 11 1 0.05 0.07

1 11 -1 0.09 0.06

1 11 1 0.04 0.06

1 11 1 -0.09 0.07

1 12 1 2.79 0.17

1 12 -1 3.56 0.20

-1 12 -1 2.90 0.17

1 12 1 3.24 0.20

-1 12 -1 3.76 0.20

-1 12 1 3.45 0.20

1 12 -1 2.99 0.17

-1 12 1 2.79 0.18

-1 12 -1 3.64 0.21

-1 12 1 3.26 0.20

1 12 1 3.16 0.20

1 12 -1 3.41 0.21

1 13 -1 0.14 0.05

-1 13 -1 0.07 0.04

-1 13 1 0.04 0.05

1 13 1 0.10 0.05

1 13 1 0.03 0.06

1 13 -1 0.01 0.07

-1 13 1 0.05 0.07

-1 13 -1 -0.05 0.07

1 14 1 0.08 0.05

-1 14 1 0.05 0.05

-1 14 -1 0.13 0.06

1 14 -1 0.08 0.06

1 15 -1 0.09 0.04

-1 15 -1 0.10 0.04

-1 0 2 -0.01 0.03

-1 1 2 2.52 0.09

1 -1 -2 2.23 0.11

-1 -1 2 2.49 0.12

1 2 2 434.07 2.76

-1 2 2 436.87 2.72

-1 -3 -2 1.37 0.08

-1 3 2 1.25 0.11

1 3 -2 1.39 0.13

1 3 2 1.39 0.07

1 -3 -2 1.10 0.08

1 3 2 1.27 0.12

1 -3 2 1.42 0.10

1 4 2 3.22 0.21

-1 4 2 3.74 0.22

-1 4 -2 3.50 0.23

1 4 -2 3.46 0.12

1 4 2 3.66 0.22

-1 4 2 3.50 0.21

1 -4 -2 3.04 0.14

1 4 -2 3.63 0.24

-1 5 2 1.98 0.20

1 5 2 1.61 0.18

-1 5 -2 1.78 0.19

-1 5 2 2.17 0.19

1 5 2 1.98 0.19

1 5 -2 1.94 0.20

1 6 2 95.80 1.46

-1 6 2 96.40 1.46

1 6 -2 93.07 1.51

1 6 2 93.24 1.44

-1 6 2 93.58 1.40

-1 6 -2 89.68 1.50

-1 7 2 2.22 0.26

1 7 2 2.27 0.25

1 7 2 2.25 0.25

-1 7 2 2.09 0.23

1 7 -2 2.12 0.26

-1 7 -2 2.14 0.26

1 7 -2 2.21 0.25

-1 7 -2 2.32 0.27

-1 8 2 2.97 0.30

1 8 2 3.38 0.33

-1 8 2 2.94 0.30

1 8 -2 2.64 0.31

1 8 2 3.29 0.32

1 8 -2 2.75 0.31

-1 8 -2 3.20 0.35

-1 8 -2 2.72 0.31

-1 9 2 0.27 0.06

-1 9 -2 0.30 0.07

1 9 2 0.32 0.07

1 9 -2 0.41 0.08

-1 9 -2 0.30 0.06

-1 9 2 0.39 0.07

-1 9 2 0.33 0.06

-1 9 -2 0.24 0.07

1 9 -2 0.24 0.07

1 9 2 0.30 0.07

1 9 2 0.25 0.06

1 9 -2 0.20 0.05

1 9 -2 0.29 0.13

-1 9 2 0.28 0.12

-1 9 -2 0.32 0.12

1 9 -2 0.10 0.11

1 9 2 0.38 0.13

-1 9 -2 0.27 0.13

1 9 -2 0.37 0.08

1 10 -2 5.30 0.49

-1 10 -2 4.83 0.46

-1 10 2 5.80 0.48

-1 10 -2 4.58 0.21

-1 10 -2 5.09 0.48

1 10 -2 5.19 0.48

1 10 2 5.83 0.47

1 10 -2 5.04 0.23

-1 10 2 5.31 0.24

1 10 2 5.38 0.25

-1 10 2 5.51 0.25

-1 10 -2 4.99 0.23

1 10 2 5.25 0.23

1 10 -2 4.64 0.21

-1 10 2 5.86 0.25

-1 10 -2 5.61 0.25

1 10 2 5.57 0.24

1 10 -2 5.34 0.25

1 11 -2 0.73 0.11

1 11 2 0.53 0.10

-1 11 -2 0.71 0.10

1 11 -2 0.51 0.08

1 11 -2 0.54 0.09

-1 11 -2 0.77 0.12

-1 11 2 0.43 0.08

-1 11 2 0.56 0.10

1 11 2 0.47 0.08

1 11 2 0.49 0.09

-1 11 -2 0.48 0.08

-1 11 2 0.47 0.09

-1 12 -2 0.46 0.08

1 12 -2 0.40 0.09

1 12 2 0.39 0.08

-1 12 2 0.56 0.10

-1 12 2 0.25 0.07

1 12 -2 0.41 0.07

1 12 -2 0.63 0.10

1 12 2 0.55 0.10

-1 12 -2 0.37 0.09

1 12 2 0.34 0.09

-1 12 2 0.39 0.10

-1 13 -2 0.08 0.05

1 13 2 0.07 0.05

-1 13 -2 0.00 0.07

-1 13 2 0.06 0.06

1 13 -2 0.08 0.04

1 13 2 0.13 0.07

1 13 -2 -0.02 0.07

-1 13 2 -0.01 0.06

1 13 2 0.06 0.07

1 14 -2 0.41 0.08

-1 14 -2 0.36 0.07

-1 14 2 0.40 0.07

1 14 2 0.42 0.07

1 15 -2 0.10 0.04

-1 15 -2 0.12 0.04

-1 0 3 0.00 0.04

1 -1 -3 0.85 0.09

-1 1 3 1.10 0.10

-1 -1 3 1.06 0.10

1 -2 -3 571.32 3.55

1 -2 3 572.87 3.56

-1 -2 3 572.49 3.57

1 2 3 563.08 3.44

-1 -3 -3 2.85 0.14

1 -3 -3 2.89 0.16

1 -3 3 2.92 0.16

1 3 3 2.92 0.13

1 4 -3 1.85 0.18

-1 4 3 2.11 0.16

1 -4 -3 1.64 0.11

-1 4 -3 1.83 0.17

1 -4 3 2.08 0.14

-1 -4 -3 1.65 0.10

1 4 3 2.21 0.17

1 4 3 2.12 0.17

-1 4 3 2.22 0.17

1 -5 -3 1.59 0.11

1 5 3 1.94 0.19

1 5 -3 1.48 0.09

-1 5 3 1.91 0.18

-1 5 -3 1.72 0.19

1 5 -3 1.69 0.19

1 5 3 1.81 0.18

-1 5 3 1.97 0.19

-1 6 3 25.20 0.70

1 6 -3 26.19 0.81

1 6 3 26.52 0.72

-1 6 -3 26.46 0.80

1 6 3 26.71 0.74

-1 6 3 26.75 0.75

-1 7 3 0.42 0.12

1 7 -3 0.29 0.13

-1 7 -3 0.42 0.14

1 7 -3 0.12 0.11

1 7 3 0.39 0.13

1 7 3 0.25 0.10

-1 7 3 0.21 0.10

-1 8 -3 12.69 0.66

1 8 -3 13.36 0.69

1 8 -3 13.95 0.70

-1 8 -3 13.68 0.70

-1 8 3 14.58 0.65

1 8 3 14.53 0.65

-1 9 -3 1.37 0.13

-1 9 3 1.35 0.12

1 9 -3 1.28 0.12

1 9 -3 1.15 0.10

-1 9 -3 1.35 0.12

-1 9 3 1.25 0.12

-1 9 3 1.72 0.26

1 9 3 1.16 0.11

-1 9 -3 1.19 0.11

1 9 3 1.35 0.12

1 9 -3 1.33 0.12

-1 9 -3 1.10 0.21

-1 9 -3 1.42 0.24

1 9 -3 1.22 0.23

1 9 3 1.38 0.22

1 9 -3 1.35 0.25

1 9 -3 1.31 0.13

-1 9 -3 1.20 0.13

-1 9 3 1.47 0.12

1 9 3 1.29 0.12

-1 10 -3 3.58 0.41

1 10 -3 3.78 0.42

-1 10 3 3.68 0.21

1 10 -3 3.63 0.20

1 10 -3 3.83 0.41

-1 10 -3 2.98 0.17

1 10 3 3.59 0.19

-1 10 -3 3.97 0.22

1 10 3 3.82 0.21

1 10 -3 3.18 0.17

-1 10 -3 3.51 0.19

1 10 -3 3.79 0.21

1 10 3 4.38 0.41

-1 10 3 3.87 0.39

-1 10 -3 3.79 0.42

-1 10 3 3.75 0.20

1 10 3 4.09 0.21

-1 10 3 4.01 0.21

1 11 3 0.04 0.05

-1 11 -3 0.05 0.06

-1 11 3 -0.03 0.07

1 11 -3 0.11 0.08

-1 11 3 0.07 0.07

1 11 3 0.09 0.07

1 11 -3 0.13 0.06

-1 11 -3 -0.04 0.06

1 11 -3 -0.03 0.05

-1 11 -3 0.16 0.08

1 11 3 0.07 0.07

-1 11 3 0.04 0.07

-1 12 3 2.08 0.16

1 12 3 1.72 0.14

-1 12 -3 1.59 0.12

1 12 3 1.85 0.16

1 12 -3 1.99 0.15

1 12 -3 1.59 0.12

-1 12 3 1.66 0.14

1 12 3 1.83 0.15

1 12 -3 1.51 0.14

-1 12 -3 1.60 0.15

-1 12 3 1.94 0.16

-1 13 3 0.14 0.06

1 13 -3 0.12 0.04

1 13 -3 0.08 0.07

-1 13 -3 0.11 0.05

1 13 3 0.19 0.06

1 13 3 0.32 0.08

-1 13 -3 0.12 0.07

1 13 3 0.12 0.07

-1 13 3 0.07 0.07

-1 14 -3 0.58 0.09

1 14 -3 0.66 0.09

1 15 -3 0.09 0.04

-1 15 -3 0.07 0.03

-1 0 4 0.01 0.06

-1 1 4 6.03 0.24

1 1 4 5.98 0.24

1 -1 4 6.00 0.25

-1 -1 4 6.22 0.26

-1 2 4 488.16 3.26

1 -2 -4 493.91 3.35

1 2 4 495.01 3.25

-1 -2 4 493.31 3.37

1 -2 4 493.19 3.36

-1 3 4 12.70 0.35

1 -3 -4 12.25 0.38

-1 -3 -4 11.79 0.35

1 -3 4 12.91 0.39

1 3 4 12.96 0.34

1 4 4 5.25 0.26

-1 4 4 5.43 0.26

1 -4 4 4.78 0.24

1 -4 -4 4.95 0.23

-1 -4 -4 4.90 0.21

-1 5 4 2.44 0.21

1 5 4 2.46 0.21

1 5 -4 2.36 0.15

-1 5 -4 2.26 0.22

1 5 -4 2.40 0.24

1 -5 -4 2.12 0.15

1 6 4 23.99 0.69

-1 6 4 22.72 0.68

-1 6 -4 23.81 0.79

1 6 -4 24.08 0.80

-1 7 4 1.76 0.22

1 7 4 1.78 0.22

-1 7 -4 1.51 0.23

1 7 -4 1.67 0.25

1 8 4 7.39 0.46

-1 8 4 7.52 0.47

-1 8 -4 7.42 0.52

-1 8 -4 6.91 0.51

1 8 -4 7.13 0.51

1 8 -4 7.40 0.52

-1 9 4 -0.03 0.05

1 9 4 0.04 0.05

-1 9 4 0.00 0.04

1 9 -4 -0.06 0.05

1 9 4 -0.02 0.05

-1 9 -4 0.05 0.05

-1 9 -4 0.00 0.04

1 9 -4 0.00 0.04

-1 9 4 -0.01 0.05

-1 9 -4 -0.02 0.05

1 9 -4 0.00 0.05

1 9 4 -0.03 0.06

1 9 4 0.01 0.09

-1 9 4 0.00 0.11

1 9 -4 -0.07 0.11

-1 9 -4 -0.05 0.11

1 9 -4 0.04 0.09

-1 9 -4 -0.15 0.14

1 9 -4 -0.02 0.05

-1 9 -4 -0.01 0.06

1 10 4 2.19 0.16

1 10 4 2.24 0.16

-1 10 4 2.36 0.30

1 10 -4 2.35 0.17

1 10 4 2.27 0.30

-1 10 4 2.22 0.16

1 10 -4 2.22 0.14

-1 10 4 2.09 0.15

-1 10 -4 2.03 0.14

-1 10 -4 2.29 0.15

-1 10 -4 2.31 0.33

-1 10 -4 2.31 0.17

1 10 -4 2.43 0.16

-1 10 -4 2.36 0.34

1 10 -4 2.24 0.33

1 10 -4 2.09 0.32

1 10 -4 2.02 0.17

-1 10 4 2.38 0.16

1 10 4 2.42 0.16

-1 11 -4 0.75 0.12

1 11 -4 0.73 0.12

-1 11 4 0.79 0.11

-1 11 -4 0.67 0.09

1 11 -4 0.63 0.08

-1 11 4 0.64 0.10

1 11 4 0.80 0.11

1 11 -4 0.78 0.10

1 11 4 0.69 0.10

1 11 4 0.76 0.11

-1 11 4 0.75 0.11

-1 12 -4 1.34 0.11

-1 12 4 1.52 0.14

-1 12 4 1.87 0.15

1 12 4 1.46 0.13

1 12 -4 1.35 0.11

1 12 4 1.55 0.14

1 12 4 1.50 0.14

-1 12 4 1.61 0.15

-1 12 -4 1.93 0.16

1 12 -4 1.95 0.16

-1 13 -4 -0.04 0.07

1 13 4 0.09 0.06

1 13 -4 0.01 0.07

-1 13 -4 0.06 0.04

-1 13 4 0.09 0.06

1 13 4 0.12 0.07

-1 13 4 0.09 0.06

1 13 4 -0.02 0.06

1 14 -4 0.94 0.10

-1 14 -4 0.89 0.09

-1 15 -4 0.11 0.04

1 15 -4 0.10 0.04

-1 0 5 0.05 0.08

1 0 5 0.06 0.07

1 1 5 9.95 0.35

-1 -1 5 9.83 0.36

-1 1 5 9.84 0.35

1 -1 5 9.86 0.36

-1 2 5 144.92 1.51

-1 -2 5 149.19 1.60

1 2 5 146.44 1.52

1 -2 5 147.70 1.59

-1 -3 5 4.52 0.26

1 -3 5 4.61 0.26

1 -3 -5 3.97 0.24

1 3 5 4.08 0.22

-1 -3 -5 4.16 0.23

1 -4 -5 4.86 0.26

-1 -4 -5 4.63 0.24

1 -4 5 4.92 0.27

-1 4 5 5.14 0.26

1 4 5 5.09 0.26

1 5 -5 3.24 0.20

-1 5 -5 3.05 0.27

1 5 -5 3.18 0.27

-1 -5 -5 2.75 0.17

1 -5 -5 2.84 0.19

1 5 5 2.91 0.22

-1 5 5 2.42 0.21

-1 6 -5 5.16 0.38

1 6 -5 5.01 0.38

-1 6 5 5.22 0.33

1 6 5 5.00 0.32

1 7 -5 1.55 0.24

-1 7 -5 1.68 0.24

-1 7 5 1.45 0.20

1 7 5 1.63 0.21

1 8 -5 10.65 0.64

-1 8 -5 10.58 0.63

1 8 -5 10.69 0.63

-1 8 5 9.89 0.29

-1 8 5 9.85 0.53

1 8 5 10.13 0.29

1 8 5 10.88 0.55

-1 8 5 10.48 0.29

1 8 5 10.22 0.29

-1 9 -5 0.50 0.08

1 9 5 0.45 0.08

1 9 -5 0.47 0.08

-1 9 -5 0.45 0.08

-1 9 5 0.44 0.08

-1 9 5 0.51 0.08

-1 9 -5 0.49 0.07

1 9 -5 0.54 0.08

1 9 5 0.49 0.08

-1 9 -5 0.31 0.15

1 9 -5 0.45 0.16

-1 9 -5 0.54 0.18

1 9 5 0.51 0.15

1 9 -5 0.49 0.18

-1 9 5 0.60 0.17

-1 9 -5 0.47 0.10

1 9 -5 0.54 0.09

1 9 5 0.59 0.08

-1 9 5 0.58 0.08

-1 10 -5 4.10 0.19

1 10 -5 4.67 0.24

-1 10 -5 4.28 0.20

1 10 5 4.86 0.24

-1 10 5 4.73 0.23

-1 10 -5 4.67 0.24

1 10 5 4.68 0.22

-1 10 5 4.89 0.23

1 10 -5 4.39 0.21

-1 10 -5 4.24 0.47

1 10 -5 4.64 0.48

1 10 -5 4.50 0.47

-1 10 -5 4.35 0.46

1 10 -5 4.33 0.24

1 10 5 4.87 0.23

-1 10 5 5.05 0.23

1 11 -5 0.24 0.07

1 11 -5 0.15 0.08

-1 11 5 0.16 0.07

1 11 5 0.14 0.07

1 11 5 0.21 0.08

-1 11 -5 0.11 0.04

-1 11 -5 0.26 0.09

-1 11 5 0.12 0.06

1 11 5 0.26 0.08

-1 11 5 0.22 0.08

1 12 5 0.33 0.08

-1 12 5 0.57 0.09

1 12 -5 0.51 0.11

-1 12 -5 0.60 0.11

1 12 5 0.48 0.09

-1 12 5 0.41 0.09

-1 12 5 0.32 0.09

1 12 5 0.30 0.08

1 13 -5 0.12 0.06

-1 13 -5 0.07 0.06

-1 13 5 0.20 0.07

1 13 5 0.20 0.07

-1 14 -5 0.45 0.07

1 14 -5 0.42 0.07

1 15 -5 0.00 0.03

-1 15 -5 0.05 0.03

1 0 6 1.30 0.24

-1 0 6 1.25 0.21

-1 1 6 45.72 0.84

1 -1 6 44.69 0.86

-1 -1 6 46.28 0.88

1 1 6 45.48 0.84

1 -2 -6 2.57 0.21

1 2 6 2.57 0.20

-1 2 6 2.53 0.20

1 -2 6 2.53 0.21

-1 -2 6 2.54 0.21

1 -3 -6 2.50 0.21

-1 -3 6 2.53 0.22

1 3 6 2.33 0.19

-1 -3 -6 2.59 0.20

1 -3 6 2.38 0.21

-1 -4 -6 7.26 0.34

1 -4 6 7.91 0.38

1 -4 -6 7.95 0.36

-1 -5 -6 0.33 0.08

1 -5 -6 0.29 0.08

1 5 -6 0.20 0.07

-1 5 6 0.37 0.10

1 6 -6 14.86 0.66

-1 6 -6 14.56 0.65

-1 6 6 14.67 0.54

1 7 -6 0.09 0.10

1 7 6 0.03 0.08

-1 7 6 -0.04 0.08

-1 7 -6 0.14 0.12

1 8 6 0.20 0.05

1 8 6 0.31 0.06

-1 8 6 0.34 0.07

-1 8 6 0.17 0.05

-1 8 -6 0.30 0.14

-1 8 6 0.47 0.15

1 8 -6 0.44 0.16

-1 8 -6 0.44 0.09

1 8 -6 0.34 0.09

-1 8 6 0.34 0.07

1 8 6 0.31 0.07

1 9 6 1.14 0.12

1 9 -6 1.07 0.10

-1 9 6 1.11 0.11

-1 9 -6 1.24 0.12

1 9 -6 1.03 0.11

-1 9 -6 1.00 0.10

1 9 6 1.25 0.11

-1 9 6 1.23 0.11

-1 9 -6 1.18 0.25

-1 9 -6 1.04 0.24

1 9 -6 0.74 0.22

1 9 -6 1.15 0.25

1 9 -6 0.97 0.12

-1 9 -6 1.09 0.13

1 9 6 1.23 0.11

-1 9 6 1.20 0.11

1 10 6 2.63 0.18

1 10 -6 2.65 0.16

1 10 6 2.80 0.18

-1 10 6 2.39 0.17

-1 10 -6 2.79 0.20

-1 10 6 2.82 0.18

1 10 -6 2.55 0.19

-1 10 -6 2.41 0.35

-1 10 -6 2.60 0.37

1 10 -6 2.55 0.37

1 10 -6 2.59 0.36

1 10 -6 2.41 0.19

-1 10 -6 2.89 0.21

1 10 6 2.78 0.18

-1 10 6 2.97 0.18

1 11 6 0.04 0.06

-1 11 6 0.10 0.06

1 11 6 0.09 0.06

-1 11 6 0.01 0.06

1 11 -6 0.03 0.07

-1 11 -6 0.05 0.07

-1 11 6 0.04 0.06

1 11 6 -0.03 0.06

1 12 6 1.61 0.13

-1 12 6 1.78 0.14

-1 12 6 1.86 0.15

1 12 6 1.61 0.14

-1 12 -6 1.91 0.16

1 12 -6 1.67 0.16

-1 13 6 0.19 0.06

1 13 6 0.18 0.07

-1 13 -6 -0.06 0.07

1 13 -6 -0.06 0.08

1 14 -6 0.06 0.05

-1 14 -6 0.07 0.05

-1 15 -6 0.01 0.02

1 15 -6 0.01 0.02

1 0 7 0.00 0.09

-1 0 7 -0.04 0.11

1 1 7 6.32 0.34

1 -1 7 6.29 0.35

-1 -1 7 6.15 0.35

-1 1 7 6.30 0.34

-1 2 7 52.80 0.99

1 -2 -7 51.69 1.01

-1 -2 7 51.64 1.02

1 2 7 52.07 0.98

1 -2 7 52.34 1.02

1 -3 7 1.30 0.16

1 -3 -7 1.36 0.18

-1 -3 7 1.31 0.17

1 3 7 1.24 0.16

-1 -3 -7 1.27 0.16

1 -4 -7 30.45 0.79

1 4 7 28.70 0.72

1 -4 7 29.49 0.80

-1 -4 -7 30.13 0.77

1 -5 -7 1.26 0.17

-1 -5 -7 1.00 0.15

1 5 -7 1.02 0.15

1 6 -7 9.51 0.55

-1 6 -7 9.30 0.54

-1 7 -7 0.09 0.10

1 7 -7 0.11 0.11

-1 8 7 1.32 0.11

-1 8 7 1.31 0.11

1 8 7 1.56 0.12

-1 8 -7 1.49 0.10

1 8 7 1.45 0.12

-1 8 -7 1.19 0.24

1 8 -7 1.57 0.27

-1 8 -7 1.70 0.15

1 8 -7 1.65 0.15

-1 8 7 1.39 0.11

1 8 7 1.30 0.11

-1 9 7 0.08 0.05

1 9 7 0.19 0.06

-1 9 7 0.11 0.05

1 9 -7 0.13 0.04

1 9 7 0.20 0.06

-1 9 -7 0.11 0.13

1 9 -7 0.14 0.10

1 9 -7 0.00 0.15

-1 9 -7 0.21 0.15

1 9 -7 0.23 0.08

-1 9 -7 0.16 0.07

-1 9 7 0.17 0.06

1 9 7 0.15 0.05

1 10 -7 2.60 0.19

-1 10 7 2.64 0.17

1 10 7 2.87 0.18

1 10 7 2.71 0.18

-1 10 -7 2.82 0.20

-1 10 7 3.07 0.19

-1 10 -7 2.92 0.39

1 10 -7 2.80 0.39

-1 10 -7 2.78 0.39

1 10 -7 2.57 0.38

1 10 -7 2.90 0.21

-1 10 -7 3.03 0.22

-1 10 7 2.92 0.18

1 10 7 2.68 0.17

-1 11 -7 0.06 0.07

-1 11 7 0.07 0.07

-1 11 7 0.09 0.06

1 11 -7 0.07 0.07

1 11 7 0.03 0.06

1 11 7 0.11 0.06

1 12 7 3.42 0.18

-1 12 7 3.26 0.18

-1 12 7 3.04 0.17

1 12 7 2.93 0.18

-1 12 -7 3.37 0.21

1 12 -7 3.42 0.21

-1 13 -7 -0.02 0.06

1 13 -7 -0.08 0.07

-1 14 -7 0.17 0.05

1 14 -7 0.30 0.06

-1 15 -7 -0.03 0.03

1 15 -7 -0.05 0.03

1 0 8 0.15 0.13

-1 0 8 0.00 0.10

1 1 8 1.80 0.21

-1 -1 8 1.86 0.22

-1 1 8 1.76 0.20

1 -1 8 1.93 0.22

1 -2 8 174.45 2.15

1 -2 -8 178.78 2.14

-1 2 8 173.73 2.08

1 2 8 174.95 2.08

-1 -2 8 173.55 2.15

1 -3 8 2.52 0.25

1 -3 -8 2.61 0.25

1 3 8 2.42 0.23

-1 -3 -8 2.64 0.25

-1 -3 8 2.70 0.26

-1 -4 -8 16.68 0.62

1 -4 -8 16.67 0.63

1 -4 8 16.47 0.64

1 5 -8 1.92 0.22

-1 -5 -8 1.86 0.21

1 -5 -8 2.01 0.22

1 6 -8 16.03 0.73

1 7 -8 0.22 0.15

-1 7 -8 0.14 0.11

-1 8 8 1.52 0.12

-1 8 8 1.55 0.12

1 8 8 1.47 0.12

1 8 8 1.60 0.12

1 8 -8 1.81 0.30

-1 8 -8 1.71 0.29

1 8 -8 1.85 0.16

-1 8 -8 1.85 0.16

-1 8 -8 1.74 0.14

1 8 8 1.37 0.11

-1 8 8 1.42 0.11

-1 9 8 0.70 0.09

1 9 8 0.79 0.10

-1 9 8 0.72 0.09

1 9 8 0.70 0.09

1 9 -8 0.78 0.20

1 9 -8 0.77 0.21

-1 9 -8 0.99 0.24

-1 9 -8 0.83 0.12

1 9 -8 0.66 0.11

1 9 8 0.72 0.09

-1 9 8 0.61 0.08

-1 10 -8 0.66 0.12

1 10 -8 0.73 0.12

-1 10 8 0.76 0.10

1 10 8 0.85 0.11

-1 10 8 0.84 0.11

1 10 8 0.68 0.11

1 10 -8 0.79 0.13

-1 10 -8 0.95 0.14

-1 11 8 0.73 0.10

-1 11 8 0.69 0.10

-1 11 -8 0.58 0.11

1 11 8 0.71 0.10

1 11 8 0.76 0.10

1 11 -8 0.75 0.12

1 11 -8 0.69 0.12

-1 12 8 2.05 0.14

1 12 8 2.15 0.15

-1 12 8 2.33 0.15

-1 12 -8 2.38 0.18

1 12 -8 2.44 0.18

1 13 -8 0.14 0.05

-1 13 -8 0.15 0.06

1 14 -8 0.09 0.04

-1 14 -8 0.12 0.05

1 0 9 -0.06 0.11

-1 0 9 -0.05 0.11

-1 -1 9 1.64 0.22

1 -1 9 1.80 0.23

1 1 9 1.59 0.21

-1 1 9 1.67 0.22

-1 2 9 100.24 1.64

1 -2 -9 103.21 1.69

1 2 9 100.82 1.63

1 -2 9 100.98 1.69

1 3 9 4.29 0.33

1 -3 -9 4.01 0.33

1 -3 9 4.42 0.35

-1 -3 -9 3.71 0.32

1 -4 9 14.70 0.65

-1 -4 -9 14.99 0.63

1 -4 -9 15.33 0.65

1 -5 -9 2.81 0.28

1 5 -9 2.19 0.24

-1 -5 -9 2.97 0.28

1 7 9 0.07 0.04

1 7 -9 0.09 0.05

-1 7 -9 0.08 0.06

-1 7 -9 0.02 0.14

1 7 -9 0.07 0.10

1 8 9 4.36 0.20

-1 8 9 4.02 0.19

1 8 9 4.39 0.21

-1 8 9 4.53 0.20

1 8 -9 4.73 0.25

-1 8 -9 5.09 0.26

-1 8 -9 3.86 0.22

-1 8 -9 4.53 0.47

1 8 -9 4.15 0.45

1 9 9 0.46 0.08

-1 9 9 0.51 0.08

-1 9 9 0.48 0.08

1 9 9 0.55 0.08

1 9 -9 0.54 0.10

-1 9 -9 0.51 0.10

-1 9 -9 0.56 0.18

1 9 -9 0.48 0.18

1 10 9 2.00 0.15

-1 10 -9 1.82 0.17

1 10 9 1.81 0.15

-1 10 9 1.69 0.14

-1 10 9 2.02 0.15

1 10 -9 1.80 0.17

-1 10 -9 2.11 0.19

1 10 -9 1.92 0.18

-1 11 9 0.23 0.06

1 11 -9 0.23 0.08

-1 11 -9 0.11 0.07

1 11 9 0.25 0.07

-1 11 9 0.20 0.07

1 11 9 0.25 0.07

1 11 -9 0.16 0.09

-1 12 9 0.69 0.09

1 12 9 0.82 0.09

-1 12 -9 0.87 0.12

1 12 -9 0.75 0.11

-1 13 -9 0.20 0.07

1 13 -9 0.22 0.07

1 14 -9 0.03 0.03

-1 14 -9 0.04 0.03

1 0 10 0.04 0.10

-1 0 10 -0.05 0.12

-1 1 10 0.29 0.13

-1 -1 10 0.33 0.14

1 -1 -10 0.36 0.14

1 -1 10 0.36 0.14

1 1 10 0.35 0.13

-1 -2 10 99.63 1.79

1 -2 -10 103.48 1.78

1 2 10 96.86 1.70

1 -2 10 98.37 1.78

1 3 10 13.68 0.61

1 -3 -10 13.99 0.65

-1 -3 -10 14.51 0.65

1 -3 10 14.53 0.67

1 -4 -10 6.82 0.46

1 4 -10 5.72 0.42

1 -4 10 5.94 0.44

-1 -4 -10 5.70 0.42

1 -5 -10 0.82 0.18

-1 -5 -10 0.72 0.17

1 5 -10 0.68 0.16

1 7 10 2.72 0.14

-1 7 10 2.61 0.14

1 7 -10 2.77 0.19

-1 7 -10 2.69 0.19

1 7 -10 2.38 0.17

-1 7 -10 2.35 0.17

-1 7 -10 2.38 0.33

1 7 -10 2.39 0.32

-1 8 10 4.10 0.19

-1 8 10 4.03 0.19

1 8 10 4.06 0.20

1 8 10 4.05 0.19

1 8 -10 3.27 0.20

1 8 -10 4.55 0.26

-1 8 -10 3.50 0.22

-1 8 -10 4.76 0.26

-1 8 -10 3.81 0.44

1 8 -10 4.35 0.47

1 9 10 0.62 0.09

1 9 10 0.62 0.09

-1 9 10 0.55 0.08

-1 9 10 0.60 0.08

-1 9 -10 0.56 0.10

-1 9 -10 0.74 0.12

1 9 -10 0.68 0.12

-1 9 -10 0.39 0.17

1 9 -10 0.64 0.18

-1 10 10 0.85 0.10

1 10 -10 0.71 0.11

-1 10 10 0.96 0.10

1 10 10 0.90 0.11

1 10 10 0.99 0.11

-1 10 -10 0.82 0.12

-1 10 -10 1.01 0.14

1 10 -10 0.93 0.14

1 11 -10 0.12 0.07

-1 11 10 0.25 0.06

1 11 10 0.14 0.06

-1 11 10 0.22 0.06

-1 11 -10 0.10 0.08

1 11 -10 0.33 0.11

-1 12 -10 0.11 0.07

1 12 10 0.15 0.05

1 12 -10 0.06 0.07

1 13 -10 0.23 0.07

-1 13 -10 0.19 0.06

1 14 -10 -0.04 0.03

-1 14 -10 -0.03 0.03

1 0 11 -0.07 0.13

-1 0 11 -0.16 0.15

1 -1 11 4.52 0.39

1 1 11 4.56 0.39

-1 -1 11 4.69 0.40

1 -1 -11 4.15 0.37

-1 1 11 4.18 0.38

1 -2 11 57.26 1.41

1 2 11 54.49 1.34

1 -2 -11 57.25 1.40

-1 -3 -11 2.05 0.27

1 -3 11 1.71 0.25

1 -3 -11 1.88 0.26

1 3 11 1.66 0.24

-1 -4 -11 14.78 0.71

1 4 -11 13.30 0.67

1 -4 -11 14.55 0.71

1 -5 -11 0.05 0.08

-1 -5 -11 0.10 0.09

1 5 -11 -0.03 0.08

-1 6 -11 3.12 0.19

1 6 -11 3.10 0.19

-1 6 11 3.38 0.21

1 7 11 0.07 0.04

-1 7 11 0.00 0.03

-1 7 -11 0.09 0.09

1 7 -11 0.02 0.09

-1 7 -11 0.16 0.07

1 7 -11 0.04 0.06

1 7 -11 -0.01 0.05

-1 7 -11 -0.03 0.06

-1 8 11 3.55 0.17

1 8 11 3.53 0.17

-1 8 -11 3.86 0.24

1 8 -11 2.67 0.19

1 8 -11 3.99 0.24

-1 8 -11 2.95 0.20

1 8 -11 3.00 0.40

-1 8 -11 3.15 0.41

-1 9 11 0.63 0.08

1 9 11 0.56 0.08

1 9 11 0.59 0.08

-1 9 -11 0.43 0.08

1 9 -11 0.65 0.12

-1 9 -11 0.78 0.13

1 10 11 0.55 0.09

-1 10 11 0.37 0.07

-1 10 11 0.47 0.08

-1 10 -11 0.51 0.12

1 10 -11 0.38 0.11

-1 11 -11 0.14 0.07

-1 11 11 0.23 0.06

1 11 -11 0.07 0.08

1 11 11 0.20 0.06

1 11 -11 0.25 0.10

1 12 -11 0.00 0.06

-1 12 -11 0.13 0.06

-1 13 -11 0.12 0.05

1 13 -11 0.06 0.05

-1 14 -11 0.16 0.04

1 14 -11 0.13 0.03

1 0 -12 0.04 0.15

1 0 12 0.05 0.13

1 -1 12 9.20 0.59

1 -1 -12 9.17 0.58

1 1 -12 8.85 0.57

1 1 12 8.85 0.57

-1 -2 -12 18.33 0.82

1 -2 12 17.62 0.82

1 2 -12 18.14 0.82

1 -2 -12 18.26 0.83

1 2 12 16.15 0.76

1 -3 -12 1.69 0.26

-1 -3 -12 1.39 0.24

1 3 -12 1.40 0.25

1 -3 12 1.52 0.25

-1 -4 -12 28.00 1.04

1 -4 -12 26.57 1.03

1 4 -12 24.93 0.97

1 -5 -12 0.48 0.16

1 5 -12 0.56 0.16

-1 5 -12 0.75 0.19

-1 -5 -12 0.78 0.19

1 6 -12 0.47 0.09

-1 6 -12 0.49 0.09

-1 6 12 0.46 0.09

1 6 -12 0.40 0.08

-1 6 -12 0.49 0.09

1 7 12 0.13 0.04

-1 7 12 0.16 0.05

1 7 -12 0.02 0.13

1 7 -12 0.20 0.08

-1 7 -12 0.10 0.07

-1 7 -12 0.11 0.07

1 7 -12 0.06 0.05

-1 8 12 3.09 0.16

1 8 12 3.02 0.15

-1 8 -12 2.21 0.18

-1 8 -12 3.11 0.22

1 8 -12 2.14 0.17

1 8 -12 3.08 0.22

-1 8 -12 2.36 0.37

1 8 -12 2.47 0.37

-1 9 12 0.42 0.07

1 9 -12 0.51 0.12

-1 9 -12 0.39 0.09

-1 9 -12 0.57 0.12

-1 10 -12 0.36 0.11

1 10 -12 0.34 0.11

-1 11 -12 -0.19 0.09

1 11 -12 -0.21 0.09

1 11 -12 0.04 0.07

1 12 -12 0.06 0.05

-1 12 -12 -0.01 0.06

-1 13 -12 -0.01 0.04

1 13 -12 0.07 0.04

1 14 -12 0.05 0.03

-1 14 -12 0.09 0.03

1 0 13 0.07 0.14

1 0 -13 0.00 0.12

-1 -1 -13 8.11 0.57

1 1 13 7.64 0.56

1 1 -13 7.64 0.56

1 -1 13 8.05 0.58

1 -1 -13 8.38 0.59

-1 -2 -13 8.04 0.58

1 -2 13 7.52 0.57

1 -2 -13 7.85 0.58

1 2 -13 6.56 0.52

1 2 13 7.18 0.54

1 -3 -13 0.20 0.13

1 3 -13 0.30 0.15

-1 -3 -13 -0.03 0.12

1 -4 -13 27.50 1.10

1 4 -13 24.63 1.02

-1 -4 -13 28.23 1.11

-1 -5 -13 0.27 0.15

-1 5 -13 0.23 0.14

1 -5 -13 0.27 0.13

1 5 -13 0.16 0.11

1 5 -13 0.38 0.09

-1 5 -13 0.43 0.08

1 5 13 0.22 0.06

-1 5 -13 0.35 0.08

1 5 -13 0.20 0.06

-1 5 13 0.28 0.07

-1 6 -13 1.50 0.14

-1 6 -13 1.20 0.13

1 6 -13 1.49 0.15

1 6 -13 1.18 0.13

-1 6 13 1.27 0.15

-1 7 13 0.18 0.05

-1 7 -13 0.16 0.13

1 7 -13 0.13 0.11

1 7 -13 0.19 0.08

-1 7 -13 0.26 0.08

-1 7 -13 0.21 0.07

1 7 -13 0.19 0.06

-1 8 13 0.63 0.08

1 8 -13 0.67 0.13

-1 8 -13 0.64 0.12

-1 8 -13 0.45 0.09

1 8 -13 0.47 0.09

-1 9 -13 0.59 0.11

-1 9 -13 0.34 0.08

1 9 -13 0.56 0.12

1 9 -13 0.31 0.08

1 10 -13 1.86 0.18

-1 10 -13 1.63 0.17

1 11 -13 -0.06 0.08

-1 11 -13 -0.07 0.08

1 11 -13 0.07 0.08

-1 12 -13 -0.01 0.05

1 12 -13 0.01 0.05

-1 13 -13 -0.02 0.04

1 13 -13 0.01 0.03

1 0 14 0.07 0.14

1 0 -14 -0.06 0.14

-1 0 -14 0.02 0.14

-1 -1 -14 1.06 0.25

1 -1 -14 1.10 0.25

1 -1 14 0.81 0.19

1 1 -14 1.06 0.23

1 1 14 0.66 0.19

1 -2 -14 8.15 0.63

-1 -2 -14 8.30 0.63

1 2 -14 8.38 0.62

1 -3 -14 1.34 0.27

-1 -3 -14 1.27 0.25

1 3 -14 0.84 0.19

-1 -4 -14 14.56 0.84

-1 4 -14 13.82 0.81

1 4 -14 14.50 0.83

1 -4 -14 15.20 0.86

1 4 -14 16.60 0.43

1 4 14 18.05 0.50

-1 4 14 16.79 0.48

1 5 -14 -0.11 0.10

-1 5 -14 0.04 0.11

1 -5 -14 -0.06 0.11

1 5 -14 0.03 0.06

-1 5 -14 0.03 0.05

1 5 14 -0.07 0.08

-1 5 14 -0.08 0.08

-1 5 -14 0.03 0.06

1 5 -14 0.06 0.06

1 6 -14 2.45 0.19

-1 6 -14 2.64 0.19

-1 6 -14 2.44 0.19

1 6 -14 2.67 0.20

-1 6 14 2.96 0.22

-1 7 -14 0.09 0.10

1 7 -14 0.20 0.14

1 7 -14 0.30 0.09

-1 7 -14 0.35 0.09

-1 7 -14 0.23 0.08

1 7 -14 0.26 0.08

-1 8 -14 1.27 0.15

-1 8 -14 1.73 0.17

1 8 -14 1.21 0.14

1 8 -14 1.75 0.18

-1 9 -14 0.83 0.12

1 9 -14 1.01 0.14

1 9 -14 0.57 0.09

-1 9 -14 0.54 0.10

-1 10 -14 1.28 0.16

1 10 -14 1.25 0.16

1 11 -14 0.23 0.07

-1 11 -14 0.17 0.06

1 12 -14 0.08 0.05

-1 12 -14 0.05 0.04

1 13 -14 -0.04 0.03

-1 13 -14 -0.05 0.04

-1 0 -15 -0.01 0.19

1 0 -15 0.12 0.18

-1 -1 -15 2.20 0.35

1 1 -15 2.09 0.34

1 -1 -15 2.59 0.39

-1 1 -15 2.24 0.35

-1 -2 -15 17.44 0.95

-1 2 -15 15.65 0.89

1 2 -15 15.92 0.90

1 -2 -15 17.57 0.96

1 2 15 18.04 0.48

-1 2 15 17.73 0.47

-1 -3 -15 1.92 0.33

1 -3 -15 1.77 0.33

-1 3 -15 1.49 0.29

1 3 -15 1.80 0.32

1 3 -15 1.68 0.14

1 3 15 1.99 0.17

-1 3 15 2.01 0.17

1 -4 -15 8.96 0.69

1 4 -15 8.14 0.65

-1 -4 -15 9.00 0.70

-1 4 -15 8.48 0.66

1 4 -15 9.12 0.33

-1 4 -15 9.30 0.32

1 4 15 10.20 0.39

-1 4 15 10.05 0.39

1 5 -15 1.54 0.29

-1 5 -15 1.63 0.30

1 5 -15 1.88 0.16

-1 5 -15 1.67 0.15

1 5 15 2.06 0.19

-1 5 15 1.74 0.17

1 5 -15 1.88 0.17

-1 5 -15 1.65 0.16

1 6 -15 2.21 0.19

1 6 -15 2.46 0.19

-1 6 -15 2.37 0.19

-1 6 -15 2.24 0.19

1 7 -15 0.23 0.08

-1 7 -15 0.21 0.08

1 7 -15 0.17 0.07

-1 7 -15 0.25 0.08

-1 8 -15 0.29 0.10

1 8 -15 0.33 0.11

-1 8 -15 0.23 0.09

1 8 -15 0.15 0.06

-1 9 -15 0.96 0.13

-1 9 -15 0.73 0.11

1 9 -15 1.06 0.14

1 9 -15 0.69 0.10

-1 10 -15 0.50 0.11

1 10 -15 0.42 0.11

-1 10 -15 0.17 0.06

-1 11 -15 -0.08 0.06

1 11 -15 -0.08 0.06

-1 12 -15 0.04 0.04

1 12 -15 -0.04 0.04

-1 13 -15 0.08 0.03

1 13 -15 0.09 0.03

-1 0 -16 0.05 0.16

1 0 -16 -0.07 0.14

1 0 16 0.15 0.07

1 -1 -16 0.57 0.20

-1 1 -16 0.90 0.26

1 1 -16 0.72 0.23

-1 -1 -16 0.54 0.21

-1 1 16 0.59 0.10

1 1 16 0.82 0.11

1 2 -16 17.11 0.98

-1 2 -16 16.57 0.95

-1 -2 -16 18.59 1.02

1 -2 -16 17.67 1.00

1 2 16 19.35 0.51

-1 2 16 19.06 0.52

-1 -3 -16 0.64 0.22

1 -3 -16 0.62 0.20

1 3 -16 0.12 0.11

-1 3 -16 0.54 0.21

1 3 -16 0.53 0.09

1 3 16 0.69 0.12

-1 3 16 0.54 0.10

1 -4 -16 2.15 0.37

-1 4 -16 2.04 0.36

1 4 -16 1.99 0.34

-1 -4 -16 2.17 0.36

-1 4 -16 2.60 0.18

1 4 -16 2.61 0.18

1 4 16 2.32 0.20

-1 4 -16 2.24 0.19

-1 4 16 2.41 0.21

1 5 -16 1.87 0.35

-1 5 -16 2.32 0.38

1 5 -16 2.29 0.18

-1 5 -16 2.17 0.17

-1 5 16 2.28 0.20

1 5 -16 2.24 0.19

-1 5 -16 2.29 0.19

1 5 16 2.22 0.20

1 6 -16 5.82 0.29

-1 6 -16 5.31 0.27

1 6 -16 5.18 0.28

-1 6 -16 5.13 0.28

-1 7 -16 0.71 0.12

1 7 -16 0.96 0.14

-1 7 -16 0.77 0.12

1 7 -16 0.74 0.12

1 8 -16 2.10 0.18

-1 8 -16 2.79 0.20

-1 8 -16 2.30 0.19

1 8 -16 2.73 0.20

1 9 -16 0.14 0.09

-1 9 -16 0.16 0.09

-1 9 -16 0.02 0.05

1 9 -16 0.10 0.05

-1 10 -16 0.28 0.09

-1 10 -16 0.06 0.05

1 10 -16 0.32 0.09

-1 11 -16 0.13 0.06

1 11 -16 0.15 0.06

-1 12 -16 0.15 0.04

1 12 -16 0.24 0.05

-1 0 -17 -0.10 0.15

1 0 -17 -0.05 0.16

-1 0 17 0.07 0.06

1 0 17 0.01 0.05

-1 1 -17 0.44 0.17

1 -1 -17 0.74 0.26

1 1 -17 0.50 0.22

-1 -1 -17 0.47 0.19

1 -1 17 0.76 0.10

1 1 17 0.79 0.12

-1 1 17 0.71 0.11

1 -2 -17 4.10 0.51

-1 -2 -17 4.16 0.52

-1 2 -17 4.05 0.50

1 2 -17 3.99 0.50

-1 2 17 4.78 0.27

1 2 17 4.76 0.27

1 3 -17 0.30 0.18

-1 3 -17 0.20 0.16

1 -3 -17 0.06 0.14

-1 -3 -17 0.13 0.16

1 3 -17 0.34 0.07

-1 3 17 0.47 0.10

1 3 17 0.44 0.10

-1 -4 -17 2.20 0.37

1 4 -17 2.39 0.39

1 -4 -17 2.34 0.39

-1 4 -17 2.07 0.36

1 4 -17 2.77 0.19

-1 4 -17 2.71 0.18

-1 4 17 3.00 0.23

-1 4 -17 2.57 0.20

1 4 17 3.16 0.23

1 5 -17 -0.01 0.14

-1 5 -17 -0.25 0.16

1 5 -17 0.01 0.07

-1 5 -17 0.08 0.06

-1 5 17 0.03 0.09

1 5 17 0.04 0.09

-1 5 -17 0.07 0.07

1 5 -17 0.08 0.07

1 6 -17 4.76 0.27

-1 6 -17 5.05 0.26

-1 6 -17 4.90 0.28

1 6 -17 5.29 0.27

1 7 -17 0.21 0.08

-1 7 -17 0.24 0.08

-1 7 -17 0.10 0.08

1 7 -17 0.30 0.09

1 8 -17 7.12 0.31

-1 8 -17 6.90 0.30

-1 8 -17 5.94 0.29

1 8 -17 5.63 0.28

1 9 -17 0.46 0.10

1 9 -17 0.39 0.08

-1 9 -17 0.45 0.10

-1 9 -17 0.47 0.09

1 10 -17 0.26 0.08

-1 10 -17 0.19 0.05

-1 11 -17 0.06 0.05

1 11 -17 0.04 0.04

1 12 -17 0.18 0.04

-1 12 -17 0.20 0.04

1 0 -18 0.08 0.22

-1 0 -18 0.04 0.15

-1 0 18 0.20 0.09

1 0 18 0.20 0.09

1 -1 -18 0.44 0.22

-1 -1 -18 0.43 0.20

-1 1 -18 0.26 0.16

1 1 -18 0.41 0.19

-1 1 18 0.48 0.10

1 1 18 0.48 0.10

1 -1 18 0.40 0.08

1 2 -18 1.71 0.34

1 -2 -18 1.37 0.31

-1 2 -18 1.13 0.31

-1 -2 -18 1.29 0.31

1 2 18 1.80 0.18

-1 2 18 1.90 0.19

1 -2 18 1.93 0.16

-1 3 -18 1.95 0.37

1 3 -18 1.78 0.35

-1 -3 -18 1.89 0.36

1 -3 -18 2.20 0.41

-1 3 18 2.49 0.21

1 3 18 2.20 0.20

1 4 -18 3.07 0.45

1 -4 -18 2.26 0.40

-1 4 -18 2.64 0.42

1 4 -18 3.02 0.19

-1 4 18 3.03 0.24

1 4 18 2.86 0.23

1 4 -18 3.07 0.22

-1 4 -18 3.09 0.23

-1 5 -18 0.68 0.10

1 5 -18 0.57 0.10

-1 5 18 0.69 0.13

-1 5 -18 0.45 0.11

1 5 -18 0.48 0.11

-1 6 -18 0.40 0.08

1 6 -18 0.53 0.10

1 6 -18 0.41 0.10

-1 6 -18 0.51 0.11

1 7 -18 0.68 0.11

-1 7 -18 0.69 0.11

1 7 -18 0.70 0.12

-1 7 -18 0.73 0.12

-1 8 -18 2.81 0.19

-1 8 -18 2.73 0.20

1 8 -18 3.00 0.20

1 8 -18 2.65 0.19

-1 9 -18 0.06 0.05

-1 9 -18 0.09 0.07

1 9 -18 0.03 0.05

1 9 -18 0.07 0.07

1 10 -18 0.79 0.10

-1 10 -18 0.83 0.10

1 11 -18 0.02 0.04

-1 11 -18 0.07 0.04

-1 0 -19 0.06 0.14

1 0 -19 0.12 0.13

-1 0 19 -0.12 0.08

1 0 19 0.01 0.06

1 -1 -19 2.14 0.40

-1 1 -19 2.06 0.39

1 1 -19 2.06 0.40

-1 -1 -19 1.84 0.37

1 -1 19 2.17 0.18

-1 -1 19 2.40 0.19

1 1 19 2.20 0.19

-1 1 19 2.23 0.19

1 2 19 1.85 0.19

-1 2 19 2.04 0.20

-1 -2 19 1.76 0.16

1 -2 19 1.79 0.16

-1 3 19 0.45 0.11

1 3 19 0.44 0.11

-1 3 -19 0.60 0.12

1 4 -19 5.91 0.27

1 4 19 6.41 0.33

1 4 -19 5.85 0.31

-1 4 -19 6.17 0.31

-1 4 19 6.84 0.35

-1 5 -19 0.08 0.06

1 5 -19 0.13 0.07

-1 5 19 0.26 0.11

-1 5 -19 0.09 0.08

1 5 -19 0.16 0.09

-1 6 -19 0.50 0.09

1 6 -19 0.60 0.11

-1 6 -19 0.46 0.11

1 6 -19 0.49 0.11

-1 7 -19 0.35 0.08

1 7 -19 0.38 0.10

-1 7 -19 0.31 0.10

1 7 -19 0.33 0.09

1 8 -19 0.67 0.10

-1 8 -19 0.71 0.10

1 8 -19 0.70 0.10

-1 8 -19 0.75 0.11

1 9 -19 0.16 0.06

-1 9 -19 0.02 0.04

-1 9 -19 0.17 0.06

1 9 -19 0.08 0.04

-1 10 -19 0.29 0.06

1 10 -19 0.20 0.06

-1 11 -19 0.12 0.04

1 11 -19 0.11 0.04

-1 0 20 0.06 0.08

1 0 20 -0.02 0.06

-1 -1 20 0.31 0.09

1 1 20 0.24 0.09

1 -1 20 0.23 0.08

-1 1 20 0.31 0.09

-1 -2 20 1.11 0.13

-1 2 20 1.21 0.16

1 -2 20 1.16 0.13

1 2 20 1.27 0.15

1 3 20 0.53 0.12

-1 3 -20 0.59 0.12

-1 3 20 0.54 0.12

-1 4 20 7.01 0.35

-1 4 -20 6.29 0.32

1 4 -20 6.04 0.31

1 4 20 6.80 0.34

1 5 -20 0.08 0.06

1 5 -20 0.00 0.08

-1 5 -20 0.16 0.09

1 6 -20 2.86 0.18

-1 6 -20 3.14 0.22

-1 6 -20 2.55 0.17

1 6 -20 2.87 0.21

1 7 -20 0.16 0.06

-1 7 -20 0.16 0.06

-1 7 -20 0.20 0.08

1 7 -20 0.23 0.08

1 8 -20 0.32 0.07

-1 8 -20 0.21 0.06

-1 8 -20 0.29 0.08

1 8 -20 0.26 0.07

-1 9 -20 -0.01 0.04

1 9 -20 -0.01 0.04

-1 10 -20 0.64 0.07

1 10 -20 0.64 0.07

-1 0 21 -0.02 0.07

1 0 21 -0.01 0.06

-1 -1 21 0.37 0.09

-1 1 21 0.33 0.10

1 -1 21 0.33 0.08

1 1 21 0.29 0.09

1 2 21 0.55 0.12

-1 2 21 0.69 0.14

-1 2 -21 0.55 0.13

-1 -2 21 0.55 0.10

-1 3 21 0.02 0.09

-1 3 -21 0.07 0.09

1 3 -21 0.18 0.10

1 3 21 0.11 0.09

-1 4 -21 2.59 0.21

-1 4 21 3.11 0.24

1 4 -21 2.52 0.21

-1 5 -21 0.31 0.10

1 5 -21 0.25 0.09

1 6 -21 0.30 0.07

1 6 -21 0.30 0.09

-1 6 -21 0.28 0.10

1 7 -21 -0.03 0.05

-1 7 -21 0.03 0.05

1 7 -21 0.09 0.06

-1 7 -21 0.13 0.07

1 8 -21 0.23 0.06

-1 8 -21 0.24 0.07

1 8 -21 0.25 0.06

-1 9 -21 0.12 0.04

1 9 -21 0.11 0.04

-1 10 -21 0.20 0.04

1 10 -21 0.15 0.04

-1 0 22 0.13 0.08

1 0 22 0.12 0.08

1 1 22 0.15 0.08

-1 1 22 0.00 0.07

-1 1 -22 0.26 0.09

1 -1 22 0.07 0.06

-1 -1 22 0.17 0.08

-1 -2 22 1.63 0.15

-1 2 22 1.72 0.18

1 2 -22 1.78 0.17

1 2 22 1.60 0.16

-1 2 -22 2.06 0.19

-1 3 22 0.16 0.10

-1 3 -22 0.14 0.09

1 3 -22 0.11 0.09

-1 4 -22 1.69 0.18

1 4 -22 1.86 0.17

1 5 -22 0.19 0.09

-1 5 -22 0.21 0.09

-1 6 -22 0.22 0.08

1 6 -22 0.25 0.08

1 7 -22 0.27 0.06

1 7 -22 0.21 0.06

-1 7 -22 0.22 0.07

-1 8 -22 0.12 0.05

1 8 -22 0.14 0.05

-1 9 -22 0.31 0.05

1 9 -22 0.29 0.05

-1 0 23 -0.01 0.06

-1 0 -23 0.12 0.08

1 0 23 -0.05 0.06

1 -1 23 -0.01 0.05

-1 1 -23 0.01 0.07

1 1 -23 0.09 0.08

-1 1 23 0.00 0.06

-1 -1 23 0.02 0.05

1 1 23 0.03 0.06

-1 2 -23 0.88 0.13

1 2 -23 0.75 0.12

-1 2 23 0.70 0.11

-1 -2 23 0.56 0.09

1 3 -23 0.10 0.08

-1 3 -23 0.00 0.07

-1 4 -23 1.36 0.14

1 4 -23 1.17 0.14

1 5 -23 0.17 0.08

-1 5 -23 0.11 0.07

-1 6 -23 0.45 0.09

1 6 -23 0.34 0.08

1 7 -23 0.22 0.06

-1 7 -23 0.14 0.06

-1 8 -23 0.96 0.08

1 8 -23 0.81 0.07

-1 0 -24 0.14 0.07

-1 0 24 -0.03 0.05

1 0 24 0.06 0.06

1 0 -24 0.22 0.08

1 1 -24 0.02 0.06

-1 -1 24 0.01 0.05

-1 -1 -24 0.05 0.05

-1 1 24 -0.01 0.06

-1 1 -24 0.03 0.06

-1 -2 -24 0.12 0.06

-1 2 -24 0.12 0.07

1 2 -24 0.09 0.07

-1 3 -24 0.34 0.09

-1 -3 -24 0.27 0.06

1 3 -24 0.28 0.09

1 4 -24 0.10 0.07

-1 4 -24 0.10 0.07

1 5 -24 0.25 0.07

-1 5 -24 0.32 0.07

-1 6 -24 0.20 0.06

1 6 -24 0.15 0.06

-1 7 -24 0.16 0.04

1 7 -24 0.16 0.04

1 0 -25 0.08 0.06

-1 0 -25 0.11 0.06

-1 -1 25 0.01 0.04

1 -1 -25 0.10 0.06

-1 -1 -25 0.13 0.06

1 1 -25 0.09 0.06

-1 1 -25 0.07 0.06

1 -2 -25 0.07 0.05

1 2 -25 0.10 0.07

-1 2 -25 0.10 0.07

-1 -2 -25 0.08 0.05

-1 3 -25 0.20 0.07

1 3 -25 0.28 0.07

1 -3 -25 0.22 0.05

-1 4 -25 0.57 0.09

1 4 -25 0.53 0.08

-1 5 -25 0.09 0.05

1 5 -25 0.08 0.05

-1 6 -25 0.32 0.06

1 6 -25 0.30 0.05

1 0 -26 -0.06 0.05

-1 0 -26 -0.01 0.04

-1 1 -26 0.09 0.05

-1 -1 -26 0.12 0.05

1 -1 -26 0.06 0.04

1 1 -26 0.06 0.05

-1 2 -26 0.20 0.06

1 -2 -26 0.10 0.04

1 2 -26 0.15 0.06

-1 -2 -26 0.09 0.04

-1 3 -26 0.34 0.06

1 3 -26 0.43 0.07

1 4 -26 1.35 0.10

-1 4 -26 1.44 0.10

1 5 -26 0.03 0.03

-1 5 -26 0.01 0.03

1 0 -27 0.02 0.03

-1 0 -27 0.00 0.03

1 -1 -27 0.00 0.03

-1 1 -27 0.04 0.03

1 1 -27 0.02 0.03

-1 -1 -27 -0.02 0.03

-1 2 -27 0.07 0.04

1 2 -27 0.02 0.03

-1 3 -27 0.02 0.03

1 3 -27 0.07 0.04

2 1 0 0.87 0.05

2 1 0 0.88 0.06

2 -1 0 0.85 0.06

2 1 0 0.93 0.05

-2 1 0 0.90 0.05

2 2 0 3.09 0.15

2 -2 0 3.02 0.10

2 -2 0 3.08 0.13

-2 2 0 3.02 0.13

2 2 0 3.00 0.14

2 3 0 0.05 0.05

2 3 0 0.01 0.02

-2 4 0 0.40 0.10

2 4 0 0.48 0.11

2 4 0 0.14 0.08

-2 5 0 0.02 0.07

2 5 0 0.00 0.08

2 5 0 0.02 0.09

-2 5 0 0.08 0.07

-2 6 0 -0.04 0.13

-2 6 0 -0.02 0.11

2 6 0 0.01 0.12

2 6 0 -0.03 0.13

-2 7 0 1.59 0.21

-2 7 0 1.71 0.23

2 7 0 1.46 0.20

2 7 0 1.43 0.21

2 8 0 0.36 0.13

-2 8 0 0.39 0.14

2 8 0 0.33 0.12

-2 8 0 0.38 0.13

2 9 0 2.83 0.32

-2 9 0 2.61 0.31

2 9 0 2.81 0.16

-2 9 0 2.96 0.17

-2 9 0 2.77 0.34

-2 9 0 2.98 0.17

2 9 0 2.99 0.33

2 9 0 2.89 0.18

2 9 0 2.95 0.17

-2 9 0 2.96 0.17

2 10 0 0.33 0.15

-2 10 0 0.42 0.16

-2 10 0 0.09 0.10

2 10 0 0.22 0.12

-2 10 0 0.38 0.09

2 10 0 0.25 0.07

2 10 0 0.51 0.10

2 10 0 0.14 0.05

-2 10 0 0.16 0.07

-2 10 0 0.27 0.07

-2 11 0 0.14 0.07

-2 11 0 -0.02 0.07

2 11 0 0.02 0.05

2 11 0 0.09 0.06

2 11 0 0.04 0.07

-2 11 0 -0.01 0.07

2 12 0 0.26 0.09

2 12 0 0.09 0.05

-2 12 0 0.19 0.07

2 12 0 0.16 0.08

-2 12 0 0.11 0.08

-2 13 0 0.37 0.07

2 13 0 0.60 0.10

2 13 0 0.43 0.07

2 13 0 0.34 0.08

-2 13 0 0.34 0.08

-2 14 0 0.07 0.04

2 14 0 0.03 0.05

-2 14 0 0.02 0.05

-2 1 1 12.37 0.21

2 -1 1 11.91 0.23

2 -1 -1 12.35 0.25

2 1 1 12.02 0.22

-2 1 1 11.81 0.18

2 1 1 11.70 0.22

2 1 1 12.21 0.20

2 2 1 484.62 3.02

-2 2 1 480.86 2.93

2 2 -1 500.61 2.74

2 2 1 482.36 2.79

2 2 -1 505.05 3.08

2 -2 -1 475.50 2.94

-2 2 1 486.96 3.00

2 2 1 477.67 2.97

2 3 -1 45.55 0.71

-2 3 -1 45.85 0.68

2 3 1 45.58 0.69

-2 3 1 43.66 0.63

2 -3 -1 42.48 0.40

2 -3 -1 43.10 0.57

-2 4 1 286.92 2.31

2 4 -1 279.65 2.40

2 -4 1 293.05 1.70

-2 4 1 284.15 2.37

2 4 1 279.62 2.36

-2 4 -1 282.02 2.35

2 4 1 272.13 2.31

-2 5 1 13.41 0.46

2 5 -1 14.27 0.51

2 5 -1 13.70 0.49

2 5 1 14.67 0.50

-2 5 1 14.14 0.50

-2 5 -1 13.30 0.48

2 5 1 13.63 0.48

2 6 -1 378.01 3.43

-2 6 1 396.51 3.47

-2 6 -1 393.22 3.55

-2 6 -1 387.37 3.39

-2 6 1 370.20 3.27

2 6 -1 387.48 3.48

2 6 1 382.78 3.38

2 6 1 387.57 3.36

2 7 1 8.28 0.46

2 7 -1 8.24 0.48

-2 7 1 8.00 0.44

2 7 -1 8.60 0.48

-2 7 -1 7.85 0.47

-2 7 -1 8.19 0.47

-2 7 1 8.93 0.48

2 7 1 8.92 0.47

2 8 -1 30.59 1.00

-2 8 -1 30.10 0.97

-2 8 -1 31.77 1.03

2 8 1 32.17 0.99

-2 8 1 31.30 0.95

2 8 1 32.06 0.98

-2 8 1 33.42 1.03

2 8 -1 31.24 0.99

-2 9 1 0.97 0.20

-2 9 -1 0.94 0.22

2 9 1 0.96 0.20

-2 9 -1 1.18 0.22

2 9 1 1.08 0.20

2 9 -1 1.22 0.23

-2 9 1 1.19 0.22

2 9 -1 1.31 0.24

-2 9 1 1.04 0.11

2 9 -1 1.15 0.11

2 9 -1 1.09 0.10

2 9 1 0.98 0.10

-2 9 -1 1.16 0.11

-2 9 -1 1.18 0.11

2 9 1 1.15 0.12

-2 9 1 1.16 0.11

-2 9 1 1.16 0.11

2 9 1 1.17 0.11

-2 9 -1 1.07 0.11

2 9 -1 1.08 0.11

2 10 -1 2.93 0.36

-2 10 -1 2.78 0.33

-2 10 1 2.38 0.16

2 10 1 2.62 0.18

2 10 1 2.43 0.15

-2 10 -1 2.56 0.16

2 10 -1 2.53 0.16

2 10 -1 2.97 0.19

-2 10 1 2.64 0.34

2 10 1 2.50 0.32

-2 10 -1 2.56 0.35

-2 10 -1 2.80 0.17

-2 10 1 2.79 0.18

2 10 -1 2.70 0.34

2 10 1 2.58 0.17

-2 10 -1 3.19 0.19

-2 10 1 2.76 0.18

2 10 -1 3.12 0.19

-2 11 1 0.06 0.07

-2 11 1 0.24 0.08

2 11 -1 0.14 0.05

-2 11 -1 0.28 0.08

-2 11 -1 0.04 0.07

2 11 1 0.14 0.06

2 11 1 0.13 0.08

2 11 -1 0.18 0.08

2 11 1 0.14 0.07

2 11 -1 -0.01 0.08

-2 11 1 -0.09 0.08

-2 11 -1 0.04 0.06

2 12 -1 5.71 0.25

-2 12 1 5.61 0.25

2 12 1 5.30 0.23

-2 12 -1 4.91 0.22

-2 12 1 6.60 0.26

2 12 1 6.24 0.27

2 12 -1 4.91 0.21

2 12 -1 5.79 0.26

-2 12 -1 5.76 0.26

2 12 1 6.14 0.27

-2 12 1 6.44 0.27

2 13 -1 0.14 0.05

2 13 1 0.21 0.07

-2 13 -1 0.06 0.05

-2 13 1 0.05 0.05

2 13 1 0.12 0.05

-2 13 1 0.07 0.06

2 13 1 0.03 0.07

-2 13 -1 0.07 0.07

2 13 -1 0.00 0.06

-2 14 1 0.21 0.05

-2 14 -1 0.15 0.04

2 14 -1 0.08 0.06

-2 14 -1 0.06 0.05

2 14 1 0.13 0.06

-2 14 1 0.15 0.06

2 0 -2 0.02 0.03

2 -1 -2 0.74 0.08

2 1 -2 0.80 0.07

-2 1 2 0.78 0.05

2 -2 -2 23.45 0.42

2 2 2 24.08 0.42

-2 2 2 24.00 0.37

2 2 -2 25.23 0.38

-2 2 2 24.65 0.40

-2 -3 -2 1.11 0.07

2 3 -2 1.15 0.12

2 3 -2 1.19 0.08

2 -3 -2 0.97 0.09

2 3 2 1.11 0.11

-2 3 2 1.08 0.10

2 4 -2 0.94 0.13

-2 4 2 0.80 0.10

-2 4 2 0.87 0.12

2 4 2 0.87 0.11

2 4 2 0.73 0.11

2 -4 -2 0.67 0.08

2 -4 2 0.94 0.09

-2 4 -2 0.83 0.12

2 4 -2 0.86 0.05

2 5 2 1.21 0.15

-2 5 -2 1.21 0.16

-2 5 2 1.23 0.14

2 5 -2 1.19 0.17

2 5 2 1.11 0.15

-2 5 2 1.20 0.15

-2 6 2 7.57 0.38

2 6 -2 8.29 0.44

-2 6 -2 8.17 0.44

2 6 -2 8.89 0.47

2 6 2 7.87 0.41

2 6 2 8.00 0.42

-2 6 2 7.95 0.42

-2 7 -2 2.18 0.25

2 7 2 2.54 0.26

-2 7 2 2.15 0.23

2 7 -2 2.21 0.26

-2 7 -2 2.54 0.29

2 7 -2 2.33 0.25

-2 7 2 2.44 0.26

2 7 2 2.55 0.26

-2 8 -2 0.58 0.17

-2 8 -2 0.54 0.15

-2 8 2 0.73 0.17

-2 8 2 0.66 0.17

2 8 -2 0.42 0.13

2 8 2 0.54 0.15

2 8 -2 0.39 0.14

2 8 2 0.74 0.18

-2 9 -2 2.01 0.14

2 9 -2 1.94 0.14

-2 9 2 1.74 0.26

2 9 -2 1.83 0.27

-2 9 2 1.90 0.14

2 9 -2 1.94 0.13

2 9 -2 1.81 0.14

-2 9 -2 1.92 0.15

-2 9 -2 1.76 0.13

2 9 2 1.85 0.13

2 9 2 1.81 0.14

-2 9 2 2.07 0.15

2 9 2 1.98 0.26

-2 9 -2 1.80 0.28

-2 9 -2 1.72 0.27

2 9 -2 2.11 0.30

2 9 -2 1.64 0.14

-2 9 2 2.07 0.14

2 9 2 1.97 0.14

2 10 -2 0.50 0.18

2 10 2 0.41 0.15

-2 10 2 0.38 0.16

-2 10 -2 0.20 0.11

-2 10 -2 0.38 0.15

2 10 -2 0.66 0.20

-2 10 -2 0.50 0.10

-2 10 2 0.49 0.10

2 10 2 0.20 0.06

-2 10 -2 0.33 0.08

2 10 -2 0.53 0.10

-2 10 -2 0.42 0.08

2 10 -2 0.16 0.05

2 10 -2 0.35 0.08

-2 10 2 0.12 0.06

2 10 2 0.29 0.08

-2 10 2 0.45 0.09

2 10 2 0.25 0.07

2 11 -2 0.75 0.10

-2 11 -2 0.76 0.12

-2 11 -2 0.47 0.08

2 11 2 0.66 0.10

-2 11 -2 0.72 0.10

-2 11 2 0.56 0.09

-2 11 2 0.71 0.11

-2 11 2 0.77 0.11

2 11 -2 0.67 0.09

2 11 -2 0.78 0.12

2 11 2 0.84 0.12

2 11 2 0.65 0.10

2 12 -2 0.04 0.07

-2 12 -2 -0.13 0.08

-2 12 2 0.32 0.09

2 12 2 0.10 0.08

2 12 2 0.01 0.05

2 12 -2 0.19 0.08

-2 12 -2 0.03 0.05

-2 12 2 -0.07 0.07

2 12 -2 0.03 0.04

2 12 2 0.06 0.07

-2 12 2 0.05 0.07

2 13 -2 0.26 0.08

2 13 2 0.35 0.08

2 13 2 0.44 0.08

-2 13 -2 0.29 0.08

-2 13 2 0.31 0.07

-2 13 -2 0.31 0.06

-2 13 2 0.35 0.09

2 13 2 0.32 0.08

-2 14 2 0.17 0.05

-2 14 2 -0.02 0.05

2 14 -2 0.02 0.05

-2 14 -2 0.00 0.05

2 14 2 0.06 0.05

-2 15 -2 0.15 0.04

2 15 -2 0.18 0.04

2 0 -3 208.00 1.64

2 -1 -3 32.44 0.55

2 1 -3 34.52 0.54

2 -2 -3 285.32 2.15

-2 2 3 290.33 1.96

2 3 3 55.89 0.66

2 -3 -3 52.39 0.75

2 -3 3 55.78 0.76

2 3 -3 55.62 0.70

-2 3 3 53.02 0.75

-2 -4 -3 24.93 0.38

-2 4 3 26.80 0.55

2 4 3 26.11 0.58

-2 4 -3 24.90 0.61

2 -4 3 27.00 0.51

2 4 -3 25.48 0.64

2 4 3 27.11 0.38

2 -4 -3 23.05 0.49

2 4 -3 25.88 0.44

2 4 3 25.42 0.56

-2 4 3 27.05 0.59

2 5 3 3.53 0.25

-2 5 3 3.34 0.23

-2 5 -3 3.67 0.27

2 5 -3 3.42 0.27

2 -5 -3 3.22 0.18

2 5 3 3.32 0.23

-2 5 3 3.63 0.25

2 6 3 10.98 0.47

-2 6 3 10.58 0.44

2 6 -3 12.39 0.55

-2 6 -3 11.93 0.53

2 6 3 11.23 0.47

-2 6 3 10.88 0.48

2 7 -3 2.75 0.30

2 7 -3 2.72 0.29

-2 7 -3 2.64 0.28

2 7 3 2.55 0.26

-2 7 3 2.77 0.27

2 7 3 2.60 0.26

2 8 -3 20.55 0.85

-2 8 -3 20.13 0.82

2 8 -3 20.91 0.84

-2 8 -3 20.86 0.86

2 8 3 20.32 0.76

-2 8 3 20.29 0.77

-2 9 -3 2.14 0.14

2 9 -3 2.32 0.15

-2 9 3 2.16 0.15

2 9 3 2.39 0.29

2 9 -3 2.30 0.30

2 9 3 2.17 0.16

-2 9 3 2.13 0.28

-2 9 -3 2.03 0.14

2 9 -3 1.98 0.13

2 9 3 2.05 0.14

-2 9 3 2.15 0.15

2 9 -3 2.10 0.15

-2 9 -3 2.37 0.16

-2 9 -3 2.00 0.29

-2 9 -3 2.05 0.30

2 9 -3 2.01 0.29

2 9 -3 2.05 0.16

2 9 3 2.17 0.15

-2 9 3 2.28 0.15

2 10 -3 2.62 0.36

-2 10 -3 2.75 0.35

2 10 -3 2.98 0.37

2 10 -3 2.93 0.19

2 10 3 2.57 0.33

-2 10 3 2.29 0.16

2 10 3 2.48 0.17

-2 10 3 2.48 0.17

-2 10 -3 3.10 0.40

2 10 3 2.41 0.16

-2 10 -3 3.22 0.20

-2 10 -3 2.74 0.17

-2 10 -3 2.75 0.17

2 10 -3 3.00 0.18

2 10 -3 2.60 0.15

-2 10 3 2.74 0.34

-2 10 3 2.68 0.18

2 10 3 2.60 0.17

2 11 3 0.91 0.11

2 11 3 0.82 0.11

2 11 -3 0.83 0.09

-2 11 -3 0.75 0.09

2 11 -3 0.93 0.11

-2 11 3 0.94 0.12

-2 11 3 0.71 0.10

-2 11 3 0.95 0.12

-2 11 -3 0.87 0.12

2 11 -3 0.91 0.12

2 11 3 0.83 0.12

2 12 3 1.83 0.14

-2 12 3 1.85 0.15

-2 12 3 2.13 0.16

2 12 -3 1.93 0.15

2 12 -3 1.54 0.12

-2 12 -3 1.69 0.13

2 12 3 2.05 0.17

2 12 3 1.67 0.15

-2 12 3 1.96 0.16

2 12 -3 1.78 0.15

-2 12 -3 1.83 0.15

-2 13 -3 0.00 0.07

2 13 3 0.13 0.07

2 13 -3 0.08 0.07

-2 13 -3 0.05 0.04

-2 13 3 0.15 0.06

-2 13 3 0.06 0.07

2 13 3 0.07 0.06

-2 14 -3 0.14 0.06

2 14 -3 0.16 0.06

-2 15 -3 0.12 0.04

2 15 -3 0.10 0.04

2 0 -4 0.79 0.10

-2 0 4 0.74 0.10

2 -1 -4 4.72 0.23

2 -2 -4 5.17 0.25

-2 3 4 9.32 0.30

2 3 4 8.98 0.29

2 -3 4 8.83 0.33

2 -3 -4 8.63 0.33

2 -4 -4 0.48 0.08

-2 4 4 0.64 0.11

2 -4 4 0.56 0.09

2 4 4 0.54 0.10

2 4 4 0.58 0.07

-2 -4 -4 0.52 0.07

2 4 -4 0.65 0.09

2 5 -4 0.16 0.05

-2 5 4 0.13 0.08

2 -5 4 0.14 0.05

2 5 4 0.17 0.09

-2 5 -4 0.16 0.09

2 -5 -4 0.23 0.07

2 5 -4 0.19 0.10

2 6 4 0.41 0.11

-2 6 -4 0.43 0.14

2 6 -4 0.44 0.14

-2 6 4 0.40 0.12

-2 7 -4 0.51 0.15

-2 7 4 0.65 0.16

2 7 -4 0.45 0.16

2 7 4 0.64 0.15

2 8 -4 0.30 0.16

-2 8 -4 0.13 0.15

2 8 4 0.10 0.10

2 8 -4 0.09 0.11

-2 8 -4 0.22 0.14

-2 8 4 0.09 0.11

-2 9 4 0.43 0.08

2 9 4 0.33 0.06

-2 9 -4 0.38 0.08

2 9 -4 0.29 0.05

2 9 -4 0.36 0.07

2 9 4 0.46 0.08

2 9 4 0.34 0.07

-2 9 -4 0.41 0.07

2 9 -4 0.35 0.08

-2 9 -4 0.50 0.08

-2 9 4 0.28 0.07

-2 9 4 0.27 0.06

2 9 4 0.51 0.15

-2 9 -4 0.29 0.15

2 9 -4 0.51 0.17

-2 9 -4 0.39 0.15

2 9 -4 0.26 0.13

-2 9 4 0.40 0.13

2 9 -4 0.32 0.08

-2 9 -4 0.43 0.09

2 10 -4 0.22 0.07

2 10 4 0.13 0.07

-2 10 4 0.04 0.06

-2 10 -4 0.08 0.07

-2 10 -4 0.08 0.05

2 10 -4 0.12 0.08

2 10 -4 0.07 0.04

2 10 4 0.02 0.06

-2 10 -4 0.15 0.06

-2 10 4 0.21 0.07

-2 10 4 0.07 0.08

2 10 -4 0.18 0.14

-2 10 -4 -0.02 0.12

-2 10 -4 0.10 0.11

2 10 -4 0.06 0.10

2 10 4 0.14 0.12

2 10 -4 0.16 0.08

-2 10 4 0.07 0.07

2 10 4 0.18 0.08

2 11 4 0.55 0.09

-2 11 -4 0.37 0.07

2 11 4 0.61 0.10

-2 11 -4 0.52 0.11

-2 11 4 0.57 0.10

2 11 -4 0.55 0.08

2 11 -4 0.61 0.11

2 11 -4 0.52 0.09

-2 11 4 0.42 0.09

-2 11 4 0.56 0.10

2 11 4 0.57 0.10

2 12 -4 0.22 0.09

-2 12 4 0.06 0.06

2 12 -4 0.12 0.05

2 12 4 0.11 0.06

-2 12 -4 0.10 0.05

-2 12 4 0.35 0.09

2 12 4 0.23 0.08

-2 12 -4 0.30 0.10

-2 12 4 0.01 0.07

2 12 4 0.15 0.07

2 13 -4 0.00 0.06

2 13 4 0.10 0.07

-2 13 -4 0.06 0.04

-2 13 -4 -0.01 0.06

-2 13 4 0.04 0.06

-2 13 4 -0.02 0.06

2 13 4 0.01 0.06

2 14 -4 0.03 0.05

-2 14 -4 0.01 0.05

-2 15 -4 0.06 0.03

2 15 -4 0.06 0.03

-2 0 5 641.23 4.27

-2 -1 5 22.59 0.56

2 -1 -5 22.23 0.55

2 2 5 219.74 1.97

2 -2 5 220.70 2.06

2 -3 -5 28.10 0.65

2 -3 5 27.97 0.65

2 3 5 27.58 0.59

2 4 -5 10.67 0.37

2 -4 -5 10.95 0.41

2 4 5 12.41 0.38

2 -4 5 12.30 0.43

-2 -4 -5 11.13 0.37

-2 4 5 11.87 0.39

2 4 5 12.33 0.40

2 -5 -5 5.15 0.26

2 5 -5 5.79 0.27

2 5 -5 6.09 0.38

2 -5 5 6.39 0.31

2 5 5 6.33 0.32

-2 5 5 6.13 0.32

2 6 -5 88.64 1.61

-2 6 -5 89.36 1.58

2 6 5 88.16 1.37

-2 6 5 87.28 1.37

-2 7 -5 2.42 0.29

2 7 -5 2.46 0.30

2 7 5 2.64 0.26

-2 7 5 2.55 0.26

2 8 5 45.29 0.64

2 8 5 42.07 1.10

-2 8 5 44.19 1.13

-2 8 5 44.75 0.66

2 8 -5 43.85 1.27

-2 8 -5 42.09 1.25

2 8 -5 44.67 1.31

-2 8 -5 44.43 0.72

2 8 5 43.29 0.62

-2 8 5 45.39 0.64

-2 9 5 0.53 0.08

-2 9 5 0.64 0.09

2 9 5 0.62 0.09

-2 9 -5 0.46 0.08

-2 9 -5 0.53 0.07

-2 9 -5 0.58 0.08

2 9 5 0.50 0.08

2 9 -5 0.46 0.08

2 9 -5 0.64 0.08

2 9 -5 0.53 0.17

2 9 -5 0.40 0.14

-2 9 -5 0.33 0.14

-2 9 -5 0.49 0.16

2 9 5 0.55 0.16

-2 9 5 0.52 0.16

-2 9 -5 0.75 0.12

2 9 -5 0.71 0.10

2 9 5 0.65 0.09

-2 9 5 0.71 0.09

-2 10 -5 9.67 0.30

2 10 -5 10.04 0.35

2 10 5 10.54 0.33

-2 10 5 10.58 0.34

2 10 5 10.36 0.35

-2 10 5 10.21 0.61

-2 10 -5 10.13 0.35

-2 10 5 9.83 0.33

2 10 -5 10.08 0.68

2 10 -5 9.67 0.31

-2 10 -5 10.09 0.71

2 10 -5 10.29 0.71

-2 10 -5 10.16 0.70

2 10 -5 9.62 0.36

-2 10 5 10.76 0.34

2 10 5 10.29 0.33

-2 11 5 1.10 0.12

-2 11 5 1.30 0.13

-2 11 -5 0.99 0.10

2 11 -5 1.23 0.12

2 11 5 1.28 0.14

2 11 5 1.19 0.12

2 11 -5 1.15 0.14

-2 11 -5 1.11 0.14

2 11 5 1.21 0.13

-2 11 5 1.32 0.13

-2 12 -5 1.41 0.11

2 12 5 1.66 0.15

-2 12 5 1.36 0.13

-2 12 5 1.62 0.14

2 12 5 1.47 0.13

-2 12 -5 1.70 0.16

2 12 5 1.37 0.13

-2 12 5 1.34 0.13

2 12 -5 1.71 0.15

-2 13 5 0.03 0.05

2 13 5 0.15 0.07

2 13 -5 -0.06 0.07

-2 13 -5 -0.06 0.07

-2 14 -5 1.11 0.10

2 14 -5 1.16 0.10

2 15 -5 0.01 0.03

-2 15 -5 0.04 0.03

-2 0 6 422.50 3.29

2 0 6 414.04 3.26

-2 -1 6 1.26 0.15

2 -1 -6 1.51 0.18

-2 1 6 1.00 0.14

2 1 6 1.16 0.14

2 -1 6 1.20 0.14

2 -2 6 50.57 0.95

2 -2 -6 51.95 0.96

2 2 6 50.00 0.90

2 -3 -6 6.15 0.33

2 3 6 5.62 0.29

2 -3 6 5.47 0.32

2 -4 6 1.18 0.16

2 4 -6 1.24 0.15

-2 -4 -6 1.22 0.14

2 4 6 1.24 0.14

2 -4 -6 1.23 0.16

2 -5 6 1.00 0.15

-2 5 6 0.86 0.13

2 5 -6 0.82 0.12

-2 -5 -6 0.84 0.11

2 -5 -6 0.83 0.13

-2 6 -6 0.25 0.13

-2 6 6 0.32 0.12

2 6 -6 0.19 0.12

-2 7 -6 0.81 0.18

2 7 -6 0.52 0.16

-2 7 6 0.76 0.15

2 8 6 0.00 0.05

2 8 6 0.06 0.05

-2 8 6 0.05 0.05

-2 8 6 0.04 0.04

-2 8 -6 0.05 0.11

-2 8 6 0.12 0.10

2 8 -6 0.18 0.16

2 8 -6 0.11 0.06

-2 8 -6 0.03 0.06

2 8 6 0.06 0.05

-2 8 6 0.05 0.05

2 9 -6 0.01 0.07

2 9 6 0.11 0.05

-2 9 6 0.19 0.06

-2 9 -6 0.01 0.07

2 9 -6 0.12 0.04

-2 9 6 0.14 0.05

2 9 6 0.08 0.05

2 9 -6 0.11 0.13

-2 9 -6 0.15 0.11

2 9 -6 0.04 0.10

-2 9 -6 0.39 0.17

-2 9 -6 0.09 0.07

2 9 -6 0.19 0.07

-2 9 6 0.15 0.06

2 9 6 0.18 0.06

-2 10 6 1.12 0.12

2 10 6 0.94 0.11

2 10 6 0.95 0.12

-2 10 -6 0.96 0.13

2 10 -6 0.92 0.12

2 10 -6 0.99 0.10

-2 10 6 1.09 0.12

2 10 -6 1.15 0.26

2 10 -6 0.74 0.21

-2 10 -6 1.11 0.26

-2 10 -6 1.11 0.25

2 10 -6 1.04 0.14

2 10 6 1.00 0.12

-2 10 6 1.01 0.12

-2 11 6 0.51 0.09

-2 11 6 0.57 0.10

2 11 -6 0.52 0.11

-2 11 -6 0.47 0.10

2 11 6 0.52 0.10

2 11 6 0.54 0.09

2 11 6 0.48 0.09

-2 11 6 0.60 0.10

-2 12 6 0.03 0.06

2 12 6 0.07 0.06

-2 12 -6 0.13 0.09

-2 12 6 0.16 0.07

2 12 6 0.04 0.06

2 12 -6 0.09 0.08

2 13 6 0.01 0.05

2 13 -6 -0.10 0.07

-2 13 -6 -0.04 0.07

-2 13 6 0.10 0.06

2 14 -6 -0.03 0.05

-2 14 -6 0.00 0.04

-2 15 -6 0.05 0.03

2 15 -6 0.04 0.03

2 0 7 456.65 3.65

-2 0 7 457.16 3.67

2 -1 -7 47.41 0.96

2 1 7 44.71 0.92

-2 -1 7 45.48 0.94

2 -1 7 45.35 0.94

-2 1 7 44.97 0.93

2 -2 7 252.50 2.51

2 2 7 250.67 2.42

2 -3 -7 28.93 0.77

2 -3 7 29.86 0.79

2 3 7 28.81 0.72

2 -4 -7 62.34 1.17

2 -4 7 60.70 1.17

2 4 -7 58.29 1.09

2 4 7 59.79 1.06

-2 -4 -7 57.40 1.07

-2 -5 -7 2.17 0.20

2 -5 7 2.65 0.25

2 -5 -7 2.14 0.22

2 5 -7 2.27 0.21

-2 6 -7 35.46 1.04

2 6 -7 36.01 1.06

-2 7 -7 3.70 0.37

2 7 -7 3.86 0.39

2 8 7 3.20 0.18

-2 8 7 3.27 0.17

2 8 7 3.29 0.17

-2 8 7 2.95 0.17

2 8 -7 3.21 0.37

-2 8 -7 3.34 0.38

-2 8 -7 3.48 0.21

2 8 -7 3.28 0.20

-2 8 7 2.88 0.16

2 8 7 2.86 0.16

2 9 7 0.43 0.08

2 9 -7 0.41 0.07

2 9 7 0.30 0.07

-2 9 7 0.50 0.08

-2 9 7 0.46 0.08

2 9 -7 0.28 0.14

2 9 -7 0.48 0.16

-2 9 -7 0.40 0.16

2 9 -7 0.51 0.10

-2 9 -7 0.47 0.10

-2 9 7 0.42 0.07

2 9 7 0.34 0.07

-2 10 7 13.81 0.39

2 10 7 13.74 0.37

2 10 -7 13.36 0.34

-2 10 7 12.34 0.37

2 10 -7 13.44 0.42

2 10 7 12.71 0.39

-2 10 -7 13.69 0.42

-2 10 -7 12.51 0.79

2 10 -7 13.63 0.82

-2 10 -7 13.86 0.85

2 10 -7 12.84 0.43

-2 10 -7 13.41 0.43

2 10 7 12.41 0.36

-2 10 7 13.01 0.37

-2 11 7 1.35 0.13

2 11 7 1.37 0.13

2 11 7 1.29 0.13

-2 11 7 1.18 0.13

2 11 -7 1.09 0.14

-2 11 -7 1.10 0.13

2 11 -7 1.11 0.14

-2 12 7 0.35 0.09

2 12 -7 0.38 0.10

-2 12 -7 0.40 0.10

2 12 7 0.24 0.07

-2 13 7 0.05 0.05

2 13 7 -0.02 0.05

-2 13 -7 -0.10 0.07

2 13 -7 0.02 0.06

-2 14 -7 0.58 0.07

2 14 -7 0.54 0.07

2 15 -7 0.03 0.02

-2 15 -7 -0.01 0.02

2 0 8 20.50 0.67

-2 0 8 20.94 0.69

2 -1 8 14.85 0.58

2 1 8 14.97 0.57

2 -1 -8 14.94 0.57

-2 -1 8 15.57 0.60

2 2 8 24.78 0.73

2 -2 8 24.98 0.76

2 -2 -8 24.88 0.76

2 -3 -8 6.73 0.40

2 3 8 6.98 0.39

2 -3 8 6.98 0.41

2 4 8 10.59 0.47

2 -4 -8 10.98 0.52

2 -4 8 10.99 0.53

-2 -4 -8 10.73 0.49

2 4 -8 10.73 0.49

-2 -5 -8 0.07 0.06

2 5 -8 0.01 0.08

2 -5 -8 0.03 0.08

2 -5 8 -0.02 0.08

2 6 -8 3.56 0.35

-2 7 -8 1.42 0.24

2 7 -8 1.44 0.25

2 8 8 1.34 0.11

2 8 8 1.39 0.12

-2 8 8 1.32 0.11

-2 8 8 1.21 0.11

2 8 -8 1.08 0.22

-2 8 -8 0.96 0.22

-2 8 -8 1.14 0.12

2 8 -8 1.12 0.13

-2 8 -8 1.35 0.14

2 8 8 1.14 0.10

-2 8 8 1.16 0.10

-2 9 8 0.56 0.08

2 9 8 0.45 0.08

-2 9 8 0.55 0.08

2 9 8 0.64 0.09

2 9 -8 0.64 0.22

-2 9 -8 0.59 0.19

2 9 -8 0.45 0.15

2 9 -8 0.47 0.10

-2 9 -8 0.69 0.12

2 9 8 0.45 0.08

-2 9 8 0.38 0.07

-2 10 -8 0.04 0.08

2 10 -8 0.07 0.07

2 10 8 0.06 0.05

-2 10 8 0.06 0.06

2 10 8 0.15 0.07

-2 10 8 0.14 0.06

2 10 -8 0.07 0.08

-2 10 -8 0.23 0.10

2 11 8 0.04 0.06

-2 11 8 0.01 0.06

2 11 8 0.05 0.06

2 11 -8 -0.02 0.08

-2 11 -8 -0.06 0.08

-2 11 8 0.09 0.06

2 12 8 0.13 0.06

2 12 -8 0.24 0.09

-2 12 -8 0.16 0.09

-2 12 8 0.07 0.06

2 13 -8 0.07 0.06

-2 13 -8 0.04 0.05

2 14 -8 0.06 0.04

-2 14 -8 -0.02 0.04

2 0 -9 0.11 0.11

-2 0 9 0.29 0.13

2 0 9 0.26 0.12

-2 -1 9 10.23 0.52

2 1 9 10.53 0.51

2 -1 9 10.13 0.51

2 -1 -9 10.88 0.53

2 -2 -9 3.08 0.31

2 -2 9 3.50 0.32

2 2 9 3.11 0.28

2 3 9 30.18 0.86

2 -3 -9 31.65 0.93

2 3 -9 30.19 0.88

2 -3 9 29.48 0.90

2 -4 -9 2.88 0.29

2 4 -9 2.91 0.28

2 -4 9 3.43 0.32

-2 -4 -9 2.89 0.28

2 4 9 3.37 0.29

-2 -5 -9 -0.07 0.09

2 5 -9 0.02 0.08

2 -5 9 -0.03 0.10

2 -5 -9 -0.01 0.08

2 7 9 1.58 0.11

-2 7 -9 1.59 0.14

2 7 -9 1.67 0.15

2 7 -9 1.41 0.25

-2 7 -9 1.35 0.24

-2 8 9 2.54 0.15

-2 8 9 2.50 0.15

2 8 9 2.48 0.16

2 8 9 2.60 0.15

-2 8 -9 3.22 0.21

-2 8 -9 2.40 0.18

2 8 -9 2.94 0.20

-2 8 -9 2.58 0.35

2 8 -9 2.72 0.36

-2 9 9 0.09 0.05

-2 9 9 0.03 0.05

2 9 9 0.09 0.06

2 9 9 0.06 0.04

-2 9 -9 0.22 0.08

-2 9 -9 0.13 0.06

2 9 -9 0.14 0.08

2 9 -9 -0.11 0.16

2 9 -9 0.16 0.11

-2 9 -9 0.18 0.10

-2 10 9 1.09 0.11

2 10 9 1.18 0.12

2 10 9 0.91 0.11

-2 10 -9 0.71 0.11

-2 10 9 1.06 0.11

2 10 -9 0.79 0.12

-2 10 -9 1.11 0.14

2 10 -9 0.99 0.14

-2 11 -9 0.19 0.08

2 11 -9 0.13 0.07

-2 11 9 0.40 0.08

-2 11 9 0.31 0.07

2 11 9 0.32 0.08

2 11 -9 0.35 0.10

2 12 9 0.05 0.05

-2 12 -9 0.06 0.07

-2 12 9 0.03 0.05

2 12 -9 0.10 0.07

2 13 -9 0.19 0.06

-2 13 -9 0.19 0.07

-2 14 -9 0.26 0.05

2 14 -9 0.39 0.06

2 0 -10 0.40 0.15

2 0 10 0.33 0.14

2 1 -10 2.67 0.29

2 1 10 2.27 0.26

2 -1 10 2.50 0.29

2 -1 -10 2.65 0.29

2 -2 10 6.83 0.46

2 2 -10 5.85 0.43

2 2 10 7.41 0.46

2 -2 -10 5.85 0.43

2 3 -10 4.23 0.35

-2 -3 -10 5.23 0.39

2 3 10 3.69 0.32

2 -3 10 4.32 0.37

2 -3 -10 4.63 0.39

2 -4 10 0.24 0.11

2 4 10 0.12 0.08

-2 -4 -10 0.30 0.12

2 4 -10 0.23 0.11

2 -4 -10 0.23 0.11

2 5 -10 0.91 0.17

2 -5 -10 1.09 0.19

-2 -5 -10 1.00 0.19

2 7 10 0.36 0.06

-2 7 10 0.31 0.06

2 7 -10 0.24 0.10

-2 7 -10 0.28 0.14

2 7 -10 0.29 0.08

-2 7 -10 0.33 0.08

-2 7 -10 0.34 0.08

-2 8 10 1.83 0.13

2 8 10 1.65 0.13

2 8 10 1.87 0.13

-2 8 -10 1.62 0.15

-2 8 -10 2.01 0.17

2 8 -10 1.86 0.17

2 8 -10 2.13 0.33

-2 8 -10 1.53 0.29

-2 9 10 0.31 0.07

2 9 10 0.20 0.06

-2 9 10 0.14 0.06

2 9 10 0.26 0.06

2 9 -10 0.13 0.08

-2 9 -10 0.19 0.07

-2 9 -10 0.18 0.09

-2 9 -10 0.10 0.09

2 9 -10 0.13 0.14

-2 10 10 0.50 0.09

-2 10 10 0.51 0.08

2 10 -10 0.32 0.09

-2 10 -10 0.20 0.08

2 10 10 0.49 0.08

2 10 10 0.42 0.08

-2 10 -10 0.65 0.13

2 10 -10 0.39 0.11

-2 11 10 0.06 0.05

2 11 -10 -0.06 0.08

-2 11 10 -0.05 0.06

2 11 10 0.04 0.05

-2 11 -10 -0.05 0.09

2 11 -10 0.19 0.10

2 12 10 0.09 0.05

2 12 -10 0.08 0.07

-2 12 -10 0.04 0.06

2 13 -10 -0.01 0.04

-2 13 -10 0.05 0.05

-2 14 -10 -0.02 0.03

2 14 -10 -0.03 0.03

2 0 11 50.09 1.29

2 0 -11 49.78 1.28

2 -1 11 7.06 0.49

2 -1 -11 6.73 0.48

2 1 -11 6.70 0.47

2 1 11 6.26 0.46

2 -2 11 155.71 2.40

2 2 -11 149.99 2.30

2 -2 -11 155.86 2.40

2 2 11 146.51 2.28

2 -3 11 3.82 0.37

2 3 11 3.72 0.35

2 3 -11 4.62 0.40

2 -3 -11 4.70 0.41

-2 -3 -11 4.90 0.41

-2 -4 -11 2.93 0.32

2 -4 -11 2.93 0.33

2 4 -11 2.74 0.31

2 -4 11 2.51 0.31

-2 -5 -11 0.53 0.15

2 5 -11 0.88 0.18

2 -5 -11 0.79 0.19

-2 6 -11 1.06 0.12

2 6 -11 1.11 0.12

-2 6 -11 1.00 0.11

-2 6 11 0.99 0.12

-2 7 11 0.48 0.07

2 7 11 0.49 0.07

2 7 -11 0.24 0.12

-2 7 -11 0.46 0.15

-2 7 -11 0.46 0.09

2 7 -11 0.35 0.08

-2 7 -11 0.36 0.08

2 7 -11 0.29 0.07

2 8 11 1.80 0.12

-2 8 11 1.66 0.12

2 8 11 1.61 0.13

2 8 -11 1.25 0.13

-2 8 -11 1.39 0.14

2 8 -11 1.69 0.17

-2 8 -11 2.02 0.17

2 8 -11 1.74 0.31

-2 8 -11 1.68 0.30

-2 9 11 0.14 0.05

2 9 11 0.21 0.06

2 9 11 0.26 0.06

-2 9 -11 0.16 0.09

-2 9 -11 0.21 0.07

2 9 -11 0.19 0.08

2 10 11 0.31 0.07

2 10 -11 0.33 0.09

-2 10 -11 0.17 0.07

-2 10 11 0.42 0.07

-2 10 11 0.34 0.07

-2 10 -11 0.49 0.11

2 10 -11 0.30 0.10

2 11 11 0.03 0.04

-2 11 11 0.05 0.05

-2 11 -11 -0.07 0.08

2 11 -11 -0.12 0.08

-2 12 -11 0.25 0.07

2 12 11 0.41 0.06

2 12 -11 0.30 0.08

-2 13 -11 0.00 0.04

2 13 -11 0.03 0.04

-2 14 -11 0.21 0.04

2 14 -11 0.26 0.04

2 0 12 34.33 1.13

2 0 -12 36.84 1.16

2 1 -12 0.18 0.14

2 -1 -12 0.20 0.14

2 1 12 0.04 0.13

2 -1 12 0.13 0.13

2 -2 -12 25.68 0.99

2 -2 12 27.91 1.03

2 2 12 25.15 0.96

2 2 -12 25.23 0.96

-2 -3 -12 0.17 0.12

2 3 12 0.18 0.10

2 -3 -12 0.09 0.10

2 3 -12 0.15 0.13

2 -3 12 0.13 0.11

-2 -4 -12 0.00 0.10

2 4 -12 0.07 0.11

2 -4 -12 0.07 0.12

2 -4 12 0.04 0.11

-2 -5 -12 0.05 0.08

2 -5 -12 0.07 0.08

2 5 -12 0.05 0.09

2 6 -12 0.27 0.07

-2 6 -12 0.32 0.08

-2 6 12 0.30 0.07

-2 6 -12 0.20 0.07

2 6 -12 0.19 0.06

-2 7 12 1.20 0.10

2 7 -12 1.09 0.23

-2 7 -12 0.90 0.22

-2 7 -12 1.20 0.13

2 7 -12 1.07 0.13

-2 7 -12 1.52 0.15

2 7 -12 1.41 0.15

-2 8 12 0.15 0.05

2 8 -12 0.05 0.11

-2 8 -12 0.15 0.14

-2 8 -12 0.30 0.10

2 8 -12 0.16 0.09

2 8 -12 0.10 0.06

-2 8 -12 0.04 0.06

-2 9 12 0.17 0.05

2 9 -12 0.23 0.10

-2 9 -12 0.15 0.07

-2 9 -12 0.28 0.10

-2 10 12 0.01 0.05

2 10 12 0.14 0.06

-2 10 -12 0.11 0.09

-2 10 -12 0.08 0.07

2 10 -12 0.02 0.08

2 11 -12 0.27 0.07

2 11 12 0.37 0.06

2 11 -12 0.50 0.11

-2 12 -12 0.02 0.05

2 12 -12 0.03 0.05

-2 13 -12 -0.08 0.05

2 13 -12 -0.04 0.04

2 14 -12 0.06 0.03

-2 14 -12 0.06 0.03

2 0 -13 7.57 0.57

2 0 13 6.16 0.50

2 -1 13 28.06 1.08

2 1 -13 27.62 1.07

2 1 13 27.57 1.06

2 -1 -13 29.85 1.12

2 2 13 9.19 0.61

2 2 -13 8.99 0.61

2 -2 -13 9.26 0.63

2 -2 13 9.46 0.64

-2 -2 -13 9.50 0.63

2 -3 13 3.67 0.39

2 -3 -13 3.90 0.42

-2 -3 -13 3.80 0.41

2 3 -13 3.47 0.39

2 3 13 3.62 0.39

2 4 -13 6.24 0.52

2 -4 -13 6.94 0.56

-2 -4 -13 7.16 0.56

2 -5 -13 0.09 0.09

2 5 -13 0.06 0.10

-2 5 -13 0.08 0.06

2 5 -13 0.08 0.05

-2 5 13 0.12 0.06

-2 5 -13 0.07 0.05

2 5 13 0.07 0.05

2 5 -13 0.22 0.07

2 6 -13 1.34 0.14

-2 6 -13 1.23 0.13

-2 6 -13 1.10 0.13

2 6 -13 1.18 0.13

-2 6 13 1.09 0.14

2 7 -13 0.21 0.13

-2 7 -13 0.16 0.15

2 7 -13 0.21 0.08

-2 7 -13 0.18 0.07

-2 7 -13 0.20 0.08

2 7 -13 0.26 0.07

-2 8 13 7.46 0.24

2 8 -13 8.41 0.36

-2 8 -13 6.79 0.31

-2 8 -13 8.17 0.34

2 8 -13 6.13 0.29

-2 9 -13 1.07 0.14

-2 9 -13 0.84 0.12

2 10 -13 1.80 0.18

-2 10 -13 1.78 0.17

-2 11 -13 -0.10 0.07

2 11 -13 -0.18 0.08

2 11 -13 0.04 0.08

2 12 -13 0.05 0.05

-2 12 -13 0.04 0.05

-2 13 -13 -0.02 0.04

2 13 -13 -0.03 0.04

2 0 -14 9.02 0.65

2 0 14 8.96 0.65

2 1 14 4.42 0.45

2 -1 -14 4.62 0.47

2 -1 14 4.76 0.47

2 1 -14 4.64 0.47

2 2 -14 6.13 0.54

2 -2 14 6.25 0.55

2 -2 -14 7.08 0.59

-2 -2 -14 6.58 0.55

2 2 14 6.12 0.53

2 -3 14 3.30 0.41

2 -3 -14 3.11 0.40

2 3 -14 2.94 0.37

-2 -3 -14 3.34 0.41

2 4 -14 3.65 0.42

-2 -4 -14 4.80 0.49

2 -4 -14 4.21 0.46

2 4 -14 4.91 0.24

2 4 14 4.95 0.27

-2 4 -14 4.77 0.24

-2 4 14 4.91 0.27

2 5 -14 0.18 0.11

-2 5 -14 0.19 0.11

2 -5 -14 0.21 0.13

2 5 -14 0.28 0.08

-2 5 -14 0.18 0.07

-2 5 -14 0.31 0.08

-2 5 14 0.20 0.07

2 5 -14 0.36 0.08

2 5 14 0.24 0.07

-2 6 -14 0.06 0.05

2 6 -14 0.14 0.08

-2 6 -14 0.11 0.07

2 6 -14 0.10 0.07

-2 6 14 0.03 0.07

2 7 -14 -0.12 0.14

-2 7 -14 -0.06 0.13

-2 7 -14 0.01 0.07

2 7 -14 0.08 0.07

-2 7 -14 0.06 0.08

2 7 -14 -0.02 0.07

-2 8 -14 1.03 0.14

2 8 -14 0.70 0.11

-2 8 -14 0.78 0.12

-2 9 -14 0.09 0.06

2 9 -14 0.25 0.10

-2 9 -14 0.18 0.09

-2 10 -14 0.28 0.10

-2 10 -14 0.13 0.06

2 10 -14 0.40 0.12

-2 11 -14 0.23 0.07

2 11 -14 0.30 0.07

2 12 -14 0.01 0.04

-2 12 -14 -0.05 0.05

2 13 -14 -0.05 0.04

-2 13 -14 -0.03 0.03

-2 0 -15 91.67 2.17

2 0 15 90.83 2.18

2 0 -15 91.61 2.20

-2 -1 -15 0.78 0.23

2 1 -15 0.60 0.20

2 1 15 0.38 0.16

2 -1 -15 0.36 0.17

2 -1 15 0.71 0.21

2 -2 -15 3.09 0.42

-2 -2 -15 3.21 0.42

2 2 -15 3.08 0.41

-2 2 15 3.91 0.22

2 2 15 4.01 0.22

2 -3 -15 3.76 0.46

-2 -3 -15 3.84 0.46

2 3 -15 3.51 0.44

-2 3 -15 3.37 0.42

2 3 -15 3.85 0.20

-2 3 15 4.12 0.24

2 3 15 3.76 0.23

2 4 -15 8.59 0.67

-2 4 -15 8.41 0.67

2 -4 -15 8.49 0.68

-2 -4 -15 9.99 0.72

2 4 -15 10.31 0.35

-2 4 -15 10.39 0.34

-2 4 15 10.60 0.40

2 4 15 10.92 0.40

-2 4 -15 10.13 0.37

-2 5 -15 0.72 0.19

2 5 -15 0.66 0.20

2 5 -15 0.86 0.12

-2 5 -15 0.80 0.11

2 5 -15 0.83 0.12

2 5 15 0.64 0.11

-2 5 15 0.69 0.11

-2 5 -15 0.90 0.13

-2 6 -15 3.26 0.21

2 6 -15 3.20 0.22

2 6 -15 2.98 0.21

-2 6 -15 3.09 0.22

-2 6 15 3.13 0.24

-2 7 -15 0.48 0.09

2 7 -15 0.59 0.12

2 7 -15 0.35 0.09

2 8 -15 11.80 0.41

-2 8 -15 11.05 0.39

-2 9 -15 0.00 0.06

-2 9 -15 0.12 0.08

2 9 -15 0.06 0.09

2 9 -15 0.03 0.05

2 10 -15 5.61 0.27

-2 10 -15 5.62 0.26

2 11 -15 0.07 0.06

-2 11 -15 0.04 0.05

-2 12 -15 0.33 0.06

2 12 -15 0.38 0.06

2 13 -15 0.03 0.02

-2 13 -15 0.12 0.03

-2 0 -16 0.63 0.21

2 0 -16 0.59 0.21

2 0 16 0.72 0.10

2 1 -16 0.74 0.24

2 -1 -16 0.95 0.27

-2 -1 -16 0.85 0.25

-2 1 -16 0.83 0.24

2 -1 16 0.87 0.10

2 1 16 1.10 0.13

-2 1 16 0.91 0.12

-2 -2 -16 0.66 0.23

-2 2 -16 0.59 0.19

2 2 -16 0.63 0.22

2 -2 -16 0.68 0.23

-2 2 16 1.00 0.13

2 2 16 0.98 0.12

-2 3 -16 2.26 0.37

2 3 -16 2.25 0.36

-2 -3 -16 2.22 0.37

2 -3 -16 2.57 0.40

2 3 -16 2.43 0.17

-2 3 16 2.46 0.20

2 3 16 2.65 0.21

-2 -4 -16 0.37 0.17

-2 4 -16 0.35 0.18

2 4 -16 0.16 0.13

2 -4 -16 0.54 0.21

-2 4 -16 0.43 0.09

2 4 -16 0.43 0.09

-2 4 16 0.58 0.11

-2 4 -16 0.49 0.10

2 4 16 0.41 0.10

-2 5 -16 0.07 0.12

2 5 -16 0.39 0.18

2 5 -16 0.34 0.09

-2 5 -16 0.30 0.08

2 5 16 0.29 0.09

-2 5 16 0.34 0.10

2 5 -16 0.30 0.09

-2 5 -16 0.35 0.10

2 6 -16 2.13 0.18

-2 6 -16 2.16 0.19

-2 6 -16 2.29 0.18

2 6 -16 2.32 0.19

2 7 -16 -0.03 0.07

-2 7 -16 0.03 0.07

2 7 -16 0.01 0.06

-2 7 -16 -0.01 0.07

-2 8 -16 1.11 0.14

2 8 -16 1.12 0.15

2 8 -16 0.86 0.13

-2 8 -16 1.03 0.14

-2 9 -16 0.33 0.08

2 9 -16 0.54 0.11

-2 9 -16 0.50 0.10

2 9 -16 0.30 0.07

-2 10 -16 0.20 0.06

2 10 -16 0.47 0.10

2 11 -16 0.12 0.05

-2 11 -16 0.19 0.06

2 12 -16 0.02 0.03

-2 12 -16 0.00 0.03

-2 0 -17 15.96 0.98

2 0 -17 16.28 1.00

2 0 17 16.35 0.45

-2 1 -17 2.97 0.44

2 -1 -17 2.95 0.44

-2 -1 -17 2.76 0.43

2 1 -17 3.09 0.44

2 -1 17 3.57 0.21

2 1 17 3.44 0.22

-2 1 17 3.69 0.23

-2 -2 -17 4.94 0.56

-2 2 -17 4.49 0.53

2 -2 -17 4.77 0.56

2 2 -17 4.71 0.54

-2 2 17 4.51 0.26

2 2 17 4.82 0.27

2 3 -17 0.48 0.19

-2 -3 -17 0.33 0.15

2 -3 -17 0.72 0.22

-2 3 -17 0.27 0.20

2 3 -17 0.49 0.09

-2 3 17 0.61 0.11

2 3 17 0.74 0.12

2 -4 -17 0.98 0.27

-2 4 -17 0.85 0.25

2 4 -17 0.87 0.25

-2 -4 -17 0.73 0.23

2 4 -17 0.95 0.12

-2 4 -17 0.89 0.13

2 4 17 1.13 0.15

-2 4 17 1.11 0.16

-2 5 -17 -0.06 0.08

2 5 -17 -0.09 0.08

-2 5 -17 0.05 0.06

-2 5 -17 0.05 0.08

2 5 -17 -0.07 0.09

-2 5 17 0.01 0.08

2 6 -17 11.23 0.40

2 6 -17 10.25 0.39

-2 6 -17 10.62 0.41

-2 6 -17 10.47 0.36

-2 7 -17 0.06 0.07

2 7 -17 0.07 0.08

-2 7 -17 0.00 0.07

2 7 -17 -0.01 0.06

-2 8 -17 0.58 0.11

2 8 -17 0.63 0.12

-2 8 -17 0.47 0.11

2 8 -17 0.44 0.10

-2 9 -17 0.00 0.05

2 9 -17 0.10 0.08

-2 9 -17 0.05 0.07

2 9 -17 0.09 0.05

-2 10 -17 1.27 0.11

2 10 -17 2.00 0.15

2 11 -17 0.06 0.05

-2 11 -17 -0.02 0.04

2 12 -17 0.03 0.03

-2 12 -17 0.01 0.03

2 0 -18 0.34 0.19

2 0 18 0.36 0.09

-2 0 18 0.39 0.09

-2 1 -18 -0.04 0.16

2 -1 -18 0.01 0.17

-2 -1 -18 -0.11 0.15

2 1 -18 0.10 0.18

2 1 18 -0.03 0.08

2 -1 18 0.03 0.05

-2 1 18 -0.02 0.06

-2 2 -18 0.21 0.18

2 2 -18 -0.02 0.13

-2 -2 -18 0.16 0.12

2 -2 -18 0.44 0.19

2 2 18 0.18 0.09

-2 2 18 0.39 0.11

-2 -3 -18 -0.21 0.16

2 3 -18 0.20 0.15

2 -3 -18 0.00 0.14

-2 3 -18 -0.07 0.16

2 3 -18 0.09 0.05

2 3 18 -0.01 0.08

-2 3 -18 0.14 0.09

-2 3 18 0.12 0.08

2 4 -18 0.16 0.07

2 4 18 0.05 0.08

-2 4 -18 0.15 0.09

-2 4 18 0.28 0.11

-2 5 -18 0.44 0.09

2 5 -18 0.31 0.08

-2 5 -18 0.50 0.11

-2 5 18 0.46 0.12

2 5 -18 0.31 0.09

-2 6 -18 0.28 0.08

2 6 -18 0.27 0.09

2 6 -18 0.23 0.09

-2 6 -18 0.25 0.10

2 7 -18 0.14 0.08

-2 7 -18 0.03 0.06

-2 7 -18 0.02 0.07

2 7 -18 0.00 0.06

2 8 -18 0.38 0.09

-2 8 -18 0.24 0.08

2 8 -18 0.30 0.08

-2 8 -18 0.27 0.09

-2 9 -18 0.22 0.07

2 9 -18 0.20 0.06

-2 9 -18 0.20 0.07

2 9 -18 0.24 0.08

-2 10 -18 0.15 0.06

2 10 -18 0.09 0.06

2 10 -18 0.07 0.06

-2 10 -18 0.01 0.04

-2 11 -18 0.03 0.04

2 11 -18 -0.04 0.04

2 0 -19 2.33 0.42

-2 0 -19 1.82 0.38

2 0 19 2.07 0.17

-2 0 19 1.82 0.17

2 1 -19 1.50 0.35

2 -1 -19 1.46 0.35

-2 1 -19 1.18 0.30

-2 -1 -19 1.39 0.32

-2 1 19 1.61 0.17

2 -1 19 1.34 0.14

-2 -1 19 1.47 0.15

2 1 19 1.57 0.16

2 2 19 32.84 0.72

-2 2 19 31.91 0.73

-2 3 -19 0.21 0.10

2 3 19 0.02 0.08

-2 3 19 0.03 0.09

2 4 -19 0.71 0.11

2 4 -19 0.89 0.14

-2 4 -19 0.73 0.14

-2 4 19 0.86 0.15

2 4 19 1.01 0.16

2 5 -19 0.05 0.06

-2 5 19 0.22 0.12

-2 5 -19 0.09 0.08

2 5 -19 0.07 0.08

2 6 -19 7.91 0.34

-2 6 -19 8.11 0.35

-2 6 -19 7.68 0.29

2 6 -19 7.66 0.31

2 7 -19 0.16 0.08

-2 7 -19 0.16 0.07

-2 7 -19 0.07 0.08

2 7 -19 0.15 0.08

-2 8 -19 0.29 0.07

2 8 -19 0.38 0.09

-2 8 -19 0.17 0.07

2 8 -19 0.29 0.07

2 9 -19 0.06 0.04

-2 9 -19 0.00 0.05

2 9 -19 0.09 0.06

2 10 -19 0.66 0.08

-2 10 -19 0.78 0.09

2 11 -19 0.03 0.03

-2 11 -19 0.06 0.03

2 0 20 0.11 0.07

-2 0 20 0.06 0.08

2 -1 20 0.79 0.11

-2 1 20 0.58 0.11

2 1 20 0.69 0.11

-2 -1 20 0.60 0.11

2 2 20 0.33 0.10

-2 -2 20 0.23 0.08

-2 2 20 0.32 0.11

-2 2 -20 0.56 0.12

-2 3 20 0.48 0.13

2 3 20 0.22 0.10

-2 -3 20 0.34 0.08

-2 3 -20 0.40 0.11

2 4 -20 0.87 0.11

-2 4 -20 1.02 0.16

-2 4 20 1.19 0.17

2 4 -20 0.90 0.14

2 5 -20 -0.02 0.05

2 5 -20 -0.05 0.09

-2 5 -20 -0.01 0.09

2 6 -20 0.10 0.07

-2 6 -20 0.07 0.05

-2 6 -20 0.13 0.09

2 6 -20 0.17 0.09

2 7 -20 0.08 0.06

-2 7 -20 0.07 0.05

-2 7 -20 0.07 0.07

2 7 -20 0.15 0.07

-2 8 -20 0.74 0.09

2 8 -20 0.74 0.10

-2 8 -20 0.85 0.11

2 8 -20 0.65 0.09

2 9 -20 0.05 0.04

-2 9 -20 0.15 0.05

2 9 -20 0.18 0.06

-2 10 -20 0.10 0.04

2 10 -20 0.03 0.04

-2 0 21 0.49 0.11

2 0 21 0.36 0.09

-2 -1 21 0.07 0.07

2 -1 21 -0.04 0.06

-2 1 21 0.00 0.08

2 1 21 -0.06 0.07

-2 -2 21 9.08 0.34

-2 2 21 10.03 0.40

-2 2 -21 10.11 0.38

2 2 21 9.30 0.38

-2 3 -21 0.21 0.10

-2 -3 21 0.12 0.05

2 3 21 0.09 0.08

2 3 -21 0.20 0.09

-2 3 21 0.30 0.12

-2 4 21 1.13 0.17

2 4 -21 0.94 0.14

-2 4 -21 1.04 0.15

2 5 -21 0.36 0.08

2 5 -21 0.25 0.10

-2 5 -21 0.32 0.10

2 6 -21 1.75 0.16

-2 6 -21 1.87 0.17

2 6 -21 1.77 0.14

2 7 -21 0.03 0.05

2 7 -21 0.12 0.07

-2 7 -21 0.16 0.07

2 8 -21 0.23 0.06

-2 8 -21 0.22 0.07

2 8 -21 0.16 0.06

-2 9 -21 0.09 0.04

-2 10 -21 1.09 0.07

2 10 -21 1.13 0.07

2 0 22 1.50 0.15

-2 0 22 1.45 0.16

2 1 22 0.35 0.09

-2 1 -22 0.57 0.11

2 -1 22 0.39 0.08

-2 1 22 0.44 0.11

-2 -1 22 0.33 0.08

-2 2 -22 0.27 0.10

-2 -2 22 0.33 0.08

2 2 22 0.25 0.09

-2 2 22 0.33 0.11

-2 3 -22 0.92 0.14

2 3 -22 0.84 0.13

-2 3 22 0.89 0.15

2 4 -22 0.22 0.09

-2 4 -22 0.24 0.10

-2 5 -22 0.06 0.08

2 5 -22 0.04 0.07

2 6 -22 1.46 0.14

-2 6 -22 1.36 0.14

2 6 -22 1.27 0.11

2 7 -22 0.18 0.05

2 7 -22 0.20 0.07

-2 7 -22 0.21 0.07

2 8 -22 0.06 0.04

-2 8 -22 0.05 0.04

2 9 -22 0.10 0.04

-2 9 -22 0.18 0.05

2 0 23 0.52 0.09

-2 0 23 0.58 0.10

-2 0 -23 0.78 0.11

-2 -1 -23 0.11 0.07

2 1 23 0.01 0.06

-2 1 23 0.06 0.07

-2 -1 23 0.08 0.06

-2 1 -23 0.10 0.08

-2 2 23 1.14 0.15

-2 2 -23 1.26 0.14

-2 -2 23 1.08 0.12

2 2 -23 1.49 0.15

-2 3 23 0.18 0.09

-2 -3 -23 0.23 0.06

2 3 -23 0.13 0.08

-2 3 -23 0.22 0.08

-2 4 -23 1.21 0.14

2 4 -23 1.26 0.14

2 5 -23 0.14 0.07

-2 5 -23 0.21 0.08

-2 6 -23 3.03 0.18

2 6 -23 2.83 0.17

2 7 -23 0.12 0.05

-2 7 -23 0.13 0.05

-2 8 -23 0.51 0.06

-2 0 -24 0.09 0.06

-2 0 24 0.10 0.07

-2 -1 24 -0.01 0.05

2 1 -24 0.01 0.06

-2 1 -24 -0.03 0.06

-2 -1 -24 0.09 0.06

-2 1 24 0.08 0.07

-2 2 -24 0.39 0.09

2 2 -24 0.26 0.08

-2 -2 24 0.24 0.06

-2 -2 -24 0.35 0.07

-2 2 24 0.38 0.09

2 3 -24 -0.05 0.07

-2 3 -24 -0.03 0.06

-2 4 -24 0.02 0.06

2 4 -24 0.14 0.07

-2 5 -24 0.12 0.06

2 5 -24 0.19 0.07

2 6 -24 0.37 0.07

-2 6 -24 0.38 0.07

-2 7 -24 0.07 0.04

2 7 -24 0.05 0.03

-2 0 -25 2.60 0.16

-2 0 25 2.77 0.17

2 0 -25 2.77 0.17

2 1 -25 0.07 0.06

2 -1 -25 0.06 0.05

-2 -1 25 -0.04 0.04

-2 1 -25 -0.01 0.05

-2 -1 -25 0.07 0.05

-2 2 -25 3.16 0.18

2 2 -25 3.08 0.18

-2 -2 -25 2.74 0.15

2 -2 -25 3.03 0.16

2 -3 -25 -0.01 0.04

2 3 -25 0.07 0.06

-2 3 -25 0.01 0.05

2 4 -25 0.33 0.08

-2 4 -25 0.27 0.07

2 5 -25 0.06 0.05

-2 5 -25 0.06 0.05

-2 6 -25 0.44 0.06

2 6 -25 0.30 0.05

-2 0 -26 -0.01 0.04

2 0 -26 0.03 0.04

2 -1 -26 0.12 0.05

-2 -1 -26 0.07 0.04

-2 1 -26 0.13 0.05

2 1 -26 0.11 0.05

-2 2 -26 0.22 0.06

2 2 -26 0.13 0.05

2 -2 -26 0.18 0.05

-2 -2 -26 0.08 0.04

-2 3 -26 0.08 0.05

2 3 -26 0.16 0.06

2 -3 -26 0.05 0.03

2 4 -26 0.23 0.06

-2 4 -26 0.26 0.06

2 5 -26 0.03 0.03

-2 5 -26 0.05 0.03

-2 0 -27 0.35 0.05

2 0 -27 0.38 0.05

2 1 -27 0.02 0.04

2 -1 -27 0.05 0.03

-2 1 -27 0.02 0.03

-2 2 -27 -0.01 0.03

2 2 -27 -0.01 0.03

-2 3 -27 0.11 0.04

2 3 -27 0.07 0.04

3 0 0 0.00 0.03

3 1 0 27.95 0.37

3 1 0 27.30 0.35

-3 1 0 27.51 0.28

3 1 0 28.16 0.31

-3 2 0 7.98 0.21

3 2 0 7.91 0.24

3 -2 0 8.21 0.25

3 2 0 8.35 0.16

3 -2 0 8.08 0.21

3 2 0 7.67 0.22

3 3 0 35.22 0.60

3 -3 0 36.14 0.45

-3 3 0 36.61 0.58

3 4 0 3.24 0.22

-3 4 0 3.36 0.20

3 4 0 3.25 0.10

3 4 0 3.26 0.21

-3 5 0 1.63 0.17

-3 5 0 1.85 0.19

3 5 0 1.65 0.18

3 5 0 1.61 0.18

-3 6 0 0.16 0.10

-3 6 0 0.10 0.10

3 6 0 0.06 0.10

3 6 0 0.18 0.10

-3 7 0 5.24 0.38

-3 7 0 5.28 0.36

3 7 0 5.78 0.40

3 7 0 5.52 0.38

-3 8 0 16.39 0.69

3 8 0 17.36 0.74

-3 8 0 18.16 0.77

3 8 0 18.01 0.73

-3 9 0 2.59 0.31

3 9 0 3.26 0.35

-3 9 0 2.77 0.17

3 9 0 3.03 0.33

-3 9 0 3.11 0.35

-3 9 0 3.07 0.17

3 9 0 2.86 0.16

3 9 0 3.03 0.18

3 9 0 2.95 0.17

-3 9 0 3.24 0.18

3 10 0 10.21 0.66

-3 10 0 10.36 0.63

-3 10 0 10.09 0.32

3 10 0 9.82 0.30

3 10 0 10.21 0.34

3 10 0 9.65 0.62

-3 10 0 10.24 0.33

-3 10 0 9.89 0.66

-3 10 0 10.85 0.35

3 10 0 10.24 0.34

-3 11 0 0.33 0.09

-3 11 0 0.09 0.07

3 11 0 0.14 0.08

3 11 0 0.31 0.09

-3 11 0 0.07 0.07

3 11 0 0.13 0.06

3 12 0 1.86 0.16

3 12 0 1.49 0.12

-3 12 0 1.42 0.13

-3 12 0 1.97 0.16

3 12 0 1.89 0.16

-3 13 0 0.00 0.05

3 13 0 0.10 0.07

3 13 0 0.06 0.07

-3 13 0 0.05 0.06

-3 14 0 0.56 0.07

-3 14 0 0.47 0.08

3 14 0 0.48 0.08

3 0 1 -0.02 0.03

3 0 -1 0.01 0.03

3 -1 1 12.72 0.28

3 1 1 13.47 0.28

3 1 1 13.54 0.24

-3 1 1 13.24 0.21

3 1 -1 13.10 0.26

3 1 1 13.82 0.22

3 2 1 14.26 0.32

3 2 -1 14.86 0.33

3 2 -1 14.40 0.25

3 -2 1 14.52 0.30

-3 2 1 14.56 0.31

3 -2 -1 13.75 0.33

3 2 1 14.72 0.28

-3 2 1 14.32 0.28

3 2 1 14.46 0.30

-3 3 1 37.30 0.59

3 3 1 39.42 0.64

3 3 1 37.59 0.46

3 3 -1 37.98 0.37

3 3 -1 37.72 0.66

-3 3 1 39.58 0.64

3 -3 -1 38.19 0.60

-3 3 -1 39.72 0.63

3 3 1 38.24 0.61

-3 4 1 20.27 0.49

3 -4 -1 20.97 0.45

-3 4 -1 21.62 0.53

-3 4 1 21.12 0.54

3 4 1 20.95 0.53

3 4 -1 21.36 0.56

3 4 1 20.25 0.51

-3 5 1 9.25 0.38

3 5 1 9.76 0.41

3 5 -1 9.38 0.42

-3 5 1 9.60 0.41

3 5 -1 9.33 0.40

-3 5 -1 9.26 0.39

3 5 1 9.30 0.40

3 6 -1 7.22 0.43

-3 6 1 7.39 0.42

3 6 1 7.29 0.40

-3 6 -1 7.55 0.39

-3 6 1 6.76 0.37

3 6 -1 7.50 0.41

3 6 1 6.72 0.37

3 7 1 5.96 0.40

-3 7 1 5.82 0.37

-3 7 -1 6.09 0.42

3 7 -1 6.26 0.42

-3 7 -1 5.85 0.39

3 7 -1 6.39 0.41

-3 7 1 6.05 0.40

3 7 1 5.93 0.39

-3 8 -1 24.74 0.92

3 8 -1 24.88 0.91

3 8 1 25.86 0.90

-3 8 1 24.82 0.84

-3 8 -1 23.75 0.85

-3 8 1 26.26 0.91

3 8 1 25.20 0.86

3 8 -1 24.92 0.88

3 9 -1 1.12 0.10

3 9 1 1.05 0.11

-3 9 -1 1.31 0.12

3 9 -1 1.28 0.12

3 9 1 1.02 0.10

-3 9 1 1.06 0.11

-3 9 -1 1.27 0.12

-3 9 1 1.03 0.11

-3 9 -1 1.16 0.21

-3 9 1 1.09 0.21

-3 9 -1 1.29 0.25

3 9 -1 1.04 0.22

-3 9 1 0.83 0.19

3 9 1 1.05 0.21

3 9 1 1.17 0.21

3 9 -1 1.25 0.23

3 9 -1 1.14 0.11

-3 9 1 1.10 0.11

3 9 1 1.14 0.11

3 9 -1 1.11 0.11

-3 9 -1 1.34 0.12

-3 10 -1 0.73 0.22

-3 10 1 0.41 0.16

3 10 -1 0.70 0.20

-3 10 -1 0.50 0.18

3 10 1 0.43 0.16

3 10 -1 0.70 0.19

3 10 1 0.57 0.10

3 10 -1 0.67 0.10

-3 10 -1 0.73 0.10

3 10 1 0.54 0.09

-3 10 -1 0.64 0.10

3 10 -1 0.64 0.10

-3 10 1 0.67 0.10

3 10 1 0.59 0.10

-3 10 1 0.72 0.10

-3 10 1 0.51 0.09

3 10 -1 0.56 0.08

-3 10 -1 0.76 0.11

3 11 -1 0.33 0.08

3 11 1 0.49 0.10

3 11 1 0.52 0.09

-3 11 1 0.48 0.10

3 11 -1 0.39 0.07

3 11 1 0.41 0.09

-3 11 -1 0.42 0.09

-3 11 -1 0.40 0.08

3 11 -1 0.52 0.10

-3 11 1 0.24 0.07

-3 11 1 0.55 0.10

-3 11 -1 0.61 0.10

3 12 1 1.69 0.13

3 12 -1 2.25 0.17

3 12 1 2.18 0.18

-3 12 1 1.84 0.15

-3 12 -1 1.73 0.14

3 12 -1 1.87 0.13

-3 12 1 2.10 0.17

-3 12 -1 2.20 0.17

3 12 1 1.93 0.16

3 12 -1 2.07 0.16

-3 13 -1 0.13 0.06

3 13 -1 0.28 0.08

-3 13 1 0.07 0.05

3 13 1 0.18 0.07

-3 13 -1 0.08 0.06

3 13 -1 0.06 0.06

3 13 1 0.05 0.06

-3 13 1 0.09 0.06

-3 14 -1 0.11 0.04

-3 14 1 0.16 0.05

3 14 1 0.06 0.05

-3 14 1 0.06 0.05

-3 14 -1 0.07 0.05

3 14 -1 0.09 0.05

3 0 -2 0.01 0.03

3 0 2 -0.04 0.04

-3 1 2 39.66 0.41

3 -1 -2 39.89 0.58

3 1 -2 40.34 0.55

3 2 2 91.67 0.86

-3 2 2 91.49 0.87

3 2 2 88.53 0.89

3 -2 -2 90.28 0.97

3 3 2 65.64 0.85

3 3 -2 68.13 0.92

3 3 -2 65.49 0.67

-3 3 2 65.77 0.85

3 4 2 14.29 0.42

3 -4 2 13.88 0.34

3 4 -2 13.22 0.21

3 -4 -2 14.75 0.39

-3 4 2 15.27 0.45

-3 4 2 14.29 0.40

3 -4 -2 14.30 0.25

-3 4 -2 15.25 0.46

3 4 -2 13.93 0.46

3 4 2 13.78 0.43

-3 5 2 3.69 0.26

3 5 2 3.59 0.25

-3 5 -2 3.56 0.25

3 5 -2 3.54 0.27

-3 5 2 3.45 0.23

3 5 2 3.39 0.25

-3 6 2 20.19 0.65

3 6 2 18.39 0.61

3 6 2 19.58 0.64

-3 6 -2 16.50 0.61

3 6 -2 17.92 0.67

-3 6 2 18.42 0.59

3 6 -2 17.28 0.63

-3 7 2 8.33 0.46

3 7 2 8.40 0.45

-3 7 2 8.07 0.43

3 7 -2 7.83 0.46

3 7 2 7.59 0.44

-3 7 -2 7.51 0.45

3 7 -2 7.74 0.47

-3 7 -2 8.32 0.50

-3 8 2 4.60 0.38

3 8 2 4.44 0.36

3 8 -2 4.47 0.39

-3 8 2 4.36 0.35

3 8 -2 4.40 0.40

3 8 2 4.10 0.35

-3 8 -2 3.99 0.36

-3 8 -2 4.60 0.40

-3 9 -2 0.14 0.05

3 9 2 0.04 0.04

-3 9 2 0.16 0.06

-3 9 2 0.08 0.04

3 9 -2 0.12 0.04

3 9 2 0.08 0.05

-3 9 -2 0.15 0.05

3 9 2 0.08 0.05

3 9 -2 0.13 0.05

-3 9 2 0.07 0.05

-3 9 -2 0.18 0.06

3 9 -2 0.14 0.06

-3 9 2 0.13 0.11

-3 9 -2 0.06 0.09

3 9 2 0.25 0.13

3 9 -2 0.17 0.13

3 9 -2 0.16 0.13

-3 9 -2 0.10 0.12

3 9 -2 0.18 0.06

-3 10 -2 7.89 0.57

3 10 -2 9.03 0.63

-3 10 -2 8.47 0.30

3 10 -2 8.94 0.32

3 10 2 9.35 0.60

3 10 2 9.29 0.30

-3 10 -2 9.10 0.30

3 10 -2 8.59 0.61

-3 10 -2 9.16 0.65

3 10 -2 8.32 0.27

-3 10 2 9.60 0.62

3 10 2 9.66 0.34

-3 10 2 9.55 0.32

-3 10 2 9.29 0.32

3 10 2 9.71 0.32

-3 10 2 10.19 0.33

3 10 -2 9.20 0.32

-3 10 -2 9.08 0.32

3 11 2 0.08 0.07

-3 11 2 0.19 0.08

3 11 -2 0.12 0.07

3 11 -2 0.22 0.09

-3 11 2 0.10 0.07

3 11 2 0.09 0.06

-3 11 -2 -0.06 0.07

3 11 2 -0.05 0.07

-3 11 2 -0.10 0.08

3 11 -2 0.05 0.05

-3 11 -2 0.14 0.08

-3 12 -2 0.26 0.06

3 12 2 0.58 0.11

3 12 -2 0.39 0.09

3 12 2 0.38 0.08

3 12 -2 0.62 0.11

-3 12 2 0.33 0.09

3 12 -2 0.55 0.08

-3 12 -2 0.36 0.09

-3 12 2 0.37 0.09

3 12 2 0.46 0.10

-3 13 -2 0.06 0.04

-3 13 2 0.07 0.05

-3 13 -2 0.11 0.07

3 13 -2 0.06 0.07

3 13 2 0.29 0.08

-3 13 2 0.07 0.06

3 13 2 0.12 0.07

-3 14 -2 0.43 0.06

-3 14 2 0.56 0.07

3 14 2 0.51 0.07

-3 14 2 0.52 0.08

-3 14 -2 0.47 0.08

3 14 -2 0.51 0.08

-3 15 -2 0.11 0.04

3 15 -2 0.13 0.04

3 0 -3 0.04 0.05

3 -1 -3 6.38 0.25

3 1 -3 6.18 0.24

3 -2 -3 133.70 1.34

3 2 -3 134.45 1.25

-3 2 3 133.41 1.12

3 3 -3 22.96 0.44

3 -3 -3 22.10 0.50

-3 3 3 22.59 0.47

3 4 3 19.35 0.50

3 4 -3 18.10 0.37

-3 4 3 18.71 0.45

3 -4 -3 17.25 0.45

3 4 -3 18.82 0.55

-3 -4 -3 18.08 0.31

3 4 3 19.35 0.36

3 -4 3 19.44 0.45

-3 4 -3 17.71 0.51

3 4 3 17.70 0.48

-3 4 3 18.47 0.50

-3 5 3 1.80 0.17

3 5 3 1.93 0.19

3 -5 3 2.07 0.14

3 -5 -3 1.66 0.14

3 5 -3 1.85 0.20

-3 5 -3 1.85 0.20

3 5 3 1.88 0.18

-3 5 3 1.97 0.20

-3 6 3 0.57 0.13

3 6 -3 0.56 0.15

-3 6 3 0.69 0.12

-3 6 -3 0.53 0.14

3 6 3 0.50 0.11

3 6 3 0.59 0.13

3 7 -3 7.93 0.47

3 7 3 7.80 0.44

3 7 -3 7.59 0.48

-3 7 -3 7.84 0.47

3 7 3 7.72 0.43

-3 7 3 7.92 0.45

3 8 -3 6.44 0.47

-3 8 -3 6.09 0.48

-3 8 -3 5.65 0.44

3 8 -3 6.81 0.49

-3 8 3 6.45 0.44

3 8 3 7.02 0.44

3 9 -3 1.11 0.11

-3 9 3 1.03 0.11

3 9 3 0.97 0.11

3 9 3 0.91 0.09

-3 9 -3 1.20 0.12

-3 9 -3 0.97 0.10

-3 9 -3 1.19 0.11

3 9 -3 0.85 0.09

3 9 -3 1.04 0.11

-3 9 3 1.07 0.11

-3 9 -3 0.89 0.21

-3 9 -3 0.86 0.19

3 9 -3 0.97 0.20

-3 9 3 0.85 0.20

3 9 3 1.01 0.21

3 9 -3 1.05 0.22

3 9 -3 0.94 0.11

3 9 3 0.94 0.10

-3 9 3 0.97 0.10

3 10 -3 8.84 0.27

-3 10 -3 8.45 0.29

-3 10 -3 8.85 0.30

3 10 3 9.29 0.30

-3 10 3 9.41 0.32

-3 10 3 9.87 0.62

3 10 -3 9.56 0.32

3 10 3 9.94 0.61

3 10 -3 9.46 0.64

3 10 3 9.21 0.33

-3 10 -3 9.53 0.33

-3 10 3 9.41 0.32

-3 10 -3 9.62 0.68

3 10 -3 9.14 0.33

-3 10 -3 8.86 0.61

3 10 -3 9.01 0.64

3 10 -3 8.34 0.32

3 10 3 9.87 0.32

-3 10 3 10.16 0.33

3 11 -3 0.12 0.07

-3 11 -3 -0.05 0.06

3 11 3 0.08 0.07

3 11 -3 0.06 0.04

-3 11 3 0.19 0.08

-3 11 -3 0.07 0.07

3 11 -3 0.12 0.08

3 11 3 0.13 0.07

-3 11 3 -0.10 0.08

-3 11 3 0.01 0.06

3 11 3 0.11 0.06

-3 12 -3 0.23 0.06

3 12 -3 0.45 0.09

-3 12 3 0.38 0.08

3 12 3 0.41 0.08

3 12 3 0.55 0.11

-3 12 -3 0.11 0.07

3 12 -3 0.18 0.07

3 12 -3 0.35 0.06

3 12 3 0.31 0.09

-3 12 3 0.36 0.09

3 13 -3 0.03 0.06

-3 13 3 0.05 0.06

-3 13 -3 0.03 0.06

-3 13 -3 -0.01 0.04

3 13 3 0.14 0.08

3 13 3 0.07 0.06

-3 13 3 0.03 0.06

-3 14 3 1.29 0.10

3 14 -3 1.24 0.11

-3 14 -3 1.22 0.11

-3 15 -3 0.03 0.03

3 15 -3 0.08 0.04

3 0 -4 0.01 0.06

3 1 -4 53.05 0.80

3 -1 -4 53.86 0.83

-3 2 4 93.46 0.91

3 2 -4 93.37 1.11

3 -2 -4 95.50 1.18

-3 3 4 60.50 0.81

3 3 -4 61.10 0.86

3 -3 -4 60.74 0.93

3 4 4 10.34 0.36

-3 4 4 10.69 0.36

3 4 -4 9.80 0.32

-3 -4 -4 9.54 0.29

3 -4 4 10.11 0.37

3 4 -4 9.89 0.41

3 4 4 10.51 0.31

-3 5 4 2.97 0.22

3 5 4 3.22 0.23

3 5 -4 3.31 0.28

3 5 -4 3.06 0.16

3 -5 -4 3.34 0.22

-3 5 -4 3.14 0.26

3 -5 4 3.28 0.21

-3 6 4 2.31 0.23

3 6 4 2.49 0.23

3 6 -4 2.15 0.25

-3 6 -4 1.92 0.23

3 7 4 7.50 0.42

-3 7 4 7.18 0.42

3 7 -4 7.95 0.50

3 7 -4 7.32 0.47

-3 7 -4 7.52 0.48

3 8 4 3.93 0.34

-3 8 4 4.16 0.35

3 8 -4 3.33 0.35

-3 8 -4 3.29 0.35

3 8 -4 4.08 0.39

3 9 4 0.08 0.04

-3 9 -4 0.14 0.05

-3 9 4 0.18 0.06

-3 9 4 0.16 0.06

3 9 4 0.06 0.05

3 9 -4 0.01 0.03

-3 9 -4 0.10 0.05

-3 9 4 0.13 0.06

3 9 4 0.10 0.05

-3 9 -4 0.05 0.05

3 9 -4 0.09 0.05

3 9 -4 0.07 0.05

3 9 -4 0.12 0.12

-3 9 -4 0.04 0.10

3 9 4 0.09 0.11

-3 9 -4 0.19 0.11

3 9 -4 0.01 0.10

-3 9 4 0.20 0.12

-3 9 -4 0.16 0.07

3 9 -4 0.12 0.07

3 10 4 9.71 0.31

-3 10 4 9.59 0.60

3 10 -4 10.07 0.67

3 10 -4 10.25 0.32

-3 10 -4 10.23 0.35

3 10 -4 9.53 0.28

-3 10 4 9.62 0.33

3 10 4 9.81 0.59

3 10 4 9.35 0.34

-3 10 -4 9.35 0.30

-3 10 4 9.14 0.32

3 10 -4 10.01 0.35

-3 10 -4 9.56 0.65

-3 10 -4 10.05 0.70

3 10 -4 9.99 0.69

3 10 -4 8.88 0.34

3 10 4 9.59 0.32

-3 10 4 10.27 0.33

3 11 -4 0.14 0.07

3 11 4 0.12 0.07

-3 11 -4 -0.04 0.06

-3 11 4 0.23 0.08

-3 11 4 -0.01 0.07

3 11 -4 0.14 0.05

3 11 -4 0.12 0.08

-3 11 -4 0.18 0.08

3 11 4 0.15 0.07

-3 11 4 0.14 0.07

3 11 4 0.09 0.07

-3 12 -4 1.93 0.13

3 12 4 2.40 0.18

-3 12 4 1.83 0.15

3 12 4 1.93 0.14

3 12 -4 2.12 0.15

3 12 -4 2.08 0.17

-3 12 4 2.06 0.16

3 12 4 2.05 0.16

-3 12 -4 2.30 0.17

-3 13 -4 0.06 0.03

3 13 4 0.15 0.06

-3 13 4 0.13 0.06

3 13 -4 -0.04 0.06

-3 13 -4 0.09 0.06

-3 13 4 0.03 0.05

3 13 4 0.04 0.06

-3 14 -4 1.11 0.10

3 14 -4 1.26 0.11

-3 15 -4 0.03 0.03

3 15 -4 0.04 0.03

3 0 -5 0.08 0.08

3 -1 -5 4.56 0.26

3 1 -5 4.57 0.25

3 -2 -5 64.97 1.03

3 2 -5 66.15 0.98

3 3 5 12.49 0.40

3 -3 -5 12.63 0.45

3 3 -5 12.56 0.41

3 -3 5 12.05 0.43

-3 3 5 12.73 0.35

3 4 5 5.43 0.26

3 -4 5 5.42 0.29

3 -4 -5 5.17 0.29

3 4 -5 4.92 0.26

-3 -4 -5 5.34 0.25

-3 4 5 5.66 0.27

3 5 -5 2.83 0.19

3 -5 -5 2.77 0.21

3 5 -5 3.14 0.28

3 -5 5 3.31 0.23

-3 5 5 3.55 0.24

3 5 5 3.02 0.23

3 6 -5 13.21 0.62

-3 6 -5 12.86 0.58

3 6 5 14.21 0.53

-3 6 5 13.45 0.53

3 7 -5 9.75 0.57

-3 7 -5 9.04 0.54

3 7 5 8.76 0.46

-3 7 5 9.12 0.47

3 8 5 6.64 0.23

-3 8 5 6.52 0.44

3 8 5 6.88 0.44

-3 8 5 6.76 0.25

3 8 5 6.78 0.26

3 8 -5 7.04 0.50

3 8 -5 7.06 0.53

-3 8 -5 6.53 0.49

-3 8 -5 6.53 0.27

-3 8 5 6.79 0.24

3 8 5 6.74 0.24

3 9 5 0.06 0.05

-3 9 -5 0.30 0.06

-3 9 -5 0.20 0.06

-3 9 -5 0.14 0.06

3 9 -5 0.23 0.06

-3 9 5 0.12 0.06

3 9 5 0.04 0.05

3 9 -5 0.13 0.05

-3 9 5 0.09 0.05

-3 9 -5 -0.05 0.14

3 9 -5 0.09 0.10

3 9 -5 0.12 0.12

-3 9 -5 0.02 0.13

-3 9 5 -0.01 0.09

3 9 -5 0.23 0.07

-3 9 -5 0.25 0.08

-3 9 5 0.17 0.06

3 9 5 0.12 0.05

-3 10 5 1.97 0.15

3 10 -5 1.98 0.32

-3 10 5 2.00 0.28

-3 10 -5 2.14 0.17

3 10 5 1.93 0.16

3 10 -5 2.00 0.17

3 10 5 2.11 0.15

-3 10 -5 2.04 0.14

3 10 -5 2.21 0.15

-3 10 5 2.03 0.16

3 10 -5 2.02 0.33

-3 10 -5 2.07 0.32

-3 10 -5 2.01 0.34

3 10 -5 2.08 0.18

3 10 5 2.01 0.15

-3 10 5 2.06 0.16

3 11 5 0.44 0.09

3 11 5 0.44 0.10

3 11 -5 0.46 0.08

-3 11 5 0.47 0.09

-3 11 -5 0.34 0.07

3 11 -5 0.48 0.11

-3 11 -5 0.38 0.09

-3 11 5 0.55 0.10

-3 11 5 0.45 0.09

3 11 5 0.55 0.10

-3 12 -5 0.04 0.04

3 12 5 0.19 0.07

3 12 -5 0.21 0.09

3 12 5 0.26 0.09

-3 12 5 0.11 0.07

-3 12 -5 0.27 0.09

-3 12 5 0.11 0.07

3 12 5 0.06 0.06

3 13 5 0.20 0.07

-3 13 5 0.22 0.07

-3 13 -5 0.12 0.06

3 13 -5 0.16 0.07

-3 14 -5 0.14 0.05

3 14 -5 0.15 0.05

-3 15 -5 0.05 0.03

3 15 -5 0.03 0.03

3 0 -6 0.21 0.12

3 1 -6 9.10 0.39

3 -1 -6 9.15 0.40

3 2 -6 54.14 0.95

3 -2 -6 53.27 0.99

3 3 6 19.71 0.55

3 3 -6 19.71 0.56

3 -3 -6 19.48 0.60

3 -3 6 19.87 0.60

3 -4 6 11.22 0.46

3 4 6 11.28 0.41

-3 -4 -6 11.33 0.42

3 -4 -6 10.15 0.44

3 4 -6 10.43 0.42

3 -5 6 0.75 0.13

3 5 -6 0.68 0.11

3 5 -6 0.84 0.17

-3 5 6 0.69 0.12

3 -5 -6 0.73 0.13

-3 6 -6 16.77 0.69

3 6 -6 18.64 0.74

-3 6 6 17.01 0.58

3 7 -6 4.51 0.40

-3 7 -6 4.26 0.38

-3 7 6 3.93 0.31

3 8 6 1.69 0.13

3 8 6 1.75 0.12

-3 8 6 1.84 0.24

-3 8 6 2.00 0.13

-3 8 6 1.90 0.14

3 8 -6 1.94 0.28

3 8 -6 1.76 0.30

-3 8 -6 1.68 0.27

-3 8 -6 1.94 0.16

3 8 -6 1.94 0.16

3 8 6 1.72 0.12

-3 8 6 1.87 0.13

-3 9 6 0.01 0.05

3 9 6 0.04 0.05

-3 9 6 0.16 0.06

3 9 6 0.06 0.05

-3 9 -6 -0.07 0.07

3 9 -6 0.08 0.05

3 9 -6 0.00 0.07

-3 9 -6 0.05 0.14

3 9 -6 0.03 0.10

-3 9 -6 0.04 0.12

3 9 -6 -0.15 0.14

3 9 -6 0.09 0.07

-3 9 -6 0.04 0.07

3 9 6 0.03 0.05

-3 9 6 0.08 0.05

3 10 6 0.37 0.08

-3 10 6 0.21 0.07

3 10 6 0.32 0.08

3 10 -6 0.33 0.08

-3 10 -6 0.39 0.09

-3 10 6 0.42 0.08

3 10 -6 0.34 0.07

3 10 -6 0.32 0.16

-3 10 -6 0.41 0.19

3 10 -6 0.17 0.14

-3 10 -6 0.05 0.11

3 10 -6 0.29 0.09

3 10 6 0.42 0.09

-3 10 6 0.36 0.08

3 11 6 0.38 0.09

3 11 6 0.39 0.09

-3 11 6 0.29 0.08

3 11 -6 0.46 0.08

-3 11 -6 0.34 0.10

3 11 -6 0.37 0.09

-3 11 6 0.45 0.09

3 11 -6 0.46 0.10

3 11 6 0.41 0.09

-3 11 6 0.43 0.09

3 12 6 1.58 0.15

-3 12 6 1.71 0.15

-3 12 -6 1.71 0.15

3 12 -6 1.64 0.15

-3 13 6 0.18 0.06

3 13 -6 0.11 0.06

3 13 6 0.22 0.07

-3 13 -6 0.15 0.06

-3 14 -6 0.02 0.04

3 14 -6 0.05 0.04

3 15 -6 0.01 0.02

-3 15 -6 -0.02 0.02

3 0 -7 -0.02 0.09

3 -1 7 4.11 0.29

3 1 7 4.15 0.28

3 1 -7 3.33 0.26

3 -1 -7 3.31 0.27

3 -2 -7 23.74 0.70

3 -2 7 22.93 0.68

3 2 7 23.01 0.66

3 2 -7 22.61 0.66

3 3 7 24.27 0.67

3 -3 7 23.13 0.70

3 -3 -7 22.72 0.70

3 3 -7 23.28 0.67

3 4 7 51.54 0.99

3 -4 7 49.13 1.07

3 4 -7 50.89 1.01

-3 -4 -7 52.68 1.01

3 -4 -7 49.26 1.07

3 -5 -7 0.66 0.13

3 5 -7 0.91 0.14

3 -5 7 0.87 0.15

-3 -5 -7 0.78 0.12

3 5 7 0.77 0.12

3 6 -7 8.00 0.51

-3 6 -7 7.36 0.47

-3 7 -7 3.48 0.36

3 7 -7 3.72 0.38

3 8 7 1.33 0.12

-3 8 7 1.45 0.12

-3 8 7 1.31 0.11

3 8 7 1.40 0.11

3 8 -7 1.61 0.28

-3 8 -7 1.13 0.23

3 8 -7 1.42 0.14

-3 8 -7 1.29 0.12

-3 8 -7 1.39 0.14

-3 8 7 1.38 0.11

3 8 7 1.43 0.11

3 9 -7 1.78 0.12

-3 9 7 1.77 0.14

-3 9 7 1.73 0.14

3 9 7 1.74 0.15

3 9 7 1.92 0.14

3 9 -7 2.01 0.33

3 9 -7 1.81 0.29

-3 9 -7 1.82 0.30

3 9 -7 1.81 0.17

-3 9 -7 1.64 0.15

-3 9 -7 1.91 0.17

-3 9 7 1.77 0.14

3 9 7 1.69 0.13

3 10 -7 1.48 0.15

3 10 7 1.26 0.13

3 10 7 1.50 0.13

-3 10 -7 1.48 0.15

-3 10 7 1.48 0.14

-3 10 7 1.27 0.12

3 10 -7 1.48 0.12

-3 10 -7 1.48 0.30

-3 10 -7 1.28 0.25

3 10 -7 1.34 0.26

3 10 -7 1.44 0.16

-3 10 7 1.49 0.13

3 10 7 1.43 0.13

3 11 7 0.84 0.11

-3 11 7 0.73 0.10

3 11 -7 0.72 0.12

3 11 7 0.59 0.10

-3 11 7 0.71 0.11

-3 11 -7 0.67 0.11

3 11 -7 0.95 0.13

-3 12 7 2.98 0.18

3 12 7 3.05 0.18

-3 12 -7 2.98 0.20

3 12 -7 3.00 0.19

-3 13 -7 0.01 0.06

-3 13 7 0.08 0.05

3 13 7 0.08 0.05

3 13 -7 0.02 0.06

-3 14 -7 0.12 0.04

3 14 -7 0.21 0.05

3 0 8 0.23 0.10

3 0 -8 -0.02 0.10

3 1 8 24.23 0.73

3 -1 8 24.79 0.75

3 -1 -8 23.61 0.73

3 1 -8 22.76 0.71

3 -2 8 17.82 0.65

3 2 8 17.77 0.62

3 2 -8 18.49 0.65

3 -2 -8 19.58 0.69

3 -3 8 15.90 0.62

3 3 -8 14.94 0.58

3 -3 -8 15.92 0.62

3 3 8 16.00 0.58

3 4 -8 47.93 1.06

3 -4 8 47.37 1.11

3 4 8 47.79 1.03

3 -4 -8 48.70 1.13

-3 -4 -8 49.58 1.07

-3 -5 -8 0.03 0.06

3 -5 -8 0.04 0.08

3 -5 8 0.11 0.09

3 5 -8 0.11 0.08

3 6 -8 10.44 0.59

3 7 -8 4.15 0.40

-3 7 -8 3.85 0.39

-3 8 8 6.82 0.25

-3 8 8 6.45 0.24

3 8 8 6.21 0.25

3 8 8 6.81 0.24

-3 8 -8 6.15 0.52

3 8 -8 6.80 0.56

-3 8 -8 6.10 0.27

3 8 -8 7.08 0.31

-3 8 -8 7.51 0.30

-3 8 8 6.22 0.23

3 8 8 6.10 0.23

3 9 8 2.00 0.15

-3 9 8 2.22 0.15

3 9 8 2.23 0.15

-3 9 8 2.19 0.15

3 9 -8 1.99 0.30

3 9 -8 2.39 0.35

-3 9 -8 1.89 0.31

3 9 -8 2.12 0.18

-3 9 -8 2.16 0.18

-3 9 -8 2.05 0.16

-3 9 8 1.94 0.14

3 9 8 1.75 0.13

3 10 8 0.40 0.08

3 10 8 0.24 0.07

-3 10 8 0.43 0.08

-3 10 -8 0.31 0.09

-3 10 8 0.40 0.08

3 10 -8 0.26 0.08

3 10 -8 0.33 0.10

-3 10 -8 0.56 0.12

-3 11 8 0.24 0.07

3 11 8 0.40 0.08

3 11 8 0.21 0.07

-3 11 -8 0.18 0.08

-3 11 8 0.30 0.08

3 11 -8 0.21 0.08

3 11 -8 0.44 0.11

3 12 8 2.65 0.17

-3 12 8 2.90 0.17

3 12 -8 2.71 0.18

-3 12 -8 3.04 0.19

3 13 -8 0.18 0.07

-3 13 -8 0.12 0.06

3 13 8 0.21 0.06

-3 14 -8 0.02 0.04

3 14 -8 0.08 0.04

3 0 9 -0.03 0.11

3 0 -9 0.07 0.12

3 1 -9 9.65 0.50

3 -1 9 9.33 0.50

3 -1 -9 9.71 0.50

3 1 9 9.80 0.50

3 -2 9 7.93 0.46

3 2 9 7.98 0.45

3 2 -9 8.98 0.48

3 -2 -9 8.55 0.48

3 -3 9 3.81 0.33

3 -3 -9 3.85 0.34

3 3 -9 3.58 0.31

3 3 9 3.75 0.31

3 -4 -9 26.27 0.88

-3 -4 -9 26.36 0.83

3 4 9 23.99 0.78

3 -4 9 24.40 0.85

3 4 -9 25.98 0.83

-3 -5 -9 0.84 0.16

3 -5 9 0.92 0.17

3 5 -9 0.68 0.14

3 -5 -9 0.74 0.16

3 6 -9 5.82 0.46

-3 7 9 4.47 0.19

3 7 9 4.71 0.19

3 7 -9 4.32 0.24

-3 7 -9 4.54 0.23

-3 7 -9 3.82 0.40

3 7 -9 4.19 0.42

-3 8 9 2.83 0.16

-3 8 9 2.82 0.16

3 8 9 2.65 0.17

3 8 9 2.67 0.15

-3 8 -9 2.72 0.19

3 8 -9 3.26 0.21

-3 8 -9 3.22 0.21

3 8 -9 3.05 0.38

-3 8 -9 2.46 0.34

-3 9 9 0.27 0.06

3 9 9 0.17 0.06

3 9 9 0.04 0.05

-3 9 9 0.15 0.06

-3 9 -9 0.16 0.08

3 9 -9 0.15 0.09

-3 9 -9 0.16 0.06

3 9 -9 0.12 0.16

3 9 -9 -0.17 0.19

-3 9 -9 0.20 0.09

3 10 9 0.59 0.09

-3 10 9 0.75 0.09

-3 10 -9 0.60 0.10

3 10 -9 0.53 0.10

-3 10 9 0.74 0.10

3 10 9 0.87 0.10

-3 10 -9 0.97 0.15

3 10 -9 0.74 0.13

-3 11 9 0.81 0.09

3 11 -9 0.62 0.11

-3 11 -9 0.56 0.11

3 11 9 0.81 0.11

-3 11 9 0.74 0.10

3 11 -9 0.74 0.12

-3 12 9 0.49 0.08

3 12 9 0.46 0.08

-3 12 -9 0.44 0.09

3 12 -9 0.63 0.10

-3 13 -9 0.06 0.05

3 13 -9 0.02 0.05

3 14 -9 0.03 0.03

-3 14 -9 0.07 0.04

3 0 10 -0.04 0.14

3 0 -10 -0.02 0.12

3 -1 10 9.64 0.54

3 1 10 9.37 0.52

3 -1 -10 8.95 0.53

3 1 -10 8.26 0.49

3 2 10 3.13 0.30

3 -2 -10 3.06 0.32

3 2 -10 3.15 0.32

3 -2 10 3.01 0.31

3 -3 -10 16.83 0.73

3 -3 10 16.59 0.72

3 3 10 15.62 0.67

3 3 -10 15.17 0.67

3 -4 10 2.32 0.28

3 -4 -10 2.59 0.30

3 4 -10 2.36 0.27

3 4 10 2.26 0.26

-3 -4 -10 2.22 0.26

-3 -5 -10 0.80 0.16

3 5 -10 0.88 0.17

3 -5 10 0.73 0.18

3 -5 -10 0.86 0.18

3 7 10 2.03 0.12

-3 7 10 1.87 0.12

3 7 10 1.88 0.13

-3 7 -10 1.91 0.30

3 7 -10 2.29 0.33

3 7 -10 2.30 0.18

-3 7 -10 2.11 0.17

-3 7 -10 2.05 0.16

3 8 10 0.75 0.08

3 8 10 0.66 0.09

-3 8 10 0.66 0.08

-3 8 -10 0.64 0.20

3 8 -10 0.70 0.21

3 8 -10 0.86 0.13

-3 8 -10 0.84 0.12

-3 8 -10 0.53 0.10

-3 9 10 0.75 0.09

3 9 10 0.66 0.09

3 9 10 0.83 0.09

-3 9 10 0.74 0.09

3 9 -10 0.73 0.13

-3 9 -10 0.64 0.10

-3 9 -10 0.95 0.13

3 9 -10 0.61 0.20

-3 9 -10 0.40 0.15

3 9 -10 0.83 0.23

-3 10 10 0.41 0.08

-3 10 -10 0.23 0.08

3 10 -10 0.33 0.09

-3 10 10 0.60 0.08

3 10 10 0.38 0.07

-3 10 -10 0.54 0.12

3 10 -10 0.47 0.11

-3 10 -10 0.55 0.09

3 11 -10 0.30 0.09

3 11 10 0.56 0.09

-3 11 -10 0.29 0.09

-3 11 10 0.55 0.09

3 11 -10 0.54 0.11

3 12 -10 0.12 0.07

3 12 10 0.05 0.04

-3 12 -10 0.03 0.06

-3 12 10 0.01 0.04

3 13 -10 0.10 0.05

-3 13 -10 0.12 0.05

-3 14 -10 0.15 0.04

3 14 -10 0.22 0.04

3 0 -11 -0.01 0.14

3 0 11 0.06 0.14

3 -1 11 5.56 0.44

3 1 11 5.58 0.43

3 1 -11 5.42 0.44

3 -1 -11 5.95 0.45

3 2 -11 1.10 0.23

3 -2 11 1.09 0.22

3 -2 -11 1.37 0.26

3 2 11 1.04 0.21

3 3 11 6.48 0.46

3 -3 11 6.52 0.49

3 3 -11 6.40 0.47

3 -3 -11 6.67 0.49

3 -4 -11 8.00 0.55

3 -4 11 6.89 0.51

-3 -4 -11 8.13 0.52

3 4 11 6.97 0.48

3 4 -11 7.08 0.49

-3 -5 -11 0.36 0.14

3 -5 11 0.14 0.09

3 5 -11 0.21 0.11

3 -5 -11 0.31 0.11

3 6 -11 1.92 0.16

-3 6 -11 1.75 0.15

-3 6 -11 1.74 0.15

-3 6 11 1.51 0.14

-3 7 11 2.36 0.14

3 7 11 2.41 0.13

-3 7 -11 2.18 0.32

3 7 -11 2.49 0.34

3 7 -11 2.59 0.19

3 7 -11 1.97 0.15

-3 7 -11 2.42 0.18

-3 7 -11 2.44 0.18

3 8 11 0.48 0.08

-3 8 11 0.59 0.08

3 8 -11 0.53 0.18

-3 8 -11 0.57 0.18

3 8 -11 0.57 0.11

-3 8 -11 0.70 0.12

-3 8 -11 0.38 0.08

3 9 11 0.50 0.08

-3 9 11 0.48 0.07

3 9 -11 0.65 0.13

-3 9 -11 0.56 0.11

-3 9 -11 0.37 0.09

3 10 -11 -0.16 0.10

-3 10 11 -0.03 0.05

-3 10 -11 -0.20 0.10

3 10 11 0.06 0.05

-3 10 -11 0.06 0.06

3 10 -11 0.03 0.08

-3 10 -11 0.07 0.09

3 11 11 0.59 0.08

-3 11 -11 0.46 0.09

-3 11 11 0.50 0.08

3 11 -11 0.45 0.09

3 11 -11 0.66 0.12

3 12 -11 -0.02 0.06

3 12 11 0.05 0.04

-3 12 -11 0.05 0.07

3 13 -11 0.03 0.04

-3 13 -11 0.01 0.04

3 14 -11 0.07 0.03

-3 14 -11 0.07 0.03

3 0 -12 -0.09 0.15

3 0 12 -0.04 0.15

3 1 12 22.95 0.92

3 -1 -12 24.19 0.96

3 -1 12 23.16 0.94

3 1 -12 23.38 0.93

3 2 -12 1.98 0.30

3 -2 -12 2.09 0.31

3 -2 12 1.81 0.29

3 2 12 1.94 0.29

-3 -3 -12 2.84 0.34

3 -3 12 2.84 0.34

3 3 12 2.50 0.30

3 3 -12 2.20 0.30

3 -3 -12 2.61 0.34

3 -4 -12 5.55 0.48

3 4 12 5.28 0.44

-3 -4 -12 6.01 0.48

3 4 -12 5.59 0.46

3 -4 12 5.51 0.48

3 5 -12 -0.08 0.09

3 -5 -12 0.19 0.12

3 5 12 0.04 0.06

-3 6 -12 3.52 0.21

3 6 -12 3.80 0.22

-3 6 -12 3.31 0.21

3 6 -12 3.15 0.20

-3 6 12 3.65 0.23

-3 7 12 2.24 0.13

3 7 -12 2.15 0.17

3 7 -12 2.93 0.21

-3 7 -12 2.41 0.19

-3 7 -12 2.60 0.19

3 7 -12 2.25 0.34

-3 7 -12 2.04 0.32

-3 8 12 2.76 0.15

-3 8 -12 3.20 0.21

3 8 -12 3.30 0.23

-3 8 -12 2.36 0.19

3 8 -12 2.83 0.40

-3 8 -12 2.37 0.37

3 9 12 0.82 0.10

-3 9 12 0.70 0.09

-3 9 -12 0.90 0.13

-3 9 -12 0.63 0.10

3 9 -12 0.80 0.14

3 10 12 3.01 0.17

-3 10 12 2.94 0.16

3 10 -12 2.91 0.22

-3 10 -12 3.20 0.22

3 11 -12 0.47 0.09

3 11 12 0.58 0.08

-3 11 -12 0.37 0.09

3 11 -12 0.67 0.12

-3 12 -12 0.10 0.06

3 12 -12 0.02 0.05

3 13 -12 0.13 0.05

-3 13 -12 0.00 0.04

3 0 -13 0.05 0.13

3 0 13 -0.24 0.21

3 1 -13 1.04 0.23

3 -1 -13 1.01 0.23

3 1 13 1.05 0.22

3 -1 13 1.17 0.23

3 -2 -13 16.23 0.84

3 -2 13 16.06 0.84

3 2 -13 15.17 0.80

3 2 13 14.68 0.78

3 -3 -13 5.81 0.51

-3 -3 -13 6.35 0.52

3 -3 13 5.54 0.50

3 3 13 5.91 0.50

3 3 -13 5.83 0.50

3 -4 -13 16.85 0.87

3 -4 13 17.36 0.90

3 4 -13 16.56 0.84

-3 -4 -13 17.69 0.87

3 -5 -13 1.01 0.22

3 5 -13 0.99 0.22

3 5 -13 1.03 0.12

-3 5 -13 1.07 0.11

-3 5 -13 0.98 0.12

-3 5 13 1.26 0.14

3 5 13 1.13 0.13

3 6 -13 0.80 0.11

-3 6 -13 0.70 0.10

-3 6 -13 0.76 0.11

3 6 -13 0.69 0.10

-3 6 13 0.76 0.12

-3 7 -13 2.11 0.17

-3 7 -13 1.98 0.17

3 7 -13 2.12 0.19

3 7 -13 1.51 0.15

3 7 -13 1.79 0.30

-3 7 -13 1.85 0.32

-3 8 13 3.30 0.16

-3 8 -13 3.31 0.22

-3 8 -13 3.01 0.21

3 8 -13 3.71 0.25

-3 9 13 0.75 0.08

-3 9 -13 0.88 0.13

3 9 -13 0.90 0.14

-3 9 -13 0.69 0.11

3 10 -13 2.30 0.20

-3 10 -13 2.57 0.20

-3 11 -13 0.31 0.07

3 11 -13 0.29 0.08

3 12 -13 0.26 0.07

-3 12 -13 0.21 0.06

3 13 -13 0.02 0.03

-3 13 -13 0.03 0.03

3 0 -14 -0.05 0.16

3 0 14 -0.06 0.16

3 1 -14 4.25 0.46

3 -1 -14 4.52 0.47

3 1 14 4.97 0.47

3 -1 14 5.47 0.51

3 -2 14 5.28 0.50

-3 -2 -14 5.75 0.52

3 2 14 4.96 0.48

3 -2 -14 5.21 0.51

3 2 -14 5.15 0.49

3 -3 14 4.43 0.48

-3 -3 -14 4.54 0.47

3 3 -14 4.04 0.45

3 3 14 4.72 0.47

3 -3 -14 4.40 0.46

-3 3 14 5.72 0.27

3 -4 -14 10.73 0.74

3 4 -14 10.13 0.69

-3 -4 -14 10.73 0.72

3 4 -14 11.87 0.37

-3 4 -14 10.93 0.37

-3 4 14 11.54 0.40

3 4 14 12.51 0.41

3 -5 -14 1.26 0.26

3 5 -14 1.58 0.29

-3 5 -14 1.92 0.16

3 5 -14 1.92 0.16

-3 5 -14 1.86 0.15

-3 5 14 1.77 0.17

3 6 -14 1.00 0.14

-3 6 -14 0.84 0.12

-3 6 -14 1.00 0.13

3 6 -14 1.03 0.13

-3 6 14 1.03 0.14

3 7 -14 2.34 0.20

3 7 -14 1.80 0.16

-3 7 -14 2.19 0.18

-3 7 -14 2.03 0.18

-3 7 -14 1.56 0.30

3 7 -14 2.17 0.35

3 8 -14 1.58 0.18

-3 8 -14 1.06 0.14

3 8 -14 0.90 0.12

-3 8 -14 1.48 0.15

3 9 -14 1.29 0.16

-3 9 -14 1.23 0.15

-3 9 -14 0.84 0.12

3 10 -14 0.79 0.13

-3 10 -14 0.51 0.09

3 11 -14 0.07 0.06

-3 11 -14 0.06 0.06

-3 12 -14 0.16 0.05

3 12 -14 0.19 0.05

-3 13 -14 -0.03 0.03

3 13 -14 0.02 0.03

3 0 -15 0.27 0.21

3 0 15 0.36 0.24

3 1 -15 1.60 0.30

3 -1 -15 1.17 0.26

3 -1 15 1.34 0.28

3 1 15 1.26 0.27

-3 -1 -15 1.65 0.32

3 -2 15 3.86 0.45

-3 -2 -15 4.23 0.47

3 -2 -15 4.46 0.50

3 2 15 4.06 0.46

3 2 -15 4.08 0.46

3 2 15 4.97 0.25

-3 2 15 4.83 0.25

3 3 -15 2.37 0.36

3 -3 15 2.34 0.36

-3 -3 -15 2.23 0.35

3 -3 -15 2.25 0.36

3 3 -15 3.09 0.18

3 3 15 2.97 0.20

-3 3 15 3.01 0.21

3 -4 -15 5.22 0.55

-3 -4 -15 5.13 0.52

3 4 -15 4.94 0.51

3 4 -15 5.76 0.27

-3 4 -15 5.59 0.24

-3 4 15 5.86 0.30

3 4 15 5.70 0.29

-3 4 -15 5.45 0.27

3 5 -15 1.39 0.28

-3 5 -15 1.77 0.32

3 -5 -15 1.61 0.31

-3 5 -15 1.65 0.16

3 5 -15 2.04 0.17

-3 5 -15 1.87 0.15

-3 5 15 1.75 0.18

3 5 -15 1.83 0.16

-3 6 -15 4.97 0.25

-3 6 -15 4.58 0.27

3 6 -15 5.10 0.27

3 6 -15 4.48 0.26

-3 6 15 4.87 0.29

-3 7 -15 0.04 0.07

3 7 -15 0.05 0.08

-3 7 -15 -0.05 0.08

3 7 -15 0.04 0.06

3 8 -15 0.91 0.15

-3 8 -15 0.78 0.13

3 8 -15 0.45 0.10

-3 8 -15 0.65 0.12

3 9 -15 1.15 0.15

-3 9 -15 1.33 0.15

-3 9 -15 0.91 0.12

3 10 -15 1.09 0.14

-3 10 -15 0.65 0.09

3 11 -15 0.01 0.05

-3 11 -15 -0.01 0.05

3 11 -15 0.13 0.07

-3 12 -15 0.07 0.04

3 12 -15 0.05 0.04

-3 13 -15 0.07 0.03

3 13 -15 0.05 0.03

3 0 16 0.11 0.12

-3 0 -16 0.05 0.13

3 0 -16 -0.06 0.14

3 0 16 0.07 0.04

3 1 -16 0.76 0.23

3 -1 -16 0.64 0.22

3 -1 16 0.49 0.16

-3 -1 -16 0.81 0.24

3 1 16 0.60 0.20

3 1 16 0.92 0.12

3 2 -16 3.98 0.47

-3 -2 -16 4.21 0.49

3 -2 -16 3.88 0.48

3 2 -16 4.47 0.21

3 2 16 4.46 0.25

-3 2 16 4.87 0.26

3 3 -16 4.13 0.50

-3 -3 -16 4.73 0.53

-3 3 -16 4.31 0.50

3 -3 -16 4.82 0.54

3 3 -16 4.72 0.23

3 3 16 5.21 0.28

-3 3 16 5.34 0.29

-3 4 -16 3.24 0.44

-3 -4 -16 3.56 0.46

3 4 -16 3.01 0.43

3 -4 -16 3.61 0.47

3 4 -16 4.08 0.23

-3 4 16 4.08 0.26

-3 4 -16 4.29 0.25

3 4 16 4.19 0.26

3 5 -16 -0.07 0.11

-3 5 -16 0.23 0.14

-3 5 -16 0.21 0.06

3 5 -16 0.29 0.09

3 5 -16 0.30 0.08

-3 5 16 0.24 0.10

-3 5 -16 0.25 0.08

3 6 -16 1.55 0.16

-3 6 -16 1.44 0.16

-3 6 -16 1.64 0.15

3 6 -16 1.57 0.16

-3 6 16 1.61 0.18

-3 7 -16 1.38 0.16

3 7 -16 1.29 0.15

-3 7 -16 1.37 0.15

3 7 -16 1.46 0.16

-3 8 -16 0.99 0.13

3 8 -16 1.17 0.15

3 8 -16 0.81 0.11

-3 8 -16 0.88 0.14

-3 9 -16 0.13 0.07

-3 9 -16 0.02 0.06

3 9 -16 0.20 0.10

-3 10 -16 0.29 0.07

3 10 -16 0.60 0.10

-3 11 -16 0.01 0.05

3 11 -16 0.05 0.05

3 12 -16 0.03 0.03

-3 12 -16 0.02 0.03

-3 0 -17 0.10 0.17

3 0 -17 -0.06 0.15

3 0 17 0.01 0.05

3 1 -17 3.21 0.46

3 -1 -17 2.60 0.41

-3 -1 -17 2.62 0.41

-3 1 -17 2.55 0.40

-3 1 17 2.69 0.20

3 -1 17 2.83 0.18

3 1 17 2.55 0.19

3 2 -17 1.28 0.28

3 -2 -17 1.29 0.32

-3 2 -17 1.38 0.30

-3 -2 -17 1.38 0.32

3 2 -17 1.57 0.13

-3 2 17 1.99 0.18

3 2 17 1.78 0.17

3 3 -17 0.25 0.17

3 -3 -17 0.05 0.13

-3 3 -17 0.18 0.15

-3 -3 -17 0.25 0.15

3 3 -17 0.34 0.07

-3 3 17 0.43 0.10

-3 3 -17 0.42 0.10

3 3 17 0.43 0.10

-3 4 -17 2.08 0.37

3 4 -17 1.54 0.32

-3 -4 -17 1.81 0.35

3 -4 -17 1.47 0.31

3 4 -17 2.19 0.17

-3 4 17 2.14 0.20

3 4 17 2.11 0.19

-3 4 -17 2.19 0.19

3 5 -17 0.21 0.08

-3 5 -17 0.09 0.06

3 5 -17 0.20 0.08

-3 5 17 0.12 0.09

-3 5 -17 0.09 0.08

-3 6 -17 0.12 0.07

3 6 -17 0.15 0.08

-3 6 -17 0.16 0.10

3 6 -17 0.22 0.09

3 7 -17 0.35 0.10

-3 7 -17 0.39 0.09

-3 7 -17 0.40 0.10

3 7 -17 0.43 0.10

3 8 -17 2.63 0.19

-3 8 -17 2.73 0.18

3 8 -17 3.18 0.21

-3 8 -17 2.75 0.20

-3 9 -17 0.39 0.09

3 9 -17 0.34 0.10

-3 9 -17 0.25 0.07

3 9 -17 0.29 0.07

-3 10 -17 0.35 0.08

3 10 -17 0.20 0.07

-3 10 -17 0.21 0.06

3 10 -17 0.35 0.09

-3 11 -17 -0.03 0.04

3 11 -17 -0.01 0.04

-3 12 -17 0.07 0.03

3 12 -17 0.09 0.03

3 0 -18 -0.09 0.16

-3 0 -18 -0.22 0.16

3 0 18 0.04 0.06

3 1 -18 2.16 0.40

-3 -1 -18 2.58 0.43

-3 1 -18 2.48 0.42

3 -1 -18 2.35 0.41

3 1 18 2.47 0.19

-3 1 18 2.69 0.21

3 -1 18 2.50 0.18

-3 2 -18 2.49 0.42

3 2 -18 2.12 0.40

-3 -2 -18 2.27 0.39

3 -2 -18 2.49 0.43

-3 2 18 2.74 0.22

3 2 18 2.67 0.21

-3 -3 -18 0.76 0.25

3 3 -18 0.64 0.24

3 -3 -18 1.12 0.29

-3 3 -18 0.69 0.24

3 3 -18 0.99 0.12

-3 3 -18 1.05 0.14

-3 3 18 0.89 0.14

3 3 18 0.94 0.13

3 4 -18 0.40 0.09

3 4 18 0.43 0.11

-3 4 18 0.41 0.12

-3 4 -18 0.43 0.11

3 5 -18 0.24 0.08

-3 5 -18 0.27 0.07

-3 5 -18 0.33 0.10

3 5 -18 0.16 0.08

-3 5 18 0.45 0.13

-3 6 -18 1.49 0.13

3 6 -18 1.49 0.15

3 6 -18 1.48 0.16

-3 6 -18 1.52 0.17

3 7 -18 0.53 0.11

-3 7 -18 0.52 0.09

-3 7 -18 0.48 0.11

3 7 -18 0.59 0.11

-3 8 -18 0.19 0.07

3 8 -18 0.46 0.11

-3 8 -18 0.33 0.09

3 8 -18 0.30 0.08

-3 9 -18 0.16 0.06

3 9 -18 0.22 0.07

3 9 -18 0.20 0.06

3 10 -18 0.13 0.06

-3 10 -18 0.11 0.06

3 10 -18 0.17 0.06

-3 10 -18 -0.02 0.04

-3 11 -18 0.13 0.04

3 11 -18 0.12 0.04

-3 0 -19 -0.07 0.14

3 0 -19 0.04 0.16

-3 0 19 -0.18 0.09

3 0 19 -0.09 0.07

-3 -1 -19 -0.04 0.13

3 1 -19 -0.03 0.14

3 -1 -19 0.26 0.17

-3 1 19 -0.01 0.08

3 1 19 0.09 0.07

3 -1 19 0.00 0.06

3 2 19 3.21 0.23

-3 2 19 3.67 0.26

3 3 -19 0.45 0.09

3 3 19 0.38 0.11

-3 3 -19 0.49 0.12

-3 3 19 0.46 0.11

3 4 -19 3.76 0.22

-3 4 -19 3.97 0.25

-3 4 19 4.25 0.28

3 4 -19 3.76 0.24

3 5 -19 0.24 0.08

-3 5 -19 0.36 0.11

-3 5 19 0.43 0.13

3 5 -19 0.17 0.09

-3 6 -19 0.28 0.07

3 6 -19 0.29 0.09

-3 6 -19 0.31 0.10

3 6 -19 0.38 0.10

-3 7 -19 0.08 0.06

3 7 -19 0.22 0.08

-3 7 -19 0.33 0.10

3 7 -19 0.16 0.07

-3 8 -19 0.59 0.09

3 8 -19 0.86 0.11

-3 8 -19 0.70 0.10

3 8 -19 0.57 0.09

-3 9 -19 0.07 0.05

3 9 -19 0.28 0.07

-3 10 -19 0.20 0.06

3 10 -19 0.15 0.05

-3 10 -19 0.06 0.04

3 11 -19 -0.02 0.03

-3 11 -19 0.05 0.03

3 0 20 0.09 0.07

-3 0 20 0.08 0.09

3 1 20 0.09 0.07

-3 1 20 0.15 0.08

-3 -1 20 0.22 0.08

3 -1 20 0.12 0.06

3 2 20 0.11 0.07

-3 -2 20 0.21 0.08

-3 2 20 0.11 0.09

-3 2 -20 0.26 0.10

3 3 20 1.04 0.15

-3 3 -20 1.04 0.15

-3 3 20 1.26 0.17

3 4 -20 1.49 0.14

-3 4 -20 1.93 0.19

3 4 -20 1.57 0.17

-3 4 20 1.85 0.20

3 5 -20 0.74 0.11

3 5 -20 0.63 0.11

-3 5 20 1.06 0.16

-3 5 -20 0.71 0.12

3 6 -20 0.76 0.11

3 6 -20 0.65 0.11

-3 6 -20 0.72 0.12

-3 7 -20 0.11 0.05

3 7 -20 0.11 0.07

-3 7 -20 0.27 0.09

3 7 -20 0.17 0.07

3 8 -20 0.11 0.06

-3 8 -20 0.10 0.06

3 8 -20 0.11 0.05

3 9 -20 0.18 0.06

-3 9 -20 0.03 0.04

3 10 -20 0.54 0.06

-3 10 -20 0.49 0.06

3 0 21 -0.02 0.06

-3 0 21 -0.03 0.08

-3 1 -21 0.23 0.09

-3 1 21 -0.02 0.08

3 1 21 0.17 0.08

-3 -1 21 0.08 0.07

3 2 21 0.31 0.10

-3 2 -21 0.42 0.11

-3 -2 21 0.28 0.09

-3 2 21 0.30 0.10

-3 -3 21 0.49 0.09

-3 3 -21 0.59 0.12

-3 3 21 0.84 0.15

-3 4 -21 0.67 0.12

3 4 -21 0.52 0.11

3 5 -21 1.03 0.11

-3 5 -21 1.11 0.14

3 5 -21 1.04 0.13

3 6 -21 0.67 0.09

3 6 -21 0.61 0.11

-3 6 -21 0.47 0.10

3 7 -21 0.02 0.05

3 7 -21 0.02 0.06

-3 7 -21 0.04 0.06

3 8 -21 0.13 0.05

-3 8 -21 0.19 0.06

3 8 -21 0.21 0.06

-3 9 -21 0.09 0.04

-3 10 -21 0.05 0.03

3 10 -21 0.06 0.03

-3 0 22 0.04 0.07

3 0 22 -0.05 0.06

-3 1 22 0.00 0.07

-3 1 -22 0.07 0.08

-3 -1 22 -0.03 0.07

3 1 22 -0.02 0.07

-3 2 22 0.87 0.14

-3 -2 22 0.86 0.11

-3 2 -22 0.97 0.13

-3 -3 22 0.49 0.09

-3 3 22 0.48 0.12

3 3 -22 0.40 0.10

-3 3 -22 0.40 0.11

3 4 -22 0.60 0.11

-3 4 22 0.74 0.14

-3 4 -22 0.50 0.11

3 5 -22 0.13 0.08

-3 5 -22 0.13 0.08

3 6 -22 0.32 0.07

-3 6 -22 0.42 0.10

3 6 -22 0.28 0.08

3 7 -22 0.04 0.04

3 7 -22 0.06 0.05

-3 7 -22 0.06 0.06

-3 8 -22 0.08 0.05

3 8 -22 0.05 0.04

3 8 -22 0.12 0.04

3 9 -22 0.14 0.04

-3 9 -22 0.16 0.04

-3 0 23 0.05 0.07

-3 0 -23 0.05 0.07

3 0 23 -0.01 0.05

-3 1 23 0.22 0.08

-3 1 -23 0.25 0.08

-3 -1 -23 0.24 0.08

-3 -1 23 0.07 0.06

-3 -2 23 0.47 0.08

-3 2 23 0.58 0.12

-3 -2 -23 0.59 0.09

-3 2 -23 0.48 0.10

3 2 -23 0.46 0.10

-3 3 23 0.07 0.08

3 3 -23 -0.09 0.09

-3 3 -23 0.18 0.08

-3 4 -23 1.04 0.13

3 4 -23 1.15 0.13

3 5 -23 0.07 0.07

-3 5 -23 0.08 0.07

3 6 -23 0.33 0.08

-3 6 -23 0.44 0.09

3 7 -23 0.03 0.04

-3 7 -23 0.03 0.04

-3 8 -23 0.50 0.06

-3 0 24 0.09 0.07

-3 0 -24 0.06 0.06

-3 -1 -24 0.14 0.06

-3 -1 24 0.10 0.06

-3 1 24 0.09 0.07

3 1 -24 0.14 0.07

-3 1 -24 0.11 0.06

-3 2 24 0.30 0.09

-3 2 -24 0.28 0.08

-3 -2 24 0.27 0.06

3 2 -24 0.36 0.09

-3 -2 -24 0.35 0.07

-3 3 -24 0.10 0.06

3 3 -24 0.10 0.06

-3 4 -24 0.63 0.10

3 4 -24 0.51 0.09

-3 5 -24 0.20 0.07

3 5 -24 0.22 0.07

3 6 -24 0.44 0.07

-3 6 -24 0.58 0.08

-3 7 -24 0.03 0.04

3 7 -24 0.09 0.04

-3 0 -25 0.02 0.05

3 0 -25 0.02 0.05

-3 0 25 0.02 0.05

-3 1 -25 -0.01 0.05

3 -1 -25 0.05 0.06

-3 -1 -25 0.06 0.05

-3 1 25 0.07 0.06

-3 -1 25 0.06 0.05

3 1 -25 0.11 0.06

3 2 -25 0.11 0.07

-3 -2 -25 0.15 0.05

-3 2 -25 0.09 0.06

-3 3 -25 0.05 0.05

3 3 -25 -0.03 0.06

-3 4 -25 0.39 0.07

3 -4 -25 0.38 0.06

3 4 -25 0.45 0.08

3 5 -25 0.20 0.06

-3 5 -25 0.21 0.06

3 6 -25 0.03 0.03

-3 6 -25 0.11 0.04

3 0 -26 -0.02 0.05

-3 0 -26 -0.02 0.04

3 -1 -26 0.03 0.04

-3 -1 -26 -0.04 0.04

3 1 -26 -0.09 0.06

-3 1 -26 -0.02 0.04

3 2 -26 0.06 0.05

-3 2 -26 0.17 0.05

3 -2 -26 0.10 0.04

3 3 -26 0.13 0.05

3 -3 -26 0.15 0.04

-3 3 -26 0.16 0.05

3 4 -26 0.30 0.06

-3 4 -26 0.30 0.06

-3 5 -26 0.09 0.04

3 5 -26 0.09 0.03

-3 0 -27 -0.02 0.03

3 0 -27 0.03 0.04

-3 1 -27 0.07 0.04

3 1 -27 0.11 0.04

3 -1 -27 0.08 0.04

-3 2 -27 0.12 0.04

3 2 -27 0.09 0.04

-3 3 -27 0.20 0.04

3 3 -27 0.21 0.05

4 0 0 1206.23 6.68

4 -1 0 40.33 0.60

4 1 0 39.76 0.45

-4 1 0 39.76 0.34

4 1 0 40.79 0.38

4 2 0 0.03 0.03

-4 2 0 0.11 0.03

4 -2 0 0.01 0.03

4 2 0 0.08 0.04

4 2 0 0.07 0.04

4 -2 0 -0.01 0.05

4 -3 0 35.93 0.52

4 -3 0 37.23 0.64

4 3 0 37.04 0.60

-4 3 0 37.02 0.57

4 4 0 1.31 0.09

4 4 0 1.21 0.13

-4 4 0 1.27 0.13

4 4 0 1.26 0.14

4 5 0 10.41 0.41

-4 5 0 10.29 0.40

4 5 0 10.28 0.43

4 6 0 1.74 0.21

-4 6 0 1.73 0.22

-4 6 0 1.59 0.18

4 6 0 1.78 0.20

-4 7 0 7.34 0.45

4 7 0 7.23 0.45

-4 7 0 7.18 0.41

4 7 0 6.92 0.41

4 8 0 34.55 1.06

-4 8 0 32.68 0.97

4 8 0 35.38 1.03

-4 8 0 34.91 1.07

-4 9 0 1.46 0.23

4 9 0 1.69 0.27

-4 9 0 1.58 0.13

4 9 0 1.59 0.14

4 9 0 1.52 0.12

-4 9 0 1.60 0.27

-4 9 0 1.64 0.13

4 9 0 1.66 0.25

4 9 0 1.61 0.13

-4 9 0 1.68 0.13

4 10 0 2.43 0.33

-4 10 0 2.52 0.32

-4 10 0 2.54 0.17

-4 10 0 2.49 0.16

4 10 0 2.31 0.32

4 10 0 2.69 0.19

-4 10 0 2.30 0.33

4 10 0 2.51 0.15

4 10 0 2.56 0.17

-4 10 0 2.65 0.18

-4 11 0 3.21 0.19

4 11 0 3.40 0.21

4 11 0 3.39 0.18

-4 11 0 3.92 0.21

4 11 0 3.54 0.21

-4 11 0 3.62 0.21

-4 12 0 5.08 0.23

4 12 0 5.86 0.26

4 12 0 4.55 0.20

4 12 0 5.13 0.25

-4 12 0 5.32 0.25

4 13 0 0.12 0.07

-4 13 0 0.05 0.05

4 13 0 -0.02 0.06

-4 13 0 0.06 0.06

-4 14 0 0.42 0.06

-4 14 0 0.41 0.07

4 14 0 0.36 0.07

4 0 -1 55.56 0.66

4 0 1 59.65 0.70

4 -1 1 0.70 0.08

4 1 -1 0.60 0.07

4 1 1 0.69 0.06

4 1 -1 0.59 0.05

4 1 1 0.68 0.08

-4 1 1 0.63 0.05

4 -1 -1 0.58 0.08

4 2 -1 13.71 0.28

-4 2 1 13.72 0.26

4 -2 -1 13.94 0.36

4 2 1 14.24 0.32

4 -2 1 14.14 0.33

4 2 -1 14.28 0.33

4 2 1 13.91 0.32

4 2 1 13.68 0.29

4 3 -1 0.51 0.09

-4 3 -1 0.51 0.08

-4 3 1 0.42 0.06

4 3 1 0.51 0.09

4 -3 -1 0.50 0.09

4 3 1 0.51 0.08

4 3 1 0.46 0.06

-4 3 1 0.45 0.08

4 3 -1 0.45 0.05

-4 4 1 7.50 0.33

-4 4 1 7.19 0.28

4 4 -1 7.87 0.32

4 -4 -1 7.61 0.21

-4 4 -1 7.51 0.31

4 -4 -1 7.75 0.30

4 4 1 7.09 0.32

4 4 -1 8.27 0.34

4 4 1 7.42 0.30

4 5 1 1.11 0.16

-4 5 1 0.99 0.13

4 5 -1 1.13 0.17

-4 5 1 0.97 0.14

4 5 -1 0.91 0.14

4 5 1 0.82 0.13

-4 5 -1 0.97 0.14

-4 6 1 0.71 0.13

-4 6 1 0.68 0.15

-4 6 -1 0.71 0.15

4 6 -1 0.71 0.15

4 6 1 0.58 0.15

4 6 -1 0.61 0.14

4 6 1 0.66 0.13

4 7 -1 1.51 0.22

-4 7 1 1.54 0.20

-4 7 -1 1.63 0.21

-4 7 -1 1.25 0.20

4 7 1 1.65 0.22

4 7 1 1.63 0.21

-4 7 1 2.01 0.24

4 7 -1 1.66 0.22

-4 8 -1 0.12 0.09

-4 8 -1 0.06 0.14

4 8 1 0.23 0.11

-4 8 1 0.24 0.11

4 8 -1 0.22 0.12

4 8 1 0.24 0.12

-4 8 1 0.18 0.11

4 8 -1 0.16 0.10

-4 9 1 0.78 0.09

4 9 1 0.66 0.08

4 9 -1 0.64 0.08

4 9 1 0.70 0.09

-4 9 -1 0.63 0.09

-4 9 1 0.81 0.10

-4 9 -1 0.73 0.09

4 9 -1 0.76 0.10

4 9 -1 0.84 0.19

4 9 1 0.66 0.17

4 9 -1 0.79 0.20

4 9 1 0.55 0.16

-4 9 1 0.84 0.18

-4 9 1 0.80 0.19

-4 9 -1 0.72 0.20

-4 9 -1 0.58 0.16

4 9 -1 0.67 0.09

4 9 1 0.69 0.09

4 9 -1 0.81 0.10

-4 9 1 0.78 0.09

-4 9 -1 0.72 0.10

4 10 1 0.55 0.17

4 10 -1 0.41 0.15

-4 10 -1 0.63 0.21

-4 10 1 0.60 0.17

-4 10 -1 0.72 0.18

4 10 -1 0.64 0.18

4 10 1 0.71 0.09

-4 10 1 0.72 0.10

-4 10 1 1.01 0.12

4 10 1 0.79 0.11

4 10 -1 0.64 0.10

4 10 -1 0.65 0.09

4 10 1 0.81 0.11

-4 10 -1 0.78 0.10

-4 10 -1 0.80 0.11

-4 10 1 0.87 0.11

-4 10 -1 0.90 0.12

4 10 -1 0.94 0.12

4 11 1 1.24 0.14

-4 11 1 1.34 0.13

4 11 -1 1.19 0.11

-4 11 1 0.99 0.12

-4 11 -1 1.09 0.12

4 11 -1 1.32 0.14

4 11 1 1.20 0.12

-4 11 1 1.18 0.13

-4 11 -1 1.16 0.13

4 11 1 1.04 0.12

4 11 -1 1.07 0.12

4 12 1 0.06 0.06

4 12 -1 0.14 0.09

4 12 -1 0.12 0.06

4 12 -1 -0.02 0.07

-4 12 1 0.00 0.06

-4 12 -1 -0.01 0.06

-4 12 -1 -0.01 0.07

4 12 1 0.12 0.08

-4 12 1 0.11 0.08

4 13 1 0.27 0.08

-4 13 1 0.08 0.05

-4 13 -1 0.09 0.05

4 13 -1 0.22 0.07

4 13 -1 0.16 0.07

4 13 1 0.08 0.06

-4 13 1 0.14 0.06

-4 13 -1 0.21 0.07

-4 14 1 0.15 0.05

-4 14 -1 0.10 0.04

4 14 1 0.09 0.05

4 14 -1 0.06 0.05

-4 14 1 0.13 0.05

-4 14 -1 0.08 0.05

4 0 -2 481.37 3.11

4 0 2 479.32 3.11

-4 1 2 25.12 0.31

4 -1 -2 26.90 0.50

4 1 2 24.94 0.47

4 1 -2 26.96 0.47

4 -1 2 24.61 0.47

-4 2 2 453.71 2.79

4 2 2 450.93 2.78

4 2 2 450.03 2.93

4 2 -2 463.11 2.88

4 2 2 464.25 2.87

4 3 2 52.64 0.72

-4 3 2 51.16 0.66

4 -3 -2 48.74 0.77

-4 3 2 53.53 0.76

4 3 -2 50.11 0.62

4 3 -2 54.73 0.82

4 3 2 53.46 0.76

4 4 2 121.12 1.32

4 4 2 121.61 1.39

4 4 -2 121.77 0.93

-4 4 2 123.94 1.39

4 4 -2 123.08 1.48

-4 4 -2 119.95 1.39

-4 4 2 119.62 1.26

4 -4 -2 120.11 1.34

-4 5 2 7.97 0.37

4 5 2 7.52 0.35

-4 5 2 7.70 0.33

4 -5 -2 7.53 0.31

4 5 2 7.73 0.37

-4 5 -2 7.64 0.37

4 5 -2 7.10 0.36

4 5 -2 7.81 0.40

4 -5 2 7.31 0.24

-4 6 2 76.91 1.32

4 6 2 77.53 1.26

4 6 2 78.95 1.33

-4 6 2 75.35 1.21

4 6 -2 81.92 1.44

4 6 -2 79.49 1.35

-4 6 -2 78.86 1.34

-4 7 2 0.37 0.13

-4 7 -2 0.36 0.11

4 7 2 0.44 0.12

4 7 -2 0.46 0.13

4 7 2 0.47 0.13

-4 7 2 0.38 0.11

-4 7 -2 0.55 0.15

4 7 -2 0.58 0.14

-4 8 2 13.52 0.64

4 8 -2 13.55 0.65

4 8 2 13.77 0.62

4 8 -2 12.85 0.67

-4 8 2 13.18 0.59

-4 8 -2 13.16 0.64

4 8 2 13.18 0.63

-4 8 -2 13.03 0.68

4 9 -2 0.56 0.07

-4 9 2 0.63 0.09

4 9 -2 0.52 0.09

-4 9 -2 0.47 0.08

4 9 -2 0.60 0.09

4 9 2 0.55 0.09

4 9 2 0.46 0.08

4 9 2 0.49 0.08

-4 9 2 0.66 0.09

-4 9 -2 0.47 0.08

-4 9 -2 0.59 0.09

-4 9 2 0.58 0.08

-4 9 -2 0.64 0.18

-4 9 2 0.64 0.17

4 9 2 0.56 0.16

4 9 -2 0.46 0.16

-4 9 -2 0.46 0.15

4 9 -2 0.67 0.18

4 9 -2 0.54 0.09

-4 10 -2 5.38 0.47

4 10 -2 5.98 0.52

-4 10 -2 5.97 0.53

4 10 2 5.94 0.27

4 10 -2 5.82 0.49

4 10 -2 5.20 0.21

-4 10 2 5.91 0.26

-4 10 2 5.10 0.46

-4 10 -2 5.65 0.24

4 10 2 6.12 0.48

-4 10 -2 5.23 0.24

4 10 2 5.55 0.23

4 10 -2 5.82 0.26

-4 10 2 5.34 0.24

-4 10 -2 5.83 0.26

4 10 -2 5.65 0.25

4 10 2 5.64 0.24

-4 10 2 6.02 0.25

4 11 2 1.73 0.16

-4 11 -2 1.41 0.13

4 11 -2 1.68 0.15

-4 11 2 1.54 0.14

4 11 -2 1.54 0.12

-4 11 2 1.81 0.15

4 11 2 1.67 0.13

4 11 -2 1.82 0.16

-4 11 2 1.74 0.15

4 11 2 1.76 0.15

-4 11 -2 1.84 0.16

4 12 2 5.93 0.27

4 12 -2 5.73 0.25

-4 12 2 5.75 0.26

4 12 2 5.33 0.22

4 12 -2 5.16 0.20

-4 12 -2 5.14 0.23

-4 12 -2 5.81 0.26

-4 12 2 6.10 0.26

4 12 2 5.83 0.26

4 12 -2 5.63 0.26

4 13 -2 0.21 0.07

-4 13 2 0.03 0.05

-4 13 -2 -0.01 0.06

4 13 -2 -0.03 0.06

-4 13 -2 0.08 0.05

4 13 2 0.05 0.07

-4 13 2 0.04 0.06

4 13 2 -0.02 0.06

-4 14 -2 0.28 0.05

-4 14 2 0.36 0.06

-4 14 -2 0.25 0.06

4 14 -2 0.31 0.07

-4 15 -2 0.03 0.03

4 15 -2 0.00 0.03

4 0 -3 9.19 0.31

4 -1 -3 2.56 0.17

4 1 -3 2.31 0.16

-4 2 3 4.41 0.17

4 -2 -3 4.76 0.24

4 3 3 9.82 0.30

-4 3 3 9.94 0.31

4 3 -3 9.57 0.30

4 4 3 0.95 0.12

-4 4 3 1.04 0.13

4 4 3 1.06 0.14

4 -4 -3 0.87 0.12

4 4 -3 0.98 0.14

-4 4 -3 1.05 0.13

-4 -4 -3 1.02 0.08

4 4 -3 0.98 0.09

4 5 3 0.21 0.09

-4 5 -3 0.32 0.09

4 5 3 0.35 0.11

4 -5 3 0.29 0.06

-4 5 3 0.34 0.11

4 5 -3 0.28 0.09

4 -5 -3 0.30 0.08

-4 5 3 0.36 0.09

4 6 -3 0.55 0.16

-4 6 -3 0.70 0.15

4 6 -3 0.30 0.11

4 6 3 0.53 0.14

4 6 3 0.60 0.13

-4 6 3 0.57 0.13

-4 7 3 1.36 0.20

4 7 3 1.27 0.18

4 7 -3 1.41 0.22

-4 7 -3 1.31 0.20

4 7 -3 1.38 0.21

-4 8 -3 0.90 0.20

-4 8 -3 1.00 0.18

4 8 -3 0.89 0.19

-4 8 3 1.17 0.20

4 8 -3 0.88 0.19

4 8 3 1.08 0.19

-4 9 3 0.40 0.08

4 9 -3 0.24 0.06

-4 9 3 0.29 0.07

-4 9 -3 0.30 0.07

-4 9 3 0.42 0.08

4 9 -3 0.34 0.07

4 9 3 0.29 0.08

4 9 3 0.30 0.07

4 9 -3 0.29 0.06

-4 9 -3 0.43 0.07

4 9 3 0.34 0.08

-4 9 -3 0.26 0.07

4 9 -3 0.27 0.07

-4 9 -3 0.26 0.13

-4 9 3 0.34 0.15

-4 9 -3 0.02 0.09

4 9 -3 0.16 0.12

4 9 -3 0.45 0.16

4 9 3 0.34 0.13

-4 10 -3 0.10 0.06

-4 10 3 0.18 0.08

-4 10 3 0.08 0.07

4 10 3 0.11 0.07

4 10 3 0.00 0.06

-4 10 -3 0.12 0.08

-4 10 3 0.06 0.06

4 10 -3 -0.03 0.05

4 10 -3 0.11 0.07

4 10 3 0.06 0.06

4 10 -3 0.07 0.07

4 10 -3 0.22 0.15

4 10 -3 0.13 0.14

-4 10 -3 0.14 0.13

4 10 3 0.12 0.12

-4 10 3 -0.06 0.11

-4 10 -3 0.07 0.14

4 10 -3 0.14 0.08

4 11 -3 0.49 0.09

-4 11 3 0.41 0.08

4 11 3 0.69 0.11

4 11 3 0.65 0.09

4 11 -3 0.44 0.07

-4 11 -3 0.50 0.10

-4 11 3 0.73 0.11

-4 11 -3 0.32 0.07

4 11 -3 0.50 0.10

-4 11 3 0.60 0.10

4 11 3 0.52 0.10

4 12 3 0.71 0.10

-4 12 3 0.65 0.10

-4 12 -3 0.61 0.09

4 12 -3 0.93 0.11

4 12 -3 0.62 0.10

-4 12 -3 0.64 0.10

-4 12 3 0.76 0.11

4 12 3 0.77 0.11

4 13 3 0.23 0.08

-4 13 -3 0.15 0.05

-4 13 -3 0.09 0.06

4 13 -3 0.10 0.06

-4 13 3 0.12 0.06

4 13 3 0.06 0.06

-4 13 3 0.12 0.07

-4 14 3 0.15 0.05

-4 14 -3 0.09 0.03

4 14 -3 0.06 0.05

-4 14 -3 0.05 0.05

4 15 -3 0.04 0.03

-4 15 -3 0.04 0.03

4 0 -4 45.67 0.79

4 1 -4 15.82 0.44

4 -1 -4 16.36 0.46

4 -2 -4 156.72 1.64

4 2 -4 157.83 1.54

-4 2 4 153.93 1.26

4 -3 -4 86.38 1.18

4 3 -4 86.81 1.09

-4 3 4 89.16 1.00

4 4 -4 83.44 1.03

4 4 -4 87.31 1.27

-4 4 4 85.01 1.09

4 4 4 86.13 1.09

4 5 -4 3.43 0.17

-4 5 -4 3.72 0.28

4 -5 4 3.28 0.22

4 -5 -4 3.62 0.24

4 5 -4 4.02 0.30

4 5 4 3.13 0.23

-4 5 4 3.16 0.23

-4 6 -4 1.42 0.19

4 6 -4 1.40 0.21

4 6 4 1.91 0.20

-4 6 4 1.84 0.20

-4 7 -4 1.19 0.20

4 7 -4 1.33 0.22

4 7 -4 1.26 0.20

-4 7 4 1.41 0.20

4 7 4 1.40 0.20

-4 8 -4 4.96 0.41

4 8 -4 5.02 0.42

4 8 -4 4.73 0.43

-4 8 4 4.79 0.21

4 8 4 4.91 0.37

4 8 4 4.56 0.19

-4 8 4 4.29 0.36

-4 9 -4 1.59 0.14

-4 9 -4 1.66 0.13

4 9 -4 1.56 0.14

4 9 4 1.95 0.14

-4 9 4 1.88 0.26

4 9 4 1.94 0.26

-4 9 4 1.97 0.15

4 9 4 1.82 0.15

-4 9 4 2.06 0.14

4 9 -4 1.71 0.13

-4 9 -4 1.79 0.13

-4 9 -4 1.49 0.25

4 9 -4 1.59 0.27

-4 9 -4 1.61 0.28

4 9 -4 1.58 0.26

4 9 -4 1.60 0.15

4 9 4 1.99 0.14

-4 9 4 1.93 0.14

-4 10 4 1.55 0.14

4 10 4 1.49 0.14

4 10 4 1.53 0.13

-4 10 4 1.27 0.13

-4 10 -4 1.48 0.14

-4 10 4 1.63 0.26

4 10 -4 1.30 0.13

4 10 -4 1.31 0.14

-4 10 -4 1.40 0.13

4 10 -4 1.39 0.26

-4 10 -4 1.21 0.24

4 10 -4 1.32 0.27

-4 10 -4 0.99 0.25

4 10 -4 1.33 0.14

4 10 4 1.49 0.14

-4 10 4 1.60 0.14

4 11 4 1.47 0.15

-4 11 -4 1.24 0.12

-4 11 4 1.42 0.13

4 11 -4 1.35 0.13

-4 11 4 1.31 0.14

4 11 4 1.38 0.13

4 11 -4 1.51 0.15

-4 11 -4 1.47 0.15

-4 11 4 1.41 0.14

4 11 4 1.29 0.13

4 12 4 1.99 0.17

4 12 4 1.93 0.14

-4 12 -4 1.64 0.13

-4 12 4 2.14 0.17

4 12 -4 1.76 0.14

4 12 4 1.93 0.15

-4 12 -4 1.88 0.16

-4 12 4 1.89 0.15

4 12 -4 1.97 0.16

-4 13 -4 0.08 0.04

-4 13 -4 0.11 0.06

-4 13 4 0.03 0.05

4 13 -4 0.10 0.06

4 13 4 0.20 0.07

4 13 4 0.13 0.06

-4 13 4 0.16 0.06

4 14 -4 0.25 0.06

-4 14 -4 0.19 0.06

4 15 -4 0.02 0.02

-4 15 -4 0.07 0.03

4 0 -5 43.71 0.82

4 1 -5 1.33 0.15

4 -1 -5 1.25 0.15

4 -2 -5 25.64 0.65

4 2 -5 25.19 0.61

4 -3 -5 8.62 0.38

4 3 -5 9.02 0.35

-4 3 5 8.83 0.30

4 -4 -5 7.70 0.37

4 4 -5 8.47 0.34

4 -4 5 8.90 0.39

4 4 5 8.80 0.34

-4 4 5 8.40 0.33

4 5 5 2.65 0.17

4 -5 -5 2.60 0.22

4 5 -5 3.13 0.20

4 5 -5 3.24 0.28

4 -5 5 2.78 0.22

-4 5 5 2.84 0.22

-4 6 -5 7.45 0.44

4 6 -5 7.78 0.48

4 6 5 8.18 0.40

-4 6 5 7.75 0.40

-4 7 -5 0.07 0.09

4 7 -5 -0.05 0.11

-4 7 5 0.09 0.08

4 7 5 0.09 0.09

-4 8 5 0.54 0.08

4 8 5 0.70 0.10

-4 8 5 0.66 0.09

4 8 5 0.56 0.08

-4 8 -5 0.69 0.19

4 8 -5 0.67 0.20

-4 8 5 0.58 0.15

4 8 -5 0.81 0.19

4 8 5 0.61 0.14

4 8 -5 0.66 0.10

-4 8 -5 0.68 0.10

-4 8 5 0.62 0.08

4 8 5 0.56 0.08

-4 9 5 0.53 0.09

-4 9 5 0.61 0.09

4 9 5 0.52 0.08

-4 9 -5 0.36 0.07

4 9 -5 0.54 0.08

4 9 -5 0.42 0.08

4 9 5 0.49 0.09

-4 9 -5 0.52 0.08

-4 9 -5 0.41 0.15

-4 9 -5 0.24 0.12

4 9 -5 0.34 0.12

-4 9 5 0.43 0.15

4 9 -5 0.49 0.17

4 9 -5 0.39 0.08

-4 9 5 0.51 0.08

4 9 5 0.43 0.07

-4 10 -5 0.29 0.07

-4 10 -5 0.27 0.09

4 10 5 0.31 0.09

4 10 -5 0.32 0.09

-4 10 5 0.31 0.08

4 10 -5 0.46 0.08

-4 10 5 0.23 0.07

4 10 5 0.35 0.08

-4 10 -5 0.29 0.16

4 10 -5 0.41 0.18

4 10 -5 0.34 0.19

-4 10 5 0.34 0.15

-4 10 -5 0.34 0.16

4 10 -5 0.31 0.10

4 10 5 0.24 0.08

-4 10 5 0.31 0.08

4 11 -5 0.44 0.08

-4 11 -5 0.39 0.10

-4 11 5 0.48 0.09

4 11 5 0.50 0.09

-4 11 5 0.33 0.08

4 11 5 0.34 0.09

-4 11 -5 0.24 0.06

4 11 -5 0.43 0.10

-4 11 5 0.35 0.09

4 11 5 0.36 0.09

-4 12 5 0.27 0.08

4 12 5 0.38 0.10

4 12 -5 0.32 0.09

-4 12 -5 0.18 0.05

-4 12 -5 0.44 0.10

-4 12 5 0.15 0.07

4 12 5 0.15 0.07

-4 13 -5 0.04 0.06

4 13 5 0.18 0.07

4 13 -5 0.16 0.07

-4 13 5 0.13 0.06

4 14 -5 0.13 0.05

-4 14 -5 0.11 0.05

-4 15 -5 0.01 0.02

4 15 -5 -0.02 0.03

4 0 -6 4.31 0.27

4 -1 -6 18.83 0.58

4 1 -6 18.39 0.56

4 2 -6 115.83 1.48

4 -2 -6 116.16 1.56

4 -3 -6 1.90 0.20

4 3 -6 1.70 0.18

4 4 -6 46.92 0.91

4 -4 6 47.23 0.97

4 4 6 46.55 0.88

4 5 -6 3.82 0.25

4 -5 -6 3.57 0.27

4 5 6 3.82 0.23

4 5 -6 4.11 0.32

4 -5 6 3.70 0.28

-4 5 6 3.89 0.26

-4 6 -6 46.34 1.15

4 6 -6 49.30 1.22

-4 6 6 49.15 1.01

-4 7 -6 2.08 0.27

4 7 -6 2.47 0.31

-4 7 6 2.72 0.26

4 8 6 4.28 0.19

-4 8 6 4.47 0.20

4 8 6 4.47 0.22

-4 8 6 4.27 0.19

-4 8 6 4.44 0.36

4 8 -6 4.33 0.43

4 8 -6 4.24 0.39

-4 8 -6 3.82 0.39

4 8 -6 4.39 0.23

-4 8 -6 4.25 0.22

-4 8 6 4.50 0.20

4 8 6 4.30 0.19

-4 9 6 4.27 0.21

4 9 -6 4.18 0.22

-4 9 6 4.32 0.21

-4 9 -6 4.41 0.23

-4 9 -6 3.76 0.18

4 9 -6 4.03 0.19

4 9 6 4.16 0.22

4 9 6 4.46 0.20

-4 9 -6 3.68 0.41

4 9 -6 4.19 0.42

-4 9 -6 3.85 0.44

4 9 -6 3.80 0.42

-4 9 -6 3.60 0.21

4 9 -6 3.96 0.23

-4 9 -6 4.16 0.23

4 9 6 4.34 0.20

-4 9 6 4.62 0.21

4 10 -6 4.11 0.20

-4 10 -6 3.93 0.19

4 10 6 4.27 0.23

4 10 6 4.02 0.20

-4 10 6 4.12 0.21

4 10 -6 3.74 0.22

-4 10 6 3.82 0.20

4 10 -6 3.79 0.41

-4 10 -6 3.83 0.22

-4 10 -6 3.86 0.43

4 10 -6 3.62 0.43

-4 10 -6 4.13 0.46

4 10 -6 3.49 0.22

-4 10 6 4.04 0.21

4 10 6 3.92 0.20

4 11 -6 0.18 0.06

4 11 6 0.09 0.07

4 11 6 0.14 0.07

4 11 -6 0.08 0.07

-4 11 6 0.01 0.07

-4 11 -6 0.11 0.08

-4 11 -6 0.02 0.04

-4 11 6 0.24 0.08

4 11 -6 0.01 0.07

4 11 6 0.07 0.07

-4 11 6 0.06 0.07

-4 12 6 1.68 0.15

4 12 6 1.70 0.15

4 12 -6 1.93 0.16

-4 12 -6 1.92 0.16

-4 13 6 0.17 0.06

4 13 -6 0.10 0.06

-4 13 -6 0.10 0.06

4 13 6 0.11 0.06

4 14 -6 0.37 0.06

-4 14 -6 0.27 0.06

-4 15 -6 -0.01 0.02

4 15 -6 -0.02 0.02

4 0 -7 30.32 0.78

4 -1 -7 1.71 0.21

4 1 -7 1.80 0.20

4 -2 -7 31.76 0.84

4 2 -7 32.06 0.80

4 -3 7 4.33 0.32

4 3 7 4.45 0.29

4 -3 -7 4.88 0.33

4 3 -7 4.99 0.32

4 -4 -7 1.76 0.21

4 4 -7 1.82 0.20

4 4 7 1.81 0.19

4 -4 7 1.88 0.22

4 5 -7 0.56 0.11

4 5 7 0.52 0.11

4 -5 -7 0.44 0.12

4 -5 7 0.52 0.12

-4 6 -7 2.43 0.27

4 6 -7 2.61 0.30

-4 7 -7 0.79 0.19

4 7 -7 0.82 0.20

-4 8 7 0.23 0.06

4 8 7 0.12 0.05

-4 8 7 0.20 0.06

4 8 7 0.10 0.05

-4 8 -7 0.22 0.13

4 8 -7 0.03 0.10

-4 8 -7 0.08 0.07

4 8 -7 0.22 0.08

-4 8 -7 0.12 0.06

-4 8 7 0.16 0.05

4 8 7 0.12 0.05

-4 9 7 1.50 0.13

4 9 7 1.41 0.13

4 9 -7 1.43 0.11

-4 9 -7 1.39 0.13

-4 9 7 1.47 0.13

4 9 -7 1.38 0.13

4 9 7 1.63 0.13

4 9 -7 1.46 0.29

4 9 -7 1.40 0.26

-4 9 -7 1.38 0.27

-4 9 -7 1.49 0.14

4 9 -7 1.39 0.15

-4 9 -7 1.59 0.16

4 9 7 1.44 0.12

-4 9 7 1.49 0.12

-4 10 7 0.25 0.07

4 10 7 0.19 0.07

4 10 7 0.45 0.09

4 10 -7 0.36 0.07

-4 10 -7 0.17 0.08

-4 10 7 0.14 0.07

4 10 -7 0.15 0.07

4 10 -7 0.21 0.14

4 10 -7 0.35 0.10

-4 10 7 0.29 0.08

4 10 7 0.26 0.08

-4 11 7 0.17 0.07

4 11 -7 0.06 0.08

4 11 7 0.00 0.06

-4 11 7 0.10 0.07

4 11 7 0.17 0.07

-4 11 -7 0.09 0.08

4 11 -7 0.21 0.09

-4 12 -7 0.30 0.09

-4 12 7 0.14 0.08

4 12 7 0.26 0.08

4 12 -7 0.30 0.09

4 13 7 0.09 0.05

4 13 -7 -0.12 0.06

-4 13 7 0.10 0.05

-4 13 -7 -0.07 0.06

4 14 -7 0.14 0.05

-4 14 -7 0.00 0.04

4 0 -8 9.87 0.48

4 -1 -8 55.61 1.16

4 1 8 51.62 1.09

4 -1 8 53.27 1.12

4 1 -8 54.77 1.14

4 -2 -8 321.10 3.18

4 2 -8 318.08 3.10

4 -2 8 323.95 3.17

4 2 8 319.45 3.06

4 3 -8 8.93 0.46

4 -3 8 10.76 0.51

4 -3 -8 9.57 0.50

4 3 8 10.54 0.48

4 -4 -8 5.77 0.39

4 -4 8 5.95 0.40

4 4 8 5.90 0.36

4 4 -8 5.77 0.37

4 -5 -8 3.99 0.33

4 5 8 4.07 0.29

4 5 -8 4.63 0.33

4 -5 8 3.97 0.33

4 6 -8 3.28 0.34

-4 7 -8 2.52 0.31

4 7 -8 2.79 0.34

4 8 8 10.57 0.30

-4 8 8 10.29 0.30

4 8 8 10.33 0.34

-4 8 8 10.65 0.31

4 8 -8 11.51 0.71

-4 8 -8 9.92 0.65

-4 8 -8 11.27 0.37

-4 8 -8 10.08 0.35

4 8 -8 11.48 0.39

4 8 8 9.20 0.28

-4 8 8 9.63 0.28

-4 9 8 2.84 0.17

-4 9 8 3.19 0.18

4 9 8 2.88 0.19

4 9 8 3.25 0.18

-4 9 -8 2.60 0.19

4 9 -8 2.87 0.21

-4 9 -8 2.96 0.21

-4 9 -8 2.20 0.34

4 9 -8 2.81 0.39

4 9 -8 2.44 0.35

-4 10 8 0.33 0.07

4 10 8 0.36 0.08

4 10 8 0.20 0.07

-4 10 8 0.18 0.07

-4 10 -8 0.22 0.08

4 10 -8 0.18 0.07

-4 10 -8 0.29 0.08

4 10 -8 0.24 0.09

-4 11 -8 1.05 0.13

4 11 8 0.96 0.12

-4 11 8 1.19 0.12

4 11 -8 1.04 0.13

4 11 -8 1.19 0.15

4 12 8 0.78 0.10

4 12 -8 0.74 0.11

-4 12 8 0.79 0.11

-4 12 -8 0.74 0.11

4 13 8 0.15 0.05

-4 13 -8 0.09 0.05

4 13 -8 0.09 0.05

-4 13 8 0.18 0.06

4 14 -8 0.13 0.04

-4 14 -8 0.11 0.04

4 0 9 2.70 0.28

4 0 -9 2.85 0.29

4 -1 -9 7.90 0.47

4 1 -9 8.08 0.46

4 -1 9 7.76 0.46

4 1 9 8.23 0.46

4 -2 9 1.40 0.20

4 -2 -9 1.70 0.25

4 2 9 1.33 0.19

4 2 -9 1.68 0.23

4 3 -9 4.81 0.36

4 -3 -9 4.88 0.38

4 3 9 4.67 0.35

4 -3 9 5.06 0.39

4 -4 9 1.51 0.22

4 -4 -9 1.61 0.23

4 4 9 1.46 0.20

4 4 -9 1.52 0.22

4 5 9 1.12 0.17

4 -5 9 1.05 0.19

4 5 -9 0.86 0.16

4 -5 -9 0.97 0.18

4 6 -9 1.61 0.25

4 7 9 0.41 0.06

-4 7 9 0.34 0.06

4 7 -9 0.25 0.12

-4 7 -9 0.28 0.12

4 7 -9 0.36 0.08

-4 7 -9 0.39 0.09

-4 7 -9 0.29 0.07

-4 8 9 1.15 0.11

-4 8 9 1.06 0.10

4 8 9 1.05 0.12

4 8 9 1.15 0.10

4 8 -9 1.20 0.14

-4 8 -9 1.22 0.13

-4 8 -9 0.94 0.12

4 8 -9 1.16 0.25

-4 8 -9 0.95 0.22

4 9 9 0.39 0.07

-4 9 9 0.38 0.07

4 9 9 0.21 0.07

-4 9 9 0.38 0.08

-4 9 -9 0.35 0.10

-4 9 -9 0.26 0.08

4 9 -9 0.26 0.10

4 9 -9 0.29 0.15

-4 9 -9 0.17 0.10

4 9 -9 -0.03 0.16

4 10 -9 0.62 0.10

4 10 9 0.79 0.11

-4 10 9 0.98 0.10

-4 10 9 0.91 0.11

-4 10 -9 0.88 0.12

-4 10 -9 0.71 0.11

4 10 -9 0.75 0.12

-4 11 -9 0.42 0.09

4 11 9 0.50 0.09

-4 11 9 0.48 0.09

4 11 -9 0.48 0.10

4 11 -9 0.51 0.12

4 12 9 0.13 0.06

-4 12 9 0.19 0.07

4 12 -9 0.35 0.09

-4 12 -9 0.24 0.08

-4 13 -9 0.03 0.05

4 13 9 0.10 0.05

4 13 -9 -0.01 0.05

-4 14 -9 -0.03 0.04

4 14 -9 0.04 0.03

4 0 10 15.37 0.67

4 0 -10 15.52 0.69

4 -1 10 36.17 1.05

4 1 -10 37.62 1.06

4 1 10 34.36 1.01

4 -1 -10 39.51 1.10

4 -2 10 21.29 0.81

4 2 10 21.23 0.78

4 -2 -10 22.08 0.84

4 2 -10 21.09 0.79

4 3 -10 18.15 0.74

4 -3 -10 19.13 0.79

4 3 10 17.68 0.72

4 -3 10 18.25 0.77

4 -4 -10 20.45 0.82

4 4 10 20.09 0.77

4 4 -10 18.19 0.75

-4 -4 -10 19.72 0.77

4 -4 10 19.80 0.81

4 5 -10 0.34 0.12

4 -5 -10 0.45 0.15

4 -5 10 0.18 0.09

4 5 10 0.18 0.08

4 6 -10 0.01 0.10

-4 7 10 1.40 0.11

4 7 10 1.45 0.12

4 7 10 1.41 0.10

-4 7 -10 1.57 0.14

-4 7 -10 1.40 0.14

4 7 -10 1.77 0.16

4 7 -10 1.37 0.26

-4 7 -10 1.17 0.23

4 8 10 1.22 0.11

-4 8 10 1.18 0.11

4 8 10 1.18 0.12

4 8 -10 1.29 0.15

-4 8 -10 1.27 0.14

-4 8 -10 1.37 0.14

-4 8 -10 0.90 0.21

4 8 -10 1.36 0.27

-4 9 10 0.53 0.08

-4 9 10 0.58 0.09

4 9 10 0.58 0.09

4 9 -10 0.47 0.11

-4 9 -10 0.64 0.12

-4 9 -10 0.38 0.09

-4 9 -10 0.28 0.14

4 9 -10 0.50 0.19

4 9 -10 0.18 0.11

-4 10 -10 0.85 0.11

-4 10 10 1.13 0.11

4 10 10 1.08 0.12

4 10 -10 1.11 0.13

-4 10 10 1.42 0.11

-4 10 -10 0.93 0.13

4 10 -10 1.09 0.15

4 11 10 0.83 0.10

-4 11 -10 0.64 0.10

-4 11 10 0.80 0.10

4 11 -10 0.77 0.11

4 11 -10 0.82 0.13

4 12 10 0.01 0.04

4 12 -10 0.02 0.06

-4 12 -10 0.06 0.06

-4 12 10 0.03 0.05

4 13 -10 0.05 0.04

-4 13 -10 -0.01 0.04

4 14 -10 0.18 0.04

-4 14 -10 0.20 0.04

4 0 11 20.39 0.83

4 0 -11 20.23 0.84

4 1 -11 1.51 0.25

4 1 11 1.49 0.23

4 -1 11 1.75 0.27

4 -1 -11 1.88 0.28

4 -2 11 36.09 1.13

4 -2 -11 36.43 1.15

4 2 11 33.82 1.06

4 2 -11 35.22 1.09

4 -3 11 3.25 0.35

4 3 11 3.30 0.34

4 3 -11 3.46 0.35

4 -3 -11 3.67 0.38

4 -4 -11 1.44 0.25

4 4 -11 1.45 0.23

-4 -4 -11 1.80 0.25

4 4 11 1.33 0.22

4 -4 11 1.30 0.23

4 -5 -11 0.93 0.20

4 -5 11 0.47 0.14

4 5 -11 0.80 0.18

-4 6 -11 1.79 0.14

-4 6 -11 1.77 0.15

4 6 -11 1.84 0.16

-4 6 11 1.86 0.16

-4 7 11 0.04 0.04

4 7 11 0.04 0.04

-4 7 -11 0.09 0.10

4 7 -11 0.11 0.12

-4 7 -11 0.16 0.07

4 7 -11 0.06 0.07

-4 7 -11 0.12 0.06

4 8 11 0.47 0.08

-4 8 11 0.46 0.07

-4 8 -11 0.37 0.15

4 8 -11 0.45 0.20

-4 8 -11 0.61 0.11

4 8 -11 0.54 0.11

-4 8 -11 0.33 0.08

4 9 11 0.63 0.09

-4 9 11 0.54 0.08

-4 9 -11 0.48 0.10

4 9 -11 0.59 0.12

-4 9 -11 0.68 0.12

-4 10 11 0.67 0.09

-4 10 -11 0.50 0.09

4 10 -11 0.56 0.10

4 10 11 0.68 0.10

4 10 -11 0.75 0.14

-4 10 -11 0.61 0.11

-4 11 11 0.07 0.05

-4 11 -11 -0.04 0.08

4 11 11 0.09 0.05

4 11 -11 -0.04 0.08

4 11 -11 0.19 0.09

4 12 11 0.02 0.04

-4 12 -11 0.00 0.05

4 12 -11 0.06 0.06

-4 13 -11 0.00 0.04

4 13 -11 0.04 0.04

-4 14 -11 0.09 0.03

4 14 -11 0.10 0.03

4 0 12 64.50 1.57

4 0 -12 64.75 1.59

4 1 12 11.04 0.64

4 -1 -12 11.59 0.68

4 -1 12 11.48 0.66

4 1 -12 12.00 0.68

4 -2 -12 67.33 1.66

4 -2 12 70.49 1.67

4 2 -12 64.73 1.58

4 2 12 67.76 1.60

4 -3 -12 6.30 0.51

4 3 -12 6.47 0.50

4 3 12 7.21 0.52

4 -3 12 8.09 0.57

4 4 12 22.56 0.92

4 4 -12 22.16 0.93

4 -4 -12 23.18 0.99

-4 -4 -12 23.06 0.94

4 -4 12 22.57 0.97

4 5 -12 1.12 0.23

4 -5 12 1.12 0.23

4 -5 -12 1.25 0.25

4 5 -12 1.48 0.13

-4 5 -12 1.38 0.13

-4 6 -12 0.13 0.06

4 6 -12 0.13 0.07

4 6 -12 0.14 0.06

-4 6 -12 0.18 0.07

-4 6 12 0.08 0.06

-4 7 12 4.44 0.18

4 7 -12 5.00 0.27

-4 7 -12 4.53 0.25

4 7 -12 3.73 0.22

-4 7 -12 4.80 0.25

4 7 -12 4.85 0.49

-4 7 -12 3.48 0.41

-4 8 12 1.24 0.11

4 8 -12 1.61 0.17

-4 8 -12 1.12 0.14

-4 8 -12 1.40 0.15

-4 8 -12 1.12 0.26

4 8 -12 1.06 0.26

4 9 12 0.42 0.08

-4 9 12 0.40 0.07

-4 9 -12 0.51 0.11

4 9 -12 0.51 0.12

-4 9 -12 0.33 0.09

4 10 -12 0.93 0.12

4 10 12 1.21 0.11

-4 10 -12 0.92 0.12

-4 10 12 1.22 0.11

-4 10 -12 0.85 0.12

4 10 -12 1.32 0.16

4 11 12 0.43 0.07

4 11 -12 0.26 0.08

4 11 -12 0.46 0.11

4 12 12 0.10 0.04

-4 12 -12 0.03 0.05

4 12 -12 0.09 0.05

-4 13 -12 0.12 0.05

4 13 -12 0.11 0.04

4 0 -13 0.46 0.19

4 0 13 0.61 0.18

4 1 -13 0.52 0.19

4 -1 -13 0.55 0.20

4 -1 13 0.50 0.15

4 1 13 0.33 0.14

4 2 13 7.59 0.57

4 -2 13 7.71 0.59

4 2 -13 7.97 0.59

4 -2 -13 8.68 0.62

4 3 13 2.27 0.31

4 3 -13 2.52 0.34

-4 -3 -13 2.55 0.35

4 -3 -13 2.23 0.34

4 -3 13 2.40 0.33

4 4 13 5.28 0.48

4 -4 -13 5.49 0.51

4 4 -13 5.30 0.48

4 -4 13 5.63 0.52

-4 -4 -13 5.34 0.49

4 -5 -13 -0.03 0.09

4 5 -13 -0.17 0.15

4 5 -13 0.05 0.06

-4 5 -13 0.03 0.05

-4 5 -13 0.03 0.05

-4 5 13 0.01 0.06

4 6 -13 0.10 0.07

-4 6 -13 0.06 0.05

-4 6 -13 0.03 0.06

4 6 -13 -0.02 0.05

-4 6 13 0.01 0.06

4 7 -13 0.89 0.23

-4 7 -13 0.76 0.22

-4 7 -13 1.27 0.14

4 7 -13 0.86 0.11

4 7 -13 1.14 0.14

-4 7 -13 1.07 0.13

-4 8 13 0.34 0.06

-4 8 -13 0.42 0.10

4 8 -13 0.48 0.11

-4 8 -13 0.33 0.08

-4 9 13 0.79 0.09

4 9 -13 0.95 0.15

-4 9 -13 0.90 0.13

-4 9 -13 0.66 0.11

4 10 -13 0.55 0.12

-4 10 -13 0.33 0.08

4 11 -13 0.35 0.08

-4 11 -13 0.32 0.08

4 11 -13 0.47 0.11

4 12 -13 0.00 0.05

-4 12 -13 0.05 0.05

-4 13 -13 0.05 0.03

4 13 -13 0.05 0.03

4 0 14 0.78 0.20

4 0 -14 0.80 0.23

4 1 14 8.87 0.64

4 1 -14 10.51 0.70

4 -1 -14 10.97 0.73

4 -1 14 9.88 0.68

4 -2 14 1.99 0.33

4 2 14 1.65 0.29

4 -2 -14 2.94 0.39

4 2 -14 2.64 0.37

4 3 14 12.64 0.76

-4 -3 -14 13.09 0.79

4 3 -14 12.09 0.76

4 -3 -14 13.14 0.81

4 -3 14 12.70 0.78

-4 3 14 13.88 0.42

4 -4 -14 0.93 0.24

4 -4 14 0.91 0.24

-4 -4 -14 1.11 0.25

4 4 -14 1.13 0.25

4 4 -14 1.24 0.13

-4 4 14 1.17 0.14

4 4 14 1.14 0.13

-4 4 -14 1.24 0.13

4 -5 -14 0.83 0.23

4 5 -14 0.34 0.15

-4 5 -14 0.62 0.09

4 5 -14 0.57 0.10

-4 5 -14 0.52 0.10

-4 5 14 0.78 0.12

-4 6 -14 15.80 0.48

-4 6 -14 15.66 0.43

-4 6 14 15.82 0.50

4 6 -14 14.41 0.44

4 7 -14 2.24 0.19

4 7 -14 1.75 0.16

-4 7 -14 1.98 0.17

-4 7 -14 1.98 0.18

4 7 -14 2.27 0.35

-4 7 -14 1.38 0.27

-4 8 -14 6.28 0.29

4 8 -14 6.83 0.33

-4 9 -14 0.06 0.08

4 9 -14 0.21 0.10

-4 9 -14 0.05 0.06

4 10 -14 3.01 0.22

-4 10 -14 2.07 0.16

-4 11 -14 0.35 0.07

4 11 -14 0.43 0.08

4 11 -14 0.60 0.11

4 12 -14 0.24 0.06

-4 12 -14 0.16 0.05

4 13 -14 0.04 0.03

-4 13 -14 0.05 0.03

4 0 15 8.43 0.66

4 0 -15 9.18 0.70

4 -1 15 2.84 0.39

4 1 -15 3.00 0.41

4 -1 -15 3.08 0.41

4 1 15 2.42 0.35

4 2 15 0.53 0.20

4 2 -15 0.66 0.21

-4 -2 -15 0.51 0.19

4 -2 15 0.52 0.18

4 -2 -15 0.59 0.20

4 2 15 0.69 0.10

4 3 -15 1.63 0.31

4 -3 15 1.34 0.29

4 -3 -15 1.27 0.28

4 3 15 1.42 0.27

-4 -3 -15 1.59 0.30

4 3 -15 1.59 0.13

4 3 15 1.56 0.15

-4 3 -15 1.68 0.15

-4 3 15 1.85 0.17

-4 -4 -15 0.67 0.21

4 4 -15 0.92 0.25

4 -4 -15 0.65 0.22

4 4 -15 0.97 0.12

-4 4 15 0.97 0.13

4 4 15 0.92 0.13

-4 4 -15 0.95 0.13

4 5 -15 0.03 0.13

-4 5 -15 0.28 0.17

4 -5 -15 0.16 0.11

4 5 -15 0.19 0.07

-4 5 -15 0.20 0.06

-4 5 15 0.33 0.10

-4 5 -15 0.26 0.09

-4 6 -15 0.37 0.09

4 6 -15 0.31 0.09

-4 6 15 0.43 0.11

-4 6 -15 0.39 0.10

4 6 -15 0.36 0.09

4 7 -15 0.46 0.11

-4 7 -15 0.43 0.10

4 7 -15 0.52 0.10

-4 7 -15 0.47 0.10

4 8 -15 2.22 0.20

-4 8 -15 1.85 0.17

-4 8 -15 2.05 0.17

-4 9 -15 0.57 0.10

-4 9 -15 0.50 0.10

4 9 -15 0.72 0.14

4 10 -15 0.10 0.09

-4 10 -15 -0.05 0.06

-4 11 -15 0.15 0.06

4 11 -15 0.16 0.06

4 11 -15 0.16 0.07

-4 12 -15 0.02 0.04

4 12 -15 0.05 0.04

4 0 -16 6.00 0.59

4 0 16 5.17 0.54

4 0 16 6.00 0.26

4 1 16 2.27 0.37

4 -1 16 2.68 0.40

4 -1 -16 2.72 0.42

4 1 -16 2.42 0.39

-4 -1 -16 2.67 0.40

4 1 16 2.81 0.19

4 -2 16 17.78 1.02

-4 -2 -16 17.89 1.00

4 2 16 16.72 0.97

4 2 -16 15.97 0.96

4 -2 -16 16.99 1.01

4 2 -16 17.35 0.42

-4 2 16 17.46 0.50

4 2 16 18.87 0.50

4 -3 -16 6.29 0.62

4 3 -16 5.55 0.57

-4 -3 -16 5.70 0.58

4 3 -16 6.20 0.27

4 3 16 6.13 0.30

-4 3 16 6.45 0.31

-4 3 -16 6.69 0.30

4 -4 -16 14.12 0.93

-4 4 -16 13.89 0.89

4 4 -16 13.36 0.88

-4 -4 -16 15.87 0.95

4 4 -16 15.60 0.45

-4 4 -16 15.74 0.47

-4 4 16 16.35 0.51

4 4 16 16.69 0.51

-4 5 -16 0.14 0.13

-4 5 -16 0.02 0.05

4 5 -16 0.02 0.07

-4 5 16 0.16 0.10

-4 5 -16 -0.06 0.08

4 5 -16 -0.01 0.06

-4 6 -16 2.18 0.17

4 6 -16 2.28 0.19

-4 6 -16 2.05 0.18

4 6 -16 1.98 0.18

-4 6 16 2.22 0.21

-4 7 -16 0.36 0.08

4 7 -16 0.26 0.10

-4 7 -16 0.35 0.10

4 7 -16 0.30 0.08

-4 8 -16 5.65 0.26

4 8 -16 6.70 0.31

-4 8 -16 5.36 0.28

4 8 -16 4.34 0.24

-4 9 -16 0.07 0.06

-4 9 -16 0.03 0.06

4 9 -16 0.17 0.09

4 10 -16 0.07 0.06

-4 10 -16 -0.01 0.06

-4 10 -16 0.03 0.05

4 10 -16 0.09 0.08

4 11 -16 0.02 0.04

-4 11 -16 0.02 0.04

4 11 -16 0.07 0.06

4 12 -16 0.53 0.06

-4 12 -16 0.53 0.06

-4 0 -17 1.42 0.30

4 0 -17 1.09 0.28

4 0 17 1.37 0.29

4 0 17 1.24 0.13

4 -1 -17 0.12 0.17

-4 1 -17 0.14 0.16

-4 -1 -17 0.07 0.14

4 1 -17 0.22 0.21

4 1 17 0.14 0.07

4 2 -17 7.09 0.67

4 -2 -17 7.31 0.69

-4 -2 -17 7.55 0.69

-4 2 -17 7.23 0.67

4 2 -17 7.96 0.29

-4 2 17 9.21 0.37

4 2 17 8.23 0.34

-4 -3 -17 0.44 0.20

4 3 -17 0.55 0.19

4 -3 -17 0.56 0.20

-4 3 -17 0.85 0.26

4 3 -17 0.55 0.09

-4 3 17 0.56 0.12

4 3 17 0.64 0.11

-4 3 -17 0.57 0.11

-4 -4 -17 1.23 0.29

4 4 -17 1.15 0.27

4 -4 -17 1.31 0.31

-4 4 -17 1.23 0.30

4 4 -17 1.42 0.15

-4 4 17 1.32 0.17

4 4 17 1.48 0.16

-4 4 -17 1.47 0.16

-4 5 -17 -0.01 0.09

4 5 -17 -0.06 0.08

-4 5 -17 0.04 0.05

4 5 -17 -0.07 0.08

-4 5 17 0.06 0.07

-4 5 -17 0.03 0.08

4 6 -17 0.16 0.07

-4 6 -17 0.24 0.07

-4 6 17 0.27 0.11

4 6 -17 0.21 0.08

-4 6 -17 0.20 0.08

-4 7 -17 0.00 0.06

4 7 -17 0.00 0.08

-4 7 -17 -0.04 0.07

4 7 -17 -0.06 0.06

4 8 -17 0.11 0.09

-4 8 -17 0.02 0.06

4 8 -17 0.09 0.07

-4 8 -17 0.13 0.08

4 9 -17 0.16 0.08

-4 9 -17 -0.04 0.06

4 10 -17 0.11 0.06

-4 10 -17 0.09 0.06

4 10 -17 0.10 0.07

-4 10 -17 -0.03 0.05

-4 11 -17 0.27 0.06

4 11 -17 0.30 0.06

4 12 -17 0.05 0.03

-4 12 -17 0.01 0.03

4 0 -18 15.92 1.04

-4 0 -18 13.67 0.95

4 0 18 15.78 0.45

4 -1 -18 1.39 0.34

4 1 -18 1.44 0.33

-4 -1 -18 1.30 0.32

-4 1 -18 1.38 0.32

-4 1 18 1.74 0.17

4 1 18 1.56 0.15

4 -2 -18 6.54 0.67

-4 -2 -18 6.24 0.65

-4 2 -18 6.63 0.66

4 2 -18 6.30 0.65

4 2 -18 6.26 0.26

4 2 18 6.88 0.32

-4 2 18 7.31 0.34

-4 -3 -18 1.45 0.33

4 3 -18 2.03 0.36

4 -3 -18 1.19 0.30

-4 3 -18 2.02 0.38

4 3 -18 1.84 0.16

-4 3 18 2.17 0.20

4 3 18 1.98 0.18

-4 3 -18 1.70 0.17

-4 4 -18 7.69 0.34

4 4 -18 7.46 0.31

-4 4 18 7.93 0.38

4 5 -18 0.30 0.08

4 5 -18 0.31 0.09

-4 5 18 0.40 0.12

-4 5 -18 0.36 0.10

4 6 -18 0.52 0.11

-4 6 -18 0.56 0.09

-4 6 -18 0.48 0.11

4 6 -18 0.50 0.11

4 7 -18 0.31 0.09

-4 7 -18 0.27 0.07

-4 7 -18 0.49 0.11

4 7 -18 0.30 0.09

-4 8 -18 5.59 0.28

-4 8 -18 5.07 0.23

4 8 -18 5.88 0.28

4 8 -18 5.05 0.24

-4 9 -18 -0.02 0.06

4 9 -18 0.09 0.06

-4 10 -18 0.40 0.07

4 10 -18 0.29 0.07

-4 10 -18 0.12 0.05

4 10 -18 0.31 0.07

-4 11 -18 0.09 0.04

4 11 -18 0.09 0.04

4 0 -19 0.36 0.21

-4 0 -19 0.44 0.22

-4 0 19 0.19 0.07

4 0 19 0.27 0.08

-4 1 19 0.12 0.07

4 1 19 0.26 0.09

-4 2 -19 3.27 0.22

4 2 19 3.33 0.23

-4 2 19 3.43 0.25

4 3 -19 0.52 0.09

4 3 19 0.35 0.09

-4 3 -19 0.48 0.11

-4 3 19 0.38 0.11

4 4 -19 0.17 0.08

-4 4 -19 0.27 0.10

-4 4 19 0.40 0.12

4 5 -19 0.12 0.06

-4 5 -19 0.11 0.08

-4 5 19 0.30 0.12

4 5 -19 0.18 0.09

4 6 -19 0.14 0.07

4 6 -19 0.02 0.06

-4 6 -19 -0.01 0.08

4 7 -19 0.20 0.08

-4 7 -19 0.05 0.05

-4 7 -19 0.15 0.08

4 7 -19 0.06 0.06

4 8 -19 0.33 0.08

4 8 -19 0.19 0.06

-4 8 -19 0.26 0.08

-4 9 -19 -0.02 0.05

4 9 -19 0.10 0.06

4 10 -19 0.12 0.05

-4 10 -19 0.07 0.04

-4 10 -19 0.07 0.04

4 10 -19 0.14 0.05

-4 11 -19 0.07 0.03

4 11 -19 0.06 0.03

4 0 20 14.01 0.43

-4 0 20 14.07 0.46

-4 1 20 0.46 0.11

4 1 20 0.41 0.09

-4 1 -20 0.62 0.12

-4 -1 20 0.41 0.10

-4 2 20 0.28 0.10

4 2 20 0.35 0.09

-4 2 -20 0.45 0.10

4 3 -20 1.17 0.12

-4 3 -20 1.32 0.16

-4 3 20 1.12 0.16

4 4 -20 7.92 0.31

-4 4 -20 7.99 0.35

-4 4 20 8.95 0.40

4 4 -20 7.79 0.34

4 5 -20 0.04 0.06

-4 5 20 0.17 0.11

4 5 -20 0.12 0.08

-4 5 -20 -0.04 0.09

4 6 -20 0.11 0.07

4 6 -20 0.08 0.07

-4 6 -20 -0.01 0.09

4 7 -20 0.64 0.10

-4 7 -20 0.68 0.12

4 7 -20 0.51 0.09

4 8 -20 0.11 0.07

4 8 -20 0.01 0.04

-4 8 -20 0.06 0.06

4 9 -20 0.13 0.06

-4 9 -20 0.02 0.04

-4 10 -20 0.08 0.03

4 10 -20 0.07 0.04

-4 10 -20 0.02 0.03

4 0 21 2.92 0.20

-4 0 -21 3.20 0.21

-4 0 21 2.91 0.21

-4 -1 21 0.41 0.10

4 1 21 0.49 0.09

-4 1 -21 0.62 0.12

-4 1 21 0.55 0.11

-4 2 21 0.56 0.12

4 2 21 0.58 0.11

-4 2 -21 0.71 0.13

-4 -2 21 0.64 0.11

-4 3 21 0.38 0.11

-4 3 -21 0.33 0.10

-4 -3 21 0.27 0.07

4 4 -21 0.03 0.05

4 4 -21 0.14 0.09

-4 -4 21 0.02 0.05

-4 4 21 0.29 0.12

-4 4 -21 0.13 0.09

4 5 -21 0.06 0.06

4 5 -21 -0.04 0.07

-4 5 -21 -0.07 0.09

4 6 -21 0.21 0.07

4 6 -21 0.17 0.08

-4 6 -21 0.06 0.08

4 7 -21 0.38 0.08

-4 7 -21 0.41 0.09

4 7 -21 0.38 0.08

-4 8 -21 0.19 0.06

4 8 -21 0.17 0.06

4 8 -21 0.09 0.04

4 9 -21 0.12 0.05

-4 9 -21 0.00 0.04

-4 0 -22 3.99 0.23

-4 0 22 3.78 0.24

4 0 22 3.54 0.21

-4 1 22 -0.05 0.08

-4 1 -22 0.07 0.07

-4 -1 22 -0.03 0.06

-4 -1 -22 0.06 0.06

-4 2 22 0.36 0.11

-4 2 -22 0.60 0.11

-4 -2 -22 0.56 0.10

-4 -2 22 0.46 0.09

-4 3 -22 0.46 0.10

-4 -3 22 0.51 0.09

-4 3 22 0.49 0.12

4 3 -22 0.41 0.11

-4 4 -22 2.22 0.18

-4 4 22 2.31 0.21

4 4 -22 1.99 0.17

4 5 -22 0.15 0.06

-4 5 -22 0.09 0.07

4 5 -22 0.09 0.07

4 6 -22 0.51 0.08

4 6 -22 0.47 0.09

-4 6 -22 0.55 0.10

4 7 -22 0.01 0.04

4 7 -22 0.03 0.04

-4 7 -22 0.04 0.06

4 8 -22 1.05 0.08

-4 8 -22 1.34 0.11

4 8 -22 1.27 0.09

4 9 -22 0.01 0.03

-4 9 -22 0.06 0.03

-4 0 23 0.04 0.07

-4 0 -23 0.07 0.06

-4 -1 23 -0.03 0.06

-4 1 -23 0.04 0.06

-4 -1 -23 0.05 0.06

-4 1 23 0.01 0.07

-4 2 23 0.19 0.09

-4 -2 -23 0.18 0.06

-4 -2 23 0.08 0.06

4 2 -23 -0.01 0.08

-4 2 -23 0.16 0.08

-4 3 -23 0.10 0.07

-4 -3 23 0.11 0.05

-4 3 23 0.11 0.08

4 3 -23 0.05 0.07

-4 4 -23 0.29 0.09

4 4 -23 0.08 0.07

-4 5 -23 0.15 0.08

4 5 -23 0.20 0.07

4 6 -23 0.16 0.06

-4 6 -23 0.28 0.08

4 7 -23 0.12 0.04

-4 7 -23 0.06 0.05

4 7 -23 0.02 0.04

-4 8 -23 0.01 0.03

-4 0 -24 2.21 0.16

-4 0 24 2.33 0.18

-4 1 -24 0.23 0.07

-4 -1 24 0.23 0.07

-4 1 24 0.32 0.09

4 1 -24 0.23 0.08

-4 -1 -24 0.29 0.07

-4 2 24 0.44 0.09

-4 -2 24 0.48 0.08

-4 -2 -24 0.45 0.07

4 2 -24 0.43 0.09

-4 2 -24 0.43 0.09

-4 3 -24 0.17 0.07

4 3 -24 0.18 0.07

-4 4 -24 0.96 0.11

4 4 -24 1.06 0.12

-4 5 -24 0.29 0.07

4 5 -24 0.28 0.07

-4 6 -24 0.35 0.07

4 6 -24 0.29 0.06

-4 7 -24 0.01 0.03

-4 0 -25 0.30 0.06

4 0 -25 0.33 0.07

-4 0 25 0.33 0.07

-4 1 25 0.12 0.06

-4 1 -25 -0.01 0.05

-4 -1 -25 0.06 0.04

-4 -1 25 0.11 0.05

4 1 -25 -0.03 0.06

4 2 -25 0.68 0.09

-4 -2 25 0.54 0.07

-4 2 -25 0.61 0.09

4 3 -25 -0.01 0.05

-4 3 -25 0.01 0.05

4 4 -25 0.04 0.05

-4 4 -25 0.12 0.05

-4 5 -25 0.02 0.04

4 5 -25 0.07 0.05

-4 6 -25 0.07 0.04

4 6 -25 0.06 0.03

-4 0 -26 0.26 0.05

4 0 -26 0.28 0.06

4 1 -26 -0.05 0.05

-4 -1 26 -0.02 0.04

-4 1 -26 -0.05 0.04

-4 -1 -26 -0.02 0.03

4 -1 -26 0.03 0.04

-4 2 -26 1.10 0.10

4 2 -26 1.23 0.11

4 -2 -26 1.15 0.09

4 -3 -26 0.09 0.03

4 3 -26 0.05 0.04

-4 3 -26 0.14 0.05

4 4 -26 0.46 0.06

-4 4 -26 0.46 0.06

4 5 -26 0.07 0.03

-4 5 -26 0.05 0.03

4 0 -27 0.14 0.04

-4 1 -27 0.08 0.04

4 -1 -27 0.02 0.03

4 1 -27 -0.01 0.03

-4 2 -27 0.07 0.04

4 2 -27 0.04 0.03

-4 3 -27 0.20 0.04

4 3 -27 0.14 0.04

5 0 0 0.01 0.05

-5 1 0 38.69 0.32

5 1 0 40.40 0.60

5 -1 0 38.95 0.65

5 1 0 38.75 0.45

5 2 0 12.43 0.31

-5 2 0 12.52 0.25

5 -2 0 12.36 0.38

5 2 0 12.85 0.30

5 2 0 12.28 0.27

5 -3 0 3.55 0.18

5 3 0 3.80 0.20

5 -3 0 3.51 0.21

-5 3 0 3.40 0.16

5 3 0 3.49 0.18

-5 4 0 98.27 1.14

5 -4 0 96.16 1.00

5 4 0 96.70 0.99

5 4 0 99.57 1.19

5 5 0 3.04 0.24

-5 5 0 2.88 0.21

5 5 0 2.92 0.22

5 6 0 9.29 0.46

-5 6 0 8.74 0.41

-5 6 0 9.31 0.47

5 6 0 9.41 0.44

5 7 0 0.38 0.12

-5 7 0 0.32 0.10

-5 7 0 0.38 0.13

5 7 0 0.32 0.12

-5 8 0 6.53 0.42

5 8 0 7.27 0.49

5 8 0 6.62 0.45

-5 8 0 6.83 0.48

-5 9 0 0.06 0.09

5 9 0 0.14 0.12

-5 9 0 0.11 0.11

5 9 0 -0.03 0.10

5 9 0 0.06 0.04

-5 9 0 0.05 0.06

-5 9 0 0.09 0.05

-5 9 0 0.04 0.05

5 9 0 0.05 0.05

5 9 0 0.05 0.05

5 10 0 8.22 0.59

5 10 0 7.77 0.31

-5 10 0 7.35 0.27

5 10 0 7.36 0.26

5 10 0 7.63 0.54

-5 10 0 7.65 0.58

-5 10 0 7.22 0.29

-5 10 0 7.73 0.29

5 10 0 7.52 0.29

5 11 0 0.16 0.08

-5 11 0 0.04 0.06

-5 11 0 0.15 0.08

5 11 0 0.03 0.07

5 11 0 0.23 0.06

-5 12 0 0.64 0.10

5 12 0 0.90 0.11

-5 12 0 0.98 0.12

5 13 0 0.19 0.07

-5 13 0 0.09 0.05

-5 13 0 0.05 0.06

5 13 0 0.08 0.06

-5 14 0 0.15 0.05

5 14 0 0.06 0.05

-5 14 0 0.14 0.05

5 0 -1 0.00 0.05

5 0 1 0.01 0.04

5 0 -1 0.01 0.01

5 -1 -1 73.77 0.94

5 1 1 76.99 0.69

5 1 -1 75.99 0.86

5 1 -1 78.39 0.58

5 1 1 80.23 0.91

5 1 -1 74.06 0.70

5 -1 1 76.86 0.92

-5 2 1 46.53 0.50

5 -2 1 46.91 0.69

5 2 -1 47.12 0.63

5 -2 -1 47.96 0.76

5 2 -1 47.42 0.64

5 2 1 47.70 0.62

5 2 -1 45.31 0.55

5 2 1 45.90 0.67

5 2 1 46.30 0.56

5 3 -1 14.83 0.37

-5 3 -1 14.80 0.36

-5 3 1 14.48 0.33

5 3 -1 15.20 0.32

5 3 1 14.94 0.36

5 -3 -1 14.70 0.43

5 -3 1 14.18 0.37

5 3 1 15.66 0.41

5 3 -1 15.47 0.42

5 3 1 15.20 0.37

-5 4 1 54.53 0.80

5 4 1 53.59 0.69

5 -4 -1 52.86 0.87

-5 4 1 57.56 0.92

-5 4 -1 53.13 0.84

5 4 -1 52.57 0.84

5 4 1 58.43 0.93

5 4 -1 54.00 0.93

5 -4 -1 49.84 0.67

5 4 1 55.27 0.85

-5 5 1 6.57 0.31

5 5 1 7.27 0.36

5 5 -1 6.41 0.33

5 5 -1 6.15 0.21

-5 5 -1 6.44 0.32

-5 5 1 6.79 0.36

5 5 -1 6.83 0.36

5 5 1 6.77 0.33

-5 6 1 0.63 0.12

-5 6 -1 0.92 0.15

5 6 -1 1.02 0.16

5 6 1 1.02 0.17

5 6 -1 1.00 0.15

5 6 1 0.81 0.14

-5 6 1 0.86 0.15

-5 7 -1 0.21 0.12

-5 7 -1 0.24 0.10

5 7 -1 0.25 0.11

5 7 1 0.17 0.11

-5 7 1 0.18 0.08

5 7 -1 0.26 0.11

5 7 1 0.16 0.10

-5 7 1 0.25 0.10

-5 8 1 8.15 0.46

5 8 -1 7.44 0.50

5 8 1 7.97 0.50

-5 8 -1 7.48 0.51

-5 8 -1 7.00 0.45

5 8 1 7.98 0.48

5 8 -1 7.43 0.47

-5 8 1 8.27 0.52

5 9 1 2.43 0.31

-5 9 -1 1.88 0.26

5 9 -1 2.41 0.32

-5 9 1 2.06 0.26

5 9 1 2.05 0.14

-5 9 -1 2.11 0.15

5 9 -1 2.15 0.13

5 9 -1 2.42 0.17

5 9 1 2.25 0.28

-5 9 1 2.29 0.16

-5 9 -1 2.06 0.30

-5 9 1 2.24 0.15

-5 9 1 2.31 0.30

-5 9 -1 2.16 0.15

5 9 -1 2.30 0.29

5 9 1 2.23 0.16

-5 9 1 2.41 0.16

5 9 -1 2.31 0.16

5 9 1 2.17 0.15

-5 9 -1 2.43 0.16

-5 10 -1 5.25 0.45

5 10 -1 5.57 0.50

-5 10 1 5.03 0.23

5 10 -1 5.77 0.26

-5 10 1 5.37 0.25

-5 10 -1 5.19 0.24

-5 10 1 4.84 0.45

5 10 1 5.26 0.26

5 10 -1 4.91 0.44

5 10 1 5.15 0.44

5 10 -1 5.12 0.21

5 10 1 5.32 0.22

-5 10 -1 5.56 0.24

-5 10 -1 5.00 0.49

-5 10 -1 5.67 0.25

-5 10 1 5.85 0.25

5 10 -1 5.54 0.25

5 10 1 5.46 0.24

5 11 -1 0.14 0.06

-5 11 -1 0.14 0.07

5 11 1 0.09 0.07

5 11 -1 0.16 0.08

5 11 1 0.17 0.09

-5 11 1 0.05 0.06

-5 11 -1 0.04 0.06

5 11 -1 0.03 0.07

-5 11 1 -0.01 0.07

5 11 1 0.29 0.07

-5 12 1 3.72 0.21

-5 12 -1 4.00 0.21

5 12 1 4.34 0.24

5 12 -1 4.87 0.24

-5 12 1 4.18 0.22

5 12 -1 4.43 0.23

5 12 1 4.08 0.22

-5 12 -1 4.36 0.23

5 13 -1 0.19 0.07

5 13 1 0.15 0.07

-5 13 1 0.05 0.05

-5 13 -1 0.01 0.04

-5 13 1 0.04 0.05

-5 13 -1 0.11 0.06

5 13 1 0.07 0.06

5 13 -1 0.05 0.06

-5 14 1 0.15 0.05

-5 14 -1 0.14 0.05

5 14 1 0.06 0.05

-5 14 1 0.05 0.05

-5 14 -1 0.03 0.05

5 14 -1 0.09 0.05

5 0 2 0.01 0.05

5 0 -2 0.04 0.05

-5 1 2 22.77 0.29

5 -1 -2 21.73 0.48

5 1 -2 22.66 0.46

5 1 2 22.74 0.48

5 -1 2 22.35 0.49

5 2 2 56.27 0.66

-5 2 2 60.08 0.67

5 2 -2 57.35 0.75

5 -2 -2 55.99 0.84

5 2 2 58.58 0.80

5 -2 2 58.63 0.82

5 2 -2 56.71 0.74

5 3 2 20.74 0.43

5 3 2 19.97 0.44

5 3 2 20.76 0.47

-5 3 2 19.38 0.38

5 3 -2 18.32 0.37

5 -3 -2 20.11 0.51

5 3 -2 21.62 0.51

-5 3 2 20.34 0.45

5 4 2 34.86 0.65

5 4 -2 34.48 0.47

-5 4 -2 34.29 0.68

5 -4 -2 32.60 0.70

5 4 -2 36.73 0.76

-5 4 2 35.08 0.70

5 4 2 35.78 0.71

-5 4 2 33.14 0.60

5 5 2 7.26 0.33

-5 5 -2 7.28 0.35

-5 5 2 7.30 0.32

5 5 2 7.68 0.37

5 5 -2 7.50 0.36

5 -5 -2 7.49 0.33

5 5 -2 8.27 0.40

-5 5 2 7.41 0.36

5 6 2 4.98 0.31

-5 6 2 4.83 0.33

-5 6 2 4.54 0.29

5 6 -2 5.48 0.35

-5 6 -2 5.47 0.34

5 6 2 5.28 0.35

5 6 -2 5.49 0.38

-5 7 -2 0.55 0.13

5 7 -2 0.75 0.16

5 7 2 0.70 0.16

5 7 -2 0.63 0.14

-5 7 2 0.77 0.16

5 7 2 0.71 0.14

-5 7 2 0.74 0.14

5 8 -2 2.17 0.27

-5 8 2 2.52 0.29

5 8 2 2.41 0.27

5 8 -2 2.28 0.29

-5 8 -2 2.31 0.26

-5 8 -2 2.01 0.28

5 8 2 2.43 0.28

-5 9 2 0.12 0.06

5 9 -2 0.14 0.06

-5 9 2 0.03 0.05

-5 9 2 0.07 0.05

5 9 -2 0.05 0.05

5 9 -2 0.09 0.04

-5 9 -2 0.20 0.07

-5 9 -2 0.06 0.05

5 9 2 0.00 0.05

5 9 2 0.06 0.05

-5 9 -2 0.09 0.05

5 9 2 0.05 0.04

5 9 2 -0.04 0.09

-5 9 -2 0.10 0.11

-5 9 2 0.05 0.11

5 9 -2 -0.04 0.08

-5 9 -2 0.07 0.11

5 9 -2 0.10 0.11

5 9 -2 0.16 0.06

-5 10 2 6.17 0.51

5 10 2 6.66 0.25

5 10 -2 6.05 0.23

-5 10 2 6.45 0.27

-5 10 -2 6.01 0.26

5 10 -2 6.01 0.50

5 10 2 6.88 0.30

-5 10 -2 6.30 0.55

5 10 -2 6.28 0.27

-5 10 2 6.48 0.26

5 10 2 6.64 0.49

-5 10 -2 5.85 0.49

5 10 -2 6.55 0.54

5 10 -2 6.05 0.26

-5 10 2 7.07 0.27

5 10 2 6.92 0.27

-5 10 -2 6.67 0.28

5 10 -2 6.50 0.27

-5 11 -2 0.70 0.10

5 11 2 0.54 0.09

5 11 -2 0.62 0.10

-5 11 -2 0.66 0.11

5 11 -2 0.58 0.10

5 11 -2 0.65 0.08

5 11 2 0.65 0.11

5 11 2 0.64 0.09

-5 11 2 0.67 0.10

-5 11 2 0.49 0.09

5 12 -2 1.31 0.14

5 12 2 1.51 0.15

-5 12 -2 1.11 0.12

-5 12 2 1.06 0.12

5 12 2 1.21 0.12

-5 12 2 1.27 0.13

5 12 -2 1.33 0.13

-5 12 -2 1.17 0.12

5 13 2 0.13 0.07

-5 13 -2 0.04 0.06

5 13 -2 0.16 0.06

-5 13 -2 -0.02 0.04

5 13 -2 0.03 0.06

-5 13 2 0.00 0.05

5 13 2 -0.01 0.05

-5 13 2 0.03 0.06

-5 14 -2 0.08 0.04

-5 14 2 0.21 0.06

-5 14 -2 0.06 0.05

5 14 -2 0.06 0.05

5 0 3 0.00 0.05

5 0 -3 0.02 0.06

5 -1 -3 28.65 0.60

5 1 -3 28.86 0.57

5 -2 -3 28.89 0.62

5 2 -3 28.48 0.56

-5 2 3 28.79 0.44

5 3 -3 36.07 0.61

5 3 -3 37.79 0.70

5 3 3 35.91 0.60

-5 3 3 35.73 0.60

5 4 3 4.05 0.24

5 -4 -3 4.12 0.26

5 4 -3 3.90 0.18

-5 4 -3 4.40 0.25

5 4 -3 4.48 0.27

-5 4 3 3.80 0.23

5 4 3 4.10 0.22

5 -5 -3 1.91 0.18

5 5 -3 2.12 0.21

5 5 3 1.92 0.19

5 -5 3 1.95 0.16

-5 5 -3 2.16 0.21

-5 5 3 1.77 0.19

5 5 3 1.66 0.17

-5 6 -3 6.98 0.40

5 6 3 6.41 0.38

5 6 -3 6.69 0.39

5 6 -3 7.25 0.43

-5 6 3 6.65 0.37

5 6 3 6.51 0.36

5 7 -3 2.14 0.25

5 7 -3 2.32 0.27

-5 7 -3 1.93 0.23

5 7 3 2.20 0.23

-5 7 3 2.09 0.25

5 8 -3 6.88 0.50

-5 8 -3 6.45 0.46

-5 8 -3 6.98 0.51

5 8 -3 6.77 0.47

-5 8 3 6.33 0.43

5 8 3 6.68 0.43

5 9 3 3.86 0.19

5 9 -3 3.88 0.17

5 9 -3 3.97 0.21

5 9 3 3.99 0.36

-5 9 3 3.65 0.37

5 9 -3 3.70 0.20

-5 9 3 4.36 0.21

-5 9 -3 3.78 0.20

5 9 3 4.08 0.22

-5 9 -3 3.97 0.21

-5 9 -3 4.02 0.19

5 9 -3 4.01 0.39

-5 9 3 3.92 0.20

-5 9 -3 3.59 0.37

5 9 -3 4.24 0.43

-5 9 -3 4.04 0.43

5 9 -3 3.72 0.21

-5 9 3 4.17 0.20

5 9 3 3.87 0.19

5 10 -3 6.67 0.23

5 10 -3 6.85 0.53

5 10 -3 7.07 0.28

-5 10 3 7.11 0.27

-5 10 3 7.42 0.29

-5 10 -3 6.57 0.27

5 10 3 7.73 0.53

5 10 3 7.17 0.26

-5 10 3 6.82 0.52

-5 10 -3 7.50 0.60

5 10 3 7.39 0.31

5 10 -3 7.00 0.57

-5 10 -3 6.35 0.51

5 10 -3 6.19 0.28

5 10 -3 6.93 0.28

-5 10 3 8.02 0.29

-5 10 -3 7.41 0.29

5 10 3 7.35 0.28

5 11 -3 1.51 0.11

5 11 -3 1.59 0.14

-5 11 -3 1.57 0.14

5 11 3 1.59 0.16

-5 11 3 1.56 0.14

5 11 3 1.56 0.13

-5 11 3 1.66 0.15

-5 11 -3 1.58 0.15

5 11 -3 1.64 0.15

5 11 3 1.59 0.14

5 12 -3 0.66 0.10

-5 12 -3 0.33 0.08

-5 12 3 0.53 0.09

5 12 3 0.66 0.11

-5 12 -3 0.36 0.07

5 12 -3 0.30 0.08

-5 12 3 0.48 0.09

5 12 3 0.57 0.10

-5 13 3 -0.04 0.05

-5 13 -3 0.02 0.05

-5 13 -3 0.04 0.04

5 13 3 0.10 0.07

5 13 -3 0.01 0.06

5 13 3 0.10 0.06

-5 13 3 0.09 0.06

-5 14 3 0.76 0.08

-5 14 -3 0.56 0.06

5 14 -3 0.62 0.08

-5 14 -3 0.62 0.08

-5 15 -3 0.10 0.03

5 15 -3 0.15 0.04

5 0 -4 0.03 0.07

5 1 -4 17.73 0.49

5 -1 -4 17.01 0.50

5 -2 -4 2.46 0.20

5 2 -4 2.63 0.19

-5 2 4 2.23 0.13

5 3 -4 6.14 0.28

5 -3 -4 6.03 0.31

-5 3 4 5.91 0.24

5 4 -4 18.58 0.47

5 4 -4 18.84 0.58

5 4 4 17.52 0.49

-5 4 4 18.66 0.49

5 -5 -4 10.24 0.43

5 5 -4 10.61 0.49

5 -5 4 10.66 0.40

5 5 4 10.45 0.32

-5 5 -4 10.25 0.45

-5 5 4 10.21 0.41

5 5 4 10.39 0.41

5 6 -4 34.03 0.96

-5 6 -4 31.74 0.88

5 6 4 33.08 0.80

-5 6 4 31.68 0.80

-5 7 4 0.56 0.13

-5 7 -4 0.32 0.12

5 7 -4 0.49 0.15

5 7 -4 0.35 0.12

5 7 4 0.59 0.14

5 8 4 1.43 0.21

-5 8 4 1.27 0.11

5 8 4 1.42 0.11

-5 8 4 1.39 0.22

5 8 -4 1.24 0.11

5 8 4 1.43 0.13

-5 8 -4 1.38 0.23

5 8 -4 1.17 0.22

5 8 -4 1.30 0.22

-5 8 -4 1.36 0.13

5 8 4 1.47 0.11

5 9 4 0.59 0.08

5 9 -4 0.55 0.09

5 9 4 0.63 0.09

-5 9 -4 0.48 0.08

-5 9 4 0.74 0.09

5 9 4 0.59 0.09

-5 9 4 0.63 0.10

-5 9 -4 0.54 0.09

5 9 -4 0.53 0.09

5 9 4 0.64 0.17

-5 9 -4 0.45 0.17

5 9 -4 0.41 0.16

-5 9 4 0.65 0.17

5 9 -4 0.55 0.18

-5 9 -4 0.46 0.17

-5 9 -4 0.65 0.10

5 9 -4 0.66 0.11

-5 9 4 0.63 0.09

-5 10 4 1.43 0.14

5 10 -4 1.36 0.13

5 10 -4 1.23 0.13

-5 10 -4 1.33 0.14

-5 10 4 1.13 0.12

-5 10 -4 1.12 0.12

5 10 4 1.10 0.13

5 10 4 1.29 0.13

5 10 -4 1.51 0.30

-5 10 -4 1.00 0.23

5 10 -4 1.05 0.23

-5 10 4 0.96 0.21

-5 10 -4 1.26 0.26

5 10 -4 1.14 0.14

5 10 4 1.15 0.12

-5 10 4 1.14 0.12

-5 11 -4 0.63 0.09

-5 11 -4 0.73 0.11

5 11 4 0.77 0.10

-5 11 4 0.76 0.11

5 11 -4 0.76 0.10

5 11 4 0.74 0.11

-5 11 4 0.62 0.10

5 11 -4 0.72 0.11

5 11 4 0.75 0.10

-5 11 4 0.71 0.10

-5 12 -4 1.60 0.12

5 12 -4 1.65 0.13

-5 12 4 1.60 0.14

5 12 4 1.90 0.17

5 12 4 1.54 0.14

-5 12 4 1.60 0.14

-5 12 -4 1.60 0.14

5 12 -4 1.56 0.14

5 13 4 0.08 0.06

-5 13 4 0.05 0.06

-5 13 -4 0.06 0.04

-5 13 -4 0.02 0.05

5 13 -4 0.06 0.06

-5 14 4 0.24 0.06

-5 14 -4 0.15 0.05

5 14 -4 0.24 0.06

5 15 -4 0.16 0.04

-5 15 -4 0.16 0.03

5 0 -5 -0.01 0.08

5 -1 -5 1.79 0.19

5 1 -5 1.64 0.17

5 -2 -5 5.59 0.32

5 2 -5 6.41 0.31

5 3 -5 32.54 0.71

5 -3 -5 32.17 0.77

-5 3 5 31.57 0.57

5 4 -5 12.30 0.42

5 -4 -5 11.56 0.47

-5 4 5 11.87 0.39

5 -5 -5 4.67 0.31

5 5 -5 4.86 0.34

5 5 5 4.43 0.23

5 -5 5 3.96 0.27

5 5 -5 4.79 0.25

-5 5 5 3.93 0.26

5 6 -5 21.66 0.79

-5 6 -5 19.39 0.71

-5 6 5 21.29 0.65

-5 7 -5 1.14 0.20

5 7 -5 1.03 0.20

5 7 -5 1.12 0.19

-5 7 5 1.05 0.17

5 8 5 0.83 0.09

-5 8 5 0.80 0.09

-5 8 5 0.91 0.10

5 8 5 1.00 0.11

5 8 -5 0.85 0.19

-5 8 -5 1.00 0.20

5 8 -5 0.90 0.20

-5 8 5 0.74 0.16

5 8 -5 1.04 0.11

-5 8 -5 1.24 0.12

-5 8 5 0.85 0.09

5 8 5 0.78 0.08

-5 9 5 4.91 0.23

5 9 5 4.73 0.24

-5 9 -5 5.12 0.24

5 9 -5 5.13 0.24

-5 9 5 4.73 0.40

5 9 -5 5.31 0.23

-5 9 -5 4.75 0.22

5 9 5 5.09 0.21

-5 9 5 4.79 0.21

-5 9 -5 4.85 0.49

-5 9 -5 4.54 0.43

5 9 -5 5.04 0.45

5 9 -5 5.09 0.48

-5 9 -5 4.99 0.24

5 9 -5 4.95 0.25

-5 9 5 5.05 0.22

5 9 5 5.06 0.22

5 10 -5 4.96 0.23

-5 10 -5 4.94 0.25

-5 10 5 4.73 0.24

-5 10 5 4.64 0.42

5 10 -5 4.33 0.44

-5 10 -5 4.64 0.22

5 10 5 4.70 0.25

5 10 5 4.95 0.22

5 10 -5 4.84 0.24

-5 10 5 4.58 0.22

5 10 -5 4.71 0.49

-5 10 -5 4.54 0.50

-5 10 -5 5.08 0.48

5 10 -5 4.25 0.24

-5 10 5 4.90 0.23

5 10 5 5.00 0.23

-5 11 -5 0.53 0.08

5 11 -5 0.62 0.09

5 11 5 0.69 0.10

5 11 5 0.58 0.11

-5 11 -5 0.59 0.11

-5 11 5 0.50 0.10

5 11 -5 0.48 0.10

5 11 -5 0.59 0.11

5 11 5 0.52 0.09

-5 11 5 0.50 0.09

5 12 -5 0.30 0.07

5 12 5 0.29 0.09

5 12 -5 0.24 0.09

-5 12 -5 0.33 0.09

-5 12 5 0.15 0.07

-5 12 -5 0.19 0.05

5 12 5 0.20 0.07

-5 12 5 0.13 0.06

5 13 5 0.15 0.07

-5 13 -5 0.06 0.04

-5 13 5 0.07 0.06

-5 13 -5 0.05 0.06

5 13 -5 0.10 0.06

-5 14 5 0.65 0.07

5 14 -5 0.58 0.07

-5 14 -5 0.50 0.07

5 15 -5 0.07 0.03

-5 15 -5 0.07 0.03

5 0 -6 0.07 0.09

5 1 -6 3.97 0.27

5 -1 -6 3.53 0.27

5 -2 -6 43.25 0.94

5 2 -6 43.06 0.89

5 3 -6 10.16 0.43

5 -3 -6 10.25 0.46

5 -4 -6 12.71 0.52

5 4 6 12.26 0.46

5 -4 6 11.80 0.49

5 4 -6 13.17 0.49

5 5 -6 0.65 0.11

5 -5 6 0.68 0.13

5 5 -6 0.91 0.17

5 -5 -6 0.67 0.13

-5 5 6 0.69 0.13

5 5 6 0.76 0.11

-5 6 -6 13.71 0.61

5 6 -6 14.27 0.66

-5 6 6 13.67 0.52

5 7 -6 2.05 0.28

-5 7 -6 1.94 0.26

-5 7 6 2.08 0.23

5 8 6 2.20 0.14

-5 8 6 2.08 0.13

5 8 6 2.21 0.16

-5 8 6 2.15 0.26

-5 8 6 2.11 0.15

5 8 -6 3.09 0.37

5 8 -6 2.35 0.30

-5 8 -6 2.18 0.30

5 8 -6 2.40 0.18

-5 8 -6 2.29 0.17

-5 8 -6 2.28 0.16

-5 8 6 2.38 0.14

5 8 6 2.22 0.14

5 9 6 2.91 0.16

-5 9 6 2.67 0.16

5 9 -6 2.75 0.16

-5 9 6 3.03 0.18

5 9 6 2.80 0.19

5 9 -6 2.86 0.19

-5 9 -6 3.04 0.19

-5 9 -6 2.82 0.16

-5 9 -6 2.77 0.38

5 9 -6 2.65 0.36

5 9 -6 2.89 0.35

-5 9 -6 2.93 0.36

-5 9 -6 2.56 0.18

5 9 -6 2.74 0.20

5 9 6 2.67 0.16

-5 9 6 2.83 0.17

-5 10 6 0.51 0.09

-5 10 -6 0.59 0.08

5 10 6 0.53 0.10

5 10 -6 0.69 0.09

-5 10 -6 0.46 0.09

5 10 6 0.57 0.09

-5 10 6 0.40 0.08

5 10 -6 0.53 0.10

-5 10 -6 0.49 0.18

5 10 -6 0.50 0.17

5 10 -6 0.48 0.18

-5 10 -6 0.24 0.12

-5 10 -6 0.64 0.10

5 10 -6 0.51 0.11

5 10 6 0.56 0.09

-5 10 6 0.53 0.09

5 11 -6 0.49 0.08

-5 11 6 0.49 0.10

5 11 6 0.44 0.10

5 11 -6 0.45 0.10

-5 11 -6 0.35 0.06

-5 11 -6 0.45 0.10

5 11 -6 0.37 0.10

5 11 6 0.44 0.09

-5 11 6 0.44 0.09

5 12 6 0.75 0.11

-5 12 6 0.96 0.12

5 12 -6 1.02 0.13

-5 12 -6 1.08 0.13

5 13 6 0.18 0.06

-5 13 6 0.25 0.07

-5 13 -6 0.16 0.07

5 13 -6 0.23 0.07

5 14 -6 0.03 0.04

-5 14 -6 -0.03 0.04

5 0 -7 0.12 0.11

5 1 -7 35.29 0.86

5 -1 -7 34.69 0.88

5 2 -7 79.87 1.32

5 -2 -7 83.32 1.41

5 -3 -7 8.19 0.44

5 3 -7 8.07 0.41

5 4 7 1.72 0.19

5 4 -7 1.62 0.19

5 -4 7 1.62 0.20

5 -4 -7 1.68 0.21

5 5 -7 2.72 0.24

5 5 7 2.24 0.21

5 5 -7 2.86 0.28

5 -5 -7 2.77 0.26

5 -5 7 2.31 0.24

-5 6 -7 5.13 0.39

5 6 -7 5.54 0.42

5 7 -7 12.11 0.67

-5 7 -7 11.24 0.63

-5 8 7 0.52 0.08

-5 8 7 0.55 0.08

5 8 7 0.57 0.08

5 8 7 0.50 0.08

-5 8 -7 0.58 0.17

5 8 -7 0.55 0.19

5 8 -7 0.31 0.13

-5 8 -7 0.45 0.08

-5 8 -7 0.50 0.09

5 8 -7 0.50 0.10

-5 8 7 0.45 0.07

5 8 7 0.49 0.07

-5 9 7 4.14 0.20

5 9 -7 4.22 0.19

5 9 7 3.73 0.21

5 9 7 4.11 0.19

-5 9 7 4.15 0.21

-5 9 -7 3.96 0.22

5 9 -7 4.08 0.22

5 9 -7 4.79 0.49

5 9 -7 3.95 0.41

-5 9 -7 3.64 0.41

5 9 -7 4.15 0.24

-5 9 -7 3.73 0.22

-5 9 -7 4.33 0.24

5 9 7 3.77 0.19

-5 9 7 3.86 0.19

-5 10 7 6.08 0.27

-5 10 7 5.59 0.23

5 10 -7 6.18 0.23

5 10 7 5.81 0.27

-5 10 -7 5.65 0.27

5 10 -7 5.80 0.27

5 10 -7 5.68 0.51

5 10 7 6.04 0.24

5 10 -7 5.76 0.29

-5 10 -7 5.29 0.26

-5 10 7 5.63 0.24

5 10 7 5.34 0.23

5 11 7 0.04 0.07

5 11 -7 0.06 0.07

-5 11 7 0.12 0.08

-5 11 -7 0.10 0.07

5 11 -7 0.17 0.06

5 11 -7 0.12 0.08

-5 12 7 2.34 0.16

5 12 7 2.27 0.17

-5 12 -7 2.48 0.18

5 12 -7 2.44 0.18

5 13 7 0.05 0.05

-5 13 7 0.05 0.05

-5 13 -7 0.01 0.05

5 13 -7 0.07 0.05

-5 14 -7 0.05 0.04

5 14 -7 0.03 0.04

5 0 -8 -0.04 0.11

5 1 -8 20.57 0.70

5 -1 -8 20.83 0.71

5 2 -8 39.50 0.97

5 -2 -8 40.25 1.03

5 -3 -8 17.36 0.68

5 -3 8 17.86 0.67

5 3 8 17.90 0.64

5 3 -8 16.84 0.63

5 -4 -8 7.70 0.46

5 4 8 7.40 0.42

5 -4 8 7.33 0.44

5 4 -8 7.67 0.43

5 -5 -8 5.35 0.39

5 -5 8 5.52 0.39

5 5 8 5.25 0.34

5 5 -8 5.82 0.37

5 6 -8 0.21 0.12

5 7 8 1.44 0.10

-5 7 -8 1.44 0.24

5 7 -8 1.58 0.27

5 7 -8 1.70 0.15

-5 7 -8 1.77 0.14

5 7 8 1.27 0.10

5 8 8 1.07 0.12

-5 8 8 1.00 0.10

5 8 8 1.23 0.11

-5 8 8 1.05 0.10

5 8 -8 1.22 0.26

-5 8 -8 1.04 0.22

-5 8 -8 1.17 0.14

5 8 -8 1.07 0.13

-5 8 -8 0.97 0.12

-5 8 8 0.91 0.09

5 8 8 0.91 0.09

-5 9 8 6.03 0.23

-5 9 8 6.49 0.26

5 9 8 6.30 0.28

5 9 8 6.45 0.24

5 9 -8 6.02 0.30

-5 9 -8 6.75 0.30

-5 9 -8 5.36 0.27

5 9 -8 5.97 0.51

-5 9 -8 5.81 0.52

5 9 -8 6.27 0.56

-5 10 8 1.28 0.12

-5 10 -8 1.25 0.14

5 10 8 1.17 0.13

-5 10 8 1.23 0.13

5 10 -8 1.16 0.13

5 10 -8 1.23 0.15

-5 10 -8 1.16 0.14

5 11 8 1.12 0.13

-5 11 8 1.36 0.13

-5 11 -8 1.15 0.14

5 11 -8 1.17 0.13

5 11 -8 1.08 0.14

-5 12 8 0.78 0.10

5 12 -8 0.83 0.11

5 12 8 0.79 0.10

-5 12 -8 0.89 0.11

5 13 8 0.15 0.05

5 13 -8 0.26 0.07

-5 13 -8 0.21 0.06

-5 13 8 0.26 0.06

-5 14 -8 0.05 0.04

5 14 -8 0.12 0.04

5 0 -9 0.17 0.13

5 0 9 0.17 0.12

5 1 9 26.37 0.84

5 -1 9 27.47 0.87

5 1 -9 25.61 0.83

5 -1 -9 25.78 0.85

5 -2 9 2.83 0.29

5 2 9 2.54 0.27

5 2 -9 2.68 0.28

5 -2 -9 2.71 0.30

5 -3 -9 22.85 0.83

5 -3 9 23.28 0.81

5 3 9 22.15 0.76

5 3 -9 22.58 0.78

5 4 9 6.89 0.43

5 -4 9 6.77 0.45

5 4 -9 6.67 0.43

5 -4 -9 7.41 0.48

5 5 -9 0.13 0.08

5 5 9 0.13 0.07

5 -5 -9 0.11 0.09

5 -5 9 0.26 0.10

5 6 -9 3.74 0.37

5 7 9 4.30 0.17

5 7 9 4.38 0.21

-5 7 9 4.45 0.19

-5 7 -9 4.79 0.24

5 7 -9 4.98 0.25

-5 7 -9 4.91 0.24

5 7 -9 4.61 0.44

-5 7 -9 4.04 0.41

-5 8 9 1.51 0.12

5 8 9 1.83 0.13

5 8 9 1.60 0.14

-5 8 9 1.74 0.13

5 8 -9 1.71 0.17

-5 8 -9 1.75 0.16

-5 8 -9 1.58 0.15

5 8 -9 1.77 0.30

-5 8 -9 1.68 0.29

-5 9 9 2.90 0.18

5 9 9 2.64 0.18

-5 9 9 2.79 0.16

5 9 -9 2.62 0.20

-5 9 -9 2.48 0.19

-5 9 -9 3.18 0.22

5 9 -9 2.78 0.36

5 9 -9 2.94 0.40

-5 9 -9 2.50 0.35

5 10 9 0.13 0.07

-5 10 -9 0.11 0.07

-5 10 9 0.12 0.06

5 10 -9 0.11 0.07

-5 10 -9 0.11 0.06

5 10 -9 0.34 0.10

5 11 9 0.39 0.09

-5 11 9 0.23 0.07

-5 11 -9 0.10 0.07

5 11 -9 0.19 0.08

5 11 -9 0.40 0.10

-5 12 9 1.04 0.11

5 12 9 0.95 0.10

5 12 -9 1.12 0.12

-5 12 -9 1.09 0.12

-5 13 -9 0.09 0.05

5 13 9 0.15 0.05

5 13 -9 0.16 0.06

-5 14 -9 0.09 0.04

5 14 -9 0.11 0.04

5 0 -10 0.00 0.12

5 0 10 0.01 0.12

5 -1 -10 8.01 0.52

5 -1 10 9.14 0.53

5 1 10 8.52 0.51

5 1 -10 7.82 0.49

5 -2 -10 5.32 0.43

5 2 -10 5.14 0.40

5 -2 10 4.39 0.37

5 2 10 4.45 0.37

5 -3 -10 1.88 0.26

5 3 10 2.17 0.27

5 -3 10 2.32 0.29

5 3 -10 1.78 0.24

5 -4 -10 4.94 0.42

5 4 10 4.86 0.39

5 -4 10 5.22 0.42

5 4 -10 4.99 0.40

5 -5 -10 3.30 0.34

5 5 -10 3.14 0.32

5 5 10 3.59 0.32

5 -5 10 3.76 0.37

5 6 -10 9.37 0.59

5 7 10 0.10 0.05

-5 7 10 0.11 0.05

-5 7 -10 0.15 0.12

5 7 -10 0.12 0.11

5 7 -10 0.18 0.07

-5 7 -10 0.25 0.07

-5 7 -10 0.16 0.06

-5 8 10 1.04 0.10

5 8 10 0.99 0.11

5 8 -10 1.05 0.14

-5 8 -10 1.16 0.13

-5 8 -10 0.93 0.12

5 8 -10 0.96 0.23

-5 8 -10 0.65 0.19

-5 9 10 2.97 0.17

5 9 10 2.65 0.18

-5 9 -10 2.51 0.19

-5 9 -10 3.12 0.22

5 9 -10 2.75 0.21

-5 9 -10 2.44 0.35

5 9 -10 2.33 0.33

-5 10 10 0.14 0.06

-5 10 -10 0.03 0.07

5 10 10 0.20 0.07

5 10 -10 0.02 0.08

5 10 -10 0.31 0.11

-5 10 -10 0.28 0.08

5 11 10 0.26 0.07

-5 11 10 0.32 0.08

-5 11 -10 0.20 0.08

5 11 -10 0.05 0.07

5 11 -10 0.43 0.11

-5 12 10 0.27 0.06

5 12 10 0.27 0.06

5 12 -10 0.43 0.09

-5 12 -10 0.39 0.08

-5 13 -10 -0.03 0.05

5 13 -10 0.00 0.04

5 13 10 0.08 0.04

-5 14 -10 -0.02 0.03

5 14 -10 0.01 0.03

5 0 -11 0.04 0.13

5 0 11 -0.09 0.13

5 1 11 12.73 0.65

5 -1 11 12.39 0.66

5 1 -11 13.71 0.68

5 -1 -11 13.78 0.70

5 -2 -11 4.05 0.39

5 -2 11 3.89 0.38

5 2 11 3.35 0.34

5 2 -11 4.15 0.39

5 -3 -11 10.18 0.62

5 -3 11 11.39 0.65

5 3 11 11.43 0.62

5 3 -11 10.26 0.60

5 -4 11 1.06 0.21

5 4 -11 1.05 0.21

5 4 11 0.97 0.18

5 -4 -11 0.94 0.21

5 -5 11 4.20 0.41

5 -5 -11 4.55 0.43

5 5 -11 4.08 0.38

5 6 -11 1.64 0.27

5 6 -11 1.72 0.15

-5 6 -11 1.66 0.15

-5 6 -11 1.82 0.15

-5 6 11 1.67 0.15

-5 7 11 3.74 0.17

5 7 11 3.64 0.19

5 7 -11 4.29 0.25

-5 7 -11 3.79 0.23

-5 7 -11 4.26 0.23

5 7 -11 4.04 0.44

-5 7 -11 3.21 0.38

-5 8 11 1.09 0.11

5 8 11 1.16 0.12

5 8 -11 1.06 0.14

-5 8 -11 1.41 0.15

-5 8 -11 1.01 0.13

-5 8 -11 0.89 0.22

5 8 -11 1.10 0.26

5 9 11 3.11 0.19

-5 9 11 3.17 0.18

-5 9 -11 3.45 0.23

5 9 -11 3.38 0.24

-5 9 -11 2.80 0.20

5 10 11 0.30 0.07

-5 10 11 0.23 0.06

5 10 -11 0.16 0.07

-5 10 -11 0.13 0.07

-5 10 -11 0.31 0.08

5 10 -11 0.42 0.12

5 11 11 0.72 0.09

5 11 -11 0.69 0.10

-5 11 11 0.79 0.09

-5 11 -11 0.57 0.10

-5 11 -11 0.41 0.08

5 11 -11 0.86 0.13

5 12 11 0.18 0.05

5 12 -11 0.22 0.07

-5 12 -11 0.15 0.06

-5 13 -11 0.15 0.05

5 13 -11 0.18 0.05

-5 14 -11 0.09 0.03

5 14 -11 0.11 0.03

5 0 -12 0.04 0.13

5 0 12 -0.01 0.12

5 1 -12 5.85 0.48

5 1 12 5.55 0.46

5 -1 12 5.79 0.48

5 -1 -12 6.45 0.51

5 2 12 6.44 0.50

5 -2 12 7.09 0.54

5 2 -12 6.78 0.52

5 -2 -12 7.17 0.55

5 -3 -12 3.83 0.41

5 3 12 3.98 0.39

5 -3 12 3.88 0.40

5 3 -12 3.68 0.39

5 -4 -12 2.15 0.32

5 -4 12 1.71 0.28

5 4 12 1.59 0.25

-5 -4 -12 2.44 0.32

5 4 -12 1.97 0.29

5 5 -12 1.47 0.25

5 -5 -12 1.32 0.25

5 -5 12 1.12 0.23

5 5 -12 1.85 0.15

-5 5 -12 1.74 0.15

-5 5 12 1.30 0.14

5 6 -12 1.73 0.16

-5 6 -12 1.45 0.13

-5 6 -12 1.45 0.15

5 6 -12 1.23 0.12

-5 6 12 1.61 0.16

-5 7 12 0.72 0.08

5 7 -12 0.69 0.19

-5 7 -12 0.55 0.18

-5 7 -12 0.82 0.12

5 7 -12 0.74 0.12

-5 7 -12 0.60 0.11

-5 8 12 1.42 0.11

5 8 12 1.42 0.12

-5 8 -12 1.55 0.16

5 8 -12 1.41 0.16

-5 8 -12 1.18 0.14

-5 8 -12 1.01 0.24

5 8 -12 0.98 0.23

-5 9 12 3.90 0.19

5 9 12 3.80 0.20

5 9 -12 4.29 0.27

-5 9 -12 4.45 0.25

-5 9 -12 3.38 0.22

-5 10 12 0.70 0.09

5 10 12 0.59 0.09

-5 10 -12 0.51 0.09

5 10 -12 0.48 0.10

-5 10 -12 0.59 0.10

5 10 -12 0.81 0.14

-5 11 12 0.30 0.06

5 11 12 0.35 0.06

-5 11 -12 0.29 0.07

5 11 -12 0.31 0.08

5 11 -12 0.53 0.11

-5 11 -12 0.23 0.06

5 12 12 0.30 0.05

-5 12 -12 0.30 0.07

5 12 -12 0.23 0.07

-5 13 -12 0.17 0.04

5 13 -12 0.16 0.04

5 0 -13 0.02 0.14

5 0 13 0.06 0.15

5 1 13 13.21 0.75

5 -1 -13 12.87 0.76

5 -1 13 14.27 0.79

5 1 -13 13.14 0.76

5 -2 -13 2.95 0.38

5 -2 13 2.84 0.37

5 2 13 2.68 0.34

5 2 -13 3.17 0.39

5 -3 -13 10.78 0.71

5 -3 13 10.68 0.70

5 3 13 9.84 0.64

5 3 -13 10.06 0.67

5 4 -13 0.52 0.15

5 4 13 0.49 0.16

-5 -4 -13 0.63 0.18

5 -4 -13 0.77 0.21

5 -4 13 0.46 0.17

-5 4 13 0.75 0.11

-5 4 -13 0.83 0.11

5 4 13 0.82 0.11

5 -5 13 3.56 0.42

5 -5 -13 3.54 0.41

5 5 -13 3.17 0.37

-5 5 -13 3.73 0.22

-5 5 -13 3.38 0.19

5 5 -13 3.96 0.23

-5 5 13 3.69 0.23

-5 6 -13 0.76 0.11

5 6 -13 0.93 0.13

-5 6 -13 0.75 0.12

5 6 -13 0.48 0.08

-5 6 13 0.67 0.11

5 7 -13 2.60 0.36

-5 7 -13 2.12 0.34

-5 7 -13 3.06 0.20

5 7 -13 3.33 0.23

5 7 -13 2.11 0.17

-5 7 -13 2.69 0.20

-5 8 13 0.27 0.06

-5 8 -13 0.34 0.09

5 8 -13 0.36 0.11

-5 8 -13 0.09 0.07

-5 9 13 1.73 0.12

-5 9 -13 1.86 0.17

-5 9 -13 1.47 0.15

5 9 -13 1.91 0.19

-5 10 -13 -0.14 0.09

-5 10 13 0.13 0.05

5 10 -13 -0.20 0.09

-5 10 -13 0.09 0.06

5 10 -13 0.19 0.11

-5 11 -13 0.00 0.06

5 11 -13 0.03 0.06

5 11 -13 0.07 0.08

-5 11 -13 -0.03 0.05

5 12 -13 0.03 0.05

-5 12 -13 0.04 0.05

-5 13 -13 0.08 0.03

5 13 -13 0.12 0.04

5 0 14 -0.08 0.16

5 0 -14 -0.06 0.17

5 1 -14 5.83 0.54

5 -1 14 5.59 0.52

5 1 14 6.26 0.55

5 -1 -14 5.64 0.53

5 -2 -14 8.35 0.65

5 -2 14 8.07 0.63

5 2 14 7.10 0.58

5 2 -14 8.42 0.64

5 3 -14 1.38 0.27

5 -3 -14 1.62 0.30

5 -3 14 1.12 0.25

5 3 14 0.97 0.22

-5 -3 -14 1.29 0.27

5 3 14 1.59 0.15

-5 3 14 1.91 0.16

-5 3 -14 1.79 0.15

5 4 -14 -0.06 0.14

5 4 14 -0.17 0.14

5 -4 14 -0.02 0.15

5 -4 -14 -0.09 0.15

-5 -4 -14 -0.13 0.13

5 4 -14 0.00 0.05

5 4 14 0.01 0.06

-5 4 -14 -0.02 0.06

-5 4 14 0.05 0.07

5 -5 -14 5.48 0.54

5 5 -14 5.24 0.51

-5 5 -14 4.99 0.23

5 5 -14 5.92 0.28

-5 5 -14 5.57 0.28

-5 5 14 5.28 0.29

-5 6 -14 2.82 0.21

-5 6 -14 2.85 0.19

-5 6 14 2.82 0.22

5 6 -14 2.47 0.19

5 7 -14 2.67 0.22

-5 7 -14 2.45 0.20

5 7 -14 1.99 0.17

-5 7 -14 2.52 0.18

5 7 -14 2.35 0.36

-5 8 -14 1.75 0.17

-5 8 -14 2.01 0.17

-5 9 -14 1.59 0.16

-5 9 -14 2.07 0.17

5 9 -14 2.19 0.20

-5 10 -14 0.16 0.07

5 10 -14 0.05 0.07

-5 10 -14 0.11 0.06

5 10 -14 0.30 0.11

5 11 -14 -0.05 0.07

-5 11 -14 -0.04 0.06

5 11 -14 0.04 0.07

-5 11 -14 0.03 0.04

5 12 -14 0.06 0.04

-5 12 -14 0.08 0.05

-5 13 -14 0.09 0.03

5 13 -14 0.13 0.03

5 0 -15 0.09 0.14

5 0 15 -0.07 0.15

5 -1 15 6.87 0.61

5 1 15 7.19 0.61

5 -1 -15 7.21 0.63

5 1 -15 7.25 0.63

5 2 -15 0.65 0.21

5 -2 -15 1.05 0.27

5 2 15 0.71 0.21

5 -2 15 0.59 0.19

5 2 -15 0.88 0.10

5 2 15 0.96 0.12

5 -3 15 8.59 0.68

5 -3 -15 8.35 0.69

5 3 15 7.66 0.63

-5 -3 -15 8.31 0.66

5 3 -15 7.54 0.63

5 3 -15 9.10 0.31

5 3 15 8.97 0.35

-5 3 15 9.15 0.36

-5 3 -15 9.25 0.34

5 -4 -15 6.35 0.60

5 4 -15 5.95 0.56

-5 -4 -15 6.42 0.59

5 -4 15 6.44 0.62

5 4 -15 6.92 0.29

-5 4 -15 6.71 0.30

-5 4 15 7.29 0.33

5 4 15 7.26 0.33

5 5 -15 0.41 0.16

5 -5 -15 0.54 0.17

-5 5 -15 0.58 0.09

5 5 -15 0.66 0.11

-5 5 15 0.45 0.10

-5 5 -15 0.55 0.11

-5 6 -15 1.04 0.12

-5 6 -15 1.11 0.14

5 6 -15 1.15 0.14

5 6 -15 1.06 0.13

-5 6 15 1.08 0.15

5 7 -15 1.34 0.16

-5 7 -15 1.34 0.14

5 7 -15 1.00 0.12

-5 7 -15 1.10 0.14

-5 8 -15 1.78 0.16

5 8 -15 2.02 0.19

5 8 -15 1.15 0.13

-5 8 -15 1.47 0.16

-5 9 -15 1.34 0.14

-5 9 -15 1.06 0.13

5 9 -15 1.44 0.16

-5 10 -15 0.25 0.08

5 10 -15 0.20 0.08

5 10 -15 0.47 0.11

-5 10 -15 0.31 0.08

5 11 -15 0.00 0.05

-5 11 -15 0.07 0.05

5 11 -15 0.11 0.07

-5 11 -15 0.02 0.04

-5 12 -15 0.04 0.03

5 12 -15 0.10 0.04

5 0 -16 -0.14 0.18

5 0 16 0.04 0.14

5 0 16 -0.01 0.05

5 1 -16 0.16 0.16

5 -1 -16 0.20 0.15

5 1 16 -0.12 0.17

5 -1 16 -0.14 0.18

5 1 -16 0.12 0.05

5 1 16 0.12 0.06

5 -2 16 0.46 0.21

5 -2 -16 0.56 0.25

5 2 16 0.46 0.21

5 2 -16 0.42 0.20

-5 -2 -16 0.44 0.20

5 2 -16 0.61 0.09

-5 2 16 0.74 0.12

5 2 16 0.60 0.11

5 3 -16 0.23 0.15

-5 -3 -16 0.42 0.20

5 -3 -16 0.30 0.17

5 -3 16 0.36 0.15

5 3 -16 0.47 0.09

-5 3 -16 0.45 0.10

5 3 16 0.44 0.10

-5 3 16 0.55 0.11

5 -4 -16 9.27 0.76

-5 -4 -16 9.75 0.74

5 4 -16 8.31 0.71

5 4 -16 9.72 0.36

-5 4 -16 9.81 0.37

-5 4 16 10.38 0.41

-5 5 -16 0.13 0.13

-5 5 -16 0.17 0.06

5 5 -16 0.21 0.08

-5 5 16 0.22 0.10

-5 5 -16 0.11 0.07

-5 6 -16 0.19 0.07

5 6 -16 0.14 0.08

5 6 -16 0.15 0.07

-5 6 16 0.17 0.09

-5 6 -16 0.14 0.08

5 7 -16 0.42 0.11

-5 7 -16 0.42 0.09

5 7 -16 0.35 0.08

-5 7 -16 0.39 0.10

5 8 -16 1.79 0.19

-5 8 -16 1.53 0.16

-5 8 -16 1.68 0.15

-5 9 -16 -0.12 0.07

5 9 -16 0.04 0.08

-5 10 -16 0.39 0.08

5 10 -16 0.48 0.09

5 10 -16 0.44 0.09

-5 10 -16 0.33 0.07

5 11 -16 0.09 0.05

-5 11 -16 0.05 0.04

-5 11 -16 0.04 0.03

5 11 -16 0.12 0.06

-5 12 -16 0.01 0.03

5 12 -16 0.03 0.03

5 0 -17 -0.10 0.17

5 0 17 0.13 0.14

5 0 17 0.02 0.05

5 1 17 5.41 0.59

5 -1 17 5.39 0.59

5 -1 -17 5.71 0.61

-5 -1 -17 5.82 0.61

5 1 -17 5.48 0.59

5 1 -17 5.50 0.24

5 1 17 5.46 0.27

5 2 -17 0.81 0.25

5 -2 17 1.00 0.27

5 -2 -17 1.00 0.30

-5 -2 -17 0.80 0.27

5 2 -17 1.09 0.12

-5 2 -17 1.23 0.15

5 2 17 1.09 0.14

-5 2 17 1.05 0.15

-5 -3 -17 1.48 0.32

5 3 -17 1.34 0.31

5 -3 -17 1.56 0.34

5 3 -17 1.53 0.14

5 3 17 1.51 0.16

-5 3 17 1.51 0.17

-5 3 -17 1.66 0.16

5 4 -17 1.52 0.32

-5 -4 -17 2.13 0.38

5 -4 -17 1.76 0.33

-5 4 -17 1.60 0.32

5 4 -17 2.04 0.17

-5 4 -17 1.92 0.18

-5 4 17 1.85 0.19

-5 5 -17 1.24 0.15

5 5 -17 1.22 0.14

5 5 -17 1.23 0.14

-5 5 17 1.51 0.18

5 6 -17 0.01 0.08

-5 6 -17 0.12 0.07

5 6 -17 0.05 0.07

-5 6 -17 0.01 0.08

-5 6 17 0.18 0.10

5 7 -17 0.35 0.10

-5 7 -17 0.37 0.08

-5 7 -17 0.61 0.12

5 7 -17 0.44 0.09

-5 8 -17 0.45 0.09

5 8 -17 0.56 0.12

-5 8 -17 0.45 0.11

5 8 -17 0.31 0.07

5 9 -17 0.85 0.12

-5 9 -17 0.55 0.10

5 10 -17 0.08 0.05

-5 10 -17 0.22 0.06

-5 10 -17 0.08 0.05

5 11 -17 0.01 0.04

-5 11 -17 0.03 0.04

-5 12 -17 0.12 0.03

5 12 -17 0.15 0.03

5 0 -18 0.08 0.16

-5 0 -18 -0.14 0.18

5 0 18 0.01 0.05

-5 -1 -18 -0.17 0.19

5 1 -18 0.19 0.19

-5 1 -18 0.04 0.16

5 -1 -18 -0.08 0.16

5 1 18 0.05 0.06

-5 1 18 0.10 0.07

5 2 -18 0.72 0.25

5 -2 -18 0.52 0.19

-5 -2 -18 0.46 0.18

-5 2 -18 0.57 0.21

5 2 -18 0.63 0.09

-5 2 -18 0.67 0.12

-5 2 18 0.77 0.13

5 2 18 0.82 0.12

-5 -3 -18 0.27 0.17

5 3 -18 0.17 0.16

5 -3 -18 0.32 0.16

-5 3 -18 0.38 0.21

5 3 -18 0.46 0.10

-5 3 -18 0.23 0.09

5 3 18 0.12 0.07

-5 3 18 0.34 0.11

5 4 -18 0.76 0.12

-5 4 18 0.86 0.14

-5 4 -18 0.61 0.12

5 5 -18 0.97 0.13

-5 5 -18 1.21 0.15

5 5 -18 1.04 0.13

-5 5 18 1.17 0.17

-5 6 -18 0.78 0.10

5 6 -18 0.83 0.12

-5 6 -18 0.67 0.12

5 6 -18 0.78 0.12

-5 6 18 0.95 0.15

5 7 -18 0.10 0.08

-5 7 -18 0.11 0.06

-5 7 -18 0.12 0.09

5 7 -18 0.17 0.08

5 8 -18 0.68 0.11

5 8 -18 0.51 0.09

-5 8 -18 0.50 0.11

-5 9 -18 0.03 0.05

5 9 -18 0.06 0.06

-5 10 -18 0.09 0.05

5 10 -18 0.02 0.04

5 10 -18 0.17 0.07

-5 10 -18 0.02 0.04

-5 11 -18 0.06 0.03

5 11 -18 0.15 0.04

5 0 19 -0.03 0.07

-5 1 -19 0.69 0.12

-5 1 19 0.47 0.11

5 1 19 0.52 0.10

5 2 -19 0.25 0.08

-5 2 19 0.27 0.10

-5 2 -19 0.27 0.09

5 2 19 0.19 0.08

5 3 -19 0.65 0.10

-5 3 19 0.59 0.13

-5 3 -19 0.63 0.11

5 4 -19 0.26 0.08

-5 4 -19 0.29 0.10

-5 4 19 0.37 0.12

5 5 -19 0.29 0.09

5 5 -19 0.19 0.08

-5 5 19 0.45 0.13

-5 5 -19 0.19 0.09

5 6 -19 0.13 0.07

-5 6 -19 0.07 0.08

5 6 -19 0.18 0.08

-5 7 -19 0.58 0.09

5 7 -19 0.44 0.09

5 7 -19 0.60 0.10

-5 7 -19 0.70 0.12

5 8 -19 0.36 0.09

5 8 -19 0.24 0.07

-5 8 -19 0.25 0.08

-5 9 -19 0.16 0.06

5 9 -19 0.16 0.06

-5 10 -19 0.08 0.04

5 10 -19 0.01 0.04

5 10 -19 0.03 0.04

-5 10 -19 -0.06 0.04

5 11 -19 0.06 0.03

-5 11 -19 0.03 0.03

-5 0 20 0.09 0.09

5 0 20 0.03 0.06

-5 1 -20 0.17 0.09

5 1 20 0.11 0.07

-5 1 20 0.02 0.08

-5 2 -20 0.11 0.08

-5 2 20 -0.07 0.10

5 2 20 0.05 0.07

5 3 -20 1.41 0.14

-5 3 -20 1.45 0.16

-5 3 20 1.62 0.18

5 4 -20 0.12 0.06

5 4 -20 0.08 0.08

-5 4 20 0.28 0.12

-5 4 -20 0.20 0.09

5 5 -20 0.75 0.11

5 5 -20 0.89 0.12

-5 5 -20 0.92 0.13

5 6 -20 0.74 0.11

-5 6 -20 0.65 0.12

5 6 -20 0.73 0.11

5 7 -20 0.00 0.06

-5 7 -20 0.05 0.07

5 7 -20 -0.01 0.05

5 8 -20 -0.05 0.05

-5 8 -20 0.02 0.06

5 8 -20 -0.05 0.04

5 9 -20 0.08 0.05

-5 9 -20 0.04 0.04

5 10 -20 0.23 0.04

-5 10 -20 0.31 0.05

-5 10 -20 0.17 0.04

-5 0 21 -0.04 0.08

5 0 21 0.00 0.05

-5 0 -21 0.11 0.08

-5 -1 21 0.04 0.08

-5 1 -21 0.13 0.08

-5 1 21 0.03 0.08

-5 -1 -21 0.12 0.07

5 1 21 0.09 0.06

-5 -2 21 0.34 0.09

-5 2 -21 0.39 0.11

-5 2 21 0.40 0.12

-5 3 -21 0.38 0.10

-5 3 21 0.36 0.11

5 4 -21 1.09 0.12

5 4 -21 1.00 0.13

-5 4 21 1.50 0.18

-5 4 -21 1.16 0.14

5 5 -21 0.17 0.06

-5 5 -21 0.17 0.08

5 5 -21 0.19 0.08

-5 6 -21 0.86 0.13

5 6 -21 0.60 0.10

5 6 -21 0.61 0.10

5 7 -21 0.27 0.07

-5 7 -21 0.22 0.07

5 7 -21 0.20 0.06

5 8 -21 0.62 0.08

-5 8 -21 0.61 0.08

5 8 -21 0.46 0.06

-5 9 -21 0.08 0.04

5 9 -21 0.01 0.03

-5 9 -21 -0.05 0.04

5 9 -21 0.01 0.04

-5 0 22 -0.03 0.07

-5 0 -22 0.10 0.07

-5 -1 22 0.21 0.08

-5 -1 -22 0.26 0.07

-5 1 -22 0.26 0.09

-5 1 22 0.34 0.10

-5 2 22 0.41 0.11

-5 -2 -22 0.42 0.08

-5 2 -22 0.36 0.10

-5 -2 22 0.33 0.08

-5 -3 22 0.10 0.07

5 3 -22 -0.05 0.09

-5 3 -22 0.03 0.07

-5 3 22 0.16 0.10

5 4 -22 0.52 0.08

5 4 -22 0.79 0.12

-5 -4 22 0.56 0.08

-5 4 22 0.89 0.15

-5 4 -22 0.82 0.12

5 5 -22 0.21 0.06

-5 5 -22 0.24 0.08

5 5 -22 0.18 0.07

5 6 -22 0.26 0.06

5 6 -22 0.29 0.07

-5 6 -22 0.30 0.08

5 7 -22 0.43 0.07

-5 7 -22 0.48 0.08

5 7 -22 0.44 0.07

5 8 -22 0.02 0.03

-5 8 -22 -0.01 0.04

-5 9 -22 0.08 0.03

5 9 -22 0.01 0.03

-5 0 23 -0.04 0.07

-5 0 -23 -0.01 0.05

-5 -1 -23 0.02 0.05

-5 -1 23 -0.02 0.06

-5 1 -23 0.06 0.06

-5 1 23 0.02 0.07

-5 -2 23 0.07 0.06

-5 -2 -23 0.17 0.06

-5 2 23 0.08 0.08

-5 2 -23 0.08 0.07

5 3 -23 0.18 0.07

-5 -3 23 0.31 0.07

-5 3 23 0.42 0.11

-5 3 -23 0.29 0.09

5 4 -23 0.98 0.12

-5 4 -23 1.03 0.12

-5 5 -23 0.06 0.06

5 5 -23 -0.01 0.05

5 6 -23 0.05 0.04

5 6 -23 0.10 0.05

-5 7 -23 0.07 0.05

5 7 -23 0.13 0.04

5 7 -23 0.06 0.04

-5 8 -23 0.36 0.06

-5 0 -24 0.00 0.06

-5 0 24 0.04 0.07

-5 1 24 0.09 0.06

-5 -1 24 0.09 0.06

-5 1 -24 0.02 0.05

-5 -1 -24 0.07 0.05

-5 2 -24 0.05 0.06

-5 2 24 0.04 0.07

-5 -2 24 0.09 0.06

5 2 -24 -0.04 0.07

5 3 -24 -0.01 0.07

-5 3 24 0.12 0.07

-5 -3 24 0.08 0.05

-5 3 -24 0.02 0.06

5 4 -24 0.30 0.07

-5 4 -24 0.18 0.06

-5 5 -24 0.06 0.05

5 5 -24 0.08 0.05

-5 6 -24 0.48 0.07

5 6 -24 0.28 0.05

-5 7 -24 0.11 0.04

5 0 -25 0.04 0.06

-5 0 25 0.00 0.05

-5 0 -25 0.01 0.04

-5 -1 -25 -0.03 0.04

-5 -1 25 -0.02 0.04

-5 1 25 0.05 0.06

-5 1 -25 -0.06 0.05

5 1 -25 -0.01 0.06

-5 -2 25 0.08 0.04

-5 2 -25 0.04 0.05

-5 2 25 0.16 0.07

5 2 -25 0.06 0.05

-5 3 -25 -0.01 0.05

5 3 -25 0.03 0.05

5 4 -25 -0.02 0.04

-5 4 -25 0.06 0.05

-5 5 -25 0.08 0.05

5 5 -25 0.03 0.04

-5 6 -25 0.11 0.04

-5 0 26 0.00 0.04

-5 0 -26 -0.05 0.04

5 0 -26 -0.06 0.05

5 -1 -26 -0.02 0.04

5 1 -26 -0.03 0.05

-5 1 -26 0.00 0.04

-5 -1 26 0.04 0.04

5 -2 -26 0.13 0.05

-5 2 -26 0.08 0.04

5 2 -26 0.10 0.05

5 -3 -26 0.20 0.04

-5 3 -26 0.24 0.05

5 3 -26 0.24 0.05

5 4 -26 0.05 0.04

-5 4 -26 0.07 0.04

-5 5 -26 0.21 0.04

5 0 -27 -0.01 0.03

5 -1 -27 0.11 0.04

5 1 -27 0.11 0.04

-5 2 -27 0.09 0.03

5 2 -27 0.10 0.04

5 3 -27 0.06 0.03

6 0 0 0.04 0.06

6 1 0 3.54 0.13

6 1 0 3.50 0.19

6 -1 0 3.44 0.21

6 -2 0 45.80 0.80

6 2 0 45.49 0.67

-6 2 0 42.52 0.49

6 2 0 43.41 0.62

6 2 0 45.03 0.53

6 -3 0 2.23 0.15

6 -3 0 2.29 0.19

-6 3 0 2.32 0.13

6 3 0 2.50 0.17

6 3 0 2.29 0.14

-6 4 0 0.41 0.09

6 4 0 0.36 0.10

6 -4 0 0.24 0.06

6 4 0 0.24 0.06

6 4 0 0.20 0.07

6 5 0 4.60 0.30

6 5 0 4.62 0.19

-6 5 0 4.55 0.26

6 5 0 4.73 0.28

-6 6 0 15.91 0.55

6 6 0 18.21 0.65

-6 6 0 17.16 0.63

6 6 0 17.13 0.58

-6 7 0 0.22 0.09

6 7 0 0.25 0.10

6 7 0 0.52 0.13

-6 7 0 0.34 0.12

6 8 0 0.22 0.10

6 8 0 0.35 0.14

-6 8 0 0.36 0.11

-6 8 0 0.25 0.14

6 9 0 2.08 0.30

-6 9 0 1.96 0.26

-6 9 0 1.95 0.14

6 9 0 1.97 0.13

6 9 0 1.60 0.24

6 9 0 1.91 0.15

-6 9 0 1.80 0.28

-6 9 0 2.02 0.15

-6 9 0 2.17 0.15

6 9 0 2.07 0.14

6 10 0 4.47 0.44

6 10 0 4.06 0.23

6 10 0 3.82 0.18

-6 10 0 4.08 0.44

-6 10 0 4.30 0.23

6 10 0 4.02 0.39

6 10 0 4.09 0.21

-6 10 0 4.25 0.21

-6 11 0 1.11 0.13

6 11 0 1.26 0.14

6 11 0 1.22 0.11

-6 11 0 1.14 0.13

6 11 0 0.99 0.12

6 12 0 0.85 0.12

6 12 0 0.67 0.10

-6 12 0 0.75 0.11

-6 12 0 0.55 0.09

6 13 0 0.22 0.07

-6 13 0 0.09 0.05

6 13 0 0.11 0.06

-6 13 0 0.12 0.06

-6 14 0 0.10 0.04

-6 14 0 0.06 0.05

6 14 0 -0.01 0.04

6 0 1 495.83 3.35

6 0 1 499.65 3.41

6 0 -1 495.80 3.38

6 0 -1 503.80 2.63

6 1 1 39.68 0.67

6 1 -1 41.56 0.67

6 -1 -1 41.35 0.72

6 -1 1 40.67 0.68

6 -1 1 39.95 0.73

6 1 -1 40.71 0.48

6 2 -1 432.74 2.91

6 -2 -1 435.79 3.16

6 2 1 433.15 2.99

6 2 -1 417.08 2.78

-6 2 1 416.33 2.47

6 -2 1 436.34 3.03

6 2 1 435.32 2.77

6 2 1 436.02 2.62

-6 3 1 23.13 0.41

6 3 1 23.72 0.51

6 3 -1 24.09 0.46

6 -3 -1 23.16 0.59

-6 3 -1 23.88 0.46

6 3 -1 24.49 0.53

6 3 1 23.00 0.50

6 -3 1 23.07 0.52

6 3 -1 21.99 0.46

6 3 1 23.31 0.45

-6 4 1 211.67 1.81

6 4 1 216.13 2.02

-6 4 1 224.42 2.03

-6 4 -1 217.41 1.90

6 4 -1 213.36 1.91

6 4 1 213.82 1.80

6 -4 -1 211.79 2.09

6 -4 -1 208.74 1.79

6 4 -1 223.82 2.07

6 4 1 206.56 1.86

-6 5 1 7.97 0.33

-6 5 1 8.10 0.38

6 5 -1 7.42 0.27

6 5 -1 7.87 0.35

6 5 1 8.66 0.40

-6 5 -1 7.64 0.35

6 5 -1 7.94 0.39

6 5 1 8.06 0.36

-6 6 1 46.26 0.92

6 6 1 49.93 1.06

6 6 -1 52.25 1.12

-6 6 -1 45.51 0.96

-6 6 1 48.90 1.06

6 6 -1 47.46 0.99

6 6 1 46.90 0.97

6 7 -1 9.06 0.51

-6 7 1 8.02 0.42

6 7 1 8.76 0.49

-6 7 -1 8.67 0.50

-6 7 -1 8.36 0.45

6 7 1 8.40 0.44

-6 7 1 8.58 0.48

6 7 -1 8.78 0.46

-6 8 1 1.42 0.23

-6 8 1 1.48 0.20

6 8 -1 1.11 0.21

6 8 1 1.19 0.21

-6 8 -1 1.04 0.20

-6 8 -1 1.29 0.20

6 8 -1 1.04 0.19

6 8 1 1.23 0.19

6 9 -1 5.02 0.44

-6 9 -1 4.68 0.40

6 9 1 5.00 0.43

-6 9 1 4.76 0.21

6 9 -1 4.61 0.19

-6 9 -1 4.93 0.21

6 9 1 4.97 0.24

6 9 -1 5.10 0.24

6 9 1 4.66 0.20

-6 9 -1 4.64 0.44

6 9 -1 5.12 0.42

-6 9 -1 4.67 0.23

-6 9 1 4.76 0.23

6 9 1 4.87 0.40

-6 9 1 5.00 0.44

6 9 1 5.00 0.22

-6 9 -1 5.01 0.22

-6 9 1 5.10 0.22

6 9 -1 4.82 0.22

-6 10 -1 2.28 0.30

6 10 -1 2.35 0.33

-6 10 1 1.63 0.27

6 10 -1 2.19 0.14

-6 10 1 2.02 0.15

-6 10 -1 2.56 0.35

6 10 -1 1.88 0.28

-6 10 1 2.10 0.17

-6 10 -1 2.35 0.17

6 10 1 1.89 0.27

6 10 1 1.95 0.14

6 10 1 1.81 0.16

6 10 -1 2.61 0.18

6 10 1 1.91 0.15

-6 10 -1 2.76 0.18

6 10 -1 2.40 0.17

-6 10 1 1.93 0.15

-6 11 -1 0.50 0.10

6 11 1 0.44 0.08

6 11 1 0.39 0.08

6 11 -1 0.40 0.09

6 11 1 0.46 0.10

-6 11 1 0.36 0.08

6 11 -1 0.48 0.07

6 11 -1 0.27 0.08

-6 11 -1 0.27 0.07

-6 11 1 0.44 0.10

6 12 -1 0.28 0.09

6 12 1 0.36 0.10

6 12 -1 0.16 0.07

-6 12 -1 0.07 0.06

-6 12 -1 0.30 0.08

-6 12 1 0.11 0.07

-6 12 1 0.28 0.08

6 12 1 0.27 0.08

6 13 -1 0.16 0.07

-6 13 -1 0.05 0.05

6 13 1 0.29 0.08

-6 13 1 0.10 0.06

6 13 1 0.07 0.06

-6 13 -1 0.05 0.06

6 13 -1 0.09 0.06

-6 13 1 0.12 0.06

-6 14 -1 0.08 0.04

-6 14 1 0.12 0.05

6 14 -1 0.02 0.04

-6 14 1 0.04 0.04

6 14 1 0.06 0.04

-6 14 -1 0.08 0.05

6 0 2 27.69 0.58

6 0 -2 26.18 0.55

-6 1 2 1.74 0.07

6 1 -2 1.73 0.14

6 -1 -2 1.75 0.15

6 -1 2 1.67 0.15

6 1 2 1.62 0.14

-6 2 2 39.71 0.54

6 2 2 38.95 0.70

6 -2 -2 39.29 0.75

6 -2 2 41.38 0.72

6 2 -2 40.75 0.61

6 3 2 9.95 0.29

-6 3 2 9.21 0.25

6 -3 -2 9.12 0.37

6 3 -2 9.72 0.34

-6 3 2 9.58 0.31

6 3 2 9.31 0.33

6 3 -2 9.01 0.29

6 3 2 9.59 0.32

6 4 2 8.94 0.33

-6 4 2 8.62 0.29

6 4 -2 9.09 0.26

6 -4 -2 8.68 0.38

6 4 2 8.74 0.35

6 4 -2 9.27 0.34

-6 4 2 9.18 0.35

-6 4 -2 9.31 0.35

6 4 -2 9.95 0.40

6 5 -2 0.87 0.15

6 5 2 0.78 0.13

6 5 2 0.84 0.12

-6 5 2 0.81 0.12

6 -5 -2 0.86 0.13

-6 5 2 0.87 0.14

6 5 -2 0.77 0.12

-6 5 -2 0.94 0.14

6 6 2 3.26 0.25

-6 6 2 3.05 0.26

6 6 2 2.88 0.26

-6 6 2 3.07 0.23

6 6 -2 2.94 0.26

-6 6 -2 3.05 0.26

6 6 -2 3.39 0.29

6 7 -2 2.17 0.24

-6 7 2 2.05 0.24

6 7 2 2.20 0.23

-6 7 2 2.23 0.22

6 7 2 2.50 0.26

-6 7 -2 2.05 0.23

6 7 -2 2.37 0.27

-6 8 -2 0.31 0.11

6 8 -2 0.50 0.16

-6 8 2 0.44 0.14

6 8 2 0.40 0.14

6 8 -2 0.37 0.12

-6 8 -2 0.37 0.14

6 8 2 0.48 0.14

-6 9 2 0.31 0.07

6 9 2 0.24 0.06

-6 9 2 0.27 0.07

6 9 2 0.24 0.06

6 9 -2 0.15 0.06

-6 9 2 0.14 0.06

6 9 2 0.20 0.06

-6 9 -2 0.18 0.06

6 9 -2 0.18 0.05

-6 9 -2 0.14 0.06

6 9 -2 0.12 0.06

6 9 2 0.35 0.13

-6 9 -2 0.17 0.11

6 9 -2 0.18 0.13

6 9 -2 0.23 0.13

-6 9 2 0.20 0.12

-6 9 -2 0.21 0.14

6 9 -2 0.16 0.06

-6 9 -2 0.11 0.05

6 10 2 1.91 0.14

-6 10 2 2.05 0.15

-6 10 -2 2.13 0.34

6 10 2 1.79 0.16

6 10 -2 2.03 0.16

6 10 2 2.05 0.28

-6 10 -2 1.72 0.15

6 10 -2 1.81 0.27

6 10 -2 1.80 0.12

-6 10 2 2.26 0.17

-6 10 -2 1.48 0.24

-6 10 2 1.74 0.28

6 10 -2 2.21 0.33

6 10 -2 1.68 0.15

6 10 -2 1.96 0.16

-6 10 2 2.09 0.16

6 10 2 2.07 0.15

-6 10 -2 2.21 0.17

-6 11 -2 0.51 0.09

6 11 -2 0.45 0.09

6 11 2 0.58 0.11

6 11 2 0.48 0.08

-6 11 2 0.36 0.09

-6 11 -2 0.53 0.10

6 11 -2 0.32 0.08

6 11 -2 0.55 0.07

6 11 2 0.38 0.09

-6 11 2 0.45 0.09

6 12 -2 0.51 0.10

6 12 2 0.34 0.10

-6 12 -2 0.28 0.08

6 12 -2 0.31 0.08

-6 12 2 0.15 0.07

-6 12 -2 0.20 0.05

6 12 2 0.32 0.08

-6 12 2 0.29 0.08

6 13 -2 0.22 0.07

6 13 2 0.30 0.08

-6 13 2 0.22 0.07

-6 13 -2 0.16 0.05

6 13 2 0.15 0.06

-6 13 -2 0.17 0.07

-6 13 2 0.07 0.05

6 13 -2 0.01 0.05

-6 14 -2 0.05 0.04

-6 14 2 0.06 0.05

-6 14 -2 -0.03 0.04

6 14 -2 0.01 0.04

6 0 -3 62.94 0.93

6 0 3 60.53 0.93

6 -1 3 46.21 0.80

6 -1 -3 49.36 0.84

6 1 -3 49.14 0.80

6 1 3 44.36 0.79

6 2 3 294.30 2.41

6 2 -3 293.84 2.33

-6 2 3 293.71 1.96

6 -3 -3 95.89 1.30

6 3 -3 94.52 1.12

6 3 -3 101.17 1.22

-6 3 3 100.48 1.07

6 -4 -3 77.98 1.21

6 4 -3 78.84 0.95

-6 4 -3 81.26 1.13

6 4 -3 84.64 1.23

6 4 3 80.85 1.05

-6 4 3 82.02 1.09

6 5 3 5.21 0.31

6 -5 -3 4.23 0.28

6 5 -3 4.74 0.32

6 5 -3 3.95 0.28

-6 5 -3 4.44 0.29

-6 5 3 4.83 0.29

6 5 3 4.80 0.28

-6 6 -3 52.96 1.10

6 6 -3 51.66 1.07

6 6 -3 54.27 1.19

-6 6 3 51.55 1.06

6 6 3 52.37 1.01

6 7 -3 2.30 0.26

-6 7 -3 2.58 0.27

6 7 -3 2.72 0.29

6 7 3 2.22 0.23

-6 7 3 2.29 0.25

6 8 -3 26.64 0.98

-6 8 -3 24.85 0.89

-6 8 -3 27.31 1.01

6 8 3 25.36 0.45

6 8 -3 25.49 0.89

-6 8 -3 26.04 0.45

6 8 3 24.60 0.82

-6 8 3 24.56 0.86

-6 8 3 26.21 0.52

6 8 3 25.52 0.53

6 9 3 2.99 0.16

6 9 3 3.04 0.19

6 9 -3 2.92 0.14

-6 9 3 2.95 0.33

6 9 -3 2.99 0.18

-6 9 -3 2.89 0.18

-6 9 -3 2.91 0.18

6 9 -3 3.10 0.34

6 9 3 2.94 0.31

-6 9 3 3.12 0.19

-6 9 3 3.04 0.17

6 9 -3 3.24 0.19

-6 9 -3 2.74 0.32

6 9 -3 3.41 0.38

-6 9 -3 3.04 0.37

6 9 -3 2.81 0.18

-6 9 -3 2.91 0.18

-6 9 3 3.30 0.18

6 9 3 3.06 0.17

6 10 3 9.74 0.30

-6 10 3 9.81 0.31

6 10 -3 9.22 0.26

6 10 -3 9.67 0.33

-6 10 3 10.25 0.35

-6 10 -3 10.15 0.70

6 10 3 10.09 0.36

-6 10 -3 9.39 0.32

-6 10 3 9.47 0.61

6 10 -3 9.30 0.61

6 10 -3 11.55 0.73

-6 10 -3 9.12 0.61

6 10 -3 9.22 0.33

-6 10 -3 10.09 0.34

6 10 3 9.97 0.32

-6 10 3 10.27 0.33

6 10 -3 9.73 0.33

6 11 -3 0.99 0.12

6 11 3 1.00 0.13

-6 11 -3 0.99 0.11

-6 11 3 0.97 0.12

6 11 3 1.02 0.11

-6 11 -3 1.09 0.13

6 11 3 0.97 0.11

6 11 -3 1.09 0.13

-6 11 3 1.02 0.12

6 12 -3 0.83 0.10

6 12 3 0.96 0.13

6 12 -3 0.69 0.10

-6 12 -3 0.64 0.09

-6 12 3 0.69 0.10

-6 12 -3 0.72 0.10

-6 12 3 0.72 0.10

6 12 3 0.69 0.10

6 13 -3 0.22 0.06

6 13 3 0.15 0.08

-6 13 -3 0.13 0.05

-6 13 -3 0.08 0.06

6 13 -3 0.10 0.06

-6 13 3 0.17 0.07

-6 13 3 0.10 0.05

6 13 3 0.11 0.06

-6 14 3 0.38 0.06

-6 14 -3 0.31 0.05

-6 14 -3 0.38 0.06

6 14 -3 0.31 0.06

6 15 -3 0.01 0.02

6 0 -4 5.25 0.28

6 1 -4 4.29 0.25

6 -1 -4 4.50 0.27

6 -2 -4 27.02 0.67

-6 2 4 26.33 0.42

6 -3 -4 8.31 0.37

6 3 -4 8.82 0.35

-6 3 4 9.45 0.30

6 4 -4 3.39 0.21

6 -4 -4 3.39 0.26

6 4 -4 3.49 0.26

-6 4 4 3.59 0.21

6 -5 -4 0.03 0.07

6 5 -4 0.04 0.08

-6 5 4 0.00 0.06

-6 5 -4 0.04 0.07

6 5 4 0.10 0.08

6 6 -4 1.24 0.18

-6 6 -4 1.30 0.19

6 6 -4 1.49 0.22

-6 6 4 1.54 0.19

6 6 4 1.61 0.20

-6 7 -4 1.06 0.18

6 7 -4 1.05 0.20

-6 7 4 1.16 0.18

6 7 -4 1.12 0.18

6 7 4 1.08 0.17

-6 8 -4 0.49 0.14

6 8 -4 0.57 0.16

6 8 -4 0.37 0.13

-6 8 4 0.45 0.14

6 8 4 0.49 0.14

-6 8 4 0.50 0.07

6 8 4 0.51 0.07

-6 8 4 0.44 0.08

6 8 4 0.46 0.07

6 8 -4 0.57 0.08

-6 8 4 0.57 0.08

6 8 4 0.39 0.08

-6 8 -4 0.53 0.08

6 9 4 2.39 0.18

-6 9 4 2.58 0.16

-6 9 -4 2.01 0.15

6 9 -4 1.93 0.14

-6 9 4 2.44 0.17

-6 9 4 2.20 0.28

6 9 -4 2.19 0.16

6 9 4 2.29 0.15

6 9 -4 2.00 0.28

-6 9 -4 2.12 0.16

-6 9 -4 2.26 0.32

-6 9 -4 2.08 0.30

6 9 -4 2.15 0.31

-6 9 -4 2.13 0.16

6 9 -4 2.03 0.17

6 9 4 2.38 0.15

-6 9 4 2.50 0.16

6 10 4 4.58 0.21

-6 10 4 4.73 0.22

-6 10 -4 4.82 0.24

6 10 -4 4.65 0.24

-6 10 4 5.17 0.25

6 10 4 4.82 0.26

-6 10 -4 4.26 0.22

-6 10 4 4.93 0.44

6 10 -4 4.63 0.23

6 10 -4 4.79 0.44

-6 10 -4 4.94 0.50

-6 10 -4 4.21 0.42

6 10 -4 5.20 0.50

6 10 -4 3.94 0.23

6 10 4 4.59 0.22

-6 10 4 5.02 0.23

6 11 -4 0.63 0.10

6 11 -4 0.72 0.10

-6 11 -4 0.70 0.10

6 11 4 0.67 0.11

-6 11 -4 0.74 0.11

-6 11 4 0.57 0.10

6 11 4 0.66 0.09

6 11 4 0.64 0.10

-6 11 4 0.69 0.10

6 12 4 0.31 0.09

-6 12 -4 0.19 0.07

-6 12 4 0.21 0.07

6 12 -4 0.34 0.08

-6 12 -4 0.23 0.06

6 12 -4 0.21 0.07

-6 12 4 0.28 0.07

6 12 4 0.20 0.07

-6 13 -4 0.04 0.06

6 13 4 0.12 0.07

-6 13 4 0.06 0.06

-6 13 -4 0.00 0.04

6 13 -4 0.00 0.05

-6 14 -4 0.16 0.03

-6 14 4 0.30 0.06

-6 14 -4 0.17 0.05

6 14 -4 0.20 0.05

-6 15 -4 0.02 0.02

6 15 -4 0.04 0.03

6 0 -5 1.73 0.19

6 -1 -5 38.85 0.84

6 1 -5 38.37 0.80

6 -2 -5 121.02 1.58

6 2 -5 119.34 1.49

6 3 -5 39.96 0.82

6 -3 -5 39.65 0.89

-6 3 5 41.55 0.65

6 4 -5 19.52 0.55

6 4 -5 20.00 0.61

-6 4 5 20.05 0.51

6 -5 5 1.86 0.19

6 5 5 1.84 0.16

6 5 -5 1.76 0.16

6 5 -5 2.36 0.25

6 -5 -5 1.92 0.21

-6 5 5 1.67 0.17

-6 6 5 0.59 0.13

6 6 -5 0.87 0.18

-6 6 -5 0.74 0.16

6 7 -5 9.95 0.53

-6 7 -5 9.40 0.54

-6 7 5 9.34 0.47

-6 8 -5 0.72 0.09

6 8 5 0.88 0.09

-6 8 5 0.93 0.10

-6 8 5 0.89 0.10

6 8 5 0.91 0.11

6 8 -5 0.70 0.09

-6 8 -5 0.50 0.16

6 8 -5 0.69 0.19

-6 8 5 0.88 0.17

6 8 -5 0.51 0.16

-6 8 -5 0.71 0.10

6 8 -5 0.66 0.10

6 8 5 0.98 0.09

-6 8 5 1.00 0.09

-6 9 5 3.46 0.18

6 9 -5 3.45 0.20

6 9 -5 3.31 0.18

6 9 5 3.37 0.21

-6 9 5 3.53 0.20

-6 9 5 3.34 0.34

6 9 -5 3.21 0.35

-6 9 -5 3.37 0.20

-6 9 -5 3.34 0.19

6 9 5 3.37 0.17

6 9 -5 3.69 0.42

-6 9 -5 3.05 0.40

-6 9 -5 2.99 0.35

-6 9 -5 3.35 0.21

6 9 -5 3.36 0.21

-6 9 5 3.53 0.19

6 9 5 3.25 0.18

6 10 -5 0.60 0.10

6 10 5 0.83 0.10

-6 10 5 0.84 0.11

-6 10 -5 0.42 0.10

6 10 -5 0.60 0.11

-6 10 5 0.74 0.10

6 10 5 0.58 0.11

-6 10 -5 0.56 0.09

-6 10 -5 0.27 0.16

-6 10 -5 0.53 0.18

6 10 -5 0.55 0.19

-6 10 5 0.84 0.20

6 10 -5 0.62 0.18

-6 10 -5 0.48 0.09

6 10 -5 0.62 0.12

-6 10 5 0.64 0.10

6 10 5 0.48 0.09

6 11 -5 2.87 0.18

-6 11 5 3.22 0.20

6 11 5 3.00 0.21

-6 11 -5 2.84 0.17

6 11 -5 2.62 0.19

6 11 -5 2.89 0.19

-6 11 5 3.19 0.19

6 11 5 2.93 0.18

-6 11 -5 2.91 0.20

-6 12 5 -0.04 0.08

6 12 -5 0.12 0.05

6 12 5 0.12 0.08

-6 12 -5 0.14 0.07

-6 12 -5 -0.06 0.05

6 12 -5 0.07 0.07

-6 12 5 0.04 0.06

6 12 5 0.02 0.05

6 13 -5 0.32 0.08

-6 13 -5 0.37 0.08

-6 13 5 0.43 0.08

-6 13 -5 0.38 0.06

6 13 5 0.41 0.08

6 14 5 0.28 0.06

-6 14 5 0.21 0.05

6 14 -5 0.13 0.05

-6 14 -5 0.12 0.05

6 15 -5 -0.02 0.02

6 0 -6 76.64 1.26

6 -1 -6 3.53 0.28

6 1 -6 3.51 0.27

6 -2 -6 0.95 0.16

6 2 -6 1.06 0.15

6 3 -6 12.74 0.49

6 -3 -6 12.56 0.53

6 4 -6 0.45 0.10

6 -4 -6 0.53 0.13

6 5 -6 0.07 0.07

-6 5 6 0.07 0.07

6 5 -6 -0.05 0.10

6 -5 -6 0.08 0.07

6 5 6 0.01 0.05

6 -5 6 -0.01 0.07

-6 6 -6 0.49 0.13

6 6 -6 0.80 0.18

-6 6 6 0.66 0.14

6 7 -6 2.94 0.34

-6 7 -6 2.53 0.29

-6 7 6 2.62 0.26

-6 8 6 0.13 0.04

6 8 6 0.19 0.07

6 8 6 0.25 0.06

-6 8 6 0.19 0.06

-6 8 -6 0.14 0.05

-6 8 6 0.29 0.13

-6 8 -6 0.18 0.12

6 8 -6 0.29 0.15

6 8 -6 0.30 0.13

6 8 -6 0.22 0.07

-6 8 -6 0.22 0.07

-6 8 -6 0.33 0.08

-6 8 6 0.20 0.05

6 8 6 0.17 0.05

-6 9 6 0.16 0.08

-6 9 6 0.15 0.06

6 9 -6 0.02 0.04

6 9 6 0.09 0.07

-6 9 -6 0.05 0.05

6 9 6 0.21 0.07

-6 9 -6 0.00 0.06

6 9 -6 -0.01 0.06

-6 9 -6 0.16 0.13

6 9 -6 0.00 0.09

6 9 -6 0.18 0.14

-6 9 -6 0.06 0.12

-6 9 -6 0.13 0.07

6 9 -6 0.08 0.07

-6 9 6 0.03 0.05

6 9 6 0.11 0.06

6 10 -6 2.89 0.17

6 10 -6 2.80 0.19

6 10 6 2.52 0.19

-6 10 6 2.49 0.16

-6 10 -6 2.83 0.19

6 10 6 2.50 0.16

-6 10 6 2.48 0.18

6 10 -6 2.52 0.34

-6 10 -6 2.51 0.16

-6 10 -6 2.66 0.35

-6 10 -6 2.86 0.41

-6 10 -6 2.43 0.18

6 10 -6 2.29 0.19

-6 10 6 2.46 0.17

6 10 6 2.21 0.16

-6 11 -6 0.17 0.06

6 11 -6 0.18 0.06

-6 11 6 0.18 0.07

6 11 6 0.21 0.08

-6 11 -6 0.21 0.08

6 11 -6 0.16 0.08

6 11 -6 0.28 0.09

-6 11 6 0.16 0.07

6 11 6 0.26 0.08

6 12 6 0.16 0.08

-6 12 6 0.05 0.06

-6 12 -6 0.11 0.05

-6 12 -6 0.22 0.08

6 12 -6 0.17 0.08

6 13 6 0.18 0.07

-6 13 6 0.23 0.07

6 13 -6 0.18 0.06

-6 13 -6 0.15 0.06

-6 14 6 0.20 0.05

-6 14 -6 0.08 0.04

6 14 -6 0.10 0.04

6 0 -7 17.58 0.62

6 1 -7 35.89 0.89

6 -1 -7 36.68 0.92

6 -2 -7 1.07 0.17

6 2 -7 1.25 0.18

6 -3 -7 42.72 1.03

6 3 -7 42.95 0.97

6 4 -7 22.22 0.69

6 4 7 22.02 0.68

6 -4 7 21.82 0.73

6 -4 -7 20.96 0.73

6 -5 -7 4.76 0.35

6 5 -7 5.20 0.33

6 5 7 5.84 0.34

6 5 -7 5.19 0.38

6 -5 7 5.69 0.38

-6 6 -7 1.72 0.24

6 6 -7 2.32 0.28

-6 7 -7 6.89 0.49

6 7 -7 7.37 0.53

6 8 7 2.64 0.15

-6 8 7 2.58 0.15

6 8 7 2.61 0.17

-6 8 7 2.67 0.16

6 8 -7 2.49 0.30

-6 8 -7 2.16 0.30

6 8 -7 2.95 0.37

-6 8 -7 2.54 0.18

6 8 -7 2.59 0.19

-6 8 -7 2.74 0.18

-6 8 7 2.67 0.15

6 8 7 2.44 0.14

6 9 -7 0.77 0.11

-6 9 7 0.90 0.10

-6 9 7 0.96 0.11

6 9 7 0.94 0.12

6 9 7 1.07 0.11

-6 9 -7 0.82 0.11

6 9 -7 0.67 0.19

-6 9 -7 1.08 0.24

6 9 -7 0.75 0.21

-6 9 -7 0.87 0.12

6 9 -7 0.99 0.13

-6 9 7 0.76 0.09

6 9 7 0.88 0.10

6 10 -7 9.24 0.29

-6 10 7 9.64 0.33

6 10 7 8.80 0.34

-6 10 -7 9.09 0.34

6 10 -7 9.20 0.34

-6 10 -7 8.90 0.34

6 10 -7 8.41 0.35

-6 10 7 8.26 0.28

6 10 7 7.84 0.27

-6 11 7 3.42 0.20

6 11 -7 3.12 0.16

6 11 7 3.07 0.21

-6 11 -7 3.03 0.20

6 11 -7 2.89 0.20

6 11 -7 2.76 0.20

6 12 7 0.55 0.10

-6 12 7 0.57 0.09

-6 12 -7 0.71 0.11

6 12 -7 0.60 0.10

-6 13 -7 0.22 0.06

-6 13 7 0.26 0.06

6 13 -7 0.16 0.06

6 13 7 0.19 0.06

6 14 -7 0.38 0.06

-6 14 -7 0.40 0.06

6 0 -8 11.54 0.55

6 1 -8 5.87 0.39

6 -1 -8 6.20 0.41

6 -2 -8 9.65 0.51

6 2 -8 9.43 0.49

6 -3 -8 3.92 0.33

6 3 -8 4.09 0.33

6 -4 -8 5.13 0.39

6 -4 8 5.13 0.38

6 4 8 5.21 0.36

6 4 -8 5.25 0.36

6 -5 8 0.76 0.16

6 5 8 0.83 0.15

6 -5 -8 0.74 0.16

6 5 -8 0.80 0.15

6 6 -8 0.09 0.11

6 7 8 0.67 0.07

-6 7 -8 0.62 0.16

6 7 -8 0.76 0.20

6 7 -8 0.69 0.10

-6 7 -8 0.83 0.10

-6 7 -8 0.75 0.10

-6 8 8 0.33 0.07

-6 8 8 0.26 0.05

6 8 8 0.45 0.07

6 8 8 0.35 0.07

-6 8 -8 0.50 0.10

6 8 -8 0.41 0.09

-6 8 -8 0.49 0.09

-6 8 -8 0.18 0.12

6 8 -8 0.36 0.17

6 8 -8 0.34 0.14

-6 9 8 0.52 0.08

6 9 8 0.22 0.07

-6 9 8 0.41 0.08

-6 9 -8 0.31 0.09

6 9 -8 0.42 0.10

-6 9 -8 0.63 0.19

6 9 -8 0.54 0.20

6 9 -8 0.57 0.18

-6 10 -8 0.59 0.10

-6 10 8 0.77 0.11

6 10 8 0.82 0.11

6 10 -8 0.61 0.11

6 10 -8 0.85 0.14

-6 10 -8 0.79 0.12

-6 11 8 0.99 0.12

6 11 8 0.88 0.12

6 11 -8 0.87 0.12

-6 11 -8 0.91 0.12

6 11 -8 0.80 0.13

6 12 8 0.86 0.11

-6 12 8 0.74 0.10

6 12 -8 0.78 0.11

-6 12 -8 0.87 0.11

6 13 8 0.06 0.05

-6 13 8 0.07 0.05

-6 13 -8 -0.02 0.05

6 13 -8 0.00 0.05

6 14 -8 0.04 0.04

-6 14 -8 0.03 0.03

6 0 -9 5.49 0.40

6 1 -9 33.33 0.98

6 -1 -9 35.07 1.01

6 2 -9 0.42 0.14

6 -2 -9 0.27 0.14

6 -3 -9 41.06 1.14

6 -3 9 41.58 1.12

6 3 9 40.49 1.06

6 3 -9 40.39 1.08

6 4 -9 20.03 0.75

6 -4 9 21.72 0.82

6 4 9 21.21 0.76

6 -4 -9 21.06 0.82

6 5 -9 4.59 0.36

6 -5 -9 4.27 0.38

6 -5 9 4.94 0.40

6 5 9 4.96 0.37

6 6 -9 1.20 0.23

-6 7 9 3.88 0.18

6 7 9 3.87 0.20

6 7 -9 3.88 0.23

-6 7 -9 3.62 0.20

-6 7 -9 3.82 0.22

6 7 -9 3.56 0.40

-6 7 -9 3.28 0.37

6 8 9 16.77 0.45

-6 8 9 17.93 0.42

-6 8 -9 16.71 0.47

6 8 -9 18.68 0.52

-6 8 -9 18.88 0.49

-6 8 -9 16.07 0.85

6 8 -9 18.56 0.94

6 8 -9 17.36 0.84

-6 9 9 0.62 0.08

-6 9 9 0.44 0.08

6 9 9 0.37 0.08

6 9 -9 0.39 0.10

-6 9 -9 0.55 0.12

-6 9 -9 0.37 0.09

-6 9 -9 0.73 0.22

6 9 -9 0.48 0.18

6 9 -9 0.42 0.18

-6 10 9 3.68 0.20

6 10 9 3.38 0.21

6 10 -9 3.24 0.21

-6 10 -9 3.39 0.21

-6 10 -9 3.14 0.21

6 10 -9 3.28 0.23

-6 11 9 1.06 0.12

6 11 9 1.00 0.12

-6 11 -9 0.95 0.12

6 11 -9 1.03 0.12

6 11 -9 0.99 0.14

-6 11 -9 0.65 0.10

-6 12 -9 0.12 0.07

6 12 9 0.05 0.05

-6 12 9 0.06 0.06

6 12 -9 0.10 0.07

6 13 9 0.22 0.06

-6 13 9 0.27 0.06

-6 13 -9 0.22 0.06

6 13 -9 0.24 0.06

-6 14 -9 0.01 0.03

6 14 -9 0.01 0.03

6 0 10 7.97 0.51

6 0 -10 7.44 0.50

6 1 -10 0.31 0.13

6 -1 10 0.27 0.14

6 1 10 0.23 0.13

6 -1 -10 0.34 0.16

6 2 -10 1.38 0.23

6 -2 10 1.28 0.22

6 2 10 1.14 0.20

6 -2 -10 1.67 0.26

6 -3 -10 7.00 0.50

6 -3 10 6.70 0.48

6 3 10 6.63 0.45

6 3 -10 6.42 0.45

6 4 10 7.63 0.49

6 -4 10 7.65 0.52

6 -4 -10 7.58 0.53

6 4 -10 7.40 0.49

6 -5 -10 0.71 0.17

6 5 10 0.61 0.12

6 5 -10 0.73 0.17

6 -5 10 0.70 0.18

-6 6 -10 1.02 0.12

6 6 -10 0.98 0.11

6 6 -10 0.91 0.20

-6 7 10 2.42 0.14

6 7 10 2.28 0.16

6 7 -10 2.48 0.19

-6 7 -10 2.40 0.18

-6 7 -10 2.41 0.17

6 7 -10 2.43 0.34

-6 7 -10 1.93 0.29

6 8 10 0.92 0.11

-6 8 10 1.07 0.11

6 8 -10 1.09 0.15

-6 8 -10 1.20 0.14

-6 8 -10 1.07 0.13

-6 8 -10 0.87 0.22

6 8 -10 1.08 0.26

6 9 10 0.27 0.07

-6 9 10 0.38 0.08

-6 9 -10 0.69 0.13

-6 9 -10 0.42 0.10

6 9 -10 0.62 0.12

-6 9 -10 0.11 0.10

6 9 -10 0.73 0.21

6 10 -10 0.37 0.09

6 10 10 0.44 0.09

-6 10 10 0.52 0.09

-6 10 -10 0.33 0.09

-6 10 -10 0.42 0.09

6 10 -10 0.48 0.12

-6 11 10 0.94 0.10

6 11 10 0.86 0.11

6 11 -10 0.90 0.12

-6 11 -10 0.82 0.11

-6 11 -10 0.69 0.10

6 11 -10 0.83 0.13

6 12 10 0.28 0.06

-6 12 10 0.29 0.06

-6 12 -10 0.23 0.07

6 12 -10 0.34 0.08

-6 13 -10 0.01 0.04

6 13 10 0.04 0.04

-6 14 -10 0.04 0.03

6 14 -10 0.07 0.03

6 0 11 12.89 0.67

6 0 -11 14.11 0.72

6 1 -11 26.21 0.97

6 -1 11 27.87 1.00

6 1 11 26.51 0.95

6 -1 -11 27.88 1.01

6 -2 -11 26.09 0.99

6 -2 11 27.45 0.99

6 2 11 26.19 0.95

6 2 -11 25.73 0.96

6 3 11 27.83 0.99

6 -3 -11 29.82 1.08

6 -3 11 28.07 1.03

6 3 -11 28.37 1.01

6 -4 11 5.81 0.48

6 -4 -11 5.70 0.47

6 4 11 5.20 0.42

6 4 -11 5.05 0.42

6 5 -11 4.41 0.40

6 -5 -11 3.97 0.40

6 -5 11 3.95 0.40

6 6 -11 5.75 0.48

-6 6 -11 5.56 0.24

6 6 -11 6.17 0.28

-6 6 -11 5.87 0.27

-6 6 11 5.31 0.27

-6 7 11 5.06 0.20

6 7 11 4.80 0.23

6 7 -11 5.48 0.29

-6 7 -11 5.36 0.26

-6 7 -11 5.26 0.27

6 7 -11 5.50 0.51

-6 8 11 7.13 0.25

6 8 11 6.91 0.28

-6 8 -11 6.94 0.31

6 8 -11 7.42 0.34

-6 8 -11 7.70 0.32

6 8 -11 7.15 0.61

-6 9 11 1.26 0.12

6 9 11 1.19 0.13

-6 9 -11 1.03 0.13

6 9 -11 1.15 0.16

-6 9 -11 1.28 0.15

-6 10 11 1.58 0.13

6 10 11 1.54 0.14

-6 10 -11 1.44 0.15

6 10 -11 1.44 0.15

6 10 -11 1.53 0.17

-6 10 -11 1.15 0.14

-6 11 11 0.11 0.06

-6 11 -11 0.05 0.07

6 11 11 0.08 0.05

6 11 -11 0.03 0.07

6 11 -11 0.11 0.09

-6 11 -11 -0.04 0.06

6 12 11 0.79 0.08

-6 12 11 0.81 0.08

6 12 -11 0.84 0.10

-6 12 -11 0.83 0.10

6 13 -11 0.28 0.06

-6 13 -11 0.15 0.04

6 0 12 2.22 0.32

6 0 -12 2.51 0.34

6 1 -12 1.46 0.27

6 -1 -12 1.20 0.25

6 -1 12 1.40 0.26

6 1 12 1.13 0.22

6 -2 -12 1.70 0.29

6 -2 12 1.58 0.28

6 2 12 1.57 0.25

6 2 -12 1.78 0.29

6 -3 -12 9.70 0.65

6 3 12 8.43 0.57

6 -3 12 9.04 0.62

6 3 -12 8.88 0.60

6 -4 -12 3.42 0.40

6 -4 12 3.36 0.39

6 4 12 3.42 0.37

6 4 -12 3.58 0.38

6 5 -12 0.67 0.17

6 -5 -12 0.77 0.20

6 -5 12 0.54 0.17

-6 5 -12 0.79 0.11

6 5 -12 0.80 0.10

-6 5 12 0.76 0.11

6 6 -12 0.32 0.13

6 6 -12 0.35 0.08

-6 6 -12 0.33 0.07

6 6 -12 0.21 0.06

-6 6 -12 0.40 0.09

-6 6 12 0.33 0.09

-6 7 -12 1.30 0.26

6 7 -12 1.65 0.29

-6 7 -12 1.86 0.16

6 7 -12 1.96 0.18

-6 7 -12 1.71 0.16

-6 8 12 0.01 0.04

6 8 12 -0.07 0.06

6 8 -12 0.07 0.11

-6 8 -12 0.08 0.13

6 8 -12 0.10 0.08

-6 8 -12 0.07 0.07

-6 8 -12 0.09 0.07

-6 9 12 0.46 0.08

6 9 12 0.44 0.08

6 9 -12 0.57 0.12

-6 9 -12 0.44 0.10

-6 9 -12 0.63 0.12

-6 10 12 0.14 0.05

6 10 12 0.09 0.05

6 10 -12 -0.01 0.08

-6 10 -12 -0.07 0.08

6 10 -12 0.23 0.10

-6 10 -12 0.14 0.07

-6 11 12 0.20 0.05

6 11 12 0.16 0.05

-6 11 -12 0.10 0.06

6 11 -12 0.10 0.06

-6 11 -12 0.07 0.05

6 11 -12 0.16 0.08

-6 12 -12 0.16 0.06

6 12 12 0.13 0.04

6 12 -12 0.16 0.06

6 13 -12 0.00 0.03

-6 13 -12 -0.01 0.03

6 0 13 82.54 1.91

6 0 -13 83.04 1.94

6 -1 13 24.96 1.05

6 1 13 23.63 1.01

6 1 -13 24.81 1.05

6 -1 -13 25.93 1.08

6 -2 13 11.23 0.71

6 2 13 11.01 0.69

6 2 -13 11.15 0.70

6 -2 -13 11.53 0.72

6 -3 -13 9.02 0.65

6 -3 13 7.63 0.59

6 3 13 7.15 0.56

6 3 -13 8.68 0.62

6 -4 -13 19.59 0.97

6 4 -13 18.95 0.93

-6 -4 -13 21.25 0.96

6 -4 13 19.92 0.97

6 4 13 20.74 0.95

-6 4 -13 21.78 0.52

6 4 -13 22.74 0.51

-6 4 13 22.18 0.54

6 -5 13 6.55 0.57

6 5 -13 6.07 0.51

6 -5 -13 6.16 0.55

-6 5 -13 6.12 0.25

6 5 -13 6.77 0.30

-6 5 -13 6.37 0.29

-6 5 13 6.82 0.31

6 6 -13 3.79 0.42

6 6 -13 4.37 0.25

-6 6 -13 4.19 0.24

-6 6 -13 3.79 0.21

-6 6 13 3.80 0.24

6 6 -13 3.23 0.20

-6 7 -13 0.51 0.17

6 7 -13 0.55 0.18

-6 7 -13 0.63 0.10

6 7 -13 0.62 0.12

-6 7 -13 0.63 0.11

-6 8 -13 0.09 0.08

6 8 -13 0.01 0.09

-6 8 -13 0.08 0.08

-6 9 13 3.10 0.16

6 9 -13 3.34 0.24

-6 9 -13 2.65 0.20

6 10 13 1.60 0.12

-6 10 13 1.70 0.12

-6 10 -13 1.41 0.14

6 10 -13 1.50 0.14

6 10 -13 1.75 0.18

-6 10 -13 1.34 0.14

6 11 13 -0.02 0.04

-6 11 -13 0.03 0.06

6 11 -13 -0.08 0.07

-6 11 -13 -0.07 0.06

6 11 -13 0.03 0.07

-6 12 -13 0.20 0.06

6 12 -13 0.09 0.05

-6 13 -13 0.22 0.04

6 13 -13 0.24 0.05

6 0 -14 0.73 0.21

6 0 14 0.71 0.21

6 1 -14 2.04 0.33

6 -1 14 2.23 0.34

6 1 14 1.84 0.31

6 -1 -14 2.55 0.38

6 -2 -14 6.92 0.60

6 -2 14 6.68 0.58

6 2 14 6.63 0.57

6 2 -14 6.27 0.56

6 -3 14 0.09 0.12

6 3 14 0.18 0.11

6 -3 -14 0.33 0.14

6 3 -14 0.25 0.12

6 3 -14 0.37 0.08

-6 3 14 0.38 0.09

6 3 14 0.33 0.07

-6 3 -14 0.40 0.09

6 4 -14 0.78 0.22

6 -4 14 0.27 0.14

6 4 14 0.56 0.18

6 -4 -14 0.36 0.15

-6 -4 -14 0.54 0.18

6 4 -14 0.67 0.10

-6 4 14 0.67 0.11

-6 4 -14 0.67 0.10

6 -5 -14 0.39 0.15

6 -5 14 0.26 0.11

6 5 -14 0.39 0.15

6 5 -14 0.57 0.11

-6 5 -14 0.45 0.08

-6 5 -14 0.51 0.10

-6 5 14 0.55 0.11

6 6 -14 0.28 0.15

6 6 -14 0.28 0.09

-6 6 -14 0.34 0.08

6 6 -14 0.28 0.07

-6 6 14 0.27 0.08

-6 6 -14 0.31 0.09

6 7 -14 0.03 0.10

-6 7 -14 0.05 0.10

-6 7 -14 0.00 0.06

6 7 -14 0.04 0.08

6 7 -14 0.03 0.05

-6 7 -14 -0.05 0.09

-6 8 -14 1.67 0.16

-6 8 -14 1.29 0.15

6 8 -14 1.84 0.19

-6 9 -14 0.34 0.09

6 9 -14 0.51 0.12

6 10 -14 0.88 0.11

-6 10 -14 0.84 0.11

-6 10 -14 0.79 0.11

6 10 -14 1.11 0.14

-6 11 -14 0.03 0.05

6 11 -14 0.02 0.05

-6 11 -14 -0.07 0.06

6 11 -14 0.00 0.06

-6 12 -14 0.06 0.04

6 12 -14 0.02 0.04

-6 13 -14 0.13 0.03

6 13 -14 0.22 0.04

6 0 -15 7.62 0.65

6 0 15 7.06 0.61

6 -1 15 20.93 1.06

6 1 15 20.64 1.04

6 1 -15 20.73 1.06

6 -1 -15 22.87 1.11

6 2 -15 0.75 0.21

6 -2 -15 1.18 0.27

6 -2 15 0.95 0.25

6 2 15 0.94 0.24

6 2 -15 1.32 0.12

6 2 15 1.13 0.12

6 3 -15 0.84 0.23

6 3 15 0.96 0.24

6 -3 -15 0.87 0.24

6 -3 15 1.06 0.24

-6 -3 -15 1.24 0.27

6 3 -15 1.30 0.13

6 3 15 1.45 0.15

-6 3 15 1.47 0.15

-6 3 -15 1.20 0.13

6 -4 15 7.18 0.64

6 4 15 7.49 0.63

6 -4 -15 7.11 0.64

-6 -4 -15 7.01 0.61

6 4 -15 6.43 0.59

-6 4 -15 7.76 0.32

6 4 -15 7.69 0.31

-6 4 15 8.31 0.35

6 5 -15 2.28 0.35

6 -5 -15 2.33 0.37

6 5 -15 3.00 0.21

-6 5 -15 2.88 0.21

-6 5 -15 2.70 0.17

-6 5 15 3.02 0.22

6 6 -15 0.39 0.16

6 6 -15 0.59 0.11

-6 6 -15 0.59 0.10

-6 6 15 0.49 0.10

6 6 -15 0.45 0.09

-6 6 -15 0.64 0.12

6 7 -15 0.51 0.11

-6 7 -15 0.50 0.09

6 7 -15 0.43 0.09

-6 7 -15 0.49 0.10

-6 8 -15 1.19 0.13

6 8 -15 1.41 0.17

-6 8 -15 1.02 0.14

-6 9 -15 0.10 0.07

6 9 -15 0.18 0.09

-6 10 -15 1.65 0.14

6 10 -15 1.43 0.13

-6 10 -15 1.54 0.14

6 10 -15 1.89 0.17

-6 11 -15 0.35 0.07

6 11 -15 0.31 0.07

6 11 -15 0.36 0.08

-6 11 -15 0.17 0.05

-6 12 -15 0.15 0.04

6 12 -15 0.15 0.04

6 0 16 6.06 0.59

6 0 -16 5.40 0.57

6 -1 -16 0.33 0.19

6 -1 16 0.41 0.18

6 1 16 0.34 0.16

6 1 -16 0.57 0.22

6 1 -16 0.57 0.08

6 1 16 0.53 0.09

6 -2 -16 2.37 0.39

6 2 16 2.14 0.36

6 2 -16 2.30 0.38

6 -2 16 2.34 0.38

6 2 -16 2.59 0.17

6 2 16 2.66 0.19

-6 2 -16 2.87 0.19

6 3 -16 0.36 0.21

6 -3 -16 0.58 0.22

6 3 16 0.65 0.21

6 -3 16 0.47 0.18

-6 -3 -16 0.45 0.18

6 3 -16 0.62 0.09

-6 3 16 0.67 0.12

6 3 16 0.82 0.12

-6 3 -16 0.67 0.11

-6 -4 -16 1.40 0.30

6 -4 -16 1.34 0.32

6 -4 16 0.90 0.25

6 4 -16 1.08 0.26

6 4 -16 1.42 0.15

-6 4 -16 1.45 0.15

-6 4 16 1.26 0.16

6 5 -16 0.06 0.07

-6 5 16 0.12 0.09

-6 5 -16 0.08 0.07

-6 6 -16 0.04 0.05

6 6 -16 0.01 0.09

-6 6 16 0.01 0.08

-6 6 -16 0.00 0.08

6 6 -16 0.03 0.06

-6 7 -16 0.18 0.07

6 7 -16 0.13 0.08

6 7 -16 0.22 0.07

-6 7 -16 0.19 0.09

-6 8 -16 0.22 0.07

6 8 -16 0.33 0.10

-6 8 -16 0.38 0.10

-6 9 -16 0.21 0.08

6 9 -16 0.19 0.09

6 10 -16 0.01 0.06

-6 10 -16 0.06 0.05

6 10 -16 0.11 0.08

-6 10 -16 0.03 0.05

6 11 -16 0.02 0.04

-6 11 -16 0.02 0.04

6 11 -16 0.10 0.06

-6 11 -16 -0.01 0.04

-6 12 -16 0.00 0.03

6 12 -16 0.02 0.03

6 0 -17 4.79 0.56

6 0 17 4.57 0.54

6 0 17 4.85 0.24

6 -1 17 9.19 0.77

6 1 -17 8.60 0.74

6 -1 -17 8.76 0.76

6 1 17 8.08 0.72

-6 -1 -17 9.54 0.77

6 1 -17 9.72 0.32

6 1 17 9.69 0.36

6 -2 17 43.00 1.67

-6 -2 -17 41.93 1.62

6 -2 -17 39.76 1.63

6 2 17 39.59 1.57

6 2 -17 40.40 1.61

6 2 -17 40.77 0.70

-6 2 17 44.62 0.83

6 2 17 43.77 0.80

-6 2 -17 43.29 0.77

6 3 -17 0.75 0.24

6 -3 17 0.33 0.16

6 -3 -17 0.56 0.21

-6 -3 -17 0.72 0.22

6 3 -17 0.62 0.10

-6 3 17 0.81 0.14

6 3 17 0.55 0.10

-6 3 -17 0.58 0.11

6 -4 -17 4.06 0.52

6 4 -17 3.65 0.48

-6 4 -17 4.55 0.27

6 4 -17 4.54 0.25

-6 4 17 4.64 0.28

6 5 -17 0.84 0.13

6 5 -17 0.79 0.12

-6 5 17 0.81 0.14

-6 5 -17 0.83 0.13

-6 6 -17 4.27 0.26

6 6 -17 3.96 0.25

-6 6 -17 3.45 0.20

6 6 -17 3.41 0.22

-6 6 17 3.86 0.27

-6 7 -17 0.48 0.09

6 7 -17 0.39 0.10

-6 7 -17 0.50 0.12

6 7 -17 0.39 0.09

-6 8 -17 2.70 0.20

6 8 -17 3.09 0.22

6 9 -17 0.08 0.08

-6 9 -17 0.02 0.06

6 10 -17 0.00 0.05

-6 10 -17 0.02 0.05

-6 10 -17 0.00 0.05

6 10 -17 0.10 0.07

-6 11 -17 0.14 0.04

6 11 -17 0.13 0.04

-6 11 -17 0.05 0.03

6 11 -17 0.10 0.05

6 0 -18 0.90 0.26

6 0 18 0.79 0.26

-6 0 -18 0.53 0.22

6 0 18 0.77 0.11

6 -1 -18 0.01 0.16

6 1 -18 0.08 0.13

6 -1 18 0.34 0.18

-6 1 -18 0.27 0.17

-6 -1 -18 0.34 0.18

6 1 -18 0.36 0.07

6 1 18 0.26 0.08

-6 1 -18 0.29 0.08

6 2 -18 2.66 0.43

6 -2 -18 2.58 0.43

-6 2 -18 2.98 0.46

-6 -2 -18 2.79 0.44

6 2 -18 2.99 0.19

-6 2 18 3.22 0.23

-6 2 -18 2.99 0.21

6 2 18 3.12 0.22

-6 -3 -18 0.95 0.26

6 -3 -18 0.93 0.28

6 3 -18 1.08 0.27

-6 3 -18 0.82 0.26

6 3 -18 1.11 0.13

-6 3 -18 1.07 0.14

-6 3 18 1.10 0.15

6 4 -18 0.89 0.13

-6 4 -18 0.72 0.13

-6 4 18 0.93 0.15

6 5 -18 -0.04 0.08

6 5 -18 -0.04 0.07

-6 5 -18 -0.04 0.09

-6 5 18 0.04 0.08

6 6 -18 0.32 0.10

-6 6 -18 0.21 0.08

-6 6 18 0.32 0.11

6 6 -18 0.32 0.09

-6 7 -18 0.01 0.05

6 7 -18 -0.02 0.08

6 7 -18 0.04 0.05

-6 7 -18 0.04 0.07

6 8 -18 0.81 0.12

6 8 -18 0.78 0.10

-6 8 -18 0.78 0.12

-6 9 -18 0.00 0.05

6 9 -18 0.19 0.08

6 10 -18 0.07 0.04

-6 10 -18 0.05 0.04

6 10 -18 0.12 0.06

-6 10 -18 0.02 0.04

-6 11 -18 0.01 0.03

6 11 -18 0.02 0.03

-6 1 19 0.43 0.10

6 1 19 0.58 0.10

-6 1 -19 0.73 0.12

6 2 -19 5.89 0.27

-6 2 19 6.17 0.32

6 2 19 6.14 0.30

-6 2 -19 5.88 0.29

6 3 -19 0.96 0.12

-6 3 -19 1.14 0.15

-6 3 19 1.30 0.17

-6 4 -19 1.52 0.16

6 4 -19 1.33 0.14

-6 4 19 1.69 0.18

6 5 -19 0.78 0.11

6 5 -19 0.90 0.12

-6 5 -19 0.74 0.12

-6 5 19 1.12 0.16

6 6 -19 1.87 0.17

-6 6 -19 1.77 0.17

6 6 -19 1.71 0.16

6 7 -19 0.12 0.07

-6 7 -19 0.13 0.08

6 7 -19 0.07 0.06

6 8 -19 0.13 0.07

-6 8 -19 0.17 0.07

6 8 -19 0.08 0.06

6 9 -19 0.10 0.06

-6 9 -19 0.08 0.06

6 10 -19 0.34 0.06

-6 10 -19 0.38 0.06

6 10 -19 0.43 0.07

-6 10 -19 0.24 0.05

-6 0 20 0.66 0.12

-6 0 -20 0.99 0.13

-6 1 20 0.07 0.08

-6 -1 -20 0.14 0.07

6 1 20 0.03 0.06

-6 1 -20 0.09 0.08

6 2 -20 0.09 0.06

-6 2 -20 0.08 0.07

-6 2 20 0.04 0.09

6 3 -20 0.43 0.09

-6 3 -20 0.38 0.10

-6 3 20 0.44 0.12

6 4 -20 0.01 0.07

-6 4 -20 -0.03 0.09

6 4 -20 -0.04 0.08

-6 4 20 0.23 0.11

6 5 -20 -0.03 0.07

6 5 -20 -0.03 0.07

-6 5 20 0.10 0.09

-6 5 -20 0.05 0.09

6 6 -20 0.32 0.08

6 6 -20 0.27 0.08

-6 6 -20 0.37 0.10

6 7 -20 0.19 0.08

6 7 -20 0.14 0.06

-6 7 -20 0.13 0.08

-6 8 -20 0.17 0.06

6 8 -20 0.12 0.06

6 8 -20 0.08 0.04

6 9 -20 0.07 0.05

-6 9 -20 -0.02 0.04

-6 10 -20 -0.01 0.03

6 10 -20 0.00 0.03

-6 10 -20 -0.02 0.03

-6 0 21 1.59 0.16

-6 0 -21 1.63 0.15

-6 -1 21 0.34 0.09

-6 1 21 0.36 0.10

-6 -1 -21 0.55 0.09

-6 1 -21 0.51 0.10

-6 2 -21 3.07 0.21

-6 -2 -21 3.07 0.19

-6 2 21 3.19 0.23

-6 -2 21 2.72 0.19

6 3 -21 0.01 0.06

-6 3 21 0.03 0.09

-6 3 -21 -0.03 0.08

6 4 -21 0.02 0.06

6 4 -21 0.03 0.07

-6 4 21 0.16 0.11

-6 4 -21 -0.02 0.08

6 5 -21 0.25 0.07

-6 5 21 0.45 0.12

-6 5 -21 0.33 0.09

6 5 -21 0.30 0.08

6 6 -21 0.61 0.10

-6 6 -21 0.73 0.12

6 6 -21 0.60 0.09

6 7 -21 0.08 0.05

-6 7 -21 0.12 0.07

6 7 -21 0.00 0.04

6 8 -21 0.11 0.05

-6 8 -21 0.13 0.05

6 9 -21 -0.01 0.03

-6 9 -21 0.03 0.03

6 9 -21 0.07 0.04

-6 9 -21 0.00 0.04

-6 0 22 0.07 0.08

-6 0 -22 0.13 0.07

-6 -1 22 0.01 0.07

-6 1 -22 -0.04 0.06

-6 1 22 -0.02 0.07

-6 -1 -22 -0.01 0.05

-6 -2 -22 0.20 0.06

-6 -2 22 0.17 0.07

-6 2 -22 0.18 0.08

-6 2 22 0.31 0.11

6 3 -22 -0.04 0.08

-6 -3 22 0.04 0.05

-6 3 -22 0.04 0.07

-6 3 22 0.03 0.08

6 4 -22 0.42 0.08

-6 -4 22 0.58 0.09

-6 4 22 0.73 0.13

-6 4 -22 0.59 0.11

6 4 -22 0.43 0.09

6 5 -22 0.00 0.05

-6 5 -22 -0.11 0.08

6 5 -22 0.01 0.06

6 6 -22 0.17 0.06

6 6 -22 0.18 0.06

-6 6 -22 0.06 0.06

6 7 -22 0.10 0.05

-6 7 -22 0.07 0.05

6 7 -22 0.06 0.04

6 8 -22 0.29 0.05

-6 8 -22 0.24 0.06

-6 9 -22 0.00 0.03

6 9 -22 -0.04 0.03

-6 0 -23 0.16 0.06

-6 0 23 0.11 0.07

-6 1 -23 0.14 0.06

-6 -1 23 0.02 0.06

-6 -1 -23 0.11 0.05

-6 1 23 -0.01 0.07

-6 2 -23 3.70 0.22

-6 2 23 4.14 0.25

-6 -2 23 3.83 0.21

-6 3 23 0.16 0.09

6 3 -23 0.07 0.07

-6 -3 23 0.19 0.06

-6 3 -23 0.13 0.07

6 4 -23 0.21 0.08

-6 4 23 0.22 0.09

-6 -4 23 0.13 0.05

-6 4 -23 0.22 0.08

6 5 -23 0.28 0.06

6 5 -23 0.21 0.07

6 6 -23 0.91 0.09

6 6 -23 0.86 0.08

-6 6 -23 1.15 0.11

-6 7 -23 -0.01 0.04

6 7 -23 0.05 0.03

6 7 -23 0.03 0.03

-6 8 -23 0.01 0.03

-6 0 -24 0.60 0.09

-6 0 24 0.62 0.10

-6 -1 24 0.20 0.07

-6 1 24 0.25 0.08

-6 -1 -24 0.23 0.06

-6 1 -24 0.14 0.06

6 2 -24 0.03 0.07

-6 2 24 0.10 0.07

-6 2 -24 0.11 0.06

-6 -2 24 0.10 0.06

-6 -3 24 0.04 0.04

-6 3 24 0.11 0.08

-6 3 -24 0.02 0.06

6 3 -24 -0.04 0.07

6 4 -24 0.14 0.06

-6 4 -24 0.14 0.06

-6 5 -24 0.12 0.06

6 5 -24 -0.02 0.04

-6 6 -24 0.01 0.04

6 6 -24 0.01 0.03

-6 7 -24 0.02 0.03

-6 0 -25 0.09 0.04

-6 0 25 0.28 0.07

-6 1 -25 0.10 0.04

-6 -1 25 0.21 0.06

6 1 -25 0.14 0.06

-6 -1 -25 0.09 0.04

-6 1 25 0.16 0.07

-6 -2 25 0.41 0.07

-6 2 25 0.57 0.09

-6 2 -25 0.41 0.07

6 2 -25 0.50 0.08

-6 3 -25 0.11 0.05

6 3 -25 -0.01 0.06

-6 4 -25 0.17 0.06

6 4 -25 0.13 0.05

-6 5 -25 0.03 0.04

6 5 -25 0.01 0.03

-6 6 -25 0.13 0.04

-6 0 -26 0.18 0.04

-6 0 26 0.32 0.06

6 0 -26 0.27 0.06

6 -1 -26 -0.04 0.05

6 1 -26 0.02 0.04

-6 1 -26 0.02 0.04

-6 -1 26 0.04 0.04

6 -2 -26 0.13 0.04

-6 -2 26 0.17 0.04

-6 2 -26 0.15 0.05

6 2 -26 0.13 0.05

6 -3 -26 0.01 0.03

6 3 -26 -0.02 0.04

-6 3 -26 -0.01 0.04

6 4 -26 0.13 0.04

-6 4 -26 0.13 0.04

-6 5 -26 0.05 0.03

6 0 -27 1.00 0.08

6 1 -27 0.01 0.03

6 -1 -27 0.05 0.03

6 2 -27 0.02 0.03

7 0 0 0.04 0.07

7 -1 0 15.20 0.47

7 1 0 15.56 0.44

7 1 0 15.41 0.28

7 2 0 10.15 0.23

7 2 0 10.08 0.28

7 -2 0 9.86 0.39

7 2 0 9.82 0.33

-7 2 0 9.51 0.20

-7 3 0 0.11 0.04

7 3 0 0.09 0.05

7 3 0 0.11 0.05

7 -3 0 0.06 0.07

7 -3 0 0.18 0.06

7 -4 0 110.93 1.28

7 4 0 108.62 1.27

-7 4 0 107.52 1.19

7 5 0 2.08 0.19

-7 5 0 2.32 0.19

7 5 0 2.44 0.17

7 5 0 2.44 0.22

7 6 0 20.20 0.63

7 6 0 20.65 0.71

-7 6 0 19.26 0.59

-7 6 0 19.94 0.68

-7 7 0 0.02 0.05

7 7 0 0.01 0.09

7 7 0 0.08 0.09

-7 7 0 0.07 0.09

-7 8 0 11.90 0.64

-7 8 0 11.93 0.30

7 8 0 11.38 0.56

-7 8 0 11.16 0.55

7 8 0 12.17 0.63

-7 9 0 0.67 0.09

7 9 0 0.44 0.07

-7 9 0 0.58 0.09

7 9 0 0.59 0.09

-7 9 0 0.55 0.09

7 9 0 0.38 0.07

7 9 0 0.40 0.14

7 9 0 0.46 0.16

-7 9 0 0.66 0.16

-7 9 0 0.51 0.18

-7 9 0 0.38 0.07

7 10 0 5.56 0.27

-7 10 0 5.45 0.26

7 10 0 5.52 0.46

-7 10 0 5.68 0.50

7 10 0 5.15 0.21

-7 10 0 5.62 0.25

7 10 0 5.55 0.24

-7 11 0 0.43 0.09

7 11 0 0.42 0.09

7 11 0 0.30 0.08

-7 11 0 0.26 0.08

7 12 0 0.14 0.07

-7 12 0 -0.04 0.08

7 12 0 0.05 0.06

-7 12 0 0.09 0.06

7 13 0 0.09 0.06

-7 13 0 -0.04 0.05

7 13 0 -0.03 0.06

-7 13 0 -0.03 0.05

-7 14 0 0.33 0.06

7 14 0 0.19 0.05

-7 14 0 0.25 0.06

7 0 1 0.01 0.07

7 0 -1 0.00 0.01

7 0 -1 -0.02 0.08

7 0 1 0.08 0.08

7 -1 -1 3.12 0.21

7 1 1 3.13 0.20

7 -1 1 3.26 0.22

7 -1 1 3.22 0.21

7 1 -1 2.78 0.19

7 1 -1 2.95 0.12

-7 2 1 8.24 0.17

7 2 -1 7.86 0.30

7 -2 -1 8.27 0.35

7 2 1 7.92 0.32

7 2 1 8.14 0.25

7 2 -1 8.00 0.25

7 -2 1 8.08 0.32

7 2 -1 8.51 0.21

7 3 1 4.83 0.23

-7 3 -1 4.56 0.19

7 -3 1 4.61 0.25

7 3 -1 4.60 0.22

7 -3 -1 4.26 0.27

-7 3 1 4.69 0.17

7 3 1 4.65 0.25

7 3 -1 4.49 0.23

7 3 -1 4.33 0.20

7 3 1 4.63 0.20

7 4 1 69.19 1.03

7 4 1 65.64 0.95

7 -4 -1 63.31 1.10

-7 4 1 64.65 0.85

7 -4 -1 62.30 0.93

7 -4 1 64.91 0.96

-7 4 1 69.43 1.03

-7 4 -1 63.45 0.90

7 4 -1 67.56 1.05

7 4 1 65.85 0.91

7 4 -1 65.02 0.93

-7 5 1 2.32 0.18

7 5 1 2.43 0.22

7 5 -1 2.27 0.16

7 -5 -1 2.13 0.15

-7 5 1 2.27 0.21

-7 5 -1 2.10 0.18

7 5 -1 2.11 0.21

7 5 1 2.20 0.19

7 5 -1 2.49 0.20

7 6 -1 8.33 0.45

7 6 1 8.01 0.43

-7 6 1 7.90 0.43

-7 6 1 7.93 0.37

-7 6 -1 7.79 0.39

7 6 1 8.01 0.39

7 6 -1 7.72 0.39

-7 7 1 1.00 0.15

-7 7 -1 0.97 0.16

7 7 -1 1.12 0.19

7 7 1 1.11 0.19

-7 7 -1 1.09 0.18

7 7 1 0.90 0.15

7 7 -1 1.00 0.16

-7 7 1 1.06 0.18

-7 8 -1 5.01 0.42

7 8 -1 5.24 0.42

7 8 1 5.29 0.41

-7 8 -1 4.76 0.37

-7 8 1 5.05 0.36

-7 8 1 5.48 0.21

7 8 -1 4.87 0.38

7 8 1 5.19 0.38

-7 8 1 5.27 0.23

-7 8 1 5.20 0.41

-7 8 -1 4.88 0.19

7 9 -1 0.95 0.09

7 9 1 0.93 0.09

-7 9 -1 0.98 0.11

7 9 1 1.04 0.12

-7 9 1 0.97 0.10

-7 9 1 1.10 0.12

7 9 -1 0.96 0.11

7 9 -1 0.96 0.22

-7 9 -1 0.91 0.18

7 9 1 1.03 0.21

-7 9 -1 0.88 0.22

7 9 1 0.85 0.18

7 9 -1 0.88 0.18

-7 9 1 1.06 0.22

-7 9 -1 1.06 0.12

-7 9 1 0.97 0.11

7 9 -1 0.99 0.11

-7 9 -1 1.06 0.11

-7 9 1 1.04 0.11

7 9 1 0.95 0.10

7 10 -1 3.01 0.37

-7 10 -1 3.24 0.35

7 10 -1 2.80 0.15

7 10 -1 3.03 0.20

7 10 1 3.08 0.20

-7 10 -1 2.99 0.38

-7 10 1 2.47 0.32

-7 10 1 2.98 0.20

7 10 1 2.82 0.16

7 10 1 2.96 0.33

-7 10 -1 2.82 0.19

7 10 -1 2.54 0.32

-7 10 1 3.01 0.18

7 10 -1 2.99 0.18

7 10 1 3.05 0.18

-7 10 -1 3.29 0.19

-7 11 1 0.48 0.10

-7 11 -1 0.40 0.09

7 11 -1 0.49 0.10

7 11 1 0.52 0.10

-7 11 -1 0.39 0.09

-7 11 1 0.46 0.09

7 11 -1 0.44 0.09

7 11 1 0.42 0.09

-7 12 -1 0.02 0.06

7 12 -1 -0.04 0.06

7 12 -1 0.15 0.08

7 12 1 0.11 0.08

-7 12 -1 -0.11 0.07

-7 12 1 -0.18 0.09

7 12 1 0.07 0.06

-7 12 1 0.01 0.06

7 13 -1 0.23 0.07

7 13 1 0.06 0.06

-7 13 1 0.01 0.05

-7 13 -1 0.00 0.05

7 13 1 0.03 0.05

-7 13 -1 0.03 0.05

-7 13 1 0.08 0.05

7 13 -1 0.03 0.06

-7 14 1 0.10 0.05

-7 14 -1 0.06 0.04

-7 14 -1 0.03 0.04

7 14 -1 0.01 0.04

7 0 2 -0.04 0.07

7 0 -2 -0.03 0.07

7 1 2 21.36 0.53

7 1 -2 17.62 0.47

7 -1 -2 17.90 0.52

7 -1 2 20.74 0.53

7 1 -2 19.46 0.34

-7 1 2 20.64 0.26

7 1 -2 18.69 0.21

7 2 -2 39.86 0.50

7 2 -2 39.10 0.70

7 -2 -2 39.20 0.80

7 -2 2 39.17 0.75

7 2 2 38.43 0.73

7 2 -2 40.34 0.62

-7 2 2 39.17 0.53

7 3 2 41.20 0.68

7 3 -2 39.82 0.69

7 -3 -2 38.47 0.84

7 3 2 40.44 0.76

-7 3 2 40.65 0.66

7 -3 2 41.02 0.78

7 3 -2 41.09 0.63

7 3 -2 41.59 0.73

7 3 2 40.87 0.62

7 4 2 36.57 0.73

7 4 2 33.93 0.68

-7 4 2 33.87 0.58

7 4 -2 36.19 0.60

7 -4 -2 34.50 0.81

-7 4 2 36.01 0.70

7 4 -2 34.28 0.67

-7 4 -2 34.35 0.67

7 4 -2 36.04 0.76

7 4 2 34.84 0.65

7 -5 -2 0.67 0.09

7 5 -2 0.82 0.13

-7 5 2 0.65 0.10

-7 5 -2 0.83 0.13

7 5 -2 0.90 0.15

7 5 2 0.73 0.12

7 5 2 0.80 0.14

7 -5 -2 0.67 0.12

-7 5 2 0.63 0.12

7 6 2 18.22 0.64

-7 6 2 16.89 0.53

7 6 -2 17.29 0.60

-7 6 -2 16.00 0.57

7 6 -2 17.80 0.67

7 6 2 17.47 0.57

-7 6 2 17.67 0.62

7 7 2 4.22 0.34

7 7 -2 3.28 0.32

-7 7 -2 3.04 0.28

7 7 -2 3.53 0.30

7 7 2 3.61 0.29

-7 7 2 3.84 0.32

-7 8 -2 0.91 0.18

7 8 -2 1.22 0.22

7 8 -2 1.18 0.20

-7 8 2 1.16 0.20

7 8 2 1.01 0.18

-7 8 -2 1.41 0.24

-7 8 2 1.09 0.10

7 8 2 1.21 0.12

-7 8 2 1.08 0.11

7 9 -2 0.99 0.09

7 9 2 1.13 0.10

-7 9 2 1.04 0.11

-7 9 2 1.05 0.12

7 9 2 1.07 0.12

-7 9 -2 0.89 0.11

7 9 -2 0.93 0.11

7 9 2 1.14 0.21

-7 9 -2 1.01 0.20

7 9 -2 1.21 0.24

-7 9 2 1.04 0.21

7 9 -2 1.02 0.21

-7 9 -2 0.76 0.21

-7 9 -2 1.01 0.12

7 9 -2 0.94 0.11

-7 9 -2 0.99 0.11

7 9 2 1.01 0.10

7 9 -2 0.92 0.11

-7 9 2 1.01 0.10

7 10 2 2.73 0.16

-7 10 2 2.40 0.32

7 10 -2 2.90 0.34

7 10 -2 2.97 0.19

-7 10 -2 2.84 0.19

-7 10 2 2.96 0.19

7 10 2 2.65 0.19

-7 10 -2 2.93 0.38

7 10 2 2.87 0.32

7 10 -2 2.85 0.15

-7 10 -2 2.69 0.32

7 10 -2 3.25 0.39

-7 10 -2 2.87 0.19

7 10 2 2.70 0.17

7 10 -2 2.97 0.18

-7 10 -2 3.33 0.20

-7 10 2 3.05 0.18

7 11 2 1.84 0.17

-7 11 2 2.09 0.17

-7 11 -2 1.70 0.15

7 11 -2 1.85 0.16

-7 11 2 2.07 0.16

-7 11 -2 1.85 0.15

7 11 -2 1.68 0.15

7 11 2 1.80 0.15

7 12 -2 1.13 0.12

7 12 2 1.21 0.14

-7 12 -2 0.84 0.10

-7 12 2 0.99 0.12

7 12 -2 0.88 0.11

-7 12 -2 1.07 0.12

-7 12 2 1.03 0.12

7 12 2 1.07 0.12

7 13 -2 0.36 0.08

7 13 2 0.40 0.09

-7 13 2 0.18 0.07

-7 13 -2 0.14 0.05

-7 13 -2 0.18 0.06

-7 13 2 0.18 0.06

7 13 -2 0.13 0.06

7 13 2 0.16 0.06

7 14 2 0.48 0.07

-7 14 -2 0.28 0.05

-7 14 2 0.48 0.07

-7 14 -2 0.24 0.05

7 14 -2 0.34 0.06

7 0 -3 -0.05 0.08

7 0 3 -0.03 0.07

7 1 -3 5.95 0.29

7 -1 -3 6.16 0.31

7 1 3 6.27 0.31

7 -1 3 6.41 0.31

7 -2 3 40.49 0.80

7 2 -3 40.24 0.64

7 2 -3 42.05 0.75

7 2 3 40.73 0.78

7 -2 -3 38.96 0.80

-7 2 3 40.20 0.52

7 3 -3 7.13 0.30

7 3 -3 8.22 0.33

7 -3 -3 7.73 0.38

-7 3 3 7.92 0.27

7 4 -3 6.81 0.28

7 -4 -3 6.48 0.36

7 4 -3 7.29 0.35

-7 4 -3 6.58 0.31

7 4 3 7.21 0.30

-7 4 3 7.05 0.30

7 -5 -3 2.91 0.25

7 5 -3 2.64 0.22

7 -5 -3 2.89 0.19

-7 5 -3 2.96 0.23

7 5 -3 3.28 0.27

-7 5 3 3.44 0.24

7 5 3 3.34 0.23

7 6 -3 3.02 0.26

-7 6 -3 2.91 0.25

7 6 -3 3.30 0.30

-7 6 3 3.20 0.27

7 6 3 3.41 0.26

7 7 -3 1.67 0.21

-7 7 -3 1.93 0.23

7 7 -3 1.66 0.23

7 7 3 1.85 0.22

-7 7 3 1.98 0.23

7 8 -3 1.84 0.27

-7 8 -3 1.71 0.27

-7 8 -3 1.66 0.24

7 8 -3 1.59 0.23

-7 8 3 1.86 0.13

7 8 3 2.09 0.13

7 8 3 1.96 0.15

-7 8 3 1.82 0.24

7 8 3 1.83 0.23

-7 8 3 1.81 0.14

7 9 3 3.30 0.17

-7 9 3 3.09 0.17

-7 9 3 3.37 0.35

-7 9 -3 3.30 0.19

7 9 3 3.22 0.20

7 9 -3 3.43 0.19

7 9 3 3.06 0.32

7 9 -3 3.25 0.15

-7 9 3 3.43 0.20

7 9 -3 3.25 0.35

7 9 -3 3.23 0.19

-7 9 -3 3.62 0.20

7 9 -3 3.32 0.38

-7 9 -3 3.11 0.34

-7 9 -3 3.05 0.38

7 9 -3 3.23 0.20

-7 9 -3 3.23 0.20

7 9 3 3.30 0.18

-7 9 3 3.33 0.18

7 10 -3 0.35 0.06

7 10 3 0.34 0.08

7 10 -3 0.34 0.08

-7 10 -3 0.33 0.09

7 10 3 0.28 0.09

-7 10 3 0.39 0.10

7 10 -3 0.41 0.09

-7 10 -3 0.33 0.08

7 10 -3 0.58 0.21

-7 10 -3 0.32 0.14

-7 10 3 0.31 0.15

-7 10 -3 0.43 0.18

7 10 -3 0.24 0.13

7 10 -3 0.34 0.09

-7 10 -3 0.29 0.08

-7 10 3 0.30 0.08

7 10 3 0.41 0.09

-7 11 -3 0.22 0.08

7 11 -3 0.19 0.07

-7 11 -3 0.17 0.07

7 11 3 0.19 0.08

-7 11 3 0.07 0.07

7 11 -3 0.19 0.08

-7 11 3 0.17 0.07

7 11 3 0.11 0.07

7 12 -3 0.78 0.10

-7 12 3 0.47 0.09

7 12 3 0.72 0.12

7 12 -3 0.55 0.09

-7 12 -3 0.57 0.09

-7 12 -3 0.55 0.09

-7 12 3 0.58 0.09

7 12 3 0.57 0.09

-7 13 -3 0.16 0.06

7 13 -3 0.29 0.06

-7 13 3 0.18 0.07

-7 13 -3 0.18 0.05

7 13 -3 0.19 0.07

-7 13 3 0.16 0.05

7 13 3 0.12 0.05

-7 14 -3 0.31 0.05

-7 14 3 0.43 0.07

-7 14 -3 0.38 0.06

7 14 -3 0.34 0.06

7 0 -4 0.04 0.08

7 -1 -4 40.75 0.84

7 1 -4 40.99 0.81

7 -2 -4 23.97 0.65

7 2 -4 24.10 0.61

7 3 -4 33.77 0.71

7 -3 -4 32.45 0.77

-7 3 4 31.86 0.55

7 4 -4 7.07 0.31

7 -4 -4 7.38 0.40

7 4 -4 7.95 0.38

-7 4 4 7.93 0.32

7 -5 -4 9.36 0.46

-7 5 -4 9.31 0.43

7 5 -4 9.50 0.47

-7 5 4 8.99 0.38

7 6 -4 5.71 0.36

-7 6 -4 5.35 0.36

7 6 -4 6.04 0.41

7 6 4 6.03 0.34

-7 6 4 6.03 0.35

7 7 -4 5.94 0.40

-7 7 -4 5.65 0.40

7 7 -4 6.36 0.45

7 7 4 6.04 0.38

-7 7 4 6.36 0.39

7 8 -4 1.43 0.24

-7 8 -4 1.56 0.23

7 8 4 1.77 0.12

-7 8 -4 1.35 0.12

7 8 4 1.87 0.15

7 8 -4 1.38 0.22

-7 8 4 1.86 0.13

-7 8 4 1.93 0.14

-7 8 -4 1.58 0.12

7 8 -4 1.52 0.12

-7 8 4 1.64 0.23

-7 8 4 2.04 0.13

7 8 4 1.95 0.13

7 9 4 4.52 0.19

-7 9 4 4.63 0.20

7 9 -4 4.40 0.21

7 9 4 4.51 0.24

-7 9 4 4.55 0.23

7 9 -4 4.27 0.40

-7 9 4 4.68 0.40

7 9 -4 4.29 0.22

-7 9 -4 4.41 0.22

-7 9 -4 4.19 0.21

-7 9 -4 3.97 0.39

7 9 -4 4.59 0.45

-7 9 -4 4.20 0.44

7 9 -4 4.15 0.23

-7 9 -4 4.05 0.22

7 9 4 4.38 0.20

-7 9 4 4.67 0.21

7 10 -4 4.70 0.23

7 10 4 4.91 0.26

7 10 4 4.85 0.21

-7 10 4 5.30 0.26

-7 10 4 4.82 0.43

-7 10 -4 4.11 0.22

7 10 -4 4.54 0.43

-7 10 -4 4.69 0.24

7 10 -4 4.51 0.23

-7 10 -4 4.79 0.49

-7 10 -4 4.71 0.44

7 10 -4 4.89 0.49

7 10 -4 4.14 0.23

-7 10 -4 4.24 0.23

-7 10 4 5.33 0.23

7 10 4 4.89 0.22

7 11 -4 0.07 0.06

7 11 4 0.03 0.06

7 11 -4 0.03 0.06

-7 11 -4 0.06 0.06

-7 11 -4 0.07 0.07

-7 11 4 -0.07 0.08

7 11 4 0.09 0.06

-7 11 4 0.05 0.06

7 12 -4 0.20 0.06

7 12 -4 0.03 0.06

-7 12 4 0.09 0.07

7 12 4 0.10 0.07

-7 12 -4 0.22 0.07

-7 12 -4 0.04 0.05

7 12 4 0.12 0.06

-7 12 4 0.12 0.06

7 13 4 0.22 0.08

-7 13 4 -0.01 0.05

-7 13 -4 0.02 0.04

7 13 -4 0.07 0.06

-7 13 -4 0.14 0.06

7 14 4 0.65 0.08

-7 14 -4 0.36 0.05

-7 14 4 0.60 0.07

-7 14 -4 0.45 0.06

7 14 -4 0.49 0.07

7 0 -5 -0.01 0.08

7 1 -5 7.80 0.37

7 -1 -5 7.69 0.38

7 2 -5 13.98 0.49

7 -2 -5 13.98 0.53

7 3 -5 1.69 0.17

7 -3 -5 1.54 0.19

-7 3 5 1.84 0.14

7 4 -5 1.24 0.15

7 -4 -5 1.07 0.16

7 4 -5 1.15 0.16

-7 4 5 1.06 0.13

7 5 -5 3.36 0.21

7 -5 -5 3.55 0.29

-7 5 -5 3.38 0.26

7 5 -5 3.95 0.32

-7 5 5 3.03 0.23

-7 6 -5 5.28 0.37

7 6 -5 6.09 0.43

-7 6 5 5.81 0.34

-7 7 -5 0.23 0.10

7 7 -5 0.27 0.14

-7 7 5 0.17 0.10

7 7 -5 0.21 0.10

-7 8 -5 0.04 0.05

7 8 -5 0.08 0.05

7 8 5 0.13 0.05

-7 8 -5 0.00 0.04

7 8 -5 0.03 0.04

-7 8 5 0.08 0.05

-7 8 5 0.08 0.04

7 8 5 0.10 0.06

-7 8 -5 0.09 0.08

7 8 -5 0.03 0.09

-7 8 5 0.10 0.08

7 8 -5 0.17 0.11

7 8 -5 0.08 0.07

-7 8 -5 0.11 0.06

7 8 5 0.12 0.05

-7 8 5 0.19 0.05

-7 9 5 1.71 0.13

7 9 -5 1.74 0.13

7 9 -5 1.61 0.26

7 9 5 1.70 0.16

-7 9 5 1.81 0.15

-7 9 5 1.47 0.24

7 9 5 1.69 0.12

7 9 -5 1.63 0.14

-7 9 -5 1.57 0.13

-7 9 -5 1.71 0.14

7 9 -5 1.94 0.32

-7 9 -5 1.52 0.26

-7 9 -5 1.38 0.27

7 9 -5 1.77 0.16

-7 9 -5 1.71 0.16

-7 9 5 1.81 0.14

7 9 5 1.73 0.13

-7 10 5 5.71 0.26

7 10 -5 5.53 0.24

7 10 5 4.99 0.26

-7 10 5 5.32 0.45

7 10 -5 5.04 0.24

-7 10 -5 4.89 0.23

7 10 -5 4.66 0.44

-7 10 -5 5.27 0.25

-7 10 -5 4.93 0.46

7 10 -5 5.57 0.53

-7 10 -5 4.95 0.52

-7 10 -5 4.98 0.26

7 10 -5 4.59 0.25

-7 10 5 5.38 0.23

7 10 5 4.78 0.22

7 11 -5 0.12 0.06

7 11 5 0.19 0.08

-7 11 5 0.03 0.08

-7 11 -5 0.20 0.07

-7 11 -5 0.18 0.08

7 11 -5 0.12 0.07

7 11 -5 0.23 0.09

-7 11 5 0.16 0.07

7 11 5 0.23 0.07

7 12 -5 0.46 0.09

7 12 5 0.55 0.11

-7 12 -5 0.41 0.09

7 12 -5 0.64 0.09

-7 12 5 0.48 0.09

-7 12 -5 0.39 0.07

7 13 5 0.25 0.08

-7 13 5 0.16 0.07

-7 13 -5 0.12 0.04

7 13 -5 0.16 0.06

-7 13 -5 0.03 0.05

7 14 5 0.12 0.05

-7 14 5 0.10 0.05

7 14 -5 0.05 0.04

-7 14 -5 0.00 0.04

7 0 -6 0.01 0.09

7 1 -6 80.57 1.32

7 -1 -6 83.49 1.37

7 -2 -6 5.16 0.35

7 2 -6 4.96 0.32

7 3 -6 20.40 0.63

7 -3 -6 19.73 0.68

7 4 -6 15.00 0.53

7 -4 -6 13.07 0.55

7 -5 -6 8.66 0.48

7 5 -6 9.08 0.40

7 5 6 9.10 0.41

7 -5 6 9.19 0.46

7 5 -6 9.04 0.48

-7 5 6 8.51 0.37

-7 6 -6 8.72 0.49

7 6 -6 9.05 0.53

-7 6 6 8.38 0.41

-7 7 -6 4.76 0.39

7 7 -6 4.91 0.38

7 7 -6 5.05 0.43

-7 7 6 4.98 0.35

7 8 6 5.98 0.22

-7 8 6 5.72 0.22

-7 8 6 5.89 0.41

-7 8 6 6.15 0.25

7 8 6 6.21 0.27

-7 8 -6 5.82 0.24

7 8 -6 6.11 0.46

-7 8 -6 5.32 0.44

7 8 -6 6.42 0.51

-7 8 -6 5.80 0.26

7 8 -6 6.21 0.28

-7 8 6 6.11 0.22

7 8 6 6.05 0.22

-7 9 6 8.34 0.27

7 9 6 8.23 0.26

7 9 -6 8.97 0.29

7 9 6 8.77 0.34

-7 9 6 9.19 0.32

7 9 -6 8.94 0.59

-7 9 -6 8.48 0.30

-7 9 -6 9.48 0.33

7 9 -6 9.25 0.32

-7 9 -6 8.74 0.60

-7 9 -6 8.54 0.66

7 9 -6 9.71 0.68

-7 9 -6 8.49 0.33

7 9 -6 8.14 0.33

-7 9 6 9.09 0.29

7 9 6 8.57 0.28

7 10 6 1.39 0.15

-7 10 6 1.31 0.14

-7 10 -6 1.46 0.13

7 10 -6 1.49 0.13

-7 10 -6 1.42 0.14

7 10 -6 1.30 0.14

7 10 -6 1.54 0.26

7 10 -6 1.17 0.14

-7 10 -6 1.37 0.14

-7 10 6 1.28 0.12

7 10 6 1.30 0.12

-7 11 -6 0.45 0.08

7 11 -6 0.45 0.08

7 11 6 0.54 0.10

-7 11 6 0.44 0.09

-7 11 -6 0.48 0.10

7 11 -6 0.37 0.09

7 11 -6 0.48 0.10

-7 11 6 0.50 0.08

7 11 6 0.46 0.08

-7 12 6 0.59 0.10

-7 12 -6 0.58 0.07

-7 12 -6 0.65 0.10

7 12 -6 0.64 0.10

7 12 6 0.65 0.11

7 13 6 0.35 0.08

-7 13 6 0.31 0.07

-7 13 -6 0.31 0.07

7 13 -6 0.34 0.07

7 14 6 0.12 0.05

-7 14 6 0.17 0.05

-7 14 -6 0.01 0.03

7 14 -6 0.06 0.04

7 0 -7 -0.02 0.10

7 -1 -7 6.42 0.40

7 1 -7 6.59 0.39

7 -2 -7 22.97 0.75

7 2 -7 23.45 0.73

7 3 -7 11.80 0.51

7 -3 -7 11.81 0.55

7 4 -7 10.82 0.49

7 -4 -7 9.74 0.51

7 5 -7 1.08 0.19

7 -5 -7 0.90 0.16

7 5 -7 1.10 0.16

7 -5 7 1.46 0.21

7 5 7 1.26 0.17

-7 6 -7 8.40 0.50

7 6 -7 10.04 0.57

-7 7 -7 2.13 0.27

7 7 -7 2.52 0.32

-7 8 7 0.71 0.08

-7 8 7 0.74 0.09

7 8 7 0.78 0.10

-7 8 -7 0.56 0.08

7 8 7 0.74 0.08

-7 8 -7 0.56 0.15

7 8 -7 0.74 0.21

7 8 -7 0.63 0.17

-7 8 -7 0.71 0.10

-7 8 -7 0.75 0.10

7 8 -7 0.70 0.10

-7 8 7 0.77 0.08

7 8 7 0.70 0.08

-7 9 7 2.35 0.15

-7 9 7 2.51 0.17

7 9 -7 2.37 0.17

7 9 7 2.20 0.17

-7 9 -7 2.15 0.15

-7 9 -7 2.31 0.17

7 9 -7 2.27 0.31

-7 9 -7 2.19 0.32

7 9 -7 2.24 0.34

7 9 -7 2.06 0.18

-7 9 -7 2.24 0.18

-7 9 7 2.14 0.14

7 9 7 2.07 0.14

7 10 -7 0.90 0.10

-7 10 7 1.07 0.12

-7 10 -7 0.88 0.10

7 10 7 0.91 0.13

-7 10 -7 1.00 0.13

7 10 -7 0.77 0.11

-7 10 -7 0.85 0.12

7 10 -7 0.89 0.13

7 11 7 0.08 0.07

7 11 -7 -0.01 0.04

-7 11 7 -0.02 0.06

-7 11 -7 0.01 0.06

7 11 -7 0.01 0.06

7 11 -7 0.10 0.08

-7 11 -7 0.04 0.05

7 12 7 0.22 0.08

-7 12 -7 0.17 0.07

-7 12 7 0.17 0.07

7 12 -7 0.28 0.08

7 13 -7 0.16 0.06

-7 13 -7 0.03 0.05

7 13 7 0.08 0.05

-7 13 7 0.16 0.06

-7 14 -7 0.09 0.04

7 14 -7 0.12 0.04

7 0 -8 0.12 0.11

7 -1 -8 62.51 1.32

7 1 -8 61.24 1.28

7 -2 -8 14.55 0.64

7 2 -8 15.27 0.63

7 -3 -8 17.97 0.72

7 3 -8 18.51 0.68

7 4 -8 14.20 0.60

7 -4 -8 14.58 0.66

7 -4 8 14.70 0.65

7 4 8 14.51 0.61

7 5 -8 2.93 0.27

7 -5 -8 2.63 0.28

7 5 8 2.79 0.27

7 5 -8 3.18 0.31

7 -5 8 2.89 0.30

7 6 -8 1.85 0.26

7 7 8 1.56 0.13

-7 7 8 1.41 0.11

-7 7 -8 1.54 0.14

7 7 -8 1.66 0.15

-7 7 -8 1.64 0.14

-7 7 -8 1.27 0.23

7 7 -8 1.60 0.26

-7 8 8 5.16 0.20

-7 8 8 5.46 0.23

7 8 8 5.23 0.25

-7 8 -8 5.18 0.26

7 8 -8 5.24 0.27

-7 8 -8 6.04 0.27

7 8 -8 5.47 0.45

-7 8 -8 4.85 0.45

7 8 -8 6.09 0.53

-7 9 -8 3.48 0.21

7 9 -8 3.58 0.21

-7 9 8 3.59 0.20

7 9 8 3.24 0.21

-7 9 -8 3.21 0.22

7 9 -8 3.34 0.23

7 9 -8 3.81 0.40

-7 9 -8 3.01 0.38

7 9 -8 3.72 0.43

-7 10 8 0.37 0.08

7 10 8 0.35 0.09

7 10 -8 0.44 0.09

-7 10 -8 0.29 0.08

-7 10 -8 0.45 0.10

7 10 -8 0.52 0.12

7 11 8 1.87 0.16

-7 11 8 1.97 0.15

7 11 -8 1.82 0.16

-7 11 -8 1.76 0.16

-7 11 -8 1.50 0.14

7 11 -8 1.58 0.16

7 12 8 0.61 0.10

-7 12 8 0.63 0.09

7 12 -8 0.59 0.10

-7 12 -8 0.67 0.10

7 13 8 0.14 0.06

-7 13 8 0.23 0.06

7 13 -8 0.11 0.05

-7 13 -8 0.14 0.05

7 14 -8 0.15 0.04

-7 14 -8 0.13 0.04

7 0 -9 -0.08 0.14

7 -1 -9 5.55 0.42

7 1 -9 5.24 0.40

7 -2 -9 6.29 0.44

7 2 -9 5.79 0.41

7 -3 -9 7.03 0.48

7 3 9 7.25 0.45

7 -3 9 7.79 0.49

7 3 -9 6.69 0.44

7 -4 -9 13.06 0.66

7 4 -9 13.06 0.62

7 -4 9 13.55 0.66

7 4 9 13.37 0.62

7 -5 -9 1.11 0.21

7 5 -9 1.13 0.18

7 -5 9 1.14 0.21

7 5 9 1.11 0.19

7 6 -9 3.58 0.37

-7 7 9 6.23 0.23

7 7 9 6.37 0.26

7 7 -9 5.96 0.28

-7 7 -9 6.03 0.26

-7 7 -9 5.90 0.27

-7 7 -9 5.12 0.46

7 7 -9 6.13 0.51

7 8 9 2.34 0.17

-7 8 9 2.58 0.16

-7 8 -9 2.64 0.19

-7 8 -9 3.10 0.20

7 8 -9 2.87 0.21

7 8 -9 2.52 0.32

7 8 -9 2.74 0.37

-7 8 -9 2.29 0.32

7 9 9 1.75 0.15

-7 9 9 2.13 0.15

7 9 -9 1.73 0.17

-7 9 -9 1.76 0.17

7 9 -9 1.81 0.29

7 9 -9 2.28 0.33

-7 9 -9 1.56 0.29

-7 10 9 0.19 0.07

7 10 9 0.23 0.08

-7 10 -9 0.35 0.09

7 10 -9 0.30 0.09

7 10 -9 0.41 0.11

-7 10 -9 0.31 0.08

7 11 9 0.52 0.10

-7 11 -9 0.65 0.10

-7 11 9 0.58 0.10

7 11 -9 0.63 0.11

7 11 -9 0.61 0.12

-7 11 -9 0.66 0.10

7 12 9 0.15 0.06

-7 12 9 0.31 0.07

-7 12 -9 0.27 0.07

7 12 -9 0.23 0.07

7 13 9 0.02 0.04

-7 13 9 0.16 0.05

7 13 -9 0.05 0.04

-7 13 -9 0.01 0.04

-7 14 -9 0.11 0.04

7 14 -9 0.08 0.03

7 0 -10 -0.01 0.13

7 1 -10 22.81 0.86

7 -1 -10 23.53 0.90

7 -2 10 0.50 0.14

7 2 -10 0.67 0.17

7 -2 -10 0.63 0.17

7 2 10 0.45 0.12

7 -3 -10 21.30 0.87

7 -3 10 20.65 0.84

7 3 10 20.49 0.81

7 3 -10 19.91 0.81

7 4 -10 8.47 0.53

7 -4 -10 8.17 0.55

7 4 10 8.13 0.52

7 -4 10 8.08 0.54

7 5 -10 14.68 0.69

7 -5 -10 15.00 0.75

7 -5 10 16.03 0.77

7 5 10 16.34 0.72

7 6 -10 -0.04 0.11

7 6 -10 0.08 0.06

-7 6 -10 0.11 0.06

7 7 10 3.63 0.20

-7 7 10 3.60 0.18

-7 7 -10 3.58 0.22

-7 7 -10 3.95 0.22

7 7 -10 4.23 0.25

7 7 -10 3.85 0.42

-7 7 -10 3.02 0.36

7 8 10 0.12 0.06

-7 8 10 0.10 0.05

-7 8 -10 0.18 0.08

-7 8 -10 0.25 0.09

7 8 -10 0.14 0.09

-7 8 -10 0.07 0.10

7 8 -10 0.06 0.12

7 8 -10 -0.03 0.09

7 9 10 7.00 0.29

-7 9 10 7.23 0.27

-7 9 -10 6.13 0.30

7 9 -10 6.32 0.32

7 10 10 5.19 0.25

7 10 -10 5.22 0.26

-7 10 10 5.52 0.24

-7 10 -10 5.34 0.26

7 10 -10 4.98 0.28

-7 10 -10 4.61 0.26

-7 11 10 0.86 0.10

-7 11 -10 0.63 0.10

7 11 10 0.65 0.10

7 11 -10 0.81 0.11

7 11 -10 0.65 0.12

-7 11 -10 0.42 0.08

7 12 10 0.16 0.06

-7 12 10 0.26 0.06

7 12 -10 0.31 0.08

-7 12 -10 0.30 0.07

-7 13 -10 0.17 0.05

7 13 -10 0.10 0.04

7 13 10 0.19 0.05

7 14 -10 0.22 0.04

-7 14 -10 0.16 0.03

7 0 -11 0.01 0.14

7 0 11 -0.22 0.17

7 -1 -11 2.10 0.30

7 -1 11 2.56 0.31

7 1 11 2.14 0.29

7 1 -11 2.27 0.31

7 -2 11 1.58 0.25

7 -2 -11 1.93 0.28

7 2 11 1.67 0.25

7 2 -11 2.00 0.28

7 3 -11 0.65 0.18

7 3 11 0.23 0.12

7 -3 -11 0.51 0.16

7 -3 11 0.30 0.15

7 4 -11 6.58 0.50

7 -4 -11 7.14 0.54

7 -4 11 7.49 0.55

7 4 11 6.56 0.49

7 5 -11 2.65 0.32

7 -5 -11 2.82 0.35

7 -5 11 2.71 0.34

7 6 -11 1.24 0.24

-7 6 -11 1.53 0.15

-7 6 -11 1.47 0.13

7 6 -11 1.48 0.14

-7 6 11 1.19 0.13

-7 7 11 1.56 0.12

7 7 11 1.67 0.14

-7 7 -11 1.97 0.16

-7 7 -11 1.71 0.16

7 7 -11 1.92 0.17

7 7 -11 1.89 0.30

-7 7 -11 1.23 0.25

7 8 11 1.11 0.12

-7 8 11 1.26 0.11

-7 8 -11 1.43 0.15

-7 8 -11 1.28 0.15

7 8 -11 1.43 0.17

-7 8 -11 0.94 0.23

7 8 -11 1.63 0.31

7 9 11 -0.01 0.05

-7 9 11 0.04 0.05

-7 9 -11 -0.01 0.06

7 9 -11 0.08 0.09

-7 10 11 2.00 0.14

7 10 11 1.81 0.15

7 10 -11 2.03 0.17

-7 10 -11 1.99 0.16

-7 10 -11 1.80 0.17

7 10 -11 2.00 0.19

-7 11 11 0.76 0.09

7 11 11 0.69 0.09

-7 11 -11 0.52 0.09

7 11 -11 0.71 0.10

7 11 -11 0.58 0.11

-7 11 -11 0.41 0.08

-7 12 11 0.49 0.07

7 12 11 0.45 0.07

-7 12 -11 0.41 0.07

7 12 -11 0.43 0.08

-7 13 -11 0.05 0.04

7 13 -11 0.02 0.04

7 0 -12 0.00 0.13

7 0 12 -0.01 0.14

7 -1 12 40.32 1.28

7 1 12 39.12 1.25

7 1 -12 37.61 1.25

7 -1 -12 40.23 1.31

7 2 12 2.19 0.31

7 -2 12 2.47 0.33

7 -2 -12 2.48 0.34

7 2 -12 2.09 0.30

7 -3 -12 13.67 0.78

7 -3 12 13.88 0.77

7 3 12 12.86 0.71

7 3 -12 12.67 0.72

7 4 -12 1.58 0.26

7 -4 12 1.45 0.26

7 -4 -12 1.72 0.29

7 4 12 1.37 0.24

7 5 -12 3.82 0.40

7 -5 -12 3.52 0.41

7 -5 12 3.50 0.40

7 5 -12 4.13 0.22

-7 5 -12 3.80 0.22

-7 5 12 3.95 0.23

7 6 -12 0.74 0.21

-7 6 -12 0.57 0.09

-7 6 -12 0.64 0.10

-7 6 12 0.69 0.11

7 7 12 4.64 0.22

-7 7 -12 4.17 0.45

7 7 -12 4.83 0.49

7 7 -12 5.40 0.28

-7 7 -12 4.92 0.27

-7 7 -12 5.16 0.25

-7 8 12 0.49 0.07

7 8 12 0.44 0.08

-7 8 -12 0.35 0.17

-7 8 -12 0.63 0.11

7 8 -12 0.47 0.11

-7 8 -12 0.43 0.10

7 9 12 7.18 0.28

-7 9 12 7.27 0.25

7 9 -12 7.82 0.36

-7 9 -12 6.70 0.32

-7 10 -12 0.22 0.08

7 10 -12 0.24 0.08

-7 10 12 0.36 0.07

7 10 12 0.34 0.07

7 10 -12 0.32 0.11

-7 10 -12 0.25 0.08

-7 11 12 0.06 0.04

7 11 12 0.07 0.05

-7 11 -12 0.06 0.06

7 11 -12 0.01 0.06

7 11 -12 0.04 0.07

-7 11 -12 0.08 0.06

7 12 12 0.07 0.04

7 12 -12 0.07 0.05

-7 12 -12 0.12 0.05

-7 13 -12 0.25 0.05

7 13 -12 0.25 0.05

7 0 -13 0.00 0.15

7 0 13 0.13 0.15

7 1 -13 1.50 0.28

7 -1 -13 1.94 0.32

7 -1 13 1.63 0.30

7 1 13 1.56 0.29

7 -2 -13 5.46 0.51

7 -2 13 5.24 0.50

7 2 13 5.11 0.48

7 2 -13 5.67 0.52

7 -3 -13 8.51 0.65

7 -3 13 7.61 0.60

7 3 13 7.74 0.58

7 3 -13 7.93 0.60

7 4 -13 4.21 0.44

7 -4 -13 4.59 0.48

7 -4 13 4.42 0.47

7 4 13 3.91 0.42

-7 4 -13 5.04 0.25

7 4 -13 4.89 0.24

-7 4 13 5.09 0.26

7 5 -13 0.00 0.11

7 -5 -13 0.24 0.14

7 -5 13 -0.06 0.18

-7 5 -13 0.09 0.05

7 5 -13 0.18 0.07

-7 5 13 0.17 0.08

-7 5 -13 0.16 0.07

7 6 -13 2.94 0.37

-7 6 -13 2.72 0.20

-7 6 -13 2.87 0.18

7 6 -13 3.19 0.22

7 6 -13 2.28 0.17

-7 6 13 2.45 0.20

-7 7 -13 1.57 0.16

7 7 -13 1.94 0.19

-7 7 -13 1.73 0.15

-7 7 -13 1.37 0.27

7 7 -13 1.68 0.32

-7 8 -13 1.33 0.15

7 8 -13 1.64 0.18

-7 8 -13 1.46 0.15

-7 9 13 0.30 0.06

7 9 13 0.20 0.06

-7 9 -13 0.25 0.09

7 9 -13 0.29 0.11

-7 10 -13 1.09 0.12

7 10 13 1.29 0.11

-7 10 13 1.22 0.10

7 10 -13 1.11 0.12

-7 10 -13 1.16 0.13

7 10 -13 1.36 0.16

7 11 13 0.20 0.05

-7 11 -13 0.17 0.06

7 11 -13 0.17 0.06

-7 11 -13 0.04 0.05

7 11 -13 0.21 0.08

7 12 -13 0.04 0.04

-7 12 -13 0.03 0.04

-7 13 -13 0.09 0.03

7 13 -13 0.10 0.03

7 0 -14 0.13 0.15

7 0 14 0.04 0.16

7 -1 -14 24.28 1.12

7 -1 14 24.57 1.10

7 1 14 22.80 1.06

7 1 -14 23.32 1.08

7 -2 -14 2.10 0.35

7 2 -14 1.59 0.29

7 -2 14 1.89 0.32

7 2 14 1.84 0.32

7 -3 -14 7.79 0.65

7 -3 14 7.44 0.62

7 3 14 7.21 0.60

7 3 -14 7.22 0.60

7 3 -14 8.03 0.30

7 3 14 8.69 0.33

-7 3 14 8.20 0.33

-7 3 -14 8.25 0.32

7 4 -14 1.09 0.26

7 -4 14 0.71 0.23

7 4 14 0.92 0.23

-7 -4 -14 1.03 0.25

7 -4 -14 1.09 0.24

7 4 -14 1.15 0.13

-7 4 -14 1.21 0.13

-7 4 14 1.14 0.14

7 -5 14 3.34 0.43

7 5 -14 3.52 0.42

7 -5 -14 3.52 0.44

7 5 -14 3.78 0.23

-7 5 -14 3.24 0.22

-7 5 -14 3.40 0.19

-7 5 14 3.56 0.23

7 6 -14 0.32 0.13

7 6 -14 0.46 0.10

-7 6 -14 0.48 0.09

-7 6 14 0.31 0.09

-7 6 -14 0.48 0.10

7 6 -14 0.40 0.09

7 7 -14 4.53 0.27

-7 7 -14 3.84 0.25

-7 7 -14 4.33 0.23

-7 8 -14 0.07 0.07

7 8 -14 0.07 0.09

-7 8 -14 0.03 0.08

-7 9 -14 1.72 0.16

7 9 -14 2.18 0.19

7 10 -14 0.27 0.07

-7 10 -14 0.27 0.07

-7 10 -14 0.25 0.08

7 10 -14 0.42 0.11

-7 11 -14 0.20 0.06

7 11 -14 0.13 0.06

7 11 -14 0.10 0.07

7 12 -14 0.38 0.06

-7 12 -14 0.39 0.06

7 0 -15 -0.03 0.16

7 0 15 -0.09 0.13

7 -1 -15 3.02 0.43

7 -1 15 2.51 0.38

7 1 15 2.16 0.35

7 1 -15 2.85 0.41

7 1 -15 2.73 0.16

7 -2 -15 0.78 0.22

7 -2 15 0.88 0.24

7 2 15 0.84 0.23

7 2 -15 0.88 0.25

7 2 -15 0.94 0.11

-7 2 -15 0.94 0.12

7 2 15 1.04 0.12

7 3 -15 1.01 0.25

7 -3 15 1.14 0.26

7 3 15 1.02 0.25

7 -3 -15 1.06 0.27

7 3 -15 1.43 0.14

-7 3 15 1.61 0.16

-7 3 -15 1.43 0.14

7 4 -15 2.84 0.40

7 4 15 3.23 0.41

7 -4 -15 3.38 0.46

-7 -4 -15 2.95 0.40

7 -4 15 2.96 0.42

-7 4 -15 3.53 0.22

7 4 -15 3.75 0.22

-7 4 15 3.88 0.25

7 -5 -15 0.38 0.17

7 -5 15 0.02 0.14

7 5 -15 0.25 0.09

-7 5 15 0.46 0.11

-7 5 -15 0.34 0.09

7 6 -15 0.94 0.23

7 6 -15 1.29 0.15

-7 6 -15 1.27 0.15

-7 6 -15 1.28 0.13

-7 6 15 1.13 0.15

7 6 -15 0.95 0.12

7 7 -15 0.29 0.10

-7 7 -15 0.24 0.07

-7 7 -15 0.21 0.09

7 7 -15 0.20 0.07

7 8 -15 0.10 0.09

-7 8 -15 0.10 0.06

-7 8 -15 0.13 0.08

-7 9 -15 0.27 0.08

7 9 -15 0.27 0.10

-7 10 -15 0.50 0.09

7 10 -15 0.39 0.08

-7 10 -15 0.52 0.09

7 10 -15 0.60 0.11

-7 11 -15 0.09 0.05

7 11 -15 0.08 0.05

7 11 -15 0.08 0.06

-7 11 -15 0.06 0.04

-7 12 -15 0.05 0.03

7 12 -15 0.08 0.04

7 0 -16 0.06 0.14

7 0 16 -0.09 0.17

7 0 -16 0.04 0.05

7 1 -16 6.93 0.65

7 -1 -16 8.01 0.70

7 -1 16 7.64 0.68

7 1 16 7.76 0.67

7 1 -16 8.16 0.28

7 1 16 7.79 0.31

7 2 -16 0.31 0.19

7 -2 -16 0.11 0.14

7 -2 16 0.25 0.17

7 2 16 0.22 0.16

7 2 -16 0.39 0.08

-7 2 -16 0.32 0.09

7 2 16 0.27 0.08

7 3 16 8.99 0.72

7 3 -16 8.45 0.71

-7 -3 -16 8.99 0.72

7 -3 -16 8.74 0.74

7 -3 16 9.09 0.75

7 3 -16 9.62 0.34

-7 3 -16 10.07 0.37

-7 3 16 9.85 0.39

7 4 -16 0.29 0.12

7 -4 -16 0.33 0.17

-7 -4 -16 0.29 0.16

7 -4 16 0.26 0.14

7 4 -16 0.43 0.10

-7 4 -16 0.37 0.09

-7 4 16 0.54 0.12

7 -5 -16 2.04 0.36

-7 5 -16 2.48 0.20

7 5 -16 2.52 0.20

-7 5 16 2.65 0.22

-7 6 -16 1.14 0.15

7 6 -16 1.19 0.15

-7 6 -16 1.07 0.12

-7 6 16 1.04 0.15

7 6 -16 1.02 0.13

-7 7 -16 2.42 0.20

-7 7 -16 2.32 0.17

7 7 -16 1.91 0.16

7 7 -16 2.49 0.21

7 8 -16 0.61 0.12

-7 8 -16 0.60 0.11

7 9 -16 0.05 0.09

-7 9 -16 -0.05 0.07

7 10 -16 0.14 0.06

-7 10 -16 0.16 0.06

-7 10 -16 0.10 0.06

7 10 -16 0.22 0.08

7 11 -16 0.10 0.04

-7 11 -16 0.03 0.04

-7 11 -16 0.04 0.04

7 11 -16 0.16 0.06

-7 12 -16 0.16 0.04

7 12 -16 0.15 0.04

7 0 -17 -0.06 0.13

7 0 17 -0.02 0.16

7 0 -17 -0.09 0.06

7 1 -17 0.56 0.24

7 -1 -17 0.28 0.18

7 -1 17 0.53 0.22

7 1 17 0.43 0.18

7 1 -17 0.42 0.08

-7 1 -17 0.54 0.10

7 1 17 0.46 0.09

7 2 -17 1.56 0.35

7 -2 -17 1.08 0.30

7 -2 17 1.60 0.35

7 2 17 1.08 0.29

-7 -2 -17 1.40 0.31

7 2 -17 1.54 0.14

7 2 17 1.41 0.15

-7 2 17 1.59 0.17

-7 2 -17 1.49 0.16

7 3 -17 0.07 0.16

7 -3 17 0.39 0.21

7 3 17 -0.08 0.16

7 -3 -17 0.18 0.14

-7 -3 -17 0.51 0.18

7 3 -17 0.33 0.08

-7 3 17 0.22 0.10

-7 3 -17 0.26 0.08

7 4 -17 0.13 0.14

7 -4 -17 0.23 0.16

7 4 -17 0.48 0.10

-7 4 -17 0.42 0.10

-7 4 17 0.41 0.11

7 5 -17 0.67 0.12

-7 5 -17 0.48 0.11

-7 5 17 0.58 0.13

-7 6 -17 1.51 0.16

7 6 -17 1.57 0.17

-7 6 17 1.69 0.18

7 6 -17 1.32 0.14

7 7 -17 0.31 0.09

-7 7 -17 0.32 0.07

-7 7 -17 0.35 0.10

7 7 -17 0.30 0.07

7 8 -17 1.67 0.17

-7 8 -17 1.35 0.15

7 9 -17 0.13 0.08

-7 9 -17 0.00 0.06

7 10 -17 0.11 0.05

-7 10 -17 0.04 0.05

7 10 -17 0.15 0.07

-7 10 -17 0.01 0.05

7 11 -17 0.02 0.03

-7 11 -17 -0.01 0.03

-7 11 -17 -0.04 0.04

7 11 -17 -0.03 0.05

7 0 -18 -0.09 0.17

7 0 18 -0.08 0.14

-7 0 -18 -0.02 0.05

7 1 -18 0.26 0.16

7 -1 -18 0.12 0.12

7 -1 18 0.38 0.18

7 1 18 0.32 0.16

-7 -1 -18 0.09 0.12

7 1 -18 0.27 0.06

7 1 18 0.19 0.07

-7 1 -18 0.26 0.08

7 2 -18 0.40 0.15

7 -2 -18 0.39 0.22

7 -2 18 0.30 0.19

-7 -2 -18 0.38 0.20

7 2 -18 0.32 0.08

-7 2 -18 0.31 0.09

-7 2 18 0.35 0.10

-7 3 -18 2.05 0.18

7 3 -18 2.26 0.18

-7 3 18 2.49 0.22

7 4 -18 0.84 0.13

-7 4 -18 0.84 0.13

-7 4 18 0.88 0.15

7 5 -18 0.67 0.11

-7 5 -18 0.72 0.12

-7 5 18 0.75 0.14

7 5 -18 0.70 0.12

7 6 -18 0.40 0.10

7 6 -18 0.56 0.10

-7 6 18 0.67 0.14

-7 6 -18 0.35 0.09

7 7 -18 0.42 0.10

-7 7 -18 0.43 0.10

7 7 -18 0.40 0.08

7 8 -18 0.32 0.09

-7 8 -18 0.27 0.09

7 9 -18 0.15 0.07

-7 9 -18 0.07 0.06

-7 10 -18 0.20 0.05

7 10 -18 0.18 0.05

7 10 -18 0.28 0.07

-7 10 -18 0.19 0.05

7 11 -18 0.01 0.03

-7 11 -18 0.01 0.03

-7 0 -19 0.18 0.08

7 1 -19 0.29 0.07

-7 1 -19 0.50 0.10

-7 1 19 0.31 0.10

7 1 19 0.30 0.08

-7 -1 -19 0.44 0.09

7 2 -19 0.12 0.07

-7 2 -19 0.14 0.08

-7 2 19 0.07 0.08

7 3 -19 0.01 0.06

-7 3 19 0.01 0.08

-7 3 -19 0.00 0.08

7 4 -19 0.13 0.07

-7 4 -19 -0.10 0.09

-7 4 19 0.02 0.09

7 5 -19 0.51 0.10

-7 5 19 0.54 0.13

7 5 -19 0.40 0.09

-7 5 -19 0.48 0.10

7 6 -19 0.19 0.08

-7 6 -19 0.16 0.09

7 6 -19 0.23 0.08

7 7 -19 0.42 0.09

7 7 -19 0.22 0.07

-7 7 -19 0.38 0.10

-7 8 -19 -0.05 0.07

7 8 -19 0.06 0.07

-7 9 -19 -0.04 0.05

7 9 -19 -0.02 0.05

7 10 -19 -0.03 0.04

-7 10 -19 0.08 0.04

7 10 -19 0.10 0.05

-7 10 -19 0.03 0.04

-7 0 20 -0.03 0.07

-7 0 -20 0.06 0.07

-7 1 20 0.26 0.10

-7 1 -20 0.24 0.08

-7 -1 -20 0.38 0.08

7 2 -20 0.25 0.07

-7 2 20 0.21 0.09

-7 2 -20 0.25 0.08

7 3 -20 0.21 0.07

-7 3 -20 0.01 0.07

-7 3 20 -0.07 0.09

7 4 -20 0.04 0.06

-7 4 -20 -0.07 0.08

7 5 -20 0.23 0.08

7 5 -20 0.19 0.07

-7 5 -20 0.30 0.10

-7 5 20 0.41 0.11

7 6 -20 1.01 0.12

7 6 -20 1.03 0.12

-7 6 -20 1.16 0.14

7 7 -20 0.02 0.06

7 7 -20 0.08 0.05

-7 7 -20 0.12 0.07

-7 8 -20 0.24 0.07

7 8 -20 0.31 0.07

7 9 -20 0.05 0.04

-7 9 -20 0.05 0.04

7 9 -20 0.16 0.05

7 10 -20 0.17 0.04

-7 10 -20 0.17 0.04

-7 10 -20 0.15 0.04

7 10 -20 0.23 0.05

-7 0 -21 0.07 0.07

-7 0 21 -0.09 0.09

-7 1 21 0.14 0.08

-7 -1 21 0.17 0.08

-7 -1 -21 0.21 0.07

-7 1 -21 0.22 0.08

7 2 -21 0.43 0.08

-7 2 -21 0.43 0.10

-7 2 21 0.42 0.12

7 3 -21 -0.06 0.07

-7 3 -21 0.04 0.07

-7 3 21 0.08 0.09

7 4 -21 0.18 0.06

7 4 -21 0.17 0.07

-7 4 21 0.37 0.11

-7 4 -21 0.16 0.08

7 5 -21 0.26 0.07

-7 5 -21 0.15 0.08

7 5 -21 0.23 0.07

7 6 -21 0.29 0.07

7 6 -21 0.32 0.07

-7 6 -21 0.38 0.09

7 7 -21 0.13 0.05

-7 7 -21 0.06 0.06

7 7 -21 0.11 0.05

-7 8 -21 0.10 0.05

7 8 -21 0.21 0.06

7 9 -21 0.02 0.03

-7 9 -21 0.06 0.03

7 9 -21 0.04 0.03

-7 0 22 -0.05 0.08

-7 0 -22 0.05 0.06

-7 1 22 0.03 0.08

-7 -1 22 -0.01 0.06

-7 1 -22 0.02 0.06

-7 -1 -22 0.03 0.05

-7 -2 22 -0.01 0.06

-7 2 -22 -0.06 0.07

-7 2 22 -0.03 0.09

7 3 -22 0.23 0.06

-7 3 -22 0.31 0.09

-7 -3 22 0.37 0.08

-7 3 22 0.36 0.10

7 4 -22 -0.02 0.05

-7 4 -22 0.00 0.07

-7 -4 22 0.05 0.04

-7 4 22 0.05 0.08

7 4 -22 -0.06 0.08

7 5 -22 0.03 0.05

7 5 -22 0.10 0.06

7 6 -22 0.28 0.07

7 6 -22 0.46 0.08

-7 6 -22 0.37 0.09

-7 7 -22 0.03 0.05

7 7 -22 0.01 0.04

7 7 -22 0.02 0.03

-7 8 -22 -0.04 0.04

7 8 -22 0.12 0.06

-7 0 23 -0.08 0.07

-7 0 -23 0.03 0.05

-7 -1 -23 0.02 0.04

-7 1 23 0.04 0.07

-7 1 -23 0.03 0.05

-7 -1 23 0.00 0.06

-7 -2 23 0.10 0.07

-7 2 23 0.17 0.09

-7 2 -23 0.19 0.08

-7 -3 23 -0.01 0.05

-7 3 -23 -0.06 0.07

-7 3 23 0.06 0.08

7 3 -23 -0.14 0.08

7 4 -23 0.26 0.07

-7 4 23 0.28 0.09

-7 4 -23 0.26 0.07

-7 -4 23 0.25 0.06

7 5 -23 0.26 0.06

7 5 -23 0.27 0.07

-7 5 -23 0.25 0.08

7 6 -23 0.18 0.05

7 6 -23 0.15 0.05

-7 6 -23 0.23 0.07

7 7 -23 0.01 0.03

-7 7 -23 -0.04 0.04

7 7 -23 -0.04 0.03

-7 8 -23 0.13 0.04

-7 0 -24 -0.07 0.05

-7 0 24 0.07 0.07

-7 1 -24 -0.05 0.06

-7 -1 -24 -0.04 0.04

-7 1 24 0.00 0.06

-7 -1 24 -0.01 0.06

7 2 -24 0.03 0.06

-7 2 24 0.17 0.08

-7 2 -24 0.03 0.05

-7 -2 24 0.11 0.05

7 3 -24 -0.10 0.07

-7 3 24 0.06 0.07

-7 3 -24 -0.07 0.06

-7 -3 24 -0.03 0.05

-7 4 -24 0.36 0.07

7 4 -24 0.18 0.06

-7 5 -24 0.10 0.05

7 5 -24 0.10 0.05

-7 6 -24 -0.02 0.04

7 6 -24 0.02 0.03

-7 7 -24 0.20 0.04

-7 0 -25 -0.07 0.05

-7 0 25 -0.01 0.05

7 1 -25 0.03 0.06

-7 -1 25 0.09 0.05

-7 1 -25 0.06 0.04

-7 1 25 0.12 0.06

-7 -2 25 0.07 0.04

7 2 -25 0.01 0.05

-7 2 25 0.11 0.06

-7 2 -25 0.05 0.04

-7 -3 25 -0.01 0.04

-7 3 -25 0.03 0.04

7 3 -25 0.02 0.04

7 4 -25 0.05 0.04

-7 4 -25 0.12 0.05

7 5 -25 0.08 0.04

-7 5 -25 0.07 0.04

-7 6 -25 0.06 0.03

-7 0 26 0.01 0.04

7 0 -26 -0.01 0.04

-7 0 -26 0.01 0.03

7 -1 -26 -0.06 0.05

-7 -1 26 0.00 0.04

-7 1 26 0.01 0.04

-7 1 -26 0.02 0.03

7 1 -26 -0.03 0.05

7 -2 -26 -0.04 0.04

-7 2 -26 -0.01 0.03

-7 -2 26 0.02 0.03

7 2 -26 -0.07 0.04

-7 3 -26 0.23 0.05

7 3 -26 0.25 0.05

7 -3 -26 0.25 0.05

-7 4 -26 0.00 0.03

7 4 -26 0.00 0.03

7 0 -27 -0.01 0.03

7 1 -27 0.15 0.04

7 -1 -27 0.18 0.04

7 2 -27 0.08 0.04

8 0 0 0.15 0.07

8 1 0 1.12 0.08

8 1 0 1.11 0.14

8 -1 0 0.99 0.14

8 2 0 0.62 0.06

8 2 0 0.59 0.07

8 -2 0 0.67 0.13

8 2 0 0.68 0.10

-8 2 0 0.58 0.05

8 3 0 2.57 0.15

8 3 0 2.76 0.18

8 -3 0 2.53 0.22

8 -3 0 2.74 0.21

-8 3 0 2.51 0.13

8 4 0 2.15 0.16

8 4 0 2.32 0.19

8 -4 0 2.32 0.19

8 4 0 2.60 0.20

-8 4 0 2.30 0.15

8 5 0 10.12 0.39

-8 5 0 9.68 0.37

8 5 0 10.20 0.44

8 5 0 10.08 0.37

8 6 0 5.25 0.31

-8 6 0 5.29 0.31

8 6 0 5.38 0.36

-8 6 0 5.27 0.35

8 7 0 9.58 0.46

-8 7 0 9.61 0.46

8 7 0 10.44 0.54

-8 7 0 9.94 0.53

-8 8 0 0.00 0.05

8 8 0 0.07 0.05

8 8 0 -0.03 0.10

-8 8 0 -0.05 0.06

-8 8 0 0.10 0.09

8 8 0 0.07 0.08

8 9 0 0.16 0.05

8 9 0 0.00 0.07

-8 9 0 0.10 0.05

8 9 0 0.11 0.05

-8 9 0 0.10 0.05

-8 9 0 -0.02 0.06

8 9 0 -0.03 0.10

-8 9 0 0.16 0.11

-8 9 0 0.12 0.11

8 9 0 0.14 0.10

8 10 0 0.62 0.09

8 10 0 0.53 0.10

-8 10 0 0.68 0.11

-8 10 0 0.72 0.10

8 10 0 0.70 0.08

-8 10 0 0.75 0.11

8 10 0 0.55 0.15

-8 10 0 0.61 0.19

-8 11 0 0.47 0.09

8 11 0 0.42 0.09

-8 11 0 0.47 0.08

8 11 0 0.44 0.08

8 12 0 2.87 0.19

-8 12 0 2.30 0.17

8 12 0 2.46 0.17

-8 12 0 2.63 0.17

8 13 0 0.16 0.07

-8 13 0 -0.02 0.05

-8 13 0 0.03 0.05

8 13 0 0.08 0.05

-8 14 0 0.54 0.07

-8 14 0 0.36 0.06

8 14 0 0.39 0.06

8 0 -1 21.17 0.56

8 0 1 20.16 0.55

8 0 1 20.29 0.57

8 1 1 0.52 0.11

8 1 -1 0.76 0.12

8 -1 1 0.56 0.11

8 -1 1 0.49 0.11

8 1 -1 0.67 0.06

8 2 1 7.20 0.24

8 -2 -1 7.13 0.36

8 2 1 7.34 0.32

-8 2 -1 6.92 0.18

8 2 -1 7.19 0.31

8 -2 1 7.07 0.32

8 2 -1 6.97 0.24

8 2 -1 7.13 0.19

8 3 -1 0.51 0.09

8 -3 1 0.58 0.11

8 3 1 0.55 0.09

8 3 1 0.51 0.09

8 -3 -1 0.44 0.10

8 3 -1 0.53 0.09

-8 3 -1 0.52 0.07

8 3 -1 0.50 0.07

-8 3 1 0.55 0.06

8 3 1 0.69 0.08

-8 4 1 1.84 0.13

8 4 1 1.87 0.17

8 4 1 1.74 0.16

8 -4 -1 1.53 0.19

8 -4 1 1.77 0.17

-8 4 1 1.86 0.17

8 -4 -1 1.82 0.17

-8 4 -1 1.64 0.14

8 4 -1 1.77 0.17

8 4 -1 1.71 0.14

8 4 1 1.78 0.15

-8 5 1 3.64 0.22

8 5 1 4.17 0.28

8 -5 -1 3.88 0.23

8 5 -1 3.89 0.24

-8 5 1 3.65 0.26

8 5 1 3.87 0.23

-8 5 -1 3.75 0.24

8 5 -1 3.84 0.28

8 5 1 3.74 0.23

8 5 -1 3.77 0.24

-8 6 1 1.01 0.14

8 6 -1 1.06 0.17

8 6 1 1.12 0.18

8 6 -1 0.92 0.14

-8 6 1 0.95 0.16

-8 6 -1 1.02 0.14

8 6 1 1.26 0.16

8 7 -1 3.00 0.30

8 7 1 2.96 0.29

-8 7 -1 2.90 0.26

-8 7 1 2.78 0.24

-8 7 -1 3.15 0.31

-8 7 1 3.16 0.30

8 7 -1 2.77 0.26

8 7 1 3.05 0.27

8 8 1 0.22 0.10

-8 8 -1 0.34 0.11

8 8 -1 0.19 0.12

8 8 1 0.28 0.13

-8 8 1 0.28 0.11

8 8 -1 0.13 0.08

-8 8 1 0.21 0.12

-8 8 -1 0.13 0.10

-8 8 1 0.15 0.05

-8 8 1 0.32 0.06

-8 8 -1 0.18 0.06

8 8 -1 0.24 0.06

8 8 1 0.32 0.07

8 9 1 0.95 0.12

8 9 -1 0.75 0.08

8 9 1 0.82 0.08

-8 9 1 0.73 0.10

-8 9 -1 0.76 0.10

8 9 -1 0.74 0.10

8 9 1 0.78 0.17

8 9 -1 0.95 0.21

-8 9 -1 0.68 0.15

-8 9 -1 0.64 0.19

8 9 -1 0.82 0.17

-8 9 1 0.73 0.19

-8 9 1 0.74 0.10

-8 9 -1 0.67 0.10

8 9 -1 0.83 0.10

-8 9 1 0.93 0.10

8 9 1 0.78 0.09

-8 9 -1 0.89 0.10

8 10 1 0.83 0.09

-8 10 1 0.84 0.11

-8 10 -1 0.76 0.11

8 10 -1 0.76 0.11

8 10 1 0.70 0.11

8 10 -1 0.88 0.21

8 10 1 0.69 0.18

8 10 -1 0.95 0.19

-8 10 1 0.83 0.20

-8 10 -1 0.93 0.22

-8 10 -1 0.76 0.10

8 10 1 0.88 0.10

-8 10 -1 0.99 0.11

8 10 -1 0.90 0.11

-8 10 1 0.94 0.11

-8 11 1 0.47 0.10

-8 11 -1 0.34 0.09

8 11 -1 0.32 0.09

8 11 1 0.43 0.10

8 11 -1 0.34 0.08

-8 11 -1 0.40 0.08

8 11 1 0.44 0.09

-8 11 1 0.44 0.08

8 12 -1 0.30 0.09

8 12 1 0.40 0.10

-8 12 -1 0.27 0.07

8 12 -1 0.14 0.06

-8 12 1 0.22 0.07

-8 12 -1 0.11 0.06

8 12 1 0.19 0.07

-8 12 1 0.28 0.07

8 13 -1 0.42 0.08

8 13 1 0.43 0.09

-8 13 1 0.35 0.08

-8 13 -1 0.18 0.06

8 13 1 0.26 0.07

-8 13 -1 0.29 0.06

8 13 -1 0.31 0.07

-8 13 1 0.33 0.07

-8 14 -1 0.06 0.04

-8 14 1 0.14 0.05

-8 14 -1 0.03 0.04

8 14 -1 0.06 0.04

8 0 -2 241.69 2.23

8 0 -2 241.16 1.43

8 0 2 246.73 2.23

8 -1 2 14.17 0.46

8 1 -2 14.90 0.47

8 -1 -2 15.72 0.50

8 1 -2 14.30 0.29

8 1 2 13.84 0.46

-8 1 2 12.91 0.19

8 2 2 27.42 0.65

8 2 -2 26.85 0.50

8 -2 2 27.86 0.66

8 -2 -2 27.03 0.69

8 2 -2 27.64 0.62

-8 2 2 25.24 0.42

8 2 -2 26.46 0.38

8 3 2 17.44 0.52

8 3 -2 16.19 0.46

8 -3 -2 16.35 0.57

8 -3 2 17.25 0.53

8 3 2 17.92 0.44

-8 3 -2 16.39 0.39

-8 3 2 17.62 0.42

8 3 -2 15.51 0.37

8 3 -2 16.18 0.45

8 4 2 72.84 1.06

8 4 2 72.07 1.08

-8 4 2 72.55 1.04

8 4 -2 71.61 0.98

8 4 -2 75.17 0.98

8 -4 -2 71.26 1.25

-8 4 -2 72.13 1.00

8 4 -2 72.53 1.12

8 4 2 69.81 0.95

8 5 2 8.03 0.38

-8 5 2 7.59 0.30

8 -5 -2 6.93 0.40

-8 5 2 7.67 0.37

8 5 -2 7.49 0.34

-8 5 -2 7.42 0.35

8 -5 -2 7.38 0.32

8 5 -2 8.28 0.41

8 5 2 7.96 0.34

8 6 2 5.91 0.37

-8 6 -2 5.24 0.32

8 6 -2 5.98 0.38

8 6 -2 5.58 0.33

-8 6 2 5.32 0.34

8 6 2 5.82 0.33

8 7 -2 2.64 0.29

8 7 2 2.59 0.27

-8 7 -2 2.33 0.24

8 7 -2 2.50 0.25

-8 7 2 2.45 0.26

8 7 2 2.33 0.23

8 8 -2 35.64 1.12

-8 8 -2 33.14 1.10

-8 8 -2 32.88 0.98

-8 8 2 31.71 0.52

8 8 -2 35.78 0.62

8 8 2 32.14 0.93

8 8 -2 33.28 0.99

-8 8 2 35.35 0.62

8 8 2 35.06 0.64

8 8 2 32.60 0.50

-8 8 2 32.48 1.02

8 9 -2 4.06 0.37

8 9 -2 4.42 0.22

-8 9 -2 4.12 0.22

8 9 2 4.59 0.24

8 9 2 4.27 0.37

-8 9 2 4.42 0.40

-8 9 2 4.31 0.22

8 9 -2 3.91 0.16

8 9 2 4.17 0.18

8 9 -2 4.69 0.44

-8 9 -2 4.35 0.38

-8 9 -2 4.04 0.42

-8 9 2 4.28 0.23

-8 9 -2 4.30 0.23

8 9 2 4.30 0.20

-8 9 -2 4.42 0.21

-8 9 2 4.46 0.20

8 9 -2 4.36 0.21

-8 10 2 6.58 0.51

-8 10 -2 6.73 0.56

8 10 -2 7.15 0.29

8 10 2 7.09 0.31

-8 10 2 7.00 0.29

-8 10 -2 6.86 0.29

8 10 -2 6.80 0.50

8 10 -2 6.98 0.56

-8 10 -2 6.99 0.30

-8 10 -2 7.70 0.29

8 10 -2 7.25 0.28

8 10 2 6.86 0.26

-8 10 2 7.55 0.28

8 11 -2 2.10 0.17

-8 11 2 2.14 0.17

-8 11 -2 1.87 0.16

8 11 2 2.18 0.18

-8 11 -2 1.98 0.15

8 11 2 2.08 0.15

-8 11 2 2.29 0.16

8 11 -2 1.95 0.15

8 12 2 0.15 0.09

8 12 -2 0.10 0.07

8 12 -2 0.05 0.06

-8 12 -2 0.00 0.06

-8 12 2 -0.02 0.08

-8 12 -2 -0.04 0.07

-8 12 2 0.03 0.06

8 12 2 0.06 0.05

8 13 -2 0.13 0.06

-8 13 2 0.05 0.05

-8 13 -2 0.01 0.05

-8 13 2 0.00 0.04

8 13 2 -0.03 0.05

-8 13 -2 0.10 0.06

8 13 -2 0.08 0.05

8 14 2 0.39 0.07

-8 14 2 0.38 0.06

-8 14 -2 0.19 0.05

-8 14 -2 0.16 0.05

8 14 -2 0.21 0.05

8 0 -3 38.89 0.79

8 0 3 41.19 0.83

8 1 -3 4.24 0.26

8 -1 -3 3.85 0.26

8 1 3 4.27 0.27

8 -1 3 3.98 0.26

8 -2 3 9.12 0.40

8 2 -3 9.05 0.36

8 -2 -3 9.31 0.42

8 2 3 9.33 0.39

8 2 -3 9.30 0.30

-8 2 3 9.31 0.24

8 3 3 6.02 0.31

8 3 -3 5.47 0.28

8 -3 -3 5.55 0.33

8 3 -3 5.97 0.28

-8 3 3 6.10 0.24

8 4 -3 8.71 0.39

8 4 -3 8.39 0.34

8 -4 -3 8.73 0.44

-8 4 -3 8.59 0.35

8 4 -3 7.99 0.32

-8 4 3 8.79 0.34

8 -5 -3 1.25 0.18

8 -5 -3 1.23 0.14

-8 5 -3 1.09 0.15

8 5 -3 1.23 0.15

8 5 -3 1.38 0.18

-8 5 3 1.07 0.15

8 5 3 0.76 0.13

8 -6 -3 5.65 0.37

8 6 -3 5.37 0.33

-8 6 -3 5.13 0.33

8 6 -3 5.83 0.40

-8 6 3 5.53 0.34

8 6 3 5.85 0.33

-8 7 -3 3.17 0.29

8 7 -3 3.17 0.32

8 7 3 3.10 0.27

-8 7 3 2.83 0.28

8 7 -3 2.99 0.27

-8 8 -3 2.67 0.29

8 8 -3 2.73 0.33

-8 8 -3 2.70 0.33

8 8 3 2.81 0.14

-8 8 3 2.79 0.15

8 8 -3 2.64 0.16

8 8 3 2.95 0.28

8 8 3 3.07 0.19

-8 8 3 2.97 0.30

8 8 -3 2.79 0.30

-8 8 -3 2.75 0.16

-8 8 3 2.97 0.18

8 8 -3 2.91 0.16

-8 8 3 3.07 0.16

8 8 3 3.04 0.15

8 9 3 1.49 0.15

-8 9 3 1.43 0.14

8 9 -3 1.34 0.12

-8 9 -3 1.41 0.13

-8 9 -3 1.15 0.12

8 9 3 1.33 0.11

8 9 -3 1.36 0.13

-8 9 -3 1.53 0.24

8 9 -3 1.26 0.24

8 9 -3 1.00 0.20

-8 9 3 1.18 0.22

-8 9 -3 1.29 0.25

-8 9 3 1.40 0.13

8 9 -3 1.21 0.13

-8 9 -3 1.16 0.12

-8 9 3 1.44 0.12

8 9 3 1.45 0.12

-8 10 3 0.19 0.08

8 10 -3 0.16 0.06

-8 10 -3 0.11 0.07

8 10 3 0.07 0.07

8 10 -3 0.20 0.07

-8 10 -3 0.24 0.07

8 10 -3 0.17 0.14

8 10 -3 -0.14 0.14

-8 10 -3 0.44 0.19

-8 10 3 0.03 0.09

8 10 -3 0.18 0.07

-8 10 -3 0.08 0.06

-8 10 3 0.18 0.06

8 10 3 0.15 0.06

-8 11 -3 0.07 0.06

-8 11 3 0.25 0.09

8 11 -3 0.08 0.06

8 11 3 0.11 0.07

8 11 -3 0.11 0.07

-8 11 -3 0.11 0.06

-8 11 3 0.11 0.06

8 11 3 0.08 0.06

8 12 -3 0.60 0.10

8 12 3 0.56 0.11

-8 12 -3 0.30 0.08

8 12 -3 0.37 0.08

-8 12 3 0.28 0.08

-8 12 -3 0.25 0.06

-8 12 3 0.37 0.08

8 12 3 0.35 0.07

8 13 3 0.21 0.08

-8 13 3 0.10 0.07

-8 13 -3 0.02 0.04

8 13 -3 0.19 0.06

8 13 -3 -0.02 0.05

-8 13 -3 -0.02 0.05

-8 14 3 0.04 0.05

-8 14 -3 0.00 0.03

8 14 3 0.13 0.05

-8 14 -3 0.00 0.04

8 14 -3 0.04 0.04

8 0 -4 49.89 0.96

8 0 4 52.54 0.97

8 1 -4 15.20 0.51

8 -1 -4 15.19 0.53

8 1 4 15.10 0.51

8 2 -4 24.87 0.63

8 -2 -4 22.79 0.65

8 3 -4 23.11 0.60

8 -3 -4 21.79 0.67

-8 3 4 23.04 0.47

8 4 -4 4.26 0.25

8 -4 -4 4.59 0.33

8 4 -4 4.46 0.29

-8 4 4 4.51 0.24

8 -5 -4 21.76 0.73

8 5 -4 22.60 0.72

-8 5 -4 21.28 0.64

8 5 -4 21.43 0.61

-8 5 4 20.93 0.59

8 -6 -4 16.01 0.64

8 6 -4 15.35 0.58

-8 6 -4 15.52 0.60

8 6 -4 16.73 0.67

-8 6 4 15.37 0.56

-8 7 -4 0.82 0.17

8 7 -4 1.13 0.20

8 7 -4 0.90 0.16

-8 7 4 0.67 0.15

-8 8 4 2.48 0.14

8 8 -4 2.57 0.15

8 8 4 2.30 0.13

8 8 -4 2.52 0.28

8 8 4 2.42 0.17

8 8 -4 2.44 0.15

-8 8 4 2.54 0.16

-8 8 -4 2.53 0.15

-8 8 4 2.77 0.29

-8 8 -4 1.93 0.25

8 8 -4 2.43 0.31

-8 8 -4 2.44 0.17

8 8 -4 2.38 0.17

-8 8 4 2.72 0.15

8 8 4 2.56 0.14

8 9 -4 3.20 0.34

8 9 -4 3.21 0.18

8 9 4 3.18 0.21

-8 9 4 3.34 0.20

8 9 -4 3.25 0.19

-8 9 -4 3.08 0.18

8 9 4 2.80 0.15

-8 9 -4 2.88 0.18

-8 9 4 2.90 0.32

-8 9 -4 2.77 0.32

8 9 -4 3.53 0.40

-8 9 -4 2.94 0.37

-8 9 -4 2.80 0.19

8 9 -4 2.90 0.19

-8 9 4 2.87 0.19

-8 9 4 3.12 0.17

8 9 4 2.87 0.17

8 10 -4 3.19 0.19

8 10 4 3.08 0.21

-8 10 4 3.33 0.35

8 10 -4 2.99 0.34

-8 10 4 3.68 0.22

-8 10 -4 2.88 0.19

-8 10 -4 3.17 0.40

8 10 -4 3.82 0.43

-8 10 -4 2.95 0.20

8 10 -4 2.96 0.20

8 10 -4 3.00 0.19

-8 10 -4 3.31 0.20

-8 10 4 3.62 0.19

8 10 4 3.17 0.18

-8 11 4 0.53 0.10

-8 11 -4 0.40 0.08

8 11 -4 0.51 0.09

8 11 4 0.47 0.10

8 11 -4 0.57 0.10

-8 11 -4 0.63 0.10

-8 11 -4 0.43 0.09

-8 11 4 0.57 0.09

8 11 4 0.50 0.09

8 12 -4 2.05 0.14

8 12 4 1.91 0.17

-8 12 -4 1.80 0.14

-8 12 4 1.95 0.16

-8 12 -4 2.06 0.16

8 12 -4 1.94 0.15

-8 12 4 1.79 0.14

8 12 4 1.66 0.13

8 13 4 0.44 0.09

-8 13 4 0.31 0.08

-8 13 -4 0.20 0.05

-8 13 -4 0.28 0.07

8 13 -4 0.26 0.07

8 14 4 0.30 0.06

-8 14 -4 0.19 0.04

-8 14 4 0.28 0.06

-8 14 -4 0.25 0.05

8 14 -4 0.24 0.05

8 0 -5 54.73 1.05

8 1 -5 11.97 0.48

8 -1 -5 12.26 0.50

8 -2 -5 3.56 0.29

8 2 -5 3.43 0.25

8 -3 -5 2.63 0.24

8 3 -5 3.16 0.24

8 4 -5 0.69 0.13

8 4 -5 0.62 0.11

8 -4 -5 0.57 0.14

-8 4 5 0.64 0.10

8 -5 -5 6.62 0.42

8 5 -5 7.15 0.42

-8 5 -5 6.66 0.37

-8 5 5 6.78 0.33

8 -6 -5 9.05 0.50

-8 6 -5 8.73 0.47

8 6 -5 9.13 0.46

8 6 -5 9.67 0.53

-8 6 5 8.97 0.42

-8 7 -5 0.17 0.08

8 7 -5 0.22 0.13

-8 7 5 0.22 0.10

8 7 -5 0.18 0.10

-8 8 5 1.56 0.11

8 8 -5 1.44 0.12

8 8 -5 1.66 0.24

8 8 5 1.65 0.14

-8 8 5 1.33 0.21

-8 8 5 1.52 0.13

-8 8 -5 1.59 0.13

8 8 -5 1.61 0.13

8 8 5 1.54 0.11

-8 8 -5 1.56 0.24

8 8 -5 1.74 0.27

8 8 -5 1.47 0.14

-8 8 -5 1.36 0.13

-8 8 5 1.51 0.13

-8 8 5 1.65 0.12

8 8 5 1.52 0.12

8 9 -5 0.98 0.10

-8 9 5 0.99 0.12

-8 9 -5 0.94 0.11

8 9 -5 0.97 0.11

8 9 5 0.95 0.12

-8 9 -5 0.99 0.11

-8 9 -5 0.98 0.25

-8 9 -5 0.73 0.18

8 9 -5 1.43 0.27

-8 9 5 1.00 0.19

8 9 -5 1.16 0.22

8 9 -5 0.92 0.12

-8 9 -5 1.13 0.13

8 9 5 0.91 0.10

-8 9 5 0.95 0.10

-8 10 5 0.62 0.11

8 10 -5 0.54 0.09

-8 10 -5 0.59 0.10

8 10 5 0.40 0.10

-8 10 -5 0.53 0.10

8 10 -5 0.65 0.11

-8 10 5 0.46 0.16

-8 10 -5 0.56 0.19

8 10 -5 0.63 0.18

-8 10 -5 0.49 0.10

8 10 -5 0.49 0.10

-8 10 5 0.57 0.09

8 10 5 0.50 0.09

-8 11 -5 0.13 0.06

8 11 -5 0.19 0.06

8 11 5 0.06 0.07

-8 11 -5 0.18 0.07

8 11 -5 0.24 0.08

-8 11 5 0.04 0.08

-8 11 -5 0.15 0.06

8 11 -5 0.31 0.09

-8 11 5 0.11 0.06

8 11 5 0.09 0.06

8 12 -5 0.41 0.08

8 12 5 0.39 0.10

-8 12 -5 0.35 0.08

-8 12 5 0.22 0.08

8 12 -5 0.38 0.08

-8 12 -5 0.21 0.06

8 13 5 0.42 0.08

8 13 -5 0.32 0.07

-8 13 5 0.22 0.07

-8 13 -5 0.18 0.05

-8 13 -5 0.26 0.07

8 14 5 0.11 0.05

-8 14 5 0.06 0.04

8 14 -5 0.02 0.04

-8 14 -5 0.01 0.04

8 0 -6 51.80 1.08

8 1 -6 99.98 1.53

8 -1 -6 99.32 1.56

8 2 -6 45.62 0.99

8 -2 -6 46.41 1.06

8 -3 -6 5.77 0.38

8 3 -6 6.47 0.36

8 4 -6 36.81 0.86

8 4 -6 38.91 0.90

8 5 -6 7.00 0.35

8 -5 -6 7.35 0.46

8 5 -6 8.20 0.46

-8 5 6 7.03 0.34

8 -6 -6 8.50 0.50

-8 6 -6 8.26 0.47

8 6 -6 9.05 0.53

-8 6 6 8.60 0.41

-8 7 -6 0.15 0.10

8 7 -6 0.24 0.13

-8 7 6 0.13 0.08

8 7 -6 0.19 0.10

-8 8 6 0.87 0.09

8 8 6 0.82 0.11

-8 8 6 0.89 0.10

8 8 6 0.87 0.09

-8 8 -6 0.87 0.19

8 8 -6 0.97 0.22

8 8 -6 0.68 0.16

-8 8 6 0.93 0.18

8 8 -6 0.75 0.11

-8 8 -6 0.89 0.12

-8 8 6 0.82 0.09

8 8 6 0.74 0.08

8 9 -6 4.59 0.21

8 9 -6 4.35 0.41

-8 9 6 5.32 0.25

8 9 6 5.10 0.26

8 9 -6 4.72 0.23

-8 9 -6 4.54 0.23

-8 9 -6 4.13 0.21

-8 9 -6 4.28 0.48

-8 9 -6 4.29 0.42

8 9 -6 4.68 0.47

8 9 -6 4.12 0.24

-8 9 -6 4.11 0.24

-8 9 6 4.97 0.21

8 9 6 4.82 0.21

8 10 -6 12.26 0.35

-8 10 6 12.66 0.39

-8 10 -6 11.14 0.35

8 10 6 11.50 0.40

8 10 -6 12.05 0.38

-8 10 -6 12.02 0.38

-8 10 -6 11.28 0.39

8 10 -6 10.86 0.38

-8 10 6 11.12 0.33

8 10 6 10.50 0.32

8 11 6 0.17 0.08

8 11 -6 0.21 0.06

-8 11 -6 0.25 0.07

-8 11 6 0.09 0.07

8 11 -6 0.17 0.07

-8 11 -6 0.22 0.07

-8 11 -6 0.18 0.07

8 11 -6 0.19 0.08

8 12 -6 1.17 0.10

8 12 6 1.14 0.13

-8 12 6 1.28 0.13

-8 12 -6 1.07 0.10

-8 12 -6 1.18 0.12

8 12 -6 1.19 0.12

8 13 6 0.58 0.09

-8 13 6 0.69 0.09

-8 13 -6 0.57 0.08

8 13 -6 0.61 0.08

8 14 6 0.22 0.05

-8 14 6 0.23 0.05

8 14 -6 0.16 0.04

-8 14 -6 0.14 0.04

8 0 -7 30.38 0.87

8 1 -7 25.30 0.78

8 -1 -7 24.43 0.79

8 2 -7 0.29 0.11

8 -2 -7 0.18 0.11

8 3 -7 0.32 0.11

8 -3 -7 0.33 0.13

8 4 -7 8.53 0.45

8 -4 -7 7.90 0.47

8 5 -7 1.63 0.19

8 -5 -7 1.65 0.23

8 -5 7 1.38 0.21

8 5 7 1.23 0.18

8 5 -7 1.75 0.23

-8 6 -7 0.62 0.15

8 6 -7 0.83 0.18

8 -6 -7 0.61 0.16

8 7 7 0.62 0.09

8 7 -7 0.64 0.17

-8 7 -7 0.49 0.15

8 7 -7 0.47 0.15

8 7 -7 0.47 0.08

-8 7 -7 0.62 0.09

-8 7 -7 0.61 0.09

8 8 7 0.02 0.06

-8 8 7 0.04 0.05

-8 8 -7 0.00 0.09

8 8 -7 0.12 0.12

8 8 -7 0.12 0.09

8 8 -7 -0.03 0.07

-8 8 -7 0.07 0.06

8 8 7 0.01 0.04

-8 8 7 0.03 0.04

8 9 7 1.20 0.14

-8 9 7 1.26 0.13

-8 9 -7 0.74 0.09

8 9 -7 0.96 0.12

-8 9 -7 0.96 0.12

-8 9 -7 0.82 0.20

8 9 -7 1.20 0.26

8 9 -7 1.09 0.23

8 9 -7 0.89 0.13

-8 9 -7 0.93 0.12

8 9 7 0.98 0.10

-8 9 7 1.11 0.11

-8 10 -7 0.81 0.10

8 10 7 0.71 0.12

-8 10 -7 0.78 0.11

8 10 -7 0.75 0.11

-8 10 7 0.83 0.11

8 10 -7 0.81 0.13

-8 10 -7 0.72 0.12

-8 11 -7 0.28 0.06

8 11 7 0.17 0.08

-8 11 7 0.06 0.06

-8 11 -7 0.14 0.07

8 11 -7 0.24 0.08

-8 11 -7 0.09 0.05

8 11 -7 0.27 0.09

8 12 7 0.19 0.08

-8 12 7 0.23 0.08

-8 12 -7 0.32 0.08

8 12 -7 0.30 0.08

8 13 7 0.17 0.06

-8 13 7 0.14 0.06

-8 13 -7 0.11 0.05

8 13 -7 0.09 0.05

8 14 7 0.11 0.04

-8 14 -7 0.03 0.03

8 14 -7 0.03 0.03

8 0 -8 46.99 1.16

8 1 -8 62.05 1.33

8 -1 -8 63.81 1.37

8 2 -8 1.53 0.22

8 -2 -8 1.69 0.24

8 3 -8 2.29 0.25

8 -3 -8 2.82 0.30

8 4 -8 0.43 0.12

8 -4 -8 0.47 0.13

8 -5 -8 7.07 0.48

8 5 -8 7.05 0.42

8 5 -8 7.07 0.45

8 -5 8 6.94 0.47

8 5 8 7.10 0.43

8 6 -8 0.36 0.14

-8 6 -8 0.37 0.13

-8 7 8 0.16 0.05

8 7 8 0.13 0.05

-8 7 -8 0.11 0.10

8 7 -8 0.27 0.13

8 7 -8 0.05 0.06

-8 7 -8 0.23 0.07

-8 7 -8 0.23 0.07

8 8 8 4.95 0.25

-8 8 8 5.36 0.23

-8 8 -8 4.56 0.25

8 8 -8 5.02 0.27

8 8 -8 5.24 0.43

-8 8 -8 4.52 0.43

8 8 -8 5.10 0.49

8 9 8 6.05 0.28

-8 9 8 6.32 0.26

8 9 -8 5.87 0.27

-8 9 -8 6.07 0.27

8 9 -8 5.78 0.30

-8 9 -8 5.55 0.28

8 9 -8 6.24 0.50

-8 9 -8 6.04 0.52

8 9 -8 6.46 0.57

8 10 8 0.37 0.09

-8 10 8 0.36 0.08

-8 10 -8 0.45 0.09

8 10 -8 0.38 0.08

8 10 -8 0.45 0.11

-8 10 -8 0.44 0.10

8 11 8 0.37 0.09

8 11 -8 0.42 0.09

-8 11 8 0.44 0.09

-8 11 -8 0.39 0.09

-8 11 -8 0.32 0.08

8 11 -8 0.42 0.10

8 12 8 0.61 0.10

-8 12 -8 0.65 0.10

-8 12 8 0.60 0.09

8 12 -8 0.75 0.10

8 13 8 0.25 0.06

-8 13 8 0.44 0.07

8 13 -8 0.34 0.06

-8 13 -8 0.40 0.07

-8 14 -8 0.12 0.04

8 14 -8 0.10 0.03

8 0 -9 10.17 0.57

8 1 -9 7.29 0.48

8 -1 -9 7.50 0.49

8 2 -9 0.95 0.19

8 -2 -9 1.03 0.21

8 3 -9 2.43 0.28

8 -3 -9 2.65 0.31

8 -4 -9 6.39 0.47

8 4 -9 6.07 0.43

8 -4 9 6.74 0.47

8 4 9 6.58 0.44

8 -5 -9 3.35 0.34

8 5 9 3.17 0.31

8 5 -9 3.51 0.32

8 5 -9 3.48 0.34

8 -5 9 3.66 0.35

8 6 -9 1.18 0.22

-8 7 9 0.38 0.07

8 7 9 0.45 0.08

-8 7 -9 0.31 0.13

8 7 -9 0.46 0.17

-8 7 -9 0.54 0.09

8 7 -9 0.44 0.09

-8 7 -9 0.54 0.09

-8 8 9 0.23 0.07

8 8 9 0.19 0.07

-8 8 -9 0.23 0.08

8 8 -9 0.11 0.07

-8 8 -9 0.07 0.11

8 8 -9 0.17 0.14

8 8 -9 0.11 0.10

-8 9 9 2.93 0.18

8 9 9 2.58 0.19

-8 9 -9 2.51 0.20

8 9 -9 2.67 0.21

-8 9 -9 2.59 0.35

8 10 -9 0.39 0.09

-8 10 9 0.48 0.09

8 10 9 0.57 0.10

-8 10 -9 0.37 0.08

-8 10 -9 0.44 0.10

8 10 -9 0.49 0.11

8 11 9 0.20 0.07

-8 11 -9 0.21 0.07

8 11 -9 0.23 0.08

-8 11 9 0.23 0.07

-8 11 -9 0.16 0.07

8 11 -9 0.25 0.09

8 12 9 0.17 0.06

8 12 -9 0.23 0.07

-8 12 9 0.20 0.06

-8 12 -9 0.27 0.07

-8 13 9 0.12 0.05

8 13 9 0.11 0.04

-8 13 -9 0.03 0.04

8 13 -9 0.10 0.05

-8 14 -9 0.14 0.03

8 14 -9 0.20 0.04

8 0 -10 9.83 0.59

8 -1 -10 51.54 1.36

8 1 -10 50.53 1.33

8 2 -10 2.44 0.30

8 -2 -10 2.86 0.33

8 3 10 0.94 0.19

8 3 -10 0.87 0.19

8 -3 10 1.25 0.23

8 -3 -10 0.91 0.20

8 4 -10 10.16 0.58

8 -4 -10 9.84 0.61

8 -4 10 10.18 0.61

8 4 10 9.96 0.58

8 -5 -10 15.74 0.78

8 5 -10 16.00 0.72

8 -5 10 16.82 0.81

8 5 10 15.69 0.72

8 6 -10 0.85 0.20

8 6 -10 1.02 0.12

-8 6 -10 0.98 0.11

-8 6 -10 1.01 0.12

-8 7 10 0.28 0.06

8 7 10 0.20 0.06

-8 7 -10 0.28 0.13

8 7 -10 0.43 0.15

-8 7 -10 0.47 0.09

8 7 -10 0.39 0.10

-8 7 -10 0.29 0.08

-8 8 10 4.47 0.21

8 8 10 4.37 0.23

-8 8 -10 4.82 0.25

-8 8 -10 4.46 0.25

8 8 -10 4.38 0.26

-8 8 -10 3.63 0.41

8 8 -10 4.94 0.49

-8 9 10 3.10 0.18

8 9 10 2.80 0.19

-8 9 -10 3.31 0.23

8 9 -10 2.84 0.22

8 10 10 0.41 0.09

8 10 -10 0.35 0.09

-8 10 10 0.45 0.08

-8 10 -10 0.37 0.09

-8 10 -10 0.38 0.10

8 10 -10 0.46 0.12

-8 11 10 1.30 0.12

8 11 10 1.12 0.12

-8 11 -10 1.22 0.13

8 11 -10 1.17 0.12

-8 11 -10 0.95 0.12

8 11 -10 1.02 0.13

-8 12 10 2.23 0.13

8 12 10 2.07 0.14

-8 12 -10 2.35 0.15

8 12 -10 2.31 0.15

8 13 10 0.16 0.05

8 13 -10 0.21 0.05

-8 13 -10 0.16 0.05

8 0 -11 20.46 0.89

8 0 11 22.01 0.90

8 -1 -11 10.16 0.64

8 1 11 9.31 0.58

8 -1 11 9.37 0.59

8 1 -11 9.81 0.61

8 -2 -11 3.48 0.38

8 -2 11 3.51 0.37

8 2 11 2.97 0.34

8 2 -11 3.36 0.37

8 3 -11 3.50 0.37

8 -3 -11 3.89 0.40

8 -3 11 3.62 0.38

8 3 11 3.58 0.37

8 4 -11 1.04 0.20

8 -4 11 0.66 0.18

8 4 11 0.75 0.18

8 -4 -11 1.03 0.23

8 5 -11 3.44 0.36

8 -5 -11 3.38 0.39

8 -5 11 3.48 0.39

8 5 -11 3.97 0.22

-8 5 -11 3.60 0.21

8 6 11 0.54 0.08

8 6 -11 0.54 0.14

-8 6 -11 0.49 0.09

8 6 -11 0.47 0.10

-8 6 11 0.47 0.09

-8 6 -11 0.59 0.10

8 7 11 0.50 0.08

-8 7 11 0.50 0.07

-8 7 -11 0.41 0.12

8 7 -11 0.59 0.21

8 7 -11 0.58 0.12

-8 7 -11 0.63 0.10

-8 7 -11 0.57 0.11

8 8 11 -0.07 0.07

-8 8 11 -0.01 0.04

8 8 -11 -0.01 0.08

-8 8 -11 0.05 0.07

-8 8 -11 0.14 0.08

-8 8 -11 0.06 0.09

8 8 -11 0.00 0.13

8 9 11 0.96 0.11

-8 9 11 0.95 0.10

8 9 -11 1.05 0.15

-8 9 -11 1.04 0.14

8 10 11 0.48 0.09

-8 10 11 0.51 0.08

-8 10 -11 0.51 0.10

8 10 -11 0.43 0.09

-8 10 -11 0.52 0.10

8 10 -11 0.47 0.11

-8 11 11 0.15 0.06

8 11 11 0.10 0.05

-8 11 -11 0.09 0.06

8 11 -11 0.00 0.06

8 11 -11 0.12 0.08

-8 11 -11 0.03 0.06

-8 12 11 0.10 0.04

8 12 11 0.13 0.05

8 12 -11 0.10 0.06

-8 12 -11 0.06 0.05

8 13 11 0.34 0.05

-8 13 -11 0.38 0.06

8 13 -11 0.37 0.06

8 0 -12 43.41 1.36

8 0 12 42.09 1.32

8 -1 -12 42.65 1.36

8 -1 12 39.95 1.29

8 1 -12 41.21 1.32

8 2 -12 10.06 0.65

8 -2 -12 10.04 0.67

8 -2 12 10.56 0.67

8 2 12 9.60 0.63

8 3 -12 5.47 0.49

8 -3 -12 5.52 0.51

8 -3 12 6.09 0.51

8 3 12 5.91 0.50

8 4 -12 15.95 0.82

8 -4 -12 16.76 0.88

8 -4 12 16.45 0.86

8 4 12 16.13 0.82

8 5 -12 4.14 0.41

8 -5 -12 4.09 0.44

8 -5 12 3.88 0.43

8 5 -12 4.50 0.24

-8 5 -12 4.21 0.23

-8 5 12 4.07 0.24

8 6 -12 1.52 0.27

-8 6 -12 1.67 0.14

-8 6 -12 1.70 0.16

8 6 -12 1.76 0.17

-8 6 12 1.62 0.16

8 7 12 2.85 0.18

8 7 -12 3.14 0.22

-8 7 -12 2.73 0.21

-8 7 -12 2.85 0.19

-8 7 -12 2.10 0.32

8 7 -12 2.76 0.37

-8 8 12 2.15 0.14

8 8 12 1.92 0.15

-8 8 -12 2.06 0.17

8 8 -12 1.98 0.19

-8 8 -12 1.89 0.18

-8 9 12 0.99 0.10

8 9 12 0.87 0.11

8 9 -12 0.88 0.15

-8 9 -12 0.73 0.12

-8 10 12 0.81 0.09

8 10 12 0.77 0.10

8 10 -12 0.56 0.10

-8 10 -12 0.62 0.10

-8 10 -12 0.40 0.09

8 10 -12 0.77 0.13

-8 11 12 0.72 0.08

8 11 12 0.81 0.09

8 11 -12 0.74 0.10

-8 11 -12 0.75 0.10

8 11 -12 0.68 0.11

-8 11 -12 0.50 0.09

8 12 -12 0.49 0.07

8 12 12 0.34 0.05

-8 12 -12 0.44 0.07

-8 13 -12 0.09 0.04

8 13 -12 0.17 0.04

8 0 -13 2.09 0.34

8 0 13 1.97 0.33

8 1 -13 4.06 0.45

8 1 13 3.79 0.43

8 -1 -13 4.60 0.48

8 -1 13 4.24 0.45

8 2 -13 9.37 0.66

8 -2 -13 9.46 0.68

8 -2 13 9.77 0.68

8 2 13 9.88 0.67

8 3 -13 3.91 0.43

8 -3 -13 4.07 0.45

8 3 13 4.00 0.43

8 -3 13 3.69 0.43

8 4 -13 3.90 0.43

8 -4 -13 3.72 0.45

8 -4 13 3.54 0.43

8 4 13 3.97 0.43

8 4 -13 4.29 0.22

-8 4 -13 4.39 0.24

-8 4 13 4.24 0.24

8 -5 -13 -0.02 0.13

8 -5 13 -0.06 0.13

8 5 -13 0.22 0.11

8 5 -13 0.04 0.06

-8 5 -13 0.02 0.04

-8 5 13 0.05 0.07

-8 5 -13 0.12 0.06

8 6 -13 0.14 0.11

8 6 -13 0.26 0.09

-8 6 -13 0.27 0.07

8 6 -13 0.30 0.08

-8 6 -13 0.37 0.09

-8 6 13 0.30 0.09

-8 7 -13 0.77 0.22

8 7 -13 0.81 0.22

8 7 -13 1.07 0.15

-8 7 -13 1.03 0.12

-8 7 -13 0.81 0.12

8 8 13 0.44 0.08

-8 8 -13 0.51 0.11

8 8 -13 0.40 0.11

-8 9 13 0.11 0.05

8 9 13 0.02 0.04

8 9 -13 0.09 0.08

-8 9 -13 0.09 0.07

8 10 -13 0.28 0.07

8 10 13 0.42 0.08

-8 10 13 0.43 0.07

-8 10 -13 0.33 0.08

8 10 -13 0.42 0.10

-8 10 -13 0.29 0.09

8 11 13 0.28 0.06

8 11 -13 0.21 0.06

-8 11 -13 0.19 0.06

-8 11 -13 0.10 0.05

8 11 -13 0.20 0.07

-8 12 -13 0.09 0.05

8 12 -13 0.17 0.05

8 13 -13 0.04 0.03

-8 13 -13 0.04 0.03

8 0 -14 0.07 0.15

8 0 14 0.27 0.15

8 1 -14 20.83 1.03

8 -1 -14 21.42 1.07

8 1 14 20.11 1.01

8 -1 14 20.78 1.02

8 2 -14 22.25 1.06

8 -2 -14 23.78 1.13

8 2 14 22.08 1.04

8 -2 14 23.27 1.10

8 2 -14 24.62 0.50

-8 2 -14 25.07 0.54

8 3 -14 5.41 0.52

8 -3 -14 5.94 0.57

8 -3 14 5.69 0.55

8 3 14 5.12 0.51

8 3 -14 6.57 0.27

-8 3 -14 6.24 0.28

8 4 -14 0.08 0.12

8 -4 -14 0.02 0.12

8 -4 14 0.19 0.12

8 4 14 0.05 0.11

-8 -4 -14 0.25 0.15

8 4 -14 0.13 0.07

-8 4 14 0.24 0.08

-8 4 -14 0.15 0.07

8 -5 -14 1.89 0.33

8 -5 14 1.68 0.31

8 5 -14 2.20 0.18

-8 5 -14 2.12 0.18

-8 5 14 1.77 0.17

8 6 -14 4.49 0.46

-8 6 -14 4.48 0.26

-8 6 -14 4.41 0.22

8 6 -14 4.99 0.28

8 6 -14 3.56 0.22

-8 6 14 4.21 0.26

8 7 -14 2.42 0.21

-8 7 -14 2.17 0.16

-8 7 -14 2.10 0.19

8 8 -14 0.36 0.10

-8 8 -14 0.31 0.10

-8 9 -14 -0.04 0.07

8 9 -14 0.03 0.08

-8 10 -14 0.80 0.10

8 10 -14 0.83 0.11

-8 10 -14 0.72 0.11

8 10 -14 0.81 0.12

-8 11 -14 0.04 0.05

8 11 -14 0.06 0.05

8 11 -14 0.12 0.06

-8 11 -14 0.02 0.05

-8 12 -14 0.16 0.04

8 12 -14 0.23 0.05

8 0 -15 0.64 0.22

8 0 15 0.43 0.19

8 0 -15 0.59 0.08

8 1 -15 0.71 0.24

8 -1 -15 0.48 0.21

8 -1 15 0.43 0.17

8 1 15 0.51 0.21

8 1 -15 0.38 0.07

8 -1 -15 0.52 0.07

-8 1 -15 0.66 0.10

8 2 -15 1.69 0.33

8 -2 -15 1.62 0.32

8 -2 15 1.72 0.32

8 2 15 1.42 0.28

8 2 -15 1.80 0.14

-8 2 -15 1.85 0.15

8 2 15 1.91 0.16

8 3 -15 1.58 0.30

8 -3 -15 1.37 0.30

8 -3 15 1.69 0.32

8 3 15 1.70 0.31

8 3 -15 1.96 0.15

-8 3 -15 1.92 0.16

-8 3 15 2.31 0.19

8 4 15 2.81 0.40

8 -4 -15 2.49 0.39

8 4 -15 1.95 0.34

-8 -4 -15 2.91 0.40

8 -4 15 2.47 0.38

-8 4 -15 2.88 0.21

8 4 -15 2.80 0.20

-8 4 15 3.03 0.22

8 -5 -15 0.22 0.15

8 -5 15 0.27 0.13

8 5 -15 0.35 0.09

-8 5 15 0.35 0.10

-8 5 -15 0.46 0.10

8 6 -15 0.05 0.12

8 6 -15 0.34 0.10

-8 6 -15 0.32 0.08

-8 6 -15 0.38 0.11

-8 6 15 0.24 0.08

8 6 -15 0.30 0.08

-8 7 -15 1.08 0.12

8 7 -15 1.06 0.15

-8 7 -15 1.08 0.14

8 8 -15 0.26 0.10

-8 8 -15 0.36 0.10

-8 9 -15 0.07 0.07

8 9 -15 0.06 0.09

-8 10 -15 -0.05 0.06

8 10 -15 -0.01 0.06

-8 10 -15 0.01 0.06

8 10 -15 0.09 0.08

-8 11 -15 0.38 0.07

8 11 -15 0.30 0.06

-8 11 -15 0.13 0.04

8 11 -15 0.35 0.07

8 12 -15 0.09 0.03

-8 12 -15 0.05 0.03

8 0 -16 8.81 0.73

8 0 16 9.59 0.75

8 0 -16 9.14 0.29

8 1 -16 2.90 0.42

8 -1 -16 3.33 0.47

8 -1 16 3.49 0.47

8 1 16 3.20 0.44

8 -1 -16 3.48 0.17

8 1 -16 3.36 0.18

8 1 16 3.54 0.21

-8 1 -16 3.48 0.21

8 2 -16 20.39 1.11

8 -2 -16 21.57 1.16

8 -2 16 22.97 1.18

8 2 16 21.51 1.12

8 2 -16 21.96 0.50

-8 2 -16 21.83 0.53

8 3 -16 3.62 0.47

8 -3 -16 3.78 0.50

-8 -3 -16 4.39 0.52

8 -3 16 3.33 0.46

8 3 16 3.72 0.48

8 3 -16 4.30 0.23

-8 3 -16 4.01 0.24

-8 3 16 4.39 0.26

8 4 -16 1.20 0.28

8 -4 -16 1.16 0.28

8 -4 16 1.42 0.31

8 4 16 1.32 0.27

-8 4 -16 1.82 0.17

8 4 -16 1.76 0.16

-8 4 16 1.79 0.18

8 5 -16 1.53 0.16

-8 5 -16 1.51 0.16

-8 5 16 1.42 0.17

-8 6 -16 3.67 0.20

-8 6 -16 3.90 0.25

8 6 -16 4.05 0.26

8 6 -16 3.42 0.22

-8 6 16 3.87 0.27

8 7 -16 -0.03 0.09

-8 7 -16 0.05 0.08

8 7 -16 0.08 0.05

8 8 -16 0.06 0.09

-8 8 -16 0.10 0.08

-8 9 -16 0.08 0.06

8 9 -16 0.14 0.09

8 10 -16 0.08 0.05

-8 10 -16 0.07 0.05

-8 10 -16 -0.02 0.06

8 10 -16 0.12 0.07

-8 11 -16 0.02 0.04

8 11 -16 -0.04 0.04

8 11 -16 -0.05 0.05

-8 11 -16 -0.08 0.04

8 12 -16 0.05 0.03

-8 12 -16 0.11 0.03

8 0 -17 0.02 0.14

8 0 17 -0.09 0.19

8 0 -17 0.00 0.05

-8 0 -17 -0.02 0.06

8 1 -17 0.46 0.20

8 -1 -17 0.31 0.16

8 -1 17 0.29 0.15

8 1 17 0.28 0.13

8 1 -17 0.44 0.08

8 1 17 0.43 0.08

-8 1 -17 0.42 0.09

8 -2 -17 2.01 0.38

8 2 -17 1.65 0.33

8 -2 17 1.61 0.34

8 2 17 1.91 0.36

8 2 -17 2.04 0.16

-8 2 -17 1.97 0.17

8 3 -17 0.53 0.21

8 -3 -17 0.35 0.17

8 -3 17 0.55 0.21

8 3 17 0.14 0.11

-8 -3 -17 0.46 0.22

8 3 -17 0.60 0.10

-8 3 17 0.62 0.12

-8 3 -17 0.53 0.10

8 4 -17 1.10 0.29

8 -4 -17 1.60 0.32

8 -4 17 0.57 0.19

-8 4 -17 1.14 0.15

8 4 -17 1.10 0.14

-8 4 17 1.33 0.16

8 5 -17 1.22 0.15

-8 5 17 1.25 0.17

-8 5 -17 1.08 0.15

8 6 -17 0.17 0.08

-8 6 17 0.17 0.09

-8 6 -17 0.14 0.08

8 6 -17 0.20 0.07

8 7 -17 0.28 0.09

-8 7 -17 0.26 0.09

8 7 -17 0.32 0.08

8 8 -17 0.11 0.09

-8 8 -17 0.11 0.07

-8 9 -17 -0.02 0.06

8 9 -17 -0.01 0.07

8 10 -17 0.11 0.05

-8 10 -17 0.04 0.04

8 10 -17 0.06 0.05

-8 10 -17 -0.06 0.05

8 11 -17 0.05 0.03

-8 11 -17 0.03 0.03

-8 11 -17 0.01 0.03

8 11 -17 -0.03 0.05

8 0 -18 1.78 0.37

8 0 18 1.90 0.37

8 0 -18 1.71 0.14

-8 0 -18 1.71 0.15

8 -1 -18 0.46 0.21

8 1 -18 0.40 0.21

8 -1 18 0.61 0.25

8 1 18 0.50 0.22

-8 -1 -18 0.37 0.15

8 1 -18 0.45 0.08

-8 -1 -18 0.53 0.09

-8 1 -18 0.48 0.09

8 2 -18 -0.08 0.13

8 -2 -18 -0.35 0.29

8 -2 18 -0.10 0.14

8 2 18 -0.38 0.20

-8 -2 -18 -0.23 0.16

8 2 -18 0.03 0.05

-8 2 18 -0.06 0.09

-8 2 -18 0.00 0.07

8 3 -18 1.22 0.13

-8 3 -18 1.35 0.15

-8 3 18 1.59 0.17

8 4 -18 3.32 0.22

-8 4 -18 3.14 0.23

-8 4 18 3.44 0.25

8 5 -18 0.52 0.11

8 5 -18 0.50 0.10

-8 5 -18 0.49 0.11

-8 5 18 0.46 0.12

-8 6 -18 1.92 0.18

8 6 -18 1.80 0.17

8 6 -18 1.58 0.15

-8 6 18 1.94 0.19

8 7 -18 0.12 0.08

8 7 -18 0.15 0.06

-8 7 -18 0.24 0.09

8 8 -18 -0.01 0.07

-8 8 -18 -0.07 0.08

8 9 -18 0.10 0.07

-8 9 -18 0.08 0.05

8 10 -18 0.14 0.04

-8 10 -18 0.14 0.04

-8 10 -18 0.12 0.05

8 10 -18 0.12 0.05

-8 11 -18 0.02 0.02

8 11 -18 0.09 0.03

-8 0 -19 3.14 0.20

8 1 -19 0.26 0.07

-8 1 19 0.20 0.08

-8 1 -19 0.22 0.08

-8 -1 -19 0.46 0.09

8 2 -19 -0.01 0.06

-8 2 -19 0.00 0.07

-8 2 19 0.02 0.09

8 3 -19 0.42 0.10

-8 3 19 0.48 0.11

-8 3 -19 0.42 0.10

8 4 -19 0.61 0.11

-8 4 -19 0.45 0.10

-8 4 19 0.50 0.12

8 5 -19 -0.13 0.09

-8 5 19 0.03 0.08

-8 5 -19 -0.03 0.09

8 5 -19 -0.06 0.07

8 6 -19 -0.04 0.08

8 6 -19 0.05 0.05

-8 6 19 0.07 0.08

-8 6 -19 -0.09 0.09

8 7 -19 0.16 0.08

-8 7 -19 0.13 0.08

8 7 -19 0.10 0.06

-8 8 -19 0.02 0.06

8 8 -19 0.02 0.06

-8 9 -19 -0.01 0.06

8 9 -19 0.07 0.06

8 10 -19 0.04 0.03

-8 10 -19 0.09 0.03

8 10 -19 0.12 0.05

-8 10 -19 0.00 0.04

-8 0 -20 6.20 0.28

8 1 -20 0.05 0.05

-8 1 -20 0.00 0.06

-8 -1 -20 0.00 0.05

8 2 -20 1.02 0.12

-8 2 -20 1.12 0.14

-8 2 20 1.16 0.15

8 3 -20 0.12 0.07

-8 3 20 0.09 0.09

-8 3 -20 0.05 0.08

8 4 -20 2.13 0.17

-8 4 -20 2.39 0.19

-8 4 20 2.61 0.22

8 5 -20 0.08 0.07

8 5 -20 0.08 0.06

-8 5 20 0.14 0.10

-8 5 -20 0.13 0.09

8 6 -20 -0.04 0.07

8 6 -20 0.15 0.06

-8 6 -20 0.10 0.08

8 7 -20 0.28 0.08

-8 7 -20 0.32 0.09

8 7 -20 0.19 0.06

8 8 -20 0.09 0.05

-8 8 -20 -0.04 0.06

8 9 -20 -0.03 0.04

-8 9 -20 -0.01 0.03

8 9 -20 -0.01 0.04

-8 9 -20 -0.05 0.05

8 10 -20 0.06 0.03

-8 0 -21 0.06 0.05

-8 0 21 0.00 0.09

-8 1 21 0.08 0.08

-8 -1 21 0.04 0.08

-8 -1 -21 0.20 0.06

-8 1 -21 0.20 0.08

8 2 -21 0.32 0.08

-8 2 -21 0.31 0.09

-8 2 21 0.35 0.11

8 3 -21 0.04 0.06

-8 3 -21 -0.05 0.08

-8 3 21 -0.02 0.10

8 4 -21 0.13 0.06

8 4 -21 0.02 0.07

-8 4 21 0.16 0.10

-8 4 -21 0.14 0.07

8 5 -21 0.00 0.06

-8 5 21 0.04 0.08

-8 5 -21 0.10 0.07

8 5 -21 0.08 0.06

8 6 -21 0.03 0.05

-8 6 -21 -0.01 0.06

8 6 -21 0.03 0.05

8 7 -21 0.08 0.06

-8 7 -21 0.04 0.06

8 7 -21 0.03 0.04

8 8 -21 0.01 0.04

-8 8 -21 -0.02 0.05

-8 9 -21 0.04 0.03

8 9 -21 -0.04 0.03

-8 9 -21 -0.08 0.04

8 9 -21 -0.03 0.03

-8 0 -22 1.11 0.12

-8 0 22 1.09 0.14

-8 -1 22 -0.07 0.08

-8 -1 -22 -0.05 0.05

-8 1 22 -0.09 0.09

-8 1 -22 0.06 0.06

-8 -2 22 0.07 0.06

-8 2 22 0.15 0.08

-8 2 -22 0.09 0.07

8 3 -22 0.10 0.05

-8 3 22 0.29 0.10

-8 3 -22 0.08 0.06

-8 -3 22 0.25 0.07

8 4 -22 0.95 0.11

-8 -4 22 0.90 0.10

-8 4 -22 0.93 0.12

-8 4 22 1.01 0.14

8 4 -22 0.92 0.12

8 5 -22 -0.03 0.05

-8 -5 22 -0.03 0.04

8 5 -22 -0.05 0.06

-8 5 -22 0.03 0.06

8 6 -22 0.12 0.05

-8 6 -22 0.14 0.07

8 6 -22 0.15 0.05

-8 7 -22 -0.02 0.05

8 7 -22 0.03 0.04

8 7 -22 -0.01 0.03

-8 8 -22 0.04 0.04

8 8 -22 0.06 0.04

-8 0 23 -0.01 0.07

-8 0 -23 0.04 0.05

-8 -1 -23 -0.10 0.05

-8 -1 23 -0.01 0.06

-8 1 -23 -0.03 0.06

-8 1 23 -0.05 0.08

-8 -2 23 0.91 0.12

-8 2 23 0.91 0.13

-8 2 -23 0.73 0.10

-8 -3 23 -0.01 0.05

-8 3 -23 -0.03 0.07

-8 3 23 -0.02 0.08

8 3 -23 -0.19 0.09

8 4 -23 0.03 0.04

-8 -4 23 0.01 0.04

-8 4 23 0.06 0.08

-8 4 -23 -0.05 0.06

8 4 -23 -0.10 0.07

8 5 -23 0.15 0.05

-8 5 -23 0.20 0.06

8 5 -23 0.09 0.05

8 6 -23 0.29 0.05

-8 6 -23 0.31 0.07

8 6 -23 0.15 0.04

-8 7 -23 0.01 0.04

8 7 -23 -0.05 0.03

-8 8 -23 -0.02 0.03

-8 0 -24 0.62 0.08

-8 0 24 0.76 0.11

-8 1 -24 0.00 0.05

-8 1 24 0.07 0.07

-8 -1 24 -0.02 0.05

-8 -2 24 0.68 0.09

8 2 -24 0.59 0.09

-8 2 -24 0.54 0.08

-8 2 24 0.71 0.11

8 3 -24 0.05 0.05

-8 -3 24 0.10 0.05

-8 3 24 0.09 0.07

-8 3 -24 0.05 0.05

8 4 -24 0.16 0.06

-8 4 -24 0.03 0.05

-8 -4 24 0.07 0.04

-8 5 -24 0.16 0.05

8 5 -24 0.07 0.04

-8 6 -24 0.02 0.04

8 6 -24 -0.02 0.03

-8 7 -24 0.00 0.03

-8 0 25 0.07 0.05

-8 0 -25 0.03 0.03

-8 1 -25 -0.10 0.05

-8 -1 25 -0.04 0.05

-8 1 25 -0.01 0.05

8 1 -25 -0.05 0.06

-8 2 25 0.41 0.08

-8 -2 25 0.34 0.06

-8 2 -25 0.31 0.06

8 2 -25 0.36 0.07

-8 3 -25 0.17 0.05

-8 -3 25 0.16 0.05

8 3 -25 0.04 0.04

8 4 -25 -0.03 0.04

-8 4 -25 -0.02 0.04

-8 5 -25 0.13 0.04

8 5 -25 0.05 0.03

-8 6 -25 -0.02 0.03

-8 0 26 0.35 0.06

8 0 -26 0.31 0.06

8 -1 -26 0.18 0.05

-8 1 -26 0.21 0.04

-8 1 26 0.18 0.05

-8 -1 26 0.21 0.05

8 1 -26 0.18 0.05

8 -2 -26 0.11 0.04

-8 2 -26 0.12 0.04

-8 -2 26 0.08 0.04

8 2 -26 0.12 0.05

8 -3 -26 0.22 0.05

-8 3 -26 0.08 0.04

8 3 -26 0.19 0.05

-8 4 -26 0.07 0.03

8 4 -26 0.07 0.03

8 0 -27 0.48 0.06

8 -1 -27 0.01 0.03

8 1 -27 0.05 0.03

8 2 -27 0.08 0.03

9 0 0 -0.01 0.08

9 1 0 3.34 0.13

9 -1 0 3.67 0.26

9 1 0 3.26 0.24

-9 2 0 4.56 0.11

9 2 0 4.62 0.20

9 -2 0 4.68 0.31

9 2 0 4.83 0.27

9 3 0 1.26 0.10

9 3 0 1.50 0.13

9 -3 0 1.44 0.18

9 -3 0 1.38 0.16

-9 3 0 1.23 0.09

9 4 0 20.45 0.48

-9 4 0 19.59 0.44

9 4 0 21.62 0.58

9 -4 0 20.65 0.59

9 4 0 20.49 0.59

9 5 0 0.22 0.07

-9 5 0 0.32 0.08

9 5 0 0.29 0.10

9 -5 0 0.25 0.08

9 5 0 0.24 0.07

-9 6 0 0.12 0.06

9 6 0 0.14 0.08

9 6 0 0.24 0.09

-9 6 0 0.16 0.08

9 7 0 0.21 0.10

-9 7 0 0.16 0.06

9 7 0 0.19 0.09

-9 7 0 0.10 0.08

-9 8 0 0.06 0.05

9 8 0 0.08 0.05

9 8 0 0.03 0.08

9 8 0 -0.06 0.11

-9 8 0 0.08 0.07

-9 8 0 -0.05 0.09

9 9 0 0.45 0.08

9 9 0 0.45 0.09

-9 9 0 0.30 0.07

-9 9 0 0.44 0.07

-9 9 0 0.32 0.07

9 9 0 0.42 0.13

9 9 0 0.32 0.13

-9 9 0 0.35 0.14

-9 10 0 0.02 0.06

9 10 0 0.04 0.07

-9 10 0 0.08 0.05

9 10 0 0.07 0.05

-9 10 0 0.07 0.06

9 10 0 -0.07 0.11

-9 10 0 0.08 0.09

-9 11 0 0.78 0.12

9 11 0 0.72 0.11

-9 11 0 0.59 0.09

9 11 0 0.70 0.10

-9 12 0 0.50 0.09

9 12 0 0.82 0.12

-9 12 0 0.54 0.09

9 12 0 0.52 0.09

9 13 0 0.92 0.11

-9 13 0 0.74 0.09

-9 13 0 0.63 0.08

9 13 0 0.63 0.08

9 14 0 0.15 0.05

-9 14 0 0.13 0.04

9 0 1 -0.02 0.08

9 0 -1 0.01 0.07

9 0 1 0.00 0.07

9 1 -1 7.22 0.19

9 1 -1 7.32 0.34

9 -1 -1 7.67 0.37

9 1 1 8.13 0.36

9 -1 1 7.69 0.35

9 -1 1 8.50 0.39

9 2 1 13.80 0.34

9 2 -1 14.76 0.47

9 -2 -1 14.29 0.52

9 2 1 14.48 0.48

-9 2 -1 13.38 0.25

9 -2 1 13.83 0.48

9 2 -1 13.98 0.35

9 2 -1 14.51 0.26

9 -3 1 6.32 0.33

9 3 1 6.72 0.27

-9 3 1 6.21 0.18

9 -3 -1 7.02 0.38

9 3 -1 6.71 0.31

9 3 1 6.27 0.32

-9 3 -1 6.38 0.22

9 3 -1 6.86 0.29

9 3 -1 6.75 0.23

9 4 1 12.55 0.46

-9 4 1 12.33 0.33

9 4 1 12.95 0.44

9 -4 -1 12.95 0.54

9 -4 1 12.40 0.47

9 -4 -1 12.07 0.45

-9 4 1 12.99 0.43

-9 4 -1 11.90 0.36

9 4 -1 12.42 0.45

9 4 1 12.76 0.37

9 4 -1 12.65 0.38

9 5 -1 4.03 0.28

9 5 1 3.80 0.27

9 -5 -1 4.18 0.25

-9 5 1 3.79 0.21

9 5 1 3.90 0.25

9 5 -1 4.19 0.27

-9 5 1 3.74 0.27

-9 5 -1 3.84 0.23

9 5 1 3.63 0.23

9 5 -1 4.11 0.25

-9 6 -1 5.55 0.32

9 6 -1 6.02 0.38

9 6 1 6.02 0.37

-9 6 1 5.75 0.30

-9 6 1 5.80 0.37

9 6 -1 5.48 0.32

9 6 1 5.84 0.33

-9 7 -1 3.22 0.27

9 7 -1 3.46 0.32

9 7 1 3.58 0.32

-9 7 1 3.45 0.26

-9 7 -1 3.37 0.33

-9 7 1 3.55 0.32

9 7 -1 3.09 0.27

9 7 1 3.44 0.28

-9 8 1 3.87 0.20

9 8 -1 4.46 0.22

9 8 1 4.07 0.22

9 8 1 4.29 0.34

-9 8 1 3.67 0.35

9 8 -1 4.04 0.33

9 8 1 3.80 0.16

9 8 -1 4.60 0.40

-9 8 -1 4.11 0.33

9 8 1 4.16 0.37

-9 8 -1 4.37 0.39

-9 8 -1 4.28 0.22

9 8 -1 4.07 0.18

9 8 1 3.69 0.17

9 9 1 0.96 0.12

9 9 -1 0.82 0.10

-9 9 1 0.84 0.10

-9 9 -1 0.87 0.11

9 9 -1 0.89 0.20

9 9 1 0.73 0.17

9 9 -1 0.72 0.16

-9 9 1 0.89 0.20

-9 9 -1 1.00 0.21

-9 9 1 1.02 0.12

-9 9 -1 0.79 0.11

9 9 1 0.96 0.10

-9 9 -1 0.93 0.10

9 9 -1 0.94 0.10

-9 9 1 0.96 0.10

9 10 -1 1.06 0.12

9 10 1 0.99 0.12

-9 10 1 1.06 0.13

-9 10 -1 1.10 0.13

9 10 -1 1.29 0.26

9 10 -1 1.01 0.21

-9 10 1 0.99 0.22

-9 10 -1 1.22 0.26

9 10 1 1.04 0.22

-9 10 -1 1.00 0.12

-9 10 1 1.12 0.13

-9 10 -1 1.12 0.11

9 10 -1 1.03 0.11

9 10 1 1.05 0.11

-9 10 1 1.23 0.12

9 11 -1 2.27 0.18

9 11 1 2.45 0.19

-9 11 1 2.18 0.18

-9 11 -1 2.32 0.17

-9 11 -1 2.24 0.16

9 11 -1 2.13 0.16

-9 11 1 2.37 0.16

9 11 1 2.28 0.16

-9 12 -1 0.75 0.11

9 12 -1 0.92 0.12

9 12 1 1.29 0.14

-9 12 1 0.95 0.11

9 12 -1 0.86 0.10

-9 12 -1 0.85 0.10

-9 12 1 0.97 0.10

9 12 1 0.87 0.10

9 13 -1 0.15 0.07

-9 13 1 0.08 0.06

-9 13 -1 0.04 0.05

-9 13 -1 0.01 0.04

9 13 -1 0.05 0.05

-9 13 1 0.06 0.05

9 13 1 0.03 0.04

-9 14 1 0.20 0.05

-9 14 -1 0.14 0.04

-9 14 -1 0.11 0.04

9 14 -1 0.10 0.04

9 0 2 0.10 0.10

9 0 -2 0.01 0.01

9 0 -2 -0.06 0.09

9 1 2 0.43 0.10

9 -1 2 0.45 0.11

9 1 -2 0.40 0.05

9 1 -2 0.34 0.10

9 -1 -2 0.41 0.10

-9 1 2 0.44 0.03

9 2 2 16.93 0.54

9 -2 2 17.29 0.54

9 2 -2 16.66 0.51

9 -2 -2 16.97 0.58

9 2 -2 16.43 0.39

9 2 -2 16.50 0.28

-9 2 2 16.71 0.33

9 3 2 3.33 0.24

9 3 -2 3.38 0.23

9 -3 -2 3.01 0.27

9 -3 2 3.21 0.24

-9 3 -2 3.27 0.17

-9 3 2 3.27 0.18

9 3 -2 3.42 0.21

9 3 -2 3.33 0.16

-9 4 -2 0.15 0.06

9 4 2 0.08 0.06

9 4 -2 0.20 0.09

9 4 2 0.24 0.08

9 -4 -2 0.04 0.09

9 4 -2 0.20 0.06

9 4 2 0.07 0.06

9 4 -2 0.12 0.06

-9 4 2 0.17 0.07

-9 5 -2 0.05 0.05

9 5 -2 -0.09 0.09

9 5 2 0.08 0.08

9 -5 -2 -0.01 0.08

9 5 2 0.12 0.07

9 5 -2 0.03 0.07

-9 5 2 0.01 0.07

9 -5 -2 -0.05 0.05

9 6 2 17.60 0.63

-9 6 -2 16.47 0.57

-9 6 2 16.16 0.60

9 6 2 16.81 0.55

9 6 -2 17.61 0.58

-9 7 -2 0.96 0.15

9 7 -2 0.97 0.19

9 7 2 1.04 0.18

-9 7 2 0.78 0.16

9 7 2 0.96 0.15

9 7 -2 0.88 0.15

-9 7 -2 0.86 0.18

-9 8 2 1.30 0.13

9 8 -2 1.41 0.11

9 8 -2 1.66 0.14

9 8 2 1.40 0.13

9 8 2 1.48 0.20

9 8 -2 1.45 0.21

-9 8 2 1.40 0.22

9 8 2 1.24 0.10

-9 8 -2 1.32 0.11

-9 8 -2 1.33 0.20

9 8 -2 1.39 0.24

-9 8 -2 1.32 0.23

-9 8 -2 1.42 0.13

9 8 2 1.51 0.11

-9 8 2 1.30 0.11

9 9 2 0.90 0.12

9 9 -2 0.81 0.10

-9 9 2 0.82 0.11

-9 9 -2 0.72 0.10

9 9 -2 0.94 0.21

9 9 2 0.73 0.17

9 9 -2 0.64 0.15

-9 9 2 0.71 0.17

-9 9 -2 0.97 0.21

-9 9 2 0.79 0.11

-9 9 -2 0.92 0.11

-9 9 -2 0.98 0.10

9 9 -2 0.84 0.10

-9 9 2 0.93 0.10

9 9 2 0.79 0.09

-9 10 2 0.07 0.06

9 10 -2 0.08 0.07

9 10 2 0.17 0.06

-9 10 2 0.16 0.07

9 10 2 0.05 0.07

-9 10 -2 0.06 0.06

9 10 -2 0.20 0.07

-9 10 -2 0.20 0.07

9 10 -2 0.27 0.13

9 10 -2 0.13 0.11

-9 10 2 0.07 0.11

-9 10 -2 0.34 0.18

-9 10 2 0.09 0.05

-9 10 -2 0.06 0.06

-9 11 2 0.14 0.08

-9 11 -2 0.09 0.07

9 11 -2 -0.01 0.06

9 11 2 -0.02 0.07

9 11 -2 -0.05 0.06

-9 11 -2 -0.06 0.07

-9 11 -2 0.02 0.05

9 11 2 -0.03 0.06

-9 11 2 -0.04 0.06

9 12 -2 1.78 0.15

9 12 2 1.93 0.17

-9 12 -2 1.58 0.14

-9 12 2 1.64 0.15

-9 12 2 1.69 0.13

9 12 2 1.46 0.13

-9 12 -2 1.59 0.13

9 12 -2 1.48 0.13

9 13 -2 0.14 0.06

-9 13 2 0.02 0.05

-9 13 -2 0.04 0.04

-9 13 -2 0.03 0.04

-9 13 2 0.02 0.04

9 13 -2 0.02 0.05

9 13 2 -0.05 0.05

9 14 2 0.25 0.06

-9 14 2 0.17 0.05

-9 14 -2 0.10 0.04

-9 14 -2 0.10 0.04

9 14 -2 0.09 0.04

9 0 -3 -0.05 0.09

9 0 3 -0.01 0.08

9 1 -3 26.68 0.68

9 -1 -3 28.53 0.73

9 1 3 29.36 0.74

9 -1 3 29.34 0.74

9 1 -3 26.76 0.45

9 2 -3 3.88 0.25

9 -2 -3 3.75 0.28

9 2 3 3.98 0.27

9 2 -3 3.68 0.14

9 2 -3 3.73 0.20

9 -2 3 4.01 0.28

-9 2 3 3.88 0.15

9 3 3 3.69 0.26

9 3 -3 3.38 0.22

9 3 -3 3.37 0.23

9 -3 -3 3.89 0.30

9 3 -3 3.56 0.18

-9 3 3 3.71 0.19

9 4 -3 3.46 0.23

9 -4 -3 3.31 0.29

-9 4 -3 3.17 0.21

9 4 -3 3.35 0.20

9 4 -3 3.44 0.25

-9 4 3 3.84 0.22

9 -5 -3 12.43 0.57

9 -5 -3 13.12 0.47

9 5 -3 12.39 0.45

-9 5 -3 12.08 0.46

9 5 -3 13.18 0.53

-9 5 3 12.91 0.47

9 -6 -3 13.46 0.60

-9 6 -3 12.49 0.52

9 6 -3 13.64 0.60

9 6 3 13.87 0.50

9 6 -3 13.26 0.52

-9 6 3 13.22 0.53

-9 7 -3 2.44 0.25

9 7 -3 2.55 0.29

9 7 -3 2.48 0.25

9 7 3 2.58 0.24

-9 7 3 2.34 0.25

9 8 -3 2.77 0.17

-9 8 3 2.47 0.28

9 8 3 2.71 0.18

-9 8 -3 2.49 0.15

9 8 -3 2.60 0.15

9 8 -3 2.48 0.27

-9 8 3 2.72 0.18

9 8 3 2.49 0.13

-9 8 -3 2.58 0.28

9 8 -3 2.81 0.33

-9 8 -3 2.27 0.30

-9 8 -3 2.52 0.17

-9 8 3 2.72 0.18

-9 8 3 2.67 0.15

9 8 3 2.56 0.14

-9 9 3 5.08 0.42

-9 9 -3 5.48 0.24

9 9 -3 5.76 0.25

9 9 3 5.33 0.27

9 9 -3 5.18 0.41

-9 9 3 5.45 0.26

9 9 -3 5.62 0.24

-9 9 -3 5.20 0.25

-9 9 -3 5.45 0.43

9 9 -3 5.58 0.48

-9 9 -3 5.18 0.48

-9 9 3 5.05 0.25

9 9 -3 5.28 0.25

-9 9 -3 5.05 0.25

9 9 3 5.14 0.22

-9 9 3 5.28 0.22

9 10 -3 3.65 0.37

9 10 -3 4.11 0.22

9 10 3 3.84 0.24

-9 10 3 4.02 0.23

-9 10 -3 3.48 0.21

-9 10 -3 3.87 0.46

-9 10 3 3.56 0.37

9 10 -3 4.49 0.45

-9 10 -3 3.80 0.23

-9 10 3 3.77 0.22

9 10 3 3.82 0.20

-9 10 3 4.16 0.20

-9 10 -3 3.90 0.21

9 10 -3 3.96 0.21

-9 11 -3 0.66 0.10

9 11 -3 0.73 0.10

9 11 3 0.49 0.10

-9 11 3 0.67 0.11

-9 11 -3 0.65 0.10

9 11 -3 0.74 0.10

-9 11 -3 0.68 0.11

-9 11 3 0.64 0.09

9 11 3 0.67 0.09

-9 12 -3 0.68 0.10

9 12 -3 1.08 0.12

9 12 3 1.02 0.14

9 12 -3 0.73 0.10

-9 12 -3 0.75 0.10

-9 12 3 0.88 0.12

-9 12 3 0.83 0.10

9 12 3 0.79 0.10

9 13 -3 0.67 0.09

9 13 3 0.79 0.11

-9 13 3 0.58 0.09

-9 13 -3 0.43 0.07

9 13 -3 0.54 0.08

-9 13 -3 0.56 0.08

9 14 3 0.26 0.06

-9 14 3 0.25 0.05

-9 14 -3 0.12 0.04

-9 14 -3 0.10 0.04

9 14 -3 0.08 0.04

9 0 -4 -0.03 0.10

9 0 4 0.07 0.08

9 1 4 1.53 0.18

9 1 -4 1.34 0.18

9 -1 -4 1.40 0.18

9 -1 4 1.50 0.19

9 2 -4 1.91 0.19

9 2 4 1.81 0.19

9 -2 -4 1.85 0.20

9 2 -4 1.79 0.15

9 3 -4 8.25 0.37

9 -3 -4 8.34 0.44

9 3 -4 8.84 0.36

-9 3 4 8.31 0.27

9 4 -4 4.60 0.28

9 -4 -4 4.49 0.34

9 4 -4 4.76 0.25

9 4 -4 4.44 0.29

-9 4 4 4.33 0.24

9 -5 -4 3.46 0.31

9 5 -4 3.04 0.23

-9 5 -4 3.06 0.24

9 5 -4 3.24 0.28

-9 5 4 3.01 0.22

9 -6 -4 20.85 0.76

9 6 -4 20.41 0.66

-9 6 -4 19.71 0.67

9 6 -4 21.24 0.77

-9 6 4 19.15 0.63

-9 7 -4 3.23 0.30

9 7 -4 3.54 0.35

-9 7 4 3.27 0.29

9 7 -4 3.50 0.30

-9 8 -4 4.40 0.21

9 8 -4 4.03 0.20

9 8 4 4.22 0.23

9 8 -4 4.07 0.35

-9 8 4 3.75 0.34

9 8 -4 4.32 0.20

-9 8 4 4.16 0.21

9 8 -4 4.32 0.41

-9 8 -4 4.08 0.37

-9 8 4 3.97 0.21

9 8 -4 4.34 0.22

-9 8 -4 4.16 0.22

9 8 4 4.11 0.18

-9 8 4 4.21 0.19

9 9 -4 0.92 0.10

9 9 4 0.96 0.12

9 9 -4 0.95 0.11

-9 9 4 1.07 0.12

-9 9 -4 0.86 0.10

-9 9 -4 0.90 0.11

-9 9 -4 1.12 0.20

9 9 -4 1.01 0.22

9 9 -4 0.86 0.19

-9 9 -4 0.87 0.20

-9 9 4 0.82 0.18

9 9 -4 0.84 0.11

-9 9 -4 0.95 0.12

-9 9 4 0.93 0.11

-9 9 4 1.04 0.10

9 9 4 0.95 0.10

9 10 -4 1.35 0.13

9 10 4 1.27 0.14

-9 10 4 1.52 0.15

-9 10 -4 1.18 0.13

-9 10 4 1.27 0.23

9 10 -4 1.09 0.20

-9 10 -4 1.35 0.27

-9 10 -4 1.21 0.14

9 10 -4 1.12 0.13

9 10 -4 1.24 0.12

-9 10 4 1.49 0.13

9 10 4 1.49 0.13

-9 10 -4 1.48 0.14

9 11 -4 0.93 0.11

-9 11 4 0.96 0.13

-9 11 -4 0.83 0.11

9 11 4 0.74 0.12

9 11 -4 0.79 0.11

-9 11 -4 0.79 0.11

-9 11 4 0.81 0.10

9 11 4 0.73 0.10

-9 11 -4 1.04 0.12

9 11 -4 0.86 0.11

9 12 -4 2.19 0.15

9 12 4 1.99 0.17

-9 12 4 2.25 0.17

-9 12 -4 1.83 0.14

-9 12 4 1.88 0.14

9 12 -4 1.94 0.15

-9 12 -4 1.99 0.15

9 12 4 1.73 0.13

9 13 -4 0.34 0.06

9 13 4 0.37 0.09

-9 13 4 0.29 0.07

-9 13 -4 0.23 0.06

9 13 -4 0.27 0.06

-9 13 -4 0.23 0.06

9 14 4 0.26 0.06

-9 14 4 0.26 0.05

-9 14 -4 0.16 0.03

9 14 -4 0.18 0.04

-9 14 -4 0.13 0.04

9 0 -5 -0.02 0.09

9 1 -5 67.35 1.20

9 -1 -5 65.36 1.23

9 2 -5 0.57 0.13

9 -2 -5 0.61 0.14

9 3 -5 48.57 0.98

9 -3 -5 47.67 1.08

9 3 -5 50.87 0.91

9 4 -5 2.10 0.20

9 -4 -5 2.44 0.26

9 4 -5 2.72 0.24

-9 4 5 2.68 0.18

9 -5 -5 24.85 0.85

-9 5 -5 24.07 0.71

9 5 -5 26.01 0.69

9 5 -5 25.86 0.80

-9 5 5 25.18 0.64

9 -6 -5 3.98 0.34

9 6 -5 4.34 0.31

-9 6 -5 4.06 0.32

9 6 -5 4.77 0.38

-9 6 5 4.60 0.30

9 7 -5 3.53 0.31

-9 7 -5 3.05 0.30

9 7 -5 3.68 0.36

-9 7 5 3.65 0.30

9 8 -5 1.32 0.11

9 8 5 1.45 0.14

-9 8 5 1.27 0.20

9 8 -5 1.42 0.12

-9 8 5 1.36 0.12

-9 8 -5 1.29 0.12

9 8 -5 1.59 0.27

9 8 -5 1.18 0.20

-9 8 -5 1.38 0.22

9 8 -5 1.22 0.13

-9 8 -5 1.24 0.13

-9 8 5 1.31 0.12

-9 8 5 1.48 0.11

9 8 5 1.36 0.11

9 9 -5 3.58 0.36

9 9 -5 3.61 0.19

9 9 5 3.13 0.21

-9 9 5 3.58 0.35

-9 9 5 3.58 0.21

-9 9 -5 3.60 0.20

9 9 -5 3.59 0.20

-9 9 -5 3.13 0.19

-9 9 -5 3.66 0.37

9 9 -5 3.98 0.43

-9 9 -5 3.29 0.40

-9 9 -5 3.34 0.21

9 9 -5 3.15 0.20

-9 9 5 3.60 0.18

9 9 5 3.40 0.18

9 10 -5 1.09 0.11

-9 10 5 1.24 0.14

9 10 5 0.83 0.12

-9 10 -5 1.07 0.12

-9 10 -5 1.07 0.12

9 10 -5 1.07 0.12

9 10 -5 1.02 0.12

-9 10 -5 0.87 0.12

-9 10 5 1.10 0.11

9 10 5 0.96 0.11

9 11 -5 0.31 0.07

-9 11 -5 0.20 0.07

9 11 5 0.29 0.09

-9 11 -5 0.23 0.07

9 11 -5 0.21 0.07

-9 11 5 0.23 0.08

-9 11 -5 0.26 0.08

9 11 -5 0.29 0.08

9 11 5 0.24 0.07

-9 11 5 0.33 0.07

9 12 -5 0.78 0.09

9 12 -5 0.74 0.10

9 12 5 0.63 0.11

-9 12 5 0.69 0.11

-9 12 -5 0.65 0.09

-9 12 -5 0.66 0.08

9 13 5 0.21 0.07

-9 13 5 0.16 0.07

-9 13 -5 0.16 0.05

-9 13 -5 0.17 0.06

9 13 -5 0.16 0.06

9 14 5 0.17 0.05

-9 14 5 0.31 0.06

-9 14 -5 0.15 0.04

9 14 -5 0.18 0.04

9 0 -6 0.06 0.10

9 1 -6 4.21 0.32

9 -1 -6 4.14 0.33

9 -2 -6 0.33 0.11

9 2 -6 0.43 0.13

9 3 -6 3.23 0.28

9 -3 -6 2.88 0.28

9 4 -6 13.12 0.53

9 -4 -6 12.59 0.60

9 4 -6 14.07 0.54

9 -5 -6 3.30 0.32

9 5 -6 3.23 0.29

-9 5 6 3.29 0.23

-9 6 -6 3.74 0.32

9 -6 -6 3.50 0.34

9 6 -6 3.90 0.31

9 6 -6 4.12 0.36

-9 6 6 4.06 0.28

-9 7 -6 3.83 0.35

9 7 -6 4.44 0.40

9 7 -6 3.85 0.33

-9 7 6 4.01 0.31

-9 8 -6 6.94 0.27

9 8 -6 7.20 0.27

9 8 -6 6.72 0.46

-9 8 6 6.87 0.44

9 8 6 6.93 0.30

-9 8 6 7.36 0.28

-9 8 -6 6.60 0.49

9 8 -6 7.26 0.55

9 8 -6 6.51 0.29

-9 8 -6 6.58 0.29

-9 8 6 6.73 0.23

9 8 6 6.72 0.23

-9 9 -6 1.09 0.12

9 9 -6 0.96 0.10

9 9 6 1.11 0.13

-9 9 6 1.08 0.12

9 9 -6 1.15 0.12

-9 9 -6 0.81 0.10

-9 9 -6 0.98 0.20

9 9 -6 1.18 0.26

9 9 -6 0.88 0.20

-9 9 -6 0.57 0.18

-9 9 -6 1.03 0.13

9 9 -6 0.84 0.12

-9 9 6 1.13 0.11

9 9 6 1.09 0.11

9 10 -6 0.54 0.08

-9 10 6 0.69 0.11

-9 10 -6 0.40 0.08

9 10 6 0.38 0.10

-9 10 -6 0.54 0.09

9 10 -6 0.48 0.09

-9 10 -6 0.37 0.08

9 10 -6 0.50 0.10

-9 10 6 0.48 0.08

9 10 6 0.41 0.08

9 11 -6 0.71 0.09

-9 11 -6 0.72 0.09

9 11 6 0.60 0.11

-9 11 6 0.56 0.10

-9 11 -6 0.72 0.11

9 11 -6 0.67 0.11

9 11 -6 0.81 0.11

-9 11 -6 0.81 0.11

9 12 -6 0.08 0.05

-9 12 -6 0.01 0.05

9 12 6 0.09 0.07

-9 12 6 -0.05 0.07

9 12 -6 0.06 0.06

-9 12 -6 0.01 0.04

9 13 6 0.17 0.07

-9 13 6 0.07 0.05

-9 13 -6 0.05 0.05

9 13 -6 -0.02 0.04

9 14 6 0.18 0.05

-9 14 6 0.17 0.04

-9 14 -6 0.08 0.03

9 14 -6 0.13 0.04

9 0 -7 0.02 0.12

9 1 -7 52.69 1.17

9 -1 -7 53.12 1.21

9 2 -7 6.53 0.41

9 -2 -7 6.43 0.42

9 -3 -7 30.98 0.95

9 3 -7 31.26 0.88

9 -4 -7 11.50 0.58

9 4 -7 12.22 0.53

9 4 -7 11.90 0.54

9 5 -7 5.94 0.36

9 -5 -7 5.85 0.44

9 5 -7 6.02 0.41

9 -6 -7 3.34 0.34

-9 6 -7 3.44 0.32

9 6 -7 3.49 0.34

9 7 7 14.60 0.41

-9 7 7 14.86 0.37

9 7 -7 14.71 0.65

-9 7 -7 13.01 0.67

9 7 -7 15.39 0.76

9 7 -7 14.73 0.43

-9 7 -7 14.86 0.42

9 8 7 5.27 0.26

-9 8 7 5.60 0.24

9 8 -7 5.98 0.44

-9 8 -7 5.08 0.44

9 8 -7 5.77 0.50

-9 8 -7 5.26 0.27

9 8 -7 5.13 0.27

-9 8 7 4.92 0.20

9 8 7 5.05 0.20

9 9 7 5.56 0.28

-9 9 7 5.54 0.25

9 9 -7 5.71 0.25

-9 9 -7 5.74 0.26

-9 9 -7 5.15 0.24

-9 9 -7 5.06 0.27

9 9 -7 4.69 0.26

-9 9 -7 5.00 0.45

9 9 -7 5.60 0.52

-9 10 7 0.05 0.06

-9 10 -7 0.07 0.05

9 10 7 0.08 0.07

-9 10 -7 0.11 0.06

9 10 -7 0.09 0.06

9 10 -7 0.14 0.08

-9 10 -7 0.19 0.08

9 11 7 0.82 0.12

9 11 -7 0.64 0.10

-9 11 -7 0.57 0.08

-9 11 7 0.82 0.10

-9 11 -7 0.72 0.10

9 11 -7 0.62 0.10

-9 11 -7 0.67 0.11

9 12 7 0.46 0.09

-9 12 7 0.48 0.09

-9 12 -7 0.47 0.09

9 12 -7 0.64 0.10

9 13 7 0.09 0.06

-9 13 7 0.14 0.06

-9 13 -7 0.16 0.06

9 13 -7 0.15 0.05

9 14 7 0.22 0.04

-9 14 7 0.32 0.05

-9 14 -7 0.17 0.04

9 14 -7 0.21 0.04

9 0 -8 -0.06 0.12

9 1 -8 5.33 0.40

9 -1 -8 5.33 0.41

9 2 -8 11.97 0.58

9 -2 -8 12.51 0.62

9 3 -8 0.14 0.10

9 -3 -8 0.06 0.11

9 4 -8 0.01 0.08

9 -4 -8 -0.01 0.11

9 5 -8 2.07 0.23

9 -5 -8 2.35 0.30

9 -5 8 2.78 0.30

9 5 8 2.41 0.26

9 5 -8 2.48 0.27

-9 6 -8 0.94 0.19

9 6 -8 0.77 0.18

9 -6 -8 0.88 0.20

-9 7 8 0.32 0.06

9 7 8 0.32 0.07

-9 7 -8 0.32 0.08

-9 7 -8 0.40 0.09

9 7 -8 0.27 0.08

-9 7 -8 0.16 0.10

9 7 -8 0.41 0.16

9 7 -8 0.24 0.10

-9 8 8 1.23 0.12

9 8 8 1.02 0.12

-9 8 -8 1.25 0.14

9 8 -8 1.13 0.14

-9 8 -8 1.25 0.23

9 8 -8 1.30 0.26

9 8 -8 1.35 0.23

-9 9 8 1.49 0.14

9 9 8 1.25 0.14

-9 9 -8 1.13 0.11

-9 9 -8 1.31 0.13

9 9 -8 1.27 0.13

-9 9 -8 1.17 0.14

9 9 -8 1.04 0.14

-9 9 -8 1.16 0.24

9 9 -8 1.21 0.25

9 10 8 0.18 0.07

-9 10 8 0.03 0.07

-9 10 -8 0.23 0.08

9 10 -8 0.22 0.07

9 10 -8 0.35 0.10

-9 10 -8 0.26 0.09

9 11 8 0.27 0.08

-9 11 8 0.36 0.08

-9 11 -8 0.31 0.08

9 11 -8 0.36 0.09

-9 11 -8 0.31 0.08

9 11 -8 0.34 0.09

9 12 8 0.57 0.09

-9 12 -8 0.66 0.09

-9 12 8 0.69 0.10

9 12 -8 0.78 0.10

9 13 8 0.17 0.06

-9 13 8 0.22 0.06

-9 13 -8 0.19 0.05

9 13 -8 0.11 0.05

9 14 -8 0.05 0.03

-9 14 -8 0.11 0.03

9 0 -9 0.04 0.12

9 1 -9 39.48 1.13

9 -1 -9 40.62 1.17

9 2 -9 7.37 0.49

9 -2 -9 8.09 0.52

9 3 -9 12.82 0.63

9 -3 -9 13.71 0.70

9 4 -9 0.81 0.18

9 -4 -9 0.82 0.19

9 5 -9 10.40 0.56

9 -5 -9 10.23 0.62

9 -5 9 10.84 0.63

9 5 9 10.58 0.58

9 5 -9 10.93 0.58

9 6 -9 6.16 0.27

-9 6 -9 6.22 0.27

9 6 -9 6.47 0.49

-9 7 9 9.49 0.29

9 7 9 9.61 0.34

9 7 -9 9.39 0.36

-9 7 -9 9.20 0.35

-9 7 -9 9.65 0.33

9 7 -9 10.01 0.65

-9 7 -9 8.42 0.57

9 7 -9 9.64 0.55

-9 8 9 1.07 0.11

9 8 9 0.87 0.11

-9 8 -9 0.92 0.13

9 8 -9 0.71 0.12

-9 8 -9 0.79 0.18

9 8 -9 1.09 0.24

-9 9 -9 4.22 0.23

9 9 -9 4.29 0.23

-9 9 9 4.34 0.22

9 9 9 4.08 0.24

9 9 -9 3.80 0.25

-9 9 -9 4.23 0.25

-9 10 9 4.09 0.21

9 10 9 3.75 0.23

9 10 -9 3.93 0.22

-9 10 -9 3.96 0.22

9 10 -9 3.29 0.23

-9 10 -9 3.64 0.23

-9 11 9 1.83 0.14

9 11 9 1.47 0.14

-9 11 -9 1.76 0.15

9 11 -9 1.74 0.14

-9 11 -9 1.58 0.15

9 11 -9 1.30 0.14

9 12 9 0.58 0.09

-9 12 9 0.62 0.09

-9 12 -9 0.50 0.08

-9 12 -9 0.70 0.09

9 12 -9 0.79 0.10

9 13 9 0.28 0.06

-9 13 9 0.34 0.06

9 13 -9 0.23 0.05

-9 13 -9 0.23 0.05

9 0 -10 -0.07 0.15

9 1 -10 1.58 0.25

9 -1 -10 1.76 0.28

9 2 -10 0.78 0.18

9 -2 -10 0.86 0.20

9 3 -10 3.81 0.37

9 -3 -10 4.26 0.41

9 -4 10 0.40 0.16

9 4 10 0.53 0.15

9 4 -10 0.56 0.16

9 -4 -10 0.65 0.19

9 5 -10 7.57 0.50

9 -5 -10 7.31 0.54

9 5 -10 7.50 0.50

9 -5 10 7.16 0.54

9 5 10 6.88 0.48

9 6 10 2.82 0.18

9 6 -10 2.81 0.33

-9 6 -10 2.92 0.20

9 6 -10 3.01 0.20

-9 6 -10 3.26 0.18

-9 7 10 0.47 0.07

9 7 10 0.36 0.08

-9 7 -10 0.14 0.09

9 7 -10 0.40 0.16

9 7 -10 0.48 0.10

-9 7 -10 0.63 0.10

-9 7 -10 0.51 0.10

-9 8 10 1.57 0.13

9 8 10 1.58 0.15

-9 8 -10 1.49 0.16

9 8 -10 1.52 0.16

-9 8 -10 1.54 0.26

9 8 -10 1.74 0.30

-9 9 10 3.67 0.20

9 9 10 3.45 0.22

-9 9 -10 3.46 0.23

9 9 -10 3.24 0.23

9 10 10 0.53 0.09

-9 10 10 0.64 0.09

-9 10 -10 0.80 0.11

9 10 -10 0.65 0.10

-9 10 -10 0.68 0.12

9 10 -10 0.72 0.12

9 11 -10 0.19 0.07

9 11 10 0.21 0.07

-9 11 10 0.18 0.06

-9 11 -10 0.27 0.07

-9 11 -10 0.14 0.07

9 11 -10 0.15 0.08

-9 12 10 0.91 0.09

9 12 10 0.74 0.09

-9 12 -10 0.54 0.08

-9 12 -10 0.71 0.09

9 12 -10 0.78 0.09

9 13 10 0.36 0.05

9 13 -10 0.31 0.06

-9 13 -10 0.30 0.05

9 0 -11 -0.02 0.16

9 1 -11 27.13 1.03

9 -1 -11 29.73 1.10

9 2 11 0.62 0.18

9 -2 11 0.81 0.19

9 2 -11 0.48 0.17

9 -2 -11 0.66 0.18

9 3 -11 23.89 0.96

9 -3 -11 26.06 1.05

9 -3 11 26.59 1.04

9 3 11 25.79 0.99

9 4 -11 3.78 0.39

9 -4 -11 3.62 0.41

9 -4 11 3.37 0.38

9 4 11 3.93 0.39

9 5 -11 6.15 0.48

9 -5 -11 5.30 0.47

9 -5 11 6.55 0.54

-9 5 -11 6.53 0.28

9 5 -11 6.52 0.28

9 6 11 2.91 0.18

9 6 -11 2.98 0.36

-9 6 -11 3.25 0.19

-9 6 -11 3.06 0.21

9 6 -11 3.24 0.21

-9 6 11 2.69 0.19

-9 7 11 10.12 0.29

9 7 11 9.87 0.34

-9 7 -11 10.27 0.34

-9 7 -11 9.73 0.37

9 7 -11 10.56 0.40

9 7 -11 10.22 0.68

-9 8 11 2.99 0.17

9 8 11 2.70 0.19

9 8 -11 2.79 0.22

-9 8 -11 2.90 0.21

-9 8 -11 2.40 0.33

9 8 -11 2.76 0.38

-9 9 11 1.91 0.14

9 9 11 1.83 0.16

9 9 -11 1.76 0.18

-9 9 -11 1.89 0.18

9 10 11 0.14 0.06

-9 10 11 0.10 0.05

-9 10 -11 0.02 0.06

9 10 -11 0.06 0.06

-9 10 -11 0.12 0.07

9 10 -11 0.13 0.08

-9 11 11 0.51 0.08

9 11 11 0.55 0.09

-9 11 -11 0.65 0.09

9 11 -11 0.56 0.09

-9 11 -11 0.42 0.08

9 11 -11 0.46 0.10

-9 12 11 0.10 0.04

9 12 11 0.03 0.04

-9 12 -11 0.11 0.05

9 12 -11 0.03 0.05

-9 12 -11 0.08 0.05

9 13 11 0.39 0.05

9 13 -11 0.34 0.05

-9 13 -11 0.34 0.05

9 0 12 -0.04 0.12

9 0 -12 -0.13 0.18

9 1 -12 7.46 0.57

9 -1 -12 7.39 0.58

9 -1 12 7.14 0.56

9 1 12 6.75 0.54

9 2 -12 2.75 0.36

9 -2 -12 2.72 0.36

9 -2 12 2.59 0.35

9 2 12 2.51 0.34

9 -3 12 -0.07 0.15

9 3 12 -0.07 0.15

9 3 -12 -0.14 0.14

9 -3 -12 0.09 0.12

9 4 -12 18.03 0.88

9 -4 -12 17.83 0.93

9 -4 12 18.02 0.91

9 4 12 17.61 0.87

9 4 -12 20.50 0.48

-9 4 -12 20.35 0.49

9 5 -12 1.18 0.23

9 -5 12 0.86 0.23

9 -5 -12 0.94 0.22

-9 5 -12 1.27 0.14

9 5 -12 1.31 0.14

-9 5 12 0.98 0.12

9 6 12 5.21 0.24

9 6 -12 5.53 0.49

-9 6 -12 5.60 0.24

-9 6 -12 5.53 0.28

9 6 -12 5.62 0.28

9 7 12 0.85 0.11

9 7 -12 0.89 0.22

-9 7 -12 0.84 0.22

9 7 -12 1.02 0.14

-9 7 -12 1.08 0.12

-9 7 -12 0.96 0.13

9 8 12 4.87 0.24

-9 8 12 5.23 0.21

9 8 -12 5.34 0.30

-9 8 -12 5.05 0.28

-9 9 12 2.55 0.15

9 9 12 2.48 0.17

-9 9 -12 2.17 0.19

9 9 -12 2.31 0.20

-9 10 -12 0.31 0.08

-9 10 12 0.25 0.06

9 10 12 0.28 0.07

9 10 -12 0.27 0.07

-9 10 -12 0.22 0.08

9 10 -12 0.33 0.10

-9 11 -12 0.01 0.05

-9 11 12 -0.02 0.04

9 11 12 0.06 0.05

9 11 -12 -0.05 0.05

-9 11 -12 -0.06 0.07

9 11 -12 -0.05 0.07

-9 12 -12 0.13 0.05

9 12 12 0.06 0.04

9 12 -12 0.07 0.04

-9 12 -12 0.09 0.04

9 13 -12 0.16 0.04

-9 13 -12 0.09 0.03

9 0 13 0.00 0.14

9 0 -13 0.08 0.16

9 1 -13 19.97 0.98

9 -1 -13 22.80 1.06

9 -1 13 20.93 0.99

9 1 13 19.73 0.95

9 2 -13 2.43 0.34

9 -2 -13 2.85 0.39

9 -2 13 2.47 0.35

9 2 13 2.54 0.34

9 3 -13 22.06 1.02

9 -3 -13 23.51 1.09

9 3 13 22.39 1.02

9 -3 13 23.41 1.07

-9 3 -13 25.18 0.55

9 3 -13 25.34 0.52

9 4 -13 4.95 0.48

9 -4 -13 5.43 0.54

9 -4 13 5.02 0.51

9 4 13 4.66 0.47

-9 4 -13 6.02 0.28

9 4 -13 5.99 0.27

-9 4 13 5.24 0.27

9 -5 -13 3.99 0.46

9 -5 13 4.27 0.47

-9 5 -13 4.17 0.24

9 5 -13 4.29 0.24

-9 5 13 4.26 0.25

9 6 -13 1.11 0.24

-9 6 -13 1.19 0.12

-9 6 -13 1.27 0.14

9 6 -13 1.52 0.16

-9 6 13 1.00 0.13

-9 7 -13 9.28 0.37

-9 7 -13 9.41 0.33

9 7 -13 10.17 0.41

9 7 -13 10.04 0.71

-9 7 -13 8.30 0.64

9 8 13 0.16 0.05

-9 8 -13 0.27 0.09

9 8 -13 0.33 0.11

-9 9 13 1.12 0.10

9 9 13 0.89 0.10

-9 9 -13 0.93 0.13

9 9 -13 1.11 0.15

-9 10 13 1.06 0.09

9 10 13 0.96 0.10

9 10 -13 0.92 0.11

-9 10 -13 0.85 0.10

-9 10 -13 0.80 0.11

9 10 -13 0.85 0.13

9 11 13 0.39 0.06

9 11 -13 0.45 0.08

-9 11 -13 0.36 0.07

-9 11 -13 0.27 0.07

9 11 -13 0.32 0.08

9 12 -13 0.41 0.06

-9 12 -13 0.39 0.06

9 0 -14 -0.14 0.15

9 0 14 0.05 0.13

9 -1 -14 2.14 0.36

9 1 -14 1.59 0.30

9 -1 14 1.84 0.33

9 1 14 2.10 0.35

9 2 -14 0.06 0.12

9 -2 14 0.02 0.14

9 2 14 0.12 0.11

9 -2 -14 0.17 0.16

9 2 -14 0.11 0.05

-9 2 -14 0.27 0.07

9 3 -14 1.21 0.27

9 -3 14 0.96 0.24

9 3 14 1.00 0.23

9 -3 -14 1.20 0.28

-9 3 -14 1.60 0.15

9 3 -14 1.62 0.14

9 4 -14 0.30 0.18

9 -4 14 0.40 0.16

9 -4 -14 0.32 0.16

9 4 14 0.46 0.18

9 4 -14 0.59 0.10

-9 4 -14 0.33 0.09

-9 4 14 0.52 0.11

9 -5 -14 1.51 0.32

9 -5 14 1.03 0.25

9 5 -14 1.67 0.16

-9 5 -14 1.57 0.16

-9 5 14 1.20 0.15

9 6 -14 3.31 0.40

-9 6 -14 3.46 0.23

-9 6 -14 3.66 0.20

9 6 -14 4.11 0.26

-9 6 14 3.12 0.23

9 6 -14 2.58 0.18

9 7 -14 1.34 0.16

-9 7 -14 1.16 0.15

9 8 -14 0.32 0.10

-9 8 -14 0.42 0.11

-9 9 -14 0.86 0.12

9 9 -14 0.83 0.13

-9 10 -14 0.51 0.08

9 10 -14 0.57 0.09

9 10 -14 0.67 0.12

-9 10 -14 0.60 0.10

9 11 -14 0.17 0.05

-9 11 -14 0.17 0.05

-9 11 -14 -0.01 0.05

9 11 -14 0.15 0.07

-9 12 -14 0.35 0.05

9 12 -14 0.51 0.06

9 0 -15 0.05 0.14

9 0 15 0.06 0.13

9 0 -15 0.03 0.04

-9 0 -15 0.04 0.05

9 1 -15 9.93 0.75

9 -1 -15 10.20 0.77

9 1 15 9.13 0.71

9 -1 15 10.24 0.76

9 1 -15 10.53 0.32

9 -1 -15 10.26 0.29

-9 1 -15 10.30 0.34

9 2 -15 0.68 0.22

9 -2 -15 0.49 0.20

9 -2 15 0.63 0.21

9 2 15 0.48 0.19

9 -2 -15 0.74 0.08

9 2 -15 0.62 0.09

-9 2 -15 0.59 0.09

9 3 -15 7.14 0.64

9 -3 -15 7.54 0.68

9 -3 15 6.82 0.64

9 3 15 7.36 0.64

9 3 -15 8.30 0.31

-9 3 -15 8.25 0.33

-9 3 15 7.95 0.34

9 4 -15 -0.02 0.10

9 -4 -15 0.07 0.14

9 -4 15 -0.17 0.17

9 4 15 -0.20 0.20

-9 -4 -15 0.17 0.14

9 4 -15 0.18 0.09

-9 4 15 0.17 0.09

-9 4 -15 0.15 0.09

9 -5 15 8.24 0.71

9 -5 -15 8.69 0.72

9 5 -15 9.89 0.38

-9 5 -15 9.76 0.38

-9 5 15 8.60 0.38

9 6 -15 -0.07 0.09

-9 6 -15 0.09 0.06

9 6 -15 0.15 0.07

-9 6 15 -0.04 0.07

-9 6 -15 -0.08 0.10

9 7 -15 0.73 0.13

-9 7 -15 0.50 0.11

-9 8 -15 0.60 0.12

9 8 -15 0.79 0.14

9 9 -15 1.24 0.15

-9 9 -15 1.16 0.13

9 10 -15 0.38 0.07

-9 10 -15 0.48 0.08

9 10 -15 0.65 0.11

-9 10 -15 0.42 0.08

-9 11 -15 0.37 0.06

9 11 -15 0.26 0.05

-9 11 -15 0.19 0.05

9 11 -15 0.26 0.07

9 12 -15 0.08 0.03

-9 12 -15 0.07 0.03

9 0 -16 0.01 0.11

9 0 16 0.03 0.12

9 0 -16 -0.05 0.06

-9 0 -16 -0.04 0.05

9 1 -16 0.43 0.20

9 -1 -16 0.35 0.17

9 -1 16 0.56 0.22

9 1 16 0.45 0.20

9 -1 -16 0.57 0.08

9 1 -16 0.45 0.08

-9 -1 -16 0.56 0.09

-9 1 -16 0.51 0.09

9 2 -16 1.02 0.26

9 -2 -16 1.16 0.28

9 -2 16 1.08 0.26

9 2 16 0.96 0.25

9 2 -16 1.27 0.13

-9 2 -16 1.47 0.15

9 -2 -16 1.31 0.11

9 3 -16 0.25 0.17

9 -3 -16 0.29 0.15

9 -3 16 0.23 0.16

9 3 -16 0.25 0.08

-9 3 16 0.37 0.10

-9 3 -16 0.18 0.07

9 -4 -16 1.26 0.31

9 -4 16 1.14 0.28

9 4 16 1.25 0.31

9 4 -16 1.12 0.27

-9 4 -16 1.14 0.14

9 4 -16 1.50 0.16

-9 4 16 1.34 0.16

9 5 -16 0.20 0.09

-9 5 16 0.24 0.10

-9 5 -16 0.27 0.09

-9 6 -16 2.20 0.19

9 6 -16 2.19 0.20

-9 6 16 2.22 0.21

9 6 -16 1.79 0.16

9 7 -16 0.76 0.12

-9 7 -16 0.72 0.12

9 8 -16 0.77 0.13

-9 8 -16 0.62 0.11

-9 9 -16 0.09 0.07

9 9 -16 0.15 0.09

-9 10 -16 0.00 0.05

9 10 -16 -0.10 0.06

-9 10 -16 -0.16 0.08

9 10 -16 0.04 0.07

-9 11 -16 0.08 0.04

9 11 -16 0.06 0.04

9 11 -16 0.02 0.05

-9 11 -16 0.02 0.04

9 0 -17 -0.07 0.15

9 0 17 -0.02 0.12

9 0 -17 -0.12 0.07

-9 0 -17 -0.06 0.06

9 1 -17 2.82 0.44

9 -1 -17 2.80 0.44

9 -1 17 2.80 0.44

9 1 17 2.59 0.40

9 1 -17 2.90 0.18

9 -1 -17 2.83 0.16

-9 1 -17 3.12 0.20

-9 -1 -17 3.25 0.19

9 2 -17 0.67 0.21

9 -2 -17 0.57 0.22

9 -2 17 0.38 0.19

9 2 17 0.62 0.22

9 2 -17 0.85 0.11

-9 2 -17 0.86 0.12

9 3 -17 1.18 0.28

9 -3 -17 1.26 0.31

9 -3 17 1.32 0.30

9 3 17 1.51 0.32

-9 -3 -17 1.70 0.35

9 3 -17 1.34 0.14

-9 3 -17 1.32 0.14

-9 3 17 1.57 0.17

9 4 -17 0.27 0.16

9 4 -17 0.17 0.08

-9 4 17 0.13 0.09

-9 4 -17 0.03 0.07

-9 5 -17 1.68 0.17

9 5 -17 2.22 0.19

-9 5 17 1.66 0.18

9 6 -17 0.77 0.12

-9 6 -17 0.95 0.14

-9 6 17 0.97 0.14

9 6 -17 0.76 0.11

9 7 -17 -0.19 0.10

-9 7 -17 0.04 0.08

-9 8 -17 -0.02 0.08

9 8 -17 0.03 0.08

9 9 -17 0.33 0.09

-9 9 -17 0.30 0.08

9 10 -17 -0.02 0.04

-9 10 -17 -0.01 0.04

9 10 -17 -0.01 0.05

-9 10 -17 -0.04 0.05

-9 11 -17 0.04 0.03

9 11 -17 0.02 0.03

-9 11 -17 0.01 0.03

9 11 -17 0.01 0.04

9 0 -18 0.19 0.17

9 0 18 -0.13 0.20

9 0 -18 -0.14 0.07

-9 0 -18 0.02 0.05

9 1 -18 0.12 0.15

9 -1 -18 0.04 0.14

9 -1 18 0.18 0.14

9 1 18 0.34 0.22

9 1 -18 0.21 0.06

9 -1 -18 0.13 0.05

-9 -1 -18 0.18 0.06

-9 1 -18 0.26 0.08

9 2 -18 0.16 0.06

-9 2 18 0.08 0.08

-9 2 -18 0.16 0.08

9 3 -18 0.08 0.07

-9 3 -18 -0.01 0.07

-9 3 18 0.12 0.08

9 4 -18 1.41 0.15

-9 4 -18 1.36 0.16

-9 4 18 1.43 0.17

9 5 -18 0.29 0.09

-9 5 18 0.33 0.10

-9 5 -18 0.11 0.09

9 5 -18 0.22 0.08

9 6 -18 0.25 0.09

-9 6 18 0.31 0.10

-9 6 -18 0.36 0.11

9 6 -18 0.27 0.08

9 7 -18 0.16 0.08

-9 7 -18 0.17 0.09

9 7 -18 0.14 0.06

-9 8 -18 0.04 0.06

9 8 -18 0.03 0.07

9 9 -18 -0.07 0.06

-9 9 -18 0.01 0.05

9 10 -18 0.01 0.03

-9 10 -18 0.01 0.03

9 10 -18 0.03 0.05

-9 10 -18 0.01 0.04

9 0 -19 -0.01 0.05

-9 0 -19 0.00 0.06

9 1 -19 0.26 0.07

-9 1 -19 0.17 0.07

-9 -1 -19 0.33 0.07

9 2 -19 0.29 0.08

-9 2 19 0.16 0.08

-9 2 -19 0.18 0.07

9 3 -19 0.47 0.10

-9 3 19 0.46 0.12

-9 3 -19 0.39 0.09

9 4 -19 0.20 0.08

-9 4 -19 0.11 0.08

-9 4 19 0.13 0.08

9 5 -19 0.14 0.08

-9 5 19 0.43 0.11

-9 5 -19 0.28 0.09

9 5 -19 0.37 0.09

9 6 -19 0.96 0.13

9 6 -19 0.81 0.11

-9 6 19 0.99 0.14

-9 6 -19 0.99 0.14

9 7 -19 0.48 0.10

9 7 -19 0.39 0.08

-9 7 -19 0.51 0.10

-9 8 -19 0.01 0.06

9 8 -19 0.10 0.06

9 9 -19 0.12 0.04

-9 9 -19 0.14 0.04

-9 9 -19 0.11 0.06

9 9 -19 0.12 0.05

-9 10 -19 0.17 0.04

9 10 -19 0.13 0.04

-9 10 -19 0.12 0.04

9 10 -19 0.15 0.05

-9 0 -20 -0.06 0.06

9 1 -20 0.27 0.07

-9 1 20 0.18 0.08

-9 1 -20 0.31 0.08

-9 -1 -20 0.40 0.08

9 2 -20 0.22 0.07

-9 2 20 0.06 0.09

-9 2 -20 0.02 0.06

9 3 -20 0.18 0.07

-9 3 -20 0.07 0.07

-9 3 20 0.16 0.09

9 4 -20 -0.04 0.06

-9 4 -20 0.06 0.07

-9 4 20 -0.07 0.08

9 5 -20 0.38 0.09

9 5 -20 0.46 0.09

-9 5 20 0.52 0.11

-9 5 -20 0.48 0.10

9 6 -20 0.41 0.09

-9 6 -20 0.43 0.10

9 6 -20 0.34 0.08

9 7 -20 0.18 0.07

-9 7 -20 0.23 0.08

9 7 -20 0.12 0.05

-9 8 -20 0.09 0.06

9 8 -20 0.12 0.05

9 9 -20 -0.02 0.04

-9 9 -20 0.02 0.03

9 9 -20 0.03 0.04

-9 9 -20 -0.07 0.05

9 10 -20 -0.01 0.03

-9 0 -21 0.01 0.05

-9 0 21 -0.18 0.10

9 1 -21 -0.03 0.05

-9 1 -21 -0.02 0.06

-9 1 21 -0.03 0.08

-9 -1 -21 0.00 0.04

9 2 -21 0.23 0.07

-9 2 21 0.13 0.09

-9 2 -21 0.15 0.07

9 3 -21 0.08 0.06

-9 3 -21 0.03 0.07

-9 3 21 0.00 0.09

9 4 -21 0.14 0.06

-9 4 21 0.20 0.10

-9 4 -21 0.19 0.08

9 4 -21 0.16 0.07

9 5 -21 0.06 0.06

-9 5 21 0.08 0.08

-9 5 -21 0.11 0.07

9 5 -21 0.10 0.06

9 6 -21 0.03 0.06

-9 6 -21 0.02 0.06

9 6 -21 0.03 0.05

9 7 -21 0.16 0.06

-9 7 -21 0.12 0.06

9 7 -21 0.16 0.05

9 8 -21 -0.12 0.05

-9 8 -21 -0.09 0.06

9 9 -21 0.00 0.02

-9 9 -21 -0.03 0.03

9 9 -21 -0.01 0.03

-9 9 -21 -0.06 0.04

-9 0 22 -0.03 0.08

-9 0 -22 -0.06 0.06

-9 1 22 0.07 0.08

-9 -1 22 0.08 0.07

-9 1 -22 0.04 0.05

-9 -1 -22 0.03 0.04

9 2 -22 0.04 0.05

-9 -2 22 -0.04 0.07

-9 2 22 -0.02 0.09

-9 2 -22 0.03 0.06

9 3 -22 0.00 0.05

-9 3 22 -0.05 0.10

-9 3 -22 -0.04 0.07

9 4 -22 0.09 0.05

-9 4 22 0.07 0.08

-9 4 -22 0.08 0.07

9 4 -22 0.05 0.06

9 5 -22 0.09 0.05

-9 5 -22 0.15 0.06

9 5 -22 -0.01 0.05

9 6 -22 0.11 0.05

-9 6 -22 0.11 0.06

9 6 -22 0.02 0.04

-9 7 -22 0.10 0.05

9 7 -22 0.05 0.04

-9 8 -22 0.02 0.03

9 8 -22 -0.03 0.03

-9 8 -22 0.02 0.04

9 8 -22 0.06 0.04

-9 0 23 -0.05 0.07

-9 0 -23 -0.05 0.05

-9 -1 23 -0.02 0.06

-9 1 -23 -0.08 0.06

-9 1 23 -0.01 0.08

-9 2 -23 -0.04 0.06

-9 -2 23 0.08 0.07

-9 2 23 -0.03 0.07

9 3 -23 -0.08 0.05

-9 3 -23 -0.11 0.07

-9 -3 23 0.04 0.05

9 3 -23 -0.11 0.08

-9 3 23 -0.03 0.08

9 4 -23 -0.02 0.04

-9 -4 23 0.04 0.04

-9 4 23 0.04 0.07

-9 4 -23 -0.03 0.06

9 4 -23 -0.08 0.06

9 5 -23 0.10 0.05

-9 -5 23 0.06 0.04

9 5 -23 0.08 0.05

-9 5 -23 0.04 0.06

-9 6 -23 0.01 0.05

9 6 -23 0.03 0.04

9 6 -23 -0.03 0.04

9 7 -23 -0.01 0.03

-9 7 -23 -0.06 0.05

-9 8 -23 0.32 0.05

-9 0 -24 -0.03 0.04

-9 0 24 -0.02 0.06

-9 -1 24 0.01 0.06

-9 1 -24 -0.03 0.05

-9 1 24 0.09 0.07

9 2 -24 -0.10 0.07

-9 2 24 -0.03 0.07

-9 -2 24 0.04 0.05

-9 2 -24 -0.06 0.05

-9 -3 24 0.17 0.06

-9 3 24 0.10 0.06

-9 3 -24 0.17 0.06

9 3 -24 0.17 0.06

9 4 -24 0.05 0.04

-9 -4 24 0.06 0.04

-9 4 -24 0.08 0.05
[truncated: 457,684 more chars]
