# Supplementary material for: Antineoplastic 4-piperidone-1-phosphonothioates with potential multi-targeted inhibitory properties
Source: Sci Rep. 2025 Nov 18;15:40363. doi: 10.1038/s41598-025-25796-6 (PMC12627723; doi:10.1038/s41598-025-25796-6)
Supplement: Supplementary file 5 — Supplementary Material 5 [file 41598_2025_25796_MOESM5_ESM.docx]

**Antineoplastic 4-piperidone-1-phosphonothioates with potential multi-targeted inhibitory properties**

Mohamed S. Bekheit^1^, Siva S. Panda^2^, Benson M. Kariuki^3^, Walid Fayad^4^, Ahmed A. F. Soliman^4^, Hanaa Farag^1^, Adel S. Girgis^1^,*

^1^ Department of Pesticide Chemistry, National Research Centre, Dokki, Giza 12622, Egypt

^2^ Department of Chemistry and Biochemistry & Department of Biochemistry and Molecular Biology, Augusta University, Augusta, GA 30912, USA

^3^ School of Chemistry, Cardiff University, Main Building, Park Place, Cardiff CF10 3AT, UK

^4^ Drug Bioassay-Cell Culture Laboratory, Pharmacognosy Department, National Research Centre, Dokki 12622, Giza, Egypt

* Corresponding author: [girgisas10@yahoo.com](mailto:girgisas10@yahoo.com), [as.girgis@nrc.sci.eg](mailto:as.girgis@nrc.sci.eg)

**Supplementary material**

**Table title**

**Table S1.** Crystal data and structure refinement for compounds **20a**, and **20b**.

**Table S2.** Total energy in production step (kcal mol^-1^) of the protein backbone (PDB: 4OAS).

**Table S3.** Total energy in production step (kcal mol^-1^) of the best conformation pose of compound **20i** docked in the active site of PDB: 4OAS.

**Table S4.** Total energy in production step (kcal mol^-1^) of the best conformation pose of compound **20k** docked in the active site of PDB: 4OAS.

**Table S5.** RMSD of the best conformation pose of compound **20i** docked in the protein active site of PDB: 4OAS.

**Table S6.** RMSD of the best conformation pose of compound **20k** docked in the protein active site of PDB: 4OAS.

**Table S7.** RMSF of the best conformation pose of compound **20i** docked in the protein active site of PDB: 4OAS.

**Table S8.** RMSF of the best conformation pose of compound **20k** docked in the protein active site of PDB: 4OAS.

**Figure captions**

**Fig. S1.** IR spectrum of compound **20a** (KBr pellet).

**Fig. S2.** ^1^H-NMR spectrum of compound **20a** in DMSO-*d6*.

**Fig. S3.** ^13^C-NMR spectrum of compound **20a** in DMSO-*d6*.

**Fig. S4.** IR spectrum of compound **20b** (KBr pellet).

**Fig. S5.** ^1^H-NMR spectrum of compound **20b** in CDCl_3_.

**Fig. S6.** ^13^C-NMR spectrum of compound **20b** in CDCl_3_.

**Fig. S7.** IR spectrum of compound **20c** (KBr pellet).

**Fig. S8.** ^1^H-NMR spectrum of compound **20c** in DMSO-*d6*.

**Fig. S9.** ^13^C-NMR spectrum of compound **20c** in DMSO-*d6*.

**Fig. S10.** IR spectrum of compound **20d** (KBr pellet).

**Fig. S11.** ^1^H-NMR spectrum of compound **20d** in DMSO-*d6*.

**Fig. S12.** ^13^C-NMR spectrum of compound **20d** in DMSO-*d6*.

**Fig. S13.** IR spectrum of compound **20e** (KBr pellet).

**Fig. S14.** ^1^H-NMR spectrum of compound **20e** in DMSO-*d6*.

**Fig. S15.** ^13^C-NMR spectrum of compound **20e** in DMSO-*d6*.

**Fig. S16.** IR spectrum of compound **20f** (KBr pellet).

**Fig. S17.** ^1^H-NMR spectrum of compound **20f** in DMSO-*d6*.

**Fig. S18.** ^13^C-NMR spectrum of compound **20f** in DMSO-*d6*.

**Fig. S19.** IR spectrum of compound **20g** (KBr pellet).

**Fig. S20.** ^1^H-NMR spectrum of compound **20g** in DMSO-*d6*.

**Fig. S21.** ^13^C-NMR spectrum of compound **20g** in DMSO-*d6*.

**Fig. S22.** IR spectrum of compound **20h** (KBr pellet).

**Fig. S23.** ^1^H-NMR spectrum of compound **20h** in DMSO-*d6*.

**Fig. S24.** ^13^C-NMR spectrum of compound **20h** in DMSO-*d6*.

**Fig. S25.** IR spectrum of compound **20i** (KBr pellet).

**Fig. S26.** ^1^H-NMR spectrum of compound **20i** in DMSO-*d6*.

**Fig. S27.** ^13^C-NMR spectrum of compound **20i** in DMSO-*d6*.

**Fig. S28.** IR spectrum of compound **20j** (KBr pellet).

**Fig. S29.** ^1^H-NMR spectrum of compound **20j** in DMSO-*d6*.

**Fig. S30.** ^13^C-NMR spectrum of compound **20j** in DMSO-*d6*.

**Fig. S31.** IR spectrum of compound **20k** (KBr pellet).

**Fig. S32.** ^1^H-NMR spectrum of compound **20k** in DMSO-*d6*.

**Fig. S33.** ^13^C-NMR spectrum of compound **20k** in DMSO-*d6*.

**Fig. S34.** IR spectrum of compound **20l** (KBr pellet).

**Fig. S35.** ^1^H-NMR spectrum of compound **20l** in DMSO-*d6*.

**Fig. S36.** ^13^C-NMR spectrum of compound **20l** in DMSO-*d6*.

**Fig. S37.** Mass spectra (EI-MS) of the synthesized compounds **20a‒l**.

**Fig. S38.** Dose response curves of **20a‒l** against MCF7 (breast) cancer cell line.

**Fig. S39.** Dose response curves of **20a‒l** against HCT116 (colon) cancer cell line.

**Fig. S40.** Dose response curves of **20a‒l** against A431 (skin/squamous) cancer cell line.

**Fig. S41.** Dose response curves of **20a‒l** against RPE1 (normal/non-cancer) cell line.

**Fig. S42.** 2D**-**docking poses of the tested compounds **20a‒l**, co-crystallized ligand, and doxorubicin (standard reference/drug) in PDB ID: 4OAS.

**Fig. S43.** 3D-docking poses of the tested compounds **20a‒l**, co-crystallized ligand, and doxorubicin (standard reference/drug) in PDB ID: 4OAS.

**Crystal structure determination**

Single-crystal X-ray diffraction data were collected on an Agilent SuperNova Dual Atlas diffractometer, equipped with a mirror monochromator and using either Cu or Mo radiation. An Oxford Cryosystems cooling apparatus was used for temperature regulation. The data were processed using CrysAlisPro^1^ and the crystal structures were solved using SHELXT^2^ and refined using SHELXL^3^. In general, non-hydrogen atoms were refined with anisotropic displacement parameters. In the final cycles of refinement, hydrogen atom geometry was idealized, and a riding model was used with U_iso_ set at 1.2 or 1.5 times the value of U_eq_ for the atom to which the hydrogen atoms are bonded. In the structure of **20a**, the diethoxythiophosphoryl groups are disordered and were refined with two components with occupancies 0.60(2)/0.40(2) and 0.540(8)/0.460(8) for the two independent molecules. In the structure of **20b**, the diethoxythiophosphoryl groups are also disordered and were refined with two components with occupancies 0.505(13)/0.495(13), 0.569(11)/0.431(11), 0.728(6)/0.272(6) and 0.777(6)/0.223(6) for the four independent molecules. The crystal structures have been deposited in the CSD under reference CCDC 2440500 and CCDC2440501.

**Biological studies**

All the biological studies conducted were approved and adhered to standards set by the Research Ethics Committee, National Research Centre, Egypt (associated with project ID: 13060103).

**Biochemical/enzymatic properties**

The synthesized analogs revealing considerable antiproliferation properties were screened for their anti-MDM2, activation of p53, anti-topo-I, anti-topo-II properties utilizing the IC_50_ values observed against MCF7 (breast cancer) cell line, following the instructions of the manufacturer^4‒7^.

**Molecular docking studies**

Molecular docking study was carried out using CDOCKER protocol in Discovery Studio 4.1 Software. The synthesized compounds were docked into the PDB: 4OAS active site. The X-ray crystallographic structure of PDB: 4OAS was downloaded, followed by removing of the unneeded water molecules. Protein structure was checked for any incomplete residues followed by addition of hydrogen atoms at the appropriate position(s) of amino acids. Optimized by the standard protocol (force field: CHARMm, Partial charge: MMFF94, resolution: 1.70 Å, radius of the active site employed: 8.427742 Å, RMS gradient: 0.09155) with identification of the active/binding site (a sphere) to be utilized in the following docking steps. The chemical structure of the tested compounds should be also optimized (force field: CHARMm, Partial charge: MMFF94) then, the binding mode of interaction of the synthesized compounds (**20a‒l**) in the protein active site was determined/studied (C-docker protocol) for determination the bonding/non-bonding interactions with the active site/pocket protein amino acid(s)^8,9^.

**Molecular dynamic simulation studies**

Molecular dynamic simulation studies using Discovery Studio 4.1 Software were carried out for the protein backbone of PDB: 4OAS and best pose detected in molecular docking studies for compounds **20i**, and **20k** utilizing the standard protocol (standard dynamic cascade). For the tested agents, the poses revealing best docking interactions were dragged and dropped in the protein of PDB: 4OAS, optimized by the standard protocol (force field: CHARMm, partial charge: MMFF94) then the standard molecular dynamic protocol was applied^9^.

Minimization 1

Algorithm: Steepest Descent, Maximum Steps: 2000, RMS Gradient: 1.0.

Minimization 2

Algorithm: Conjugate Gradient, Max. Steps: 2000, RMS Gradient: 0.1

Heating

Simulation Time (ps): 4, Time Steps (fs): 2, Initial Temperature: 50, Target Temperature: 300, Adjust Velocity Frequency: 1000, Save Results Interval (ps): 2.

Equilibration

Simulation Time (ps): 20, Time Step (fs): 2, Target Temperature: 300, Adjust Velocity Frequency: 1000, Save Results Intervals (ps): 2.

Production

Simulation Time (ps): 200, Time Steps (fs): 2, Target Temperature: 300, Type: NVT.

Implicit Solvent Model

Generalized Born with a simple Switching (GBSW).

RMSD and RMSF were obtained upon applying trajectory analysis on the output files obtained from the molecular dynamic simulation studies.

**References**

1. CrysAlisPro, Rigaku OD, Yarnton, England, 2024.
2. Sheldrick, G. M. SHELXT – Integrated space-group and crystal-structure determination. *Acta Crystallogr., Sect. A* **71**, 3‒8. <https://doi.org/10.1107/S2053273314026370> (2015).
3. Sheldrick, G. M. Crystal structure refinement with SHELXL. *Acta Crystallogr., Sect. C* **71**, 3‒8. <https://doi.org/10.1107/S2053229614024218> (2015).
4. Human MDM2 (Mdm2 p53 Binding Protein Homolog) ELISA Kit, Cat: ELK4557, ELK Biotechnology, <http://www.elkbiotech.com>.
5. Human TP53 (Tumor Protein p53) ELISA Kit, Cat: ELK1507, ELK Biotechnology, <http://www.elkbiotech.com>.
6. Human TOP1 (Topoisomerase I) ELISA Kit, Cat: ELK3045, <http://www.elkbiotech.com>.
7. Human DNA Topoisomerase 2-alpha, TOP2A ELISA Kit, Cat: E6652Hu, [www.bt-laboratory.com](http://www.bt-laboratory.com).
8. Co-crystal structure of MDM2 (17-111) in complex with compound 25, <https://www.rcsb.org/structure/4OAS>.
9. Aboshouk, D. R., Hamed, A. R., Panda, S. S., Bekheit, M. S., Youssef, M. A., Girgis, A. S. Curcumin mimics of potential chemoprevention with NQO1 induction properties. Sci. Rep. **15**, 2332. <https://doi.org/10.1038/s41598-025-85588-w> (2025).

**Table S1.** Crystal data and structure refinement for compounds **20a**, and **20b**.

|  | Compound **20a** | Compound **20b** |
| --- | --- | --- |
| Empirical formula | C_23_H_26_NO_3_PS | C_23_H_24_F_2_NO_3_PS |
| Formula weight | 427.48 | 463.46 |
| Temperature | 293 K | 293 K |
| Wavelength | 0.71073 Å | 1.54184 Å |
| Crystal system | Monoclinic | Orthorhombic |
| Space group | P2_1_/c | Pna2_1_ |
| a | 15.9985(6) Å | 32.9400(5) Å |
| b | 23.6855(8) Å | 12.5414(2) Å |
| c | 12.8351(5) Å | 22.4554(4) Å |
| α | 90° | 90° |
| β | 112.788(4)° | 90° |
| γ | 90° | 90° |
| Volume | 4484.0(3) Å3 | 9276.6(3) Å3 |
| Z | 8 | 16 |
| Density (calculated) | 1.266 mg/m3 | 1.327 mg/m3 |
| Absorption coefficient | 0.239 mm-1 | 2.245 mm-1 |
| F(000) | 1810.50 | 3872.0 |
| Crystal size | 0.280 x 0.410 x 0.440 mm3 | 0.100 x 0.12 x 0.590 mm3 |
| Theta range for data collection | 3.441 to 29.779 | 3.771 to 72.795 |
| Index ranges | -21<=h<=22,  -32<=k<=32,  -17<=l<=17 | -36<=h<=40,  -12<=k<=15,  -27<=l<=26 |
| Reflections collected | 11415 | 37910 |
| Independent reflections | 5914 [R(int) = 0.0838] | 12652 [R(int) = 0.0567] |
| Completeness to theta | 99.7 % | 99.8 % |
| Refinement method | Full-matrix least-squares on F2 | Full-matrix least-squares on F2 |
| Data / restraints / parameters | 5914 / 652 / 677 | 12652 / 1317 / 1389 |
| Goodness-of-fit on F2 | 1.078 | 1.027 |
| Final R indices [I>2sigma(I)] | R1 = 0.0838, wR2 = 0.2925 | R1 = 0.0567, wR2 = 0.1709 |
| R indices (all data) | R1 = 0.1403, wR2 = 0.2925 | R1 = 0.0705, wR2 = 0.1709 |
| Absolute structure parameter | - | 0.082(9) |
| Largest diff. peak and hole | 0.335 and -0.331 e.Å-3 | 0.520 and -0.456 e.Å-3 |

**Table S2.** Total energy in production step (kcal mol^-1^) of the protein backbone (PDB: 4OAS).

| Time (*p*s) | Total Energy |
| --- | --- |
| 26 | -9261.74 |
| 28 | -9272.16 |
| 30 | -9275.95 |
| 32 | -9291.11 |
| 34 | -9304.39 |
| 36 | -9310.93 |
| 38 | -9328.61 |
| 40 | -9333.86 |
| 42 | -9341.52 |
| 44 | -9346.33 |
| 46 | -9349.09 |
| 48 | -9357.28 |
| 50 | -9365.05 |
| 52 | -9368.05 |
| 54 | -9377.83 |
| 56 | -9376.76 |
| 58 | -9377.71 |
| 60 | -9378.98 |
| 62 | -9381.37 |
| 64 | -9380.21 |
| 66 | -9384.21 |
| 68 | -9387.44 |
| 70 | -9389.64 |
| 72 | -9386.19 |
| 74 | -9388.31 |
| 76 | -9393.78 |
| 78 | -9390.21 |
| 80 | -9390.55 |
| 82 | -9389.43 |
| 84 | -9388.96 |
| 86 | -9390.72 |
| 88 | -9389.67 |
| 90 | -9388.51 |
| 92 | -9390.87 |
| 94 | -9389.37 |
| 96 | -9390.86 |
| 98 | -9392.09 |
| 100 | -9393.9 |
| 102 | -9395.08 |
| 104 | -9397.17 |
| 106 | -9395.98 |
| 108 | -9397.34 |
| 110 | -9400.89 |
| 112 | -9399.86 |
| 114 | -9400.58 |
| 116 | -9399.47 |
| 118 | -9396.57 |
| 120 | -9395.62 |
| 122 | -9392.69 |
| 124 | -9391.24 |
| 126 | -9393.33 |
| 128 | -9396.44 |
| 130 | -9400.14 |
| 132 | -9404.27 |
| 134 | -9408.8 |
| 136 | -9410.65 |
| 138 | -9409.05 |
| 140 | -9409.99 |
| 142 | -9409.62 |
| 144 | -9407.89 |
| 146 | -9406.09 |
| 148 | -9407.04 |
| 150 | -9405.21 |
| 152 | -9405.54 |
| 154 | -9408 |
| 156 | -9405.47 |
| 158 | -9407.21 |
| 160 | -9402.74 |
| 162 | -9397.83 |
| 164 | -9400.43 |
| 166 | -9399.16 |
| 168 | -9400.69 |
| 170 | -9398.83 |
| 172 | -9403.38 |
| 174 | -9399 |
| 176 | -9397.26 |
| 178 | -9398.73 |
| 180 | -9397.86 |
| 182 | -9397.31 |
| 184 | -9400.92 |
| 186 | -9399.07 |
| 188 | -9404.77 |
| 190 | -9400 |
| 192 | -9401.2 |
| 194 | -9401.46 |
| 196 | -9402.4 |
| 198 | -9401.66 |
| 200 | -9397.47 |
| 202 | -9397.65 |
| 204 | -9395.79 |
| 206 | -9397.75 |
| 208 | -9398.77 |
| 210 | -9396.14 |
| 212 | -9391.76 |
| 214 | -9392.27 |
| 216 | -9396.97 |
| 218 | -9397.48 |
| 220 | -9391.05 |
| 222 | -9397.99 |
| 224 | -9395.87 |

**Table S3.** Total energy in production step (kcal mol^-1^) of the best conformation pose of compound **20i** docked in the active site of PDB: 4OAS.

| Time (*p*s) | Total Energy |
| --- | --- |
| 26 | -5584.01 |
| 28 | -5584.26 |
| 30 | -5583.76 |
| 32 | -5583.37 |
| 34 | -5583.09 |
| 36 | -5582.51 |
| 38 | -5582.94 |
| 40 | -5583.14 |
| 42 | -5583.43 |
| 44 | -5583.12 |
| 46 | -5582.73 |
| 48 | -5582.6 |
| 50 | -5582.82 |
| 52 | -5582.83 |
| 54 | -5583.27 |
| 56 | -5582.88 |
| 58 | -5583.11 |
| 60 | -5582.86 |
| 62 | -5583.26 |
| 64 | -5583.88 |
| 66 | -5584.07 |
| 68 | -5583.97 |
| 70 | -5583.74 |
| 72 | -5583.99 |
| 74 | -5584.33 |
| 76 | -5584.31 |
| 78 | -5584.39 |
| 80 | -5583.88 |
| 82 | -5583.87 |
| 84 | -5583.54 |
| 86 | -5583.41 |
| 88 | -5583.55 |
| 90 | -5583.32 |
| 92 | -5583.07 |
| 94 | -5582.9 |
| 96 | -5582.9 |
| 98 | -5582.6 |
| 100 | -5582.71 |
| 102 | -5582.88 |
| 104 | -5583.1 |
| 106 | -5583.02 |
| 108 | -5583.08 |
| 110 | -5582.95 |
| 112 | -5582.55 |
| 114 | -5582.42 |
| 116 | -5582.35 |
| 118 | -5582.76 |
| 120 | -5582.2 |
| 122 | -5582.31 |
| 124 | -5582.27 |
| 126 | -5581.99 |
| 128 | -5582.57 |
| 130 | -5581.76 |
| 132 | -5581.52 |
| 134 | -5581.22 |
| 136 | -5581.23 |
| 138 | -5581.4 |
| 140 | -5581.41 |
| 142 | -5581.41 |
| 144 | -5581.91 |
| 146 | -5581.6 |
| 148 | -5581.4 |
| 150 | -5581.82 |
| 152 | -5581.8 |
| 154 | -5582.16 |
| 156 | -5582.97 |
| 158 | -5582.99 |
| 160 | -5583.69 |
| 162 | -5583.51 |
| 164 | -5583.98 |
| 166 | -5583.42 |
| 168 | -5584.04 |
| 170 | -5584.23 |
| 172 | -5584.24 |
| 174 | -5584.34 |
| 176 | -5584.02 |
| 178 | -5583.85 |
| 180 | -5584.07 |
| 182 | -5583.91 |
| 184 | -5583.57 |
| 186 | -5583.11 |
| 188 | -5582.76 |
| 190 | -5583.03 |
| 192 | -5583.61 |
| 194 | -5583.35 |
| 196 | -5583.93 |
| 198 | -5583.89 |
| 200 | -5584.1 |
| 202 | -5583.99 |
| 204 | -5584 |
| 206 | -5584.36 |
| 208 | -5584.14 |
| 210 | -5584.18 |
| 212 | -5584.24 |
| 214 | -5583.89 |
| 216 | -5583.58 |
| 218 | -5583.32 |
| 220 | -5583.23 |
| 222 | -5583.54 |
| 224 | -5583.7 |

**Table S4.** Total energy in production step (kcal mol^-1^) of the best conformation pose of compound **20k** docked in the active site of PDB: 4OAS.

| Time (*p*s) | Total Energy |
| --- | --- |
| 26 | -5535.43 |
| 28 | -5535.1 |
| 30 | -5535.5 |
| 32 | -5535.59 |
| 34 | -5535.66 |
| 36 | -5535.73 |
| 38 | -5535.6 |
| 40 | -5535.51 |
| 42 | -5535.67 |
| 44 | -5535.99 |
| 46 | -5535.67 |
| 48 | -5535.6 |
| 50 | -5535.9 |
| 52 | -5535.5 |
| 54 | -5535.53 |
| 56 | -5535.39 |
| 58 | -5535.33 |
| 60 | -5534.84 |
| 62 | -5535.78 |
| 64 | -5535.76 |
| 66 | -5535.5 |
| 68 | -5535.62 |
| 70 | -5534.92 |
| 72 | -5535.28 |
| 74 | -5535.13 |
| 76 | -5535.06 |
| 78 | -5535.05 |
| 80 | -5535.22 |
| 82 | -5535.57 |
| 84 | -5535.45 |
| 86 | -5535.35 |
| 88 | -5535.71 |
| 90 | -5535.49 |
| 92 | -5534.69 |
| 94 | -5535.44 |
| 96 | -5535.4 |
| 98 | -5534.96 |
| 100 | -5535.01 |
| 102 | -5534.48 |
| 104 | -5534.69 |
| 106 | -5534.98 |
| 108 | -5534.81 |
| 110 | -5535.29 |
| 112 | -5535.51 |
| 114 | -5535.74 |
| 116 | -5535.43 |
| 118 | -5535.39 |
| 120 | -5535.34 |
| 122 | -5535.72 |
| 124 | -5535.02 |
| 126 | -5535.82 |
| 128 | -5535.56 |
| 130 | -5535.52 |
| 132 | -5534.89 |
| 134 | -5535.16 |
| 136 | -5535.26 |
| 138 | -5534.81 |
| 140 | -5535 |
| 142 | -5535.27 |
| 144 | -5535.4 |
| 146 | -5535.71 |
| 148 | -5536.16 |
| 150 | -5536.27 |
| 152 | -5535.55 |
| 154 | -5535.44 |
| 156 | -5536.05 |
| 158 | -5535.49 |
| 160 | -5535.51 |
| 162 | -5535.44 |
| 164 | -5535.51 |
| 166 | -5535.58 |
| 168 | -5535.88 |
| 170 | -5535.82 |
| 172 | -5535.69 |
| 174 | -5535.74 |
| 176 | -5536.02 |
| 178 | -5536.06 |
| 180 | -5535.69 |
| 182 | -5536.13 |
| 184 | -5536.32 |
| 186 | -5535.86 |
| 188 | -5535.37 |
| 190 | -5535.23 |
| 192 | -5535.52 |
| 194 | -5535.43 |
| 196 | -5535.57 |
| 198 | -5535.75 |
| 200 | -5535.83 |
| 202 | -5536.15 |
| 204 | -5536.22 |
| 206 | -5535.94 |
| 208 | -5535.89 |
| 210 | -5535.4 |
| 212 | -5535.7 |
| 214 | -5535.48 |
| 216 | -5535.28 |
| 218 | -5535.41 |
| 220 | -5535.25 |
| 222 | -5535.42 |
| 224 | -5535.89 |

**Table S5.** RMSD of the best conformation pose of compound **20i** docked in the protein active site of PDB: 4OAS.

| Entry | Item | RMSD (Å) |
| --- | --- | --- |
| 1 | Conformation 1 | 0 |
| 2 | Conformation 2 | 0.096491 |
| 3 | Conformation 3 | 0.080753 |
| 4 | Conformation 4 | 0.092139 |
| 5 | Conformation 5 | 0.14195 |
| 6 | Conformation 6 | 0.146615 |
| 7 | Conformation 7 | 0.234928 |
| 8 | Conformation 8 | 0.239662 |
| 9 | Conformation 9 | 0.154994 |
| 10 | Conformation 10 | 0.15051 |
| 11 | Conformation 11 | 0.201862 |
| 12 | Conformation 12 | 0.100676 |
| 13 | Conformation 13 | 0.108419 |
| 14 | Conformation 14 | 0.144634 |
| 15 | Conformation 15 | 0.149393 |
| 16 | Conformation 16 | 0.174765 |
| 17 | Conformation 17 | 0.181723 |
| 18 | Conformation 18 | 0.104021 |
| 19 | Conformation 19 | 0.119746 |
| 20 | Conformation 20 | 0.146634 |
| 21 | Conformation 21 | 0.137767 |
| 22 | Conformation 22 | 0.108664 |
| 23 | Conformation 23 | 0.117652 |
| 24 | Conformation 24 | 0.141351 |
| 25 | Conformation 25 | 0.182665 |
| 26 | Conformation 26 | 0.165343 |
| 27 | Conformation 27 | 0.176328 |
| 28 | Conformation 28 | 0.179074 |
| 29 | Conformation 29 | 0.180643 |
| 30 | Conformation 30 | 0.139767 |
| 31 | Conformation 31 | 0.104516 |
| 32 | Conformation 32 | 0.101404 |
| 33 | Conformation 33 | 0.078526 |
| 34 | Conformation 34 | 0.107291 |
| 35 | Conformation 35 | 0.098588 |
| 36 | Conformation 36 | 0.125222 |
| 37 | Conformation 37 | 0.136165 |
| 38 | Conformation 38 | 0.115057 |
| 39 | Conformation 39 | 0.12971 |
| 40 | Conformation 40 | 0.131145 |
| 41 | Conformation 41 | 0.121435 |
| 42 | Conformation 42 | 0.116816 |
| 43 | Conformation 43 | 0.104728 |
| 44 | Conformation 44 | 0.118498 |
| 45 | Conformation 45 | 0.136935 |
| 46 | Conformation 46 | 0.132492 |
| 47 | Conformation 47 | 0.126775 |
| 48 | Conformation 48 | 0.128822 |
| 49 | Conformation 49 | 0.113807 |
| 50 | Conformation 50 | 0.14048 |
| 51 | Conformation 51 | 0.173582 |
| 52 | Conformation 52 | 0.196015 |
| 53 | Conformation 53 | 0.205018 |
| 54 | Conformation 54 | 0.202407 |
| 55 | Conformation 55 | 0.208917 |
| 56 | Conformation 56 | 0.193128 |
| 57 | Conformation 57 | 0.16246 |
| 58 | Conformation 58 | 0.141132 |
| 59 | Conformation 59 | 0.102598 |
| 60 | Conformation 60 | 0.092161 |
| 61 | Conformation 61 | 0.108557 |
| 62 | Conformation 62 | 0.094456 |
| 63 | Conformation 63 | 0.105684 |
| 64 | Conformation 64 | 0.117411 |
| 65 | Conformation 65 | 0.158094 |
| 66 | Conformation 66 | 0.137037 |
| 67 | Conformation 67 | 0.17518 |
| 68 | Conformation 68 | 0.146358 |
| 69 | Conformation 69 | 0.140234 |
| 70 | Conformation 70 | 0.156385 |
| 71 | Conformation 71 | 0.128036 |
| 72 | Conformation 72 | 0.139806 |
| 73 | Conformation 73 | 0.118416 |
| 74 | Conformation 74 | 0.14194 |
| 75 | Conformation 75 | 0.143443 |
| 76 | Conformation 76 | 0.16766 |
| 77 | Conformation 77 | 0.163773 |
| 78 | Conformation 78 | 0.14356 |
| 79 | Conformation 79 | 0.119296 |
| 80 | Conformation 80 | 0.126619 |
| 81 | Conformation 81 | 0.139828 |
| 82 | Conformation 82 | 0.141652 |
| 83 | Conformation 83 | 0.164682 |
| 84 | Conformation 84 | 0.13262 |
| 85 | Conformation 85 | 0.155176 |
| 86 | Conformation 86 | 0.176584 |
| 87 | Conformation 87 | 0.239894 |
| 88 | Conformation 88 | 0.212614 |
| 89 | Conformation 89 | 0.169295 |
| 90 | Conformation 90 | 0.197981 |
| 91 | Conformation 91 | 0.209465 |
| 92 | Conformation 92 | 0.168841 |
| 93 | Conformation 93 | 0.170051 |
| 94 | Conformation 94 | 0.103303 |
| 95 | Conformation 95 | 0.095692 |
| 96 | Conformation 96 | 0.101359 |
| 97 | Conformation 97 | 0.158013 |
| 98 | Conformation 98 | 0.151034 |
| 99 | Conformation 99 | 0.143443 |
| 100 | Conformation 100 | 0.127999 |
| 101 | Average RMSD | 0.142365 |

**Table S6.** RMSD of the best conformation pose of compound **20k** docked in the protein active site of PDB: 4OAS.

| Entry | Item | RMSD (Å) |
| --- | --- | --- |
| 1 | Conformation 1 | 0 |
| 2 | Conformation 2 | 0.132994 |
| 3 | Conformation 3 | 0.143438 |
| 4 | Conformation 4 | 0.153849 |
| 5 | Conformation 5 | 0.138669 |
| 6 | Conformation 6 | 0.144192 |
| 7 | Conformation 7 | 0.160259 |
| 8 | Conformation 8 | 0.147927 |
| 9 | Conformation 9 | 0.159902 |
| 10 | Conformation 10 | 0.158916 |
| 11 | Conformation 11 | 0.176557 |
| 12 | Conformation 12 | 0.169282 |
| 13 | Conformation 13 | 0.169525 |
| 14 | Conformation 14 | 0.151714 |
| 15 | Conformation 15 | 0.16214 |
| 16 | Conformation 16 | 0.180517 |
| 17 | Conformation 17 | 0.162908 |
| 18 | Conformation 18 | 0.160273 |
| 19 | Conformation 19 | 0.168796 |
| 20 | Conformation 20 | 0.167284 |
| 21 | Conformation 21 | 0.171369 |
| 22 | Conformation 22 | 0.178306 |
| 23 | Conformation 23 | 0.176327 |
| 24 | Conformation 24 | 0.174209 |
| 25 | Conformation 25 | 0.159927 |
| 26 | Conformation 26 | 0.143182 |
| 27 | Conformation 27 | 0.152381 |
| 28 | Conformation 28 | 0.18488 |
| 29 | Conformation 29 | 0.192655 |
| 30 | Conformation 30 | 0.176201 |
| 31 | Conformation 31 | 0.174655 |
| 32 | Conformation 32 | 0.17459 |
| 33 | Conformation 33 | 0.167571 |
| 34 | Conformation 34 | 0.164411 |
| 35 | Conformation 35 | 0.174111 |
| 36 | Conformation 36 | 0.187226 |
| 37 | Conformation 37 | 0.189239 |
| 38 | Conformation 38 | 0.173607 |
| 39 | Conformation 39 | 0.205494 |
| 40 | Conformation 40 | 0.200912 |
| 41 | Conformation 41 | 0.167413 |
| 42 | Conformation 42 | 0.20206 |
| 43 | Conformation 43 | 0.17509 |
| 44 | Conformation 44 | 0.174134 |
| 45 | Conformation 45 | 0.187711 |
| 46 | Conformation 46 | 0.179109 |
| 47 | Conformation 47 | 0.179429 |
| 48 | Conformation 48 | 0.179597 |
| 49 | Conformation 49 | 0.1935 |
| 50 | Conformation 50 | 0.199533 |
| 51 | Conformation 51 | 0.199144 |
| 52 | Conformation 52 | 0.183206 |
| 53 | Conformation 53 | 0.212297 |
| 54 | Conformation 54 | 0.20199 |
| 55 | Conformation 55 | 0.189424 |
| 56 | Conformation 56 | 0.188179 |
| 57 | Conformation 57 | 0.193555 |
| 58 | Conformation 58 | 0.171031 |
| 59 | Conformation 59 | 0.188678 |
| 60 | Conformation 60 | 0.171977 |
| 61 | Conformation 61 | 0.170077 |
| 62 | Conformation 62 | 0.165281 |
| 63 | Conformation 63 | 0.158312 |
| 64 | Conformation 64 | 0.169908 |
| 65 | Conformation 65 | 0.177093 |
| 66 | Conformation 66 | 0.141428 |
| 67 | Conformation 67 | 0.162222 |
| 68 | Conformation 68 | 0.192731 |
| 69 | Conformation 69 | 0.148433 |
| 70 | Conformation 70 | 0.193876 |
| 71 | Conformation 71 | 0.184391 |
| 72 | Conformation 72 | 0.1748 |
| 73 | Conformation 73 | 0.152273 |
| 74 | Conformation 74 | 0.155626 |
| 75 | Conformation 75 | 0.16927 |
| 76 | Conformation 76 | 0.147215 |
| 77 | Conformation 77 | 0.168927 |
| 78 | Conformation 78 | 0.164445 |
| 79 | Conformation 79 | 0.143668 |
| 80 | Conformation 80 | 0.175982 |
| 81 | Conformation 81 | 0.157878 |
| 82 | Conformation 82 | 0.247905 |
| 83 | Conformation 83 | 0.217874 |
| 84 | Conformation 84 | 0.213966 |
| 85 | Conformation 85 | 0.222375 |
| 86 | Conformation 86 | 0.21871 |
| 87 | Conformation 87 | 0.205379 |
| 88 | Conformation 88 | 0.219191 |
| 89 | Conformation 89 | 0.223111 |
| 90 | Conformation 90 | 0.211785 |
| 91 | Conformation 91 | 0.206641 |
| 92 | Conformation 92 | 0.217034 |
| 93 | Conformation 93 | 0.224223 |
| 94 | Conformation 94 | 0.233273 |
| 95 | Conformation 95 | 0.227584 |
| 96 | Conformation 96 | 0.220882 |
| 97 | Conformation 97 | 0.223112 |
| 98 | Conformation 98 | 0.235633 |
| 99 | Conformation 99 | 0.230201 |
| 100 | Conformation 100 | 0.235503 |
| 101 | Average RMSD | 0.179757 |

**Table S7.** RMSF of the best conformation pose of compound **20i** docked in the protein active site of PDB: 4OAS.

| Entry | Item | RMSF |
| --- | --- | --- |
| 1 | GLN18 | 0.011331 |
| 2 | ILE19 | 0.010232 |
| 3 | PRO20 | 0.010008 |
| 4 | ALA21 | 0.009318 |
| 5 | SER22 | 0.009112 |
| 6 | GLU23 | 0.00891 |
| 7 | GLN24 | 0.008353 |
| 8 | GLU25 | 0.008069 |
| 9 | THR26 | 0.008002 |
| 10 | LEU27 | 0.00718 |
| 11 | VAL28 | 0.007217 |
| 12 | ARG29 | 0.006585 |
| 13 | PRO30 | 0.006738 |
| 14 | LYS31 | 0.007463 |
| 15 | PRO32 | 0.006716 |
| 16 | LEU33 | 0.007188 |
| 17 | LEU34 | 0.007069 |
| 18 | LEU35 | 0.006046 |
| 19 | LYS36 | 0.006215 |
| 20 | LEU37 | 0.006684 |
| 21 | LEU38 | 0.006271 |
| 22 | LYS39 | 0.005289 |
| 23 | SER40 | 0.005712 |
| 24 | VAL41 | 0.005839 |
| 25 | GLY42 | 0.005296 |
| 26 | ALA43 | 0.004883 |
| 27 | GLN44 | 0.004826 |
| 28 | LYS45 | 0.005271 |
| 29 | ASP46 | 0.005307 |
| 30 | THR47 | 0.006012 |
| 31 | TYR48 | 0.006191 |
| 32 | THR49 | 0.006953 |
| 33 | MET50 | 0.007832 |
| 34 | LYS51 | 0.007452 |
| 35 | GLU52 | 0.00654 |
| 36 | VAL53 | 0.007235 |
| 37 | LEU54 | 0.00793 |
| 38 | PHE55 | 0.00693 |
| 39 | TYR56 | 0.006329 |
| 40 | LEU57 | 0.007519 |
| 41 | GLY58 | 0.007571 |
| 42 | GLN59 | 0.006839 |
| 43 | TYR60 | 0.007008 |
| 44 | ILE61 | 0.007854 |
| 45 | MET62 | 0.007665 |
| 46 | THR63 | 0.006772 |
| 47 | LYS64 | 0.007124 |
| 48 | ARG65 | 0.007976 |
| 49 | LEU66 | 0.008206 |
| 50 | TYR67 | 0.008945 |
| 51 | ASP68 | 0.009059 |
| 52 | GLU69 | 0.009376 |
| 53 | LYS70 | 0.009788 |
| 54 | GLN71 | 0.009868 |
| 55 | GLN72 | 0.010789 |
| 56 | HIS73 | 0.010318 |
| 57 | ILE74 | 0.010112 |
| 58 | VAL75 | 0.009222 |
| 59 | TYR76 | 0.009593 |
| 60 | CYS77 | 0.008856 |
| 61 | SER78 | 0.009158 |
| 62 | ASN79 | 0.008432 |
| 63 | ASP80 | 0.007897 |
| 64 | LEU81 | 0.007582 |
| 65 | LEU82 | 0.008155 |
| 66 | GLY83 | 0.008749 |
| 67 | ASP84 | 0.008501 |
| 68 | LEU85 | 0.008163 |
| 69 | PHE86 | 0.008908 |
| 70 | GLY87 | 0.009663 |
| 71 | VAL88 | 0.009802 |
| 72 | PRO89 | 0.009964 |
| 73 | SER90 | 0.010038 |
| 74 | PHE91 | 0.009585 |
| 75 | SER92 | 0.010277 |
| 76 | VAL93 | 0.009681 |
| 77 | LYS94 | 0.010496 |
| 78 | GLU95 | 0.010766 |
| 79 | HIS96 | 0.009967 |
| 80 | ARG97 | 0.010994 |
| 81 | LYS98 | 0.01065 |
| 82 | ILE99 | 0.009448 |
| 83 | TYR100 | 0.009307 |
| 84 | THR101 | 0.009865 |
| 85 | MET102 | 0.009383 |
| 86 | ILE103 | 0.00851 |
| 87 | TYR104 | 0.008784 |
| 88 | ARG105 | 0.009421 |
| 89 | ASN106 | 0.008118 |
| 90 | LEU107 | 0.007929 |
| 91 | VAL108 | 0.007662 |
| 92 | VAL109 | 0.007989 |
| 93 | VAL110 | 0.007274 |

**Table S8.** RMSF of the best conformation pose of compound **20k** docked in the protein active site of PDB: 4OAS.

| Entry | Item | RMSF |
| --- | --- | --- |
| 1 | GLN18 | 0.010478 |
| 2 | ILE19 | 0.009449 |
| 3 | PRO20 | 0.009206 |
| 4 | ALA21 | 0.008499 |
| 5 | SER22 | 0.008355 |
| 6 | GLU23 | 0.008243 |
| 7 | GLN24 | 0.007641 |
| 8 | GLU25 | 0.007368 |
| 9 | THR26 | 0.007405 |
| 10 | LEU27 | 0.006669 |
| 11 | VAL28 | 0.006799 |
| 12 | ARG29 | 0.006276 |
| 13 | PRO30 | 0.006526 |
| 14 | LYS31 | 0.007308 |
| 15 | PRO32 | 0.006747 |
| 16 | LEU33 | 0.007242 |
| 17 | LEU34 | 0.006934 |
| 18 | LEU35 | 0.006017 |
| 19 | LYS36 | 0.006386 |
| 20 | LEU37 | 0.006609 |
| 21 | LEU38 | 0.006089 |
| 22 | LYS39 | 0.005326 |
| 23 | SER40 | 0.005663 |
| 24 | VAL41 | 0.005577 |
| 25 | GLY42 | 0.00506 |
| 26 | ALA43 | 0.004659 |
| 27 | GLN44 | 0.004793 |
| 28 | LYS45 | 0.004986 |
| 29 | ASP46 | 0.005114 |
| 30 | THR47 | 0.005655 |
| 31 | TYR48 | 0.005849 |
| 32 | THR49 | 0.006412 |
| 33 | MET50 | 0.007285 |
| 34 | LYS51 | 0.00676 |
| 35 | GLU52 | 0.006022 |
| 36 | VAL53 | 0.006798 |
| 37 | LEU54 | 0.007368 |
| 38 | PHE55 | 0.006322 |
| 39 | TYR56 | 0.005905 |
| 40 | LEU57 | 0.007155 |
| 41 | GLY58 | 0.007079 |
| 42 | GLN59 | 0.006327 |
| 43 | TYR60 | 0.006836 |
| 44 | ILE61 | 0.007487 |
| 45 | MET62 | 0.007139 |
| 46 | THR63 | 0.006428 |
| 47 | LYS64 | 0.007015 |
| 48 | ARG65 | 0.007589 |
| 49 | LEU66 | 0.007982 |
| 50 | TYR67 | 0.008389 |
| 51 | ASP68 | 0.008516 |
| 52 | GLU69 | 0.008563 |
| 53 | LYS70 | 0.009179 |
| 54 | GLN71 | 0.009375 |
| 55 | GLN72 | 0.010091 |
| 56 | HIS73 | 0.0096 |
| 57 | ILE74 | 0.009642 |
| 58 | VAL75 | 0.008861 |
| 59 | TYR76 | 0.009343 |
| 60 | CYS77 | 0.008768 |
| 61 | SER78 | 0.009224 |
| 62 | ASN79 | 0.008565 |
| 63 | ASP80 | 0.007969 |
| 64 | LEU81 | 0.007688 |
| 65 | LEU82 | 0.008042 |
| 66 | GLY83 | 0.008719 |
| 67 | ASP84 | 0.008583 |
| 68 | LEU85 | 0.008077 |
| 69 | PHE86 | 0.008668 |
| 70 | GLY87 | 0.009535 |
| 71 | VAL88 | 0.009625 |
| 72 | PRO89 | 0.009843 |
| 73 | SER90 | 0.009775 |
| 74 | PHE91 | 0.009198 |
| 75 | SER92 | 0.009716 |
| 76 | VAL93 | 0.00905 |
| 77 | LYS94 | 0.009715 |
| 78 | GLU95 | 0.010141 |
| 79 | HIS96 | 0.009267 |
| 80 | ARG97 | 0.010296 |
| 81 | LYS98 | 0.010113 |
| 82 | ILE99 | 0.008938 |
| 83 | TYR100 | 0.008721 |
| 84 | THR101 | 0.00939 |
| 85 | MET102 | 0.009022 |
| 86 | ILE103 | 0.008121 |
| 87 | TYR104 | 0.008321 |
| 88 | ARG105 | 0.009071 |
| 89 | ASN106 | 0.007902 |
| 90 | LEU107 | 0.007584 |
| 91 | VAL108 | 0.007309 |
| 92 | VAL109 | 0.007518 |
| 93 | VAL110 | 0.006809 |


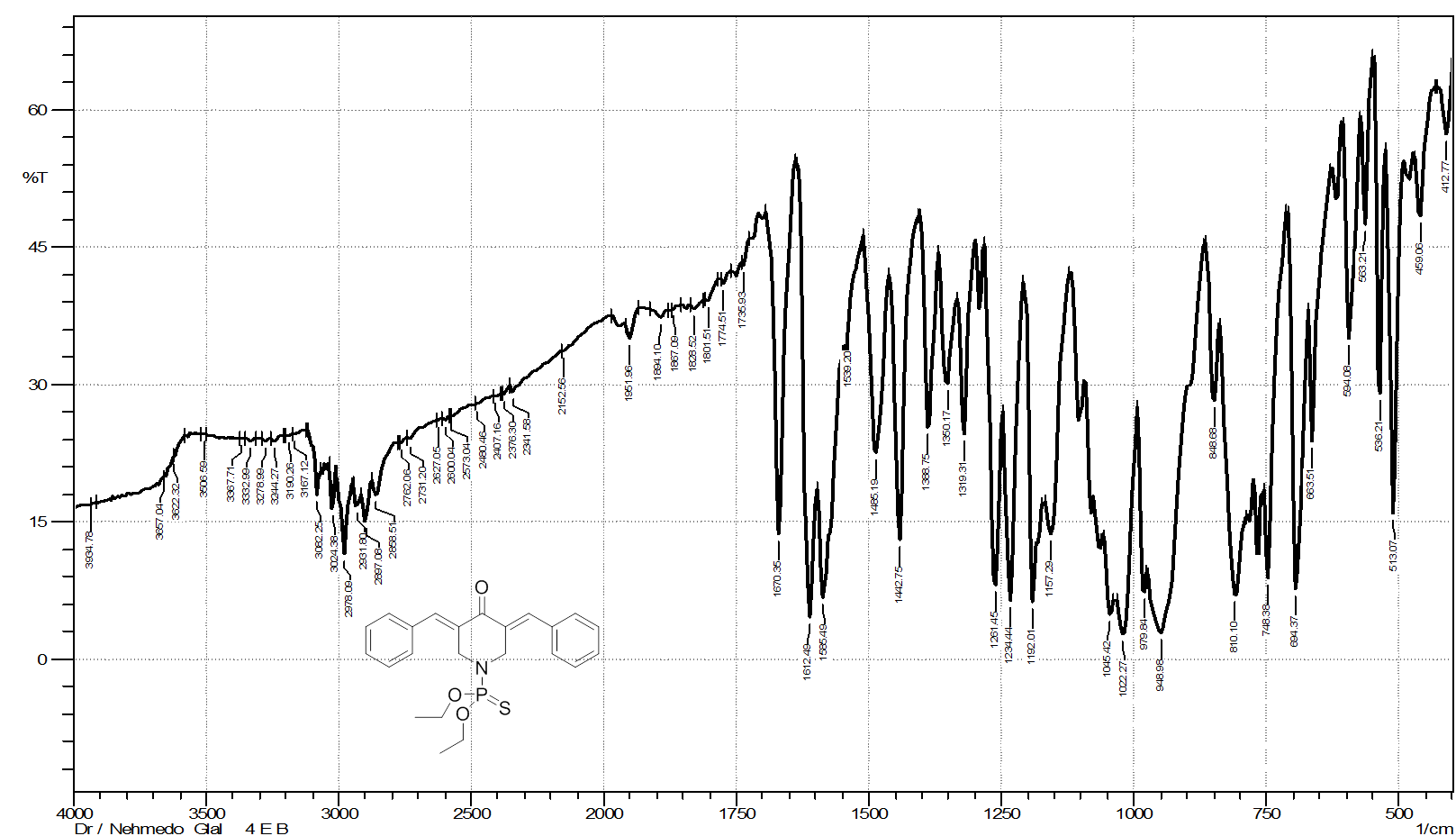


**Fig. S1.** IR spectrum of compound **20a** (KBr pellet).


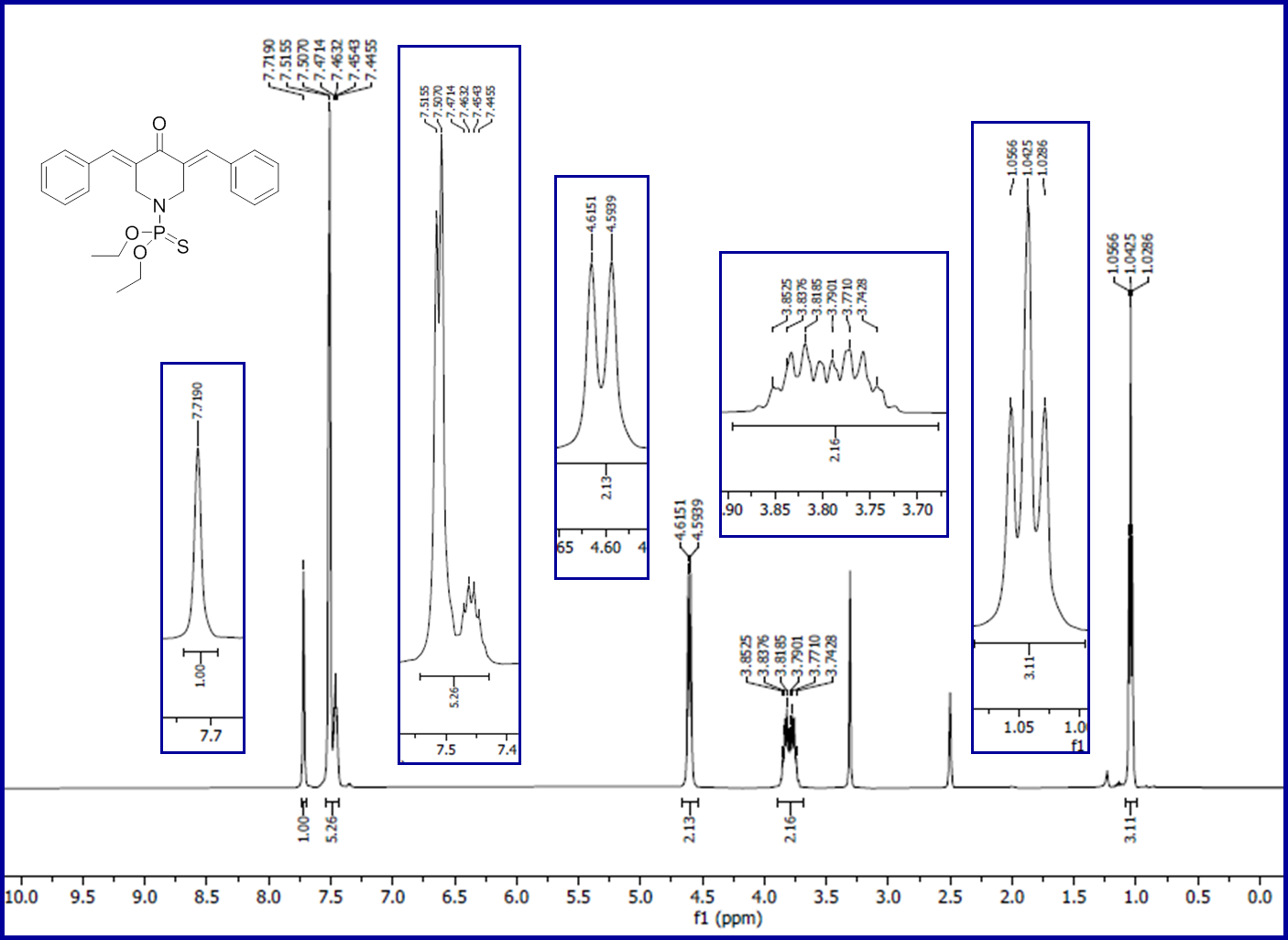


**Fig. S2.** ^1^H-NMR spectrum of compound **20a** in DMSO-*d6*.


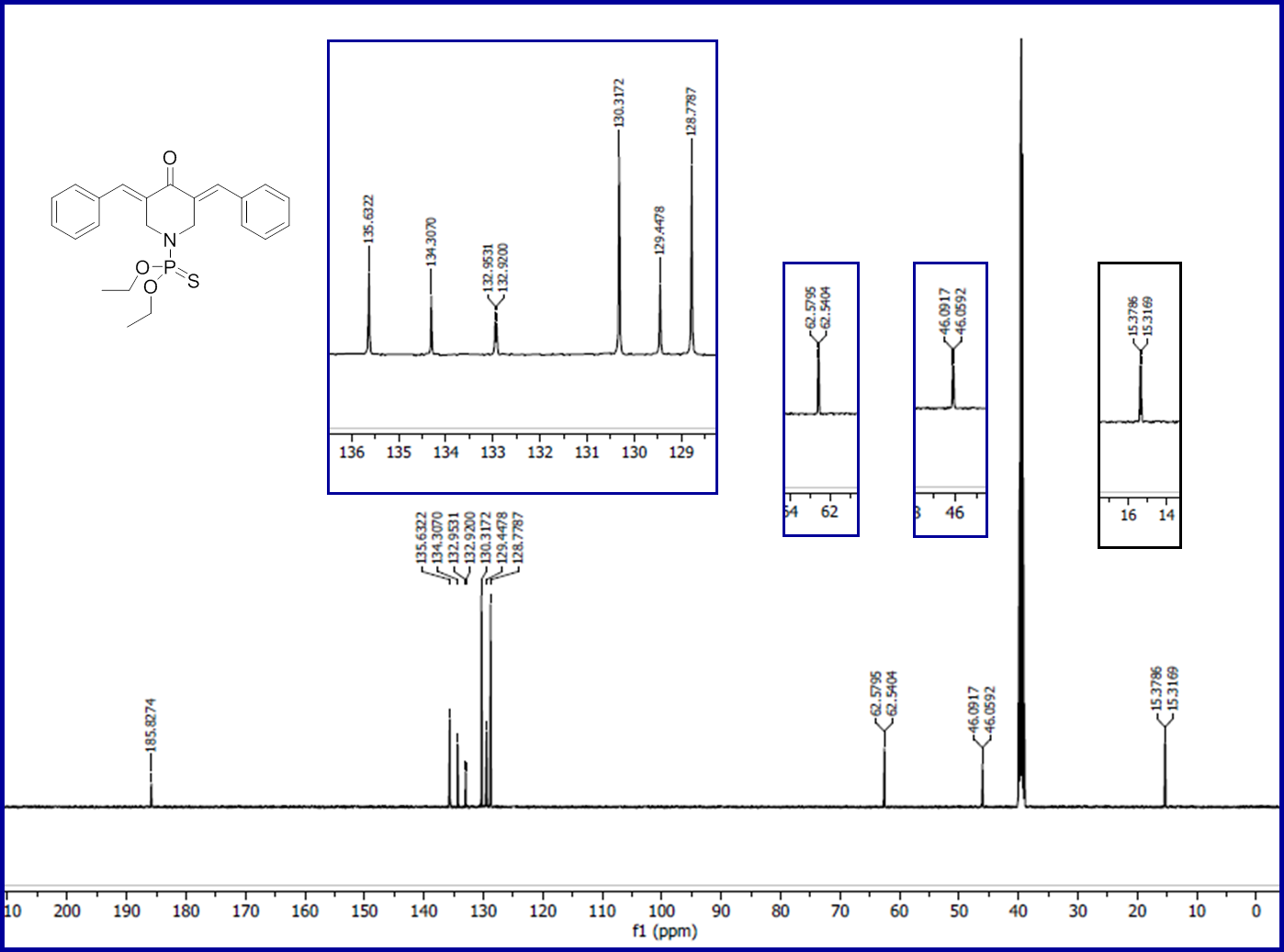


**Fig. S3.** ^13^C-NMR spectrum of compound **20a** in DMSO-*d6*.


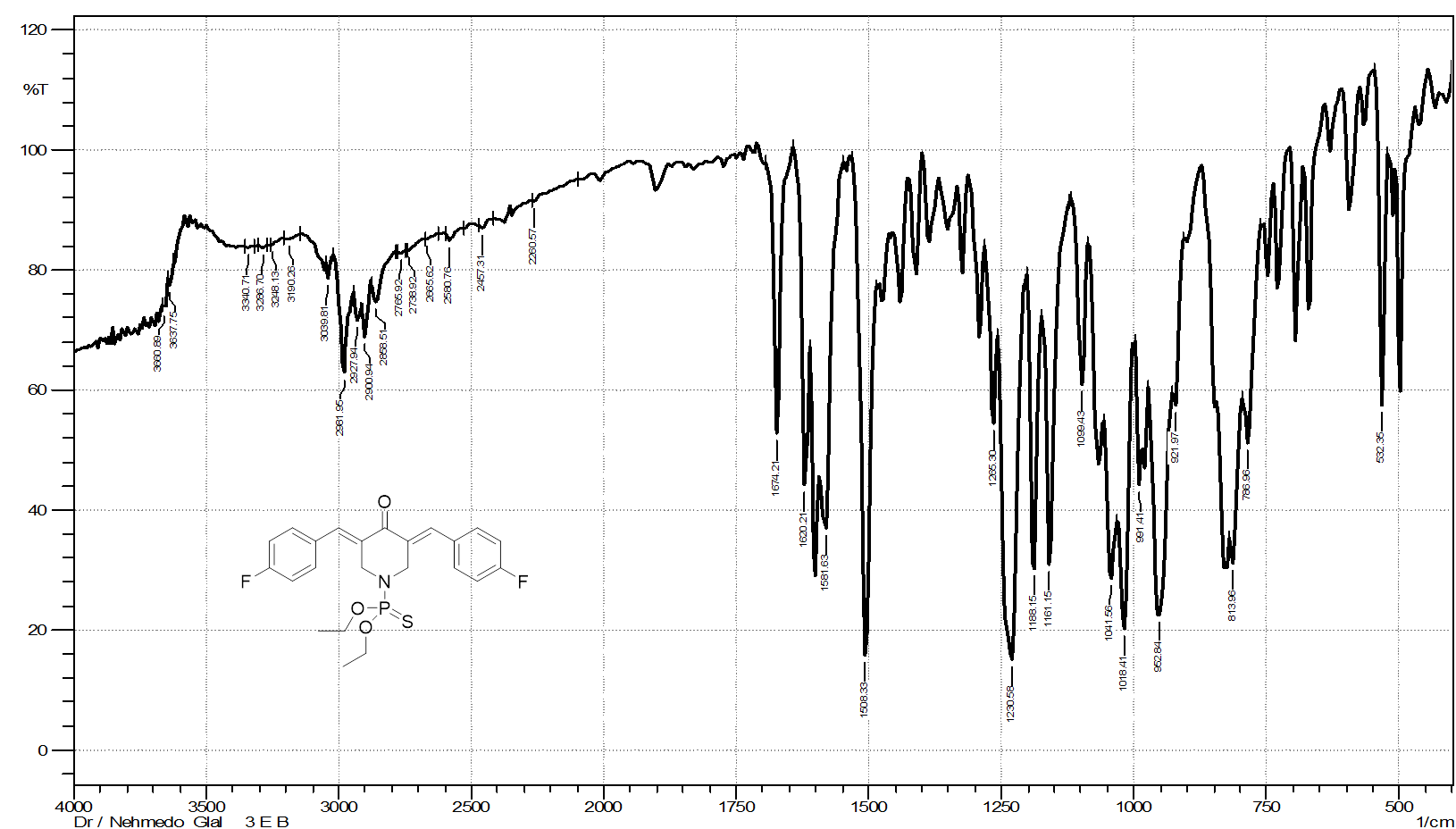


**Fig. S4.** IR spectrum of compound **20b** (KBr pellet).


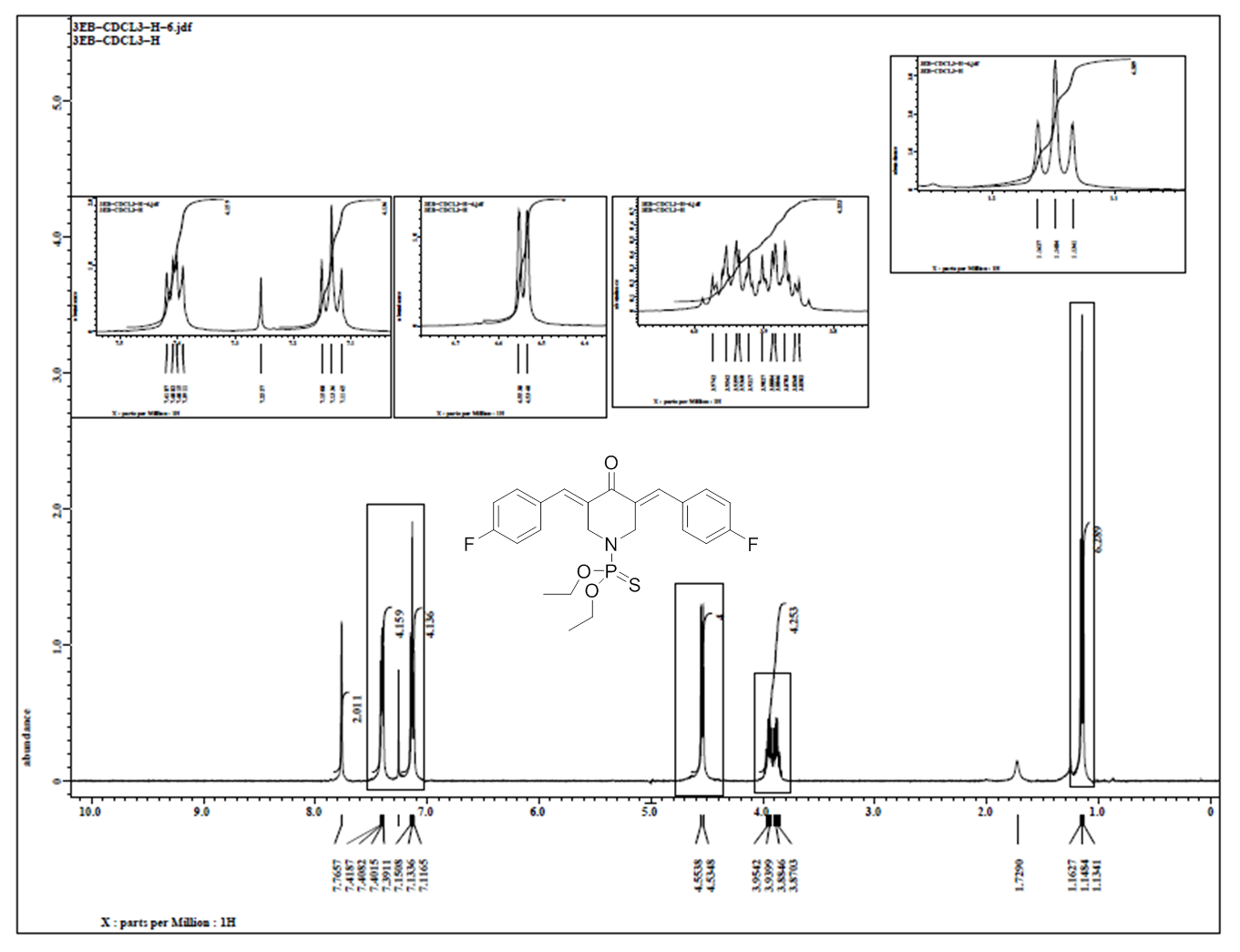


**Fig. S5.** ^1^H-NMR spectrum of compound **20b** in CDCl_3_.


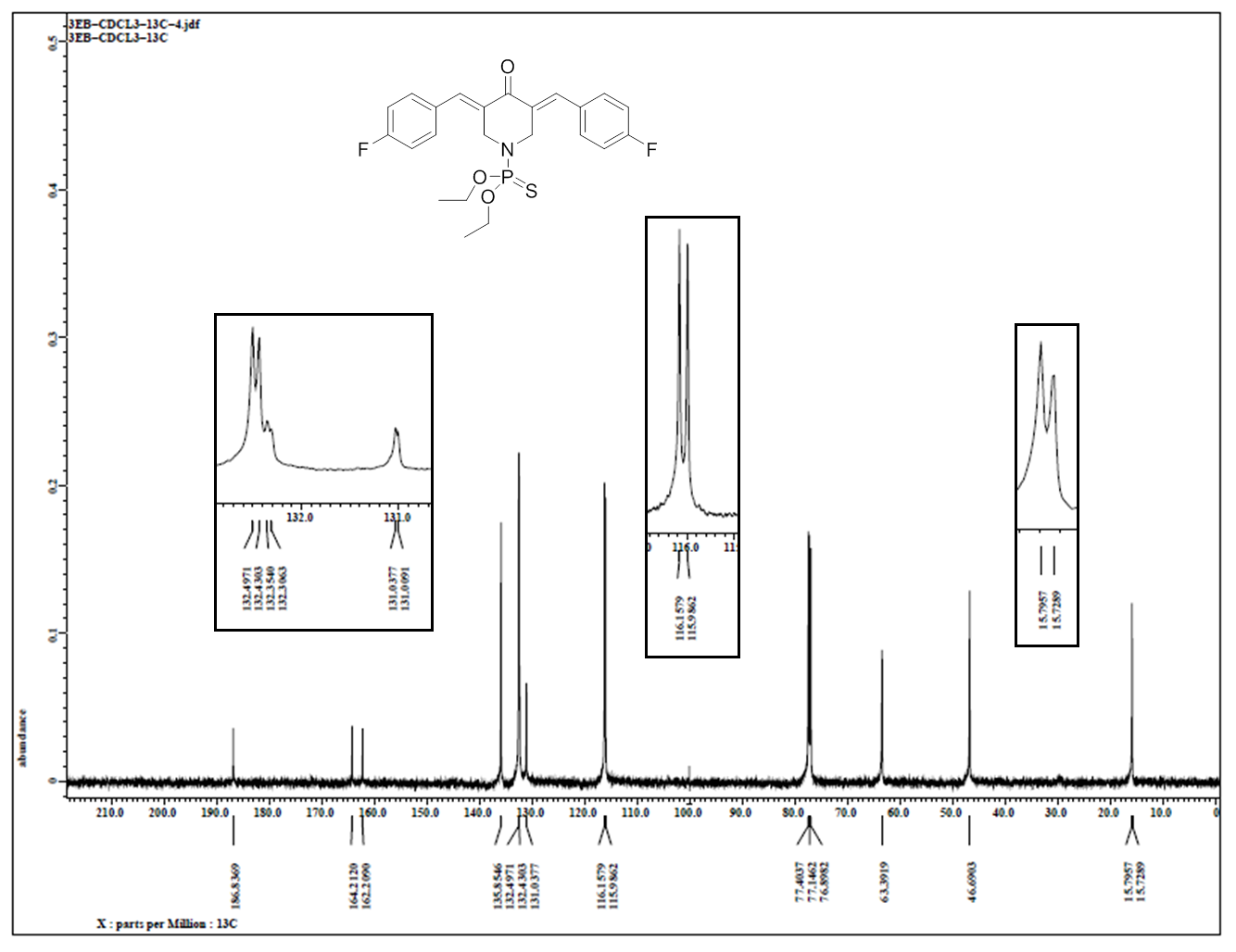


**Fig. S6.** ^13^C-NMR spectrum of compound **20b** in CDCl_3_.


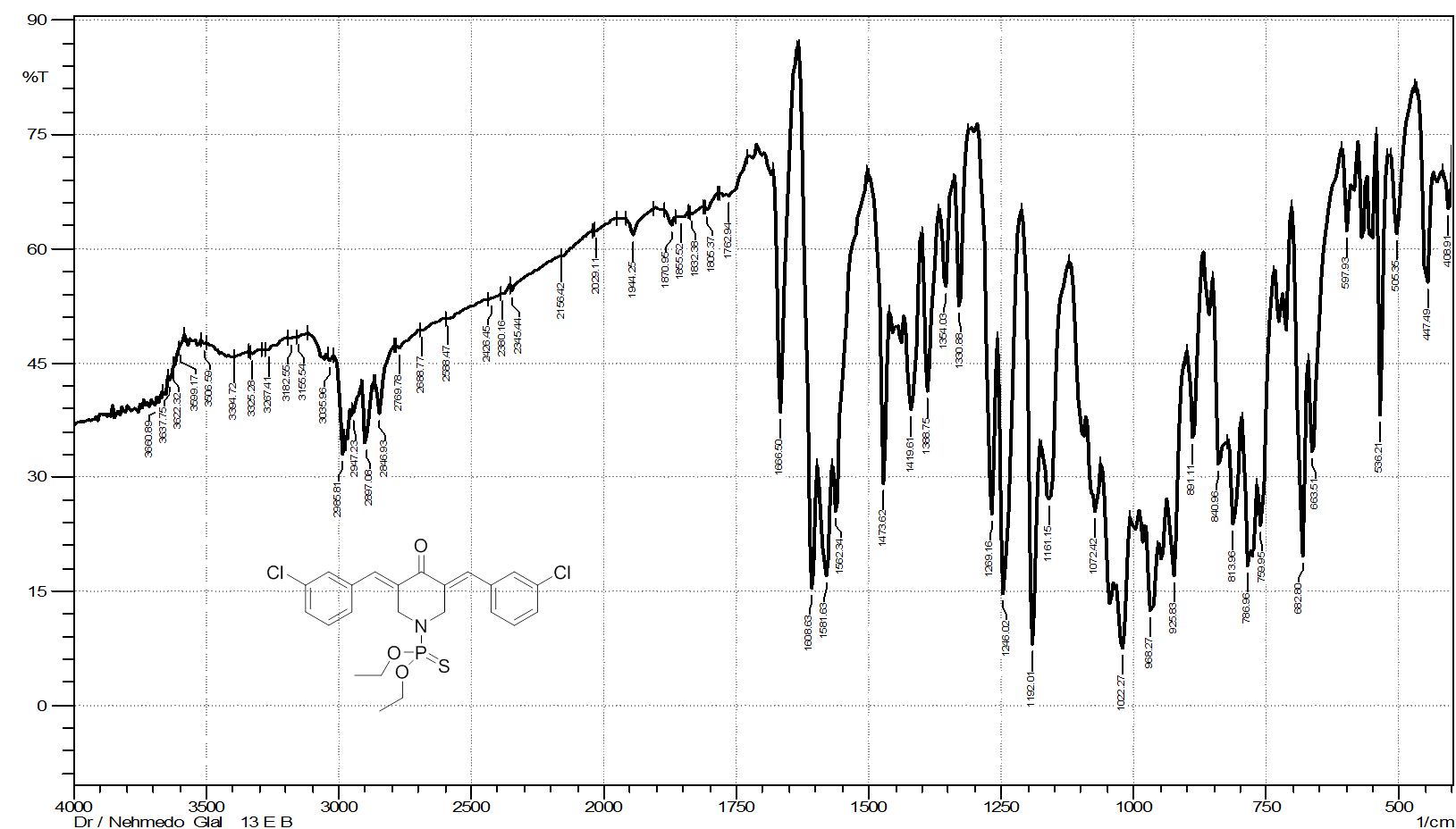


**Fig. S7.** IR spectrum of compound **20c** (KBr pellet).


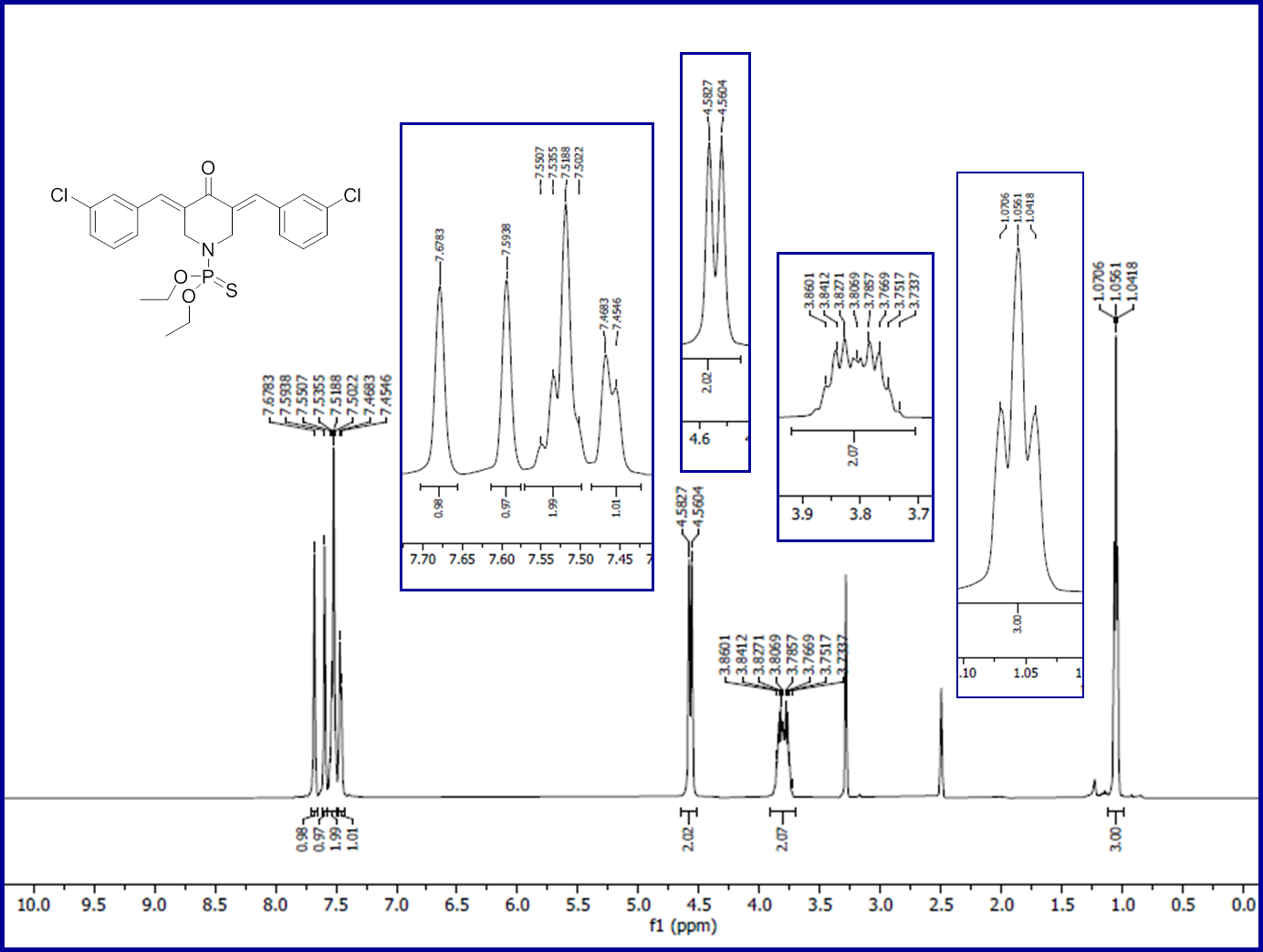


**Fig. S8.** ^1^H-NMR spectrum of compound **20c** in DMSO-*d6*.


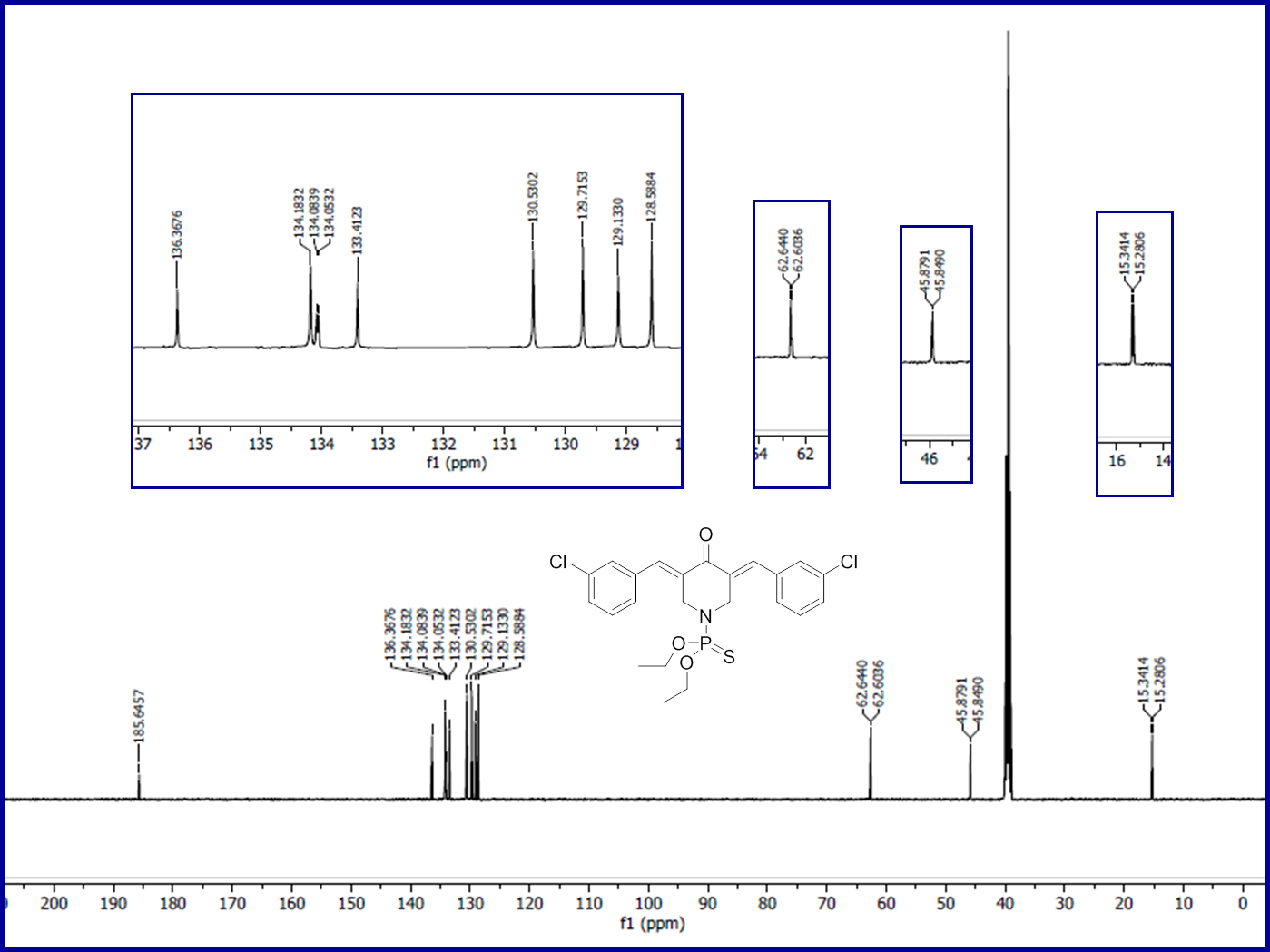


**Fig. S9.** ^13^C-NMR spectrum of compound **20c** in DMSO-*d6*.


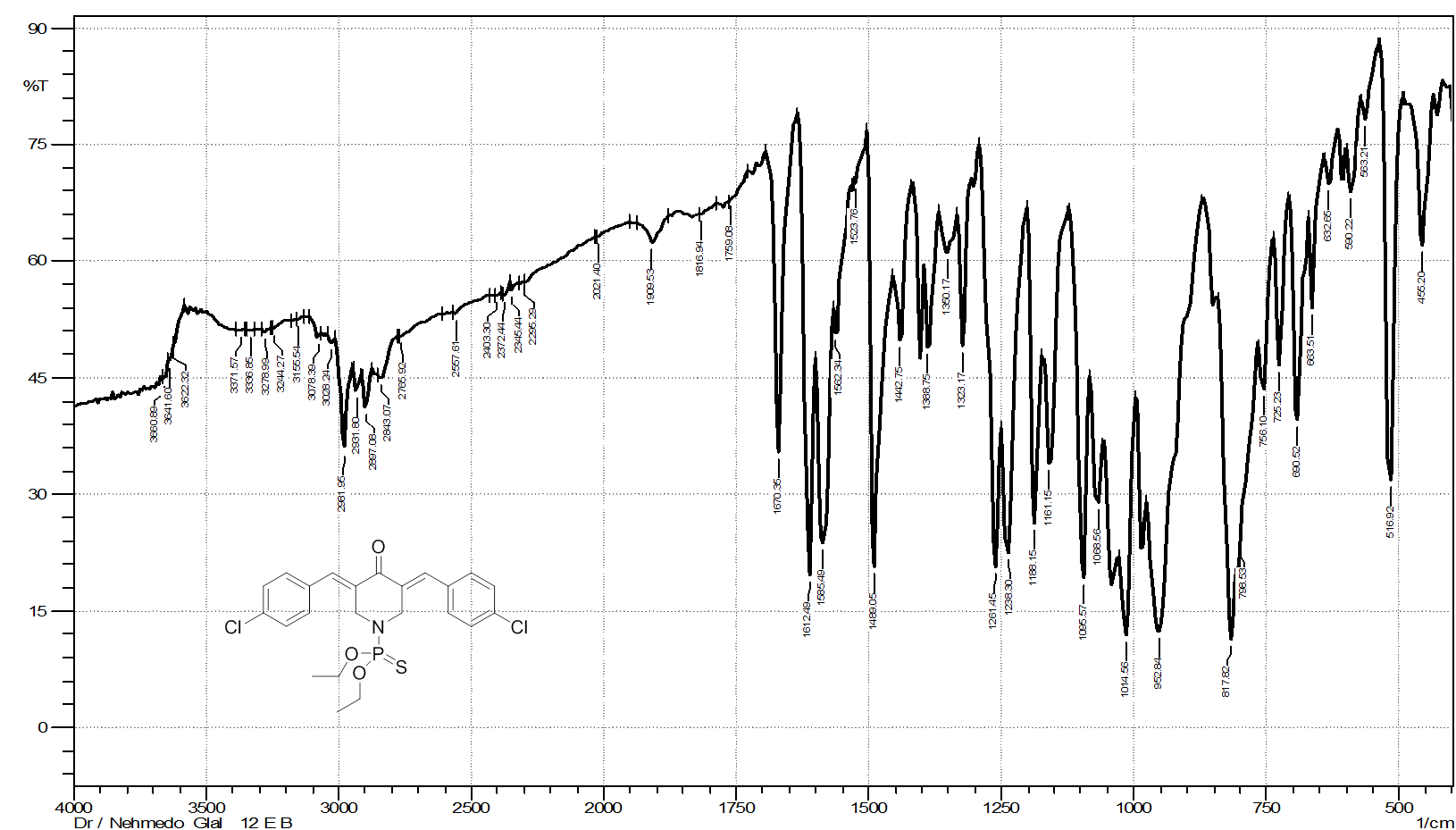


**Fig. S10.** IR spectrum of compound **20d** (KBr pellet).


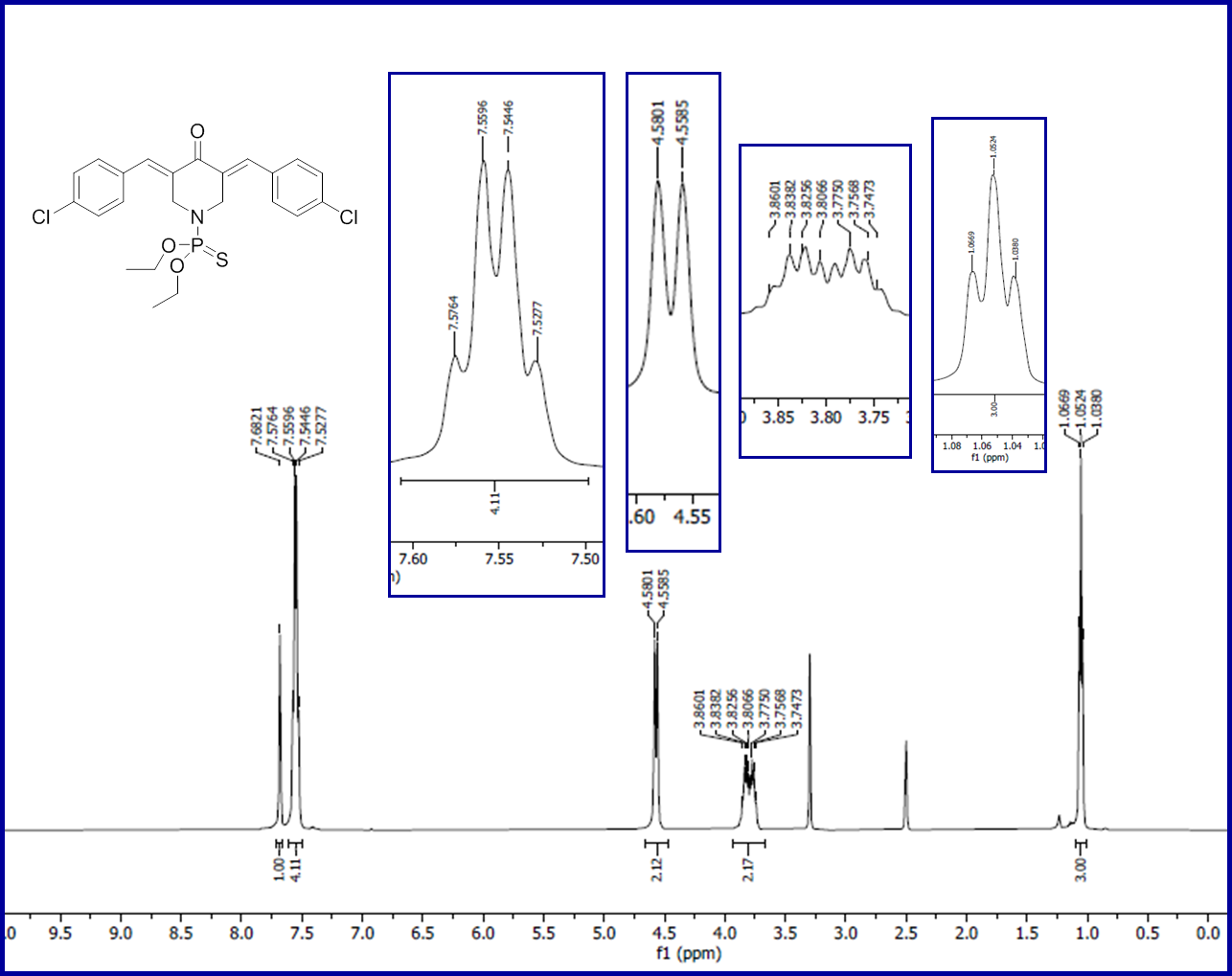


**Fig. S11.** ^1^H-NMR spectrum of compound **20d** in DMSO-*d6*.


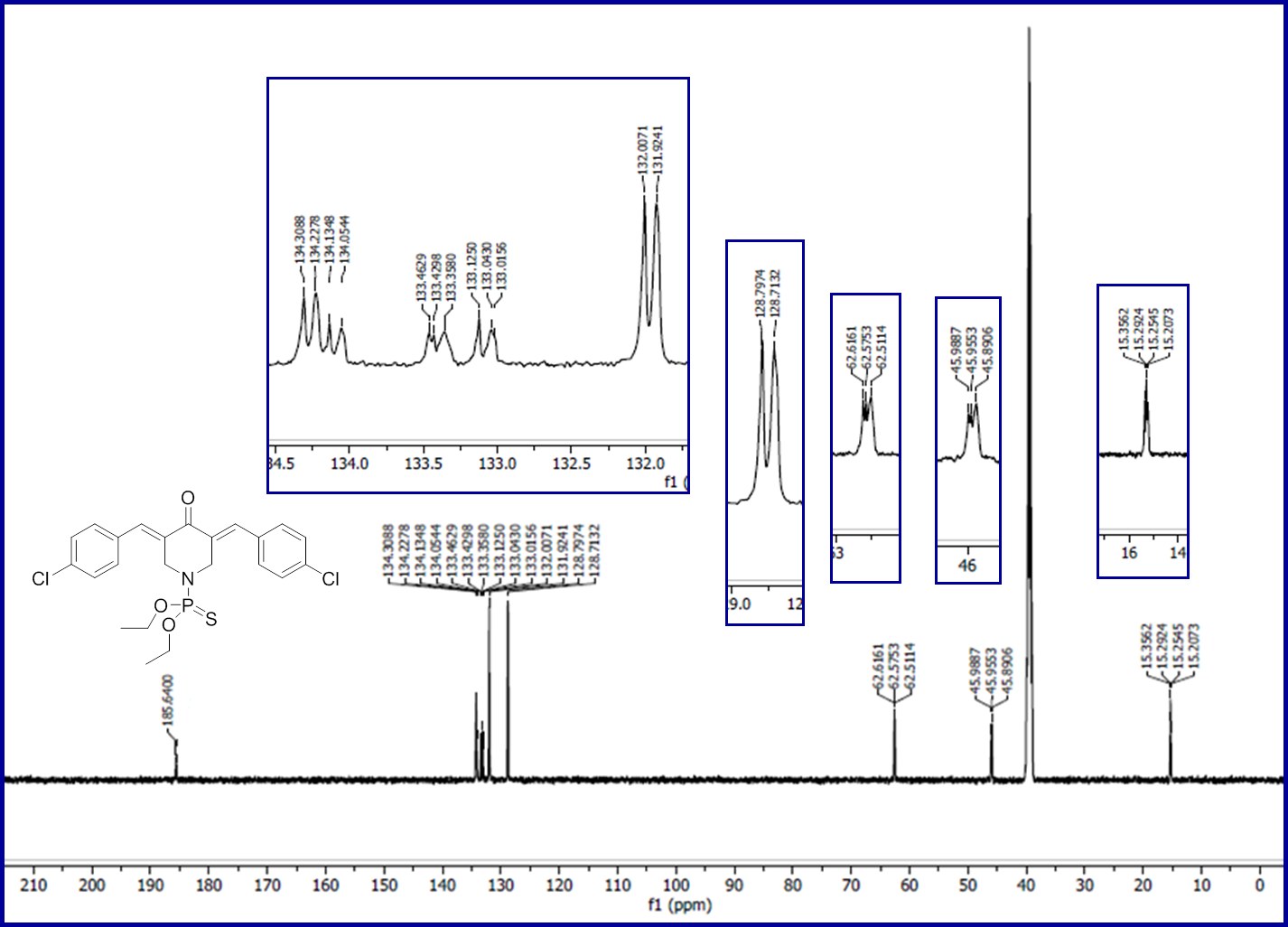


**Fig. S12.** ^13^C-NMR spectrum of compound **20d** in DMSO-*d6*.


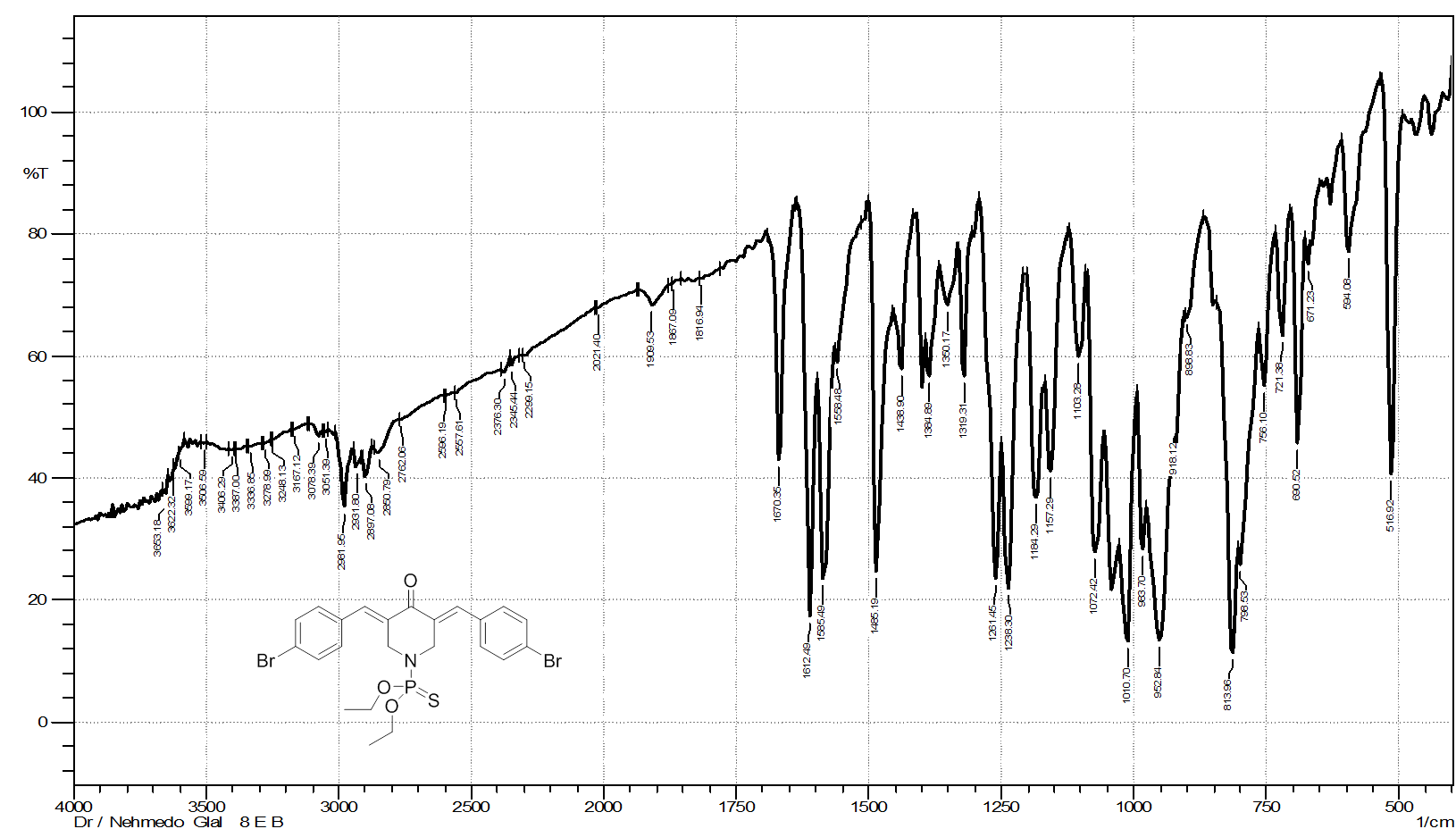


**Fig. S13.** IR spectrum of compound **20e** (KBr pellet).


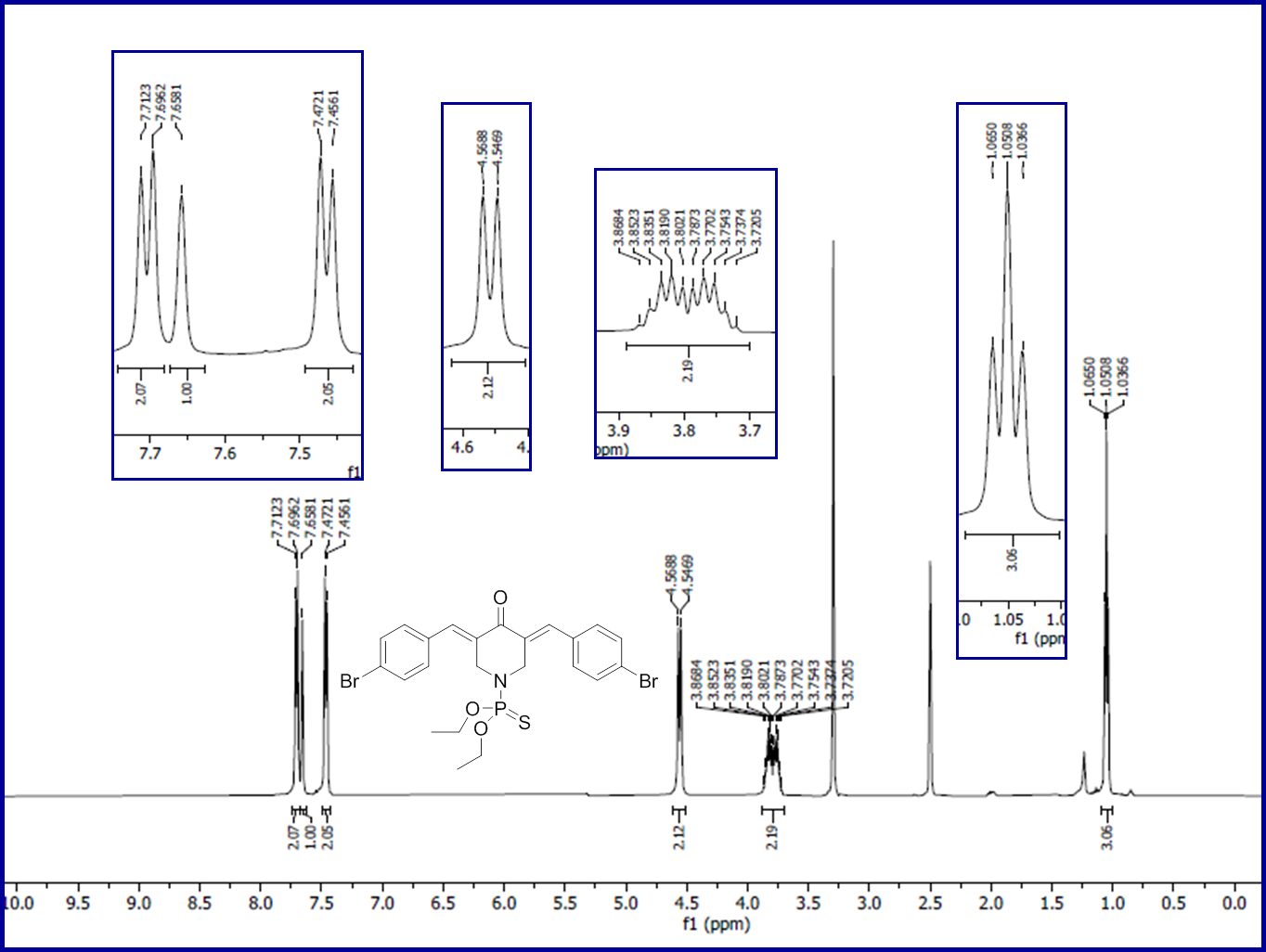


**Fig. S14.** ^1^H-NMR spectrum of compound **20e** in DMSO-*d6*.


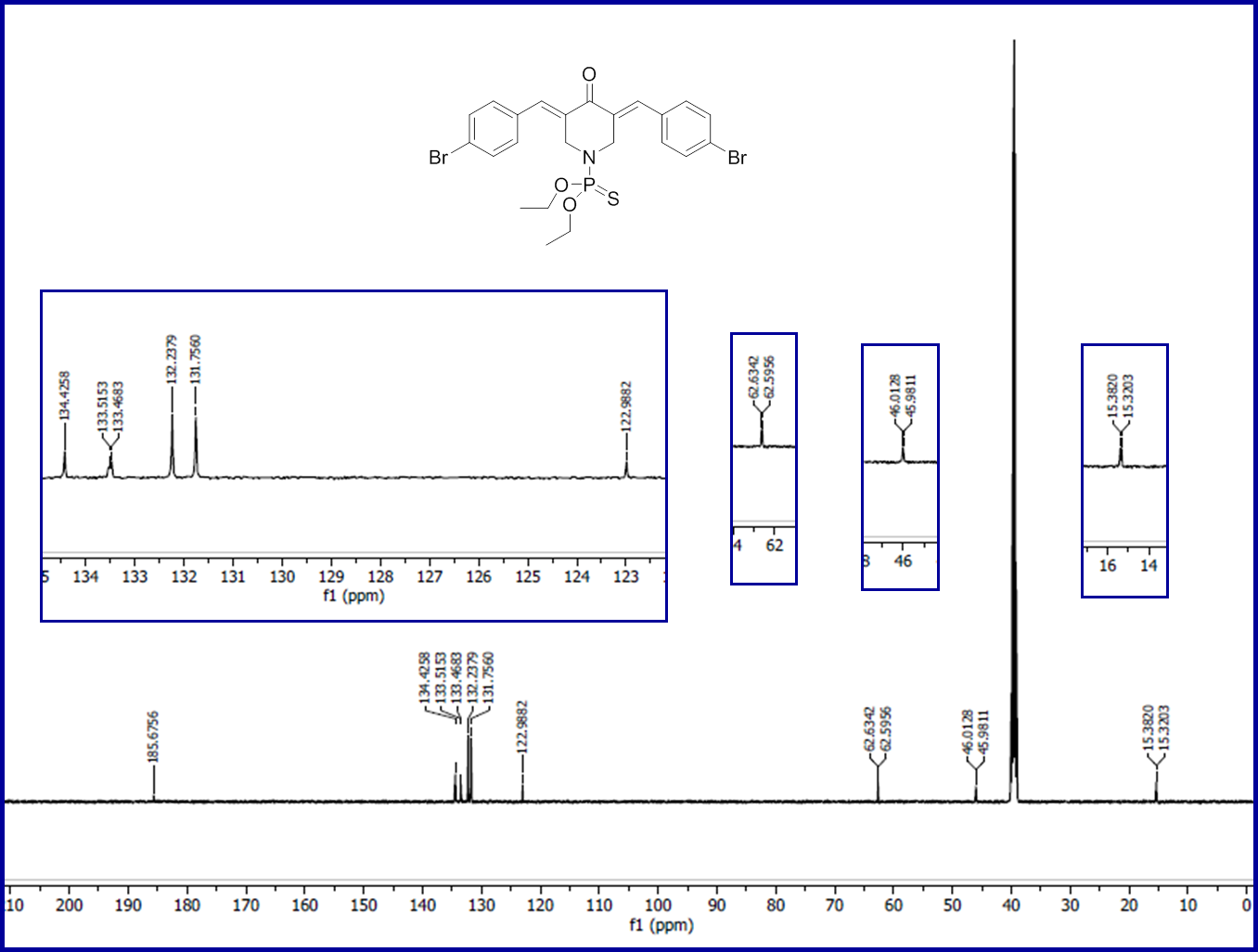


**Fig. S15.** ^13^C-NMR spectrum of compound **20e** in DMSO-*d6*.


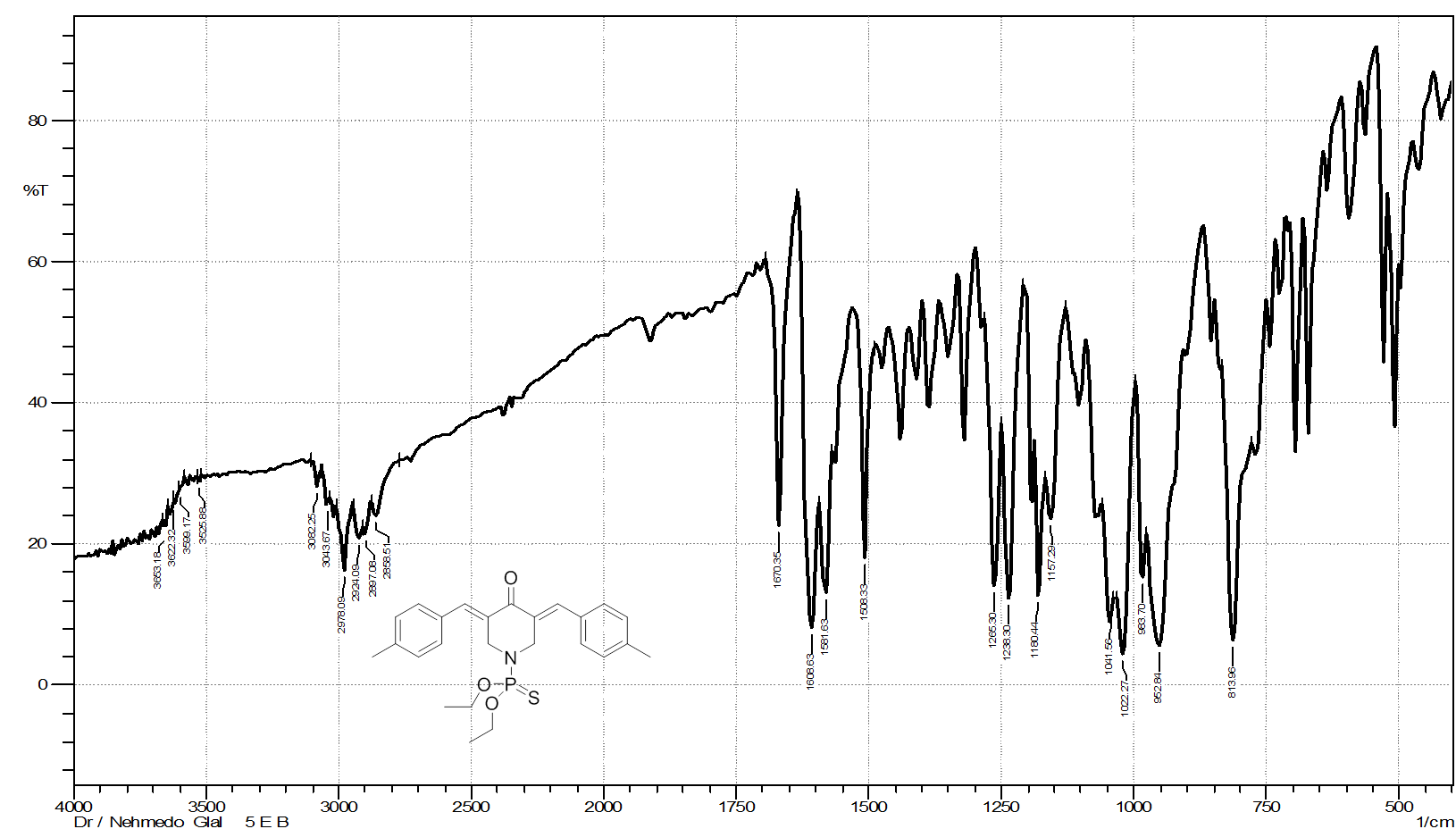


**Fig. S16.** IR spectrum of compound **20f** (KBr pellet).


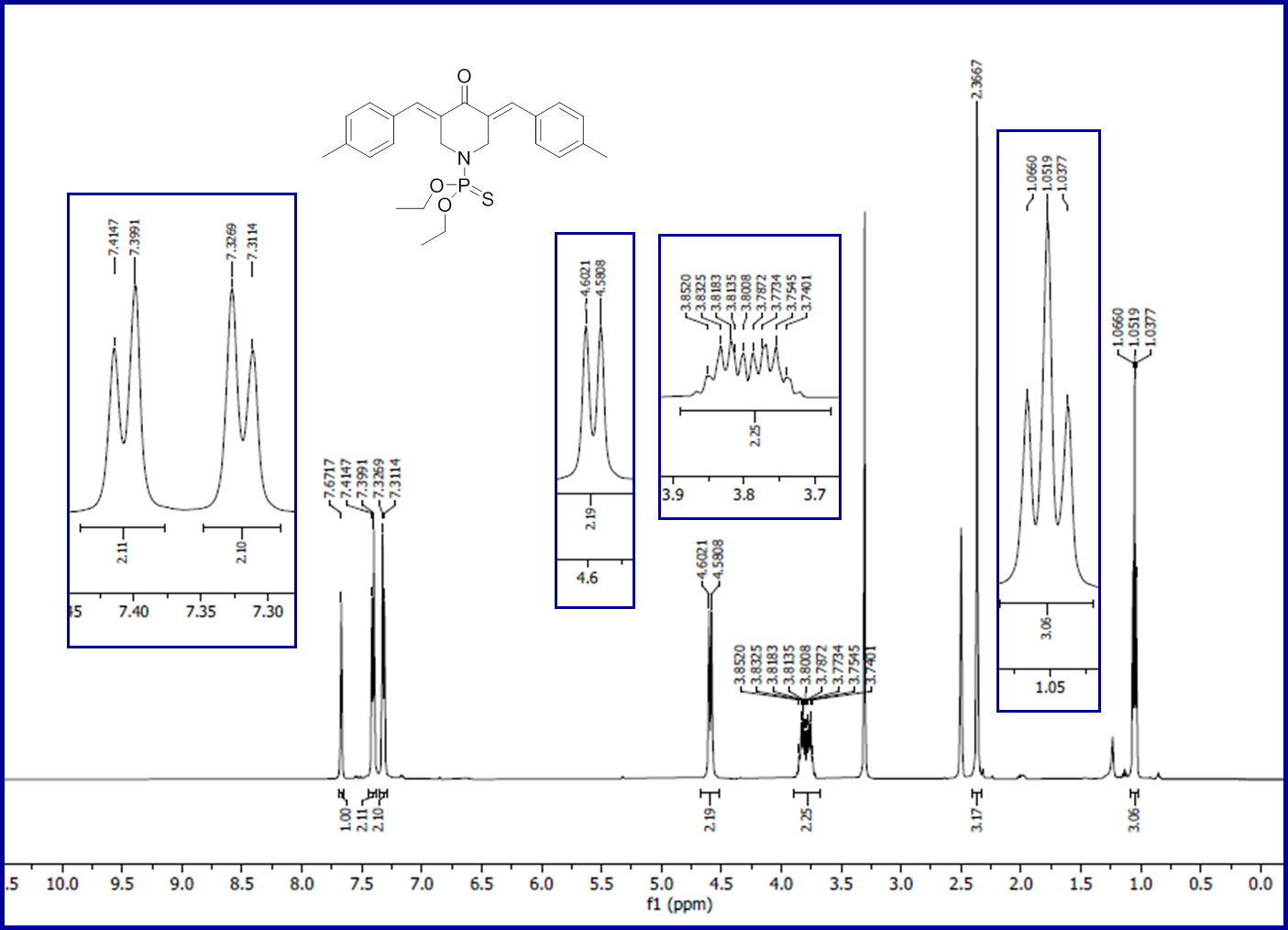


**Fig. S17.** ^1^H-NMR spectrum of compound **20f** in DMSO-*d6*.


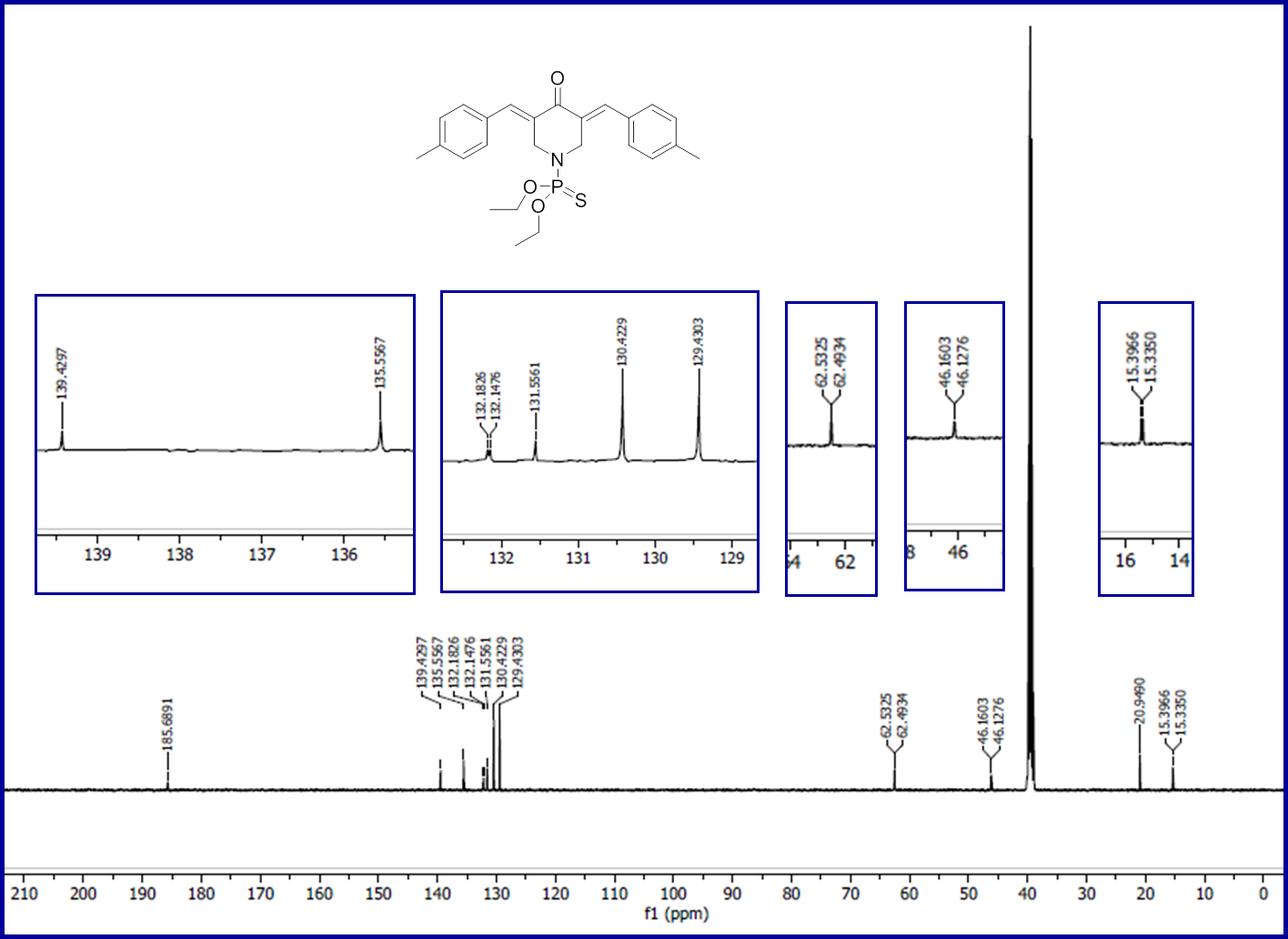


**Fig. S18.** ^13^C-NMR spectrum of compound **20f** in DMSO-*d6*.


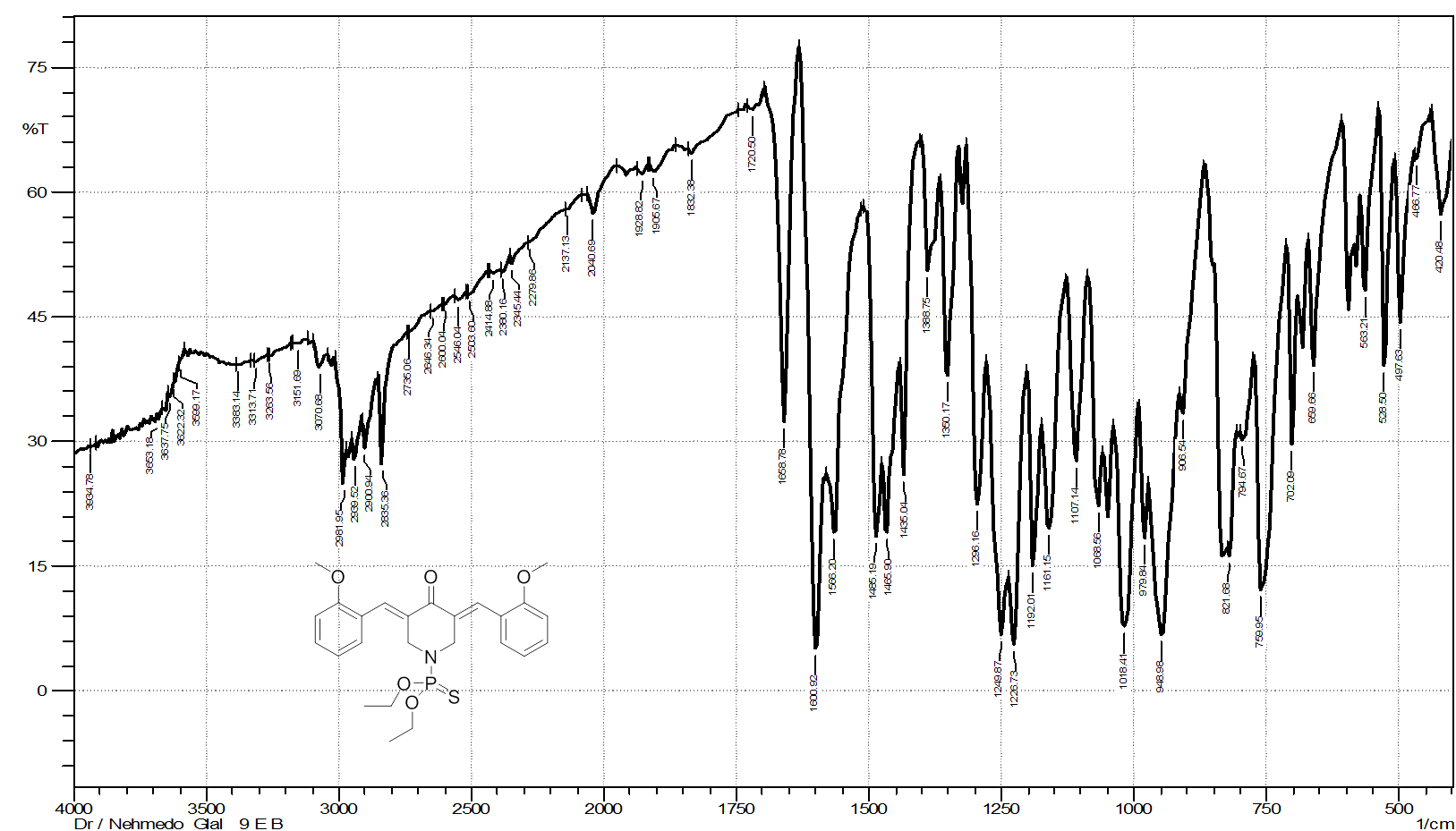


**Fig. S19.** IR spectrum of compound **20g** (KBr pellet).


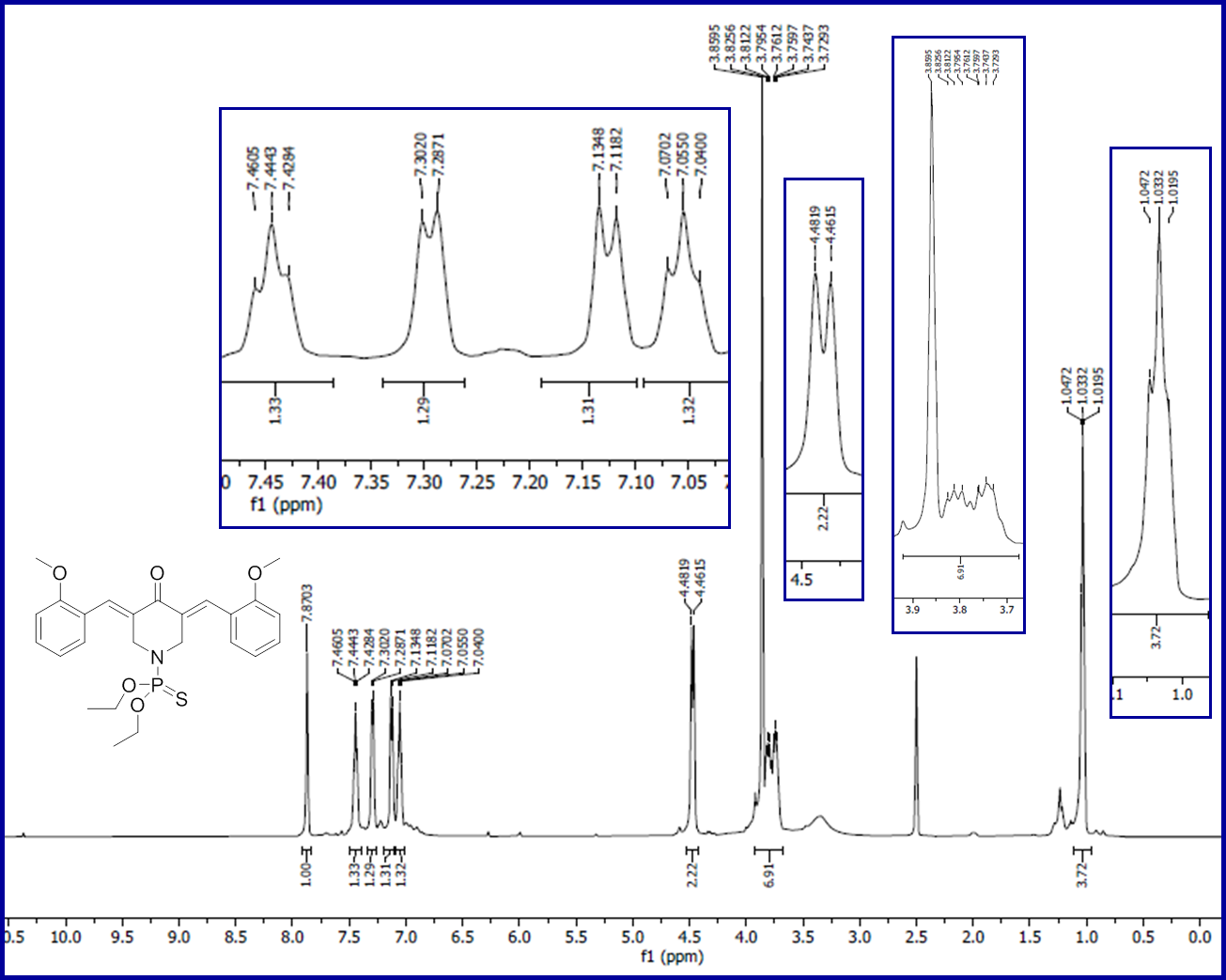


**Fig. S20.** ^1^H-NMR spectrum of compound **20g** in DMSO-*d6*.


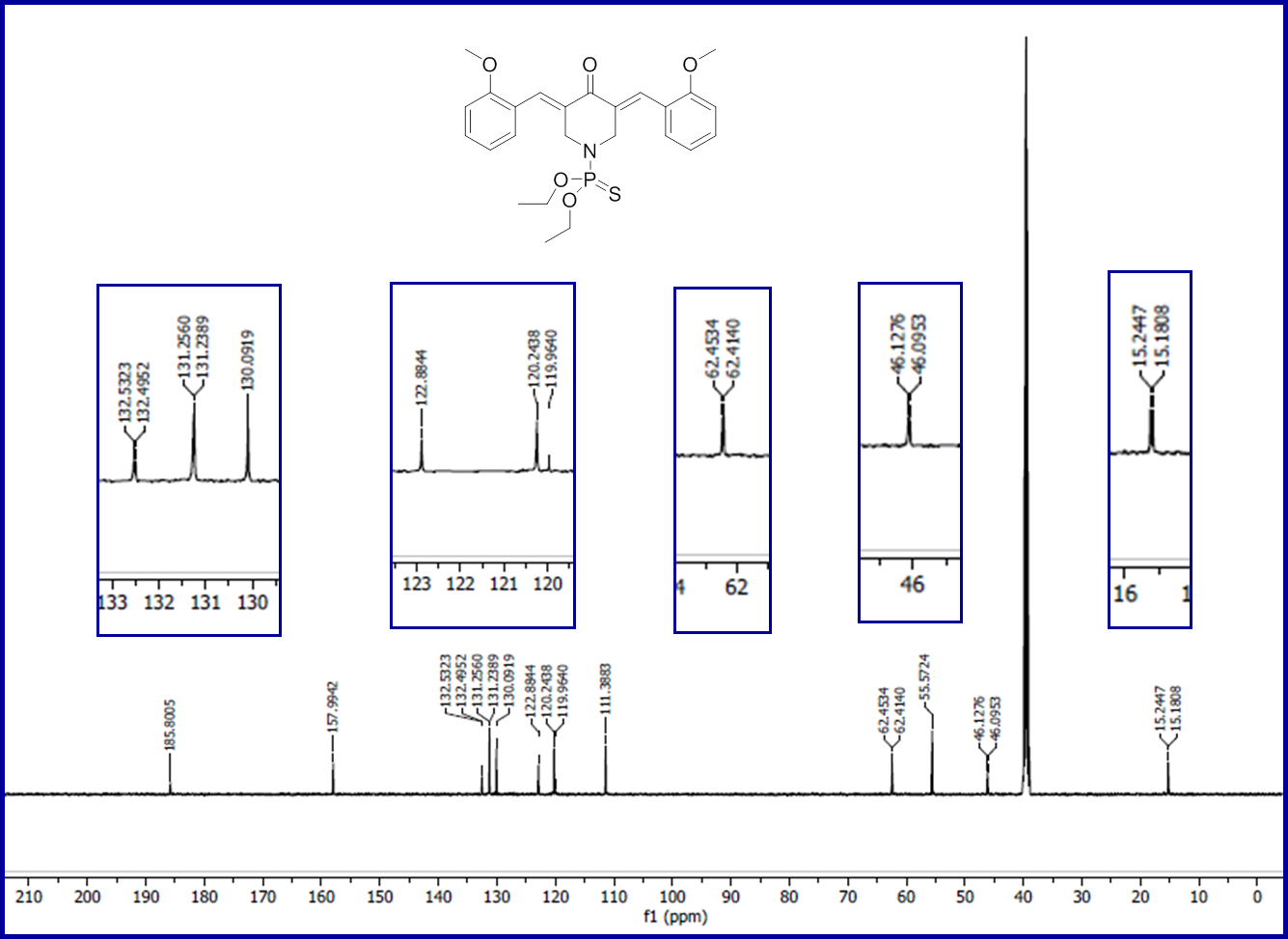


**Fig. S21.** ^13^C-NMR spectrum of compound **20g** in DMSO-*d6*.


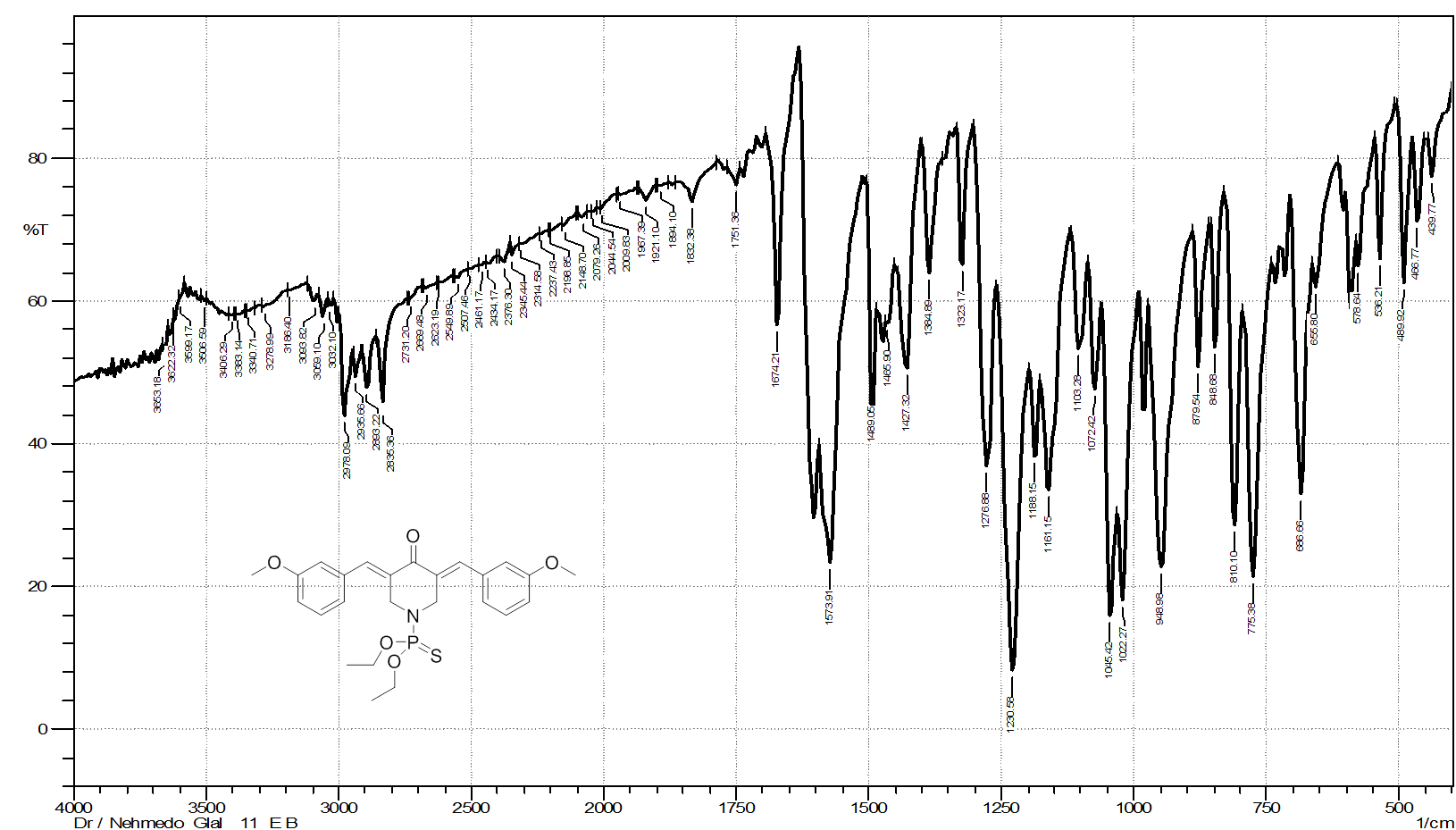


**Fig. S22.** IR spectrum of compound **20h** (KBr pellet).


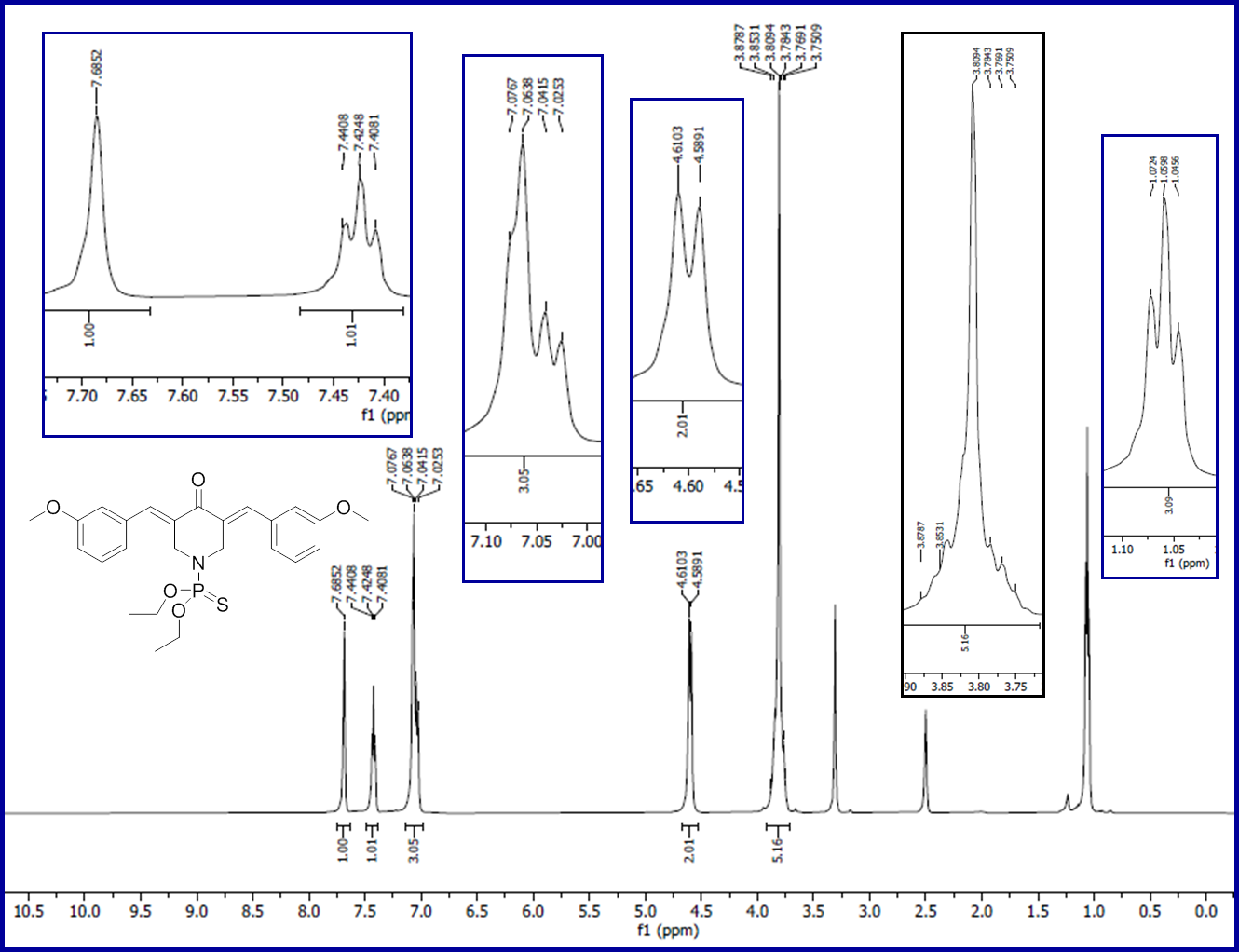


**Fig. S23.** ^1^H-NMR spectrum of compound **20h** in DMSO-*d6*.


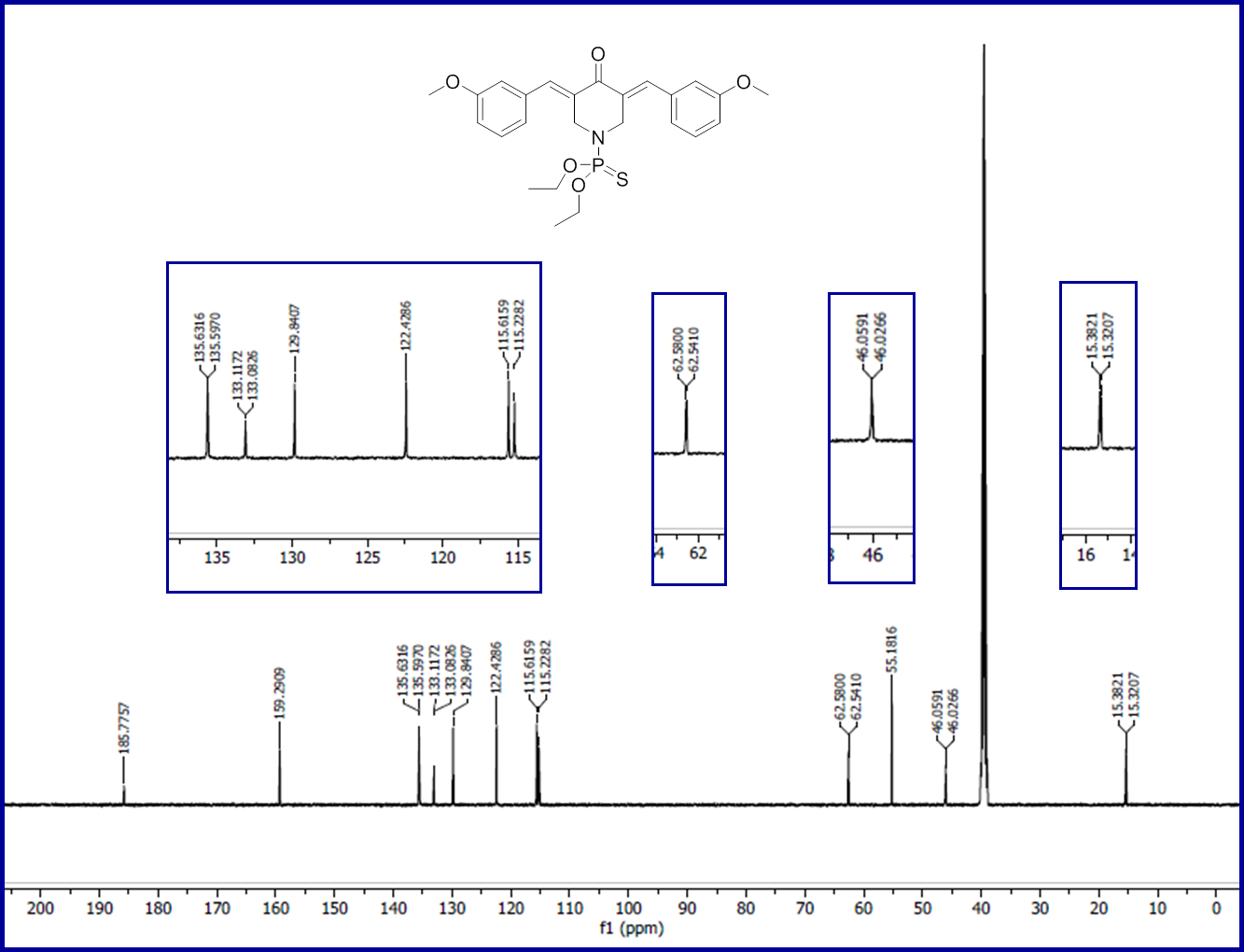


**Fig. S24.** ^13^C-NMR spectrum of compound **20h** in DMSO-*d6*.


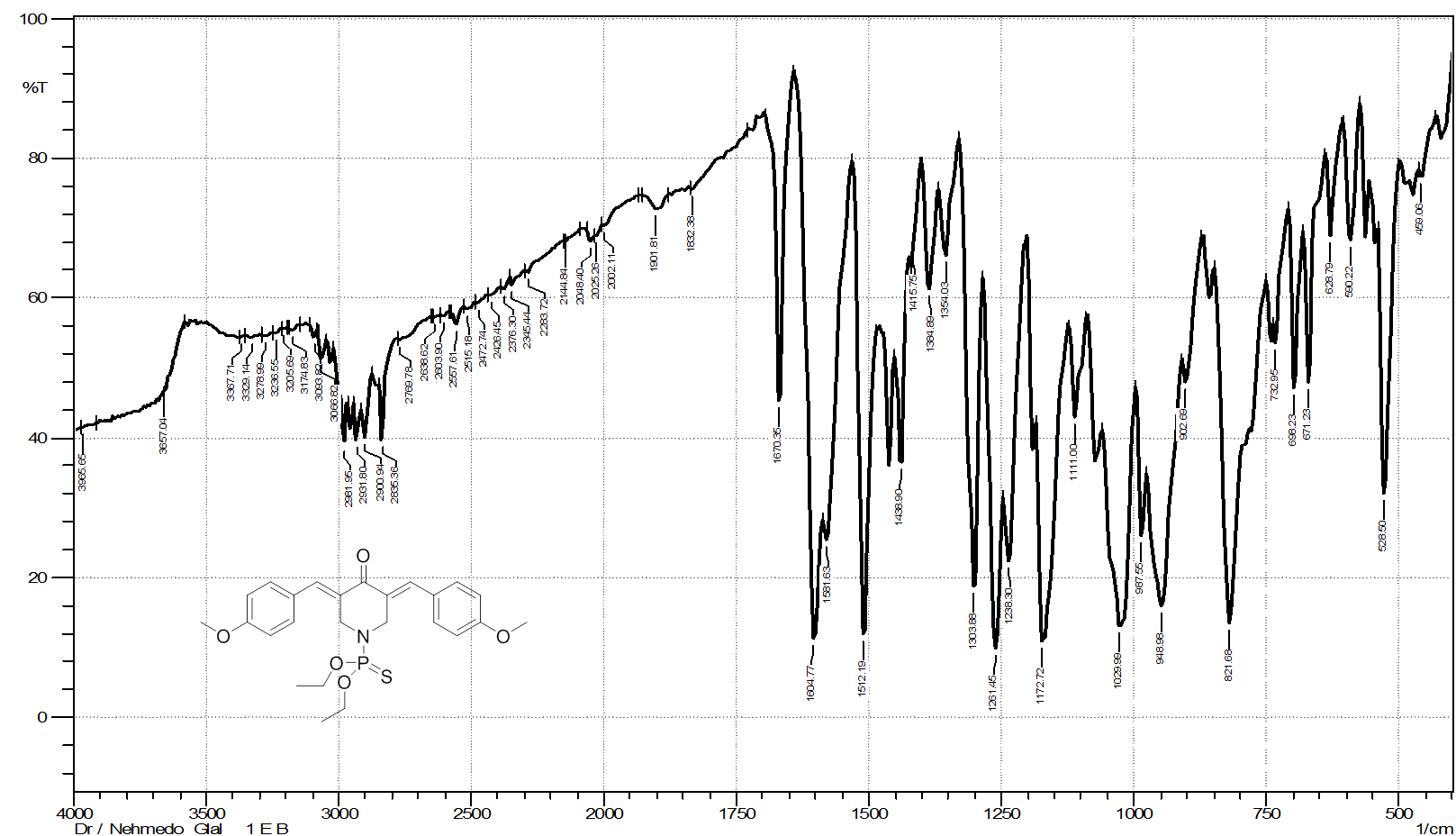


**Fig. S25.** IR spectrum of compound **20i** (KBr pellet).


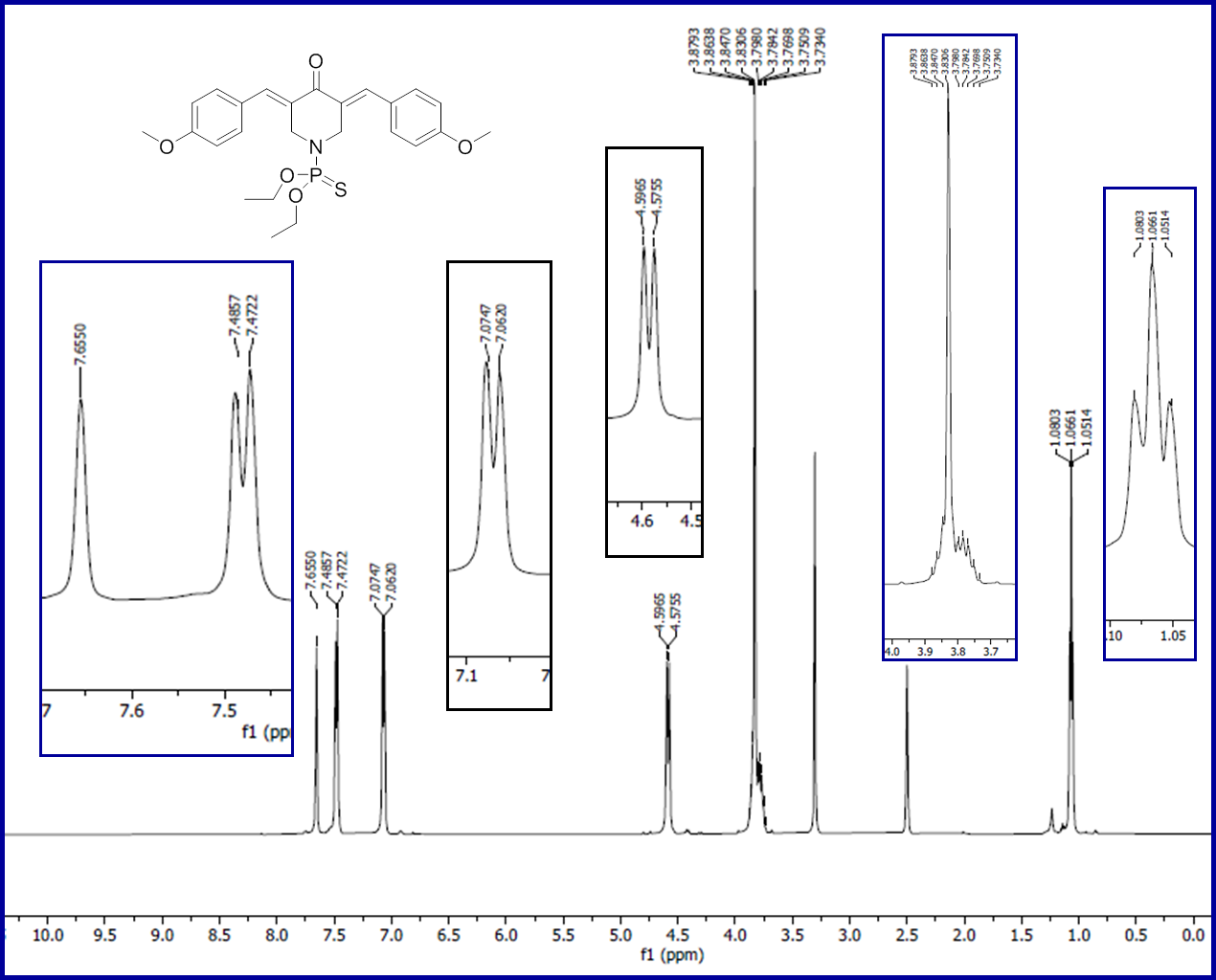


**Fig. S26.** ^1^H-NMR spectrum of compound **20i** in DMSO-*d6*.


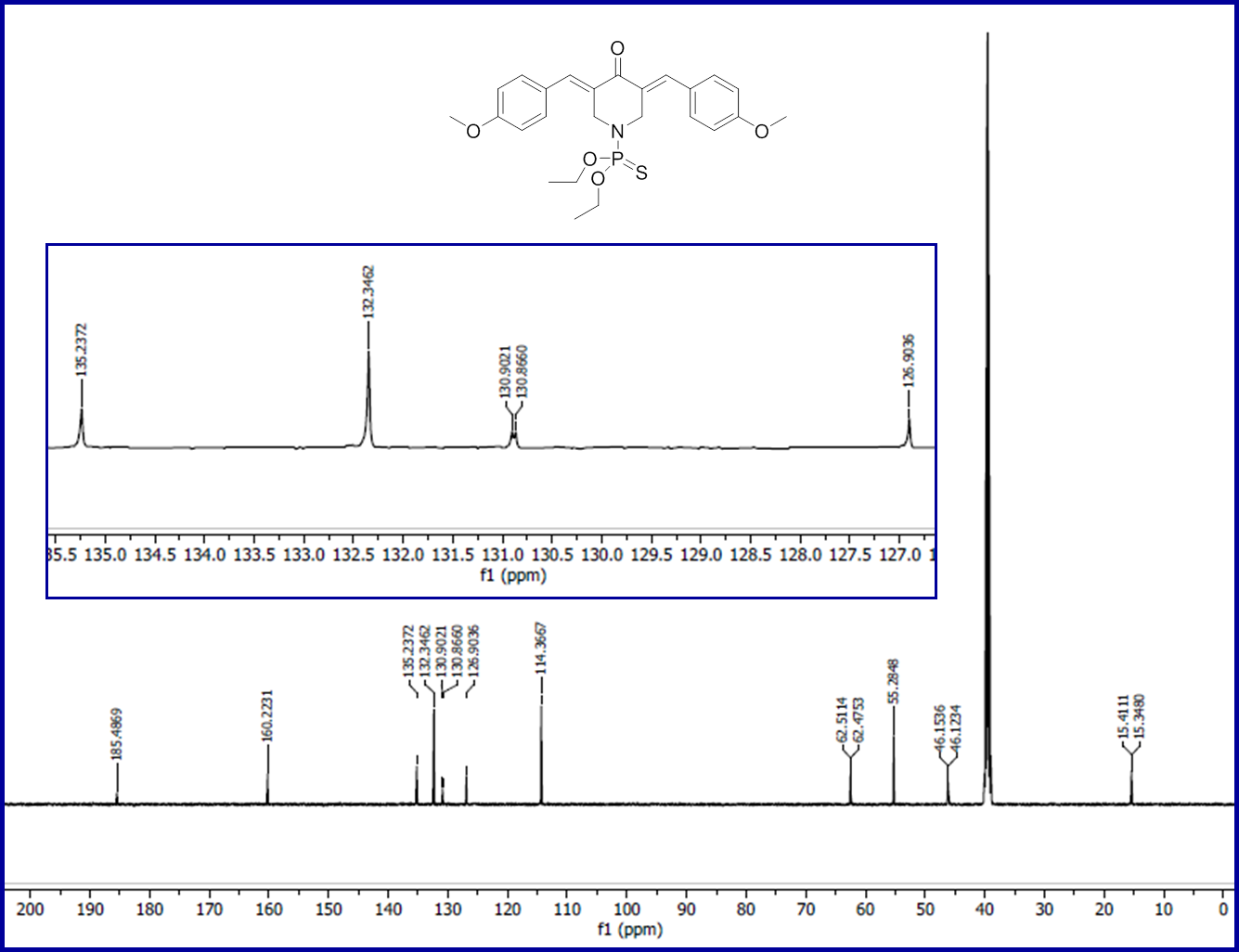


**Fig. S27.** ^13^C-NMR spectrum of compound **20i** in DMSO-*d6*.


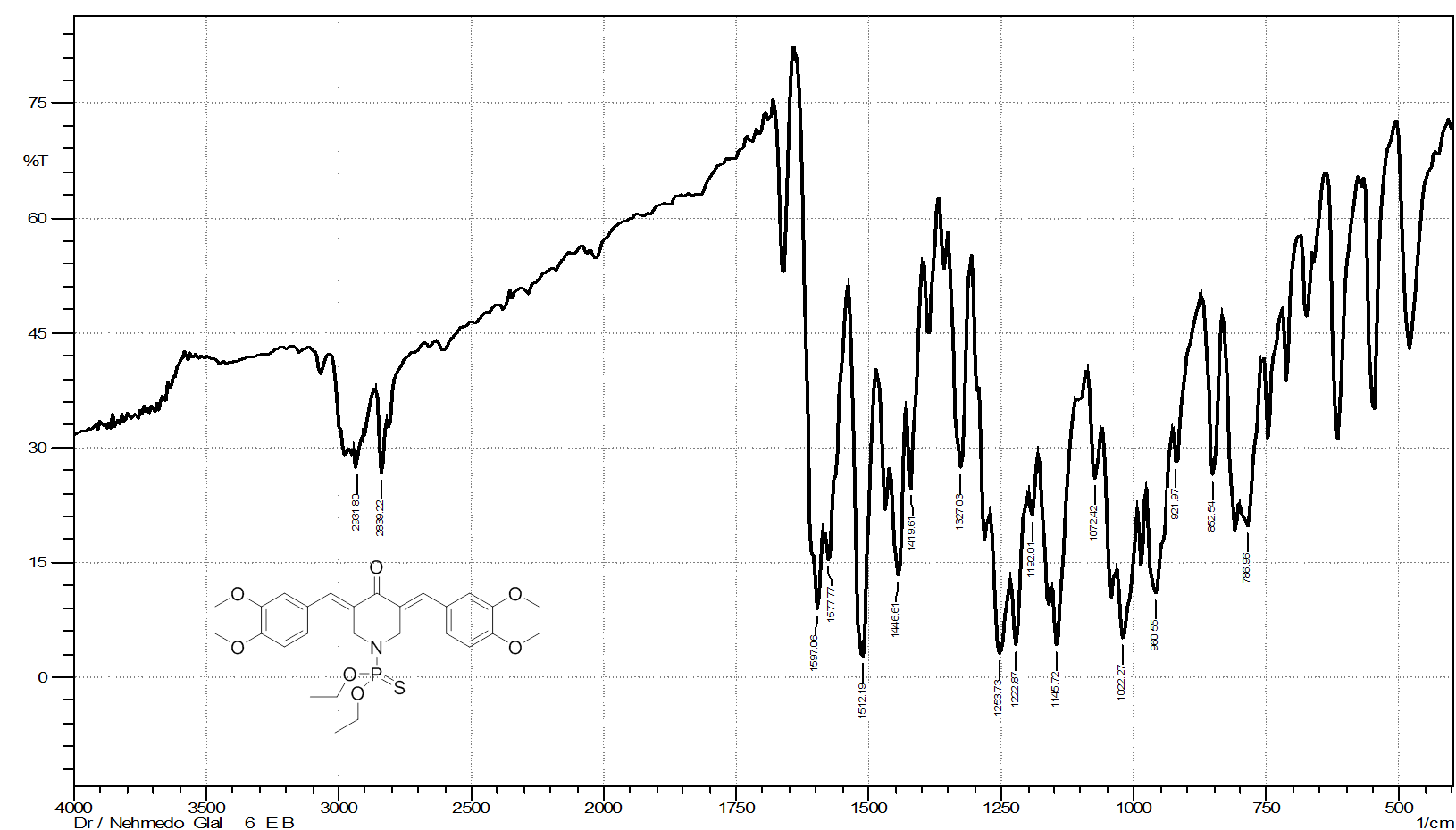


**Fig. S28.** IR spectrum of compound **20j** (KBr pellet).


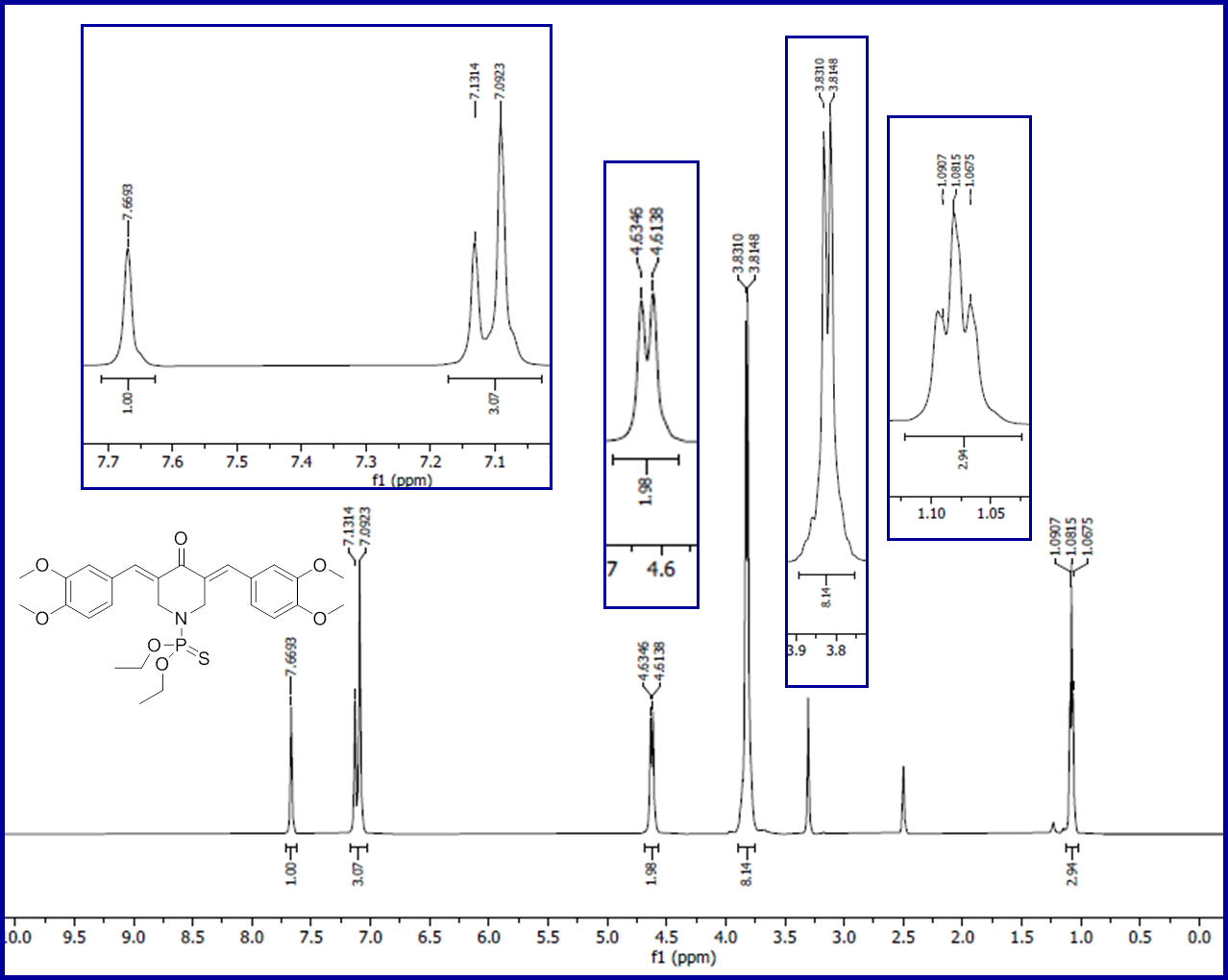


**Fig. S29.** ^1^H-NMR spectrum of compound **20j** in DMSO-*d6*.


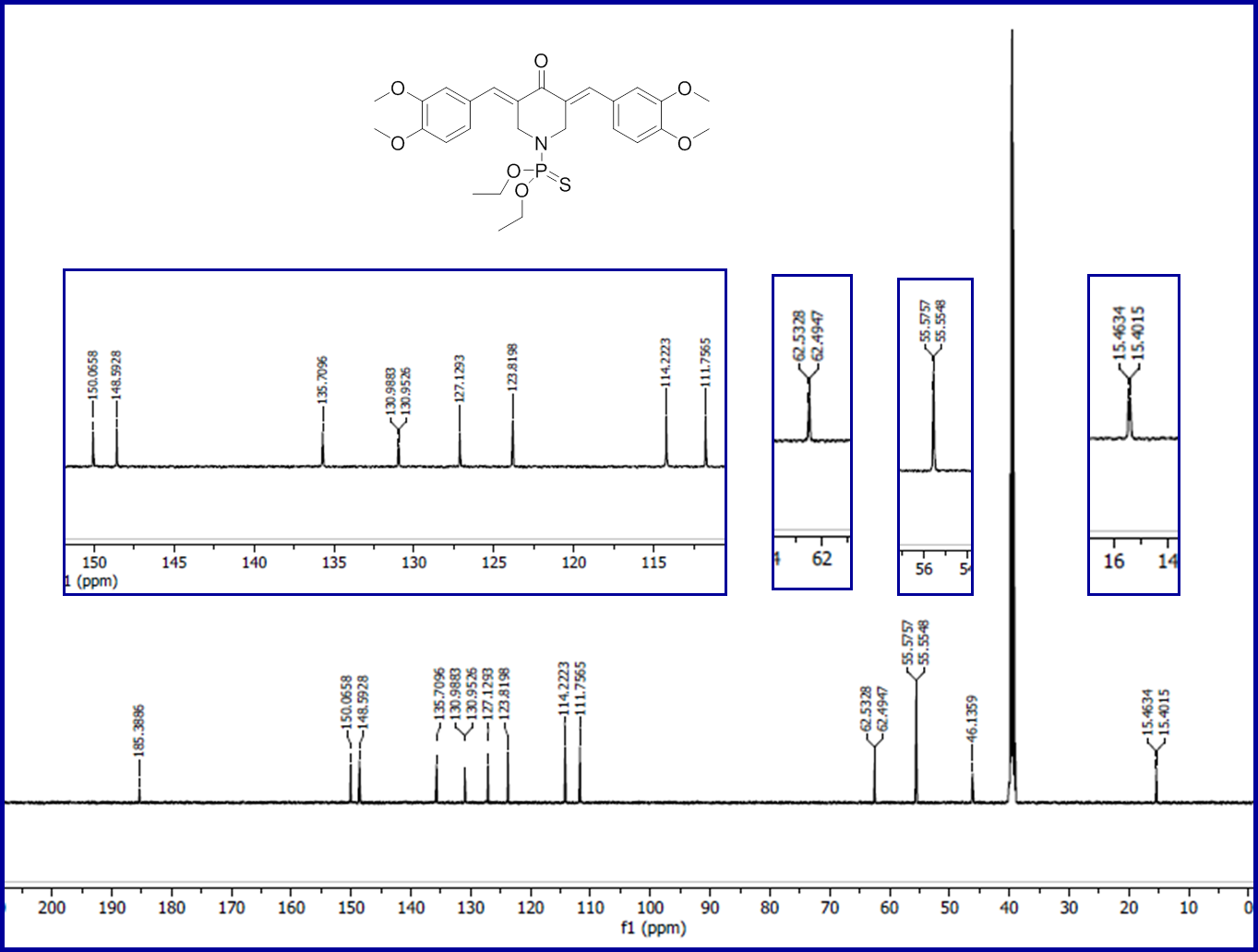


**Fig. S30.** ^13^C-NMR spectrum of compound **20j** in DMSO-*d6*.


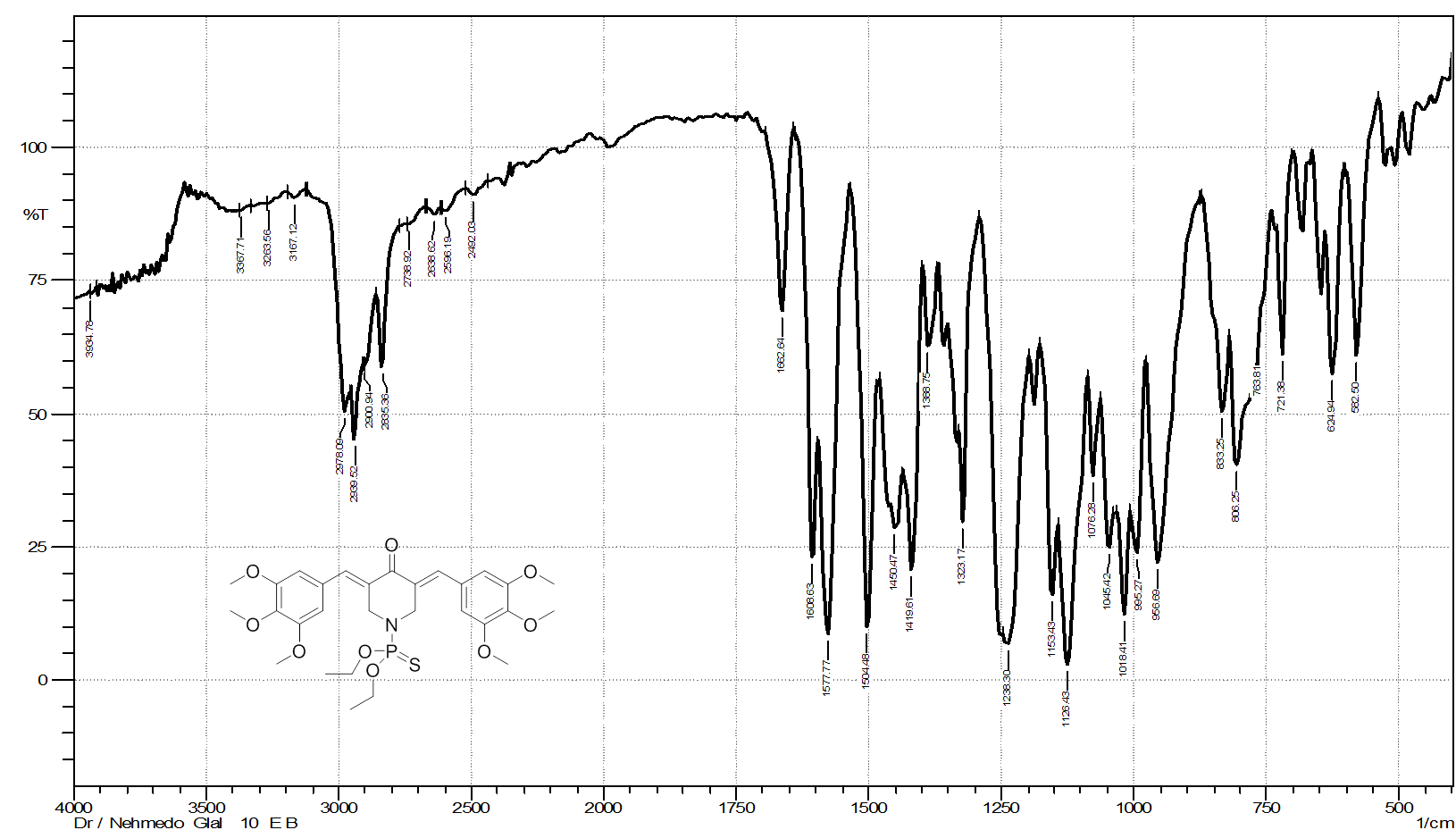


**Fig. S31.** IR spectrum of compound **20k** (KBr pellet).


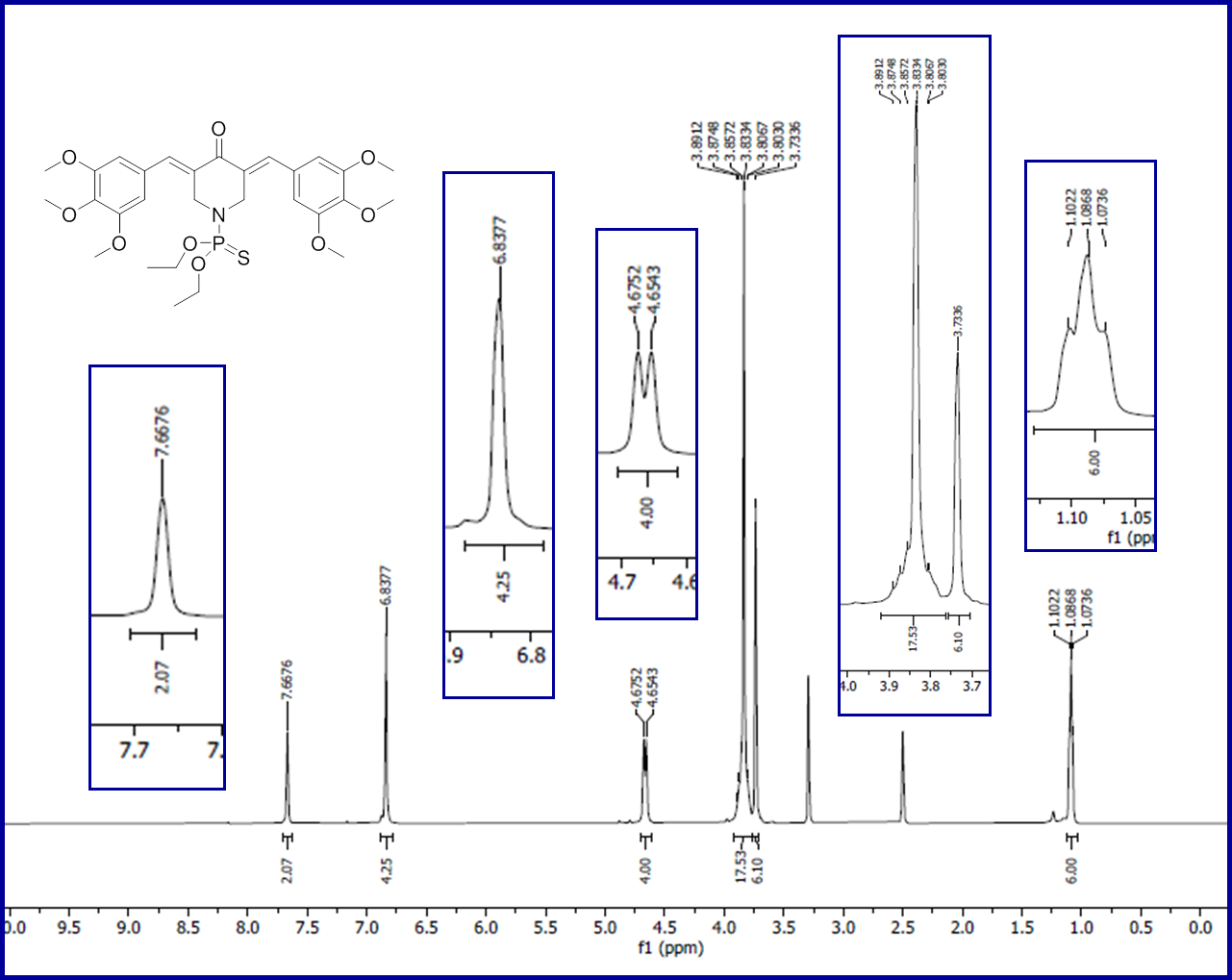


**Fig. S32.** ^1^H-NMR spectrum of compound **20k** in DMSO-*d6*.


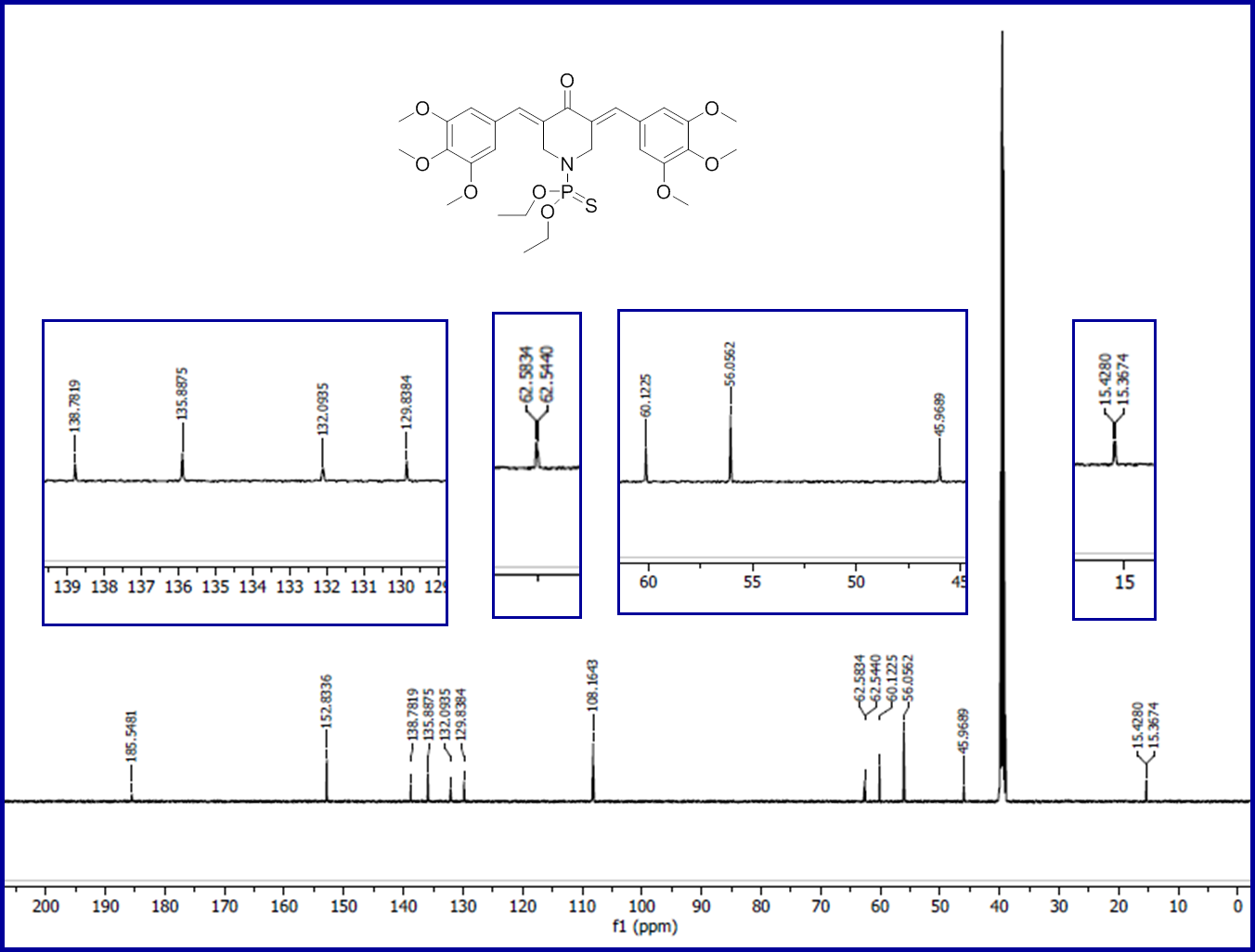


**Fig. S33.** ^13^C-NMR spectrum of compound **20k** in DMSO-*d6*.


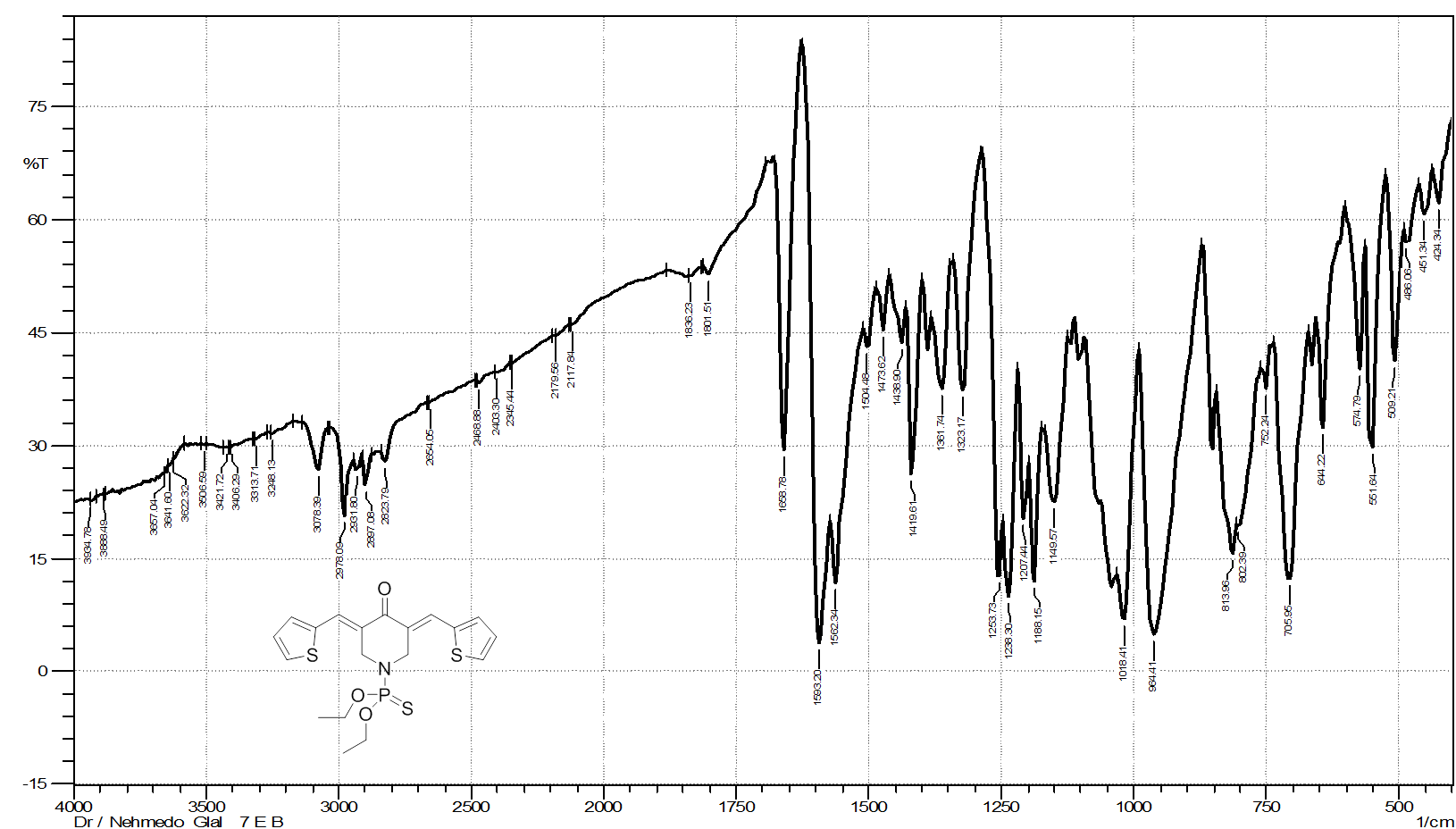


**Fig. S34.** IR spectrum of compound **20l** (KBr pellet).


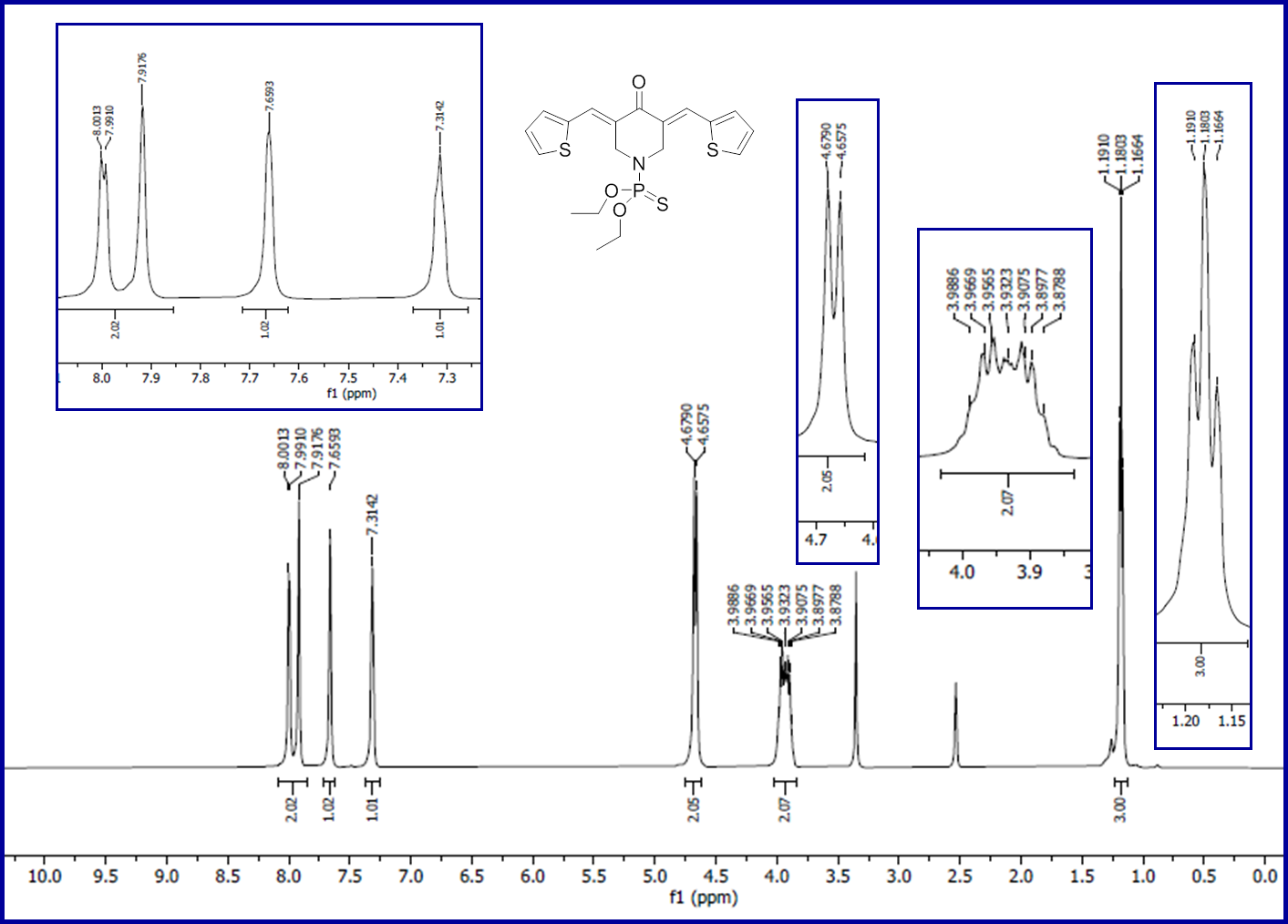


**Fig. S35.** ^1^H-NMR spectrum of compound **20l** in DMSO-*d6*.


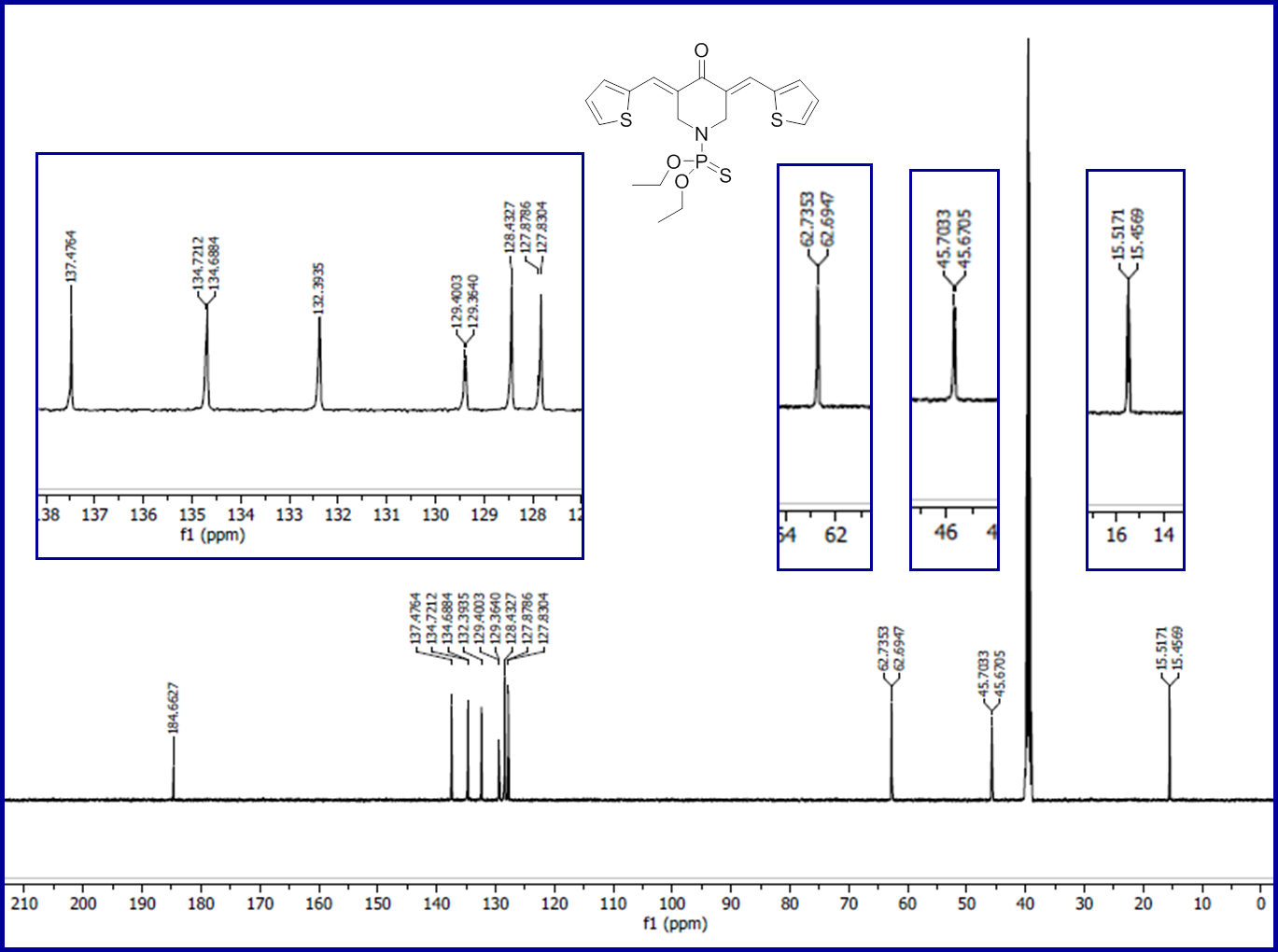


**Fig. S36.** ^13^C-NMR spectrum of compound **20l** in DMSO-*d6*.


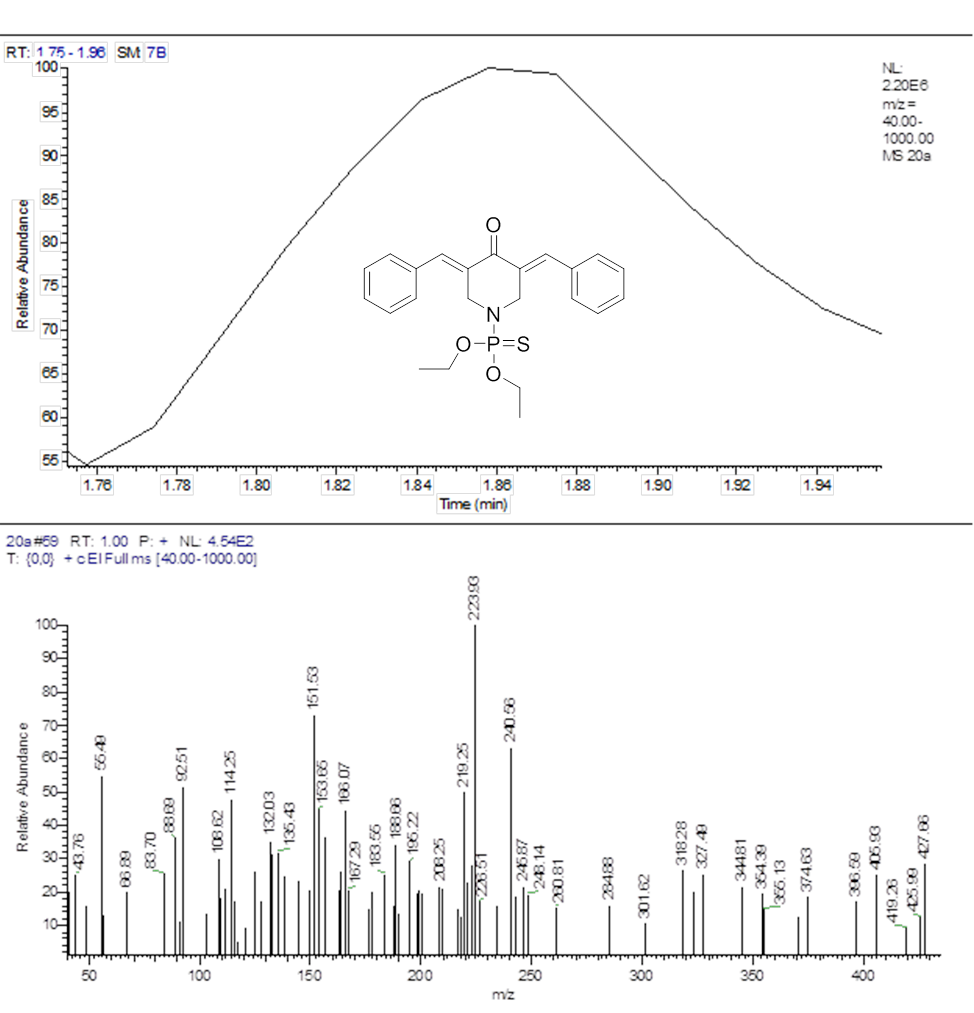


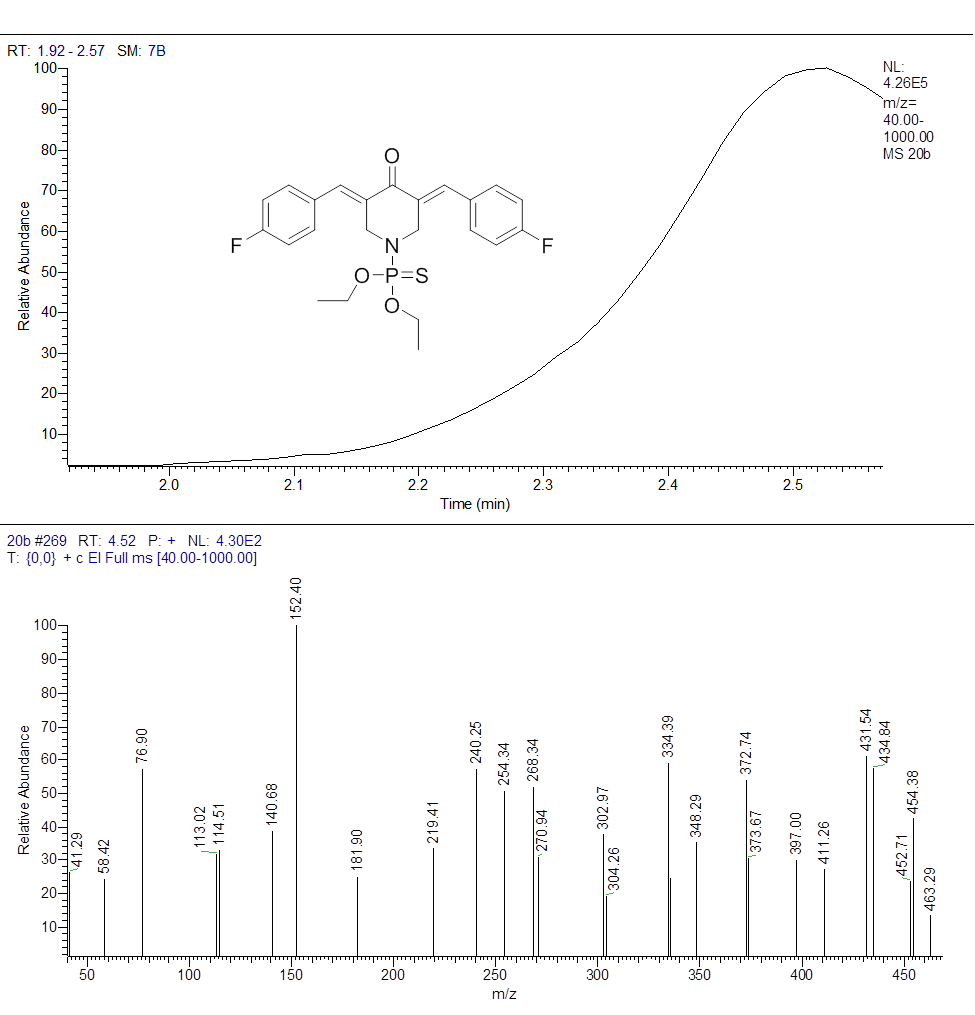


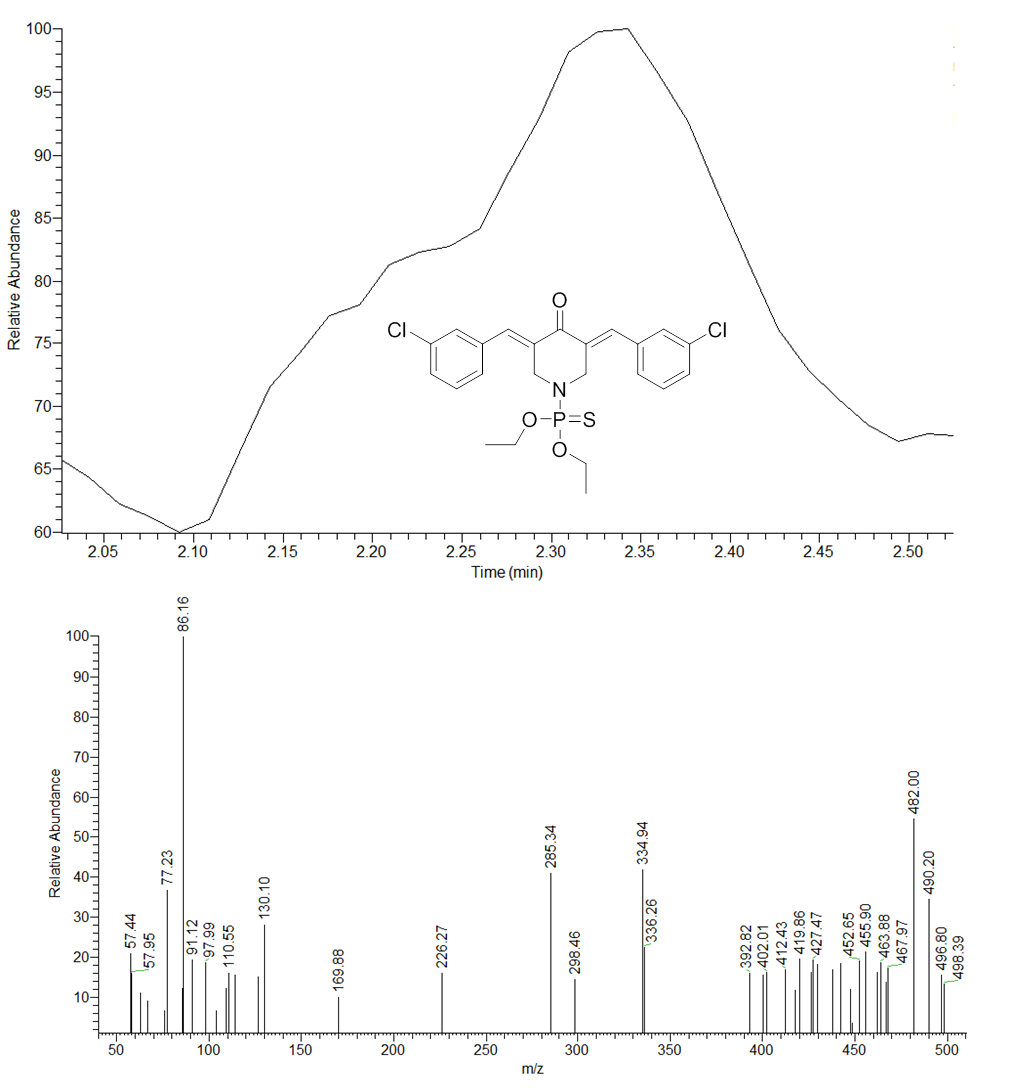


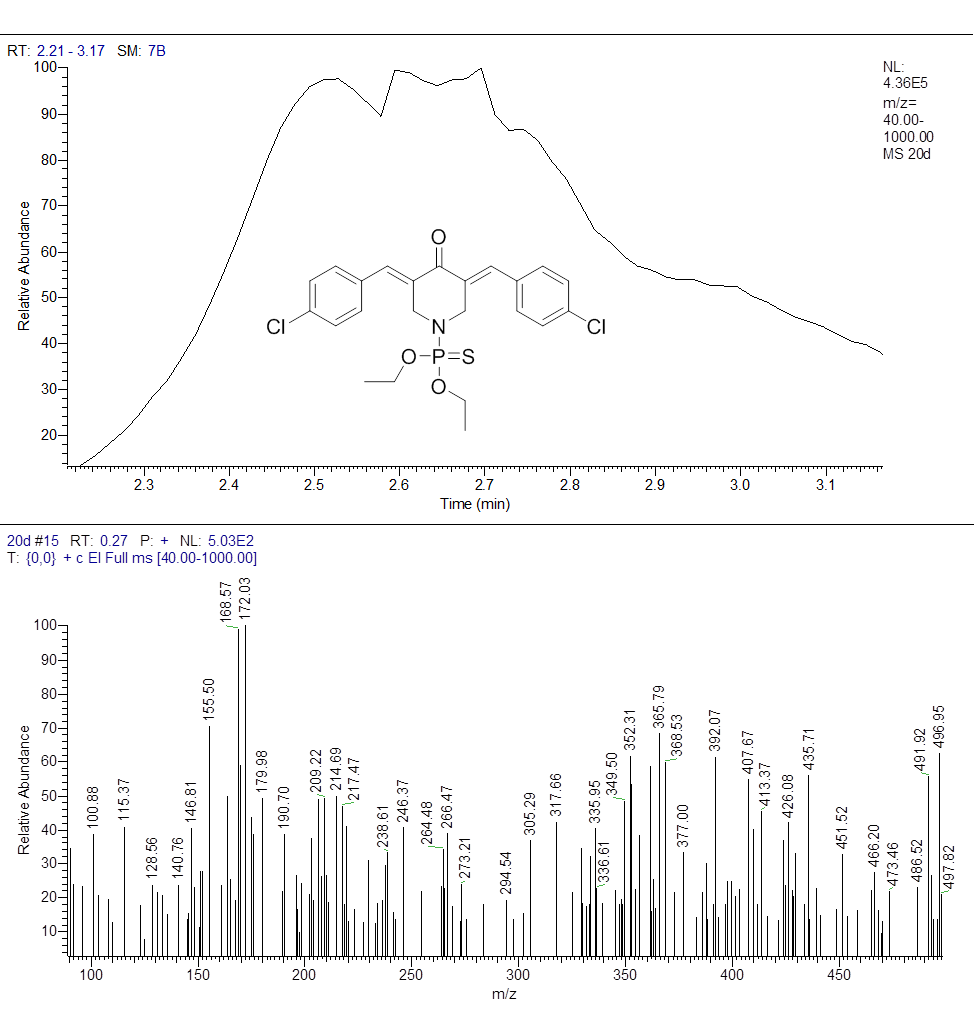


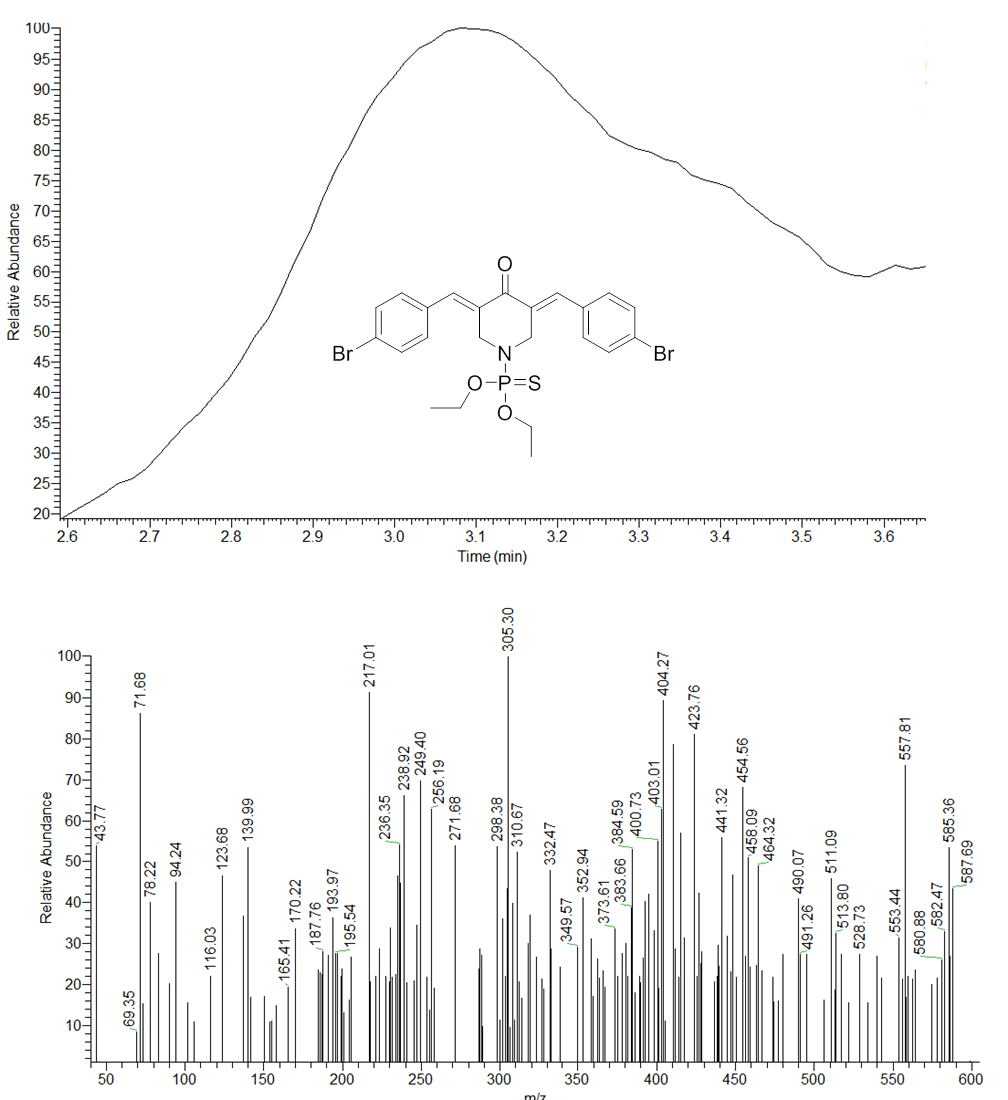


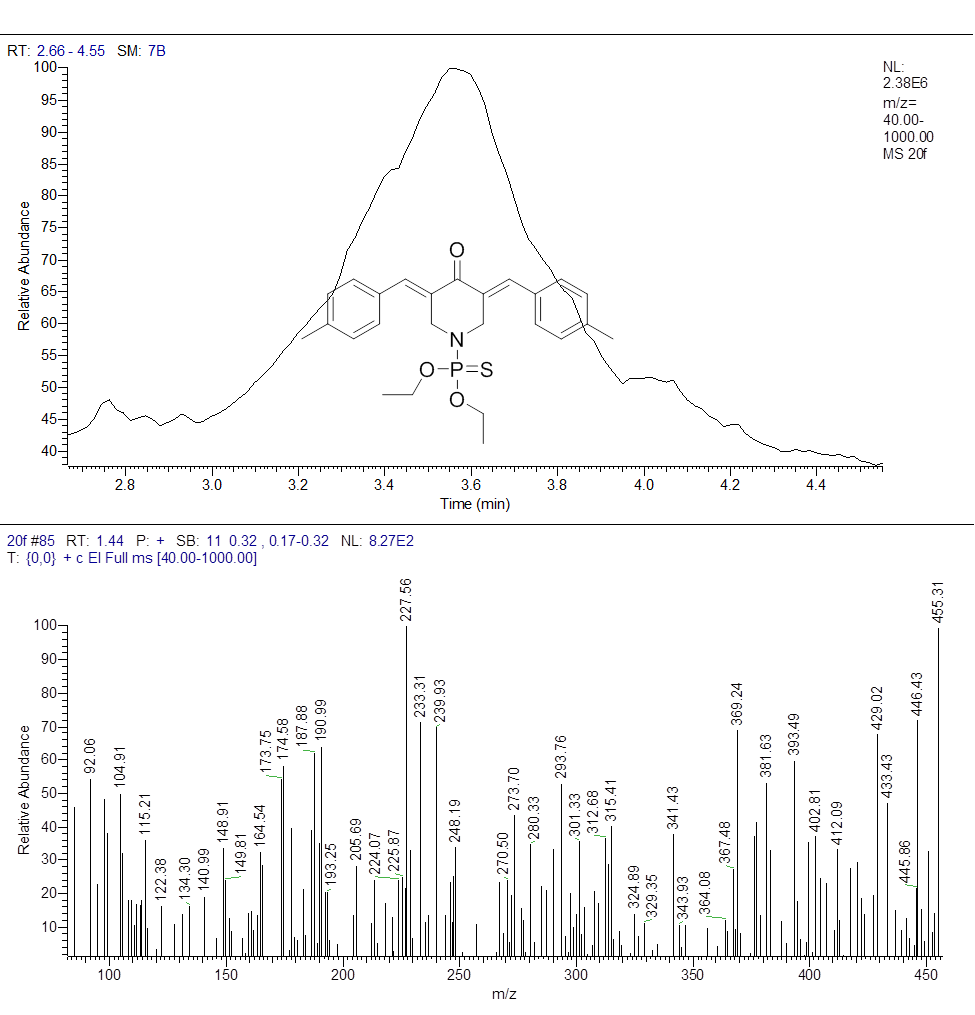


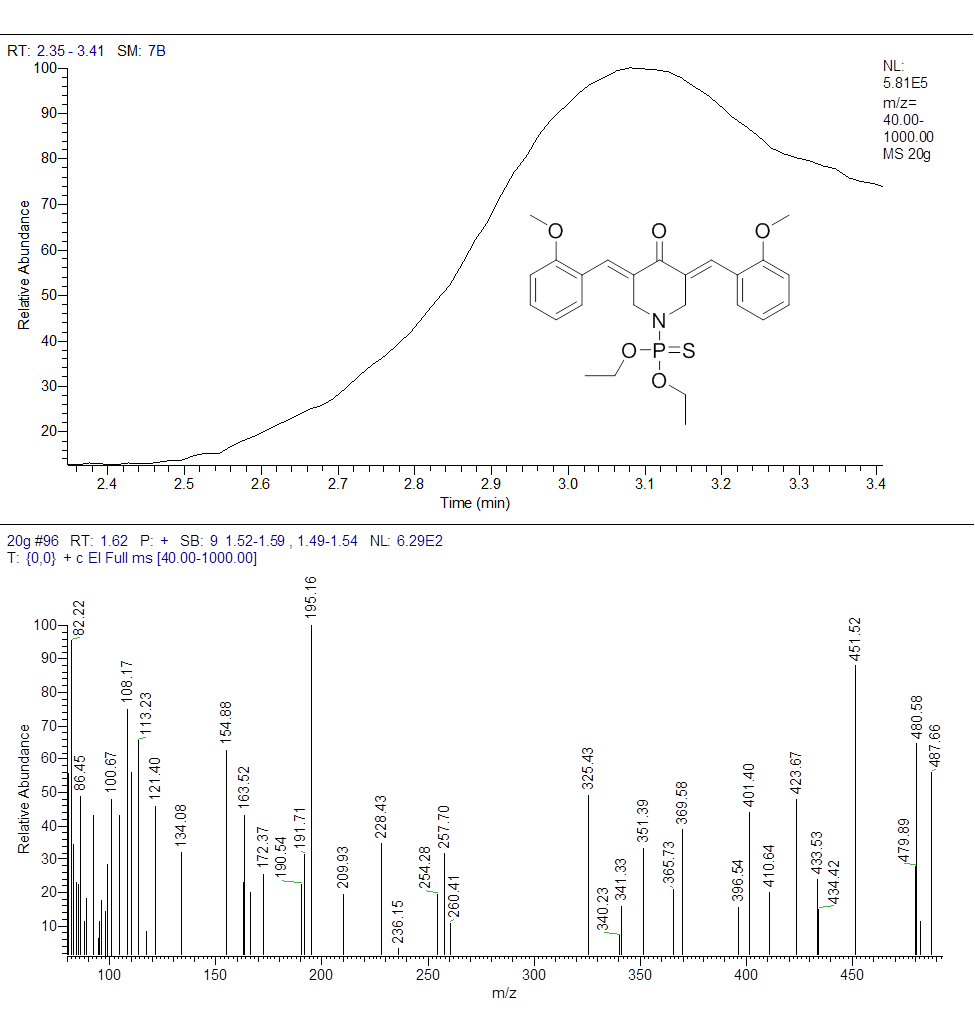


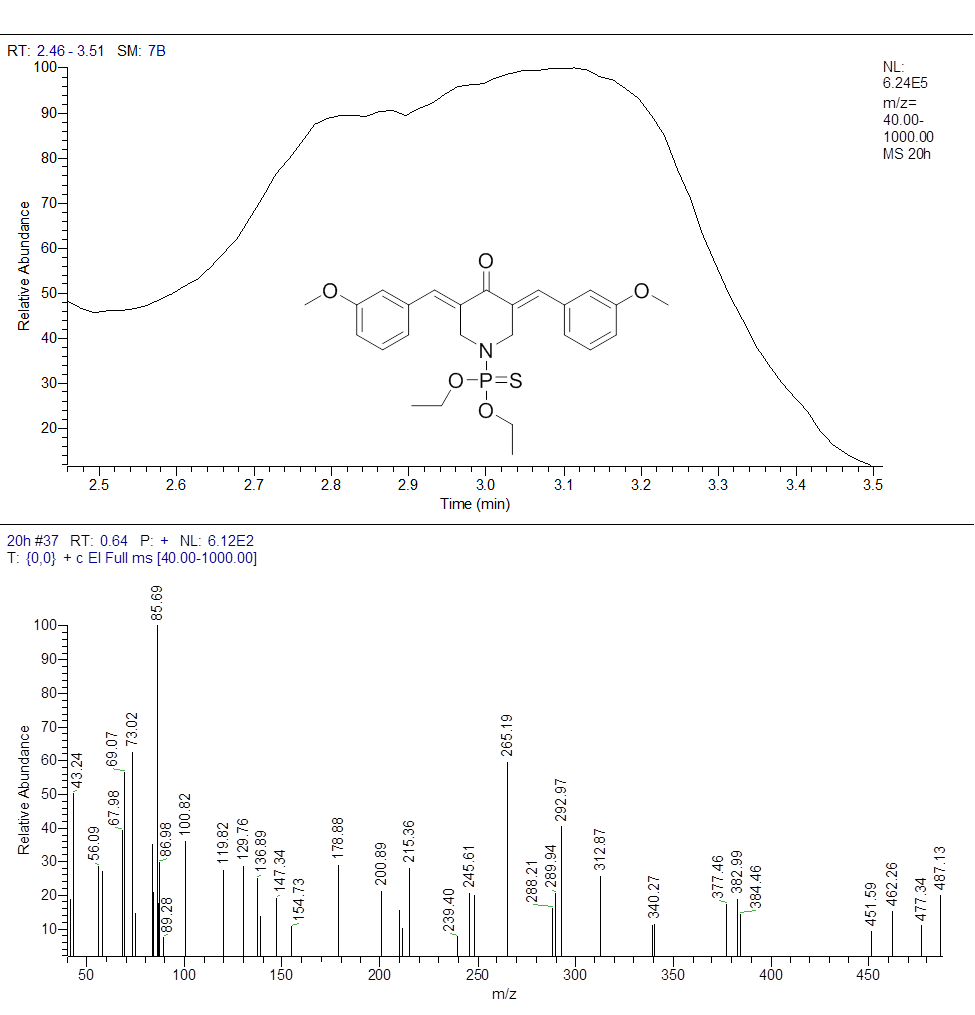


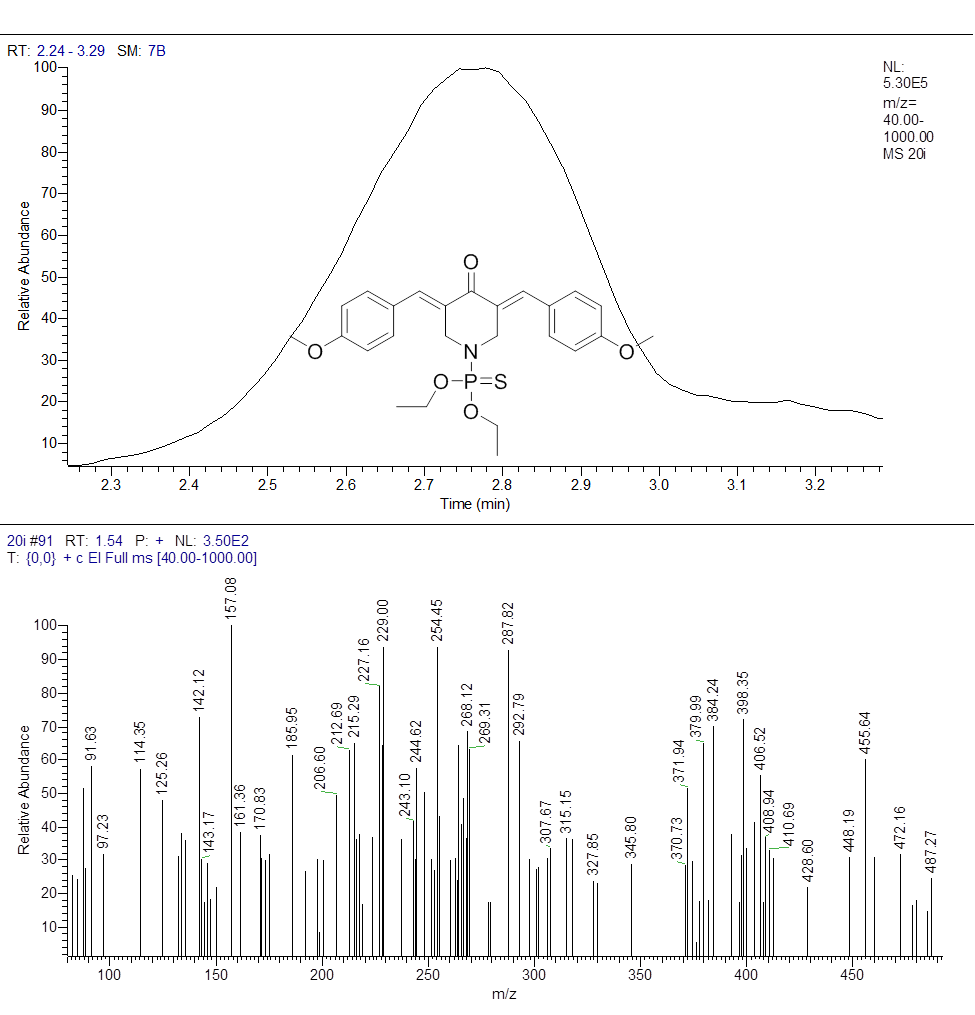


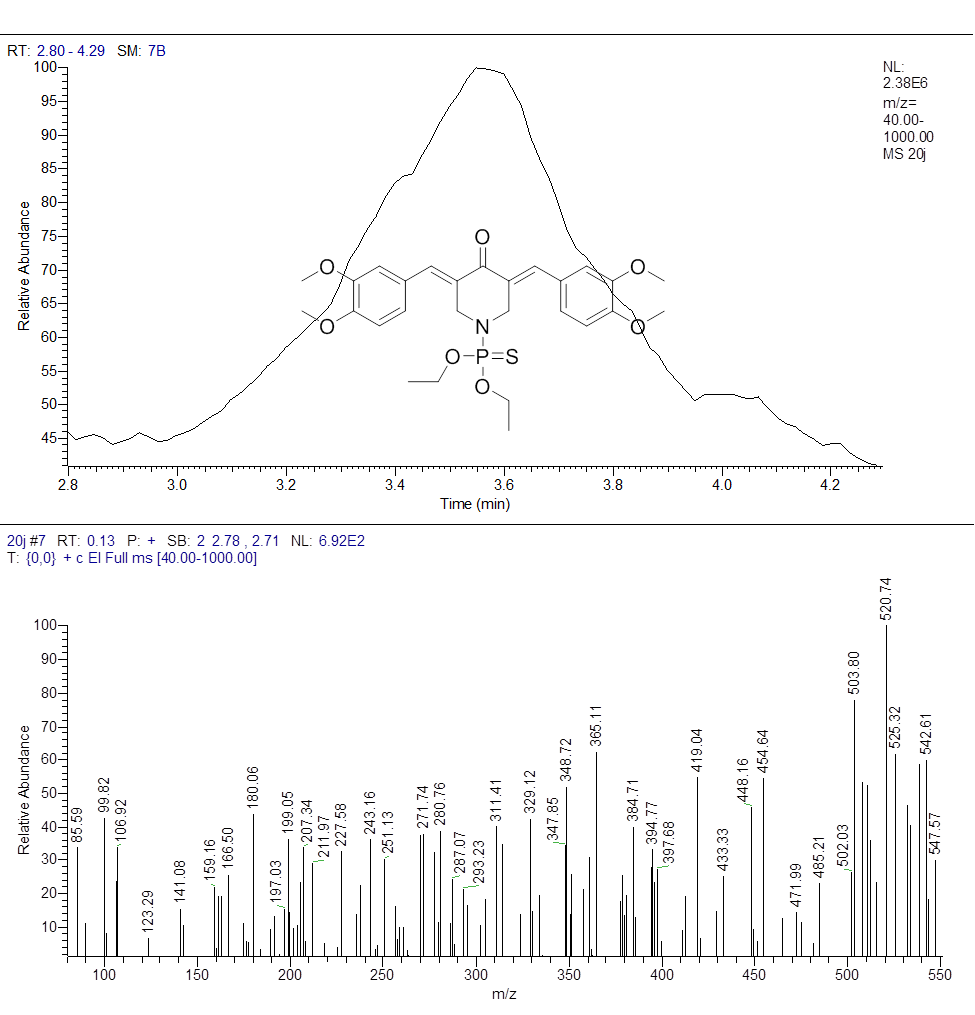


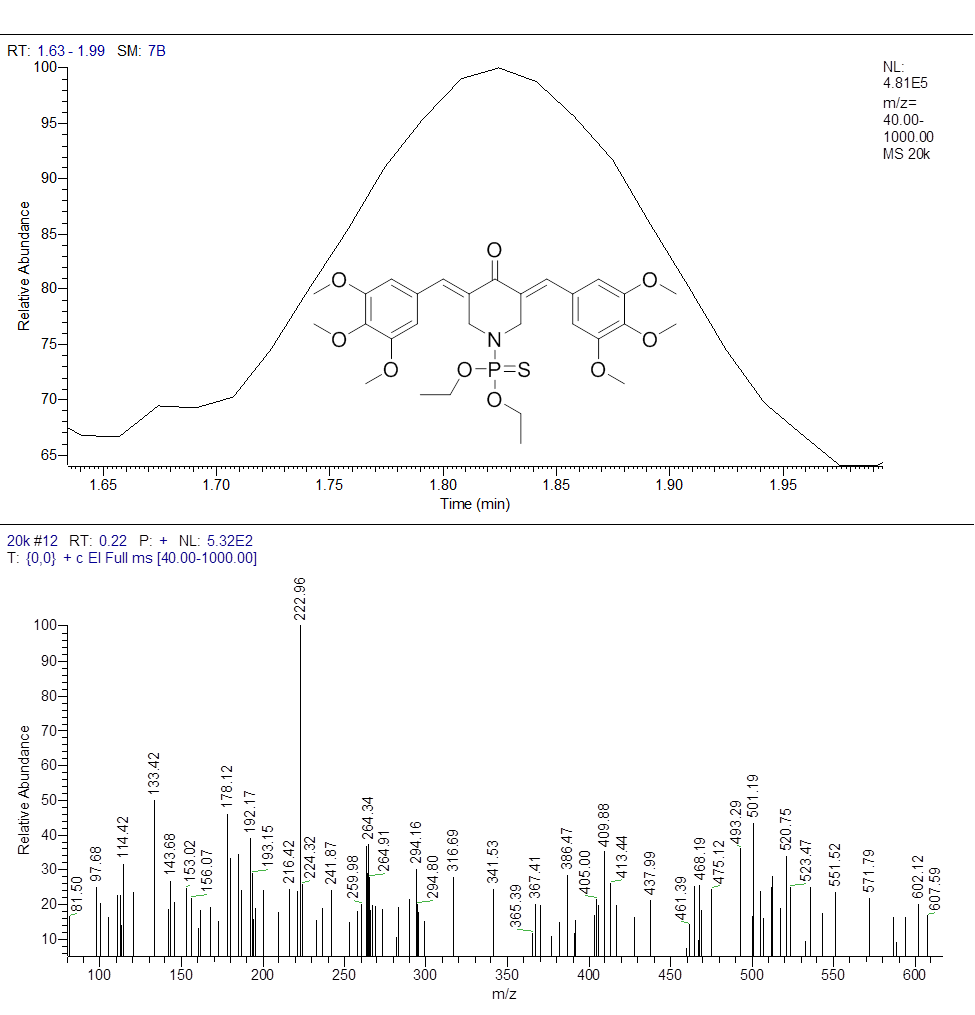


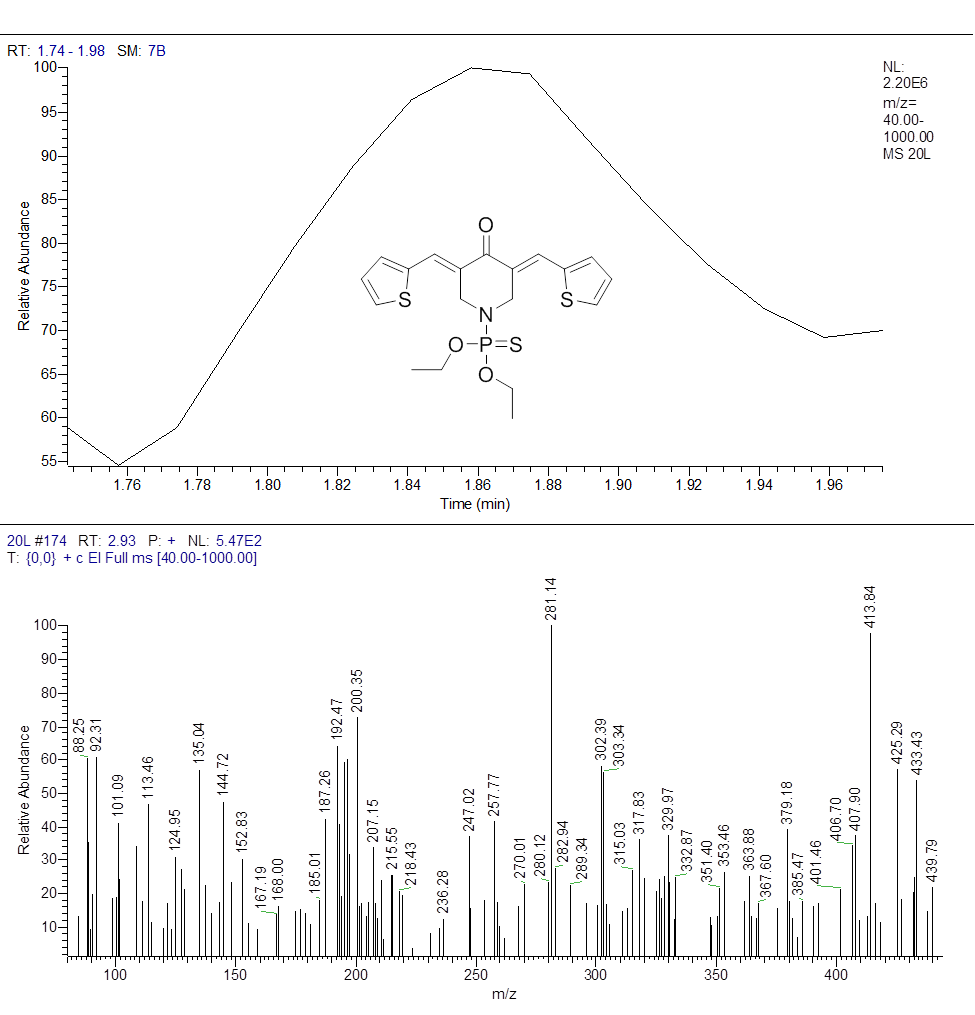


**Fig. S37.** Mass spectra (EI-MS) of the synthesized compounds **20a‒l**.


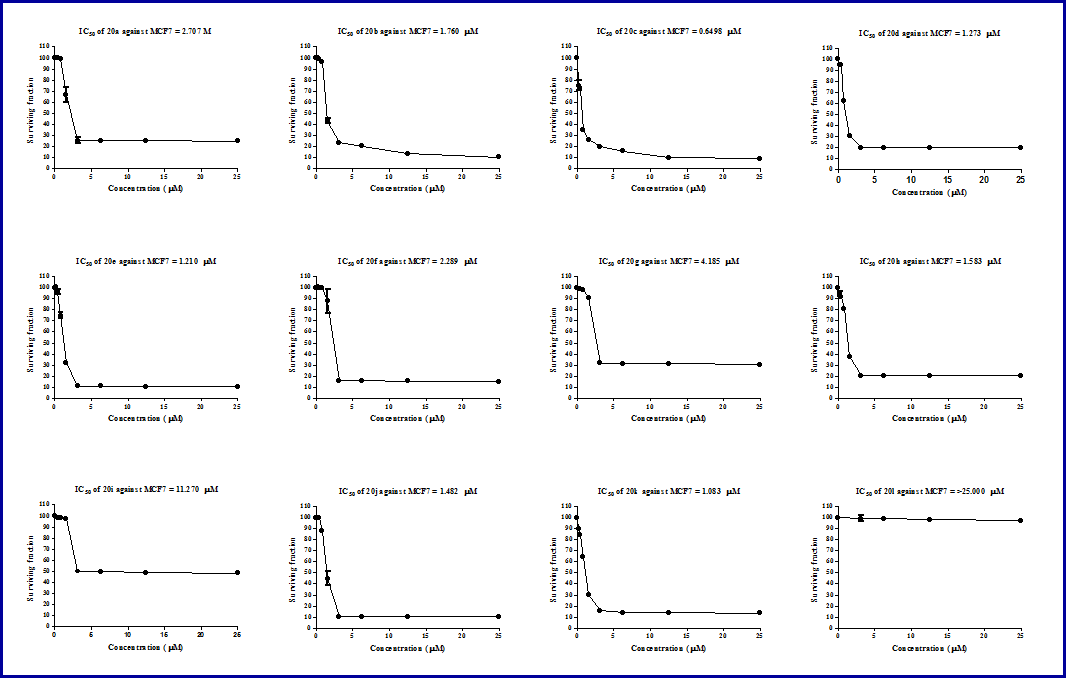


**Fig. S38.** Dose response curves of **20a‒l** against MCF7 (breast) cancer cell line.


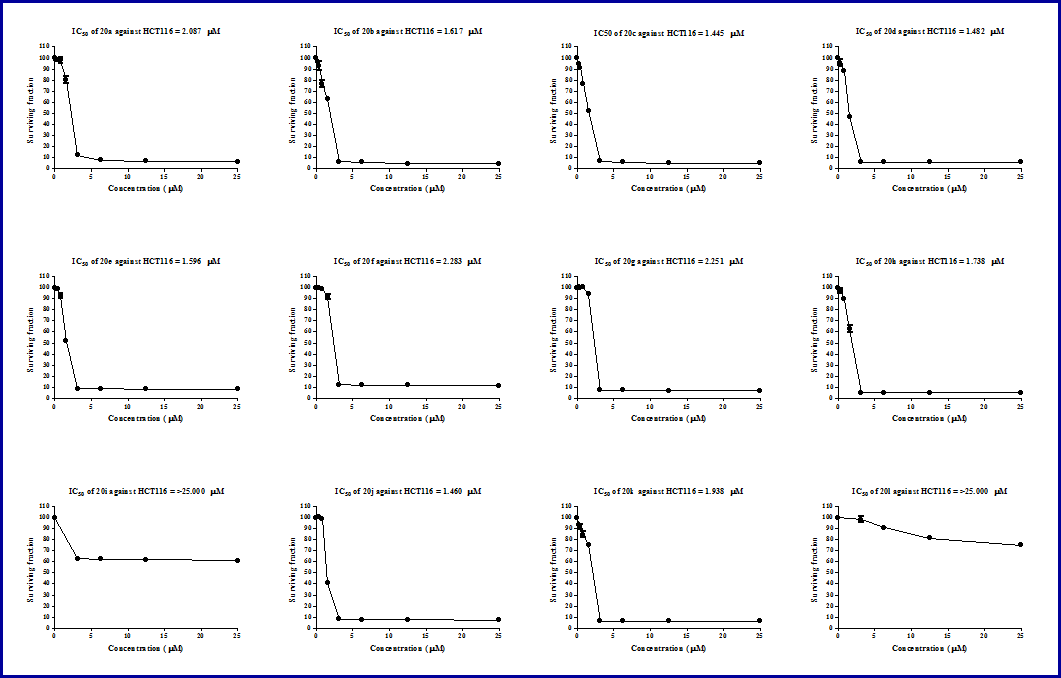


**Fig. S39.** Dose response curves of **20a‒l** against HCT116 (colon) cancer cell line.


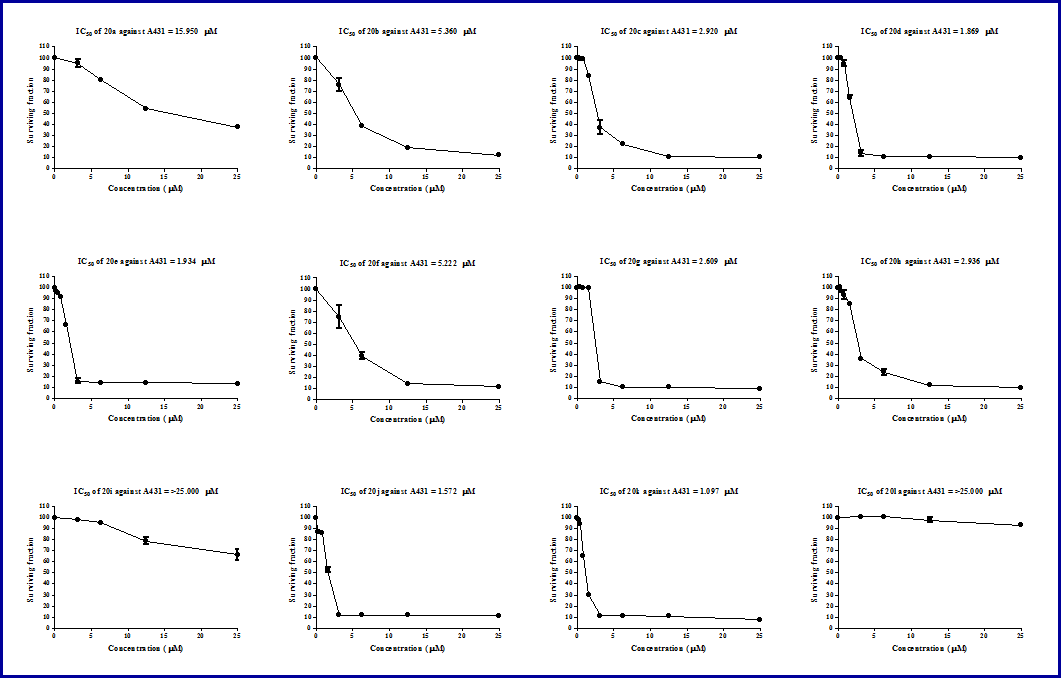


**Fig. S40.** Dose response curves of **20a‒l** against A431 (skin/squamous) cancer cell line.


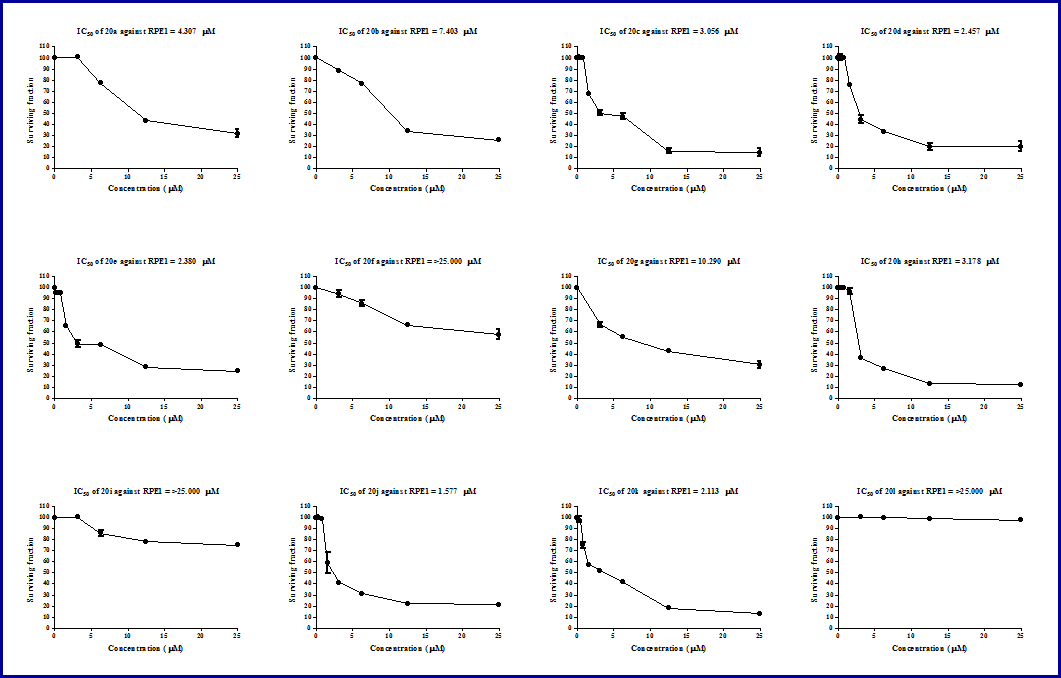


**Fig. S41.** Dose response curves of **20a‒l** against RPE1 (normal/non-cancer) cell line.


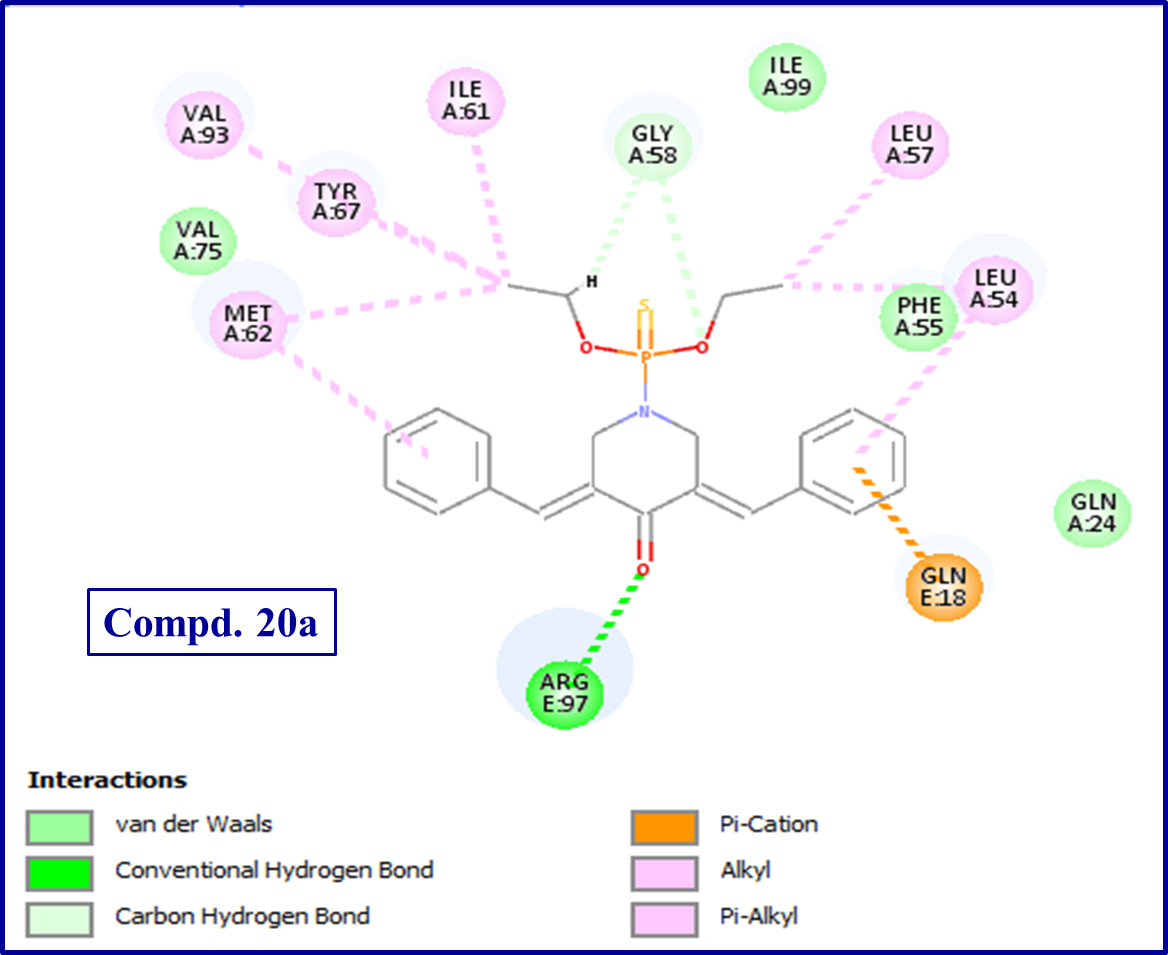


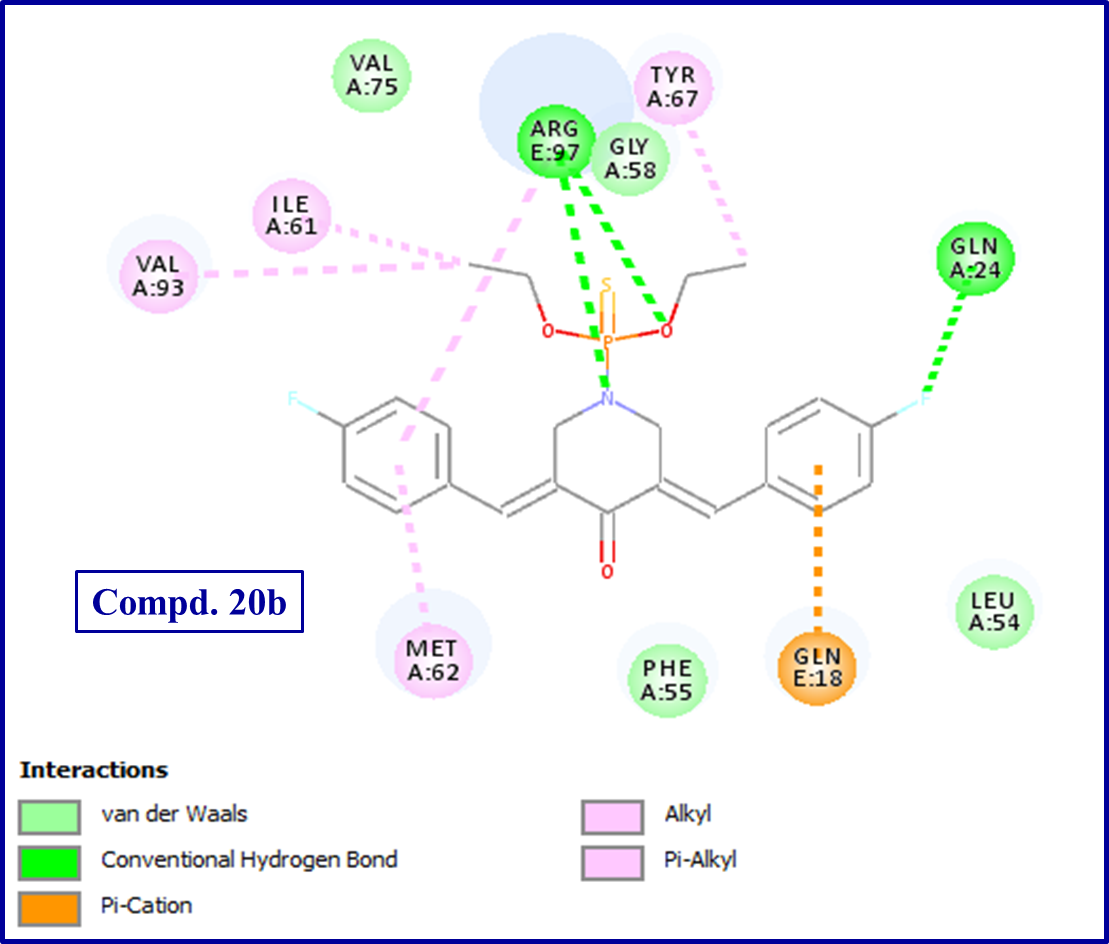


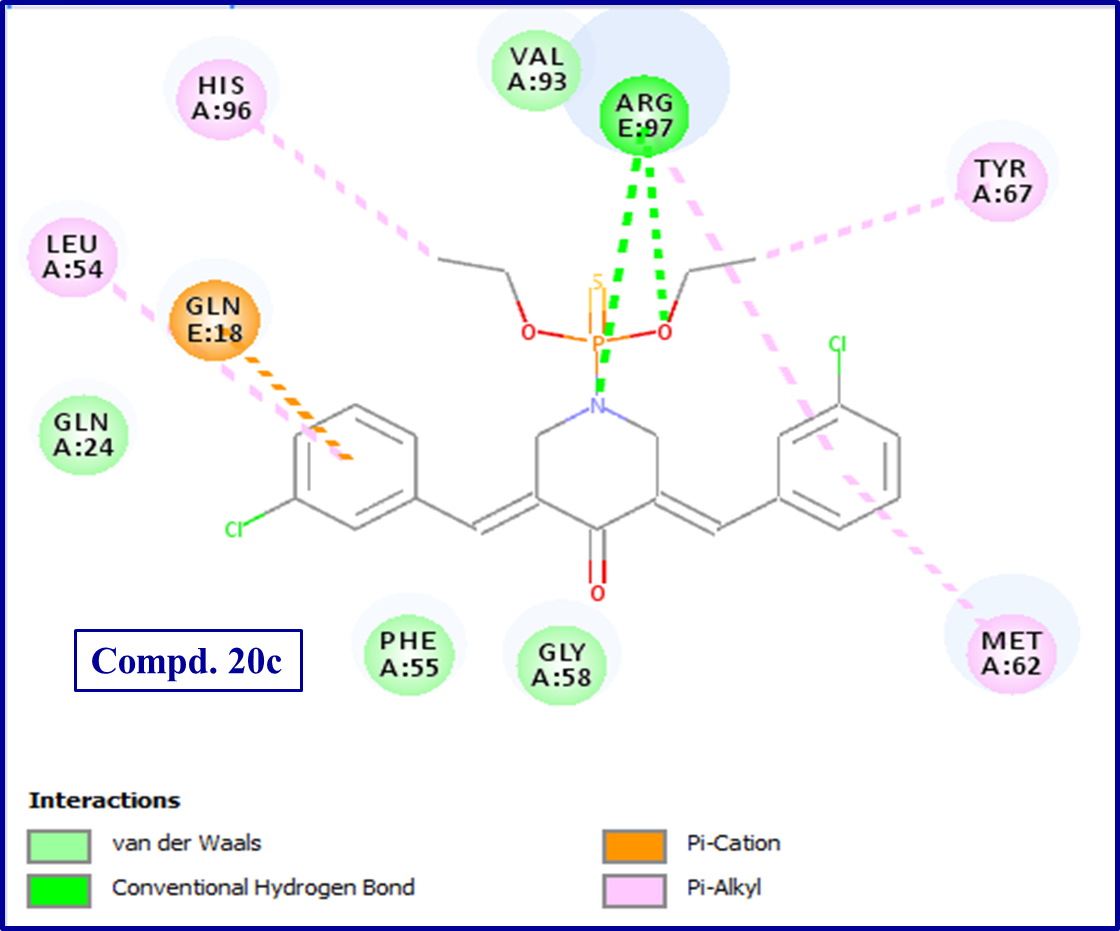


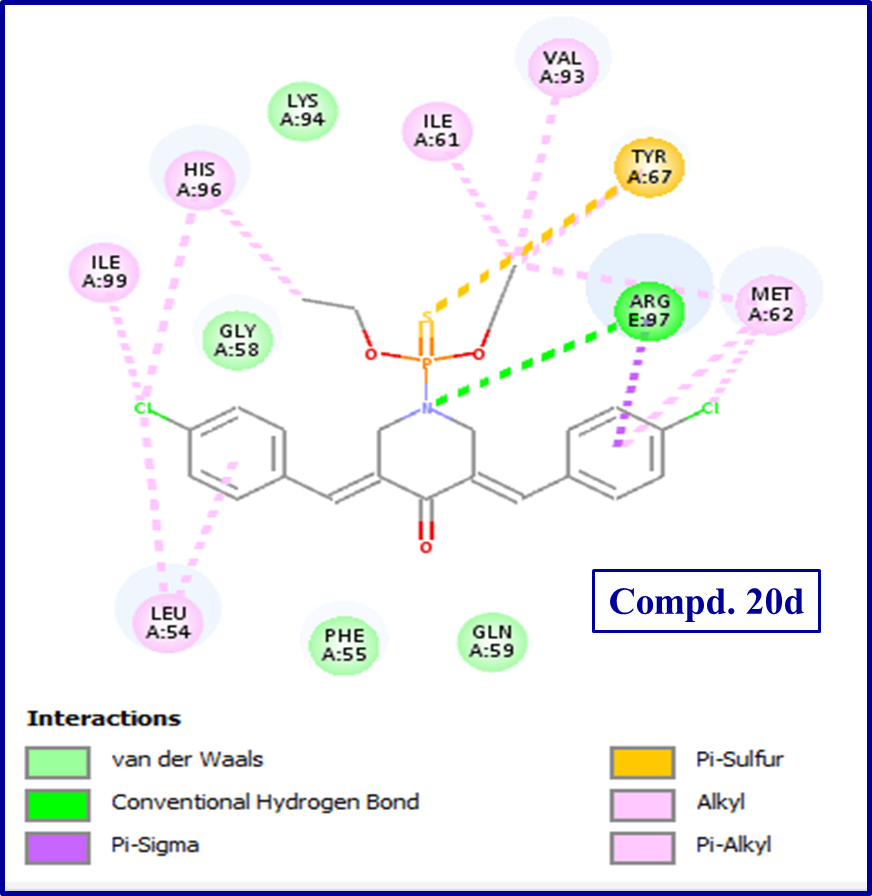


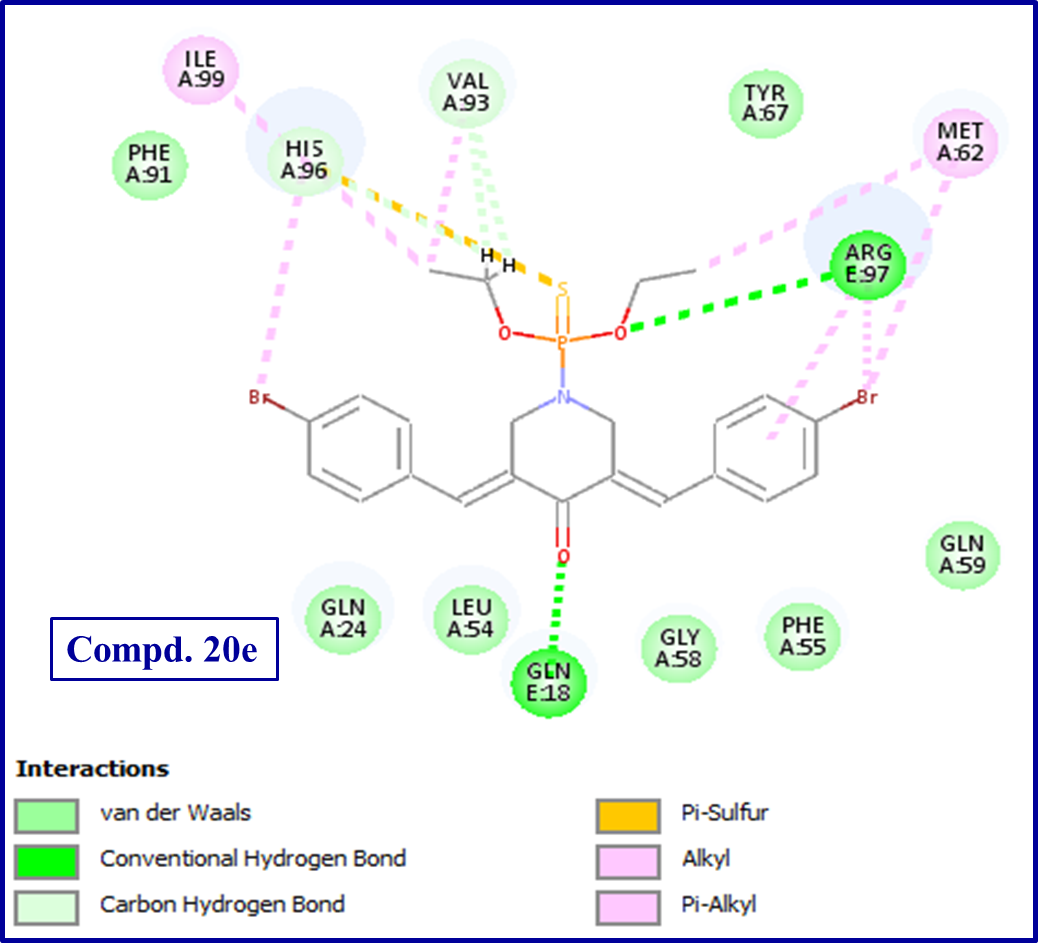


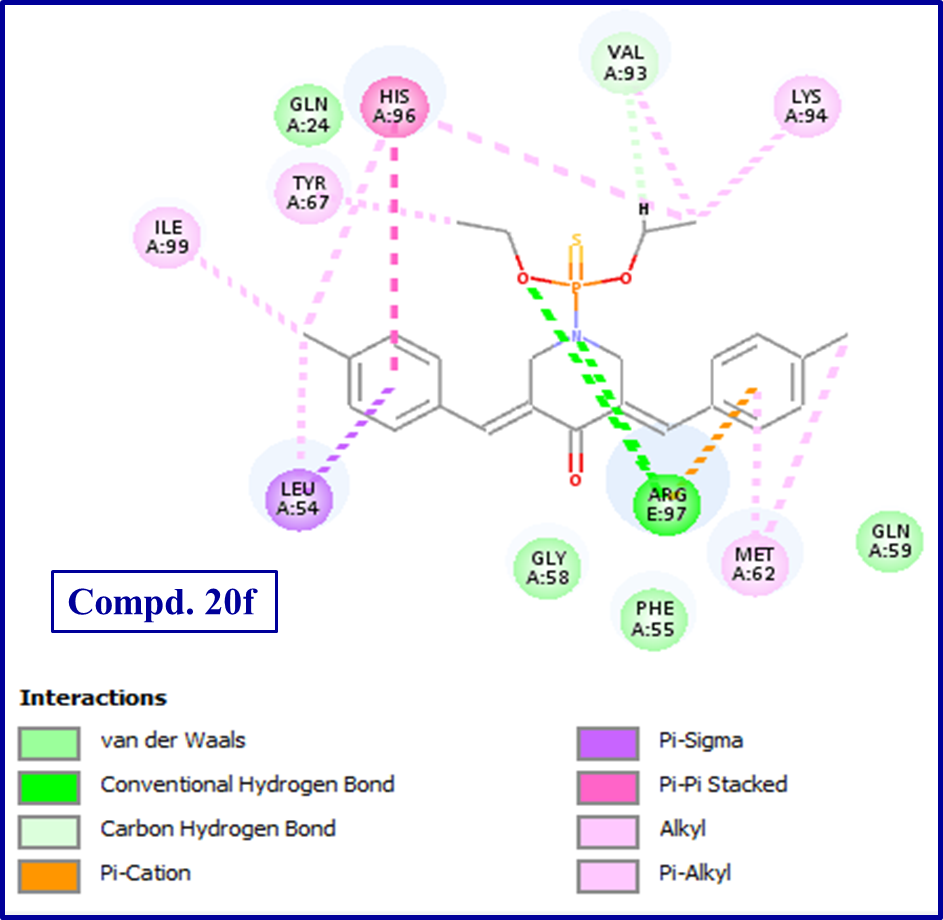


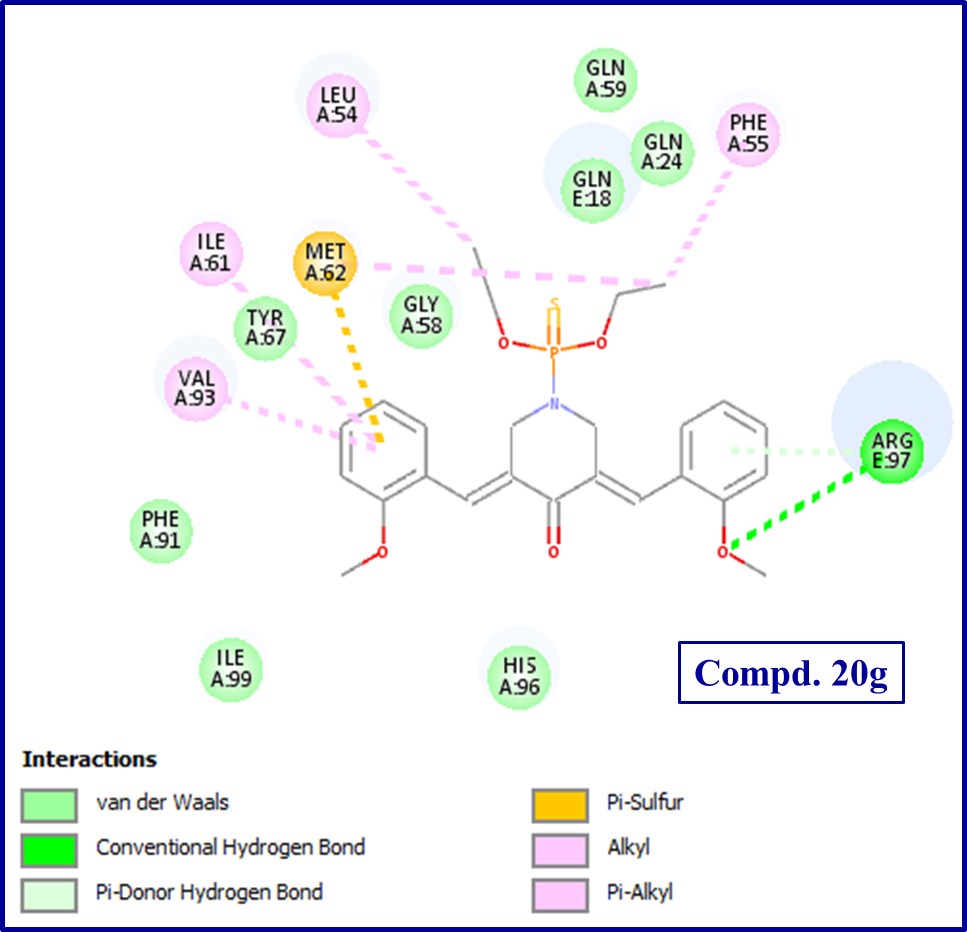


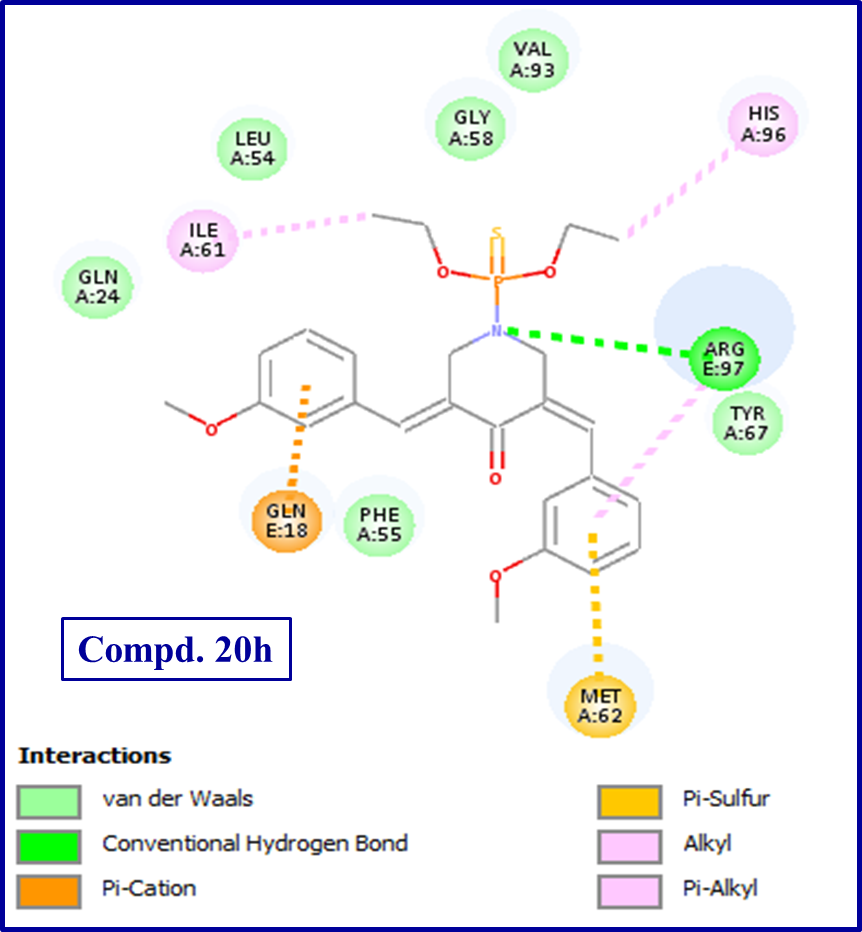


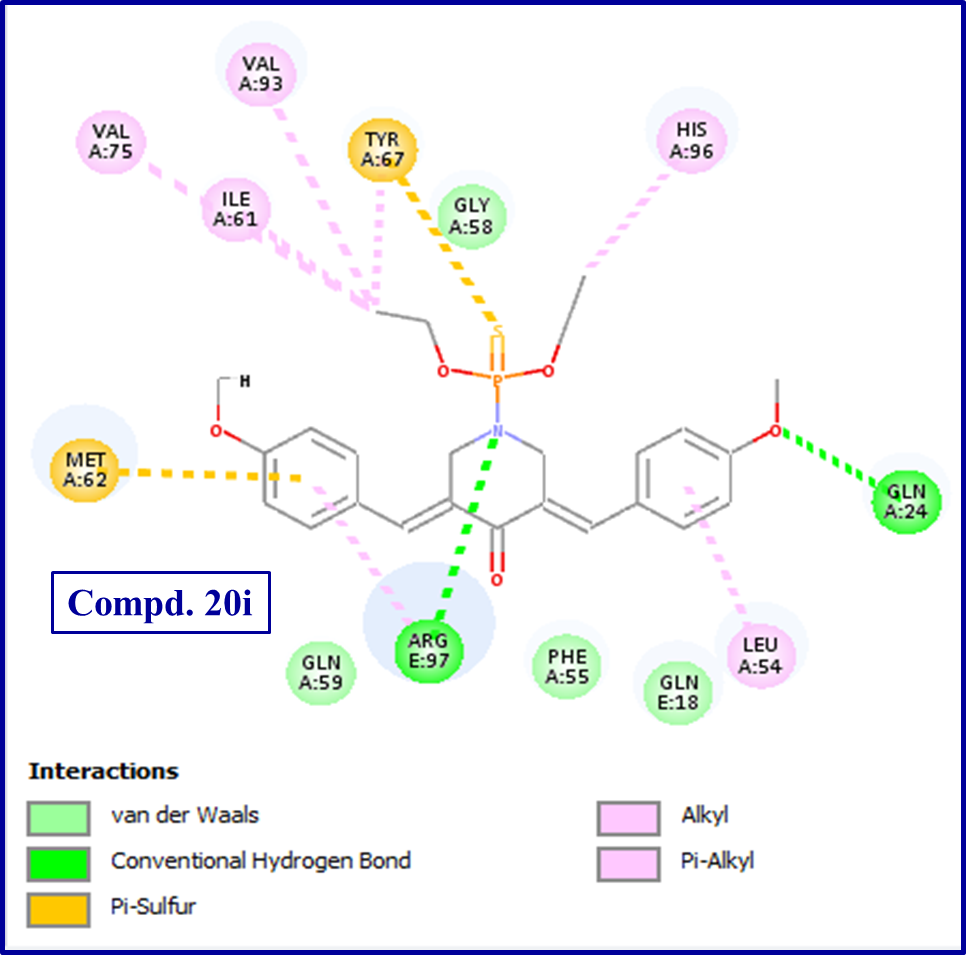


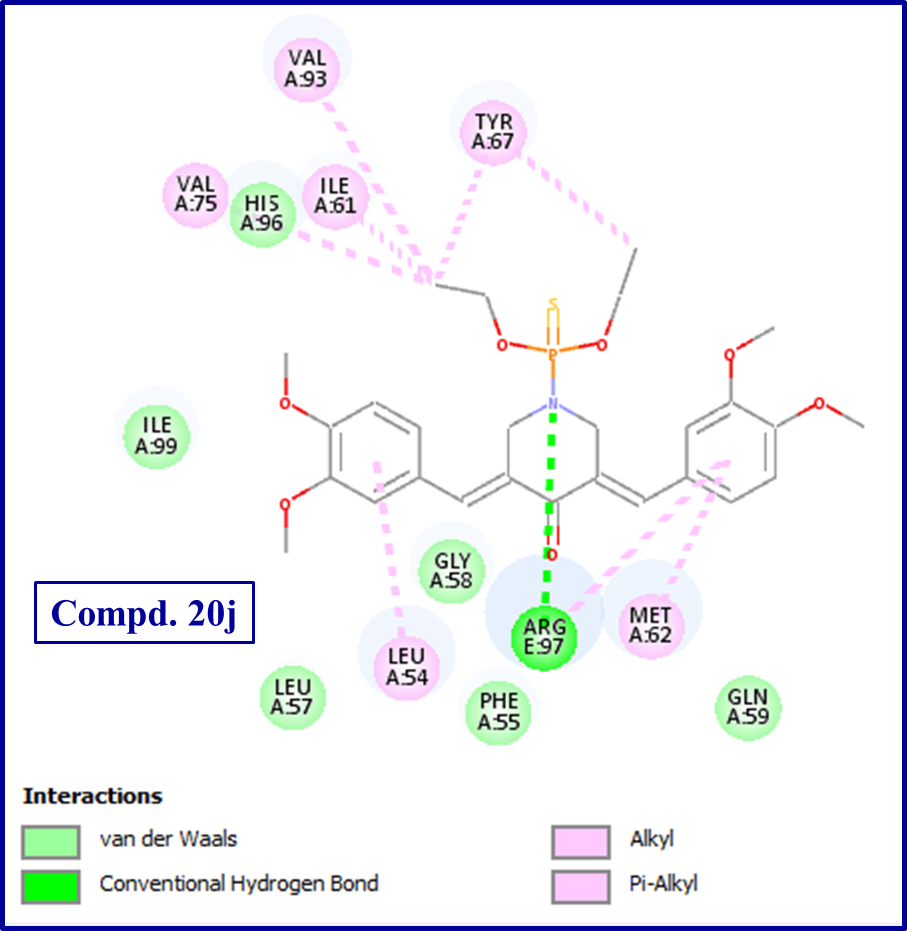


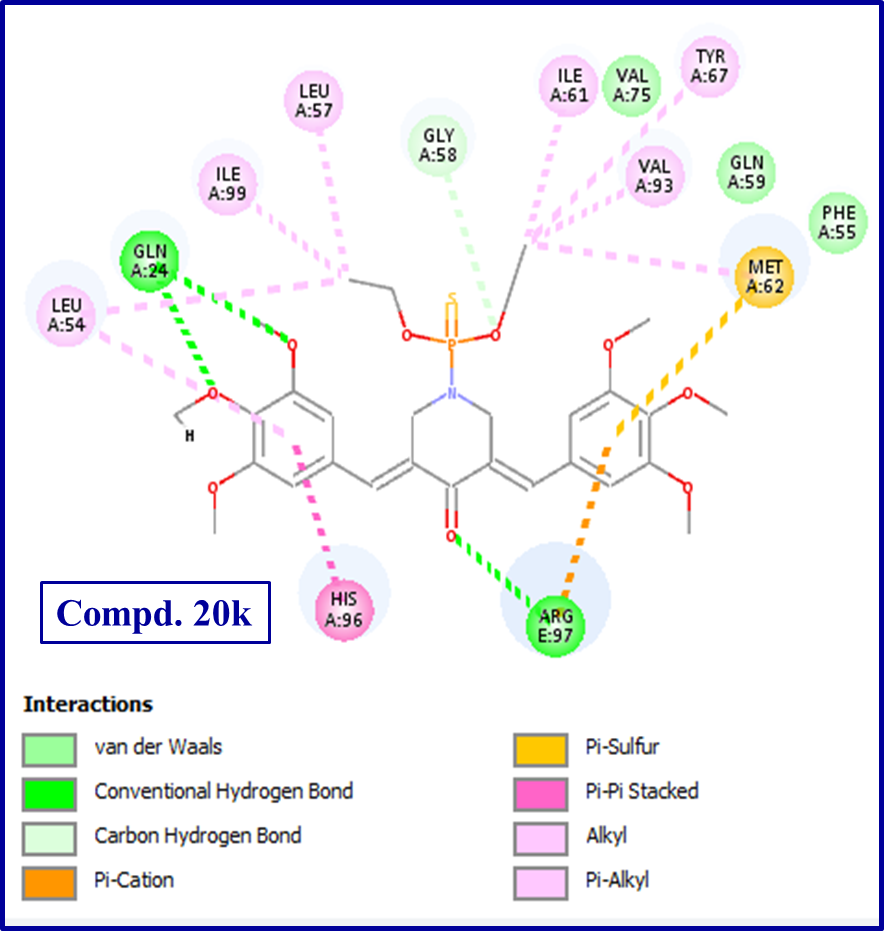


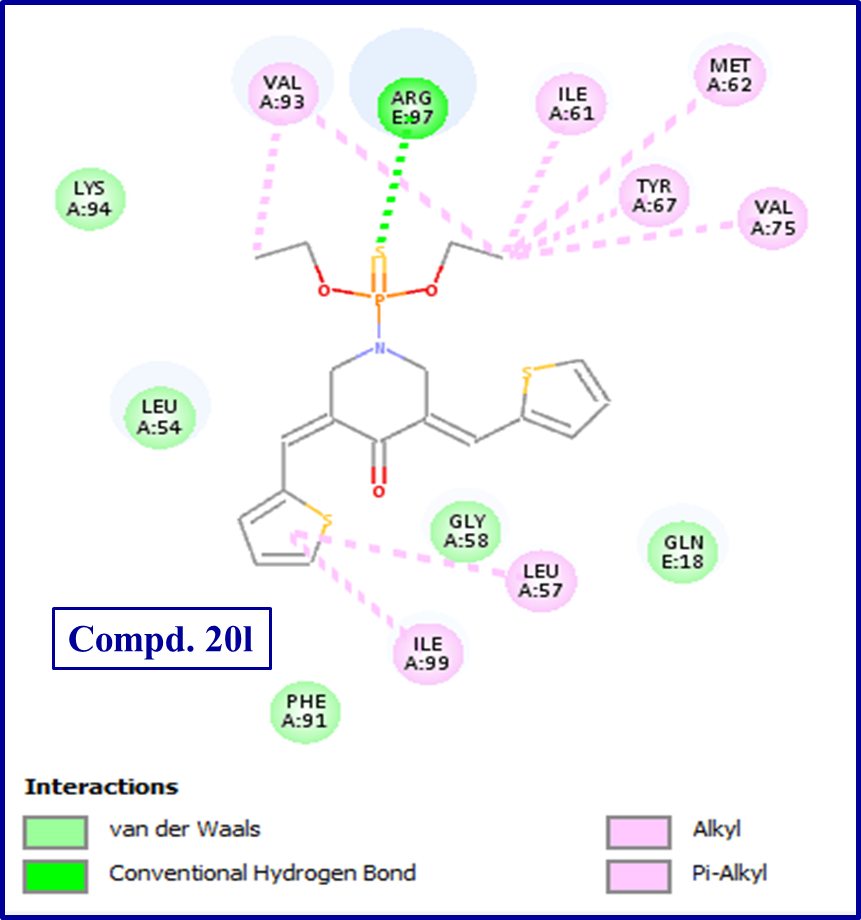


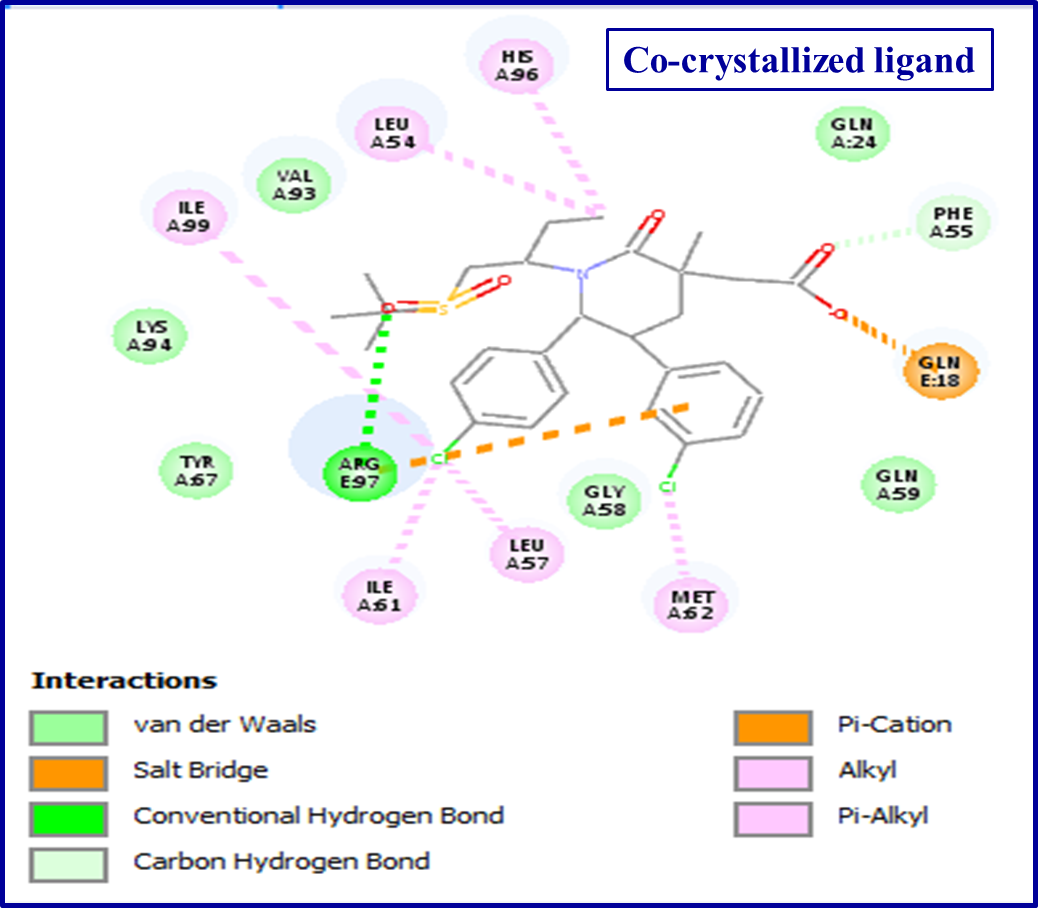


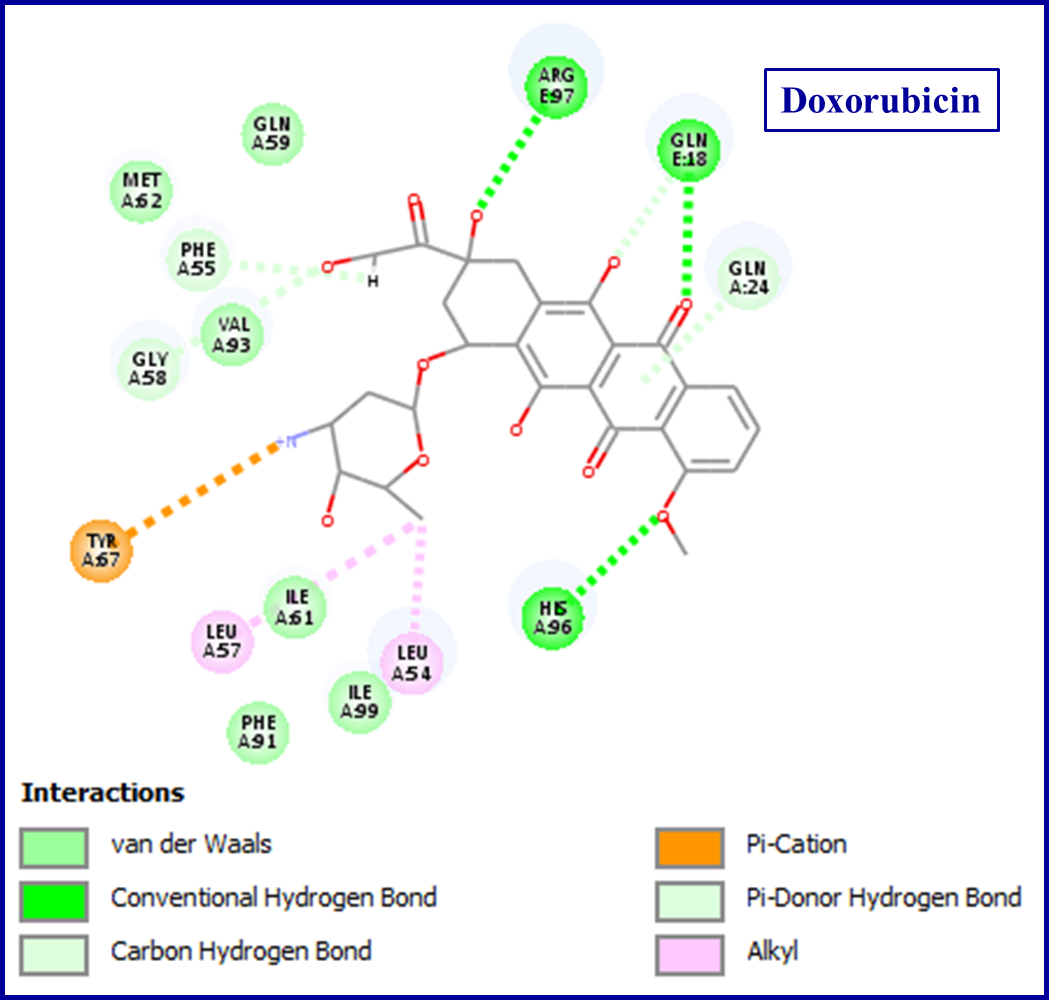


**Fig. S42.** 2D**-**docking poses of the tested compounds **20a‒l**, co-crystallized ligand, and doxorubicin (standard reference/drug) in PDB ID: 4OAS.


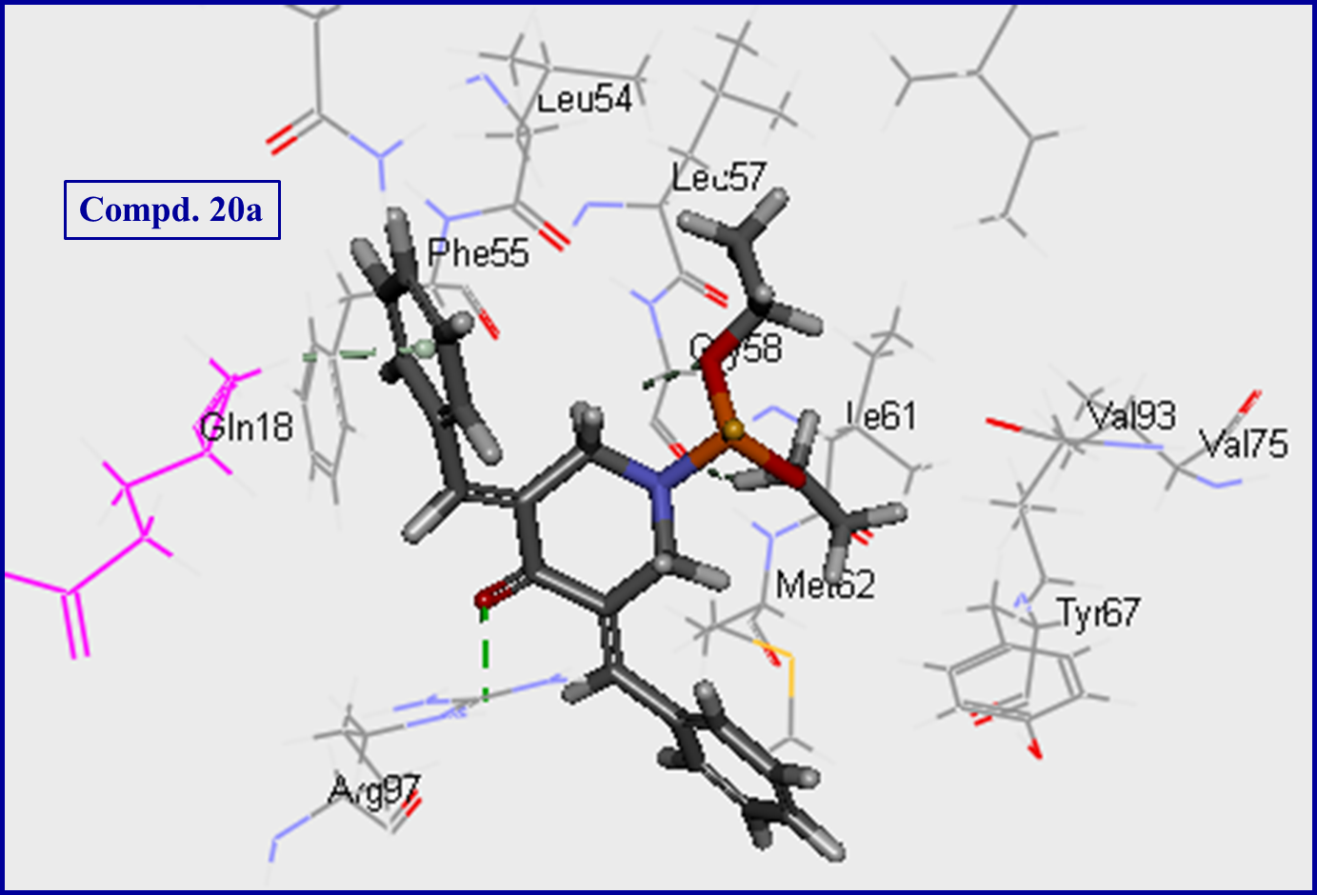


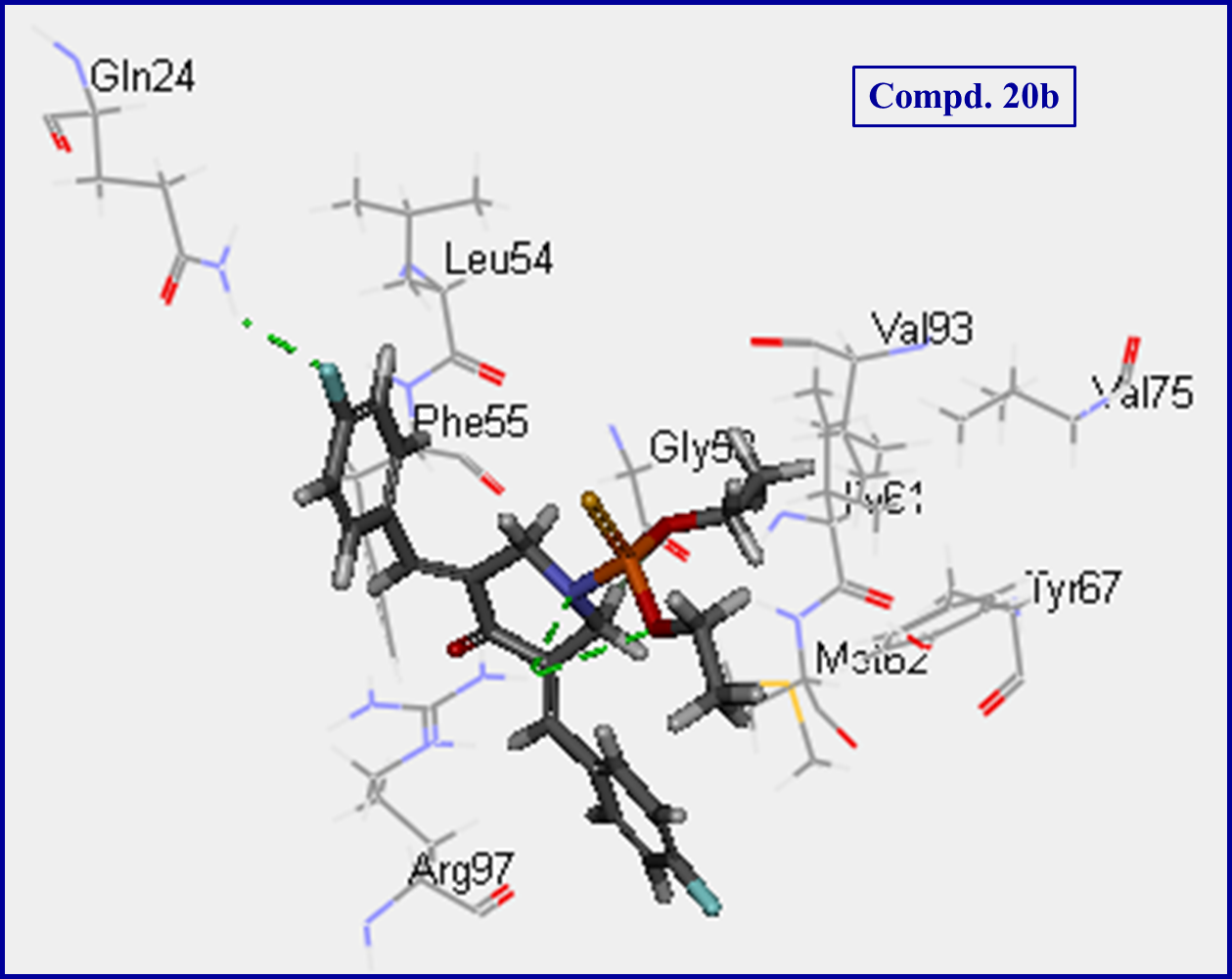


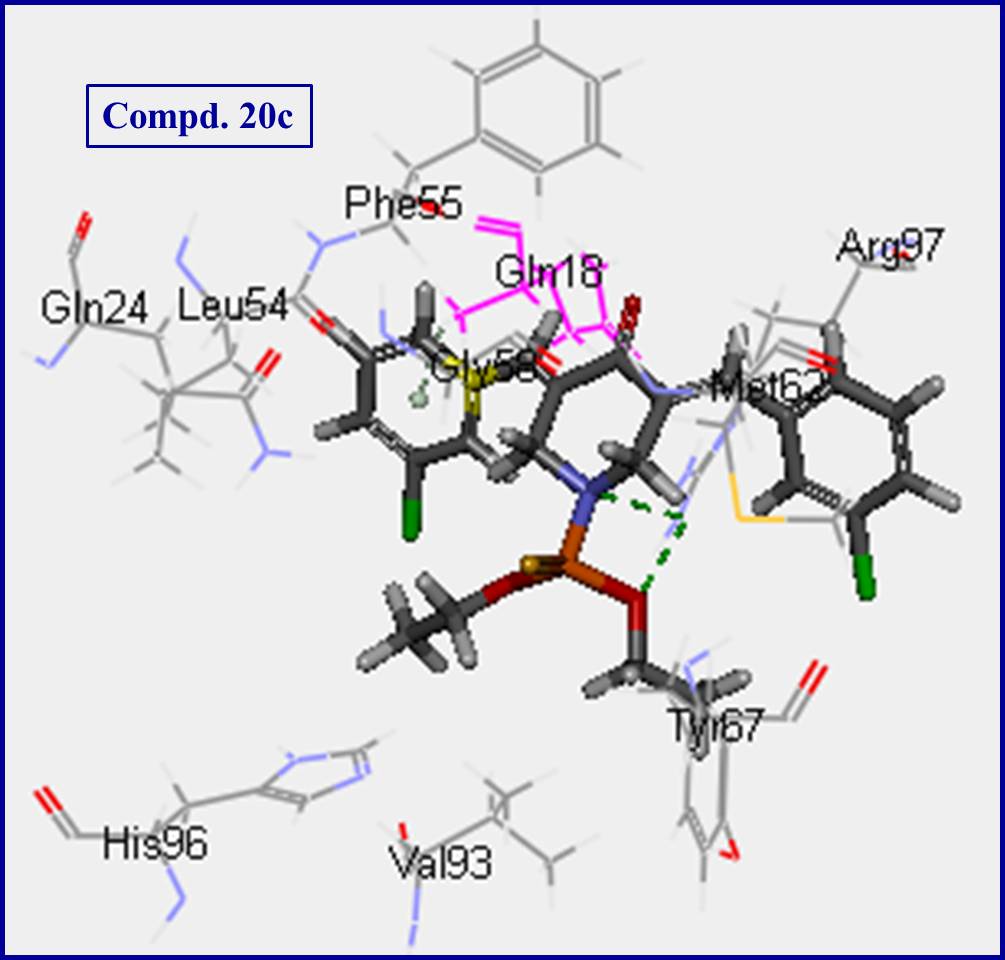

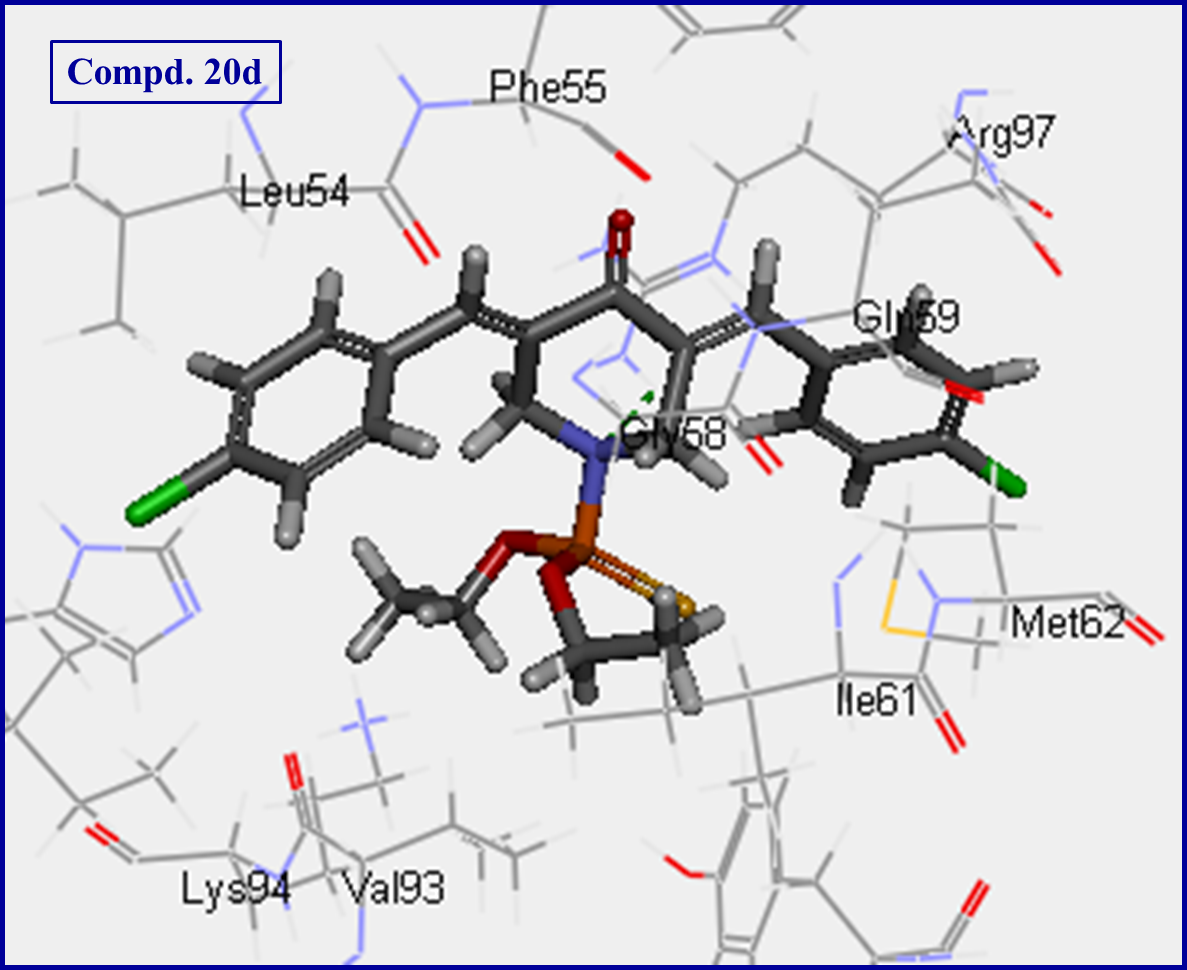


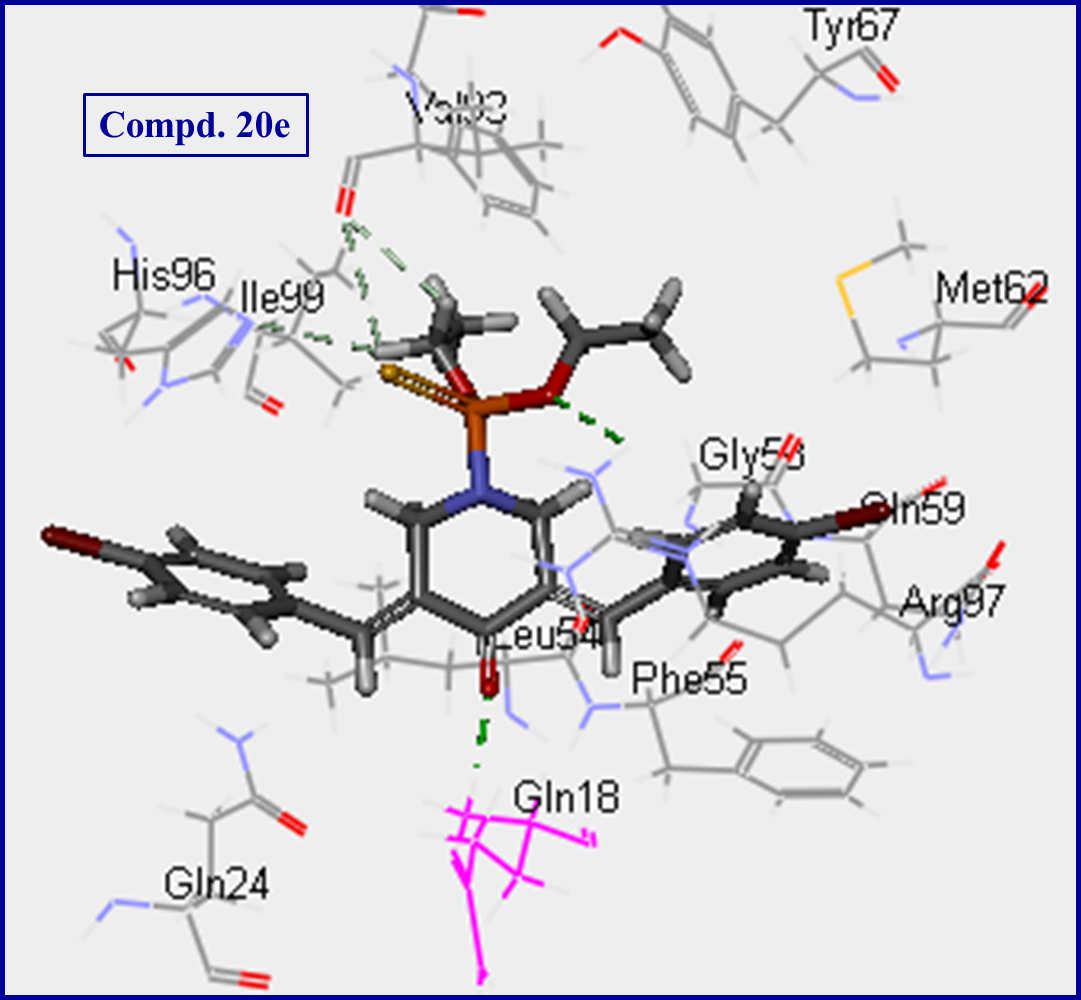


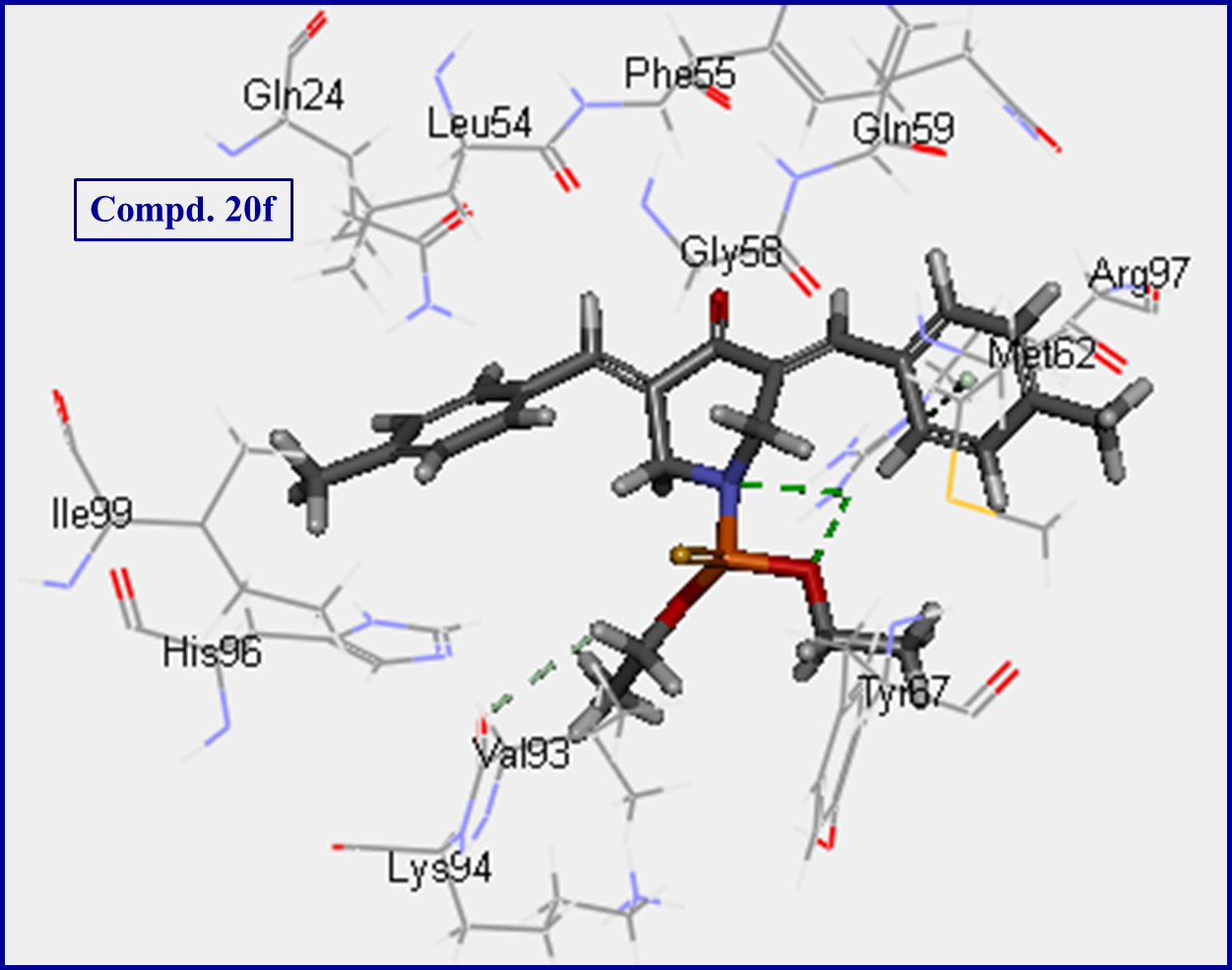


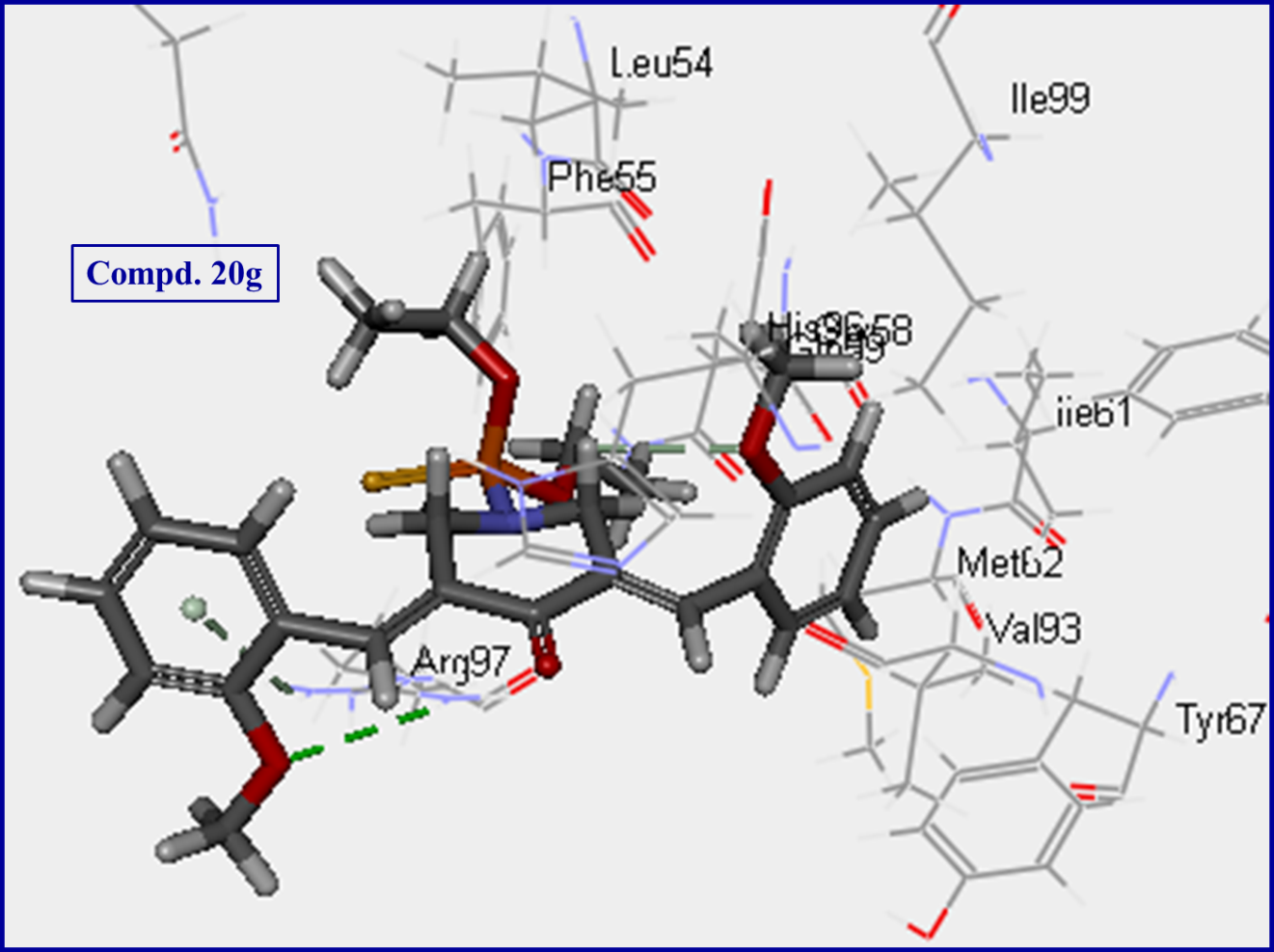


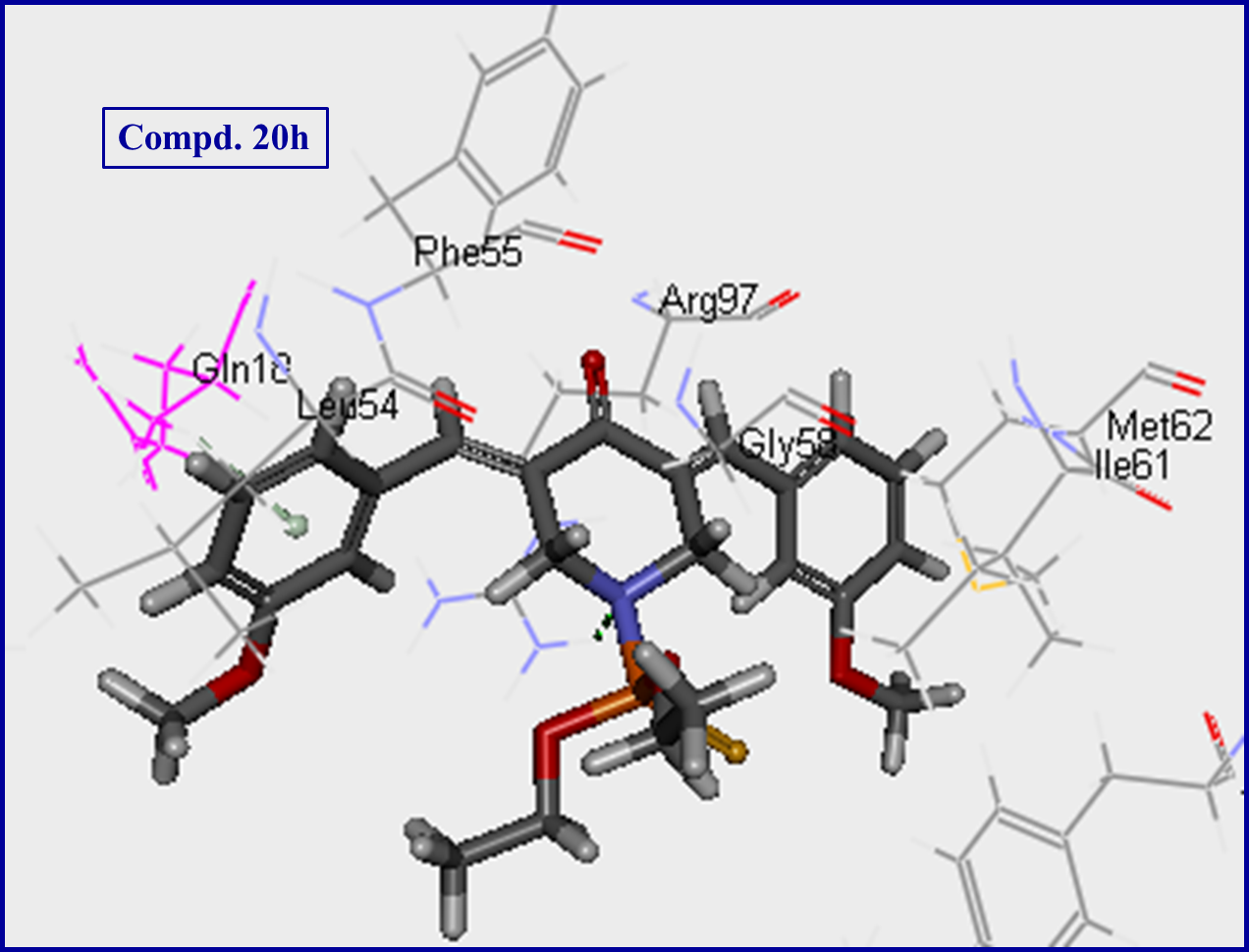


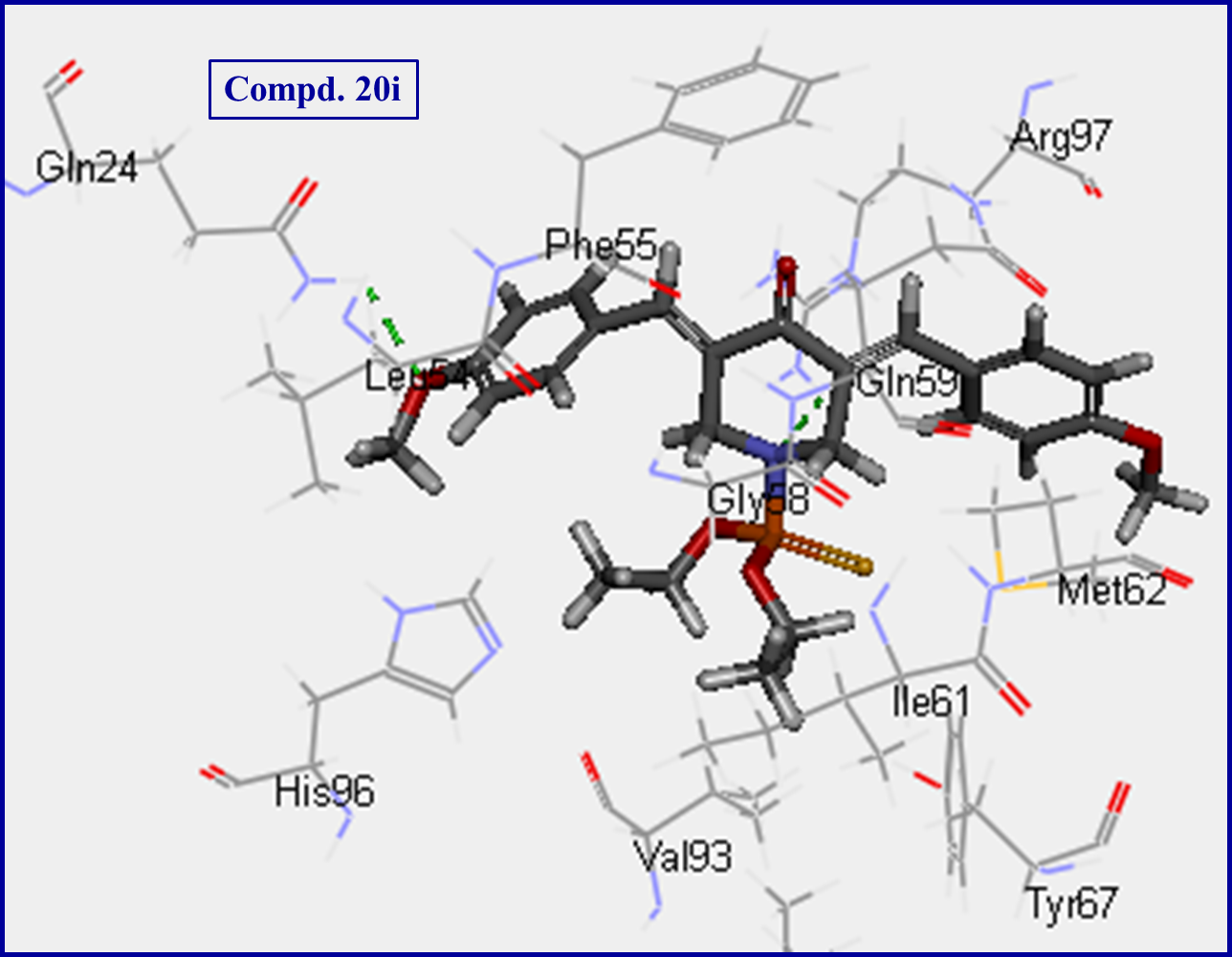


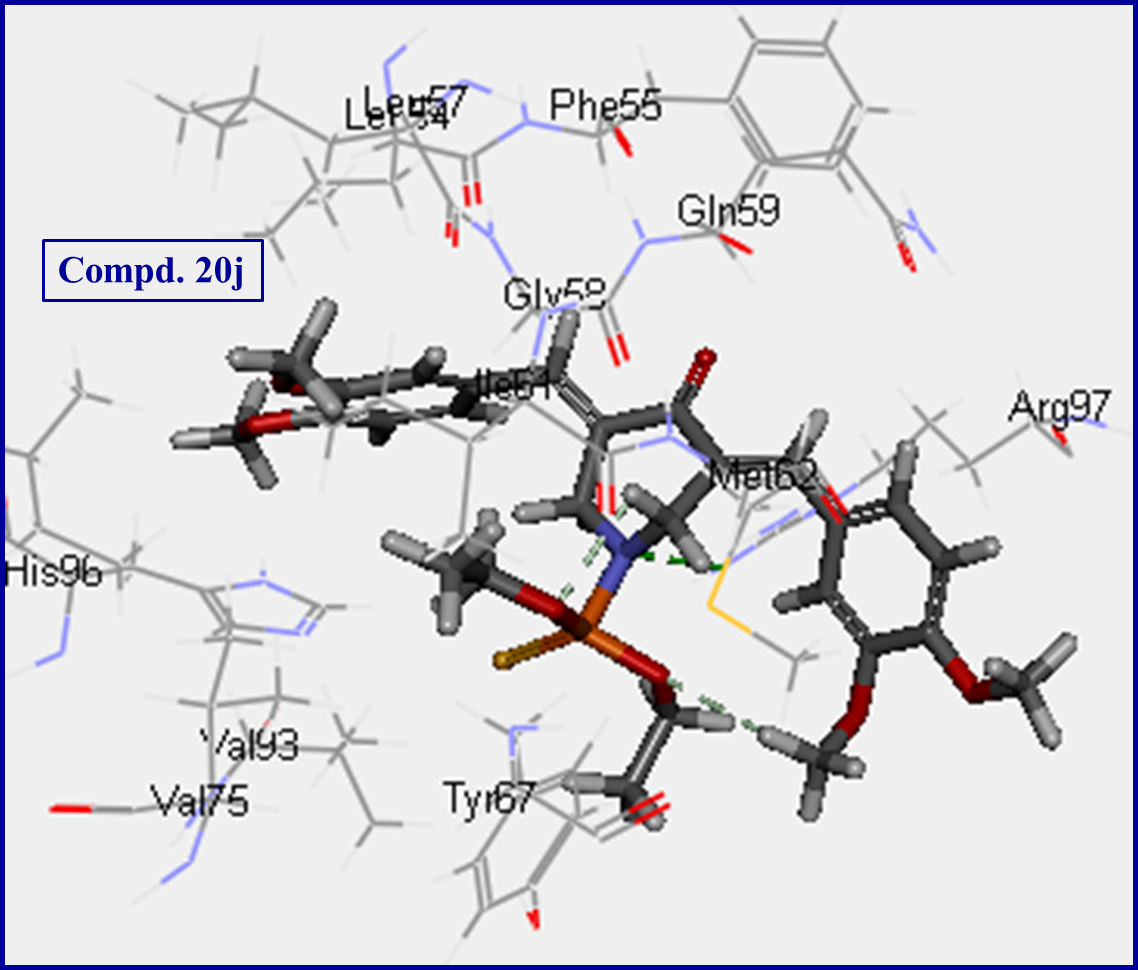


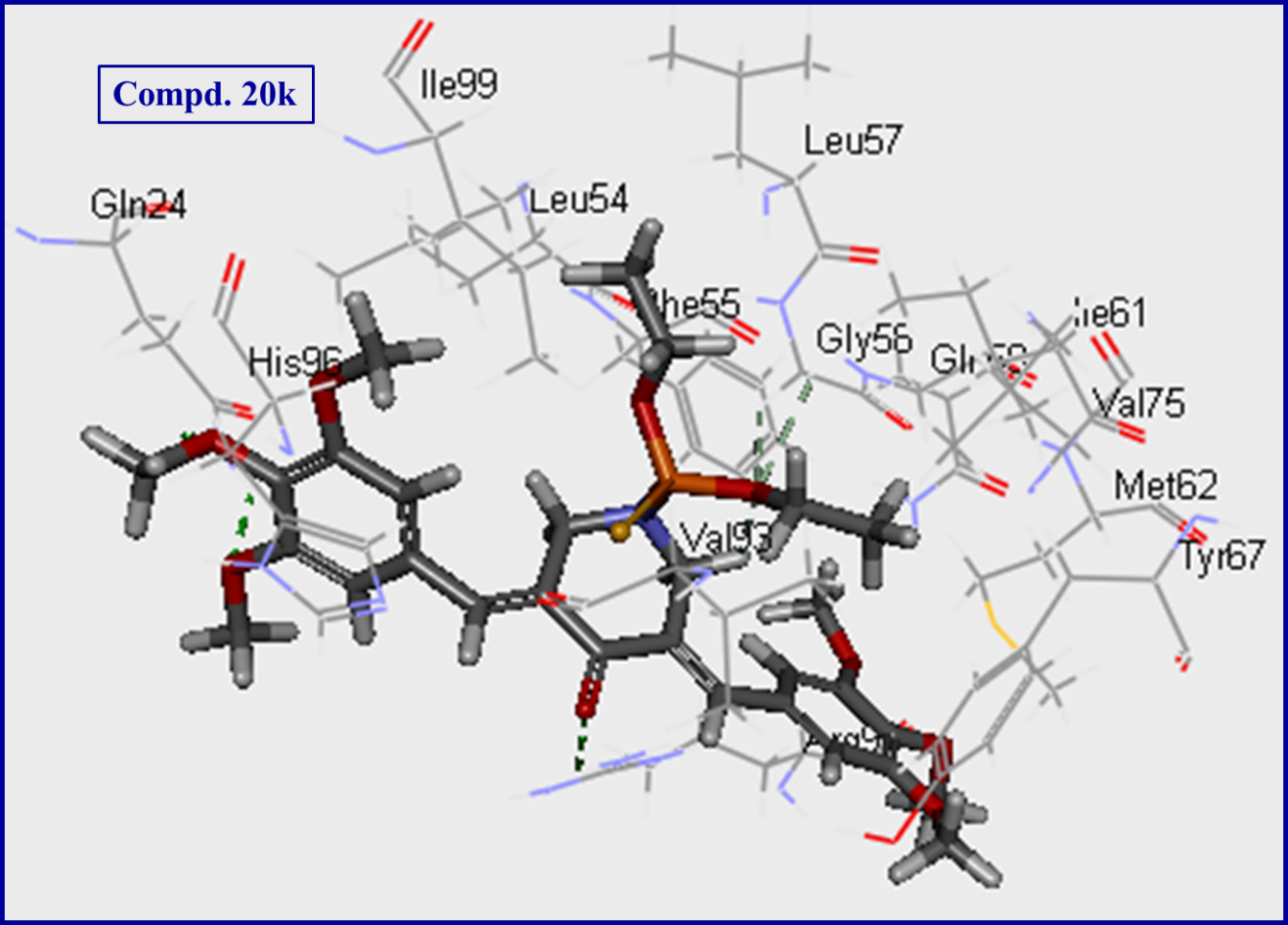


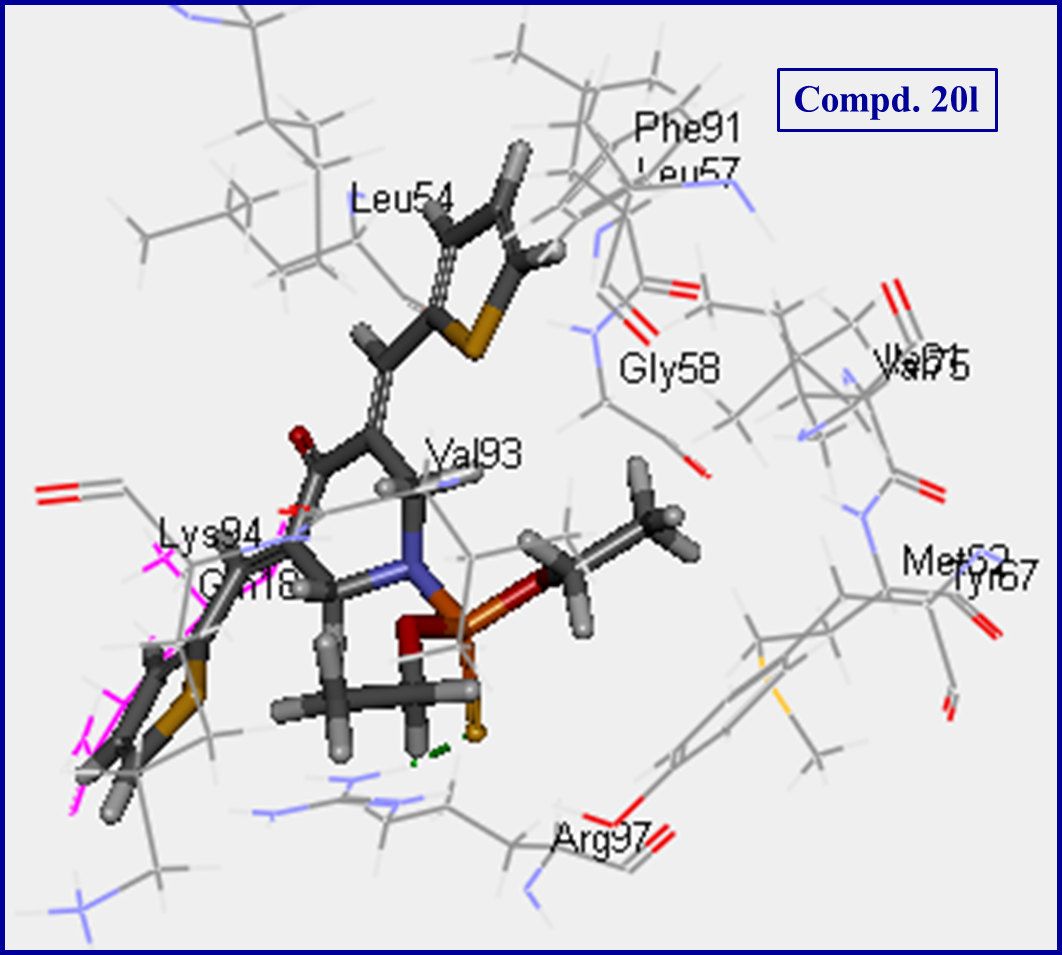


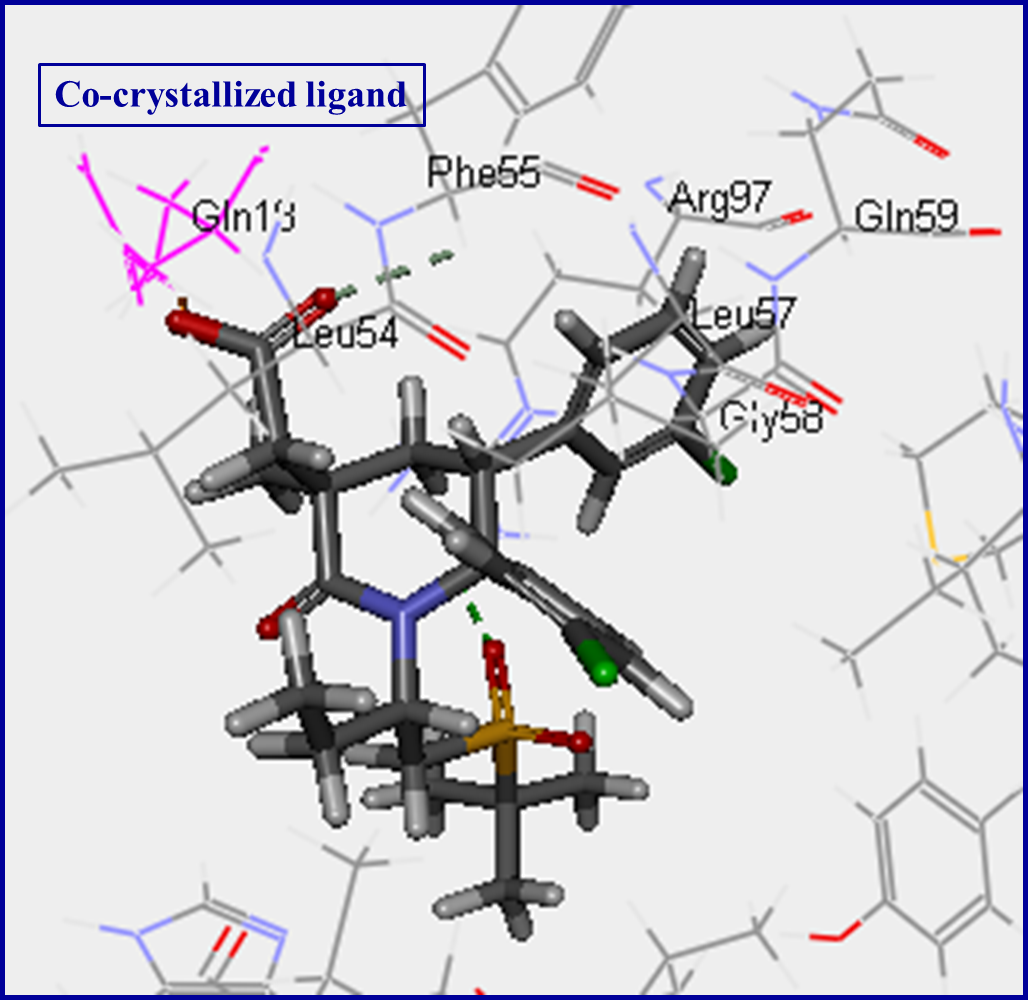


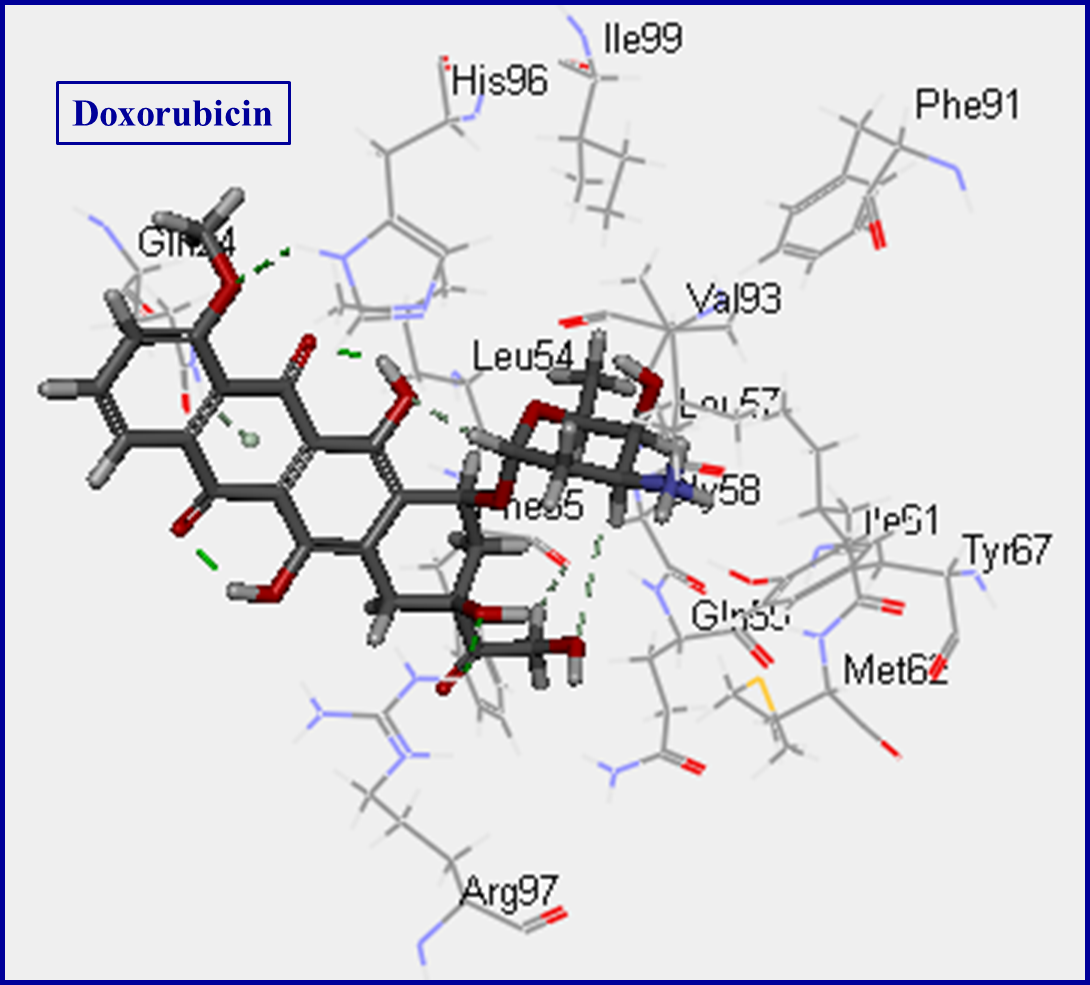


**Fig. S43.** 3D-docking poses of the tested compounds **20a‒l**, co-crystallized ligand, and doxorubicin (standard reference/drug) in PDB ID: 4OAS.
